# Identification and Validation of Apparent Imbalanced Epi-lncRNAs Prognostic Model Based on Multi-Omics Data in Pancreatic Cancer

**DOI:** 10.3389/fmolb.2022.860323

**Published:** 2022-05-12

**Authors:** Mujing Ke

**Affiliations:** Department of Ultrasound, Xiangya Hospital, Central South University, Changsha, China

**Keywords:** long non-coding RNA, pancreatic cancer, TCGA, apparent modification, prognosis

## Abstract

**Background:** Globally, pancreatic adenocarcinoma is a recognized cause of pancreatic death (PAAD) associated with high mortality. Long non-coding RNAs (lncRNAs) play an important role in several biological processes in pancreatic cancer.

**Methods:** The gene expression profile of PAAD patients were obtained from The Cancer Genome Atlas (TCGA) database. The *limma* package was used to identify epigenetic disorders of lncRNAs and PCG. Subsequently, the genomic characteristics and landscape of lncRNAs were explored. The pancreatic cancer-related lncRNAs gene set from Lnc2Cancer v3.0 were collected and the difference between cancer samples and normal samples were analysed. A prognostic model consisting of five epigenetic lncRNA (epi-lncRNAs) was established by univariate and multivariate Cox proportional hazards regression analyses and was verified across different data sets. Finally, the expression of core epi-lncRNAs was identified by PCR experiment.

**Results:** A total of 2237 epi-lncRNAs, 11855 non-epi-lncRNAs, 13518 epi-PCGs, and 6097 non-epi-PCGs, were identified. The abnormal frequency of lncRNAs in pancreatic cancer was much lower than that in PCG, and 138 epi-lncRNAs were enriched in human cancer-related lncRNAs. Epi-lncRNAs had a higher number with longer lengths and a greater number of transcripts. Epi-lncRNAs associated with epigenetic disorders had a higher number of exons, gene length, and isomers as compared to non-epi-lncRNAs. Further, the five pancreatic cancer-specific epi-lncRNA genes (AL161431.1, LINC00663, LINC00941, SNHG10, and TM4SF1-AS1) were identified. Based on these five pancreatic cancer-specific epis-lncRNAs, a prognostic model for pancreatic cancer was established. The RT-PCR result confirmed that AL161431.1, LINC00663, LINC00941, and SNHG10 expressions in pancreatic cancer samples were higher as compared to normal pancreatic samples; the expression of TM4SF1-AS1 in pancreatic cancer cells was significantly lower than that in normal pancreatic samples.

**Conclusions:** Epigenetic abnormalities could promote abnormal lncRNA expression in pancreatic cancer and may play an important role in its progression.

## Introduction

Pancreatic cancer is a highly malignant gastrointestinal tumor. Although its incidence rate is low, it is an important cause of cancer-related deaths. The prognosis of pancreatic cancer remains poor. The overall 5-year survival rate is approximately 5–7%, with an average survival time of 6 months (Rahib et al., 2014; Allemani et al., 2018; Orth et al., 2019). This can be attributed to the high invasiveness of pancreatic cancer and insensitivity towards various chemotherapeutic agents. Additionally, pancreatic cancer patients do not show any specific symptoms at the early stage; due to the lack of reliable methods for screening and detection, most patients are first diagnosed in the advanced stage of the disease (Tesfaye et al., 2018). Serum carbohydrate antigen (CA19-9) is the most commonly used tumor marker for pancreatic cancer. However, due to its low sensitivity and accuracy in clinical settings, many patients remain undiagnosed in the early stage (O'Brien et al., 2015; Locker et al., 2006). Unlike other solid tumors, there is no effective gene target and a clinically relevant biomarker for pancreatic cancer (Mayerle et al., 2018). Pancreatic cancer has strong genetic heterogeneity and a complex tumor microenvironment, making it difficult to study (Krempley and Yu, 2017). To improve its detection and treatment strategies, effective early diagnostic biomarkers and therapeutic targets need to be identified to guide clinical treatment, improve the survival rate, and prolong the survival time.

Long non-coding RNAs (lncRNAs) are RNA transcripts that do not encode polypeptides and are usually greater than 200 nucleotides in length (Jathar et al., 2017). Previous studies show that these play an important role in the occurrence and development of many diseases, including tumors. lncRNAs are distributed in the nucleus and cytoplasm of eukaryotic cells; these interact with several biomolecules, including DNA, RNA, and proteins, thereby, driving important cancer phenotypes and promoting tumor progression (Yan et al., 2015; Fu et al., 2016; Li et al., 2016). In pancreatic cancer, studies show that lncRNAs promote tumor progression. For example, AFAP1-AS1 can promote IGF1R oncogene by isolating miR-133a, thereby, promoting the growth and invasion of pancreatic cancer cells (Chen et al., 2018). lnc-Sox2ot functions as a competitive endogenous (ce) RNA in pancreatic cancer and promotes EMT and stemness (Li et al., 2018). At present, the mechanism of action on lncRNAs is best explained by the ceRNA hypothesis, which elucidates the relationship between coding and noncoding genes in cells and the regulation of mRNA expression. lncRNAs act as miRNA “sponges” and function as a microRNA response element (MRE), causing changes in the level of mRNAs regulated by miRNAs, imbalance in the expression of oncogenic proteins or tumor suppressor proteins, and promoting tumor progression (Salmena et al., 2011; Tay et al., 2014; Li et al., 2020). Although lncRNAs have been used as diagnostic and prognostic biomarkers in some tumors, such as hepatocellular carcinoma, gastric cancer, ovarian cancer, and prostate cancer (Cheng et al., 2014; Gu et al., 2015; Guo et al., 2015; Li et al., 2015), these studies are limited to the role of dysregulated noncoding RNAs in the tumor and lack a systematic analysis of the underlying mechanism of such dysregulation.

In this study, the abnormally expressed genes in pancreatic cancer were systematically analyzed and found a large number of epigenetic abnormalities in lncRNAs. I also focused on the abnormal modification of lncRNA expression. The histone abnormalities mainly included H3K36me3, H3K27me3, H3K27ac, H3K9me3, H3K4me1, and H3K4me3. 138 epi-lncRNAs known to be associated with human cancer were also identified. These abnormal epi-lncRNAs had longer exons and higher numbers of exons and transcripts; the epi-lncRNAs in epigenetic disorders had a higher number of exons, gene lengths, and isoforms as compared to non-epi-lncRNAs. Based on these lncRNAs, the prognosis of pancreatic cancer patients was analyzed and identified five pancreatic cancer-specific epi-lncRNA genes as prognostic markers. Based on these epi-lncRNAs, I constructed a prognostic model for pancreatic cancer. My findings provided a better understanding of the abnormal epigenetic regulation of lncRNA expression in pancreatic cancer, and underlying mechanism of non-coding RNA regulation, and the mechanism of tumor progression in pancreatic cancer.

## Methods

### Expression Profiles and Data Preprocessing

The gene expression profiles of pancreatic cancer (PAAD) and the corresponding Fragments per kilo base per million mapped reads (FPKM) expression profiles along with the clinical information of normal samples were downloaded from The Cancer Genome Atlas (TCGA) database. FPKM data were transformed into Transcript per Million (TPM) format.

### The Genotype-Tissue Expression (GTEx) Data Processing

The expression profile data of GETx normal samples were downloaded from the University of California Santa Cruz (UCSC) Xena database. Pancreatic tissue samples were screened for further processing.

### Combination of GETx Data and TCGA Data

The data for PAAD tumor samples from TCGA and the normal samples from GETx were combined and corrected by the “normalizebeweenarrays” function of the limma package. Then, based on the gene annotation file from GENCODE, the expression profiles were divided into lncRNAs and PCGs and converted the Ensemble ID of these genes into symbol format. Based on the combined sample number, the 177 pancreatic cancer and 166 and normal tissue samples were identified along with their lncRNAs and PCGs expression profiles.

### 450K Methylation Chip Data and Data Preprocessing

The 450K chip data of pancreatic cancer (PAAD) were downloaded from the TCGA database. The chip data were divided into 184 pancreatic cancer samples and 10 normal samples based on the PAAD sample number. The KNN method was used to fill in the missing value in the data for pancreatic cancer.

#### Histone Modification Data and Preprocessing

I downloaded hg38 histone modified replicated “narrowPeak” data from the hg38 versions of the pancreatic cancer cell line, Panc1, and a normal pancreatic cell line from the Encyclopedia of DNA Elements (ENCODE) database. The data included information on six histone modifications, namely, H3K36me3, H3K27me3, H3K27ac, H3K9me3, H3K4me1, and H3K4me3.

### Identification of lncRNAs and Protein Coding Genes (PCGs) With Epigenetic Abnormalities

To explore the correlation between epigenetic variation and pancreatic cancer, first, I transformed the FPKM expression profile data downloaded from TCGA into TPM format. The log-base 2 value was calculated for the expression profile data, and the differentially expressed lncRNAs and PCGs were further identified using the limma package in R. The lncRNAs or PCGs with false discovery rate (FDR) < 0.05 were statistically significant. Second, I screened the specific peaks for pancreatic cancer according to the physical location of histone modification, and only retained those with *p*-value < 0.01 peak as the “differential peak.” The GENCODE GTF files were combined to obtain differentially histone modified genes. I downloaded the human enhancer database from FANTOM5 to identify gene enhancers; Enhancers are located near structural genes and are a class of non-coding DNA cis-acting elements that act on promoters by binding transcription factors, co-factors and chromatin complexes during eukaryotic development, which can activate or enhance transcription of genes. In simple terms, enhancers are DNA sequences that increase the activity of a promoter and thereby increase the frequency of gene transcription.

The promoter of the gene was defined as being located 2 kb upstream and 0.5 kb downstream of the transcription initiation site (TSS). ChIPseeker package in R and human “lincRNAsTranscripts” database was used to identify the gene promoters. Subsequently, the Bumphunter method of the CHAMP package in R was used to identify the DMRs. The regions with BumphunterDMR.p.value <0.01 were considered as significant DMRs. Finally, I defined lncRNAs and PCGs as epigenetic abnormal regulatory lncRNAs or PCGs based on the following criteria: *1*) the significant differential expression of lncRNAs and PCGs in pancreatic cancer tissues as compared to normal tissue samples; *2*) the promoter or enhancer had at least one overlapping differential histone modification or differential methylation region (named epi-lncRNA, non-epi-lncRNA, epi-PCG, or non-epi-PCG).

### Genomic Signal Characterization for Epigenetically Dysregulated lncRNAs

To compare the genomic characteristics of epigenetically dysregulated and non-dysregulated lncRNAs/PCGs, I compared the exons, transcripts, numbers, and lengths of the epi-lncRNA, non-epi-lncRNA, epi-PCG, and non-epi-PCG genes.

### Genomic Map of Epigenetically Dysregulated lncRNAs Modified by Different Histones

To explore the epigenetic characteristics of lncRNAs influenced by histone modification, the distributional characteristics of promoters was analyzed and enhancers of epi-lncRNAs were modified by different histones throughout the genome.

### lncRNA-Based Recognition of Pancreatic Cancer-specific Epigenetic Aberrations

To understand the relationship between epigenetically dysregulated lncRNAs and cancer, I analyzed the enrichment of epi-lncRNAs and known cancer lncRNAs using Lnc2Cancer v3.0. To further explore the regulation by epi-lncRNAs in pancreatic cancer, I identified epi-lncRNAs associated with pancreatic cancer. In addition, given that most of the genes affecting the disease state show dysregulation, the TPM expression profile data from TCGA in combination was used to identify the epi-lncRNAs exhibiting significant changes in pancreatic cancer.

### The Relationship Between the Epigenetic Imbalance in lncRNAs and the Prognosis of Pancreatic Cancer

To understand the potential prognostic value of epi-lncRNA imbalance in-depth, based on the above-derived expression values of epi-lncRNAs in cancer tissues, I divided the samples into high expression and low expression subgroups and analyzed the survival time (OS) and survival status for each sample category. The survival rates and survival times were estimated using the Kaplan-Meier method, and the significant differences between subgroups consisting of different samples were compared based on the log-rank test. I also identified epi-lncRNA markers for prognostic prediction of pancreatic cancer.

### RT-PCR

Total RNA was extracted from the cells as follows: The cells from each experimental group were collected and RNA was extracted according to the manufacturer’s instructions using the Trizol kit. The RNA quality was measured using Nanodrop by estimating its concentration at 260/280 nm. The RNA concentration was determined as 1000 ng. One Step PrimeScript^®^ miRNA cDNA Synthesis Kit (Takara, Japan) was used for reverse transcribing the RNA to cDNA; SYBR^®^ Premix Ex Taq^™^ II (Perfect Real Time) (Takara, Japan) was used to detect the relative expression of mRNA; PCR conditions were as follows: 95°C for 15 s, 58°C for 34 s; 45 cycles in total. Using threshold cycle and 2- ΔΔ methods, the relative expression of mRNA was calculated by CT method (β- Act was used as the internal reference). Primer sequences for LINC00663: Forward (5′-GGC​AGC​GAT​GAT​GAC​CGT​AA-3′), Reverse (5′-GGT​AGT​GCA​GGT​CCA​CTG​AA-3′); primer sequences for TM4SF1-AS1: Forward (5′-TGC​AAG​TCA​CTC​TGA​TGC​CG-3′), Reverse (5′-AGC​TCT​GAG​CAA​ACC​ATC​CTC-3′); primer sequences for AL161431.1: Forward (5′-GGC​AGC​GAT​GAT​GAC​CGT​AA-3′), Reverse (5′-AGG​TAC​CAC​AGG​AGG​CAC​AA-3′); primer sequences for LINC00941: Forward (5′-CAG​GTC​AGG​TTA​TGC​AAC​GC-3′), Reverse (5′-GGG​TTG​GTC​TCA​GAG​GGA​CT-3′), and primer sequences for SNHG10: Forward (5′-GCC​CTC​CAG​CCT​TTT​AAC​CT-3′), Reverse (5′-TTC​TCA​CGA​TGG​GTC​CAA​GC-3′).

## Results

### Identification of lncRNAs and PCGs With Epigenetic Aberrations

**FIGURE 1:**
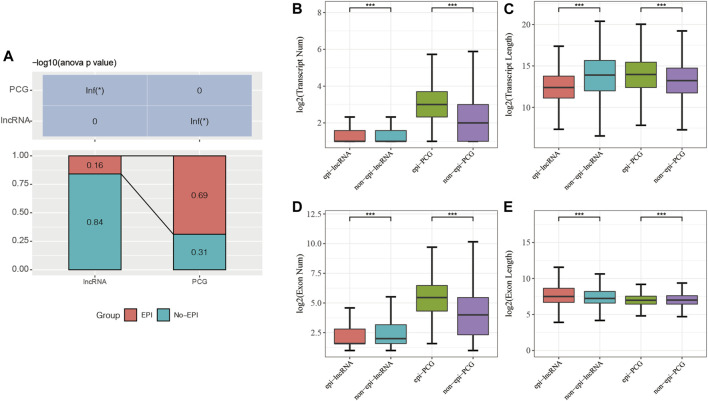
**(A)** Percentage of epi-lncRNAs and epi-PCGs for all lncRNAs and PCGs in the genome; **(B**–**E)**: Comparison of the genomic characteristics of epigenetically dysregulated lncRNAs/PCGs and non-epigenetically dysregulated lncRNAs/PCGs.

My goal was to analyze the relationship between lncRNA expression and epigenetic alterations in pancreatic cancer. The limma package was used and identified significantly differentially expressed genes, including 16805 PCGs (Supplementary Table S1) and 9167 lncRNAs (Supplementary Table S2). Combined with the histone modification data and 450K methylation microarray data, the 2237 epi-lncRNAs, 11855 non-epi-lncRNAs, 13518 epi-PCGs, and 6097 non-epi-PCGs (Supplementary Table S3) were identified and found that these lncRNAs showed abnormally low frequency in pancreatic cancer than PCGs (Figure 1A).

I compared the numbers and lengths of exons and transcripts of epi-lncRNAs, non-epi-lncRNAs, epi-PCGs, and non-epi-PCGs to elucidate the genomic characteristics of epigenetically dysregulated lncRNAs. The lengths of transcripts of epi-lncRNAs were lesser than those of non-epi-lncRNAs. Epi-PCGs had a greater number of transcripts; the lengths of transcripts were greater as compared to those of the non-epi-PCGs (Figures 1B,C). The number of exons in epi-lncRNAs was low, but the lengths of the exons were high. The number of exons in epi-PCGs was greater than that in non-epi-PCGs (Figures 1D,E).

### Genomic Landscape of Epigenetically Dysregulated lncRNAs

**FIGURE 2:**
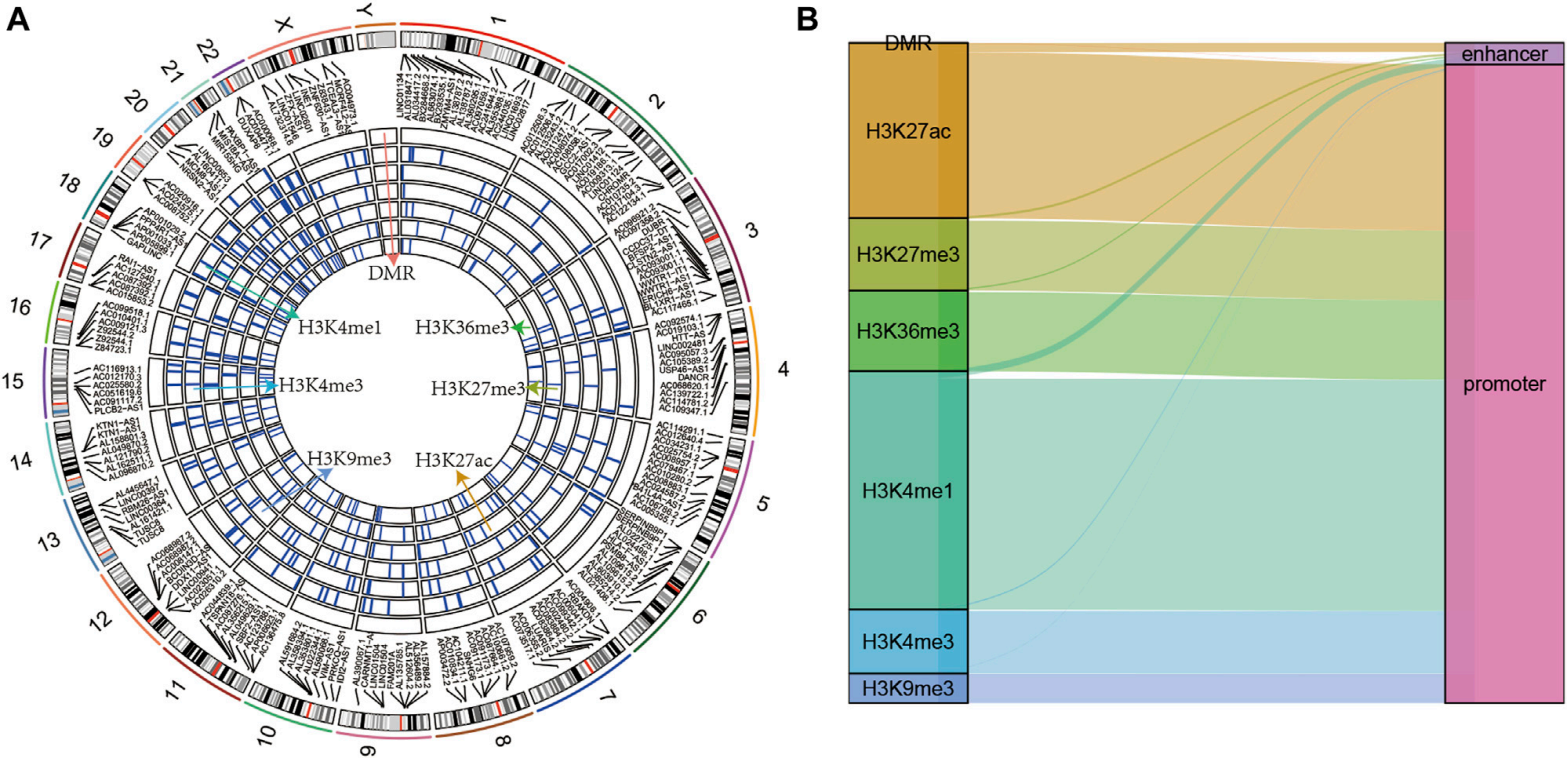
**(A)** Genomic landscape of epi-lncRNAs having differential histone modifications and differential methylation regions; **(B)** Type distribution of epi-lncRNAs having differential apparent dysregulations.

The epi-lncRNAs in pancreatic cancer were systematically analyzed and found epi-lncRNAs landscapes having differential histone modifications and differential methylation regions (Figure 2A). I found that the abnormal histone modifications in these lncRNAs mainly included H3K36me3, H3K27me3, H3K27ac, H3K9me3, H3K4me1, and H3K4me3. Most apparently dysregulated lncRNAs were accompanied by several abnormalities in histone modifications. In addition, I also observed that the regions with abnormal histone modifications were mainly concentrated in the promoter regions (Figure 2B).

### ssGSEA Analysis of Dysregulated Epi-lncRNAs

**FIGURE 3:**
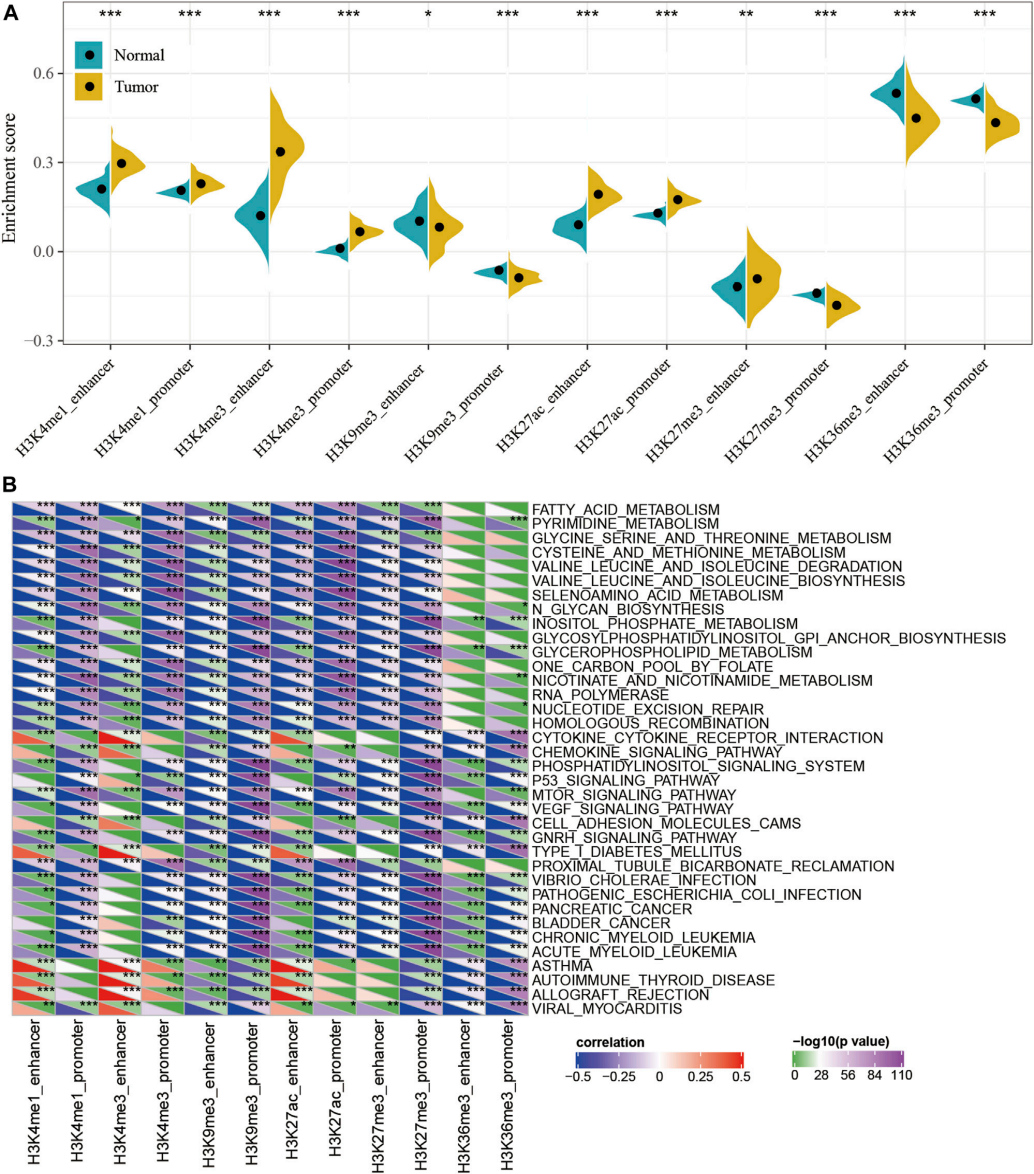
Functional analysis for epi-lncRNAs. **(A)** Differences in 12 kinds of apparent epigenetic dysregulations in lncRNAs between cancerous and adjacent non-cancerous samples; **(B)** KEGG pathways most significantly associated with the 12 apparent epigenetically dysregulated lncRNAs.

To characterize the potential roles of lncRNA dysfunction induced by the abnormalities in histone modifications, the relationship between the expression of epi-lncRNAs and pathways in pancreatic cancer were systematically analyzed. Specifically, I extracted the expression profiles of lncRNAs induced by histone modifications and calculated the enrichment score for each sample using Single-sample GSEA (ssGSEA). It was observed that the enrichment scores of H3K4me1_enhancer, H3K4me1_promoter, H3K4me3_enhancer, H3K4me3_promoter, H3K27ac_enhancer, H3K27ac_promoter, and H3K27me3_enhancer in tumor samples were significantly higher than those in the corresponding adjacent samples, while the enrichment scores for H3K9me3_ enhancer, H3K9me3_ promoter,H3K27me3_promoter, H3K36me3_enhancer, and H3K36me3_promoter in adjacent samples were significantly higher than those in tumor samples (Figure 3A). This suggested that H3K9me3_enhancer, H3K9me3_promoter, H3K27me3_promoter, H3K36me3_enhancer, and H3K36me3_promoter may exert protective effects, while H3K4me1_enhancer, H3K4me1_promoter, H3K4me3_enhancer, H3K4me3_promoter, H3K27ac_enhancer, H3K27ac_promoter, and H3K27me3_enhancer may have cancer-promoting effects.

In addition, the Kyoto Encyclopedia of Genes and Genomes (KEGG) pathway scores for each sample were also evaluated and calculated the relationship between the enrichment score of each epi-lncRNA and the corresponding KEGG pathway to obtain the relevant significant enriched KEGG pathway for each epi-lncRNA. A total of 36 pathways were most significantly related to 12 epi-lncRNAs (Figure 3B and Supplementary Table S4), which suggest that different types of epi-lncRNA-related pathways were consistent. Among these 36 pathways, there were tumor-related pathways, including those in PANCREATIC_CANCER, BLADDER_CANCER, and metabolism-related pathways, including FATTY_ ACID_ METABOLISM, PYRIMIDINE_METABOLISM, GLYCINE_ SERINE_ AND_ THREONINE_ METABOLISM, CYSTEINE_ AND_ METHIONINE_ METABOLISM, SELENOAMINO_ ACID_ METABOLISM, INOSITOL_ PHOSPHATE_ METABOLISM, GLYCEROPHOSPHOLIPID_ METABOLISM, and NICOTINATE_ AND_ NICOTINAMIDE_ METABOLISM pathways. These results implied that epi-lncRNAs were closely related to the occurrence, development, and metabolism of tumors.

### Relationship Between Apparently Dysregulated lncRNAs and RNA Modifications

**FIGURE 4:**
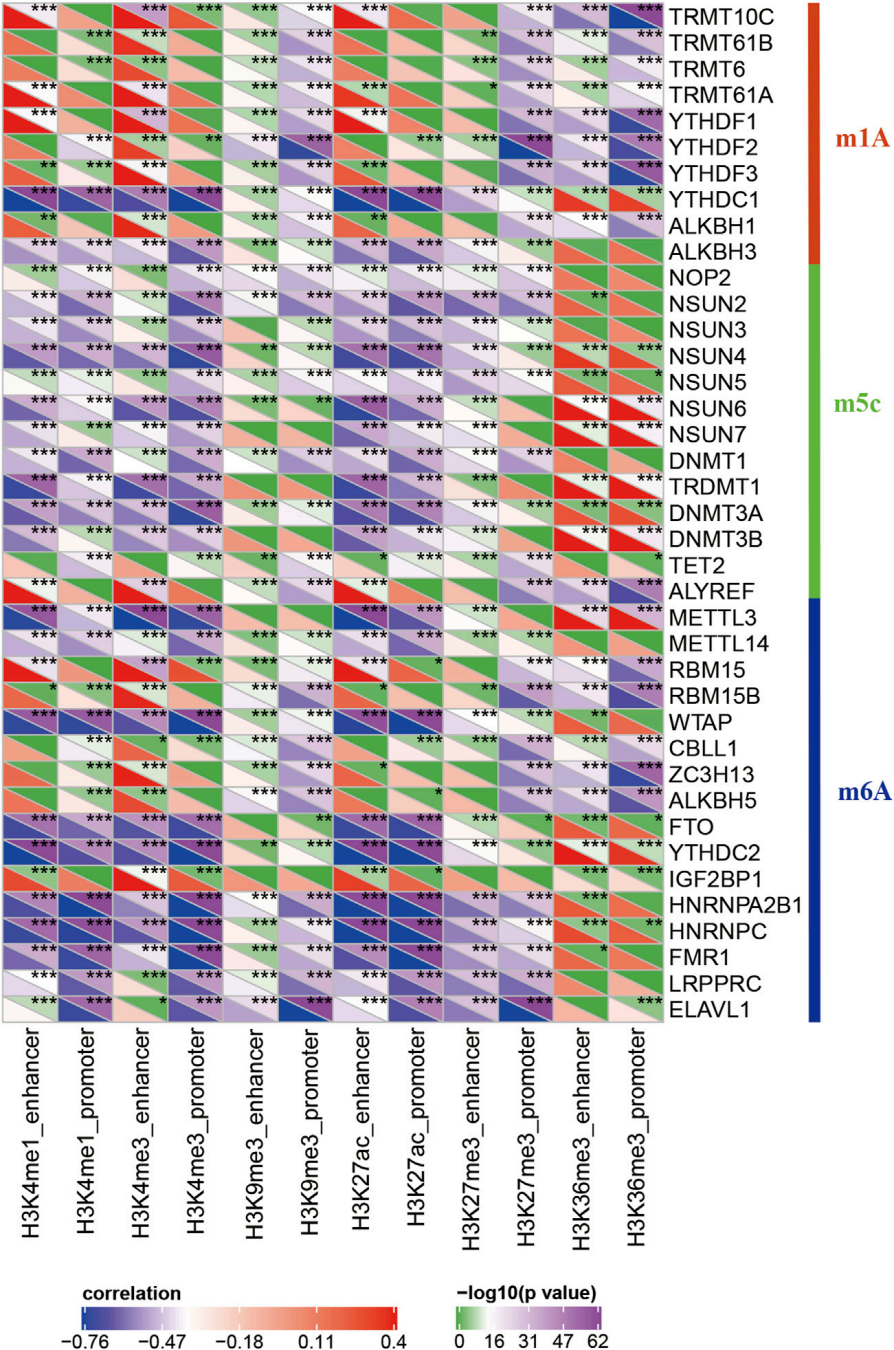
Correlation between enrichment scores of 12 epi-lncRNAs and m6A-, m5C-, and m1A-related genes.

RNA modifications are important epigenetic features related to several important biological processes. Thus, I analyzed the relationship between 20 different epi-lncRNAs, m6A, and m5C genes. Specifically, the m6A, m5C, and m1A gene expression profiles for TCGA pancreatic cancer cohort were extracted and calculated the correlation for the 12 epi-lncRNA enrichment scores and m6A, m5C, and m6A genes. The enrichment scores were significantly correlated with m6A-, m5C-, and m1A-related genes. Among them, H3K36me3, H3K27me3, H3K27ac, H3K9me3, H3K4me1, and H3K4me3 had both similar and unique correlations with these genes, which suggest that there may be different regulatory modes of lncRNA imbalance caused by histone modifications in enhancer and promoter regions. These epi-lncRNAs were closely related to RNA modifications (Figure 4).

### Identification of Pancreatic Cancer-specific Epigenetically Dysregulated lncRNAs

**FIGURE 5:**
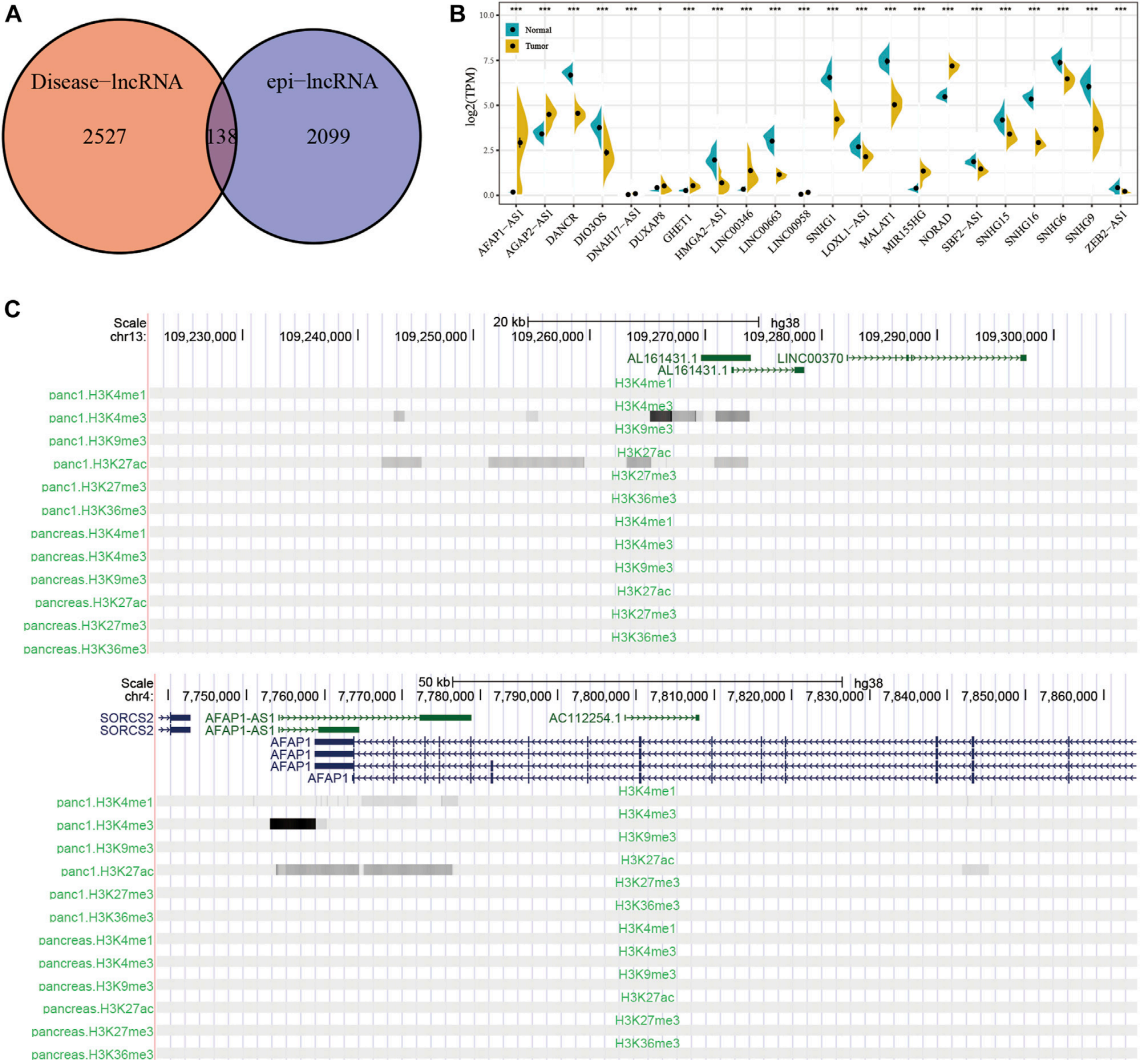
**(A)** Intersection of disease-related lncRNAs with pancreatic cancer-specific epi-lncRNAs. **(B)** The expression differences between epi-lncRNAs related to pancreatic cancer in tumor and normal samples. **(C)** Histone modification profiles for AL161431.1 and AFAP1-AS1.

Using Lnc2Cancer V3.0 for analysis, 138 epi-lncRNAs known human cancer-related lncRNAs were obtained (Figure 5A and Supplementary Table S4). To further confirm the regulatory role of candidate lncRNAs in pancreatic cancer, I collected a set of pancreatic cancer-associated lncRNAs genes from Lnc2Cancer V3.0 and found 22 previously reported epi-lncRNAs directly associated with pancreatic cancer. These 22 genes are shown in Supplementary Table S5. Then, the differences between expressions of the 22 epi-lncRNAs in pancreatic cancer and normal tissue samples were calculated (Figure 5B). Further, I selected the histone modification profiles of AL161431.1 and AFAP1-AS1. The results showed that the expression levels of histones H3K27ac and H3K4me3 for AL161431.1 and AFAP1-AS1 in tumor samples were higher as compared to the normal samples (Figure 5C).

### Epigenetically Aberrant lncRNAs Are Associated With the Prognosis of Pancreatic Cancer

**FIGURE 6:**
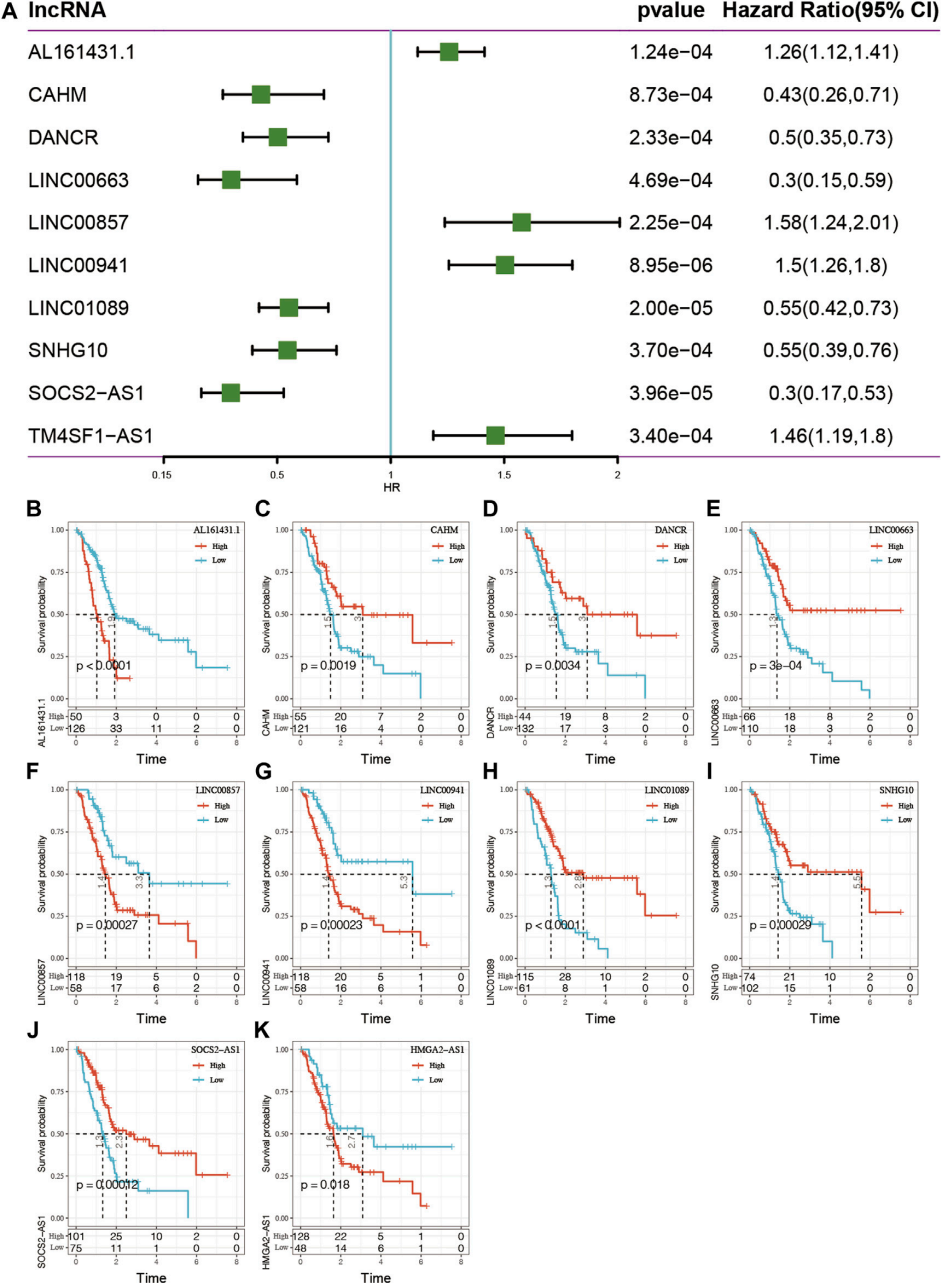
**(A)** Single-factor analysis of 10 epi-lncRNA genes related to pancreatic cancer. **(B–K)** Survival curve analyses for the 10 genes.

To understand the potential prognostic value of epi-lncRNA imbalance, the 138 above-mentioned epi-lncRNA genes was used to analyze the survival of TCGA pancreatic cancer patients. The results identified significantly (*p* < 0.001) related genes and found the following: *1*) in univariate Cox analysis, 10 genes were significantly associated with survival, including AL161431.1, CAHM, DANCR, LINC00663, LINC00857, LINC00941, LINC01089, SNHG10, SOCS2-AS1, and TM4SF1-AS1 (Figure 6A); *2*) using the *max_stat* package in R software, these 10 genes were truncated to obtain high and low expression subgroups. It found that the survival curves for high- and low-expression subgroups for these 10 genes were significant (Figure 6B).

### Five Epi-lncRNA Signatures as Prognostic Markers for Pancreatic Cancer

To establish a prognostic model based on epi-lncRNA maladjustment, I prioritized the 138 pancreatic cancer-related epi-lncRNAs genes to a final 10 using the single factor COX analysis. Survival analysis was performed for the 10 genes. To further prioritize the most relevant genes, a stepwise regression using the AIC Akaike information criterion based on the statistical fit of the model and the number of parameters used for fitting was performed. The stepAIC method of the MASS package starts from the most complex model and successively deletes a variable each time to reduce the AIC. The smaller the value, the better the model as it was indicative of the model obtaining sufficient fit with fewer parameters. Using this algorithm, the five pancreatic cancer-specific epi-lncRNAs, including, AL161431.1, LINC00663, LINC00941, SNHG10, and TM4SF1-AS1 genes were finally obtained. These were selected as prognostic markers. Calculation was as follows: RiskScore =0.1479814*AL161431.1– 0.6410330*LINC00663+0.2429150* LINC00941–0.6692016*SNHG10+0.2734249*TM4SF1-AS1.

**FIGURE 7:**
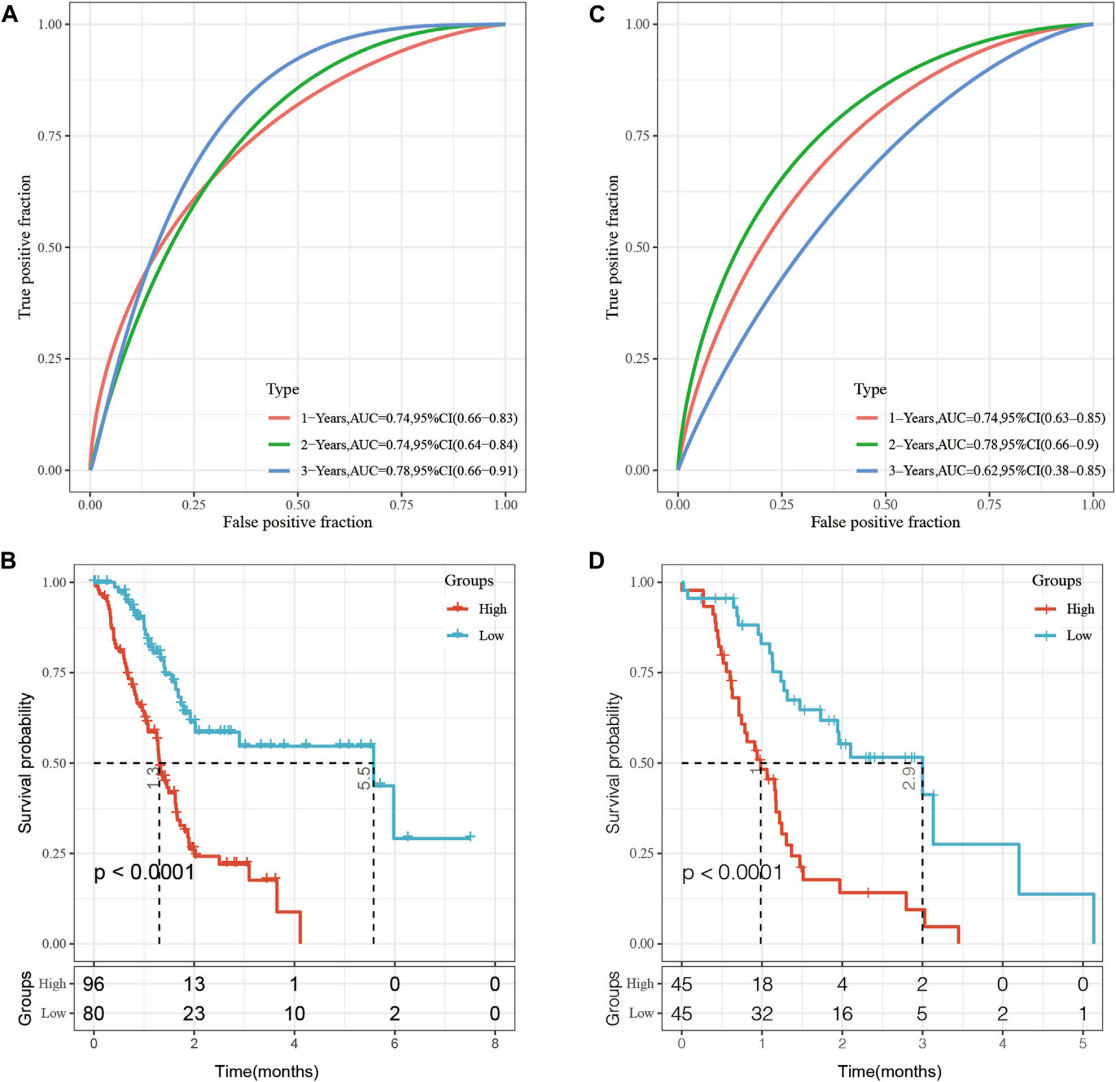
**(A)** ROC analysis of five-epi-lncRNA scores in TCGA dataset; **(B)** Prognostic differences between patients in high- and low-five-epi-lncRNA subgroups in the TCGA dataset; **(C)** ROC analysis of five-epi-lncRNA scores in ICGC-AU dataset; **(D)** Prognostic differences between patients in high- and low-five-epi-lncRNA subgroups in the ICGC dataset.

In the TCGA dataset, Receiver operating characteristic curve (ROC) analysis showed that the AUC for the five-epi-lncRNA score at 1-, 2- and 3-year was 0.74, 0.74, and 0.78, respectively, which indicated good prognostic prediction performance (Figure 7A). The prognosis of patients in the high five-epi-lncRNA RiskScore subgroup was significantly worse than those in the low five-epi-lncRNA RiskScore subgroup (Figure 7B). To evaluate the stability of five-epi-lncRNA RiskScore, the ICGC-AU data set along with prognostic information was obtained and the same method to evaluate the ROC for five-epi-lncRNA RiskScore was used. The results showed that the AUC of RiskScore was 0.74 in 1-year, 0.78 in 2-year, and 0.62 in 3-year, which indicated good prognostic prediction performance (Figure 7C). The prognosis of patients in the high five-epi-lncRNA subgroup was significantly worse than those in the low five-epi-lncRNA RiskScore subgroup (Figure 7D), consistent with the previous results.

### Risk Model and Prognostic Analysis of Clinical Characteristics

**FIGURE 8:**
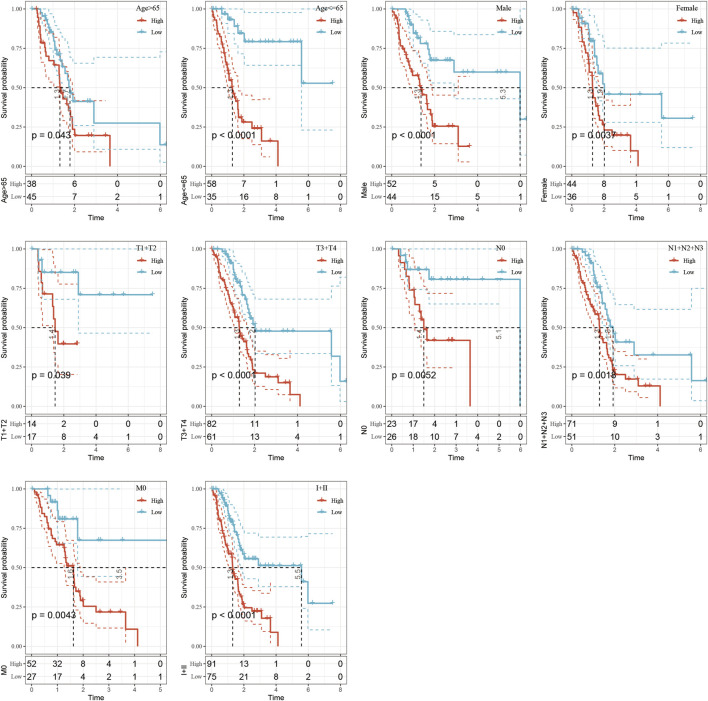
Performance of the prognostic risk model for different clinical characteristics.

The clinical subgroup survival analysis based on five-epi-lncRNA scores showed that the prognostic signature could significantly distinguish between age, gender, T stage, N0 stage, M0 stage, and stage I + II subgroups (Figures 8A–I, *p* < 0.05); this further showed that my model had good prediction ability across different clinical parameters.

### Performance of Risk Score for Different Clinical Characteristics

**FIGURE 9:**
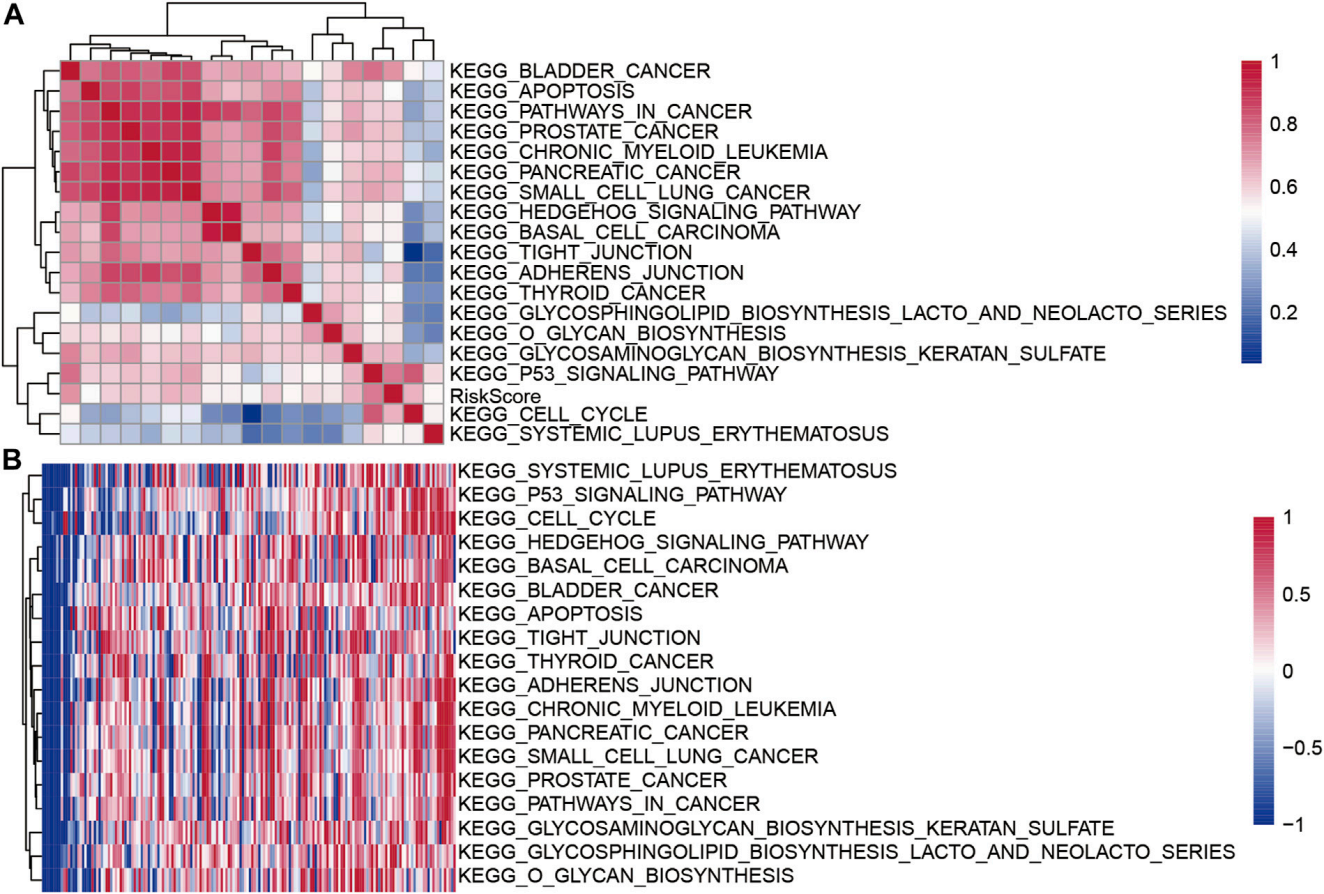
**(A)** Clustering correlation coefficients for KEGG pathways and RiskScores >0.5; **(B)** For KEGG pathways with risk score correlations >0.5, the change in the relationship of ssGSEA score for each sample with the increase in risk score was calculated. The horizontal axis represents the sample, and the risk score increases from left to right.

To evaluate the relationship between the risk scores for different samples and biological functions, the gene expression profiles corresponding to these samples were selected and the GSVA package in R software was used for ssGSEA analysis, I calculated the score for each sample for different functions, and finally selected the functions with correlation >0.5 (Figure 9A). A total of 18 pathways were positively correlated with the sample risk scores. Cluster analysis was performed according to their enrichment scores (Figure 9B). Tumor-related pathways KEGG_CELL_CYCLE, KEGG_BASAL_CELL_CARCINOMA, and KEGG_P53_SIGNALING_PATHWAY among the 18 pathways were upregulated with the increase in RiskScore.

### Univariate and Multivariate Analysis of Five-Epi-lncRNA Signature

**FIGURE 10:**
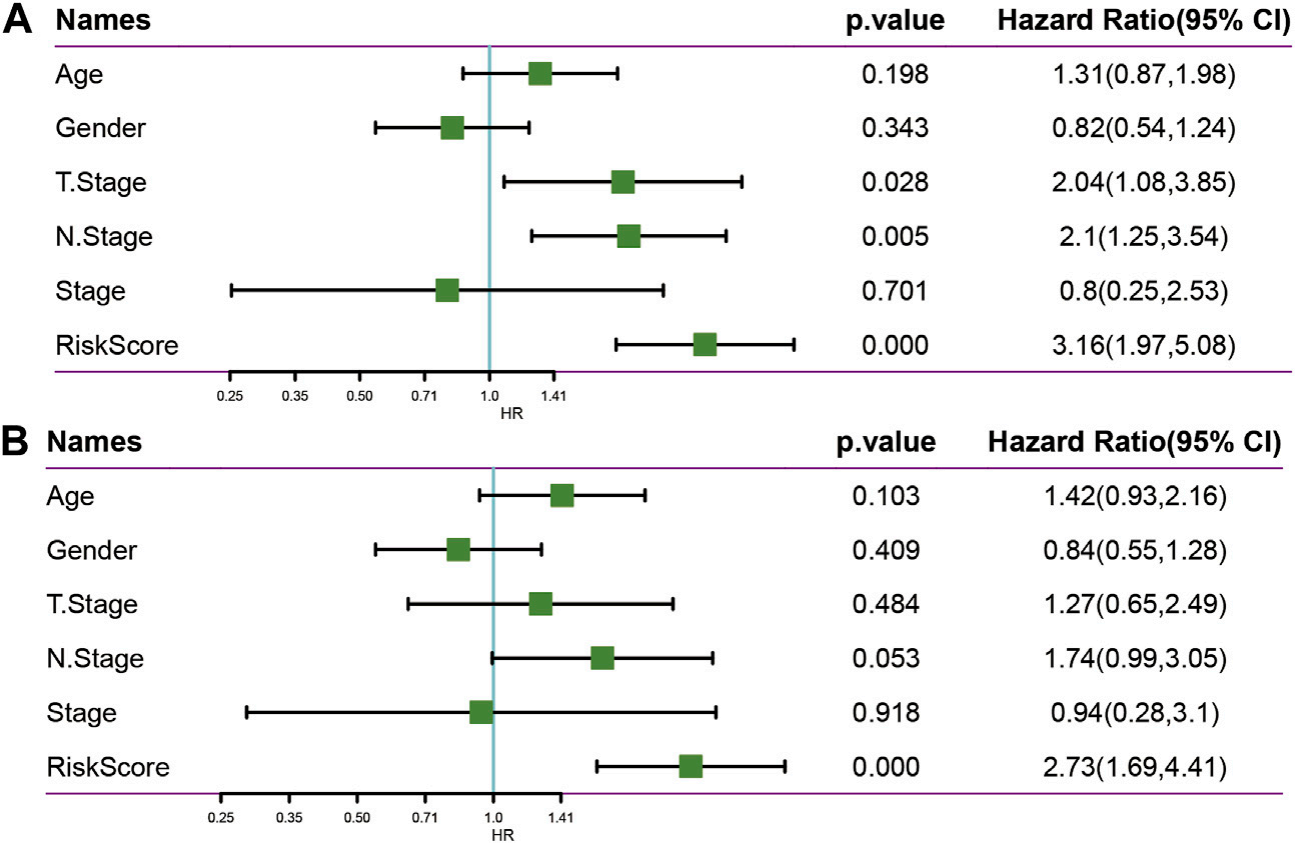
**(A)** Univariate analysis in TCGA data set; **(B)** Multivariate analysis in TCGA data set.

To identify the independence of the 5-epi-lncRNA signature model in clinical settings, the univariate and multivariate Cox regression analyses were used. The results demonstrated that the RiskScore was significantly correlated with prognosis in univariate and multivariate cox analysis (Figures 10A,B). Therefore, the 5-epi-lncRNA characteristic model is an independent risk factor for predicting prognosis of PAAD patients.

### Construction of Nomogram Based on RiskScore

**FIGURE 11:**
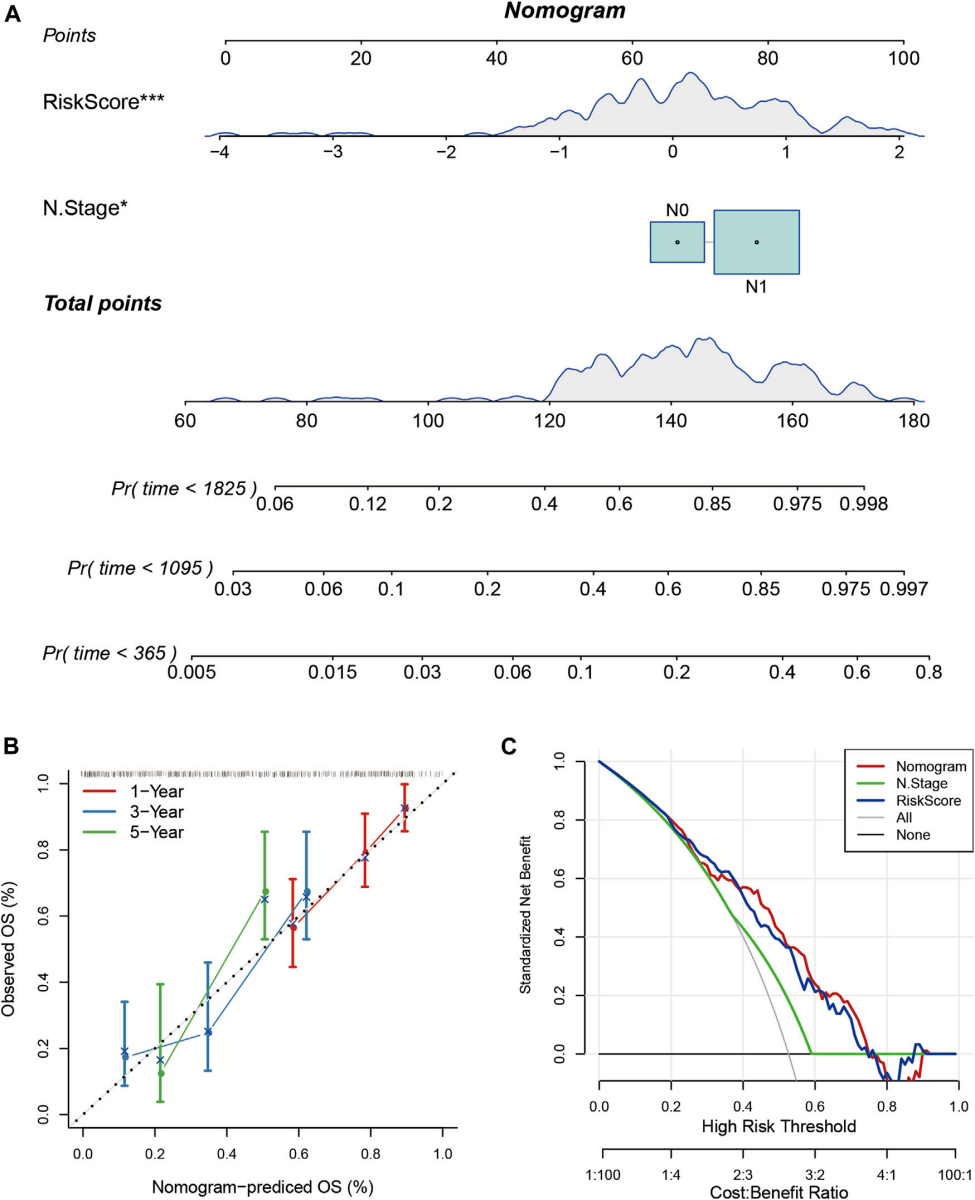
**(A)** The nomogram model based on RiskScore and N stage; **(B)** The calibration plot of the nomogram; **(C)** The decision curve analysis of the nomogram model.

Nomogram helps to display the results of the risk model intuitively and effectively. It uses the length of the straight line to represent the impact of different variables on the outcome. I combined RiskScore and N stage to build a nomogram model (Figure 11A). It showed that the RiskScore had a significantly high impact on the prognosis of patients. The calibration plot showed that my nomogram model had high accuracy (Figure 11B). The Decision Curve Analysis (DCA) curve showed that the nomogram had higher net benefit and better clinical applicability (Figure 11C).

### Expression Verification of lncRNA

**FIGURE 12:**
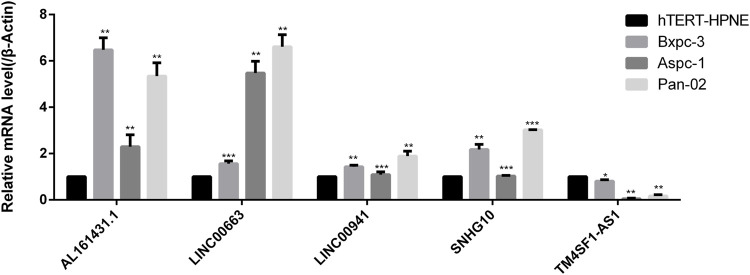
RT-PCR was used to detect the expression levels of AL161431.1, LINC00663, LINC00941, SNHG10, and TM4SF1-AS1 in normal pancreatic and pancreatic cancer samples (*p < 0.05, **p < 0.01, ***p < 0.001).

The expressions of lncRNAs in normal pancreatic and pancreatic cancer samples were detected by RT-PCR. The results showed that AL161431.1, LINC00663, LINC00941, and SNHG10 were highly expressed in pancreatic cancers samples; in particular, AL161431.1 and LINC00663 had significantly higher levels. However, the expression of TM4SF1-AS1 was lower in normal cells (Figure 12).

## Discussion

As pancreatic cancer patients do not exhibit any clinical symptoms at the early stages, detection inability and lack of early treatment lead to a low 5-year survival rate among them (Luo et al., 2019). Thus, it is important to identify potential molecular markers and therapeutic targets for pancreatic cancer.

To date, studies have reported abnormal expression of lncRNAs in several diseases. With the deepening of my understanding of their functions, their role in tumors has also been gradually uncovered. Abnormally expressed lncRNAs are key regulators of important biological functions in cancer cells (Harries, 2012). For example, linc01207 regulates ARHGAP11A and promotes the progression of non-small cell lung cancer by secreting mir-525-5p (Zhang et al., 2022); long noncoding RNA, SNHG10, promotes the malignant progression of colorectal cancer cells by targeting mir-3690 (Zhang et al., 2021a). The important functions of lncRNAs are also described in pancreatic cancer. Different lncRNAs can either inhibit or promote cancer progression. For example, lncRNA-ZNFTR inhibits pancreatic cancer cells by regulating the ATF3/ZNF24/VEGFA pathway (Li et al., 2021a). lncRNA LINC00857 acts as a competitive endogenous RNA of miR-340-5p and upregulates the expression of TGFA, thereby, enhancing the malignant behavior of pancreatic cancer cells (Li et al., 2021b). However, most of these current studies focus on the function of a particular lncRNA. There is no systematic analysis of lncRNAs and PCGs aberrations in pancreatic cancer. In this study, the 2237 epi-lncRNAs, 11855 non-epi-lncRNAs, 13518 epi-PCGs, and 6097 non-epi-PCGs differentially expressed genes in pancreatic cancer were identified by differential analysis. The abnormal frequency of lncRNAs in pancreatic cancer was much lower than that of PCGs. Further analysis of these specific epigenetically dysfunctional lncRNAs in pancreatic cancer showed that 138 epi-lncRNAs were related to known human cancers; among them, 22 were directly related to pancreatic cancer. There were significant differences in the expression of the 22 epi-lncRNAs between normal and tumor tissue samples. To further understand the potential prognostic value of imbalance in epi-lncRNAs, I analyzed the survival of 138 differentially expressed epi-lncRNAs genes; among them, 10 genes were related to survival, including AL161431.1, CAHM, DANCR, LINC00663, LINC00857, LINC00941, LINC01089, SNHG10, SOCS2-AS1, and TM4SF1-AS1. Further, AL161431.1, LINC00663, LINC00941, SNHG10, and TM4SF1-AS1 were selected as prognostic markers, and they had good prognostic prediction performance. Subgroup analysis for different clinical characteristics also showed good prognostic prediction performance, which indicated that the constructed prognostic model had good clinical applicability. Previous studies reported that AL161431.1 is highly expressed in pancreatic cancer cells and tissues and enhances tumor progression by promoting the cell cycle (Ma et al., 2021). AL161431.1 also promotes cell proliferation and migration in endometrial cancer by targeting mir-1252-5p and MAPK signaling pathways (Gu and Liu, 2020). The expression of LINC00663 in glioma tissues and cell lines is higher as compared to the normal brain tissues and human astrocytes. LINC0066312 may play a cancer-promoting role by accelerating the development and progression of glioma through the regulation of the Akt/mTOR pathway. Its high expression is associated with a low overall survival rate in glioma patients (Pan et al., 2021). Additionally, LINC00663 expression is low in DU-145 and PC3 prostate cancer, HGC-27 stomach carcinoma, CRL-1469 pancreatic carcinoma, A549 lung cancer, MCF7 breast cancer, and BCPAP thyroid cancer cell lines (Bozgeyik et al., 2016). LINC00941 promotes the progression of pancreatic cancer by regulating the Hippo pathway and promoting glycolysis in pancreatic cancer cells (Xu et al., 2021). LINC00941 promotes the progression of esophageal squamous cell carcinoma by regulating PMEPA1 expression as a competitive endogenous RNA of mir-877-3p (Zhang et al., 2021b). LINC00941 regulates VEGFA expression by adsorbing mir-877-3p and promotes the progression of non-small cell lung cancer (Ren et al., 2021). SNHG10 can promote tumor progression in colorectal cancer (Zhang et al., 2021a) and acute myoid leukemia (AML) through microRNA interaction (Xiao et al., 2021). However, as compared to non-tumor tissues, the expression of SNHG10 in non-small cell lung cancer (NSCLC) is down-regulated, and its underlying mechanism could be the regulation of miR-21 gene methylation to enhance NSCLC cell proliferation (Liang et al., 2020). TM4SF1-AS1 promotes the progression of gastric cancer (He et al., 2021) and lung cancer (Zhou et al., 2020) by activating the PI3K-Akt signaling pathway. The expression of these five lncRNAs in pancreatic cancer cells and normal pancreatic cells were obtained by RT-PCR. AL161431.1, LINC00663, LINC00941, and SNHG10 expressions in pancreatic cancer cells were relatively higher as compared to the normal cells. This was consistent with the findings of previous reports; however, the specific mechanisms promoting pancreatic cancer need further investigation.

Regarding the mode and mechanism of lncRNAs in the tumor, the current research provided theoretical support for the hypothesis that the dysregulated lncRNAs, miRNAs, and mRNAs form a regulatory ceRNA network to promote or inhibit cancer; such a mechanism also exists in pancreatic cancer (Song et al., 2016; Zhou et al., 2016; Fu et al., 2017). However, previous studies are limited to genes that are abnormally expressed, and the mechanism of their actions remains unclear. In this study, I not only detected the abnormally expressed genes in pancreatic cancer but also combined histone methylation and acetylation abnormalities in tumors, the most common modifications, for my analyses. DNA methylation is mediated by methyltransferases and plays an important role in the occurrence and development of tumors (Jaenisch and Bird, 2003; Pfeifer, 2018). Common methylation is methylation at different sites on gene promoters, while hypermethylation can inhibit gene expression (Gujar et al., 2019). Gene methylation is an important factor in tumorigenesis, but its specific underlying mechanism remains unclear (Pfeifer, 2018). Acetylation occurs on the specific lysine residues of the four histones and usually promotes gene transcription (Bauer et al., 2021; Gen et al., 2021).

In this study, I identified the differentially expressed genes and further found that the abnormal histone modifications in the lncRNAs mainly included H3K36me3, H3K27me3, H3K27ac, H3K9me3, H3K4me1, and H3K4me3. The expression of these modifications at promoter sites was significantly different between tumors and normal tissues, and these participated in several tumor-related pathways, such as in “PANCREATIC_CANCER” and “BLADDER_CANCER.” These modifications not only regulated gene expression, but also were related to RNA modifications, including m6A, m5C, and m1A RNA-related modifications. This showed that abnormal epigenetic modifications may be the initial factors of gene expression imbalance which could potentially run through multiple steps of gene expression regulation.

I further refined my knowledge of the mechanism of abnormal methylation and acetylation. Enhancer is a cis-acting element that binds to transcription factor (TF) and activates gene transcription even at long distances (Calo and Wysocka, 2013; Shlyueva et al., 2014; Nizovtseva et al., 2017). Abnormal histones modifications at gene enhancers and transcription factors (TF) can lead to abnormal gene expression. For example, if H3K27ac exists at this site, the gene is easier to be transcribed and the expression increases. However, if H3K27me3 replaces H3K27ac in this region, the gene is in a state of transcriptional inhibition and the expression is downregulated (Kemmeren et al., 2014; McDaniel et al., 2017). My results showed that for H3K4me1_enhancer,H3K4me1_promoter, H3K4me3_ enhancer, H3K4me3_promoter, H3K27ac_enhancer, H3K27ac_promoter, and H3K27me3_enhancer, the enrichment scores at enhancer in tumor samples were significantly higher as compared to the adjacent normal samples. For H3KH3K9me3_enhancer, H3KH3K9me3_promoter, H3K27me3_promoter,H3K36me3_ enhancer, and H3K36me3_promoter, the enrichment scores at promoters in adjacent samples were significantly higher as compared to the tumor samples. On the one hand, it implied that the abnormal methylation and acetylation of these enhancers and TFs were cancer-promoting or tumor-inhibiting. On the other hand, I also found that there are characteristic abnormal modification sites in tumors and normal tissues, which made us propose the above-mentioned hypothesis. Whether these modification sites can be used as therapeutic targets or site-specific modification inhibitors can be used simultaneously, needs further basic experiments and clinical data for validation.

In this study, the target lncRNA is mainly identified through bioinformatic data analysis combined with the validation of cell lines. However, given the limited laboratory conditions, it is hard for us to conduct more in-depth experiments. I will conduct a more in-depth mechanism study once the conditions are in place.

In conclusion, the pancreatic cancer-specific epi-lncRNAs through a systematic analysis of 100s of candidates lncRNAs from the TCGA database were identified. I examined the abnormal expression profiles of cancer-specific lncRNAs related to prognosis and clinicopathological parameters. Importantly, I constructed a five-epi-lncRNA signature based on the representative lncRNAs to evaluate the prognostic prediction in pancreatic cancer patients. It found that it could be used as an independent prognostic marker for PAAD. In addition, the aberrant modification methods and loci of pancreatic cancer were systematically analyzed, which indicated aberrant epigenetic modifications between tumors and normal tissues. The findings may have implications for identifying effective therapeutic targets for clinical applicability.

## Data Availability

The original contributions presented in the study are included in the article/Supplementary Material, further inquiries can be directed to the corresponding author.
