# Supplementary material for: Identification and Validation of Apparent Imbalanced Epi-lncRNAs Prognostic Model Based on Multi-Omics Data in Pancreatic Cancer
Source: Front Mol Biosci. 2022 May 12;9:860323. doi: 10.3389/fmolb.2022.860323 (PMC9133386; doi:10.3389/fmolb.2022.860323)
Supplement: Supplementary file 1 [file Table1.DOCX]

Supplementary Table 1. 16805 PCGs

logFC AveExpr t P.Value adj.P.Val B

TOMM6 -7.15011585 3.471131815 -118.8562376 4.88E-281 9.58E-277 633.9249946

XBP1 -9.053009124 4.39492012 -110.9690868 4.67E-271 4.58E-267 611.0565388

POLR2F -5.290504973 2.662699045 -110.3177488 3.34E-270 2.18E-266 609.0986439

EEF1G -8.986248599 4.898024538 -106.6514643 2.64E-265 1.29E-261 597.8670224

AC138969.1 -6.903076591 3.515903278 -103.7228946 2.81E-261 1.10E-257 588.6273489

NPIPA9 -6.923658051 3.407511064 -102.7765123 5.93E-260 1.94E-256 585.5883865

C11orf52 -4.194535248 2.182960977 -102.3036993 2.75E-259 7.71E-256 584.0601547

U2AF1 -5.403989732 2.864685641 -102.0806347 5.68E-259 1.39E-255 583.3368352

RGPD6 -4.184080038 2.041010915 -101.2913775 7.50E-258 1.63E-254 580.7654701

FAM156A -4.214557614 2.127096576 -98.62602283 5.25E-254 1.03E-250 571.939536

OVCA2 -4.422200212 2.146823933 -97.19349524 6.71E-252 1.20E-248 567.102506

AC026464.4 -4.768862506 2.366917517 -96.86368504 2.07E-251 3.38E-248 565.9793796

AC010132.3 -2.865492237 1.416028532 -94.8161057 2.44E-248 3.68E-245 558.9251817

U2AF1L5 -4.991849849 2.532432999 -94.39313325 1.07E-247 1.50E-244 557.4501986

TRAPPC5 -5.055945012 2.859032818 -94.10760217 2.92E-247 3.81E-244 556.4509972

TEN1 -4.363412456 2.322734045 -93.99450476 4.34E-247 5.32E-244 556.0544341

EIF3CL -5.216243006 2.692943049 -93.83286712 7.67E-247 8.84E-244 555.486895

TREX1 -4.354969167 2.114185605 -93.0866735 1.07E-245 1.17E-242 552.8549702

AC011462.1 -4.20564252 2.073842266 -92.8580847 2.42E-245 2.49E-242 552.0447635

AP003419.1 -5.494729441 2.764145521 -89.87672991 1.14E-240 1.12E-237 541.3040271

CMC4 -3.477977027 1.688436514 -89.47798636 4.94E-240 4.62E-237 539.8424713

CBWD3 -4.226683551 2.1947891 -89.21682321 1.29E-239 1.15E-236 538.8819273

PTRH1 -3.908216387 2.020926132 -89.06985217 2.23E-239 1.90E-236 538.3402296

SLX1A -4.903155846 2.418958182 -88.66459864 9.99E-239 8.16E-236 536.8422701

NPIPB5 -6.20452692 3.488467121 -88.60047887 1.27E-238 9.94E-236 536.6046805

ZNF410 -3.4902774 2.446614482 -88.07688075 8.90E-238 6.72E-235 534.6585652

SERF1B -4.142274875 2.380724652 -87.76831337 2.82E-237 2.05E-234 533.5066651

ATRIP -2.183242804 1.138585404 -87.35199586 1.35E-236 9.44E-234 531.9465869

ANKHD1 -4.783363145 3.360198656 -87.14481852 2.94E-236 1.99E-233 531.1676671

TOP3B -3.619589586 1.945551859 -86.85707259 8.72E-236 5.70E-233 530.082998

NPIPB12 -5.375955529 2.736140582 -86.02692492 2.04E-234 1.29E-231 526.9350717

EIF3C -6.568086062 4.64637798 -85.79369491 4.97E-234 3.05E-231 526.0456318

ISY1-RAB43 -2.801653958 1.379186526 -85.42762138 2.02E-233 1.20E-230 524.6450914

AGAP5 -3.424283039 1.894684285 -84.07219338 3.82E-231 2.21E-228 519.4110289

AC074143.1 -4.007038764 1.946658355 -83.99992598 5.07E-231 2.84E-228 519.1297979

AP003108.2 -3.694466953 2.064227419 -83.94380085 6.31E-231 3.44E-228 518.9112323

ACAD11 -4.205997015 2.301808636 -82.96066235 2.98E-229 1.58E-226 515.0608072

NPIPA7 -4.393259397 2.147957866 -82.52498896 1.67E-228 8.61E-226 513.3411675

ARL6IP4 -6.088176697 4.39569365 -82.32316075 3.72E-228 1.87E-225 512.5417266

CBWD5 -4.433911115 2.869433276 -82.31112884 3.90E-228 1.91E-225 512.4940118

INO80B-WBP1 -3.708351075 1.832252539 -82.1960831 6.16E-228 2.95E-225 512.0374555

NPIPB4 -4.820999034 2.873496363 -81.63513109 5.77E-227 2.69E-224 509.8029606

C1QTNF5 -4.014861633 1.949075262 -80.97970255 8.02E-226 3.66E-223 507.174368

NPIPA1 -5.452795851 3.588192732 -80.5685701 4.22E-225 1.88E-222 505.5156447

SNX15 -2.997047931 1.990007255 -80.2078454 1.82E-224 7.95E-222 504.0539577

MATR3 -3.478486724 2.391647541 -80.03322621 3.71E-224 1.58E-221 503.344245

POLR2J3 -5.085825943 3.119122737 -79.60140087 2.16E-223 9.03E-221 501.5831193

SEC16B -3.214290376 1.757485507 -78.83189182 5.11E-222 2.09E-219 498.4232862

MEMO1 -3.16666206 2.485864282 -78.79908731 5.86E-222 2.34E-219 498.287963

NDST2 -3.206804809 2.563509528 -78.36699457 3.50E-221 1.37E-218 496.5007619

ANKHD1-EIF4EBP3 -3.194487473 2.34927834 -78.07062454 1.20E-220 4.62E-218 495.2697885

NPIPB3 -4.956876155 2.988317597 -77.49346158 1.34E-219 5.05E-217 492.8604222

PLSCR3 -3.366819829 2.306624841 -77.30125451 3.00E-219 1.11E-216 492.0544722

GTF2H2C -3.139097716 2.190251124 -76.93471287 1.40E-218 5.10E-216 490.5125133

C17orf49 -4.198318629 3.308984783 -76.76236788 2.91E-218 1.04E-215 489.7852167

ZNRF3 -1.861564637 0.966603382 -76.62884134 5.12E-218 1.79E-215 489.2207268

DDX47 -4.034319822 2.927842594 -76.37093341 1.53E-217 5.26E-215 488.1279087

ARPIN-AP3S2 -3.742626947 2.069268085 -76.30962154 1.98E-217 6.70E-215 487.8676293

ADIRF -5.192298183 2.522910686 -76.12945304 4.27E-217 1.42E-214 487.1016982

PIK3R2 -3.365111514 2.198633229 -76.03801239 6.30E-217 2.06E-214 486.7123457

NACA -2.565630186 8.107507514 -75.4998638 6.29E-216 2.02E-213 484.4124016

SCLY -3.614549554 2.392231965 -75.29215865 1.54E-215 4.86E-213 483.5207914

RTEL1 -2.807295136 1.831272455 -75.17267707 2.57E-215 8.00E-213 483.0069011

OGFOD2 -3.702978145 2.605351515 -75.1105021 3.36E-215 1.03E-212 482.7391983

CIDEB -3.539302205 2.180744639 -75.07288766 3.95E-215 1.19E-212 482.5771483

EIF4A1 -6.169496931 4.942878176 -75.04884954 4.38E-215 1.30E-212 482.4735496

SARNP -3.759356142 3.066203524 -74.35732137 8.76E-214 2.56E-211 479.4804957

JMJD7 -3.445110764 1.882240039 -74.33528045 9.64E-214 2.78E-211 479.384692

ZNF23 -2.597322327 1.688579024 -74.24263237 1.44E-213 4.10E-211 478.9817086

BBS1 -3.742171214 2.686335509 -74.05885507 3.21E-213 9.00E-211 478.1810238

WDR73 -3.168052003 2.639129377 -73.99171637 4.31E-213 1.19E-210 477.8880721

DNLZ -4.113873678 2.176052775 -73.23486251 1.19E-211 3.25E-209 474.5692082

CBS -6.704711853 3.446122521 -73.11857727 1.99E-211 5.35E-209 474.0565961

RBM34 -3.48990996 2.81146034 -72.50926855 2.96E-210 7.85E-208 471.3587712

FAM156B -3.732207881 1.818489864 -72.50133102 3.07E-210 8.02E-208 471.3234942

GOLGA8N -4.098305784 2.475956859 -72.14690689 1.49E-209 3.84E-207 469.7448345

NOTCH2NLA -3.168260176 1.643393742 -71.94556802 3.66E-209 9.33E-207 468.8449898

CBWD1 -3.851500725 2.903400266 -71.90734828 4.35E-209 1.09E-206 468.6739235

KREMEN1 -2.027420585 0.984241962 -71.05288694 2.04E-207 5.06E-205 464.8284156

SPSB3 -5.019128247 3.71847606 -70.74738142 8.14E-207 2.00E-204 463.4436163

CCDC180 -2.667829143 1.469079493 -70.62722358 1.41E-206 3.40E-204 462.897525

COMMD3-BMI1 -3.956604568 1.940396548 -70.58307319 1.72E-206 4.11E-204 462.6966664

MTCP1 -3.549376696 2.616283633 -70.46712552 2.91E-206 6.88E-204 462.1686469

ALG9 -3.26719659 3.072879706 -69.80995163 5.90E-205 1.38E-202 459.1614564

GTF2IRD2 -3.321356314 2.534190139 -69.00190071 2.47E-203 5.69E-201 455.4297697

BOLA2 -2.487225331 1.207461127 -68.92265851 3.56E-203 8.12E-201 455.0617704

PLA2G4B -3.385531531 1.962850847 -68.850364 4.99E-203 1.12E-200 454.7257146

AC011511.4 -2.879946502 1.426936997 -68.62844877 1.40E-202 3.12E-200 453.6922364

ARL17A -2.937337111 1.671698489 -68.62196082 1.44E-202 3.18E-200 453.6619777

GOLGA6L4 -2.487414565 1.267071448 -68.11591978 1.54E-201 3.36E-199 451.2941996

GTF2IRD2B -3.315842622 3.094541757 -67.45814819 3.43E-200 7.39E-198 448.1935614

RGPD5 -3.110919239 1.518087878 -67.37786152 5.01E-200 1.07E-197 447.8133116

C2orf92 -3.359953912 2.218454642 -66.92584724 4.30E-199 9.06E-197 445.6651682

CTRL -9.283052641 4.990411566 -66.74251851 1.03E-198 2.15E-196 444.7903497

RASA4 -4.109349709 2.325584638 -66.50533743 3.21E-198 6.62E-196 443.6554773

AARSD1 -3.721301309 3.681947671 -66.14860568 1.78E-197 3.64E-195 441.9419964

CNBD2 -2.579082172 1.344767322 -65.79952769 9.59E-197 1.94E-194 440.257573

RASA4B -3.713674017 1.881693406 -65.7977004 9.67E-197 1.94E-194 440.2487355

ZBTB9 -1.844386381 0.922404492 -65.49286166 4.24E-196 8.40E-194 438.7714633

GET4 -3.765299785 3.419757726 -65.14706784 2.28E-195 4.47E-193 437.0885724

BOLA2B -4.023269316 2.888615554 -64.90155869 7.57E-195 1.47E-192 435.8890976

HTD2 -1.947628006 1.012575642 -64.59651435 3.38E-194 6.49E-192 434.3933439

NAIP -2.895241366 1.623610209 -64.54177035 4.42E-194 8.42E-192 434.1242749

CBWD2 -3.043607791 3.16304011 -64.4968228 5.51E-194 1.04E-191 433.9032103

NOMO3 -4.823675397 3.204824089 -64.47848194 6.03E-194 1.13E-191 433.8129671

RBM4 -3.721234928 4.4691789 -64.35443985 1.11E-193 2.06E-191 433.2020636

CYB5D1 -2.7090995 2.163751406 -64.16528093 2.83E-193 5.18E-191 432.2685285

NOMO2 -5.385750779 4.649672873 -64.13056361 3.36E-193 6.09E-191 432.0969377

NBPF10 -1.990320653 1.310779981 -63.90491133 1.03E-192 1.85E-190 430.979719

UBE2V1 -3.739433597 4.008890505 -63.6733841 3.24E-192 5.77E-190 429.8299241

HNRNPUL2-BSCL2 -3.368332016 2.237487192 -63.46239196 9.26E-192 1.64E-189 428.7790156

MAGED4B -3.672472442 1.794173672 -63.35113615 1.61E-191 2.82E-189 428.2236802

DNAJC25-GNG10 -2.357892077 1.157332319 -63.28232539 2.27E-191 3.95E-189 427.8797965

SULT1A3 -3.670062344 1.856862215 -62.85956958 1.89E-190 3.26E-188 425.7600922

GPS2 -3.988988639 3.753491465 -62.78965146 2.69E-190 4.59E-188 425.4083612

SPNS1 -3.023789895 2.728166666 -62.34348027 2.56E-189 4.33E-187 423.1560322

AQP12B -7.494202587 4.105460776 -61.8728725 2.79E-188 4.68E-186 420.7655874

RPL17 -5.991157357 7.523463903 -61.56765176 1.33E-187 2.20E-185 419.2070455

TTC4 -2.236184664 2.33106691 -61.54500725 1.49E-187 2.45E-185 419.0911584

DNAH14 -2.644615601 1.913224127 -61.50361542 1.84E-187 3.01E-185 418.8792362

RNASEK -4.417206876 5.207894284 -61.43818952 2.57E-187 4.17E-185 418.5440179

ASB3 -2.035901884 1.844750789 -61.34811549 4.08E-187 6.57E-185 418.0820223

C18orf32 -3.444186378 3.318183652 -61.12375158 1.29E-186 2.06E-184 416.9287723

ZSCAN32 -2.108028035 1.869135378 -61.06430937 1.76E-186 2.78E-184 416.6226411

CCZ1B -3.388247679 3.182919558 -60.95516298 3.08E-186 4.84E-184 416.0598812

MIA2 -3.318338533 3.152497881 -60.91445965 3.80E-186 5.92E-184 415.8497989

SMIM11B -3.6822785 1.792495913 -60.86796494 4.84E-186 7.47E-184 415.6096822

CBWD6 -3.149814142 1.995670437 -60.68667631 1.23E-185 1.89E-183 414.6719731

MTMR3 -2.65999484 2.783444487 -60.45540766 4.10E-185 6.23E-183 413.472349

ANKRD36B -2.604012588 1.796837041 -60.41585009 5.03E-185 7.59E-183 413.2667755

DIABLO -3.002013523 3.290090259 -59.98640308 4.72E-184 7.06E-182 411.0277842

CHMP4A -3.605438196 3.839021489 -59.7636367 1.51E-183 2.25E-181 409.8611089

NHEJ1 -2.351786413 1.739423876 -59.31897601 1.57E-182 2.32E-180 407.5215139

GPR89A -2.851746337 2.624379583 -59.13847503 4.07E-182 5.96E-180 406.567664

STIMATE -2.679251411 2.27557071 -59.01986504 7.63E-182 1.11E-179 405.9395654

TICAM2 -1.780640002 1.024564903 -58.85630124 1.82E-181 2.62E-179 405.0717049

GLS2 -4.984605596 2.802964739 -58.26527211 4.24E-180 6.08E-178 401.9191044

FAM47E-STBD1 -2.226552728 1.137811793 -58.00946892 1.67E-179 2.38E-177 400.5464835

AGAP4 -2.826984822 2.417858835 -57.88042134 3.35E-179 4.73E-177 399.8521419

AC027796.3 -2.37566871 1.206868561 -57.6437128 1.20E-178 1.68E-176 398.5752323

ZACN -3.174945396 1.76622011 -57.36388333 5.46E-178 7.59E-176 397.0601762

DTWD1 -3.027592211 3.341305316 -57.1108411 2.16E-177 2.98E-175 395.6849579

CAPN3 -2.793660783 1.99210593 -57.09348728 2.37E-177 3.25E-175 395.5904629

MRPL53 -3.565327206 4.011436164 -56.92859913 5.82E-177 7.93E-175 394.6914473

AS3MT -2.905767628 1.616988123 -56.57473736 4.03E-176 5.46E-174 392.7549374

ANKRD36 -2.403277992 1.881021121 -56.47467979 6.99E-176 9.39E-174 392.2055921

STRADA -2.594219099 3.074218654 -56.41878308 9.50E-176 1.27E-173 391.8983597

CCZ1 -3.150912274 3.228163449 -56.3503233 1.38E-175 1.83E-173 391.5217398

ARF6 2.647334359 5.142237926 56.10602882 5.32E-175 7.00E-173 390.1747737

CELF6 -2.066213495 1.026594199 -56.01663479 8.71E-175 1.14E-172 389.6806988

LAT -2.643376936 1.694235949 -55.78915304 3.07E-174 3.99E-172 388.4205495

TXNDC5 -4.63492327 4.343418873 -55.75546466 3.70E-174 4.77E-172 388.2335791

GOLGA8R -2.083788955 1.208777484 -55.66941372 5.97E-174 7.65E-172 387.7555835

PRPF40B -2.998317475 3.013349144 -55.56904765 1.04E-173 1.33E-171 387.1973193

ABHD16A -2.910063338 3.544705815 -55.55202047 1.15E-173 1.45E-171 387.102529

AL049839.2 -8.651745114 5.226244555 -55.52985402 1.30E-173 1.63E-171 386.9790934

ADO 1.812246852 3.299999775 55.50980952 1.45E-173 1.81E-171 386.86744

MISP 5.599985189 3.637173585 55.50293803 1.51E-173 1.87E-171 386.8291564

GPR89B -2.759253638 2.519358248 -55.32808443 3.99E-173 4.92E-171 385.8537011

AL662899.2 -2.499425114 1.273827876 -55.30862737 4.45E-173 5.45E-171 385.7450033

PLIN5 -5.477952873 3.830777055 -55.25527227 5.99E-173 7.30E-171 385.4467749

CMC2 -2.904158458 3.586186408 -55.24670164 6.29E-173 7.61E-171 385.3988479

AC011498.4 -3.475957278 2.063678471 -55.09399809 1.48E-172 1.78E-170 384.5439281

NBPF9 -2.364161875 2.521763665 -55.00050263 2.50E-172 2.98E-170 384.0195519

TTC7B -3.141850504 2.899916024 -54.87855354 4.95E-172 5.88E-170 383.3345192

WDR27 -3.021240288 3.266024324 -54.8508605 5.78E-172 6.83E-170 383.1787879

BLOC1S5-TXNDC5 -3.321363686 1.808873904 -54.83048257 6.48E-172 7.62E-170 383.0641528

FMC1-LUC7L2 -3.530130348 1.735750193 -54.70666568 1.30E-171 1.52E-169 382.3668949

IPO4 -3.264917514 3.225712659 -54.68487286 1.47E-171 1.71E-169 382.2440413

PYURF -3.318666497 3.673470812 -54.54055504 3.32E-171 3.83E-169 381.429486

SARM1 -2.776099862 2.758442907 -54.44393567 5.73E-171 6.58E-169 380.8831892

NBPF19 -2.240391305 1.853389426 -54.36515085 8.95E-171 1.02E-168 380.4371596

PSMD9 -2.91387519 3.913986904 -54.30448121 1.26E-170 1.43E-168 380.0933362

SH3KBP1 2.767686566 3.450352215 54.23822837 1.84E-170 2.07E-168 379.7175231

COX20 -2.776629203 3.207144344 -54.23169772 1.91E-170 2.14E-168 379.6804589

GSTM2 -4.780625487 4.233395422 -54.21166326 2.14E-170 2.38E-168 379.5667326

RGPD8 -1.738457347 1.178087467 -54.12686186 3.46E-170 3.83E-168 379.0849846

IFI30 -4.233248326 3.26917987 -54.11649739 3.67E-170 4.04E-168 379.0260641

AC022400.5 -1.213904444 0.676250581 -54.05448171 5.22E-170 5.72E-168 378.6733265

TUBA8 -1.931524203 1.109943362 -54.02995441 6.00E-170 6.54E-168 378.5337296

PSMA2 -3.258380778 4.415470546 -53.88866455 1.34E-169 1.45E-167 377.7286005

NBPF20 -1.290652934 0.908593878 -53.79865766 2.24E-169 2.42E-167 377.2148313

UPK3BL1 -4.604351841 2.75034384 -53.76998684 2.64E-169 2.83E-167 377.0510322

KCNMB3 -1.857191226 1.442699034 -53.6405547 5.53E-169 5.90E-167 376.3107137

AQP12A -6.609467955 3.636012702 -53.60800659 6.66E-169 7.07E-167 376.1243247

NUDT4B -2.163139568 1.063857116 -53.49759384 1.25E-168 1.32E-166 375.4913722

RAP2B 2.609803296 3.300214773 53.23254857 5.75E-168 6.03E-166 373.9677572

CMC1 -2.625781653 3.139761251 -53.22356093 6.05E-168 6.32E-166 373.915987

NBPF14 -2.64572135 2.341214115 -53.20968317 6.56E-168 6.81E-166 373.8360354

SDR39U1 -3.443020913 4.226855696 -53.2085275 6.60E-168 6.82E-166 373.8293768

BRSK2 -5.534026485 3.674678423 -53.16526722 8.47E-168 8.70E-166 373.5800392

CHKB -3.670700571 3.904050972 -52.611949 2.08E-166 2.12E-164 370.3767638

NPHP3 -2.627345674 2.779762846 -52.37537422 8.24E-166 8.37E-164 368.9991257

PSMC1 -3.128495451 4.207052775 -52.34966451 9.57E-166 9.68E-164 368.8491186

ZNF280D -2.333316966 3.426865875 -52.33380674 1.05E-165 1.06E-163 368.7565654

FGFR1OP -1.928117377 2.341604993 -52.26246658 1.59E-165 1.59E-163 368.3399207

CEMP1 -2.028593186 0.984811219 -52.23894374 1.83E-165 1.82E-163 368.2024441

CAMK2N1 3.929315162 4.938036108 52.20447761 2.23E-165 2.21E-163 368.0009235

LUC7L2 -2.056978895 4.697033242 -52.10852011 3.91E-165 3.86E-163 367.4393213

RABIF 1.87591558 2.826129432 52.07623644 4.73E-165 4.64E-163 367.2501964

KLC1 -3.23465862 4.12481118 -52.05921112 5.23E-165 5.10E-163 367.1504215

TUT4 -2.851561979 3.394775524 -51.88541957 1.45E-164 1.41E-162 366.1304823

AC010422.3 -3.099363117 2.416206762 -51.8644704 1.64E-164 1.58E-162 366.0073576

ELF4 2.858751104 3.142798239 51.83316622 1.97E-164 1.89E-162 365.8233013

GSS 2.321017636 4.823527687 51.74783672 3.25E-164 3.11E-162 365.3211588

GOLGA8B -5.42460539 5.195696636 -51.71994362 3.83E-164 3.65E-162 365.1568757

CDK3 -2.67667556 2.057043323 -51.63446081 6.34E-164 6.00E-162 364.6529757

GATD3B -4.388823846 2.946187189 -51.58528825 8.47E-164 7.99E-162 364.3628227

BEGAIN -3.62594417 2.654207811 -51.53404915 1.15E-163 1.08E-161 364.0602477

EML5 -1.534979982 0.898684011 -51.52050435 1.24E-163 1.16E-161 363.9802247

CHKB-CPT1B -2.542753002 1.887109216 -51.46089722 1.76E-163 1.64E-161 363.6278702

ZNF337 -2.29601152 2.381190097 -51.35839931 3.24E-163 2.99E-161 363.0212377

RILPL2 1.900661854 2.43298709 51.32220305 4.01E-163 3.69E-161 362.8067871

ZC3H14 -2.698023806 3.552467977 -51.25446654 5.99E-163 5.49E-161 362.405157

STIMATE-MUSTN1 -2.079674405 1.22564704 -51.19767672 8.39E-163 7.65E-161 362.0681175

HAUS7 -2.526014974 2.409671807 -51.17968842 9.33E-163 8.47E-161 361.9612993

CYB5A -4.410677993 5.680821979 -51.14680556 1.13E-162 1.03E-160 361.7659592

NUTM2D -1.949209966 1.244263134 -51.13678091 1.20E-162 1.08E-160 361.7063888

GTF2H4 -2.491694269 2.850139224 -51.103412 1.47E-162 1.31E-160 361.5080325

PPT2-EGFL8 -1.9663216 1.429949939 -51.08150149 1.67E-162 1.49E-160 361.3777345

PRORP -1.971181153 2.166841381 -51.0477698 2.04E-162 1.81E-160 361.1770539

MRPL38 -3.582694801 3.969122248 -51.02471764 2.34E-162 2.07E-160 361.0398505

WDR92 -1.477357012 1.439249487 -50.88645249 5.34E-162 4.69E-160 360.2159119

CPT1B -3.550865728 2.966207777 -50.86858823 5.94E-162 5.20E-160 360.1093311

NPIPB13 -2.43524502 1.313872214 -50.82856885 7.54E-162 6.57E-160 359.8704651

ARPC4-TTLL3 -2.342031122 1.441062092 -50.79061101 9.45E-162 8.20E-160 359.6437704

CDRT4 -3.270501379 2.112293513 -50.7568816 1.16E-161 9.99E-160 359.4422198

A1BG -3.564973806 1.947669596 -50.7540358 1.18E-161 1.01E-159 359.42521

MTPN 2.483107294 5.193620214 50.75025648 1.20E-161 1.03E-159 359.4026192

GTF2H2 -2.609906083 1.768013636 -50.74503635 1.24E-161 1.06E-159 359.3714139

SPDYE16 -0.87700417 0.451016084 -50.69598967 1.66E-161 1.41E-159 359.0780986

ZNF493 -2.155838456 2.227784909 -50.63360997 2.42E-161 2.04E-159 358.7047328

PLGLB1 -2.198983945 1.185115443 -50.44457789 7.50E-161 6.31E-159 357.5711484

VPS18 2.029899298 3.552663 50.44222871 7.60E-161 6.37E-159 357.5570404

ECHDC2 -4.256280836 5.575197503 -50.42863382 8.25E-161 6.89E-159 357.4753866

ZNF564 -1.495915396 1.249950292 -50.42526928 8.42E-161 7.00E-159 357.4551759

BRF1 -2.525849495 3.019748628 -50.39480028 1.01E-160 8.37E-159 357.2721021

SRXN1 -2.64998081 2.251199154 -50.32488486 1.54E-160 1.27E-158 356.8516932

CDK11A -3.495969632 3.720372821 -50.30446342 1.74E-160 1.43E-158 356.7288127

NPIPA3 -2.611621079 1.527212835 -50.22440158 2.82E-160 2.30E-158 356.2466944

PNMA1 3.11000026 4.253866322 49.97178287 1.29E-159 1.05E-157 354.7216158

ITIH4 -5.3554199 3.125445636 -49.84236716 2.83E-159 2.29E-157 353.938048

RABGEF1 -2.312833884 2.640388093 -49.82397465 3.16E-159 2.55E-157 353.8265622

ACADL -4.173282686 2.907197151 -49.78726513 3.95E-159 3.17E-157 353.6039547

RPL36A -4.626657038 7.290131977 -49.67236762 7.92E-159 6.34E-157 352.9064065

SLC2A11 -2.964434191 3.178717591 -49.59872999 1.24E-158 9.88E-157 352.4587052

PSMA6 -3.141828825 4.4934176 -49.53437908 1.83E-158 1.46E-156 352.0670525

EEF1D -4.192543239 7.023160022 -49.52270236 1.97E-158 1.56E-156 351.9959444

EFNB2 3.355810846 3.757126948 49.51525785 2.06E-158 1.62E-156 351.9506026

APEX2 1.768275089 3.718453962 49.48617287 2.46E-158 1.93E-156 351.7734073

MKNK1 -4.229017982 5.232699278 -49.45633394 2.95E-158 2.30E-156 351.5915367

FUNDC2 -3.476354202 5.346884015 -49.25489561 1.01E-157 7.84E-156 350.361579

ILK -3.395727706 4.91848344 -49.19337705 1.47E-157 1.14E-155 349.9851971

JMJD7-PLA2G4B -2.440913439 2.279838485 -49.14492598 1.97E-157 1.52E-155 349.6885146

FP565260.6 -1.709417231 1.673858809 -49.11324741 2.40E-157 1.84E-155 349.4944165

SMN1 -2.600909577 3.062739482 -49.09195912 2.73E-157 2.09E-155 349.3639279

UBQLN2 1.927980728 3.938592946 48.96192401 6.06E-157 4.62E-155 348.5659385

STX6 1.845926069 3.189932248 48.78107548 1.84E-156 1.40E-154 347.4534675

RNF19B 2.463591353 4.193292421 48.71784263 2.72E-156 2.06E-154 347.0637664

BAG5 1.665473687 3.705299257 48.68207683 3.39E-156 2.56E-154 346.8431755

H3F3A -3.526248393 6.002309549 -48.63876747 4.42E-156 3.32E-154 346.5758959

RNPC3 -3.038295634 3.564282358 -48.63815278 4.44E-156 3.32E-154 346.5721011

ARHGAP8 -2.901961921 2.36186685 -48.55499397 7.42E-156 5.53E-154 346.0583893

FAM204A -2.226231076 3.504923679 -48.48413363 1.15E-155 8.54E-154 345.6201311

RYBP 1.814132363 3.154984526 48.47682357 1.20E-155 8.90E-154 345.5748925

PDXP -2.691385694 1.931688667 -48.46024846 1.33E-155 9.82E-154 345.4722978

BUB1B-PAK6 -1.796028118 0.880639313 -48.43480575 1.56E-155 1.15E-153 345.3147644

NAGA 2.103217378 4.1255001 48.41939053 1.72E-155 1.26E-153 345.219288

HEMK1 -2.422018488 3.023001275 -48.296593 3.67E-155 2.68E-153 344.4579122

TBC1D3B -2.026627654 1.082195756 -48.28486327 3.95E-155 2.87E-153 344.3851093

B4GALT5 2.833434669 4.572521906 48.26068383 4.59E-155 3.32E-153 344.2349932

NDUFA7 -3.066302673 3.660026604 -48.24008939 5.21E-155 3.76E-153 344.1070901

BORCS8-MEF2B -1.885371904 1.573610041 -48.1672528 8.19E-155 5.89E-153 343.6544068

NPIPB2 -2.74768083 1.797776741 -48.04896244 1.71E-154 1.22E-152 342.9181387

EDRF1 -2.20041305 3.338833039 -47.95163536 3.13E-154 2.24E-152 342.3113395

BCKDHA -2.665258202 3.13515538 -47.92432201 3.72E-154 2.64E-152 342.1408862

RPS29 -4.673533149 8.244473849 -47.91486068 3.94E-154 2.79E-152 342.0818244

USP15 -2.403224069 3.724275008 -47.88757755 4.67E-154 3.30E-152 341.9114628

FRMD4A -2.598707453 2.992601737 -47.85190636 5.84E-154 4.10E-152 341.6886155

KRTCAP2 -3.897320336 5.644709629 -47.77285145 9.57E-154 6.70E-152 341.1942994

NBDY 2.14317918 4.631133371 47.66127151 1.92E-153 1.34E-151 340.4955777

SLC46A1 -2.851975615 2.853613401 -47.50355673 5.17E-153 3.59E-151 339.5058891

MYEF2 -3.28363233 2.976324315 -47.47627168 6.13E-153 4.25E-151 339.3344243

RIC3 -3.745449546 2.92814869 -47.45569453 6.98E-153 4.82E-151 339.2050651

SETD6 -2.502858135 3.736190294 -47.35972391 1.28E-152 8.78E-151 338.6011945

PSMD6 -3.86216556 5.581178498 -47.29690937 1.89E-152 1.30E-150 338.2054615

SKAP2 2.487644367 3.51989028 47.29616763 1.90E-152 1.30E-150 338.2007862

ARHGDIG -5.39281633 3.762150625 -47.10839394 6.22E-152 4.24E-150 337.0154829

METTL22 -2.263566516 3.023174876 -47.10235231 6.46E-152 4.39E-150 336.977288

ST7L -1.900013281 2.638153431 -47.09995316 6.56E-152 4.44E-150 336.9621197

OSTF1 2.218658449 5.066325139 47.00764197 1.18E-151 7.93E-150 336.378064

TRIM14 2.552611315 2.964961818 46.98903046 1.32E-151 8.89E-150 336.2602065

TRIM34 -1.38217879 1.201775511 -46.97565838 1.44E-151 9.64E-150 336.1755065

COA1 -2.29414298 3.863958926 -46.93285264 1.89E-151 1.26E-149 335.9042523

ANKRD23 -1.701603851 1.376044301 -46.89504677 2.40E-151 1.59E-149 335.6645308

GPKOW 1.690326484 4.236070894 46.8163966 3.95E-151 2.62E-149 335.1653677

TTLL3 -2.837339151 3.773615735 -46.80656885 4.20E-151 2.78E-149 335.1029515

GOLGA6L9 -2.038280122 1.863936957 -46.76388629 5.51E-151 3.63E-149 334.8317628

ANP32E 2.128327538 4.038915917 46.75569492 5.81E-151 3.81E-149 334.7796972

HYI -3.321506643 4.850151303 -46.73980488 6.42E-151 4.20E-149 334.6786787

TM9SF1 -2.768508506 3.926066231 -46.71854195 7.35E-151 4.79E-149 334.5434637

ITGA2 3.965278115 3.070697496 46.65961576 1.07E-150 6.94E-149 334.1685058

ZNFX1 1.925351196 3.636926729 46.64646787 1.16E-150 7.52E-149 334.0847963

FNBP4 -1.88481289 5.134133413 -46.62288575 1.35E-150 8.71E-149 333.9346112

ZNF177 -1.624461079 0.809243794 -46.57229844 1.86E-150 1.20E-148 333.6122539

ZNF333 -1.860879017 2.417442632 -46.5706776 1.88E-150 1.21E-148 333.6019213

GPR146 -2.120570049 1.826569321 -46.5487224 2.17E-150 1.38E-148 333.4619333

MTERF4 -2.186933688 3.66511433 -46.5365853 2.34E-150 1.49E-148 333.3845255

KSR1 -4.373857689 4.658857669 -46.52496089 2.52E-150 1.60E-148 333.3103738

TRIM73 -2.523050945 1.321686638 -46.48266959 3.30E-150 2.09E-148 333.0404852

GPR137B 2.530514073 3.231031275 46.47753297 3.41E-150 2.15E-148 333.0076928

RAP1B -2.793797626 4.065317824 -46.4646876 3.70E-150 2.33E-148 332.9256761

EMB 3.145681623 2.919865198 46.42550086 4.75E-150 2.98E-148 332.6753695

OPN1SW 2.265771243 1.329423189 46.42341535 4.82E-150 3.01E-148 332.6620439

FAHD2A -2.356584036 3.696698888 -46.3708892 6.73E-150 4.19E-148 332.3262792

S100A6 6.222367911 10.02217757 46.24371985 1.52E-149 9.43E-148 331.5122239

LTC4S -1.995418414 1.068791259 -46.1981575 2.03E-149 1.26E-147 331.220168

SLC27A5 -2.343923861 2.349709664 -46.08353018 4.24E-149 2.62E-147 330.484479

FARP1 -2.654740696 3.86396209 -46.07831176 4.38E-149 2.70E-147 330.4509552

SARS2 -2.722874269 3.391330359 -46.02713879 6.09E-149 3.73E-147 330.1220672

ABRAXAS2 1.483491718 3.228365263 45.97523072 8.50E-149 5.19E-147 329.7881845

ZFPL1 -2.351740278 3.814976191 -45.91094624 1.29E-148 7.83E-147 329.3743163

SLC38A9 -1.986439139 2.744156169 -45.81374934 2.40E-148 1.46E-146 328.7477602

MTG1 -2.815845347 3.491685672 -45.81275028 2.42E-148 1.46E-146 328.741315

NRG4 -3.821266967 2.662867976 -45.81129087 2.44E-148 1.47E-146 328.7318998

RBM28 -2.077776112 3.141815647 -45.80840136 2.49E-148 1.50E-146 328.7132579

ZBTB7A 1.868711725 3.994943891 45.74420585 3.76E-148 2.26E-146 328.2988764

VIPR2 -3.148643302 2.094818219 -45.73359328 4.03E-148 2.41E-146 328.230332

SLC35F6 1.767472492 4.147159805 45.72852451 4.16E-148 2.48E-146 328.1975897

PSMG4 -2.756821292 3.174260636 -45.72614764 4.23E-148 2.51E-146 328.1822352

RALA 2.159101086 4.488020885 45.68806636 5.41E-148 3.20E-146 327.9361528

C15orf40 -1.990396525 2.749182135 -45.65346094 6.76E-148 3.99E-146 327.7124034

MPPE1 -2.411747987 3.70352992 -45.64851141 6.98E-148 4.11E-146 327.6803911

SPTLC2 2.010540987 3.535835734 45.49553291 1.88E-147 1.10E-145 326.6897319

NUDT6 -1.568157143 1.484984817 -45.40362617 3.41E-147 1.99E-145 326.0934111

GIT2 -2.500374844 3.4603599 -45.39683115 3.56E-147 2.08E-145 326.0492884

CORO2A 3.880767427 2.51669898 45.34237707 5.07E-147 2.95E-145 325.6955263

NDUFV2 -3.206031891 4.712232014 -45.32783972 5.57E-147 3.23E-145 325.6010326

LRRC24 -1.66864058 0.848267772 -45.3277457 5.58E-147 3.23E-145 325.6004214

PILRB -4.162256747 3.672310899 -45.30237868 6.57E-147 3.79E-145 325.435482

UBE2T 3.200045645 2.489843071 45.30217063 6.58E-147 3.79E-145 325.434129

GVQW3 -2.179889723 1.43911549 -45.2907073 7.09E-147 4.07E-145 325.3595711

ADHFE1 -3.416616492 3.25852417 -45.28313717 7.45E-147 4.26E-145 325.3103273

CLK3 -2.474710718 3.911865411 -45.25555372 8.91E-147 5.08E-145 325.1308468

EFCAB2 -2.410158173 2.432513954 -45.24753582 9.39E-147 5.34E-145 325.0786611

MSTO1 -2.868978879 3.854561037 -45.21442291 1.16E-146 6.60E-145 324.8630707

POLI -2.434937878 3.241430825 -45.12877627 2.03E-146 1.15E-144 324.3049218

BSCL2 -3.05404548 3.955737281 -45.04792819 3.44E-146 1.94E-144 323.7773504

NBPF26 -2.185661739 1.798603371 -44.98501603 5.19E-146 2.92E-144 323.366352

CCNK -2.196365115 2.759827406 -44.85922265 1.18E-145 6.62E-144 322.5433286

SGCB 2.246180851 3.698876684 44.85057661 1.25E-145 6.99E-144 322.4867003

MARCKSL1 4.159429916 6.284826438 44.80690267 1.66E-145 9.27E-144 322.2005335

RALGAPA1 -2.047894891 2.803594216 -44.79910956 1.75E-145 9.73E-144 322.1494495

LIX1L 2.134883075 3.572968522 44.7917541 1.84E-145 1.02E-143 322.1012286

PPP3CA 2.060201274 3.921990103 44.75674835 2.31E-145 1.28E-143 321.8716606

FAM47E -2.427542639 1.989481966 -44.74019244 2.58E-145 1.42E-143 321.7630425

VDR 2.808423353 2.89259874 44.73497702 2.67E-145 1.46E-143 321.7288198

TUBE1 -2.182535787 3.038226912 -44.72477361 2.85E-145 1.56E-143 321.6618587

COQ6 -1.868462083 2.84000453 -44.71173937 3.11E-145 1.70E-143 321.5763041

SYAP1 1.75729223 3.896539501 44.68683811 3.66E-145 1.99E-143 321.4128072

DGCR6 -2.766991869 2.186885909 -44.63488821 5.14E-145 2.79E-143 321.0715062

SERPINA3 -6.986528588 5.184858209 -44.59643827 6.62E-145 3.59E-143 320.8187162

ELK3 2.658325031 3.887659181 44.58930339 6.94E-145 3.75E-143 320.7717908

RAP2A 1.821964329 2.894761936 44.56931661 7.91E-145 4.26E-143 320.6403114

BBIP1 -2.106459904 2.976591552 -44.56615306 8.08E-145 4.34E-143 320.6194967

DPH1 -2.837259522 4.253155924 -44.48179255 1.41E-144 7.54E-143 320.0640586

SLC31A2 -2.753515003 2.49023621 -44.48097528 1.41E-144 7.56E-143 320.058674

SMAD2 -2.154515463 3.320658024 -44.45326967 1.70E-144 9.05E-143 319.8760927

DTNA -3.161375131 2.699493063 -44.44636425 1.78E-144 9.44E-143 319.8305732

APOL1 4.809112962 5.310286756 44.3557177 3.23E-144 1.71E-142 319.2325805

TMSB10 4.898584781 11.1067718 44.31229986 4.30E-144 2.27E-142 318.945849

TSPOAP1 -3.108343931 3.442112427 -44.29983992 4.67E-144 2.46E-142 318.863527

HYPK -1.848775223 1.700583716 -44.28427872 5.17E-144 2.72E-142 318.7606922

PI4K2A 1.722347312 3.438364854 44.25044445 6.47E-144 3.39E-142 318.5370137

ABCA5 -3.365282374 3.839950957 -44.2486071 6.54E-144 3.42E-142 318.5248636

LY6G5B -2.324037807 2.394885153 -44.2423782 6.82E-144 3.56E-142 318.4836699

ALKBH6 -2.138659933 2.357086759 -44.19722582 9.19E-144 4.78E-142 318.1849415

ERVK3-1 -2.139465689 3.709072866 -44.18056185 1.03E-143 5.32E-142 318.0746382

IL13RA1 2.24758001 5.111472207 44.15901508 1.18E-143 6.12E-142 317.9319711

SLC7A6OS -1.611563629 2.330642187 -44.13557659 1.38E-143 7.13E-142 317.7767229

AC015813.2 -2.345923374 1.223460734 -44.13394732 1.40E-143 7.19E-142 317.7659291

DMRTC1B -1.929694459 0.978484865 -44.10269571 1.72E-143 8.82E-142 317.558834

AHR 3.219671475 4.191325404 44.09479205 1.81E-143 9.27E-142 317.5064425

AC006254.1 -2.774714316 1.824594973 -44.01300118 3.11E-143 1.59E-141 316.9638825

TOMM5 -2.706583294 4.628430689 -43.90843664 6.23E-143 3.17E-141 316.2692231

ACAD8 -2.217151051 3.992436523 -43.80771657 1.22E-142 6.18E-141 315.5990083

AC026464.6 -2.405069254 1.167577217 -43.78140501 1.45E-142 7.35E-141 315.4237477

POLR2J2 -1.652734766 0.813284484 -43.72916038 2.05E-142 1.04E-140 315.0755294

CKS2 3.51789957 3.888250803 43.65704049 3.32E-142 1.67E-140 314.5943623

SMS 2.07568074 5.274986409 43.62440044 4.13E-142 2.08E-140 314.3764134

FAM133B -1.907135515 3.016548717 -43.61719598 4.33E-142 2.17E-140 314.3282914

CCDC189 -1.985824254 1.872686392 -43.61458412 4.41E-142 2.20E-140 314.3108442

MARVELD1 2.978952727 4.366349459 43.59301516 5.09E-142 2.54E-140 314.1667358

FAM20B 1.714181079 3.882485341 43.55951528 6.36E-142 3.17E-140 313.9428152

HSFX1 -0.954501872 0.50195692 -43.53349583 7.57E-142 3.76E-140 313.7688128

ANKRD53 -2.459191678 2.079735046 -43.50078765 9.42E-142 4.67E-140 313.5499775

PHC1 -2.573430948 3.323680107 -43.45002965 1.32E-141 6.54E-140 313.2101527

CCDC117 -2.026994187 1.956332342 -43.43490333 1.46E-141 7.21E-140 313.1088287

MICOS10 -2.60925973 4.799895339 -43.42939234 1.52E-141 7.47E-140 313.0719071

TNFRSF21 3.591759821 5.205310083 43.41868302 1.63E-141 8.00E-140 313.0001493

RP2 2.045858685 2.608627544 43.40730019 1.76E-141 8.61E-140 312.9238652

ENDOD1 2.496476514 3.717154054 43.36326542 2.36E-141 1.15E-139 312.6286274

PCSK7 -2.441223567 3.393270901 -43.32785449 3.00E-141 1.46E-139 312.3910587

TMED3 -3.330261825 5.531254881 -43.30193241 3.57E-141 1.73E-139 312.2170645

APOL6 2.481327704 2.783504852 43.2994814 3.63E-141 1.76E-139 312.2006092

CYP2E1 -2.954440384 2.132089662 -43.26516325 4.57E-141 2.21E-139 311.9701388

BMPR2 2.140271926 3.447626988 43.25760945 4.80E-141 2.31E-139 311.9193928

ACTR3C -2.13986599 2.095710089 -43.22715426 5.89E-141 2.83E-139 311.7147342

FUCA1 2.415796121 5.15320544 43.14631559 1.01E-140 4.86E-139 311.1710151

PIGL -2.698080899 3.211084588 -43.09512239 1.43E-140 6.85E-139 310.8263267

RABL2A -1.907131237 2.636989506 -43.08230981 1.56E-140 7.44E-139 310.7400143

LRRC37A2 -2.532952205 2.229642698 -43.04802523 1.96E-140 9.35E-139 310.5089677

PIGP -2.385413664 3.363206933 -43.04037229 2.07E-140 9.83E-139 310.4573767

INTS6 -2.217992677 2.952225496 -42.94372835 3.97E-140 1.88E-138 309.8053221

MRPS24 -4.196473653 4.63508766 -42.9427982 3.99E-140 1.89E-138 309.7990415

DPYSL3 3.841902278 4.126567156 42.88372986 5.95E-140 2.80E-138 309.4000053

ZNF596 -1.57723941 1.753952575 -42.83886427 8.05E-140 3.79E-138 309.0966632

SPEF2 -2.038993208 1.949844391 -42.82074734 9.10E-140 4.27E-138 308.9741104

CNKSR3 -2.373617306 2.654146343 -42.76013443 1.37E-139 6.42E-138 308.5638327

ZNF625-ZNF20 -1.146954109 0.597552669 -42.75870399 1.38E-139 6.47E-138 308.5541455

PTBP3 2.089688691 4.317861543 42.75577323 1.41E-139 6.58E-138 308.5342972

SLC2A14 -2.352110456 1.342444848 -42.74061061 1.56E-139 7.27E-138 308.4315946

AL163636.2 -2.894910808 2.028451491 -42.50810229 7.57E-139 3.51E-137 306.8535879

SERTAD1 2.66311869 3.903908398 42.43798412 1.22E-138 5.64E-137 306.3765469

SDHC -2.528704286 4.769809026 -42.41851396 1.39E-138 6.42E-137 306.2439885

SULT1A4 -2.193703196 1.076872866 -42.39111087 1.68E-138 7.72E-137 306.0573502

PIK3C3 -1.936174514 3.130016306 -42.3501443 2.22E-138 1.02E-136 305.7781798

MAN2C1 -3.2626503 5.526857298 -42.34597421 2.28E-138 1.04E-136 305.749752

F2R 3.698978984 3.887264075 42.29646178 3.19E-138 1.46E-136 305.4120769

HNRNPA0 1.643933049 4.606419025 42.28438645 3.47E-138 1.58E-136 305.3296823

ATXN3 -1.778704938 2.349173874 -42.23561287 4.84E-138 2.20E-136 304.9967187

SPX -4.47113381 2.601406238 -42.1886101 6.67E-138 3.03E-136 304.6755967

PMM2 -2.633437622 3.649299549 -42.15566095 8.35E-138 3.78E-136 304.450344

SNX12 1.744856617 4.918030293 42.0993711 1.23E-137 5.54E-136 304.0652492

FAM219B -2.11871004 3.534670653 -42.08809324 1.32E-137 5.97E-136 303.9880522

SLX1B -2.841742762 1.426804377 -42.00746492 2.30E-137 1.03E-135 303.4357427

MYO15A -1.224666753 0.795492191 -41.95770902 3.23E-137 1.45E-135 303.0945536

HLA-A 3.768941267 8.869342099 41.93859799 3.69E-137 1.65E-135 302.9634316

WBP1 -2.858261888 4.7544541 -41.92854846 3.95E-137 1.76E-135 302.8944649

CCDC155 -1.85844793 0.970079813 -41.86981453 5.91E-137 2.63E-135 302.4911698

FLRT2 -3.885769529 3.600128107 -41.86596693 6.07E-137 2.70E-135 302.4647371

RPS27L -3.231803041 5.781075919 -41.85461814 6.56E-137 2.91E-135 302.386762

RFESD -1.600701539 1.316790552 -41.85208685 6.67E-137 2.95E-135 302.3693682

RAB22A 1.474462487 3.213448243 41.84450219 7.03E-137 3.11E-135 302.3172456

SOWAHC 2.070763956 2.696762299 41.82182603 8.21E-137 3.62E-135 302.1613746

SREK1 -2.380318435 4.209529403 -41.7771624 1.12E-136 4.91E-135 301.8542

SRP19 -2.610226701 4.753913411 -41.77474167 1.14E-136 4.98E-135 301.8375451

CHFR -1.896636507 3.105495732 -41.77193425 1.16E-136 5.07E-135 301.8182289

RNASE4 -3.108835607 3.393047642 -41.75492865 1.30E-136 5.68E-135 301.7012047

MST1 -3.1390668 3.339383268 -41.67966229 2.18E-136 9.51E-135 301.1828733

EGFL8 -2.400725869 2.340456498 -41.66435055 2.43E-136 1.05E-134 301.07735

GNAQ 1.783421966 4.084177235 41.64609956 2.75E-136 1.19E-134 300.9515365

PLEKHG1 2.241491065 2.267844968 41.63673394 2.93E-136 1.27E-134 300.88696

KDM4C -1.877235031 3.056671556 -41.63167045 3.04E-136 1.31E-134 300.8520429

NRBF2 1.572732982 3.90483133 41.60938477 3.54E-136 1.53E-134 300.6983301

ACTB 4.107416916 10.87982854 41.6035743 3.69E-136 1.59E-134 300.658244

YWHAG 2.24726876 5.774527442 41.58946983 4.06E-136 1.74E-134 300.5609225

ADAMTS17 -2.058122705 1.662203894 -41.4976899 7.65E-136 3.28E-134 299.9270954

NPHS1 -3.737834385 2.68924123 -41.41576297 1.35E-135 5.76E-134 299.3605192

WBP1L 2.101191067 4.599582954 41.32542351 2.52E-135 1.07E-133 298.7348962

ANKAR -1.462803998 1.623048742 -41.29002892 3.22E-135 1.37E-133 298.489531

AC012651.1 -1.560126221 1.450741604 -41.26869246 3.73E-135 1.58E-133 298.341553

CDR2L 2.91847662 3.618867525 41.2652379 3.82E-135 1.62E-133 298.3175893

FAM153B -2.991228537 1.770174761 -41.22335179 5.11E-135 2.16E-133 298.0269255

PAK2 1.850698134 4.322889649 41.22121885 5.18E-135 2.19E-133 298.012119

RAB8B 2.104560743 3.101142425 41.20065852 5.98E-135 2.52E-133 297.8693661

AC244197.3 -2.702075132 2.131092974 -41.19475328 6.23E-135 2.62E-133 297.8283566

EFNB1 3.391325744 4.527290091 41.14576187 8.75E-135 3.67E-133 297.4879797

ZNF98 -2.716337511 1.542317962 -41.13384681 9.50E-135 3.97E-133 297.4051569

GIMAP5 -2.204176332 1.718741253 -41.07096665 1.47E-134 6.14E-133 296.9678062

CCPG1 -2.615239875 4.275187087 -41.06570223 1.53E-134 6.35E-133 296.9311704

CHIC2 1.740764381 3.997731523 41.05577621 1.63E-134 6.79E-133 296.8620855

CLK4 -2.286171987 3.782797388 -40.99323327 2.53E-134 1.05E-132 296.4265323

ANAPC5 -2.566301599 5.508314879 -40.93879634 3.69E-134 1.53E-132 296.0470714

MRNIP -2.631121196 3.712196357 -40.91607529 4.32E-134 1.78E-132 295.8885921

LRRC28 -1.976400578 2.999492353 -40.91055467 4.49E-134 1.85E-132 295.850077

GTF2I -3.043012904 4.505529015 -40.88061303 5.53E-134 2.28E-132 295.6411264

TSSK3 -1.4134257 1.021713015 -40.86733187 6.07E-134 2.49E-132 295.5484102

MAPKAPK5 -1.907723217 3.397804802 -40.8647351 6.18E-134 2.53E-132 295.5302797

BPNT1 2.008126169 3.815117025 40.86064054 6.36E-134 2.60E-132 295.5016903

IKBKG -1.804833379 2.988557118 -40.85180097 6.77E-134 2.76E-132 295.4399633

REEP3 2.280802907 3.938317994 40.84634225 7.03E-134 2.86E-132 295.4018404

MB21D2 1.742687918 1.787608578 40.82301125 8.27E-134 3.36E-132 295.2388624

TMEM266 -2.017155212 1.620614274 -40.81996038 8.45E-134 3.42E-132 295.2175461

RNF212 -3.601668821 2.744187358 -40.81261035 8.89E-134 3.60E-132 295.1661874

INAFM2 2.211196078 4.1021914 40.75877565 1.30E-133 5.23E-132 294.7898286

ZNF585A -1.740195142 2.07195215 -40.72006164 1.70E-133 6.84E-132 294.5189761

HINFP -1.842971071 3.239890541 -40.71748772 1.73E-133 6.95E-132 294.5009623

ZDHHC20 2.114834253 4.163634125 40.71226632 1.79E-133 7.19E-132 294.4644176

CTTNBP2NL 1.937036358 2.936517215 40.69914457 1.96E-133 7.86E-132 294.3725646

LYRM9 -2.357404044 2.876883759 -40.66758723 2.45E-133 9.79E-132 294.1515815

KPNA3 1.684195598 3.643747788 40.65951672 2.59E-133 1.03E-131 294.0950489

FAM243B -1.43498182 0.696633608 -40.62379927 3.33E-133 1.32E-131 293.8447655

HIST1H4I 3.135570951 2.055943517 40.60013993 3.93E-133 1.56E-131 293.6788973

DDX39B -3.553583902 6.238913088 -40.59866338 3.97E-133 1.57E-131 293.6685436

STK38 2.140134476 4.494217291 40.58668276 4.32E-133 1.71E-131 293.5845251

RPL17-C18orf32 -3.04740142 1.502275664 -40.5711231 4.81E-133 1.90E-131 293.4753828

CCDC71 1.528120033 3.846232134 40.55453885 5.41E-133 2.13E-131 293.3590234

TWSG1 2.393183221 3.532375133 40.54659729 5.72E-133 2.25E-131 293.3032922

SYT15 -2.173505546 1.144052704 -40.52279641 6.75E-133 2.65E-131 293.136223

MRPL14 2.154892953 5.328580838 40.49323984 8.31E-133 3.25E-131 292.9286623

CARD11 3.70683375 3.071381262 40.49091963 8.45E-133 3.30E-131 292.9123644

NBPF11 -1.66852394 2.43665091 -40.46554652 1.01E-132 3.94E-131 292.7340961

SLC25A45 -3.308357656 3.317461896 -40.46243722 1.03E-132 4.02E-131 292.7122454

THOC1 -2.21829618 3.772669366 -40.4551238 1.09E-132 4.22E-131 292.6608461

KLHDC2 -2.52957773 4.805735187 -40.45173906 1.11E-132 4.31E-131 292.6370557

LRCH4 -2.650769831 4.204446222 -40.45061418 1.12E-132 4.34E-131 292.629149

GOLGA8S -0.705467685 0.373218806 -40.44953831 1.13E-132 4.36E-131 292.6215866

HRH1 2.520029129 1.732961788 40.439475 1.21E-132 4.67E-131 292.5508445

B3GALT4 1.996428609 3.114893754 40.4338362 1.26E-132 4.85E-131 292.5112003

TVP23C-CDRT4 -2.327743057 1.27490031 -40.36588592 2.03E-132 7.80E-131 292.0331838

IFRD1 -3.222747359 5.079383448 -40.36344423 2.07E-132 7.92E-131 292.0159971

TWF2 2.296961221 4.999108188 40.35401392 2.21E-132 8.45E-131 291.9496126

SLC9A6 1.375074053 2.682219908 40.33857472 2.46E-132 9.40E-131 291.8409067

BCL10 2.344022591 3.52411105 40.32340514 2.74E-132 1.04E-130 291.7340727

SPRED1 1.991171215 2.985395441 40.30442473 3.13E-132 1.19E-130 291.6003633

SPIN2A -0.762045073 0.535093008 -40.27648834 3.81E-132 1.45E-130 291.4034879

TPX2 3.524287256 2.27370439 40.25109101 4.56E-132 1.73E-130 291.2244286

CASP3 1.682901146 4.012343055 40.23923674 4.96E-132 1.87E-130 291.140827

FBXO9 -2.615223406 4.750398022 -40.23454696 5.12E-132 1.93E-130 291.1077481

C5orf15 2.092116954 5.163587199 40.19969072 6.55E-132 2.47E-130 290.8618146

CFAP44 -2.057290226 2.433938808 -40.19116436 6.96E-132 2.61E-130 290.8016344

RCC2 2.222499009 5.19074108 40.10382455 1.29E-131 4.83E-130 290.1846994

ACTR2 2.451992422 5.831100889 40.10267951 1.30E-131 4.86E-130 290.1766055

ENOSF1 -2.752259411 4.314398186 -40.08284876 1.49E-131 5.58E-130 290.0364044

BATF 3.793382064 2.927386882 40.07478367 1.58E-131 5.90E-130 289.9793722

TM2D3 -1.875499337 3.890702618 -40.05576093 1.81E-131 6.73E-130 289.8448237

IGFN1 -3.688523023 2.261865708 -40.01916795 2.34E-131 8.70E-130 289.5858835

RABL2B -2.037042216 3.55968791 -39.97298554 3.25E-131 1.20E-129 289.2588671

B3GNT3 4.424672928 4.137032323 39.93194429 4.34E-131 1.61E-129 288.9680495

NCBP3 -1.985686149 3.795563365 -39.9151571 4.89E-131 1.81E-129 288.84904

ARIH1 -1.865342402 3.693135112 -39.879042 6.32E-131 2.33E-129 288.5928992

RBM14-RBM4 -1.454606669 1.567704749 -39.86812066 6.82E-131 2.51E-129 288.5154118

CSTF2 1.615684399 3.077505814 39.86050088 7.20E-131 2.65E-129 288.4613409

ZNF814 -1.845808749 2.353818894 -39.84590757 7.99E-131 2.93E-129 288.3577664

ADPRHL2 1.736219473 4.683268622 39.83871833 8.41E-131 3.08E-129 288.3067325

NPEPL1 -2.73407909 3.769751149 -39.82863531 9.03E-131 3.30E-129 288.2351466

AL132780.3 -2.828134625 1.373355717 -39.8187994 9.68E-131 3.53E-129 288.1653039

GNA13 2.006556491 3.978948 39.81759534 9.76E-131 3.55E-129 288.1567534

LGALS3BP 3.46277229 7.530121665 39.81426324 1.00E-130 3.63E-129 288.1330898

TAF10 -2.890393637 5.735169919 -39.79929867 1.11E-130 4.03E-129 288.0268004

ZNF717 -1.574192465 2.173047575 -39.7816912 1.26E-130 4.56E-129 287.901706

WDFY2 -2.138570255 2.965109399 -39.77623492 1.31E-130 4.73E-129 287.8629339

ABCB6 -2.044660212 2.521467194 -39.77533298 1.32E-130 4.75E-129 287.8565245

E2F3 1.841537369 2.195669339 39.73337057 1.78E-130 6.39E-129 287.5582227

CCDC66 -1.955339442 2.721842552 -39.73312012 1.78E-130 6.39E-129 287.5564418

CAMTA1 -2.099413875 3.794845373 -39.69570162 2.32E-130 8.32E-129 287.2902693

TMEM134 -2.920975657 5.126096993 -39.67163854 2.75E-130 9.85E-129 287.1190138

MRPS18C -1.852433515 3.396404623 -39.64796685 3.26E-130 1.16E-128 286.9504786

MON2 -2.193287052 3.791591789 -39.63933106 3.46E-130 1.23E-128 286.8889783

GNA15 3.404393787 2.759426316 39.63913152 3.47E-130 1.23E-128 286.8875572

SAMD9 2.910462927 2.670119378 39.63695348 3.52E-130 1.25E-128 286.8720447

C22orf39 -2.010151589 3.673518351 -39.62125012 3.94E-130 1.40E-128 286.7601859

POMP 1.98930827 6.058504329 39.54930545 6.58E-130 2.33E-128 286.247342

HMCES 1.724885907 4.522444539 39.51533608 8.38E-130 2.96E-128 286.0049896

HLA-DRB1 5.121833458 7.323541837 39.49479141 9.70E-130 3.42E-128 285.8583501

RGS10 2.77309092 3.928061293 39.4597394 1.25E-129 4.38E-128 285.6080502

MTHFD2L -1.546506645 2.115364129 -39.45354512 1.30E-129 4.57E-128 285.5638032

SFT2D2 1.293850942 3.235718389 39.43931506 1.44E-129 5.05E-128 285.4621381

OLFML2B 4.138553948 3.395985414 39.43756285 1.46E-129 5.11E-128 285.449618

UNC5B 2.677988413 3.435696003 39.43431782 1.49E-129 5.22E-128 285.4264303

HYDIN -1.121092778 0.715467353 -39.42953601 1.54E-129 5.39E-128 285.3922591

SERTAD3 2.122675193 4.16114512 39.34468351 2.83E-129 9.87E-128 284.7854559

UBTD2 1.621509978 3.727207844 39.33745587 2.98E-129 1.04E-127 284.7337304

RLIM 1.416845541 3.194041782 39.33710814 2.99E-129 1.04E-127 284.7312417

RALB 2.012149714 4.99185056 39.32828057 3.18E-129 1.10E-127 284.6680576

SLC24A3 2.644418504 2.170131638 39.32258441 3.32E-129 1.15E-127 284.627282

NACC1 1.75259538 4.090854384 39.29522786 4.03E-129 1.39E-127 284.4313994

DMKN -3.521227231 5.29593265 -39.28849929 4.23E-129 1.46E-127 284.3832071

UBA5 -2.16158807 4.52115937 -39.28222774 4.43E-129 1.52E-127 284.3382834

SPRY4 2.142563515 3.343781159 39.2641635 5.04E-129 1.73E-127 284.208862

GRB10 -2.697356608 5.205921611 -39.19609899 8.20E-129 2.81E-127 283.7208723

SELPLG 3.701977298 3.369306568 39.18781315 8.70E-129 2.98E-127 283.66143

COL5A2 4.687691582 4.705386903 39.15824849 1.08E-128 3.68E-127 283.4492689

N4BP2L2 -2.298623096 4.450404487 -39.15295616 1.12E-128 3.81E-127 283.4112796

PAN2 -2.952554708 4.849294651 -39.1145475 1.47E-128 5.01E-127 283.1354767

FAM214A -2.232525538 3.962094327 -39.09300181 1.72E-128 5.84E-127 282.9806873

GM2A 2.303062322 4.249956848 39.09175631 1.73E-128 5.88E-127 282.9717376

YWHAB 2.142565528 6.438527136 39.09146786 1.74E-128 5.88E-127 282.9696649

DERL2 -2.422962198 4.891515942 -39.07434958 1.96E-128 6.64E-127 282.8466414

ZNF19 -1.331734943 1.630871287 -39.06606279 2.08E-128 7.04E-127 282.7870746

ING5 -2.092078774 3.614696479 -39.03888785 2.53E-128 8.54E-127 282.5916805

CMTM6 2.267066622 4.941536268 39.02951231 2.71E-128 9.12E-127 282.5242483

SYNE1 -2.580548824 3.144976371 -39.02794357 2.74E-128 9.20E-127 282.5129643

DYRK4 -2.42053615 3.735663389 -39.01310008 3.05E-128 1.02E-126 282.4061808

RPAP2 -1.449970542 2.289210332 -38.9910378 3.57E-128 1.20E-126 282.247418

RBPJL -7.32958073 5.048293076 -38.97961591 3.88E-128 1.30E-126 282.1652024

IRF9 -3.081043702 3.831805749 -38.94066896 5.13E-128 1.71E-126 281.8847446

CCDC32 -1.762144004 3.051064696 -38.93905956 5.19E-128 1.73E-126 281.8731515

CTHRC1 5.069475077 4.371919225 38.9255402 5.72E-128 1.90E-126 281.7757543

MSH5 -2.107266324 2.055889737 -38.87487493 8.24E-128 2.74E-126 281.4105574

HOMER2 -4.332762434 4.601032366 -38.84331392 1.03E-127 3.43E-126 281.1829126

AFAP1 2.078155598 2.971428196 38.83998024 1.06E-127 3.51E-126 281.1588604

APOBEC3C 2.664923963 3.684619442 38.82419651 1.19E-127 3.92E-126 281.0449649

FAM227B -1.703092745 1.822542547 -38.78900406 1.53E-127 5.05E-126 280.7909105

TMEM52 -5.434866939 4.465083002 -38.76618762 1.80E-127 5.94E-126 280.626121

PRDM5 -1.852068977 1.943427737 -38.75412715 1.97E-127 6.47E-126 280.5389907

TP53RK 1.373545528 3.064481141 38.73565109 2.25E-127 7.38E-126 280.4054783

MRRF -1.966156793 3.642998389 -38.73227892 2.30E-127 7.55E-126 280.3811058

EIF3F -2.844627715 6.430116424 -38.72715576 2.39E-127 7.82E-126 280.3440755

MCM6 1.988491519 3.232515214 38.71251866 2.66E-127 8.67E-126 280.2382612

HMGN4 1.982782039 5.344140123 38.705546 2.80E-127 9.11E-126 280.1878457

HDAC10 -2.780030566 3.712434673 -38.691098 3.10E-127 1.01E-125 280.083362

MTO1 -1.724566632 2.762378932 -38.67178486 3.57E-127 1.16E-125 279.9436566

B3GNT9 1.949814026 2.920232543 38.62408086 5.04E-127 1.63E-125 279.5983927

FEM1B 1.473923309 3.554557279 38.62306018 5.07E-127 1.64E-125 279.5910024

ZNF703 2.566754164 4.302554226 38.60400081 5.82E-127 1.88E-125 279.4529806

ABCC6 -2.647853285 2.633850913 -38.57486022 7.19E-127 2.32E-125 279.2418712

DCAF8 -2.56195004 5.576609856 -38.56869735 7.52E-127 2.42E-125 279.1972114

DMD -2.940405826 2.953026804 -38.5615186 7.92E-127 2.55E-125 279.1451843

EHD3 1.971762769 2.123243761 38.55664855 8.20E-127 2.63E-125 279.1098858

DPP10 -2.230546481 1.397488564 -38.55448535 8.33E-127 2.67E-125 279.0942058

RNF31 -2.163047732 4.186826967 -38.54913166 8.66E-127 2.77E-125 279.0553972

CHMP4B 2.432978953 7.150504952 38.54059252 9.22E-127 2.94E-125 278.9934905

TBC1D2 2.579102431 3.077080369 38.52852557 1.01E-126 3.21E-125 278.9059933

BGN 4.878755354 8.013279538 38.50421426 1.20E-126 3.82E-125 278.7296605

POLR3E -1.950183791 4.191029669 -38.50371975 1.20E-126 3.83E-125 278.726073

ZNF706 -2.991291745 5.988567505 -38.48652414 1.36E-126 4.33E-125 278.6013082

NDUFA11 -3.075737658 5.678708468 -38.4821851 1.41E-126 4.46E-125 278.5698202

ARL4C 3.455449101 4.073580962 38.41885371 2.23E-126 7.05E-125 278.1099784

ZSWIM1 1.370595659 2.919292255 38.4107969 2.36E-126 7.46E-125 278.0514449

FBXW12 -2.436444534 1.410313139 -38.40856359 2.40E-126 7.57E-125 278.0352183

TMEM138 -1.877333438 3.927438847 -38.40572819 2.45E-126 7.71E-125 278.0146163

MAN2A2 -2.605882489 4.827785545 -38.40261846 2.51E-126 7.88E-125 277.99202

TPD52L1 -2.955830796 3.956013936 -38.39124691 2.72E-126 8.54E-125 277.9093805

CWC22 1.305859379 3.602209344 38.34183527 3.90E-126 1.22E-124 277.5501186

RBBP9 1.449289486 2.887700985 38.3300173 4.25E-126 1.33E-124 277.4641497

PHYKPL -2.378133952 4.49342803 -38.31678111 4.67E-126 1.46E-124 277.3678446

CHMP1B 1.851522795 4.762290663 38.31168181 4.85E-126 1.51E-124 277.3307371

NME1-NME2 -2.631068858 2.33632505 -38.24221617 8.04E-126 2.50E-124 276.8249308

STARD5 -1.589927813 1.608340196 -38.23622472 8.40E-126 2.61E-124 276.7812779

ZNF7 -1.70872952 3.166631061 -38.22592741 9.05E-126 2.81E-124 276.7062433

DCLRE1B 1.431979016 1.754274741 38.21566587 9.75E-126 3.02E-124 276.6314569

CECR2 -1.463402993 0.9545574 -38.19265771 1.15E-125 3.57E-124 276.4637272

LAMP5 4.179175993 2.615637523 38.18261574 1.24E-125 3.83E-124 276.3905016

C6orf47 1.498021245 4.193054322 38.18154374 1.25E-125 3.86E-124 276.3826839

DPH6 -1.855263885 2.193604226 -38.16572249 1.40E-125 4.32E-124 276.2672896

HSF4 -3.763296437 4.69787482 -38.16526478 1.41E-125 4.33E-124 276.2639509

PGRMC1 2.12474936 5.929512423 38.13957616 1.70E-125 5.21E-124 276.0765232

DBNDD2 -2.573806466 3.566531264 -38.11663337 2.01E-125 6.15E-124 275.9090633

NDUFAF5 -1.706640425 2.75547181 -38.11425783 2.04E-125 6.25E-124 275.8917207

SPSB4 -2.844458794 2.131215514 -38.08053084 2.61E-125 7.98E-124 275.6454238

TOMM34 1.749915509 4.701330384 38.06664213 2.89E-125 8.81E-124 275.54396

PTPN1 1.714226316 4.420859195 38.05378702 3.17E-125 9.67E-124 275.4500267

GOSR2 -1.969454015 4.134190303 -38.04613491 3.36E-125 1.02E-123 275.3941029

AOPEP -2.660099951 4.768632718 -38.04509107 3.38E-125 1.03E-123 275.3864736

TRIM50 -3.84134874 2.512604975 -38.03083338 3.75E-125 1.14E-123 275.2822539

AC135050.2 -1.548428875 0.84183127 -38.03071504 3.76E-125 1.14E-123 275.2813888

FAM86B1 -1.937858978 1.66067686 -38.02815596 3.83E-125 1.16E-123 275.2626801

ABHD13 1.245415452 2.689328419 38.0179821 4.12E-125 1.24E-123 275.188294

ADORA2A -1.361740955 0.844586584 -37.99252734 4.96E-125 1.50E-123 275.002128

OSBPL11 1.425188785 2.571963773 37.98888313 5.10E-125 1.53E-123 274.9754694

TTYH3 2.682692637 4.497612016 37.94834915 6.86E-125 2.06E-123 274.6788434

TLCD4-RWDD3 -1.154459506 1.172579111 -37.94478523 7.04E-125 2.11E-123 274.6527535

MICAL3 -1.88680147 2.447894541 -37.91435393 8.79E-125 2.63E-123 274.4299171

SCCPDH 2.144510564 4.918237746 37.90839705 9.18E-125 2.75E-123 274.3862843

ATP9B -1.639308415 2.705729842 -37.89895251 9.84E-125 2.94E-123 274.3170966

UPF3A -2.434781715 4.525148379 -37.8934418 1.02E-124 3.05E-123 274.276722

GOLGA8K -1.049548477 0.567241847 -37.89283109 1.03E-124 3.06E-123 274.2722474

TIMM23 1.730478957 5.335002211 37.88930869 1.06E-124 3.14E-123 274.2464381

SMIM8 -1.233739499 1.680037593 -37.86268957 1.28E-124 3.81E-123 274.0513471

ATP6AP2 2.26511685 5.886103463 37.8384815 1.53E-124 4.54E-123 273.8738534

EDNRA 3.212140537 2.637391251 37.83666344 1.55E-124 4.59E-123 273.8605206

PLEKHH1 -3.053362041 3.637491981 -37.82553941 1.68E-124 4.98E-123 273.7789333

ISLR 4.493017557 5.309861112 37.82063909 1.75E-124 5.15E-123 273.7429881

FAM221A -2.369080769 2.957178253 -37.81188017 1.86E-124 5.48E-123 273.6787317

PNPLA7 -2.480781851 3.318972744 -37.78720627 2.23E-124 6.56E-123 273.4976725

MRPL46 -1.637313093 2.930174153 -37.78116775 2.33E-124 6.85E-123 273.4533503

TSPAN1 5.149331772 5.258444437 37.77795343 2.39E-124 7.00E-123 273.4297557

LPAR5 2.635369446 2.022029818 37.76572603 2.61E-124 7.64E-123 273.3399895

KPNA4 1.493807804 3.726255735 37.75601565 2.80E-124 8.20E-123 273.2686891

NDUFA9 -2.454684326 4.224310909 -37.7360599 3.25E-124 9.47E-123 273.1221247

SLC6A6 2.945883838 3.554139961 37.72512718 3.52E-124 1.03E-122 273.0418095

LAMC2 5.645125531 4.624031854 37.71824979 3.70E-124 1.08E-122 272.9912787

DPYSL2 2.132809529 4.237708613 37.71030941 3.92E-124 1.14E-122 272.9329307

MCUB 2.111763651 2.705960279 37.70046874 4.21E-124 1.22E-122 272.8606085

SNAPC1 1.415163492 2.367595943 37.69515826 4.38E-124 1.27E-122 272.8215753

ASB7 1.066558432 2.314585575 37.68858891 4.60E-124 1.33E-122 272.7732844

CXCL16 2.591837209 5.28385555 37.611624 8.10E-124 2.34E-122 272.2071363

NQO2 -2.192211708 3.588147816 -37.60531906 8.48E-124 2.45E-122 272.1607262

POSTN 5.384421222 4.476403309 37.60228243 8.67E-124 2.50E-122 272.1383722

ZNF26 -1.33728168 1.862582046 -37.59367147 9.24E-124 2.66E-122 272.074977

DRG2 -1.742285238 3.699099888 -37.59271188 9.30E-124 2.67E-122 272.0679118

SGPP2 3.241780334 3.366264756 37.57733996 1.04E-123 2.99E-122 271.9547175

AC010522.1 -1.409261771 1.016363636 -37.57553998 1.06E-123 3.02E-122 271.9414611

GDPD5 -2.72572067 3.640339073 -37.56624345 1.13E-123 3.23E-122 271.8729885

BLOC1S4 1.575167934 3.655394839 37.566135 1.13E-123 3.23E-122 271.8721897

GOLGA8Q -1.220229257 0.629804898 -37.544005 1.33E-123 3.80E-122 271.7091515

ATP5MF-PTCD1 -1.387052646 0.792745308 -37.51918212 1.60E-123 4.55E-122 271.5262044

SECISBP2 -2.013227541 4.391721836 -37.51767299 1.62E-123 4.59E-122 271.5150796

SFN 6.150761309 4.450604917 37.50512865 1.77E-123 5.03E-122 271.4225963

KCTD5 1.63034472 3.928308938 37.48380625 2.07E-123 5.88E-122 271.2653533

ZSCAN5A -1.394268265 1.688048301 -37.46210813 2.43E-123 6.89E-122 271.1052836

CERS4 -3.491986476 4.650205527 -37.46118741 2.45E-123 6.92E-122 271.0984901

ALAS1 1.906603748 4.739795551 37.45698712 2.53E-123 7.13E-122 271.0674971

KLHDC4 -2.093398559 3.82787021 -37.45177298 2.63E-123 7.40E-122 271.0290201

TFPT 2.0200603 4.081773818 37.37348222 4.68E-123 1.32E-121 270.4508934

PEA15 2.388712365 6.250119565 37.36441112 5.00E-123 1.41E-121 270.3838616

FAM187A -0.702862042 0.341214995 -37.35734657 5.27E-123 1.48E-121 270.3316507

NTPCR -2.033805896 3.46153019 -37.3449357 5.78E-123 1.62E-121 270.2399128

MFSD14B 1.676957795 4.58238839 37.33010578 6.44E-123 1.80E-121 270.1302698

INO80B -2.37066332 3.734395799 -37.32219546 6.83E-123 1.91E-121 270.0717751

UBR4 -2.208270087 4.982268089 -37.32140682 6.87E-123 1.92E-121 270.0659429

TEPSIN -2.375671107 3.900881814 -37.32066151 6.91E-123 1.93E-121 270.0604311

PANX1 1.929234299 3.322062728 37.31955821 6.97E-123 1.94E-121 270.0522717

NEDD4L -2.902807456 4.111327076 -37.30071088 8.01E-123 2.22E-121 269.9128645

ICMT 1.814066871 4.748484717 37.29824461 8.15E-123 2.26E-121 269.8946192

BIK 3.575811454 3.384353424 37.29140681 8.58E-123 2.38E-121 269.8440298

EGF -4.173623361 2.836840927 -37.28385969 9.07E-123 2.51E-121 269.7881859

DNAJC24 -1.641913968 2.411203093 -37.26448726 1.05E-122 2.89E-121 269.644811

PDCL 1.336482621 3.175546424 37.24318395 1.23E-122 3.38E-121 269.4870937

GOLGA6L10 -1.67237865 1.092037083 -37.24313135 1.23E-122 3.38E-121 269.4867042

INHBA 4.131566484 2.585669025 37.23252006 1.33E-122 3.65E-121 269.4081239

RBM5 -2.644284544 5.559302461 -37.22863005 1.36E-122 3.75E-121 269.3793137

ANO1 3.399846969 3.880274178 37.21224855 1.54E-122 4.22E-121 269.2579688

FNDC10 2.496415485 2.159145295 37.20762373 1.59E-122 4.37E-121 269.2237049

POLR1D -2.754893504 6.199579664 -37.20213 1.66E-122 4.54E-121 269.1830002

EIF4E -1.757352367 3.15360962 -37.17666468 2.00E-122 5.47E-121 268.9942723

HMBOX1 -1.528430358 3.085179953 -37.17273759 2.06E-122 5.63E-121 268.9651611

ARL8A 1.732406629 5.325003613 37.16543589 2.18E-122 5.93E-121 268.9110291

PURB 1.213434463 3.451154392 37.1513504 2.42E-122 6.58E-121 268.8065866

TAMM41 -1.467667226 2.615648142 -37.13739057 2.68E-122 7.28E-121 268.7030523

ZNF709 -0.808753045 0.598343138 -37.12735338 2.89E-122 7.83E-121 268.6285961

UHMK1 1.686422644 3.844874211 37.12700538 2.90E-122 7.84E-121 268.6260143

LAMB3 5.752975466 4.876692589 37.12617673 2.91E-122 7.88E-121 268.6198668

L3HYPDH -2.112037643 3.370346748 -37.1209394 3.03E-122 8.18E-121 268.5810105

ACVR1 1.847099098 4.076064476 37.08327304 4.00E-122 1.08E-120 268.3014629

LRCH1 1.565125809 2.92408706 37.07536146 4.25E-122 1.14E-120 268.242724

AZI2 -1.992840651 3.732675077 -37.05701273 4.86E-122 1.31E-120 268.1064661

SPTSSA 1.947404838 4.372210298 37.05663484 4.88E-122 1.31E-120 268.1036595

FO681492.1 -2.005999779 1.063504944 -37.04493963 5.32E-122 1.43E-120 268.0167891

STX16-NPEPL1 -2.180971012 1.662748362 -37.04103295 5.48E-122 1.47E-120 267.9877671

WDR13 -2.901799454 6.235399404 -37.03445927 5.75E-122 1.54E-120 267.9389284

GPR153 2.760027197 2.625433849 37.03331381 5.80E-122 1.55E-120 267.9304177

TXNL1 -2.187882516 4.501183653 -37.0206811 6.37E-122 1.70E-120 267.8365474

ZNF629 1.351632043 3.184443851 37.00800306 7.00E-122 1.87E-120 267.742321

PBDC1 1.656181586 4.738764602 37.00543822 7.13E-122 1.90E-120 267.7232561

TRMU -2.31649881 3.900777821 -37.00466238 7.17E-122 1.91E-120 267.7174889

PDE4DIP -2.184458182 3.475344911 -36.99760441 7.56E-122 2.01E-120 267.665021

MMP9 4.586255282 2.868419633 36.9953154 7.69E-122 2.04E-120 267.6480036

ZNF224 -1.677479583 2.964637392 -36.98072662 8.57E-122 2.27E-120 267.5395296

FAM92A -1.994222502 3.350980508 -36.97114758 9.20E-122 2.43E-120 267.4682913

LPCAT3 -2.657184566 4.188912489 -36.96597646 9.56E-122 2.52E-120 267.4298296

ASF1B 2.736424217 2.121359478 36.96425304 9.68E-122 2.55E-120 267.4170105

UQCRB -2.695280766 6.444785569 -36.96091865 9.93E-122 2.61E-120 267.3922076

ARL17B -1.405753549 1.011367337 -36.94762351 1.10E-121 2.88E-120 267.2932983

CAMSAP2 1.651817081 2.885887124 36.93657266 1.19E-121 3.12E-120 267.2110692

SMU1 1.382897692 3.567924081 36.91730655 1.37E-121 3.60E-120 267.0676752

ZNF625 -1.184901571 0.801839758 -36.82866935 2.65E-121 6.95E-120 266.4073875

MOB1A 1.79454058 4.559665363 36.82575218 2.71E-121 7.10E-120 266.3856405

ERICH6B -1.273632089 0.89626252 -36.82403655 2.75E-121 7.18E-120 266.3728502

EFNA5 2.707986574 2.120771554 36.81690254 2.90E-121 7.56E-120 266.3196613

GJB3 4.405007149 2.687761593 36.81270799 2.99E-121 7.79E-120 266.2883852

MORN1 -1.417894586 1.695342973 -36.80269349 3.22E-121 8.38E-120 266.2137049

CCDC171 -1.171551482 1.039000271 -36.7985856 3.32E-121 8.63E-120 266.1830679

LTO1 -1.698744873 3.22207412 -36.78098897 3.79E-121 9.83E-120 266.0518078

ZNF75A -1.623758556 2.912534509 -36.76089614 4.40E-121 1.14E-119 265.9018817

GABARAP -2.894411515 7.170684534 -36.7434112 5.01E-121 1.30E-119 265.771375

YAF2 -1.36624203 2.509253076 -36.7227922 5.84E-121 1.51E-119 265.6174284

FKBP11 -3.734554039 5.780377921 -36.70563998 6.64E-121 1.71E-119 265.4893264

RNF26 1.457849197 4.266252195 36.67679797 8.24E-121 2.12E-119 265.2738383

LBH 2.770130601 4.661089945 36.66767013 8.82E-121 2.27E-119 265.2056202

NDUFAF6 -1.809939233 2.82364991 -36.66223792 9.18E-121 2.36E-119 265.1650171

MRPS35 1.890742427 5.073337158 36.63563205 1.12E-120 2.88E-119 264.9660996

FTL 4.228505823 11.7475225 36.63302304 1.14E-120 2.93E-119 264.9465889

B3GALNT2 -2.487741441 3.160444556 -36.62837175 1.18E-120 3.03E-119 264.9118034

DESI2 1.53923614 3.752734528 36.62762317 1.19E-120 3.04E-119 264.9062048

ANKRD20A1 -1.5508025 0.781007014 -36.60840776 1.37E-120 3.51E-119 264.7624697

CLASRP -2.230086196 5.275186537 -36.5812481 1.68E-120 4.29E-119 264.5592336

COL3A1 6.349604717 8.085117491 36.57041245 1.82E-120 4.65E-119 264.4781253

NAA16 -2.177991595 3.692729704 -36.56303005 1.93E-120 4.90E-119 264.4228575

PTAFR 2.725561823 2.584536889 36.55971733 1.98E-120 5.02E-119 264.3980549

EDIL3 3.365758771 2.864799569 36.54924739 2.14E-120 5.42E-119 264.3196569

ITFG2 -2.061250371 4.114571862 -36.53597652 2.36E-120 5.98E-119 264.2202667

FSCN1 3.279477145 4.282258436 36.52848819 2.50E-120 6.31E-119 264.1641745

ATM -2.305849941 3.560102913 -36.52349839 2.59E-120 6.55E-119 264.1267941

CEP95 -2.094445586 3.913175858 -36.50524647 2.97E-120 7.50E-119 263.9900364

FER1L5 -1.438016945 0.860778411 -36.50040312 3.08E-120 7.76E-119 263.9537395

DPH7 -2.454448693 4.57248028 -36.47909727 3.61E-120 9.09E-119 263.7940357

MREG 1.572222533 2.285757841 36.47123196 3.83E-120 9.63E-119 263.7350652

MAPRE1 2.009022401 5.668194954 36.46435308 4.03E-120 1.01E-118 263.6834843

NDUFA10 -2.198446098 5.319837915 -36.45455973 4.34E-120 1.09E-118 263.6100395

LHFPL5 -2.015687613 1.238620152 -36.4491181 4.52E-120 1.13E-118 263.5692252

CNTNAP3B -1.486335147 0.92856787 -36.44682916 4.60E-120 1.15E-118 263.5520562

CPA4 -3.194824808 2.276974473 -36.42155465 5.56E-120 1.39E-118 263.3624336

CCDC57 -2.322107946 3.830096722 -36.40956106 6.08E-120 1.52E-118 263.2724242

C9orf64 1.473224744 3.178590124 36.39936904 6.56E-120 1.64E-118 263.1959215

TTC17 -2.203420318 4.96628249 -36.39326259 6.87E-120 1.71E-118 263.1500795

WASL 1.763801119 4.538416535 36.3904175 7.02E-120 1.74E-118 263.1287195

SLC35E2A -1.516160743 1.7303279 -36.39007711 7.04E-120 1.75E-118 263.1261639

MAATS1 -2.624899395 2.681394081 -36.36906027 8.24E-120 2.04E-118 262.9683449

NEIL1 -2.727087302 3.915800175 -36.33724902 1.05E-119 2.59E-118 262.7293666

PERP 2.798923679 5.913423272 36.33258273 1.08E-119 2.68E-118 262.6943013

NXT1 1.74993784 4.257848162 36.32716831 1.13E-119 2.79E-118 262.6536107

LMNB1 2.17052032 2.988206751 36.32049383 1.19E-119 2.93E-118 262.6034456

ARHGEF4 -2.918201153 3.227012841 -36.31487593 1.24E-119 3.05E-118 262.5612176

TICAM1 2.03598223 4.180603695 36.29603541 1.43E-119 3.51E-118 262.4195709

PLA2G6 -2.797785183 3.889311461 -36.28999522 1.49E-119 3.67E-118 262.3741504

RWDD3 -1.703220954 3.411137909 -36.28134535 1.59E-119 3.90E-118 262.3090982

PIGF -1.680677113 3.18689785 -36.28128407 1.59E-119 3.90E-118 262.3086373

TSEN2 -1.755757306 2.869197646 -36.27888917 1.62E-119 3.97E-118 262.2906246

LMNB2 1.806438736 3.947419658 36.25867404 1.89E-119 4.61E-118 262.138553

COL1A1 6.236519449 7.985419161 36.25708164 1.91E-119 4.66E-118 262.1265719

B3GAT2 -0.900858537 0.729292152 -36.24988018 2.02E-119 4.92E-118 262.0723843

STK19 -1.771977054 3.806992216 -36.23929093 2.18E-119 5.32E-118 261.9926937

COBLL1 -2.323654741 3.614043996 -36.20896782 2.74E-119 6.67E-118 261.7644182

GNAI3 1.261831601 2.37331533 36.20251627 2.88E-119 7.00E-118 261.7158358

KIF11 2.206582579 1.756990217 36.19736232 2.99E-119 7.26E-118 261.6770212

SDC1 4.105343112 5.741175735 36.1484457 4.32E-119 1.05E-117 261.3084662

VKORC1L1 1.481111676 3.75999775 36.14698186 4.37E-119 1.06E-117 261.2974327

ECE2 -2.953018444 3.386788862 -36.14599513 4.40E-119 1.07E-117 261.2899951

HTRA3 4.298239816 4.706324629 36.12860142 5.02E-119 1.21E-117 261.1588692

UTP3 1.498089013 4.351510154 36.12518393 5.15E-119 1.24E-117 261.1331015

FXYD1 -2.571740729 2.246430375 -36.11823396 5.43E-119 1.31E-117 261.0806946

FBXO25 -1.966783444 3.509396363 -36.09405635 6.51E-119 1.57E-117 260.898335

CENPW 2.550139896 2.789466547 36.08844574 6.79E-119 1.63E-117 260.8560068

TVP23C -1.620640215 1.72790112 -36.08678552 6.88E-119 1.65E-117 260.8434808

GCSH -2.006990929 3.09630298 -36.04757723 9.24E-119 2.22E-117 260.5475657

AOC1 4.646115145 2.825627783 36.02068184 1.13E-118 2.71E-117 260.3444707

CCDC110 -2.415527641 2.037615475 -36.01776288 1.16E-118 2.77E-117 260.3224235

ZNF416 1.190850391 1.702423158 36.01693631 1.16E-118 2.78E-117 260.3161801

HLA-DRA 5.193745663 8.186950618 35.99099187 1.42E-118 3.38E-117 260.1201703

SKP1 -2.434969985 6.308015274 -35.99062146 1.42E-118 3.39E-117 260.1173712

ANKRD62 -0.921043531 0.488927039 -35.95122165 1.91E-118 4.55E-117 259.8195472

BASP1 3.326412372 3.48604142 35.93348565 2.19E-118 5.20E-117 259.6854183

OSGIN2 1.562773274 2.844996735 35.92383748 2.35E-118 5.59E-117 259.6124377

SHISA2 2.638753341 1.778393691 35.92367511 2.36E-118 5.59E-117 259.6112094

H2BFS -1.651860586 0.82290171 -35.91814531 2.46E-118 5.82E-117 259.5693757

NRAS 1.770210247 3.890438701 35.91123826 2.59E-118 6.12E-117 259.5171176

RTP4 2.698559505 2.933630129 35.89427198 2.94E-118 6.95E-117 259.3887276

IL2RG 4.117149834 3.636101792 35.87903854 3.30E-118 7.79E-117 259.2734206

IGFBP3 4.469462121 5.675056148 35.87567219 3.39E-118 7.98E-117 259.2479358

NT5E 3.221230512 2.922076236 35.85535637 3.95E-118 9.30E-117 259.0941059

PPIP5K2 -1.836050427 3.214316233 -35.83318466 4.67E-118 1.10E-116 258.9261657

DENND6A 1.208883825 3.119142692 35.82355929 5.02E-118 1.18E-116 258.8532393

RPL39 -3.431260765 8.403913089 -35.81020538 5.56E-118 1.30E-116 258.752045

TIMM21 -1.632340892 3.219958273 -35.79008228 6.47E-118 1.52E-116 258.5995133

CAVIN1 3.089356894 5.969340167 35.77683619 7.15E-118 1.67E-116 258.4990817

RNASEH2B -1.933929062 3.680760307 -35.76509709 7.82E-118 1.83E-116 258.4100581

STAG3 -2.367974513 2.35990627 -35.75519812 8.43E-118 1.97E-116 258.3349761

TPST2 -4.02358694 5.711717755 -35.74784554 8.91E-118 2.08E-116 258.2792002

MDP1 -2.077870746 3.036255577 -35.74018224 9.44E-118 2.20E-116 258.2210602

HK1 2.129948986 4.477430113 35.73519845 9.81E-118 2.28E-116 258.1832452

KLF16 2.06912076 3.60809544 35.72408066 1.07E-117 2.48E-116 258.098877

DNHD1 -1.947714721 1.93267594 -35.69585471 1.32E-117 3.07E-116 257.8846141

NBPF1 -1.907812263 2.422805948 -35.69345406 1.35E-117 3.12E-116 257.8663863

PTPRCAP -1.601505994 0.777475286 -35.67597084 1.54E-117 3.56E-116 257.7336171

MRPL47 1.605041332 4.599775688 35.66099575 1.72E-117 3.98E-116 257.6198652

ZNF273 -1.401185752 1.757534239 -35.65641167 1.78E-117 4.12E-116 257.5850386

SERPINB9 2.40707037 2.7148529 35.63102958 2.16E-117 4.99E-116 257.3921571

RND3 2.462919812 3.657348496 35.62163699 2.32E-117 5.35E-116 257.3207616

MRPS33 -2.294701193 4.633374797 -35.6118343 2.50E-117 5.76E-116 257.2462372

MCOLN3 -2.877980916 2.472243034 -35.55201433 3.94E-117 9.06E-116 256.791204

ANAPC15 -1.959886276 3.869506292 -35.54949874 4.01E-117 9.22E-116 256.7720589

FAM184A -1.537571365 1.388057567 -35.52791518 4.73E-117 1.09E-115 256.6077645

CLCN7 -1.491681826 4.784656591 -35.5210822 4.98E-117 1.14E-115 256.5557397

PLIN3 2.234396786 5.038330802 35.50478717 5.64E-117 1.29E-115 256.43165

CCDC30 -1.1680984 1.177157222 -35.48935431 6.34E-117 1.45E-115 256.3140957

TOR4A 2.815104995 3.458653687 35.45612694 8.17E-117 1.86E-115 256.0608986

MYO19 -2.046166596 3.827712227 -35.4556072 8.20E-117 1.87E-115 256.056937

ZNF526 1.129418486 2.475231524 35.45400437 8.30E-117 1.89E-115 256.0447198

MPI -1.730409244 3.929177231 -35.43617934 9.51E-117 2.16E-115 255.90883

PAQR8 2.785564373 2.55928581 35.39975351 1.25E-116 2.85E-115 255.6310147

UBE2C 3.859541785 2.828021064 35.39246396 1.33E-116 3.01E-115 255.5753986

VAMP3 1.79546249 5.505969871 35.39211461 1.33E-116 3.02E-115 255.5727331

ZNF519 -0.91230087 0.890515595 -35.39005474 1.35E-116 3.06E-115 255.5570159

TCHP -1.655901701 3.045923532 -35.3889868 1.36E-116 3.08E-115 255.5488671

COQ8A -2.995272531 5.409962247 -35.38344905 1.42E-116 3.21E-115 255.5066097

DPH3 1.385384246 3.315762757 35.37761425 1.49E-116 3.35E-115 255.4620816

POLE -1.835743057 3.058709378 -35.37414789 1.53E-116 3.44E-115 255.4356261

YTHDF1 1.451101666 4.865790512 35.37231648 1.55E-116 3.48E-115 255.4216481

MRGBP 1.585135373 3.495367541 35.36591259 1.62E-116 3.65E-115 255.3727679

PAAF1 -1.687973685 3.26202339 -35.36152551 1.68E-116 3.77E-115 255.3392789

IFIT5 1.62579016 3.017008704 35.35908045 1.71E-116 3.84E-115 255.3206134

FBXL8 -2.682773807 3.957974504 -35.34438501 1.91E-116 4.29E-115 255.2084132

THBS2 4.998915342 4.817395136 35.33913597 1.99E-116 4.46E-115 255.1683302

RAB31 3.273948406 4.123844342 35.30844193 2.52E-116 5.63E-115 254.9338746

CRYZL1 -1.49051984 3.425714355 -35.30458176 2.59E-116 5.79E-115 254.9043805

ADAT2 -1.788634538 2.444369973 -35.30363515 2.61E-116 5.83E-115 254.8971476

CYP46A1 -1.961494224 1.308827321 -35.2665614 3.47E-116 7.72E-115 254.613784

IFT20 -2.498671254 5.212262318 -35.24638939 4.04E-116 9.00E-115 254.4595332

CEP83 -1.682576441 2.713107493 -35.24423707 4.11E-116 9.14E-115 254.443072

L3MBTL1 -1.790168958 2.150171285 -35.24063294 4.22E-116 9.38E-115 254.4155058

TBCD -2.011168 4.783419258 -35.23729913 4.33E-116 9.62E-115 254.3900057

TMEM218 -1.602303004 3.457971531 -35.21905206 4.98E-116 1.10E-114 254.2504109

ACTN2 -1.729792718 1.1280414 -35.21365999 5.19E-116 1.15E-114 254.2091523

SIX5 -2.529433267 3.675161564 -35.20398871 5.59E-116 1.24E-114 254.1351414

DEPTOR 2.182803313 3.606827797 35.1931175 6.07E-116 1.34E-114 254.0519341

SH3YL1 -2.852002512 5.193123439 -35.19114117 6.17E-116 1.36E-114 254.0368059

STK33 -2.038523628 2.112197594 -35.19090437 6.18E-116 1.36E-114 254.0349932

LTBP1 3.202793224 3.701165713 35.17663839 6.89E-116 1.52E-114 253.9257766

TNFRSF1B 2.610442671 3.925893983 35.175558 6.95E-116 1.53E-114 253.9175044

ACAA1 -2.300843022 5.20009927 -35.15989195 7.83E-116 1.72E-114 253.7975382

UBE3D -1.191950533 1.504750449 -35.15416364 8.18E-116 1.80E-114 253.7536648

RAC2 3.172243302 4.029040166 35.15345353 8.23E-116 1.80E-114 253.7482258

PFKP 2.679514215 4.808211061 35.12951255 9.88E-116 2.16E-114 253.5648149

FDX2 -2.027399139 2.985273857 -35.12503989 1.02E-115 2.24E-114 253.5305423

DYRK2 1.451668298 3.17785426 35.1093762 1.15E-115 2.52E-114 253.4104964

CNOT6 1.24640175 3.100733948 35.10616037 1.18E-115 2.58E-114 253.3858467

ANTXR1 3.799387732 4.053212015 35.10570918 1.19E-115 2.58E-114 253.3823881

METTL3 -1.89432509 4.34693377 -35.07804487 1.47E-115 3.19E-114 253.1702836

DDT -2.971066072 5.931298065 -35.07328073 1.52E-115 3.30E-114 253.133747

TRMT10C 1.489217568 3.698122541 35.06824346 1.58E-115 3.43E-114 253.0951127

MMD 2.17947211 2.832742152 35.06423668 1.63E-115 3.53E-114 253.0643797

RPP21 -2.344918512 4.514119415 -35.04251887 1.92E-115 4.17E-114 252.8977642

ZNF20 -1.065223007 0.844855061 -35.04216967 1.93E-115 4.18E-114 252.8950847

FAM13A -2.376166313 3.784485194 -35.03580485 2.03E-115 4.38E-114 252.8462434

SPAST 1.226829423 2.715922306 35.01773398 2.33E-115 5.03E-114 252.7075472

SLC16A1 2.632721688 2.80195436 35.0151035 2.37E-115 5.12E-114 252.6873546

PRXL2B 2.323084284 4.255399125 34.97805205 3.15E-115 6.80E-114 252.4028417

CNOT1 -1.44945556 5.474753949 -34.97462016 3.24E-115 6.97E-114 252.3764801

ARMT1 1.40932207 3.257951531 34.96315633 3.54E-115 7.60E-114 252.2884117

ASPHD2 2.642622101 2.005974528 34.94413692 4.09E-115 8.79E-114 252.1422633

OBSCN -2.104309551 2.348606313 -34.93419175 4.42E-115 9.48E-114 252.0658252

ICE2 -1.3878417 2.981293034 -34.92815324 4.63E-115 9.92E-114 252.0194074

COMMD3 -2.094317198 4.832403701 -34.91130217 5.26E-115 1.13E-113 251.8898503

RPS15A -3.132198294 8.407833643 -34.90593751 5.49E-115 1.17E-113 251.8485975

CEP55 2.765189245 1.752818278 34.88785389 6.30E-115 1.35E-113 251.7095129

SDC3 2.096602322 3.768291576 34.83758783 9.28E-115 1.98E-113 251.3226945

TXNL4A -2.290993803 5.033348983 -34.83731111 9.30E-115 1.98E-113 251.3205641

TMPRSS4 4.725772602 2.913196931 34.83010217 9.83E-115 2.09E-113 251.2650625

CCL14 -3.128587197 2.415152292 -34.82603164 1.01E-114 2.16E-113 251.2337206

ERICH1 -1.540697241 2.910679159 -34.81569871 1.10E-114 2.33E-113 251.1541508

BCS1L -2.099570024 4.810000115 -34.81486502 1.10E-114 2.35E-113 251.1477302

AXL 2.872611943 3.584822318 34.8140168 1.11E-114 2.36E-113 251.1411978

TMEM120B -1.780650272 2.973146776 -34.80954107 1.15E-114 2.44E-113 251.106727

ZMPSTE24 1.692206662 4.527625424 34.80542279 1.19E-114 2.51E-113 251.0750069

SULF2 2.910169198 4.683838452 34.79076555 1.33E-114 2.81E-113 250.962096

KCTD11 1.655077588 3.716762463 34.78535412 1.39E-114 2.93E-113 250.9204028

RAB14 1.588420293 5.159955278 34.78356497 1.41E-114 2.96E-113 250.9066172

PHF13 1.444431489 3.522884833 34.77442068 1.51E-114 3.18E-113 250.8361532

TATDN1 -2.29029663 4.081191587 -34.76696737 1.60E-114 3.36E-113 250.7787119

TOP2A 3.146847178 2.343502642 34.75724518 1.72E-114 3.62E-113 250.7037744

COL10A1 5.52519926 3.3025942 34.75133684 1.80E-114 3.78E-113 250.6582279

ZNF260 1.190675564 2.360122756 34.74901112 1.83E-114 3.85E-113 250.6402981

LYRM4 -1.777871109 3.236714694 -34.7373662 2.01E-114 4.20E-113 250.5505131

BTBD10 1.416939605 3.749563728 34.73717881 2.01E-114 4.21E-113 250.5490682

SHPK -1.582207326 2.383554986 -34.73367106 2.06E-114 4.32E-113 250.5220192

RALBP1 1.654351204 4.372246677 34.72336229 2.23E-114 4.67E-113 250.4425176

STK25 -2.230430007 5.802580037 -34.72181296 2.26E-114 4.72E-113 250.4305679

NKTR -2.305771569 4.315520839 -34.71112083 2.46E-114 5.12E-113 250.3480938

COQ10B 1.524559804 4.112997368 34.6967032 2.74E-114 5.71E-113 250.2368605

ZNF839 -1.583321351 2.665635591 -34.67733942 3.19E-114 6.63E-113 250.0874265

HNRNPH1 -2.784750858 7.174089498 -34.66884306 3.40E-114 7.07E-113 250.0218439

PTBP2 -1.49475984 2.534177768 -34.6642399 3.52E-114 7.32E-113 249.9863087

FZD7 2.410384566 2.511690207 34.66285436 3.56E-114 7.39E-113 249.9756122

WDPCP -1.014249373 1.205783103 -34.65907557 3.67E-114 7.60E-113 249.9464384

MCM3 1.739325573 4.680242563 34.63921184 4.27E-114 8.84E-113 249.7930529

ANAPC1 -1.379639092 2.407149651 -34.6317818 4.53E-114 9.36E-113 249.7356665

MGAT2 -2.382723084 2.982293471 -34.62494275 4.77E-114 9.85E-113 249.6828385

PJA1 1.589946374 4.068459137 34.6241877 4.80E-114 9.90E-113 249.6770058

CDC42EP2 2.435012276 3.59719811 34.60495003 5.57E-114 1.15E-112 249.5283722

TAF1D -2.944128616 5.595105979 -34.5992013 5.82E-114 1.20E-112 249.4839476

AKT2 -2.072186823 5.01184381 -34.59509386 6.01E-114 1.24E-112 249.4522039

FGF12 -2.005805929 1.677517259 -34.5870605 6.39E-114 1.31E-112 249.3901134

SF3B4 1.789495542 5.401364952 34.5545922 8.21E-114 1.68E-112 249.1390814

CNPY2 -2.508609481 5.44903033 -34.55458643 8.21E-114 1.68E-112 249.1390368

TRIM52 -1.800288311 3.160828569 -34.54879735 8.59E-114 1.76E-112 249.0942642

HOXB7 3.165949447 2.672073344 34.54229039 9.03E-114 1.85E-112 249.0439346

C11orf98 -2.138999873 4.100015633 -34.5365632 9.44E-114 1.93E-112 248.9996318

PRAG1 2.139783958 2.925333056 34.52194103 1.06E-113 2.16E-112 248.8865036

MAML2 1.846404321 2.602916274 34.50174173 1.24E-113 2.52E-112 248.7301827

MMP23B -1.885284756 1.303556786 -34.49904685 1.26E-113 2.57E-112 248.7093234

BNIP3 -4.214309162 5.479871301 -34.49561885 1.30E-113 2.64E-112 248.6827882

HOOK2 -2.855245701 5.432336443 -34.49508944 1.30E-113 2.64E-112 248.6786901

AD000671.1 -1.912313972 1.053298831 -34.49417346 1.31E-113 2.66E-112 248.6715994

TTC21A -1.616224574 2.210840768 -34.48940072 1.36E-113 2.76E-112 248.6346517

ZNF558 -1.710509686 3.072509528 -34.48641317 1.39E-113 2.82E-112 248.6115223

RSKR -1.480000313 1.763597159 -34.48206776 1.44E-113 2.91E-112 248.5778786

CTSK 4.141170951 5.305578707 34.4767314 1.50E-113 3.03E-112 248.5365594

ENY2 -1.886703378 4.800284989 -34.47381615 1.53E-113 3.10E-112 248.5139853

NSMCE3 1.509295894 3.77399013 34.46363565 1.66E-113 3.35E-112 248.4351446

COQ10A -2.134108647 3.546883703 -34.45977878 1.71E-113 3.45E-112 248.4052726

LRMDA -2.101948251 2.696501859 -34.45622416 1.76E-113 3.54E-112 248.3777399

PARP6 -2.248092952 4.868303688 -34.44908858 1.86E-113 3.74E-112 248.3224657

CDC5L 1.256413184 3.453649946 34.44202092 1.96E-113 3.94E-112 248.2677114

BLOC1S3 1.354713387 3.236020933 34.43849366 2.02E-113 4.05E-112 248.2403829

MMP11 5.841007687 4.319189348 34.4362163 2.05E-113 4.11E-112 248.2227374

FAM153A -2.561254265 1.499936792 -34.4236981 2.26E-113 4.53E-112 248.1257327

TMTC3 1.372803495 2.221037119 34.40896064 2.53E-113 5.07E-112 248.0115057

AP002495.1 -0.99107423 1.146239037 -34.38239607 3.11E-113 6.22E-112 247.8055411

MRPS10 1.49796867 4.481211147 34.3687205 3.46E-113 6.91E-112 247.6994754

PAM16 -2.306547266 4.133498064 -34.36541331 3.55E-113 7.08E-112 247.6738217

MRPS11 -1.724078332 3.883277096 -34.35842428 3.75E-113 7.47E-112 247.619604

NQO1 3.965515103 4.955523197 34.35680241 3.79E-113 7.56E-112 247.6070214

AGBL3 -1.279525995 1.41200588 -34.34115304 4.28E-113 8.52E-112 247.4855954

MAP1A 2.145499295 1.977020507 34.30993681 5.46E-113 1.08E-111 247.2432925

KIF26B 2.404211994 1.663702458 34.29510399 6.12E-113 1.22E-111 247.1281166

KPNA2 2.178100418 4.012492398 34.27993602 6.89E-113 1.37E-111 247.0103099

SLC22A5 -1.621533825 2.72180576 -34.26285647 7.86E-113 1.56E-111 246.877622

ZMAT2 1.657519762 5.762835322 34.25271919 8.51E-113 1.68E-111 246.7988502

ACTR3B -1.475328969 2.353144121 -34.24861688 8.78E-113 1.74E-111 246.7669696

CDYL 1.229146839 2.995063728 34.24817763 8.81E-113 1.74E-111 246.7635558

CSTB 2.904580165 6.191280064 34.24167448 9.27E-113 1.83E-111 246.7130127

FOXQ1 3.843773335 3.326338084 34.23989007 9.40E-113 1.85E-111 246.6991431

RBM20 -3.882389827 2.166812787 -34.21842985 1.11E-112 2.19E-111 246.5323098

TOP1 1.774962971 5.060350396 34.21393295 1.15E-112 2.26E-111 246.4973434

CDH23 -2.359265939 2.002145968 -34.21390718 1.15E-112 2.26E-111 246.497143

KCNN4 4.508499412 3.42447719 34.19507018 1.33E-112 2.61E-111 246.350645

SHPRH -1.233655573 1.52503922 -34.18485134 1.44E-112 2.83E-111 246.2711531

COL5A1 4.225065275 4.928332758 34.17191026 1.59E-112 3.12E-111 246.1704664

NAGK -1.993281535 4.867798896 -34.17091606 1.61E-112 3.14E-111 246.1627303

AL121845.3 -1.366959748 1.365436969 -34.16985676 1.62E-112 3.17E-111 246.1544875

CDCA8 2.437295197 2.060724401 34.15778173 1.78E-112 3.47E-111 246.0605171

NDUFS7 -2.656935925 4.817511866 -34.15105509 1.87E-112 3.66E-111 246.0081612

SLC25A27 -2.887529671 3.476342047 -34.14788609 1.92E-112 3.74E-111 245.9834937

KIF4A 2.146328425 1.351170856 34.14738753 1.93E-112 3.76E-111 245.9796128

TIMP1 4.107202697 8.755727005 34.14715694 1.93E-112 3.76E-111 245.9778178

U2AF1L4 -2.264770768 3.941785865 -34.14060784 2.03E-112 3.95E-111 245.9268351

CENPT -2.316254688 4.784889567 -34.13519937 2.12E-112 4.12E-111 245.8847278

AC010547.4 -0.772733707 0.406283794 -34.13351089 2.15E-112 4.17E-111 245.8715816

CXXC1 -2.338360644 5.515841558 -34.12201962 2.35E-112 4.55E-111 245.7821025

CCDC149 -1.730060332 3.230274487 -34.11352535 2.51E-112 4.86E-111 245.7159497

PLEKHO2 2.246459428 3.711455204 34.10994232 2.58E-112 4.99E-111 245.6880425

ERG28 1.722763493 4.518644248 34.10581993 2.66E-112 5.15E-111 245.6559326

STS 2.113182887 2.613190191 34.10459036 2.69E-112 5.19E-111 245.6463548

MRPL22 -1.790423097 3.990469367 -34.09197699 2.97E-112 5.72E-111 245.548092

PTCD3 -1.656525098 4.107178778 -34.08807747 3.06E-112 5.89E-111 245.5177093

PYGB 2.708602128 5.807196681 34.0861246 3.10E-112 5.98E-111 245.502493

MYOF 2.90406642 4.425147051 34.05780561 3.87E-112 7.44E-111 245.2817848

HIST2H2AA3 -3.630000123 1.79539683 -34.05536614 3.94E-112 7.58E-111 245.2627677

DEXI -1.688290445 3.739376988 -34.05410539 3.98E-112 7.65E-111 245.2529392

VASP 2.535163021 5.719012738 34.04515306 4.27E-112 8.19E-111 245.1831429

PTTG1IP 2.33187895 7.107209236 34.04016306 4.44E-112 8.51E-111 245.1442344

FBXO34 1.678951693 3.740131633 34.03928482 4.47E-112 8.56E-111 245.1373862

SULF1 4.33949466 4.163636787 34.02238849 5.10E-112 9.75E-111 245.0056155

MYADM 2.672433977 5.776643855 34.02213064 5.11E-112 9.76E-111 245.0036043

ENO3 -1.867131097 2.350091641 -33.99893676 6.12E-112 1.17E-110 244.8226618

LMO3 -3.06392877 2.504998843 -33.98866547 6.63E-112 1.26E-110 244.7425109

ZNHIT3 -1.798807456 3.95349563 -33.98578228 6.78E-112 1.29E-110 244.7200099

PDCD4 -3.296150089 7.080710733 -33.9772495 7.25E-112 1.38E-110 244.6534122

TTYH1 -3.281075061 2.894528066 -33.97081354 7.62E-112 1.45E-110 244.6031741

HENMT1 2.155062201 2.280192091 33.95598514 8.56E-112 1.63E-110 244.487406

LSMEM1 -2.050634667 2.154044997 -33.95239616 8.80E-112 1.67E-110 244.4593821

ELK1 1.389046476 4.10777829 33.95179658 8.84E-112 1.68E-110 244.4547002

MINDY4 -1.236103868 1.472762946 -33.94178199 9.56E-112 1.81E-110 244.3764938

TMEM87B 1.58733674 4.081556359 33.93703829 9.92E-112 1.88E-110 244.3394447

INTS10 -2.119446989 5.033807197 -33.93029342 1.05E-111 1.98E-110 244.2867612

ATP6V1G2-DDX39B -2.755439986 1.351425297 -33.92736722 1.07E-111 2.02E-110 244.2639032

CYB5RL -1.247515816 1.535816516 -33.92307681 1.11E-111 2.09E-110 244.2303868

PMFBP1 -1.620608201 1.241647703 -33.91319125 1.19E-111 2.25E-110 244.1531526

MMP28 3.516272083 3.080541837 33.91312469 1.20E-111 2.25E-110 244.1526325

ZDHHC5 1.480854243 5.003203305 33.90030449 1.32E-111 2.49E-110 244.0524522

PROB1 0.985857474 1.044455414 33.88164375 1.53E-111 2.87E-110 243.9065958

MCF2L -2.33425045 3.52110766 -33.87255517 1.64E-111 3.08E-110 243.8355418

TMEM45B 3.597591293 2.974270158 33.86594442 1.73E-111 3.24E-110 243.7838528

ZNF326 -1.547441614 3.301206715 -33.86059953 1.80E-111 3.38E-110 243.7420576

CDCA4 1.665879375 2.711001883 33.83733795 2.16E-111 4.04E-110 243.5601181

MCM9 -1.200249536 2.197485626 -33.80679575 2.74E-111 5.13E-110 243.321131

DMTF1 -2.1234413 4.484643937 -33.79918099 2.91E-111 5.44E-110 243.2615288

PRRG4 2.090819071 2.564927425 33.79653508 2.97E-111 5.55E-110 243.2408171

DNTTIP1 1.992673043 4.759886655 33.79097523 3.10E-111 5.79E-110 243.1972926

AP1G2 -2.665286358 5.416192079 -33.78686743 3.21E-111 5.97E-110 243.1651328

CSAD -2.552849094 4.208053757 -33.78018428 3.38E-111 6.29E-110 243.1128062

SH3PXD2A 2.080043967 3.969470897 33.77889152 3.41E-111 6.34E-110 243.1026838

MKI67 2.717164859 1.85256796 33.76375377 3.84E-111 7.13E-110 242.9841377

ZNF577 -1.853372136 2.61397945 -33.73371534 4.86E-111 9.02E-110 242.7488175

PPAN -2.13774191 3.566257926 -33.72194072 5.33E-111 9.88E-110 242.6565447

SKIL 2.211704894 3.700215426 33.71059705 5.82E-111 1.08E-109 242.5676328

NSDHL 1.637668582 4.065425487 33.69569294 6.54E-111 1.21E-109 242.4507895

HSD17B4 -2.32130165 5.845922026 -33.66379894 8.40E-111 1.55E-109 242.200658

TGFBR1 2.058640426 3.786568728 33.65813725 8.78E-111 1.62E-109 242.1562423

LRRC8E -1.520508286 1.313756241 -33.6512033 9.27E-111 1.71E-109 242.1018405

EPSTI1 2.637789552 2.757103491 33.63929429 1.02E-110 1.88E-109 242.0083917

NPIPB9 -1.020031329 0.527630626 -33.63599757 1.04E-110 1.92E-109 241.9825196

GRAMD1C -1.84924492 2.853937194 -33.63217707 1.08E-110 1.98E-109 241.9525351

ELP2 -1.85954759 4.372039509 -33.62593468 1.13E-110 2.08E-109 241.9035391

GNS 1.925236965 5.192261885 33.61305695 1.25E-110 2.30E-109 241.8024474

PDHA1 -2.162725048 5.754336706 -33.60845007 1.30E-110 2.38E-109 241.7662778

RTEL1-TNFRSF6B -1.744971167 1.933939138 -33.59151996 1.48E-110 2.71E-109 241.6333331

ZNF559-ZNF177 -1.099168877 0.770729543 -33.59108874 1.49E-110 2.72E-109 241.6299465

ASPN 3.972352663 4.092541195 33.56715205 1.79E-110 3.28E-109 241.4419199

POP4 -1.91538877 4.198951191 -33.56339639 1.85E-110 3.37E-109 241.412412

ACSL5 3.577006453 4.095589512 33.55877644 1.91E-110 3.50E-109 241.3761112

ZMYM6 -1.180112181 2.578134574 -33.54397277 2.15E-110 3.92E-109 241.2597745

INTS11 -2.21648257 5.589004776 -33.54335451 2.16E-110 3.94E-109 241.2549152

MRPL44 1.407352407 4.352338011 33.54293769 2.17E-110 3.95E-109 241.2516392

C1GALT1C1 1.547854265 4.435493991 33.51761745 2.64E-110 4.81E-109 241.0525899

HLA-DQB2 3.562239269 2.348804656 33.50877139 2.83E-110 5.15E-109 240.9830297

RIOX2 -1.606076139 3.556359589 -33.50856165 2.84E-110 5.16E-109 240.9813803

KRT19 5.777664742 7.994315159 33.50681261 2.88E-110 5.22E-109 240.9676257

DCK 1.658028867 3.194596202 33.49857475 3.07E-110 5.57E-109 240.9028373

HLA-E 2.796915178 8.272210886 33.49764261 3.09E-110 5.60E-109 240.8955057

BNIP3L 1.812316682 4.702252605 33.49392995 3.19E-110 5.76E-109 240.8663034

PLAU 4.26363719 4.704345221 33.49254942 3.22E-110 5.82E-109 240.8554443

CDC20 3.237884894 2.472672128 33.48536093 3.41E-110 6.15E-109 240.7988965

METAP1D -1.437460852 2.635161141 -33.48485897 3.42E-110 6.17E-109 240.7949477

PDZD11 1.499223715 4.91521045 33.47676963 3.64E-110 6.57E-109 240.7313051

ZBTB2 1.212386762 3.013531466 33.45719761 4.25E-110 7.66E-109 240.5772891

RPS10-NUDT3 -2.337728527 1.261950847 -33.45181085 4.43E-110 7.98E-109 240.5348913

UMOD -2.147799228 1.231504225 -33.43510426 5.06E-110 9.09E-109 240.4033748

PHKA2 -2.011153873 4.213391283 -33.43228681 5.17E-110 9.29E-109 240.3811921

ALDH1L1 -3.506587635 3.26319362 -33.42561224 5.45E-110 9.78E-109 240.3286369

BHLHA15 -5.498605379 4.714144555 -33.42036798 5.68E-110 1.02E-108 240.28734

GTF2H5 1.168830201 2.328947131 33.4178144 5.79E-110 1.04E-108 240.2672301

CEACAM6 7.314269107 5.926288584 33.40550148 6.38E-110 1.14E-108 240.1702522

ABT1 1.287440436 3.71086071 33.38656856 7.41E-110 1.32E-108 240.0210974

CCDC159 -2.13785112 4.206733895 -33.3790923 7.86E-110 1.40E-108 239.9621866

RTRAF -2.137226452 5.818762039 -33.36840634 8.55E-110 1.53E-108 239.8779721

PYGO2 1.379514845 4.535475468 33.3675846 8.60E-110 1.53E-108 239.8714955

PRELID3B 1.723423507 4.881119756 33.36312432 8.91E-110 1.59E-108 239.83634

RFLNA 3.718294972 2.400838639 33.35342889 9.62E-110 1.71E-108 239.7599129

CKAP2 1.705298565 2.190588126 33.35241249 9.69E-110 1.72E-108 239.7519002

PRR4 -1.842073047 1.649511274 -33.34756734 1.01E-109 1.79E-108 239.7137019

RPAIN -1.740572795 3.713150515 -33.34358946 1.04E-109 1.84E-108 239.6823388

IPPK -1.237753911 1.86441165 -33.33110146 1.15E-109 2.03E-108 239.5838658

ARNTL2 2.411451444 2.294719611 33.32838257 1.17E-109 2.08E-108 239.5624236

C19orf57 -1.816538874 1.90792992 -33.30582461 1.40E-109 2.48E-108 239.3844877

ZSCAN2 -1.668866295 2.647983738 -33.29927617 1.47E-109 2.61E-108 239.3328221

ARMCX4 -1.534704071 2.419073787 -33.29343988 1.54E-109 2.73E-108 239.2867706

BOLA2-SMG1P6 -1.839089063 2.411304938 -33.28389553 1.66E-109 2.94E-108 239.2114513

MPV17 -1.877963329 4.883398363 -33.28343971 1.67E-109 2.94E-108 239.2078539

VXN -2.110555052 1.295131301 -33.26527939 1.93E-109 3.39E-108 239.0645092

MZT1 1.539768014 3.264763568 33.25861174 2.03E-109 3.57E-108 239.0118691

C12orf76 -1.606013907 2.757491646 -33.24764522 2.21E-109 3.89E-108 238.925278

ACSM3 -3.222097603 3.911177987 -33.24266862 2.30E-109 4.05E-108 238.885978

AC024592.3 -1.036102345 0.508100225 -33.23393589 2.47E-109 4.33E-108 238.8170086

ZNF543 1.007570962 1.359344498 33.23003754 2.54E-109 4.46E-108 238.786217

SERINC3 1.694683476 5.702233365 33.20975525 2.98E-109 5.23E-108 238.6259846

TRMT9B -1.52951193 1.545449348 -33.20128255 3.19E-109 5.59E-108 238.559034

XKR6 -1.516931648 1.389602471 -33.17546115 3.91E-109 6.85E-108 238.3549401

CEP63 -1.407770669 3.157481315 -33.17370024 3.97E-109 6.94E-108 238.3410187

PSTK -1.327677048 1.596045332 -33.16357974 4.30E-109 7.51E-108 238.2610006

SFXN2 -1.919942103 2.630077362 -33.15967511 4.43E-109 7.74E-108 238.2301251

CHSY1 2.047537285 3.519398241 33.11333058 6.39E-109 1.11E-107 237.8635139

CHN2 -2.258781956 2.939868767 -33.1052953 6.81E-109 1.19E-107 237.799923

CBFA2T3 -2.718197241 3.151319764 -33.10086268 7.05E-109 1.23E-107 237.7648399

COL8A2 3.342672558 2.512350355 33.10061798 7.07E-109 1.23E-107 237.7629031

DEGS1 1.648854361 4.846120401 33.09415211 7.44E-109 1.29E-107 237.7117227

PEX5L -1.810338822 1.048012311 -33.09370761 7.47E-109 1.30E-107 237.7082041

FXYD2 -5.143009056 5.565214481 -33.09159634 7.59E-109 1.32E-107 237.6914911

RRAS 2.628367215 5.948045697 33.0871107 7.87E-109 1.36E-107 237.6559807

PRR5-ARHGAP8 -1.085845773 0.74211866 -33.08414921 8.05E-109 1.39E-107 237.6325348

ADAM19 2.831889149 2.23019844 33.08140932 8.23E-109 1.42E-107 237.6108423

DNAH7 -1.330119185 1.015395573 -33.08011665 8.31E-109 1.44E-107 237.6006075

TMCC3 1.913606561 2.248501036 33.07830594 8.43E-109 1.46E-107 237.5862708

FOXS1 2.802633417 2.260930599 33.06370383 9.46E-109 1.63E-107 237.4706401

HECA 1.536285496 3.478657345 33.05334989 1.03E-108 1.77E-107 237.3886335

CARD8 -1.605616397 3.290283764 -33.05110664 1.05E-108 1.80E-107 237.3708644

PFDN2 2.103220801 6.172523404 33.04325815 1.11E-108 1.91E-107 237.3086906

RBBP5 1.157537213 3.119858116 33.03901987 1.15E-108 1.98E-107 237.2751128

CLIC1 2.829831382 8.224834629 33.03750203 1.16E-108 2.00E-107 237.2630871

NAB2 1.919949463 3.732961001 33.0112605 1.43E-108 2.46E-107 237.0551332

PRODH2 -2.413512339 1.383595038 -33.00963248 1.45E-108 2.49E-107 237.0422289

KCTD12 2.670280272 4.084611249 32.99488516 1.63E-108 2.80E-107 236.9253213

MDK 3.239456764 6.168136574 32.98546294 1.76E-108 3.01E-107 236.8506135

BST2 3.81996379 6.281090223 32.98167089 1.81E-108 3.10E-107 236.8205435

TTLL7 -2.268853247 2.514680237 -32.97019031 1.98E-108 3.39E-107 236.7294947

AMY2B -7.418621826 6.419203375 -32.96578741 2.05E-108 3.51E-107 236.6945723

P2RX1 -3.862203813 3.589838321 -32.96480115 2.07E-108 3.53E-107 236.6867493

HLA-C 2.986264444 8.953614212 32.95937738 2.16E-108 3.68E-107 236.6437256

GNG12 2.03002574 4.926167162 32.95904708 2.17E-108 3.69E-107 236.6411055

S100A16 3.797187344 6.816984574 32.94393386 2.44E-108 4.16E-107 236.521201

C19orf33 5.706268856 5.330393238 32.93693792 2.58E-108 4.39E-107 236.4656872

DENND10 -1.404597712 2.956755814 -32.93367827 2.65E-108 4.50E-107 236.4398194

APOBR 2.334758316 2.255840163 32.93359201 2.65E-108 4.50E-107 236.4391349

ACBD3 1.713058958 4.729490018 32.92431947 2.85E-108 4.84E-107 236.3655426

ZNF451 -1.323224001 2.856697785 -32.91002897 3.20E-108 5.41E-107 236.2521039

COG8 -1.369061552 3.279740414 -32.90893299 3.22E-108 5.46E-107 236.2434029

EIF3M -2.392296946 6.335263737 -32.90884015 3.23E-108 5.46E-107 236.2426658

PRMT7 -1.719249743 3.754646272 -32.90636511 3.29E-108 5.56E-107 236.2230159

OGG1 -1.306418779 2.920552404 -32.90004851 3.46E-108 5.84E-107 236.1728633

SCART1 -1.586505335 1.440945642 -32.8979755 3.52E-108 5.93E-107 236.1564029

FUNDC1 1.477673535 3.798152466 32.890005 3.75E-108 6.31E-107 236.0931096

ZSWIM8 -2.096655433 5.139975162 -32.87578319 4.19E-108 7.06E-107 235.980155

AP2B1 1.627737024 5.504191566 32.87467748 4.23E-108 7.12E-107 235.971372

NADSYN1 -2.027441299 4.354937498 -32.87334397 4.28E-108 7.19E-107 235.9607793

GDF10 -2.694372045 1.918544975 -32.86877134 4.43E-108 7.45E-107 235.9244551

ACSL4 1.906975691 3.709949369 32.83727133 5.69E-108 9.55E-107 235.674153

CYP51A1 -2.33696088 3.149945126 -32.83520357 5.79E-108 9.70E-107 235.657718

AL157935.2 -1.838538157 1.874506686 -32.82845156 6.11E-108 1.02E-106 235.604048

LARP1B -2.049470374 3.687035677 -32.82215409 6.42E-108 1.07E-106 235.5539858

CDS1 2.061202186 3.212515145 32.82137528 6.46E-108 1.08E-106 235.5477942

SIDT2 -2.532008746 5.216828296 -32.81768466 6.65E-108 1.11E-106 235.5184527

CMTM3 2.874031817 4.202728002 32.81253183 6.93E-108 1.16E-106 235.4774833

ARL3 1.495595473 3.311599618 32.80949135 7.10E-108 1.18E-106 235.4533073

PKD1 -2.194497246 4.250494393 -32.7946779 7.99E-108 1.33E-106 235.3355036

PASK -1.492117503 2.59493709 -32.77355906 9.44E-108 1.57E-106 235.1675088

NAA10 -2.175772843 5.073946664 -32.7618042 1.04E-107 1.73E-106 235.0739777

IFNGR2 2.033629263 5.611303299 32.75601083 1.09E-107 1.80E-106 235.0278747

ACAT1 -2.52333832 4.971718233 -32.7219505 1.42E-107 2.36E-106 234.7567408

ZNF286A -1.262361546 1.651307163 -32.71303351 1.53E-107 2.54E-106 234.685734

ZNF181 -1.555685945 2.680173092 -32.71059142 1.56E-107 2.58E-106 234.6662857

SH3RF1 1.939133707 3.615354495 32.71027221 1.56E-107 2.59E-106 234.6637435

SYNE2 -2.383356174 4.804887321 -32.69580041 1.75E-107 2.90E-106 234.5484769

RAD51C -1.435918214 2.798407815 -32.69399302 1.78E-107 2.94E-106 234.5340793

GSTZ1 -1.91105563 3.118715749 -32.68004598 1.99E-107 3.28E-106 234.4229644

DYNLT3 1.701458321 4.193612781 32.67279569 2.10E-107 3.48E-106 234.3651923

GRB2 1.544612463 5.33206039 32.66827115 2.18E-107 3.60E-106 234.3291363

SLC45A3 3.061571249 2.716712217 32.667741 2.19E-107 3.61E-106 234.3249114

HK2 3.378903715 3.275114626 32.65250506 2.47E-107 4.07E-106 234.2034765

BMT2 1.05367369 2.385770543 32.65228504 2.48E-107 4.08E-106 234.2017226

INPP5J -2.137156569 2.577440048 -32.63716771 2.80E-107 4.60E-106 234.0812039

FBXO28 1.285079345 3.325226706 32.63649024 2.81E-107 4.62E-106 234.0758023

STK26 1.825956359 2.701685653 32.63544848 2.83E-107 4.65E-106 234.0674959

DPY19L1 1.695418654 3.325150559 32.62525352 3.07E-107 5.04E-106 233.986201

ZNF765 -1.107558762 1.918165093 -32.624317 3.10E-107 5.07E-106 233.9787326

INTS8 -1.753510947 4.01692526 -32.62286586 3.13E-107 5.13E-106 233.9671599

SMIM7 -1.929947132 5.01582464 -32.61971827 3.21E-107 5.25E-106 233.9420573

FAN1 -1.545226996 3.129060081 -32.61009167 3.47E-107 5.67E-106 233.8652759

SMIM15 1.611428295 4.690043688 32.61004819 3.47E-107 5.67E-106 233.864929

SMIM11A -2.385418367 1.167858348 -32.60026217 3.75E-107 6.12E-106 233.7868641

PELO 1.303311672 3.424584754 32.5937644 3.95E-107 6.44E-106 233.7350235

ZCCHC3 1.313005258 3.518112063 32.57531896 4.58E-107 7.45E-106 233.587833

PPP1R3B 1.951764709 3.094481876 32.57376838 4.63E-107 7.54E-106 233.5754577

PTPRJ 1.447316753 3.316437354 32.56834612 4.84E-107 7.87E-106 233.5321802

GPSM3 2.623784294 3.963992664 32.56691037 4.89E-107 7.95E-106 233.5207201

KIAA0232 1.332761081 3.299079329 32.56394331 5.01E-107 8.13E-106 233.4970365

TBC1D30 -1.955473231 2.875307816 -32.55077899 5.56E-107 9.03E-106 233.3919432

AC009690.1 -0.916881708 0.615949524 -32.5355945 6.28E-107 1.02E-105 233.2706956

APBB3 -2.092623361 3.876219716 -32.53390018 6.37E-107 1.03E-105 233.2571647

S100A11 4.434262912 9.611232299 32.52472732 6.85E-107 1.11E-105 233.1839037

TRAPPC12 -1.794992949 4.24541764 -32.51106814 7.64E-107 1.24E-105 233.0747923

IL3RA 1.995158388 3.119407284 32.49823745 8.46E-107 1.37E-105 232.9722776

ARID5B 2.081068366 2.894573665 32.49485924 8.69E-107 1.40E-105 232.9452829

SPCS2 -2.203398705 5.954794734 -32.49460675 8.71E-107 1.41E-105 232.9432653

ASPSCR1 -2.007833084 3.506824439 -32.49236238 8.87E-107 1.43E-105 232.92533

C16orf91 1.409664284 3.663031544 32.48984953 9.05E-107 1.46E-105 232.9052485

MASP2 -1.245481076 1.08577141 -32.47892844 9.87E-107 1.59E-105 232.8179634

LUZP1 1.431810409 2.768289823 32.46801039 1.08E-106 1.73E-105 232.7306876

TSR2 1.542118663 5.289621149 32.45735822 1.17E-106 1.88E-105 232.6455227

GJB2 4.605588752 3.495513615 32.45459505 1.20E-106 1.92E-105 232.6234286

GOLGA8A -3.331769794 4.478212524 -32.45270958 1.22E-106 1.95E-105 232.608352

USP8 -1.397602223 3.228139616 -32.44888367 1.25E-106 2.01E-105 232.5777579

SORBS2 -2.830842198 4.666129704 -32.44783048 1.27E-106 2.03E-105 232.5693357

PPP2R2D -2.623992311 4.442800897 -32.4405341 1.34E-106 2.15E-105 232.5109836

FITM1 -2.520389997 2.082819236 -32.43550285 1.40E-106 2.23E-105 232.4707428

PLPP5 -2.525960532 5.739661179 -32.43297679 1.42E-106 2.28E-105 232.4505378

PCBP2 -2.436012385 7.261543488 -32.41416026 1.66E-106 2.64E-105 232.3000059

SEMA7A 3.540938096 2.810957838 32.40955985 1.72E-106 2.74E-105 232.2631959

PRSS16 -2.564183416 3.774515137 -32.40576013 1.77E-106 2.82E-105 232.2327906

ATXN2 -1.56850142 3.517387415 -32.39238354 1.97E-106 3.14E-105 232.125737

HLA-B 3.393696155 9.364475126 32.36895396 2.38E-106 3.78E-105 231.9381746

REPS1 -1.542938609 3.659904213 -32.3648158 2.46E-106 3.91E-105 231.9050399

RAB32 2.259546551 4.425044355 32.36333688 2.49E-106 3.95E-105 231.8931975

PDRG1 1.383448421 3.826603701 32.36180088 2.52E-106 3.99E-105 231.8808978

GRPEL2 1.083784592 2.597249335 32.35640388 2.63E-106 4.17E-105 231.8376782

STK36 -2.098128156 4.216373539 -32.33415302 3.14E-106 4.98E-105 231.659453

HCCS 1.345639333 3.752318405 32.33139376 3.21E-106 5.08E-105 231.6373475

CARS2 -1.82760191 4.211151346 -32.31751773 3.59E-106 5.67E-105 231.5261669

ANGPTL2 2.928152804 3.737667409 32.30703238 3.90E-106 6.17E-105 231.4421377

CEACAM1 2.967707796 3.19001181 32.30650862 3.92E-106 6.19E-105 231.43794

EMC6 -2.33365469 3.843542719 -32.30622296 3.93E-106 6.20E-105 231.4356505

DAPP1 2.342206849 1.695865514 32.3052107 3.96E-106 6.24E-105 231.4275375

BMS1 1.087381769 3.516783712 32.30488847 3.97E-106 6.25E-105 231.4249549

PVRIG -1.685635815 1.183260018 -32.30297296 4.03E-106 6.34E-105 231.409602

RNF215 -1.510453055 2.873735743 -32.29837147 4.18E-106 6.58E-105 231.3727192

NPIPB6 -1.020060515 0.601748525 -32.29619865 4.25E-106 6.69E-105 231.3553022

HPSE 1.689088308 1.473070101 32.29430777 4.32E-106 6.78E-105 231.3401448

PRDM1 2.074426043 2.122464998 32.2913051 4.42E-106 6.94E-105 231.3160742

POC1A 1.965983234 1.894816836 32.27974618 4.85E-106 7.61E-105 231.2234027

ANKRD50 1.770802946 2.495103605 32.26457143 5.48E-106 8.59E-105 231.1017164

MIPOL1 -1.51120703 1.534663374 -32.26234263 5.58E-106 8.73E-105 231.0838413

GOLGA8T -0.867921437 0.457538543 -32.25778627 5.79E-106 9.05E-105 231.047297

MGLL 2.575128169 4.124728261 32.24676271 6.32E-106 9.88E-105 230.9588716

OBSL1 -2.479725807 5.447124571 -32.24449671 6.44E-106 1.01E-104 230.940693

SMARCE1 -1.728115842 4.423825866 -32.2330816 7.05E-106 1.10E-104 230.8491075

FAHD2B -1.823135543 3.054059179 -32.2266648 7.43E-106 1.16E-104 230.7976172

UBAP1 1.575231235 5.047935412 32.2245644 7.55E-106 1.18E-104 230.7807618

TMEM43 1.813965391 5.287169125 32.21960511 7.86E-106 1.22E-104 230.7409622

PNISR -2.382654605 5.120890167 -32.21846145 7.93E-106 1.23E-104 230.7317835

TRIM3 -1.672181797 3.313968782 -32.21832001 7.94E-106 1.23E-104 230.7306483

SUGT1 -1.631390015 3.799325943 -32.21434893 8.20E-106 1.27E-104 230.6987765

PACS2 -1.965326252 4.833024264 -32.20024764 9.18E-106 1.42E-104 230.5855836

AGAP6 -2.072976323 3.454804667 -32.19121578 9.87E-106 1.53E-104 230.5130704

SEMA3C 3.6930139 2.863723707 32.17905885 1.09E-105 1.69E-104 230.4154513

ENOPH1 1.433809461 4.686238991 32.16805504 1.19E-105 1.84E-104 230.3270756

PROCR 2.440605774 4.258056237 32.16447546 1.22E-105 1.89E-104 230.2983234

ZKSCAN5 0.932641746 2.199587046 32.15869245 1.28E-105 1.98E-104 230.2518692

IZUMO4 -2.622586279 2.919578378 -32.14524702 1.43E-105 2.20E-104 230.1438475

ICAM3 -2.4369555 2.429271319 -32.13836866 1.51E-105 2.33E-104 230.0885774

GPR68 2.449904886 2.016621373 32.12978693 1.62E-105 2.49E-104 230.0196118

PLCXD2 -2.316674866 2.071689238 -32.11273595 1.85E-105 2.85E-104 229.8825571

USP44 -1.20455455 1.145780553 -32.11038715 1.89E-105 2.91E-104 229.8636747

CEP164 -1.944919789 3.730897716 -32.10393756 1.99E-105 3.06E-104 229.8118219

NDC80 2.005421339 1.512032544 32.0964048 2.11E-105 3.25E-104 229.7512541

RFTN1 2.231712789 3.388087284 32.09015966 2.22E-105 3.41E-104 229.7010341

LRRC37A -1.222627451 0.822971394 -32.07450786 2.52E-105 3.86E-104 229.5751494

NGLY1 -1.495844635 3.630716743 -32.07339312 2.54E-105 3.90E-104 229.5661826

TIPRL 1.335939006 4.192439171 32.0660094 2.70E-105 4.13E-104 229.5067849

PABPC4 -2.744702191 6.910481894 -32.06170913 2.79E-105 4.27E-104 229.4721886

ZNF90 -1.450093787 1.387179896 -32.05512746 2.94E-105 4.50E-104 229.4192336

NDUFA13 -2.319692697 5.753596298 -32.04837027 3.11E-105 4.75E-104 229.3648607

HM13 -2.366811974 6.450869955 -32.03064534 3.58E-105 5.47E-104 229.222207

PAFAH1B2 1.270550311 4.504076118 32.02676872 3.70E-105 5.64E-104 229.1910018

MTA1 -2.130986957 5.073965387 -32.02173792 3.85E-105 5.87E-104 229.1505033

LRRC42 1.4867594 4.173214799 32.02060339 3.88E-105 5.92E-104 229.1413698

SRSF10 -1.775722405 4.961972287 -32.01869272 3.94E-105 6.01E-104 229.1259875

TMSB15B -0.720846419 0.455273914 -32.01625176 4.02E-105 6.12E-104 229.1063354

SPG7 -1.981661753 4.851011133 -32.01602845 4.03E-105 6.13E-104 229.1045375

HEPH 2.831187767 2.693673546 32.01159756 4.18E-105 6.34E-104 229.0688626

CAP1 2.310040479 6.821366459 32.00120706 4.54E-105 6.89E-104 228.9851945

GSK3B 1.221727324 3.484560932 31.98238053 5.28E-105 8.01E-104 228.833562

ZYG11B 1.144513208 2.632396392 31.97911172 5.42E-105 8.22E-104 228.8072299

GNMT -5.860587468 4.632827089 -31.97593698 5.56E-105 8.43E-104 228.7816542

UBE2F -1.972866793 4.940815502 -31.97184367 5.75E-105 8.70E-104 228.7486766

RAB24 -2.835272921 4.730702246 -31.96884629 5.89E-105 8.91E-104 228.7245271

NBPF15 -1.858300298 3.325485109 -31.96493295 6.08E-105 9.18E-104 228.6929961

ZNF775 -1.91504972 3.449786925 -31.96036228 6.31E-105 9.52E-104 228.6561663

SEPHS2 1.805358672 5.44703536 31.94829102 6.95E-105 1.05E-103 228.5588852

SPDYE2 -1.061838228 0.703458533 -31.94413774 7.19E-105 1.08E-103 228.5254101

TSGA10 -1.53817597 1.989903645 -31.94263837 7.27E-105 1.10E-103 228.5133248

IGFLR1 -2.180095019 2.355604332 -31.9370866 7.61E-105 1.14E-103 228.4685736

HERC2 -1.584900876 3.745737849 -31.93106058 7.98E-105 1.20E-103 228.4199952

LIG3 -1.478138004 2.926979623 -31.92795036 8.19E-105 1.23E-103 228.3949205

KCNN2 -1.649895294 1.355253423 -31.92462122 8.41E-105 1.26E-103 228.3680796

ITGAE -1.544113725 3.18660817 -31.92421928 8.44E-105 1.27E-103 228.3648389

CRIPT 1.180410486 3.125214171 31.92107152 8.65E-105 1.30E-103 228.3394589

TRAF3 1.333084556 2.692772097 31.91782008 8.88E-105 1.33E-103 228.3132417

PPIE -1.720262889 4.375010497 -31.91252112 9.27E-105 1.39E-103 228.2705118

PGAM2 -1.219227125 0.591892231 -31.90151675 1.01E-104 1.52E-103 228.1817633

IL27RA 1.881480607 2.663420232 31.88150875 1.19E-104 1.78E-103 228.0203631

AKIRIN2 1.363109595 4.385392757 31.87636837 1.24E-104 1.85E-103 227.9788887

PRSS53 -1.311287718 1.355718475 -31.87496975 1.25E-104 1.87E-103 227.9676035

KIF5B 1.748658475 5.245604923 31.87432943 1.26E-104 1.88E-103 227.9624368

PDCD2 -1.805981038 4.546931799 -31.87044327 1.30E-104 1.94E-103 227.9310787

C1orf216 1.379728551 2.923312801 31.8599665 1.42E-104 2.11E-103 227.8465302

GJA1 2.943318427 4.539576106 31.85830569 1.43E-104 2.14E-103 227.8331261

SLC30A1 1.791513359 3.516863951 31.84926438 1.54E-104 2.29E-103 227.760149

EIF2AK2 1.403820449 3.267617761 31.84383982 1.61E-104 2.40E-103 227.7163596

CETN2 1.6776794 5.052609065 31.84356161 1.62E-104 2.40E-103 227.7141136

PRNP 2.114624828 5.250156296 31.83986462 1.66E-104 2.47E-103 227.6842677

CLEC11A 2.586227083 4.102852314 31.83650938 1.71E-104 2.54E-103 227.6571793

GSTO2 -2.133027564 3.556966347 -31.83453356 1.74E-104 2.57E-103 227.6412269

GPRC5A 4.931124426 4.340221337 31.83296074 1.76E-104 2.61E-103 227.6285278

MSLN 6.500017437 3.984406084 31.8323315 1.77E-104 2.62E-103 227.6234472

BTBD1 1.382606656 4.397978534 31.8257131 1.87E-104 2.76E-103 227.5700061

CBR3 1.817433428 2.146219858 31.80788652 2.15E-104 3.18E-103 227.4260357

GALNT5 3.527859005 1.991882404 31.80657327 2.18E-104 3.21E-103 227.4154281

ZNF431 -1.447292259 2.309361072 -31.80491065 2.21E-104 3.25E-103 227.4019982

CYHR1 -2.142791024 4.855335779 -31.80162841 2.27E-104 3.34E-103 227.3754848

CCNA2 2.168728602 2.154616616 31.79944258 2.31E-104 3.40E-103 227.3578271

APOBEC3D 1.649906597 1.641929224 31.76833287 2.96E-104 4.36E-103 227.1064515

FERMT1 2.998446928 2.957457301 31.76626055 3.01E-104 4.43E-103 227.0897022

RCOR3 -1.619280201 4.007064998 -31.75516439 3.30E-104 4.84E-103 227.0000097

CBFB 1.451429521 4.432429231 31.75381495 3.33E-104 4.89E-103 226.9891008

KIF2C 2.252148244 1.762918806 31.74663415 3.53E-104 5.18E-103 226.9310475

WDR59 -1.784226081 4.228998092 -31.74295641 3.64E-104 5.33E-103 226.9013122

RAB3IP -1.902258288 3.409143945 -31.74223485 3.66E-104 5.36E-103 226.895478

SAMD8 1.208571932 2.788633352 31.7411843 3.69E-104 5.40E-103 226.8869837

CTSE 7.098452415 5.231015941 31.72307631 4.27E-104 6.25E-103 226.7405481

FAM220A 1.190612277 3.561053458 31.71273588 4.65E-104 6.79E-103 226.6569087

DYNLL2 1.51254043 4.482329738 31.70663342 4.88E-104 7.13E-103 226.6075422

PLEKHB2 1.420418478 4.714817395 31.70464552 4.96E-104 7.24E-103 226.5914599

LEO1 1.353966099 3.634493759 31.70287905 5.03E-104 7.34E-103 226.5771685

SERPINB5 4.105178965 2.433920505 31.66028169 7.10E-104 1.03E-102 226.2324221

MEIS1 -2.406265428 4.360171011 -31.65622345 7.33E-104 1.07E-102 226.1995664

CHRM3 -2.219064397 1.963284954 -31.64291899 8.17E-104 1.19E-102 226.0918381

MMP14 3.629661627 6.712819749 31.64158671 8.26E-104 1.20E-102 226.0810492

AC040162.1 -2.072634076 2.022900324 -31.63911854 8.42E-104 1.22E-102 226.0610612

RAB20 1.763564066 5.096619522 31.62921621 9.13E-104 1.32E-102 225.9808613

C5orf63 -1.295529204 1.905972482 -31.61510045 1.02E-103 1.48E-102 225.8665153

HS3ST1 2.876954874 2.078764447 31.61469852 1.03E-103 1.49E-102 225.8632591

SEC11C -3.240889932 6.024485496 -31.60702027 1.09E-103 1.58E-102 225.8010498

MTHFS -2.09475673 3.95419695 -31.59825539 1.17E-103 1.70E-102 225.7300276

BPHL -1.648200409 3.144091877 -31.59193208 1.23E-103 1.78E-102 225.6787835

TRIM21 1.663352537 3.974606498 31.58232805 1.33E-103 1.93E-102 225.6009429

GCC1 1.13493386 3.162879367 31.57737244 1.39E-103 2.01E-102 225.5607732

MAF 2.016782861 2.907552384 31.56796434 1.50E-103 2.16E-102 225.4845035

PCNX4 -1.462261493 3.008325766 -31.56435181 1.54E-103 2.23E-102 225.4552145

JARID2 1.153716599 2.538536855 31.56359735 1.55E-103 2.24E-102 225.4490974

NIT2 -1.641896681 3.952935971 -31.55911643 1.61E-103 2.32E-102 225.4127651

GRK4 -1.465569061 2.174664645 -31.5587536 1.61E-103 2.32E-102 225.4098231

PIK3IP1 2.134673268 4.347529065 31.55010741 1.73E-103 2.49E-102 225.3397103

ANAPC4 -1.390185351 3.680676739 -31.53197734 2.01E-103 2.88E-102 225.1926614

CUL1 1.300966043 4.687475641 31.53014963 2.04E-103 2.92E-102 225.1778349

PDK2 -2.531948795 4.290935178 -31.52968813 2.04E-103 2.93E-102 225.1740912

ECM1 3.440572661 3.976252729 31.52041449 2.20E-103 3.16E-102 225.0988562

ZNF780A -1.677943096 2.864199949 -31.51268516 2.34E-103 3.36E-102 225.0361416

ZNF385A 2.119572522 3.191619976 31.50988533 2.40E-103 3.43E-102 225.0134223

LRRC37B -1.337364487 2.30168612 -31.50949983 2.41E-103 3.44E-102 225.0102941

RABGGTB -2.004210434 5.216653963 -31.49634799 2.68E-103 3.83E-102 224.9035596

GLE1 1.247460037 3.699409104 31.49413856 2.73E-103 3.89E-102 224.8856267

SLC43A1 -3.450668786 5.454998734 -31.49394108 2.73E-103 3.90E-102 224.8840238

WNK2 -3.50795929 4.096890791 -31.49331895 2.74E-103 3.91E-102 224.8789741

TKFC -1.906912428 4.439859743 -31.49129588 2.79E-103 3.98E-102 224.8625531

FAM186B -0.692052978 0.588291169 -31.48902144 2.84E-103 4.05E-102 224.8440912

ARMCX6 -1.596897951 4.282226932 -31.48517552 2.93E-103 4.17E-102 224.8128717

TTC9 2.39307042 2.382127443 31.48276603 2.99E-103 4.25E-102 224.7933117

HBS1L -1.631960903 3.536077809 -31.47879001 3.09E-103 4.39E-102 224.7610329

CCDC141 -1.587029626 1.035672746 -31.45776462 3.66E-103 5.20E-102 224.5903088

FBRSL1 -2.274351294 4.746916111 -31.4510517 3.86E-103 5.49E-102 224.5357888

IMPA2 -3.399046178 6.224769376 -31.44820675 3.96E-103 5.61E-102 224.5126815

GIMAP1-GIMAP5 -1.127312385 0.549928863 -31.43453481 4.42E-103 6.26E-102 224.4016205

SNRPB 2.06328097 6.884704895 31.43424837 4.43E-103 6.27E-102 224.3992934

TRERF1 1.288516619 1.964734623 31.43224421 4.50E-103 6.37E-102 224.383011

SYTL1 -2.991420531 5.466923498 -31.42763213 4.67E-103 6.61E-102 224.3455391

CCDC85C -1.693848082 2.827714947 -31.42178834 4.90E-103 6.93E-102 224.2980563

RBIS -2.05708777 5.338363884 -31.40816971 5.47E-103 7.73E-102 224.1873832

FBXO16 -1.652505977 1.715077552 -31.40592367 5.57E-103 7.87E-102 224.1691283

ARHGEF18 -1.694504159 3.182906404 -31.40027426 5.84E-103 8.23E-102 224.1232095

GATB -1.238445753 2.362721953 -31.39171185 6.26E-103 8.82E-102 224.0536059

RAB43 -1.586942465 1.76627574 -31.38390146 6.67E-103 9.39E-102 223.9901074

C11orf1 -2.060275171 4.009717861 -31.38137104 6.80E-103 9.57E-102 223.9695335

NFE2L1 1.82754075 5.97360033 31.37856302 6.96E-103 9.79E-102 223.9467015

BROX 1.39122086 3.99793257 31.3779408 7.00E-103 9.83E-102 223.9416421

MAP4K4 1.681056385 4.330957285 31.37056407 7.43E-103 1.04E-101 223.8816568

TRIM38 1.372702687 2.547711562 31.37055055 7.43E-103 1.04E-101 223.8815469

GUCA1C -3.975907095 2.485026741 -31.36523259 7.76E-103 1.09E-101 223.8382986

FAM222B 1.297015863 3.54437417 31.3542266 8.48E-103 1.19E-101 223.7487811

ATP6V1A 1.407369891 4.270919455 31.34750779 8.96E-103 1.25E-101 223.6941261

FAM193B -2.594287363 5.295761522 -31.34448919 9.18E-103 1.28E-101 223.669569

CTC1 -1.821771705 3.753603117 -31.34366141 9.24E-103 1.29E-101 223.6628346

FOXL1 3.371677434 1.99021485 31.33351471 1.00E-102 1.40E-101 223.5802794

WBP4 1.060836028 3.184748331 31.32998145 1.03E-102 1.44E-101 223.5515291

ARFIP1 1.441109581 3.966703726 31.32354209 1.09E-102 1.52E-101 223.4991278

VSIG2 5.061139817 3.86128289 31.32012753 1.12E-102 1.56E-101 223.4713392

LPAR3 -2.191427827 1.671586047 -31.28912254 1.44E-102 2.01E-101 223.2189454

FAAP20 -2.120487538 4.075791784 -31.28453524 1.49E-102 2.08E-101 223.1815925

PTGER2 2.268664834 1.942238777 31.28427843 1.50E-102 2.08E-101 223.1795013

ABCG1 1.902908854 2.951363233 31.2768885 1.59E-102 2.21E-101 223.1193219

SARDH -2.744091607 2.985390559 -31.27644207 1.60E-102 2.22E-101 223.1156861

C16orf95 -1.285093923 1.500644715 -31.27382783 1.63E-102 2.26E-101 223.0943954

TP53INP2 1.878361212 3.672476097 31.27329456 1.64E-102 2.27E-101 223.0900523

COL8A1 3.67025134 2.842825269 31.27055402 1.67E-102 2.32E-101 223.0677319

ARL6IP5 1.968279512 6.118222294 31.26861586 1.70E-102 2.36E-101 223.051946

SRSF11 -2.293707743 6.170979415 -31.26692735 1.73E-102 2.39E-101 223.038193

NOMO1 -2.3254004 5.246835138 -31.25755756 1.86E-102 2.58E-101 222.961869

RHBDL2 2.56060109 1.973887154 31.25243792 1.94E-102 2.68E-101 222.9201611

TMEM262 -0.917170539 1.096691169 -31.24442246 2.07E-102 2.86E-101 222.8548553

FNTA -1.75832699 4.398707942 -31.2434722 2.09E-102 2.88E-101 222.8471126

FAM83E 3.75939019 3.149435607 31.24095837 2.13E-102 2.94E-101 222.8266293

NRP2 2.429344725 2.683616424 31.23620934 2.21E-102 3.05E-101 222.7879309

MAT1A -3.296019527 2.67360447 -31.23317797 2.27E-102 3.13E-101 222.7632276

RYR2 -2.18208993 1.674836084 -31.23260145 2.28E-102 3.14E-101 222.7585293

CST2 4.145760777 2.437687127 31.23171791 2.30E-102 3.16E-101 222.7513289

CST1 5.850108657 3.138396403 31.22694609 2.39E-102 3.28E-101 222.7124392

ACSF3 -1.49410857 3.358311761 -31.22272904 2.47E-102 3.40E-101 222.6780684

EIF2B4 -1.706355228 4.769115519 -31.21483854 2.64E-102 3.62E-101 222.6137514

STK4 1.294965175 3.105786784 31.21364994 2.66E-102 3.65E-101 222.6040623

SYPL2 -1.585524062 1.633362545 -31.20625848 2.83E-102 3.87E-101 222.5438049

GRN 2.494932715 7.390840276 31.20481083 2.86E-102 3.92E-101 222.5320024

IFNAR1 1.365337805 4.186897561 31.20192203 2.93E-102 4.01E-101 222.5084497

KIFC1 2.360131282 1.942940823 31.19607555 3.07E-102 4.20E-101 222.4607794

ZNF384 -1.800666427 4.131147641 -31.19554964 3.08E-102 4.22E-101 222.4564911

EPO -2.284369311 1.446910929 -31.18230272 3.44E-102 4.69E-101 222.3484634

COL4A1 3.114257703 5.952629725 31.1741956 3.67E-102 5.01E-101 222.2823395

SLC1A2 -2.346119879 1.66865611 -31.17383039 3.68E-102 5.02E-101 222.2793605

ALB -5.929538494 5.433157193 -31.16527217 3.95E-102 5.38E-101 222.2095481

TMEM116 -1.263617464 2.035203511 -31.1620197 4.05E-102 5.52E-101 222.1830141

PABPC1L -2.953009377 4.706711581 -31.16053519 4.10E-102 5.58E-101 222.1709028

ILRUN 1.649084257 5.274509348 31.15525999 4.28E-102 5.83E-101 222.1278635

SCAPER -1.503710782 2.633891077 -31.14009423 4.85E-102 6.59E-101 222.0041093

ERI2 -1.473916275 2.731580814 -31.12780683 5.36E-102 7.28E-101 221.9038218

ACER3 1.270308598 2.506507064 31.12336899 5.55E-102 7.54E-101 221.8675963

SATB1 -1.886481437 3.531638422 -31.12150926 5.64E-102 7.65E-101 221.8524148

ATP6V1F 1.886692312 7.014522876 31.11826197 5.79E-102 7.85E-101 221.8259053

CCDC122 -1.262517469 1.950793563 -31.10846376 6.27E-102 8.50E-101 221.7459088

WDR90 -2.065948136 3.74069971 -31.10699364 6.35E-102 8.59E-101 221.7339051

CDCP1 2.349259528 3.984534484 31.1010442 6.66E-102 9.01E-101 221.6853245

CATSPER2 -1.521200356 1.812911328 -31.10091909 6.67E-102 9.02E-101 221.6843029

EPN1 -2.346797187 5.526591467 -31.09713786 6.88E-102 9.29E-101 221.6534246

COL4A2 2.920995118 6.232217496 31.0852844 7.58E-102 1.02E-100 221.5566153

SOX4 2.334693714 4.236956682 31.08395178 7.66E-102 1.03E-100 221.5457305

CTAGE4 -1.880143931 1.199162486 -31.07894313 7.98E-102 1.08E-100 221.5048178

WDR19 -1.711732746 3.348350564 -31.06303475 9.09E-102 1.22E-100 221.374851

SLC39A5 -5.205764281 5.449767446 -31.05899439 9.39E-102 1.26E-100 221.3418374

MDFI 2.892789462 3.259012882 31.04383079 1.06E-101 1.43E-100 221.217918

TRMT12 1.258808406 2.997863482 31.04318727 1.07E-101 1.44E-100 221.2126584

FOXF2 2.024687634 1.965294236 31.04262343 1.07E-101 1.44E-100 221.2080501

LSM12 -1.436658 3.401081799 -31.03828636 1.11E-101 1.49E-100 221.1726009

PPP1R9B 1.440369231 4.781757616 31.03409774 1.15E-101 1.54E-100 221.1383628

ZSWIM4 1.704536287 2.889115094 31.03282271 1.16E-101 1.56E-100 221.1279402

DLGAP5 2.228110586 1.397672931 31.03098277 1.18E-101 1.58E-100 221.1128994

VMA21 1.143877171 3.520423974 31.03089662 1.18E-101 1.58E-100 221.1121951

RHOG 1.712175521 5.406247617 31.01964988 1.29E-101 1.73E-100 221.0202481

RPL37A -3.108710933 9.280009767 -31.01230133 1.37E-101 1.84E-100 220.960162

WDR61 -1.57837708 4.383686021 -31.01200738 1.38E-101 1.84E-100 220.9577584

MSANTD3-TMEFF1 -0.549310309 0.354167564 -31.01189012 1.38E-101 1.84E-100 220.9567995

ANK3 -1.607645256 2.327218508 -30.99832987 1.54E-101 2.06E-100 220.8459044

ETV6 1.397824323 3.729424736 30.99779931 1.55E-101 2.06E-100 220.841565

RCHY1 -1.290637445 3.26327781 -30.98841719 1.67E-101 2.23E-100 220.7648243

IER5 2.050277174 3.39069828 30.97952327 1.80E-101 2.39E-100 220.6920666

E2F1 2.247465807 2.016131868 30.97762156 1.83E-101 2.43E-100 220.6765082

GPC6 2.492267453 1.917651272 30.9742577 1.88E-101 2.49E-100 220.6489864

UBAP1L -1.121350088 1.40550238 -30.96974829 1.95E-101 2.59E-100 220.61209

GABRD 1.608029709 1.095807035 30.94226893 2.44E-101 3.24E-100 220.3871959

ALG13 -1.580587465 3.797488521 -30.93559787 2.57E-101 3.42E-100 220.3325851

CBL 1.269519845 2.23285912 30.93297961 2.63E-101 3.49E-100 220.3111499

TNFAIP6 3.038660635 2.018080403 30.92509258 2.80E-101 3.72E-100 220.2465749

METTL17 -1.993550552 5.077694917 -30.91630589 3.01E-101 3.99E-100 220.1746249

FBF1 -1.407154681 1.978800117 -30.90888198 3.20E-101 4.24E-100 220.1138265

USP34 -2.052285497 3.865937781 -30.90408853 3.33E-101 4.40E-100 220.0745667

GIMAP2 2.146144 2.602373144 30.90398206 3.33E-101 4.41E-100 220.0736946

GTF3C4 1.161811923 2.635599509 30.88767762 3.81E-101 5.03E-100 219.9401345

IFIT2 2.147616919 2.479245375 30.87114137 4.36E-101 5.76E-100 219.8046416

TMEM250 1.39220401 4.28645058 30.86829059 4.46E-101 5.89E-100 219.7812797

ZNF599 -1.097463708 1.839801374 -30.85271898 5.07E-101 6.68E-100 219.6536539

ZNF852 -1.104465201 1.449605284 -30.84355857 5.47E-101 7.20E-100 219.5785605

PTTG1 2.46932163 2.646389268 30.83866701 5.69E-101 7.49E-100 219.5384571

MMAB -1.959056077 3.998662429 -30.83435931 5.89E-101 7.75E-100 219.503138

RXYLT1 -1.538943379 3.129766694 -30.82698629 6.26E-101 8.23E-100 219.4426808

NUDT15 1.366827205 3.720473428 30.825887 6.32E-101 8.30E-100 219.4336663

GATD3A -3.076173728 2.073738267 -30.81965626 6.65E-101 8.73E-100 219.3825693

NOL12 -1.607082303 3.028660021 -30.81367599 6.98E-101 9.16E-100 219.333522

RRP9 1.516266311 4.306512647 30.81280014 7.03E-101 9.22E-100 219.3263382

ZCCHC8 -1.318657115 3.380948475 -30.80796177 7.32E-101 9.59E-100 219.2866523

BAG4 1.300287188 2.668864799 30.80770468 7.33E-101 9.60E-100 219.2845435

SYT13 3.24417692 3.426393053 30.80385767 7.57E-101 9.90E-100 219.2529869

WDR91 -1.996054179 4.123806762 -30.80134009 7.72E-101 1.01E-99 219.2323344

ABCA10 -2.109303077 1.994135284 -30.79943613 7.84E-101 1.03E-99 219.2167151

EZR 3.029495168 7.400629352 30.79702453 8.00E-101 1.04E-99 219.1969307

PLEKHF2 1.452081149 3.522667093 30.79131713 8.38E-101 1.09E-99 219.1501052

CDH3 3.597610812 2.976305346 30.77843696 9.32E-101 1.22E-99 219.0444167

PAIP2B -3.136677063 3.20260283 -30.77703545 9.43E-101 1.23E-99 219.0329154

CDA 3.781794563 2.94065894 30.76919595 1.01E-100 1.31E-99 218.9685766

NPIPA5 -2.987321522 2.640003717 -30.76578885 1.03E-100 1.35E-99 218.9406122

EXOC7 -1.657149387 4.559542542 -30.76386896 1.05E-100 1.37E-99 218.9248537

PRPF6 1.621965356 5.876394658 30.76038437 1.08E-100 1.40E-99 218.896251

PNLIPRP1 -8.356891991 6.936176756 -30.75955996 1.09E-100 1.41E-99 218.8894837

NUSAP1 2.397520875 2.603279637 30.75951222 1.09E-100 1.41E-99 218.8890918

MMGT1 1.321645374 3.883920243 30.75844147 1.10E-100 1.42E-99 218.8803022

RACGAP1 1.779400727 2.411191803 30.75813057 1.10E-100 1.43E-99 218.8777502

ATF7IP2 -2.273844114 3.382340882 -30.74961397 1.18E-100 1.53E-99 218.807834

HPN -4.462479542 5.276246429 -30.74228104 1.25E-100 1.62E-99 218.7476278

INTS5 1.221352201 3.783893372 30.74140752 1.26E-100 1.63E-99 218.7404555

IFI6 4.706752492 7.075789556 30.74035974 1.27E-100 1.65E-99 218.7318521

EPB41L4B -3.388371768 4.872172646 -30.7340055 1.34E-100 1.73E-99 218.6796744

NDUFA8 1.617358467 5.614359402 30.7312372 1.37E-100 1.77E-99 218.656941

LEF1 2.337228675 2.130757708 30.72775251 1.41E-100 1.82E-99 218.6283232

SPCS1 -2.162779874 6.216000831 -30.72751375 1.41E-100 1.82E-99 218.6263623

LCN10 -2.237629096 1.306252801 -30.72727803 1.42E-100 1.83E-99 218.6244264

CHRFAM7A -1.057482968 0.630098524 -30.72269403 1.47E-100 1.90E-99 218.586778

NPIPA2 -1.655317773 0.804120443 -30.71693558 1.54E-100 1.99E-99 218.5394801

MUC13 4.601250454 4.102590592 30.71160296 1.61E-100 2.07E-99 218.4956761

HDAC6 -1.639731686 4.33701 -30.70842314 1.65E-100 2.13E-99 218.4695543

FSBP -0.770586185 0.376510095 -30.70385769 1.72E-100 2.21E-99 218.4320475

TIMP3 -3.181867434 4.131128599 -30.70293122 1.73E-100 2.22E-99 218.4244358

TPMT 1.549027078 3.733667799 30.69538864 1.84E-100 2.36E-99 218.3624642

NRDE2 -0.99922328 2.120232758 -30.69356736 1.87E-100 2.40E-99 218.3474992

PFN1 2.293425173 8.591804172 30.68952923 1.93E-100 2.48E-99 218.314317

SLC16A10 -2.486393572 2.392162876 -30.68146992 2.06E-100 2.64E-99 218.2480862

GARNL3 -1.3651409 1.581688311 -30.67571421 2.16E-100 2.77E-99 218.2007811

OAS3 2.578876921 3.015621786 30.65598888 2.54E-100 3.25E-99 218.0386311

LETMD1 -1.990573605 5.201837574 -30.65330223 2.60E-100 3.32E-99 218.016542

MTA3 -1.486236571 3.72136105 -30.64831943 2.71E-100 3.46E-99 217.975572

ADAM32 -1.440178371 0.999202697 -30.64738501 2.73E-100 3.49E-99 217.9678886

HMGA1 3.21551923 6.180061585 30.64088917 2.88E-100 3.67E-99 217.9144727

PTGES 3.85970599 3.102176769 30.63630239 2.99E-100 3.81E-99 217.8767519

HOXB5 2.487459076 2.094645858 30.6316693 3.11E-100 3.96E-99 217.8386476

TRIOBP -1.739776317 3.730836101 -30.62846501 3.19E-100 4.06E-99 217.8122927

LRRC8A 2.236114168 4.979700876 30.625643 3.27E-100 4.15E-99 217.789081

KIF20A 2.153902003 1.508933449 30.62345885 3.32E-100 4.23E-99 217.7711152

PLAUR 3.13290299 4.066432847 30.62194075 3.37E-100 4.28E-99 217.7586276

SERPINI2 -6.322514708 4.89897819 -30.61791322 3.48E-100 4.42E-99 217.7254966

CXCR4 3.655311427 4.610530777 30.60357535 3.91E-100 4.97E-99 217.6075349

DNASE2 1.571955289 5.278673633 30.59874103 4.07E-100 5.16E-99 217.5677558

IL12RB2 -1.664818587 1.112694241 -30.59610904 4.16E-100 5.27E-99 217.5460973

DMXL2 -1.791774121 3.234637682 -30.58051455 4.73E-100 5.99E-99 217.4177534

MRPL15 1.623334766 5.182059026 30.57550504 4.93E-100 6.24E-99 217.3765183

CCDC14 -1.794392875 3.725470994 -30.57454964 4.97E-100 6.28E-99 217.3686537

AP1S1 1.703816024 5.399804066 30.55789868 5.70E-100 7.20E-99 217.231569

CYP2S1 4.512574231 2.990308369 30.5563524 5.77E-100 7.29E-99 217.218837

STYK1 2.504715992 1.555292204 30.55213022 5.98E-100 7.54E-99 217.1840702

QTRT1 -2.392383526 5.571283658 -30.53835768 6.69E-100 8.44E-99 217.070647

NCEH1 2.136749033 3.225965023 30.53653747 6.79E-100 8.56E-99 217.055655

CDK1 2.355150451 2.126665922 30.52710268 7.34E-100 9.25E-99 216.9779393

H2AFY2 1.86092507 3.742725026 30.50896689 8.52E-100 1.07E-98 216.8285211

MAGIX -1.930170282 2.854036366 -30.50149213 9.06E-100 1.14E-98 216.7669257

MICU1 1.304841406 4.525080463 30.49553079 9.52E-100 1.20E-98 216.7177967

POLN -0.966370527 0.827850658 -30.4954545 9.53E-100 1.20E-98 216.7171679

DHRS12 -2.347511571 4.474416766 -30.48858064 1.01E-99 1.27E-98 216.6605128

SLC44A4 4.019167026 5.111223549 30.48726648 1.02E-99 1.28E-98 216.6496807

SNX9 1.58729047 4.90327635 30.47909836 1.09E-99 1.37E-98 216.5823493

SULT1A1 -2.131437882 3.064688135 -30.47868199 1.09E-99 1.37E-98 216.5789168

CRKL 1.329931789 4.344985087 30.45840429 1.29E-99 1.62E-98 216.4117265

HIP1 1.638730212 2.927780624 30.45189352 1.36E-99 1.71E-98 216.3580341

PTF1A -4.233067219 2.973783321 -30.4464 1.43E-99 1.78E-98 216.3127266

COA8 -1.865732091 4.323702785 -30.42669132 1.68E-99 2.10E-98 216.1501493

CTSO 1.963842634 4.408654607 30.42207489 1.74E-99 2.18E-98 216.1120612

SHOC2 1.228155954 4.076942022 30.42180597 1.75E-99 2.18E-98 216.1098424

NBPF12 -1.902685318 2.609325119 -30.41667208 1.82E-99 2.27E-98 216.0674817

SMN2 -2.858254176 2.777397547 -30.41201397 1.89E-99 2.36E-98 216.029044

BAZ1B 1.310339263 4.370931201 30.40623026 1.99E-99 2.48E-98 215.9813143

SKI 1.553538684 4.316700442 30.39536806 2.17E-99 2.71E-98 215.8916633

CFAP70 -2.050216444 1.917862472 -30.38826681 2.30E-99 2.87E-98 215.8330454

TGS1 1.170322761 3.016730377 30.38635022 2.34E-99 2.91E-98 215.8172236

ETV3 1.362665683 2.980948428 30.3860956 2.35E-99 2.92E-98 215.8151216

TMEM185B 1.387581814 3.691947342 30.3851128 2.36E-99 2.94E-98 215.8070083

FBXW7 -1.419040821 3.155736857 -30.3809 2.45E-99 3.04E-98 215.7722287

TLCD2 1.686810188 1.89592911 30.38070091 2.45E-99 3.04E-98 215.770585

DST -1.979514067 4.482135415 -30.37346543 2.60E-99 3.23E-98 215.7108457

SYT11 2.356068834 2.668729488 30.36766176 2.73E-99 3.38E-98 215.6629234

BUD13 1.048916387 3.853770703 30.36183498 2.86E-99 3.55E-98 215.614806

BAZ2B -1.417292558 3.045638619 -30.36158417 2.87E-99 3.55E-98 215.6127347

GPR132 2.042816921 1.644895661 30.35728384 2.97E-99 3.68E-98 215.5772198

CD109 2.545199089 1.864179047 30.3447793 3.30E-99 4.08E-98 215.4739363

LXN 2.01174711 4.425201057 30.34367039 3.33E-99 4.11E-98 215.4647761

AHI1 -1.815213285 3.28024103 -30.34218726 3.37E-99 4.16E-98 215.4525244

SOGA1 1.248049743 2.326077157 30.34008652 3.43E-99 4.23E-98 215.4351704

MBOAT2 2.28699059 2.913672663 30.33749561 3.50E-99 4.32E-98 215.4137663

CXorf40A -1.43909676 3.374158423 -30.33732589 3.51E-99 4.32E-98 215.4123642

BPGM 1.419569915 3.58984199 30.3342021 3.60E-99 4.43E-98 215.3865568

BOLA3 -1.803818074 3.776517036 -30.32230777 3.97E-99 4.89E-98 215.2882796

MTERF2 -1.886997316 3.637189232 -30.31996625 4.05E-99 4.98E-98 215.2689306

NR2C1 -1.583029452 3.690625113 -30.3155618 4.20E-99 5.16E-98 215.232533

GON4L -1.439948591 3.708205047 -30.31474263 4.23E-99 5.19E-98 215.2257632

LRRC20 1.509556982 2.611755198 30.30699098 4.50E-99 5.53E-98 215.1616983

SLFN5 1.979383905 2.758124462 30.30366086 4.63E-99 5.68E-98 215.1341736

N4BP3 1.261820679 1.350226592 30.29419114 5.01E-99 6.14E-98 215.0558952

PLXDC2 2.660877869 3.027782108 30.29038548 5.17E-99 6.33E-98 215.0244338

ANKMY1 -1.423053244 2.59129806 -30.28841766 5.25E-99 6.43E-98 215.0081651

NUCB2 -3.162986208 5.579914783 -30.28678754 5.32E-99 6.51E-98 214.994688

PMVK 1.75276961 5.534864321 30.28651675 5.33E-99 6.52E-98 214.9924492

CCNB2 2.413928354 1.998099612 30.2833695 5.48E-99 6.69E-98 214.966428

PPP1R14C 2.709010743 2.093600882 30.27111632 6.06E-99 7.40E-98 214.8651079

PRR15 2.963311172 3.313649702 30.26919588 6.16E-99 7.51E-98 214.8492264

CLHC1 -1.497206332 2.141195771 -30.25346524 7.01E-99 8.55E-98 214.7191205

STXBP2 -2.273490142 5.646090504 -30.25096639 7.16E-99 8.72E-98 214.6984499

CALCOCO1 -2.140275453 5.56688788 -30.23401076 8.23E-99 1.00E-97 214.5581726

MYH3 -1.240280133 1.457209158 -30.23011379 8.50E-99 1.04E-97 214.5259271

CYP27A1 2.20085839 4.110187239 30.21926822 9.30E-99 1.13E-97 214.4361754

TIGAR 1.493423058 2.24669119 30.21895727 9.32E-99 1.13E-97 214.433602

KRR1 -1.344469111 3.317082429 -30.21887933 9.33E-99 1.13E-97 214.4329569

AMHR2 -2.542843618 1.581353214 -30.20392765 1.06E-98 1.28E-97 214.3092008

GANC -1.243248514 2.640699984 -30.20065067 1.08E-98 1.32E-97 214.2820733

CCL5 3.26399567 3.552664538 30.19969627 1.09E-98 1.33E-97 214.2741723

HDAC2 -1.57635405 4.305311993 -30.19198132 1.17E-98 1.41E-97 214.2103001

ZNF638 -1.665002334 4.8495604 -30.19147209 1.17E-98 1.42E-97 214.2060839

TWISTNB 1.199627335 2.993818167 30.18649884 1.22E-98 1.48E-97 214.1649061

ETS2 2.302586209 5.178168057 30.17132995 1.38E-98 1.67E-97 214.0392907

COL1A2 4.785519118 7.753679657 30.16406165 1.47E-98 1.78E-97 213.979091

TPRG1L 1.688473136 5.620503281 30.16032968 1.51E-98 1.83E-97 213.9481783

MARCKS 1.782004889 5.805444493 30.15537294 1.58E-98 1.91E-97 213.907118

RBM39 -2.143072348 6.587069487 -30.14797969 1.68E-98 2.02E-97 213.8458686

GRB14 -2.774768394 3.255785488 -30.14178601 1.77E-98 2.13E-97 213.7945517

SPARC 4.09901802 8.71489254 30.13072377 1.93E-98 2.33E-97 213.7028853

POGLUT2 1.714459325 2.359304153 30.12567138 2.02E-98 2.43E-97 213.661014

ACBD5 1.498241081 3.615545466 30.12267196 2.07E-98 2.49E-97 213.636155

FAM228B -1.481847777 2.322541262 -30.10877042 2.32E-98 2.79E-97 213.5209255

KDM2B -1.137018097 2.847125435 -30.09827322 2.53E-98 3.04E-97 213.4338985

ENTPD1 1.487435113 2.585101242 30.09703534 2.56E-98 3.07E-97 213.4236349

POGLUT3 1.723646232 3.744260924 30.09582836 2.58E-98 3.10E-97 213.4136275

RNF139 1.340849795 4.071973238 30.07813298 2.99E-98 3.59E-97 213.2668876

PAK6 -1.336865921 0.813603129 -30.06470565 3.34E-98 4.01E-97 213.1555149

CDCA5 2.145288878 1.81364785 30.04982857 3.78E-98 4.53E-97 213.0320909

RBM18 1.167519033 3.139700436 30.04762938 3.85E-98 4.61E-97 213.0138436

ACBD6 -1.511005145 4.113377717 -30.03556064 4.26E-98 5.10E-97 212.9136949

CSTF2T 1.164582463 3.705048337 30.03233753 4.37E-98 5.23E-97 212.8869458

CYB561A3 -1.487587488 3.707471453 -30.03111597 4.41E-98 5.28E-97 212.8768076

ATPAF2 -1.365328911 3.122696946 -30.02932145 4.48E-98 5.36E-97 212.8619138

MPLKIP 0.998516609 2.309092333 30.02611651 4.60E-98 5.50E-97 212.8353131

SEM1 -2.152647701 5.115816308 -30.02601745 4.61E-98 5.50E-97 212.8344908

ZNF76 -1.642952311 4.174158387 -30.00932662 5.29E-98 6.31E-97 212.6959376

MARK3 -1.808117409 4.983623924 -30.00360339 5.55E-98 6.61E-97 212.6484201

BAK1 1.631960242 4.23131465 29.99315892 6.05E-98 7.21E-97 212.5616939

CHST12 -1.401621299 2.649098634 -29.9854192 6.45E-98 7.68E-97 212.4974179

KIAA0408 -0.915227718 0.484154817 -29.98120995 6.68E-98 7.95E-97 212.4624583

RNF214 -1.475393388 3.653100743 -29.97799569 6.86E-98 8.16E-97 212.4357609

GZMA 2.787447815 2.321894536 29.96948375 7.36E-98 8.75E-97 212.3650555

SFI1 -1.806235378 3.419944928 -29.96446368 7.67E-98 9.12E-97 212.3233515

ZNF354B -1.31029461 2.908212016 -29.96141931 7.87E-98 9.34E-97 212.298059

NAA60 -1.546214614 4.506330918 -29.9572086 8.15E-98 9.67E-97 212.2630747

AVPI1 1.952736297 3.997522626 29.95051066 8.62E-98 1.02E-96 212.207421

PHF20L1 -1.249011702 2.773908798 -29.94075441 9.34E-98 1.11E-96 212.1263455

LNPK 1.230420742 2.570207396 29.93382644 9.90E-98 1.17E-96 212.0687661

ZNF433 -1.357264031 2.254161476 -29.9294042 1.03E-97 1.22E-96 212.0320091

RAB7A 1.728637892 6.855858813 29.92220323 1.09E-97 1.29E-96 211.9721506

HIST2H4A -2.31258991 1.391725217 -29.9134067 1.17E-97 1.39E-96 211.8990201

HERC1 -1.676124368 3.754955349 -29.90883116 1.22E-97 1.44E-96 211.8609772

DUS1L -2.188856231 5.903182566 -29.90092793 1.30E-97 1.54E-96 211.7952606

WDR12 -1.501991134 3.290919606 -29.89433179 1.37E-97 1.62E-96 211.7404067

NDC1 1.350399581 2.827252518 29.89388321 1.38E-97 1.63E-96 211.7366761

CSGALNACT2 1.764983005 3.618007179 29.87793423 1.57E-97 1.86E-96 211.6040202

RPL14 -2.581448853 8.186722288 -29.87779344 1.58E-97 1.86E-96 211.602849

ACYP2 -1.323810008 2.828944381 -29.85300858 1.94E-97 2.28E-96 211.3966374

ARHGDIB 2.614771329 5.894072748 29.85066873 1.97E-97 2.32E-96 211.3771658

RBM15 0.981246365 2.145061562 29.84923737 2.00E-97 2.35E-96 211.3652541

NCAPH 1.84843887 1.404741438 29.84326354 2.10E-97 2.47E-96 211.3155373

SHROOM3 2.110137227 3.466384624 29.84050575 2.15E-97 2.52E-96 211.2925843

VAMP7 1.400462139 4.20540784 29.83683376 2.22E-97 2.60E-96 211.2620209

TMEM258 -2.16175808 6.670825312 -29.83665939 2.22E-97 2.60E-96 211.2605695

MLYCD -1.263330247 2.189312356 -29.83197764 2.31E-97 2.70E-96 211.221599

PTPMT1 -1.594165789 4.334609306 -29.83033844 2.34E-97 2.74E-96 211.2079539

FGL1 -5.922407331 4.728504654 -29.82723792 2.40E-97 2.81E-96 211.1821432

SAP30BP -1.684130818 4.885605452 -29.82372384 2.47E-97 2.89E-96 211.1528885

RARB 1.56171099 1.829947803 29.81740107 2.60E-97 3.05E-96 211.1002475

VEGFC 2.063996218 2.327539067 29.8166437 2.62E-97 3.06E-96 211.0939417

INTS4 -1.240390547 3.677162196 -29.81437829 2.67E-97 3.12E-96 211.0750793

LSM3 1.224854257 3.612303838 29.80990694 2.77E-97 3.24E-96 211.0378478

EIF3L -2.512502973 7.191007704 -29.80975484 2.77E-97 3.24E-96 211.0365813

NUFIP2 1.264219052 3.948787664 29.80715415 2.84E-97 3.31E-96 211.0149251

GNPDA1 1.300866214 3.988018302 29.80681227 2.84E-97 3.31E-96 211.0120781

TGOLN2 1.691496574 5.844992369 29.79727911 3.08E-97 3.59E-96 210.9326868

ALKAL2 -3.723134628 3.720041326 -29.79356572 3.17E-97 3.70E-96 210.901759

DIPK1A 1.514622866 2.494424734 29.79264183 3.20E-97 3.72E-96 210.8940639

RNF130 -1.888678788 4.710492144 -29.78319978 3.46E-97 4.02E-96 210.8154148

PBXIP1 1.996361916 5.774216292 29.77582281 3.68E-97 4.28E-96 210.7539595

COMP 4.696798346 3.994412503 29.77338153 3.76E-97 4.36E-96 210.7336203

UBXN10 2.059164515 1.774644166 29.76854125 3.91E-97 4.54E-96 210.6932923

LRRC27 -1.159506151 2.128793281 -29.76369693 4.07E-97 4.72E-96 210.6529276

HLA-DRB5 5.188724166 5.260074466 29.76142651 4.15E-97 4.81E-96 210.6340087

F11 -3.26410905 3.047431978 -29.75209045 4.48E-97 5.19E-96 210.5562064

EGLN2 -1.698825395 4.020786129 -29.74469749 4.77E-97 5.52E-96 210.4945894

CES4A -2.334283438 2.595835755 -29.74397214 4.80E-97 5.55E-96 210.4885436

GLO1 1.435826978 5.486754921 29.72387134 5.67E-97 6.56E-96 210.3209763

TRPV6 -3.938221943 4.210110762 -29.71617028 6.05E-97 6.99E-96 210.2567642

DNAJB6 -1.523455036 4.312424115 -29.69891578 6.98E-97 8.07E-96 210.1128684

WDR82 1.429752613 4.85315262 29.69523146 7.20E-97 8.31E-96 210.0821378

FGFR1 -2.770127797 5.597355322 -29.69433644 7.25E-97 8.37E-96 210.0746723

MUL1 1.345014953 4.561807663 29.69334537 7.31E-97 8.43E-96 210.0664054

PATL1 1.438276251 4.07440602 29.69163989 7.42E-97 8.55E-96 210.0521792

HEXA -1.728695113 4.698830713 -29.69155467 7.42E-97 8.55E-96 210.0514684

ADAMTS14 2.372738064 1.559068563 29.67109384 8.80E-97 1.01E-95 209.8807666

SUPT20H -1.361884295 3.783094915 -29.66542927 9.23E-97 1.06E-95 209.8334987

SLC2A1 3.67352802 4.710989358 29.66241005 9.46E-97 1.09E-95 209.8083033

NUTM2B -0.970499396 0.619348976 -29.65866161 9.76E-97 1.12E-95 209.7770209

PAX2 -0.466861469 0.295090754 -29.65339033 1.02E-96 1.17E-95 209.7330269

YBX3 -2.797510137 6.758031512 -29.64831987 1.06E-96 1.22E-95 209.6907057

PIGH -1.554530553 4.053511316 -29.64603217 1.08E-96 1.24E-95 209.6716101

KCNIP3 -1.449889808 1.509331339 -29.64115831 1.13E-96 1.30E-95 209.6309254

WDR44 1.275815366 2.908429924 29.63276748 1.21E-96 1.39E-95 209.5608759

SERINC4 -0.707291687 0.347225388 -29.63127806 1.23E-96 1.40E-95 209.5484407

PRPSAP1 -1.732795362 4.306470303 -29.62287462 1.32E-96 1.51E-95 209.4782757

LSM5 -1.744712951 4.503934487 -29.61996202 1.35E-96 1.54E-95 209.4539547

KLF5 3.328986114 4.740118501 29.61267518 1.43E-96 1.64E-95 209.3931031

PDPK1 -1.435263837 3.387655537 -29.61255558 1.43E-96 1.64E-95 209.3921042

LTBP2 2.719740627 4.155880931 29.6073266 1.50E-96 1.71E-95 209.3484335

PPHLN1 -1.237767156 3.609127458 -29.6009811 1.58E-96 1.80E-95 209.2954333

SLPI 5.017344691 6.965441309 29.59790592 1.62E-96 1.85E-95 209.2697464

OBI1 0.845783963 2.335267156 29.59629799 1.64E-96 1.87E-95 209.2563149

TEN1-CDK3 -1.259546321 1.386374558 -29.58792373 1.76E-96 2.01E-95 209.1863573

RAB35 1.191484416 4.368259358 29.58729018 1.77E-96 2.02E-95 209.1810644

PELI1 1.769985364 3.921280122 29.57508941 1.96E-96 2.23E-95 209.079124

SLC22A3 2.77977385 2.178475631 29.57356239 1.99E-96 2.26E-95 209.0663642

FP565260.1 -1.923626011 1.908842899 -29.57293797 2.00E-96 2.27E-95 209.0611464

KRT17 6.427173453 4.806248067 29.56944186 2.06E-96 2.33E-95 209.0319312

IDH3A -1.649793809 3.450245541 -29.56918871 2.06E-96 2.34E-95 209.0298157

KIAA1328 -0.889985398 1.385277818 -29.559098 2.24E-96 2.54E-95 208.9454842

DNASE1 -3.075637632 3.501891518 -29.54159396 2.59E-96 2.94E-95 208.7991671

SMIM10L1 1.179694162 2.877288319 29.53849198 2.66E-96 3.02E-95 208.7732335

APLNR 3.036478706 2.837525536 29.53474968 2.75E-96 3.11E-95 208.7419451

DUSP6 1.944140005 4.425780688 29.52818383 2.90E-96 3.28E-95 208.6870456

AL133352.1 -1.368504396 0.785425644 -29.52172804 3.06E-96 3.46E-95 208.6330611

TTN -1.082000058 0.746886406 -29.52171318 3.06E-96 3.46E-95 208.6329368

GSR 1.644587737 4.978945463 29.51699687 3.19E-96 3.60E-95 208.593495

CDK5RAP3 -2.553140544 6.692043361 -29.51637957 3.20E-96 3.62E-95 208.5883324

SDR16C5 3.627061659 2.251248143 29.51525768 3.23E-96 3.65E-95 208.5789497

TRIM66 -1.440318112 2.431396362 -29.50696435 3.46E-96 3.91E-95 208.5095851

HDDC2 -1.704675842 4.521991201 -29.49747143 3.75E-96 4.23E-95 208.430177

CHCHD5 -1.940265492 4.44687571 -29.49621979 3.79E-96 4.27E-95 208.4197062

NSMCE4A -1.883673458 4.843998507 -29.49234047 3.91E-96 4.41E-95 208.387252

ERCC5 -1.614637826 4.287014857 -29.48841236 4.04E-96 4.55E-95 208.3543877

PCM1 -1.746241621 4.864973445 -29.48612507 4.12E-96 4.64E-95 208.3352503

HGS -1.944894003 5.1947366 -29.48530029 4.15E-96 4.67E-95 208.3283493

DND1 -0.754128661 0.366103151 -29.48342131 4.22E-96 4.74E-95 208.3126276

SLC16A4 2.594049199 2.345705611 29.48296183 4.23E-96 4.75E-95 208.308783

RCOR1 1.465441925 3.48054179 29.48204882 4.27E-96 4.79E-95 208.3011434

LUC7L -2.249276441 5.061795433 -29.47241896 4.62E-96 5.18E-95 208.2205601

FASTKD5 1.186573298 2.954709063 29.46003282 5.13E-96 5.75E-95 208.1168952

MMP12 3.409737859 1.796986503 29.45842067 5.20E-96 5.82E-95 208.103401

ABI1 1.33204811 4.605175765 29.45128493 5.52E-96 6.18E-95 208.043669

CTSS 3.084237382 4.985574636 29.4445134 5.84E-96 6.53E-95 207.9869798

TMEM173 1.987263993 4.620024473 29.44219021 5.95E-96 6.66E-95 207.9675295

RBM17 -1.728587002 5.449941905 -29.43670779 6.23E-96 6.96E-95 207.9216267

PRDX5 2.257245459 8.446776864 29.43359273 6.40E-96 7.14E-95 207.8955435

INS-IGF2 -2.347878658 1.277922809 -29.42923976 6.63E-96 7.41E-95 207.859093

ZNF184 0.978100613 2.198830961 29.4270974 6.75E-96 7.53E-95 207.8411526

LIN37 -1.372300554 2.625367768 -29.4252872 6.86E-96 7.65E-95 207.8259934

IGFBP4 3.265179286 8.418403126 29.42207698 7.04E-96 7.85E-95 207.7991088

FEN1 1.719367847 3.405199236 29.4121963 7.65E-96 8.52E-95 207.7163534

VCPIP1 1.071269808 2.345736851 29.41213769 7.65E-96 8.52E-95 207.7158625

MATN3 3.621983359 2.463780955 29.40599387 8.06E-96 8.96E-95 207.664399

LIPH 3.044629135 3.666271839 29.40468535 8.15E-96 9.06E-95 207.6534376

TTLL1 1.268322823 2.722122321 29.38962886 9.24E-96 1.03E-94 207.5272952

MFHAS1 1.440153271 2.973246073 29.38882736 9.30E-96 1.03E-94 207.5205795

C5orf51 1.061334034 3.202907545 29.38427997 9.66E-96 1.07E-94 207.4824758

ONECUT1 -2.652547478 2.371449528 -29.37834404 1.02E-95 1.13E-94 207.4327333

ARHGAP31 1.533149612 2.214755996 29.37804104 1.02E-95 1.13E-94 207.4301942

FOXP2 -1.514984795 1.470025898 -29.36370676 1.15E-95 1.27E-94 207.310056

HTATSF1 1.577211505 4.99547978 29.36135494 1.17E-95 1.30E-94 207.2903426

PLA2G7 2.696060412 1.859852757 29.34852373 1.30E-95 1.44E-94 207.1827768

CENPS -1.566218403 2.854439912 -29.34010241 1.40E-95 1.55E-94 207.1121687

IRAK2 2.287874035 2.508698325 29.33782085 1.43E-95 1.58E-94 207.0930375

CDKN2D 1.730752129 3.387451292 29.3284438 1.54E-95 1.70E-94 207.0144032

SNX18 1.372066912 3.05479074 29.32088057 1.64E-95 1.81E-94 206.9509715

HNRNPAB 1.680889928 6.283224384 29.31624957 1.71E-95 1.89E-94 206.9121285

KHDRBS1 1.397918188 5.777996549 29.29980607 1.96E-95 2.16E-94 206.7741854

PLAAT4 3.285447084 4.990482594 29.29130455 2.11E-95 2.32E-94 206.7028538

RPUSD3 -1.484036744 3.812992139 -29.28886756 2.15E-95 2.37E-94 206.6824047

NOXO1 -0.732899505 0.363349954 -29.28843105 2.16E-95 2.38E-94 206.6787419

OSBPL10 1.542542912 2.031251189 29.28584583 2.20E-95 2.43E-94 206.6570481

SUMO1 1.47417753 5.856437727 29.28484975 2.22E-95 2.44E-94 206.6486892

ZWINT 2.033120681 2.964657384 29.2822419 2.27E-95 2.50E-94 206.6268044

EGR2 2.840873533 2.242639058 29.28131226 2.29E-95 2.52E-94 206.6190027

IGFBP5 4.096492261 6.455993738 29.27180506 2.48E-95 2.72E-94 206.5392108

NEMF -1.520500981 3.559668067 -29.2626058 2.68E-95 2.94E-94 206.4619928

CLEC18A -1.14922808 0.633460117 -29.26112288 2.71E-95 2.97E-94 206.4495443

MEA1 1.450461701 5.879511647 29.25578287 2.84E-95 3.11E-94 206.404715

SCD 3.374429445 4.576705465 29.25166622 2.94E-95 3.22E-94 206.3701532

CCDC103 -0.676520763 0.343883456 -29.23875573 3.27E-95 3.58E-94 206.2617486

PRTFDC1 1.494269235 2.65020484 29.23720551 3.31E-95 3.63E-94 206.2487306

DIP2A -1.526087305 3.495849178 -29.23448507 3.39E-95 3.71E-94 206.2258849

AP002990.1 -1.051950613 1.841157083 -29.22747431 3.60E-95 3.93E-94 206.1670058

TRPV1 -1.508520341 0.818443618 -29.21205513 4.09E-95 4.47E-94 206.0374884

BUB1 1.796755495 1.32591793 29.19652827 4.66E-95 5.09E-94 205.9070369

RIPOR3 -1.722155255 1.70246783 -29.18565364 5.11E-95 5.58E-94 205.8156544

IRF2 1.310955149 4.275537317 29.17402111 5.63E-95 6.14E-94 205.7178869

UBE2L6 2.040584677 5.273574777 29.16755515 5.95E-95 6.48E-94 205.6635355

ZNF207 -1.601812991 4.967008135 -29.16614885 6.02E-95 6.56E-94 205.6517137

S100P 6.851979417 4.637790713 29.16052033 6.31E-95 6.87E-94 205.6043965

FNDC1 3.494402694 2.698121447 29.15749192 6.47E-95 7.04E-94 205.5789359

CALR 2.193531997 8.907261127 29.15724683 6.48E-95 7.05E-94 205.5768754

CDC73 1.294105272 3.433999671 29.15605792 6.55E-95 7.12E-94 205.5668796

NAB1 1.443795649 3.95151894 29.15233648 6.76E-95 7.34E-94 205.5355905

IL1RN 3.637529152 2.96263375 29.14907304 6.94E-95 7.54E-94 205.5081506

C1orf54 1.859079021 3.164690492 29.14666037 7.09E-95 7.69E-94 205.4878635

UQCC2 -1.902522623 4.562808501 -29.14410552 7.24E-95 7.85E-94 205.46638

APOL2 1.878274615 4.69405567 29.14358734 7.27E-95 7.88E-94 205.4620226

CENPB 1.543861121 5.667125338 29.1343682 7.86E-95 8.52E-94 205.3844926

FZD2 1.948789639 2.194977867 29.13090845 8.09E-95 8.76E-94 205.3553945

TUBGCP4 -1.027205515 2.371251046 -29.12940461 8.19E-95 8.87E-94 205.3427461

RNF220 -1.548382403 4.622808195 -29.12329489 8.62E-95 9.33E-94 205.2913559

CATSPERG -1.137683127 1.127319488 -29.12181705 8.73E-95 9.44E-94 205.2789248

SLC25A48 -1.745898799 1.022956533 -29.11318553 9.39E-95 1.01E-93 205.2063137

KIF9 -1.609968597 2.632127629 -29.11059517 9.59E-95 1.04E-93 205.184521

HIST2H4B -1.813330605 0.888806281 -29.11004515 9.64E-95 1.04E-93 205.1798935

PEX11B 1.414425958 4.935026137 29.10422671 1.01E-94 1.09E-93 205.1309397

KRBA2 -0.764636586 0.739604775 -29.10420144 1.01E-94 1.09E-93 205.130727

NES 2.200379424 3.518931041 29.09543118 1.09E-94 1.17E-93 205.0569298

CD55 3.118257658 5.329955042 29.08916816 1.15E-94 1.24E-93 205.004224

CCL18 4.322947269 2.544988899 29.08022887 1.24E-94 1.33E-93 204.9289879

CCDC7 -0.81423532 1.022949636 -29.07007176 1.35E-94 1.45E-93 204.8434904

ZNF438 1.045585193 2.416666689 29.05819377 1.49E-94 1.60E-93 204.7434913

RPGR -1.404057033 2.579493424 -29.05699637 1.51E-94 1.62E-93 204.7334096

TMEM203 1.302330356 4.985714129 29.05235573 1.57E-94 1.68E-93 204.6943354

XXYLT1 1.200021651 2.431738893 29.05095967 1.58E-94 1.70E-93 204.68258

TNPO3 1.219821621 4.246463093 29.04721677 1.63E-94 1.75E-93 204.6510623

TSC2 -1.700137089 4.941201059 -29.040081 1.74E-94 1.86E-93 204.5909693

FGGY -1.587595883 2.896311414 -29.03738625 1.78E-94 1.90E-93 204.5682742

OGDH 1.425195223 5.161003217 29.03422171 1.82E-94 1.95E-93 204.5416215

HARS -1.487222894 4.760889571 -29.03010156 1.89E-94 2.02E-93 204.5069183

GBP4 2.24671495 2.67290253 29.02679268 1.94E-94 2.08E-93 204.4790469

PLPP4 3.263147674 1.902422654 29.02205427 2.02E-94 2.16E-93 204.4391319

CTSZ 2.440248417 7.304232613 29.01645462 2.12E-94 2.26E-93 204.3919585

ARL15 1.507920898 3.109180058 29.01266879 2.19E-94 2.34E-93 204.3600632

WBP2NL -0.90086496 0.934016343 -29.00488266 2.33E-94 2.49E-93 204.2944601

AAR2 1.183611224 4.473947482 28.98578562 2.74E-94 2.93E-93 204.1335241

SOCS4 1.004937671 2.590219946 28.98084773 2.86E-94 3.05E-93 204.0919038

RAB7B 2.086679423 2.048388288 28.97999193 2.88E-94 3.07E-93 204.0846902

CORO1C 1.758535077 4.461460353 28.97774371 2.93E-94 3.13E-93 204.0657393

MDN1 -1.411614285 3.158074186 -28.96855305 3.17E-94 3.38E-93 203.9882621

CHRD -2.533160862 3.532583667 -28.9648763 3.27E-94 3.48E-93 203.9572643

CTCF 1.154982575 4.396306062 28.96146293 3.36E-94 3.58E-93 203.9284854

AMT -2.120216635 4.09404632 -28.95941567 3.42E-94 3.64E-93 203.9112239

FAM240C -3.640451531 2.704987325 -28.95809347 3.46E-94 3.68E-93 203.9000754

DNAJC7 -1.486138833 4.786162581 -28.95589827 3.53E-94 3.74E-93 203.8815655

ZBTB6 1.024788927 2.343644494 28.93843319 4.09E-94 4.34E-93 203.7342796

HBEGF 2.604232801 3.330513425 28.9305514 4.37E-94 4.63E-93 203.6677989

USP5 1.461671361 4.888068173 28.91849741 4.83E-94 5.12E-93 203.5661122

AMIGO2 2.703661841 3.355134266 28.91411881 5.01E-94 5.31E-93 203.5291703

KCNT1 -0.488626106 0.33998984 -28.91057678 5.17E-94 5.47E-93 203.4992846

CGAS 1.542879026 1.831632325 28.90727484 5.31E-94 5.62E-93 203.4714234

IQGAP3 2.565486892 1.720896994 28.9069334 5.33E-94 5.64E-93 203.4685423

SRF 1.406793956 4.44885611 28.90219316 5.54E-94 5.86E-93 203.4285423

MED17 -1.221791508 3.246671041 -28.89575253 5.85E-94 6.19E-93 203.3741896

PSAP 2.450900827 9.463676712 28.8862334 6.34E-94 6.70E-93 203.2938478

TRIM32 1.048445076 2.93416268 28.88504267 6.41E-94 6.76E-93 203.2837972

SH3RF3 1.521630685 2.219716446 28.88203988 6.57E-94 6.93E-93 203.2584508

ZNF322 -1.104377796 1.990374681 -28.88073898 6.64E-94 7.01E-93 203.2474696

ARHGAP42 1.598847685 1.626160231 28.88044206 6.66E-94 7.02E-93 203.2449633

ORMDL1 -1.757648954 5.184855799 -28.87513527 6.97E-94 7.34E-93 203.2001653

SLC6A16 -1.828803581 1.806674334 -28.87280675 7.10E-94 7.48E-93 203.1805076

GCOM1 -1.127941609 0.901217671 -28.8650654 7.58E-94 7.98E-93 203.1151496

PROS1 1.979067839 3.505990952 28.85526338 8.24E-94 8.66E-93 203.0323833

ZNF777 1.047244391 3.307727665 28.85378407 8.34E-94 8.77E-93 203.0198913

MSN 2.484520741 5.953332027 28.8504257 8.58E-94 9.01E-93 202.9915307

TBCC 1.351458633 4.244914473 28.84793639 8.76E-94 9.20E-93 202.9705081

PCDH7 2.4874955 1.754625194 28.83693119 9.61E-94 1.01E-92 202.8775586

MSH5-SAPCD1 -1.448890587 1.336500975 -28.8360308 9.69E-94 1.02E-92 202.8699533

MED29 1.431309402 4.644756777 28.81842548 1.12E-93 1.18E-92 202.7212269

EHMT1 -1.267931989 3.675823361 -28.811178 1.19E-93 1.25E-92 202.6599905

TRIM41 -1.308956805 3.530591726 -28.81076385 1.20E-93 1.26E-92 202.656491

TAGLN2 2.642002875 8.885364115 28.80600978 1.25E-93 1.31E-92 202.6163186

PHF23 1.235748933 4.271670479 28.79363886 1.39E-93 1.45E-92 202.51177

JUP 2.242949633 6.716302249 28.78911677 1.44E-93 1.50E-92 202.4735485

CENPC -1.298219135 3.130899606 -28.78668815 1.47E-93 1.54E-92 202.4530204

OTUD1 1.1835584 3.280556943 28.78198366 1.53E-93 1.60E-92 202.4132531

LMF1 -1.539862217 3.369028574 -28.77000423 1.69E-93 1.77E-92 202.3119783

FAIM -1.852889371 3.969612509 -28.76544742 1.76E-93 1.83E-92 202.2734502

TAF7 1.583161192 5.805271008 28.76300677 1.79E-93 1.87E-92 202.2528133

LAPTM5 3.269781715 5.547900534 28.75861048 1.86E-93 1.94E-92 202.2156387

GIPC3 1.112210773 1.313800322 28.75834588 1.87E-93 1.94E-92 202.2134012

OASL 3.14923419 2.147680707 28.75472123 1.92E-93 2.00E-92 202.1827496

RAB10 1.60386514 5.581622829 28.74878061 2.02E-93 2.11E-92 202.1325097

IFI35 1.879768026 4.78189805 28.73447772 2.28E-93 2.37E-92 202.0115324

CYP2R1 -1.478318333 3.346047939 -28.73234023 2.33E-93 2.42E-92 201.9934508

GMPPB -1.946005785 4.3824567 -28.72840607 2.40E-93 2.50E-92 201.9601693

TSTD2 -1.539398478 2.842285983 -28.72714534 2.43E-93 2.52E-92 201.9495037

CD93 2.310357648 3.885434346 28.72357 2.50E-93 2.60E-92 201.9192555

ZNF665 -1.174448118 1.23356259 -28.71097873 2.79E-93 2.89E-92 201.8127182

DNAJC17 -1.416961774 3.538139333 -28.71013603 2.81E-93 2.91E-92 201.8055873

SFRP4 4.388913554 4.249920741 28.70446309 2.94E-93 3.05E-92 201.7575804

GCNT3 4.165785493 3.251116321 28.70089148 3.03E-93 3.14E-92 201.727354

SLC17A5 1.526911757 3.746152112 28.70065656 3.04E-93 3.15E-92 201.7253657

SLC37A3 -1.378264813 3.876437067 -28.69329667 3.23E-93 3.35E-92 201.663074

AC010422.6 -0.638535589 0.696815093 -28.6929281 3.24E-93 3.35E-92 201.6599544

GNE 1.822243617 3.446053596 28.69282101 3.25E-93 3.36E-92 201.659048

C3orf38 1.050346336 2.905024968 28.68968332 3.33E-93 3.44E-92 201.6324894

PRAF2 1.843543203 4.111008311 28.68818092 3.38E-93 3.49E-92 201.6197721

KLF15 -2.837898391 3.712139259 -28.68718432 3.41E-93 3.51E-92 201.611336

MYOM2 -1.614906456 1.614558912 -28.67898671 3.65E-93 3.76E-92 201.5419403

MSRB2 1.55813867 4.062044136 28.6749397 3.78E-93 3.89E-92 201.5076778

TRAM2 1.668311482 3.472179744 28.66913948 3.97E-93 4.09E-92 201.4585691

CTDSPL 1.508894543 4.216136097 28.66764994 4.02E-93 4.14E-92 201.4459569

KIRREL2 -3.667520544 3.714282873 -28.65893301 4.33E-93 4.45E-92 201.372144

AKIRIN1 1.444845103 4.653981703 28.6560673 4.43E-93 4.56E-92 201.3478758

NUPR1 -3.378209245 6.317462751 -28.65121127 4.62E-93 4.75E-92 201.3067504

AGGF1 1.037743451 3.294840628 28.64627 4.81E-93 4.95E-92 201.2649

LY96 2.583056054 3.244980567 28.64296336 4.95E-93 5.08E-92 201.2368927

NDUFB1 -2.005467391 6.166468367 -28.6366151 5.22E-93 5.36E-92 201.1831189

GEM 3.253588504 3.84652043 28.63659676 5.22E-93 5.36E-92 201.1829635

RNF145 1.585911161 4.69141077 28.6346948 5.31E-93 5.44E-92 201.1668518

BBS12 1.156877054 1.779185084 28.62404352 5.81E-93 5.95E-92 201.0766153

GALT -1.765848729 4.176071522 -28.62391422 5.82E-93 5.96E-92 201.0755198

KCNK1 2.606935364 3.694295194 28.62163935 5.93E-93 6.07E-92 201.0562455

SNX27 1.074200481 3.466355617 28.62032414 6.00E-93 6.14E-92 201.0451018

NFKBIE 1.909427442 3.736634963 28.61140648 6.47E-93 6.61E-92 200.9695378

ZNF598 -1.486702335 4.968106735 -28.60906706 6.60E-93 6.74E-92 200.949713

LAPTM4B 1.931368058 5.567846474 28.60847812 6.63E-93 6.77E-92 200.9447221

SOCS5 1.273969068 3.088932814 28.59889017 7.19E-93 7.34E-92 200.8634641

STRADB -1.367734928 3.528821595 -28.59209661 7.62E-93 7.77E-92 200.8058818

PAQR7 1.33295746 3.225323328 28.583802 8.17E-93 8.33E-92 200.7355691

UNG 1.203437267 4.123747063 28.58144122 8.33E-93 8.50E-92 200.7155554

TPGS1 -2.232777495 2.641680817 -28.5783625 8.55E-93 8.72E-92 200.6894543

ZNF10 -1.476872375 2.716634439 -28.56342385 9.71E-93 9.89E-92 200.5627896

RNGTT 1.153689839 2.296059856 28.56141533 9.87E-93 1.01E-91 200.5457574

ZNF281 1.357252996 2.744424851 28.54633583 1.12E-92 1.14E-91 200.4178673

ELOVL5 1.888472177 4.408814595 28.54382227 1.15E-92 1.17E-91 200.396547

DALRD3 -1.641697126 4.594944276 -28.54356331 1.15E-92 1.17E-91 200.3943504

C5 -2.399030719 2.685620574 -28.54169077 1.17E-92 1.19E-91 200.3784667

ATP9A 1.803306833 4.332497138 28.54143781 1.17E-92 1.19E-91 200.376321

HNRNPH2 1.453073042 5.709199174 28.52516936 1.34E-92 1.36E-91 200.2383068

SPIN2B -1.217096233 2.412799045 -28.52436708 1.35E-92 1.37E-91 200.2314998

NEK2 2.23157402 1.453564546 28.51871059 1.42E-92 1.44E-91 200.1835046

TNFAIP8L1 1.265511679 2.384139541 28.51743977 1.43E-92 1.45E-91 200.1727212

SMIM19 -1.772296683 4.693806904 -28.50546253 1.59E-92 1.61E-91 200.07108

ARF3 1.48865664 5.619650397 28.50520436 1.59E-92 1.61E-91 200.0688889

SEMA6D -2.192229701 2.298289756 -28.50273224 1.62E-92 1.64E-91 200.0479078

CHST14 1.28332796 3.571552484 28.49759666 1.70E-92 1.71E-91 200.0043192

EIF2D -1.456015925 4.461970908 -28.49029518 1.80E-92 1.82E-91 199.9423419

NYNRIN 1.691664804 2.959518655 28.48948153 1.82E-92 1.84E-91 199.935435

SMC2 1.285348847 2.162416734 28.48153641 1.94E-92 1.96E-91 199.8679862

VMO1 2.114709519 2.192293614 28.4806482 1.96E-92 1.98E-91 199.8604454

TRMT44 -1.063859479 2.492320485 -28.47770237 2.01E-92 2.02E-91 199.835435

MYD88 1.613651844 4.586711759 28.47616363 2.03E-92 2.05E-91 199.8223706

SUFU 0.945938135 2.629593257 28.47531999 2.05E-92 2.06E-91 199.8152076

CLCNKA -1.910757648 1.386897727 -28.47409709 2.07E-92 2.08E-91 199.8048244

ZNF888 1.800042563 1.52052359 28.47324093 2.09E-92 2.10E-91 199.7975549

RUNX1 2.255288902 3.215042132 28.47070468 2.13E-92 2.14E-91 199.7760197

BHLHE41 2.254698895 3.804245494 28.46479045 2.24E-92 2.25E-91 199.7257991

PPARD 1.562929753 4.119836641 28.46395916 2.26E-92 2.27E-91 199.7187399

ECT2 2.122629026 2.630680957 28.46303469 2.27E-92 2.28E-91 199.7108892

ZNF677 -1.366264354 1.848133333 -28.45898872 2.35E-92 2.36E-91 199.6765297

POM121 -1.412652585 3.587267069 -28.45117077 2.51E-92 2.52E-91 199.6101316

TPGS2 -1.551658244 4.019564274 -28.44676493 2.61E-92 2.62E-91 199.5727095

CD74 3.572936192 9.387725637 28.44020814 2.76E-92 2.77E-91 199.5170134

DCT -0.653199138 0.388076103 -28.43581028 2.86E-92 2.87E-91 199.4796532

TYRO3 -1.501538767 2.737613789 -28.43430135 2.90E-92 2.91E-91 199.4668341

TMEM150A -1.559351634 4.495076532 -28.43183645 2.96E-92 2.97E-91 199.4458931

HAGH -1.880966054 4.663926283 -28.42898887 3.04E-92 3.04E-91 199.4217001

P2RX4 -1.703241096 4.369893063 -28.42603897 3.11E-92 3.11E-91 199.3966367

NANP 0.913236174 1.814576563 28.42284549 3.20E-92 3.20E-91 199.3695025

AL031708.1 -0.316208036 0.216106498 -28.41113635 3.53E-92 3.53E-91 199.2700027

GPATCH11 0.974823216 2.177222309 28.40915038 3.59E-92 3.59E-91 199.253125

PGM3 -1.832987609 4.435059065 -28.40713492 3.65E-92 3.65E-91 199.2359962

NFS1 -1.320289791 3.698818532 -28.40704058 3.66E-92 3.65E-91 199.2351944

HTT -1.581565935 4.165802303 -28.39962588 3.89E-92 3.88E-91 199.1721748

MOCS3 0.993190578 1.868290285 28.39832934 3.94E-92 3.92E-91 199.1611545

VCAN 3.584379078 4.09945583 28.38972443 4.24E-92 4.22E-91 199.0880093

ABHD17C 2.548166589 3.945942094 28.38119461 4.55E-92 4.53E-91 199.0154935

LIF 3.387202415 3.271937048 28.37462682 4.82E-92 4.79E-91 198.9596519

AFMID -2.038483931 4.691550514 -28.36587614 5.19E-92 5.16E-91 198.8852425

SERTAD4 1.626354431 1.188138751 28.36568499 5.20E-92 5.16E-91 198.883617

DSG2 2.31420379 4.962571455 28.35926609 5.49E-92 5.45E-91 198.8290293

PARP3 1.613810205 3.64895872 28.35348838 5.76E-92 5.72E-91 198.7798902

PDGFB 1.845712275 3.596694596 28.34761235 6.06E-92 6.01E-91 198.7299107

PTPRA 1.298016046 4.802906119 28.34686887 6.10E-92 6.05E-91 198.7235867

SLC25A3 -2.058676575 7.097784903 -28.34376683 6.26E-92 6.20E-91 198.6971998

NUTM2A -1.038232663 0.673532648 -28.3382502 6.56E-92 6.50E-91 198.6502709

HPS6 1.394065619 3.632983807 28.33783883 6.58E-92 6.52E-91 198.6467714

CST7 2.680589429 2.557828594 28.33625725 6.67E-92 6.60E-91 198.6333164

MGAT5 1.462503638 3.854633411 28.33148895 6.95E-92 6.87E-91 198.5927493

FOXF1 1.984666025 2.105065353 28.32972501 7.05E-92 6.97E-91 198.5777417

GMIP 1.550033755 3.516574827 28.32526322 7.33E-92 7.24E-91 198.5397788

MRPL42 -1.323049383 3.196672803 -28.32229849 7.51E-92 7.42E-91 198.5145522

S100A10 3.207570646 8.29113899 28.32104203 7.59E-92 7.50E-91 198.5038608

LRTOMT -1.219981252 2.569410411 -28.31914186 7.72E-92 7.61E-91 198.4876918

SRPX2 2.721666289 3.283757551 28.31225134 8.18E-92 8.07E-91 198.4290544

SLC44A1 1.695920893 4.113481494 28.30863086 8.44E-92 8.32E-91 198.3982423

GPD2 1.305625807 3.342905865 28.30734301 8.53E-92 8.40E-91 198.3872817

SAPCD2 2.150729489 1.585739464 28.29754304 9.27E-92 9.13E-91 198.3038698

ZBTB33 1.119304633 3.020407507 28.29710306 9.31E-92 9.16E-91 198.3001246

HMOX1 2.486550487 3.645851085 28.29564374 9.42E-92 9.27E-91 198.2877026

MOB3A 1.367586799 4.211444557 28.28792069 1.01E-91 9.89E-91 198.2219583

BRCC3 1.16065378 3.51777886 28.28600134 1.02E-91 1.01E-90 198.2056182

CD58 1.996837977 3.713703229 28.28359418 1.04E-91 1.03E-90 198.1851247

KBTBD2 1.117736971 4.013526632 28.27448818 1.13E-91 1.11E-90 198.1075938

PEX1 -1.444430651 3.436176205 -28.27429676 1.13E-91 1.11E-90 198.1059638

SLC51A -0.995118193 1.508972595 -28.26340063 1.24E-91 1.22E-90 198.0131778

GLI2 1.606843889 1.368270339 28.26035414 1.27E-91 1.25E-90 197.9872329

FRZB 2.654476111 2.99296933 28.25803746 1.30E-91 1.27E-90 197.9675025

FADS3 -1.838502739 3.832953331 -28.25184621 1.37E-91 1.34E-90 197.9147705

MEX3D 1.511985902 3.355937945 28.25167124 1.37E-91 1.34E-90 197.9132802

SENP3 -1.502768041 4.232642601 -28.24172556 1.49E-91 1.46E-90 197.8285612

TRAFD1 1.17657871 4.087074513 28.24090589 1.50E-91 1.47E-90 197.8215787

TRO -2.236855557 3.393408692 -28.23792267 1.54E-91 1.50E-90 197.7961645

WDR74 -1.652076852 4.509292107 -28.23392254 1.59E-91 1.56E-90 197.7620855

RNASEL 1.213440549 2.489537017 28.23012158 1.65E-91 1.61E-90 197.7297016

C7orf25 -0.872746214 0.890192144 -28.22974465 1.65E-91 1.61E-90 197.7264901

VRK3 -1.405080659 3.767518087 -28.22434701 1.73E-91 1.69E-90 197.6804993

TMEM158 2.67040582 2.713911285 28.21834129 1.82E-91 1.77E-90 197.6293232

MIB2 -2.209954336 4.24050532 -28.21801219 1.82E-91 1.78E-90 197.6265188

OTUD7A -0.763868191 0.774234572 -28.20779376 1.99E-91 1.94E-90 197.5394349

TGFB1 2.144578598 4.771965334 28.20188987 2.09E-91 2.04E-90 197.4891149

MAP3K2 1.06472595 3.330955934 28.18828965 2.35E-91 2.29E-90 197.3731815

EPC1 -1.153294072 3.314755201 -28.18629354 2.39E-91 2.32E-90 197.3561641

PIP4K2A 1.561440655 3.510746855 28.16928782 2.76E-91 2.68E-90 197.2111656

PMAIP1 2.154263809 2.027215834 28.16158937 2.95E-91 2.87E-90 197.1455137

ZC3H8 -1.245293438 2.597192984 -28.16058717 2.97E-91 2.89E-90 197.1369664

FXYD5 2.683103466 5.754117721 28.16037042 2.98E-91 2.89E-90 197.1351178

NMRK1 -1.739265641 4.55464431 -28.15426929 3.14E-91 3.05E-90 197.0830818

APMAP 1.561110791 5.845385966 28.15284086 3.18E-91 3.08E-90 197.0708982

TRIM15 3.197743887 1.89427242 28.14641566 3.36E-91 3.25E-90 197.0160923

MAP6D1 -1.476989341 1.943053691 -28.14600739 3.37E-91 3.26E-90 197.0126097

TNFRSF10A 2.132602238 2.698868209 28.14582511 3.37E-91 3.27E-90 197.0110548

SYCP2L -1.467387023 0.982585223 -28.14405887 3.42E-91 3.31E-90 196.9959881

MYH7 -1.687603104 1.136120364 -28.11356703 4.44E-91 4.30E-90 196.7358214

NPHP1 -1.077149527 1.694749294 -28.0971998 5.11E-91 4.94E-90 196.5961247

CCN4 2.86741541 2.200773174 28.0957008 5.17E-91 5.00E-90 196.5833289

ADAM9 2.422922335 4.876811894 28.09262995 5.31E-91 5.13E-90 196.5571146

GSTA2 -4.915964174 4.325940884 -28.08952443 5.45E-91 5.26E-90 196.5306032

ZNF578 -0.66464738 0.439284063 -28.08947112 5.45E-91 5.26E-90 196.5301482

VAPA -1.791920215 5.627045336 -28.08789792 5.53E-91 5.33E-90 196.5167175

FGD1 1.138073734 2.4360024 28.08017096 5.90E-91 5.69E-90 196.450747

CPXM1 3.362575329 3.37566229 28.07361091 6.24E-91 6.02E-90 196.3947336

TRIM56 1.175240014 3.68048778 28.07338768 6.26E-91 6.02E-90 196.3928274

LAMA4 2.165525464 3.242202284 28.06289515 6.84E-91 6.58E-90 196.3032254

PLCB3 1.89200441 4.017237748 28.05584466 7.27E-91 6.99E-90 196.2430095

PCED1B 1.961230789 2.675175471 28.05363195 7.40E-91 7.12E-90 196.2241102

SRGN 3.233975217 5.773170744 28.05354132 7.41E-91 7.12E-90 196.2233361

UBTD1 1.57894811 3.856974152 28.05054406 7.60E-91 7.30E-90 196.197735

ZFP1 0.985279229 2.00599105 28.05054095 7.60E-91 7.30E-90 196.1977084

HEXD -2.431342616 4.943522685 -28.04903706 7.70E-91 7.39E-90 196.1848625

STXBP6 -2.348043596 3.642179138 -28.04770273 7.79E-91 7.47E-90 196.1734646

BAIAP2L2 3.319956136 3.345879494 28.02619215 9.36E-91 8.97E-90 195.9896923

TEAD3 1.825620189 4.210235697 28.02322997 9.60E-91 9.20E-90 195.964381

ZCCHC17 1.128362976 4.382802423 28.01756367 1.01E-90 9.65E-90 195.9159606

LNPEP 1.038332432 2.909261759 28.01420196 1.04E-90 9.92E-90 195.8872319

C11orf68 1.301180005 5.069706248 28.01376731 1.04E-90 9.96E-90 195.8835174

NME2 -3.140727338 6.814749025 -28.0024683 1.15E-90 1.10E-89 195.786947

PHF7 -1.403882027 2.304022978 -27.99916763 1.18E-90 1.13E-89 195.7587339

PPP1R8 1.146766358 4.279204934 27.99863876 1.18E-90 1.13E-89 195.7542133

MFN2 1.271512028 4.772551125 27.99729344 1.20E-90 1.14E-89 195.7427134

SERF1A -1.354133303 0.732538682 -27.98803021 1.30E-90 1.24E-89 195.6635254

GTF2A1 1.191877581 3.160041513 27.98614842 1.32E-90 1.26E-89 195.6474373

VPS51 -2.044076474 6.15092453 -27.98344572 1.35E-90 1.29E-89 195.6243304

DTX3L 1.595606276 4.302727284 27.97353054 1.47E-90 1.40E-89 195.5395523

ZNF276 -1.628755615 3.695047586 -27.96734368 1.55E-90 1.47E-89 195.4866466

AHNAK 2.238828623 5.744659373 27.96316324 1.60E-90 1.53E-89 195.4508957

RUFY3 -1.772536461 3.898086718 -27.95429511 1.73E-90 1.65E-89 195.3750493

TREM2 3.117226501 3.386589789 27.94815228 1.82E-90 1.73E-89 195.322506

SLC35G1 -1.704750082 2.044675401 -27.94703509 1.84E-90 1.75E-89 195.3129496

NEURL4 -1.590407018 3.755562566 -27.94679486 1.84E-90 1.75E-89 195.3108946

SYNDIG1 2.343332043 1.659408975 27.94065181 1.94E-90 1.85E-89 195.258344

UNKL -1.502409179 2.74710846 -27.9362636 2.02E-90 1.92E-89 195.2208023

OAS2 3.074387108 2.863789579 27.93556564 2.03E-90 1.93E-89 195.2148309

BCL11B 1.452061814 0.999620402 27.92310545 2.26E-90 2.14E-89 195.1082189

EPB41L5 -1.37405491 3.210128658 -27.91980288 2.32E-90 2.20E-89 195.0799583

S1PR2 1.429430638 2.498577304 27.91165844 2.49E-90 2.36E-89 195.0102596

USP3 -1.449229345 4.180514468 -27.90949376 2.54E-90 2.40E-89 194.9917333

KANSL1 -1.383863649 3.921696933 -27.90811714 2.57E-90 2.43E-89 194.9799513

PCDH1 2.176057832 4.731969302 27.90694393 2.59E-90 2.45E-89 194.96991

PSMB8 2.045778529 5.641357003 27.90680247 2.60E-90 2.45E-89 194.9686992

DTL 1.618410016 1.279481502 27.90050705 2.74E-90 2.59E-89 194.914815

SPDEF 3.803957932 2.565725628 27.90000003 2.75E-90 2.60E-89 194.9104751

MR1 1.374200463 2.631284815 27.89855239 2.78E-90 2.63E-89 194.8980836

CRABP2 4.429118263 4.011747498 27.89380948 2.90E-90 2.74E-89 194.8574835

MRPL23 -2.084200153 5.366814757 -27.88448557 3.14E-90 2.96E-89 194.7776615

DDX55 -1.408720859 3.568662591 -27.88358783 3.17E-90 2.98E-89 194.7699754

FMOD 2.557023442 4.877508847 27.88245426 3.20E-90 3.01E-89 194.7602701

AFDN -1.852635932 5.080551783 -27.87267992 3.47E-90 3.27E-89 194.6765785

ZDHHC11B -2.341645626 2.391333814 -27.87249523 3.48E-90 3.28E-89 194.6749969

ZKSCAN4 0.833450629 2.019225211 27.87034015 3.54E-90 3.34E-89 194.6565427

HAAO -2.072317205 3.740928667 -27.86985859 3.56E-90 3.35E-89 194.6524191

PPT1 1.734756296 5.526949261 27.8696564 3.57E-90 3.35E-89 194.6506876

AAK1 -1.299534846 2.859800741 -27.86661935 3.66E-90 3.44E-89 194.6246798

PHYHD1 -2.795868191 4.592464202 -27.86035321 3.86E-90 3.63E-89 194.5710162

ABCD4 -1.572887674 4.311909336 -27.85582599 4.01E-90 3.77E-89 194.5322419

ZNF655 -1.661919058 4.664862727 -27.85520887 4.03E-90 3.79E-89 194.5269563

MRPL57 1.417813977 4.472988555 27.84737854 4.31E-90 4.05E-89 194.4598855

AURKA 1.94696758 2.072032667 27.84132597 4.54E-90 4.26E-89 194.4080372

CNPY4 1.51439444 3.232792381 27.84063264 4.57E-90 4.28E-89 194.4020976

HNRNPA1P48 -1.620550587 2.9549885 -27.83552066 4.78E-90 4.47E-89 194.3583028

XPNPEP1 -1.872097985 5.516802281 -27.82350099 5.29E-90 4.96E-89 194.255317

ANLN 2.478377386 1.878475264 27.8211646 5.40E-90 5.05E-89 194.2352965

PCNA 1.720966543 5.506339593 27.81913888 5.49E-90 5.14E-89 194.2179377

IFIT3 2.507329955 3.617429448 27.81705995 5.59E-90 5.23E-89 194.2001224

PHLDB1 -2.115095837 4.577398289 -27.80539269 6.18E-90 5.78E-89 194.1001307

AL136295.4 -1.102006956 0.569637857 -27.80356109 6.28E-90 5.86E-89 194.0844319

SLC26A10 -1.067741235 0.850118087 -27.79671254 6.66E-90 6.22E-89 194.0257289

PARP4 1.772538856 4.571461375 27.79493093 6.76E-90 6.31E-89 194.0104567

RHOA 2.066903047 8.200479175 27.79466483 6.78E-90 6.32E-89 194.0081756

SMIM13 0.957265513 2.317335313 27.78653511 7.26E-90 6.77E-89 193.9384817

NOP10 1.796294161 6.972124698 27.78590534 7.30E-90 6.80E-89 193.9330825

HYKK -1.336368909 1.815165868 -27.7796877 7.70E-90 7.17E-89 193.8797746

BCAS2 1.326609602 4.852113722 27.77675372 7.90E-90 7.35E-89 193.854618

CUL3 -1.358398143 4.250861852 -27.77214718 8.22E-90 7.65E-89 193.8151186

SPTY2D1 1.186310449 3.149832586 27.77202445 8.22E-90 7.65E-89 193.8140662

RWDD1 -1.742931875 4.81240556 -27.76830795 8.49E-90 7.89E-89 193.7821966

PRRX2 2.746137987 2.243468179 27.75894981 9.20E-90 8.55E-89 193.7019417

ASNSD1 1.178346823 4.427249379 27.75205056 9.76E-90 9.06E-89 193.6427675

SPRYD4 -1.264866002 2.699097449 -27.75191708 9.77E-90 9.07E-89 193.6416227

AEBP1 3.687306329 6.628916864 27.73794246 1.10E-89 1.02E-88 193.5217461

GAL3ST4 1.90751223 2.132424733 27.73466934 1.13E-89 1.05E-88 193.4936655

LZTS1 1.997940048 2.19682857 27.73325706 1.15E-89 1.06E-88 193.4815488

NDRG3 1.277237054 4.205001864 27.72677584 1.21E-89 1.12E-88 193.4259405

FAM160B2 -1.82967264 5.063406954 -27.71852322 1.30E-89 1.20E-88 193.3551264

PRCD -1.24822971 1.162526754 -27.71815979 1.30E-89 1.21E-88 193.3520077

ZNF789 -1.455043031 2.837918521 -27.71683148 1.32E-89 1.22E-88 193.3406089

RIT1 1.18743249 3.537791617 27.71144526 1.38E-89 1.28E-88 193.2943854

RSRC2 -1.501040356 4.914388874 -27.70817589 1.42E-89 1.31E-88 193.2663266

TSN 1.148657021 4.679941549 27.70773113 1.43E-89 1.32E-88 193.2625094

TTC1 1.22447095 4.986597288 27.70157657 1.50E-89 1.39E-88 193.2096852

PPOX -1.519169386 3.585668644 -27.69580629 1.58E-89 1.46E-88 193.1601552

STRN 1.175072948 2.70508592 27.69201199 1.63E-89 1.51E-88 193.1275841

GPX8 2.133335886 3.616151893 27.68484094 1.74E-89 1.60E-88 193.0660216

DGKH 1.139347034 1.561053517 27.68296518 1.76E-89 1.63E-88 193.0499175

PLAT 3.656742405 4.84022188 27.67091833 1.96E-89 1.80E-88 192.9464806

HOXC4 1.563448125 1.244964874 27.66351278 2.09E-89 1.92E-88 192.8828864

NPAS2 -2.164857357 4.271030977 -27.66199848 2.11E-89 1.94E-88 192.8698818

CASTOR1 -1.391015385 2.066763461 -27.65533853 2.24E-89 2.06E-88 192.8126835

SPDYE5 -0.521299261 0.528849229 -27.65222233 2.30E-89 2.11E-88 192.7859186

TCEAL9 1.696422568 5.968104615 27.64997655 2.34E-89 2.15E-88 192.7666289

SLC22A18AS 1.977985479 1.336514585 27.6434221 2.48E-89 2.28E-88 192.7103276

MYL12B 2.294129455 8.464483801 27.6394165 2.56E-89 2.35E-88 192.6759177

VSTM4 1.711353374 1.908411446 27.63774501 2.60E-89 2.39E-88 192.6615583

SRRM2 -2.373711954 7.64852282 -27.63735776 2.61E-89 2.39E-88 192.6582315

COL17A1 4.979607694 2.981119949 27.63458827 2.67E-89 2.45E-88 192.6344387

GOLM1 2.521980402 6.170524218 27.63331227 2.70E-89 2.48E-88 192.6234761

PKIA 1.812295812 1.521301764 27.63326318 2.70E-89 2.48E-88 192.6230544

PINX1 1.315557515 1.112213198 27.62981453 2.78E-89 2.55E-88 192.5934249

CCDC196 -1.792356901 0.977900752 -27.62845397 2.82E-89 2.58E-88 192.5817352

ADARB2 -1.777768845 1.279340639 -27.62747455 2.84E-89 2.60E-88 192.57332

ITGB5 2.107326408 5.640613415 27.62370219 2.93E-89 2.68E-88 192.5409068

PGA3 -3.603935576 1.767530374 -27.62344933 2.94E-89 2.69E-88 192.538734

HAPLN3 2.46689803 2.754832187 27.61548673 3.15E-89 2.88E-88 192.4703112

ACCS -1.970385801 3.720701879 -27.61342941 3.20E-89 2.93E-88 192.4526314

KCTD20 1.392820441 4.018703279 27.60488094 3.45E-89 3.15E-88 192.3791638

CCNL1 -2.071753809 5.450838713 -27.60438194 3.46E-89 3.16E-88 192.3748751

FAM76B -1.352323328 3.299045336 -27.60231098 3.53E-89 3.22E-88 192.3570753

ALDH1B1 2.061567512 3.97742474 27.60068081 3.58E-89 3.26E-88 192.3430637

USP38 1.055704196 2.72569761 27.59941889 3.61E-89 3.29E-88 192.3322172

CAMK2N2 -2.929522892 3.013419147 -27.59094175 3.89E-89 3.54E-88 192.2593486

F2RL2 2.657581746 2.12206749 27.58227373 4.19E-89 3.81E-88 192.1848305

MPRIP -1.784223823 5.552087334 -27.58074846 4.24E-89 3.86E-88 192.1717169

GGNBP2 -1.350078722 4.64143465 -27.57277105 4.54E-89 4.13E-88 192.1031266

TFDP1 1.221935752 4.731363626 27.56717748 4.77E-89 4.33E-88 192.0550282

BTBD9 0.87754178 2.179804684 27.5654184 4.84E-89 4.40E-88 192.0399014

ADAMTS12 2.639335393 2.020662701 27.55972568 5.08E-89 4.62E-88 191.9909454

RAB29 1.358133403 3.692042075 27.55575899 5.26E-89 4.77E-88 191.9568306

TOR1B 1.250236066 4.195822768 27.55504206 5.29E-89 4.80E-88 191.9506646

DDHD2 -1.5857504 3.888572817 -27.5531675 5.38E-89 4.88E-88 191.934542

PPP1R16A -2.466837344 4.839219529 -27.54774959 5.63E-89 5.11E-88 191.8879416

MRC2 2.682034017 4.291020372 27.54671371 5.68E-89 5.15E-88 191.8790314

USHBP1 -1.363262559 1.789672012 -27.54662485 5.69E-89 5.15E-88 191.8782671

DKK3 2.235830258 4.628080041 27.54626659 5.71E-89 5.17E-88 191.8751855

CXorf38 1.005483029 3.046906142 27.53986212 6.03E-89 5.45E-88 191.8200939

ARL2BP -1.458526375 3.460974574 -27.53623745 6.22E-89 5.62E-88 191.7889122

HLA-DOA 2.766966484 2.859834795 27.5359336 6.24E-89 5.64E-88 191.7862982

EPS8L3 4.159161663 3.283136461 27.53193346 6.45E-89 5.83E-88 191.7518845

SLC25A36 -1.821424867 4.623434436 -27.53058942 6.53E-89 5.90E-88 191.7403212

FZD1 1.689265756 3.098571433 27.52522864 6.84E-89 6.17E-88 191.694198

DUSP5 2.71415591 3.992061429 27.52142409 7.06E-89 6.37E-88 191.6614622

TRIM45 -1.667854206 2.52526701 -27.51503829 7.46E-89 6.73E-88 191.6065125

GMNN -2.371063719 4.862883939 -27.51329416 7.58E-89 6.83E-88 191.5915035

SNN 1.554954077 3.396400012 27.50908365 7.86E-89 7.08E-88 191.5552687

ETS1 2.006398038 4.41285863 27.50862913 7.89E-89 7.10E-88 191.551357

TAPBP 1.847238693 6.078424422 27.50665931 8.02E-89 7.22E-88 191.5344044

LPA -0.328768837 0.211492777 -27.50279613 8.29E-89 7.46E-88 191.5011557

LY86 2.581661079 2.518028622 27.49958178 8.52E-89 7.67E-88 191.4734898

TMEM54 2.101782761 5.748924582 27.49760842 8.67E-89 7.79E-88 191.4565046

NPIPB8 -1.151152361 0.573171152 -27.49663293 8.74E-89 7.86E-88 191.448108

LCP1 2.919411709 4.035334165 27.49060825 9.21E-89 8.27E-88 191.3962483

RANBP17 -1.454973554 1.599035516 -27.4881879 9.40E-89 8.44E-88 191.3754131

ADAMTS2 2.444431775 3.740092776 27.48548643 9.62E-89 8.63E-88 191.352157

IZUMO1 -2.452495282 1.385311074 -27.48395874 9.75E-89 8.74E-88 191.3390052

OAS1 2.665227535 3.527414733 27.47815215 1.02E-88 9.19E-88 191.2890143

DDX11 -2.233287026 3.724434674 -27.46922106 1.11E-88 9.92E-88 191.2121157

RAD54B -1.083694914 1.242166282 -27.46543149 1.14E-88 1.02E-87 191.1794838

PRRG1 1.447952121 2.024995997 27.46247678 1.17E-88 1.05E-87 191.1540397

METTL7B 2.685785819 3.044769988 27.45802652 1.22E-88 1.09E-87 191.115715

TMED6 -4.151163399 4.247556765 -27.4578366 1.22E-88 1.09E-87 191.1140793

MAOB 2.397016422 4.05405005 27.45780085 1.22E-88 1.09E-87 191.1137714

GORASP1 -1.455875699 4.947147133 -27.45401856 1.26E-88 1.13E-87 191.0811972

MFAP1 1.242227162 4.330956125 27.44890143 1.32E-88 1.18E-87 191.0371242

SFRP2 5.437409673 6.711638089 27.44689307 1.34E-88 1.20E-87 191.0198257

PPP1CB 1.581335368 5.796373081 27.44619942 1.35E-88 1.20E-87 191.013851

RUNX2 1.86834814 1.702473322 27.44610386 1.35E-88 1.20E-87 191.013028

MRPS22 -1.437323331 4.127352345 -27.44542494 1.36E-88 1.21E-87 191.0071801

ORAI1 1.47887138 3.887707564 27.44359592 1.38E-88 1.23E-87 190.9914255

MZT2A -2.309213621 5.166628083 -27.43675103 1.46E-88 1.30E-87 190.9324625

VLDLR -2.119062569 3.39453988 -27.43436677 1.49E-88 1.33E-87 190.9119228

CAPN5 2.737745982 4.06240675 27.42340422 1.64E-88 1.46E-87 190.8174751

WNT2 2.717011283 1.599152858 27.42038164 1.68E-88 1.50E-87 190.7914317

PIM2 1.967671809 3.329594584 27.41800301 1.72E-88 1.53E-87 190.7709359

ZSWIM3 1.07035704 1.763569494 27.41749208 1.73E-88 1.54E-87 190.7665333

ZSCAN30 -1.236387494 2.715571493 -27.41266304 1.80E-88 1.60E-87 190.7249209

SGK3 -1.469821375 2.647942 -27.39638953 2.07E-88 1.84E-87 190.5846702

PSMC6 -1.390180593 4.826766955 -27.39432972 2.11E-88 1.87E-87 190.5669158

LATS2 1.610466954 3.076555089 27.39065025 2.18E-88 1.93E-87 190.5351996

SP2 1.046555194 3.721964093 27.38492726 2.29E-88 2.03E-87 190.4858657

ARL6IP1 1.928800725 6.175825914 27.37220998 2.55E-88 2.26E-87 190.376225

MTUS2 -2.494578704 2.00968912 -27.36428371 2.73E-88 2.42E-87 190.3078799

PPP3R1 1.097435244 4.524001217 27.35407407 2.98E-88 2.64E-87 190.2198353

HMMR 1.750661868 1.370643492 27.33991528 3.37E-88 2.98E-87 190.0977144

RPA2 1.396045618 4.753716139 27.33714513 3.45E-88 3.05E-87 190.0738189

NOTCH3 2.051135569 4.279856165 27.33209923 3.60E-88 3.19E-87 190.0302902

INCA1 1.082155165 1.849021904 27.33138769 3.63E-88 3.21E-87 190.0241518

FABP3 2.23114754 2.723048683 27.32390007 3.87E-88 3.42E-87 189.9595534

MAPK6 1.337300106 3.375402519 27.31413296 4.21E-88 3.72E-87 189.8752791

CPD 1.631378845 4.157265269 27.31040765 4.35E-88 3.84E-87 189.8431329

PWP2 -1.839539587 1.284139367 -27.30678337 4.48E-88 3.96E-87 189.8118567

PLEKHH3 -2.502272035 4.944315252 -27.29392942 5.01E-88 4.42E-87 189.7009201

IGSF6 2.161477261 1.974440273 27.28959972 5.20E-88 4.58E-87 189.663548

FAM168A 1.285620676 3.698019783 27.28365148 5.47E-88 4.82E-87 189.6122018

ZNF444 -1.802779236 4.404083987 -27.27945935 5.67E-88 5.00E-87 189.5760122

TAF6L -1.434245016 3.692058376 -27.27642423 5.82E-88 5.13E-87 189.5498095

B3GLCT 0.915744114 2.243166232 27.27582465 5.85E-88 5.15E-87 189.5446331

DACT1 2.199334444 2.552055539 27.25541599 6.98E-88 6.14E-87 189.368412

PDCL3 1.146958704 3.795973776 27.25239384 7.17E-88 6.30E-87 189.3423128

RNFT1 -1.48543864 3.189219531 -27.25101168 7.25E-88 6.37E-87 189.3303762

WNT7B 2.52597017 1.88066107 27.24893385 7.38E-88 6.49E-87 189.3124311

CRADD -1.263007805 2.798773027 -27.24516389 7.63E-88 6.70E-87 189.2798708

CELSR1 2.042445868 2.230723644 27.24482012 7.65E-88 6.71E-87 189.2769017

ZNF140 -1.147528942 3.055376703 -27.24429576 7.69E-88 6.74E-87 189.2723727

SH3BGRL 1.705703018 6.017315038 27.23986438 7.98E-88 7.00E-87 189.2340972

HS2ST1 1.225423161 3.265149648 27.23491972 8.33E-88 7.30E-87 189.1913857

LHX4 -0.695445814 0.78490207 -27.2347138 8.35E-88 7.31E-87 189.1896069

PRR11 1.75527984 1.367722394 27.23352867 8.43E-88 7.38E-87 189.1793694

MELK 2.1268779 1.5670499 27.22903727 8.77E-88 7.67E-87 189.1405698

AL139353.1 -0.895519598 0.664518959 -27.22140405 9.36E-88 8.19E-87 189.074624

SLC4A5 -0.749291772 0.665065541 -27.22008622 9.47E-88 8.28E-87 189.0632381

ZNF208 -1.066455773 0.86038825 -27.21875672 9.58E-88 8.37E-87 189.0517512

TAF13 1.361181644 4.55510367 27.21357107 1.00E-87 8.75E-87 189.0069451

GALNT1 1.47641136 4.537166886 27.20961621 1.04E-87 9.05E-87 188.9727715

STOM 2.10264473 5.657478706 27.20105432 1.12E-87 9.74E-87 188.8987826

AD000671.2 -0.859921381 0.493207047 -27.19387588 1.19E-87 1.04E-86 188.8367425

FBLN2 2.984305648 4.089008456 27.19213165 1.21E-87 1.05E-86 188.8216669

SYBU -3.214915969 4.616595863 -27.191998 1.21E-87 1.05E-86 188.8205117

BAG3 1.924310116 4.849170981 27.18364608 1.30E-87 1.13E-86 188.7483203

GID8 1.17918355 4.693345519 27.18168404 1.32E-87 1.15E-86 188.7313598

FXR1 -1.5807687 4.945995112 -27.16549751 1.52E-87 1.32E-86 188.5914216

KIF3C 1.566048832 2.085827716 27.16307566 1.55E-87 1.35E-86 188.5704813

CCDC51 1.170066239 2.975013844 27.15914329 1.60E-87 1.39E-86 188.5364788

HJURP 1.976052992 1.437071854 27.15853523 1.61E-87 1.40E-86 188.5312209

BRD8 -1.563795255 4.629348868 -27.15732563 1.63E-87 1.42E-86 188.5207612

TRAF2 1.361955544 3.5754461 27.15444767 1.67E-87 1.45E-86 188.4958744

MN1 1.820565107 1.756268458 27.15312223 1.69E-87 1.47E-86 188.4844124

TRMT2A -1.715247897 5.010803246 -27.1529449 1.69E-87 1.47E-86 188.4828789

AC093323.1 1.383521691 3.288564639 27.15097035 1.72E-87 1.49E-86 188.4658033

ERC2 -1.012177513 0.742369935 -27.15077789 1.72E-87 1.49E-86 188.4641388

CSE1L 1.273486403 4.707776117 27.1499219 1.74E-87 1.50E-86 188.4567362

CUZD1 -6.682804094 6.473337373 -27.14599669 1.80E-87 1.56E-86 188.4227897

YWHAH 1.77447548 6.607058156 27.14361437 1.83E-87 1.59E-86 188.4021857

TSPAN15 2.441811106 5.22768484 27.13657164 1.95E-87 1.69E-86 188.3412714

C6orf163 -0.883019449 0.756555465 -27.13338801 2.00E-87 1.73E-86 188.3137335

TJAP1 -1.361890751 4.113245458 -27.13012656 2.06E-87 1.78E-86 188.2855213

EED -1.093024213 3.170413028 -27.12934748 2.07E-87 1.79E-86 188.2787818

PCF11 -1.400190898 4.259006431 -27.12146172 2.22E-87 1.92E-86 188.2105625

EPHX2 -2.587494742 4.381821611 -27.12145534 2.22E-87 1.92E-86 188.2105073

PRSS50 -1.781426443 1.537853051 -27.11437388 2.36E-87 2.04E-86 188.1492397

EFCAB14 1.372455407 4.984404186 27.104554 2.57E-87 2.22E-86 188.0642702

GPR176 1.700652064 1.935236677 27.10403798 2.58E-87 2.23E-86 188.0598048

UBE2L3 1.178178088 5.046766957 27.09665324 2.75E-87 2.37E-86 187.9958982

TNS4 4.196034953 2.478516146 27.09143461 2.88E-87 2.48E-86 187.9507331

KIRREL1 2.041651416 3.152649076 27.08275312 3.10E-87 2.67E-86 187.8755913

IWS1 1.058856126 4.459411438 27.07443081 3.33E-87 2.87E-86 187.8035501

FRA10AC1 -1.269915939 3.16329638 -27.07190777 3.41E-87 2.93E-86 187.7817081

BRD9 -1.266270922 3.769147284 -27.06199662 3.71E-87 3.19E-86 187.6958999

ZNF169 -1.011592469 1.57981827 -27.05453749 3.96E-87 3.41E-86 187.6313131

TMBIM4 -1.744766367 5.508236988 -27.05212781 4.04E-87 3.48E-86 187.6104469

ITSN2 -1.50906215 4.122588445 -27.042534 4.39E-87 3.77E-86 187.5273644

MTF1 0.942124477 2.211986132 27.03520138 4.68E-87 4.02E-86 187.4638566

PHLDA2 3.980311086 4.005003227 27.03279151 4.78E-87 4.10E-86 187.4429833

SLC6A14 3.832853331 2.138596571 27.0314005 4.84E-87 4.15E-86 187.4309347

TMC5 3.601663782 3.563866496 27.02415516 5.15E-87 4.42E-86 187.3681735

DIPK2A 1.183318706 2.616535818 27.02141442 5.27E-87 4.52E-86 187.3444308

PLEC 2.301622969 5.557827758 27.01872638 5.40E-87 4.63E-86 187.3211438

IGFBP7 2.839579475 8.967895109 27.01468002 5.59E-87 4.79E-86 187.2860879

CRACR2B -3.373962502 6.043363479 -27.01178325 5.73E-87 4.91E-86 187.2609904

ERVW-1 -0.316915597 0.204388788 -27.00959746 5.84E-87 5.00E-86 187.2420522

OARD1 -1.943307214 4.192954049 -27.0070152 5.97E-87 5.11E-86 187.2196781

CHAD -2.832051853 2.717442878 -27.0052972 6.06E-87 5.18E-86 187.204792

PGA4 -3.34678484 1.625069318 -27.00122744 6.28E-87 5.37E-86 187.1695271

NRG2 -1.39167625 1.275788066 -27.00052661 6.32E-87 5.40E-86 187.1634541

CEMIP 2.986848813 2.447437376 26.99877002 6.42E-87 5.48E-86 187.1482323

GOLIM4 1.704940061 4.162985782 26.99433144 6.67E-87 5.69E-86 187.109768

POLR3K 1.201107826 3.315673588 26.99002355 6.92E-87 5.91E-86 187.0724341

AKAP9 -1.647616511 4.045175531 -26.9755806 7.84E-87 6.69E-86 186.94725

DYM -1.616767544 4.27479918 -26.96690508 8.46E-87 7.21E-86 186.8720434

CCNB1 1.972245203 3.168362486 26.9627479 8.77E-87 7.47E-86 186.8360025

FAM83F -1.759630105 2.52785406 -26.96168991 8.85E-87 7.53E-86 186.8268299

ZBTB12 1.196751443 2.074470956 26.95844723 9.10E-87 7.75E-86 186.7987155

SORT1 1.448022905 4.167451916 26.95742846 9.18E-87 7.81E-86 186.7898824

LRRFIP2 -1.059485785 3.874701659 -26.9490873 9.87E-87 8.39E-86 186.717557

SPATA5 0.850963233 1.312293673 26.9421967 1.05E-86 8.90E-86 186.6578033

MMP1 5.839573162 3.844220935 26.93339502 1.13E-86 9.61E-86 186.581469

CROCC -1.708169672 3.856388553 -26.9297933 1.17E-86 9.91E-86 186.5502299

ALG12 -1.277954018 3.220634563 -26.92810642 1.18E-86 1.00E-85 186.5355984

CD86 2.154180641 2.000741255 26.92629778 1.20E-86 1.02E-85 186.5199104

MAGEF1 1.422915584 5.127179699 26.91673646 1.31E-86 1.11E-85 186.4369701

AC011511.1 -0.871953534 0.742517236 -26.91503294 1.33E-86 1.12E-85 186.4221918

RNF168 0.933494795 2.771376688 26.91405621 1.34E-86 1.13E-85 186.4137183

PGD 1.607674397 5.490711431 26.90856477 1.40E-86 1.19E-85 186.3660759

BCL2L2-PABPN1 -1.6758967 1.35448919 -26.90018259 1.51E-86 1.28E-85 186.2933477

ACY1 -1.837460609 2.991263111 -26.8978038 1.54E-86 1.30E-85 186.2727065

TBRG1 -1.352575172 4.101002465 -26.89324149 1.60E-86 1.35E-85 186.2331168

POMT2 -1.462067951 3.306540714 -26.8898503 1.65E-86 1.39E-85 186.203688

TRAF5 -2.032328433 3.990866672 -26.88794932 1.68E-86 1.42E-85 186.1871907

BRI3 -2.012932322 6.424012776 -26.88471182 1.72E-86 1.46E-85 186.1590936

GNB4 1.795782879 2.298769584 26.88345998 1.74E-86 1.47E-85 186.148229

UHRF2 -1.277687579 3.736221499 -26.88332519 1.74E-86 1.47E-85 186.1470592

SLC35C1 1.441749806 4.195419936 26.87455912 1.88E-86 1.59E-85 186.0709744

TPRN -2.474746614 4.948412711 -26.8709424 1.94E-86 1.64E-85 186.0395807

CRIM1 1.728269793 4.052888158 26.86425648 2.06E-86 1.74E-85 185.9815416

FBXO3 -1.192893103 3.57821914 -26.85934983 2.15E-86 1.81E-85 185.9389449

NAPA -1.671581235 5.626276789 -26.85426985 2.24E-86 1.89E-85 185.8948404

CALML6 -1.306926025 1.002966842 -26.8521364 2.29E-86 1.93E-85 185.8763169

HIF1A 2.054636364 5.366744952 26.84178395 2.50E-86 2.11E-85 185.7864251

JRKL 1.262066173 2.985170241 26.8381171 2.58E-86 2.17E-85 185.7545823

PIGG -1.504682204 4.053688351 -26.8329697 2.70E-86 2.27E-85 185.7098799

THOC2 -1.361879583 4.270379266 -26.82863222 2.80E-86 2.36E-85 185.6722089

CDKN2B 2.572477538 2.600974334 26.82724904 2.84E-86 2.38E-85 185.6601955

TCEAL8 1.440665841 5.650844666 26.82633144 2.86E-86 2.40E-85 185.6522257

SLC33A1 -1.594049552 4.07759656 -26.8258941 2.87E-86 2.41E-85 185.6484272

KCTD17 1.499896462 3.20961224 26.81532176 3.15E-86 2.64E-85 185.556594

RTL8C 1.796694927 6.424413577 26.81485638 3.16E-86 2.65E-85 185.5525513

OGT -2.080854782 6.001830726 -26.810775 3.27E-86 2.75E-85 185.5170961

AKAP11 1.29128047 3.232177741 26.80634766 3.40E-86 2.85E-85 185.4786333

FGD5 1.667175493 2.819660117 26.80581999 3.42E-86 2.86E-85 185.474049

N4BP2L1 -1.559646958 3.409400085 -26.80251632 3.52E-86 2.95E-85 185.4453465

SOX10 -0.845621566 0.410519764 -26.80216849 3.53E-86 2.95E-85 185.4423245

PXDN 2.215665999 3.224026678 26.79862392 3.64E-86 3.04E-85 185.4115275

CD248 2.826781165 4.700372223 26.7879726 3.99E-86 3.34E-85 185.3189749

CYP27B1 -1.460260344 2.13870702 -26.78577198 4.07E-86 3.40E-85 185.2998514

NCOA3 1.133659962 3.554670716 26.77742007 4.37E-86 3.65E-85 185.2272679

THAP12 -1.596116872 4.154774618 -26.7771929 4.38E-86 3.66E-85 185.2252935

TK1 2.560410293 3.697624972 26.77691938 4.39E-86 3.67E-85 185.2229163

CCDC84 -1.836145348 3.892224424 -26.76345106 4.94E-86 4.12E-85 185.1058502

INIP 0.946809547 3.010331446 26.76289979 4.96E-86 4.14E-85 185.1010581

EXOC2 0.998932253 3.194756476 26.76196271 5.00E-86 4.17E-85 185.0929122

RBM23 -1.658102412 4.245454176 -26.75508897 5.31E-86 4.43E-85 185.0331568

ZNF496 -1.24424466 3.514337134 -26.75415662 5.35E-86 4.46E-85 185.0250512

FARSB 1.214270036 4.244672387 26.75311844 5.40E-86 4.50E-85 185.0160254

CSNK2B -1.778861877 6.007879047 -26.74440433 5.83E-86 4.85E-85 184.9402613

ZNF469 1.823491728 1.267810803 26.73760257 6.18E-86 5.14E-85 184.8811179

TUT1 -1.399046158 3.764530243 -26.73417332 6.37E-86 5.30E-85 184.8512976

ZSCAN18 -2.034594258 3.821037746 -26.73019231 6.59E-86 5.48E-85 184.8166775

CORO7 -1.440591079 3.105894794 -26.72872175 6.68E-86 5.55E-85 184.8038886

SNTG2 -2.010406402 1.360583768 -26.72473038 6.91E-86 5.74E-85 184.7691759

PGM2L1 1.68631255 2.51797897 26.72434228 6.93E-86 5.76E-85 184.7658005

OXCT1 1.592281406 2.973658598 26.72382086 6.97E-86 5.78E-85 184.7612656

B4GALNT3 2.277521907 3.512921151 26.72330327 7.00E-86 5.81E-85 184.7567639

ITPKC 1.729498328 4.670783393 26.72093308 7.14E-86 5.92E-85 184.7361493

FAM43A 1.640246434 2.602306284 26.71790312 7.33E-86 6.08E-85 184.7097954

AOX1 -3.427372037 4.255877976 -26.71300451 7.65E-86 6.34E-85 184.6671862

MTFR1L -1.478241181 4.699095664 -26.71037619 7.83E-86 6.49E-85 184.6443234

ACAD10 -1.306790196 3.505927624 -26.71022498 7.84E-86 6.49E-85 184.6430081

RPL18A -2.667638559 9.12776818 -26.70832802 7.97E-86 6.60E-85 184.6265066

ZNF429 -1.369098874 2.362663921 -26.7056553 8.16E-86 6.75E-85 184.603256

CD83 2.193521215 2.51959985 26.70527048 8.18E-86 6.77E-85 184.5999084

MACF1 -1.708279593 4.731636044 -26.70146845 8.46E-86 6.99E-85 184.5668321

AC114490.2 -0.558531404 0.419227401 -26.69411866 9.02E-86 7.45E-85 184.5028872

DEF8 -1.418493787 4.628496284 -26.6824371 9.98E-86 8.24E-85 184.4012421

EAF1 1.027441016 3.195040008 26.67578306 1.06E-85 8.73E-85 184.3433362

FOSL1 3.177193206 2.864587665 26.67482317 1.07E-85 8.80E-85 184.3349825

C4A -3.619039734 4.055275768 -26.67072277 1.11E-85 9.12E-85 184.2992962

MCM2 1.730736688 2.760147425 26.66649345 1.15E-85 9.45E-85 184.2624861

GAPDH 2.67872431 9.866206556 26.6641677 1.17E-85 9.64E-85 184.2422429

C2orf74 -1.718314532 3.088688763 -26.66222753 1.19E-85 9.80E-85 184.2253552

BCAR3 1.744782423 3.070144663 26.66194141 1.19E-85 9.82E-85 184.2228648

ZNF771 -1.15705273 1.747930667 -26.65279765 1.29E-85 1.06E-84 184.1432697

RBPMS -1.937581279 5.158139663 -26.64879816 1.34E-85 1.10E-84 184.1084517

CD300A 2.01029863 2.588995828 26.64615022 1.37E-85 1.13E-84 184.0853987

ENDOV -1.277332101 3.06214232 -26.64505866 1.38E-85 1.14E-84 184.0758954

NME6 -1.111304015 3.175073693 -26.64369183 1.40E-85 1.15E-84 184.0639953

ARMC1 1.099404765 3.698051316 26.63777165 1.47E-85 1.21E-84 184.0124497

TRAF3IP1 1.004006938 2.974373914 26.63237418 1.54E-85 1.27E-84 183.9654518

WRAP73 -1.338103529 3.524463999 -26.63132781 1.56E-85 1.28E-84 183.9563403

AP3S2 -1.300618563 3.314538451 -26.62967344 1.58E-85 1.30E-84 183.9419342

FSTL1 2.643553917 5.42292487 26.62680355 1.62E-85 1.33E-84 183.9169426

PIGU 1.163964541 3.902681746 26.62642202 1.62E-85 1.33E-84 183.9136201

CLCN4 1.437402623 1.582055383 26.62641209 1.62E-85 1.33E-84 183.9135337

RBM25 -1.605518042 5.030135151 -26.62190226 1.69E-85 1.38E-84 183.8742592

ADSS 1.329767175 4.598600401 26.61062706 1.86E-85 1.53E-84 183.7760574

ZNF141 -1.22165055 2.177045499 -26.60845548 1.90E-85 1.55E-84 183.7571423

EID2 1.211994799 3.226132204 26.60714421 1.92E-85 1.57E-84 183.7457205

SKA3 1.585760587 1.173565228 26.60342831 1.98E-85 1.62E-84 183.713352

TCF19 1.446325219 2.570046813 26.59967913 2.05E-85 1.68E-84 183.6806922

ATXN7L3B 1.264949561 4.471003044 26.59944604 2.05E-85 1.68E-84 183.6786616

ANKRD20A2 -0.845412334 0.418881971 -26.58204577 2.39E-85 1.95E-84 183.5270628

TMEM92 2.742931082 2.398896304 26.56373868 2.80E-85 2.29E-84 183.3675261

AASS -2.34192792 3.130440974 -26.56118457 2.87E-85 2.34E-84 183.3452654

AC105052.3 -2.65748564 1.528059797 -26.55849031 2.93E-85 2.39E-84 183.3217823

ITM2C 2.009493521 6.3843894 26.55652568 2.99E-85 2.43E-84 183.3046582

DOP1B 1.486961055 2.541464543 26.55186053 3.11E-85 2.53E-84 183.2639939

AGAP9 -2.223595797 3.302100559 -26.54903748 3.19E-85 2.60E-84 183.2393854

RHNO1 1.274239938 3.784076781 26.54478091 3.31E-85 2.69E-84 183.202279

POLL -1.505413744 4.327817747 -26.54397722 3.33E-85 2.71E-84 183.1952727

FLCN -1.543548202 3.636815483 -26.54325663 3.35E-85 2.73E-84 183.1889907

SH3BGRL2 1.783116704 4.160119331 26.53775169 3.52E-85 2.86E-84 183.1409979

SUSD6 1.452241011 4.856923597 26.53377469 3.64E-85 2.96E-84 183.1063236

RPL37 -2.393052859 8.478080192 -26.53018353 3.76E-85 3.05E-84 183.0750119

C1QA 3.389739963 6.183744214 26.51736788 4.20E-85 3.41E-84 182.9632588

SOCS6 1.236386236 3.147918977 26.5128921 4.37E-85 3.54E-84 182.9242254

ZNF426 0.831305352 1.920346111 26.50550792 4.66E-85 3.78E-84 182.8598229

RCN3 2.425526336 4.783500836 26.50505266 4.67E-85 3.79E-84 182.855852

GLIPR2 2.166321268 3.263533417 26.49649742 5.04E-85 4.08E-84 182.7812277

KLK10 4.348449613 2.946921378 26.49419617 5.14E-85 4.16E-84 182.7611533

DCAF7 1.295798132 4.53418135 26.49213423 5.23E-85 4.24E-84 182.7431658

NBL1 2.257311162 5.373536401 26.49131914 5.27E-85 4.27E-84 182.7360553

DCAF12 1.189961739 4.198470373 26.49029518 5.32E-85 4.30E-84 182.7271225

ZNF148 0.980398053 2.773968751 26.4887664 5.39E-85 4.36E-84 182.7137856

NUDT22 -1.768003653 4.78546017 -26.4835458 5.64E-85 4.56E-84 182.6682394

NOSIP -1.980203961 5.622184163 -26.48259574 5.69E-85 4.60E-84 182.6599504

ENKD1 -2.155552468 5.370612523 -26.47793631 5.92E-85 4.79E-84 182.619297

DENR 1.110377194 4.61197909 26.4761012 6.02E-85 4.86E-84 182.6032851

LMBR1L -1.591676679 4.337091597 -26.47372269 6.14E-85 4.96E-84 182.5825313

MAL2 2.628675521 5.927794599 26.47361796 6.15E-85 4.96E-84 182.5816174

TNRC6A -1.275092185 3.953326073 -26.47358318 6.15E-85 4.96E-84 182.5813139

FAM229A -1.513004563 2.24362765 -26.47343712 6.16E-85 4.97E-84 182.5800394

ESYT2 1.391117171 4.926778687 26.46558045 6.59E-85 5.32E-84 182.5114806

MALSU1 -1.410011728 4.048960564 -26.46488902 6.63E-85 5.35E-84 182.5054467

ATG12 -1.232952004 4.045284159 -26.46274743 6.76E-85 5.44E-84 182.4867575

ITGA1 1.574857112 3.006585069 26.46230195 6.79E-85 5.46E-84 182.4828697

MED18 1.207487714 3.370135864 26.46159486 6.83E-85 5.50E-84 182.476699

SCFD1 -1.492797551 4.77853473 -26.45795793 7.05E-85 5.67E-84 182.4449584

BANP -1.33647848 2.694567529 -26.45645045 7.14E-85 5.74E-84 182.4318017

TRAK1 1.428525039 4.093480636 26.45330766 7.34E-85 5.90E-84 182.404372

RPS6KA4 1.548090487 4.428156197 26.44684052 7.76E-85 6.24E-84 182.3479243

ESM1 2.512751071 1.847318727 26.44240926 8.07E-85 6.48E-84 182.3092438

ZNF185 2.220764141 2.982920179 26.44218313 8.09E-85 6.49E-84 182.3072698

NUFIP1 0.836117836 2.109004312 26.44137163 8.14E-85 6.54E-84 182.300186

DIS3L2 -1.091191331 3.108012184 -26.43157781 8.87E-85 7.12E-84 182.2146867

CREBL2 1.468364413 4.550764472 26.4258951 9.32E-85 7.47E-84 182.1650721

PM20D1 -4.490728901 3.122105279 -26.42567585 9.34E-85 7.49E-84 182.1631579

JPT1 2.153661349 5.165134053 26.42235244 9.61E-85 7.70E-84 182.1341401

CEACAM5 6.907044937 3.954060202 26.41712851 1.01E-84 8.06E-84 182.0885257

PDIA2 -6.126826027 6.284672169 -26.41125147 1.06E-84 8.48E-84 182.0372048

ABLIM3 2.069513594 2.968064146 26.40854949 1.08E-84 8.68E-84 182.0136086

RBP1 -3.316342894 6.663093882 -26.40755862 1.09E-84 8.75E-84 182.0049553

STC1 2.560211129 3.259636922 26.40647238 1.10E-84 8.83E-84 181.9954689

TMEM115 1.221500464 5.137275713 26.40078738 1.16E-84 9.28E-84 181.9458182

PLEKHA2 1.868327294 3.265361001 26.3959668 1.21E-84 9.67E-84 181.9037142

BCL2A1 2.630398797 2.196902638 26.39380136 1.23E-84 9.85E-84 181.8847999

SGSH -1.544061345 4.251094082 -26.39139448 1.26E-84 1.01E-83 181.8637761

KPNA6 1.0607231 3.983426198 26.38524884 1.33E-84 1.06E-83 181.8100918

TCEAL7 1.897851182 2.005462219 26.3763889 1.44E-84 1.15E-83 181.7326896

WDR45 -1.542642446 5.158237793 -26.37451484 1.46E-84 1.16E-83 181.7163163

PGM2 1.298450913 3.490544321 26.37423067 1.46E-84 1.17E-83 181.7138336

SHCBP1 1.520939021 1.272817231 26.37357815 1.47E-84 1.17E-83 181.7081325

MET 2.638970158 3.935815559 26.36164154 1.63E-84 1.30E-83 181.6038342

LGMN 1.700407635 5.733471387 26.36111436 1.64E-84 1.31E-83 181.5992275

ILKAP -1.365980241 4.297284277 -26.35451207 1.74E-84 1.38E-83 181.5415314

LAYN 1.706224101 2.428157562 26.352654 1.77E-84 1.40E-83 181.5252933

IFT80 -1.181851718 3.016128294 -26.35239606 1.77E-84 1.41E-83 181.5230391

SNIP1 0.836138459 2.651947158 26.35220442 1.77E-84 1.41E-83 181.5213643

SLC25A22 -1.942555759 4.920132123 -26.35000045 1.81E-84 1.44E-83 181.5021026

FHL2 2.286993642 4.156187259 26.34614725 1.87E-84 1.48E-83 181.468426

TMEM154 1.542761105 1.191922838 26.3459245 1.87E-84 1.49E-83 181.4664792

RASL12 1.751200821 2.444996274 26.34237674 1.93E-84 1.53E-83 181.4354706

LIMS4 -1.879315882 0.912606002 -26.34049958 1.96E-84 1.56E-83 181.4190631

FAM83D 2.590922356 1.771866609 26.33953891 1.98E-84 1.57E-83 181.410666

C15orf48 3.424188176 4.576630264 26.33903859 1.99E-84 1.58E-83 181.4062929

UGCG 1.602716149 4.447264289 26.33383971 2.08E-84 1.65E-83 181.3608485

SLIRP -1.733936132 5.418295202 -26.33154815 2.12E-84 1.68E-83 181.3408165

RPL31 -2.492035089 9.140214485 -26.32284893 2.29E-84 1.81E-83 181.264766

TMEM184B 1.389193064 4.894457752 26.31905103 2.37E-84 1.87E-83 181.2315612

RPL26 -2.548358878 9.034554618 -26.31764938 2.40E-84 1.90E-83 181.2193063

BCAT2 -2.05128 4.935547573 -26.31554415 2.44E-84 1.93E-83 181.2008994

RFLNB 1.841950888 2.844008586 26.31371163 2.48E-84 1.96E-83 181.1848765

GEMIN5 0.922809167 2.766541015 26.31275572 2.50E-84 1.98E-83 181.1765182

NDUFC2 -1.683704211 5.652761222 -26.31057166 2.55E-84 2.01E-83 181.1574208

MDM1 -1.16050988 2.415858497 -26.29051122 3.04E-84 2.40E-83 180.9819879

ETV7 1.936864904 2.137799499 26.29014626 3.05E-84 2.41E-83 180.9787959

CXCR3 1.847747241 1.326080866 26.28970319 3.06E-84 2.41E-83 180.9749205

TTLL11 -1.039274694 1.949935973 -26.2829282 3.25E-84 2.56E-83 180.915661

ZNF398 0.848486948 2.486969967 26.28093941 3.30E-84 2.60E-83 180.8982644

RBM6 -1.943430191 5.519448879 -26.27996591 3.33E-84 2.63E-83 180.8897488

ZBTB41 1.025933138 2.367460967 26.2776072 3.40E-84 2.68E-83 180.8691156

ABRACL 1.856955057 4.598517409 26.27222585 3.57E-84 2.81E-83 180.8220391

CAB39 1.324486812 4.814940394 26.27151763 3.59E-84 2.82E-83 180.8158432

ATG2B -1.396989306 3.37065199 -26.26714768 3.73E-84 2.93E-83 180.7776118

MTMR2 1.065684309 3.491071417 26.25856276 4.02E-84 3.16E-83 180.7024985

KIFC3 -2.074684031 5.03988513 -26.25644467 4.09E-84 3.22E-83 180.6839652

PMEPA1 2.861885076 5.840891622 26.25446117 4.17E-84 3.27E-83 180.666609

SLC16A13 1.259351248 1.877620074 26.24627972 4.47E-84 3.51E-83 180.5950145

HAUS3 -0.991998314 2.699128109 -26.24077837 4.69E-84 3.69E-83 180.5468688

DOK5 2.129968915 1.771783164 26.24039871 4.71E-84 3.70E-83 180.543546

STOX2 -1.165782861 1.657432159 -26.2371451 4.85E-84 3.80E-83 180.5150701

PIP4K2C 1.33345547 4.71718668 26.23423916 4.97E-84 3.90E-83 180.4896358

SSR2 -1.978113953 7.229573925 -26.2334729 5.00E-84 3.92E-83 180.482929

RRM2 2.360646014 2.214704621 26.22651716 5.32E-84 4.17E-83 180.4220446

CD14 2.51964324 5.045315902 26.22257148 5.51E-84 4.31E-83 180.3875052

CDNF -1.446342114 1.991188975 -26.22034949 5.61E-84 4.39E-83 180.3680537

SDC4 2.801605312 7.234742153 26.21826489 5.72E-84 4.47E-83 180.3498045

CLIC3 3.950334689 2.889517263 26.20762624 6.27E-84 4.91E-83 180.2566629

TTC14 -1.496781616 4.079048349 -26.19580702 6.96E-84 5.44E-83 180.1531706

FOXC1 1.883270522 2.621756173 26.19273936 7.15E-84 5.59E-83 180.1263067

GCNA -1.133563168 1.344549784 -26.18427879 7.70E-84 6.01E-83 180.0522111

HLA-DQA2 3.913197573 2.643006234 26.18308556 7.78E-84 6.07E-83 180.0417606

STK17B 1.811570277 3.146912931 26.18163079 7.88E-84 6.15E-83 180.029019

PHF12 -1.204979652 3.968959752 -26.17836724 8.10E-84 6.32E-83 180.0004345

ITPRIP 1.729788506 3.168831867 26.17286148 8.50E-84 6.63E-83 179.9522086

HECTD4 -1.419291773 3.35134726 -26.16457842 9.14E-84 7.13E-83 179.8796494

GPR183 2.831587957 2.751039101 26.15379392 1.00E-83 7.83E-83 179.7851662

PBK 1.87578259 1.431232773 26.15324326 1.01E-83 7.87E-83 179.7803415

STRA6 2.515318002 1.805685879 26.14964217 1.04E-83 8.12E-83 179.7487892

CD2 2.682583238 2.450336436 26.14739533 1.06E-83 8.27E-83 179.7291019

NUCKS1 1.596873829 5.706807094 26.14674607 1.07E-83 8.32E-83 179.7234128

ATP4A -2.844963319 1.481922496 -26.14502683 1.09E-83 8.44E-83 179.7083481

SPINDOC 1.11565946 3.419225172 26.14385817 1.10E-83 8.52E-83 179.6981075

INTU -1.092345236 1.805171055 -26.14008274 1.13E-83 8.81E-83 179.6650236

CLIP3 2.02315018 3.037441666 26.13169237 1.22E-83 9.47E-83 179.5914937

ATP6V1G1 1.648400987 6.572261236 26.1267746 1.27E-83 9.89E-83 179.5483927

1-Mar -1.543214716 2.291983747 -26.12614544 1.28E-83 9.94E-83 179.5428783

SF3B3 -1.507185512 5.371452177 -26.12564425 1.29E-83 9.98E-83 179.5384855

MXRA5 3.341712032 4.114774902 26.12394597 1.31E-83 1.01E-82 179.5236003

ERCC6L2 -1.026904956 2.253966502 -26.11669915 1.39E-83 1.08E-82 179.4600795

TM2D1 -1.190227001 3.871341685 -26.10888576 1.49E-83 1.15E-82 179.3915859

HLA-DPB1 2.941411623 6.167537573 26.10397273 1.55E-83 1.20E-82 179.3485139

CDK10 -2.110374732 5.583556305 -26.10138374 1.59E-83 1.23E-82 179.3258155

FLYWCH2 1.36857636 4.266300099 26.09886993 1.63E-83 1.26E-82 179.3037755

SERPINH1 2.4863721 5.920122502 26.09572347 1.67E-83 1.29E-82 179.2761876

TAFA5 1.95586593 2.631204946 26.09227363 1.72E-83 1.33E-82 179.2459385

ARL4D 2.131102014 2.628743321 26.08654486 1.81E-83 1.40E-82 179.1957042

MINDY2 1.260089481 2.58154287 26.08019393 1.91E-83 1.48E-82 179.1400101

GCNT1 2.132982769 2.239284826 26.07705742 1.97E-83 1.52E-82 179.112503

RGN -2.610050599 3.47931551 -26.07469527 2.01E-83 1.55E-82 179.0917863

AL162231.1 -1.444272406 1.803277012 -26.07164921 2.06E-83 1.59E-82 179.0650706

LCK 2.368062594 2.195675855 26.06951342 2.10E-83 1.62E-82 179.0463379

CRISPLD1 1.84196174 1.434354516 26.06933544 2.11E-83 1.62E-82 179.0447768

MRM2 1.013863738 3.873010905 26.06764845 2.14E-83 1.65E-82 179.0299801

GATA2 -1.697183205 2.958939728 -26.06561752 2.18E-83 1.68E-82 179.0121662

SSPO -0.949253567 0.711288993 -26.06392486 2.21E-83 1.70E-82 178.997319

SIVA1 -1.97146964 5.303753743 -26.06379009 2.21E-83 1.70E-82 178.9961368

RRAGA 1.441623866 5.858840678 26.06274481 2.23E-83 1.72E-82 178.986968

FIGN -1.006036374 1.318650347 -26.06005026 2.28E-83 1.76E-82 178.9633317

CHPF2 1.382736634 4.955071878 26.05813251 2.32E-83 1.79E-82 178.946509

SNURF -1.784686313 1.450191192 -26.05596226 2.37E-83 1.82E-82 178.9274708

KALRN -1.774259921 2.660135409 -26.05591105 2.37E-83 1.82E-82 178.9270215

NAP1L1 -1.804886466 6.237307519 -26.05468447 2.39E-83 1.84E-82 178.9162613

SIAH1 -1.060722732 2.982308314 -26.05351019 2.42E-83 1.86E-82 178.9059597

GOLPH3 1.423094366 5.854970342 26.05272705 2.44E-83 1.87E-82 178.8990894

EWSR1 -1.672252728 6.229298492 -26.04690189 2.56E-83 1.97E-82 178.8479842

MSANTD1 -0.812657524 0.57925544 -26.04600902 2.58E-83 1.98E-82 178.8401507

PLEKHA1 -1.334230767 3.343551615 -26.04532888 2.60E-83 1.99E-82 178.8341834

ME2 -1.403192099 3.520086237 -26.04318104 2.65E-83 2.03E-82 178.8153387

KLK1 -6.113235266 7.372393522 -26.03861283 2.76E-83 2.11E-82 178.7752565

HECTD2 -1.038936208 2.007720064 -26.03634428 2.81E-83 2.15E-82 178.7553511

SLBP 1.295278485 4.860732199 26.0345528 2.86E-83 2.19E-82 178.7396313

IRS1 1.605767042 2.894116998 26.02932694 2.99E-83 2.29E-82 178.6937736

MOK -1.796106683 2.956545391 -26.02689494 3.05E-83 2.34E-82 178.6724314

ENTPD2 2.222733714 2.917915774 26.02620047 3.07E-83 2.35E-82 178.6663369

SRCIN1 -1.845178523 2.304401784 -26.02310029 3.16E-83 2.41E-82 178.6391299

SCAF1 1.321378492 4.733764087 26.01986786 3.25E-83 2.48E-82 178.6107612

ISG15 3.637685185 5.356490659 26.01713824 3.33E-83 2.54E-82 178.5868043

CINP -1.467437547 4.165456551 -26.00717402 3.63E-83 2.77E-82 178.4993451

MATN4 -1.886586806 1.4269918 -26.00690342 3.64E-83 2.78E-82 178.4969699

RPS7 -2.233771125 8.209905772 -26.00448012 3.72E-83 2.84E-82 178.475698

ADGRF4 1.99661805 1.171257174 25.99824653 3.93E-83 3.00E-82 178.4209763

TM4SF5 3.898098112 2.649942024 25.99545397 4.02E-83 3.07E-82 178.3964604

SLAMF8 2.24842891 2.504375119 25.98995615 4.22E-83 3.22E-82 178.3481924

PRSS3 -4.960979548 8.514982794 -25.98864113 4.27E-83 3.25E-82 178.3366467

CLPSL2 -2.004127503 1.260106442 -25.98729847 4.32E-83 3.29E-82 178.3248582

PCBD2 -1.066831952 2.456045008 -25.98522771 4.40E-83 3.35E-82 178.3066765

CANT1 1.602499083 4.857932479 25.98078834 4.58E-83 3.48E-82 178.2676965

SLC39A1 1.568352735 5.947984768 25.98041879 4.59E-83 3.49E-82 178.2644515

AGR2 4.919481435 5.943116832 25.97902768 4.65E-83 3.53E-82 178.2522363

STMND1 -1.636128685 1.175378042 -25.97817764 4.68E-83 3.56E-82 178.2447721

ANKZF1 -1.809465072 4.877226342 -25.9730688 4.90E-83 3.72E-82 178.1999095

GAS8 -1.289080759 3.675185182 -25.9683353 5.11E-83 3.88E-82 178.1583403

CABP1 -0.914377156 0.844980843 -25.96707389 5.16E-83 3.92E-82 178.1472623

DDX51 -1.441030462 3.770460135 -25.96547218 5.24E-83 3.97E-82 178.1331955

MFSD2A 2.307206716 1.838481342 25.96217396 5.39E-83 4.09E-82 178.1042283

PI3 4.816230917 3.494433996 25.94977934 6.01E-83 4.56E-82 177.9953599

INO80E -1.526684778 4.734272732 -25.94542081 6.24E-83 4.73E-82 177.9570726

COMMD9 -1.376923993 4.212683492 -25.94146782 6.46E-83 4.90E-82 177.922346

GPX2 4.625990316 6.08803232 25.93492418 6.85E-83 5.18E-82 177.8648569

SLC52A3 2.062287716 2.18777595 25.93384271 6.91E-83 5.23E-82 177.8553552

ZNF595 -1.292263973 2.590624226 -25.92595031 7.41E-83 5.60E-82 177.7860094

SLC22A4 1.228059084 1.152233519 25.92172579 7.69E-83 5.81E-82 177.7488883

LYZ 5.355741946 7.497260042 25.91843656 7.91E-83 5.98E-82 177.7199843

PTGS1 1.752559402 2.285564289 25.9169605 8.01E-83 6.06E-82 177.7070131

ZNF763 -1.019246129 1.675092038 -25.91452535 8.19E-83 6.19E-82 177.6856131

TMOD3 1.436029467 3.654308918 25.90854141 8.63E-83 6.52E-82 177.6330237

KANK1 -1.610949579 3.56312735 -25.90322087 9.04E-83 6.83E-82 177.5862613

RGL3 -2.567411961 4.571409037 -25.90070635 9.24E-83 6.98E-82 177.56416

MAEA -1.525018323 4.621155751 -25.89987779 9.31E-83 7.03E-82 177.5568772

WWC3 1.157263284 3.46098721 25.89589096 9.64E-83 7.27E-82 177.5218333

SZT2 -1.059743941 3.100117894 -25.89252197 9.93E-83 7.49E-82 177.4922189

KBTBD3 -1.152624454 2.115601534 -25.88870461 1.03E-82 7.74E-82 177.4586615

FBXO10 -1.345555035 2.195370043 -25.88724303 1.04E-82 7.84E-82 177.4458128

PCYOX1 1.436213179 4.226496718 25.88366712 1.07E-82 8.08E-82 177.414376

MAZ -1.643766564 5.565123886 -25.88354141 1.07E-82 8.09E-82 177.4132708

RPS6KB2 -1.407674684 4.565019813 -25.8832771 1.08E-82 8.11E-82 177.4109471

EPHA2 2.907318463 4.731745706 25.87547711 1.15E-82 8.68E-82 177.34237

SPDYE2B -0.521139355 0.268783635 -25.86303523 1.29E-82 9.68E-82 177.2329679

CBSL -4.132533742 2.121890264 -25.86223043 1.30E-82 9.74E-82 177.2258906

CERKL 1.605402245 1.613051188 25.85683325 1.36E-82 1.02E-81 177.1784271

GHITM 1.443140776 6.358328196 25.85493982 1.38E-82 1.04E-81 177.1617754

ANKS3 -1.467615315 3.479921072 -25.85388981 1.39E-82 1.05E-81 177.1525409

RMND5B -1.236880566 3.893758252 -25.85304837 1.41E-82 1.05E-81 177.1451405

TMEM18 -1.364305011 3.971820186 -25.85267464 1.41E-82 1.06E-81 177.1418536

SH3GLB2 -1.992248272 5.893532034 -25.85162956 1.42E-82 1.07E-81 177.1326622

SPDYE9P -0.196150905 0.095432982 -25.84988075 1.44E-82 1.08E-81 177.1172813

AC013394.1 -0.885373692 0.763881955 -25.84926319 1.45E-82 1.09E-81 177.1118497

FAM114A1 1.433464718 4.448841731 25.84421856 1.52E-82 1.14E-81 177.0674796

MFF -1.397689249 5.034752434 -25.83960863 1.58E-82 1.18E-81 177.0269304

PPP2R3B -1.662733557 2.734812318 -25.83876477 1.59E-82 1.19E-81 177.0195075

CTAGE8 -1.554672914 0.962594777 -25.83734548 1.61E-82 1.21E-81 177.0070228

LIMS3 -1.259316497 0.612262765 -25.83370059 1.67E-82 1.25E-81 176.9749595

RDH5 -1.456712102 1.972545202 -25.8301181 1.72E-82 1.28E-81 176.9434439

GRPR -2.141305583 1.525705599 -25.82771699 1.76E-82 1.31E-81 176.9223202

SSBP2 -1.342783573 2.854052765 -25.82437717 1.81E-82 1.35E-81 176.8929371

ZNF91 -1.155372703 2.621412372 -25.81118491 2.03E-82 1.52E-81 176.7768625

TXNDC17 -1.768965458 5.159330702 -25.80922057 2.07E-82 1.54E-81 176.7595773

PCCA -1.364742896 3.532306239 -25.80404228 2.16E-82 1.61E-81 176.7140088

GTF2H3 1.054499529 3.185567477 25.79880715 2.26E-82 1.69E-81 176.6679371

PRKCD 1.577207073 4.942765689 25.78975972 2.45E-82 1.83E-81 176.5883084

PPP1R3E -1.742080034 3.284293463 -25.78936963 2.46E-82 1.83E-81 176.5848749

ARHGAP30 2.138646884 2.676853703 25.78813688 2.49E-82 1.85E-81 176.5740244

CALCB -0.940387542 0.625126205 -25.78664776 2.52E-82 1.88E-81 176.5609171

CDH11 2.626151303 3.002248618 25.78077685 2.65E-82 1.97E-81 176.509239

TRANK1 1.307497272 2.42083741 25.77149294 2.88E-82 2.14E-81 176.4275105

RUFY2 -0.944792524 2.693139207 -25.76583624 3.02E-82 2.25E-81 176.3777086

DCAF4 -1.268051975 3.235690306 -25.76481669 3.05E-82 2.27E-81 176.3687321

SHC2 -2.760412942 5.800735383 -25.76227816 3.12E-82 2.32E-81 176.3463813

ZDHHC11 -2.336198367 2.06082388 -25.76221797 3.12E-82 2.32E-81 176.3458513

IFT27 -1.453801709 3.53506661 -25.75682137 3.27E-82 2.43E-81 176.298334

EDC4 -1.41608746 4.517646446 -25.75494059 3.33E-82 2.47E-81 176.2817729

SENP6 -1.333353012 4.29572256 -25.75015981 3.47E-82 2.58E-81 176.2396743

BMP4 2.634422701 3.302972687 25.75014035 3.47E-82 2.58E-81 176.2395029

PFDN1 1.124270106 5.20656657 25.74902628 3.51E-82 2.60E-81 176.2296922

CDT1 1.91899841 1.727439163 25.74862519 3.52E-82 2.61E-81 176.2261601

GGA2 -1.733264752 5.344635229 -25.73200966 4.07E-82 3.02E-81 176.0798249

NUF2 1.620584982 1.301328448 25.72037942 4.51E-82 3.34E-81 175.9773778

SLC38A3 -3.390130364 3.391199947 -25.71759239 4.62E-82 3.42E-81 175.9528256

PRELID1 -1.869702441 6.604059382 -25.70752939 5.05E-82 3.74E-81 175.864169

ZNF860 1.133840139 0.84955296 25.70728453 5.06E-82 3.75E-81 175.8620115

NGRN -1.320339228 4.117924446 -25.70515441 5.16E-82 3.82E-81 175.8432434

ZSCAN22 0.691276561 1.542517445 25.70514861 5.16E-82 3.82E-81 175.8431923

SAP30 1.250752052 3.441812625 25.70493636 5.17E-82 3.82E-81 175.8413222

TMEM200A 1.967669954 1.929836968 25.70258859 5.28E-82 3.90E-81 175.8206357

COL12A1 3.20197543 4.096791498 25.69830525 5.48E-82 4.05E-81 175.7828932

ATP5PO -1.735624684 6.026293328 -25.69066563 5.86E-82 4.33E-81 175.7155719

SLC35B2 1.304161997 5.554717606 25.68360054 6.24E-82 4.60E-81 175.6533079

KIAA1211 2.09962112 1.597023627 25.67995005 6.44E-82 4.75E-81 175.6211343

TMEM140 1.352214292 3.902308728 25.67233506 6.89E-82 5.08E-81 175.5540149

NPRL2 -1.476645043 4.263681853 -25.67105163 6.97E-82 5.14E-81 175.542702

BORCS8 -1.280931372 3.260487196 -25.66349962 7.45E-82 5.49E-81 175.4761305

CFAP410 -1.814894306 3.864857788 -25.66304281 7.48E-82 5.51E-81 175.4721035

HDDC3 -1.408807208 3.776544318 -25.66220859 7.53E-82 5.55E-81 175.4647493

AKAP8L -1.69034337 5.190148418 -25.65480877 8.04E-82 5.92E-81 175.3995124

MDFIC 1.790688932 3.053369988 25.65078782 8.33E-82 6.13E-81 175.3640611

RASSF2 2.250346949 2.669620816 25.65055021 8.35E-82 6.14E-81 175.3619661

ELL3 -1.370430271 1.575892107 -25.6480032 8.54E-82 6.28E-81 175.3395091

PGGHG -3.878736259 6.898719122 -25.64715792 8.60E-82 6.32E-81 175.3320561

RSPH3 0.963273703 2.368612592 25.64381909 8.86E-82 6.51E-81 175.3026161

PAK3 -2.562503941 2.722569385 -25.635206 9.55E-82 7.02E-81 175.2266653

FOXP3 1.649462527 1.134197764 25.63327942 9.72E-82 7.13E-81 175.2096754

SLC1A1 2.658154498 2.846946945 25.63191642 9.84E-82 7.22E-81 175.1976554

LUC7L3 -1.880536431 5.819151887 -25.6300294 1.00E-81 7.34E-81 175.1810138

UHRF1 1.554690188 1.308609 25.62260472 1.07E-81 7.83E-81 175.1155317

SNX4 1.150999765 4.302671931 25.61775043 1.11E-81 8.17E-81 175.0727161

GRHPR -1.499305087 5.09709636 -25.61668162 1.12E-81 8.24E-81 175.0632887

IKBKE 1.443446923 2.701320204 25.61462991 1.15E-81 8.39E-81 175.0451913

SSC5D 2.125945453 2.359768029 25.60996178 1.19E-81 8.74E-81 175.0040136

NELFB 1.266464281 5.490307848 25.60810278 1.21E-81 8.88E-81 174.9876147

SUMO3 1.347488374 5.609476679 25.60539091 1.24E-81 9.09E-81 174.9636916

RBM27 0.8526846 2.635910112 25.59476707 1.36E-81 9.98E-81 174.8699645

NOL8 -1.155996498 3.711950121 -25.59057491 1.42E-81 1.04E-80 174.8329765

GPT2 -2.962030274 4.871539957 -25.58863057 1.44E-81 1.05E-80 174.8158207

TOMM40L 1.035039085 2.823331696 25.5839354 1.50E-81 1.10E-80 174.7743913

C8orf34 -0.519576776 0.417924402 -25.57834407 1.58E-81 1.15E-80 174.7250513

TMEM159 1.735148145 4.307102111 25.57757529 1.59E-81 1.16E-80 174.718267

NOP53 -1.927415676 7.964151374 -25.57288441 1.65E-81 1.21E-80 174.6768699

NUMA1 -1.75247904 5.890591154 -25.57141014 1.68E-81 1.22E-80 174.663859

SFSWAP -1.243495078 3.93133164 -25.56945658 1.71E-81 1.24E-80 174.6466177

SLC5A1 2.953459054 2.662062929 25.56708601 1.74E-81 1.27E-80 174.6256957

CLRN3 3.336516074 2.050462488 25.5639615 1.79E-81 1.30E-80 174.5981186

KIF18B 1.63601875 1.176927755 25.55991561 1.86E-81 1.35E-80 174.5624078

HPGD 2.893599077 2.441704184 25.55902494 1.87E-81 1.36E-80 174.554546

SLC4A11 2.417330158 1.931897585 25.55835507 1.88E-81 1.37E-80 174.5486332

PALLD 2.226460338 4.789045677 25.55669087 1.91E-81 1.39E-80 174.5339435

PAXBP1 -1.256672742 3.699514407 -25.5380903 2.25E-81 1.64E-80 174.3697374

TVP23B -1.43932548 4.041654172 -25.53459407 2.32E-81 1.69E-80 174.3388685

FAXC -1.21917816 1.229800606 -25.52912566 2.43E-81 1.77E-80 174.2905841

CRBN -1.12402167 3.756285266 -25.52589777 2.50E-81 1.82E-80 174.2620813

WDCP 0.76045298 2.06196187 25.52167593 2.60E-81 1.89E-80 174.2248002

RAB42 1.58503313 1.386738823 25.51888514 2.66E-81 1.93E-80 174.2001549

EFNA4 2.09278999 3.549159215 25.50869172 2.92E-81 2.12E-80 174.1101305

SAE1 1.234101991 5.123899857 25.50745062 2.95E-81 2.14E-80 174.0991688

VBP1 1.141000469 4.533032577 25.50682015 2.96E-81 2.15E-80 174.0936003

NUDT7 -1.330169982 2.37300543 -25.50563877 3.00E-81 2.17E-80 174.0831658

CA4 -3.066954749 2.92742627 -25.50275552 3.07E-81 2.23E-80 174.0576991

ITGAV 1.938575105 4.866970439 25.50119313 3.11E-81 2.26E-80 174.0438986

CUL9 -1.743328344 3.640830779 -25.50099077 3.12E-81 2.26E-80 174.0421112

SLC10A3 1.314353115 4.800801637 25.48697104 3.53E-81 2.56E-80 173.9182645

RAC1 1.676145997 7.262206959 25.48555992 3.58E-81 2.59E-80 173.9057978

TFE3 1.102791769 4.651868809 25.48280093 3.66E-81 2.65E-80 173.8814227

GATAD2B 0.852440463 3.173399035 25.48245516 3.68E-81 2.66E-80 173.8783678

ARHGEF28 -1.641072608 3.448885234 -25.48012282 3.75E-81 2.71E-80 173.8577613

TSPAN9 1.478665472 4.201653702 25.47718699 3.85E-81 2.78E-80 173.8318221

TECPR1 -2.210054826 4.755475694 -25.47491474 3.93E-81 2.84E-80 173.8117452

PRF1 1.778885745 1.808160312 25.47433283 3.95E-81 2.85E-80 173.8066037

TMEM171 2.265999444 1.791733642 25.47261959 4.01E-81 2.89E-80 173.7914656

PPP1R14D 3.065984425 1.97404656 25.46813257 4.17E-81 3.01E-80 173.751817

PBX1 -1.700619317 3.901049549 -25.46805173 4.17E-81 3.01E-80 173.7511026

EFR3A 1.351511584 4.233670267 25.46360237 4.34E-81 3.13E-80 173.7117847

ALYREF 1.405353625 5.300709224 25.45832395 4.55E-81 3.28E-80 173.6651378

FAM241A 1.00558275 1.645514766 25.45426166 4.71E-81 3.40E-80 173.6292362

ELMOD3 -1.134661667 3.234114339 -25.44931615 4.92E-81 3.55E-80 173.5855265

ZRANB3 -0.862480832 1.335060034 -25.44424927 5.15E-81 3.71E-80 173.5407413

TRHDE -2.033957876 1.59381933 -25.44180335 5.26E-81 3.79E-80 173.5191213

STON2 1.260031191 1.321462363 25.44146947 5.28E-81 3.80E-80 173.51617

RPL27A -2.380055702 8.858877034 -25.4400356 5.35E-81 3.84E-80 173.5034954

DGKZ -1.495344926 4.742223401 -25.43772455 5.46E-81 3.92E-80 173.4830666

ATP5F1A -1.881443996 6.756773808 -25.43579183 5.55E-81 3.99E-80 173.4659816

TFF1 7.785029775 5.411540853 25.42814046 5.94E-81 4.26E-80 173.3983405

TCN1 5.297564129 4.454154172 25.4244606 6.13E-81 4.40E-80 173.3658069

CIAO3 -1.379709679 3.728755738 -25.41734147 6.53E-81 4.69E-80 173.3028627

AIFM2 1.107730935 2.922572745 25.41656412 6.58E-81 4.72E-80 173.2959894

TCF20 1.051692399 3.413002824 25.41237904 6.82E-81 4.89E-80 173.2589838

TNFSF11 2.103715644 1.313786606 25.40987239 6.98E-81 5.00E-80 173.2368185

PITX1 3.234242899 2.057580877 25.40753328 7.12E-81 5.10E-80 173.216134

ARMC7 1.118428849 3.303642555 25.40129897 7.53E-81 5.39E-80 173.161002

SCT 2.530382934 1.649263407 25.39593748 7.89E-81 5.65E-80 173.1135853

EIF6 1.559131146 6.702687568 25.39326196 8.08E-81 5.78E-80 173.0899221

PLAGL1 -2.134551758 3.528471681 -25.38866156 8.42E-81 6.02E-80 173.0492327

RAI14 1.521969134 3.60580447 25.38258163 8.88E-81 6.35E-80 172.9954537

EP400 -1.09567892 3.341690806 -25.38158101 8.96E-81 6.40E-80 172.9866025

GPATCH2L -0.944343003 2.461496732 -25.38157578 8.96E-81 6.40E-80 172.9865562

CRK 1.153497379 4.540818792 25.3806777 9.03E-81 6.45E-80 172.978612

VAV1 2.020901226 2.170463027 25.37972914 9.11E-81 6.51E-80 172.9702212

ATP5F1E 1.57964569 5.86624512 25.37108311 9.83E-81 7.02E-80 172.8937348

ZNF267 1.05543388 2.371084175 25.37077708 9.86E-81 7.04E-80 172.8910274

MCCC1 -1.609000605 4.407017439 -25.37052932 9.88E-81 7.05E-80 172.8888355

BRK1 1.557480122 7.196133994 25.36050892 1.08E-80 7.70E-80 172.8001804

EMP3 2.208042033 4.789719091 25.36003645 1.08E-80 7.73E-80 172.7959999

CPPED1 1.265242846 2.490234813 25.35834113 1.10E-80 7.84E-80 172.7809996

IFT122 -1.279489935 3.630355152 -25.35725109 1.11E-80 7.91E-80 172.7713545

MXD3 -1.796354037 2.87439093 -25.35330688 1.15E-80 8.19E-80 172.7364541

GCFC2 -1.140919536 3.309841463 -25.3527876 1.16E-80 8.23E-80 172.7318591

AMMECR1L 0.893087748 3.624303871 25.35070875 1.18E-80 8.38E-80 172.7134635

AMY2A -8.827673642 7.590105264 -25.35041915 1.18E-80 8.40E-80 172.7109008

GAK -1.810871356 5.088619373 -25.33907765 1.30E-80 9.28E-80 172.6105323

GJC3 -2.192132768 1.908612506 -25.33847043 1.31E-80 9.32E-80 172.6051581

RUNX3 2.11682112 1.889104397 25.32993505 1.41E-80 1.01E-79 172.5296133

RIN2 1.390850754 4.261318685 25.32899314 1.43E-80 1.01E-79 172.5212762

LIN7C 0.949430444 3.504094408 25.32386028 1.49E-80 1.06E-79 172.4758421

KIF1BP 1.094682213 3.770170385 25.32096178 1.53E-80 1.09E-79 172.4501845

FGF17 -1.262927777 0.960620432 -25.31881252 1.56E-80 1.11E-79 172.4311586

PYROXD1 -1.294635063 3.109459389 -25.31715665 1.58E-80 1.12E-79 172.4165

DBN1 1.687700156 4.736427826 25.31346168 1.64E-80 1.16E-79 172.3837892

TMEM63B 1.232245951 4.189131388 25.31324171 1.64E-80 1.16E-79 172.3818418

SIRT3 -1.187391424 3.803127863 -25.31164979 1.66E-80 1.18E-79 172.3677484

PLAC8 2.933953569 2.789931101 25.31126803 1.67E-80 1.18E-79 172.3643686

SESN3 1.699961027 2.700759961 25.30950335 1.69E-80 1.20E-79 172.3487453

MED27 1.067561922 3.514327704 25.30936777 1.70E-80 1.20E-79 172.347545

CACNB2 -1.719727527 1.996932495 -25.30915583 1.70E-80 1.20E-79 172.3456686

CDKN3 1.786882298 1.928561297 25.30577289 1.75E-80 1.24E-79 172.3157173

ABHD17A -1.646122135 4.395771294 -25.30407587 1.78E-80 1.26E-79 172.3006921

TENT5A 1.686232461 3.036103177 25.30298639 1.80E-80 1.27E-79 172.2910458

DEPDC4 -0.804139364 0.78444158 -25.30022556 1.84E-80 1.30E-79 172.2666008

MAPK8IP3 -2.078517127 4.982544888 -25.29438019 1.94E-80 1.37E-79 172.2148418

NARF -1.628938243 4.362218104 -25.2927649 1.97E-80 1.39E-79 172.2005383

CYBRD1 2.531159664 4.731814836 25.29276485 1.97E-80 1.39E-79 172.2005378

MAP7D1 1.442467985 5.016353763 25.27356144 2.33E-80 1.64E-79 172.0304687

TBC1D24 -1.185595488 2.763805942 -25.270913 2.38E-80 1.68E-79 172.0070105

ST6GALNAC1 3.447049704 2.848296743 25.26962884 2.41E-80 1.70E-79 171.995636

RSAD1 -1.515795616 4.86233291 -25.26698361 2.47E-80 1.74E-79 171.9722052

EHD1 1.452154072 4.251399896 25.26527367 2.51E-80 1.77E-79 171.9570585

ZGLP1 -1.441054596 1.69805859 -25.26205948 2.58E-80 1.82E-79 171.9285865

LCA5L -0.877256212 1.088676197 -25.25970106 2.63E-80 1.85E-79 171.9076943

ING3 -1.113503503 2.821267775 -25.2587125 2.66E-80 1.87E-79 171.8989369

EHD2 1.812726159 5.392740029 25.25236559 2.81E-80 1.98E-79 171.8427089

TRA2B -1.365541072 4.933974305 -25.25184135 2.82E-80 1.99E-79 171.8380644

MOXD1 2.596483469 2.782331057 25.25172194 2.83E-80 1.99E-79 171.8370065

ALMS1 -1.263912569 2.657024374 -25.25133663 2.84E-80 1.99E-79 171.8335929

PLEKHA6 1.794113888 4.00327504 25.25021937 2.86E-80 2.01E-79 171.8236943

CLN3 -1.440091566 4.127072881 -25.25010143 2.87E-80 2.01E-79 171.8226494

ACADVL -2.35026512 8.116377938 -25.2474418 2.93E-80 2.06E-79 171.7990856

C1GALT1 1.472532195 3.213780755 25.24330978 3.04E-80 2.14E-79 171.7624752

OCLM -1.051784407 1.097871114 -25.2408644 3.11E-80 2.18E-79 171.7408078

FAM200A 0.928850598 2.435896636 25.23994986 3.14E-80 2.20E-79 171.7327043

MFSD6 1.311726032 3.972437528 25.23293873 3.34E-80 2.34E-79 171.6705778

SYTL3 1.643316981 1.911056905 25.22972895 3.43E-80 2.41E-79 171.6421338

NUDCD2 -1.033003782 3.162437307 -25.2275853 3.50E-80 2.45E-79 171.6231369

PECAM1 1.896353698 4.78355428 25.22660529 3.53E-80 2.47E-79 171.614452

SLC25A53 -1.160611567 1.576180022 -25.22091782 3.71E-80 2.60E-79 171.564047

ATG16L2 -1.740319782 3.57602719 -25.21910139 3.77E-80 2.64E-79 171.5479483

CD82 1.800786661 5.370797727 25.21827558 3.80E-80 2.66E-79 171.5406291

FSCN2 -1.697136069 1.890147719 -25.21756517 3.82E-80 2.67E-79 171.5343327

CEP19 0.970440611 1.239755761 25.21602823 3.88E-80 2.71E-79 171.5207105

SRFBP1 1.049307879 2.348906571 25.21019107 4.08E-80 2.85E-79 171.4689723

PLS1 2.248358591 3.903301935 25.20851775 4.14E-80 2.89E-79 171.45414

CDHR2 3.349756024 2.192072148 25.2084928 4.14E-80 2.89E-79 171.4539189

CCNL2 -2.280615154 6.301744863 -25.20789828 4.17E-80 2.91E-79 171.448649

FHDC1 1.579937403 1.846047208 25.19529076 4.66E-80 3.25E-79 171.336886

SNX14 -1.321477505 4.713372382 -25.19452434 4.69E-80 3.27E-79 171.3300913

NEURL2 -1.017537142 1.615775869 -25.19227061 4.78E-80 3.34E-79 171.3101105

LACC1 1.01093172 2.281697866 25.18498601 5.10E-80 3.56E-79 171.2455241

AC005833.1 -1.436399841 0.733311807 -25.16649532 6.01E-80 4.19E-79 171.0815578

NPAP1 -0.503400257 0.262812433 -25.16301568 6.20E-80 4.32E-79 171.0506981

AREG 3.410463832 3.15219942 25.15766606 6.50E-80 4.53E-79 171.0032516

RTL6 1.249811876 3.427218281 25.15760418 6.50E-80 4.53E-79 171.0027028

ATP6V1E2 -1.10350854 1.926375491 -25.14756859 7.11E-80 4.95E-79 170.9136877

SP1 1.224416405 4.480511534 25.1449714 7.27E-80 5.06E-79 170.8906491

TFCP2 1.045287505 3.987594668 25.14142275 7.51E-80 5.22E-79 170.8591693

NPEPPS -1.428396275 4.786540248 -25.14123889 7.52E-80 5.23E-79 170.8575383

PLXNC1 1.756775869 2.158826111 25.14029141 7.58E-80 5.27E-79 170.849133

ITGB3BP -1.394520478 2.897306301 -25.13894932 7.67E-80 5.33E-79 170.8372269

NCAPG 1.545152228 1.243968802 25.13589918 7.88E-80 5.47E-79 170.8101675

IL2RB 1.794218397 1.556911027 25.12766297 8.48E-80 5.89E-79 170.7370949

CD4 2.213536589 3.654563812 25.12658088 8.56E-80 5.94E-79 170.727494

FXYD6-FXYD2 -0.954511653 0.463382103 -25.12431719 8.74E-80 6.06E-79 170.7074088

AQP8 -5.791628444 5.003750697 -25.1146608 9.52E-80 6.60E-79 170.6217238

RAB1B 1.414644057 6.878425047 25.11096969 9.83E-80 6.82E-79 170.5889685

ECI2 -2.099601994 5.226224576 -25.09704922 1.11E-79 7.71E-79 170.4654242

LTB4R2 -1.137387793 1.634176542 -25.09682275 1.11E-79 7.72E-79 170.4634141

NDUFA5 -1.404144882 4.346452527 -25.09392642 1.14E-79 7.92E-79 170.4377065

DIO2 2.077999388 1.797574016 25.09297792 1.15E-79 7.98E-79 170.4292874

LAMC1 1.782094837 5.129450481 25.07857651 1.31E-79 9.07E-79 170.3014473

DGCR8 -1.2434648 3.923311786 -25.07716392 1.33E-79 9.18E-79 170.2889067

ATAD3B -1.803160977 3.634811919 -25.0750236 1.35E-79 9.35E-79 170.2699051

NDUFB2 -1.713536014 5.939450381 -25.06578621 1.47E-79 1.01E-78 170.1878908

CCL22 2.369659175 1.389437133 25.06566516 1.47E-79 1.02E-78 170.1868159

HFM1 -0.882188114 0.626231572 -25.05570153 1.61E-79 1.11E-78 170.0983436

CRELD2 -2.218209511 5.289927915 -25.04924508 1.70E-79 1.17E-78 170.0410078

NFAM1 1.68891113 1.683470838 25.0483035 1.71E-79 1.18E-78 170.0326458

TBC1D7 -1.15924686 2.542175746 -25.0452091 1.76E-79 1.22E-78 170.0051645

TOM1L1 -1.647250748 4.007504974 -25.04510019 1.76E-79 1.22E-78 170.0041973

RAD21 1.376073713 5.124568828 25.04469697 1.77E-79 1.22E-78 170.0006162

ABCC1 1.260375501 3.884206348 25.04180557 1.82E-79 1.25E-78 169.9749367

ZNF658 -0.734318835 1.087089455 -25.03792658 1.88E-79 1.30E-78 169.9404847

CNOT2 -1.177628994 3.891531631 -25.03765132 1.88E-79 1.30E-78 169.9380399

PDCD6 -1.447966334 5.049023952 -25.03526147 1.92E-79 1.33E-78 169.916813

SCARA3 2.14848798 3.057012499 25.03422123 1.94E-79 1.34E-78 169.9075734

LUM 3.812859727 7.78187572 25.03391296 1.95E-79 1.34E-78 169.9048353

NPC1L1 2.870210831 2.16480264 25.03295905 1.96E-79 1.35E-78 169.8963623

ST8SIA4 1.486314981 1.379914342 25.0292894 2.03E-79 1.40E-78 169.8637662

RAB33A 1.312768996 0.992353827 25.0280927 2.05E-79 1.41E-78 169.8531361

CHMP5 1.299510275 5.412254103 25.02320742 2.14E-79 1.47E-78 169.8097393

SLC9A1 1.692425538 4.308995911 25.02064908 2.19E-79 1.51E-78 169.7870122

TRIL 1.378101832 1.322502611 25.01420226 2.32E-79 1.59E-78 169.7297384

TIMM23B -1.044766932 2.742406359 -25.00542545 2.51E-79 1.72E-78 169.6517581

C3orf80 1.497332138 1.134272738 25.00377466 2.55E-79 1.75E-78 169.6370902

BICDL2 -2.913989343 5.472294742 -25.00036505 2.62E-79 1.80E-78 169.6067937

CD300LF 2.044609922 1.674469666 24.99904297 2.65E-79 1.82E-78 169.5950459

ZNF33B -1.706729365 4.165463937 -24.98651631 2.97E-79 2.03E-78 169.4837267

MGST3 -1.634336983 5.461377738 -24.98421024 3.03E-79 2.07E-78 169.4632318

FAF1 -1.362419813 4.371111887 -24.9830858 3.06E-79 2.10E-78 169.4532384

GINS1 1.409521202 1.267542421 24.98104244 3.11E-79 2.13E-78 169.4350776

SPAG4 -2.443872865 4.476982261 -24.97798278 3.20E-79 2.19E-78 169.4078835

TYW5 -0.853253414 1.927641298 -24.97752588 3.21E-79 2.20E-78 169.4038225

MTRF1 -1.303721392 3.124704336 -24.9754194 3.27E-79 2.24E-78 169.3850995

SNTB2 0.990183476 2.670379388 24.97372517 3.32E-79 2.27E-78 169.3700404

DDX28 1.119085877 3.004209954 24.96357141 3.64E-79 2.49E-78 169.2797829

RNASE6 2.64715661 3.615043179 24.96288554 3.66E-79 2.50E-78 169.2736857

PARS2 0.910171195 2.043917362 24.96146172 3.71E-79 2.53E-78 169.2610283

MRPL50 1.035118556 3.577532791 24.9608899 3.73E-79 2.54E-78 169.2559449

BTNL9 -2.201137983 3.009377445 -24.96056358 3.74E-79 2.55E-78 169.253044

ETV1 1.6361893 2.10667882 24.95652781 3.87E-79 2.64E-78 169.2171655

KIF7 1.243703185 1.652212951 24.95427694 3.95E-79 2.69E-78 169.1971543

PRUNE1 1.032782774 3.823974933 24.95316703 3.99E-79 2.72E-78 169.1872864

HSD17B3 -1.47135294 1.446600957 -24.95174387 4.04E-79 2.75E-78 169.1746335

PLA2G12A -1.665626087 4.489847679 -24.94706026 4.21E-79 2.87E-78 169.1329912

LRRC32 2.028640977 4.782762287 24.94562417 4.27E-79 2.91E-78 169.1202224

SPOCK2 2.562312517 2.644336306 24.9415847 4.42E-79 3.01E-78 169.0843047

KRCC1 1.278185774 4.821761965 24.93910528 4.52E-79 3.08E-78 169.0622577

PLEK2 3.037712005 4.318154828 24.93500156 4.69E-79 3.19E-78 169.0257661

ZNF862 -1.307218568 3.046584764 -24.93144165 4.84E-79 3.29E-78 168.9941087

CKAP4 1.531161388 5.602497754 24.9308909 4.86E-79 3.31E-78 168.989211

OXCT2 -0.688417416 0.682558036 -24.92899722 4.95E-79 3.36E-78 168.9723703

IFRD2 -1.696121954 5.573059559 -24.92872518 4.96E-79 3.37E-78 168.969951

ADAM7 -0.257571936 0.133943207 -24.92793137 4.99E-79 3.39E-78 168.9628915

VSTM2L 3.686461427 3.370565671 24.9275781 5.01E-79 3.40E-78 168.9597498

RGMB -1.092032075 2.494477794 -24.925842 5.09E-79 3.45E-78 168.9443099

DIAPH2 1.041912686 2.458439828 24.91958386 5.38E-79 3.65E-78 168.8886511

EMSY -0.949969707 2.452087682 -24.91858325 5.43E-79 3.68E-78 168.8797515

TYK2 -1.660586682 5.563024984 -24.91775328 5.47E-79 3.71E-78 168.8723695

MCEE -1.3174058 4.074565297 -24.91732208 5.49E-79 3.72E-78 168.8685342

TSPAN5 1.558820227 1.942512596 24.91605432 5.55E-79 3.76E-78 168.8572583

NMU 3.311864493 1.889731444 24.91603554 5.55E-79 3.76E-78 168.8570912

PTPRH 2.422153798 3.663667635 24.91444025 5.63E-79 3.81E-78 168.8429018

KIF27 -1.137511212 1.739569426 -24.9106769 5.82E-79 3.94E-78 168.8094273

GALNT10 1.430238203 3.822987087 24.91008798 5.85E-79 3.96E-78 168.8041888

NINJ2 1.756118759 2.057834369 24.90621445 6.06E-79 4.10E-78 168.7697325

MPHOSPH9 -1.100968919 2.050724824 -24.89934257 6.44E-79 4.35E-78 168.7086012

TMEM14B -1.444202192 4.734503643 -24.8943891 6.73E-79 4.55E-78 168.6645327

SUB1 -1.489384287 5.431781217 -24.8867482 7.20E-79 4.86E-78 168.5965507

PPIL2 -1.360375165 4.409803955 -24.87741757 7.82E-79 5.28E-78 168.5135267

RARA 1.358399306 4.154188417 24.87550724 7.96E-79 5.37E-78 168.4965275

TM9SF3 1.573014339 5.922217885 24.87429859 8.04E-79 5.43E-78 168.485772

SMG6 -1.232688 2.939515492 -24.86899232 8.43E-79 5.69E-78 168.4385511

KLF4 2.138833711 4.010460603 24.86274313 8.91E-79 6.01E-78 168.3829355

DNAH12 -0.749803232 0.599529594 -24.8577851 9.32E-79 6.28E-78 168.3388079

PARP14 1.484957742 3.832196946 24.85601752 9.46E-79 6.38E-78 168.3230754

RMI2 1.73772983 2.035603979 24.84723037 1.02E-78 6.89E-78 168.2448599

KLK6 4.668681942 2.552847148 24.84643457 1.03E-78 6.94E-78 168.2377759

SERINC5 1.41507898 3.357174357 24.84335315 1.06E-78 7.13E-78 168.2103457

ITGA11 2.630501695 2.262985698 24.84214553 1.07E-78 7.21E-78 168.1995955

STAT1 1.937378383 5.125220861 24.8414085 1.08E-78 7.25E-78 168.1930343

SETD4 -1.067037227 2.983907427 -24.8370646 1.12E-78 7.53E-78 168.1543633

CSKMT -1.355963509 2.284895015 -24.83087747 1.18E-78 7.96E-78 168.0992796

PRDM11 -0.789640641 1.827025876 -24.8224895 1.28E-78 8.57E-78 168.0245959

TKT -2.036187761 6.656768705 -24.82023129 1.30E-78 8.74E-78 168.0044883

RENBP 1.855243233 2.693908269 24.81850063 1.32E-78 8.87E-78 167.9890777

HEXIM2 -1.240868363 2.48802361 -24.81848824 1.32E-78 8.87E-78 167.9889675

H2AFX 1.697829147 4.454915043 24.81757145 1.33E-78 8.94E-78 167.9808038

FGF11 -0.718172433 0.57183494 -24.81385332 1.38E-78 9.24E-78 167.9476946

HOXB6 2.340796522 2.328061865 24.81362567 1.38E-78 9.26E-78 167.9456674

DIP2B 1.139991094 3.158286894 24.81209409 1.40E-78 9.38E-78 167.9320286

ATE1 1.12122843 3.059437589 24.81124328 1.41E-78 9.45E-78 167.9244519

SNX7 1.279289408 4.169049775 24.81096084 1.41E-78 9.47E-78 167.9219367

AFAP1L1 1.292484191 1.903795838 24.8108214 1.41E-78 9.48E-78 167.920695

TMEM191C -0.709096346 0.542903969 -24.81064147 1.42E-78 9.49E-78 167.9190926

TAP1 1.889975195 4.848517982 24.81026428 1.42E-78 9.52E-78 167.9157336

AK4 2.16759992 2.033486484 24.80643525 1.47E-78 9.84E-78 167.881634

TRDMT1 -0.822887665 1.253054572 -24.80459334 1.50E-78 1.00E-77 167.8652302

COPS5 -1.27810805 4.483717309 -24.7951619 1.63E-78 1.09E-77 167.7812299

JOSD1 1.245716788 4.898556158 24.77943521 1.87E-78 1.25E-77 167.6411414

DRC3 -1.212585727 1.845007109 -24.77930474 1.87E-78 1.25E-77 167.639979

ZBTB25 -0.840385757 2.23415116 -24.77700305 1.91E-78 1.28E-77 167.6194741

AC091167.2 -0.319445187 0.27472319 -24.77631988 1.92E-78 1.28E-77 167.6133879

BTN2A1 1.062244374 3.915967031 24.77386576 1.97E-78 1.31E-77 167.5915243

TSHZ3 1.47938256 1.992851081 24.77325565 1.98E-78 1.32E-77 167.5860888

RPS6KL1 -1.685414145 2.40285439 -24.7699608 2.04E-78 1.36E-77 167.556734

PRDM15 -0.78761033 1.644091655 -24.7692929 2.05E-78 1.37E-77 167.5507834

METTL18 1.028058028 2.818113724 24.76842655 2.06E-78 1.38E-77 167.5430645

NHS 1.44981713 1.367163674 24.76776417 2.08E-78 1.38E-77 167.537163

EVI2B 2.474306791 2.82155097 24.75268789 2.37E-78 1.58E-77 167.4028264

GJA5 1.765963193 2.186000138 24.75058904 2.42E-78 1.61E-77 167.3841229

SAP130 0.884324886 3.409077946 24.74700889 2.50E-78 1.66E-77 167.3522181

LY6G5C -1.187012503 2.061530907 -24.74653776 2.51E-78 1.67E-77 167.3480194

BMP2K -1.132693412 2.006197727 -24.74432462 2.56E-78 1.70E-77 167.328296

ATF7-NPFF -1.341973528 1.999430364 -24.74289135 2.59E-78 1.72E-77 167.3155225

UCP2 2.199491292 4.468357949 24.74161681 2.62E-78 1.74E-77 167.3041635

ZBTB8OS -1.288703118 4.142822098 -24.74109235 2.63E-78 1.75E-77 167.2994892

C4B -3.427133525 4.117888702 -24.74026774 2.65E-78 1.76E-77 167.2921399

ZBTB22 0.953904565 3.841273268 24.73888418 2.68E-78 1.78E-77 167.2798089

NCAPD2 1.34030189 3.752968192 24.73506352 2.78E-78 1.84E-77 167.2457561

NRN1L -0.873515827 0.460747305 -24.73044113 2.89E-78 1.92E-77 167.2045556

IP6K2 -1.576266956 5.422641337 -24.72743539 2.97E-78 1.97E-77 167.1777635

PSMB3 1.59509828 6.763028027 24.72503353 3.04E-78 2.01E-77 167.1563536

PEF1 1.178564319 5.57837273 24.72329609 3.08E-78 2.04E-77 167.1408658

CALHM5 1.20839289 0.917081227 24.71691828 3.26E-78 2.16E-77 167.0840106

FPR3 2.572164426 2.472388778 24.71681434 3.27E-78 2.16E-77 167.083084

MEGF9 1.235692966 2.707851174 24.71375626 3.36E-78 2.22E-77 167.0558212

SPATA13 -1.42300142 4.023978618 -24.71184208 3.41E-78 2.26E-77 167.0387557

SLC11A2 -1.292147521 4.242035232 -24.71064402 3.45E-78 2.28E-77 167.0280746

CILP2 2.290389781 1.377213812 24.70546862 3.61E-78 2.39E-77 166.981932

NDUFB8 -1.589390923 5.723880391 -24.70207479 3.73E-78 2.46E-77 166.951672

ZC3HAV1 0.956911957 3.238960336 24.69685582 3.90E-78 2.58E-77 166.9051364

ZNF597 0.763713582 1.414591588 24.69582737 3.94E-78 2.60E-77 166.8959658

RPH3AL -2.173670148 4.11545268 -24.68396624 4.38E-78 2.89E-77 166.7901931

ATAD1 1.025700457 4.027705229 24.68300108 4.42E-78 2.92E-77 166.7815856

CTNND2 -2.366256986 2.71653055 -24.67495469 4.74E-78 3.13E-77 166.7098223

CEP57L1 -0.911164419 1.956080854 -24.67163083 4.89E-78 3.23E-77 166.6801759

DUSP7 1.332356758 3.317686772 24.66949335 4.98E-78 3.29E-77 166.6611106

ANAPC2 -1.48273851 4.814696064 -24.66759314 5.07E-78 3.34E-77 166.6441612

TMEM127 1.158200892 5.042730994 24.66586155 5.14E-78 3.39E-77 166.6287155

SSBP1 -1.49965496 5.281608992 -24.66576299 5.15E-78 3.39E-77 166.6278364

MYT1L -1.14645972 0.758530469 -24.66511894 5.18E-78 3.41E-77 166.6220914

METTL21A -1.012799739 2.556496531 -24.65586044 5.62E-78 3.71E-77 166.5395004

SLC35E2B -1.503897261 4.007730496 -24.65290939 5.77E-78 3.80E-77 166.5131736

C16orf58 -1.543102483 5.407987277 -24.65189811 5.83E-78 3.84E-77 166.5041516

HHIPL1 1.145414624 0.945224008 24.65157759 5.84E-78 3.85E-77 166.5012921

C1orf116 2.05785668 4.250971864 24.65062394 5.89E-78 3.88E-77 166.4927841

GATM -4.006930724 7.095213511 -24.65013413 5.92E-78 3.89E-77 166.4884142

GTSE1 1.507142571 1.264766099 24.64772371 6.05E-78 3.98E-77 166.4669091

EPHX4 1.71277108 1.021035072 24.64405465 6.25E-78 4.11E-77 166.4341737

UBASH3B 1.578430822 1.557113462 24.64284686 6.32E-78 4.15E-77 166.4233975

HERPUD1 -2.132144509 6.965975424 -24.61976779 7.76E-78 5.10E-77 166.2174513

ASPM 1.376943054 0.970549323 24.61944433 7.78E-78 5.11E-77 166.2145645

SFR1 1.305433243 2.682126576 24.61897209 7.81E-78 5.13E-77 166.21035

PIMREG 1.393095123 0.987365749 24.61650103 7.99E-78 5.24E-77 166.1882961

GKAP1 -1.465644661 2.470277705 -24.60762897 8.65E-78 5.67E-77 166.1091091

SPC24 1.722286407 1.506349738 24.60737202 8.67E-78 5.68E-77 166.1068156

ODF2L -1.287733248 3.090892871 -24.60724399 8.68E-78 5.69E-77 166.1056729

MASTL 1.010272132 2.35900831 24.60502828 8.85E-78 5.80E-77 166.0858953

CLEC14A 1.605757848 3.664428511 24.60280674 9.03E-78 5.91E-77 166.0660653

ZNF692 -2.139942692 4.726665822 -24.58632147 1.05E-77 6.85E-77 165.9188982

HEXIM1 1.211221622 4.520773504 24.58218546 1.08E-77 7.10E-77 165.8819709

VPS26A 1.155906586 4.627371007 24.57987024 1.11E-77 7.25E-77 165.8612993

NAGS 1.671812474 1.607635175 24.57739876 1.13E-77 7.41E-77 165.839232

BRD7 -1.178519804 4.494227519 -24.57176486 1.19E-77 7.79E-77 165.7889259

TLCD3A 1.42566937 3.696804924 24.56755084 1.24E-77 8.08E-77 165.7512959

HAVCR2 2.018241616 2.344429726 24.56586074 1.26E-77 8.20E-77 165.7362034

COL4A3 -1.56897594 1.46111854 -24.5658137 1.26E-77 8.20E-77 165.7357833

ARL8B 1.154008855 5.136321524 24.56484678 1.27E-77 8.27E-77 165.7271486

NRDC -0.722527766 5.567114934 -24.5619539 1.30E-77 8.49E-77 165.7013143

ABTB2 1.700853881 2.382387985 24.56043272 1.32E-77 8.60E-77 165.6877293

BLACAT1 2.363110888 1.491584786 24.55930666 1.33E-77 8.68E-77 165.6776729

HTR1D 2.053166772 1.186444504 24.55840679 1.34E-77 8.75E-77 165.6696363

KIF20B 1.158854393 1.346197184 24.55406951 1.39E-77 9.09E-77 165.6308999

MND1 1.454767692 1.183912829 24.54890791 1.46E-77 9.52E-77 165.584799

SYT6 -1.47500635 1.024875208 -24.54321238 1.54E-77 1.00E-76 165.5339263

ADGRA2 1.981666693 3.414433733 24.53934126 1.59E-77 1.04E-76 165.4993474

BUB1B 1.534717396 1.456378936 24.53849988 1.60E-77 1.04E-76 165.4918316

ACTG1 2.563980282 10.96525407 24.53417955 1.67E-77 1.08E-76 165.453238

PPTC7 1.166656771 3.475204246 24.53368019 1.67E-77 1.09E-76 165.4487771

DPEP1 -3.541013047 5.826521242 -24.53328507 1.68E-77 1.09E-76 165.4452473

SLC38A5 -3.659806771 5.928055618 -24.52895982 1.74E-77 1.13E-76 165.4066075

EXOC3 -1.293221141 4.756043009 -24.52861361 1.75E-77 1.14E-76 165.4035146

ARPP19 1.193788668 4.827135147 24.525067 1.81E-77 1.17E-76 165.3718293

EMILIN1 2.630372493 5.151612864 24.51637213 1.95E-77 1.27E-76 165.2941443

WDFY1 1.268714575 4.030443977 24.51037999 2.06E-77 1.34E-76 165.2406026

POGK 0.998805905 3.814665668 24.50972272 2.07E-77 1.35E-76 165.2347295

GALK2 -1.241113061 3.083026018 -24.50675889 2.13E-77 1.38E-76 165.2082452

IGSF3 1.700345447 2.425790356 24.50060693 2.25E-77 1.46E-76 165.1532696

ZNF662 -1.468244748 2.659698109 -24.49895054 2.28E-77 1.48E-76 165.138467

ANKRD18A -1.484523928 1.53713766 -24.497963 2.30E-77 1.49E-76 165.1296416

FAM102B 1.292193309 2.487448289 24.49702539 2.32E-77 1.50E-76 165.1212623

TNFAIP8L2 1.932698802 2.120172383 24.49426351 2.38E-77 1.54E-76 165.0965792

ZNF385C -1.155839904 1.452701606 -24.49129417 2.44E-77 1.58E-76 165.0700411

DDAH2 1.71977842 5.182889643 24.48975688 2.48E-77 1.60E-76 165.0563015

CERS6 1.419373395 2.894326469 24.48891608 2.49E-77 1.61E-76 165.0487866

FPGT-TNNI3K -0.444677837 0.395095872 -24.48612997 2.56E-77 1.65E-76 165.0238847

GATA3 2.101439552 1.443922577 24.47480035 2.83E-77 1.83E-76 164.9226139

GJB4 2.526094051 1.448729359 24.47416245 2.85E-77 1.84E-76 164.9169115

XDH 2.343209588 1.702510802 24.46960925 2.96E-77 1.92E-76 164.8762086

SRSF9 -1.537119058 6.050589015 -24.46756275 3.02E-77 1.95E-76 164.8579133

CHCHD7 -1.227331827 3.932072168 -24.46045994 3.22E-77 2.08E-76 164.7944126

KLHL25 1.304993609 2.046227079 24.44949907 3.55E-77 2.29E-76 164.6964102

ROCK2 1.052576497 3.347454624 24.44349923 3.74E-77 2.42E-76 164.6427599

OSBPL2 -1.433413905 4.610220441 -24.44113705 3.82E-77 2.47E-76 164.6216365

ATL3 1.276990128 4.287900663 24.43916161 3.89E-77 2.51E-76 164.6039709

ITGB4 3.091728489 5.587249386 24.43580163 4.01E-77 2.59E-76 164.573923

SSC4D -1.315783584 1.554429597 -24.43524505 4.03E-77 2.60E-76 164.5689456

PRKG1 1.380266477 1.824949315 24.42496212 4.42E-77 2.85E-76 164.4769794

IMMP1L -1.335939861 3.450810607 -24.42373119 4.47E-77 2.88E-76 164.4659697

BMP2 1.71957226 3.119167736 24.42044258 4.60E-77 2.96E-76 164.4365552

SLC25A32 1.033494358 3.388978391 24.41964405 4.63E-77 2.98E-76 164.4294127

ARFIP2 -1.525579766 5.314182745 -24.41886262 4.66E-77 3.00E-76 164.422423

ADCK2 1.165845672 4.059025124 24.41881328 4.67E-77 3.00E-76 164.4219817

PXDC1 1.59028207 4.622701124 24.4059614 5.23E-77 3.37E-76 164.3070175

THY1 2.773157008 4.972592969 24.39927009 5.56E-77 3.57E-76 164.247155

SLC16A3 2.602531628 3.849505526 24.39819751 5.61E-77 3.61E-76 164.2375589

EML6 -1.131561206 0.992660808 -24.39181764 5.94E-77 3.82E-76 164.1804779

SSH1 1.056649464 2.767221619 24.38745085 6.18E-77 3.97E-76 164.1414058

BEST1 -1.020758144 1.339480729 -24.38335414 6.41E-77 4.12E-76 164.1047484

MMP15 1.627112665 4.633972642 24.38326581 6.41E-77 4.12E-76 164.103958

TRIM31 3.293852606 2.232841243 24.38099943 6.54E-77 4.20E-76 164.0836778

CCDC183 -1.930037773 2.168976636 -24.37862545 6.68E-77 4.29E-76 164.0624341

EXOC8 0.856201216 2.707194659 24.37181686 7.10E-77 4.56E-76 164.0015041

FCHSD2 1.146534114 3.527151655 24.37080621 7.17E-77 4.60E-76 163.9924593

SLC25A43 1.13608686 3.293176649 24.36933021 7.26E-77 4.66E-76 163.9792499

RPS10 -2.299904711 8.576242015 -24.36208353 7.75E-77 4.97E-76 163.9143926

CKAP2L 1.330844148 0.905232918 24.36009537 7.89E-77 5.05E-76 163.8965978

MCU 1.662524066 4.021365704 24.35971901 7.92E-77 5.07E-76 163.8932292

TSPAN18 1.693947757 2.139821442 24.34385576 9.12E-77 5.84E-76 163.7512325

RBBP6 -1.090234727 4.111553143 -24.33784382 9.63E-77 6.16E-76 163.6974114

CPSF4 -1.338661177 4.285495072 -24.33772478 9.64E-77 6.17E-76 163.6963457

ARHGAP33 -1.683958526 3.103740805 -24.33676718 9.72E-77 6.22E-76 163.6877725

GPATCH3 0.97087978 3.324884008 24.33670053 9.73E-77 6.22E-76 163.6871758

GTPBP3 -1.322795116 3.697051496 -24.33475171 9.90E-77 6.33E-76 163.6697282

RGS11 -2.240132673 3.076743569 -24.32754587 1.06E-76 6.74E-76 163.605212

BACE1 -2.492842845 5.255605544 -24.32135728 1.12E-76 7.13E-76 163.5497995

FERMT3 2.332173979 3.894547538 24.32102664 1.12E-76 7.14E-76 163.5468388

ADAM12 2.628074213 1.665902596 24.31979814 1.13E-76 7.22E-76 163.5358384

BET1 -1.214847868 3.777627499 -24.31879007 1.14E-76 7.28E-76 163.5268116

SPATA7 -1.102515556 2.541363937 -24.31852306 1.14E-76 7.30E-76 163.5244206

KLF12 1.155541789 1.493999826 24.3171875 1.16E-76 7.38E-76 163.5124613

ITCH 1.029648708 3.857362226 24.31627276 1.17E-76 7.44E-76 163.50427

BLOC1S5 0.875258889 2.683705311 24.31568605 1.17E-76 7.48E-76 163.4990161

IAH1 -1.184201781 4.134949829 -24.3120528 1.21E-76 7.72E-76 163.4664803

SEC63 -1.642967823 5.458874506 -24.3095886 1.24E-76 7.89E-76 163.4444126

ZNF654 0.857209159 2.353974872 24.30162986 1.33E-76 8.47E-76 163.3731355

PPP5D1 -0.623483545 0.516987876 -24.30143224 1.33E-76 8.49E-76 163.3713656

PDGFRB 2.160093062 5.276481913 24.2979015 1.38E-76 8.76E-76 163.3397428

SPOPL 1.048747074 2.98983073 24.28775978 1.51E-76 9.58E-76 163.2489027

CHSY3 1.413350095 1.150014125 24.2872397 1.51E-76 9.63E-76 163.244244

TXN 1.94775376 7.463523468 24.2796702 1.62E-76 1.03E-75 163.1764364

DPH5 -1.357293717 4.171081104 -24.27727154 1.66E-76 1.05E-75 163.1549481

HLA-G 2.379785348 2.078412329 24.27592723 1.68E-76 1.06E-75 163.1429049

DDX60 1.96567869 3.004880225 24.27491437 1.69E-76 1.07E-75 163.1338309

COCH -3.145076659 2.651847112 -24.26972802 1.77E-76 1.12E-75 163.087366

LEKR1 -0.717990603 0.676475448 -24.26774522 1.80E-76 1.14E-75 163.0696013

TRIM26 1.087904867 4.484008466 24.26532171 1.84E-76 1.17E-75 163.0478874

GPR107 1.131932967 4.243773041 24.25687086 1.99E-76 1.26E-75 162.9721663

MPPED2 -0.978581473 1.093685158 -24.25465709 2.03E-76 1.28E-75 162.9523295

CLIC4 2.01543805 5.3287184 24.25321831 2.05E-76 1.30E-75 162.9394367

SWSAP1 1.02502331 1.864680877 24.25295355 2.06E-76 1.30E-75 162.9370642

HTRA1 2.466013125 6.431783695 24.24351506 2.24E-76 1.42E-75 162.8524819

GP2 -8.189009309 8.948491725 -24.23941751 2.32E-76 1.47E-75 162.8157594

VEPH1 -1.754444114 2.139063078 -24.23707985 2.37E-76 1.50E-75 162.7948083

PIK3C2B 1.338950018 3.161700332 24.22905004 2.55E-76 1.61E-75 162.722838

IL4I1 2.031851485 1.767777533 24.22893637 2.55E-76 1.61E-75 162.721819

LGALS1 2.999878963 8.214628715 24.22612729 2.62E-76 1.65E-75 162.6966401

C4orf3 1.371786867 5.800435187 24.22398696 2.67E-76 1.69E-75 162.6774549

BANF2 -1.925938455 1.12854105 -24.22296734 2.69E-76 1.70E-75 162.6683151

POMT1 -1.342252603 4.104350513 -24.21855765 2.80E-76 1.77E-75 162.6287862

GADD45A 1.615204819 4.579833421 24.21777757 2.82E-76 1.78E-75 162.6217934

PNO1 1.049958881 3.560398174 24.21490631 2.89E-76 1.83E-75 162.5960538

KLF7 1.32473613 2.791849039 24.21327945 2.94E-76 1.85E-75 162.5814694

EGFL6 1.721922377 1.018214297 24.20911499 3.05E-76 1.92E-75 162.544135

RMND5A 1.124047466 3.762245206 24.20473663 3.17E-76 2.00E-75 162.5048811

PSMC4 1.36937798 5.693221578 24.20449943 3.18E-76 2.00E-75 162.5027545

IMPAD1 1.210172618 4.373394333 24.20340842 3.21E-76 2.02E-75 162.4929728

COX6A2 -1.498669598 0.966285972 -24.20148825 3.26E-76 2.06E-75 162.4757568

TUBGCP6 -1.643160162 4.696425155 -24.20085231 3.28E-76 2.07E-75 162.4700549

KCTD21 0.857624712 2.391779673 24.19754967 3.38E-76 2.13E-75 162.4404429

ZXDC -1.162651702 3.69991406 -24.19380796 3.50E-76 2.20E-75 162.4068927

RIOX1 1.006907222 3.078721785 24.19202955 3.55E-76 2.24E-75 162.3909461

FAM171B 1.434291021 1.429418095 24.18737503 3.70E-76 2.33E-75 162.3492085

AC005832.4 -1.811196587 0.911650621 -24.18676865 3.72E-76 2.34E-75 162.3437709

ZGPAT -1.320217281 3.410301288 -24.18202684 3.88E-76 2.44E-75 162.3012481

SKIDA1 -0.524577188 0.466762449 -24.17802615 4.03E-76 2.53E-75 162.2653697

GRIN2D 2.287872064 1.467419568 24.17521702 4.13E-76 2.59E-75 162.2401765

KCNAB3 -1.090815667 1.439605711 -24.17238844 4.24E-76 2.66E-75 162.2148079

SHH 2.467113195 1.56689573 24.16599458 4.49E-76 2.82E-75 162.1574608

LY75 1.652079458 1.994551005 24.15952051 4.75E-76 2.98E-75 162.0993904

PPP1R12C -1.692228716 5.371784609 -24.1518867 5.09E-76 3.19E-75 162.0309121

OSER1 1.206919151 4.814282588 24.14902283 5.22E-76 3.28E-75 162.0052206

PSMD10 1.045686104 4.433393503 24.14542263 5.39E-76 3.38E-75 161.9729224

UBE2G2 -1.398562687 5.136354122 -24.13826446 5.75E-76 3.61E-75 161.9087013

DLG2 -0.982183562 1.029318868 -24.13762916 5.78E-76 3.62E-75 161.9030013

NDUFA1 1.600359736 6.929356205 24.13016552 6.18E-76 3.87E-75 161.8360339

ARIH2 -1.592834796 4.835693827 -24.12993938 6.20E-76 3.88E-75 161.8340048

STRC -0.53113403 0.370296989 -24.12599736 6.42E-76 4.02E-75 161.7986328

CCDC43 0.886004571 3.57426815 24.11281757 7.23E-76 4.52E-75 161.6803589

ZNF223 -0.883171316 1.388829271 -24.10873964 7.49E-76 4.69E-75 161.6437606

TBPL1 -1.099417957 3.235559828 -24.10579181 7.70E-76 4.81E-75 161.6173037

TNFRSF18 1.763062501 1.340465623 24.10446339 7.79E-76 4.87E-75 161.6053809

CELA3B -8.638499562 8.610058332 -24.09879042 8.19E-76 5.12E-75 161.5544626

PHF11 -1.386466365 4.541852906 -24.09876432 8.20E-76 5.12E-75 161.5542283

TOB2 1.288366002 4.691218101 24.09715914 8.31E-76 5.19E-75 161.5398203

TDRD7 0.991766339 3.201180421 24.09391103 8.56E-76 5.35E-75 161.5106647

CHST11 2.115903005 3.050590712 24.08751758 9.07E-76 5.66E-75 161.453273

SNAI1 2.076315201 2.813794547 24.08635509 9.16E-76 5.72E-75 161.4428374

MGST1 -2.573759252 5.24550985 -24.08620861 9.17E-76 5.72E-75 161.4415224

VILL 2.808632549 3.625630212 24.08610027 9.18E-76 5.73E-75 161.4405498

GGCX -1.221610956 3.955651558 -24.08222833 9.51E-76 5.93E-75 161.4057904

OSCAR 2.064315772 2.090132807 24.08147655 9.57E-76 5.97E-75 161.3990413

EFCAB1 -1.580983168 1.053751343 -24.07647749 1.00E-75 6.24E-75 161.3541609

CADPS -2.391025657 2.896656592 -24.06251938 1.13E-75 7.07E-75 161.2288357

DNAJC19 -1.177251834 4.206890899 -24.06218886 1.14E-75 7.09E-75 161.2258678

FGL2 2.335052249 2.703956467 24.06134664 1.15E-75 7.14E-75 161.2183052

AMACR -1.375720931 2.277430178 -24.05799066 1.18E-75 7.35E-75 161.1881698

COMTD1 -2.426715417 5.723134341 -24.0570305 1.19E-75 7.41E-75 161.1795478

SUSD1 1.208547594 2.874643879 24.05427443 1.22E-75 7.60E-75 161.1547983

TMEM185A -0.934042961 2.862351341 -24.04345696 1.35E-75 8.37E-75 161.0576505

FIP1L1 -1.116885081 4.005601128 -24.03127898 1.50E-75 9.33E-75 160.9482711

BCL2L1 1.635862934 6.089725376 24.02965759 1.52E-75 9.47E-75 160.9337071

SCAMP1 1.34544454 3.899020455 24.02818139 1.54E-75 9.59E-75 160.9204471

TTLL4 -1.29258817 3.580061951 -24.0165344 1.71E-75 1.06E-74 160.8158203

RBMS2 1.170530945 3.315367874 24.01623674 1.72E-75 1.07E-74 160.8131462

PAK1IP1 1.085822337 3.561518016 24.01426385 1.75E-75 1.09E-74 160.795422

KIAA1109 -1.352356751 3.644417916 -24.01295758 1.77E-75 1.10E-74 160.7836865

LCN6 -1.988595252 1.450531111 -24.0128201 1.77E-75 1.10E-74 160.7824514

RPF1 1.037417118 4.688093159 24.01039458 1.81E-75 1.12E-74 160.76066

ITGA9 1.491849958 1.814145526 24.00821509 1.85E-75 1.14E-74 160.7410786

GDF1 -0.232632613 0.112935011 -24.00330128 1.93E-75 1.20E-74 160.6969293

STEAP1 2.167505167 3.71938417 24.00189794 1.95E-75 1.21E-74 160.6843202

CEPT1 -1.104493515 3.313286355 -24.00054538 1.98E-75 1.23E-74 160.6721672

HACD2 1.186722601 4.019833035 23.99837196 2.02E-75 1.25E-74 160.6526383

SRGAP2B -0.987920501 1.136090023 -23.9928956 2.12E-75 1.31E-74 160.6034293

HERC3 -1.652241866 3.945183467 -23.99266361 2.12E-75 1.31E-74 160.6013446

C1QC 3.142029057 6.013836838 23.99262803 2.12E-75 1.31E-74 160.6010249

C9orf131 -0.340062665 0.193316832 -23.98811214 2.21E-75 1.37E-74 160.5604442

SERPINE1 3.732782526 5.620916031 23.98528967 2.27E-75 1.40E-74 160.5350799

VPS13A -1.370123738 3.216964827 -23.98193909 2.34E-75 1.44E-74 160.5049688

BCAS1 2.973778797 2.35134494 23.98023568 2.37E-75 1.47E-74 160.4896601

TMEM176B 2.753767265 6.425115466 23.97289687 2.54E-75 1.57E-74 160.4237024

PTPN2 -1.297799477 4.196826841 -23.97212267 2.55E-75 1.58E-74 160.416744

GINS2 1.437739764 1.57413427 23.97202545 2.56E-75 1.58E-74 160.4158702

PRXL2C 1.120403734 3.019916274 23.9697422 2.61E-75 1.61E-74 160.3953482

PRH1 -0.967666457 0.82570523 -23.96952247 2.61E-75 1.61E-74 160.3933732

KLRB1 2.114098057 1.58820324 23.9660519 2.70E-75 1.66E-74 160.3621785

LAMA3 2.652050429 3.654708489 23.96212632 2.79E-75 1.72E-74 160.3268927

SPATA1 -0.858353419 0.862986872 -23.95501599 2.98E-75 1.83E-74 160.2629764

CXCL14 4.087877973 5.047692817 23.95391628 3.01E-75 1.85E-74 160.2530905

MYH14 2.035528123 5.084838976 23.95382814 3.01E-75 1.85E-74 160.2522981

RGPD2 -1.953791864 1.06083239 -23.9534847 3.02E-75 1.86E-74 160.2492107

LARGE2 -2.10429757 3.626549676 -23.94769987 3.18E-75 1.96E-74 160.1972052

OTUD7B 0.849312165 2.417723411 23.94681375 3.21E-75 1.97E-74 160.1892387

ZFAND1 -1.368886677 4.706043565 -23.94399427 3.29E-75 2.02E-74 160.1638903

SMAP1 -1.323928252 3.605464473 -23.94206232 3.35E-75 2.06E-74 160.1465208

TRAP1 -1.509428233 5.242827242 -23.94165391 3.36E-75 2.06E-74 160.1428488

C16orf96 -0.385139508 0.311275727 -23.94062901 3.39E-75 2.08E-74 160.1336341

PSMD1 1.217739555 4.918782478 23.93699713 3.50E-75 2.15E-74 160.1009795

AC008770.2 -0.805373859 0.526910327 -23.93668441 3.51E-75 2.16E-74 160.0981678

OPA3 0.8368132 2.493689341 23.92665626 3.84E-75 2.36E-74 160.007997

CXCL5 4.867205896 2.979012788 23.92181121 4.01E-75 2.46E-74 159.9644282

S100A5 1.387353078 0.77935784 23.91908443 4.11E-75 2.52E-74 159.9399067

TFAP4 -1.21042753 2.800882269 -23.91793035 4.16E-75 2.55E-74 159.9295281

HCK 2.117146511 2.856962009 23.91302478 4.34E-75 2.66E-74 159.8854109

CPXM2 2.282732177 2.887040858 23.91168453 4.40E-75 2.69E-74 159.8733573

SP110 -1.366698409 2.626728482 -23.90933734 4.49E-75 2.75E-74 159.8522473

MYO1D 1.308934264 5.128099154 23.89891422 4.93E-75 3.02E-74 159.7584981

TMEM248 1.139661577 5.647299593 23.89807161 4.97E-75 3.04E-74 159.7509188

KLF2 2.531900742 4.574143205 23.8914072 5.28E-75 3.23E-74 159.690971

FCGR3A 2.788547306 3.878613928 23.88895677 5.39E-75 3.30E-74 159.6689277

RBM42 1.366070484 5.604447883 23.88738204 5.47E-75 3.35E-74 159.6547616

HPS4 -1.202863561 3.784967873 -23.88036066 5.83E-75 3.56E-74 159.5915955

PLGLB2 -1.165517802 0.609530061 -23.87946281 5.87E-75 3.59E-74 159.5835179

GPR150 -1.88207175 1.564812288 -23.8750821 6.11E-75 3.73E-74 159.5441052

EID1 1.627416107 6.292737578 23.87172576 6.30E-75 3.85E-74 159.5139074

UFD1 -1.291507242 4.374131799 -23.8716597 6.30E-75 3.85E-74 159.5133131

RABL6 -1.564359074 5.376181746 -23.86828075 6.49E-75 3.96E-74 159.4829108

TMEM80 -1.450620201 3.972360884 -23.86771452 6.53E-75 3.98E-74 159.477816

AURKB 1.985182449 1.833160643 23.85958277 7.02E-75 4.28E-74 159.4046452

FRMD5 1.364901481 1.040098936 23.8469162 7.87E-75 4.80E-74 159.2906572

CTBP1 -1.675044635 6.021096101 -23.84629636 7.91E-75 4.83E-74 159.2850788

HSD17B14 2.018419614 2.936893936 23.84457432 8.04E-75 4.90E-74 159.2695807

RPL38 -2.159714736 8.402905625 -23.84079547 8.32E-75 5.07E-74 159.2355706

MRFAP1L1 1.152194143 5.372953792 23.83933281 8.43E-75 5.13E-74 159.2224062

TRMT13 -1.027813301 3.065683167 -23.83187711 9.01E-75 5.49E-74 159.1552992

IKBKB -1.380819371 4.374944115 -23.83082177 9.10E-75 5.54E-74 159.1457998

MTUS1 -2.015650783 5.347286101 -23.82430111 9.65E-75 5.87E-74 159.087104

ECHS1 1.45088914 6.452675926 23.8211013 9.93E-75 6.04E-74 159.0582993

SLC35E3 -1.106320045 1.920337476 -23.81942747 1.01E-74 6.13E-74 159.0432312

SPC25 1.578974569 1.780046492 23.81310196 1.07E-74 6.49E-74 158.9862853

EVI2A 2.117627007 2.246683658 23.81255327 1.07E-74 6.52E-74 158.9813455

CFAP69 -1.116578536 1.785958184 -23.79953862 1.21E-74 7.32E-74 158.8641677

SLAMF7 2.01850078 1.613286874 23.79384431 1.27E-74 7.71E-74 158.8128939

UTP6 -1.168346369 4.340418868 -23.79060044 1.31E-74 7.93E-74 158.7836834

ATP5MPL -1.561180699 5.828263225 -23.77486884 1.50E-74 9.14E-74 158.6420095

ELAC1 -1.210731082 2.487499611 -23.77240869 1.54E-74 9.34E-74 158.619852

SNF8 -1.401454094 5.271548274 -23.7712767 1.55E-74 9.43E-74 158.6096566

COLGALT1 1.451944606 5.311851548 23.76873099 1.59E-74 9.65E-74 158.5867277

KDELR3 2.018475627 4.544142205 23.76484824 1.65E-74 9.99E-74 158.5517551

SAP25 -1.69787234 1.386980121 -23.75666762 1.77E-74 1.07E-73 158.4780662

TUBG2 -2.224659645 4.883518396 -23.75263805 1.84E-74 1.11E-73 158.4417667

HLA-DQA1 2.893630538 3.621536923 23.74982185 1.89E-74 1.14E-73 158.4163966

PPP1R3D 0.96720242 2.446210731 23.74895427 1.90E-74 1.15E-73 158.4085807

DHRS11 -1.565537599 3.03292448 -23.7480473 1.92E-74 1.16E-73 158.40041

CD3D 2.520653875 2.38978206 23.74375171 1.99E-74 1.21E-73 158.3617106

LRRC37A3 -1.282420062 1.695936136 -23.74355899 1.99E-74 1.21E-73 158.3599743

LEAP2 -1.202186239 1.642592119 -23.743441 2.00E-74 1.21E-73 158.3589113

FBXO15 -0.919227817 0.752091495 -23.73761245 2.10E-74 1.27E-73 158.3063984

S100A14 4.086964668 5.775216984 23.73728233 2.11E-74 1.28E-73 158.303424

SRGAP1 -0.970899289 1.990365648 -23.73663805 2.12E-74 1.28E-73 158.2976191

CYBB 2.530566944 3.192068574 23.72900509 2.27E-74 1.37E-73 158.2288437

STAU1 1.254373507 5.795600864 23.71625948 2.55E-74 1.54E-73 158.1139899

NGDN -1.126548589 4.177400939 -23.71182944 2.65E-74 1.60E-73 158.0740661

GLT8D2 1.950441431 2.834761318 23.70805257 2.75E-74 1.66E-73 158.0400273

PLXNB1 -1.954020655 5.749471569 -23.70724992 2.77E-74 1.67E-73 158.0327934

FAM53A -0.859697743 1.035785242 -23.70605271 2.80E-74 1.69E-73 158.0220032

THAP3 -1.164401193 3.480603868 -23.70426456 2.84E-74 1.71E-73 158.0058869

ZNF720 -0.884954666 2.388694172 -23.70391629 2.85E-74 1.72E-73 158.002748

TAZ -1.57046075 4.612788362 -23.70240419 2.89E-74 1.74E-73 157.9891193

SLK 1.427016581 4.34062985 23.69684625 3.04E-74 1.83E-73 157.9390235

P4HA1 1.812625235 4.72829672 23.69403349 3.12E-74 1.88E-73 157.91367

NOP56 -1.583423299 5.82856829 -23.69380012 3.12E-74 1.88E-73 157.9115664

CYP2C18 2.824258559 1.782353088 23.69282127 3.15E-74 1.90E-73 157.9027431

TIMM44 -1.273311339 4.322204234 -23.69030391 3.22E-74 1.94E-73 157.8800513

PCID2 -1.117530899 4.214306613 -23.69012362 3.23E-74 1.94E-73 157.8784261

ZNF786 0.864577222 2.148534918 23.68984192 3.24E-74 1.95E-73 157.8758869

WDR97 -1.262302013 1.081399724 -23.68696826 3.32E-74 2.00E-73 157.8499825

RPS6KA3 1.19174242 3.884186411 23.68461318 3.39E-74 2.04E-73 157.8287523

CCDC200 -0.857474912 1.205581605 -23.67811763 3.60E-74 2.16E-73 157.7701946

CENPE 1.129923804 0.886642245 23.67127279 3.83E-74 2.30E-73 157.7084838

FAM200B -1.213432586 3.719034072 -23.66926178 3.90E-74 2.34E-73 157.6903524

NEDD8 -1.440338823 5.770831355 -23.66410497 4.08E-74 2.45E-73 157.6438566

BIVM -1.032990218 3.334640667 -23.66349203 4.10E-74 2.46E-73 157.6383299

NCF2 2.415738487 3.174414204 23.6627531 4.13E-74 2.48E-73 157.6316671

E2F8 1.240953825 0.832532967 23.66059357 4.21E-74 2.53E-73 157.612195

ANKRD6 -1.179763725 2.662105903 -23.66014151 4.23E-74 2.54E-73 157.6081188

ANKRD35 1.239621228 1.36703821 23.6583822 4.30E-74 2.58E-73 157.5922549

SERGEF -1.352021439 4.093326215 -23.657727 4.32E-74 2.59E-73 157.5863469

EIF2B5 -1.262436766 4.910430634 -23.6573799 4.34E-74 2.60E-73 157.5832171

RAB12 1.161401095 4.41187649 23.65240694 4.53E-74 2.72E-73 157.5383735

SLC29A3 1.227633645 3.03745202 23.64726829 4.75E-74 2.84E-73 157.4920335

TRIM27 -1.311999564 4.841585328 -23.64677241 4.77E-74 2.85E-73 157.4875615

IMP4 -1.499639334 5.709927463 -23.64097285 5.03E-74 3.01E-73 157.4352583

CTSD 2.203983934 9.018057963 23.63318737 5.39E-74 3.22E-73 157.3650402

RTTN -0.863817623 1.840195377 -23.6314756 5.48E-74 3.27E-73 157.3496008

TMEM33 1.100199777 3.687703979 23.63131767 5.48E-74 3.28E-73 157.3481763

S100A3 1.862448693 1.734879685 23.63062862 5.52E-74 3.30E-73 157.3419613

CLK1 -1.927950338 6.196636673 -23.61989775 6.08E-74 3.63E-73 157.245167

CTRC -8.060995411 8.227618123 -23.61889652 6.13E-74 3.66E-73 157.2361351

FAM32A 1.161189766 5.890758784 23.61702104 6.24E-74 3.72E-73 157.2192167

DLAT 0.999344121 3.473691797 23.6127367 6.48E-74 3.87E-73 157.1805671

CEP295 -0.881971357 2.070422896 -23.60775836 6.78E-74 4.05E-73 157.1356548

CCDC6 1.173291542 4.428399026 23.60767492 6.79E-74 4.05E-73 157.134902

COL11A1 3.924324164 2.579186375 23.60548865 6.92E-74 4.13E-73 157.1151777

RNF5 1.207363544 5.584875054 23.60260943 7.10E-74 4.23E-73 157.0892011

HINT3 1.053965488 3.964064338 23.59142194 7.86E-74 4.68E-73 156.9882591

AC005041.1 1.277642728 2.165840044 23.59038714 7.93E-74 4.72E-73 156.9789217

CHMP1A 1.247906663 5.814041477 23.59020511 7.94E-74 4.73E-73 156.9772792

PPP1R15A 1.934744833 5.30135616 23.58718948 8.16E-74 4.86E-73 156.9500676

C15orf39 1.117236587 3.789048879 23.58655407 8.21E-74 4.89E-73 156.9443339

CAPG 2.604538385 6.266759742 23.58613918 8.24E-74 4.90E-73 156.9405901

HIST1H2AC 2.45041599 4.400308798 23.58564926 8.28E-74 4.92E-73 156.9361691

KIF13B 1.42872781 3.926846174 23.57970344 8.73E-74 5.19E-73 156.8825138

HOXB4 1.293366254 1.718964109 23.57889004 8.80E-74 5.23E-73 156.8751733

MDM2 -1.596594213 4.094620426 -23.56940158 9.58E-74 5.69E-73 156.7895415

AGPS 1.077750383 3.336579053 23.56768176 9.73E-74 5.78E-73 156.7740196

CENPA 1.555453042 1.191840897 23.56648575 9.84E-74 5.84E-73 156.7632251

GMEB1 0.74900354 2.337828057 23.5652863 9.95E-74 5.90E-73 156.7523993

MYCBP2 -1.38649182 3.896925888 -23.56483797 9.99E-74 5.93E-73 156.7483528

RAB11FIP3 -1.434755434 4.791374771 -23.56256834 1.02E-73 6.05E-73 156.7278676

INPP4B 1.685698034 2.349071693 23.56065929 1.04E-73 6.15E-73 156.7106367

ERN2 3.644203345 2.910457448 23.55496211 1.09E-73 6.47E-73 156.6592121

LOX 2.457296236 3.191817906 23.55323353 1.11E-73 6.57E-73 156.6436089

ESYT3 -1.07681938 1.199225963 -23.54848556 1.16E-73 6.86E-73 156.6007492

CHRNA7 -1.141196872 0.933314472 -23.54434449 1.20E-73 7.12E-73 156.5633664

KHDC1 -1.010647742 1.706491907 -23.54283423 1.22E-73 7.21E-73 156.5497323

RBM45 -0.839989626 2.67040675 -23.53850472 1.27E-73 7.50E-73 156.5106461

DNAJB11 -1.537695287 5.664077861 -23.52494456 1.43E-73 8.47E-73 156.3882159

RLF 0.928799028 2.66200397 23.51911377 1.51E-73 8.93E-73 156.3355666

TNC 2.891875336 2.883509509 23.51889352 1.51E-73 8.94E-73 156.3335778

DISP1 0.935643713 2.058908474 23.51835816 1.52E-73 8.98E-73 156.3287435

GDA 2.339418916 2.334634869 23.51254565 1.60E-73 9.46E-73 156.2762558

FBXO22 -1.060083575 2.858905213 -23.51063359 1.63E-73 9.63E-73 156.258989

NMI 1.326213506 3.53521822 23.51007407 1.64E-73 9.67E-73 156.2539362

TRUB1 1.007362303 2.92144181 23.4979851 1.83E-73 1.08E-72 156.1447587

TMEM204 1.78240313 4.594033166 23.49567606 1.86E-73 1.10E-72 156.123904

PMM1 -1.868280449 4.315871327 -23.49124988 1.94E-73 1.15E-72 156.0839262

TIGD7 -0.804889643 1.422928849 -23.49110475 1.94E-73 1.15E-72 156.0826153

P4HA2 -1.784654789 5.188176057 -23.49043591 1.95E-73 1.15E-72 156.0765741

BLZF1 1.094491006 3.426874332 23.48742436 2.01E-73 1.18E-72 156.0493723

PTK6 3.061269666 3.336887789 23.48446 2.06E-73 1.22E-72 156.0225958

MXD1 1.89688334 3.200616328 23.48133507 2.12E-73 1.25E-72 155.9943682

SH2B3 1.303070003 3.441087728 23.47928297 2.16E-73 1.27E-72 155.9758309

TAF1C -1.525596272 4.727376741 -23.47804902 2.19E-73 1.29E-72 155.9646841

LHFPL6 2.057186387 4.507960511 23.47553419 2.24E-73 1.32E-72 155.9419662

PHLDA1 2.012698128 4.196844461 23.47469651 2.25E-73 1.33E-72 155.9343988

CDYL2 1.062246303 1.524969347 23.46906722 2.37E-73 1.39E-72 155.8835435

INPP5B -0.94257478 2.936538848 -23.46230835 2.52E-73 1.48E-72 155.8224797

DDTL -1.58579304 2.596962224 -23.45358245 2.73E-73 1.60E-72 155.7436387

UROS -1.249075696 4.032831631 -23.45256033 2.75E-73 1.62E-72 155.7344031

ZMAT1 -1.754095521 2.935727792 -23.45125331 2.78E-73 1.64E-72 155.7225931

TUBB 1.8363123 7.547535056 23.4437274 2.98E-73 1.75E-72 155.6545873

AC005520.1 -0.303603721 0.176008301 -23.4417337 3.03E-73 1.78E-72 155.6365709

CDCA2 1.225269549 0.875210948 23.43468833 3.23E-73 1.90E-72 155.5729014

GOLGA8M -0.646526223 0.389639079 -23.43007435 3.37E-73 1.98E-72 155.5312023

ZNF736 -1.020566305 2.034755751 -23.42317665 3.59E-73 2.11E-72 155.4688603

ZNF35 0.752574947 2.0834147 23.42216562 3.62E-73 2.12E-72 155.4597221

NUAK1 1.625897825 2.69489336 23.42195939 3.63E-73 2.13E-72 155.4578581

PPP1R18 1.835002613 4.708147469 23.4167583 3.80E-73 2.23E-72 155.4108468

TAF3 0.718346621 2.330252689 23.41546579 3.85E-73 2.25E-72 155.3991638

ZBED5 -1.071280822 3.96683978 -23.41410765 3.89E-73 2.28E-72 155.3868874

SELL 2.666320161 2.197694221 23.41046763 4.02E-73 2.36E-72 155.3539839

CBX1 1.117669101 4.675998213 23.40581774 4.20E-73 2.46E-72 155.3119502

CTRB2 -9.043519924 9.778083019 -23.40503527 4.23E-73 2.47E-72 155.3048767

POLD4 -1.87873364 5.568636184 -23.40420774 4.26E-73 2.49E-72 155.2973957

FAM114A2 -1.041495474 3.253386668 -23.40382411 4.27E-73 2.50E-72 155.2939277

TRIM74 -1.148248178 0.764334844 -23.39837932 4.49E-73 2.63E-72 155.2447048

6-Mar -1.428479252 5.40984144 -23.39790885 4.51E-73 2.64E-72 155.2404515

PIGT 1.565812969 6.366951062 23.38980542 4.85E-73 2.84E-72 155.1671884

COTL1 1.778251586 5.205142855 23.38741426 4.96E-73 2.90E-72 155.1455687

CIP2A 1.093615832 1.295168619 23.37181182 5.71E-73 3.33E-72 155.0044874

THAP6 -0.838641704 2.558037178 -23.36925832 5.84E-73 3.41E-72 154.9813959

COL28A1 -2.05392952 1.975699945 -23.36655319 5.98E-73 3.49E-72 154.9569327

DLL4 1.58387073 2.581371006 23.36343142 6.15E-73 3.59E-72 154.9287007

NCS1 1.545596389 3.001654757 23.36297222 6.18E-73 3.61E-72 154.9245478

KIZ -1.332926124 3.509239383 -23.36226221 6.22E-73 3.63E-72 154.9181267

CCDC157 -0.791635427 1.30910816 -23.35628696 6.57E-73 3.83E-72 154.8640862

CIRBP -2.228141431 7.56814708 -23.3545144 6.67E-73 3.89E-72 154.8480544

ZNF792 0.797266704 1.771955413 23.35298746 6.76E-73 3.94E-72 154.8342439

F2RL1 2.095679275 4.557113549 23.35186964 6.83E-73 3.98E-72 154.8241336

ADSL -1.295297033 4.67020638 -23.35166014 6.85E-73 3.99E-72 154.8222387

CENPQ 0.937313009 2.039393398 23.34441077 7.31E-73 4.26E-72 154.7566678

CLCC1 -1.11489568 3.736939895 -23.33757065 7.78E-73 4.53E-72 154.6947943

UTP14C 0.952229113 3.130578728 23.3270341 8.55E-73 4.98E-72 154.599476

DPY19L2 -1.437610667 1.692574015 -23.32222274 8.93E-73 5.20E-72 154.555947

ANKS1A 0.974650987 3.476080556 23.3152733 9.51E-73 5.53E-72 154.4930711

ATG4B -1.498274224 5.019837063 -23.31188192 9.81E-73 5.70E-72 154.4623855

RPL34 -2.256071699 9.353789934 -23.31170489 9.82E-73 5.71E-72 154.4607837

SPON1 2.669344823 3.710686542 23.29511406 1.14E-72 6.63E-72 154.3106537

TBC1D3L -2.595847496 2.02651668 -23.29402346 1.15E-72 6.70E-72 154.300784

LRP2BP -1.039007271 1.328952967 -23.2862146 1.24E-72 7.19E-72 154.2301127

ZBTB24 0.774065724 2.040096706 23.28379608 1.26E-72 7.34E-72 154.2082237

CEP112 -1.177202201 2.543705131 -23.28175779 1.29E-72 7.48E-72 154.1897756

PPP2R5E 0.874475497 3.158620424 23.27384846 1.38E-72 8.03E-72 154.1181865

PTPRE 1.251783271 2.221316288 23.26540381 1.49E-72 8.66E-72 154.041746

BRI3BP 1.25351262 2.794639733 23.25926237 1.58E-72 9.16E-72 153.9861501

LOXL2 2.296640998 3.735025769 23.25201858 1.69E-72 9.77E-72 153.920571

TLR4 1.571492175 2.046342418 23.25054255 1.71E-72 9.90E-72 153.9072078

MAST2 1.217649953 3.824267093 23.24690987 1.77E-72 1.02E-71 153.8743183

SH3BGRL3 2.157766266 7.547426456 23.24077326 1.87E-72 1.08E-71 153.8187564

TIMM22 0.967805191 3.211979448 23.2394176 1.89E-72 1.09E-71 153.8064815

TIAL1 -1.238125371 5.032537311 -23.2371093 1.93E-72 1.12E-71 153.7855806

L3MBTL3 1.013450722 2.166053421 23.23704993 1.93E-72 1.12E-71 153.785043

GOLGA8H -0.613296554 0.591538583 -23.23270733 2.01E-72 1.16E-71 153.745721

THEM4 -1.156648309 3.59613566 -23.22563004 2.14E-72 1.24E-71 153.6816328

SGK1 -2.382298601 5.510408387 -23.22540865 2.14E-72 1.24E-71 153.679628

GBP2 1.929900162 4.187929615 23.22103688 2.23E-72 1.29E-71 153.6400373

NLE1 -1.453834593 3.607715035 -23.21698933 2.31E-72 1.34E-71 153.6033813

MRPL52 -1.606608932 5.573561617 -23.21640422 2.33E-72 1.34E-71 153.5980823

ZNF302 -1.313869688 4.043974878 -23.20384582 2.61E-72 1.51E-71 153.4843394

ZNF382 -0.750675918 0.908599495 -23.19039381 2.94E-72 1.70E-71 153.3624879

IL2RA 2.119813755 1.391230648 23.18704285 3.04E-72 1.75E-71 153.3321317

TMCO6 -1.162633845 3.515304581 -23.18531938 3.08E-72 1.78E-71 153.3165184

ZNF85 -1.56270294 2.06183039 -23.17730578 3.32E-72 1.91E-71 153.2439184

PKM 2.025597364 7.668795361 23.17446124 3.40E-72 1.96E-71 153.2181467

CHORDC1 -1.269743439 3.01167749 -23.16882519 3.58E-72 2.06E-71 153.1670816

TNFSF9 2.319697586 1.435109436 23.16664244 3.65E-72 2.10E-71 153.1473042

VNN1 3.328029998 2.717505122 23.1662441 3.66E-72 2.11E-71 153.1436948

ARHGEF10L -1.604800463 4.994982017 -23.16342235 3.76E-72 2.17E-71 153.1181269

PLAGL2 0.921835249 2.860131001 23.16114186 3.84E-72 2.21E-71 153.0974628

DDX58 1.342095572 3.156965497 23.15867842 3.92E-72 2.26E-71 153.0751405

ANGEL1 -1.588356957 4.276056283 -23.15567414 4.03E-72 2.32E-71 153.0479167

MRPL48 -1.515981657 4.006500522 -23.14867238 4.30E-72 2.47E-71 152.9844659

SERTAD2 1.278489248 3.128768564 23.14404345 4.48E-72 2.58E-71 152.9425157

MSRB3 2.006952483 3.116042535 23.14098597 4.61E-72 2.65E-71 152.914806

RNF11 1.31515067 5.119249884 23.13598708 4.82E-72 2.77E-71 152.8694996

PYCARD 1.796763525 4.23989554 23.13568167 4.83E-72 2.78E-71 152.8667315

IFNGR1 1.517467661 5.64020867 23.13551086 4.84E-72 2.78E-71 152.8651833

AC010531.1 -0.867777724 0.587408728 -23.13481042 4.87E-72 2.80E-71 152.8588348

TMEM119 2.628802086 3.968076361 23.1347802 4.87E-72 2.80E-71 152.8585609

CCDC198 -2.552099999 4.426027669 -23.13003073 5.09E-72 2.92E-71 152.8155126

JAM3 1.451653615 3.415431793 23.12871337 5.15E-72 2.95E-71 152.8035719

IBA57 -1.04295791 2.043806244 -23.12629059 5.26E-72 3.02E-71 152.7816114

ANKRD39 -0.946847929 2.632588162 -23.12319905 5.41E-72 3.10E-71 152.7535882

AHNAK2 2.637109602 2.596606945 23.12023267 5.56E-72 3.19E-71 152.7266988

PRDX1 1.783161867 7.401731352 23.11956393 5.59E-72 3.21E-71 152.7206367

CYREN -1.095168889 3.889435914 -23.11932414 5.60E-72 3.21E-71 152.718463

PLEKHJ1 -1.645699286 5.379931017 -23.1179855 5.67E-72 3.25E-71 152.7063282

EFL1 1.022075555 3.075139312 23.11707692 5.72E-72 3.28E-71 152.6980919

HSD11B1 2.327211783 2.485306222 23.11364646 5.90E-72 3.38E-71 152.6669939

TCF25 -1.3546375 5.915048869 -23.10617839 6.31E-72 3.61E-71 152.5992903

RTL8B 1.294482594 3.273470905 23.10188623 6.56E-72 3.76E-71 152.5603765

TMEM60 1.046144033 4.400216441 23.10031983 6.66E-72 3.81E-71 152.5461748

DARS2 1.14273868 3.195450636 23.10031285 6.66E-72 3.81E-71 152.5461115

NDRG2 -2.016310733 5.683472481 -23.09993307 6.68E-72 3.82E-71 152.5426682

TCN2 1.46316984 4.102074923 23.09884503 6.75E-72 3.86E-71 152.5328033

ZNF396 -0.900992018 1.333189243 -23.09752871 6.83E-72 3.90E-71 152.5208686

TPCN1 -1.636153183 5.081949222 -23.09748336 6.83E-72 3.90E-71 152.5204574

CEBPB 1.891083801 5.657172819 23.09486968 7.00E-72 3.99E-71 152.4967595

TSPYL2 -2.090815789 5.070622372 -23.09294567 7.12E-72 4.06E-71 152.4793142

ARSI 1.802542917 1.334203271 23.09260625 7.14E-72 4.07E-71 152.4762366

HSPE1-MOB4 -1.480394937 0.858706617 -23.09183414 7.19E-72 4.10E-71 152.4692357

EIF4G3 1.043857479 3.832110112 23.09076341 7.26E-72 4.14E-71 152.4595271

FYB1 2.071886262 1.972265766 23.07566921 8.32E-72 4.75E-71 152.3226526

SNX29 1.097807639 2.265784049 23.07246411 8.57E-72 4.89E-71 152.2935863

ARL9 1.284284277 0.878280052 23.07113645 8.67E-72 4.94E-71 152.2815457

MPST -1.987128703 5.290520712 -23.06801876 8.92E-72 5.08E-71 152.2532708

ZZEF1 -1.272041846 3.669331712 -23.06565067 9.12E-72 5.19E-71 152.2317937

ZMAT5 -1.100220094 3.45300869 -23.06309876 9.33E-72 5.31E-71 152.2086488

CCDC3 1.884378767 2.93770724 23.0630831 9.33E-72 5.31E-71 152.2085068

UVRAG 0.800744231 2.918120189 23.06197447 9.42E-72 5.36E-71 152.1984517

KCNA2 -0.553682127 0.388253969 -23.06052669 9.55E-72 5.43E-71 152.1853206

MROH7 -0.878528591 0.815121473 -23.05698181 9.86E-72 5.61E-71 152.1531682

PSMC2 1.107331527 5.041224708 23.0523289 1.03E-71 5.85E-71 152.1109642

DEPDC1B 1.435143705 1.049004098 23.05217556 1.03E-71 5.85E-71 152.1095734

MTA2 1.036860349 5.133397852 23.05113841 1.04E-71 5.91E-71 152.1001656

GNB5 -1.168727821 3.220217008 -23.04947515 1.06E-71 6.00E-71 152.0850785

SEC31B -1.360820249 2.195360131 -23.04015474 1.15E-71 6.52E-71 152.0005305

CCDC71L 1.371895109 3.568302496 23.03725449 1.18E-71 6.69E-71 151.9742201

DGUOK 1.157899664 5.3687929 23.02667837 1.30E-71 7.37E-71 151.8782696

CD52 3.033695351 4.107228386 23.02294742 1.34E-71 7.62E-71 151.8444188

DUOX2 4.15184763 2.920492247 23.01768963 1.41E-71 7.99E-71 151.796713

SSPN 1.614968453 2.653499652 23.00811563 1.54E-71 8.71E-71 151.7098388

IRS2 1.427647855 2.883004414 23.00746996 1.54E-71 8.76E-71 151.7039798

CCND1 1.937796844 5.499588338 23.00509123 1.58E-71 8.95E-71 151.6823939

RASA3 1.517153917 3.975621189 23.00398878 1.59E-71 9.03E-71 151.6723895

PRPF39 -1.232260138 4.173193418 -23.00141918 1.63E-71 9.24E-71 151.6490708

SEC61G -1.751689439 6.834789223 -22.99881354 1.67E-71 9.46E-71 151.6254244

GGA1 -1.264863869 4.531993056 -22.99564523 1.72E-71 9.74E-71 151.5966711

STX8 -1.326967143 4.500722565 -22.99543027 1.72E-71 9.75E-71 151.5947203

SERPINI1 -1.987811207 3.496624542 -22.99501727 1.73E-71 9.79E-71 151.5909721

MZF1 -1.741602583 3.881629346 -22.99056236 1.80E-71 1.02E-70 151.5505408

HOXD9 1.318697676 1.127268313 22.98963513 1.82E-71 1.03E-70 151.5421253

RAD51 1.411619605 1.411777459 22.98659958 1.87E-71 1.06E-70 151.5145745

BLVRA 1.245895346 4.801720021 22.98310296 1.93E-71 1.09E-70 151.482838

EVL -1.888594734 4.651752913 -22.98151191 1.96E-71 1.10E-70 151.4683968

KATNA1 0.784163632 3.35817612 22.97876043 2.00E-71 1.13E-70 151.4434223

ABI3 1.575553493 2.919781144 22.97830403 2.01E-71 1.14E-70 151.4392797

TXNRD2 -1.11467001 3.427807004 -22.97584144 2.06E-71 1.16E-70 151.4169267

GAS2 -1.597270947 2.068589215 -22.96978206 2.17E-71 1.23E-70 151.3619237

THBD 1.802577614 3.616768441 22.96531513 2.26E-71 1.28E-70 151.3213738

DMAP1 -1.372480896 4.518516709 -22.9610942 2.35E-71 1.33E-70 151.2830555

FAM117B 0.937915901 2.475307589 22.95930842 2.39E-71 1.35E-70 151.2668436

ZGRF1 -0.838194619 1.28682933 -22.95405433 2.51E-71 1.41E-70 151.2191432

GAMT -2.781696537 5.615841886 -22.95377854 2.51E-71 1.42E-70 151.2166394

C1orf131 -1.096716103 3.104271542 -22.95362385 2.52E-71 1.42E-70 151.2152349

CALU 1.737830149 5.815405371 22.95360053 2.52E-71 1.42E-70 151.2150232

TXNRD1 1.328981808 4.295981739 22.94926008 2.62E-71 1.47E-70 151.1756157

RGS19 1.399492661 3.534168453 22.93611084 2.95E-71 1.66E-70 151.0562223

CRLF3 0.889177349 2.712455604 22.93271679 3.04E-71 1.71E-70 151.0254025

FUS -1.544496578 6.405905666 -22.93077983 3.10E-71 1.74E-70 151.0078134

EHBP1L1 1.541499152 4.693179387 22.9293686 3.14E-71 1.76E-70 150.9949981

CMSS1 -1.315480555 3.676620066 -22.92561715 3.25E-71 1.83E-70 150.9609307

MT1X -3.098666113 7.484761572 -22.92547345 3.25E-71 1.83E-70 150.9596257

ZNF334 -1.307204881 2.2929006 -22.92546125 3.25E-71 1.83E-70 150.9595149

SLC26A2 1.103645294 2.297915525 22.92537543 3.25E-71 1.83E-70 150.9587355

PDK3 1.043021576 2.329362902 22.9241564 3.29E-71 1.85E-70 150.9476651

RPE 1.13002803 3.836123953 22.92034307 3.41E-71 1.91E-70 150.913034

PPARG 2.296210833 2.881669757 22.90959168 3.76E-71 2.11E-70 150.815388

SMAD4 -1.339313185 3.828274148 -22.90211463 4.02E-71 2.25E-70 150.7474745

RAB28 0.855516693 3.616552549 22.89747755 4.19E-71 2.35E-70 150.7053538

HAUS4 -1.281636486 4.63734805 -22.89515999 4.28E-71 2.40E-70 150.6843017

DOP1A -1.112426551 2.897028157 -22.89368117 4.34E-71 2.43E-70 150.6708683

ABHD1 -0.672441736 0.601376131 -22.88496778 4.70E-71 2.63E-70 150.5917132

KRT16 4.219999076 2.768261255 22.87793656 5.01E-71 2.80E-70 150.5278349

SMIM12 -1.098926276 3.696137975 -22.87740709 5.03E-71 2.82E-70 150.5230245

TERF1 -0.859612958 2.930510152 -22.87456534 5.16E-71 2.89E-70 150.497206

COPS9 -1.571616315 5.953066204 -22.87394518 5.19E-71 2.91E-70 150.4915715

ZNF319 0.81323659 2.614881199 22.87043506 5.36E-71 3.00E-70 150.4596795

MT-CO3 2.800244024 13.93207993 22.86657031 5.55E-71 3.10E-70 150.4245643

SLC30A2 -3.745317183 3.976227877 -22.86409167 5.68E-71 3.17E-70 150.4020427

ZC2HC1C -1.160817308 2.152339267 -22.86246083 5.76E-71 3.22E-70 150.3872241

TSPAN2 2.009470693 2.363493413 22.85981206 5.90E-71 3.30E-70 150.3631557

DMXL1 -1.164509004 3.118907072 -22.85617865 6.10E-71 3.41E-70 150.3301393

GASK1B 1.805914104 3.512010364 22.85201605 6.33E-71 3.54E-70 150.2923129

THOC3 -1.28722124 3.045596783 -22.85092262 6.40E-71 3.57E-70 150.2823763

MUS81 -1.19409164 4.47643506 -22.85090897 6.40E-71 3.57E-70 150.2822523

FLOT2 1.393587012 5.808473435 22.84804681 6.57E-71 3.67E-70 150.2562422

ST7 -1.185135102 3.833606197 -22.84703578 6.63E-71 3.70E-70 150.2470542

AGAP3 -1.496665452 4.951591172 -22.84521846 6.74E-71 3.76E-70 150.2305386

POFUT1 1.015768174 4.073097457 22.83977688 7.08E-71 3.95E-70 150.1810846

RPS23 -2.149364235 9.065182998 -22.83752276 7.23E-71 4.03E-70 150.160598

AP1M1 -1.061572605 3.57616712 -22.8310922 7.66E-71 4.27E-70 150.1021517

NPHP3-ACAD11 -0.941552764 0.472646304 -22.82947885 7.77E-71 4.33E-70 150.0874876

GPHA2 -4.087327548 3.09081122 -22.82899655 7.81E-71 4.35E-70 150.0831039

ANKRD11 -1.132226519 4.278238442 -22.82865646 7.83E-71 4.36E-70 150.0800127

RUNDC1 0.911854789 3.354610721 22.82653653 7.98E-71 4.44E-70 150.0607439

DCP1A 0.852689084 3.114943283 22.82532039 8.07E-71 4.49E-70 150.0496897

SYK 1.581441483 3.829612814 22.8241759 8.16E-71 4.54E-70 150.0392867

KCMF1 -1.55399452 4.908757389 -22.80671751 9.56E-71 5.32E-70 149.8805831

TMX4 1.474667896 3.946921937 22.80181357 9.99E-71 5.56E-70 149.8359998

FAF2 0.969130424 4.23233722 22.80063395 1.01E-70 5.62E-70 149.8252753

DNAJC4 -1.534275326 5.553700532 -22.79796872 1.03E-70 5.75E-70 149.8010437

ZNF296 1.364723515 1.422641045 22.79761793 1.04E-70 5.77E-70 149.7978544

LIN54 0.796245233 1.924981048 22.79742658 1.04E-70 5.78E-70 149.7961147

SSR4 -2.33664695 7.816612723 -22.7875939 1.14E-70 6.32E-70 149.7067133

S1PR4 1.894609115 1.719788561 22.78512643 1.16E-70 6.46E-70 149.6842772

PPP1R2 0.969045088 4.310811413 22.78498942 1.16E-70 6.46E-70 149.6830313

PHACTR1 -1.250758842 1.811545435 -22.78482368 1.17E-70 6.47E-70 149.6815243

NFIX 1.373138433 4.26484023 22.78204203 1.20E-70 6.64E-70 149.6562307

INKA1 1.245313131 1.748281919 22.78084842 1.21E-70 6.71E-70 149.6453771

ERP27 -4.381836298 5.462345226 -22.78024842 1.22E-70 6.74E-70 149.639921

ZNF782 -0.866779679 1.724218358 -22.77962173 1.22E-70 6.78E-70 149.6342224

NMNAT3 -1.226433673 2.552873393 -22.77893366 1.23E-70 6.82E-70 149.6279655

GON7 1.014722476 3.695143549 22.77548854 1.27E-70 7.03E-70 149.5966373

ITPKB 1.167902508 3.081805281 22.77483851 1.28E-70 7.07E-70 149.5907261

ATP1B1 2.46325295 7.459438059 22.77101765 1.32E-70 7.32E-70 149.5559797

RTF1 0.90037075 4.000640236 22.76765588 1.36E-70 7.54E-70 149.5254072

IARS2 1.233718649 5.136869169 22.76258409 1.43E-70 7.90E-70 149.4792818

BICD2 1.049138556 3.274660481 22.76169063 1.44E-70 7.96E-70 149.4711561

WNT10A 2.44855315 1.482617928 22.76006496 1.46E-70 8.08E-70 149.4563709

CHID1 -1.547620354 5.648398125 -22.75997157 1.46E-70 8.08E-70 149.4555215

SGTB 0.995510001 1.584272373 22.75865631 1.48E-70 8.18E-70 149.4435592

KIF14 1.024594897 0.693884426 22.75207525 1.57E-70 8.68E-70 149.3837026

FKBP10 1.953043166 5.102402077 22.74815878 1.63E-70 8.99E-70 149.3480794

ARHGAP28 -1.125256266 1.316244745 -22.7479099 1.63E-70 9.01E-70 149.3458157

ADNP 0.960032296 4.448614108 22.74740672 1.64E-70 9.05E-70 149.3412387

ULK1 -1.764022214 5.070867302 -22.74342993 1.70E-70 9.38E-70 149.3050655

ZNF606 -0.946078297 2.228822926 -22.73836205 1.78E-70 9.82E-70 149.2589656

HOXA3 1.608735153 1.629094733 22.73635444 1.81E-70 9.99E-70 149.240703

CCL24 3.228888629 1.813672446 22.73184146 1.89E-70 1.04E-69 149.1996483

MAD2L1BP 1.011521442 4.148849633 22.72598577 1.99E-70 1.10E-69 149.1463765

MIS18A 1.037440937 2.72449389 22.72138957 2.08E-70 1.14E-69 149.1045609

CNOT8 0.975226553 4.644917497 22.71561728 2.19E-70 1.21E-69 149.052043

ZFP91 1.029624823 4.418891861 22.71469126 2.21E-70 1.22E-69 149.0436176

TMEM86A 1.068994969 2.265656722 22.71336316 2.23E-70 1.23E-69 149.0315336

TOMM22 1.145242694 5.279189955 22.70961083 2.31E-70 1.27E-69 148.9973918

CDC45 1.645519779 1.437253234 22.70604245 2.39E-70 1.31E-69 148.9649227

FAM217A -0.866149059 0.535642963 -22.69725305 2.59E-70 1.42E-69 148.8849423

CRYBG2 2.27176019 1.744078644 22.69711262 2.59E-70 1.42E-69 148.8836644

PRKCI 1.424603463 3.748024458 22.69640855 2.61E-70 1.43E-69 148.8772573

TCERG1 -1.199163051 4.262774512 -22.69127834 2.73E-70 1.50E-69 148.830571

ERH 1.266616609 6.221850111 22.68978852 2.77E-70 1.52E-69 148.8170129

TARBP2 -1.246223354 4.265176984 -22.68947893 2.78E-70 1.52E-69 148.8141955

DMPK -2.090067661 4.894845569 -22.68389029 2.92E-70 1.60E-69 148.7633341

ST14 2.002657896 6.433672369 22.68350078 2.93E-70 1.61E-69 148.7597892

POU6F1 -1.073550402 2.744277086 -22.67289499 3.23E-70 1.77E-69 148.6632603

SPIN1 1.188928131 4.830769983 22.67240991 3.24E-70 1.78E-69 148.6588451

ATAD2 1.26393564 2.257197441 22.67220154 3.25E-70 1.78E-69 148.6569485

DHRS4 -1.368509597 4.105628497 -22.66794256 3.38E-70 1.85E-69 148.6181826

NKG7 2.156005978 2.467100722 22.66722387 3.40E-70 1.86E-69 148.6116409

GABRP 4.415380561 2.804051341 22.66637595 3.42E-70 1.88E-69 148.6039227

FXN -1.141792225 3.106313132 -22.6635488 3.51E-70 1.93E-69 148.5781883

ASTN2 -1.428091819 1.788645646 -22.65684496 3.73E-70 2.05E-69 148.5171635

SCFD2 1.042196038 3.06686352 22.65316751 3.86E-70 2.12E-69 148.4836863

SLC4A2 -1.528098061 5.7053867 -22.64787347 4.05E-70 2.22E-69 148.4354907

KPNA7 1.823883325 1.141963528 22.64742031 4.07E-70 2.23E-69 148.4313652

USH1C 2.303476321 3.892501133 22.64354148 4.21E-70 2.31E-69 148.3960518

VPS52 -1.248001798 5.10637364 -22.64167766 4.29E-70 2.35E-69 148.3790829

RNF187 1.122975668 5.728313723 22.64133642 4.30E-70 2.35E-69 148.3759761

SLC41A1 -1.672087132 4.572005038 -22.64018816 4.35E-70 2.38E-69 148.3655217

CXorf40B -1.203667285 4.332301295 -22.63967641 4.37E-70 2.39E-69 148.3608625

CELA2A -8.417933067 8.130021492 -22.63294646 4.64E-70 2.54E-69 148.2995871

SERF2 -1.845543543 7.919167409 -22.63027131 4.76E-70 2.60E-69 148.2752292

TCEA3 -2.582087044 5.917177564 -22.62749766 4.88E-70 2.66E-69 148.2499739

LITAF 1.601798299 5.755985779 22.62541976 4.97E-70 2.71E-69 148.2310532

PLAAT2 2.927045231 1.664174661 22.62477732 5.00E-70 2.73E-69 148.2252034

TM7SF2 -2.487940296 4.657747022 -22.62073265 5.19E-70 2.83E-69 148.1883728

HAS2 1.92669706 1.35654977 22.60987143 5.73E-70 3.12E-69 148.0894646

C1QTNF2 1.130690437 1.032968688 22.60923754 5.76E-70 3.14E-69 148.0836918

ERMARD -1.097363587 3.578144682 -22.60714472 5.87E-70 3.20E-69 148.0646323

BCO1 1.372830415 0.953790343 22.60647969 5.91E-70 3.22E-69 148.0585757

ARHGAP10 -1.045345329 2.563356951 -22.60546344 5.96E-70 3.25E-69 148.0493204

TGFBR2 1.710552842 5.817348974 22.60471407 6.00E-70 3.27E-69 148.0424956

RPS6KA5 -0.652346097 0.913982692 -22.60113805 6.20E-70 3.38E-69 148.0099269

RFX5 1.124519643 3.84177135 22.59881891 6.33E-70 3.45E-69 147.9888048

KAT8 -1.14186898 4.864932747 -22.59665914 6.46E-70 3.52E-69 147.9691338

DCTPP1 1.200394595 4.902737406 22.59616654 6.49E-70 3.53E-69 147.9646471

ADAMTS7 1.433771699 1.518749652 22.59572109 6.51E-70 3.54E-69 147.96059

ALKBH8 0.731325994 2.038018699 22.59313104 6.67E-70 3.63E-69 147.9369993

GNG11 1.591650888 3.724696511 22.59031713 6.84E-70 3.72E-69 147.911369

OLFML1 1.764225009 2.418737848 22.58957449 6.89E-70 3.74E-69 147.9046047

WIPF2 0.930780357 3.75307671 22.58911716 6.92E-70 3.76E-69 147.900439

ADAM8 2.220038169 3.805554834 22.58780024 7.00E-70 3.80E-69 147.8884437

LYN 1.399813891 3.719992781 22.58543504 7.15E-70 3.89E-69 147.8668995

CLEC2B 1.700154373 2.65876587 22.58535459 7.16E-70 3.89E-69 147.8661666

RHEBL1 1.04636494 1.084759194 22.58475783 7.20E-70 3.91E-69 147.8607308

CWF19L2 0.849601513 2.714837662 22.58341657 7.29E-70 3.95E-69 147.8485132

WHRN -1.586011164 3.138609136 -22.58260846 7.34E-70 3.98E-69 147.8411521

PIK3R6 0.942115498 0.991405685 22.57930039 7.56E-70 4.10E-69 147.811018

HERC4 -0.993514989 3.258160795 -22.57841771 7.62E-70 4.13E-69 147.8029773

XYLT1 1.42756864 2.318410174 22.5783751 7.63E-70 4.14E-69 147.8025891

MSX2 1.967938027 1.58197974 22.57474245 7.88E-70 4.27E-69 147.7694972

FAM3B -3.031785575 5.697635586 -22.57170868 8.11E-70 4.39E-69 147.7418599

PLA2R1 1.307307374 1.548932709 22.56978021 8.25E-70 4.47E-69 147.7242914

OSBPL3 1.665966413 2.952601991 22.56785864 8.39E-70 4.55E-69 147.7067855

BCL11A -1.422347035 1.838128169 -22.56728492 8.44E-70 4.57E-69 147.7015587

TMEM47 1.797792637 3.364238865 22.55855792 9.14E-70 4.95E-69 147.6220498

RAD51D -0.765674087 2.206410899 -22.55499881 9.44E-70 5.11E-69 147.5896221

ATXN7L2 -0.95763792 2.016282718 -22.55013592 9.86E-70 5.34E-69 147.5453139

EPS15 0.993302812 3.967768118 22.54647117 1.02E-69 5.52E-69 147.5119214

FMNL3 1.21691498 2.5512389 22.54136856 1.07E-69 5.78E-69 147.4654255

CAMK2D 1.094893283 3.619144634 22.53023383 1.18E-69 6.39E-69 147.3639568

TGFBRAP1 0.788918921 3.326791144 22.52589151 1.23E-69 6.65E-69 147.3243834

ZNF367 1.033802562 1.305658849 22.52548984 1.23E-69 6.67E-69 147.3207227

HOXB2 1.87684128 3.128801722 22.51825879 1.32E-69 7.12E-69 147.2548197

CXCL10 2.957700245 2.160403083 22.51190187 1.40E-69 7.54E-69 147.19688

HACD4 1.090735923 1.186288576 22.50840833 1.44E-69 7.79E-69 147.1650369

PTGIS 2.838492262 3.072438779 22.50429974 1.50E-69 8.08E-69 147.1275867

LEMD1 2.576653531 1.562490628 22.50425001 1.50E-69 8.08E-69 147.1271334

RPUSD4 -1.153215957 4.185463739 -22.50134397 1.54E-69 8.30E-69 147.1006437

SWAP70 1.273138575 3.728184753 22.49987069 1.56E-69 8.41E-69 147.0872139

LCAT -1.511421238 3.569314894 -22.4979243 1.59E-69 8.56E-69 147.0694712

ZNF446 -1.064850382 2.844721861 -22.49519636 1.63E-69 8.77E-69 147.0446036

FBXO45 0.810227036 2.510060615 22.49141133 1.68E-69 9.07E-69 147.0100988

KDM4A 0.933239441 3.666492133 22.49100366 1.69E-69 9.10E-69 147.0063824

TCTN1 -1.393012801 3.487525831 -22.49084628 1.69E-69 9.12E-69 147.0049477

RNF24 1.328462657 2.831643091 22.49014428 1.70E-69 9.17E-69 146.998548

CENPH 1.148866629 1.904569816 22.48429203 1.80E-69 9.67E-69 146.9451954

RAD51AP1 1.3996049 1.560326312 22.4821283 1.83E-69 9.86E-69 146.9254688

TUBB4B 1.916665054 7.157498577 22.48007153 1.87E-69 1.00E-68 146.906717

CD300C 1.439148134 1.321715956 22.47690125 1.92E-69 1.03E-68 146.8778127

PRR5L 1.534642504 2.117035593 22.47604547 1.94E-69 1.04E-68 146.8700102

PLK1 1.648247935 2.315478988 22.46703482 2.10E-69 1.13E-68 146.7878527

PPL 2.017252374 4.303158706 22.46677356 2.11E-69 1.13E-68 146.7854705

RPL39L 1.96324494 2.515114857 22.46527764 2.14E-69 1.15E-68 146.7718303

BCL9 1.013636265 3.099019762 22.45717304 2.30E-69 1.24E-68 146.6979276

PGAP2 -1.287829649 4.296773001 -22.45562733 2.33E-69 1.25E-68 146.6838322

MAP1B 1.883747967 2.73267699 22.44509353 2.57E-69 1.38E-68 146.5877697

SLC37A4 -1.798888446 4.949885303 -22.44388433 2.60E-69 1.39E-68 146.5767419

RBM26 -1.010630834 3.960285775 -22.44056235 2.68E-69 1.44E-68 146.5464451

CD1C 2.308849259 1.600761858 22.43823239 2.73E-69 1.47E-68 146.5251952

DDX19B -1.069124248 3.695111686 -22.43484007 2.82E-69 1.51E-68 146.4942555

IFT52 0.951101567 4.180599624 22.43273621 2.88E-69 1.54E-68 146.4750667

RIN3 1.244527057 2.559159933 22.43064483 2.93E-69 1.57E-68 146.4559914

RBM4B -1.043725607 3.477934171 -22.4285912 2.99E-69 1.60E-68 146.4372601

TNFSF13B 1.618007611 2.241950531 22.42637611 3.05E-69 1.63E-68 146.4170558

SAMD9L 1.677890836 2.693105809 22.42202575 3.17E-69 1.70E-68 146.3773741

ZNF432 -1.32978758 3.151680058 -22.41931624 3.25E-69 1.74E-68 146.3526586

AQP5 3.957914242 3.290929007 22.41929352 3.25E-69 1.74E-68 146.3524514

CEP78 -0.747520942 1.863005709 -22.41609046 3.35E-69 1.79E-68 146.3232332

RSRP1 -1.885889312 5.105264685 -22.41596356 3.35E-69 1.79E-68 146.3220756

ROR2 1.66667769 2.186199275 22.40871878 3.58E-69 1.91E-68 146.2559859

MIB1 1.105026276 3.241498854 22.40276901 3.78E-69 2.02E-68 146.2017068

IQCK -1.087689266 2.698426435 -22.40032395 3.86E-69 2.06E-68 146.1793999

NAPSA -1.008247349 1.228526027 -22.39608237 4.02E-69 2.15E-68 146.1407021

BEND7 -1.308667272 3.038968518 -22.39537749 4.04E-69 2.16E-68 146.1342709

NECTIN2 1.463805419 5.965893094 22.38689091 4.37E-69 2.33E-68 146.0568393

TAOK1 0.932542362 2.976523709 22.38541402 4.43E-69 2.36E-68 146.0433636

BRAP 0.833799238 3.12592419 22.38315479 4.52E-69 2.41E-68 146.0227492

PRKACB 1.620577011 3.708124107 22.38023308 4.64E-69 2.48E-68 145.9960893

NR5A2 -2.532150747 3.858770053 -22.37837885 4.72E-69 2.52E-68 145.9791697

ATP6V0A2 -0.901681147 2.534646598 -22.37638184 4.81E-69 2.56E-68 145.9609468

ARNTL -1.406264781 2.929879253 -22.37340443 4.94E-69 2.63E-68 145.9337772

ICA1 -1.714230274 5.331293882 -22.37214663 5.00E-69 2.66E-68 145.9222993

PLBD2 1.244197677 4.741559718 22.37021317 5.08E-69 2.71E-68 145.9046554

RNPS1 -1.247177314 5.438963789 -22.36923489 5.13E-69 2.73E-68 145.895728

MT-CO1 3.152213058 13.87212754 22.36694336 5.24E-69 2.79E-68 145.874816

WDR11 -1.024773406 4.033095138 -22.36687049 5.24E-69 2.79E-68 145.874151

PWWP3A -1.232110776 3.883389371 -22.36381188 5.39E-69 2.87E-68 145.8462382

CDC25C 1.216182659 0.840690325 22.36358461 5.40E-69 2.87E-68 145.8441642

IL10RB 1.162844441 4.895605153 22.36353052 5.40E-69 2.87E-68 145.8436705

AL136295.5 -0.514061843 0.580529014 -22.3601308 5.57E-69 2.96E-68 145.8126438

CEL -7.88383191 8.926239524 -22.35547733 5.82E-69 3.09E-68 145.7701737

PARP2 -1.157649373 3.811102713 -22.35362284 5.92E-69 3.14E-68 145.7532481

TWIST1 2.232902622 1.809380196 22.35358484 5.92E-69 3.14E-68 145.7529013

TMEM131L -1.698267153 3.573628057 -22.35328762 5.93E-69 3.15E-68 145.7501886

PAGR1 -1.188714347 3.705356367 -22.34848114 6.20E-69 3.29E-68 145.7063195

FLACC1 -0.575003376 0.575256135 -22.34592449 6.35E-69 3.37E-68 145.6829841

ERCC6 -0.717958803 1.668208847 -22.33347634 7.11E-69 3.77E-68 145.5693583

ZNF408 0.980406944 3.106201831 22.32918264 7.39E-69 3.92E-68 145.530163

ITPRIPL2 1.355402599 3.609088509 22.32256418 7.85E-69 4.17E-68 145.4697433

ANOS1 1.504325248 1.115472936 22.32117568 7.95E-69 4.22E-68 145.4570673

TSNARE1 -1.365005805 3.888569243 -22.3179506 8.19E-69 4.34E-68 145.4276241

MRPS5 -1.155419238 4.50026093 -22.31346602 8.53E-69 4.52E-68 145.3866811

MTHFSD -0.954940823 3.112995672 -22.30870222 8.91E-69 4.72E-68 145.3431871

CXCL9 3.105681684 2.097160663 22.30613499 9.12E-69 4.83E-68 145.3197474

SETDB1 -1.013140439 3.991261198 -22.3051882 9.20E-69 4.87E-68 145.3111028

CLTB 1.946061638 6.678238501 22.3040179 9.30E-69 4.92E-68 145.3004172

RRM1 1.062170305 4.173926289 22.29646221 9.97E-69 5.27E-68 145.231427

PDLIM2 -1.492540111 3.27763176 -22.29593041 1.00E-68 5.30E-68 145.226571

UBE2Q2 1.032266544 4.169762471 22.28802335 1.08E-68 5.69E-68 145.1543676

GTF2E1 0.785494375 2.608521289 22.28754733 1.08E-68 5.72E-68 145.1500206

SEC31A -1.545530672 6.458261169 -22.28196728 1.14E-68 6.01E-68 145.0990632

FKBPL 1.010757183 2.933785448 22.27930885 1.17E-68 6.16E-68 145.0747855

ZBTB7C 1.665791861 1.598324859 22.27926236 1.17E-68 6.16E-68 145.0743609

PIP4K2B 0.981485852 4.279984242 22.27821729 1.18E-68 6.22E-68 145.0648167

GAS7 1.705556086 2.734249053 22.27617353 1.20E-68 6.33E-68 145.0461518

AP4E1 0.68652067 1.882611619 22.27453597 1.22E-68 6.43E-68 145.0311964

TIFA -1.445914588 3.374250559 -22.26958746 1.27E-68 6.72E-68 144.9860015

C11orf58 -1.307746245 5.370717862 -22.26347659 1.35E-68 7.11E-68 144.9301884

FHIT -1.579243783 2.781335851 -22.26343747 1.35E-68 7.11E-68 144.9298311

SOX6 -1.078574395 1.693324079 -22.26226633 1.36E-68 7.18E-68 144.9191343

TBC1D25 0.814722114 3.189358159 22.26189094 1.37E-68 7.21E-68 144.9157055

ZFC3H1 -1.223670518 3.936521687 -22.25879473 1.41E-68 7.41E-68 144.887425

AIP 1.173548273 5.816295292 22.25772637 1.42E-68 7.48E-68 144.8776666

CCDC107 -1.885356471 4.466837723 -22.25757321 1.42E-68 7.49E-68 144.8762676

SEPTIN6 1.487819265 3.137703928 22.253943 1.47E-68 7.74E-68 144.8431084

KIF1A -2.852111574 3.496164771 -22.24321718 1.62E-68 8.53E-68 144.7451305

COMMD4 -1.320013311 4.68692787 -22.24272574 1.63E-68 8.57E-68 144.7406412

ZNF830 0.900827094 3.629266422 22.24215613 1.64E-68 8.61E-68 144.7354376

BRWD1 -0.936995989 3.196569773 -22.23791385 1.70E-68 8.95E-68 144.6966827

IGFL2 2.787231423 1.576359359 22.23574661 1.73E-68 9.13E-68 144.6768836

EXO1 1.15696884 0.893165833 22.23356086 1.77E-68 9.31E-68 144.656915

ANGPT2 1.416810218 1.432480678 22.23066782 1.82E-68 9.55E-68 144.6304842

PHYHIP 1.312245591 1.977989226 22.22628393 1.89E-68 9.94E-68 144.5904318

KCNV1 -0.332786957 0.206249285 -22.22352006 1.94E-68 1.02E-67 144.5651797

USP36 -1.221951297 4.391735178 -22.20955457 2.20E-68 1.16E-67 144.4375749

EIF5A2 1.164027539 1.949442982 22.20730687 2.25E-68 1.18E-67 144.417036

RNF122 1.458816626 2.985335601 22.20281683 2.34E-68 1.23E-67 144.3760061

CHP1 1.4218497 5.667440172 22.20215937 2.36E-68 1.24E-67 144.3699981

ALDH1L2 -2.077388767 3.293494222 -22.1966932 2.48E-68 1.30E-67 144.3200459

SZRD1 1.064363389 5.24531352 22.19624776 2.49E-68 1.31E-67 144.3159752

AP3B2 -1.725343368 1.68041072 -22.19468966 2.52E-68 1.32E-67 144.3017362

LRRC15 2.771902256 1.556756087 22.19356153 2.55E-68 1.34E-67 144.2914265

PHGDH -2.34316674 4.724592354 -22.18542393 2.75E-68 1.44E-67 144.2170558

CHRNB1 -1.429353804 3.094060992 -22.18443592 2.77E-68 1.45E-67 144.2080258

IHH 3.044549271 1.736431909 22.1768991 2.97E-68 1.56E-67 144.1391409

TNFSF4 1.560131632 1.484548168 22.16913459 3.19E-68 1.67E-67 144.0681705

MTX1 -1.135671276 3.984378188 -22.16675182 3.26E-68 1.71E-67 144.0463902

COMMD8 0.974820756 3.547837878 22.1635693 3.35E-68 1.76E-67 144.017299

FAM83H 1.777002797 4.494763664 22.16321243 3.37E-68 1.76E-67 144.0140369

ACP6 -1.486167055 3.160408121 -22.16229695 3.39E-68 1.78E-67 144.0056684

COL27A1 -1.675184263 3.840855174 -22.15873956 3.51E-68 1.83E-67 143.9731493

PDLIM4 2.108689815 3.612217018 22.15797934 3.53E-68 1.85E-67 143.9661998

SLC46A3 1.322824478 3.600311917 22.15614511 3.59E-68 1.88E-67 143.9494321

ZNF593 -1.459051275 3.028140033 -22.14946039 3.82E-68 1.99E-67 143.8883215

GSG1 -1.771522534 1.045954501 -22.14022646 4.15E-68 2.17E-67 143.803901

ABRAXAS1 -0.958597336 1.897736134 -22.1393322 4.19E-68 2.19E-67 143.7957251

PAF1 1.248716272 5.320062161 22.13658865 4.29E-68 2.24E-67 143.770641

STARD10 -2.05450251 6.843912812 -22.13404973 4.39E-68 2.29E-67 143.7474273

ANKRD20A3 -0.698193919 0.340622024 -22.12943308 4.58E-68 2.39E-67 143.7052155

AMMECR1 0.932098393 2.148547939 22.12193694 4.91E-68 2.56E-67 143.6366723

APIP -1.247827492 3.550511526 -22.11994772 5.00E-68 2.61E-67 143.6184826

SEPTIN10 1.220331444 4.198186755 22.11743235 5.11E-68 2.67E-67 143.5954812

PRSS45P -1.028382869 0.899968449 -22.10849847 5.55E-68 2.89E-67 143.5137831

MPEG1 2.291092808 2.580687421 22.10560103 5.70E-68 2.97E-67 143.4872856

ZNF146 1.078062449 4.79605925 22.10409064 5.78E-68 3.01E-67 143.4734726

SLC13A3 -1.358620011 1.80521298 -22.10141215 5.92E-68 3.08E-67 143.4489765

ESF1 0.961245817 3.136450646 22.10110842 5.94E-68 3.09E-67 143.4461987

ANAPC11 -1.476148528 5.577756716 -22.10058228 5.97E-68 3.11E-67 143.4413868

TRAPPC4 -1.232517001 4.868208251 -22.09834938 6.09E-68 3.17E-67 143.4209654

KMT2D -1.240673226 3.982633641 -22.0983082 6.09E-68 3.17E-67 143.4205888

RAB38 1.517704677 2.032572129 22.097582 6.13E-68 3.19E-67 143.4139471

SLC7A4 1.874796783 1.283187603 22.09721321 6.15E-68 3.20E-67 143.4105741

IRF6 1.692158692 4.711393527 22.09525369 6.26E-68 3.26E-67 143.3926525

DELE1 -1.144548153 4.911454761 -22.09373503 6.35E-68 3.30E-67 143.3787627

ARHGAP17 -1.036267159 4.182768429 -22.09372991 6.35E-68 3.30E-67 143.3787158

FAM24B -1.331870355 2.721151811 -22.09279032 6.41E-68 3.33E-67 143.3701222

SQLE 1.871721619 3.994508508 22.08889385 6.64E-68 3.45E-67 143.3344838

EPHA4 1.627640031 1.879301827 22.08571729 6.83E-68 3.55E-67 143.305429

FBXL7 1.412663303 2.124535749 22.08112803 7.13E-68 3.70E-67 143.2634516

DMAC2L -1.087031987 2.808023087 -22.07818779 7.32E-68 3.80E-67 143.2365569

CRNKL1 0.891606692 3.60991333 22.0779852 7.33E-68 3.81E-67 143.2347037

XRCC4 1.534652175 2.499890087 22.07569356 7.49E-68 3.89E-67 143.2137413

SPATA6L -1.334848962 2.028686924 -22.07078995 7.83E-68 4.06E-67 143.1688849

HIST3H2A 2.036033562 3.115109434 22.06996369 7.89E-68 4.09E-67 143.1613265

MAPRE2 1.151166772 3.788311994 22.06200063 8.49E-68 4.40E-67 143.0884793

TMEM94 -1.212063494 4.563270432 -22.05954444 8.68E-68 4.50E-67 143.0660089

C10orf71 -0.723911784 0.387954843 -22.05463643 9.08E-68 4.70E-67 143.0211066

MICB 1.433412883 1.638981698 22.05180555 9.32E-68 4.83E-67 142.9952067

SPACA3 -2.072493036 1.460784746 -22.05129915 9.36E-68 4.85E-67 142.9905736

GRAMD1B 1.755234401 2.038692193 22.04738549 9.70E-68 5.02E-67 142.9547663

C12orf29 -0.901274669 2.654825455 -22.04655171 9.78E-68 5.06E-67 142.9471376

AKT1 -1.261780595 5.41907702 -22.0429876 1.01E-67 5.23E-67 142.9145272

MOCS2 -1.146554792 3.903003331 -22.03932628 1.04E-67 5.40E-67 142.8810264

THEM5 1.302075444 1.027735028 22.03901259 1.05E-67 5.42E-67 142.8781562

CSNK1D -1.379318367 5.657744313 -22.03334066 1.10E-67 5.70E-67 142.8262564

FOXN2 0.981899796 2.615337882 22.03004978 1.14E-67 5.88E-67 142.796143

RPL15 -1.889598562 8.655603941 -22.01689022 1.28E-67 6.63E-67 142.6757175

LY6E 2.881942727 6.851902393 22.01190547 1.34E-67 6.93E-67 142.630098

OCIAD2 1.659006458 5.707913809 22.0093188 1.37E-67 7.10E-67 142.6064246

ANXA7 1.250846458 6.043021399 22.00909695 1.38E-67 7.11E-67 142.6043942

RECQL5 -1.296527612 3.722727362 -22.00877679 1.38E-67 7.13E-67 142.601464

TNFSF15 1.131037017 0.848143981 22.00174643 1.47E-67 7.60E-67 142.5371188

TIPARP 1.367910479 3.606564332 22.0012178 1.48E-67 7.64E-67 142.5322804

DOLPP1 1.087136368 3.787771662 21.99814308 1.52E-67 7.85E-67 142.5041378

PPP6R1 1.099719896 4.689898182 21.99399998 1.58E-67 8.15E-67 142.4662155

CARD6 1.86948399 2.779370094 21.98292147 1.75E-67 9.02E-67 142.3648066

ARHGAP1 1.156205299 5.463561358 21.97983883 1.80E-67 9.28E-67 142.3365876

METTL1 -1.366520958 4.421344194 -21.9791127 1.81E-67 9.34E-67 142.3299404

GRK6 0.977691456 4.007544003 21.97846917 1.82E-67 9.39E-67 142.3240494

REV1 -0.987788204 3.700010112 -21.97670328 1.85E-67 9.54E-67 142.3078836

FLNC 2.307680362 2.019638295 21.97576783 1.87E-67 9.62E-67 142.29932

TAF4 -0.858731091 2.802296219 -21.97460663 1.89E-67 9.72E-67 142.2886897

CDK19 0.861967153 2.549588868 21.97427363 1.89E-67 9.75E-67 142.2856412

TCIRG1 -2.375219123 7.097528935 -21.97268867 1.92E-67 9.88E-67 142.2711313

BAMBI 1.607606533 3.158087785 21.97108508 1.95E-67 1.00E-66 142.2564507

MPZL1 1.239315363 5.172257492 21.97027949 1.96E-67 1.01E-66 142.2490756

AK1 -1.531033551 4.115804312 -21.96886507 1.99E-67 1.02E-66 142.2361266

TPM4 1.837694748 6.845090113 21.96753586 2.01E-67 1.04E-66 142.2239576

NME9 -0.775759081 0.919723543 -21.95869761 2.18E-67 1.12E-66 142.1430394

ORC1 1.051432694 1.028981901 21.95308322 2.30E-67 1.18E-66 142.0916343

GFI1 -1.631302458 2.267992848 -21.94617911 2.45E-67 1.26E-66 142.0284174

CYTH3 1.301305602 3.580458989 21.93148075 2.80E-67 1.44E-66 141.8938223

RPL41 -2.301795405 10.09995971 -21.93136566 2.80E-67 1.44E-66 141.8927684

SLC12A2 1.809637945 3.488496367 21.92546249 2.96E-67 1.52E-66 141.8387078

FBXO11 -1.158248397 3.626658943 -21.92266916 3.04E-67 1.56E-66 141.8131259

PPCDC -1.160532916 3.283318491 -21.91605164 3.23E-67 1.65E-66 141.7525192

PPIA -1.721802765 7.973883432 -21.91072898 3.39E-67 1.74E-66 141.7037694

OXSR1 0.973847271 3.897603044 21.91017522 3.41E-67 1.75E-66 141.6986974

PLA2G15 1.059437377 3.488901834 21.90628042 3.53E-67 1.81E-66 141.6630238

GSTT2B -2.20343572 2.955802185 -21.90466068 3.58E-67 1.83E-66 141.6481878

SPAG16 -1.157978732 3.123291632 -21.90097552 3.70E-67 1.90E-66 141.614433

C6orf201 -0.623485544 0.477318097 -21.90043567 3.72E-67 1.91E-66 141.609488

FREM1 -1.902071009 2.200317158 -21.89692553 3.84E-67 1.97E-66 141.5773353

TMSB4X 2.449716446 10.73044655 21.89682089 3.85E-67 1.97E-66 141.5763768

AKAP12 2.221104857 3.456656463 21.89576697 3.89E-67 1.99E-66 141.5667228

ENO1 2.08194992 8.345439699 21.8954568 3.90E-67 1.99E-66 141.5638815

OIP5 1.261398251 1.115308532 21.89113284 4.05E-67 2.07E-66 141.5242726

TYROBP 2.473095654 5.675993011 21.88874092 4.14E-67 2.12E-66 141.5023613

PMP22 1.949352896 5.158203471 21.88806268 4.17E-67 2.13E-66 141.4961482

SFPQ -1.48763203 6.636072224 -21.88258858 4.38E-67 2.24E-66 141.4460006

CRYBG1 1.635789613 2.871089681 21.87798801 4.57E-67 2.34E-66 141.4038537

ADGRG6 1.93284355 2.539821941 21.87758585 4.59E-67 2.34E-66 141.4001694

RPL13 -2.384246015 9.932323583 -21.8775462 4.59E-67 2.34E-66 141.3998061

MSMO1 1.786239386 4.940624854 21.8758017 4.67E-67 2.38E-66 141.3838239

THOC5 -0.980608948 3.315130147 -21.87468487 4.71E-67 2.40E-66 141.373592

RBM12 0.898182648 3.926885201 21.87080101 4.88E-67 2.49E-66 141.338009

ABHD18 -0.983207187 2.670812293 -21.86541481 5.13E-67 2.62E-66 141.2886602

LRRC8C 1.067610894 1.576019882 21.86088461 5.35E-67 2.73E-66 141.2471527

TARDBP -1.231518391 5.77788692 -21.860228 5.38E-67 2.74E-66 141.2411364

CELA2B -6.778100472 6.516519574 -21.85427697 5.68E-67 2.89E-66 141.1866082

H2AFJ -1.753379491 6.173353316 -21.85426643 5.68E-67 2.89E-66 141.1865117

TOPORS 0.895858938 3.084996567 21.8483258 6.00E-67 3.06E-66 141.1320763

KCTD15 -1.407798534 3.861596339 -21.84068555 6.43E-67 3.28E-66 141.0620633

TRMT1L 0.814557773 2.944293678 21.83729809 6.64E-67 3.38E-66 141.0310204

TEX11 -2.317190708 1.980144738 -21.83701462 6.65E-67 3.39E-66 141.0284226

UQCR10 1.249711225 6.346605918 21.83656127 6.68E-67 3.40E-66 141.024268

RPL23 -1.900044413 8.632174245 -21.83489558 6.79E-67 3.45E-66 141.0090031

SGSM2 -1.621356522 4.3785851 -21.83212594 6.96E-67 3.54E-66 140.9836209

DUOXA2 4.024349273 2.548786574 21.83058287 7.06E-67 3.59E-66 140.9694793

CCNF 1.007499078 1.559463952 21.82547165 7.40E-67 3.76E-66 140.9226359

NDNF 1.88441856 1.353188376 21.82457222 7.46E-67 3.79E-66 140.9143925

ZNF605 -0.763563097 2.178703188 -21.82123417 7.69E-67 3.91E-66 140.8837988

ANKS4B 2.111026344 1.384114138 21.8191705 7.84E-67 3.98E-66 140.8648845

CAMK1 -1.041033995 2.641907211 -21.81387796 8.23E-67 4.18E-66 140.8163751

TM9SF4 1.024788282 5.014814074 21.81155065 8.40E-67 4.27E-66 140.7950433

GPR35 2.22843274 2.449615031 21.80470597 8.95E-67 4.54E-66 140.7323037

TMED8 1.133255757 2.716672607 21.8033737 9.06E-67 4.60E-66 140.7200915

ST5 -1.257873128 4.379839996 -21.80315487 9.08E-67 4.60E-66 140.7180856

REX1BD -1.889894872 4.987391084 -21.80177269 9.19E-67 4.66E-66 140.7054158

AAAS -1.215518583 4.869394531 -21.79359818 9.91E-67 5.02E-66 140.6304809

ANKRD46 -1.076132764 3.077326823 -21.7912554 1.01E-66 5.13E-66 140.609004

PTGS2 2.638143672 1.885179303 21.79057174 1.02E-66 5.16E-66 140.6027367

PLEK 2.123533236 2.372182056 21.78890606 1.03E-66 5.24E-66 140.5874667

TCP11L2 -1.124020993 2.026579609 -21.78639041 1.06E-66 5.36E-66 140.5644043

NUP88 -1.128506718 4.253951007 -21.78586642 1.06E-66 5.38E-66 140.5596005

SHANK2 -1.208453961 2.124383841 -21.7853492 1.07E-66 5.41E-66 140.5548588

RPL22 -1.647869904 7.558904194 -21.78509031 1.07E-66 5.42E-66 140.5524853

RELL1 1.186059702 2.016686695 21.78397109 1.08E-66 5.47E-66 140.5422246

VTI1B -1.195425633 5.313237206 -21.78383893 1.08E-66 5.48E-66 140.5410129

WDR47 0.888914112 2.535518834 21.77642647 1.16E-66 5.86E-66 140.4730549

OAZ3 -0.765679866 1.131081714 -21.7754154 1.17E-66 5.92E-66 140.463785

MTM1 0.910871146 2.666802497 21.76834376 1.25E-66 6.31E-66 140.3989477

DHX36 -1.097282508 3.859571719 -21.76833783 1.25E-66 6.31E-66 140.3988933

CHRAC1 1.029809883 4.143541683 21.76732042 1.26E-66 6.37E-66 140.3895648

RPS19BP1 -1.435961776 5.640714209 -21.7596613 1.35E-66 6.83E-66 140.3193367

DUSP14 1.226815854 3.206908311 21.75686676 1.39E-66 7.00E-66 140.293712

KATNBL1 -0.988066363 3.31864265 -21.75079706 1.47E-66 7.40E-66 140.2380537

SLC18B1 1.128579368 3.654628829 21.74523358 1.54E-66 7.79E-66 140.1870352

KHK -1.538607628 2.787932845 -21.74387129 1.56E-66 7.88E-66 140.1745424

REG1B -8.079052197 8.522934059 -21.74179765 1.59E-66 8.03E-66 140.1555259

STK38L 1.299560469 3.913364507 21.74152571 1.60E-66 8.05E-66 140.1530321

KAZN -0.94367298 1.933109922 -21.73901687 1.63E-66 8.24E-66 140.130024

MCRIP2 -1.720790611 4.394406819 -21.73578196 1.68E-66 8.48E-66 140.1003568

ATG5 0.834505632 3.811602709 21.73554471 1.69E-66 8.50E-66 140.098181

DCTN3 -1.357808206 5.231024527 -21.73461632 1.70E-66 8.57E-66 140.0896666

VAMP5 1.468809766 5.311303999 21.73446959 1.70E-66 8.58E-66 140.0883209

PTPN11 1.192914696 4.327215187 21.73062931 1.76E-66 8.88E-66 140.0531006

NLGN3 0.94578155 1.000070856 21.72977933 1.78E-66 8.95E-66 140.045305

MRVI1 1.759483851 2.723103363 21.72601789 1.84E-66 9.26E-66 140.0108067

CCR1 1.816737065 1.966009746 21.72579433 1.84E-66 9.28E-66 140.0087563

G6PD 1.10954072 4.266429609 21.72212242 1.91E-66 9.59E-66 139.975078

SPPL2B -1.879156786 5.22977104 -21.72084269 1.93E-66 9.70E-66 139.9633403

B4GALT1 1.802475075 5.576575297 21.71550653 2.03E-66 1.02E-65 139.9143957

ERN1 -1.67224456 3.906370139 -21.71542755 2.03E-66 1.02E-65 139.9136713

SERINC1 1.652916495 6.336866352 21.71489351 2.04E-66 1.02E-65 139.9087728

MAP3K12 -1.252510687 2.888075322 -21.70910453 2.15E-66 1.08E-65 139.8556725

ATP5F1D -1.883844644 6.640128611 -21.70774535 2.18E-66 1.09E-65 139.8432049

ZBTB34 0.667146212 2.214085403 21.70219895 2.29E-66 1.15E-65 139.792327

CCDC90B -1.021165776 4.022184069 -21.69542534 2.44E-66 1.22E-65 139.7301891

XRN2 1.187861094 5.208207344 21.69520744 2.44E-66 1.23E-65 139.7281902

HBA1 -3.81208819 2.217025169 -21.69349048 2.48E-66 1.24E-65 139.7124391

ZBTB20 -0.73844023 0.814511399 -21.6930229 2.49E-66 1.25E-65 139.7081495

TIMP2 2.094181528 6.893125172 21.6849843 2.68E-66 1.34E-65 139.6344019

CLASP2 -0.970636532 3.015539753 -21.67148435 3.03E-66 1.52E-65 139.5105413

VAT1 1.423988755 6.379490776 21.66782632 3.14E-66 1.57E-65 139.4769771

NPY5R -0.630722754 0.45289227 -21.66588725 3.19E-66 1.60E-65 139.4591849

CSF2RB 1.925262564 2.048099435 21.66318309 3.27E-66 1.64E-65 139.434372

AGBL2 -0.898635385 1.168398746 -21.66206523 3.31E-66 1.66E-65 139.4241147

NEURL1B 1.443938781 3.167306321 21.66031372 3.36E-66 1.68E-65 139.4080428

CDR2 1.04303854 4.041623119 21.65749116 3.45E-66 1.73E-65 139.3821425

MUSTN1 -1.662271475 0.990361097 -21.65721127 3.46E-66 1.73E-65 139.3795741

FKBP7 1.28301391 2.88580485 21.65601 3.50E-66 1.75E-65 139.3685509

RNF6 0.969427488 3.76404956 21.65092922 3.66E-66 1.83E-65 139.321927

CLDN23 2.182845069 3.150526456 21.64318553 3.93E-66 1.97E-65 139.2508635

RRP8 -0.931067605 3.191133485 -21.64315012 3.94E-66 1.97E-65 139.2505385

PNMA2 1.991366506 2.30335505 21.64253616 3.96E-66 1.98E-65 139.2449041

IL17D -1.020171137 1.746891628 -21.63607835 4.20E-66 2.10E-65 139.1856378

NECAB3 -1.415693141 3.836382463 -21.62909229 4.48E-66 2.24E-65 139.1215205

DOCK11 1.501757386 2.06957864 21.62492778 4.65E-66 2.32E-65 139.0832975

IER3IP1 1.065834966 3.857006656 21.61762799 4.97E-66 2.48E-65 139.0162954

SPI1 2.058932746 3.718911202 21.61230474 5.22E-66 2.61E-65 138.9674331

TP53BP2 0.976905982 3.756952974 21.61100803 5.29E-66 2.64E-65 138.9555302

CD5 1.806502346 1.516108459 21.60891485 5.39E-66 2.69E-65 138.9363161

HIST4H4 -1.294167625 1.61422359 -21.60319952 5.68E-66 2.83E-65 138.8838515

MTMR11 2.228702084 4.024159233 21.59881714 5.91E-66 2.95E-65 138.8436215

FCER1A 2.217356502 1.609176582 21.59475546 6.14E-66 3.06E-65 138.8063344

THAP11 0.930539674 4.40434825 21.58948727 6.44E-66 3.21E-65 138.7579695

FAM3D 3.798471205 2.771367612 21.58622835 6.64E-66 3.31E-65 138.72805

JAZF1 1.135011439 3.100588743 21.58490209 6.72E-66 3.35E-65 138.7158737

CCDC96 1.243039642 1.492538232 21.57860718 7.12E-66 3.54E-65 138.6580789

ASXL1 -1.101928048 4.683780314 -21.56965419 7.73E-66 3.85E-65 138.5758753

CXorf21 1.403035947 1.139077745 21.56959595 7.73E-66 3.85E-65 138.5753406

FBXO33 0.767006084 2.731777029 21.56507623 8.06E-66 4.01E-65 138.5338399

EFS 1.354622469 2.208140846 21.56468098 8.09E-66 4.02E-65 138.5302106

AGR3 3.197359564 3.903978133 21.56320072 8.20E-66 4.08E-65 138.5166184

P4HB -1.247560866 9.378318031 -21.56152138 8.32E-66 4.14E-65 138.5011979

SEMA3A 1.317189556 0.972230377 21.56132433 8.34E-66 4.15E-65 138.4993885

TTC30B 0.85380991 1.90294072 21.56018479 8.43E-66 4.19E-65 138.4889246

LIPA 1.469632419 4.707224015 21.5553016 8.81E-66 4.38E-65 138.4440835

CNNM4 1.244743215 3.39778518 21.55332392 8.97E-66 4.46E-65 138.4259225

MCC 1.044480446 1.963420348 21.55267071 9.03E-66 4.48E-65 138.419924

DDX42 -1.313445271 5.655274398 -21.55132931 9.14E-66 4.54E-65 138.4076058

LEMD2 -1.172791683 4.768956288 -21.54605335 9.59E-66 4.76E-65 138.359155

PPP2R5C -1.131916181 4.696865009 -21.54416866 9.76E-66 4.84E-65 138.3418468

LPCAT2 1.52120937 2.806485785 21.54376094 9.80E-66 4.86E-65 138.3381025

ZNF768 1.088019947 4.872969192 21.53901018 1.02E-65 5.08E-65 138.2944725

KLHDC3 1.155031593 5.842871391 21.53881953 1.03E-65 5.08E-65 138.2927216

RORA -1.210459358 2.526023756 -21.53564809 1.06E-65 5.23E-65 138.2635948

ZDHHC17 -1.111146176 3.383332677 -21.53515832 1.06E-65 5.25E-65 138.2590967

TBC1D15 -1.111846858 4.233172524 -21.53485022 1.06E-65 5.27E-65 138.2562671

KMT5A -1.152019152 4.25963977 -21.53401069 1.07E-65 5.31E-65 138.2485566

GMPPA -1.332235904 5.063514149 -21.53354325 1.08E-65 5.33E-65 138.2442636

TAB3 -0.91501076 3.002684554 -21.53354109 1.08E-65 5.33E-65 138.2442437

ZNF362 1.027206759 4.012683912 21.53015452 1.11E-65 5.49E-65 138.2131401

SEC22C -1.121568966 3.967581704 -21.52810427 1.13E-65 5.60E-65 138.1943094

HINT2 -1.552618414 4.926338422 -21.52413117 1.17E-65 5.80E-65 138.1578174

ZMAT3 1.074976989 2.623064048 21.52403009 1.17E-65 5.81E-65 138.156889

AP1S3 1.569974081 2.231686332 21.52160417 1.20E-65 5.94E-65 138.134607

SPDYE8P -0.183806299 0.089231539 -21.52005066 1.22E-65 6.02E-65 138.1203378

LYSMD1 0.769720296 2.736716306 21.51738362 1.25E-65 6.17E-65 138.0958404

THOP1 -1.2953876 3.79240827 -21.51366413 1.29E-65 6.38E-65 138.0616752

ELOF1 -1.246772166 5.189336463 -21.50929351 1.34E-65 6.64E-65 138.021528

SYNJ2BP 0.855448337 3.312830585 21.5075733 1.37E-65 6.75E-65 138.0057264

DPAGT1 -1.272670222 4.824077372 -21.50532234 1.39E-65 6.88E-65 137.985049

RPIA 1.004975053 4.315775231 21.5017676 1.44E-65 7.11E-65 137.9523944

DAPL1 -1.970406762 2.277395319 -21.50157545 1.44E-65 7.12E-65 137.9506293

DDX4 -0.368729866 0.221330063 -21.50045888 1.46E-65 7.19E-65 137.9403721

ENOX1 1.197676378 1.406312106 21.50031805 1.46E-65 7.20E-65 137.9390784

CCDC116 -0.37307626 0.355276934 -21.49804541 1.49E-65 7.35E-65 137.9182008

HOGA1 -1.921427069 2.262881382 -21.49450157 1.54E-65 7.59E-65 137.8856447

PRPF3 -1.317299663 4.834760467 -21.49156833 1.58E-65 7.80E-65 137.8586975

FAM189A1 -1.329792766 1.702735943 -21.49004375 1.60E-65 7.91E-65 137.8446911

DHX29 0.839581885 3.727702914 21.48515779 1.68E-65 8.27E-65 137.7998029

TENM4 1.194932735 1.050967065 21.48228658 1.72E-65 8.49E-65 137.7734237

HACE1 -0.777331249 1.855677145 -21.47917495 1.77E-65 8.73E-65 137.7448351

YAP1 1.548224697 4.453654215 21.46956355 1.94E-65 9.53E-65 137.6565252

UVSSA -1.358105356 2.861557604 -21.46808047 1.96E-65 9.66E-65 137.6428981

CDC42EP5 2.272219084 4.768455287 21.46586794 2.00E-65 9.86E-65 137.6225683

LRRC40 0.913857872 2.696387178 21.46187016 2.08E-65 1.02E-64 137.5858338

GCAT -2.165647889 4.587646896 -21.45933218 2.13E-65 1.05E-64 137.5625126

ANKMY2 0.902572277 3.6911364 21.459184 2.13E-65 1.05E-64 137.561151

DNAH1 -1.268313414 2.238564125 -21.4580171 2.15E-65 1.06E-64 137.5504283

NFATC2 1.440663289 2.336653789 21.45745854 2.16E-65 1.06E-64 137.5452956

VASN 1.792709401 4.047343691 21.45695744 2.17E-65 1.07E-64 137.540691

UBALD2 1.551068611 5.789503854 21.45446123 2.22E-65 1.09E-64 137.5177528

B9D1 -1.573106162 3.350255224 -21.44816491 2.36E-65 1.16E-64 137.4598929

EEF1E1 -1.193448569 3.747995027 -21.44750978 2.37E-65 1.16E-64 137.4538723

CRCP 0.906077668 4.210966401 21.4468137 2.39E-65 1.17E-64 137.4474756

CFAP221 -1.664776341 2.548100783 -21.44422754 2.44E-65 1.20E-64 137.4237092

STAC3 1.097370617 1.931321544 21.43918678 2.56E-65 1.26E-64 137.3773841

EVPL 2.393926517 3.013203603 21.4381166 2.59E-65 1.27E-64 137.3675489

TMEM72 -1.488645215 1.219971244 -21.4379606 2.59E-65 1.27E-64 137.3661152

CYP17A1 -1.214846518 0.815347557 -21.4304804 2.77E-65 1.36E-64 137.297368

CD2AP 1.378183567 4.305775159 21.43039251 2.78E-65 1.36E-64 137.2965603

SP6 1.401273656 1.231950673 21.42669849 2.87E-65 1.41E-64 137.2626089

KLK7 4.034319291 2.340962058 21.42243062 2.99E-65 1.46E-64 137.2233822

ZNF568 -0.846190032 1.599713603 -21.41765015 3.12E-65 1.53E-64 137.1794428

CSPG4 1.562670476 2.283561159 21.41361063 3.24E-65 1.59E-64 137.1423127

LGALS3 2.605839061 7.319302844 21.41197451 3.29E-65 1.61E-64 137.1272737

MMP10 2.421860695 1.542735517 21.41119755 3.31E-65 1.62E-64 137.120132

RTKN2 1.24702564 0.888762652 21.41015234 3.34E-65 1.64E-64 137.1105244

SOWAHD 0.984123355 0.904093333 21.40771333 3.42E-65 1.67E-64 137.0881047

KDM8 -0.942328815 2.271675529 -21.40763065 3.42E-65 1.67E-64 137.0873447

RAB41 0.548279915 0.478786923 21.40658123 3.45E-65 1.69E-64 137.0776982

L3MBTL4 -0.992334469 1.604510014 -21.40364866 3.55E-65 1.73E-64 137.0507409

EXD3 -1.358073134 3.169834808 -21.39513778 3.84E-65 1.87E-64 136.9725029

CST6 3.593436833 2.51360942 21.39277276 3.92E-65 1.92E-64 136.9507611

EIF2AK3 -1.624285539 4.190816048 -21.39119363 3.98E-65 1.94E-64 136.936244

SLC39A8 -1.785156157 3.974324772 -21.38932036 4.05E-65 1.98E-64 136.9190225

TMEM123 1.583600973 6.091582444 21.38558144 4.19E-65 2.04E-64 136.8846489

RNF144B 1.199534519 2.547880404 21.37894212 4.45E-65 2.17E-64 136.8236086

SYPL1 1.317579591 6.037438515 21.37588298 4.58E-65 2.23E-64 136.7954827

PPIC 1.580087064 5.301457667 21.37297024 4.70E-65 2.29E-64 136.7687023

CCDC82 -1.06614581 3.553679524 -21.37198713 4.75E-65 2.31E-64 136.7596632

CAPZA1 1.247554926 5.565205244 21.36904236 4.88E-65 2.38E-64 136.7325876

PARN 0.876074597 4.36833029 21.36870572 4.89E-65 2.38E-64 136.7294923

CNDP1 -0.426991325 0.296547416 -21.36475584 5.07E-65 2.47E-64 136.6931743

TFF2 5.740112908 4.603211322 21.36474728 5.07E-65 2.47E-64 136.6930955

TMEM232 -0.739473161 0.674435589 -21.36364552 5.13E-65 2.50E-64 136.6829651

SLC25A26 -1.049555288 3.507940866 -21.36118703 5.24E-65 2.55E-64 136.6603593

ICAM1 2.134923913 4.054477139 21.35716028 5.44E-65 2.65E-64 136.6233326

DCDC1 -0.381872322 0.335481381 -21.35692774 5.45E-65 2.65E-64 136.6211943

HIBCH -1.328552666 3.734658387 -21.35666962 5.47E-65 2.66E-64 136.6188208

CHIC1 0.741734144 1.980200174 21.35246051 5.68E-65 2.76E-64 136.5801162

MNX1 -1.80873681 3.152684702 -21.35213207 5.70E-65 2.77E-64 136.577096

YIPF1 0.993101453 4.620451754 21.35193514 5.71E-65 2.77E-64 136.5752851

SLC7A5 2.44199049 3.59348922 21.34972349 5.83E-65 2.83E-64 136.5549474

ZNF397 -0.978918074 3.104317101 -21.34815032 5.91E-65 2.87E-64 136.5404809

OSGEP -1.185428074 4.36912221 -21.34322537 6.18E-65 3.00E-64 136.4951911

SDHAF4 1.00115512 3.236410371 21.34128975 6.30E-65 3.06E-64 136.4773907

RTN4IP1 0.918815574 2.36584661 21.34012132 6.36E-65 3.09E-64 136.4666455

RNF2 0.922842393 3.183258428 21.33868241 6.45E-65 3.13E-64 136.4534127

ANKRD37 -1.643539914 3.34136943 -21.33583327 6.62E-65 3.21E-64 136.4272106

RASSF3 1.470189813 4.141122448 21.33335041 6.77E-65 3.29E-64 136.4043765

ACACB -1.632350845 3.393413943 -21.33103732 6.92E-65 3.36E-64 136.3831035

IQCG -1.270988642 2.414382837 -21.33067513 6.94E-65 3.37E-64 136.3797725

NRCAM -2.349886284 3.448724057 -21.32886389 7.06E-65 3.42E-64 136.3631146

PRSS1 -8.30680681 10.97428258 -21.32806317 7.11E-65 3.45E-64 136.3557504

CCL20 3.224079926 2.738819031 21.32787795 7.12E-65 3.45E-64 136.3540469

CXorf56 0.886200463 3.753797971 21.32615616 7.23E-65 3.50E-64 136.3382113

KLHL12 0.868280319 4.103076396 21.32384332 7.39E-65 3.58E-64 136.3169396

CSDC2 -2.807608817 3.302809683 -21.32087361 7.59E-65 3.68E-64 136.2896259

ATR -0.877199279 2.983722347 -21.31993776 7.66E-65 3.71E-64 136.2810184

LRFN4 1.930768149 2.906021621 21.3174994 7.83E-65 3.79E-64 136.2585913

SLC16A2 1.256697587 3.138961752 21.31747114 7.84E-65 3.79E-64 136.2583314

TES 1.372034644 4.914574804 21.31399032 8.09E-65 3.91E-64 136.2263154

CLPS -8.754080303 10.04246887 -21.30750077 8.59E-65 4.15E-64 136.1666239

BIRC5 2.221796174 2.039997082 21.30496095 8.79E-65 4.25E-64 136.1432616

GSPT2 1.429179994 2.973496168 21.3048386 8.80E-65 4.25E-64 136.1421362

WBP11 1.039690141 4.451728169 21.30458079 8.82E-65 4.26E-64 136.1397648

PEX19 0.895357315 4.334819883 21.30394911 8.87E-65 4.29E-64 136.1339543

ZNF721 -0.985791803 3.057437268 -21.30389415 8.88E-65 4.29E-64 136.1334487

SLC25A13 0.893694631 3.744890015 21.30331698 8.93E-65 4.31E-64 136.1281396

C3AR1 2.144515767 2.911914376 21.30264947 8.98E-65 4.34E-64 136.1219995

CDKN1B 1.120897249 4.804745648 21.29798738 9.37E-65 4.52E-64 136.0791141

PRKCA 1.090219361 2.94807822 21.29787225 9.38E-65 4.53E-64 136.078055

POLG2 -1.040887882 3.188743668 -21.29555927 9.59E-65 4.62E-64 136.0567781

ZEB1 1.473741783 2.99068232 21.28167155 1.09E-64 5.25E-64 135.9290189

DCAF11 -1.331799697 5.417129125 -21.27862393 1.12E-64 5.40E-64 135.900981

MAP3K21 -1.432314286 3.21239343 -21.27536544 1.15E-64 5.56E-64 135.8710024

FAM177A1 -1.248017559 4.591767677 -21.2740951 1.17E-64 5.63E-64 135.859315

LENG8 -2.096653697 6.893752121 -21.27325393 1.18E-64 5.67E-64 135.8515759

SRRM1 -1.159595623 4.98060324 -21.24927379 1.47E-64 7.07E-64 135.6309331

NCF4 1.794808696 2.992027942 21.24788613 1.49E-64 7.16E-64 135.6181642

PANK3 1.006793301 3.328906109 21.24704648 1.50E-64 7.21E-64 135.6104378

PPP1R13B -1.149936752 3.79589683 -21.23533237 1.67E-64 8.03E-64 135.5026416

ATOX1 -1.265185533 4.705118814 -21.23235254 1.71E-64 8.25E-64 135.4752191

POF1B 2.199771819 3.239604027 21.22558132 1.82E-64 8.78E-64 135.4129038

UCHL3 -1.294266514 3.963896867 -21.22556567 1.82E-64 8.78E-64 135.4127598

ABCF2 0.962679906 4.266597744 21.22106241 1.90E-64 9.15E-64 135.3713149

GNB3 -1.332547828 1.681428719 -21.21513114 2.01E-64 9.66E-64 135.3167259

DSN1 1.024040802 2.944247498 21.21462197 2.02E-64 9.70E-64 135.3120395

SASH3 2.148228473 2.708992332 21.21408037 2.03E-64 9.75E-64 135.3070548

UBE4B -1.144102059 4.116836577 -21.21203356 2.07E-64 9.93E-64 135.2882161

FAHD1 1.09271563 3.798285035 21.20984491 2.11E-64 1.01E-63 135.2680717

NUBP2 -1.367353811 4.858840486 -21.2093439 2.12E-64 1.02E-63 135.2634603

MRPS28 -1.07986364 4.056358234 -21.20870762 2.13E-64 1.02E-63 135.2576039

ZFP36L2 1.793017972 6.73480272 21.20677988 2.17E-64 1.04E-63 135.2398606

STPG3 -1.039611887 0.966282712 -21.20669274 2.17E-64 1.04E-63 135.2390586

CASP4 -1.625715885 5.110181887 -21.19856604 2.34E-64 1.12E-63 135.1642562

CCL17 2.897615579 1.673142137 21.19513762 2.41E-64 1.16E-63 135.1326982

IL17RD 0.897501193 1.132258905 21.18856645 2.57E-64 1.23E-63 135.0722095

MAFG 0.972525068 3.599472327 21.18799865 2.58E-64 1.24E-63 135.0669827

ITGA3 2.010601096 5.445697635 21.1864336 2.62E-64 1.25E-63 135.0525758

FEM1C 1.022353102 3.276442662 21.18236129 2.72E-64 1.30E-63 135.0150879

KCND1 0.813491089 1.41811987 21.18220597 2.72E-64 1.30E-63 135.013658

AC087289.3 -0.52840446 0.301631961 -21.18178529 2.73E-64 1.31E-63 135.0097854

YLPM1 -0.971333322 3.925881013 -21.18024098 2.77E-64 1.33E-63 134.9955688

KRAS 1.086270968 3.594838402 21.17728049 2.85E-64 1.36E-63 134.9683149

MAT2A -2.066596615 7.372067521 -21.17667673 2.86E-64 1.37E-63 134.9627566

RNF207 -1.726164777 3.561139716 -21.17136723 3.01E-64 1.44E-63 134.9138764

JPT2 1.191844554 5.089098034 21.17097527 3.02E-64 1.44E-63 134.9102679

GPRC5C -1.981777075 5.18300862 -21.17026901 3.04E-64 1.45E-63 134.9037658

NDUFA3 -1.515303489 5.909023382 -21.16877412 3.08E-64 1.47E-63 134.8900032

TAGAP 1.644114728 1.466075383 21.16797209 3.10E-64 1.48E-63 134.8826193

IL12RB1 1.148263021 1.122917966 21.16514683 3.18E-64 1.52E-63 134.8566083

HIST2H2AA4 -2.614369966 1.286831982 -21.15926719 3.36E-64 1.60E-63 134.8024752

A4GALT 1.690758087 3.49863284 21.15807886 3.40E-64 1.62E-63 134.7915342

BCL6B 1.474070599 2.418634409 21.15803171 3.40E-64 1.62E-63 134.7911001

TMEM50A 1.154376232 5.752044132 21.15466199 3.50E-64 1.67E-63 134.7600744

SLC25A24 1.276989052 3.403358294 21.15282424 3.56E-64 1.70E-63 134.7431536

ZNF682 -1.012421247 1.459639516 -21.14988628 3.66E-64 1.75E-63 134.7161024

CHML 1.078506769 1.919861587 21.1431616 3.90E-64 1.86E-63 134.6541832

HOXA5 1.397309075 1.940353528 21.14016341 4.00E-64 1.91E-63 134.6265757

SOCS1 1.756210839 2.601438936 21.13862516 4.06E-64 1.94E-63 134.6124112

SUSD3 1.577510028 1.761785585 21.13223017 4.31E-64 2.05E-63 134.5535237

GPHN -1.15686538 3.32667972 -21.12796103 4.48E-64 2.14E-63 134.5142104

CASC4 1.201823741 4.861335568 21.127476 4.50E-64 2.14E-63 134.5097438

SOCS7 -1.158251044 2.914455701 -21.12438712 4.63E-64 2.21E-63 134.4812986

SLC37A1 1.219351187 3.35854767 21.12078476 4.79E-64 2.28E-63 134.4481239

KLRK1 -0.921920364 0.782463371 -21.11785048 4.92E-64 2.34E-63 134.4211012

CYP4F3 2.088843686 1.546166906 21.11687875 4.96E-64 2.36E-63 134.4121521

CCDC59 -1.031039043 3.962664857 -21.11423186 5.08E-64 2.42E-63 134.3877755

DNAAF2 0.787178351 2.826916359 21.11252684 5.17E-64 2.46E-63 134.3720728

WDR89 0.74197177 2.8989941 21.11007542 5.28E-64 2.51E-63 134.3494958

ANXA1 2.425598303 5.824011277 21.10802752 5.38E-64 2.56E-63 134.3306349

CLEC18B -1.109158698 0.847468004 -21.10616836 5.48E-64 2.60E-63 134.313512

KNL1 0.977936429 0.873479844 21.10265954 5.66E-64 2.69E-63 134.2811952

VKORC1 -1.47946163 5.907810909 -21.09413237 6.12E-64 2.91E-63 134.2026557

NEDD1 0.866449004 2.7369774 21.09356928 6.15E-64 2.92E-63 134.1974692

PXMP2 -1.435685348 4.23686085 -21.08959497 6.38E-64 3.03E-63 134.1608623

ACAP3 -1.488888133 4.173590726 -21.08557546 6.62E-64 3.14E-63 134.1238381

MEPCE 0.999029326 4.632922548 21.08356298 6.74E-64 3.20E-63 134.1053006

FAM162A -1.634592717 5.57598182 -21.08249727 6.81E-64 3.23E-63 134.095484

GLA 1.013494519 3.985757275 21.08234391 6.82E-64 3.24E-63 134.0940713

OLR1 2.79423146 2.672675988 21.08218459 6.83E-64 3.24E-63 134.0926037

FAM98B 0.777704341 2.91567273 21.0744385 7.34E-64 3.48E-63 134.0212496

PLA2G4A 1.637966924 2.76983902 21.07095032 7.57E-64 3.59E-63 133.9891166

COX11 -1.089411631 4.006876794 -21.0682952 7.76E-64 3.68E-63 133.9646574

ADGRV1 -1.149605329 0.9340797 -21.06757989 7.81E-64 3.70E-63 133.9580677

GPNMB 2.467685384 4.822867427 21.06664057 7.88E-64 3.73E-63 133.9494145

NTM 2.697360471 2.186905125 21.06259077 8.18E-64 3.88E-63 133.9121061

HIF3A -1.824793724 2.128885658 -21.06092871 8.31E-64 3.93E-63 133.8967943

ZNF514 -1.150418196 3.088063895 -21.0587829 8.47E-64 4.01E-63 133.8770257

PUM1 0.954774336 4.562939845 21.05594394 8.70E-64 4.12E-63 133.8508708

PDPN 2.265608074 3.464211768 21.04952458 9.23E-64 4.37E-63 133.7917289

SYCN -7.506861454 7.860634768 -21.04921612 9.25E-64 4.38E-63 133.7888869

EEF1AKMT2 -0.911607114 2.987215429 -21.04158537 9.93E-64 4.70E-63 133.7185812

NPIPB11 -1.078344979 1.033973141 -21.03982076 1.01E-63 4.77E-63 133.7023226

ADGRL4 1.463127887 3.08797643 21.03869923 1.02E-63 4.82E-63 133.6919889

CPB1 -8.055462389 9.239809868 -21.03278671 1.08E-63 5.09E-63 133.637511

CEBPG 1.210705824 4.163198628 21.02753711 1.13E-63 5.34E-63 133.5891394

C11orf65 -0.664478885 0.692539267 -21.02235842 1.19E-63 5.60E-63 133.5414198

EEFSEC 0.88053296 3.798241132 21.01571308 1.26E-63 5.95E-63 133.4801834

ZNF83 -1.565902066 4.34236284 -21.01464563 1.27E-63 6.01E-63 133.4703467

SF3B1 -1.528781991 6.912234165 -21.01380903 1.28E-63 6.05E-63 133.4626373

SDE2 1.050084529 3.351216194 21.0127487 1.29E-63 6.11E-63 133.4528661

HDLBP -1.672062198 7.700776074 -21.00808076 1.35E-63 6.38E-63 133.4098491

UNC93B1 1.601466575 4.856099603 21.00807047 1.35E-63 6.38E-63 133.4097542

RCOR2 1.158873733 1.32595276 21.00521198 1.39E-63 6.55E-63 133.3834115

HPGDS 1.442587786 1.480336301 21.00378435 1.41E-63 6.63E-63 133.3702548

PAH -2.510993133 2.491690176 -21.00316939 1.41E-63 6.67E-63 133.3645875

CCRL2 1.467201441 1.959258523 21.00171258 1.43E-63 6.76E-63 133.3511617

N4BP1 0.784125317 3.688462958 20.99877268 1.47E-63 6.94E-63 133.3240677

RARG 1.406959382 3.864483884 20.99746214 1.49E-63 7.02E-63 133.3119897

ZBED1 1.10567364 4.687638565 20.98819662 1.62E-63 7.65E-63 133.2265952

POMC -2.405425149 2.40488587 -20.98816853 1.62E-63 7.65E-63 133.2263362

ACTR1A 1.02682967 5.570811575 20.98733363 1.64E-63 7.70E-63 133.2186413

ADAM17 1.084704914 2.826842835 20.98355454 1.69E-63 7.97E-63 133.1838102

INPP5F 1.076822424 2.970576666 20.98258303 1.71E-63 8.04E-63 133.1748559

GZMH 1.824383826 1.463556026 20.98107478 1.73E-63 8.15E-63 133.1609544

APOE 3.150924902 6.216029286 20.98078312 1.74E-63 8.17E-63 133.1582662

AC026464.1 -0.739732059 0.415006254 -20.97293053 1.87E-63 8.79E-63 133.0858869

POLR3GL 1.003746722 4.677496736 20.96979585 1.92E-63 9.04E-63 133.0569928

MYRIP -1.305635362 1.586218074 -20.96917651 1.93E-63 9.09E-63 133.051284

ZNF484 0.620073794 1.438665405 20.96512999 2.01E-63 9.43E-63 133.013984

ARL13B 0.893262141 2.187293256 20.96091639 2.09E-63 9.81E-63 132.9751431

RGL1 1.326852921 2.882507134 20.95492879 2.21E-63 1.04E-62 132.9199478

THRAP3 1.02070422 5.401159659 20.94818854 2.35E-63 1.10E-62 132.857812

KRTDAP -1.317667367 1.056223625 -20.94627119 2.39E-63 1.12E-62 132.8401363

CUL5 0.734291343 3.172851653 20.94492859 2.42E-63 1.14E-62 132.8277588

UPF3B 0.806125563 3.326500467 20.94452518 2.43E-63 1.14E-62 132.8240399

AGMAT 1.463734553 1.383222713 20.94147603 2.50E-63 1.17E-62 132.7959295

COL6A2 2.39762996 7.239093446 20.93966624 2.54E-63 1.19E-62 132.7792446

RNF186 -2.703583846 3.465724625 -20.93427685 2.67E-63 1.25E-62 132.7295577

NUTM2G -0.291625741 0.282209488 -20.92692954 2.86E-63 1.34E-62 132.6618173

PDCD1LG2 1.264722127 1.009130641 20.92290101 2.96E-63 1.39E-62 132.6246741

AIF1 2.089966788 4.295435891 20.91428184 3.21E-63 1.50E-62 132.545202

PHKG2 -1.118116044 3.77377814 -20.91231323 3.27E-63 1.53E-62 132.5270501

PTDSS2 -1.149573218 3.824872207 -20.91195653 3.28E-63 1.53E-62 132.523761

CDC42 1.224780154 6.404974986 20.91091304 3.31E-63 1.55E-62 132.5141393

LTB 2.748701803 2.907127567 20.91080139 3.31E-63 1.55E-62 132.5131097

GNAI1 1.519747005 3.080435441 20.90966686 3.35E-63 1.57E-62 132.5026485

SLC35F2 1.585414587 3.205826824 20.90871126 3.38E-63 1.58E-62 132.493837

EIF3G -1.526415084 7.106699908 -20.90294055 3.56E-63 1.67E-62 132.4406249

IL23A 1.442223907 1.75522757 20.8996785 3.67E-63 1.72E-62 132.4105445

RAB5A 0.963760185 4.66060291 20.89717453 3.76E-63 1.76E-62 132.3874542

MTRNR2L12 1.329675511 0.918210742 20.8964783 3.78E-63 1.77E-62 132.3810339

NECTIN4 2.558118433 3.645037295 20.89191826 3.94E-63 1.84E-62 132.3389827

STMN2 2.41662646 2.848312814 20.88951769 4.03E-63 1.88E-62 132.3168449

DOK2 1.701891105 2.401926373 20.87986905 4.41E-63 2.06E-62 132.2278634

TGM2 2.840716095 5.764857722 20.87673472 4.54E-63 2.12E-62 132.1989569

CYP1B1 2.580192401 2.974660018 20.87061197 4.80E-63 2.24E-62 132.1424882

WDR88 0.603700982 0.532378735 20.86545768 5.03E-63 2.35E-62 132.0949498

MPP3 -1.281567712 2.904985275 -20.8640499 5.10E-63 2.38E-62 132.0819656

ZBTB16 -2.249727107 2.78372725 -20.86073958 5.26E-63 2.45E-62 132.0514333

DOLK 1.053939761 3.801597451 20.85356322 5.62E-63 2.62E-62 131.9852412

C17orf53 0.991128103 1.074678377 20.85299531 5.65E-63 2.63E-62 131.9800029

SLC7A7 1.627169125 3.515447701 20.85183422 5.71E-63 2.66E-62 131.9692931

CTXN1 1.900601867 2.38656254 20.84970868 5.82E-63 2.71E-62 131.9496872

SCEL 2.640537731 1.717073367 20.84868785 5.87E-63 2.74E-62 131.940271

NSA2 -1.387550632 5.609993503 -20.8451752 6.07E-63 2.83E-62 131.9078697

EXOSC8 -1.092138586 4.140215269 -20.84391975 6.14E-63 2.86E-62 131.8962891

AC091551.1 -0.528016226 0.324512412 -20.8418224 6.26E-63 2.91E-62 131.8769423

FAM189A2 -1.67455216 2.595021085 -20.83472485 6.68E-63 3.11E-62 131.8114702

AKR1B10 3.706520525 2.820840982 20.83208329 6.85E-63 3.19E-62 131.7871021

KCNJ1 -0.238860034 0.167334119 -20.83058297 6.94E-63 3.23E-62 131.7732617

SPDYE3 0.664313246 1.494169249 20.82551961 7.27E-63 3.38E-62 131.7265515

PRR26 -1.201676262 1.811466488 -20.82190912 7.52E-63 3.50E-62 131.6932433

CTNNB1 1.32161584 6.536426062 20.81792803 7.80E-63 3.63E-62 131.6565155

PIAS2 -0.725787687 1.89994233 -20.81543415 7.98E-63 3.71E-62 131.6335077

ZNRD2 -1.30080842 4.952421122 -20.81117832 8.30E-63 3.86E-62 131.5942437

SORL1 1.329977027 3.174638529 20.81110274 8.31E-63 3.86E-62 131.5935464

KATNAL2 -0.859635656 1.294056012 -20.81051137 8.35E-63 3.88E-62 131.5880904

ZC3H7A -1.022384809 4.434063728 -20.81008795 8.39E-63 3.89E-62 131.584184

2-Mar 1.062724378 4.447513247 20.80264487 8.98E-63 4.17E-62 131.515512

TTC26 0.770683381 1.683083634 20.80092821 9.13E-63 4.24E-62 131.4996734

TMC7 2.042895494 1.77101172 20.78587724 1.05E-62 4.87E-62 131.360799

CCDC91 -1.086569898 3.466002988 -20.78585524 1.05E-62 4.87E-62 131.3605961

CACNA2D1 1.294426653 1.51102211 20.78263796 1.08E-62 5.01E-62 131.330909

KRIT1 -1.023948446 3.87559471 -20.77648428 1.14E-62 5.30E-62 131.2741251

ARL11 1.002461979 0.811502683 20.77549854 1.15E-62 5.35E-62 131.2650288

DUS3L -1.272952302 4.331515488 -20.77225602 1.19E-62 5.51E-62 131.2351071

TNFRSF6B -1.733144958 0.841381411 -20.76370679 1.29E-62 5.96E-62 131.1562128

DEK 1.085345508 5.008070299 20.75930464 1.34E-62 6.21E-62 131.1155874

KTI12 0.845189685 3.115021083 20.75917783 1.34E-62 6.21E-62 131.1144171

DHCR24 2.117135502 5.369200871 20.75799224 1.36E-62 6.28E-62 131.1034757

ZNF217 1.319887181 3.605189712 20.7531152 1.42E-62 6.57E-62 131.0584662

B3GNT8 1.534564705 2.157057924 20.74799455 1.49E-62 6.88E-62 131.0112072

ECD 0.768151466 3.806275973 20.74745762 1.49E-62 6.92E-62 131.0062517

PRKCZ -1.223877579 3.284406788 -20.74634602 1.51E-62 6.99E-62 130.9959924

PHYHIPL -1.676786091 1.998546112 -20.7454843 1.52E-62 7.04E-62 130.9880393

CCDC22 0.881832206 4.037528698 20.74169517 1.58E-62 7.29E-62 130.9530678

ZNF641 -0.860131308 3.060568625 -20.74147993 1.58E-62 7.30E-62 130.9510812

MBD1 -1.046150261 4.573951514 -20.74017192 1.60E-62 7.39E-62 130.9390088

KIF15 1.051936678 1.038449666 20.7339307 1.69E-62 7.82E-62 130.8814036

NUP214 -0.952208509 3.93887994 -20.72878345 1.78E-62 8.20E-62 130.8338942

ZNF623 0.824552108 2.84182932 20.71856489 1.95E-62 9.01E-62 130.7395723

CATIP -0.845329372 0.92950028 -20.71679414 1.98E-62 9.16E-62 130.7232269

MLLT6 -1.409860844 5.346884622 -20.71606419 2.00E-62 9.22E-62 130.716489

ZNF571 -0.663657835 1.418181943 -20.71466829 2.02E-62 9.34E-62 130.7036036

SLC9B2 1.770367142 2.674320113 20.71406018 2.03E-62 9.39E-62 130.6979902

USP40 -0.988305603 3.961460088 -20.71331771 2.05E-62 9.45E-62 130.6911364

IPO5 -1.322367072 5.426262638 -20.70986671 2.11E-62 9.75E-62 130.6592801

NSMAF -1.006300882 4.024621506 -20.70268889 2.26E-62 1.04E-61 130.5930194

SMARCAL1 0.733080377 3.253670964 20.70219605 2.27E-62 1.05E-61 130.5884697

STX11 1.304545754 1.654051026 20.70191167 2.28E-62 1.05E-61 130.5858445

RITA1 0.918737775 3.644544685 20.69792988 2.36E-62 1.09E-61 130.5490861

MFNG 1.39835778 3.015679229 20.69775762 2.36E-62 1.09E-61 130.5474958

SVBP 0.964334072 3.886528459 20.69653965 2.39E-62 1.10E-61 130.5362518

DKK1 3.08060427 2.061259519 20.69303271 2.47E-62 1.14E-61 130.5038761

ADCY4 -1.399452316 2.954046111 -20.69047695 2.53E-62 1.16E-61 130.4802813

STK35 0.814612986 3.356291997 20.68861218 2.57E-62 1.18E-61 130.4630654

NFKBIB 1.116913198 4.354490899 20.68550066 2.65E-62 1.22E-61 130.4343391

MNS1 1.190203285 1.907859235 20.68495793 2.66E-62 1.22E-61 130.4293285

TRIB1 1.630085339 4.532549297 20.68399106 2.69E-62 1.23E-61 130.4204019

ACSF2 -1.646345611 4.232843949 -20.68070779 2.77E-62 1.27E-61 130.3900891

LAPTM4A 1.597889396 8.082395057 20.68028556 2.78E-62 1.28E-61 130.3861909

C3orf70 1.131031328 0.992769149 20.67412066 2.94E-62 1.35E-61 130.3292719

YJEFN3 -1.398150061 2.244117951 -20.67207523 3.00E-62 1.38E-61 130.3103866

PLA1A 1.531635983 2.490809397 20.67175339 3.01E-62 1.38E-61 130.307415

RAB11FIP4 -1.206632064 3.30227192 -20.67136469 3.02E-62 1.39E-61 130.3038261

CLNS1A -1.290189239 5.529806895 -20.67105957 3.03E-62 1.39E-61 130.301009

TTC33 0.919673852 2.873608866 20.66223482 3.28E-62 1.51E-61 130.2195277

LRRC58 0.950036834 3.115803914 20.66064703 3.33E-62 1.53E-61 130.2048669

SPATA9 -0.595065013 0.650817625 -20.65944373 3.37E-62 1.55E-61 130.1937561

RHBDD2 1.455584254 6.07055954 20.65788885 3.42E-62 1.57E-61 130.1793988

LRRC25 1.603577171 2.057583613 20.65594746 3.48E-62 1.60E-61 130.1614726

DTX4 1.399218124 3.733888518 20.65186627 3.61E-62 1.66E-61 130.1237873

TPP1 1.226391376 5.768015927 20.65157906 3.62E-62 1.66E-61 130.1211353

CD53 2.340924779 4.246118273 20.64866797 3.72E-62 1.70E-61 130.0942541

SNAPIN 0.917887889 4.701631773 20.64832784 3.73E-62 1.71E-61 130.0911132

NUDT19 0.984803556 3.047420383 20.64647728 3.80E-62 1.74E-61 130.0740248

CALB2 2.742484179 2.078624879 20.64630279 3.80E-62 1.74E-61 130.0724135

NPTX1 1.807199418 1.285868907 20.64478857 3.86E-62 1.77E-61 130.0584308

NDUFS5 1.626300385 8.224857972 20.64418226 3.88E-62 1.77E-61 130.0528319

RGMA -1.744197651 2.349103671 -20.64288227 3.92E-62 1.80E-61 130.0408273

RPP25 1.309065327 3.438672426 20.64223274 3.95E-62 1.81E-61 130.0348293

ANKDD1A -0.95434582 2.190653193 -20.64137564 3.98E-62 1.82E-61 130.0269144

ADGRB2 1.007720658 1.135980033 20.6386048 4.08E-62 1.87E-61 130.0013269

OR6M1 -0.526957229 0.27029659 -20.63569515 4.19E-62 1.92E-61 129.9744573

BHLHE40 2.499638859 6.632716868 20.63313111 4.29E-62 1.96E-61 129.9507788

GJC2 1.961018685 1.59569552 20.63259163 4.32E-62 1.97E-61 129.9457967

ICA1L -1.14807605 1.595460803 -20.6302985 4.41E-62 2.01E-61 129.9246198

LASP1 1.184028779 5.994058817 20.62894768 4.46E-62 2.04E-61 129.9121448

MBD2 -1.24690191 5.117376238 -20.62826235 4.49E-62 2.05E-61 129.9058157

MBD5 -0.764284643 2.14326706 -20.62802152 4.50E-62 2.06E-61 129.9035916

GTPBP10 -0.828111182 2.650992871 -20.62589613 4.59E-62 2.10E-61 129.8839633

RHOJ 1.396985113 2.703980724 20.62395209 4.67E-62 2.13E-61 129.8660096

GBP3 2.199742183 3.544934886 20.62009095 4.84E-62 2.21E-61 129.8303503

EPHA3 1.830016294 2.075009446 20.61918015 4.88E-62 2.23E-61 129.8219386

SYNGR1 -1.528350856 3.523337818 -20.61593022 5.03E-62 2.30E-61 129.7919235

WDR66 -1.240880264 1.386236373 -20.61506679 5.07E-62 2.31E-61 129.783949

ZER1 1.010605489 4.597184355 20.61383991 5.13E-62 2.34E-61 129.7726178

MEX3A 0.970383574 1.06039601 20.61314389 5.16E-62 2.35E-61 129.7661895

GLI1 1.528001772 1.767854242 20.59950653 5.86E-62 2.67E-61 129.6402326

LMAN2L 0.960755863 4.010207645 20.59584987 6.06E-62 2.76E-61 129.6064576

ARHGAP23 1.648156517 2.875445075 20.59511037 6.10E-62 2.78E-61 129.599627

IL17RA 0.825487188 2.906359767 20.59302959 6.22E-62 2.83E-61 129.5804074

POLA1 0.704739299 2.201260864 20.59210249 6.27E-62 2.86E-61 129.5718439

CNST 0.777646193 2.974513424 20.59010929 6.39E-62 2.91E-61 129.553433

MKS1 -1.016953866 3.37452941 -20.58996002 6.40E-62 2.91E-61 129.5520541

HOMER1 0.886930803 1.231034622 20.58869993 6.47E-62 2.94E-61 129.5404147

COL22A1 1.778452729 1.35709595 20.58842162 6.49E-62 2.95E-61 129.537844

NRGN 1.461539102 2.387662975 20.58802636 6.51E-62 2.96E-61 129.534193

TGFA 1.735563739 3.28552173 20.58455169 6.73E-62 3.06E-61 129.502097

PCDHB7 0.907343358 1.012029121 20.58378893 6.77E-62 3.08E-61 129.4950512

SMAD7 1.144375838 3.731141948 20.57936505 7.06E-62 3.21E-61 129.4541863

RELT 1.15123717 1.65459748 20.57860304 7.11E-62 3.23E-61 129.4471473

QPCT 2.524647982 3.622659882 20.57631605 7.26E-62 3.30E-61 129.4260211

CCDC142 -0.801465844 2.106500425 -20.57572008 7.30E-62 3.31E-61 129.4205158

ZNF773 -0.780100883 1.584494203 -20.56998735 7.69E-62 3.49E-61 129.3675583

PLS3 1.261664662 4.925798805 20.56810256 7.83E-62 3.55E-61 129.3501467

MYSM1 -1.082619067 3.215389832 -20.56803595 7.83E-62 3.55E-61 129.3495314

CDK6 1.192610206 2.960806732 20.56779923 7.85E-62 3.56E-61 129.3473446

HNRNPD -1.331489181 6.555701286 -20.56443739 8.10E-62 3.67E-61 129.3162877

NCOA4 1.267947131 5.775308134 20.56366276 8.16E-62 3.70E-61 129.3091315

PLA2G1B -7.760788236 9.316055778 -20.5633017 8.18E-62 3.71E-61 129.3057959

SEMA6B 1.47789077 2.867570094 20.55840612 8.56E-62 3.88E-61 129.260569

YTHDF3 0.943682321 4.067418184 20.55601611 8.75E-62 3.97E-61 129.238489

TIGD2 0.818469813 2.34524477 20.5540958 8.91E-62 4.04E-61 129.220748

GNAI2 0.980908945 6.230698104 20.55399773 8.92E-62 4.04E-61 129.219842

GPAT4 -1.17638915 5.092004094 -20.55136824 9.14E-62 4.14E-61 129.1955489

CHST1 1.436593246 1.926608644 20.55043085 9.22E-62 4.17E-61 129.1868886

MUCL3 4.737999388 2.745403655 20.54860698 9.37E-62 4.24E-61 129.1700382

PPWD1 -1.048891651 4.211291574 -20.54799934 9.43E-62 4.27E-61 129.1644242

NDUFB5 -1.110795022 4.973634583 -20.54759734 9.46E-62 4.28E-61 129.1607102

ELF2 -0.839657388 3.556541771 -20.54561029 9.64E-62 4.36E-61 129.1423518

MSC 1.819736557 3.14991084 20.5454418 9.65E-62 4.36E-61 129.1407952

SMIM24 3.13838344 3.932171074 20.54493214 9.70E-62 4.38E-61 129.1360864

ST20-MTHFS -0.875621685 0.618600316 -20.54349043 9.83E-62 4.44E-61 129.1227663

ATP2B4 1.587476277 4.491953689 20.53395654 1.07E-61 4.85E-61 129.0346795

ZNF774 -0.893604908 1.624264347 -20.53164939 1.10E-61 4.95E-61 129.0133623

TMEM102 1.150163374 3.01402098 20.53102719 1.10E-61 4.98E-61 129.0076133

CSRP2 -1.358751322 3.073575902 -20.53020296 1.11E-61 5.02E-61 128.9999977

TRRAP 0.858278161 3.297310943 20.52655717 1.15E-61 5.19E-61 128.9663112

SRSF5 -1.560630483 6.95661099 -20.52607072 1.15E-61 5.21E-61 128.9618165

ARAP3 1.109231176 2.861933704 20.52562364 1.16E-61 5.23E-61 128.9576854

CFAP298 -1.101089092 4.27626762 -20.5216305 1.20E-61 5.43E-61 128.9207887

HIST1H4H 2.150437443 1.846507414 20.5111242 1.33E-61 5.98E-61 128.8237065

TPST1 1.347697504 3.601934178 20.51090961 1.33E-61 5.99E-61 128.8217236

RASSF4 -1.83807886 4.382486862 -20.51070029 1.33E-61 6.00E-61 128.8197894

CPA1 -8.520631773 9.93652212 -20.50889009 1.35E-61 6.10E-61 128.8030619

CATSPERB -2.071605527 2.408490542 -20.50287358 1.43E-61 6.44E-61 128.7474641

DOK4 1.362041446 4.526621581 20.50259078 1.43E-61 6.46E-61 128.7448508

LCN2 4.99074369 7.416878804 20.50249791 1.44E-61 6.46E-61 128.7439925

SON -1.347875411 5.8877684 -20.5005415 1.46E-61 6.58E-61 128.7259133

GOLGA8O -0.940757045 0.508483618 -20.50017048 1.47E-61 6.60E-61 128.7224846

ADAR 1.13282532 5.868034759 20.4974612 1.50E-61 6.77E-61 128.6974477

MAPKAPK2 1.120872549 5.885974039 20.49578436 1.53E-61 6.87E-61 128.6819515

CLDN18 5.290256656 3.358366125 20.49213808 1.58E-61 7.11E-61 128.6482549

DDX10 -0.900345606 3.246334279 -20.48756811 1.65E-61 7.41E-61 128.6060211

C10orf142 -0.976001124 0.555116876 -20.48579679 1.67E-61 7.53E-61 128.589651

MXRA8 2.072043225 5.553959473 20.48453362 1.69E-61 7.62E-61 128.5779771

GNL3L 0.978797044 2.576663106 20.47923502 1.78E-61 8.00E-61 128.5290077

ABHD17B 0.869947996 2.983333362 20.47767499 1.81E-61 8.11E-61 128.5145897

WAS 1.856080361 2.775678849 20.47346204 1.88E-61 8.43E-61 128.4756526

CHST2 1.162290278 1.678094132 20.47068365 1.93E-61 8.65E-61 128.4499736

OSBPL9 -1.109223197 4.680079689 -20.46931431 1.95E-61 8.76E-61 128.4373176

FBXO17 -1.276003408 2.986228872 -20.46777026 1.98E-61 8.88E-61 128.4230466

CSNK1G3 0.807125232 3.419206048 20.46771304 1.98E-61 8.88E-61 128.4225177

PIP4P2 1.065658608 2.761683922 20.46635014 2.00E-61 8.99E-61 128.409921

METRN -1.805541719 4.42273626 -20.46569476 2.02E-61 9.05E-61 128.4038636

AL589666.1 -0.596824706 0.315543101 -20.46263222 2.07E-61 9.30E-61 128.3755573

SLC16A6 0.991138363 0.867934271 20.4577233 2.17E-61 9.73E-61 128.3301844

WDR6 -1.544614729 6.41061858 -20.45180478 2.29E-61 1.03E-60 128.2754786

SH2B1 -1.208922686 4.829459114 -20.45137272 2.30E-61 1.03E-60 128.2714849

SLAMF6 1.872801008 1.558284959 20.45028356 2.33E-61 1.04E-60 128.2614175

PFKFB3 1.799058615 4.625307128 20.44946798 2.34E-61 1.05E-60 128.2538787

EFNA2 2.121267797 1.17867145 20.44921294 2.35E-61 1.05E-60 128.2515213

ABCC10 -1.002779604 3.615216085 -20.44874163 2.36E-61 1.06E-60 128.2471648

FOXM1 1.716622869 2.14764979 20.44840302 2.37E-61 1.06E-60 128.2440348

LLPH -0.86793845 3.103494845 -20.44698236 2.40E-61 1.07E-60 128.2309031

ADGRF1 1.993243045 1.457577637 20.44506793 2.44E-61 1.09E-60 128.2132069

SYCE1 -1.716308982 1.004948272 -20.4446845 2.45E-61 1.10E-60 128.2096626

SUGP2 -1.274295568 4.289500397 -20.44432856 2.46E-61 1.10E-60 128.2063725

MPV17L -1.649238454 2.771364884 -20.43327712 2.72E-61 1.22E-60 128.1042142

SLC35B3 0.961364295 4.345531552 20.42936947 2.82E-61 1.26E-60 128.0680912

LGALS8 -1.497191206 4.510913206 -20.4264061 2.90E-61 1.30E-60 128.0406966

C1orf198 1.184874383 4.703207671 20.42555428 2.92E-61 1.31E-60 128.0328221

AACS -1.078919347 3.301874159 -20.42122883 3.04E-61 1.36E-60 127.9928352

DHRS4L2 -1.237160589 3.95930353 -20.42042388 3.06E-61 1.37E-60 127.9853937

SLC35B4 0.779141006 2.283998984 20.41923042 3.10E-61 1.38E-60 127.9743605

TARBP1 -1.318863856 4.049350044 -20.4166691 3.17E-61 1.42E-60 127.9506816

HPRT1 1.109829698 4.931550534 20.40890384 3.41E-61 1.52E-60 127.8788915

TMEM243 -1.112987561 4.207635593 -20.40519308 3.53E-61 1.57E-60 127.8445846

MAU2 -0.97177965 4.163359219 -20.40403499 3.57E-61 1.59E-60 127.8338775

FAM210B 1.172539751 5.082555291 20.40309985 3.60E-61 1.60E-60 127.8252318

CLIC2 1.580697155 2.594170295 20.40245257 3.62E-61 1.61E-60 127.8192473

IL11RA -1.289599514 3.1878106 -20.40130522 3.66E-61 1.63E-60 127.8086395

VDAC2 -1.406128449 6.191576528 -20.39873679 3.74E-61 1.67E-60 127.7848929

LYAR 0.979845069 3.355001148 20.39686548 3.81E-61 1.70E-60 127.7675914

ZNF44 -0.782391508 2.791479457 -20.39457878 3.89E-61 1.73E-60 127.746449

UBE2D4 -1.010373698 3.606217298 -20.39098956 4.02E-61 1.79E-60 127.7132635

SUGP1 -0.988775735 4.105903 -20.39075359 4.03E-61 1.80E-60 127.7110817

DDR2 1.725800779 2.59965074 20.38539979 4.24E-61 1.89E-60 127.66158

PROSER3 -0.961377235 2.791989212 -20.38122603 4.40E-61 1.96E-60 127.622988

UGT1A10 2.620883277 1.414445667 20.37559193 4.64E-61 2.06E-60 127.5708922

BCAS3 -0.831526144 2.551714837 -20.37483612 4.67E-61 2.08E-60 127.5639035

ZBTB4 1.026249535 4.689854527 20.37302029 4.75E-61 2.11E-60 127.547113

FCHO1 1.334880306 1.693386222 20.36978113 4.89E-61 2.18E-60 127.5171611

CAPZB 1.323052584 6.870100345 20.36817453 4.97E-61 2.21E-60 127.502305

ACSS1 -1.776015134 4.509061268 -20.36362421 5.18E-61 2.30E-60 127.4602279

SNRNP70 -1.708902246 7.229802297 -20.36306586 5.21E-61 2.31E-60 127.4550647

NEIL3 0.777020102 0.512216982 20.3617655 5.27E-61 2.34E-60 127.44304

TMEM220 -1.310119648 2.917311303 -20.35603017 5.56E-61 2.47E-60 127.3900034

EDN1 1.775366392 3.462562514 20.35342035 5.69E-61 2.53E-60 127.3658689

FBN1 2.248072297 3.93669065 20.35124698 5.81E-61 2.58E-60 127.3457704

SLC30A6 0.776093968 3.31107278 20.34596379 6.10E-61 2.71E-60 127.2969125

FAAP24 0.938683894 1.445047217 20.34417386 6.20E-61 2.75E-60 127.2803593

SNX6 1.041243875 4.492029334 20.34337172 6.25E-61 2.77E-60 127.2729411

IRF8 1.879294527 2.792967149 20.34162621 6.35E-61 2.82E-60 127.2567985

EME2 -1.272217241 2.943642742 -20.33826413 6.55E-61 2.91E-60 127.2257054

PRR35 -0.824529796 0.469302332 -20.33475415 6.77E-61 3.00E-60 127.193244

LRRC14 -1.137048066 4.000132426 -20.33337611 6.85E-61 3.04E-60 127.1804993

YPEL5 1.042266937 5.486318346 20.33242487 6.91E-61 3.06E-60 127.1717017

EIF2AK1 1.056917731 5.461495022 20.33102086 7.00E-61 3.10E-60 127.1587167

PKNOX2 -1.078997309 1.649383538 -20.32753728 7.23E-61 3.20E-60 127.1264985

CENPM 2.017831644 1.912455624 20.32446253 7.44E-61 3.30E-60 127.0980609

GRAMD1A -1.49097862 5.817964945 -20.32313402 7.53E-61 3.34E-60 127.0857737

MSL2 0.784145721 3.48989633 20.32191747 7.62E-61 3.37E-60 127.0745218

HRNR -0.423345574 0.303109753 -20.32011114 7.75E-61 3.43E-60 127.0578152

SMURF1 1.271955758 4.269026874 20.31864284 7.85E-61 3.48E-60 127.0442348

TCFL5 0.786896166 2.588030269 20.31669767 8.00E-61 3.54E-60 127.0262437

GIMAP4 1.679627595 3.730805229 20.31489075 8.13E-61 3.60E-60 127.0095312

LYPLA2 1.131909088 5.718491271 20.31346696 8.24E-61 3.64E-60 126.9963621

TNFRSF11B 2.427685768 2.374166791 20.30976021 8.53E-61 3.77E-60 126.9620771

SGSM3 -1.600705495 6.33294794 -20.30926998 8.56E-61 3.79E-60 126.9575427

B9D2 1.036162666 2.873483595 20.30584642 8.84E-61 3.91E-60 126.9258764

DNAJB7 -0.211372909 0.198010442 -20.30453941 8.95E-61 3.95E-60 126.913787

CTRB1 -8.287047401 10.0253001 -20.29787617 9.52E-61 4.20E-60 126.8521534

CRAMP1 -0.776067302 2.756019276 -20.29660788 9.63E-61 4.25E-60 126.8404217

GTF3C6 1.174933603 5.532204924 20.29474033 9.80E-61 4.33E-60 126.8231468

PGP 1.063458431 3.360341648 20.29256931 9.99E-61 4.41E-60 126.8030647

C2CD2L -0.948262822 3.063620521 -20.28862784 1.04E-60 4.58E-60 126.7666051

ENO2 1.953268135 4.07902 20.28106578 1.11E-60 4.91E-60 126.6966524

SGK2 -1.692951102 3.115500305 -20.27787792 1.14E-60 5.05E-60 126.6671625

PPP4R2 0.857742136 3.807349437 20.27777752 1.15E-60 5.05E-60 126.6662337

DIAPH3 0.990028483 0.852711094 20.27590245 1.17E-60 5.14E-60 126.6488878

ZNF573 -0.817966696 1.098373561 -20.2758825 1.17E-60 5.14E-60 126.6487033

DTNB -1.187267219 2.807165122 -20.27445753 1.18E-60 5.21E-60 126.635521

HSD17B6 1.498068912 1.231681721 20.27159642 1.21E-60 5.35E-60 126.609053

NET1 1.702916259 5.488881712 20.27059938 1.22E-60 5.40E-60 126.5998293

ANAPC7 -1.040202598 4.333258303 -20.26897044 1.24E-60 5.48E-60 126.5847599

CENPI 0.80031973 0.685506839 20.26318943 1.31E-60 5.78E-60 126.5312782

PHLDA3 1.630922331 5.034963087 20.26228867 1.32E-60 5.82E-60 126.522945

SLC35B1 -1.332723375 4.804249314 -20.25206185 1.45E-60 6.40E-60 126.4283304

THOC6 0.98603778 4.552543747 20.25075877 1.47E-60 6.48E-60 126.4162745

SEL1L -2.11062929 6.087868315 -20.24926863 1.49E-60 6.56E-60 126.4024878

SPG11 -1.024575082 4.39605558 -20.24885832 1.50E-60 6.59E-60 126.3986917

CHD1L 1.005783353 4.221681282 20.24186524 1.60E-60 7.03E-60 126.333991

MGME1 0.912701314 3.507353581 20.23697185 1.67E-60 7.35E-60 126.2887158

SBNO1 0.72312777 2.73084431 20.23677405 1.67E-60 7.36E-60 126.2868856

RGPD3 -0.276294039 0.197227197 -20.23363171 1.72E-60 7.58E-60 126.257811

NCOR1 -1.154765951 4.358969101 -20.23279011 1.74E-60 7.63E-60 126.2500241

HNF1A -1.403784693 2.502278622 -20.2309041 1.77E-60 7.77E-60 126.2325735

LIMK1 1.048187339 3.825476942 20.23034558 1.78E-60 7.81E-60 126.2274057

PCDHA10 -0.802212389 0.697948423 -20.22720775 1.83E-60 8.03E-60 126.1983721

DAPK2 -1.116276467 1.835987347 -20.22204918 1.92E-60 8.42E-60 126.1506402

INTS3 -1.237918708 4.360239616 -20.22033703 1.95E-60 8.56E-60 126.1347975

ADAM22 1.120616601 1.310297293 20.21865743 1.98E-60 8.69E-60 126.119256

KLHL2 0.910776761 3.432634209 20.2185557 1.98E-60 8.70E-60 126.1183147

IER5L 1.895947768 3.181733442 20.21596103 2.03E-60 8.90E-60 126.0943056

ZCCHC24 1.600967043 3.768885529 20.21414851 2.06E-60 9.05E-60 126.0775337

SYCP2 -1.410561815 1.593438947 -20.21037947 2.14E-60 9.37E-60 126.0426572

SLC16A14 0.863909947 1.225019229 20.20866862 2.17E-60 9.52E-60 126.0268258

ATP5MC2 -1.554720215 7.598130098 -20.20311374 2.29E-60 1.00E-59 125.9754227

TRIP13 1.401261293 1.571801515 20.19899572 2.38E-60 1.04E-59 125.937315

UBE2M 1.122700982 5.61586834 20.19665817 2.43E-60 1.06E-59 125.9156834

RBM33 -0.943691053 3.720416131 -20.19486993 2.47E-60 1.08E-59 125.8991349

EIF3E -1.525170445 7.196907703 -20.1938598 2.49E-60 1.09E-59 125.889787

SAMD5 1.449190364 1.378843033 20.19078965 2.56E-60 1.12E-59 125.8613752

AZGP1 -3.249633856 6.766977244 -20.18917563 2.60E-60 1.14E-59 125.8464385

SERP1 -1.485402672 6.70780536 -20.18765605 2.64E-60 1.15E-59 125.8323757

HID1 -1.932328771 5.963326386 -20.18736453 2.65E-60 1.16E-59 125.8296779

STIL 0.945891887 1.238924054 20.18523117 2.70E-60 1.18E-59 125.8099348

CEACAM19 -1.610842479 2.833334954 -20.18386265 2.73E-60 1.19E-59 125.7972698

MFN1 -1.048419297 4.387209165 -20.18233801 2.77E-60 1.21E-59 125.7831599

TIMM10 1.109160279 5.136639299 20.18180643 2.78E-60 1.22E-59 125.7782403

MGAT3 1.800025509 2.724118866 20.17353695 3.01E-60 1.31E-59 125.7017079

HNRNPF 1.289737176 6.54129877 20.17053257 3.09E-60 1.35E-59 125.6739024

GNL1 -1.160473413 4.996099187 -20.16985547 3.11E-60 1.36E-59 125.6676357

TPO -1.01259556 0.811893154 -20.16799854 3.16E-60 1.38E-59 125.6504496

B3GALT6 1.058560269 3.884625874 20.15869351 3.45E-60 1.51E-59 125.5643283

SGCD 1.465270366 1.251942425 20.15750293 3.49E-60 1.52E-59 125.5533088

FAS 1.220084236 3.025694661 20.15739326 3.49E-60 1.52E-59 125.5522938

AP1AR 0.976706158 3.403831535 20.15653835 3.52E-60 1.54E-59 125.5443811

SEC11A -1.25321235 5.964811313 -20.15015258 3.73E-60 1.63E-59 125.485276

SLC16A12 -2.547116651 2.702785361 -20.14431082 3.94E-60 1.72E-59 125.4312048

TTK 1.190231244 1.044022401 20.14109342 4.06E-60 1.77E-59 125.4014241

PIDD1 -1.34668966 3.383383069 -20.1396663 4.11E-60 1.79E-59 125.3882143

SPN 1.466992424 1.480549406 20.13918306 4.13E-60 1.80E-59 125.3837413

C16orf54 2.007388398 1.432625684 20.1391353 4.13E-60 1.80E-59 125.3832992

MAB21L4 2.088891917 1.846636106 20.1385965 4.15E-60 1.81E-59 125.3783119

GREM1 2.899188463 2.894246647 20.13682152 4.22E-60 1.84E-59 125.3618821

RAF1 -1.087113343 5.16010471 -20.13645684 4.24E-60 1.84E-59 125.3585065

SIPA1L2 1.337924996 2.875473466 20.13109826 4.45E-60 1.94E-59 125.3089048

ALDOC 1.653405038 3.12019354 20.12385093 4.76E-60 2.07E-59 125.2418181

SPPL2A -1.308134793 4.831152724 -20.1127867 5.28E-60 2.30E-59 125.1393957

FLII -1.498959162 6.665640676 -20.11128555 5.35E-60 2.33E-59 125.1254991

APBA3 -1.019460818 3.318296827 -20.10690473 5.57E-60 2.42E-59 125.0849439

TTC21B -0.789882867 2.660038609 -20.10574502 5.63E-60 2.45E-59 125.0742079

ESRRG -1.057983203 1.233059916 -20.10299373 5.78E-60 2.51E-59 125.0487374

GYS1 0.919036221 4.175870508 20.09752291 6.08E-60 2.64E-59 124.9980899

GINM1 1.066210958 5.014894787 20.0964161 6.14E-60 2.67E-59 124.9878432

CDC123 0.957240659 5.034320167 20.09598734 6.16E-60 2.68E-59 124.9838738

SH3PXD2B 1.563632815 3.823141155 20.09488962 6.23E-60 2.70E-59 124.9737111

TCTN3 0.877424118 4.641529827 20.09050001 6.48E-60 2.82E-59 124.9330719

PPP1R27 -0.46919238 0.289939865 -20.08754569 6.66E-60 2.89E-59 124.9057202

VPS4B 0.935101117 4.216353721 20.08743791 6.67E-60 2.90E-59 124.9047224

NADK2 -1.097364523 3.920578223 -20.08698318 6.70E-60 2.91E-59 124.9005124

ABHD4 0.994288589 4.310135064 20.08560755 6.78E-60 2.94E-59 124.8877764

LOXL1 1.652820887 4.114517015 20.08341899 6.92E-60 3.00E-59 124.8675139

APLP2 1.489040589 6.979644216 20.08337494 6.93E-60 3.00E-59 124.8671062

TBXA2R 0.861171999 1.34329102 20.08232475 6.99E-60 3.03E-59 124.857383

SEMA3F 1.306336702 3.31601256 20.08182803 7.03E-60 3.05E-59 124.8527842

DNAJA1 1.444837452 6.125532315 20.07863234 7.24E-60 3.14E-59 124.8231968

UEVLD 0.763650843 2.918923404 20.07838948 7.25E-60 3.14E-59 124.8209483

PUDP 1.148536065 3.242598422 20.07813322 7.27E-60 3.15E-59 124.8185757

OCLN -1.552929618 3.453539588 -20.07098814 7.77E-60 3.36E-59 124.7524213

FIG4 0.781394011 3.207797275 20.07001726 7.84E-60 3.39E-59 124.7434321

MYCBPAP -0.806744396 0.67748086 -20.07000657 7.84E-60 3.39E-59 124.7433331

GTF3C2 -1.015888852 4.674841337 -20.06988182 7.85E-60 3.40E-59 124.742178

SGO2 0.814913513 1.0475428 20.06226582 8.42E-60 3.64E-59 124.6716613

TMEM167B 0.869506304 4.315909087 20.05961983 8.63E-60 3.73E-59 124.6471614

TACC2 -1.25130176 3.413125354 -20.05749908 8.80E-60 3.81E-59 124.6275247

DUSP22 -1.173917427 4.520124843 -20.05482831 9.02E-60 3.90E-59 124.602795

ACIN1 -1.255739429 5.802934957 -20.05444815 9.05E-60 3.91E-59 124.5992749

MITD1 -0.98680952 3.671535243 -20.05377683 9.11E-60 3.94E-59 124.5930588

ITPR3 1.750322544 4.289770235 20.05244781 9.22E-60 3.99E-59 124.5807527

ANGEL2 -0.861122263 3.362180054 -20.05219587 9.24E-60 3.99E-59 124.5784199

DNAAF4 -0.72466773 1.09932169 -20.05206152 9.26E-60 4.00E-59 124.5771759

SPRYD7 0.816591416 2.891319798 20.05045565 9.39E-60 4.06E-59 124.5623062

MAPK3 1.16052337 5.434518853 20.04833713 9.58E-60 4.14E-59 124.5426894

TNFSF10 1.567050202 4.925329045 20.04013993 1.03E-59 4.46E-59 124.4667847

COQ4 -1.279999758 5.13657389 -20.03766122 1.06E-59 4.56E-59 124.4438318

ARMCX5-GPRASP2 -0.806919274 2.117998031 -20.03620153 1.07E-59 4.63E-59 124.4303149

SERAC1 -1.014581073 2.540665449 -20.03587823 1.08E-59 4.64E-59 124.4273211

EFCAB7 -0.658513071 1.862796858 -20.03483006 1.09E-59 4.68E-59 124.4176149

FAM149A -1.486802888 3.442222111 -20.03320992 1.10E-59 4.75E-59 124.4026121

HDHD2 -1.09238687 3.557903413 -20.02929075 1.14E-59 4.93E-59 124.3663193

SEZ6L2 1.627231192 4.966048667 20.02852784 1.15E-59 4.96E-59 124.3592545

RRP7A 1.034983385 4.255242103 20.02837868 1.15E-59 4.97E-59 124.3578732

GSKIP 1.09516353 3.6073523 20.02677821 1.17E-59 5.04E-59 124.3430521

FRY -1.170443098 3.134276447 -20.02548633 1.18E-59 5.10E-59 124.3310886

RAPH1 -1.049328756 1.549855428 -20.02529296 1.19E-59 5.11E-59 124.3292979

SOCS3 2.425963912 5.3636112 20.02457256 1.19E-59 5.14E-59 124.3226267

SFTA2 3.188264551 3.786932359 20.01952198 1.25E-59 5.38E-59 124.275855

RALGPS2 0.978868835 2.693717489 20.01922021 1.25E-59 5.40E-59 124.2730604

DMRTC1 -0.617070355 0.302331452 -20.01784132 1.27E-59 5.47E-59 124.2602908

MAPK8 -0.858611238 3.29018733 -20.01673368 1.28E-59 5.52E-59 124.2500332

C5AR1 1.84838707 3.176076166 20.01346532 1.32E-59 5.69E-59 124.2197653

ETF1 0.999347785 5.231229025 20.01016609 1.36E-59 5.87E-59 124.1892111

HTR2B 1.540939753 1.172359528 20.00919838 1.38E-59 5.92E-59 124.180249

HOXA1 0.847865821 0.634356788 20.00864715 1.38E-59 5.95E-59 124.1751441

PREX1 1.461509572 3.078012234 20.00830561 1.39E-59 5.96E-59 124.171981

PLEKHG4 -1.242239345 2.546675277 -20.00695789 1.41E-59 6.04E-59 124.1594996

RIMS1 -0.588987739 0.391656804 -20.00456306 1.44E-59 6.17E-59 124.1373205

OR6T1 -0.419671997 0.238894762 -19.99241225 1.61E-59 6.90E-59 124.024786

NRTN -1.655425975 2.754462134 -19.98912875 1.66E-59 7.12E-59 123.9943751

DAZAP1 -1.169160523 5.146658718 -19.98905774 1.66E-59 7.12E-59 123.9937173

ABCB10 0.828880258 3.070989201 19.98427723 1.73E-59 7.44E-59 123.9494408

GOT2 0.937039154 5.148948371 19.98154935 1.78E-59 7.63E-59 123.924175

HIBADH 0.984633349 4.766029178 19.98035347 1.80E-59 7.71E-59 123.9130987

SMIM10 1.101998013 2.528439244 19.97942859 1.81E-59 7.78E-59 123.9045323

ZDHHC7 1.29954155 5.253682418 19.97724474 1.85E-59 7.93E-59 123.8843051

CTSB 1.864687663 7.786182225 19.97403392 1.91E-59 8.17E-59 123.8545655

ZNF705E -0.201496244 0.15298758 -19.97260038 1.93E-59 8.28E-59 123.8412876

STMP1 0.847207363 4.219066751 19.97198086 1.94E-59 8.33E-59 123.8355493

ERGIC2 -1.180938093 4.480929363 -19.9718061 1.95E-59 8.34E-59 123.8339306

SLC17A4 -2.272216993 2.753014818 -19.97015887 1.98E-59 8.46E-59 123.8186732

ZNF521 1.399659203 1.814700411 19.96894323 2.00E-59 8.56E-59 123.8074134

UBE2D1 0.908104565 3.299856983 19.96800642 2.02E-59 8.63E-59 123.7987361

ARPIN -1.290581925 4.154504139 -19.96489885 2.07E-59 8.88E-59 123.7699519

FN1 3.38664103 6.991151031 19.96473961 2.08E-59 8.89E-59 123.768477

EXOSC1 -1.058914479 4.429596349 -19.96416933 2.09E-59 8.94E-59 123.7631946

P2RY2 1.47316285 1.478382729 19.96329707 2.11E-59 9.01E-59 123.7551152

NFKB1 0.96476395 4.221313094 19.96169897 2.14E-59 9.14E-59 123.7403124

SSU72 -1.218508604 5.794760244 -19.95539713 2.27E-59 9.68E-59 123.6819392

JAK3 1.446874364 2.354676216 19.95539196 2.27E-59 9.68E-59 123.6818913

HSPB2-C11orf52 -0.848440069 0.426446926 -19.95504073 2.27E-59 9.71E-59 123.6786379

PDLIM7 1.816940316 5.176160402 19.95295631 2.32E-59 9.90E-59 123.6593298

EDAR 1.676143213 1.090445082 19.95238605 2.33E-59 9.95E-59 123.6540474

SNRPB2 0.938134616 4.705911477 19.95227634 2.33E-59 9.96E-59 123.6530311

SCML2 -0.751074024 0.89537029 -19.9514626 2.35E-59 1.00E-58 123.6454934

HAT1 -1.008380156 3.935379426 -19.95096251 2.36E-59 1.01E-58 123.640861

ERCC3 -0.881635554 4.287853579 -19.95049468 2.37E-59 1.01E-58 123.6365274

TMEM109 1.107052805 6.204339828 19.94727572 2.44E-59 1.04E-58 123.6067095

STK10 1.009293823 3.334057549 19.94704407 2.45E-59 1.04E-58 123.6045637

NOLC1 1.111947133 5.138563738 19.94663306 2.46E-59 1.05E-58 123.6007564

DIP2C 0.917296318 2.92610744 19.94551365 2.48E-59 1.06E-58 123.5903869

NEK6 1.235887975 4.327465733 19.94464996 2.50E-59 1.07E-58 123.5823863

KATNAL1 0.918574057 1.730384422 19.94162221 2.57E-59 1.10E-58 123.554339

MOB3C -1.174490194 4.044786969 -19.93836426 2.65E-59 1.13E-58 123.5241589

GOLGA7 0.931364438 4.778543921 19.93630464 2.70E-59 1.15E-58 123.5050794

CAV1 1.955334404 4.605081258 19.93607573 2.71E-59 1.15E-58 123.5029588

ERP44 0.967796184 4.777850236 19.93094274 2.84E-59 1.21E-58 123.4554081

CTSW 1.726386947 1.700881168 19.93024476 2.86E-59 1.22E-58 123.4489421

ATP5MC3 -1.267374103 5.977868535 -19.92698721 2.95E-59 1.25E-58 123.4187645

FGF1 1.336083253 1.217399035 19.9227731 3.07E-59 1.30E-58 123.3797249

ZNF793 -0.732972466 1.173334663 -19.91399752 3.32E-59 1.41E-58 123.2984257

SPIN3 -0.732592057 2.331479877 -19.91043562 3.44E-59 1.46E-58 123.2654266

ATP1B3 1.415258173 5.606788682 19.90571021 3.59E-59 1.53E-58 123.2216476

MSL1 -1.131361142 5.292637348 -19.90354762 3.66E-59 1.56E-58 123.2016118

WDR70 -0.851331354 3.440083761 -19.90351924 3.66E-59 1.56E-58 123.2013489

CLMN -1.378848796 3.929952177 -19.9029745 3.68E-59 1.57E-58 123.196302

OSBPL8 1.144638771 3.310663147 19.9025357 3.70E-59 1.57E-58 123.1922366

AC012254.2 -1.646622002 0.80321734 -19.89758207 3.87E-59 1.64E-58 123.1463418

STKLD1 -0.740585612 0.785410218 -19.89668949 3.90E-59 1.66E-58 123.1380722

PLCB4 1.466216626 1.931821388 19.89258634 4.05E-59 1.72E-58 123.1000563

GLTP 0.907236062 4.622659313 19.89118512 4.11E-59 1.74E-58 123.0870737

NXF2B -0.304207659 0.155358475 -19.88981805 4.16E-59 1.77E-58 123.0744076

AKAP7 -2.114439742 3.777179144 -19.88838517 4.22E-59 1.79E-58 123.0611316

RAD18 0.735591069 1.762735239 19.88833768 4.22E-59 1.79E-58 123.0606916

DKK2 1.269267106 0.881009881 19.88683308 4.28E-59 1.81E-58 123.0467511

TXNDC9 0.876179308 3.767008411 19.88588284 4.31E-59 1.83E-58 123.0379468

ATPAF1 -1.032674776 4.263359974 -19.8845918 4.37E-59 1.85E-58 123.0259849

RDH13 -1.281268574 3.416787001 -19.8844049 4.37E-59 1.85E-58 123.0242532

RAB36 1.284682922 2.30017065 19.88247582 4.45E-59 1.89E-58 123.0063794

IQCD 0.953558807 1.232761639 19.8747196 4.78E-59 2.03E-58 122.9345136

PVR 1.241445502 4.594884029 19.87381659 4.82E-59 2.04E-58 122.9261465

SYDE1 1.183575001 3.152589183 19.87228319 4.89E-59 2.07E-58 122.9119384

SLC26A6 -1.453320164 3.662441187 -19.87166955 4.92E-59 2.08E-58 122.9062526

HLA-DMA 1.883705002 5.296527876 19.87095163 4.95E-59 2.10E-58 122.8996004

TBC1D9 1.296998358 3.371601845 19.86922238 5.03E-59 2.13E-58 122.8835774

ERMP1 1.026563189 3.31151997 19.86348504 5.31E-59 2.25E-58 122.8304154

CH25H 1.879291395 2.01837303 19.86314591 5.33E-59 2.25E-58 122.827273

TIA1 -1.320381137 5.184640088 -19.86256745 5.35E-59 2.26E-58 122.8219129

TTC30A 0.845207413 1.815491773 19.8608466 5.44E-59 2.30E-58 122.8059673

LPIN2 1.330180468 4.031741702 19.85865397 5.55E-59 2.35E-58 122.78565

COIL 0.699495298 3.310687632 19.85845506 5.56E-59 2.35E-58 122.7838068

CPNE5 1.327282753 1.495471325 19.8581475 5.58E-59 2.36E-58 122.7809569

APOBEC3F 0.756805814 1.92899663 19.85756264 5.61E-59 2.37E-58 122.7755375

SLA2 1.137733827 1.018802217 19.8566104 5.66E-59 2.39E-58 122.7667137

PRPS1 1.100815937 4.465114892 19.85429156 5.78E-59 2.44E-58 122.7452266

GNB1 1.161541232 6.445632204 19.85398638 5.80E-59 2.45E-58 122.7423987

TMEM117 0.729179638 2.070988852 19.85259124 5.87E-59 2.48E-58 122.7294707

HSPBAP1 -0.780960946 2.712484825 -19.85202069 5.90E-59 2.49E-58 122.7241838

ARPC1B 1.509322025 6.419328625 19.84778421 6.14E-59 2.59E-58 122.6849264

MYLK2 -2.010562977 1.785976901 -19.845719 6.26E-59 2.64E-58 122.6657888

YPEL1 -0.805687834 1.366377393 -19.84295631 6.42E-59 2.71E-58 122.6401879

GDF9 -0.608161268 0.651916691 -19.84201293 6.48E-59 2.73E-58 122.6314458

DAD1 1.3966864 7.879442545 19.84127687 6.52E-59 2.75E-58 122.6246249

INO80C -1.026688693 3.098419138 -19.84015695 6.59E-59 2.78E-58 122.6142468

AC069503.2 -0.677544584 0.570583532 -19.83710598 6.78E-59 2.86E-58 122.5859738

WSB1 -1.471638439 5.412929551 -19.83693849 6.79E-59 2.86E-58 122.5844218

USP1 0.869730354 3.794174897 19.83275599 7.06E-59 2.97E-58 122.5456625

PTPN9 0.764714489 3.436712588 19.83259119 7.07E-59 2.98E-58 122.5441353

CTH -1.516848896 2.445429194 -19.8319303 7.11E-59 2.99E-58 122.5380108

CCDC167 1.242118657 4.42932306 19.82848165 7.34E-59 3.09E-58 122.5060516

PCDHB10 0.892313213 1.289836078 19.82843619 7.35E-59 3.09E-58 122.5056303

FHL3 1.423938971 4.426105987 19.82280161 7.74E-59 3.26E-58 122.4534131

IFT43 -1.045959275 3.97583174 -19.82211185 7.79E-59 3.28E-58 122.4470208

NDN 1.484312703 4.100007777 19.82075583 7.89E-59 3.32E-58 122.434454

PAPOLA -1.149401519 5.352975973 -19.82023481 7.93E-59 3.33E-58 122.4296255

MMP2 2.968872718 6.631613332 19.82012619 7.93E-59 3.34E-58 122.4286188

FAM110A 1.245931472 3.044660455 19.81987058 7.95E-59 3.34E-58 122.4262499

APOC1 2.890915705 4.883162655 19.81701548 8.17E-59 3.43E-58 122.3997902

ACSM6 -1.662470938 0.880434058 -19.81683276 8.18E-59 3.44E-58 122.3980969

BBX 0.887147139 3.246599606 19.81076894 8.65E-59 3.64E-58 122.3418994

PSMB9 1.696388855 4.17178063 19.80211043 9.38E-59 3.94E-58 122.2616533

ZNF304 0.820303291 2.088146921 19.79978125 9.58E-59 4.02E-58 122.2400663

KRT1 -1.41871394 1.002091679 -19.79144144 1.04E-58 4.35E-58 122.162771

TAF9B 1.001193525 3.55552416 19.77741001 1.18E-58 4.95E-58 122.0327196

ARHGEF7 -0.918042544 4.059455032 -19.77370886 1.22E-58 5.12E-58 121.9984142

MAIP1 0.831332412 3.659696129 19.77298254 1.23E-58 5.15E-58 121.991682

ABCF3 -1.064490943 5.12495868 -19.76921564 1.27E-58 5.34E-58 121.9567666

HMGB1 -1.259155666 6.381643195 -19.76789798 1.29E-58 5.40E-58 121.9445532

MROH6 2.18982935 2.83374597 19.76203819 1.36E-58 5.70E-58 121.8902377

RORC -1.935490784 4.126525431 -19.76016869 1.38E-58 5.80E-58 121.8729088

KIAA1549L 1.218073898 1.057913518 19.75977112 1.39E-58 5.82E-58 121.8692236

CMTM1 1.077048519 1.210582746 19.75801465 1.41E-58 5.91E-58 121.8529422

HEPN1 -0.288474956 0.140044521 -19.75332845 1.47E-58 6.17E-58 121.8095037

HIPK3 1.055450976 4.208907996 19.74784118 1.55E-58 6.49E-58 121.7586389

JCHAIN 4.325469303 4.81149655 19.7452988 1.59E-58 6.65E-58 121.7350717

LIPJ -0.47258956 0.283361176 -19.73817293 1.70E-58 7.10E-58 121.6690157

RAD9A -1.209636087 3.842321634 -19.73755472 1.71E-58 7.14E-58 121.6632849

PIGR 4.132488988 5.262174737 19.7349823 1.75E-58 7.31E-58 121.6394386

QPRT -1.91017223 3.904366855 -19.73472796 1.75E-58 7.33E-58 121.6370808

A2M 2.057860596 6.905222501 19.73325355 1.78E-58 7.43E-58 121.6234129

GULP1 -1.503587551 3.016023917 -19.73217829 1.79E-58 7.50E-58 121.6134451

TRAPPC2L -1.19100083 4.826114677 -19.72931398 1.84E-58 7.70E-58 121.5868923

IFI16 1.709549859 4.673082433 19.7285493 1.85E-58 7.75E-58 121.5798035

ITGB2 2.183858635 4.120375186 19.72841658 1.86E-58 7.76E-58 121.5785732

S100A4 3.253455319 6.844427029 19.72612153 1.90E-58 7.92E-58 121.5572973

CAMKMT -0.859689377 2.43865984 -19.724984 1.92E-58 8.01E-58 121.546752

RASA2 0.905568346 2.897414647 19.72237593 1.96E-58 8.20E-58 121.5225742

NT5C1A -0.809227285 0.508264391 -19.72123648 1.98E-58 8.29E-58 121.5120109

NOX4 1.471772922 1.445020733 19.71974469 2.01E-58 8.40E-58 121.4981813

ZNF609 0.766530767 2.961676308 19.71813949 2.04E-58 8.52E-58 121.4833002

TULP3 0.87898471 3.588587054 19.71632098 2.08E-58 8.67E-58 121.4664415

SPIN4 1.093641864 1.875471982 19.71623154 2.08E-58 8.67E-58 121.4656124

POLDIP3 0.944577857 5.217832495 19.71618625 2.08E-58 8.67E-58 121.4651925

ZNF84 -0.892849612 3.115956077 -19.71081707 2.19E-58 9.12E-58 121.4154164

PHF5A 0.937487717 4.819851613 19.71012864 2.20E-58 9.17E-58 121.4090341

TBC1D8 -1.429685822 4.596428893 -19.70803384 2.24E-58 9.35E-58 121.3896135

ECM2 1.549373374 2.151702258 19.70453231 2.32E-58 9.66E-58 121.3571511

NCBP2AS2 1.059069319 4.548176972 19.70148361 2.38E-58 9.93E-58 121.3288866

TMEM163 -1.74139741 3.643476988 -19.69183308 2.61E-58 1.09E-57 121.2394146

FAM155A 0.880589173 0.712068959 19.68278574 2.83E-58 1.18E-57 121.1555324

OR10G4 -0.577451861 0.293133687 -19.6821594 2.85E-58 1.19E-57 121.1497252

ZNF419 -0.81420253 2.002164535 -19.68187339 2.86E-58 1.19E-57 121.1470735

KRBOX1 -1.465369829 1.46426095 -19.67708292 2.99E-58 1.24E-57 121.1026577

LRP11 1.333489164 4.357553151 19.67480687 3.05E-58 1.27E-57 121.0815546

KIF18A 0.974053048 0.806557469 19.67314832 3.10E-58 1.29E-57 121.0661769

KIF3B 1.011895259 4.586512582 19.66996871 3.19E-58 1.33E-57 121.0366957

ZNF778 -0.981405776 1.668834053 -19.66828037 3.24E-58 1.35E-57 121.0210414

SHROOM4 0.867038938 1.26930027 19.65962966 3.51E-58 1.46E-57 120.9408309

RHOT2 -1.436251235 5.688630355 -19.65880057 3.54E-58 1.47E-57 120.9331434

AC099489.1 -0.593654461 0.640826233 -19.65815467 3.56E-58 1.48E-57 120.9271544

ANKRD13C 0.70788822 3.042748169 19.6533741 3.72E-58 1.55E-57 120.8828273

YOD1 0.960826696 2.31390723 19.65119976 3.80E-58 1.58E-57 120.8626657

UBLCP1 0.853716054 4.138943419 19.64974089 3.85E-58 1.60E-57 120.8491384

HBG2 -1.025799462 0.551929817 -19.64903497 3.88E-58 1.61E-57 120.8425927

ZNF131 -0.924011847 3.144028332 -19.64894936 3.88E-58 1.61E-57 120.8417989

DZIP1 -1.501999195 3.453006989 -19.64447554 4.04E-58 1.68E-57 120.8003149

CCNDBP1 -1.039025005 4.57874479 -19.63950298 4.23E-58 1.76E-57 120.7542056

RRP1B 0.793267682 3.466765138 19.63859438 4.27E-58 1.77E-57 120.7457803

GYS2 -0.490642922 0.329379781 -19.63409284 4.45E-58 1.85E-57 120.704038

OR2I1P 2.896439908 3.111085492 19.6252836 4.83E-58 2.00E-57 120.6223491

ZNF843 -0.708523322 1.090253616 -19.62476503 4.85E-58 2.01E-57 120.6175403

ACSM2B -0.506885112 0.282194878 -19.62353361 4.91E-58 2.04E-57 120.606121

PKHD1L1 -0.78831428 0.500087043 -19.62316182 4.93E-58 2.04E-57 120.6026733

CD180 1.332099173 0.994484623 19.62279397 4.94E-58 2.05E-57 120.5992622

TBCK -1.035906293 2.885193274 -19.62079698 5.04E-58 2.09E-57 120.5807435

EDA -1.024708782 1.57533734 -19.6192247 5.11E-58 2.12E-57 120.5661632

ERO1A 1.509079024 4.760745396 19.61638174 5.25E-58 2.17E-57 120.5397992

ZNF37A -0.852303921 2.847845649 -19.61275996 5.43E-58 2.25E-57 120.5062126

FAM214B 1.209331109 3.335919266 19.6120018 5.46E-58 2.26E-57 120.4991819

RSAD2 1.768591502 2.305428073 19.61179613 5.47E-58 2.27E-57 120.4972746

EEF1AKNMT 0.844404632 4.030752785 19.60633192 5.76E-58 2.38E-57 120.4466014

CPA2 -7.110681191 8.454854509 -19.60544029 5.81E-58 2.40E-57 120.4383326

TFAP2A 1.542094975 1.318267083 19.60333425 5.92E-58 2.45E-57 120.4188017

GAL3ST3 -0.585634959 0.359852781 -19.60204191 5.99E-58 2.48E-57 120.4068168

MBP -1.164499677 3.258087076 -19.60076442 6.06E-58 2.51E-57 120.3949696

NDUFB6 1.011941386 4.990322635 19.60010002 6.10E-58 2.52E-57 120.388808

ARRDC2 1.368386199 4.56878499 19.59892909 6.17E-58 2.55E-57 120.3779489

MARS -1.430709434 5.498644047 -19.59654143 6.31E-58 2.61E-57 120.3558059

CDH17 3.30044492 2.193838596 19.59528239 6.38E-58 2.64E-57 120.3441295

RBM43 0.883755364 2.306036071 19.5934773 6.49E-58 2.68E-57 120.327389

ATP5PD -1.348160041 6.655586595 -19.59127471 6.62E-58 2.73E-57 120.306962

ERCC1 -1.059143336 4.861613315 -19.58868056 6.78E-58 2.80E-57 120.2829035

TMED7-TICAM2 -0.80446696 0.648420361 -19.58774511 6.84E-58 2.82E-57 120.2742279

SNRNP35 -1.002154601 3.668794852 -19.5868079 6.90E-58 2.85E-57 120.265536

PIM1 1.804657292 4.809432064 19.58680143 6.90E-58 2.85E-57 120.2654761

COA7 0.893254258 2.906866325 19.57844184 7.46E-58 3.08E-57 120.1879463

AP2A2 -1.073603603 4.883445038 -19.5775298 7.52E-58 3.10E-57 120.1794876

EDEM2 0.993690221 5.040301193 19.57641226 7.60E-58 3.13E-57 120.169123

CYGB 1.440938233 4.168998676 19.57462115 7.73E-58 3.19E-57 120.1525112

KITLG 1.613938254 2.940529342 19.57165442 7.94E-58 3.27E-57 120.1249961

SH2B2 1.042646271 1.989060521 19.57135148 7.97E-58 3.28E-57 120.1221864

DEPDC1 1.026911504 0.727550654 19.56961417 8.10E-58 3.34E-57 120.1060734

VEGFA -1.867503734 5.773949616 -19.56618368 8.36E-58 3.44E-57 120.0742565

MUTYH -1.085224004 3.615582637 -19.56420818 8.51E-58 3.51E-57 120.0559342

ST6GALNAC4 -1.439483075 5.027231375 -19.56177561 8.71E-58 3.58E-57 120.0333724

TRIM29 3.025303025 3.079418105 19.55690208 9.11E-58 3.75E-57 119.9881706

NT5C3B -1.287160152 5.099774113 -19.55279827 9.46E-58 3.89E-57 119.9501075

EHD4 1.063714103 4.204585401 19.5517524 9.55E-58 3.93E-57 119.9404069

C1orf52 -0.882946722 3.716845067 -19.54941254 9.76E-58 4.02E-57 119.9187043

AC090004.1 0.680973444 0.459839423 19.54863165 9.83E-58 4.05E-57 119.9114613

CENPF 1.860690775 1.639432844 19.54596419 1.01E-57 4.15E-57 119.8867199

ZNF845 0.689033975 1.866311976 19.54499898 1.02E-57 4.18E-57 119.8777673

PSEN2 -1.427771126 4.735002484 -19.54450914 1.02E-57 4.20E-57 119.8732239

TMEM161B -1.091808724 2.500374007 -19.53989494 1.07E-57 4.38E-57 119.8304254

SMIM27 -1.181544886 2.77098192 -19.53822056 1.08E-57 4.45E-57 119.8148947

OR2A7 -1.338176947 1.68259375 -19.53627653 1.10E-57 4.53E-57 119.7968628

CLCF1 1.736909778 3.422388274 19.53232366 1.14E-57 4.70E-57 119.7601976

RAB34 1.653006089 4.142527394 19.52363851 1.24E-57 5.09E-57 119.6796362

PSCA 5.124934899 3.628302699 19.51989404 1.28E-57 5.27E-57 119.6449029

SLC25A16 -0.84815075 2.975543812 -19.51904479 1.29E-57 5.31E-57 119.6370253

MRPL49 0.862463637 4.89835586 19.50018114 1.54E-57 6.33E-57 119.4620421

MRC1 2.418971062 2.972706444 19.49971855 1.55E-57 6.35E-57 119.4577509

JAK2 1.032403404 2.584160243 19.49446246 1.63E-57 6.67E-57 119.4089927

GTDC1 -0.836260513 2.22763564 -19.49417509 1.63E-57 6.68E-57 119.4063268

FLT3LG -0.984475421 1.9014741 -19.49351558 1.64E-57 6.72E-57 119.4002089

PPP1R11 1.002652884 5.552879985 19.48511745 1.77E-57 7.27E-57 119.3223019

CCDC40 -1.111138817 1.802988551 -19.4847265 1.78E-57 7.29E-57 119.3186751

MAFB 2.175272666 4.183471641 19.48467097 1.78E-57 7.29E-57 119.3181599

SLTM -0.889163259 5.033430247 -19.48112758 1.84E-57 7.54E-57 119.2852885

CHMP2B 1.094414368 4.919286797 19.48009097 1.86E-57 7.61E-57 119.2756719

PDE1C -0.787786878 0.933915355 -19.479425 1.87E-57 7.65E-57 119.2694938

TRAPPC10 -0.978975626 2.823438858 -19.47687079 1.91E-57 7.84E-57 119.2457984

CDAN1 -0.738781167 2.925295969 -19.47482351 1.95E-57 7.98E-57 119.2268057

GALNT11 -1.274457382 4.435907613 -19.4745585 1.96E-57 8.00E-57 119.2243472

GPAT3 1.34727544 1.773534457 19.4679138 2.08E-57 8.51E-57 119.1627034

ABCA6 -1.275302403 1.732939534 -19.46754131 2.09E-57 8.54E-57 119.1592477

DNAH9 -0.780104791 0.604386725 -19.46635772 2.11E-57 8.63E-57 119.1482673

GOLGA5 0.964583663 4.900209071 19.4661812 2.11E-57 8.64E-57 119.1466297

ABHD15 1.121303844 3.45288922 19.46337856 2.17E-57 8.87E-57 119.1206288

RIPK2 1.178759448 3.5320991 19.46178777 2.20E-57 9.00E-57 119.1058705

YES1 1.094458293 4.214823592 19.46012234 2.24E-57 9.14E-57 119.0904196

SLC31A1 1.046858681 3.727771432 19.45558535 2.33E-57 9.53E-57 119.0483278

TOE1 0.784175935 2.859255084 19.44994314 2.46E-57 1.00E-56 118.9959818

CNDP2 -1.312175177 5.626637692 -19.44944165 2.47E-57 1.01E-56 118.9913292

PLBD1 2.026506122 5.045363827 19.44226511 2.64E-57 1.08E-56 118.9247471

WNT8B -0.371579272 0.295887891 -19.44056636 2.68E-57 1.09E-56 118.9089864

CCSER2 0.824474601 3.087479625 19.43710758 2.77E-57 1.13E-56 118.8768962

CENPU 1.354825031 2.083452955 19.43336724 2.87E-57 1.17E-56 118.8421934

MYLK3 -0.217485584 0.175299191 -19.43215543 2.90E-57 1.18E-56 118.8309502

CSF2 1.597064865 0.848148809 19.42741393 3.03E-57 1.24E-56 118.7869581

CHST3 1.35512201 2.845885265 19.4246435 3.11E-57 1.27E-56 118.7612535

HSD17B7 -1.059090852 2.980778586 -19.42113919 3.21E-57 1.31E-56 118.7287396

C1QB 2.686692058 6.250980476 19.42050648 3.23E-57 1.32E-56 118.722869

LMNA 1.501228912 6.828967964 19.42045796 3.23E-57 1.32E-56 118.7224189

NDP 1.070632707 0.642610426 19.42024778 3.24E-57 1.32E-56 118.7204688

LAMA1 -1.631335745 1.408591522 -19.41968397 3.25E-57 1.33E-56 118.7152375

USP47 -0.932833181 4.445317645 -19.41486203 3.40E-57 1.39E-56 118.6704977

FOXA1 1.694948245 1.528560927 19.41462891 3.41E-57 1.39E-56 118.6683347

THTPA -0.902245557 2.860744908 -19.41191697 3.50E-57 1.42E-56 118.6431721

CSNK1G1 0.597195763 2.223574415 19.40949924 3.58E-57 1.46E-56 118.620739

FOLR2 2.141729668 3.180522631 19.40523833 3.72E-57 1.51E-56 118.5812037

ABL1 1.037643314 4.655212808 19.40327212 3.79E-57 1.54E-56 118.5629599

FGF18 1.183544464 1.091950387 19.4025297 3.81E-57 1.55E-56 118.5560712

TRMT2B 0.696841767 3.050333705 19.39829771 3.97E-57 1.61E-56 118.5168035

TRPS1 1.100055033 1.558605811 19.39722252 4.01E-57 1.63E-56 118.506827

PPDPF 2.010199151 8.499207514 19.39623265 4.04E-57 1.64E-56 118.4976421

C18orf54 0.569360304 0.731686749 19.3947109 4.10E-57 1.67E-56 118.4835221

MZT2B -2.105645086 6.730814283 -19.39415427 4.12E-57 1.68E-56 118.4783572

GPSM1 -1.642362189 3.953600817 -19.39205858 4.20E-57 1.71E-56 118.4589114

BBS2 -0.937247903 3.717584033 -19.38451679 4.51E-57 1.83E-56 118.3889309

VSIG1 3.472644075 2.141018087 19.38449712 4.51E-57 1.83E-56 118.3887483

FBXO30 0.711092908 1.834866842 19.3805544 4.68E-57 1.90E-56 118.3521633

ZHX2 0.963834564 4.043325432 19.37405558 4.97E-57 2.02E-56 118.2918589

MED1 0.783810634 3.229239355 19.37345834 5.00E-57 2.03E-56 118.2863169

PRRX1 1.981187383 2.830312591 19.37308573 5.01E-57 2.03E-56 118.2828593

SERINC2 2.224828483 5.837044858 19.37127896 5.10E-57 2.07E-56 118.2660937

MYO15B -1.9388329 4.822309302 -19.37005467 5.16E-57 2.09E-56 118.254733

NXF1 -1.241966571 5.444120958 -19.36997372 5.16E-57 2.09E-56 118.2539817

IKBIP 1.034085604 2.729325368 19.36982748 5.17E-57 2.10E-56 118.2526248

FAM3A -1.219994955 5.004115471 -19.36867145 5.22E-57 2.12E-56 118.2418975

ANKLE2 -1.04323565 4.199228071 -19.36544807 5.38E-57 2.18E-56 118.2119861

FBXL19 0.996963823 3.326982764 19.36512111 5.40E-57 2.19E-56 118.2089521

SGO1 0.905647199 0.763845769 19.36414936 5.45E-57 2.21E-56 118.1999347

C2 1.611969018 2.380476791 19.36343619 5.48E-57 2.22E-56 118.1933168

RAB26 -2.413354982 3.913268148 -19.36129604 5.59E-57 2.26E-56 118.1734571

MTMR6 0.887248535 3.475877671 19.35628325 5.86E-57 2.37E-56 118.12694

POLE3 0.974158065 4.937816453 19.35157807 6.12E-57 2.48E-56 118.0832771

ZFYVE21 -1.003671897 4.177858957 -19.35141409 6.13E-57 2.48E-56 118.0817553

UIMC1 -0.921738047 4.077934299 -19.35115949 6.14E-57 2.49E-56 118.0793927

PGA5 -1.884418603 1.026274072 -19.35020649 6.20E-57 2.51E-56 118.070549

TLR6 0.892655803 0.956247175 19.34752119 6.36E-57 2.57E-56 118.0456298

NIP7 0.954007426 3.746342142 19.34627806 6.43E-57 2.60E-56 118.0340936

SDK1 -1.89811112 2.913172951 -19.34610163 6.44E-57 2.60E-56 118.0324564

THAP10 0.716013876 1.35990828 19.34590731 6.45E-57 2.61E-56 118.0306531

GNG8 1.167578088 0.678694878 19.3431287 6.62E-57 2.68E-56 118.0048677

KXD1 -1.071505269 5.536241229 -19.343042 6.63E-57 2.68E-56 118.0040632

CALHM6 1.732572379 2.103166524 19.34179091 6.70E-57 2.71E-56 117.992453

RFK 1.0348384 3.922798695 19.34168737 6.71E-57 2.71E-56 117.9914922

LSM8 -0.899416649 3.742476083 -19.33366082 7.23E-57 2.92E-56 117.9170049

ZC3HAV1L 0.959373074 1.832049359 19.33125236 7.39E-57 2.98E-56 117.8946539

MTDH 1.153814389 4.83264811 19.33119144 7.40E-57 2.99E-56 117.8940885

CST4 2.045412103 1.059901787 19.3310418 7.41E-57 2.99E-56 117.8926998

CCKBR -2.229622351 1.762098358 -19.33103113 7.41E-57 2.99E-56 117.8926008

NOSTRIN -1.572248845 3.99843026 -19.32864592 7.57E-57 3.05E-56 117.8704654

ANO2 -0.633650858 0.638350686 -19.32668592 7.71E-57 3.11E-56 117.852276

CFAP43 -0.949379418 1.143558942 -19.32625178 7.74E-57 3.12E-56 117.848247

AMBRA1 0.693388899 3.366073667 19.32381207 7.92E-57 3.19E-56 117.8256057

FGD6 1.308019888 2.594153115 19.3232899 7.96E-57 3.21E-56 117.8207596

WNT7A 2.278709646 1.30780638 19.32005731 8.20E-57 3.30E-56 117.7907598

MCM10 0.914208101 0.720483628 19.3176486 8.39E-57 3.38E-56 117.7684059

MACROD1 -1.631829715 4.757724654 -19.31667974 8.46E-57 3.41E-56 117.7594143

COL14A1 2.407977615 3.56880012 19.31354809 8.71E-57 3.51E-56 117.7303508

PRKAR2A 0.872491979 3.519058462 19.31288124 8.77E-57 3.53E-56 117.7241621

CSF2RA 1.577561053 2.01487656 19.31121378 8.90E-57 3.58E-56 117.708687

CDK5RAP1 -0.961682736 3.962206403 -19.31006933 9.00E-57 3.62E-56 117.6980657

MAGED2 1.298452962 6.431746566 19.30673246 9.28E-57 3.73E-56 117.6670972

CD1A 1.514922283 0.837537069 19.30604885 9.34E-57 3.76E-56 117.6607528

PUS10 -0.695247115 1.863586259 -19.30596235 9.35E-57 3.76E-56 117.65995

XIAP 0.81879393 3.741368975 19.30174666 9.72E-57 3.91E-56 117.6208251

KCND2 1.178001563 0.934207257 19.30154264 9.74E-57 3.92E-56 117.6189316

ABAT -2.203095296 3.899067714 -19.29688476 1.02E-56 4.09E-56 117.5757024

PDP1 1.15531244 3.340178305 19.29217478 1.06E-56 4.27E-56 117.5319891

TRNP1 1.910702925 4.984942913 19.29168568 1.07E-56 4.29E-56 117.5274498

B2M 2.178738335 10.53100351 19.28460505 1.14E-56 4.58E-56 117.4617337

KAT5 -0.948566737 4.569260537 -19.28381896 1.15E-56 4.61E-56 117.4544379

ACLY 1.23283368 5.590626158 19.2808027 1.18E-56 4.74E-56 117.4264434

SP100 -1.290136088 4.567695627 -19.28050495 1.18E-56 4.75E-56 117.4236798

PLAAT5 -1.798330571 2.050050141 -19.27953735 1.19E-56 4.79E-56 117.4146993

THADA -0.814320457 3.30757786 -19.27806948 1.21E-56 4.86E-56 117.4010756

SORCS2 1.420381721 1.554761641 19.27661939 1.23E-56 4.92E-56 117.3876168

CCNT2 -0.919759518 3.80017218 -19.27228048 1.28E-56 5.13E-56 117.3473458

MYL9 2.314727152 6.987197664 19.27045876 1.30E-56 5.21E-56 117.3304377

SLC7A6 -0.929324478 2.307892344 -19.26979327 1.31E-56 5.24E-56 117.3242609

FA2H 2.121411512 3.742643601 19.26899237 1.32E-56 5.28E-56 117.3168274

CAMKK1 -1.276239869 2.785472581 -19.26615386 1.35E-56 5.42E-56 117.2904818

NKX3-2 1.25231133 1.010528187 19.26497995 1.37E-56 5.48E-56 117.2795861

MT-CO2 2.351718938 13.91240698 19.26418242 1.38E-56 5.52E-56 117.2721838

E2F2 0.877111695 0.667455974 19.26272569 1.40E-56 5.59E-56 117.2586631

MAP2K1 0.880404527 4.65539333 19.260792 1.42E-56 5.69E-56 117.2407153

LGR6 1.984405484 1.390745854 19.25961176 1.44E-56 5.75E-56 117.2297608

MAP3K13 -0.898889092 3.434164456 -19.25721117 1.47E-56 5.88E-56 117.2074792

CELA3A -8.0752653 10.21638514 -19.25508212 1.50E-56 6.00E-56 117.1877181

PPM1H 1.118873562 2.552675474 19.25284956 1.53E-56 6.12E-56 117.166996

FBXO32 1.718070679 3.919941426 19.2525166 1.54E-56 6.14E-56 117.1639056

SNX13 -0.799267895 2.862075219 -19.24999354 1.57E-56 6.29E-56 117.1404869

SLC39A14 -1.786979136 6.382762276 -19.2468027 1.62E-56 6.47E-56 117.1108701

BBS4 -0.868961466 3.707991973 -19.2453609 1.64E-56 6.56E-56 117.0974874

RAB3IL1 1.327279892 3.169604629 19.2400993 1.72E-56 6.89E-56 117.0486494

AP4B1 -1.100079883 3.830590433 -19.2398087 1.73E-56 6.90E-56 117.0459521

LAP3 1.228258478 5.10607638 19.23717664 1.77E-56 7.07E-56 117.0215212

HS3ST3B1 1.008908847 1.343452781 19.23482269 1.81E-56 7.23E-56 116.9996716

INSL3 0.831226752 0.768264684 19.23104546 1.87E-56 7.48E-56 116.9646109

SDS 1.966372317 1.83720083 19.22735847 1.94E-56 7.74E-56 116.9303875

ULBP2 1.597947737 1.366619583 19.22335306 2.01E-56 8.04E-56 116.8932082

EPS8 1.409664191 4.447320057 19.21542317 2.17E-56 8.65E-56 116.8195999

USP14 0.872151669 4.129532109 19.21499255 2.18E-56 8.68E-56 116.8156027

PINK1 -1.17296026 4.446755251 -19.21306052 2.21E-56 8.84E-56 116.7976687

BIRC3 2.144856816 3.887160812 19.20903113 2.30E-56 9.17E-56 116.7602657

ZDBF2 -1.01327922 1.797665871 -19.20450114 2.40E-56 9.56E-56 116.7182156

RGS16 2.076014668 3.693784623 19.20277015 2.44E-56 9.72E-56 116.7021474

SRP68 -1.138207891 5.236827181 -19.20249369 2.44E-56 9.74E-56 116.6995811

ATP13A5 -0.291476849 0.199731739 -19.20209927 2.45E-56 9.77E-56 116.6959198

DRICH1 -0.800494829 0.791840046 -19.1961466 2.59E-56 1.03E-55 116.6406627

OR8D4 -0.279144092 0.139740585 -19.19521284 2.61E-56 1.04E-55 116.6319948

MYCL -2.121761751 2.989326426 -19.19506474 2.62E-56 1.04E-55 116.6306201

ATP7A 0.776870877 2.015185311 19.19340522 2.66E-56 1.06E-55 116.615215

MIOS -0.904063866 3.522471994 -19.1891004 2.77E-56 1.10E-55 116.5752539

C12orf57 -1.566399892 6.855225188 -19.18901362 2.77E-56 1.10E-55 116.5744484

RCCD1 -1.164612895 3.222933571 -19.18878341 2.77E-56 1.10E-55 116.5723113

SLC25A12 0.926462169 2.81828635 19.18746494 2.81E-56 1.12E-55 116.560072

H1F0 1.728013457 7.308938843 19.18709818 2.82E-56 1.12E-55 116.5566675

CAPN10 -1.104007649 3.435714021 -19.1852417 2.87E-56 1.14E-55 116.5394338

GOLGA8J -0.267908078 0.133415808 -19.18392435 2.90E-56 1.15E-55 116.5272048

ACOT4 1.029764499 1.705779604 19.18089334 2.99E-56 1.19E-55 116.4990679

PCDH12 1.30976463 1.921114009 19.18083051 2.99E-56 1.19E-55 116.4984847

PTCH1 -1.076612997 2.55409371 -19.18005821 3.01E-56 1.20E-55 116.4913154

NXT2 0.907851444 2.448747139 19.17130302 3.26E-56 1.30E-55 116.4100397

U2SURP -0.98205353 4.480747305 -19.16743348 3.38E-56 1.34E-55 116.3741178

ACTA2 2.601227884 7.116514653 19.16538225 3.45E-56 1.37E-55 116.3550756

C3orf14 -1.264676949 2.74233039 -19.16381568 3.50E-56 1.39E-55 116.3405327

AAGAB 0.862192929 4.413984924 19.1630517 3.52E-56 1.40E-55 116.3334404

PIGQ -1.377683083 4.853146771 -19.1630462 3.52E-56 1.40E-55 116.3333894

ECHDC3 -1.841396003 3.461280302 -19.15499519 3.80E-56 1.51E-55 116.2586485

HAPLN4 -0.449978856 0.219228201 -19.15167588 3.92E-56 1.55E-55 116.2278336

EIF2B2 -0.924671398 4.096773425 -19.15164628 3.92E-56 1.55E-55 116.2275589

SAPCD1 -1.075053255 1.327799273 -19.15090186 3.94E-56 1.56E-55 116.2206479

FAM118B 0.764019781 3.167573145 19.14793878 4.05E-56 1.61E-55 116.19314

PCMTD1 -1.221089667 5.012640624 -19.1439615 4.21E-56 1.67E-55 116.1562164

NOG 1.320798975 0.907010212 19.14337262 4.23E-56 1.68E-55 116.1507495

UGGT2 -0.931368561 3.188074598 -19.141167 4.32E-56 1.71E-55 116.1302732

GPR65 1.041724081 1.026161646 19.13969121 4.38E-56 1.73E-55 116.1165724

PRPF4 0.855717099 3.894459025 19.13742885 4.47E-56 1.77E-55 116.0955692

CEP152 -0.650480403 1.488606785 -19.13513424 4.57E-56 1.81E-55 116.0742665

COPA 1.101237496 5.842136911 19.12992577 4.79E-56 1.90E-55 116.025912

ROBO3 -1.196207723 2.333551844 -19.12961062 4.81E-56 1.90E-55 116.0229861

CDH6 1.219466907 1.350976907 19.12682131 4.93E-56 1.95E-55 115.9970904

MIA3 -1.166843062 4.774494171 -19.12678981 4.93E-56 1.95E-55 115.9967979

PLGRKT 0.932621196 3.883737208 19.1260421 4.97E-56 1.97E-55 115.9898563

GTF2F2 0.806388271 3.605328972 19.12460078 5.03E-56 1.99E-55 115.9764751

LRCH2 0.917978324 0.916590517 19.12440249 5.04E-56 2.00E-55 115.9746342

CCDC13 -0.585904346 0.731774047 -19.12129669 5.19E-56 2.05E-55 115.9458

ANKRD28 -0.954447339 3.727646908 -19.11715492 5.39E-56 2.13E-55 115.9073476

KIF6 -0.402960103 0.375258577 -19.11412765 5.55E-56 2.19E-55 115.8792421

CD207 1.65367954 1.051056777 19.11282742 5.62E-56 2.22E-55 115.8671706

STX12 0.945384522 4.645276378 19.1072874 5.91E-56 2.34E-55 115.8157361

MT-ND4 2.289382149 13.68891229 19.10568295 6.00E-56 2.37E-55 115.80084

EFNA3 1.836399715 2.124056881 19.10534202 6.02E-56 2.38E-55 115.7976747

ZNF875 -0.975163223 3.834548618 -19.10347876 6.13E-56 2.42E-55 115.7803757

S100PBP -0.773421274 2.965320977 -19.09826143 6.43E-56 2.54E-55 115.7319363

TSPAN8 3.146636855 6.367560723 19.09727617 6.49E-56 2.56E-55 115.7227887

ABHD6 0.899227136 2.159890225 19.09453143 6.66E-56 2.63E-55 115.6973055

KIF21B 0.987570172 1.336838916 19.09220616 6.80E-56 2.68E-55 115.6757167

HLA-DQB1 2.794811322 4.427138199 19.09010288 6.94E-56 2.74E-55 115.6561889

NFYC -0.919923832 4.432828085 -19.0893613 6.98E-56 2.75E-55 115.6493037

PPP1R15B 0.993578244 4.618476978 19.08772476 7.09E-56 2.80E-55 115.6341092

ST3GAL1 1.440521228 4.06025685 19.08473072 7.29E-56 2.87E-55 115.6063109

FAM49A 1.252226096 2.103712169 19.0844401 7.31E-56 2.88E-55 115.6036126

KNTC1 -1.042140361 2.478247657 -19.07974168 7.64E-56 3.01E-55 115.5599897

TMEM91 -1.477076697 3.167425211 -19.07512224 7.97E-56 3.14E-55 115.5170997

MCM3AP -0.945477365 4.497467358 -19.07161417 8.23E-56 3.24E-55 115.4845282

RRM2B 1.009481698 3.288776353 19.07071156 8.30E-56 3.27E-55 115.4761477

EFHD2 1.565295945 6.489497447 19.06588377 8.68E-56 3.42E-55 115.4313226

C9orf85 -0.722963917 2.569202732 -19.06013873 9.16E-56 3.61E-55 115.3779808

RNF128 1.761275631 4.370726988 19.05970176 9.20E-56 3.62E-55 115.3739235

VCL 1.254523952 5.027295813 19.05787154 9.36E-56 3.68E-55 115.35693

C6orf223 1.92534116 1.709580937 19.05218235 9.86E-56 3.88E-55 115.3041059

LRRC3 0.862303463 1.255014098 19.05080207 9.99E-56 3.93E-55 115.2912901

RNASE10 -0.396043321 0.293304986 -19.04955942 1.01E-55 3.98E-55 115.2797519

PTPN20 -1.282953133 1.505927239 -19.0481674 1.02E-55 4.03E-55 115.266827

PHACTR2 0.976308749 2.329298856 19.0473409 1.03E-55 4.06E-55 115.2591529

AATK -1.747595013 3.486099984 -19.04435915 1.06E-55 4.17E-55 115.2314669

LRPAP1 -1.407747373 5.860594392 -19.04299861 1.07E-55 4.22E-55 115.2188342

RPP30 -0.81467626 3.60600922 -19.04278238 1.08E-55 4.23E-55 115.2168265

JADE1 -1.401538183 4.261158631 -19.0380483 1.12E-55 4.42E-55 115.1728696

ANPEP -3.224525337 7.005845022 -19.03638115 1.14E-55 4.49E-55 115.1573898

CCDC170 0.883932269 0.805060054 19.03572613 1.15E-55 4.51E-55 115.1513078

AC124312.1 -0.360637234 0.177284999 -19.03414445 1.17E-55 4.58E-55 115.1366215

RASGEF1B 0.879109744 1.801831299 19.02948014 1.22E-55 4.78E-55 115.093312

MOB3B 1.061382532 2.485613421 19.02717079 1.24E-55 4.88E-55 115.0718689

CYP4V2 -1.170481767 3.726815398 -19.02706852 1.25E-55 4.89E-55 115.0709192

C3orf52 -1.675295232 3.524459103 -19.02431732 1.28E-55 5.01E-55 115.0453733

SMUG1 -1.007130488 4.147600602 -19.01871383 1.35E-55 5.28E-55 114.9933425

FAM102A 1.369434035 5.4642806 19.01610527 1.38E-55 5.41E-55 114.9691207

UMAD1 -1.009097467 3.90598615 -19.01563739 1.38E-55 5.43E-55 114.9647762

FGFBP1 2.71184903 1.862466366 19.01423504 1.40E-55 5.50E-55 114.9517548

KRT6B 2.768544762 2.065580043 19.01272727 1.42E-55 5.58E-55 114.9377542

PTPRR 1.593228187 1.146420465 19.00969789 1.46E-55 5.73E-55 114.9096248

MT-ND5 2.374115681 11.14640326 18.99879656 1.62E-55 6.34E-55 114.8083988

GPRIN1 1.615773168 1.501632062 18.99642545 1.66E-55 6.48E-55 114.7863814

TMEM59 -1.410047487 7.011335617 -18.99546304 1.67E-55 6.54E-55 114.7774446

CEP192 -0.963379128 3.027938005 -18.99032466 1.75E-55 6.86E-55 114.7297307

TSEN15 0.946655978 4.080997757 18.98477052 1.84E-55 7.22E-55 114.6781558

IRAK3 1.268330014 2.104766425 18.98421595 1.85E-55 7.26E-55 114.6730061

METTL5 -1.048839925 4.796556398 -18.97878777 1.95E-55 7.63E-55 114.6226004

RANBP9 0.883421056 4.441226339 18.97651307 1.99E-55 7.79E-55 114.6014776

HSPA12B 1.158203285 2.277816477 18.97649513 1.99E-55 7.79E-55 114.601311

ALOX5AP 2.128210187 4.45889054 18.97590613 2.00E-55 7.83E-55 114.5958415

DACT3 1.276883305 1.643229796 18.97393061 2.04E-55 7.98E-55 114.5774968

SV2A 1.489674188 1.776173048 18.97104773 2.10E-55 8.19E-55 114.5507263

IGSF11 -1.057497778 1.403570471 -18.96339693 2.25E-55 8.79E-55 114.4796802

CTLA4 1.326661971 0.953842364 18.96223327 2.27E-55 8.89E-55 114.4688743

TACC3 1.360023751 3.027250424 18.96091955 2.30E-55 8.99E-55 114.4566749

BST1 1.689591867 2.077253133 18.95892797 2.35E-55 9.16E-55 114.4381807

CCDC130 -1.275353612 4.860738434 -18.95685107 2.39E-55 9.34E-55 114.4188942

FAM83B 1.362524516 1.503338005 18.95551153 2.42E-55 9.45E-55 114.4064549

SMC3 0.924765566 4.398601989 18.95545441 2.42E-55 9.45E-55 114.4059245

SLC16A5 1.888994194 3.231795114 18.94600274 2.64E-55 1.03E-54 114.3181536

FLVCR2 1.088581983 1.379328551 18.94534507 2.66E-55 1.04E-54 114.3120463

SLC25A14 -0.93616814 3.284821844 -18.94048142 2.78E-55 1.09E-54 114.2668806

FAR2 1.231816952 1.78938328 18.93885703 2.83E-55 1.10E-54 114.2517958

EIF3H -1.32883286 6.784974042 -18.93671345 2.88E-55 1.12E-54 114.2318896

TMEM14C 1.09728123 6.531232732 18.92591693 3.19E-55 1.24E-54 114.1316277

SCARF2 1.615811491 3.140142009 18.92213952 3.30E-55 1.29E-54 114.0965486

ZNF202 -0.753840653 3.005080927 -18.91977178 3.37E-55 1.31E-54 114.0745603

FDX1 0.819241302 3.672130215 18.91965331 3.38E-55 1.32E-54 114.0734601

NEMP2 0.70027247 1.458856014 18.91941357 3.39E-55 1.32E-54 114.0712337

ARL5C -0.957675346 0.482888441 -18.91578065 3.50E-55 1.36E-54 114.0374961

LRRC7 -0.70282994 0.50562978 -18.91458149 3.54E-55 1.38E-54 114.0263599

FIBIN 2.053888547 3.075309611 18.91053288 3.68E-55 1.43E-54 113.9887617

LSM11 0.614735925 1.713287633 18.90975879 3.70E-55 1.44E-54 113.9815729

ERLIN1 0.91441252 3.863992647 18.90788329 3.77E-55 1.47E-54 113.9641557

ABL2 0.835903102 2.632445244 18.90714371 3.79E-55 1.48E-54 113.9572873

DNAAF1 -0.736684377 0.703298351 -18.90594359 3.84E-55 1.49E-54 113.9461422

PROX2 -0.305220892 0.314978873 -18.90436356 3.89E-55 1.51E-54 113.9314688

KANSL1L -1.011364633 3.107521028 -18.90360928 3.92E-55 1.52E-54 113.9244639

SMARCD3 -1.510300314 4.369114637 -18.90308913 3.94E-55 1.53E-54 113.9196334

FUCA2 1.019047103 5.161070019 18.89966341 4.07E-55 1.58E-54 113.8878194

ADAMTS6 0.760465316 0.687943278 18.89739975 4.15E-55 1.61E-54 113.8667972

LRRC8B 0.841907564 2.440623083 18.89674322 4.18E-55 1.62E-54 113.8607001

ZMYND8 0.929079664 3.74276204 18.89136145 4.39E-55 1.71E-54 113.8107203

ZNRF2 0.76428268 3.219157891 18.88774765 4.54E-55 1.76E-54 113.7771592

LIMS1 1.204918697 3.926965249 18.8858302 4.62E-55 1.80E-54 113.759352

DNAJC10 -1.19853856 4.572690048 -18.88142553 4.82E-55 1.87E-54 113.7184459

FOXRED1 -0.94392305 3.58601374 -18.8811124 4.83E-55 1.88E-54 113.7155378

CTTNBP2 -1.18562798 1.946145016 -18.87563252 5.08E-55 1.97E-54 113.664646

NPR3 1.754938766 1.571258686 18.87373906 5.17E-55 2.01E-54 113.6470613

SUCLG1 -1.116327632 5.572154764 -18.87165567 5.28E-55 2.05E-54 113.6277127

ROBO1 1.31316792 2.189173985 18.87080356 5.32E-55 2.06E-54 113.619799

ERO1B -2.818506588 5.230416553 -18.86879355 5.42E-55 2.10E-54 113.6011319

C5orf30 1.05115992 2.819787082 18.86781307 5.47E-55 2.12E-54 113.5920261

NPM2 -1.66912091 2.961846842 -18.86281682 5.73E-55 2.22E-54 113.5456251

DFFB -0.750505739 2.631750164 -18.85616977 6.09E-55 2.36E-54 113.4838926

CMPK1 1.257077476 6.298713234 18.85592328 6.11E-55 2.37E-54 113.4816035

KDELR2 1.308827367 6.828281211 18.85325425 6.26E-55 2.42E-54 113.4568155

CDC42BPA 0.97942082 3.755017697 18.85073213 6.41E-55 2.48E-54 113.4333919

ULBP3 0.985717155 0.986506321 18.84945259 6.48E-55 2.51E-54 113.4215085

ZFAND4 -0.646455801 1.914671543 -18.84945033 6.48E-55 2.51E-54 113.4214875

FOSL2 1.662217267 5.097422142 18.84558127 6.72E-55 2.60E-54 113.3855544

ZNF66 -0.736137086 0.912521358 -18.84465442 6.78E-55 2.62E-54 113.3769464

AP001931.1 0.840055564 0.491980864 18.83738782 7.25E-55 2.81E-54 113.3094588

PPT2 -0.885163797 3.295030835 -18.83657171 7.31E-55 2.83E-54 113.3018793

CALCRL 1.518881872 2.594594599 18.83293886 7.56E-55 2.92E-54 113.2681396

MNDA 1.9211963 2.398845066 18.8298537 7.78E-55 3.01E-54 113.2394864

KLHL13 -0.931401379 1.36858338 -18.82906075 7.84E-55 3.03E-54 113.2321218

SETD1A 0.655294238 3.418601749 18.82776543 7.93E-55 3.07E-54 113.2200916

SAT2 -1.45067977 6.279285637 -18.82643472 8.03E-55 3.10E-54 113.2077327

FAM217B 0.798187095 2.002719895 18.81286632 9.11E-55 3.52E-54 113.0817158

SRGAP3 -0.869589141 1.159854449 -18.80659438 9.65E-55 3.73E-54 113.0234644

NRIP3 0.916941986 0.880669868 18.80081535 1.02E-54 3.94E-54 112.9697908

CTF1 1.091520122 2.556429159 18.79761457 1.05E-54 4.05E-54 112.9400629

VDAC1 1.214785187 6.715901957 18.79470298 1.08E-54 4.16E-54 112.9130209

ACTRT3 0.780075786 1.080937879 18.79432564 1.08E-54 4.18E-54 112.9095163

PPP6C 0.84788962 4.532326544 18.78920708 1.13E-54 4.38E-54 112.8619764

DENND4B -1.069641903 4.421899455 -18.78557805 1.17E-54 4.53E-54 112.8282708

SELENOP -1.935960036 5.229884374 -18.78538651 1.18E-54 4.54E-54 112.8264918

FBXO44 -1.277034115 4.282135373 -18.7846967 1.18E-54 4.56E-54 112.8200851

MAP2K6 -1.21093875 2.26706921 -18.78404078 1.19E-54 4.59E-54 112.8139929

PAQR3 -0.734874267 1.68880392 -18.78135748 1.22E-54 4.71E-54 112.789071

TRIM10 0.948100599 0.575137675 18.781242 1.22E-54 4.71E-54 112.7879985

ZNF365 1.145125991 0.926435898 18.78065729 1.23E-54 4.74E-54 112.7825677

EPOR -1.37768361 3.549755642 -18.77959155 1.24E-54 4.78E-54 112.7726693

ANKRD34A 0.658683859 0.826166395 18.77719617 1.27E-54 4.89E-54 112.7504215

GFER -1.159651802 4.691271953 -18.77462432 1.30E-54 5.01E-54 112.7265345

LRMP -1.711396019 3.367899578 -18.77313366 1.32E-54 5.07E-54 112.7126895

TLNRD1 0.96061833 3.585145954 18.77208503 1.33E-54 5.12E-54 112.70295

DDB2 -1.346754951 4.231606648 -18.77091897 1.34E-54 5.18E-54 112.6921197

VIRMA -0.936155569 3.976016099 -18.77073303 1.35E-54 5.19E-54 112.6903927

ZNF506 -0.805795479 2.591378523 -18.76984307 1.36E-54 5.23E-54 112.6821269

CCNH -0.943617626 4.057949493 -18.76823848 1.38E-54 5.31E-54 112.6672236

CCN2 2.800794858 7.083641929 18.76635528 1.40E-54 5.40E-54 112.6497326

MED23 -0.916384942 3.59196713 -18.76588945 1.41E-54 5.42E-54 112.6454061

STUB1 -1.255388595 5.696919208 -18.76577071 1.41E-54 5.42E-54 112.6443031

STPG2 -0.259036453 0.217196601 -18.76571977 1.41E-54 5.43E-54 112.64383

HIST1H3H 1.849769773 1.248490145 18.76161661 1.47E-54 5.64E-54 112.6057202

DYSF 1.126567644 2.291221471 18.75952343 1.50E-54 5.75E-54 112.5862789

C1QTNF3-AMACR -0.748762332 0.40249473 -18.75948427 1.50E-54 5.75E-54 112.5859151

LIN9 0.731988313 1.418305161 18.75717392 1.53E-54 5.87E-54 112.5644566

ADORA2B 1.482374235 1.927455654 18.75708934 1.53E-54 5.87E-54 112.5636711

LRP5L -1.198273374 2.540110712 -18.75610945 1.54E-54 5.93E-54 112.5545699

GPBAR1 1.511431469 2.056757325 18.75426626 1.57E-54 6.03E-54 112.5374503

COX6C -1.352457981 6.601190419 -18.75183344 1.61E-54 6.16E-54 112.5148544

UNC5C 0.666493448 0.669006137 18.74964643 1.64E-54 6.29E-54 112.4945414

UBA52 -1.579829714 8.550656126 -18.74020545 1.79E-54 6.86E-54 112.4068531

TBXAS1 1.513014703 2.741185126 18.73669749 1.85E-54 7.09E-54 112.3742709

MAGT1 1.016987854 5.035533559 18.73321988 1.91E-54 7.32E-54 112.3419705

WDR1 1.060410383 6.067388274 18.73294054 1.91E-54 7.34E-54 112.3393759

SULT2B1 1.9120764 1.76132249 18.72924791 1.98E-54 7.59E-54 112.3050784

YPEL3 -1.456328231 5.946340271 -18.72811284 2.00E-54 7.67E-54 112.2945357

H6PD 1.139995888 4.427039374 18.72619782 2.04E-54 7.81E-54 112.2767487

RNF20 0.839975594 4.064448307 18.72085215 2.14E-54 8.21E-54 112.2270972

P4HTM -1.316118286 4.637499708 -18.71991739 2.16E-54 8.27E-54 112.218415

RASL11B 1.427605831 1.802812649 18.71990355 2.16E-54 8.27E-54 112.2182864

PPIP5K1 -0.994383277 2.998779365 -18.71906213 2.18E-54 8.34E-54 112.2104711

MAD2L1 1.129713203 1.675647545 18.71852717 2.19E-54 8.38E-54 112.2055024

RBMXL1 0.787689775 2.678844576 18.70888854 2.39E-54 9.16E-54 112.1159767

C12orf45 -1.144766459 4.253122095 -18.70392239 2.51E-54 9.59E-54 112.0698498

STK24 -1.138724718 4.602473308 -18.70294248 2.53E-54 9.68E-54 112.0607482

ESPL1 0.907283928 0.959056605 18.6926602 2.78E-54 1.06E-53 111.9652433

SLC40A1 1.791354523 5.782399901 18.68800617 2.91E-54 1.11E-53 111.9220152

CD27 1.835025638 1.743290924 18.67711499 3.22E-54 1.23E-53 111.8208539

PCDHGC3 -1.472984967 3.635053867 -18.67630172 3.24E-54 1.24E-53 111.8132999

IL22RA1 -2.713844801 5.597249274 -18.67582152 3.25E-54 1.24E-53 111.8088397

ZNF737 -1.160720512 2.250411159 -18.67183757 3.38E-54 1.29E-53 111.7718351

TNFAIP8L3 1.332861458 1.615765574 18.66621113 3.56E-54 1.36E-53 111.7195744

CFAP300 1.014852203 1.332737976 18.66615993 3.56E-54 1.36E-53 111.7190988

CXCR5 -1.097444263 0.736324351 -18.66387827 3.64E-54 1.39E-53 111.6979058

RGCC 1.651427478 4.889924744 18.66136987 3.72E-54 1.42E-53 111.6746067

RABL3 0.695939761 3.051975415 18.65569306 3.92E-54 1.50E-53 111.6218778

ALG1 -0.959826091 3.871639865 -18.65509359 3.95E-54 1.51E-53 111.6163097

COL16A1 1.721770802 4.450934264 18.65420337 3.98E-54 1.52E-53 111.6080409

CZIB -0.977914503 4.601184059 -18.65415655 3.98E-54 1.52E-53 111.6076061

C1QTNF9 -0.498122595 0.428968139 -18.64530713 4.32E-54 1.65E-53 111.5254084

GOPC 0.807545717 3.851737306 18.6409873 4.50E-54 1.72E-53 111.4852835

APOBEC1 2.507306524 1.340814407 18.63937956 4.57E-54 1.74E-53 111.47035

MRPS21 1.108766152 5.790319166 18.63728954 4.65E-54 1.77E-53 111.4509368

WFDC2 2.786271087 5.747948771 18.63284853 4.85E-54 1.85E-53 111.4096864

TOR3A 0.829334881 4.508639555 18.63268375 4.86E-54 1.85E-53 111.4081558

DNAJC8 0.917727527 5.335100032 18.63197411 4.89E-54 1.86E-53 111.4015643

PPP1R3G 0.918349646 0.686979201 18.63078268 4.94E-54 1.88E-53 111.3904976

DEPDC7 -1.072502718 1.796862571 -18.63034869 4.96E-54 1.89E-53 111.3864665

CD3E 1.976144951 2.488345107 18.63012148 4.98E-54 1.89E-53 111.384356

G3BP2 0.992299667 4.859390459 18.62855224 5.05E-54 1.92E-53 111.36978

ABLIM1 1.204274714 4.791570818 18.62528504 5.20E-54 1.98E-53 111.3394324

MAP2K3 1.086715489 5.00665102 18.61711959 5.61E-54 2.14E-53 111.263587

SEC24B 0.821446702 3.927495387 18.61551077 5.70E-54 2.17E-53 111.2486434

CDC25B 1.245442945 4.211159997 18.6140916 5.77E-54 2.20E-53 111.2354614

VSIR 1.244391731 4.40937121 18.61177636 5.90E-54 2.24E-53 111.2139561

ATP6AP1L -1.008130623 1.694785926 -18.61019927 5.99E-54 2.28E-53 111.199307

MIER1 0.741649326 3.239176677 18.60751549 6.14E-54 2.33E-53 111.1743785

TMEM37 1.690879607 4.225725471 18.60663785 6.19E-54 2.35E-53 111.1662264

ZNRD1 -1.055084925 4.003756741 -18.60258261 6.43E-54 2.44E-53 111.1285589

TET3 0.772745944 2.073928912 18.6018788 6.47E-54 2.46E-53 111.1220215

RAPGEF2 -0.904403133 3.356592247 -18.60134356 6.50E-54 2.47E-53 111.1170498

UBE2Z 0.869441788 5.257994113 18.5997903 6.59E-54 2.51E-53 111.1026222

ID1 2.187084798 5.95018522 18.59948351 6.61E-54 2.51E-53 111.0997726

GJB5 2.534544955 1.680195677 18.59888235 6.65E-54 2.53E-53 111.0941886

KDELR1 1.232889694 7.313464755 18.59825413 6.69E-54 2.54E-53 111.0883533

AC005726.1 -0.727580215 1.237523417 -18.59683369 6.78E-54 2.57E-53 111.0751593

NUP93 -0.856424982 3.443515664 -18.59314961 7.01E-54 2.66E-53 111.0409392

VANGL1 1.15203153 2.36691398 18.58922143 7.28E-54 2.76E-53 111.0044518

MYH9 1.690747095 7.846018578 18.58355119 7.67E-54 2.91E-53 110.9517829

COL15A1 1.850689602 4.577321734 18.58229854 7.76E-54 2.94E-53 110.9401475

ENC1 1.719607738 4.875096991 18.57853727 8.03E-54 3.05E-53 110.9052104

MAGED4 -1.351694882 0.664678692 -18.57630101 8.20E-54 3.11E-53 110.8844385

LLGL1 0.838190007 2.974413337 18.57577337 8.24E-54 3.13E-53 110.8795374

COL9A2 1.713223207 2.592655673 18.57548371 8.27E-54 3.13E-53 110.8768469

TNIK 1.190097338 2.780996853 18.57459529 8.33E-54 3.16E-53 110.8685946

TBC1D17 -1.145535985 5.062471657 -18.57435713 8.35E-54 3.16E-53 110.8663824

UTP25 0.792978079 2.344006558 18.57088756 8.63E-54 3.27E-53 110.8341547

TBK1 0.714783824 3.45568858 18.56220398 9.35E-54 3.54E-53 110.7534958

MSRB1 -1.510813455 5.773511444 -18.56171591 9.39E-54 3.56E-53 110.7489622

HOXB9 2.099114986 1.150911645 18.56112056 9.45E-54 3.58E-53 110.7434322

PDF -1.182738297 2.296346456 -18.5603548 9.51E-54 3.60E-53 110.7363193

DLEU7 0.523841303 0.414508797 18.55876338 9.66E-54 3.65E-53 110.7215371

MRPS25 -0.985712378 4.240939206 -18.55829229 9.70E-54 3.67E-53 110.7171613

SLC5A9 -1.522757812 1.940245972 -18.55234872 1.02E-53 3.88E-53 110.6619533

CAPN9 2.720953328 1.682551217 18.55005356 1.05E-53 3.96E-53 110.6406343

FARSA 0.907010859 5.307982767 18.54904005 1.06E-53 4.00E-53 110.6312201

RER1 -1.132391489 5.822724494 -18.54853567 1.06E-53 4.01E-53 110.6265351

DSTYK 0.67393917 2.447236128 18.54773494 1.07E-53 4.04E-53 110.6190974

CCDC144A -1.341809343 0.743199317 -18.5449506 1.10E-53 4.15E-53 110.5932344

RNF223 1.428717577 0.962974686 18.54142698 1.13E-53 4.29E-53 110.5605046

SH2D2A 1.348653269 1.896029765 18.53934394 1.16E-53 4.37E-53 110.5411559

RFX4 -0.225918139 0.14104856 -18.53726648 1.18E-53 4.45E-53 110.5218591

ZNF134 0.83756814 2.701964457 18.53694149 1.18E-53 4.47E-53 110.5188403

SNW1 0.867993438 5.364387167 18.53375249 1.22E-53 4.60E-53 110.4892186

WASF2 1.068280103 5.259074009 18.52938318 1.27E-53 4.79E-53 110.4486334

CSF1R 1.914297692 3.688943784 18.52731907 1.29E-53 4.88E-53 110.4294605

MRPL33 -1.183128016 5.879981785 -18.52060246 1.38E-53 5.19E-53 110.3670719

CDHR5 2.480653001 2.706683375 18.51908827 1.40E-53 5.27E-53 110.353007

TEAD1 1.032780202 3.537064063 18.5184653 1.40E-53 5.30E-53 110.3472205

NDUFS8 -1.333412585 5.573637027 -18.51614793 1.43E-53 5.41E-53 110.3256951

C1QTNF1 1.506848851 4.230335295 18.5159177 1.44E-53 5.42E-53 110.3235566

RBL2 -1.3265709 5.025672664 -18.51161427 1.50E-53 5.64E-53 110.2835833

CNOT3 -0.935738298 4.443547465 -18.50891863 1.53E-53 5.78E-53 110.2585443

RGPD1 -1.832091678 0.915286 -18.50862046 1.54E-53 5.80E-53 110.2557747

RIPK1 0.797906231 4.146707825 18.50815592 1.55E-53 5.82E-53 110.2514597

FADD 0.930574638 3.360993596 18.5077992 1.55E-53 5.84E-53 110.2481463

TP53TG3D -0.577698403 0.341059913 -18.50685079 1.56E-53 5.89E-53 110.2393367

ATG14 0.758449245 3.238052744 18.5033773 1.62E-53 6.08E-53 110.2070726

FSD2 -0.257262097 0.15907448 -18.50311755 1.62E-53 6.10E-53 110.2046599

ZDHHC23 -0.846591254 2.209014687 -18.50004003 1.67E-53 6.27E-53 110.1760737

PTPN12 1.053493842 4.759641648 18.4970027 1.71E-53 6.45E-53 110.1478608

SKA1 0.99072884 0.970491359 18.48683388 1.88E-53 7.09E-53 110.0534058

LGALSL 0.957981517 2.939305519 18.48646747 1.89E-53 7.11E-53 110.0500023

KDM3A -0.960327545 3.885653512 -18.48315736 1.95E-53 7.33E-53 110.0192559

CDK20 -0.895957568 2.670051469 -18.47856666 2.03E-53 7.65E-53 109.9766142

SCGB1C1 -0.941253026 0.52048138 -18.47727488 2.06E-53 7.74E-53 109.9646154

ERCC6L 0.740989043 0.632666453 18.47479524 2.11E-53 7.92E-53 109.9415828

UBR5 -1.012501136 4.516823104 -18.47352527 2.13E-53 8.01E-53 109.9297865

JAK1 1.124711427 5.619438777 18.46961512 2.21E-53 8.31E-53 109.8934664

EEA1 0.851318566 2.357519906 18.4640768 2.33E-53 8.74E-53 109.8420229

DBH -0.909533726 0.912583269 -18.46354947 2.34E-53 8.79E-53 109.8371247

DHRS9 3.140973403 2.056963757 18.4633536 2.34E-53 8.80E-53 109.8353052

ASXL2 0.820150775 2.469940223 18.46244228 2.36E-53 8.87E-53 109.8268404

BIRC7 1.981209499 1.11986675 18.46176702 2.38E-53 8.93E-53 109.820568

BAHCC1 -0.897451071 2.608215707 -18.46169058 2.38E-53 8.93E-53 109.819858

GBP1 1.620398682 3.183492673 18.46067604 2.40E-53 9.01E-53 109.8104344

ZNF639 -0.762473397 3.473745409 -18.45729116 2.48E-53 9.30E-53 109.7789934

GPR4 1.084565982 2.528652813 18.45566257 2.52E-53 9.44E-53 109.763866

VPS8 -0.773698746 3.360542067 -18.45111164 2.63E-53 9.85E-53 109.721594

SLC2A6 1.293346716 2.346949503 18.4506751 2.64E-53 9.88E-53 109.7175392

RHOB 1.980576048 7.112763265 18.43672566 3.00E-53 1.13E-52 109.5879682

ZNF528 -1.031083612 2.466617925 -18.43576201 3.03E-53 1.13E-52 109.5790173

BRD2 -1.234438146 6.592365543 -18.43552109 3.03E-53 1.14E-52 109.5767795

GREB1 -1.458961568 1.792228445 -18.43514002 3.05E-53 1.14E-52 109.5732399

UCP3 -0.517843488 0.87862888 -18.43454913 3.06E-53 1.15E-52 109.5677513

SF3B6 0.996941993 6.043630875 18.43412489 3.07E-53 1.15E-52 109.5638107

LZTR1 -1.021241356 4.580099289 -18.43377885 3.08E-53 1.15E-52 109.5605965

LRRC74A -0.622437937 0.440778913 -18.43359625 3.09E-53 1.16E-52 109.5589004

BPTF -0.950016682 3.827684832 -18.43105706 3.16E-53 1.18E-52 109.5353149

AL121753.1 0.646573445 0.545092084 18.42734262 3.27E-53 1.23E-52 109.5008131

ORC4 -0.787782805 3.099679838 -18.42666092 3.30E-53 1.23E-52 109.494481

NHLRC4 0.598978735 0.668223548 18.42200907 3.44E-53 1.29E-52 109.451272

PPP4C 1.005813463 5.812029328 18.42052202 3.49E-53 1.30E-52 109.4374595

IMMP2L -0.827592309 3.026968307 -18.41873103 3.55E-53 1.33E-52 109.4208238

MFSD5 0.890183765 4.57038982 18.41800034 3.57E-53 1.34E-52 109.4140367

ANKRD20A4 -0.667306523 0.372102747 -18.4144035 3.69E-53 1.38E-52 109.3806273

DROSHA -0.945660087 4.287029741 -18.41271001 3.75E-53 1.40E-52 109.3648972

SIRPA 1.347861032 4.121226729 18.41254313 3.76E-53 1.40E-52 109.3633472

LRIG1 -1.562400725 5.310382588 -18.41114123 3.81E-53 1.42E-52 109.3503256

ETFDH -1.120288677 3.764345714 -18.40884412 3.89E-53 1.45E-52 109.3289888

RAB27B 1.549257864 2.497811551 18.40817934 3.91E-53 1.46E-52 109.322814

OXTR 1.300088254 0.974398052 18.40637408 3.98E-53 1.49E-52 109.3060457

KIFAP3 0.808543561 3.947399238 18.40612865 3.99E-53 1.49E-52 109.3037661

EMILIN2 1.258238233 2.514451174 18.40498094 4.03E-53 1.50E-52 109.2931056

CSK 1.022545399 4.652952983 18.40404532 4.07E-53 1.52E-52 109.2844151

MAMDC4 -1.667034186 3.015897784 -18.39624788 4.37E-53 1.63E-52 109.2119886

LOXL3 1.001993313 1.989884732 18.39377705 4.47E-53 1.67E-52 109.1890383

DLX5 1.08813255 0.662922979 18.39239705 4.53E-53 1.69E-52 109.1762202

ZNF511 -1.079964322 3.915467517 -18.3894204 4.66E-53 1.74E-52 109.1485718

MUC5AC 3.746886642 2.261827957 18.38525809 4.84E-53 1.81E-52 109.1099103

TMEM184A -1.623560724 4.008411672 -18.38472053 4.87E-53 1.81E-52 109.1049172

CPSF3 0.790921588 3.907730848 18.3836542 4.91E-53 1.83E-52 109.0950127

MT-ND6 2.461385105 11.60279354 18.3815376 5.01E-53 1.87E-52 109.0753528

LPP 1.11484814 3.182120709 18.37937821 5.11E-53 1.90E-52 109.0552955

DHCR7 1.243284674 4.074387926 18.37521693 5.31E-53 1.98E-52 109.0166438

OLFML2A 1.287655376 2.706466557 18.37099438 5.53E-53 2.06E-52 108.977423

TAPT1 -0.818293086 3.424065379 -18.36862168 5.65E-53 2.10E-52 108.9553845

CLEC18C -0.458028489 0.256267711 -18.36430694 5.88E-53 2.19E-52 108.9153077

MROH1 -1.207316345 4.257183283 -18.36137948 6.04E-53 2.25E-52 108.8881164

ZNF418 -0.905886986 1.695342188 -18.35793381 6.24E-53 2.32E-52 108.8561118

ZNF462 0.777288102 1.678713169 18.35747507 6.27E-53 2.33E-52 108.8518509

GPR61 -0.451811747 0.358734923 -18.35664071 6.32E-53 2.35E-52 108.8441011

KCNMB4 1.160004592 1.324875631 18.35407828 6.47E-53 2.41E-52 108.8203004

PIK3CG 1.099172629 0.952354068 18.35333716 6.51E-53 2.42E-52 108.8134167

EMP2 1.223580181 4.056680934 18.35136353 6.63E-53 2.47E-52 108.7950851

HDGF 1.228266059 6.904881495 18.35020911 6.71E-53 2.49E-52 108.7843624

ENG 1.172370397 5.953678097 18.34864962 6.80E-53 2.53E-52 108.7698775

TSKU 1.513249279 4.419818865 18.34270462 7.19E-53 2.67E-52 108.7146587

HLA-DPA1 2.125450723 5.415529558 18.33621874 7.64E-53 2.84E-52 108.6544163

SLC39A6 1.186450344 4.48114748 18.33393024 7.80E-53 2.90E-52 108.6331602

TFF3 4.150909672 5.281934444 18.33137902 7.99E-53 2.97E-52 108.6094639

ADAMTSL2 1.687176689 3.506140468 18.33129012 7.99E-53 2.97E-52 108.6086382

SULT1B1 2.371469261 1.524198855 18.3312815 7.99E-53 2.97E-52 108.6085581

EXOG -0.707768268 2.252789432 -18.32961312 8.12E-53 3.01E-52 108.5930619

POM121C -0.790968032 3.586169603 -18.3286009 8.20E-53 3.04E-52 108.5836602

WIPF1 1.382481514 3.449296629 18.3257802 8.41E-53 3.12E-52 108.5574611

LRRC66 1.756638609 1.359408776 18.32425558 8.53E-53 3.17E-52 108.5433001

SMCR8 0.848485171 2.79016221 18.32013881 8.87E-53 3.29E-52 108.505063

HASPIN 0.674223366 0.669702524 18.31818589 9.03E-53 3.35E-52 108.486924

MRPL43 -1.072260867 5.430484842 -18.31801726 9.04E-53 3.35E-52 108.4853577

GAB2 0.995074002 3.159905231 18.31667653 9.16E-53 3.39E-52 108.4729049

PTOV1 -1.375903109 6.133260635 -18.31429557 9.36E-53 3.47E-52 108.4507903

PLXNA2 -1.747821376 4.583398198 -18.31425261 9.36E-53 3.47E-52 108.4503913

PGBD5 -1.528633512 2.272229278 -18.3098373 9.76E-53 3.61E-52 108.4093815

HSP90AB1 1.569003694 9.340375628 18.30942428 9.79E-53 3.63E-52 108.4055453

C11orf53 1.786078051 1.382956048 18.30752897 9.97E-53 3.69E-52 108.3879415

NKIRAS2 0.733903534 3.975719552 18.3070602 1.00E-52 3.71E-52 108.3835875

VPS26C -0.811058421 3.873294132 -18.30701035 1.00E-52 3.71E-52 108.3831246

NDUFV1 -1.246759778 6.534327879 -18.30595444 1.01E-52 3.74E-52 108.3733172

MICOS10-NBL1 -0.890063947 0.46067975 -18.30576009 1.01E-52 3.75E-52 108.371512

ZMYM4 0.732260986 3.608819742 18.30526846 1.02E-52 3.77E-52 108.3669458

PCDH18 1.297521637 1.977308462 18.30232234 1.05E-52 3.87E-52 108.3395821

RC3H2 0.699341712 3.031076081 18.30048532 1.06E-52 3.94E-52 108.3225198

CCDC163 -1.513800145 2.098067876 -18.29950611 1.07E-52 3.97E-52 108.3134249

C1orf74 0.640372776 1.202769127 18.29781573 1.09E-52 4.03E-52 108.2977247

MYL2 -1.647689928 0.992366326 -18.29566926 1.11E-52 4.11E-52 108.2777882

RPS3A -1.787075907 9.367582032 -18.29526709 1.12E-52 4.13E-52 108.2740529

C5orf46 1.879328362 1.51804095 18.2944575 1.13E-52 4.16E-52 108.2665334

SNRPA1 -1.081793062 4.547077789 -18.29276488 1.14E-52 4.22E-52 108.2508124

EP300 0.931932479 3.898892776 18.29113868 1.16E-52 4.29E-52 108.2357084

HACD3 0.972674123 4.48868831 18.28749571 1.20E-52 4.43E-52 108.2018726

TSC22D1 1.232922753 5.556196556 18.28586934 1.22E-52 4.50E-52 108.186767

SEC23A 1.051367882 3.492649315 18.28413638 1.24E-52 4.57E-52 108.1706714

HOXC8 1.205696637 0.760616462 18.28343552 1.25E-52 4.60E-52 108.1641619

CLMP 1.822308519 2.359707975 18.28255767 1.26E-52 4.64E-52 108.1560084

NPHP4 -0.797212114 2.242295441 -18.28026228 1.28E-52 4.74E-52 108.1346891

ATXN7 -1.001530695 3.314229878 -18.27996407 1.29E-52 4.75E-52 108.1319194

MAPK8IP1 -1.76794245 4.106001009 -18.27951215 1.29E-52 4.77E-52 108.127722

GTF3C1 -0.99771849 4.824227544 -18.2770325 1.32E-52 4.88E-52 108.1046913

ZC3H13 0.764153046 3.496278052 18.27561328 1.34E-52 4.94E-52 108.0915098

ATOH8 -1.373670436 2.164354452 -18.27308729 1.37E-52 5.06E-52 108.0680487

SLC22A31 -3.183527881 2.127523224 -18.27058844 1.41E-52 5.18E-52 108.0448399

PHRF1 0.820022271 4.051336321 18.26855063 1.43E-52 5.28E-52 108.0259131

MTCH2 0.943693646 5.080742318 18.2665755 1.46E-52 5.37E-52 108.0075684

FAM149B1 -0.737810177 3.302248071 -18.26513737 1.48E-52 5.44E-52 107.9942114

VPS53 -0.782241403 2.590680531 -18.26386227 1.50E-52 5.51E-52 107.9823685

PABPN1L -0.363265689 0.224921682 -18.26206759 1.52E-52 5.60E-52 107.9656999

CHRNE -0.954334973 1.311539594 -18.2605272 1.54E-52 5.68E-52 107.9513932

KEAP1 0.879211566 5.01415712 18.2590171 1.56E-52 5.76E-52 107.9373677

DECR2 -1.400434277 4.253564966 -18.25546431 1.62E-52 5.95E-52 107.9043704

GRM6 -0.225588486 0.228569308 -18.25202331 1.67E-52 6.14E-52 107.8724114

ETFBKMT -0.608224748 1.449195734 -18.25127489 1.68E-52 6.18E-52 107.8654604

DDX59 -0.789186647 3.292293205 -18.25113983 1.68E-52 6.19E-52 107.8642059

KNOP1 -0.877116958 2.90096227 -18.25073389 1.69E-52 6.21E-52 107.8604357

PRICKLE4 -0.870108298 1.177631766 -18.24950177 1.71E-52 6.28E-52 107.8489922

SEC14L2 1.293325149 2.807358157 18.2486243 1.72E-52 6.33E-52 107.8408425

DPP4 1.682618174 2.360310688 18.24526167 1.78E-52 6.53E-52 107.8096116

SYVN1 -1.18312513 5.729142319 -18.23468869 1.96E-52 7.21E-52 107.711414

SLC1A4 -1.276298654 3.8848907 -18.23331927 1.99E-52 7.30E-52 107.6986955

FOPNL 0.736948253 4.310337479 18.23301317 1.99E-52 7.32E-52 107.6958525

PSAT1 -2.31496658 4.286126932 -18.23195011 2.01E-52 7.39E-52 107.6859793

COX8A 1.447863814 8.322388446 18.23094998 2.03E-52 7.46E-52 107.6766906

FRMD8 0.992337112 4.657356651 18.23091843 2.03E-52 7.46E-52 107.6763976

MAGOHB -0.866520531 2.964416506 -18.23031013 2.04E-52 7.50E-52 107.670748

IFITM5 -1.949671647 1.140576724 -18.22810064 2.09E-52 7.65E-52 107.6502273

NID1 1.682397843 4.613822487 18.22396791 2.17E-52 7.95E-52 107.6118446

SMOX 1.32064933 3.954114549 18.22340599 2.18E-52 7.99E-52 107.6066259

ASF1A 0.762428885 3.201167096 18.22263957 2.19E-52 8.04E-52 107.5995077

DOK1 0.897437442 3.510440823 18.21457279 2.36E-52 8.67E-52 107.5245882

GAP43 1.589607125 1.474219022 18.2137969 2.38E-52 8.73E-52 107.5173821

STN1 1.100342633 4.704022974 18.20885078 2.49E-52 9.14E-52 107.4714457

AC105052.1 -1.145612039 0.561485055 -18.20637256 2.55E-52 9.35E-52 107.4484297

ERBB4 -0.424460842 0.409726091 -18.20487361 2.59E-52 9.48E-52 107.4345085

TNFRSF4 1.312039285 1.857400518 18.20355964 2.62E-52 9.59E-52 107.4223053

PPIL4 0.805627846 3.936662487 18.20003531 2.71E-52 9.91E-52 107.3895739

IFITM10 1.599783729 1.758859805 18.1987418 2.74E-52 1.00E-51 107.3775607

MDC1 -0.866523922 3.699759716 -18.19825644 2.75E-52 1.01E-51 107.373053

BIRC6 -0.984366962 3.971628371 -18.19512449 2.83E-52 1.04E-51 107.3439659

ATP6V1B1 -1.39316046 1.89031515 -18.1932837 2.88E-52 1.05E-51 107.3268701
[truncated: 862,681 more chars]
