# Supplementary material for: Identification and Validation of Apparent Imbalanced Epi-lncRNAs Prognostic Model Based on Multi-Omics Data in Pancreatic Cancer
Source: Front Mol Biosci. 2022 May 12;9:860323. doi: 10.3389/fmolb.2022.860323 (PMC9133386; doi:10.3389/fmolb.2022.860323)
Supplement: Supplementary file 3 [file Table2.DOCX]

Supplementary Table 2. 9167 lncRNAs

logFC AveExpr t P.Value adj.P.Val B

AC008894.2 -3.937928258 1.98582074 -107.1553797 1.04E-265 1.47E-261 598.5971614

AL160408.2 -4.873173278 2.365755625 -100.3497411 3.07E-256 2.17E-252 576.9073467

AC020978.5 -4.02921049 1.956444211 -95.64270623 2.49E-249 1.08E-245 561.072568

AC012321.1 -4.161801105 2.02040925 -95.58306549 3.06E-249 1.08E-245 560.8672522

AC008581.1 -4.199251444 2.038590083 -94.98446754 2.44E-248 6.87E-245 558.7998512

FP671120.6 -9.340768408 4.561860753 -93.70038222 2.18E-246 5.12E-243 554.3233405

FP236383.4 -9.339143034 4.562697065 -93.25020763 1.07E-245 2.15E-242 552.7403299

AP001267.1 -3.734141029 1.862929632 -92.58376189 1.14E-244 2.01E-241 550.3836221

FP236383.5 -9.330630959 4.567076825 -92.5237292 1.41E-244 2.21E-241 550.1705536

AC005523.2 -3.549592601 1.723203377 -90.49547781 2.09E-241 2.95E-238 542.8948839

AC011472.3 -5.333758385 2.589353627 -89.42282936 1.06E-239 1.35E-236 538.9854329

LIMD1-AS1 -3.059687411 1.677902898 -89.03227189 4.46E-239 5.23E-236 537.5511418

AC099518.5 -3.560348224 1.728424857 -88.29775837 6.78E-238 7.35E-235 534.8378031

AL138478.1 -3.49255367 1.695512966 -87.37793232 2.11E-236 2.13E-233 531.4102439

AC016026.1 -3.158228694 1.615467714 -87.3271079 2.56E-236 2.40E-233 531.2198838

AC008738.7 -4.110195398 1.995356479 -86.20826135 1.76E-234 1.55E-231 527.0031493

AL583722.2 -4.46672978 2.168441485 -85.2561004 6.68E-233 5.54E-230 523.3746549

GUSBP11 -4.236240319 2.500140385 -84.05187557 7.03E-231 5.50E-228 518.7318011

TTN-AS1 -3.31818666 2.074707578 -83.32741684 1.19E-229 8.83E-227 515.9091608

AC093484.3 -5.544472792 2.691648121 -81.85596187 4.00E-227 2.82E-224 510.1062333

AL354740.1 -3.405675863 1.740430274 -81.51066814 1.59E-226 1.07E-223 508.7307146

AC146944.4 -3.277855678 1.640367213 -81.39739905 2.50E-226 1.60E-223 508.2783363

AC092683.1 -4.309936982 2.26311921 -79.68502899 2.54E-223 1.56E-220 501.3686896

AC008264.2 -2.431069688 1.28589773 -78.42580487 4.52E-221 2.65E-218 496.201059

LINC01001 -4.042568891 2.364522198 -78.30737763 7.38E-221 4.16E-218 495.7112014

SLX1A-SULT1A3 -3.690495001 1.846521929 -75.87707228 2.03E-216 1.10E-213 485.5082811

HCG25 -3.76596125 2.426086158 -73.44277534 7.63E-212 3.98E-209 474.990756

AC010733.2 -1.585025389 0.769474527 -73.08894256 3.62E-211 1.82E-208 473.4362345

AL157392.3 -3.940274475 3.155852067 -72.84458864 1.06E-210 5.17E-208 472.3587922

AC048382.6 -2.464060458 1.196215389 -72.71624733 1.88E-210 8.83E-208 471.7916063

AC007009.1 -3.921804488 1.906216572 -72.43663215 6.50E-210 2.95E-207 470.5528102

AC087292.1 -3.141047842 1.524869148 -71.2930369 1.08E-207 4.78E-205 465.4418601

AL513314.2 -3.006155697 1.616871066 -71.19987691 1.65E-207 7.05E-205 465.0223305

CERS6-AS1 -2.725024702 1.376958747 -70.29418299 1.01E-205 4.17E-203 460.9183314

AC125611.3 -4.257021455 2.074805174 -70.16285709 1.83E-205 7.37E-203 460.3193992

FAM226B -2.131658103 1.035859984 -69.82583514 8.57E-205 3.35E-202 458.7778483

AC138035.1 -3.1844862 1.908792503 -69.77461105 1.08E-204 4.13E-202 458.5429769

AC125494.1 -3.45549527 1.86524642 -68.84386258 7.98E-203 2.96E-200 454.2488561

AF127577.1 -3.226436514 1.56632237 -68.60858675 2.38E-202 8.62E-200 453.1553614

AC023509.1 -2.629494061 1.556185249 -68.38782165 6.68E-202 2.35E-199 452.1263336

AC018557.1 -3.020698254 1.466443622 -67.37121215 7.96E-200 2.74E-197 447.3501368

AL136131.3 -5.256414425 2.551805832 -66.38466047 8.75E-198 2.93E-195 442.6550902

SNHG14 -3.700271106 3.039004981 -66.14462927 2.77E-197 9.07E-195 441.5036663

AL139317.3 -3.25335218 1.579388987 -66.12899606 2.98E-197 9.56E-195 441.4285494

AL008729.2 -2.78668579 1.466502609 -65.93698282 7.52E-197 2.36E-194 440.5046849

AC139256.2 -2.987316602 2.23194643 -64.97941699 7.83E-195 2.40E-192 435.8625951

STAG3L5P-PVRIG2P-PILRB -4.709496672 3.644303262 -64.94217183 9.40E-195 2.82E-192 435.6808558

AC091057.3 -3.485339571 1.936195968 -64.50624414 7.95E-194 2.33E-191 433.5470747

SLX1B-SULT1A4 -2.735190413 1.334037714 -64.31748562 2.01E-193 5.78E-191 432.6193149

AP005329.3 -2.558971925 1.242291596 -63.98360167 1.04E-192 2.94E-190 430.9725535

ZFHX2-AS1 -2.375177845 1.654642716 -63.70024225 4.25E-192 1.18E-189 429.5692358

PCBP1-AS1 -3.264406728 2.784572126 -63.66908614 4.97E-192 1.35E-189 429.4146135

AC068831.4 -3.966477334 1.925586372 -63.52413887 1.02E-191 2.71E-189 428.6944193

AC026362.1 -2.150743303 1.280107136 -63.10329536 8.34E-191 2.18E-188 426.5954557

AC034102.4 -2.547788836 1.236862596 -62.6780342 7.05E-190 1.81E-187 424.4623764

AC083798.2 -2.877160497 2.202754588 -62.4826823 1.89E-189 4.75E-187 423.4784006

EDRF1-DT -2.430790593 1.530812775 -61.87007217 4.20E-188 1.04E-185 420.3757986

NUTM2B-AS1 -2.731069808 2.579716789 -61.07453787 2.46E-186 5.98E-184 416.3079784

AF111169.1 -2.92193001 1.418495085 -59.37973177 1.66E-182 3.97E-180 407.4921048

CU633967.1 -3.548641172 1.746121202 -59.25724737 3.17E-182 7.44E-180 406.846912

SEMA6A-AS1 -2.176023768 1.562006459 -58.96979638 1.45E-181 3.34E-179 405.3284072

NDUFA6-DT -2.304654863 1.80625017 -58.89479276 2.15E-181 4.89E-179 404.9311832

AC087190.3 -1.823034545 0.88501967 -58.73932562 4.91E-181 1.10E-178 404.1064883

AC008738.3 -2.678331308 1.300236412 -58.54007546 1.42E-180 3.12E-178 403.0469064

AC123768.4 -2.71300552 1.443810969 -58.12857486 1.28E-179 2.77E-177 400.8491836

AC240565.2 -2.560474898 1.402892617 -57.80090093 7.42E-179 1.58E-176 399.0900058

AC091564.4 -2.294351524 1.113827622 -57.46815208 4.46E-178 9.39E-176 397.2952066

GARS-DT -3.403364596 3.170096656 -56.70244935 2.87E-176 5.94E-174 393.1326889

AC006027.1 -2.281522383 1.107599522 -56.69967817 2.91E-176 5.95E-174 393.1175413

AC136624.2 -2.223128138 1.080501813 -56.07657653 8.91E-175 1.79E-172 389.6962661

VASH1-AS1 -3.264978498 2.668427454 -55.71670549 6.52E-174 1.29E-171 387.7062896

STRA6LP -1.130406381 0.613221075 -55.69772923 7.24E-174 1.42E-171 387.6010693

SOX9-AS1 -3.251030047 2.166816257 -55.45973082 2.72E-173 5.24E-171 386.27895

MUC20-OT1 -4.199954052 3.8569467 -55.43607132 3.10E-173 5.90E-171 386.1472685

CRYZL2P-SEC16B -2.451157381 1.564318783 -55.34781821 5.07E-173 9.52E-171 385.6556798

AC093525.7 -1.215321612 0.638327954 -55.12448896 1.76E-172 3.27E-170 384.4088708

TTC28-AS1 -2.452830107 2.697081976 -54.67573382 2.19E-171 4.00E-169 381.8912616

GMDS-DT -2.356130898 1.915188633 -54.30781388 1.74E-170 3.15E-168 379.8148113

AL627309.5 -3.118841193 1.60184664 -53.97369194 1.16E-169 2.07E-167 377.9193826

AC025259.1 -2.92430544 1.463979892 -52.82666571 8.33E-167 1.47E-164 371.3409528

AC015688.6 -4.60159866 2.233915621 -52.33451563 1.45E-165 2.52E-163 368.4838724

LINC01106 -3.361352954 2.635226996 -52.14956718 4.26E-165 7.32E-163 367.4047561

AC093495.1 -2.355764763 1.945638806 -52.03356894 8.40E-165 1.43E-162 366.7264179

ENTPD1-AS1 -1.441004787 0.93038017 -51.65546347 7.72E-164 1.30E-161 364.5071167

AL021368.2 -1.384071039 1.18689843 -51.50153789 1.91E-163 3.17E-161 363.6000308

AC009630.3 -2.5506672 1.238259941 -51.48073533 2.16E-163 3.54E-161 363.4772798

AL441883.1 -1.653602033 0.802766096 -51.10241493 2.03E-162 3.28E-160 361.2381756

AC093525.6 -2.243927348 1.375924677 -51.0326364 3.07E-162 4.91E-160 360.8237907

FAM157C -2.750848073 1.547844386 -50.59848933 4.08E-161 6.45E-159 358.2357266

AL513548.1 -2.038099589 1.009705878 -50.2628747 3.05E-160 4.77E-158 356.2233299

AC012358.3 -2.088834676 1.408588817 -49.94351909 2.09E-159 3.23E-157 354.2988608

INTS6-AS1 -2.110310708 1.527343057 -49.72643058 7.76E-159 1.19E-156 352.9853014

LINC01123 -3.118542952 2.334768057 -49.42980114 4.70E-158 7.12E-156 351.1833927

AC239868.1 -1.421171773 0.692652505 -49.32727103 8.77E-158 1.31E-155 350.5586559

AC004466.1 -1.577407806 1.079065645 -49.30196118 1.02E-157 1.52E-155 350.4042866

FAM215B -1.465164705 0.779354392 -49.10069202 3.50E-157 5.14E-155 349.1745732

AL355816.2 -2.806650766 1.362531033 -48.60269995 7.44E-156 1.08E-153 346.1155325

AC245297.2 -1.769117432 1.097060088 -48.04492323 2.35E-154 3.38E-152 342.6612199

AL627309.1 -1.260594278 0.627354727 -47.64542689 2.84E-153 4.04E-151 340.1687256

AP001318.2 2.031513154 1.409582799 47.56521017 4.69E-153 6.61E-151 339.6663783

LINC01409 -1.622180534 1.09208169 -47.33989335 1.93E-152 2.69E-150 338.2519996

AC012615.3 -2.529611808 1.228653116 -47.3293423 2.06E-152 2.85E-150 338.1856458

FP236383.3 -1.000866825 0.492694236 -47.15578951 6.14E-152 8.40E-150 337.0926336

AC159540.2 -3.405087432 1.669113276 -47.02385305 1.41E-151 1.91E-149 336.2597324

MCM3AP-AS1 -1.562256176 1.561626155 -46.91167712 2.87E-151 3.85E-149 335.5502256

AL590399.1 -1.767409518 0.874945786 -46.91019866 2.90E-151 3.85E-149 335.5408661

MAP4K3-DT -2.028296291 2.591623134 -46.64109775 1.59E-150 2.10E-148 333.8336925

AL928970.1 -0.803062679 0.405849946 -46.56658249 2.56E-150 3.34E-148 333.359695

TPT1-AS1 -2.514250097 2.858083489 -46.45912645 5.08E-150 6.56E-148 332.6751825

FAM223A -0.813768788 0.396573424 -46.34307752 1.06E-149 1.36E-147 331.9346344

AP000873.2 -1.791112914 1.738633904 -46.24854033 1.95E-149 2.47E-147 331.3303618

FAM223B -0.813192008 0.396870198 -46.2141564 2.43E-149 3.05E-147 331.1103603

AC022400.4 -1.996099867 1.878804515 -46.11879094 4.47E-149 5.57E-147 330.499553

AC026150.1 -1.917523951 1.069636825 -46.01900171 8.47E-149 1.05E-146 329.8594301

AC093673.1 2.913427353 3.28072582 45.78630249 3.78E-148 4.63E-146 328.3628094

AL358781.1 -3.123867699 1.516528788 -45.77179494 4.15E-148 5.04E-146 328.2693212

AL021707.2 -1.726602195 1.710425234 -45.72701325 5.54E-148 6.67E-146 327.9806084

TRHDE-AS1 -2.977790562 1.918263797 -45.6589561 8.59E-148 1.03E-145 327.5414452

LINC01624 -3.869794644 2.277687848 -44.94565945 8.79E-146 1.04E-143 322.9101092

AC055720.2 2.298479987 1.223504662 44.28898271 6.52E-144 7.66E-142 318.5998404

AC024267.6 -2.198026971 1.094498178 -43.75355149 2.26E-142 2.63E-140 315.0518942

CU633906.2 -1.929228278 0.950768312 -43.51968804 1.07E-141 1.24E-139 313.4926941

AC008443.1 -2.033231611 2.112223634 -43.05901573 2.35E-140 2.69E-138 310.4042052

LINC01829 -4.48697076 3.116627536 -43.01944233 3.07E-140 3.49E-138 310.1378279

MMP25-AS1 -2.399818938 2.688553089 -43.01162524 3.23E-140 3.64E-138 310.0851893

LINC00240 -1.565104147 1.04640504 -42.99790911 3.55E-140 3.97E-138 309.9928119

BX664727.3 -2.035916067 1.143046248 -42.85615517 9.21E-140 1.02E-137 309.0369155

AL590999.1 -1.752979678 1.1706595 -42.81272076 1.24E-139 1.36E-137 308.7435864

RBM5-AS1 -1.589151336 1.142955034 -42.7328922 2.12E-139 2.31E-137 308.20394

LINC02193 -3.439652254 1.828722799 -42.69590925 2.72E-139 2.95E-137 307.953698

AC005562.1 -1.371758793 1.279589514 -42.67392818 3.16E-139 3.40E-137 307.8048945

LINC00893 -2.838032203 2.569448872 -42.61544464 4.69E-139 5.01E-137 307.4087272

AC109587.1 -1.443242713 1.365947349 -42.59066094 5.55E-139 5.88E-137 307.2407301

AC005062.1 -2.638259355 1.810662747 -42.58312998 5.84E-139 6.14E-137 307.1896679

MEG3 -4.818911924 5.668513903 -42.20006822 7.88E-138 8.23E-136 304.5842199

SUGT1P4-STRA6LP -1.228487963 1.018308045 -42.1623756 1.02E-137 1.06E-135 304.326979

THUMPD3-AS1 -2.646048085 3.602751953 -42.03893969 2.37E-137 2.44E-135 303.4834712

AL161457.2 -0.872410974 0.58999967 -42.0262845 2.58E-137 2.64E-135 303.3968962

AC074212.1 -2.897359865 2.403618557 -41.93488926 4.83E-137 4.89E-135 302.7711307

AC115618.2 2.309362748 3.175750935 41.9237617 5.21E-137 5.24E-135 302.6948794

CBR3-AS1 -1.910811566 1.550938837 -41.37942804 2.20E-135 2.20E-133 298.9480819

AC011451.1 1.990683163 1.451066404 41.30406523 3.71E-135 3.68E-133 298.4267378

ZSWIM8-AS1 -1.484742045 0.833950512 -41.14402164 1.12E-134 1.11E-132 297.3174804

AC156455.1 -3.268272192 2.875586872 -41.02092367 2.64E-134 2.58E-132 296.4623359

AC005329.1 -1.701634684 0.934702776 -41.0174309 2.70E-134 2.63E-132 296.4380473

AC026401.3 2.607967828 2.449570559 40.74770775 1.77E-133 1.71E-131 294.5582437

SATB1-AS1 -1.541331026 1.04601023 -40.62969647 4.03E-133 3.86E-131 293.7331918

AC084018.2 -1.622945043 1.324511431 -40.59123979 5.28E-133 5.02E-131 293.4639887

FAM106A -3.104070206 1.750349153 -40.45889593 1.33E-132 1.26E-130 292.5362764

AL355574.1 1.959447897 1.158626957 40.44177865 1.50E-132 1.41E-130 292.4161412

AZIN1-AS1 -2.539397269 2.467431079 -40.42782029 1.66E-132 1.55E-130 292.3181517

NUTM2A-AS1 -2.103892475 2.942562566 -40.42535345 1.69E-132 1.56E-130 292.3008319

AP000759.1 2.160127498 1.781137476 40.40166975 1.99E-132 1.83E-130 292.1345118

AC084125.2 -1.94997738 1.730202319 -40.29509147 4.21E-132 3.85E-130 291.3852691

AC091563.1 2.434794928 2.349397773 40.21375133 7.46E-132 6.78E-130 290.8125778

AC048382.2 -2.176946322 1.959726159 -40.10255983 1.63E-131 1.48E-129 290.0284881

LAMTOR5-AS1 -1.494360369 0.942985447 -40.02583513 2.81E-131 2.52E-129 289.4866225

SNHG16 -2.41757566 4.103548436 -39.73795874 2.15E-130 1.92E-128 287.4474767

FAM153CP -2.924085325 1.600531255 -39.7175409 2.49E-130 2.21E-128 287.3024864

AC105339.2 -1.238546237 0.863848988 -39.50713147 1.11E-129 9.78E-128 285.8055309

AC011298.1 -4.268572219 2.73776674 -39.45524052 1.61E-129 1.41E-127 285.4355658

AC009022.1 -2.899645542 3.426982058 -39.38014905 2.75E-129 2.39E-127 284.8996361

LINC01237 -3.026613526 2.063448884 -39.36557293 3.05E-129 2.63E-127 284.7955301

AC126755.2 -1.917049684 0.937199295 -39.0708446 2.51E-128 2.16E-126 282.6852114

AP006621.1 -3.734126654 2.501849796 -38.95436495 5.79E-128 4.94E-126 281.8483994

AC005288.1 1.685526528 3.635033691 38.86291557 1.12E-127 9.48E-126 281.1902978

AC017116.1 -1.942211866 0.942876102 -38.74965979 2.53E-127 2.13E-125 280.3739115

ZNF433-AS1 -1.464397842 1.54774207 -38.72657699 2.98E-127 2.50E-125 280.2073382

AC022336.2 -0.742451556 0.360434323 -38.71206814 3.31E-127 2.76E-125 280.1026054

AC022167.2 -2.115261996 2.317226134 -38.56295325 9.72E-127 8.06E-125 279.0247789

AL355310.2 -1.278376579 0.620607227 -38.52634444 1.27E-126 1.04E-124 278.7597644

CU634019.5 -2.019320166 1.033793185 -38.51335834 1.39E-126 1.14E-124 278.6657189

AC135048.1 -1.635056813 1.109870161 -38.4187732 2.76E-126 2.25E-124 277.9801325

BMS1P4 -1.317858301 1.075246056 -38.35630752 4.34E-126 3.52E-124 277.5267804

EDRF1-AS1 -1.401363628 0.904874134 -38.12816202 2.28E-125 1.84E-123 275.8670666

COX10-AS1 -1.388319164 1.864217637 -38.10121929 2.77E-125 2.22E-123 275.6706564

AL513327.1 -1.815068219 2.104991424 -38.0607946 3.72E-125 2.97E-123 275.3758023

AC016876.2 -1.707243547 1.497427221 -38.03719134 4.42E-125 3.50E-123 275.2035525

AL135999.1 -2.012030259 2.436394832 -37.32519991 8.20E-123 6.46E-121 269.9764281

AC245052.4 -1.438741497 1.149337972 -37.26045864 1.32E-122 1.04E-120 269.4981169

AC239804.1 -1.101465619 0.547198201 -37.24840018 1.45E-122 1.13E-120 269.4089728

OR2A1-AS1 -2.288778967 2.202120457 -37.04211007 6.65E-122 5.15E-120 267.8812305

CU633904.1 -3.171392046 1.540499499 -37.04119085 6.70E-122 5.16E-120 267.8744115

AC087393.2 -2.959669049 1.436816072 -37.00096079 9.02E-122 6.91E-120 267.5758739

AGAP11 -1.275062894 0.690059806 -36.86264782 2.52E-121 1.92E-119 266.5479963

AC104794.5 -2.858608968 2.070972768 -36.78157295 4.61E-121 3.49E-119 265.9444106

AC091053.1 -2.735278267 1.327882174 -36.76903875 5.06E-121 3.81E-119 265.8510251

AC087623.1 -2.824048535 2.301849802 -36.72747563 6.89E-121 5.16E-119 265.5412248

MAMDC2-AS1 -0.831314128 0.586881258 -36.57209839 2.20E-120 1.64E-118 264.3812304

LINC00663 -1.858602873 2.052628468 -36.51165778 3.45E-120 2.56E-118 263.9292098

NEAT1 -4.755700817 7.852964256 -36.49800698 3.82E-120 2.80E-118 263.8270573

AL356599.1 -1.378734352 1.313121395 -36.49800313 3.82E-120 2.80E-118 263.8270285

AC132217.1 -3.712331883 1.802207622 -36.36288635 1.05E-119 7.67E-118 262.8146931

C8orf34-AS1 -1.688511046 1.02029762 -36.3418352 1.23E-119 8.93E-118 262.6567715

RNF144A-AS1 -2.729695364 2.111586604 -36.28662774 1.86E-119 1.34E-117 262.2423599

USP3-AS1 -1.486308915 1.404124021 -36.26983532 2.11E-119 1.52E-117 262.1162349

AP001453.1 -1.451042813 0.977647842 -36.21091459 3.28E-119 2.35E-117 261.6734196

ERVE-1 -3.501121469 2.604902626 -36.12604468 6.22E-119 4.42E-117 261.0348404

AC079329.1 -2.144237541 1.04095252 -36.04271902 1.16E-118 8.24E-117 260.4070249

KLF3-AS1 -2.157682637 2.188025571 -36.00164986 1.59E-118 1.12E-116 260.097278

AC009955.4 -1.868912981 1.359310303 -35.96864499 2.03E-118 1.43E-116 259.8482032

MRPL23-AS1 -1.270439807 0.736490547 -35.96692862 2.06E-118 1.44E-116 259.8352468

AC012181.1 -2.62710575 2.604160701 -35.94419563 2.45E-118 1.70E-116 259.6636075

AC016722.2 -1.388686179 1.127503804 -35.94133598 2.50E-118 1.73E-116 259.642012

AL162258.1 -1.09804896 0.541573836 -35.92497269 2.83E-118 1.94E-116 259.5184206

AC015674.1 -3.175551167 1.541619309 -35.86629229 4.40E-118 3.01E-116 259.0749398

AL359076.1 1.764535034 1.843528833 35.76053902 9.80E-118 6.67E-116 258.2746377

AC004951.1 -0.812730947 0.674775915 -35.60527537 3.18E-117 2.15E-115 257.0971737

BAIAP2-DT 1.594600238 2.927138384 35.55396817 4.69E-117 3.16E-115 256.7074274

EPB41L4A-AS1 -3.255895699 5.352790383 -35.47851704 8.32E-117 5.58E-115 256.1336881

AC011603.2 -3.41768169 1.829671053 -35.47400003 8.61E-117 5.75E-115 256.099318

LINC00894 -1.847470285 1.625262884 -35.47191742 8.75E-117 5.81E-115 256.0834705

AL671710.1 -1.250407998 1.144249605 -35.34955322 2.22E-116 1.47E-114 255.1514099

AC144548.1 -1.186888829 0.989818064 -35.3134998 2.92E-116 1.92E-114 254.8764356

ERVK13-1 -1.785791351 2.400268899 -35.31159797 2.96E-116 1.94E-114 254.8619262

SPAG5-AS1 -1.084305759 1.189019605 -35.23745811 5.22E-116 3.40E-114 254.2959511

AL136317.2 -0.683116513 0.339086968 -35.20630612 6.62E-116 4.30E-114 254.0579382

LIPE-AS1 -1.951903758 1.746185673 -35.10628449 1.42E-115 9.19E-114 253.2929255

LINC01128 -1.859675553 2.729388929 -34.83948806 1.10E-114 7.07E-113 251.2462929

ZNF451-AS1 -1.227729621 0.821683021 -34.75903887 2.04E-114 1.31E-112 250.6274272

AL356056.1 -0.912436104 0.453352143 -34.55212128 1.00E-113 6.40E-112 249.0320009

AC025271.4 -1.030362107 0.65334073 -34.52160264 1.27E-113 8.06E-112 248.796239

PPP3CB-AS1 -1.317140561 2.014741659 -34.39787957 3.30E-113 2.09E-111 247.839271

LINC01515 -1.320391795 0.912403639 -34.31046988 6.50E-113 4.09E-111 247.162031

AC239809.3 -1.347274913 0.76970316 -34.23686048 1.15E-112 7.20E-111 246.5909768

AC019205.1 -0.850029494 0.981222189 -34.23356124 1.18E-112 7.35E-111 246.5653658

AL390726.3 -1.644056036 0.798131847 -34.20759176 1.44E-112 8.95E-111 246.3637256

HPN-AS1 -1.894237227 1.436637722 -34.0745069 4.06E-112 2.51E-110 245.329069

AC021016.2 1.110863027 1.304775808 34.01102004 6.65E-112 4.09E-110 244.8347183

AL450384.2 -1.774692233 2.221686979 -33.88981651 1.71E-111 1.05E-109 243.8895515

AL031282.2 -1.221700303 1.361709311 -33.77406915 4.22E-111 2.57E-109 242.9852214

PSMD6-AS2 -1.478399614 1.166264396 -33.73532577 5.71E-111 3.47E-109 242.682147

AL591684.2 -1.496994997 0.982737201 -33.66065441 1.02E-110 6.19E-109 242.0974928

AL354892.2 1.682820765 1.62534644 33.65104999 1.10E-110 6.65E-109 242.0222426

AC092718.4 1.901779427 2.359420929 33.57997253 1.93E-110 1.15E-108 241.4649948

AL512625.1 -1.745564951 1.450856109 -33.56256229 2.21E-110 1.32E-108 241.3284021

TMEM161B-AS1 -1.581649906 2.474130768 -33.52948186 2.86E-110 1.70E-108 241.0687639

PWRN1 -1.998020194 1.043420894 -33.494933 3.75E-110 2.22E-108 240.7974543

Z94721.2 -0.274772739 0.197227919 -33.46771075 4.65E-110 2.74E-108 240.5835748

AC012065.4 -1.353985298 0.657312622 -33.42961617 6.27E-110 3.68E-108 240.284118

LINC01152 -1.878707917 1.457090712 -33.31210786 1.58E-109 9.23E-108 239.3592553

C5orf56 -1.470506333 2.273186659 -33.24626237 2.65E-109 1.54E-107 238.840255

CU638689.5 -1.494675861 0.776084946 -33.24133814 2.76E-109 1.60E-107 238.8014199

AC007368.1 -2.798759076 1.934894197 -33.2336402 2.93E-109 1.69E-107 238.7407038

F11-AS1 -1.919487785 1.443596654 -33.23349391 2.93E-109 1.69E-107 238.7395499

AL390195.1 -1.735774035 1.254628166 -33.13352928 6.45E-109 3.69E-107 237.9504211

AL137802.2 -1.769228189 1.182329678 -33.07798232 1.00E-108 5.70E-107 237.5113877

AF117829.1 -1.397720832 1.873474921 -33.03977808 1.35E-108 7.68E-107 237.2092037

AL590399.3 -1.129659735 0.55969209 -33.00894557 1.73E-108 9.77E-107 236.9651943

ZRANB2-AS2 -0.839549818 0.523961262 -32.94791515 2.80E-108 1.58E-106 236.4818463

AC099850.3 2.410326611 1.347623309 32.93224137 3.17E-108 1.78E-106 236.3576379

AC092802.2 -1.349070746 1.078292236 -32.8146597 8.04E-108 4.50E-106 235.4248677

DNAJC27-AS1 -2.004750543 1.469339704 -32.7881255 9.92E-108 5.53E-106 235.2141333

TRAF3IP2-AS1 -1.251958758 1.581873429 -32.73348835 1.53E-107 8.50E-106 234.779927

AC090114.2 1.014462182 1.393931176 32.5563065 6.27E-107 3.46E-105 233.3692694

HCG18 -1.29988614 2.745381846 -32.50394153 9.51E-107 5.24E-105 232.9516039

NAV2-AS1 -1.610299304 0.781744132 -32.49265757 1.04E-106 5.71E-105 232.8615574

LINC02609 -1.728307057 1.99958019 -32.42427542 1.80E-106 9.81E-105 232.3155229

AL050343.2 -1.138752823 1.070198782 -32.42385131 1.80E-106 9.81E-105 232.3121345

AL450306.1 -1.823592473 1.191476505 -32.35795602 3.05E-106 1.65E-104 231.7853986

TGFB2-OT1 -1.390899522 0.675233191 -32.32990261 3.82E-106 2.06E-104 231.5609873

PROX1-AS1 -1.430948435 1.013836498 -32.32356428 4.02E-106 2.16E-104 231.5102706

CU634019.1 -2.168747109 1.053855324 -32.29687897 4.97E-106 2.66E-104 231.2966904

AC243919.2 -1.315168419 0.931953596 -32.29438645 5.07E-106 2.71E-104 231.2767365

MKNK1-AS1 -1.305918213 0.920976233 -32.26942805 6.19E-106 3.29E-104 231.0768891

VPS33B-DT -0.779783159 0.53662579 -32.10516205 2.31E-105 1.22E-103 229.759623

AC026356.1 1.372561981 1.069872812 32.09739217 2.46E-105 1.30E-103 229.6972316

PRDM16-DT -3.179614298 3.149687501 -31.99946309 5.39E-105 2.84E-103 228.9102199

LINC00937 -1.184797516 0.813612196 -31.87398855 1.48E-104 7.75E-103 227.9000772

TMEM147-AS1 -1.789909768 2.842402328 -31.82720241 2.16E-104 1.13E-102 227.5229155

AL158163.2 -2.003104809 1.593435634 -31.80918781 2.49E-104 1.30E-102 227.3776194

AC147067.1 2.072637178 1.9426204 31.7609063 3.68E-104 1.91E-102 226.9880054

AL355312.4 4.168237814 2.236133152 31.74470851 4.19E-104 2.17E-102 226.8572296

ZBED3-AS1 -0.971910186 0.808839957 -31.68919773 6.57E-104 3.38E-102 226.4088033

AC004982.2 -2.705905747 3.280443906 -31.64138314 9.66E-104 4.95E-102 226.0222382

AC015802.1 -0.951616349 0.571587906 -31.63088493 1.05E-103 5.37E-102 225.9373252

AC091057.2 -0.956291556 0.716775022 -31.50495023 2.91E-103 1.48E-101 224.917645

LINC01828 -0.952649239 0.544236633 -31.47764427 3.63E-103 1.84E-101 224.6962889

HCP5 2.453406751 3.465727526 31.47062511 3.85E-103 1.94E-101 224.6393727

AC008969.1 -1.387455741 1.666204194 -31.37445566 8.39E-103 4.22E-101 223.8589438

ZNF337-AS1 -0.977558574 1.055787082 -31.35924808 9.49E-103 4.76E-101 223.7354258

AC010336.3 -1.368237901 0.664231764 -31.19804815 3.52E-102 1.76E-100 222.4243549

AC080112.1 1.355956945 2.316030585 31.11755021 6.77E-102 3.37E-100 221.7684292

AC109460.3 -0.747786291 0.789799024 -31.09613517 8.06E-102 4.00E-100 221.5937951

CU633906.1 -2.395411968 1.190342563 -30.92851809 3.17E-101 1.56E-99 220.224936

AL604028.1 1.342641114 2.129007191 30.92463764 3.27E-101 1.61E-99 220.1932042

LINC01140 -1.316292075 1.214489863 -30.9235013 3.30E-101 1.62E-99 220.1839117

SFTPD-AS1 -0.623068624 0.333034593 -30.89258816 4.25E-101 2.08E-99 219.9310531

AL022341.2 -1.040247871 0.658301123 -30.86116848 5.49E-101 2.68E-99 219.6739285

LINC02447 -1.664688465 1.16580496 -30.84046571 6.50E-101 3.16E-99 219.5044388

AC073896.4 2.048008222 2.965651604 30.82469115 7.40E-101 3.58E-99 219.3752594

DPP10-AS1 -2.910818941 2.094236495 -30.79943386 9.10E-101 4.39E-99 219.1683602

CCDC18-AS1 -2.155679962 3.481372783 -30.63992387 3.36E-100 1.62E-98 217.8598647

AC084855.2 -0.372277477 0.22476873 -30.53276202 8.11E-100 3.89E-98 216.9790062

GATA2-AS1 -2.068583796 2.215191241 -30.45144838 1.58E-99 7.56E-98 216.3096599

AC005307.1 -1.356857741 0.830142279 -30.43932496 1.75E-99 8.32E-98 216.2097933

AC107884.1 -1.44039326 1.28787128 -30.30395095 5.33E-99 2.53E-97 215.0934052

AC003681.1 -0.312987895 0.330304611 -30.27496384 6.77E-99 3.20E-97 214.8540604

AC021016.3 -1.646350968 1.38874897 -30.25310837 8.11E-99 3.82E-97 214.6735318

AC093525.4 -1.164547378 0.760804616 -30.24573659 8.62E-99 4.05E-97 214.6126266

AC020978.7 -0.663889337 0.584335442 -30.23602416 9.34E-99 4.37E-97 214.5323729

RBFADN -0.94997604 0.719647836 -30.1366425 2.12E-98 9.91E-97 213.7105075

LINC01354 -1.627085199 1.16108369 -30.13401051 2.17E-98 1.01E-96 213.6887248

AC073864.1 -1.2554948 0.609498921 -30.10117181 2.85E-98 1.32E-96 213.4168747

RNF139-AS1 -1.200692368 1.258548191 -30.08548961 3.24E-98 1.50E-96 213.2870047

TIPARP-AS1 -1.723576456 1.887010579 -29.98181255 7.65E-98 3.52E-96 212.4276478

THRB-AS1 -0.815706083 0.493555654 -29.94588933 1.03E-97 4.73E-96 212.1295763

AL157932.1 1.059712399 0.592535834 29.94481907 1.04E-97 4.76E-96 212.1206934

AL162274.1 -2.202761684 1.303385882 -29.9350202 1.13E-97 5.14E-96 212.0393586

AC064807.1 -1.618198821 2.419010592 -29.7651446 4.62E-97 2.10E-95 210.6274236

CU638689.4 -2.070985151 1.041197965 -29.75393609 5.07E-97 2.30E-95 210.5341373

AP005264.4 -1.424911093 0.718312569 -29.55178218 2.73E-96 1.23E-94 208.8489787

AC068385.1 -2.919853752 1.417487134 -29.54615237 2.86E-96 1.29E-94 208.8019762

AC136443.3 -1.166050573 0.61029362 -29.54002652 3.01E-96 1.35E-94 208.7508278

SNHG7 -2.715139894 5.33375983 -29.48905524 4.61E-96 2.06E-94 208.3250586

PRKCQ-AS1 -1.631014915 1.857917394 -29.47324339 5.26E-96 2.35E-94 208.1929152

AP000695.1 1.851383624 1.030140995 29.47169234 5.33E-96 2.37E-94 208.179951

LINC00662 -1.070279677 1.968040807 -29.45864643 5.94E-96 2.63E-94 208.0708974

AC000120.1 -0.736990534 0.619191888 -29.45037832 6.37E-96 2.81E-94 208.0017716

AL136982.1 -0.329085258 0.213472322 -29.40070934 9.65E-96 4.25E-94 207.586335

DTX2P1-UPK3BP1-PMS2P11 -1.207721291 1.479594617 -29.32651339 1.79E-95 7.88E-94 206.9651859

TNFRSF10A-AS1 1.979137338 1.775822708 29.31690631 1.94E-95 8.51E-94 206.8847083

AC087482.1 -2.444168343 1.640574766 -29.29667098 2.30E-95 1.00E-93 206.7151618

CCR5AS 1.689816976 1.00554894 29.23992656 3.70E-95 1.61E-93 206.2394462

AC006026.3 1.906200353 0.981702312 29.18313848 5.96E-95 2.59E-93 205.7629683

AC105446.1 2.625924633 1.546983091 29.17294322 6.50E-95 2.81E-93 205.6773834

AL589765.4 1.26148275 0.767137743 29.14600875 8.14E-95 3.51E-93 205.4512186

ZNF710-AS1 -2.405458781 3.21888503 -29.12077857 1.01E-94 4.32E-93 205.2392837

MAP3K14-AS1 -1.231202889 1.715915904 -29.08010305 1.42E-94 6.07E-93 204.8974425

BX088651.4 -1.036034231 0.610134587 -29.07634157 1.46E-94 6.24E-93 204.8658204

BX284668.2 -1.325224354 0.946288533 -29.07266545 1.51E-94 6.42E-93 204.8349142

AC138207.5 1.684782471 1.216316835 29.03121965 2.14E-94 9.06E-93 204.486353

AC092979.1 -4.778706846 3.517562023 -28.95380707 4.09E-94 1.73E-92 203.8347459

AC010976.1 -1.131478797 1.020080834 -28.93975098 4.61E-94 1.94E-92 203.7163525

AC026992.2 -0.518834197 0.320412123 -28.84911152 9.89E-94 4.16E-92 202.9523228

AC012254.3 -0.719715325 0.487606287 -28.83320527 1.13E-93 4.74E-92 202.8181402

AP002807.1 -2.39154256 3.014653222 -28.83271208 1.14E-93 4.75E-92 202.8139793

AC108471.2 -1.019337415 0.883180003 -28.82958027 1.17E-93 4.86E-92 202.787556

AC093752.3 -1.546977905 1.765703052 -28.79052039 1.62E-93 6.74E-92 202.457905

AC091132.4 -1.575880004 0.929566757 -28.75567416 2.17E-93 9.01E-92 202.1636584

AC016727.1 -1.461630086 2.054584629 -28.73120311 2.67E-93 1.10E-91 201.956933

GTF3C2-AS1 -1.087673866 0.71946656 -28.70332345 3.38E-93 1.39E-91 201.7213235

AP000350.7 -0.161996064 0.12435793 -28.58471902 9.22E-93 3.79E-91 200.717947

AC002116.1 -1.327373899 0.645315383 -28.57152652 1.03E-92 4.22E-91 200.6062347

AL391422.4 1.262738247 1.80832165 28.5711908 1.03E-92 4.22E-91 200.6033916

AP000446.1 -1.04209461 0.573144864 -28.55185563 1.22E-92 4.96E-91 200.439625

TMCC1-AS1 -0.958087377 1.06938238 -28.52101336 1.58E-92 6.42E-91 200.1783007

AC242842.1 2.061269778 1.316997314 28.50210116 1.86E-92 7.51E-91 200.018002

AC007192.2 -2.391538071 1.1610083 -28.49149866 2.03E-92 8.20E-91 199.9281168

NORAD 1.70791297 6.353425627 28.47991975 2.24E-92 9.01E-91 199.8299384

BMS1P4-AGAP5 -1.116663519 0.578635175 -28.41399531 3.91E-92 1.57E-90 199.2706506

AL592435.1 -1.099624739 1.109994112 -28.40912682 4.08E-92 1.63E-90 199.2293266

AL121603.2 0.940248539 1.318324409 28.39180831 4.73E-92 1.89E-90 199.0823029

LINC01137 1.608352603 2.973316647 28.36858314 5.75E-92 2.29E-90 198.8850784

MIR3936HG -1.696513141 2.518465926 -28.35346914 6.54E-92 2.60E-90 198.7566976

SNHG1 -2.310553126 5.353827774 -28.34365615 7.11E-92 2.81E-90 198.6733297

LINC02041 2.894079041 1.539077981 28.33177399 7.87E-92 3.10E-90 198.5723672

AC090617.5 1.768111454 1.496714414 28.3106943 9.41E-92 3.70E-90 198.3932116

AL589935.1 -0.90583668 0.581547327 -28.28497395 1.17E-91 4.58E-90 198.1745425

AC087741.1 -2.09445449 2.450323762 -28.28490263 1.17E-91 4.58E-90 198.173936

AC087623.3 -1.324542264 1.007619144 -28.28152557 1.21E-91 4.70E-90 198.145219

AL731566.2 -0.788576882 1.019015213 -28.25054411 1.57E-91 6.11E-90 197.8817023

AC025580.3 -0.913823864 0.541405868 -28.20211384 2.37E-91 9.19E-90 197.4695408

AC008522.1 -1.063716316 0.546265368 -28.18410086 2.76E-91 1.07E-89 197.3161709

AL158163.1 -1.813231216 1.6360245 -28.12608976 4.52E-91 1.75E-89 196.8219754

DARS-AS1 -1.078817292 0.915753305 -28.11770682 4.86E-91 1.87E-89 196.7505278

AC007620.2 -1.070701774 0.519788354 -28.10890367 5.23E-91 2.01E-89 196.6754897

FAM27E3 -1.540328143 1.33016446 -28.09423774 5.93E-91 2.27E-89 196.5504567

LINC01133 4.145706236 2.682130608 28.08551358 6.39E-91 2.44E-89 196.4760674

AC005586.1 1.185588931 0.781544507 28.06546244 7.58E-91 2.89E-89 196.3050604

GLIDR -1.238722475 1.621070474 -28.0422078 9.24E-91 3.51E-89 196.1066718

PRMT5-AS1 -0.892242443 0.674196928 -28.00113157 1.31E-90 4.97E-89 195.7560865

LINC00476 -1.321091811 1.784501721 -27.98423514 1.51E-90 5.72E-89 195.611817

CD2BP2-DT 1.366091525 1.916847518 27.94521511 2.11E-90 7.96E-89 195.2785154

LINC00671 -3.622693296 2.667397433 -27.93179913 2.37E-90 8.91E-89 195.1638767

LINC02614 -1.130161446 1.171093299 -27.91662808 2.70E-90 1.01E-88 195.034215

PSMA3-AS1 -1.614909837 4.336049914 -27.87903334 3.72E-90 1.39E-88 194.7127875

DLEU1 -1.117357537 1.491641099 -27.87707847 3.78E-90 1.41E-88 194.6960691

AC018904.1 1.61977056 1.984382949 27.80936741 6.75E-90 2.51E-88 194.1167122

AL355612.1 -1.792265526 1.039921116 -27.79768391 7.46E-90 2.77E-88 194.0166892

AC233263.6 -0.297413425 0.155647164 -27.79443959 7.67E-90 2.84E-88 193.9889116

LINC02532 -3.730133347 4.004177841 -27.79292952 7.77E-90 2.87E-88 193.975982

BX470102.1 3.42103021 2.109320918 27.76589537 9.79E-90 3.60E-88 193.7444639

TMEM92-AS1 1.687435849 0.986557746 27.75660937 1.06E-89 3.89E-88 193.6649194

AC008014.1 -1.794526246 1.902900769 -27.72735324 1.36E-89 4.98E-88 193.4142424

LINC02610 -1.925419659 1.507577986 -27.72517696 1.39E-89 5.06E-88 193.3955911

DM1-AS -1.723206285 2.111750377 -27.7206789 1.44E-89 5.25E-88 193.35704

AP002490.1 -0.735287103 0.80196126 -27.66624085 2.30E-89 8.35E-88 192.8902815

CR381653.1 -1.565168285 0.989895306 -27.66374656 2.35E-89 8.51E-88 192.8688867

AC073349.1 -1.031905507 0.787778992 -27.66252076 2.37E-89 8.57E-88 192.8583721

AL031600.3 -2.241018465 1.087936282 -27.62666192 3.23E-89 1.16E-87 192.5507063

LINC02585 1.370001392 0.725246567 27.59320354 4.30E-89 1.55E-87 192.2634989

LINC02145 -1.078338481 0.747346908 -27.54374261 6.57E-89 2.36E-87 191.8386829

AC004241.1 -0.97505579 0.883690145 -27.53436191 7.12E-89 2.55E-87 191.7580801

AC136475.3 -4.684134847 6.009772896 -27.50636876 9.06E-89 3.23E-87 191.5174901

AC015712.2 -0.952191795 1.417055162 -27.48320684 1.11E-88 3.93E-87 191.3183528

MIR497HG -1.267232662 1.372023604 -27.47104812 1.23E-88 4.36E-87 191.2137914

AC016738.1 -1.118676187 1.073085052 -27.44389767 1.55E-88 5.49E-87 190.9802427

AP000525.1 -1.960547436 1.690984629 -27.38882726 2.49E-88 8.79E-87 190.5062598

AL356966.1 -0.389383614 0.25657873 -27.33494058 3.96E-88 1.39E-86 190.0421202

CR559946.2 -0.930882956 0.858943454 -27.33465206 3.97E-88 1.39E-86 190.0396341

AC008556.1 -1.951130309 1.9649064 -27.32570765 4.28E-88 1.50E-86 189.9625604

DANCR -2.122318904 5.58473691 -27.2894236 5.86E-88 2.05E-86 189.6498056

AP001267.3 -1.377439634 1.299818504 -27.24579483 8.53E-88 2.98E-86 189.273538

AF131216.3 -3.17423447 2.046339762 -27.12418139 2.44E-87 8.47E-86 188.2235337

AC135457.1 -1.138332551 0.552620737 -27.10517392 2.87E-87 9.96E-86 188.0592684

DLEU2L -0.445991167 0.444772245 -27.09893194 3.03E-87 1.05E-85 188.0053152

AC025917.1 -1.326129596 1.470824593 -27.06716592 3.99E-87 1.38E-85 187.7306719

EML2-AS1 -1.751050374 0.88946224 -27.05333868 4.49E-87 1.55E-85 187.6110875

FTX -0.786407969 1.112323088 -27.03619485 5.21E-87 1.79E-85 187.4627888

AC019069.1 1.344364683 1.159889728 27.0274928 5.62E-87 1.93E-85 187.3875008

ADIRF-AS1 -2.256083018 2.245261328 -27.01191741 6.42E-87 2.20E-85 187.2527243

LINC01783 -1.072396709 0.701728585 -27.00861767 6.61E-87 2.26E-85 187.2241675

FAM13A-AS1 -1.16035312 1.660846425 -26.94456943 1.15E-86 3.92E-85 186.6696274

AP001189.3 1.884766266 1.327625326 26.9385382 1.21E-86 4.12E-85 186.6173836

LINC00857 1.867629552 1.448214958 26.90308533 1.65E-86 5.58E-85 186.3101983

EPCAM-DT -1.209293418 0.894298189 -26.81799311 3.44E-86 1.16E-84 185.5723161

AC108134.1 -2.108007363 2.519979749 -26.81307987 3.59E-86 1.21E-84 185.5296852

AC015922.2 2.181783365 2.579762402 26.80102431 3.99E-86 1.34E-84 185.4250705

LINC01614 3.097595825 1.695107936 26.65705348 1.39E-85 4.67E-84 184.1744447

AC104506.1 -1.204502358 0.58474387 -26.64833957 1.50E-85 5.03E-84 184.0986738

AC013460.1 -0.752730802 0.41088851 -26.62034499 1.92E-85 6.40E-84 183.8551912

AC078795.2 -0.657946467 0.319410051 -26.5929855 2.43E-85 8.10E-84 183.617146

AC020659.1 -2.524399298 2.313901255 -26.5658167 3.08E-85 1.02E-83 183.3806755

TMC3-AS1 -1.433067762 1.166120373 -26.51740162 4.69E-85 1.56E-83 182.9590747

LPP-AS2 0.851896047 1.253448939 26.51620593 4.74E-85 1.57E-83 182.9486593

PGM5P3-AS1 -0.891409766 0.522749978 -26.51119223 4.95E-85 1.63E-83 182.9049839

RNFT1-DT -0.948644758 0.81714495 -26.50336631 5.30E-85 1.74E-83 182.8368049

AC240565.1 -1.074782902 0.547831411 -26.46189505 7.60E-85 2.50E-83 182.4753937

ALOX12-AS1 -1.065724542 1.966093176 -26.43183332 9.88E-85 3.24E-83 182.2132919

AC007744.1 -0.487384318 0.237396406 -26.41198141 1.17E-84 3.84E-83 182.0401512

LINC00472 -1.283221993 1.285222278 -26.39682907 1.34E-84 4.37E-83 181.9079686

AC012146.1 -1.684553105 2.027827332 -26.39548821 1.36E-84 4.41E-83 181.8962702

AL132656.3 -0.375848373 0.224205786 -26.37796963 1.58E-84 5.13E-83 181.7434104

MALAT1 -2.415177989 6.206003041 -26.32645387 2.48E-84 8.02E-83 181.2937052

LINC01625 -1.517742079 0.916650856 -26.29390797 3.29E-84 1.06E-82 181.0094429

GAS5 -2.551895118 7.728995265 -26.25186305 4.75E-84 1.53E-82 180.6420383

TP53TG1 1.39247161 4.23552957 26.21293236 6.67E-84 2.15E-82 180.3016703

NAPA-AS1 -1.3568671 2.108109118 -26.20213015 7.34E-84 2.35E-82 180.2071973

AC004596.1 0.825266447 0.485485147 26.16911728 9.79E-84 3.14E-82 179.9183957

RPARP-AS1 -1.641075981 3.228316733 -26.15813932 1.08E-83 3.44E-82 179.8223319

AP003396.5 -2.005414547 0.973558799 -26.15085901 1.15E-83 3.66E-82 179.7586174

FP236241.1 -2.016296064 0.982210008 -26.1392525 1.27E-83 4.04E-82 179.6570293

AL109910.2 -1.618818152 0.940871181 -26.13677654 1.30E-83 4.12E-82 179.6353562

LINC00891 -0.25840708 0.174848992 -26.10438345 1.72E-83 5.46E-82 179.3517416

AC010719.1 -2.94734661 3.390295842 -26.10112765 1.77E-83 5.61E-82 179.3232293

LINC01138 -1.340748915 2.550923464 -26.06534842 2.43E-83 7.65E-82 179.0098191

AL132656.2 -0.780396497 0.991689568 -26.06288731 2.48E-83 7.80E-82 178.9882556

AC026471.4 1.586354263 2.125368874 26.06168135 2.51E-83 7.87E-82 178.9776892

AL691459.1 -1.141368808 0.554094734 -26.04110039 3.00E-83 9.40E-82 178.7973364

YEATS2-AS1 -0.839660258 1.011801469 -26.0407356 3.01E-83 9.41E-82 178.7941393

LINC01089 -2.021652236 3.397877744 -26.0299013 3.31E-83 1.03E-81 178.699178

FAM27C -0.696751839 0.444400963 -26.01313619 3.83E-83 1.19E-81 178.5522083

LINC02256 -1.493796122 1.502568059 -26.01046129 3.93E-83 1.22E-81 178.5287562

AL606489.1 1.723295189 1.042013215 26.00560118 4.10E-83 1.27E-81 178.4861433

AC087203.3 -0.754807212 0.51986323 -25.96495962 5.85E-83 1.81E-81 178.1297001

AC016590.1 -0.988927715 0.624909451 -25.91864979 8.78E-83 2.71E-81 177.7233218

AC010969.2 0.916660906 1.448741344 25.90313285 1.01E-82 3.09E-81 177.5871045

AC012557.1 -1.257229514 1.542287039 -25.89509393 1.08E-82 3.31E-81 177.5165235

AC009120.2 -1.558629713 2.814349464 -25.8678218 1.37E-82 4.20E-81 177.2770238

AL136526.1 -0.278570481 0.179017261 -25.8672699 1.38E-82 4.21E-81 177.2721763

AL132712.1 1.586490132 0.908079406 25.85915177 1.48E-82 4.51E-81 177.2008678

AC008467.1 -0.438649451 0.39299586 -25.8540429 1.55E-82 4.71E-81 177.1559885

AC090510.3 -1.128991409 0.548085938 -25.85321384 1.56E-82 4.73E-81 177.1487052

WAC-AS1 1.059436028 3.757508213 25.8417887 1.72E-82 5.22E-81 177.0483288

RARA-AS1 1.242675809 1.796234524 25.79198965 2.67E-82 8.07E-81 176.6106483

AC092384.2 -1.119505154 0.758958751 -25.7308934 4.57E-82 1.38E-80 176.0733073

AC097376.3 -0.912118975 1.361199743 -25.729708 4.61E-82 1.39E-80 176.0628777

SH3RF3-AS1 0.900616538 0.830543369 25.69435926 6.30E-82 1.89E-80 175.7517955

AC009065.2 2.546615996 2.088136275 25.66195669 8.37E-82 2.51E-80 175.4665213

RBM26-AS1 -1.005425191 1.221897326 -25.66148227 8.41E-82 2.52E-80 175.4623436

AP000879.1 -1.525499383 0.740576728 -25.63958559 1.02E-81 3.04E-80 175.2694983

LINC02389 -0.568434714 0.349608984 -25.62975851 1.11E-81 3.31E-80 175.1829337

SH3BP5-AS1 -1.576970161 2.683621003 -25.6157717 1.26E-81 3.74E-80 175.059709

AP001062.3 -1.334686912 0.64794393 -25.60354824 1.40E-81 4.15E-80 174.9520023

AC046143.2 1.627498754 0.94996558 25.58018784 1.72E-81 5.09E-80 174.7461176

LINC00920 1.636484897 1.10655644 25.57341208 1.83E-81 5.39E-80 174.6863889

AL096870.2 -0.706636059 1.144653196 -25.5234432 2.84E-81 8.36E-80 174.2457588

SPDYE11 -0.209793197 0.101847271 -25.52057091 2.91E-81 8.56E-80 174.2204224

SNHG21 -1.181665154 2.115769081 -25.51106582 3.16E-81 9.29E-80 174.1365723

AC027644.3 -1.528373841 2.643081209 -25.5078806 3.25E-81 9.53E-80 174.1084714

AC005034.3 0.943920946 2.749516146 25.46145419 4.90E-81 1.43E-79 173.6987607

AC068888.1 -1.483904366 2.86282738 -25.44250252 5.79E-81 1.69E-79 173.5314468

AL049838.1 1.34426266 1.24612501 25.41926181 7.11E-81 2.07E-79 173.3262148

GPRC5D-AS1 -1.415888846 2.585113994 -25.38076458 9.99E-81 2.90E-79 172.9861299

KDM7A-DT 1.490512638 1.927551655 25.33652474 1.48E-80 4.28E-79 172.5951197

AC009955.3 -1.268526116 0.902662018 -25.30479947 1.95E-80 5.65E-79 172.3145905

AC025857.2 2.253030003 1.868860523 25.26974471 2.66E-80 7.69E-79 172.0044961

HCG11 1.430285234 1.762450908 25.26837652 2.70E-80 7.77E-79 171.9923905

TMEM51-AS1 -1.811883848 2.47831443 -25.24821857 3.22E-80 9.27E-79 171.8140115

SERTAD4-AS1 1.890942212 1.896668238 25.22645118 3.91E-80 1.12E-78 171.6213423

AC015971.1 -1.111799897 0.8317973 -25.22598655 3.92E-80 1.12E-78 171.6172291

AL031320.2 -0.485726128 0.537282961 -25.22115693 4.09E-80 1.17E-78 171.5744739

AL161421.1 1.282571466 1.61489741 25.20461531 4.74E-80 1.35E-78 171.4280167

NRAV 1.119681578 2.728377057 25.19250479 5.27E-80 1.50E-78 171.3207735

SNHG26 -1.599784998 1.488198656 -25.18440086 5.67E-80 1.61E-78 171.2490017

LINC01560 0.897513974 1.714039969 25.16134617 6.95E-80 1.97E-78 171.0447816

AP001107.2 -2.297101021 1.115162407 -25.14498057 8.03E-80 2.27E-78 170.8997801

AC096677.1 1.511829179 1.205823285 25.13899753 8.47E-80 2.39E-78 170.8467626

AC139099.1 -1.090208066 0.551304584 -25.12843302 9.30E-80 2.62E-78 170.7531379

RAB30-DT -1.301839807 2.963290494 -25.1198611 1.00E-79 2.82E-78 170.6771635

AP003555.1 1.497889933 0.828989499 25.1004635 1.19E-79 3.35E-78 170.5052106

LINC00173 -1.501844464 1.030781052 -25.09228793 1.28E-79 3.59E-78 170.4327253

FOXD2-AS1 1.433018431 1.414540483 25.06571428 1.62E-79 4.53E-78 170.1970727

AC005944.1 -2.325097752 1.128753843 -25.0585588 1.73E-79 4.82E-78 170.133606

U73166.1 -1.075735146 1.080543264 -25.02529004 2.32E-79 6.46E-78 169.8384533

LINC00963 -1.80198184 4.273810204 -25.01987119 2.43E-79 6.77E-78 169.7903675

LINC01670 -0.952861038 0.470833139 -24.96551129 3.94E-79 1.09E-77 169.3078209

LINC02568 -2.032480741 1.548367531 -24.88853948 7.81E-79 2.16E-77 168.6240279

AL009179.1 -0.944907649 0.891836206 -24.86452745 9.67E-79 2.67E-77 168.4105876

PXN-AS1 -1.375021512 2.360856012 -24.83575442 1.25E-78 3.44E-77 168.1547494

PGM5P4-AS1 -1.085711908 0.670276566 -24.82261171 1.40E-78 3.86E-77 168.0378614

SEMA3F-AS1 -0.991905607 1.508885531 -24.8060073 1.63E-78 4.47E-77 167.8901607

LINC02825 -0.740788207 0.415066328 -24.76559103 2.33E-78 6.39E-77 167.5305292

AC010401.1 -0.188547878 0.129290037 -24.74725613 2.74E-78 7.50E-77 167.3673269

AP001350.1 -0.425163564 0.350696315 -24.73267207 3.12E-78 8.53E-77 167.2374871

LINC01251 -2.415958387 1.624826909 -24.7317244 3.15E-78 8.58E-77 167.2290493

AC104758.2 1.03442178 0.557701076 24.73088268 3.17E-78 8.63E-77 167.2215549

C8orf49 -1.401729627 1.305068618 -24.70133144 4.13E-78 1.12E-76 166.9583927

AC110048.2 -0.174250833 0.143369729 -24.67854712 5.05E-78 1.37E-76 166.7554313

WARS2-AS1 -1.054417702 1.983721777 -24.66940569 5.48E-78 1.48E-76 166.6739852

AL354733.3 -1.614916735 2.298674827 -24.62608411 8.07E-78 2.18E-76 166.2878943

LINC01578 -1.554506962 5.089848257 -24.61838868 8.64E-78 2.33E-76 166.2192912

AC123768.3 -0.84048313 0.673104851 -24.61029851 9.28E-78 2.50E-76 166.1471627

AC005253.1 -1.115044679 1.861597737 -24.59133786 1.10E-77 2.95E-76 165.9780917

AL132989.1 1.393719255 1.300685229 24.58759961 1.14E-77 3.04E-76 165.9447538

RGMB-AS1 -1.191142331 1.196496952 -24.57043004 1.32E-77 3.54E-76 165.7916161

AP000892.3 -2.319070993 2.080543984 -24.54123619 1.72E-77 4.59E-76 165.5311644

NAV2-AS2 -0.935802048 0.569808851 -24.53406686 1.83E-77 4.88E-76 165.4671906

BX005266.2 -1.326155875 0.736107145 -24.49692887 2.55E-77 6.78E-76 165.1357157

AC005381.1 -1.27701868 0.943561397 -24.46850051 3.29E-77 8.72E-76 164.8818857

AC010210.1 -1.139001423 1.059932963 -24.4624542 3.47E-77 9.19E-76 164.8278893

AL110504.1 -1.464434353 0.710931786 -24.43780423 4.32E-77 1.14E-75 164.6077156

AC061975.6 -0.795093343 0.441991528 -24.41995119 5.07E-77 1.34E-75 164.4482146

AC092384.1 -0.602829441 0.481072503 -24.41821642 5.15E-77 1.36E-75 164.4327143

DICER1-AS1 -1.601709596 2.516943161 -24.3966287 6.25E-77 1.64E-75 164.2398013

AC025171.1 1.082199062 1.274764588 24.38535245 6.91E-77 1.81E-75 164.1390157

AL121839.2 -1.799741645 2.431301915 -24.36087037 8.60E-77 2.25E-75 163.9201552

AC239799.1 -0.338474235 0.200226187 -24.34879772 9.58E-77 2.50E-75 163.8122084

AP002748.4 -1.223274811 2.18973684 -24.34811055 9.63E-77 2.51E-75 163.8060637

AC016735.1 2.985386353 2.069248677 24.34607078 9.81E-77 2.56E-75 163.7878236

AL138756.1 0.797089223 1.346888334 24.33223208 1.11E-76 2.89E-75 163.6640646

AP000695.2 1.57769237 0.973527963 24.31906075 1.25E-76 3.24E-75 163.5462563

Z84484.1 0.672075623 0.360488127 24.30459153 1.42E-76 3.68E-75 163.4168196

AC114730.3 -1.48417373 1.815688906 -24.29442182 1.56E-76 4.02E-75 163.3258327

AL049555.1 1.92301396 2.131598016 24.29438846 1.56E-76 4.02E-75 163.3255341

FAM87A -1.423106642 0.871162814 -24.2858918 1.68E-76 4.33E-75 163.2495079

AL392089.1 -1.312500645 0.777292445 -24.28466716 1.70E-76 4.37E-75 163.2385495

AC005670.3 -1.050804832 2.059218321 -24.27133423 1.91E-76 4.91E-75 163.1192336

LINC00689 -0.519608649 0.307102467 -24.24915345 2.33E-76 5.98E-75 162.9207

AC005261.3 1.167750053 2.563235955 24.22327203 2.94E-76 7.53E-75 162.6889825

MIR17HG -1.482186009 1.414989433 -24.22076513 3.01E-76 7.68E-75 162.6665346

AL049597.2 0.843641382 1.071610667 24.17154463 4.68E-76 1.19E-74 162.2256695

AC133785.1 -0.946634449 0.710183327 -24.11278993 7.91E-76 2.01E-74 161.6991002

AL139246.3 2.269531705 1.485795307 24.11140836 8.01E-76 2.03E-74 161.6867143

AGBL5-IT1 1.214477312 0.976503491 24.07635209 1.10E-75 2.78E-74 161.3723712

SACS-AS1 -0.199713273 0.121481048 -24.04592947 1.44E-75 3.65E-74 161.0994816

AL022069.1 -0.399768491 0.336779996 -24.0328065 1.62E-75 4.09E-74 160.9817418

LINC02477 -0.876154073 0.459041108 -24.00456172 2.09E-75 5.26E-74 160.7282726

AL050309.1 0.85224227 0.965114055 23.99748704 2.23E-75 5.60E-74 160.6647724

AC004889.1 -1.167171183 0.851676169 -23.99455095 2.28E-75 5.74E-74 160.6384176

AC012181.2 -1.647062975 2.274328392 -23.9191809 4.49E-75 1.13E-73 159.9616027

AC091132.1 -0.939310432 0.518760157 -23.88144558 6.31E-75 1.58E-73 159.6225419

AC098484.1 1.082566846 1.28169605 23.84053269 9.11E-75 2.28E-73 159.2547786

LINC02062 1.202981169 0.781597693 23.83884336 9.25E-75 2.31E-73 159.23959

AC008079.1 -0.153691648 0.188183565 -23.83735508 9.37E-75 2.33E-73 159.2262087

ALG13-AS1 1.270681097 0.661966586 23.81975925 1.10E-74 2.73E-73 159.0679875

AC078795.1 -0.479970768 0.521645936 -23.80084115 1.30E-74 3.23E-73 158.8978441

LINC01389 1.116271984 0.685802499 23.77904997 1.58E-74 3.92E-73 158.7018199

LINC01359 -0.803900374 0.653641407 -23.77408934 1.66E-74 4.09E-73 158.65719

AC115522.1 0.931468783 0.544181071 23.7529667 2.00E-74 4.94E-73 158.4671279

SUCLA2-AS1 0.886578452 0.511180794 23.7082807 2.99E-74 7.37E-73 158.0649054

SEPTIN4-AS1 -0.810448491 0.922070425 -23.70280898 3.14E-74 7.73E-73 158.0156414

AC006504.5 -0.89850534 1.633945631 -23.67845294 3.91E-74 9.61E-73 157.7963207

AL117339.4 -0.414678291 0.400137074 -23.67021818 4.21E-74 1.03E-72 157.7221562

LINC02347 -0.725906608 0.438078271 -23.61333679 7.03E-74 1.72E-72 157.2096969

LINC02381 2.281934972 3.286718562 23.59080024 8.62E-74 2.10E-72 157.0065774

AC034229.3 -0.243783545 0.118348399 -23.57050843 1.03E-73 2.52E-72 156.8236499

SNHG9 -2.35672119 4.825875565 -23.55224346 1.22E-73 2.97E-72 156.6589622

AC009065.1 -0.945402454 0.583040587 -23.54229586 1.33E-73 3.24E-72 156.5692559

KDM4A-AS1 -0.955816592 1.136163612 -23.5398102 1.36E-73 3.31E-72 156.5468391

AC040977.1 1.585445409 2.4113745 23.52715401 1.53E-73 3.70E-72 156.4326914

LINC01238 -1.595505925 1.406742596 -23.50890165 1.80E-73 4.36E-72 156.2680457

AC090425.2 1.432225142 0.822220842 23.49146877 2.11E-73 5.09E-72 156.110764

ADD3-AS1 -0.553302263 0.430168796 -23.47604936 2.42E-73 5.84E-72 155.9716253

LINC01843 1.6549113 1.537161221 23.46410879 2.70E-73 6.49E-72 155.8638636

GOLGA8M -0.646526223 0.389639079 -23.4625959 2.74E-73 6.57E-72 155.8502091

AC139887.2 -1.291389727 2.115932145 -23.45562301 2.91E-73 6.99E-72 155.7872731

AL590666.2 1.971150687 1.357877286 23.45106688 3.04E-73 7.27E-72 155.7461479

AC130324.3 -1.235886264 0.599979661 -23.42916203 3.70E-73 8.84E-72 155.5484014

LINC00624 -0.383400971 0.34783551 -23.39615166 4.99E-73 1.19E-71 155.2503187

ERVH48-1 -0.422540118 0.288978753 -23.3951498 5.03E-73 1.20E-71 155.2412704

AC019080.1 -0.604916188 0.918651512 -23.37142098 6.23E-73 1.48E-71 155.0269378

SDCBP2-AS1 -0.953965725 1.674432802 -23.35311578 7.35E-73 1.74E-71 154.8615603

SNHG5 -2.37895382 7.126843494 -23.35114341 7.49E-73 1.77E-71 154.8437392

AC106038.1 -0.281749793 0.222942851 -23.34671181 7.79E-73 1.84E-71 154.8036969

ROCR -2.482656425 2.155224862 -23.32600588 9.39E-73 2.22E-71 154.6165828

FREM2-AS1 -2.472918972 1.371277437 -23.31695759 1.02E-72 2.40E-71 154.5348038

AC007686.3 -0.964008853 1.275261959 -23.29935115 1.20E-72 2.81E-71 154.3756551

AC092683.2 -0.749308794 0.387460488 -23.29764774 1.21E-72 2.85E-71 154.3602562

AC011700.1 -0.739930067 0.606765834 -23.29302661 1.27E-72 2.97E-71 154.3184795

AL353150.1 -2.387559443 2.442858058 -23.28547085 1.35E-72 3.17E-71 154.2501687

AF001548.2 1.522134435 1.846981707 23.27295842 1.52E-72 3.55E-71 154.1370341

AC010329.1 -1.908449897 1.107527602 -23.26649567 1.61E-72 3.75E-71 154.078594

LINC02334 -0.298086619 0.241367529 -23.23517436 2.13E-72 4.97E-71 153.7953161

AC245140.2 -1.049440142 1.708670712 -23.21543182 2.55E-72 5.93E-71 153.6167155

AC025811.1 -1.661986625 1.13917659 -23.2128888 2.61E-72 6.06E-71 153.5937076

LINC00339 -2.221607263 5.360556676 -23.19745485 3.00E-72 6.96E-71 153.4540577

LINC00940 -0.879342739 0.584027397 -23.19231726 3.15E-72 7.28E-71 153.407567

AL122035.1 1.127839728 1.527342511 23.18611544 3.33E-72 7.69E-71 153.3514429

LINC02728 -0.725530541 0.540528585 -23.16302925 4.10E-72 9.45E-71 153.1424924

AL020996.1 -0.65015379 0.873088372 -23.11404375 6.39E-72 1.47E-70 152.6989779

MIR217HG -2.897055055 2.146009841 -23.10125409 7.17E-72 1.65E-70 152.5831466

LINC02417 -1.667190433 0.986103813 -23.05680373 1.07E-71 2.46E-70 152.1804664

AL162274.2 -2.136046087 2.757925798 -23.04826022 1.16E-71 2.65E-70 152.1030507

AC008763.1 -1.216377808 1.513000579 -23.04387777 1.21E-71 2.76E-70 152.0633373

AL365203.2 1.475600038 2.063453351 23.02140123 1.48E-71 3.38E-70 151.8596315

ARHGAP5-AS1 1.275113646 2.161677247 23.00727092 1.68E-71 3.83E-70 151.731546

AC004982.1 -1.507406575 1.681962559 -22.99419438 1.89E-71 4.31E-70 151.6129973

AC068987.1 -0.807806522 0.392161879 -22.97800543 2.19E-71 4.98E-70 151.4662125

BX571846.1 -0.537047011 0.277852461 -22.94162384 3.05E-71 6.91E-70 151.1362609

BX293535.1 1.592726015 0.904689837 22.93951285 3.10E-71 7.03E-70 151.1171125

AC108463.2 0.998920718 0.646346494 22.93843752 3.13E-71 7.09E-70 151.1073583

SRRM2-AS1 -0.570521432 0.591900328 -22.93530767 3.23E-71 7.28E-70 151.078967

AC091982.3 1.143809866 1.681829343 22.91347208 3.93E-71 8.86E-70 150.8808711

MEG8 -1.568185575 0.927276633 -22.88716221 4.99E-71 1.12E-69 150.642131

AL359715.1 -1.213811041 1.458942313 -22.87800821 5.42E-71 1.22E-69 150.5590527

AC127502.2 -1.379090013 2.666904464 -22.87510146 5.57E-71 1.25E-69 150.5326706

AC134312.5 1.506952571 0.873199098 22.87338234 5.66E-71 1.27E-69 150.5170673

AC019197.1 -1.211932491 1.026348821 -22.86744582 5.97E-71 1.34E-69 150.4631836

LINC02028 -0.622765091 0.394086857 -22.85447875 6.71E-71 1.50E-69 150.3454759

AC079779.2 -1.117696664 0.91687311 -22.8460661 7.25E-71 1.62E-69 150.2691032

LINC02544 2.423065811 1.266824382 22.83066179 8.33E-71 1.86E-69 150.1292428

AC004951.4 -0.591501942 0.650052624 -22.81108795 9.95E-71 2.21E-69 149.9514979

AC025580.1 1.895227063 1.102173502 22.77105293 1.43E-70 3.18E-69 149.5878524

AP002409.1 -0.939332847 0.501914766 -22.74700907 1.78E-70 3.95E-69 149.3693947

CTBP1-AS -0.956647937 1.487743311 -22.74625688 1.79E-70 3.97E-69 149.3625596

AP005233.2 3.560171519 2.082801907 22.69281875 2.91E-70 6.44E-69 148.8768598

AC073569.2 1.159395267 0.656944168 22.67340887 3.48E-70 7.67E-69 148.7003861

LINC00667 -1.205230524 3.849338778 -22.67149584 3.54E-70 7.79E-69 148.6829913

MEIS1-AS2 -0.750418137 0.623231015 -22.67027484 3.58E-70 7.86E-69 148.6718888

AC009065.5 2.26893891 2.29583615 22.65767751 4.01E-70 8.80E-69 148.5573351

ZNF213-AS1 -1.210193784 2.919699428 -22.63300502 5.02E-70 1.10E-68 148.3329391

AC087500.2 -1.14517055 1.300618341 -22.6218278 5.56E-70 1.22E-68 148.2312664

AC092143.2 -1.048530126 0.533533651 -22.6121812 6.07E-70 1.33E-68 148.1435089

AC080013.6 1.51660824 1.171235203 22.60478193 6.49E-70 1.42E-68 148.0761908

AC092535.2 -0.92737852 0.550627125 -22.59342121 7.19E-70 1.57E-68 147.9728233

AC003965.2 1.873207673 1.07517404 22.56630235 9.21E-70 2.00E-68 147.7260362

U62317.1 1.831841285 1.20624723 22.56141076 9.63E-70 2.09E-68 147.6815156

AC015813.1 -1.545380446 2.919911117 -22.56044055 9.71E-70 2.11E-68 147.672685

AC100827.4 -0.178971199 0.136291297 -22.55927217 9.82E-70 2.12E-68 147.6620507

LINC00205 1.071134668 2.273331853 22.54550955 1.11E-69 2.40E-68 147.5367782

AL365181.3 2.25769225 1.590719769 22.5272595 1.31E-69 2.83E-68 147.3706364

AC104964.3 -0.129899815 0.100907096 -22.5209505 1.39E-69 3.00E-68 147.3131955

TTLL7-IT1 -0.177106319 0.098793969 -22.48284117 1.97E-69 4.23E-68 146.9661591

AL161756.1 -0.349861334 0.337870933 -22.46833009 2.25E-69 4.83E-68 146.8339865

SEPSECS-AS1 -1.048955519 1.207207746 -22.45489795 2.54E-69 5.44E-68 146.7116267

GAS1RR -0.719607838 0.659352181 -22.44876893 2.68E-69 5.75E-68 146.6557899

CHMP1B-AS1 -1.644545768 0.798369595 -22.42771799 3.25E-69 6.95E-68 146.4639887

Z93930.2 -1.124307214 1.548615742 -22.4230249 3.39E-69 7.25E-68 146.421224

AC147651.4 1.465531894 1.263376937 22.39666608 4.31E-69 9.20E-68 146.1810034

ZNF503-AS1 -1.676821435 1.606587939 -22.37866053 5.08E-69 1.08E-67 146.0168795

AC245060.6 -0.525306008 0.662311472 -22.37227275 5.39E-69 1.15E-67 145.9586477

AC008438.1 1.153029128 0.921079493 22.37123895 5.44E-69 1.15E-67 145.9492233

AC092040.1 -0.160008982 0.100067896 -22.36806615 5.60E-69 1.19E-67 145.9202982

AL449403.2 -1.408208047 1.215833575 -22.35635152 6.23E-69 1.32E-67 145.8134944

C11orf72 -0.260632552 0.165557343 -22.31449944 9.12E-69 1.93E-67 145.4318382

AC073842.1 -1.463910363 1.034240097 -22.30540588 9.91E-69 2.09E-67 145.348895

FGF10-AS1 -0.396745155 0.281107318 -22.26661654 1.41E-68 2.97E-67 144.9950235

MIR1915HG 0.677305434 0.703515921 22.22110593 2.14E-68 4.50E-67 144.5796907

AC009318.1 -0.933678741 0.960932097 -22.21040203 2.36E-68 4.95E-67 144.4819837

AP007216.2 1.308856836 0.733165164 22.197194 2.66E-68 5.58E-67 144.361407

AL627309.3 -0.690009163 0.334975371 -22.19547933 2.70E-68 5.66E-67 144.3457528

AC093797.1 -1.240671498 1.505516892 -22.18223619 3.05E-68 6.38E-67 144.2248408

AC092611.1 -0.490137372 0.380749235 -22.16827316 3.46E-68 7.23E-67 144.0973422

AL357140.2 0.812860342 0.598570833 22.16421239 3.59E-68 7.49E-67 144.0602599

NPTN-IT1 -0.889483161 1.244293343 -22.14675619 4.22E-68 8.78E-67 143.9008392

EXTL3-AS1 -0.662554068 0.623698839 -22.14295356 4.37E-68 9.07E-67 143.8661083

AL357033.4 1.260438661 1.775418907 22.11759061 5.50E-68 1.14E-66 143.6344315

AC022150.2 1.151881346 1.717879148 22.1127475 5.75E-68 1.19E-66 143.5901869

AL022323.1 1.200584642 1.088904635 22.10176186 6.36E-68 1.32E-66 143.4898205

AL445231.1 -0.705568884 0.727972054 -22.09380522 6.84E-68 1.41E-66 143.4171221

AL109811.2 -1.637150687 3.21754113 -22.0730679 8.26E-68 1.71E-66 143.2276272

PARD6G-AS1 -1.236135985 1.23533813 -22.06495175 8.90E-68 1.83E-66 143.1534544

AC010226.1 -1.121184555 1.583393159 -22.06432584 8.95E-68 1.84E-66 143.147734

C4B-AS1 -1.123796939 0.54557726 -22.04529254 1.07E-67 2.19E-66 142.9737708

ADAMTS9-AS2 -0.817295313 0.816664123 -22.04512108 1.07E-67 2.19E-66 142.9722037

C4A-AS1 -1.123732801 0.545610262 -22.04401684 1.08E-67 2.21E-66 142.9621102

AC136944.2 -0.135171988 0.06668133 -21.99539716 1.68E-67 3.44E-66 142.5176069

CHROMR -1.025020673 2.671708759 -21.95722799 2.38E-67 4.87E-66 142.168529

AC060766.4 1.212357693 1.438487251 21.92887462 3.09E-67 6.30E-66 141.9091554

ATP1A1-AS1 -1.015625719 1.932408813 -21.92345153 3.24E-67 6.61E-66 141.8595392

AC021491.4 -0.646302014 0.456306627 -21.91540417 3.49E-67 7.10E-66 141.7859094

MATN1-AS1 -0.887496828 1.353811708 -21.90835243 3.73E-67 7.56E-66 141.7213854

AP006621.2 1.524441912 1.201313523 21.8834495 4.68E-67 9.49E-66 141.4934937

SNHG30 1.194420556 2.872455085 21.87407214 5.10E-67 1.03E-65 141.4076685

AL157394.1 0.790024608 0.951234273 21.87127044 5.23E-67 1.06E-65 141.3820251

SNHG12 -1.792893336 4.314923849 -21.8664899 5.46E-67 1.10E-65 141.3382684

NKILA 1.963783685 2.003352086 21.86457906 5.56E-67 1.12E-65 141.3207779

AL031722.1 0.801171746 0.413126546 21.8512434 6.28E-67 1.26E-65 141.1987055

FAM182A -0.388060838 0.235786737 -21.84107248 6.90E-67 1.39E-65 141.1055943

AC008736.1 1.102152392 1.370622356 21.83378932 7.37E-67 1.48E-65 141.0389152

AC114291.1 -1.365556905 1.0414569 -21.82845732 7.74E-67 1.55E-65 140.9900972

AC145207.3 -2.405009614 1.167548265 -21.82148671 8.25E-67 1.65E-65 140.9262735

EAF1-AS1 -0.583242716 0.365158516 -21.81924055 8.42E-67 1.68E-65 140.9057067

C3orf35 -0.886191515 1.024904796 -21.81345109 8.88E-67 1.77E-65 140.8526944

AC244090.1 1.241503323 3.127989912 21.77501117 1.26E-66 2.51E-65 140.5006537

AC020656.2 -1.489484788 1.367659687 -21.77499167 1.26E-66 2.51E-65 140.5004752

AC097662.1 -0.737319455 0.795086328 -21.74372102 1.68E-66 3.34E-65 140.2140181

AC008655.1 -1.869632522 0.907641362 -21.73837513 1.77E-66 3.50E-65 140.16504

LINC00324 0.859984907 1.291920568 21.69789818 2.56E-66 5.07E-65 139.7941357

AL117190.1 -1.842478363 1.159889667 -21.69656341 2.59E-66 5.13E-65 139.7819029

AC010680.4 -0.99264576 1.092124032 -21.68951426 2.76E-66 5.46E-65 139.7172972

BX322234.1 1.159584364 0.991364012 21.65203087 3.90E-66 7.69E-65 139.3737056

AC040162.3 -0.771459243 1.198130805 -21.63578262 4.52E-66 8.91E-65 139.2247371

CU638689.1 -0.728082108 0.353458458 -21.61988163 5.23E-66 1.03E-64 139.0789356

AC095057.3 -1.370422785 2.057942526 -21.55985465 9.07E-66 1.78E-64 138.5283793

AC133550.2 -2.720732553 1.32316723 -21.54827023 1.01E-65 1.98E-64 138.4221023

CFLAR-AS1 -0.809463785 0.786167993 -21.52836266 1.21E-65 2.37E-64 138.2394475

LINC00923 -1.04883454 0.704488531 -21.52076666 1.30E-65 2.54E-64 138.1697464

PTOV1-AS2 -1.57004925 3.657160925 -21.50646344 1.48E-65 2.89E-64 138.0384897

CATIP-AS1 -0.912721156 0.901813198 -21.49074424 1.71E-65 3.34E-64 137.8942239

AC126773.4 0.803849493 0.507008508 21.48772095 1.76E-65 3.43E-64 137.8664753

AP006545.1 -0.863970181 0.833035921 -21.48419075 1.82E-65 3.53E-64 137.8340735

AC023157.3 1.209895201 1.534850948 21.47582525 1.96E-65 3.81E-64 137.7572877

AL845552.2 -0.984943161 0.804913499 -21.46165814 2.23E-65 4.33E-64 137.6272396

AC024361.1 -1.025969369 1.377554108 -21.44490612 2.60E-65 5.05E-64 137.473447

AC022509.3 1.343840814 1.240027404 21.43934769 2.74E-65 5.30E-64 137.4224137

AC023355.1 -0.399365533 0.385405199 -21.43812044 2.77E-65 5.36E-64 137.4111457

AC062037.2 1.578642867 0.813162559 21.42812446 3.04E-65 5.86E-64 137.3193646

LINC01705 2.099650138 1.10101768 21.42271158 3.19E-65 6.15E-64 137.2696619

SNHG22 -1.107022791 1.240997034 -21.42252809 3.20E-65 6.16E-64 137.2679771

AC009542.1 -0.446753442 0.330688623 -21.40412905 3.79E-65 7.28E-64 137.0990179

AC009093.1 1.15734882 0.749719257 21.39574012 4.09E-65 7.85E-64 137.0219751

ERICD 0.689823504 0.720671802 21.39447006 4.14E-65 7.93E-64 137.0103106

AFAP1-AS1 2.748829904 1.589556775 21.39094084 4.27E-65 8.18E-64 136.9778971

AC243585.2 -0.987413799 0.479354947 -21.38312561 4.59E-65 8.78E-64 136.9061168

AC010615.2 -0.651527512 0.651330618 -21.38040805 4.71E-65 8.99E-64 136.881156

AC022509.2 1.108176012 0.917752206 21.3706633 5.15E-65 9.82E-64 136.7916468

MORF4L2-AS1 -0.662021448 0.799900434 -21.36382879 5.48E-65 1.04E-63 136.7288657

LINC00899 -1.096588591 1.824415653 -21.36382639 5.48E-65 1.04E-63 136.7288437

STX18-AS1 -0.654341229 0.886603907 -21.35941607 5.71E-65 1.08E-63 136.6883294

AC023830.3 -0.357580582 0.294488557 -21.33514248 7.14E-65 1.35E-63 136.4653253

AC068446.2 -0.530307567 0.264400117 -21.30788236 9.17E-65 1.74E-63 136.2148407

AL590705.3 0.88432828 0.547611067 21.29392213 1.04E-64 1.97E-63 136.0865472

AC104211.1 -0.240050869 0.280494576 -21.24404064 1.65E-64 3.11E-63 135.6280444

AC040970.1 1.233893123 1.39351307 21.22797139 1.91E-64 3.60E-63 135.4803068

AL139393.3 1.632092491 1.35967415 21.22335275 1.99E-64 3.76E-63 135.4378411

AC005920.3 -0.629733746 0.408593352 -21.21068611 2.24E-64 4.21E-63 135.3213723

HECW2-AS1 1.009933729 0.708687856 21.20017243 2.47E-64 4.64E-63 135.2246926

DBH-AS1 -1.765863847 2.150602519 -21.18838368 2.75E-64 5.16E-63 135.1162802

AC093157.1 -1.03998644 2.251855074 -21.17095775 3.23E-64 6.05E-63 134.9560118

MEG9 -2.284695137 1.822995664 -21.1542789 3.76E-64 7.04E-63 134.802598

AL357033.3 1.170606332 1.105282406 21.15226695 3.83E-64 7.16E-63 134.7840908

AC010913.1 -0.900616577 0.636513354 -21.14104127 4.25E-64 7.93E-63 134.6808254

AC090510.1 -0.478864575 0.232472039 -21.1195633 5.18E-64 9.65E-63 134.4832287

SRP14-AS1 -1.090677705 2.139953985 -21.09548726 6.46E-64 1.20E-62 134.2616984

PKP4-AS1 -0.718576286 0.527631193 -21.0941406 6.54E-64 1.22E-62 134.2493064

AC011444.1 0.690808092 0.42576786 21.08621655 7.04E-64 1.31E-62 134.1763872

GLYCTK-AS1 -0.728195222 0.600732837 -21.06052448 8.91E-64 1.65E-62 133.9399376

LINC01559 2.699456647 1.485129154 21.06035572 8.93E-64 1.65E-62 133.9383843

AC004585.1 1.254704543 0.707532834 21.04925129 9.89E-64 1.83E-62 133.8361762

GAPLINC 1.182234335 0.756491404 21.02093887 1.28E-63 2.37E-62 133.5755503

AC084876.1 -1.131955297 1.118978021 -21.02076261 1.29E-63 2.37E-62 133.5739275

AC092287.1 0.88772589 0.550893878 21.01573252 1.35E-63 2.48E-62 133.527619

HLA-DQB1-AS1 1.941174484 1.421386016 21.00624506 1.47E-63 2.70E-62 133.4402708

LINC01589 1.324220977 0.838655712 20.99477585 1.63E-63 3.00E-62 133.3346706

AL359220.1 -0.498774613 0.632449718 -20.99458997 1.64E-63 3.00E-62 133.3329591

LINC01503 1.841515914 2.192777762 20.99382574 1.65E-63 3.02E-62 133.3259223

AL031846.2 -0.704697574 1.141397561 -20.97923191 1.88E-63 3.45E-62 133.1915416

AC234582.1 -1.205628295 2.140503929 -20.97149736 2.02E-63 3.70E-62 133.1203167

RRS1-AS1 -0.152929858 0.118002338 -20.96945632 2.06E-63 3.76E-62 133.101521

MIRLET7BHG -1.172432707 1.53108975 -20.9424555 2.64E-63 4.82E-62 132.852851

SLC25A21-AS1 -1.236994823 1.941345839 -20.92492389 3.11E-63 5.66E-62 132.6913687

AC009690.2 -0.618789413 1.070911843 -20.91080636 3.54E-63 6.43E-62 132.561321

UBXN10-AS1 1.260433381 0.925131497 20.90812427 3.63E-63 6.58E-62 132.536613

AC091100.1 -0.279578727 0.211367709 -20.90203875 3.84E-63 6.96E-62 132.4805504

AC015917.2 -1.084735439 0.99994627 -20.8982483 3.97E-63 7.19E-62 132.44563

AL138976.2 0.861304439 1.017547619 20.85863711 5.72E-63 1.03E-61 132.0806572

AC036108.2 -0.364934066 0.225338588 -20.85780858 5.76E-63 1.04E-61 132.0730223

AL158166.1 1.150140482 0.600835232 20.85731769 5.79E-63 1.04E-61 132.0684987

AC002519.1 -0.211298119 0.165449901 -20.85401989 5.97E-63 1.08E-61 132.0381093

ITGA9-AS1 -0.862142555 1.35201545 -20.83521806 7.10E-63 1.28E-61 131.8648379

AL592430.1 -0.455824703 0.373178801 -20.83328817 7.23E-63 1.30E-61 131.8470516

AL356417.2 1.203851715 0.662145808 20.79855962 9.95E-63 1.79E-61 131.5269531

AC092118.1 -1.147633569 1.206508312 -20.79068187 1.07E-62 1.92E-61 131.454334

NCK1-DT 0.828320532 1.8608465 20.78765988 1.10E-62 1.97E-61 131.4264756

LBX2-AS1 1.299121207 2.949261917 20.76906111 1.31E-62 2.34E-61 131.2550117

AL158071.5 -0.316402455 0.359075549 -20.75614205 1.47E-62 2.63E-61 131.1358994

MIR29B2CHG -1.551438478 1.681857833 -20.75348193 1.51E-62 2.69E-61 131.1113722

HMGA2-AS1 -1.268671292 1.306419418 -20.72050725 2.04E-62 3.64E-61 130.8073062

AL606537.1 -0.235851637 0.144776756 -20.69216279 2.65E-62 4.72E-61 130.5458929

FLG-AS1 -0.900812311 0.764749992 -20.64873863 3.96E-62 7.04E-61 130.1453264

TONSL-AS1 1.373657691 1.187560894 20.64649585 4.04E-62 7.18E-61 130.1246354

AC026356.2 0.628803699 0.632302488 20.63456092 4.52E-62 8.00E-61 130.0145239

LINC01914 0.910561645 0.675909935 20.62507815 4.93E-62 8.73E-61 129.9270312

AC011450.1 -1.333083036 1.832817864 -20.60148433 6.13E-62 1.08E-60 129.7093243

AL359715.2 -0.724130391 0.78935176 -20.59832611 6.31E-62 1.11E-60 129.6801804

AC234781.1 -0.453373565 0.259195697 -20.55181681 9.69E-62 1.71E-60 129.2509409

FAM167A-AS1 -1.583692022 0.919949989 -20.54321028 1.05E-61 1.85E-60 129.171499

AC020915.2 0.735668137 1.608364033 20.53296481 1.15E-61 2.03E-60 129.0769246

AC010547.2 2.494641423 2.048376416 20.51237763 1.39E-61 2.45E-60 128.8868726

BNC2-AS1 0.981383134 0.671475465 20.4639724 2.18E-61 3.83E-60 128.4399395

AL162727.2 0.631166464 0.336179309 20.42888517 3.01E-61 5.28E-60 128.1159073

AC004707.1 -0.78525401 0.561889219 -20.42825234 3.03E-61 5.31E-60 128.1100625

DLG5-AS1 0.994423899 1.185672241 20.39686395 4.05E-61 7.09E-60 127.8201418

AC254633.1 1.642529577 1.524212178 20.38629089 4.47E-61 7.80E-60 127.7224734

ELOA-AS1 -1.002182656 1.715004781 -20.366513 5.36E-61 9.36E-60 127.5397624

LINC00930 -1.729273895 1.391195225 -20.36179712 5.60E-61 9.76E-60 127.4961939

KTN1-AS1 -0.761767873 1.361030726 -20.35168444 6.15E-61 1.07E-59 127.4027628

AC090907.1 0.670519707 0.41276823 20.34607918 6.48E-61 1.13E-59 127.3509739

CPNE8-AS1 1.009088722 1.061566057 20.31861375 8.35E-61 1.45E-59 127.0971922

AC012464.3 -0.182771929 0.152177901 -20.31854157 8.36E-61 1.45E-59 127.0965252

AC022395.1 -0.458040955 0.305487918 -20.30814324 9.20E-61 1.59E-59 127.0004358

PCOTH -0.789815147 0.859820317 -20.2924834 1.06E-60 1.84E-59 126.855717

AC092296.2 -1.015752652 0.7247885 -20.2708239 1.30E-60 2.24E-59 126.655536

AC083843.3 -1.0791091 2.001715015 -20.24123636 1.71E-60 2.94E-59 126.3820513

AC010326.3 1.31765489 2.839589574 20.23607198 1.79E-60 3.09E-59 126.334312

AC244453.2 -0.191080209 0.130913902 -20.19784493 2.55E-60 4.39E-59 125.9809093

AC104958.2 -1.648555487 2.338753664 -20.18980311 2.75E-60 4.72E-59 125.9065566

AC004923.4 1.102450179 0.840138335 20.17793561 3.07E-60 5.26E-59 125.7968279

AC017076.1 -1.512801597 1.484634048 -20.17159585 3.25E-60 5.57E-59 125.7382072

AC243772.2 -0.38711565 0.312120337 -20.15907632 3.65E-60 6.25E-59 125.6224406

ZBTB44-DT -0.672084088 0.681903022 -20.15838372 3.67E-60 6.28E-59 125.616036

AC020916.1 2.139574861 3.920778144 20.15211 3.89E-60 6.65E-59 125.5580212

AL031587.3 -0.714058242 0.34665036 -20.12824451 4.85E-60 8.28E-59 125.3373162

LINC01269 1.360987597 0.717473646 20.10068357 6.26E-60 1.07E-58 125.0824091

AC015802.4 -0.774439968 0.806615578 -20.08490731 7.25E-60 1.23E-58 124.9364837

AC025576.1 -1.20191778 0.583489148 -20.0794111 7.62E-60 1.30E-58 124.8856433

AL121928.1 -0.866620458 0.420713994 -20.05745167 9.34E-60 1.59E-58 124.6825055

ARMCX5-GPRASP2 -0.806919274 2.117998031 -20.04150283 1.08E-59 1.84E-58 124.534958

AC123912.4 -1.85771654 1.916970747 -20.04007555 1.10E-59 1.86E-58 124.5217533

LYPLAL1-AS1 -2.759912339 2.088349107 -20.01624003 1.37E-59 2.31E-58 124.3012252

AC011447.3 -0.52353262 0.475495952 -20.00960987 1.45E-59 2.46E-58 124.2398788

AC084375.1 -3.322958299 3.106018199 -20.00208901 1.56E-59 2.63E-58 124.1702891

AC009275.1 1.177288937 0.983594533 19.99127804 1.72E-59 2.91E-58 124.0702529

AL049775.1 -0.836105188 0.934947073 -19.98558597 1.82E-59 3.06E-58 124.0175812

FP671120.4 -0.548802111 0.725297301 -19.98300147 1.86E-59 3.13E-58 123.9936652

CAMTA1-DT 0.942503459 0.564725079 19.97740586 1.96E-59 3.29E-58 123.9418845

LINC02432 -1.855764524 2.421125658 -19.97629209 1.98E-59 3.32E-58 123.9315777

AC091544.2 -0.995655753 0.689330853 -19.96096232 2.28E-59 3.82E-58 123.7897128

BCDIN3D-AS1 -0.620503966 0.755423997 -19.95830038 2.34E-59 3.91E-58 123.7650778

AC245041.2 1.595028714 1.091542928 19.89850191 4.07E-59 6.80E-58 123.2116068

SMIM25 1.744293615 1.839441307 19.89799752 4.09E-59 6.82E-58 123.2069378

AC008537.2 0.922961178 0.993119147 19.89151854 4.34E-59 7.24E-58 123.1469635

AC148476.1 -2.021488578 2.548744601 -19.88888123 4.45E-59 7.41E-58 123.1225501

AC090204.1 1.70034176 1.192507888 19.88515342 4.60E-59 7.66E-58 123.0880417

CARD8-AS1 0.980273819 1.632575273 19.88197193 4.74E-59 7.88E-58 123.0585903

AC080037.2 1.376596058 1.04155406 19.87195792 5.20E-59 8.63E-58 122.9658872

LINC02204 -0.155464667 0.134867374 -19.86639781 5.48E-59 9.08E-58 122.9144139

AC005519.1 -0.966023683 1.609001843 -19.83301163 7.46E-59 1.24E-57 122.6053165

STARD13-AS -0.586751622 0.368333372 -19.82810815 7.81E-59 1.29E-57 122.5599158

AF131216.1 -0.214562747 0.229861383 -19.82629159 7.94E-59 1.31E-57 122.5430963

AC211433.1 -0.370662983 0.318658577 -19.76151674 1.45E-58 2.39E-57 121.9432795

AL139275.2 -0.901538564 0.593002856 -19.74941536 1.62E-58 2.67E-57 121.8312059

AC080013.4 1.158155788 1.430166199 19.72279845 2.07E-58 3.41E-57 121.5846852

AC007938.2 -0.279119022 0.22580215 -19.7226191 2.07E-58 3.41E-57 121.5830241

AP000346.1 -0.609834048 0.476037741 -19.68642088 2.90E-58 4.76E-57 121.247729

AL121929.2 -1.728480437 2.150704238 -19.67781976 3.14E-58 5.15E-57 121.1680534

AC243562.3 -0.394040009 0.249127466 -19.67303371 3.28E-58 5.38E-57 121.1237173

AL122010.1 0.818238497 1.943693164 19.64317853 4.33E-58 7.09E-57 120.8471362

AC024958.1 -0.39565569 0.201263632 -19.63250298 4.78E-58 7.81E-57 120.7482308

AL133371.2 1.552119796 0.865216949 19.62805637 4.98E-58 8.13E-57 120.7070335

AC087645.2 0.555885717 0.347691135 19.61942393 5.39E-58 8.80E-57 120.6270535

AC008663.2 -0.35017919 0.367245531 -19.61411867 5.67E-58 9.23E-57 120.5778989

AC011445.1 -0.653995853 0.532539234 -19.6128138 5.74E-58 9.33E-57 120.5658088

LINC02657 1.635226594 0.872387469 19.61063549 5.85E-58 9.51E-57 120.545626

AP003555.2 1.448698575 0.807697374 19.60233005 6.32E-58 1.03E-56 120.4686717

NADK2-AS1 -0.727367282 0.77106688 -19.601836 6.35E-58 1.03E-56 120.464094

AC124312.3 -0.953014083 0.995589982 -19.58328786 7.54E-58 1.22E-56 120.2922286

AL596087.2 0.798853438 0.42830631 19.58265035 7.59E-58 1.23E-56 120.2863214

LINC02014 0.98945458 0.548526746 19.57925476 7.83E-58 1.27E-56 120.2548571

AC106017.2 -0.385783629 0.187284487 -19.5694479 8.57E-58 1.38E-56 120.1639829

SYNPR-AS1 1.430145382 0.817523101 19.56925436 8.59E-58 1.38E-56 120.1621894

AC009831.1 0.513885434 0.719297084 19.56811564 8.68E-58 1.40E-56 120.1516374

LINC00519 0.897403246 0.51810048 19.56226674 9.16E-58 1.47E-56 120.0974378

FGF12-AS2 -0.715742461 0.528614421 -19.55511072 9.79E-58 1.57E-56 120.0311244

TRIM31-AS1 1.434313471 0.839843469 19.54281849 1.10E-57 1.76E-56 119.9172115

MAP3K2-DT 0.95293543 0.833986401 19.542468 1.10E-57 1.76E-56 119.9139635

AC022211.3 1.167019887 0.797750809 19.53502476 1.18E-57 1.89E-56 119.8449845

AP000787.1 -0.506261097 0.795809785 -19.52579263 1.28E-57 2.06E-56 119.7594255

AC091982.1 0.540520087 0.30830362 19.52375845 1.31E-57 2.09E-56 119.7405733

AC129507.1 -1.516427222 1.753294734 -19.51386752 1.44E-57 2.29E-56 119.6489058

LINC02313 1.206150969 0.673430881 19.46700272 2.22E-57 3.53E-56 119.2145377

OBSCN-AS1 -0.727370191 0.815733521 -19.46466331 2.26E-57 3.61E-56 119.1928534

AC046143.1 0.785472525 1.172744328 19.46106492 2.34E-57 3.72E-56 119.1594991

AL139288.1 1.104640039 0.612659774 19.45699218 2.43E-57 3.86E-56 119.1217477

AC245041.1 1.908847991 1.0928801 19.44297222 2.77E-57 4.39E-56 118.9917895

AL359258.2 -0.448239187 0.217604482 -19.44118639 2.82E-57 4.46E-56 118.9752354

AL603756.1 -0.390085421 0.449650937 -19.43678693 2.93E-57 4.64E-56 118.9344534

AC018521.5 -0.973693647 1.563519738 -19.43608766 2.95E-57 4.67E-56 118.9279713

AC127459.1 -0.71712677 0.348140023 -19.43589569 2.96E-57 4.67E-56 118.9261917

LINC01278 -0.935840008 3.610475572 -19.39060711 4.50E-57 7.10E-56 118.5063499

AP001062.1 -0.802351836 1.629847263 -19.38089493 4.92E-57 7.76E-56 118.4163085

LINC01638 1.011171346 0.69776526 19.37033693 5.43E-57 8.55E-56 118.3184231

AC108488.1 -0.882943132 2.272037024 -19.3637054 5.77E-57 9.08E-56 118.2569394

ALG1L9P 0.383783598 0.390319303 19.36166231 5.89E-57 9.25E-56 118.237997

AC026150.3 -0.685936811 0.591796732 -19.35346524 6.35E-57 9.96E-56 118.161997

RUSC1-AS1 -1.324959712 2.749917241 -19.3137191 9.18E-57 1.44E-55 117.7934664

TBILA -1.574079553 2.589438008 -19.30432655 1.00E-56 1.57E-55 117.7063729

AC108010.1 -1.279439405 2.455438222 -19.28922185 1.15E-56 1.80E-55 117.566309

INE2 -0.52287972 0.253839856 -19.27427138 1.32E-56 2.07E-55 117.4276709

AC022424.1 -0.855658608 0.583752766 -19.26359653 1.46E-56 2.28E-55 117.3286785

AL158835.1 -0.408923538 0.26273071 -19.25102517 1.64E-56 2.56E-55 117.2120962

TGFB2-AS1 1.601860377 0.934949284 19.24640775 1.71E-56 2.67E-55 117.1692752

AC004816.1 1.455507267 1.62273838 19.24456547 1.74E-56 2.71E-55 117.1521902

AC008735.2 -1.565641417 3.465495686 -19.24218049 1.78E-56 2.77E-55 117.1300721

AL137058.2 -0.656869269 1.082970949 -19.23526527 1.90E-56 2.95E-55 117.0659403

LINC00524 1.523703197 0.881721843 19.22731144 2.05E-56 3.17E-55 116.9921755

AC115989.1 -0.558712861 0.409375586 -19.22414936 2.11E-56 3.26E-55 116.9628497

AC074117.1 -0.89818327 2.2347616 -19.19409082 2.78E-56 4.31E-55 116.6840707

AP4B1-AS1 -0.88870844 1.277055852 -19.19020663 2.89E-56 4.46E-55 116.6480456

VLDLR-AS1 -0.983524266 0.787709622 -19.17389063 3.36E-56 5.18E-55 116.4967144

AP001363.2 0.722488232 0.448443982 19.17106119 3.45E-56 5.32E-55 116.4704709

AL162231.4 -0.235045381 0.252514292 -19.16309558 3.71E-56 5.71E-55 116.3965877

AP001767.3 0.766383348 0.408023382 19.16309014 3.71E-56 5.71E-55 116.3965373

AC138028.1 -0.15803904 0.13224934 -19.16269725 3.73E-56 5.73E-55 116.3928931

AL844908.1 1.269726097 0.93439785 19.15191241 4.12E-56 6.32E-55 116.292859

LINC02827 -0.694591253 0.566611418 -19.14224891 4.51E-56 6.91E-55 116.2032243

AL589743.1 -0.174486975 0.085006416 -19.13312187 4.90E-56 7.51E-55 116.1185641

WEE2-AS1 -0.894453838 1.273563567 -19.11985261 5.55E-56 8.49E-55 115.9954793

AC009318.2 0.694356801 1.376183225 19.11455716 5.82E-56 8.90E-55 115.9463582

AD000671.3 -0.332672288 0.27303781 -19.11143184 6.00E-56 9.15E-55 115.9173673

AP003096.1 -0.728179327 0.814738734 -19.10581627 6.32E-56 9.63E-55 115.8652761

SH3PXD2A-AS1 1.890248177 1.076114418 19.09963433 6.69E-56 1.02E-54 115.8079305

AC004461.2 -0.564933159 0.550934539 -19.09873892 6.75E-56 1.03E-54 115.7996244

AC010542.4 -0.213121677 0.161623986 -19.09629276 6.90E-56 1.05E-54 115.7769329

AC108449.3 -1.473819439 0.715487918 -19.0825882 7.84E-56 1.19E-54 115.6498024

GHRLOS -0.693739555 1.032967799 -19.07169968 8.67E-56 1.32E-54 115.5487929

AC004921.1 0.725319145 0.507284482 19.07131203 8.70E-56 1.32E-54 115.5451968

LINC02724 -0.664295977 0.637488387 -19.05379652 1.02E-55 1.55E-54 115.382707

LINC02166 0.867362335 0.807935301 19.05244094 1.04E-55 1.57E-54 115.3701313

AP001922.6 0.703398369 0.626564987 19.05084025 1.05E-55 1.59E-54 115.3552816

LINC01711 1.605803453 0.944310843 19.04051295 1.16E-55 1.75E-54 115.2594738

AC245100.6 1.229029496 1.01494733 19.03894988 1.17E-55 1.77E-54 115.2449728

AC000403.1 0.54324951 0.527438079 19.03804119 1.18E-55 1.78E-54 115.2365427

AL662797.1 -0.921346987 1.184644956 -19.00128769 1.67E-55 2.51E-54 114.8955621

AC010336.1 -0.178554718 0.116881097 -18.99674436 1.74E-55 2.61E-54 114.8534102

AC007952.1 -0.226775021 0.110377839 -18.99524547 1.76E-55 2.65E-54 114.8395038

HEXD-IT1 -0.450943001 0.513615003 -18.98667631 1.91E-55 2.86E-54 114.7600002

LCMT1-AS2 -0.2577395 0.164450172 -18.98323278 1.97E-55 2.95E-54 114.7280513

AL603839.2 0.610802794 0.479387819 18.98068845 2.02E-55 3.02E-54 114.7044451

AC123912.2 -0.343035873 0.183766981 -18.97167744 2.19E-55 3.28E-54 114.6208404

AC051619.6 -0.591966812 0.404103198 -18.96946801 2.24E-55 3.34E-54 114.600341

UBA6-AS1 -0.809234988 2.321342222 -18.96208109 2.40E-55 3.58E-54 114.5318039

AC087521.3 0.273385463 0.15154601 18.95549428 2.55E-55 3.80E-54 114.4706897

AC020931.1 -0.551293066 0.649137081 -18.94201051 2.89E-55 4.30E-54 114.3455822

AC011481.1 0.882573247 0.608993143 18.9393237 2.96E-55 4.40E-54 114.3206527

AC003070.1 1.747589573 1.924127957 18.93877168 2.98E-55 4.42E-54 114.3155308

FGD5-AS1 1.050655582 5.261302689 18.93165616 3.18E-55 4.72E-54 114.2495092

MIR155HG 0.95610238 0.881576551 18.92535186 3.37E-55 5.00E-54 114.191014

LINC01219 -0.537793716 0.332717625 -18.92167086 3.49E-55 5.16E-54 114.1568593

USP30-AS1 1.235527466 1.249367186 18.92083913 3.52E-55 5.20E-54 114.1491419

AC021218.1 2.023023031 1.129991586 18.90062319 4.24E-55 6.27E-54 113.9615623

AC078909.1 -0.779756436 0.510584318 -18.88682618 4.82E-55 7.11E-54 113.8335401

SNHG8 -1.755811215 7.421717829 -18.84870116 6.87E-55 1.01E-53 113.4797693

NR2F2-AS1 -0.800826352 1.070229133 -18.83101259 8.09E-55 1.19E-53 113.3156285

LINC01116 1.232502406 1.256349779 18.82398721 8.64E-55 1.27E-53 113.2504359

AC114488.1 1.670954491 1.328204029 18.81894035 9.05E-55 1.33E-53 113.2036028

RNASEH1-AS1 0.885983606 2.407827668 18.78317916 1.26E-54 1.85E-53 112.8717459

AC007952.2 -0.365692174 0.250170177 -18.78239186 1.27E-54 1.86E-53 112.8644398

AC009630.2 -0.362710232 0.193757669 -18.77351887 1.38E-54 2.02E-53 112.7820986

CFAP58-DT 0.610367366 0.507798121 18.77180043 1.40E-54 2.05E-53 112.7661514

SZT2-AS1 -0.729316239 0.517556623 -18.76828934 1.45E-54 2.12E-53 112.7335684

AL390208.1 -1.003083176 1.133793502 -18.75358603 1.66E-54 2.43E-53 112.5971203

STIM2-AS1 -1.503034492 1.654804843 -18.7306479 2.06E-54 3.00E-53 112.3842494

AC012414.5 -0.420269415 0.308553406 -18.73002015 2.07E-54 3.01E-53 112.3784238

AL157871.1 -1.696103764 0.823399203 -18.70899537 2.51E-54 3.66E-53 112.1833063

MCCC1-AS1 -1.047818782 1.5210132 -18.69035024 2.99E-54 4.35E-53 112.010271

H19 -2.763735609 5.714007826 -18.68055572 3.27E-54 4.75E-53 111.9193726

AC079061.1 -1.126450793 0.719365881 -18.66802579 3.68E-54 5.33E-53 111.8030876

LINC02678 1.132103593 0.596050309 18.64221496 4.67E-54 6.77E-53 111.563546

AC025048.4 0.754166373 0.689686041 18.62218327 5.63E-54 8.15E-53 111.377637

AC092069.1 -1.594328973 0.773991093 -18.60953416 6.33E-54 9.15E-53 111.2602432

AC009560.1 -0.866802048 0.853186912 -18.60154197 6.81E-54 9.85E-53 111.1860691

BX324167.2 -0.145260607 0.106965207 -18.59713374 7.10E-54 1.03E-52 111.1451571

AC079921.1 0.368889177 0.238153172 18.56045695 9.98E-54 1.44E-52 110.804764

FAM245A -0.807085887 0.61500689 -18.55791664 1.02E-53 1.47E-52 110.7811876

AC079328.2 -2.258621297 1.096481843 -18.55004855 1.10E-53 1.58E-52 110.7081645

GABPB1-AS1 -1.052194871 2.001545415 -18.53102864 1.31E-53 1.89E-52 110.5316418

AC005332.4 0.851605848 1.175091686 18.52410484 1.40E-53 2.01E-52 110.4673824

AC009318.3 0.665137816 0.839094489 18.4980993 1.78E-53 2.56E-52 110.2260262

AL358115.1 -0.694483786 0.765648545 -18.4897261 1.92E-53 2.76E-52 110.148315

AL136304.1 -1.029474142 1.495084894 -18.48788224 1.96E-53 2.80E-52 110.1312022

PITPNA-AS1 -1.256122138 3.718256809 -18.48682712 1.98E-53 2.83E-52 110.1214097

AL022316.1 1.098620312 0.855950931 18.48305156 2.05E-53 2.93E-52 110.086369

LINC00222 -0.344379639 0.266570702 -18.48234228 2.06E-53 2.94E-52 110.0797861

AL352979.2 -1.164067323 0.565114071 -18.48057661 2.10E-53 2.99E-52 110.0633991

AL109741.1 0.928660943 0.698925106 18.46860155 2.34E-53 3.34E-52 109.9522592

XXYLT1-AS2 1.611271866 0.901007784 18.46583586 2.40E-53 3.42E-52 109.926591

AC092681.2 -0.168848695 0.155814945 -18.46164814 2.50E-53 3.55E-52 109.8877252

AL137847.1 0.714041544 0.554075459 18.45865636 2.57E-53 3.65E-52 109.8599587

FAM201A -1.0444641 1.913006126 -18.45737488 2.60E-53 3.69E-52 109.8480654

AC021097.1 -1.381667409 0.670751322 -18.41763951 3.76E-53 5.33E-52 109.479286

CASC8 1.383972383 0.819506782 18.41554583 3.83E-53 5.43E-52 109.4598549

AC011468.1 1.028278405 1.713710326 18.38635202 5.03E-53 7.11E-52 109.1889131

AP002026.1 -0.617638419 0.788586652 -18.38278124 5.20E-53 7.34E-52 109.1557736

AL450326.1 0.801043609 1.37114664 18.37184189 5.75E-53 8.12E-52 109.0542486

LINC02177 -0.341413978 0.213338726 -18.37092792 5.80E-53 8.18E-52 109.0457663

LINC02773 0.487258418 0.297481327 18.36360363 6.21E-53 8.75E-52 108.9777919

AC023043.4 1.037278135 0.991730355 18.33337082 8.22E-53 1.16E-51 108.6972131

AC008610.1 1.333584701 1.623518754 18.33275039 8.27E-53 1.16E-51 108.6914552

AC138207.2 1.038360138 0.873937615 18.32785698 8.65E-53 1.22E-51 108.6460418

AC139720.1 0.873529446 0.479314581 18.32444907 8.93E-53 1.25E-51 108.6144146

AP002761.4 1.317727014 1.112184486 18.3161292 9.65E-53 1.35E-51 108.5372024

AL137026.1 -0.987860425 0.704525981 -18.31394347 9.84E-53 1.38E-51 108.5169178

AC025257.1 -1.607134376 0.780207669 -18.29589584 1.16E-52 1.63E-51 108.3494288

LINC02595 0.777084592 0.414749028 18.28883547 1.24E-52 1.74E-51 108.2839064

LINC02747 1.477014393 0.795382325 18.28051099 1.34E-52 1.88E-51 108.2066529

LINC01725 -0.504179624 0.368315687 -18.2780597 1.37E-52 1.92E-51 108.1839044

AC064836.3 0.924271702 1.713944755 18.27350503 1.43E-52 2.00E-51 108.1416361

AC007349.1 -1.445654883 1.25969892 -18.25577022 1.69E-52 2.35E-51 107.9770546

AC108673.2 0.85663103 0.984545983 18.23832066 1.99E-52 2.76E-51 107.8151224

AC004263.1 -0.570130638 0.558175051 -18.22523314 2.24E-52 3.12E-51 107.6936714

AC007349.3 -0.764915334 0.522656285 -18.21174937 2.54E-52 3.53E-51 107.5685448

AL512598.1 1.227922385 1.129800959 18.20754685 2.64E-52 3.67E-51 107.5295465

LINC01426 1.017535805 1.111302228 18.20077476 2.82E-52 3.90E-51 107.4667037

AC084809.1 0.715242077 0.682123881 18.19849346 2.88E-52 3.98E-51 107.445534

LINC01615 1.292859481 0.756862938 18.19310128 3.02E-52 4.18E-51 107.3954967

AC005104.1 -1.117573155 2.187304985 -18.19082311 3.09E-52 4.27E-51 107.3743562

BCRP3 -1.146915522 1.209859847 -18.15581439 4.27E-52 5.90E-51 107.0494961

Z98259.1 -1.041063782 0.644540072 -18.15543961 4.29E-52 5.91E-51 107.0460185

RASAL2-AS1 0.610158586 0.843847331 18.15429898 4.33E-52 5.97E-51 107.0354343

AL355073.2 -0.93280011 1.190909571 -18.13469898 5.20E-52 7.16E-51 106.8535634

AC126773.6 -0.184530351 0.165873661 -18.11957583 5.98E-52 8.23E-51 106.7132365

LINC00346 1.033234554 0.866197816 18.11709245 6.12E-52 8.41E-51 106.6901936

LINC01871 1.660072097 1.375543812 18.11320235 6.35E-52 8.71E-51 106.6540981

AC021087.3 -0.399281392 0.56203242 -18.10952522 6.57E-52 9.01E-51 106.6199788

AC012313.1 -0.838076379 2.394766275 -18.09689601 7.39E-52 1.01E-50 106.5027964

AC079193.2 -0.163230063 0.143796747 -18.09342292 7.63E-52 1.04E-50 106.470571

AC126763.1 -0.221473971 0.113656153 -18.08114002 8.55E-52 1.17E-50 106.3566042

LINC00578 1.243927642 0.850399815 18.0787527 8.74E-52 1.19E-50 106.3344537

AC012615.6 -0.850033017 1.525631956 -18.0683029 9.63E-52 1.31E-50 106.2374969

AP001120.2 -1.781148333 0.913817361 -18.0485229 1.16E-51 1.58E-50 106.0539752

LAMC1-AS1 0.650658128 0.520751825 18.02690981 1.41E-51 1.93E-50 105.8534516

AL357146.1 -0.563869256 0.326623962 -18.02048104 1.50E-51 2.04E-50 105.7938075

AC005229.4 0.636794145 1.565647587 18.01416791 1.59E-51 2.16E-50 105.7352367

AC130456.3 1.298474493 0.884135373 17.99700026 1.87E-51 2.53E-50 105.575965

AC007952.5 -0.215257665 0.105088086 -17.97924267 2.20E-51 2.99E-50 105.4112246

AC017083.2 -1.229987224 0.597115883 -17.96840577 2.43E-51 3.30E-50 105.3106909

AC012313.3 -0.31994682 0.402477731 -17.96693906 2.47E-51 3.34E-50 105.2970844

UCA1 2.756932881 1.99456113 17.94467732 3.03E-51 4.10E-50 105.0905691

CRIM1-DT 1.200773392 2.365979134 17.94336248 3.07E-51 4.15E-50 105.078372

AL445426.1 0.778368879 0.465034726 17.94258952 3.09E-51 4.18E-50 105.0712016

AC021205.3 -0.43865096 0.599877185 -17.92838771 3.53E-51 4.76E-50 104.9394604

AF131215.7 -0.818339574 1.045084904 -17.92500541 3.64E-51 4.91E-50 104.9080854

AL391056.1 1.264704864 0.782899123 17.91800183 3.89E-51 5.23E-50 104.8431192

HAND2-AS1 -1.263403408 2.001164953 -17.90400348 4.43E-51 5.95E-50 104.713271

AC006994.2 -0.554517402 0.377460069 -17.8854429 5.26E-51 7.06E-50 104.541109

AC005920.4 -1.186140564 0.910673366 -17.87844985 5.61E-51 7.53E-50 104.4762453

AP003469.2 0.852874242 0.639549724 17.87563734 5.76E-51 7.72E-50 104.4501582

AC005726.2 -0.388609439 0.305708334 -17.85919136 6.71E-51 8.99E-50 104.2976186

AL024508.1 0.707516846 0.434494292 17.84487396 7.66E-51 1.03E-49 104.1648259

ENTPD3-AS1 -0.822307833 1.942718561 -17.84186997 7.88E-51 1.05E-49 104.1369648

AL049830.3 -1.370296912 0.726289992 -17.84084503 7.96E-51 1.06E-49 104.1274587

STXBP5-AS1 -0.897288957 1.053408251 -17.83379647 8.49E-51 1.13E-49 104.062086

HIPK1-AS1 -0.460413741 0.375062612 -17.82293264 9.39E-51 1.25E-49 103.9613298

AC105942.1 1.10481547 2.551719342 17.82284708 9.40E-51 1.25E-49 103.9605363

AC009962.1 -0.747325115 0.857912379 -17.7927397 1.24E-50 1.65E-49 103.681319

AC002064.2 0.751636908 0.411975352 17.78933104 1.28E-50 1.71E-49 103.6497081

AL583722.1 0.770304414 0.462385958 17.77202747 1.51E-50 2.00E-49 103.4892436

AL450998.2 0.766543827 1.877764626 17.76181124 1.66E-50 2.20E-49 103.3945064

SNHG29 -1.557235205 7.784780933 -17.7501538 1.85E-50 2.45E-49 103.2864073

AC087276.1 0.992752334 0.640366544 17.73573623 2.11E-50 2.79E-49 103.1527178

AC099518.1 0.581580845 0.359890504 17.730945 2.21E-50 2.92E-49 103.1082912

AC027601.4 -1.11089135 0.539298991 -17.72331779 2.37E-50 3.13E-49 103.0375692

RFPL3S -1.219970607 0.971662131 -17.72017071 2.44E-50 3.22E-49 103.0083888

LINC02188 1.753712106 0.980380892 17.71658858 2.52E-50 3.33E-49 102.9751748

AC105285.1 -0.725360341 0.942108897 -17.70872963 2.71E-50 3.57E-49 102.9023067

LINC01422 -0.2184175 0.290593533 -17.69762652 3.01E-50 3.96E-49 102.7993611

LINC02195 0.845985626 0.453641237 17.69753243 3.01E-50 3.96E-49 102.7984887

DCST1-AS1 1.010549104 1.286065374 17.69611031 3.05E-50 4.01E-49 102.7853033

CARMN -0.621589232 0.660942772 -17.6956181 3.06E-50 4.02E-49 102.7807397

AC105020.4 -0.744354337 0.361358057 -17.69037567 3.21E-50 4.22E-49 102.7321343

AC100835.2 -0.708431939 0.343918986 -17.67886746 3.58E-50 4.69E-49 102.6254378

AC138356.1 -0.496166269 0.508765621 -17.67575089 3.68E-50 4.82E-49 102.5965435

AC025165.1 -0.501506745 0.733190248 -17.67084953 3.85E-50 5.04E-49 102.5511027

AC010185.1 -0.92133311 0.570553864 -17.66440156 4.09E-50 5.35E-49 102.4913239

SND1-IT1 -0.157007792 0.105371744 -17.6636443 4.12E-50 5.38E-49 102.4843035

AC060766.7 0.704958297 1.198692703 17.66261923 4.16E-50 5.43E-49 102.4748002

AC073476.3 -0.175020171 0.153100181 -17.64819118 4.75E-50 6.20E-49 102.3410431

AC007383.3 -0.621504102 0.301718553 -17.64647944 4.83E-50 6.29E-49 102.3251746

MINCR -1.180380329 3.108483041 -17.63649134 5.30E-50 6.90E-49 102.232582

AC116914.2 1.086571505 1.122440775 17.61720845 6.34E-50 8.24E-49 102.0538313

BX640514.2 1.241269876 0.758595104 17.61446041 6.50E-50 8.44E-49 102.028358

CYP4A22-AS1 0.622115665 0.393351769 17.6051868 7.08E-50 9.19E-49 101.9423965

AP003064.1 -1.275687152 0.619301604 -17.58779618 8.32E-50 1.08E-48 101.7812008

LINC01094 1.006088439 1.003300101 17.58763988 8.34E-50 1.08E-48 101.7797521

AL136295.2 -0.79194325 1.409387143 -17.57419316 9.44E-50 1.22E-48 101.6551184

AC091045.1 -0.458503368 0.423211167 -17.57166086 9.67E-50 1.25E-48 101.6316478

SNHG17 -1.320852156 4.107854343 -17.5673667 1.01E-49 1.30E-48 101.5918479

AL445309.1 -0.454931891 0.588487353 -17.56313069 1.05E-49 1.35E-48 101.5525874

AC022916.1 0.531654729 0.875878428 17.56114282 1.07E-49 1.37E-48 101.5341634

LINC02246 -0.441686894 0.318101595 -17.5494842 1.19E-49 1.53E-48 101.4261114

AC025265.1 -0.956221096 1.236961531 -17.54269373 1.26E-49 1.63E-48 101.3631791

AC023906.4 -0.508730519 0.354061578 -17.5413019 1.28E-49 1.65E-48 101.3502801

ITGB1-DT 1.186497483 0.87408536 17.53340456 1.38E-49 1.77E-48 101.2770916

Z97832.2 -0.461777338 0.784359513 -17.53147831 1.40E-49 1.80E-48 101.2592403

AC005261.1 0.920212527 3.431612761 17.52915464 1.43E-49 1.84E-48 101.2377062

AC127164.1 -0.386118315 0.4242702 -17.51436407 1.65E-49 2.11E-48 101.1006414

AL512274.1 1.419034418 1.613950065 17.49617272 1.95E-49 2.49E-48 100.9320701

OGFR-AS1 0.569243359 0.409633821 17.48743315 2.11E-49 2.70E-48 100.8510878

GAS5-AS1 0.608732787 1.398795093 17.48704937 2.12E-49 2.71E-48 100.8475317

LINC02097 -0.293371413 0.285896761 -17.48485358 2.16E-49 2.76E-48 100.8271855

AC007566.1 -0.976029197 2.062160949 -17.47573999 2.35E-49 3.00E-48 100.7427407

AL353194.1 0.976073384 1.224744835 17.46215364 2.67E-49 3.40E-48 100.6168567

LINC002481 0.655291004 0.621873547 17.45523419 2.85E-49 3.62E-48 100.552747

LINP1 1.587851063 0.863986427 17.44422361 3.15E-49 4.01E-48 100.4507353

LINC01150 0.865393669 0.583551083 17.43867451 3.32E-49 4.22E-48 100.3993251

C1RL-AS1 -1.105348669 2.038253189 -17.43333191 3.49E-49 4.43E-48 100.3498289

SBNO1-AS1 -0.715967442 1.089383631 -17.42383654 3.81E-49 4.83E-48 100.2618618

AC103702.2 1.982838435 1.04814878 17.41559734 4.11E-49 5.21E-48 100.1855344

AL158211.1 0.590852273 0.577741197 17.41097779 4.29E-49 5.43E-48 100.1427403

SAMD12-AS1 0.708708823 0.562235877 17.4105184 4.31E-49 5.45E-48 100.1384847

AL441992.1 1.003778876 2.267277633 17.40887673 4.37E-49 5.53E-48 100.1232768

AC125257.1 0.775279152 2.771694538 17.40601472 4.49E-49 5.67E-48 100.0967645

FAM66C -0.707191385 1.091553091 -17.39857075 4.81E-49 6.07E-48 100.0278084

AL590764.1 0.963451174 0.676577501 17.38090126 5.67E-49 7.15E-48 99.86413694

MED14OS 0.587095063 0.825318433 17.37616123 5.93E-49 7.46E-48 99.82023207

AC100786.2 -1.194993037 0.604447592 -17.3745528 6.01E-49 7.57E-48 99.80533404

AC078777.1 0.308844498 0.17396662 17.37412438 6.04E-49 7.59E-48 99.80136581

AC106900.1 1.539473543 0.793008576 17.36493003 6.58E-49 8.26E-48 99.71620536

AC005330.1 0.511594108 0.312507307 17.36177535 6.77E-49 8.50E-48 99.68698649

AC002398.1 1.193515458 1.753769803 17.35765186 7.03E-49 8.82E-48 99.64879504

MYCBP2-AS1 -0.617822239 0.404430779 -17.35663471 7.10E-49 8.89E-48 99.63937433

LINC01484 -0.80781504 0.734554622 -17.35594165 7.15E-49 8.94E-48 99.6329553

AC090510.2 -0.599631959 0.834331508 -17.31574126 1.04E-48 1.30E-47 99.26065528

AC051619.5 -0.825362506 0.899104768 -17.3150975 1.04E-48 1.30E-47 99.25469379

CU639417.5 -1.202016857 0.60405125 -17.31321733 1.06E-48 1.33E-47 99.23728281

COL18A1-AS1 -0.202320663 0.142451088 -17.30844625 1.11E-48 1.38E-47 99.1931015

AL049836.1 1.431062645 0.82362684 17.29323237 1.28E-48 1.59E-47 99.05222308

AC130352.1 -0.485271804 0.336937843 -17.29005042 1.32E-48 1.64E-47 99.02275967

AC004594.1 -0.596952325 0.432412586 -17.28692305 1.35E-48 1.69E-47 98.99380207

AL049646.1 -0.155445617 0.090882817 -17.27859476 1.46E-48 1.82E-47 98.91668873

AC087071.2 0.374271991 0.204855048 17.26747027 1.62E-48 2.01E-47 98.81368878

AC005339.1 -0.977926637 0.474749261 -17.26612515 1.64E-48 2.04E-47 98.80123481

AC098850.1 -0.686640116 0.383113634 -17.25730779 1.78E-48 2.21E-47 98.71959991

LINC01504 0.8869828 0.815852163 17.25293683 1.86E-48 2.30E-47 98.67913276

C5orf66 -0.679054455 0.80427474 -17.22904439 2.32E-48 2.87E-47 98.45794502

AC022034.1 1.112647941 0.914850197 17.22036134 2.51E-48 3.10E-47 98.37756585

AC098851.1 0.671645036 0.538509976 17.21755323 2.58E-48 3.18E-47 98.35157171

AC027601.1 0.965604259 0.655063682 17.21405214 2.66E-48 3.28E-47 98.31916329

AL357992.1 0.93595444 0.482476944 17.19188057 3.27E-48 4.03E-47 98.11393933

AC009950.1 -0.711856767 0.845205752 -17.18048515 3.63E-48 4.47E-47 98.00846888

AL158152.1 -1.00842385 0.79398159 -17.17995683 3.65E-48 4.49E-47 98.00357916

LINC01857 1.587379989 0.898233127 17.17592294 3.79E-48 4.66E-47 97.96624479

AC005697.2 -0.202341015 0.124430473 -17.14947282 4.84E-48 5.95E-47 97.72146034

CD27-AS1 -1.040962474 4.159788115 -17.14831331 4.89E-48 6.01E-47 97.71073024

AC097532.2 -0.224995991 0.193331973 -17.14741182 4.93E-48 6.05E-47 97.70238794

AC009053.2 -0.234831865 0.263956433 -17.13391727 5.59E-48 6.85E-47 97.57751392

AC124248.1 1.043874816 0.676131719 17.13266495 5.66E-48 6.93E-47 97.56592573

HOXB-AS1 0.996122477 1.309877205 17.12242105 6.22E-48 7.61E-47 97.4711378

KCNK4-TEX40 -0.38243065 0.199570936 -17.10290811 7.45E-48 9.11E-47 97.29059448

AF186192.1 -0.789119112 0.945651883 -17.0945038 8.05E-48 9.84E-47 97.21283866

AL135818.2 0.504455425 0.355258317 17.09090713 8.33E-48 1.02E-46 97.17956362

MIR181A2HG 0.908456923 0.86396957 17.08254077 9.00E-48 1.10E-46 97.10216318

AC120498.8 0.877665488 0.550020995 17.07476274 9.67E-48 1.18E-46 97.03020843

POLR2J4 -0.716706106 2.085520426 -17.07106718 1.00E-47 1.22E-46 96.9960216

AL359232.1 -0.976555198 0.81150005 -17.04434141 1.28E-47 1.56E-46 96.74880523

AC002091.1 0.763983017 0.52790091 17.04420497 1.28E-47 1.56E-46 96.74754321

Z98885.3 -0.309140628 0.389002321 -17.0359083 1.39E-47 1.68E-46 96.67080458

NAMA -0.26367536 0.163213752 -17.01773717 1.64E-47 1.99E-46 96.5027447

AC023449.2 0.50490614 0.425967477 16.99129841 2.09E-47 2.54E-46 96.25824663

CYP1B1-AS1 -0.486312702 0.555804315 -16.99009384 2.12E-47 2.56E-46 96.24710785

AC087392.3 -0.354717729 0.477218854 -16.9866266 2.19E-47 2.65E-46 96.2150464

AC013652.1 -1.212336172 1.048248071 -16.98114722 2.30E-47 2.78E-46 96.16437993

MAGI1-IT1 -0.233211186 0.248221433 -16.98088525 2.31E-47 2.78E-46 96.16195757

Z82217.1 -0.111480702 0.102348967 -16.97831152 2.36E-47 2.85E-46 96.13815944

AC009019.1 -0.606919904 0.49604075 -16.97729859 2.38E-47 2.87E-46 96.12879345

AGAP2-AS1 1.083361502 3.967971815 16.97220603 2.50E-47 3.01E-46 96.08170593

AC145207.6 -0.584767117 0.283884029 -16.96933396 2.57E-47 3.09E-46 96.0551504

AC084117.1 1.193136724 0.837859242 16.95478042 2.94E-47 3.53E-46 95.92059228

RNF185-AS1 -1.39439577 0.676930497 -16.95294638 2.99E-47 3.59E-46 95.90363591

LINC02449 -1.135134656 1.556528208 -16.94530671 3.21E-47 3.85E-46 95.83300634

AC090515.4 1.520110032 2.219051399 16.93065159 3.67E-47 4.40E-46 95.69752596

UNC5B-AS1 1.918392744 1.423625723 16.91461423 4.26E-47 5.10E-46 95.54927924

AC245884.9 -1.120556759 1.091742025 -16.91263359 4.34E-47 5.19E-46 95.53097148

AC005070.3 -0.380677161 0.734146015 -16.91256769 4.34E-47 5.19E-46 95.53036233

LINC00853 0.960582353 0.995979271 16.91223546 4.35E-47 5.20E-46 95.52729137

AC104109.4 -0.357529944 0.409234917 -16.90132992 4.82E-47 5.75E-46 95.42649095

AC010618.2 0.649587793 0.491739372 16.89922355 4.91E-47 5.86E-46 95.40702227

AC007952.7 -0.352875372 0.174386534 -16.89635873 5.04E-47 6.01E-46 95.38054388

AC099811.3 0.421747337 0.244287505 16.88682368 5.51E-47 6.56E-46 95.29241816

AC103810.2 -1.02150905 0.753814681 -16.86962239 6.46E-47 7.68E-46 95.13345021

FAM138B -0.378756047 0.20334273 -16.86535152 6.72E-47 7.99E-46 95.09398274

AC093423.2 0.343309288 0.190509317 16.86218662 6.92E-47 8.22E-46 95.0647362

AC106795.2 -2.115993397 2.362284638 -16.85895411 7.13E-47 8.46E-46 95.03486539

LINC01252 -0.759683702 0.821443166 -16.8427497 8.28E-47 9.82E-46 94.88513283

AC068580.1 1.547989517 1.377027402 16.83707989 8.72E-47 1.03E-45 94.83274562

AC020913.3 0.709166213 0.36578649 16.82191426 1.00E-46 1.19E-45 94.69262834

AF131215.4 -0.275494262 0.255690461 -16.81179621 1.10E-46 1.30E-45 94.59915307

TMEM72-AS1 -0.196467417 0.219596396 -16.80790082 1.14E-46 1.35E-45 94.563167

AC091544.4 -1.107697515 1.022166297 -16.79321817 1.31E-46 1.55E-45 94.42753445

LINC02086 2.549545427 1.354233403 16.79105591 1.34E-46 1.58E-45 94.40756127

LINC01694 -0.582422262 0.440317872 -16.78977969 1.35E-46 1.59E-45 94.39577272

FAM111A-DT 0.625442099 1.903480341 16.78715087 1.38E-46 1.63E-45 94.37149039

AC004264.1 1.294201307 1.030293863 16.78417296 1.42E-46 1.68E-45 94.34398396

AL391069.2 0.733791211 0.709926746 16.78239915 1.45E-46 1.70E-45 94.32759985

AC011472.2 0.709874463 0.522641395 16.7814323 1.46E-46 1.72E-45 94.31866949

ERVK9-11 -0.459509931 0.526347381 -16.77815998 1.50E-46 1.77E-45 94.28844465

AC018926.3 -0.866156997 1.539447236 -16.77342177 1.57E-46 1.84E-45 94.24468122

AC002116.2 0.63373054 0.65503678 16.76166087 1.75E-46 2.05E-45 94.13605938

SNHG20 -0.912307132 2.64464685 -16.75037501 1.95E-46 2.28E-45 94.03183214

AC025043.1 0.294565097 0.176101188 16.74631944 2.02E-46 2.36E-45 93.99437981

AC011330.2 -0.837720784 1.454405132 -16.74054856 2.13E-46 2.49E-45 93.94108847

PCAT6 1.040665175 2.358131951 16.7198182 2.58E-46 3.01E-45 93.74966877

AC010761.4 -1.080618875 1.216234055 -16.71682347 2.65E-46 3.10E-45 93.72201805

PWRN2 -0.400283855 0.198464494 -16.71330394 2.74E-46 3.20E-45 93.68952243

AL590723.1 0.576357301 0.462532473 16.69758308 3.17E-46 3.69E-45 93.54438113

UCKL1-AS1 -0.572656698 0.768098108 -16.69421016 3.27E-46 3.81E-45 93.51324286

LINC00941 1.51730226 1.243879738 16.68611338 3.52E-46 4.10E-45 93.43849718

AC003991.1 -0.602624528 0.359316732 -16.67657656 3.85E-46 4.47E-45 93.35046253

SLCO4A1-AS1 1.886919973 1.628993151 16.67228901 4.00E-46 4.65E-45 93.31088574

AL451165.2 -1.047631792 3.071791556 -16.67146167 4.03E-46 4.68E-45 93.30324891

LINC01637 1.089151073 1.735276714 16.67020141 4.08E-46 4.73E-45 93.29161621

AC008514.1 1.366003163 0.796711972 16.66980587 4.10E-46 4.75E-45 93.2879652

AL022323.4 -0.099284529 0.096823942 -16.66853823 4.14E-46 4.80E-45 93.27626452

AC004988.1 0.456470481 0.273172325 16.658905 4.53E-46 5.24E-45 93.1873497

AP001020.2 -0.359045364 0.36779821 -16.65704047 4.61E-46 5.33E-45 93.17014068

AC010618.3 -0.633956219 1.417248139 -16.6565315 4.63E-46 5.35E-45 93.16544306

AC096642.1 -0.464857953 0.639868123 -16.62161334 6.39E-46 7.38E-45 92.84319832

AC096586.1 -0.669596615 0.325065791 -16.61710513 6.66E-46 7.69E-45 92.80159921

AL121845.1 -0.892334035 0.433197038 -16.6127424 6.94E-46 8.00E-45 92.76134372

LINC01287 -0.160526867 0.104308576 -16.6122303 6.97E-46 8.03E-45 92.75661859

AL355075.2 -0.960780933 1.727425655 -16.60707905 7.31E-46 8.41E-45 92.70908894

AC037487.1 -0.28780512 0.238563769 -16.60136561 7.71E-46 8.86E-45 92.65637388

FAM41C -0.660637647 0.350753435 -16.59550695 8.14E-46 9.34E-45 92.60232101

AC093484.4 1.070266034 0.889815269 16.5945048 8.21E-46 9.42E-45 92.59307519

AC100771.2 -0.312984819 0.314588969 -16.59267534 8.35E-46 9.58E-45 92.57619683

RMST -1.309145168 1.155265291 -16.586518 8.84E-46 1.01E-44 92.51939158

AL512770.1 -0.526570478 0.985923575 -16.58241934 9.18E-46 1.05E-44 92.4815802

LIMS1-AS1 0.558852885 0.35468573 16.55910441 1.14E-45 1.30E-44 92.26651246

LINC02550 0.866226496 0.651783319 16.55728462 1.16E-45 1.32E-44 92.24972726

AP000757.1 -1.323273351 1.991284112 -16.55519863 1.18E-45 1.35E-44 92.23048699

AL109811.1 -0.353526587 0.39541929 -16.54695218 1.27E-45 1.45E-44 92.15442788

AC002070.1 0.811466384 0.685024158 16.54329944 1.32E-45 1.50E-44 92.12073908

AC016629.2 -0.31596506 0.189136939 -16.53905919 1.37E-45 1.56E-44 92.08163283

LINC00174 -0.928495283 2.35491816 -16.52691211 1.53E-45 1.74E-44 91.96961095

AL512306.2 -0.796827185 1.042208743 -16.5218438 1.61E-45 1.83E-44 91.92287312

KCNQ1OT1 -0.243095871 0.378862077 -16.52032036 1.63E-45 1.85E-44 91.90882489

AC124283.2 0.901373333 0.736667474 16.51854827 1.66E-45 1.88E-44 91.89248403

HAS2-AS1 0.689230568 0.488585741 16.51205747 1.76E-45 2.00E-44 91.83263229

FOXP4-AS1 -1.229868666 1.564240486 -16.51174106 1.76E-45 2.00E-44 91.82971474

OBI1-AS1 -0.236905575 0.142813736 -16.51061572 1.78E-45 2.02E-44 91.81933828

AC069200.1 0.973533056 0.868814862 16.49313848 2.09E-45 2.37E-44 91.65819543

RAMP2-AS1 -0.467141226 0.634728753 -16.49128279 2.13E-45 2.41E-44 91.64108679

TMEM220-AS1 -0.705361712 1.362962636 -16.46234255 2.78E-45 3.14E-44 91.37430004

AC113346.1 1.577441559 0.812544444 16.46158358 2.80E-45 3.16E-44 91.36730417

AL139274.2 -0.254569213 0.355333815 -16.448147 3.17E-45 3.58E-44 91.24345776

AC099518.2 0.916167144 0.544389917 16.42321105 3.99E-45 4.50E-44 91.01365193

AP001628.1 0.774685516 0.467828804 16.38308592 5.78E-45 6.51E-44 90.64395246

AC109809.1 -0.701261407 0.541435556 -16.36463392 6.85E-45 7.71E-44 90.47397866

MAN1B1-DT 0.797201078 1.367936448 16.36008125 7.15E-45 8.04E-44 90.43204454

AC120498.4 1.542677314 0.933260636 16.33044926 9.39E-45 1.06E-43 90.15914302

AL162724.2 0.923288984 0.780680694 16.32529562 9.85E-45 1.11E-43 90.11168582

AL590822.2 -0.114307824 0.108077784 -16.32489138 9.89E-45 1.11E-43 90.10796351

AC008543.1 -0.217244235 0.317709941 -16.31408711 1.09E-44 1.22E-43 90.00847902

AF131215.2 -0.188343521 0.134487745 -16.30953574 1.14E-44 1.28E-43 89.96657299

AP001029.2 -0.488974877 0.397482879 -16.30429393 1.20E-44 1.34E-43 89.91831168

AC025423.4 -1.05106293 0.51025438 -16.29870679 1.26E-44 1.41E-43 89.86687306

ZNF503-AS2 -0.749619449 1.665858458 -16.29282075 1.33E-44 1.48E-43 89.81268502

AL021392.1 0.69297097 0.442451424 16.26683971 1.69E-44 1.88E-43 89.5735282

AC016907.2 -0.134886248 0.113564891 -16.25442506 1.89E-44 2.11E-43 89.459268

AC234775.3 -0.767608544 1.130891295 -16.25277168 1.92E-44 2.14E-43 89.44405177

AC048344.4 0.700191661 0.593569583 16.24658257 2.03E-44 2.27E-43 89.3870942

AL109910.1 -0.508346897 0.281629308 -16.2422944 2.12E-44 2.36E-43 89.34763249

Z95331.1 -0.109477703 0.154433241 -16.24200154 2.12E-44 2.36E-43 89.34493752

AC009041.2 -1.560448424 3.056202475 -16.23246184 2.32E-44 2.58E-43 89.25715376

MIATNB -0.671493193 1.971196428 -16.22040952 2.59E-44 2.88E-43 89.14625863

AC111000.4 -0.514631758 0.331997044 -16.2143437 2.74E-44 3.04E-43 89.09045023

AC138932.2 -0.425618514 0.306819063 -16.21066098 2.83E-44 3.14E-43 89.05656876

LINC02818 0.459700569 0.262722119 16.19312682 3.33E-44 3.69E-43 88.89526669

AC004943.1 -0.359941839 0.421039017 -16.19214118 3.36E-44 3.72E-43 88.88620011

AC007878.1 -0.473395126 0.683217541 -16.18205456 3.69E-44 4.08E-43 88.79342142

AC022784.1 1.470041958 1.003539418 16.17764465 3.84E-44 4.24E-43 88.75286071

AL354733.2 -0.277918319 0.190746846 -16.17632098 3.89E-44 4.29E-43 88.74068634

AC073611.1 0.775325354 1.633879455 16.15399626 4.77E-44 5.27E-43 88.53537654

AL133338.1 0.597779352 1.057092391 16.14983829 4.96E-44 5.47E-43 88.49714191

AL031651.2 0.720499869 0.697768601 16.14672411 5.10E-44 5.62E-43 88.46850631

AL360219.1 0.699696163 0.661433906 16.1419872 5.33E-44 5.87E-43 88.42495086

HMGA1P4 1.107224824 1.098373798 16.13810817 5.53E-44 6.08E-43 88.38928481

LINC01320 -1.34077 1.328112167 -16.13692861 5.59E-44 6.14E-43 88.37843946

LINC02323 0.926226293 0.538982428 16.12429646 6.27E-44 6.89E-43 88.26230127

APTR -1.026034375 3.666061747 -16.12091006 6.47E-44 7.10E-43 88.23116934

AC022613.3 -0.337069564 0.370443078 -16.12056033 6.49E-44 7.12E-43 88.22795423

AL391883.1 -0.669687289 0.451736093 -16.11381787 6.91E-44 7.57E-43 88.16597219

LINC00332 -0.689065866 0.352546496 -16.11170478 7.05E-44 7.71E-43 88.14654763

AC007376.2 0.291065833 0.170495031 16.1091578 7.21E-44 7.89E-43 88.12313514

AL358472.3 0.936141424 1.53766762 16.1076672 7.31E-44 7.99E-43 88.10943333

AC009107.2 0.465108314 0.507824309 16.09543694 8.18E-44 8.94E-43 87.99701808

AC097382.2 -0.217357761 0.155381498 -16.08253545 9.22E-44 1.01E-42 87.87844595

LINC00460 1.578643887 0.842635774 16.07919874 9.50E-44 1.04E-42 87.8477818

UBE2D3-AS1 0.585920436 1.421691964 16.07177322 1.02E-43 1.11E-42 87.779545

AL590133.1 0.381317165 0.214293776 16.0664781 1.07E-43 1.16E-42 87.73088821

LINC00922 0.709329909 0.378479543 16.0635858 1.10E-43 1.19E-42 87.70431186

AC002401.4 1.344529425 0.782743821 16.05737287 1.16E-43 1.26E-42 87.64722569

AC104083.1 1.290231961 3.12905533 16.04922524 1.25E-43 1.36E-42 87.57236766

AC132872.2 -0.444484386 0.49657404 -16.04042523 1.36E-43 1.47E-42 87.49152174

TARID -0.296763623 0.287817747 -16.02254944 1.60E-43 1.74E-42 87.32731592

AC009139.2 -0.987882713 0.482873966 -16.00797357 1.83E-43 1.98E-42 87.19344228

CCDC84-DT 0.513468134 0.792793096 15.98990822 2.16E-43 2.34E-42 87.0275436

EIF1B-AS1 -0.373198224 0.636366887 -15.97864656 2.40E-43 2.59E-42 86.92413875

AC048382.1 -0.295824315 0.280301764 -15.9780975 2.41E-43 2.60E-42 86.91909755

AC012073.1 0.574943851 0.826728374 15.9703019 2.59E-43 2.80E-42 86.84752471

AC022706.1 0.860351493 0.852558579 15.95579289 2.96E-43 3.19E-42 86.71432845

PAPPA-AS1 -0.430683934 0.209082018 -15.94554356 3.25E-43 3.51E-42 86.62024788

C10orf25 0.544922387 1.179547693 15.92324937 3.99E-43 4.30E-42 86.4156361

AC004112.1 0.297795384 0.163513851 15.92220698 4.03E-43 4.34E-42 86.40607035

AC007608.3 -0.335616011 0.304853373 -15.92088794 4.07E-43 4.39E-42 86.3939659

AC025048.2 -0.619712902 1.223417128 -15.9196447 4.12E-43 4.43E-42 86.3825572

AC244517.11 -0.731056451 0.375307348 -15.90595293 4.67E-43 5.02E-42 86.25692224

AC124067.2 1.230041892 0.79088409 15.90506199 4.71E-43 5.06E-42 86.24874756

LINC02202 -0.645406458 0.720971084 -15.86848335 6.59E-43 7.08E-42 85.91318689

ZNF236-DT 0.607528845 1.014613773 15.86288968 6.94E-43 7.45E-42 85.86188276

AF124730.1 1.063555804 0.588501716 15.85218907 7.66E-43 8.21E-42 85.76374632

CDC37L1-DT -1.158545771 2.000189894 -15.8498696 7.82E-43 8.38E-42 85.74247558

AC015660.1 1.10742449 0.573243587 15.84800673 7.96E-43 8.52E-42 85.72539243

AP000345.2 -0.656706931 0.826111069 -15.84596728 8.11E-43 8.67E-42 85.70669029

AC145098.1 0.94949518 1.013997969 15.84486205 8.19E-43 8.75E-42 85.69655539

SCAT1 0.950960937 0.551732671 15.83650666 8.85E-43 9.44E-42 85.61993974

AC022126.1 0.439119569 0.252149931 15.81463173 1.08E-42 1.15E-41 85.41938504

AC027763.2 0.790663706 0.473882517 15.80448202 1.19E-42 1.27E-41 85.32634467

AC026803.2 0.545343455 0.70806994 15.80123237 1.22E-42 1.30E-41 85.2965578

AL135960.1 -0.177220731 0.198904558 -15.79807497 1.26E-42 1.34E-41 85.26761743

Z95114.4 -0.126458608 0.082304411 -15.7943865 1.30E-42 1.38E-41 85.23381039

AC063948.1 0.88046666 0.754846886 15.79008167 1.35E-42 1.44E-41 85.19435564

AC007383.2 0.707131421 2.440776791 15.78512188 1.42E-42 1.51E-41 85.1489002

STPG3-AS1 -0.758043481 0.89040918 -15.77253068 1.59E-42 1.69E-41 85.03351459

MANCR 0.974088174 0.620098115 15.76518486 1.70E-42 1.81E-41 84.96620425

LINC00886 -0.795876042 1.490143931 -15.74487443 2.05E-42 2.17E-41 84.78012437

PICART1 0.613897454 0.550855035 15.73150198 2.32E-42 2.46E-41 84.65762971

AC099343.3 0.533207678 1.549653049 15.73032055 2.34E-42 2.48E-41 84.64680834

AC010186.3 0.793551974 1.176294584 15.72725954 2.41E-42 2.55E-41 84.61877154

AL033384.2 0.757968902 0.823374698 15.71669458 2.66E-42 2.81E-41 84.52201028

ZNRF3-AS1 -0.133339636 0.064731734 -15.71505892 2.70E-42 2.85E-41 84.5070307

AC124798.1 0.914986555 1.436006905 15.70943678 2.84E-42 2.99E-41 84.45554435

AC107223.1 -0.427961186 0.326820882 -15.70486248 2.96E-42 3.12E-41 84.41365615

AF186192.3 -0.637654468 0.68085044 -15.69449773 3.26E-42 3.43E-41 84.3187504

CASC19 1.326688347 0.707712963 15.68370793 3.59E-42 3.78E-41 84.21996354

AC068870.1 -0.200869136 0.190298149 -15.68046022 3.70E-42 3.89E-41 84.19023111

LINC00910 -0.54364006 1.105612986 -15.6799817 3.72E-42 3.91E-41 84.18585033

AL008729.1 -0.411818282 0.648468522 -15.67611896 3.85E-42 4.05E-41 84.15048883

AP001094.2 -0.366911501 0.556876039 -15.67149269 4.02E-42 4.22E-41 84.10813948

AL355376.1 -0.656165872 0.408658048 -15.6622051 4.38E-42 4.59E-41 84.02312605

AL161725.1 -0.367386419 0.348391335 -15.6561887 4.63E-42 4.85E-41 83.96805974

AC108058.1 0.607886194 0.491278953 15.65242629 4.79E-42 5.01E-41 83.93362538

AC005387.1 0.777639249 0.763181041 15.65152751 4.83E-42 5.05E-41 83.92539968

AP001434.1 0.890098295 0.479652946 15.64993442 4.90E-42 5.12E-41 83.91081998

AC012186.2 0.670346209 0.515224129 15.64901219 4.94E-42 5.16E-41 83.9023799

AC107032.2 0.492258489 0.341717964 15.63427535 5.66E-42 5.90E-41 83.76752319

LINC01561 0.840930016 0.468766294 15.62926165 5.92E-42 6.18E-41 83.72164779

AC021231.1 -0.180829305 0.130007758 -15.61926676 6.49E-42 6.76E-41 83.63020159

C15orf56 -0.504888966 0.691856329 -15.59335135 8.23E-42 8.57E-41 83.39313996

AC018647.2 0.633533514 2.268549818 15.58683451 8.73E-42 9.09E-41 83.33353754

AC005225.2 -0.462500737 0.437116685 -15.58195325 9.13E-42 9.50E-41 83.28889682

LINC00612 -0.22929665 0.221830608 -15.56684594 1.05E-41 1.09E-40 83.15075054

AF230666.1 -0.450980036 0.668375181 -15.56507641 1.07E-41 1.11E-40 83.13457084

TMEM99 -0.983802374 3.947595896 -15.55202143 1.20E-41 1.25E-40 83.01521259

EBLN3P -0.973296993 4.775868349 -15.53030036 1.47E-41 1.52E-40 82.81666081

AC003956.1 0.445724997 0.2856788 15.527552 1.50E-41 1.56E-40 82.79154154

AC068580.2 1.264057024 0.651297169 15.52474832 1.54E-41 1.60E-40 82.76591755

LINC02249 -0.228983154 0.117000391 -15.51099027 1.75E-41 1.81E-40 82.64018833

LINC02844 -0.631224691 0.342872198 -15.5106441 1.75E-41 1.81E-40 82.63702504

AC017033.1 0.600431471 0.359601738 15.50699308 1.81E-41 1.87E-40 82.60366329

AC073575.2 -0.758483384 1.179394168 -15.48911643 2.14E-41 2.21E-40 82.4403325

AL021807.1 1.104513819 0.783997596 15.48345408 2.25E-41 2.32E-40 82.38860504

AC027117.1 -1.380972 2.007335877 -15.48130425 2.30E-41 2.37E-40 82.3689665

LINC00319 -0.941819741 0.470300704 -15.47988813 2.33E-41 2.40E-40 82.35603064

AC092171.2 0.845535579 2.324313698 15.47852574 2.35E-41 2.42E-40 82.34358581

BACH1-IT1 0.365104275 0.416331286 15.4707507 2.53E-41 2.60E-40 82.27256771

RPS6KA2-IT1 0.395906069 0.232835573 15.46849888 2.58E-41 2.65E-40 82.25200051

AL033527.3 -0.492064315 0.565481469 -15.4659973 2.64E-41 2.71E-40 82.22915277

AP001363.1 0.478006081 0.246846538 15.45387626 2.95E-41 3.03E-40 82.11845661

AL162377.1 -0.832405266 1.873788109 -15.44994777 3.06E-41 3.14E-40 82.08258273

LINC02577 1.129082051 0.59459663 15.43489284 3.51E-41 3.60E-40 81.9451204

BDNF-AS -0.562021832 1.400925108 -15.42985642 3.67E-41 3.76E-40 81.89913973

AL121761.1 1.562331644 1.180928314 15.42782187 3.74E-41 3.83E-40 81.88056573

AP000944.1 -0.711921569 0.611587044 -15.42781022 3.74E-41 3.83E-40 81.88045938

AL355987.4 -1.125708287 1.429613934 -15.41689237 4.14E-41 4.23E-40 81.78079487

JPX -0.807819534 3.402017524 -15.4072686 4.52E-41 4.61E-40 81.69295414

ERICH6-AS1 0.797176472 1.323894756 15.39737669 4.94E-41 5.05E-40 81.60267631

AC090579.1 0.531205889 0.402917363 15.39669995 4.98E-41 5.07E-40 81.5965005

AC022929.2 -0.408564257 0.245435209 -15.39606642 5.00E-41 5.10E-40 81.59071905

AC005726.5 -0.479053814 0.27127134 -15.39453667 5.07E-41 5.17E-40 81.57675906

AC242426.2 -0.556572974 1.394911311 -15.3919542 5.20E-41 5.29E-40 81.55319278

AC112491.1 -1.691135766 2.971195359 -15.38922048 5.33E-41 5.42E-40 81.52824721

LINC00996 0.618189428 0.433056048 15.38440587 5.57E-41 5.66E-40 81.48431501

MIR31HG 1.45054418 0.834780631 15.38416203 5.58E-41 5.66E-40 81.48209009

LINC01136 -0.984764674 0.627197185 -15.37995977 5.80E-41 5.88E-40 81.44374766

LINC01605 0.937962206 0.599742341 15.37736313 5.94E-41 6.02E-40 81.42005626

AC005746.2 0.29361912 0.178259978 15.37194952 6.24E-41 6.32E-40 81.37066561

AL662884.1 0.266152977 0.1561283 15.36519116 6.64E-41 6.72E-40 81.30901062

WAKMAR2 -0.65819993 1.201145973 -15.35731326 7.13E-41 7.21E-40 81.23714863

AC009403.1 -0.844391686 2.536450546 -15.35639627 7.19E-41 7.27E-40 81.22878435

AC019254.1 0.447815154 0.345678787 15.35472602 7.30E-41 7.38E-40 81.21354938

KMT2E-AS1 1.127177853 2.80893127 15.35249549 7.45E-41 7.52E-40 81.1932044

AC026304.1 -0.681643823 1.033737416 -15.30977392 1.10E-40 1.11E-39 80.80364136

AC074091.2 -0.292203623 0.151379061 -15.30683229 1.13E-40 1.14E-39 80.77682512

LINC01118 -0.259257097 0.200265909 -15.30000172 1.20E-40 1.21E-39 80.7145606

LINC00271 -0.537090205 0.631796823 -15.29998742 1.20E-40 1.21E-39 80.71443021

AC096531.2 -0.2148104 0.185301224 -15.29900126 1.21E-40 1.22E-39 80.70544129

AL445250.1 0.929804953 0.596508354 15.29309041 1.28E-40 1.29E-39 80.65156542

AL390955.2 0.528032212 0.505627577 15.289383 1.33E-40 1.33E-39 80.61777534

AC103740.1 -0.697573479 0.506378695 -15.28759824 1.35E-40 1.35E-39 80.6015092

AC026191.1 -0.640220586 0.561040935 -15.27823293 1.47E-40 1.47E-39 80.51616068

AC007743.1 -0.420868147 0.563522822 -15.27702925 1.48E-40 1.49E-39 80.50519195

MAFG-DT 0.861000559 2.0811783 15.27443726 1.52E-40 1.52E-39 80.48157261

AC010680.2 -0.405706149 0.720018305 -15.27055822 1.57E-40 1.58E-39 80.44622649

AC064807.2 -1.249706875 1.599831008 -15.26988924 1.58E-40 1.58E-39 80.44013086

Z94721.1 0.444916207 0.294252089 15.26593184 1.64E-40 1.64E-39 80.40407275

ABALON 0.639070837 0.771144359 15.26572681 1.65E-40 1.64E-39 80.40220465

AC135803.1 -1.053503087 1.720780426 -15.26195429 1.70E-40 1.70E-39 80.36783286

AC007066.2 -0.658756429 1.494502617 -15.25514725 1.81E-40 1.81E-39 80.30581727

AC008440.1 0.464088387 0.296209339 15.25335471 1.84E-40 1.84E-39 80.28948727

AC002451.1 -0.927420956 0.940739151 -15.25136595 1.88E-40 1.87E-39 80.27137006

WNT5A-AS1 1.028295767 0.959693929 15.24859805 1.92E-40 1.92E-39 80.24615582

AC067930.1 -0.68625401 0.333152375 -15.23868062 2.11E-40 2.09E-39 80.15582003

AC026470.2 0.418175874 0.22864678 15.23104066 2.26E-40 2.24E-39 80.0862369

C2CD4D-AS1 1.049628421 1.529904054 15.22424471 2.40E-40 2.39E-39 80.02434642

AC008543.5 -0.1176848 0.075255213 -15.21108766 2.71E-40 2.69E-39 79.90454091

AC004801.6 0.452509107 0.607436024 15.19601328 3.11E-40 3.08E-39 79.7673013

DCXR-DT -0.729809817 0.617216076 -15.19559839 3.12E-40 3.09E-39 79.76352449

AL021937.1 -0.264769088 0.317834167 -15.19432145 3.16E-40 3.13E-39 79.75190023

ACBD3-AS1 0.786953449 0.960997654 15.1801038 3.59E-40 3.56E-39 79.62248759

AC005041.3 0.856303371 1.020847546 15.17245295 3.85E-40 3.81E-39 79.55285732

AL731571.1 -0.628797338 1.239164389 -15.17077674 3.91E-40 3.87E-39 79.53760312

LINC02212 -0.53396007 0.363083625 -15.16921432 3.97E-40 3.92E-39 79.52338472

SOX21-AS1 1.18219442 0.663921679 15.16188779 4.24E-40 4.19E-39 79.45671542

AC008083.3 0.585751683 0.389291081 15.15666216 4.45E-40 4.39E-39 79.40916757

GNG12-AS1 -0.560236564 0.832484097 -15.15662197 4.45E-40 4.39E-39 79.40880194

AL359317.2 -0.366996397 0.318279773 -15.14886594 4.78E-40 4.71E-39 79.33823601

AC130456.2 1.140847035 0.688758625 15.14703262 4.86E-40 4.78E-39 79.32155716

AL008727.1 -0.105791341 0.109401599 -15.14645215 4.88E-40 4.80E-39 79.31627635

AL021328.1 -1.006326533 1.090441866 -15.14570238 4.92E-40 4.83E-39 79.30945539

TRAPPC12-AS1 0.744434134 1.298715224 15.11249778 6.66E-40 6.54E-39 79.007447

PCCA-DT 0.866462465 2.320383028 15.11144905 6.72E-40 6.59E-39 78.99791061

FAM66B -0.396622645 0.541262598 -15.10896111 6.87E-40 6.74E-39 78.97528752

AC006378.1 0.595693767 0.733980126 15.10736114 6.97E-40 6.83E-39 78.9607392

HDAC2-AS2 -0.355975153 0.356911421 -15.10207164 7.32E-40 7.17E-39 78.91264465

AC093726.1 0.664325724 2.230798575 15.09691613 7.67E-40 7.51E-39 78.86577172

AL133245.1 -0.18098189 0.16132156 -15.09649385 7.70E-40 7.53E-39 78.8619326

AC005329.3 -0.270175804 0.224110686 -15.091247 8.08E-40 7.89E-39 78.81423277

LINC02408 0.330588742 0.193812183 15.09122257 8.08E-40 7.89E-39 78.81401067

AC011379.2 -0.874159166 0.88096147 -15.07302601 9.54E-40 9.31E-39 78.64860926

AL161452.1 -0.533269419 0.843462102 -15.05025214 1.17E-39 1.14E-38 78.44165839

AC068580.3 0.983226966 1.656731726 15.04880287 1.19E-39 1.16E-38 78.4284908

TMLHE-AS1 -0.338653626 0.227998567 -15.03144514 1.39E-39 1.36E-38 78.27080353

AC113383.1 0.690541431 0.520650178 15.0287271 1.43E-39 1.39E-38 78.2461147

AC007405.1 -0.356034679 0.380591692 -15.02593309 1.46E-39 1.42E-38 78.22073677

AL365181.2 1.467256811 1.028389773 15.01896909 1.56E-39 1.52E-38 78.1574872

LINC02331 1.232077073 0.980754327 15.01556761 1.61E-39 1.56E-38 78.12659591

AL354919.2 0.74794364 0.397520595 15.01326264 1.64E-39 1.59E-38 78.10566366

NSMCE1-DT -0.570409846 0.557588334 -15.01121689 1.67E-39 1.62E-38 78.08708603

CPEB1-AS1 -0.167842766 0.128197851 -15.00115936 1.83E-39 1.78E-38 77.99576028

AC135050.5 -0.771210769 1.789820494 -15.00087171 1.84E-39 1.78E-38 77.99314853

OCIAD1-AS1 0.867618511 0.736679013 14.99550399 1.93E-39 1.87E-38 77.94441321

AC119396.2 0.66766865 0.557587661 14.99540441 1.93E-39 1.87E-38 77.94350919

AC020663.2 0.70689289 0.675101196 14.99530435 1.93E-39 1.87E-38 77.9426007

AC092118.2 -0.817045839 1.242213705 -14.98799513 2.07E-39 2.00E-38 77.87624393

AC009133.4 -0.856836971 0.415964452 -14.97582303 2.31E-39 2.23E-38 77.76575435

LINC01050 0.837605152 0.443443516 14.96944119 2.45E-39 2.36E-38 77.70783202

AC067852.2 -0.634935687 2.725016739 -14.9620103 2.62E-39 2.52E-38 77.64039501

AC009118.3 -0.791765964 2.234317721 -14.95835654 2.71E-39 2.61E-38 77.60723891

AC067930.4 0.790181807 0.424133773 14.93828623 3.25E-39 3.13E-38 77.42514071

AC068234.2 0.645895481 0.539481473 14.93550924 3.33E-39 3.20E-38 77.39994905

AC016957.2 0.843494359 1.164595169 14.92949492 3.52E-39 3.38E-38 77.34539324

AC008870.4 0.276087968 0.17021595 14.92714824 3.59E-39 3.45E-38 77.32410784

LINC02731 -0.526272042 0.601104566 -14.92183578 3.77E-39 3.62E-38 77.27592402

LINC00641 -0.84336425 2.185652191 -14.92066981 3.81E-39 3.66E-38 77.26534924

AL357054.4 0.484998493 0.479576919 14.91086887 4.17E-39 4.00E-38 77.17646623

AC020907.1 -1.267266401 1.619885718 -14.88599275 5.22E-39 5.00E-38 76.95092487

AL359962.1 0.958911986 0.96702481 14.88295174 5.37E-39 5.14E-38 76.92335887

AC099518.6 0.754040683 0.836398069 14.87322513 5.87E-39 5.61E-38 76.83519757

AL121820.1 0.416340624 0.260649875 14.87142987 5.96E-39 5.70E-38 76.81892679

AC025164.1 -0.520436676 0.509844119 -14.8704747 6.01E-39 5.75E-38 76.81027011

HIF1A-AS3 0.783161838 0.500065332 14.86353548 6.41E-39 6.12E-38 76.74738369

LINC02257 0.884547511 0.470563686 14.859822 6.63E-39 6.32E-38 76.71373298

AP002954.1 0.652186173 0.409062665 14.85938909 6.65E-39 6.34E-38 76.70981013

AC243967.2 0.810512181 0.422748856 14.85422168 6.97E-39 6.64E-38 76.66298757

ZNF30-AS1 -0.500105499 0.490381251 -14.84171783 7.81E-39 7.44E-38 76.54970324

SLC8A1-AS1 -0.379734888 0.246376156 -14.83515263 8.29E-39 7.89E-38 76.49023112

AC006435.1 -0.637446654 0.309458106 -14.83475176 8.32E-39 7.91E-38 76.48659991

AC022075.1 0.793531432 0.892892462 14.81667071 9.80E-39 9.31E-38 76.32283992

DLEU2 -0.509703524 1.239294001 -14.81068021 1.03E-38 9.83E-38 76.2685936

AC008946.1 0.65063252 0.758710279 14.78721837 1.28E-38 1.22E-37 76.05618388

AC005609.4 -0.541659285 0.26295668 -14.77505238 1.43E-38 1.36E-37 75.94606944

AC097534.2 -0.756337047 1.696698998 -14.76861355 1.52E-38 1.44E-37 75.88779969

LINC02150 -0.155970778 0.087450407 -14.76104134 1.62E-38 1.54E-37 75.81928032

AL359878.1 -0.234196755 0.311753206 -14.75839118 1.66E-38 1.57E-37 75.79530145

AC061975.1 -0.26806535 0.160226559 -14.75236178 1.76E-38 1.66E-37 75.74075046

SNHG18 -1.232453852 3.980638502 -14.74658022 1.85E-38 1.75E-37 75.6884465

AL391832.3 0.317411328 0.244011358 14.7463747 1.85E-38 1.75E-37 75.68658728

SENCR 0.57899815 0.674651603 14.74458797 1.88E-38 1.78E-37 75.67042429

AC019257.1 -0.880385554 1.391927466 -14.74239487 1.92E-38 1.81E-37 75.65058576

AC010266.2 -0.135571695 0.0865791 -14.73099504 2.13E-38 2.01E-37 75.547475

LINC02100 0.519478203 0.268185392 14.73071879 2.14E-38 2.01E-37 75.54497658

AC011352.3 0.864541541 0.596293156 14.72713807 2.21E-38 2.08E-37 75.51259302

AC016590.3 0.637302389 0.919763287 14.72317681 2.29E-38 2.15E-37 75.47677001

AC025271.2 -0.277114861 0.169649138 -14.71720214 2.42E-38 2.27E-37 75.42274311

HOXB-AS4 1.061833589 0.55202681 14.70511141 2.70E-38 2.53E-37 75.3134258

AL022311.1 -0.241363157 0.402851786 -14.70477177 2.70E-38 2.54E-37 75.31035532

AC034102.6 -0.482816126 0.555881844 -14.68959788 3.10E-38 2.91E-37 75.17319142

AC027601.2 -0.431134805 0.866426362 -14.68916418 3.11E-38 2.92E-37 75.16927153

AC009549.1 1.026378495 0.901501046 14.68635743 3.19E-38 2.99E-37 75.14390373

AC023510.2 -0.869060686 1.219534474 -14.68509384 3.23E-38 3.03E-37 75.13248352

AC010999.1 -0.759192558 0.776730427 -14.68332822 3.28E-38 3.07E-37 75.11652646

AL109936.2 -1.046825721 1.666366667 -14.67412153 3.57E-38 3.34E-37 75.03332673

AC009093.2 0.627889291 0.701613887 14.65122499 4.39E-38 4.10E-37 74.82646508

LINC02542 0.741305167 0.562418741 14.64911015 4.48E-38 4.18E-37 74.807362

LINC02288 -0.177740597 0.179417708 -14.64618387 4.60E-38 4.29E-37 74.78093038

RTCA-AS1 0.760169271 1.288798533 14.64206597 4.77E-38 4.45E-37 74.74373752

AC004854.2 0.435919086 0.593580319 14.63533397 5.07E-38 4.72E-37 74.68293928

AC174065.1 -0.147134475 0.116880327 -14.63107997 5.27E-38 4.91E-37 74.64452373

AC018816.1 0.863749095 0.968876812 14.62317887 5.66E-38 5.27E-37 74.57318018

PSMG3-AS1 -0.821087641 1.847996479 -14.61728352 5.97E-38 5.55E-37 74.51995345

SLC9A3-AS1 -1.603192443 3.780424467 -14.60780779 6.50E-38 6.05E-37 74.43441133

AC015961.2 -0.716382418 0.951001717 -14.60452637 6.70E-38 6.22E-37 74.40479131

AC011337.1 -0.239096215 0.419275742 -14.58904278 7.71E-38 7.15E-37 74.26504843

AC092802.1 -0.443761543 0.500849689 -14.58821281 7.76E-38 7.20E-37 74.25755869

AC093278.2 0.736008837 1.574344289 14.56608778 9.48E-38 8.79E-37 74.05793816

AL121895.2 1.003719984 1.150917106 14.54720113 1.12E-37 1.04E-36 73.88759185

AC093297.2 0.608465562 2.569560155 14.54483487 1.15E-37 1.06E-36 73.86625326

FBXL19-AS1 0.472526087 0.83475792 14.54282412 1.17E-37 1.08E-36 73.84812128

AC145124.1 -0.516239223 0.755205323 -14.54148718 1.18E-37 1.10E-36 73.83606571

BACE1-AS -0.88281185 3.333360496 -14.54113024 1.19E-37 1.10E-36 73.83284719

AP001020.3 -0.253382796 0.312312894 -14.53563402 1.25E-37 1.15E-36 73.78328927

AC026202.2 -0.777059398 1.070134317 -14.53102791 1.30E-37 1.20E-36 73.74176066

LINC00316 -0.692675143 0.608351195 -14.50341059 1.67E-37 1.54E-36 73.49282913

AC000068.3 -0.907405537 0.440513727 -14.49834154 1.75E-37 1.61E-36 73.447151

AC006059.1 0.483175785 0.621940379 14.49646263 1.78E-37 1.64E-36 73.43022075

AC011374.2 0.694802812 1.596316705 14.49473545 1.81E-37 1.66E-36 73.41465821

AL354696.2 -0.380868566 0.551901503 -14.48287271 2.01E-37 1.85E-36 73.30778233

AC025682.1 -0.598993002 0.837498341 -14.45962202 2.48E-37 2.28E-36 73.09836914

AC012063.1 0.291349692 0.52142768 14.43698647 3.04E-37 2.80E-36 72.89457474

AC135507.1 -0.661676431 1.19263508 -14.43621115 3.06E-37 2.81E-36 72.88759577

AC025171.4 0.684962006 1.038504881 14.43377432 3.13E-37 2.87E-36 72.86566112

LINC00261 -2.044202712 3.674458647 -14.43114962 3.21E-37 2.94E-36 72.84203649

AL352984.1 1.007054431 0.526388782 14.43039945 3.23E-37 2.96E-36 72.83528456

LINC01942 -0.939570036 1.162945063 -14.42640927 3.35E-37 3.07E-36 72.79937184

AL118505.1 0.884522224 0.88387697 14.42569894 3.37E-37 3.08E-36 72.79297896

AL139220.2 -0.519746615 0.556682873 -14.42326702 3.44E-37 3.15E-36 72.77109248

AP003032.2 -1.050181849 0.509826646 -14.41189918 3.82E-37 3.49E-36 72.66879762

AC112487.1 -0.345856353 0.247681514 -14.41106957 3.84E-37 3.51E-36 72.66133307

EPB41L4A-DT 0.585820829 1.240719892 14.39976503 4.26E-37 3.89E-36 72.55962889

LINC01166 -0.196775289 0.142746555 -14.39591648 4.41E-37 4.02E-36 72.5250089

DUBR 0.591948804 1.264914623 14.39068019 4.62E-37 4.21E-36 72.47790903

AL117382.1 0.869550626 0.658370578 14.38474813 4.87E-37 4.44E-36 72.42455584

AL359317.1 0.239579939 0.158910505 14.37868123 5.15E-37 4.69E-36 72.3699956

AC060766.6 0.37195748 0.261144851 14.37652695 5.25E-37 4.78E-36 72.35062328

LINC01750 -0.651412233 0.73451281 -14.37548453 5.30E-37 4.82E-36 72.34124959

ITFG1-AS1 0.31951737 0.260242371 14.37213754 5.46E-37 4.96E-36 72.31115386

AL049840.5 0.85952211 4.018333718 14.36636996 5.75E-37 5.22E-36 72.25929658

FOXCUT 0.646437453 0.377122056 14.36096573 6.04E-37 5.48E-36 72.21071084

AP002498.1 1.608037288 0.853052767 14.35771217 6.22E-37 5.64E-36 72.18146255

LINC02562 1.462711893 1.188987106 14.35495427 6.38E-37 5.78E-36 72.15667132

C5orf60 -0.186872252 0.11374246 -14.35320442 6.48E-37 5.87E-36 72.14094228

AL596094.1 0.82258634 0.822389444 14.34680457 6.86E-37 6.21E-36 72.08341925

SEPTIN9-DT 0.515233049 0.281150147 14.3459804 6.91E-37 6.25E-36 72.07601191

AC092447.5 -0.15672081 0.097540025 -14.34274604 7.12E-37 6.43E-36 72.04694378

AP002893.1 -0.472124761 0.229200094 -14.33207673 7.83E-37 7.08E-36 71.95106713

AC114296.1 0.973832816 0.509491974 14.32631139 8.25E-37 7.45E-36 71.899266

AP001372.1 0.297792674 0.168294414 14.32535244 8.32E-37 7.51E-36 71.89065047

AC243965.2 -0.144698978 0.194046908 -14.31915403 8.80E-37 7.94E-36 71.83496505

AC109479.1 0.555021172 0.43309723 14.31455791 9.17E-37 8.27E-36 71.79367821

AC116158.1 -0.260127338 0.313913725 -14.29850736 1.06E-36 9.54E-36 71.64952262

AC021078.1 -0.815848364 2.752688369 -14.28358751 1.21E-36 1.09E-35 71.51555902

ARF4-AS1 -0.711944238 0.905488986 -14.28204802 1.23E-36 1.11E-35 71.50173807

AL096828.3 0.667120601 0.654804571 14.2724895 1.34E-36 1.20E-35 71.41593419

AC092171.5 0.885974991 1.501266587 14.26867959 1.39E-36 1.25E-35 71.38173785

AP001107.3 0.389918276 0.225610339 14.26794555 1.40E-36 1.25E-35 71.37514965

AC091132.2 0.385876288 0.243257832 14.25774955 1.53E-36 1.37E-35 71.28364688

ZKSCAN7-AS1 0.476714847 0.480583723 14.25403439 1.58E-36 1.42E-35 71.25030976

AC006111.2 -0.576740672 0.916593731 -14.25110602 1.62E-36 1.45E-35 71.22403427

AC078993.1 -1.15662435 1.143529548 -14.24991641 1.64E-36 1.47E-35 71.21336063

ST7-OT4 -0.348817945 0.251159154 -14.24942439 1.65E-36 1.47E-35 71.2089461

ETV5-AS1 -0.644352373 0.512013023 -14.24900639 1.65E-36 1.48E-35 71.20519566

AC019117.1 1.639613012 2.069634035 14.24180739 1.77E-36 1.58E-35 71.14060926

LINC00623 -0.78036636 3.069516887 -14.22397105 2.07E-36 1.85E-35 70.98062532

AL731567.1 1.143342376 1.450282831 14.21997091 2.15E-36 1.92E-35 70.94475288

DIO3OS -1.382795771 3.046480858 -14.219453 2.16E-36 1.92E-35 70.94010859

CASC2 -0.445304232 0.832928861 -14.21686843 2.21E-36 1.97E-35 70.91693232

AC130371.2 0.751473728 1.25524523 14.21583853 2.23E-36 1.99E-35 70.90769736

AL022322.2 -0.079528622 0.073271327 -14.2044647 2.47E-36 2.20E-35 70.80572118

DGCR9 -0.699618403 0.913525099 -14.18352427 2.98E-36 2.65E-35 70.61802749

AP000487.1 -0.452017391 0.692951852 -14.17980304 3.08E-36 2.74E-35 70.58468081

AL139423.1 -0.330930955 0.516555525 -14.17805509 3.13E-36 2.78E-35 70.56901787

AC079601.1 -0.210400373 0.14165107 -14.17180363 3.31E-36 2.94E-35 70.51300425

LINC02328 0.436319035 0.432462394 14.15762105 3.76E-36 3.34E-35 70.3859511

AC016924.1 -0.814126024 0.648486678 -14.15439784 3.87E-36 3.43E-35 70.35708088

MGAT3-AS1 0.589657618 0.413624563 14.15425887 3.88E-36 3.44E-35 70.3558362

FO393415.1 -0.18056558 0.135175072 -14.13848046 4.47E-36 3.96E-35 70.21453488

HSD17B3-AS1 -0.273494402 0.232654145 -14.13126684 4.76E-36 4.22E-35 70.14994815

LINC02285 0.503364332 0.394381678 14.12265326 5.15E-36 4.55E-35 70.07283834

AL591848.3 -0.216239595 0.314893606 -14.12233166 5.16E-36 4.56E-35 70.06995953

LINC00863 -0.526390268 1.331486073 -14.12130444 5.21E-36 4.60E-35 70.06076465

ARMCX3-AS1 -0.517055891 0.264044596 -14.11293179 5.62E-36 4.96E-35 69.98582547

AC105339.3 -0.322341311 0.391406219 -14.10644508 5.95E-36 5.25E-35 69.92777445

NAGPA-AS1 -0.180925355 0.103685803 -14.09542798 6.57E-36 5.80E-35 69.82919615

AC092756.1 0.524969077 0.28699656 14.09224298 6.76E-36 5.96E-35 69.80070133

LINC01842 0.841816786 0.440290103 14.08785612 7.03E-36 6.19E-35 69.76145689

AC069281.2 -0.981124526 2.556329943 -14.07005898 8.25E-36 7.26E-35 69.60227865

AL139339.1 0.413573437 0.401940229 14.06417862 8.70E-36 7.65E-35 69.5496963

AC112721.2 0.802172502 0.430612897 14.04980555 9.89E-36 8.70E-35 69.42119684

AP000550.1 -0.098379897 0.048869501 -14.04864867 1.00E-35 8.78E-35 69.4108555

TEX41 -0.446678196 0.315806384 -14.04830803 1.00E-35 8.80E-35 69.40781053

Z98257.1 1.552939759 0.937649632 14.04725886 1.01E-35 8.88E-35 69.39843237

AC009404.1 -0.598203738 1.339643993 -14.04514043 1.03E-35 9.05E-35 69.37949687

LIX1L-AS1 -0.523593542 0.656371446 -14.04413412 1.04E-35 9.12E-35 69.37050227

AL117381.1 0.443911086 0.284299539 14.044051 1.04E-35 9.12E-35 69.36975934

LINC02126 0.418671442 0.2459268 14.04140475 1.07E-35 9.34E-35 69.34610754

DIAPH2-AS1 0.425990426 0.299172522 14.0344507 1.14E-35 9.93E-35 69.283959

AC016747.1 0.681927171 2.282086494 14.03406459 1.14E-35 9.96E-35 69.28050851

AL589843.1 0.942597503 1.667605463 14.03091838 1.17E-35 1.02E-34 69.25239369

HOXB-AS2 0.704862047 0.651549714 14.03085011 1.17E-35 1.02E-34 69.25178367

AC104653.1 0.500283195 0.413101861 14.0291827 1.19E-35 1.04E-34 69.23688424

AC108047.1 0.749545956 0.759002176 14.02450247 1.24E-35 1.08E-34 69.1950658

AC087672.2 0.321158803 0.257498047 14.01121772 1.40E-35 1.22E-34 69.07638547

AC136475.1 -0.678265681 1.157814166 -14.00597636 1.46E-35 1.28E-34 69.02956978

AC015922.3 0.86217692 3.444347543 14.00176849 1.52E-35 1.32E-34 68.99198867

AC004009.1 1.056834021 0.544673705 14.00053531 1.54E-35 1.34E-34 68.9809755

AC021483.2 -0.342919845 0.594507129 -13.9930548 1.64E-35 1.43E-34 68.91417516

LINC02693 -0.685586882 1.610111893 -13.98939359 1.70E-35 1.48E-34 68.88148433

AP005019.1 0.608517182 0.504836692 13.98255855 1.81E-35 1.57E-34 68.82046084

FO393418.1 0.30838585 0.222255028 13.97605862 1.91E-35 1.66E-34 68.76243674

AC002091.2 0.607984276 0.443277458 13.96490245 2.12E-35 1.83E-34 68.66286425

AC004812.2 0.598361207 1.670156913 13.95072581 2.40E-35 2.08E-34 68.53636459

AC022211.1 0.616393631 0.550620598 13.94724439 2.48E-35 2.15E-34 68.50530489

OSGEPL1-AS1 -0.540885186 0.933841625 -13.93806921 2.69E-35 2.33E-34 68.42345828

LINC01394 0.254978237 0.144391821 13.91948593 3.18E-35 2.75E-34 68.25773294

AC084864.1 0.721904276 0.376292701 13.90949878 3.47E-35 3.00E-34 68.16869317

AL356356.1 0.800577851 1.172156492 13.90668642 3.56E-35 3.08E-34 68.14362288

TGFB3-AS1 -0.635613288 0.451151548 -13.90431059 3.64E-35 3.14E-34 68.12244518

AL162258.2 -0.76887216 1.446485273 -13.89086361 4.10E-35 3.54E-34 68.00260001

AL031289.1 0.296052591 0.153225121 13.88977927 4.14E-35 3.57E-34 67.99293729

LINC01772 -0.311866111 0.787856318 -13.88963284 4.15E-35 3.57E-34 67.99163246

FALEC 0.542854867 0.481828978 13.88841767 4.19E-35 3.61E-34 67.98080425

LRRC2-AS1 -0.227031956 0.160636717 -13.8860139 4.28E-35 3.69E-34 67.95938526

AP006545.2 -0.866162886 1.039172877 -13.8823984 4.42E-35 3.81E-34 67.92717106

AL157373.2 -0.866146909 1.108128702 -13.87942993 4.54E-35 3.91E-34 67.90072365

AL161669.1 0.785689108 0.511885884 13.86579787 5.13E-35 4.41E-34 67.7792902

AC022201.2 -0.672876171 0.326657901 -13.85035689 5.89E-35 5.06E-34 67.64178345

ALDH1L1-AS2 -0.407158756 0.432240521 -13.84609705 6.12E-35 5.25E-34 67.60385588

AL162586.1 -0.930216806 2.590270501 -13.84591835 6.13E-35 5.26E-34 67.60226484

AC010904.2 -0.585403475 0.663048538 -13.83788963 6.58E-35 5.64E-34 67.53079018

AL354872.2 -0.811753076 1.156326351 -13.8332778 6.86E-35 5.88E-34 67.48973922

AC005291.2 0.765734124 0.528839331 13.83264556 6.90E-35 5.91E-34 67.48411182

SLC2A1-AS1 0.485291697 0.50461278 13.82687968 7.26E-35 6.21E-34 67.43279451

AC027309.1 -0.740098918 0.391602299 -13.82650995 7.29E-35 6.23E-34 67.42950406

SNHG19 -1.574807104 6.246808729 -13.82480603 7.40E-35 6.32E-34 67.41434019

AC007038.1 -0.423762278 0.977768888 -13.82314508 7.51E-35 6.41E-34 67.39955921

AL096865.1 0.701151369 0.885100313 13.81621974 7.99E-35 6.82E-34 67.33793534

MORC2-AS1 -0.311695472 0.325596302 -13.81618126 7.99E-35 6.82E-34 67.33759294

AL133227.1 0.227616332 0.153499468 13.81046935 8.41E-35 7.17E-34 67.28677318

AC084824.3 -0.487124233 1.000627542 -13.80917252 8.50E-35 7.25E-34 67.27523587

LINC01352 0.50134 0.436939539 13.80491158 8.83E-35 7.52E-34 67.23733053

AC015961.1 -0.274462291 0.238795592 -13.79781392 9.41E-35 8.01E-34 67.17419717

AC012615.4 0.310518895 0.200339029 13.79495797 9.65E-35 8.21E-34 67.14879621

AP005482.3 0.663984036 1.325177515 13.79132178 9.97E-35 8.48E-34 67.11645794

MYOSLID 0.654384786 0.510168619 13.79059927 1.00E-34 8.53E-34 67.11003258

AC036103.1 0.250068481 0.17091109 13.78795162 1.03E-34 8.72E-34 67.08648773

AC009299.3 -0.25922169 0.278235749 -13.77895783 1.11E-34 9.45E-34 67.00651809

AL513165.1 1.051067405 2.482609319 13.77816543 1.12E-34 9.51E-34 66.99947308

LINC00630 -0.433346855 0.960126816 -13.77655486 1.14E-34 9.64E-34 66.98515422

AC079907.1 0.576830947 0.554534605 13.77341674 1.17E-34 9.91E-34 66.95725605

MACORIS 0.767441736 0.714529223 13.77065964 1.20E-34 1.01E-33 66.9327467

SMC2-AS1 -0.319663162 0.234921737 -13.76855914 1.22E-34 1.03E-33 66.91407516

LINC02320 0.912650403 0.517764397 13.76371124 1.28E-34 1.08E-33 66.87098483

LINC01655 0.681739033 0.353916507 13.74529978 1.50E-34 1.27E-33 66.70737544

AL049775.3 -0.544469314 0.417908383 -13.73591257 1.63E-34 1.38E-33 66.62398242

LINC00884 0.498051433 0.675348154 13.73196116 1.69E-34 1.43E-33 66.58888431

LINC01547 0.605087146 1.308940327 13.73111521 1.71E-34 1.44E-33 66.58137058

AC024075.3 -0.944332073 2.064870699 -13.73091483 1.71E-34 1.44E-33 66.5795908

AC117394.2 0.306162378 0.158426959 13.72311379 1.83E-34 1.54E-33 66.51030867

AC009108.3 -0.263046282 0.291060803 -13.71963867 1.89E-34 1.59E-33 66.47944935

AC007663.2 1.074512331 0.637209443 13.70740814 2.11E-34 1.77E-33 66.37085946

MALINC1 -0.500199468 1.358506242 -13.70365995 2.18E-34 1.83E-33 66.33758646

LINC02535 0.665712343 0.425972012 13.6935333 2.38E-34 2.00E-33 66.24770459

AC104695.3 0.826733191 1.277101951 13.69236313 2.41E-34 2.02E-33 66.23731963

PRC1-AS1 -0.336521217 0.339071747 -13.69176532 2.42E-34 2.03E-33 66.23201438

LINC00844 -1.195720719 0.784697547 -13.67022373 2.93E-34 2.46E-33 66.04088806

AC006942.1 -1.030191532 2.048446697 -13.66444866 3.09E-34 2.59E-33 65.98966417

AL008628.1 -0.107793314 0.075620394 -13.66148096 3.17E-34 2.66E-33 65.96334366

AL080312.2 -0.604094647 0.509186304 -13.66069324 3.19E-34 2.67E-33 65.95635774

AC079145.1 0.51207253 0.530167948 13.64426772 3.69E-34 3.09E-33 65.81071293

AC005224.3 0.583407111 0.56106508 13.63926399 3.86E-34 3.23E-33 65.76635515

ZFPM2-AS1 1.083016779 0.79100906 13.63693391 3.94E-34 3.29E-33 65.74570085

AC004990.1 0.674842476 0.355954884 13.62407568 4.42E-34 3.69E-33 65.63174154

AC010519.1 0.545653801 0.322854324 13.61884346 4.63E-34 3.87E-33 65.58537883

AC018362.2 -0.709035375 0.344211933 -13.60895726 5.05E-34 4.22E-33 65.49779159

AL122008.3 -0.908776997 0.809104387 -13.60660298 5.16E-34 4.30E-33 65.47693651

RHPN1-AS1 0.549599957 0.809166919 13.60065308 5.44E-34 4.53E-33 65.42423499

LINC01232 0.690715471 0.993071771 13.59337033 5.81E-34 4.83E-33 65.35973708

AC090241.3 -0.172968115 0.17266481 -13.5833941 6.34E-34 5.28E-33 65.27140181

AL162424.1 0.592352211 0.428574606 13.57625348 6.76E-34 5.62E-33 65.2081866

LINC01864 -0.201958165 0.116940058 -13.57012655 7.14E-34 5.93E-33 65.15395344

LINC00944 0.484907774 0.332701304 13.56402857 7.53E-34 6.26E-33 65.09998386

AL136368.1 -0.751753153 1.167149364 -13.55737601 7.99E-34 6.64E-33 65.04111439

LINC02038 1.003815261 1.079505058 13.55657529 8.05E-34 6.68E-33 65.03402931

AC090260.1 -0.192666505 0.244988669 -13.55583613 8.10E-34 6.72E-33 65.027489

AC009237.15 0.816020914 1.279076829 13.5535242 8.27E-34 6.85E-33 65.00703309

FOXP1-IT1 -0.097210246 0.075719628 -13.55015119 8.52E-34 7.06E-33 64.9771907

RAD51-AS1 -0.852558785 3.176799895 -13.54788132 8.69E-34 7.20E-33 64.95710956

AC099560.1 0.319938205 0.168681993 13.535081 9.74E-34 8.06E-33 64.84388613

AC026369.3 0.65807468 0.384165772 13.53095801 1.01E-33 8.35E-33 64.80742371

AC090907.2 0.189303987 0.108069136 13.52640312 1.05E-33 8.69E-33 64.76714569

LINC01117 0.433148221 0.304655449 13.5258681 1.06E-33 8.73E-33 64.76241483

AC008686.1 -0.844746479 0.445929088 -13.52360606 1.08E-33 8.90E-33 64.74241384

LINC02561 0.281651623 0.170458475 13.51952889 1.12E-33 9.22E-33 64.70636585

FAM182B -0.509276551 0.514745206 -13.51919218 1.12E-33 9.24E-33 64.70338906

AL590440.1 -0.66931978 0.324931397 -13.51899174 1.12E-33 9.26E-33 64.70161696

AC005606.1 -0.769188369 0.373414113 -13.51806889 1.13E-33 9.33E-33 64.69345826

AC053527.2 0.505866437 0.510882245 13.51789179 1.13E-33 9.34E-33 64.69189253

AL591895.1 1.316721207 5.030081529 13.50396706 1.28E-33 1.06E-32 64.56880783

AC092620.2 -0.096288049 0.068523161 -13.49794747 1.35E-33 1.11E-32 64.51561094

LINC01679 0.54170195 0.542317855 13.49097103 1.44E-33 1.18E-32 64.45396713

AC010320.3 0.395038402 0.300078238 13.49051258 1.45E-33 1.19E-32 64.44991667

AQP4-AS1 -0.250202657 0.186040221 -13.48476621 1.52E-33 1.25E-32 64.39914959

AC009005.1 0.827412283 1.280819718 13.47974738 1.59E-33 1.30E-32 64.35481555

LINC02289 -0.28110223 0.35511286 -13.47628272 1.64E-33 1.34E-32 64.32421325

AC009090.3 -0.770841786 1.359475574 -13.47322968 1.68E-33 1.38E-32 64.29724868

AC068305.2 0.808575044 0.546794765 13.470837 1.72E-33 1.41E-32 64.27611778

AL133215.2 0.574534901 0.473912065 13.46758338 1.77E-33 1.45E-32 64.24738533

LINC02004 0.640972583 0.437026711 13.46335461 1.84E-33 1.50E-32 64.21004457

AC092115.3 0.466814647 0.286140189 13.45303911 2.01E-33 1.65E-32 64.11897218

AC139887.1 0.781571901 0.84479088 13.45148357 2.04E-33 1.67E-32 64.10524068

AC023509.2 -0.161553745 0.099666612 -13.44078776 2.25E-33 1.83E-32 64.01083682

LINC00960 -1.120884454 1.720360408 -13.43101458 2.45E-33 2.00E-32 63.92459659

LINC01786 -0.770736123 1.592448006 -13.42798403 2.51E-33 2.05E-32 63.89785847

AC093752.2 -0.410110041 0.368042599 -13.4273612 2.53E-33 2.06E-32 63.89236351

DNAJC9-AS1 -0.489098002 0.398756096 -13.42351781 2.62E-33 2.13E-32 63.85845707

AL355803.1 0.787001747 0.589091556 13.42167649 2.66E-33 2.16E-32 63.84221389

POLH-AS1 0.362590195 0.547991376 13.42081996 2.68E-33 2.18E-32 63.83465826

AL355388.1 0.928675952 0.495790456 13.41678183 2.78E-33 2.26E-32 63.79903927

AC068473.4 0.473324018 0.285659493 13.40399054 3.11E-33 2.53E-32 63.68623339

AL355001.2 0.780345465 2.046533264 13.40097873 3.19E-33 2.59E-32 63.65967723

TFAP2A-AS1 0.589955408 0.464979721 13.40093711 3.19E-33 2.59E-32 63.65931032

AC018521.2 -0.970491172 0.471139602 -13.39907282 3.25E-33 2.63E-32 63.64287322

AC012435.3 0.339855048 0.255985834 13.39328175 3.42E-33 2.77E-32 63.59181886

ATP1B3-AS1 0.799344582 0.798639025 13.39031326 3.51E-33 2.84E-32 63.56565125

LUNAR1 -0.166682831 0.168487908 -13.3863342 3.63E-33 2.94E-32 63.53057813

BX284613.2 0.547397781 0.311151963 13.3815335 3.79E-33 3.07E-32 63.48826706

AC239802.2 -0.321606961 0.178766764 -13.38147969 3.79E-33 3.07E-32 63.4877928

TTC39C-AS1 0.456532129 0.26009176 13.3807327 3.82E-33 3.09E-32 63.48120963

LINC00443 1.708382892 0.896080112 13.38011596 3.84E-33 3.10E-32 63.47577449

AP001596.1 -0.231356642 0.19750015 -13.36770538 4.29E-33 3.46E-32 63.3664193

AC011472.1 0.859963799 1.490512671 13.3661635 4.34E-33 3.51E-32 63.35283536

PSMB8-AS1 0.906698671 3.261831328 13.36129123 4.54E-33 3.66E-32 63.30991393

AC107375.1 0.588814597 1.579729911 13.35982586 4.59E-33 3.70E-32 63.29700592

AL136988.2 -0.43095848 0.603673201 -13.35818662 4.66E-33 3.76E-32 63.28256694

AL162231.2 0.731543203 1.664938704 13.3485543 5.08E-33 4.09E-32 63.19773327

AL355102.4 -1.291894872 1.954189032 -13.34097113 5.43E-33 4.37E-32 63.13096056

AC023043.1 0.936775215 1.713723708 13.3302665 5.96E-33 4.80E-32 63.03672258

AC009065.6 -0.517376519 0.441742532 -13.32941767 6.01E-33 4.83E-32 63.02925096

AL109947.1 0.335474971 0.192207901 13.32517373 6.24E-33 5.01E-32 62.99189687

URB1-AS1 0.754431712 3.040346523 13.32185646 6.42E-33 5.16E-32 62.96270174

LINC-PINT -0.829617433 2.491427928 -13.31907744 6.58E-33 5.28E-32 62.93824557

AC091230.1 -0.100152938 0.059027218 -13.31535357 6.80E-33 5.46E-32 62.90547688

PACRG-AS1 -0.126354539 0.117302772 -13.30645361 7.36E-33 5.90E-32 62.82717237

AC006333.2 0.558062689 2.082359368 13.30550002 7.42E-33 5.95E-32 62.81878343

AC011899.2 0.5712195 0.737064095 13.30506479 7.45E-33 5.97E-32 62.81495469

LMCD1-AS1 -0.25548524 0.368555183 -13.30276576 7.60E-33 6.08E-32 62.79473052

LINC00964 -0.442858863 0.305477343 -13.30144756 7.69E-33 6.15E-32 62.78313505

AC139149.1 -0.607603471 0.969499816 -13.30125996 7.71E-33 6.16E-32 62.78148482

AC027682.4 -0.462511143 0.67520581 -13.29735795 7.98E-33 6.37E-32 62.74716337

LINC01096 0.436846871 0.31883536 13.2958985 8.08E-33 6.45E-32 62.73432706

HOXB-AS3 1.018000971 1.423793472 13.29208109 8.36E-33 6.67E-32 62.700754

AC244517.7 0.68336741 0.599682174 13.28979668 8.53E-33 6.80E-32 62.68066466

AC005695.1 -0.089346613 0.070431385 -13.28895363 8.59E-33 6.85E-32 62.67325111

AL138921.1 0.339669803 0.356634121 13.28754533 8.70E-33 6.93E-32 62.66086724

TMEM202-AS1 -0.262719611 0.643082074 -13.2808055 9.23E-33 7.35E-32 62.6016062

LINC01686 0.419851452 0.403040987 13.27649201 9.59E-33 7.63E-32 62.56368423

AL031123.1 0.427317275 0.331146745 13.27289042 9.90E-33 7.87E-32 62.53202399

AC063949.2 -0.118145441 0.057355483 -13.27242003 9.94E-33 7.90E-32 62.52788917

JMJD1C-AS1 0.546307518 0.804965809 13.27232985 9.95E-33 7.90E-32 62.52709645

AC092598.1 0.164387013 0.092955513 13.27224945 9.95E-33 7.90E-32 62.52638972

AC092111.2 -0.225623827 0.269349873 -13.26945582 1.02E-32 8.09E-32 62.50183422

LINC02084 0.60725407 0.536567991 13.25704836 1.14E-32 9.02E-32 62.39279488

DLG3-AS1 -0.712463239 0.945482183 -13.24297316 1.29E-32 1.02E-31 62.26913898

AC025154.2 1.244808817 1.31336826 13.2378076 1.35E-32 1.07E-31 62.2237682

GRIK1-AS1 -0.529819449 0.606496794 -13.23719189 1.36E-32 1.07E-31 62.21836057

HNF1A-AS1 -1.257175116 2.840447609 -13.2359945 1.37E-32 1.08E-31 62.20784454

AC010327.6 -0.442317022 1.200644511 -13.2284114 1.46E-32 1.16E-31 62.14125306

LINC02809 -0.100725882 0.087763879 -13.22641291 1.49E-32 1.18E-31 62.12370523

AC116407.2 0.820818608 1.164319792 13.21174821 1.70E-32 1.34E-31 61.99496769

AC120498.9 -0.133543323 0.166233116 -13.21085128 1.71E-32 1.35E-31 61.98709532

AP001107.5 0.58911576 0.482873645 13.20983774 1.72E-32 1.36E-31 61.97819959

AL132780.1 -0.505174384 1.088082806 -13.20624515 1.78E-32 1.40E-31 61.94666973

LINC00266-1 -0.300937421 0.199564472 -13.20562777 1.79E-32 1.41E-31 61.9412517

AL592494.2 0.791559499 0.911447672 13.20258702 1.84E-32 1.45E-31 61.91456758

LINC01467 -0.406806009 0.201517807 -13.18466901 2.15E-32 1.69E-31 61.75736872

AC083805.3 -0.18406255 0.118281242 -13.17989467 2.24E-32 1.77E-31 61.71549404

AC006237.1 0.918422815 0.580776947 13.17873205 2.27E-32 1.78E-31 61.70529769

LINC02391 0.311885794 0.22681704 13.17674135 2.31E-32 1.81E-31 61.68783973

AC023794.2 0.371924164 0.266850156 13.17519977 2.34E-32 1.84E-31 61.67432101

AC027288.3 0.529379249 0.421148411 13.16288212 2.61E-32 2.05E-31 61.56632129

ITIH4-AS1 -0.587089319 0.430573827 -13.16090104 2.65E-32 2.08E-31 61.54895454

AC037459.2 -0.51700492 1.906070271 -13.15966782 2.68E-32 2.10E-31 61.53814418

AC018755.1 -0.182643403 0.153951875 -13.15862005 2.71E-32 2.12E-31 61.5289597

AL157938.3 -0.108715538 0.118348135 -13.14747439 2.99E-32 2.34E-31 61.43127491

C21orf62-AS1 -0.426226741 0.774487567 -13.14565535 3.03E-32 2.37E-31 61.41533472

AL080250.1 -0.433947898 0.707824145 -13.14220698 3.13E-32 2.45E-31 61.3851188

AC107072.2 0.407877248 0.315058491 13.12985148 3.49E-32 2.73E-31 61.27687665

AC021092.1 -0.352311445 0.287208649 -13.12893373 3.51E-32 2.75E-31 61.26883791

AL133520.1 0.615613465 1.182149738 13.1268556 3.58E-32 2.80E-31 61.25063591

AC107214.1 0.423236248 0.48297966 13.12360244 3.68E-32 2.87E-31 61.22214387

AC062028.1 -0.288605282 0.271715907 -13.12228188 3.73E-32 2.91E-31 61.21057872

NOP14-AS1 -0.608599518 2.740338406 -13.12040268 3.79E-32 2.95E-31 61.19412181

AC016747.3 -0.392136931 0.682804745 -13.11855562 3.85E-32 3.00E-31 61.17794714

AC017002.3 0.450399544 0.444695387 13.1152848 3.96E-32 3.09E-31 61.1493064

AC241644.3 -0.539282552 0.26180286 -13.10960622 4.16E-32 3.24E-31 61.09958802

AP001458.1 0.617202442 0.318467922 13.10772021 4.23E-32 3.29E-31 61.08307677

AC110285.2 -1.194784071 2.401274731 -13.10613788 4.29E-32 3.34E-31 61.0692247

AC093388.1 0.645284192 0.772268586 13.10357063 4.39E-32 3.41E-31 61.0467516

AL590004.3 0.844541957 0.771638449 13.10217097 4.44E-32 3.45E-31 61.03449996

AC007613.1 -0.136071743 0.191683153 -13.10210681 4.45E-32 3.45E-31 61.03393834

AP000320.1 -0.472768304 0.346230381 -13.09733187 4.64E-32 3.60E-31 60.9921452

AC012557.2 -0.601304579 0.688627369 -13.08315345 5.25E-32 4.07E-31 60.86807692

AC061975.7 -0.367043325 0.363632625 -13.08076555 5.36E-32 4.16E-31 60.84718599

DNMBP-AS1 0.650320317 0.741129741 13.07206307 5.79E-32 4.49E-31 60.77106178

AC008555.1 0.314404169 0.364879187 13.06569276 6.12E-32 4.74E-31 60.71534883

AL049552.1 0.639283361 0.801757705 13.06404992 6.21E-32 4.81E-31 60.70098241

AC011467.1 -0.134866151 0.091755201 -13.05679857 6.62E-32 5.12E-31 60.63757787

KANSL1-AS1 -1.251441255 2.769692966 -13.05551509 6.69E-32 5.17E-31 60.62635658

AL023653.1 0.601128555 0.382872656 13.04918455 7.08E-32 5.47E-31 60.5710149

AC090236.2 -0.331957258 0.289641391 -13.04849579 7.12E-32 5.50E-31 60.56499427

ITGB2-AS1 1.110636986 1.534771457 13.04843915 7.12E-32 5.50E-31 60.56449915

AL683807.1 0.709387349 0.541117939 13.04217052 7.52E-32 5.80E-31 60.50970862

AC104117.3 0.47268063 0.652458259 13.04091658 7.61E-32 5.86E-31 60.49874973

AC022098.1 -0.361274849 1.343433973 -13.04021666 7.65E-32 5.90E-31 60.4926329

AC004477.3 0.527336928 0.737422874 13.03856602 7.77E-32 5.98E-31 60.47820778

AC110597.3 -0.443603414 0.215353975 -13.03723791 7.86E-32 6.05E-31 60.46660173

LINC01933 -0.383585935 0.347029824 -13.02954328 8.41E-32 6.47E-31 60.39936804

AL391001.1 -0.861275839 1.549294091 -13.02657737 8.63E-32 6.63E-31 60.3734563

Z98885.2 -0.189583684 0.168147217 -13.02027199 9.12E-32 7.01E-31 60.31837573

AC007773.1 0.545618159 0.621528547 12.99399897 1.15E-31 8.81E-31 60.08896533

LINC02332 0.54009715 0.294906511 12.98751361 1.21E-31 9.32E-31 60.03236076

AC012236.1 0.860116113 1.104101352 12.98653053 1.23E-31 9.40E-31 60.02378128

AC097534.1 0.386015879 0.227392063 12.98627364 1.23E-31 9.42E-31 60.02153941

AC124242.1 -0.42854908 0.599167055 -12.98050075 1.29E-31 9.90E-31 59.97116298

MIR3142HG 0.799047256 0.690257643 12.97006609 1.42E-31 1.08E-30 59.88012569

AC131009.1 0.519472808 0.442713505 12.95611117 1.60E-31 1.22E-30 59.75841501

ODC1-DT 0.418729879 0.799897098 12.95548026 1.61E-31 1.23E-30 59.75291342

AC092755.1 0.696222476 0.359126486 12.95250181 1.65E-31 1.26E-30 59.72694253

AC096772.1 -0.878655275 0.426556478 -12.94815716 1.71E-31 1.31E-30 59.68906252

AC139887.4 -0.606688788 1.148844185 -12.94754032 1.72E-31 1.32E-30 59.68368481

AC099066.2 0.421581233 0.272692487 12.94447284 1.77E-31 1.35E-30 59.65694321

AL645568.1 -0.423844249 1.105053889 -12.9426281 1.80E-31 1.37E-30 59.64086223

FEZF1-AS1 1.269448514 0.750646268 12.93947903 1.85E-31 1.41E-30 59.61341296

HOXC-AS2 0.449895102 0.264164803 12.93376297 1.94E-31 1.48E-30 59.56359406

AL132639.3 0.387050789 0.383441641 12.92287077 2.14E-31 1.63E-30 59.46868303

CNTN4-AS1 -0.145608507 0.095067974 -12.92150479 2.16E-31 1.65E-30 59.45678226

FOXN3-AS1 0.644025248 1.703784515 12.91440006 2.30E-31 1.75E-30 59.39489104

AL160153.1 0.520935681 0.328019872 12.91353621 2.32E-31 1.76E-30 59.38736666

AC026355.2 0.404171756 0.249505285 12.91203076 2.35E-31 1.79E-30 59.37425407

AL023803.2 0.689482887 0.386847699 12.91037074 2.39E-31 1.81E-30 59.35979585

LINC02197 -0.216188191 0.183482717 -12.90696321 2.46E-31 1.87E-30 59.33011927

AC090912.1 0.475789534 0.484407767 12.9063761 2.47E-31 1.87E-30 59.32500639

AP000866.5 0.623635129 0.704940346 12.90168711 2.57E-31 1.95E-30 59.28417457

AC025176.1 0.417131013 0.237216098 12.90095187 2.59E-31 1.96E-30 59.27777255

AC008758.4 -0.230444905 0.282010853 -12.8960074 2.70E-31 2.05E-30 59.23472245

AC010536.2 0.478038726 0.275777671 12.89262488 2.79E-31 2.11E-30 59.20527503

HYI-AS1 0.631115976 0.590198299 12.89035485 2.84E-31 2.15E-30 59.18551421

AC024592.2 0.787938846 0.683087495 12.886756 2.93E-31 2.22E-30 59.15418838

AL035409.1 0.49692412 0.269262518 12.88670662 2.93E-31 2.22E-30 59.15375861

AC141002.1 0.66804344 0.609528679 12.88540165 2.97E-31 2.24E-30 59.14240035

LINC00398 0.344200738 0.282465546 12.88347717 3.02E-31 2.28E-30 59.12565076

AC005392.2 1.046461405 0.60084051 12.88255724 3.04E-31 2.30E-30 59.11764456

AC092375.2 -0.489208836 0.770146292 -12.87695225 3.19E-31 2.41E-30 59.06886806

LINC01775 0.278588626 0.163498198 12.87488131 3.25E-31 2.45E-30 59.05084794

AC005841.1 0.536809716 0.27710306 12.87104192 3.36E-31 2.53E-30 59.0174424

AC118344.1 -0.395404201 0.684847889 -12.87069051 3.37E-31 2.54E-30 59.01438503

LINC01355 -0.705321375 2.066745696 -12.86498198 3.55E-31 2.67E-30 58.96472347

ZBTB11-AS1 0.390266054 1.266546948 12.86331048 3.60E-31 2.71E-30 58.95018371

AL606760.2 -0.840647461 0.939428575 -12.85673985 3.81E-31 2.87E-30 58.89303444

AL161772.1 -0.390553566 0.57019334 -12.84894378 4.08E-31 3.07E-30 58.82523988

AC083967.1 1.028220749 0.53639824 12.84841808 4.10E-31 3.08E-30 58.82066885

RNF213-AS1 -0.524772081 0.990395898 -12.84200775 4.33E-31 3.25E-30 58.76493631

ALKBH3-AS1 -0.331914928 0.430612733 -12.83931199 4.44E-31 3.33E-30 58.74150172

AC005476.2 0.647353821 1.100314172 12.83730005 4.51E-31 3.39E-30 58.72401286

AL022067.1 0.514482185 0.386321581 12.83714884 4.52E-31 3.39E-30 58.72269847

AC025171.5 0.562504953 0.560765376 12.83533259 4.59E-31 3.44E-30 58.70691155

KIAA1614-AS1 -0.249032603 0.220278877 -12.83414438 4.64E-31 3.47E-30 58.696584

AC092336.1 -0.762295978 0.524656344 -12.83394775 4.65E-31 3.48E-30 58.69487495

LIFR-AS1 -0.436568439 0.72095682 -12.83181042 4.74E-31 3.54E-30 58.67629884

AL353804.1 0.617430065 0.884901714 12.83125497 4.76E-31 3.56E-30 58.67147152

AC007390.2 -0.296529418 0.796577167 -12.83012169 4.81E-31 3.59E-30 58.66162246

AC093849.1 -0.112005652 0.094078458 -12.82953659 4.83E-31 3.61E-30 58.65653765

AL109659.2 -0.453039632 0.822092771 -12.82594641 4.98E-31 3.72E-30 58.62533881

AC005753.1 0.603715073 0.638545893 12.82496444 5.03E-31 3.75E-30 58.61680602

AC112220.2 0.560887021 1.550484356 12.82290076 5.12E-31 3.82E-30 58.59887442

AL513365.2 0.407193166 0.401578294 12.81972509 5.26E-31 3.92E-30 58.57128254

AC104806.2 -0.387427815 0.29894647 -12.8087998 5.79E-31 4.31E-30 58.47637645

SPATA41 -0.396117477 0.680537821 -12.78592222 7.06E-31 5.26E-30 58.27773549

AC006213.3 0.420215062 0.385792372 12.78475873 7.14E-31 5.31E-30 58.2676365

AP001107.8 -0.611130128 0.72787111 -12.77905516 7.50E-31 5.58E-30 58.21813478

AL596244.1 -0.61807504 1.597444964 -12.77568825 7.72E-31 5.74E-30 58.18891684

C8orf31 1.272681835 1.375316205 12.77453043 7.80E-31 5.80E-30 58.17886992

AC012358.1 -0.350716045 0.501981477 -12.75192156 9.50E-31 7.05E-30 57.98274781

AL365330.1 -0.668569003 2.320795188 -12.75145858 9.54E-31 7.08E-30 57.97873288

LINC02115 0.300267799 0.15989716 12.75114726 9.56E-31 7.09E-30 57.97603322

AC106795.5 -0.563149788 0.409969869 -12.74836813 9.80E-31 7.26E-30 57.95193456

AL603910.1 0.534454628 0.485303524 12.74677997 9.93E-31 7.36E-30 57.93816396

AL928654.1 0.435208102 0.83219767 12.74250949 1.03E-30 7.63E-30 57.90113863

AC008083.2 0.594435091 0.322774807 12.73581645 1.09E-30 8.09E-30 57.84311845

TSPEAR-AS2 -0.621955078 0.689736053 -12.73370435 1.11E-30 8.23E-30 57.82481147

AL449403.1 -0.217387342 0.174733825 -12.72755988 1.17E-30 8.68E-30 57.77155945

LINC02057 0.613292036 0.388225165 12.72731302 1.18E-30 8.69E-30 57.76942022

LINC01081 0.367545643 0.220205633 12.72413148 1.21E-30 8.93E-30 57.74185067

AC007384.1 0.720975237 0.537153362 12.72283381 1.22E-30 9.03E-30 57.7306065

LINC02640 -0.673230381 0.635020269 -12.72212991 1.23E-30 9.08E-30 57.72450747

AC022613.2 0.300090031 0.515889173 12.71954709 1.26E-30 9.28E-30 57.70212914

AL445931.1 -0.211388873 0.294561396 -12.71691872 1.29E-30 9.49E-30 57.67935799

LINC00665 -1.005873301 2.429483715 -12.71366841 1.32E-30 9.76E-30 57.6512008

AP001372.2 0.619102762 2.324518406 12.70242103 1.46E-30 1.08E-29 57.55378569

AL162311.3 -0.147522973 0.262026571 -12.69661048 1.54E-30 1.13E-29 57.50347184

LINC02604 -0.855187083 2.922752674 -12.69572065 1.55E-30 1.14E-29 57.49576747

AC090515.2 0.386196092 0.646220012 12.69083713 1.62E-30 1.19E-29 57.45348832

AC010503.4 1.012553324 4.984918525 12.68424799 1.71E-30 1.26E-29 57.39645202

LINC00837 -0.099038569 0.05335097 -12.68223209 1.74E-30 1.28E-29 57.37900434

AC008945.1 -0.456128358 0.221434399 -12.66472435 2.03E-30 1.49E-29 57.22751618

AC010976.2 -0.884167888 1.422771188 -12.66449216 2.03E-30 1.49E-29 57.22550767

AC107959.3 0.912792783 0.938260223 12.66283385 2.06E-30 1.51E-29 57.21116294

AL355802.3 -0.683116429 1.103775865 -12.65648777 2.18E-30 1.60E-29 57.15627433

AC074011.1 0.307185815 0.220362469 12.65458051 2.21E-30 1.62E-29 57.13977997

AC018926.1 -0.51781743 0.949210277 -12.65392776 2.23E-30 1.63E-29 57.13413501

AC004830.2 -0.552126956 0.736772156 -12.65007979 2.30E-30 1.68E-29 57.10086039

AL050341.2 0.603347699 2.779686538 12.64807091 2.34E-30 1.71E-29 57.08349044

AC025171.3 0.388251316 0.246558259 12.64177626 2.47E-30 1.81E-29 57.02906962

AL080317.2 -0.30842454 0.742853718 -12.63262646 2.68E-30 1.96E-29 56.94998211

TBC1D8-AS1 0.331990565 0.701353463 12.62964586 2.75E-30 2.01E-29 56.92422337

AC118754.1 0.465526006 0.356865694 12.6272639 2.81E-30 2.05E-29 56.90363976

FAM83A-AS1 1.204182669 0.669728183 12.62632552 2.83E-30 2.06E-29 56.89553119

AC132938.1 -0.91051554 1.112990117 -12.62397675 2.89E-30 2.10E-29 56.87523635

AC078795.3 -0.384620451 0.186719805 -12.62386357 2.89E-30 2.11E-29 56.87425844

AC009171.2 0.556327759 0.523347631 12.6125764 3.19E-30 2.32E-29 56.77674974

AC021739.5 0.485761449 0.454824755 12.61212871 3.20E-30 2.33E-29 56.77288282

AC002553.2 -0.82096043 1.888701865 -12.61145449 3.22E-30 2.34E-29 56.76705935

AL121821.2 0.373185697 0.26762961 12.61141764 3.22E-30 2.34E-29 56.7667411

B4GALT1-AS1 0.640100247 1.436078509 12.61059189 3.24E-30 2.36E-29 56.75960906

AC245060.5 -0.468925428 0.983743508 -12.60835427 3.30E-30 2.40E-29 56.74028334

AC007014.2 0.496305674 0.379106116 12.60807927 3.31E-30 2.40E-29 56.73790831

AC012317.1 0.795509431 0.448806509 12.60802961 3.31E-30 2.40E-29 56.73747946

AL357673.2 -0.086254486 0.064292188 -12.60134646 3.51E-30 2.55E-29 56.67976712

AL391845.2 0.558824796 0.344305397 12.60022286 3.55E-30 2.57E-29 56.6700653

AC007952.6 -0.159948253 0.119320628 -12.59850025 3.60E-30 2.61E-29 56.655192

AC104699.1 0.625146511 0.333714972 12.59633143 3.67E-30 2.66E-29 56.63646709

AC100803.3 0.608323491 0.860628054 12.59046382 3.86E-30 2.79E-29 56.58581404

AP005432.2 0.266164714 0.182892882 12.58526345 4.04E-30 2.92E-29 56.54092819

LINC02739 0.267718537 0.223599579 12.58498503 4.05E-30 2.93E-29 56.53852526

HOXC-AS1 0.787998236 0.552355267 12.57899361 4.26E-30 3.08E-29 56.48682053

LINC01233 -0.351001175 0.216287671 -12.57268631 4.50E-30 3.25E-29 56.43239968

AC104031.1 0.811656156 0.483116868 12.56319589 4.89E-30 3.53E-29 56.35053306

LSAMP-AS1 0.931991672 0.525346755 12.56287337 4.90E-30 3.54E-29 56.34775133

AP005264.1 0.542797188 0.301961314 12.56123339 4.97E-30 3.58E-29 56.33360691

AC015819.1 0.706078246 0.601722672 12.56079705 4.99E-30 3.60E-29 56.32984365

AL354893.2 -0.460338154 0.47000716 -12.5606643 5.00E-30 3.60E-29 56.32869878

AC016877.3 0.491302491 0.351076843 12.56052213 5.00E-30 3.60E-29 56.32747269

AC008543.4 0.318752613 0.232071381 12.55793 5.11E-30 3.68E-29 56.30511809

AL357568.2 -0.129218083 0.075504772 -12.55539665 5.23E-30 3.76E-29 56.28327204

AC005828.5 0.439197405 0.314799935 12.55453179 5.27E-30 3.79E-29 56.27581435

LINC00525 0.403280355 0.248868359 12.5504421 5.46E-30 3.92E-29 56.24055176

AC092295.2 0.434218412 0.978915822 12.5484325 5.55E-30 3.99E-29 56.22322595

AC100861.1 0.731444086 1.431485671 12.54279292 5.83E-30 4.19E-29 56.17460949

AL133355.1 0.560208941 1.986793797 12.54046609 5.95E-30 4.27E-29 56.15455329

AL049840.4 -0.647884605 2.469820521 -12.53923509 6.01E-30 4.31E-29 56.14394319

AL391807.1 -0.528554595 0.480026804 -12.53879564 6.04E-30 4.33E-29 56.14015558

AC007406.5 0.620400276 2.249571821 12.53796763 6.08E-30 4.36E-29 56.13301919

AC011939.3 -0.471784055 0.229034694 -12.53136344 6.44E-30 4.61E-29 56.07610577

AC087500.1 -0.534650294 1.452385107 -12.52764754 6.65E-30 4.76E-29 56.04408786

AC004846.1 0.454767204 0.542493911 12.52242845 6.95E-30 4.97E-29 55.99912378

ZNF667-AS1 -0.911087041 2.866667055 -12.52150554 7.01E-30 5.01E-29 55.99117326

AC135012.2 0.211727009 0.115229526 12.52047191 7.07E-30 5.05E-29 55.98226931

AC087277.2 0.501822608 0.259100972 12.52032162 7.08E-30 5.06E-29 55.98097472

AC091180.4 0.393857717 0.232697049 12.51161189 7.64E-30 5.45E-29 55.90595789

AC026471.1 0.406729474 1.846706856 12.51159182 7.64E-30 5.45E-29 55.905785

AC004231.1 0.556816291 0.318646106 12.51023431 7.73E-30 5.51E-29 55.89409457

CCDC39 -0.409339937 1.029980905 -12.51009739 7.74E-30 5.51E-29 55.89291543

WWTR1-AS1 -0.313308173 0.439962604 -12.50742492 7.92E-30 5.64E-29 55.86990246

AL031772.1 -0.231363918 0.168420711 -12.50306805 8.22E-30 5.85E-29 55.83238871

AL135925.1 -0.443122227 1.478246334 -12.50188861 8.30E-30 5.91E-29 55.82223426

AC006270.1 0.336572763 0.177102978 12.4850349 9.61E-30 6.83E-29 55.67717089

AL121917.1 -0.493547758 0.887498971 -12.48363187 9.72E-30 6.91E-29 55.66509808

AC109347.1 0.333703363 0.644467983 12.46555554 1.14E-29 8.08E-29 55.50959947

AC010834.3 0.522648878 1.238441377 12.46403545 1.15E-29 8.18E-29 55.49652699

AC005609.3 -0.266857764 0.129550128 -12.45392697 1.26E-29 8.92E-29 55.40961141

AC136475.4 -0.707911098 0.400911742 -12.45391888 1.26E-29 8.92E-29 55.40954186

LINC02518 -0.613138409 0.52739406 -12.44607229 1.34E-29 9.54E-29 55.34209297

UBE2Q1-AS1 0.572176657 0.606550299 12.44596525 1.35E-29 9.54E-29 55.34117295

AC005786.3 -1.189330262 0.577378347 -12.44146233 1.40E-29 9.91E-29 55.30247344

AC005479.2 0.668039811 0.673165015 12.43981908 1.42E-29 1.00E-28 55.28835216

AC013565.1 0.341689652 0.215028768 12.43826665 1.44E-29 1.02E-28 55.27501202

AL138689.1 0.641591262 0.534344629 12.43760128 1.45E-29 1.02E-28 55.26929463

AC133644.2 0.708904387 0.507643425 12.43664736 1.46E-29 1.03E-28 55.26109799

AP002340.1 0.392848008 0.278119213 12.43623003 1.46E-29 1.03E-28 55.25751212

AC105219.2 1.01015824 0.660852835 12.43229181 1.51E-29 1.07E-28 55.2236755

AP001816.1 0.843410023 3.660286058 12.42938146 1.55E-29 1.10E-28 55.19867284

IQCH-AS1 -0.468548033 1.820029396 -12.41927238 1.69E-29 1.20E-28 55.11184348

AC092747.4 0.518134187 2.727805292 12.40161807 1.97E-29 1.39E-28 54.96027064

AC091564.2 -0.579531762 0.290621587 -12.39764868 2.04E-29 1.44E-28 54.92620231

AC127024.4 0.72455793 0.941907402 12.39425931 2.10E-29 1.48E-28 54.89711548

AC092164.1 -0.559389454 1.035191722 -12.39423886 2.10E-29 1.48E-28 54.89693999

AC015660.2 0.735672833 0.412161363 12.39372459 2.11E-29 1.49E-28 54.89252691

AC104653.2 0.302199373 0.195159874 12.38781444 2.22E-29 1.56E-28 54.84181535

AC073370.1 0.175943728 0.093424678 12.38527191 2.27E-29 1.60E-28 54.82000224

AC009509.4 0.588440657 0.792056868 12.38386182 2.30E-29 1.62E-28 54.8079054

AC005696.1 0.635032684 1.141664283 12.37921679 2.39E-29 1.68E-28 54.7680604

AC010834.1 -0.491049472 0.238387381 -12.3731787 2.52E-29 1.77E-28 54.71627433

AL365259.1 -0.489929102 0.315959964 -12.36293993 2.75E-29 1.93E-28 54.62848296

AC089998.1 -0.100551776 0.069277561 -12.3570508 2.90E-29 2.03E-28 54.57799977

AC006042.1 1.000866248 2.676077226 12.35072686 3.06E-29 2.14E-28 54.52379951

AC011352.1 0.551513046 0.36416308 12.34064793 3.33E-29 2.34E-28 54.43743865

LINC02454 0.43232956 0.260553537 12.34006162 3.35E-29 2.35E-28 54.43241575

LINC01220 0.487631812 0.476914898 12.33860909 3.39E-29 2.38E-28 54.41997221

AC010201.2 -0.59084323 1.302221798 -12.33498638 3.50E-29 2.45E-28 54.3889398

GCC2-AS1 0.371673567 0.50008848 12.32376006 3.86E-29 2.70E-28 54.29279656

AC020909.2 -0.415353232 0.201639498 -12.32327785 3.87E-29 2.71E-28 54.28866755

AC002553.1 -0.720796107 2.034970035 -12.32300236 3.88E-29 2.71E-28 54.28630871

LINC00313 -0.600465364 0.365366036 -12.31912058 4.01E-29 2.80E-28 54.2530734

AC011676.1 0.761509853 0.556686399 12.31227829 4.26E-29 2.97E-28 54.19450052

N4BP2L2-IT2 -0.451214405 1.236412892 -12.30822193 4.41E-29 3.07E-28 54.15978222

AC008555.4 0.77942349 1.44661226 12.30776681 4.42E-29 3.08E-28 54.15588718

AC011815.2 -0.281384231 0.359253114 -12.30469711 4.54E-29 3.17E-28 54.12961707

AC107464.1 -0.425773727 0.310463962 -12.29390219 4.98E-29 3.47E-28 54.03725553

AC000067.1 0.31198354 0.183224512 12.29217749 5.06E-29 3.52E-28 54.02250192

AP000569.1 0.275622984 0.15450478 12.27936523 5.65E-29 3.93E-28 53.91292677

LINC00664 -0.205872808 0.192882512 -12.27533351 5.84E-29 4.06E-28 53.87845526

AC010273.2 0.215522343 0.179504319 12.26899998 6.17E-29 4.29E-28 53.82431195

ITPK1-AS1 -0.07306215 0.058969824 -12.26604461 6.33E-29 4.40E-28 53.79905117

EZR-AS1 0.332550711 0.187752488 12.26457689 6.41E-29 4.45E-28 53.78650684

AC018752.1 0.671195403 0.498425525 12.26142413 6.59E-29 4.57E-28 53.75956269

AC010768.2 0.225950001 0.125654023 12.26062119 6.63E-29 4.60E-28 53.75270107

AC084757.3 0.184468829 0.160149426 12.25404252 7.02E-29 4.87E-28 53.69648864

AL031775.1 -0.523091387 1.794024705 -12.25286471 7.09E-29 4.91E-28 53.68642592

AC005256.1 1.020991592 0.575437246 12.24988532 7.27E-29 5.04E-28 53.66097292

Z84468.1 -0.21078045 0.135751038 -12.24869878 7.34E-29 5.09E-28 53.65083693

AC018529.1 0.421478707 0.32511411 12.24632014 7.50E-29 5.19E-28 53.63051869

AL356056.2 -0.374128005 0.427424572 -12.24504199 7.58E-29 5.24E-28 53.61960139

AC104825.1 -0.652603422 2.291982655 -12.24229225 7.76E-29 5.36E-28 53.59611604

AL512506.1 -0.453586743 0.516418346 -12.24228025 7.76E-29 5.36E-28 53.59601359

AC020656.1 -1.439149162 0.698656708 -12.23296536 8.41E-29 5.81E-28 53.51647126

AC027228.2 0.647745846 0.807899538 12.22337816 9.13E-29 6.30E-28 53.43462836

SHANK2-AS1 -0.103538475 0.077691351 -12.22235374 9.21E-29 6.35E-28 53.42588462

AC123768.2 -0.494222111 0.6214658 -12.22231943 9.21E-29 6.35E-28 53.42559178

AC093249.2 0.32965177 0.271962207 12.21893537 9.48E-29 6.54E-28 53.39671014

AC006213.2 -0.448005724 0.68249294 -12.21475153 9.83E-29 6.77E-28 53.36100708

AL360270.1 0.322237898 0.498015403 12.20083409 1.11E-28 7.62E-28 53.24227611

AC006116.10 -0.542961784 0.607846523 -12.19553942 1.16E-28 7.97E-28 53.19712084

LERFS -1.003224028 0.768429967 -12.18813932 1.23E-28 8.49E-28 53.13402247

AL132657.1 0.393605385 0.466956402 12.18416882 1.28E-28 8.78E-28 53.1001735

AC005387.2 0.503104749 0.619059191 12.17951592 1.33E-28 9.13E-28 53.06051252

AC009506.1 0.444503277 1.917360615 12.1750361 1.38E-28 9.48E-28 53.0223326

AC004847.1 0.571362466 0.548185248 12.16551308 1.50E-28 1.03E-27 52.9411896

SLC12A9-AS1 0.853412437 1.162541227 12.16007056 1.57E-28 1.08E-27 52.89482675

AC007611.1 0.311598827 0.376116617 12.15848279 1.59E-28 1.09E-27 52.88130261

AC004908.1 -0.653802403 1.697345468 -12.15037679 1.70E-28 1.17E-27 52.81226946

AC079630.1 -0.335500311 0.585073481 -12.1490559 1.72E-28 1.18E-27 52.80102206

AL353807.2 0.302662799 0.156626303 12.14597269 1.77E-28 1.21E-27 52.77477044

AP001505.1 0.777793012 2.426880813 12.14278056 1.82E-28 1.25E-27 52.7475942

LINC02415 -0.158342952 0.076869972 -12.1392269 1.88E-28 1.28E-27 52.71734341

AC025279.1 -0.2731101 0.379071201 -12.13846182 1.89E-28 1.29E-27 52.71083113

AC007785.1 0.938214346 0.540718179 12.13194792 2.00E-28 1.36E-27 52.65539161

AL139099.1 -0.721558859 0.350291648 -12.13058064 2.02E-28 1.38E-27 52.64375628

LINC01357 0.578680639 0.386361209 12.12894489 2.05E-28 1.40E-27 52.62983703

AC092354.2 0.598198071 0.806256868 12.12487575 2.12E-28 1.45E-27 52.59521423

AC013553.3 0.445614753 0.467645915 12.12296919 2.15E-28 1.47E-27 52.57899364

AP000696.1 0.486250388 0.254595792 12.11893686 2.23E-28 1.52E-27 52.54469081

AC026471.5 -0.352860251 0.21467224 -12.11854573 2.24E-28 1.53E-27 52.54136374

LINC01088 -0.904925853 1.115011409 -12.11488998 2.31E-28 1.57E-27 52.51026874

GASAL1 0.387422444 0.538983909 12.11409639 2.32E-28 1.58E-27 52.50351917

AC079313.1 0.409002708 0.27277297 12.1034163 2.55E-28 1.73E-27 52.41270083

SNRK-AS1 -0.307725791 0.353944282 -12.09542762 2.73E-28 1.85E-27 52.34478985

AC100861.2 -0.24964618 0.121194504 -12.08210506 3.05E-28 2.08E-27 52.23157629

ACVR2B-AS1 -0.494833694 1.164861764 -12.08052174 3.10E-28 2.10E-27 52.21812477

LINC02615 -0.660325302 1.50783596 -12.07964137 3.12E-28 2.12E-27 52.21064562

USP27X-AS1 0.518869871 1.091529512 12.07780793 3.17E-28 2.15E-27 52.19507049

AF129408.1 -0.661723968 0.321243895 -12.06798763 3.44E-28 2.34E-27 52.11166277

AL133370.1 1.387322536 0.73855729 12.06010347 3.68E-28 2.50E-27 52.04471933

PTPRG-AS1 -0.339277036 0.473686506 -12.05494525 3.85E-28 2.61E-27 52.00093109

AC096564.2 -0.087758417 0.070842002 -12.05151441 3.96E-28 2.69E-27 51.97181078

LINC02175 -0.266001566 0.499985901 -12.04504778 4.19E-28 2.84E-27 51.91693251

AC002044.1 0.41611913 0.215003544 12.04426022 4.22E-28 2.86E-27 51.91024973

AC010168.2 -0.667044485 1.567041059 -12.04084148 4.34E-28 2.94E-27 51.88124255

AC016394.2 -0.601178188 1.682845622 -12.03952903 4.39E-28 2.97E-27 51.87010766

AL355336.1 -0.144693465 0.131475369 -12.03616315 4.52E-28 3.06E-27 51.84155349

AC004888.1 0.284189884 0.154770733 12.03591624 4.53E-28 3.06E-27 51.83945897

AP001372.3 -0.605231058 0.293818558 -12.03309989 4.64E-28 3.13E-27 51.81556946

AL732437.1 -0.254097457 0.123355444 -12.03185783 4.69E-28 3.16E-27 51.80503442

AP000424.2 0.54188616 0.348786654 12.02037697 5.17E-28 3.49E-27 51.70767613

AC233992.1 -0.262635024 0.127500135 -12.01974049 5.20E-28 3.51E-27 51.70227981

PKN2-AS1 -0.246678694 0.177074938 -12.00975695 5.66E-28 3.81E-27 51.61765147

CPB2-AS1 -0.195041093 0.167927158 -12.00791605 5.75E-28 3.87E-27 51.60204968

AL356489.1 -0.201621894 0.117300585 -12.00639778 5.82E-28 3.92E-27 51.58918291

FAM99A -0.29368013 0.153199561 -12.00480708 5.90E-28 3.97E-27 51.57570308

USP46-AS1 0.474830203 1.915362101 12.00442245 5.92E-28 3.98E-27 51.57244379

AL358334.2 -0.185711352 0.199841642 -11.99538523 6.39E-28 4.30E-27 51.49587576

CASK-AS1 0.265908888 0.190681303 11.99529047 6.40E-28 4.30E-27 51.49507304

AC092755.2 0.467266934 0.308672864 11.9907892 6.65E-28 4.47E-27 51.45694487

AC089983.1 0.228633584 0.133360404 11.98185635 7.17E-28 4.82E-27 51.38129611

AC010680.3 -0.423050191 0.344720949 -11.98081351 7.24E-28 4.86E-27 51.37246621

LINC02102 -0.170564145 0.168591406 -11.97750328 7.44E-28 4.99E-27 51.34444012

AC139769.2 -0.099203895 0.082399606 -11.97602046 7.54E-28 5.06E-27 51.33188683

AC009229.2 0.258972636 0.140698391 11.97531442 7.58E-28 5.08E-27 51.32590983

MIR133A1HG -0.118319752 0.092525456 -11.96748767 8.10E-28 5.43E-27 51.25966206

AC114956.3 0.421955571 0.553924691 11.96611453 8.20E-28 5.49E-27 51.2480412

AC103925.1 -0.392002908 0.237484254 -11.96031322 8.61E-28 5.77E-27 51.1989511

AC011899.3 0.496869584 0.43416188 11.95946346 8.68E-28 5.81E-27 51.19176137

LINC01048 0.344323635 0.182606045 11.95272951 9.19E-28 6.14E-27 51.13479326

AL118558.3 0.629735718 1.891116554 11.95073328 9.34E-28 6.25E-27 51.11790807

AP005131.2 0.3516348 0.271258936 11.94682635 9.66E-28 6.45E-27 51.08486442

AC007403.1 -0.415101982 0.310162526 -11.94561059 9.76E-28 6.52E-27 51.07458277

ILF3-DT 0.746761427 4.291531882 11.94081354 1.02E-27 6.79E-27 51.03401855

AC046134.2 -0.560177812 1.492562764 -11.93847083 1.04E-27 6.92E-27 51.01421093

AL117327.1 0.541394365 0.631028773 11.9350536 1.07E-27 7.12E-27 50.98532102

AL035530.1 0.309679676 0.168824019 11.93373304 1.08E-27 7.20E-27 50.97415768

AP002761.3 0.321823306 0.544939263 11.9236125 1.18E-27 7.84E-27 50.88862059

LINC01963 0.678297611 2.010847315 11.92040955 1.21E-27 8.05E-27 50.86155608

AL445248.1 0.225872745 0.174065471 11.91898962 1.22E-27 8.14E-27 50.84955884

LINC01436 1.08073595 0.768646198 11.91420761 1.27E-27 8.48E-27 50.80915901

AF127936.2 -0.95802897 0.790504045 -11.9116644 1.30E-27 8.66E-27 50.78767599

LINC02244 0.592162625 0.357437487 11.91136609 1.31E-27 8.68E-27 50.78515624

BX537318.1 0.577306084 2.009238237 11.9097006 1.32E-27 8.80E-27 50.77108867

AC011483.1 0.449511639 0.232185154 11.90765672 1.35E-27 8.95E-27 50.75382622

SNHG15 -0.78309782 3.781849017 -11.90535445 1.37E-27 9.12E-27 50.73438278

AC108693.2 0.250315363 0.134203481 11.89094112 1.55E-27 1.03E-26 50.61269307

AL136309.2 -0.177124052 0.265556556 -11.88986677 1.57E-27 1.04E-26 50.60362497

AL162171.3 -0.146015001 0.284792063 -11.88590729 1.62E-27 1.07E-26 50.57020766

LINC01819 1.767935925 1.245738336 11.88275417 1.66E-27 1.10E-26 50.54359914

AC022154.1 -0.382777577 0.504235955 -11.88209187 1.67E-27 1.11E-26 50.53801049

AC010761.6 0.560033516 0.325042076 11.86513317 1.93E-27 1.28E-26 50.39495359

AL355581.1 -0.287641261 0.267590084 -11.86373124 1.95E-27 1.29E-26 50.38313131

AC098869.2 -0.45818547 0.717930163 -11.86280643 1.97E-27 1.30E-26 50.37533279

AC010973.2 -0.695184688 1.878567702 -11.86136292 1.99E-27 1.32E-26 50.36316083

AC104411.1 0.507534165 0.451718557 11.83977328 2.39E-27 1.58E-26 50.18118697

AC004069.1 0.492118863 0.529797858 11.83569534 2.48E-27 1.64E-26 50.14683059

AC005291.1 0.554174803 0.380594647 11.83399831 2.51E-27 1.66E-26 50.13253468

LINC02712 0.394048967 0.222115554 11.83188123 2.56E-27 1.69E-26 50.11470148

AC073896.2 -0.655848409 2.218857499 -11.82896182 2.62E-27 1.73E-26 50.090112

LINC00989 -0.197512955 0.25340646 -11.82712325 2.66E-27 1.76E-26 50.07462752

AC073115.2 0.237723141 0.138641942 11.82576564 2.70E-27 1.78E-26 50.06319429

SSBP3-AS1 -0.284061515 0.716574383 -11.81521004 2.95E-27 1.94E-26 49.97431854

AL391987.3 -0.308590899 0.155015554 -11.8135737 2.99E-27 1.97E-26 49.96054388

AC017100.1 0.597369376 0.956680932 11.80773741 3.14E-27 2.07E-26 49.91142086

AC006017.1 -0.513804149 0.755316724 -11.80636883 3.18E-27 2.09E-26 49.89990328

AL121827.2 0.622403053 0.374128642 11.79918843 3.37E-27 2.22E-26 49.83948414

AC005480.1 -0.17887471 0.142317642 -11.79032235 3.64E-27 2.39E-26 49.76490228

AL078581.4 0.229657989 0.187299945 11.78761689 3.72E-27 2.44E-26 49.74214858

AC010680.1 -0.0850544 0.07572319 -11.78593474 3.77E-27 2.48E-26 49.72800232

AC113189.4 -0.125429569 0.073189233 -11.78570735 3.78E-27 2.48E-26 49.72609007

AC087491.1 0.714314682 0.491047983 11.78536085 3.79E-27 2.49E-26 49.72317624

LINC02565 0.660410019 0.41593449 11.78009654 3.97E-27 2.60E-26 49.67891181

LINC02182 0.879218112 0.674596333 11.77882542 4.01E-27 2.63E-26 49.66822494

AL645608.1 0.300769107 0.216556056 11.7764387 4.09E-27 2.68E-26 49.64816007

AC073115.1 0.281890227 0.16481149 11.77379744 4.18E-27 2.74E-26 49.6259574

AL606834.2 0.589480518 1.090853317 11.77370696 4.18E-27 2.74E-26 49.6251968

AC113143.1 0.374272457 0.215650928 11.76512077 4.50E-27 2.94E-26 49.55303517

AL049840.3 -0.659532517 2.30843828 -11.76362544 4.56E-27 2.98E-26 49.54047016

LINC01544 -0.073874097 0.043863719 -11.76347295 4.56E-27 2.98E-26 49.53918883

LINC00310 -0.237041888 0.276591839 -11.75986386 4.70E-27 3.07E-26 49.50886512

LINC00839 0.613649782 0.64624723 11.75469244 4.91E-27 3.21E-26 49.46542157

AC114956.1 0.2962364 0.226794245 11.75227592 5.01E-27 3.27E-26 49.44512387

AL031848.2 -0.266223673 0.129242299 -11.74927225 5.14E-27 3.35E-26 49.41989692

AC010542.5 0.969234502 2.739732035 11.74890859 5.16E-27 3.36E-26 49.4168428

LINC00242 0.340508669 0.560769469 11.74267316 5.44E-27 3.54E-26 49.36448263

AC004975.2 0.589536049 0.556383207 11.74229063 5.46E-27 3.55E-26 49.36127081

KRTAP5-AS1 -0.676439152 1.175371269 -11.73277629 5.91E-27 3.85E-26 49.28140091

AC108134.2 0.504209578 0.793167313 11.71896651 6.64E-27 4.32E-26 49.16552124

AC096536.1 0.365268013 0.243130805 11.71491728 6.87E-27 4.47E-26 49.13155473

AL512408.1 0.35723327 0.649406226 11.70800143 7.28E-27 4.73E-26 49.07355343

AC107980.1 -0.073652089 0.038334819 -11.70169735 7.68E-27 4.99E-26 49.02069576

AC069222.1 -0.197712034 0.243880203 -11.69772935 7.94E-27 5.16E-26 48.98743152

AC010504.1 0.445029485 0.861411986 11.69746854 7.96E-27 5.17E-26 48.98524533

AC010149.1 0.27260416 0.142123194 11.69597567 8.06E-27 5.23E-26 48.97273182

AP001453.2 0.704945705 1.811649014 11.69156202 8.37E-27 5.42E-26 48.93573976

LNCOG 0.526453971 0.674102158 11.68837438 8.59E-27 5.57E-26 48.90902702

AL391244.1 0.567766334 0.777145686 11.68370961 8.94E-27 5.79E-26 48.86994141

C1QTNF9-AS1 -0.296170795 0.161384752 -11.68304749 8.99E-27 5.82E-26 48.86439409

AC006252.1 0.283230481 0.359398037 11.66909903 1.01E-26 6.54E-26 48.74756432

KCNMA1-AS1 -0.121641367 0.089110219 -11.66719019 1.03E-26 6.64E-26 48.7315809

LINC02584 0.252398424 0.162955744 11.66460737 1.05E-26 6.79E-26 48.70995581

AC100830.3 -0.372662795 0.18091478 -11.66126968 1.08E-26 6.98E-26 48.68201348

AC025766.1 0.365399675 0.352833315 11.65799145 1.11E-26 7.17E-26 48.65457236

AL138767.3 0.4244456 0.388748749 11.65310617 1.16E-26 7.47E-26 48.61368516

AC021683.2 -0.130924814 0.119947501 -11.65263202 1.16E-26 7.49E-26 48.60971718

AP001107.6 0.292673779 0.212109981 11.65147313 1.17E-26 7.56E-26 48.60001917

AL136982.6 -0.247262765 0.166618897 -11.65076003 1.18E-26 7.60E-26 48.59405183

ZRANB2-AS1 -0.308303817 0.312473267 -11.64599102 1.23E-26 7.91E-26 48.55414841

U62317.4 0.568249175 0.470881989 11.64539639 1.23E-26 7.95E-26 48.54917353

ZNF252P-AS1 -0.426358687 0.664841652 -11.64361659 1.25E-26 8.06E-26 48.53428369

AC078802.1 0.293222793 0.299554839 11.64262264 1.26E-26 8.13E-26 48.52596866

PITRM1-AS1 -0.267424795 0.558824845 -11.64226411 1.27E-26 8.15E-26 48.52296946

AC004067.1 0.447360434 1.147757359 11.64100008 1.28E-26 8.23E-26 48.51239565

AJ239328.1 -0.113777977 0.155828838 -11.63888985 1.30E-26 8.38E-26 48.49474438

PACERR 0.606889294 0.479157242 11.63267889 1.37E-26 8.82E-26 48.44280017

LINC01754 -0.50461174 0.485703426 -11.62207789 1.50E-26 9.64E-26 48.35416853

AC006213.4 0.440575424 0.961855676 11.62134391 1.51E-26 9.69E-26 48.34803322

LINC02345 0.430820876 0.306787968 11.62126136 1.51E-26 9.69E-26 48.34734322

LINC02593 0.731699759 1.054673237 11.61890175 1.54E-26 9.88E-26 48.32762059

AL109976.1 0.39479899 0.389524226 11.61278364 1.62E-26 1.04E-25 48.27649105

AC122129.1 -0.361581677 0.762671142 -11.60985964 1.66E-26 1.07E-25 48.25205905

AC010931.2 0.295528697 0.292006117 11.60872499 1.68E-26 1.08E-25 48.24257899

AC009095.1 -0.257409279 0.433397338 -11.60770886 1.69E-26 1.08E-25 48.23408957

AL132800.1 0.436262308 0.548685764 11.60246267 1.77E-26 1.13E-25 48.19026436

ZNF561-AS1 -0.46929856 1.728950275 -11.59689109 1.85E-26 1.19E-25 48.14373043

TMEM78 -0.091621789 0.062559905 -11.59197057 1.93E-26 1.23E-25 48.10264229

RDH10-AS1 -0.489279457 0.542002298 -11.59195077 1.93E-26 1.23E-25 48.10247696

Z99289.1 0.212996855 0.166664639 11.5895952 1.97E-26 1.26E-25 48.08280978

AL137186.1 0.258278669 0.154723537 11.5889807 1.98E-26 1.26E-25 48.07767953

LINC00638 0.399909551 0.768451921 11.58820588 1.99E-26 1.27E-25 48.07121087

AC087286.2 0.574225414 0.537485643 11.58660717 2.02E-26 1.29E-25 48.05786458

AC011498.6 -0.606012996 1.272075015 -11.58248817 2.09E-26 1.33E-25 48.02348228

AC105345.1 -0.429754422 0.857886555 -11.57993324 2.14E-26 1.36E-25 48.00215827

AC018521.7 -0.088974177 0.084615304 -11.57651137 2.20E-26 1.40E-25 47.97360187

LINC01016 -0.155704407 0.099739758 -11.57558351 2.22E-26 1.41E-25 47.96585927

AL031716.1 0.47400658 0.771874229 11.57519483 2.22E-26 1.42E-25 47.962616

LRP4-AS1 -0.340221655 0.350883614 -11.57434785 2.24E-26 1.42E-25 47.95554859

AC005034.5 0.519819969 1.315820592 11.57207004 2.28E-26 1.45E-25 47.93654328

AC069360.1 0.354653478 0.240113202 11.57057056 2.31E-26 1.47E-25 47.92403302

AC106786.1 0.522860907 0.757497108 11.56754674 2.37E-26 1.51E-25 47.89880716

AC006033.2 0.311831425 0.321644505 11.56525432 2.42E-26 1.53E-25 47.87968486

TSC22D1-AS1 -0.49987754 1.177088002 -11.56498427 2.42E-26 1.54E-25 47.87743236

AC093330.1 -0.349406294 0.192297061 -11.56409289 2.44E-26 1.55E-25 47.86999743

KF459542.1 -0.352227624 0.170994217 -11.56286574 2.47E-26 1.56E-25 47.85976226

HOMER3-AS1 0.288740244 0.26450814 11.55576904 2.62E-26 1.66E-25 47.80058079

AL162419.1 -0.23471106 0.228063597 -11.55412479 2.65E-26 1.68E-25 47.78687116

AC079807.1 0.460387494 1.417559822 11.55222503 2.70E-26 1.71E-25 47.77103227

FO393401.1 0.448728774 0.473978444 11.54934038 2.76E-26 1.75E-25 47.74698415

AC112721.1 0.528954257 0.278345029 11.54737074 2.81E-26 1.78E-25 47.73056561

AC092139.1 -0.342513113 0.166278161 -11.5446695 2.87E-26 1.82E-25 47.70805056

AC092338.1 0.359938119 0.304985166 11.53460772 3.12E-26 1.98E-25 47.62420528

SOX2-OT -0.220443835 0.216120037 -11.52146962 3.49E-26 2.20E-25 47.51477341

AC022784.5 0.433678444 0.414056426 11.51993802 3.53E-26 2.23E-25 47.5020198

AC027237.2 0.302892403 0.229400871 11.51088522 3.81E-26 2.41E-25 47.42665235

SNHG6 -0.902445565 6.911489283 -11.51058761 3.82E-26 2.41E-25 47.42417504

AC093535.1 0.271900172 0.375572406 11.50993471 3.84E-26 2.42E-25 47.41874053

AC012636.1 0.471925095 0.658508813 11.49528731 4.34E-26 2.74E-25 47.29685622

AL160314.2 -0.454280574 0.568995117 -11.49061299 4.51E-26 2.84E-25 47.25797471

AC107072.1 0.43650567 0.22549313 11.48771555 4.62E-26 2.91E-25 47.23387696

RASSF8-AS1 -0.533674592 1.830108759 -11.4861679 4.68E-26 2.95E-25 47.22100638

ZNF571-AS1 -0.450268023 0.557411597 -11.48496862 4.73E-26 2.98E-25 47.21103343

MIR3945HG 0.368643606 0.255543374 11.48314648 4.80E-26 3.02E-25 47.19588185

AL731533.2 0.630422087 0.917440413 11.47729469 5.05E-26 3.17E-25 47.14722986

AC239803.1 -0.511260834 0.414352985 -11.47697525 5.06E-26 3.18E-25 47.14457439

AC092903.2 0.380478617 0.375196212 11.47290665 5.23E-26 3.29E-25 47.11075478

AC007216.3 -0.256033564 0.488808176 -11.46996877 5.36E-26 3.37E-25 47.08633751

HCG27 -0.645121518 1.545003004 -11.46888192 5.41E-26 3.40E-25 47.07730515

AC008966.2 0.526805286 0.806693721 11.46866857 5.42E-26 3.40E-25 47.07553218

AC006141.1 -0.092170701 0.063191747 -11.46492672 5.59E-26 3.51E-25 47.04443848

SNAI3-AS1 -0.456978323 1.072743213 -11.4606652 5.80E-26 3.63E-25 47.00903197

AC025180.1 0.190086075 0.104801338 11.46041618 5.81E-26 3.64E-25 47.00696318

LINC01480 0.667368612 1.015448406 11.45936298 5.86E-26 3.67E-25 46.99821376

GHET1 0.267991357 0.401432914 11.45533784 6.06E-26 3.79E-25 46.96477833

AC093591.2 -0.102698406 0.116108618 -11.45484264 6.09E-26 3.81E-25 46.96066521

AC093732.1 0.674613157 0.424104127 11.45160739 6.25E-26 3.91E-25 46.93379546

ZNF426-DT 0.552485746 1.099327528 11.44125956 6.82E-26 4.26E-25 46.84787619

AF131215.5 -0.692217148 2.052414748 -11.43979691 6.90E-26 4.31E-25 46.83573443

LINC02019 -0.554313467 1.247429743 -11.43960638 6.91E-26 4.32E-25 46.83415287

LINC02601 0.251659319 0.174187074 11.43688024 7.07E-26 4.41E-25 46.81152462

AC124067.4 0.732206492 0.709261988 11.43624158 7.11E-26 4.44E-25 46.80622385

AC092809.2 0.451822212 0.359184369 11.43572816 7.14E-26 4.45E-25 46.80196255

AC004771.5 -0.516777476 0.765546725 -11.43143688 7.40E-26 4.61E-25 46.76634945

AC008393.1 0.461633416 1.228789969 11.42991495 7.49E-26 4.67E-25 46.75372049

ST6GAL2-IT1 0.221807999 0.115023688 11.42717565 7.67E-26 4.78E-25 46.73099167

LINC02582 -0.529838349 0.313387292 -11.42371441 7.89E-26 4.91E-25 46.70227618

AC093904.2 0.504143123 0.267041657 11.42145666 8.04E-26 5.00E-25 46.68354728

AC027801.4 0.182212029 0.09465018 11.4167201 8.36E-26 5.20E-25 46.6442612

AC007036.3 -0.345189583 0.167577493 -11.41510124 8.48E-26 5.27E-25 46.63083569

LINC01366 0.194487669 0.154001376 11.41189802 8.71E-26 5.41E-25 46.60427323

AC023825.2 0.279283138 0.171027596 11.40992958 8.85E-26 5.50E-25 46.58795183

LINC01588 0.329861788 0.488483265 11.40357147 9.33E-26 5.80E-25 46.5352418

AC068533.3 0.234138601 0.154175422 11.40178666 9.47E-26 5.88E-25 46.52044772

AL139021.2 0.265774939 0.208117041 11.39072058 1.04E-25 6.44E-25 46.42874567

AC104024.2 0.346030932 0.2897228 11.38891012 1.05E-25 6.54E-25 46.41374665

SCN1A-AS1 -0.229855365 0.144521618 -11.38795682 1.06E-25 6.59E-25 46.40584929

MAP3K20-AS1 1.097610832 0.665552171 11.38351621 1.10E-25 6.83E-25 46.36906632

AC011477.3 0.674649282 1.525433002 11.38218447 1.12E-25 6.91E-25 46.35803633

AC009118.2 -0.333447501 0.359479598 -11.38140686 1.12E-25 6.95E-25 46.3515962

AC026254.2 0.331205592 0.253608782 11.3792926 1.14E-25 7.07E-25 46.3340868

AC021188.1 0.355421583 0.367840644 11.36675161 1.27E-25 7.84E-25 46.2302582

AL135910.1 0.418890817 0.601203976 11.36074813 1.33E-25 8.24E-25 46.18057279

AC113139.1 0.549065837 0.462690744 11.34803747 1.48E-25 9.16E-25 46.07541752

AC133919.2 0.653196392 0.441483933 11.34336949 1.54E-25 9.51E-25 46.03681272

PURPL -0.520160922 0.377652695 -11.338519 1.60E-25 9.90E-25 45.9967062

AC009041.4 0.415577143 0.563844935 11.33844097 1.60E-25 9.90E-25 45.99606105

AC018755.4 1.008447711 1.032080913 11.33663592 1.63E-25 1.00E-24 45.98113793

UBE2R2-AS1 -0.692489393 0.838325691 -11.32994415 1.72E-25 1.06E-24 45.92582366

BACH1-IT2 -0.37539562 0.493544792 -11.32863847 1.74E-25 1.07E-24 45.91503263

AC024361.3 -0.617271973 0.874680838 -11.32305208 1.82E-25 1.12E-24 45.86886922

AL731684.1 -0.556118975 0.387723183 -11.32271198 1.83E-25 1.13E-24 45.86605915

AC008050.1 0.485269889 0.318037899 11.32100843 1.85E-25 1.14E-24 45.85198405

AC026369.2 0.464176496 0.6066248 11.3199455 1.87E-25 1.15E-24 45.84320237

AP001462.1 0.464110353 0.74644236 11.31646769 1.93E-25 1.18E-24 45.81447217

AC005899.6 0.814555638 1.251442268 11.31552318 1.94E-25 1.19E-24 45.80667022

ADAMTSL4-AS1 -0.363563288 0.654408406 -11.31254545 1.99E-25 1.22E-24 45.78207534

AL136141.1 0.32586834 0.226240144 11.31115807 2.01E-25 1.24E-24 45.77061716

LINC01910 0.283021595 0.169094092 11.30919828 2.05E-25 1.26E-24 45.7544325

AL590133.2 -0.637875657 0.309666372 -11.30596798 2.10E-25 1.29E-24 45.72775846

AC010754.1 0.486011877 0.345096206 11.30587145 2.10E-25 1.29E-24 45.72696136

AC124276.2 0.28235628 0.163415179 11.30548009 2.11E-25 1.29E-24 45.72372999

TPM1-AS -0.832883791 1.040504856 -11.30530016 2.11E-25 1.29E-24 45.72224432

AC010809.1 -0.433576699 1.179142207 -11.30508504 2.12E-25 1.30E-24 45.72046818

LINC02185 -0.43988454 0.499112196 -11.30066967 2.20E-25 1.34E-24 45.68401535

AC011465.1 0.395183459 0.242311463 11.29850414 2.24E-25 1.37E-24 45.66613936

LINC01126 -0.292093277 0.613913563 -11.29621681 2.28E-25 1.39E-24 45.64725961

AL034550.1 0.410704958 0.615783986 11.29601303 2.28E-25 1.40E-24 45.64557766

AL161891.1 -0.224360613 0.421179273 -11.29271551 2.35E-25 1.43E-24 45.61836304

AC004908.2 -0.653957129 1.67071415 -11.27978777 2.61E-25 1.60E-24 45.51170477

AC073254.1 -0.352863883 0.615043964 -11.27204035 2.78E-25 1.70E-24 45.44781281

AL355140.1 -0.420723483 0.204246567 -11.27136721 2.80E-25 1.71E-24 45.4422625

SNHG10 -0.668267388 2.640244718 -11.26883326 2.86E-25 1.74E-24 45.42137026

AC027031.2 0.833573056 1.996926484 11.25746392 3.14E-25 1.92E-24 45.3276575

AC123788.1 -0.214616352 0.165078951 -11.25600608 3.18E-25 1.94E-24 45.31564424

AL445228.2 -0.346352282 0.506020166 -11.2543102 3.22E-25 1.97E-24 45.30167036

AC020634.2 0.335040682 0.332258553 11.25407929 3.23E-25 1.97E-24 45.29976777

LINC01915 0.309708556 0.249840681 11.25378803 3.24E-25 1.97E-24 45.29736794

AC098848.1 0.230583514 0.126184574 11.2489844 3.37E-25 2.05E-24 45.25779267

AP001351.1 0.168171528 0.179379002 11.24638213 3.44E-25 2.09E-24 45.23635678

AC007098.1 -0.392313479 1.073577943 -11.24627044 3.45E-25 2.10E-24 45.23543684

AC092159.2 -0.137443082 0.160344993 -11.23635148 3.74E-25 2.27E-24 45.15375195

AC107294.2 0.547640929 1.062468571 11.23532189 3.77E-25 2.29E-24 45.14527494

AC099668.1 -0.566317054 0.309704089 -11.23208199 3.88E-25 2.35E-24 45.11860199

NDUFB2-AS1 0.292318054 0.826973524 11.22346708 4.16E-25 2.53E-24 45.04769574

AL513320.1 0.629933119 1.117844203 11.22021727 4.28E-25 2.59E-24 45.02095427

AC092301.1 -0.35965352 0.742655802 -11.219921 4.29E-25 2.60E-24 45.01851659

MNX1-AS2 -0.939227514 2.499698761 -11.21421465 4.49E-25 2.72E-24 44.97157034

AC082651.3 0.357835315 0.304252354 11.20624206 4.80E-25 2.91E-24 44.90599827

BBOX1-AS1 1.098691266 0.617387061 11.20175454 4.98E-25 3.02E-24 44.86909939

AC083799.1 0.654289202 3.537041812 11.19099757 5.44E-25 3.30E-24 44.7806774

AC093627.5 -0.48652484 1.007332843 -11.18696492 5.63E-25 3.41E-24 44.74753928

PHKA2-AS1 -0.366773938 0.714511508 -11.18303723 5.81E-25 3.52E-24 44.71526906

AC064836.2 -0.359586977 0.305902512 -11.17899986 6.01E-25 3.64E-24 44.68210312

AL031714.1 -0.5620086 1.759494249 -11.17818426 6.05E-25 3.66E-24 44.6754039

LINC02768 0.439707187 0.27978426 11.17562936 6.18E-25 3.73E-24 44.65441965

AL158166.2 0.321903472 0.207171239 11.16993641 6.48E-25 3.91E-24 44.60766966

AL031186.1 0.546215709 1.015531064 11.16907467 6.52E-25 3.94E-24 44.60059413

LINC02633 0.307032972 0.173758622 11.16811781 6.58E-25 3.97E-24 44.59273783

AC118758.3 -0.080029878 0.055266306 -11.16145196 6.95E-25 4.19E-24 44.53801676

AC092718.5 0.359307105 0.470220257 11.16045112 7.00E-25 4.22E-24 44.52980205

AC008972.2 0.502519006 0.919429588 11.15724335 7.19E-25 4.34E-24 44.50347548

FAM198B-AS1 -0.668021761 1.171209137 -11.15503237 7.32E-25 4.41E-24 44.48533169

LINC01556 0.566885103 0.337586422 11.15406704 7.38E-25 4.45E-24 44.47741054

AC073288.2 0.298955901 0.287957534 11.15158173 7.54E-25 4.54E-24 44.45701842

AC008781.2 -0.089081734 0.067436123 -11.15064038 7.60E-25 4.57E-24 44.44929519

KIF26B-AS1 0.292574729 0.215066879 11.15002896 7.63E-25 4.59E-24 44.44427899

AL358472.2 0.40923959 1.354507691 11.14722657 7.81E-25 4.70E-24 44.42128923

AC020658.5 0.529346707 0.495945441 11.14598021 7.89E-25 4.74E-24 44.41106543

AC011247.1 0.485061652 0.37423287 11.14561296 7.92E-25 4.76E-24 44.40805304

AC034199.1 0.167339696 0.124484797 11.14097645 8.23E-25 4.94E-24 44.37002539

ID2-AS1 -0.394224369 0.667334326 -11.13948026 8.33E-25 5.00E-24 44.35775557

Z69706.1 0.612946104 1.288826964 11.13875245 8.38E-25 5.03E-24 44.35178724

AL445471.1 0.219950995 0.176647333 11.1357235 8.59E-25 5.15E-24 44.32695085

AC009090.1 0.568798924 0.71426716 11.1340138 8.71E-25 5.22E-24 44.31293316

LINC01695 -0.217107066 0.129286205 -11.12755548 9.19E-25 5.50E-24 44.25999133

AC112503.1 -0.608178646 0.29524951 -11.12031101 9.75E-25 5.84E-24 44.20062226

AC005046.1 -0.670237372 2.248310613 -11.11944675 9.82E-25 5.88E-24 44.19354073

AC011476.2 0.269657788 0.191125366 11.11926443 9.84E-25 5.89E-24 44.19204692

AL162171.1 0.669650584 0.954409834 11.11870136 9.88E-25 5.91E-24 44.18743345

AC092849.2 -0.471694662 0.303018959 -11.11853912 9.90E-25 5.92E-24 44.18610418

LINC02416 0.325648506 0.174145157 11.11592553 1.01E-24 6.04E-24 44.16469161

AC009034.1 -0.542306216 0.374170757 -11.11104154 1.05E-24 6.29E-24 44.12468458

LINC02823 0.263031045 0.205643945 11.1102797 1.06E-24 6.32E-24 44.11844467

MIR600HG -0.568353204 1.412184144 -11.10888791 1.07E-24 6.39E-24 44.10704579

AC097641.2 -0.539307504 1.294680824 -11.10605544 1.10E-24 6.54E-24 44.08384954

AC005618.3 -0.240701399 0.116852125 -11.09300456 1.22E-24 7.28E-24 43.97700675

AL356481.1 0.359334022 1.016682695 11.09246305 1.23E-24 7.31E-24 43.97257489

AC018362.1 -0.6602298 0.320518529 -11.08966842 1.26E-24 7.48E-24 43.94970447

LINC02560 0.714967732 0.535603917 11.08469836 1.31E-24 7.79E-24 43.9090378

LINC01189 -0.084120731 0.041824744 -11.0791056 1.37E-24 8.15E-24 43.86328621

AC010547.1 -0.335833924 0.265276572 -11.07892912 1.37E-24 8.16E-24 43.86184268

AL122023.1 0.42976318 0.901135833 11.07429706 1.42E-24 8.47E-24 43.82395871

AC009087.1 0.325058693 0.249406462 11.06875479 1.49E-24 8.86E-24 43.77864023

ELFN1-AS1 0.93630328 0.513081381 11.06193872 1.58E-24 9.37E-24 43.72292078

FAM160A1-DT 0.569936256 0.641617513 11.05971018 1.61E-24 9.54E-24 43.70470662

AP003392.3 0.394928137 0.300988364 11.05660907 1.65E-24 9.78E-24 43.67936381

LINC01841 0.16924256 0.099698895 11.05169065 1.71E-24 1.02E-23 43.63917635

AC022819.1 -0.131436955 0.166976858 -11.05066162 1.73E-24 1.03E-23 43.63076948

CCNT2-AS1 -0.464575367 1.617280058 -11.05061561 1.73E-24 1.03E-23 43.63039361

AL590226.2 -0.324015545 0.328501453 -11.04901044 1.75E-24 1.04E-23 43.61728046

AC254629.1 0.652534158 0.466602943 11.0391039 1.90E-24 1.13E-23 43.53637131

AL591623.1 -0.083867628 0.063678941 -11.03677254 1.94E-24 1.15E-23 43.51733553

SERPINB9P1 0.902894351 1.476370295 11.03610306 1.95E-24 1.15E-23 43.5118695

AC083806.2 -0.10431678 0.140112161 -11.03074084 2.04E-24 1.21E-23 43.46809493

AC245452.1 -0.111952715 0.324507289 -11.02982733 2.05E-24 1.21E-23 43.46063849

SLC7A11-AS1 -0.281297263 0.232952896 -11.02800048 2.08E-24 1.23E-23 43.44572788

LINC01564 0.497689593 0.282992153 11.02770336 2.09E-24 1.23E-23 43.44330291

AL354950.2 -0.527155738 0.255915714 -11.02467556 2.14E-24 1.26E-23 43.41859317

H1FX-AS1 -0.587943047 1.358163365 -11.02288092 2.17E-24 1.28E-23 43.40394873

AC004584.3 -0.095825023 0.096800495 -11.02054958 2.21E-24 1.31E-23 43.38492637

AC009961.1 -0.374360942 0.579543407 -11.01941796 2.24E-24 1.32E-23 43.3756938

AC004906.1 0.185591609 0.114076143 11.01804423 2.26E-24 1.33E-23 43.36448644

AL137779.2 0.494495071 0.78204505 11.01346789 2.35E-24 1.38E-23 43.3271559

C5orf64-AS1 -0.161458859 0.113379846 -11.01211089 2.37E-24 1.40E-23 43.31608791

AC005180.2 0.400978549 0.277190919 11.00206116 2.58E-24 1.52E-23 43.23414026

AC008074.2 0.312936261 0.524517393 10.99981239 2.62E-24 1.55E-23 43.21580819

AL022324.3 -0.210867699 0.150799782 -10.99947182 2.63E-24 1.55E-23 43.21303197

LINC01554 -0.473207046 0.59968914 -10.99366736 2.76E-24 1.62E-23 43.16572282

AC025175.1 0.499416478 0.835621261 10.99351623 2.76E-24 1.63E-23 43.16449113

AC090186.1 -0.369338072 0.471910847 -10.99233435 2.79E-24 1.64E-23 43.15485976

AL021368.3 -0.63635977 0.653001751 -10.99128743 2.81E-24 1.65E-23 43.14632866

AP000322.2 -0.272016827 0.132054673 -10.98402799 2.99E-24 1.75E-23 43.08718359

AC002401.1 0.135734151 0.072887697 10.9826848 3.02E-24 1.77E-23 43.07624227

AC125807.2 -0.604860743 1.72091529 -10.98069925 3.07E-24 1.80E-23 43.06006951

AC007347.1 0.150370812 0.078266764 10.97405446 3.24E-24 1.90E-23 43.00595655

AC012368.2 0.249226379 0.194416674 10.97192428 3.30E-24 1.93E-23 42.98861234

AC010203.2 0.39874347 0.335114941 10.96919801 3.37E-24 1.98E-23 42.96641715

AL121894.2 0.490237641 0.833860598 10.96664235 3.44E-24 2.02E-23 42.94561327

AC245100.1 0.14177616 0.097223159 10.96327371 3.54E-24 2.07E-23 42.91819507

AC011477.2 0.525480517 2.335597769 10.96001181 3.64E-24 2.13E-23 42.89164951

LINC02829 0.415283817 0.352501278 10.9569677 3.73E-24 2.18E-23 42.86687977

AL035071.2 0.26699949 0.204074645 10.95457868 3.80E-24 2.22E-23 42.84744282

AC087164.1 -0.18550587 0.199587305 -10.95403639 3.82E-24 2.23E-23 42.84303099

AL359921.1 -0.52710492 1.395072205 -10.95339409 3.84E-24 2.24E-23 42.83780572

LINC00847 0.537889336 3.70516833 10.94723161 4.04E-24 2.36E-23 42.78768002

LINC01703 -0.575632285 1.204560299 -10.94216905 4.21E-24 2.46E-23 42.74651128

Z83843.1 -0.494796029 1.88215741 -10.93949965 4.30E-24 2.51E-23 42.72480737

COA6-AS1 0.742646573 2.056003774 10.93848615 4.34E-24 2.53E-23 42.71656769

AL021391.1 0.201396192 0.11218647 10.9360916 4.42E-24 2.58E-23 42.69710156

AC000061.1 0.654157335 0.455649305 10.93494146 4.46E-24 2.60E-23 42.68775236

AP003071.4 0.609614982 0.684348513 10.93487629 4.47E-24 2.60E-23 42.68722259

AC243964.3 -0.928336741 1.588355799 -10.92890975 4.69E-24 2.73E-23 42.63873007

ATP6V1B1-AS1 -0.144750801 0.111382133 -10.92776685 4.73E-24 2.76E-23 42.62944274

AP003774.2 0.468346118 0.338134973 10.92180268 4.97E-24 2.89E-23 42.58098469

DGUOK-AS1 0.619551489 1.600798161 10.91426799 5.29E-24 3.08E-23 42.51978467

AL353803.5 0.431469562 0.222901876 10.91179673 5.39E-24 3.14E-23 42.49971644

AL132655.2 -0.45570461 0.546850003 -10.91155892 5.40E-24 3.14E-23 42.4977854

AC009686.2 0.698035792 1.242215223 10.91097663 5.43E-24 3.16E-23 42.49305724

HHIP-AS1 -1.149571229 2.335938262 -10.91053372 5.45E-24 3.17E-23 42.48946089

AC096751.2 0.386169415 0.235153153 10.90985952 5.48E-24 3.18E-23 42.48398661

AC078889.1 0.103898823 0.054355304 10.90909382 5.51E-24 3.20E-23 42.47776965

AC097639.1 -0.795964111 2.400260156 -10.90842436 5.54E-24 3.22E-23 42.47233423

C17orf82 0.452923674 0.505032484 10.90370154 5.76E-24 3.34E-23 42.43399388

AC068870.2 0.745959169 2.564365445 10.90285939 5.80E-24 3.36E-23 42.42715797

AC000123.1 0.501828927 1.898072794 10.89266951 6.30E-24 3.65E-23 42.34446592

NFYC-AS1 -0.44060708 1.262594703 -10.89015013 6.44E-24 3.73E-23 42.32402664

AC109446.3 0.628078048 0.425304568 10.88951217 6.47E-24 3.75E-23 42.31885133

AC097468.3 0.596923663 1.934736055 10.88584377 6.67E-24 3.86E-23 42.28909518

AC137932.3 -0.345804466 0.925039235 -10.88506556 6.71E-24 3.88E-23 42.28278339

AC100774.1 -0.160495877 0.152085173 -10.88395133 6.77E-24 3.91E-23 42.27374662

AC008687.3 0.679027968 0.401677707 10.878581 7.07E-24 4.09E-23 42.23019784

AP000255.1 0.322914642 0.37788385 10.8693406 7.63E-24 4.41E-23 42.15529055

IBA57-DT 0.613511805 0.671723276 10.86807355 7.70E-24 4.45E-23 42.14502162

AC074183.1 -0.234687614 0.113932642 -10.86682215 7.78E-24 4.49E-23 42.13488013

AL606807.1 -0.347865283 0.429529639 -10.86235171 8.07E-24 4.66E-23 42.0986557

AC011468.2 -0.496459288 0.387365654 -10.84932707 8.98E-24 5.18E-23 41.99315733

LINC01550 -0.429385552 0.615365605 -10.84707381 9.14E-24 5.27E-23 41.97491241

LOXL1-AS1 -0.551899688 2.407791948 -10.84657435 9.18E-24 5.29E-23 41.97086849

AC019131.2 0.441079602 1.13910969 10.84386263 9.38E-24 5.41E-23 41.94891426

AC026803.1 0.42466245 0.327183661 10.84010423 9.68E-24 5.57E-23 41.91849059

AC023090.1 0.623692362 0.359822091 10.83780132 9.86E-24 5.68E-23 41.89985137

AC006160.1 0.240302255 0.265999746 10.83691429 9.93E-24 5.71E-23 41.89267252

AC084809.2 0.381228916 0.370429695 10.83686264 9.93E-24 5.71E-23 41.89225448

AC006449.5 0.374019987 0.68838409 10.83665681 9.95E-24 5.72E-23 41.89058874

FIRRE -0.467472691 0.66104084 -10.83472678 1.01E-23 5.81E-23 41.87496991

AC011468.3 0.301797219 0.202483143 10.82928286 1.06E-23 6.07E-23 41.83092201

LINC00337 0.314014385 0.227448029 10.82857191 1.06E-23 6.10E-23 41.82517039

AC008750.3 0.202716975 0.106604422 10.826348 1.08E-23 6.21E-23 41.80717994

AC006273.1 -0.433299425 0.59388562 -10.82335073 1.11E-23 6.36E-23 41.7829363

AL035416.1 -0.218120605 0.269945879 -10.81930062 1.15E-23 6.57E-23 41.75018181

U47924.3 0.56733744 0.77670698 10.81814094 1.16E-23 6.63E-23 41.74080427

AP000808.1 0.312425558 0.326920059 10.81612007 1.18E-23 6.74E-23 41.72446402

AP003059.2 0.271764029 0.251922509 10.81096814 1.23E-23 7.03E-23 41.68281353

AL139807.1 0.103794161 0.061590776 10.81064546 1.23E-23 7.04E-23 41.68020523

LINC01290 0.311143582 0.238492414 10.80257042 1.31E-23 7.52E-23 41.61494365

AL929472.2 -0.737824695 0.358188144 -10.80240612 1.31E-23 7.52E-23 41.613616

AC022467.1 0.27170357 0.200410339 10.80206659 1.32E-23 7.54E-23 41.6108725

AC090061.1 -0.256709763 0.353776298 -10.79719708 1.37E-23 7.84E-23 41.5715302

AC083949.1 0.342837975 0.225782018 10.79619665 1.38E-23 7.90E-23 41.5634485

AC012360.3 -0.322286062 1.15711883 -10.78987836 1.46E-23 8.32E-23 41.51241631

AC008870.2 -0.448712165 1.436547558 -10.78927554 1.46E-23 8.35E-23 41.50754817

AC022217.3 0.221338396 0.135621111 10.7823403 1.55E-23 8.83E-23 41.45155153

DGCR11 0.450289706 1.258314428 10.77996075 1.58E-23 9.00E-23 41.43234256

VIPR1-AS1 -0.342734651 0.428108704 -10.77938321 1.59E-23 9.04E-23 41.42768066

LINC02450 0.196101298 0.121069369 10.77777655 1.61E-23 9.16E-23 41.41471248

AC120498.1 -0.443094938 0.400850255 -10.77635804 1.62E-23 9.26E-23 41.40326366

AC015911.3 0.492725135 0.569998576 10.77356178 1.66E-23 9.47E-23 41.38069721

AL512422.1 0.29033609 0.166706158 10.77299916 1.67E-23 9.51E-23 41.3761571

ADPGK-AS1 -0.122190751 0.188982404 -10.77188633 1.68E-23 9.59E-23 41.36717734

AC110772.2 0.465149905 0.394144441 10.76916623 1.72E-23 9.80E-23 41.34523005

AC004687.1 0.901540849 1.066736017 10.76855246 1.73E-23 9.84E-23 41.34027815

AC092681.3 -0.224562755 0.185054064 -10.7669968 1.75E-23 9.96E-23 41.32772779

LINC02356 -0.836574206 1.179790207 -10.76134537 1.84E-23 1.04E-22 41.28214213

AC016866.1 0.371229519 0.43050043 10.75828932 1.88E-23 1.07E-22 41.2574963

AC006369.1 1.022229514 0.752236865 10.75505175 1.93E-23 1.10E-22 41.23139037

AC011510.1 0.396325519 0.264572288 10.75427211 1.94E-23 1.10E-22 41.22510435

AL354811.1 0.407052719 0.342617228 10.75280601 1.97E-23 1.12E-22 41.21328426

AC112715.1 0.504557963 0.545560198 10.75064541 2.00E-23 1.14E-22 41.19586633

AL355997.1 -0.233906431 0.216187236 -10.73905965 2.20E-23 1.25E-22 41.10249621

AL445645.1 0.522336117 0.857280271 10.73799623 2.22E-23 1.26E-22 41.09392852

AC009237.14 0.624382534 2.473191447 10.73719196 2.23E-23 1.26E-22 41.08744909

MIR222HG 0.853578177 1.84795732 10.73268468 2.32E-23 1.31E-22 41.05114133

RUNDC3A-AS1 -0.394247515 0.506764028 -10.72889848 2.39E-23 1.35E-22 41.02064801

AC008494.3 0.256136199 0.464565415 10.72729611 2.42E-23 1.37E-22 41.0077444

ASMTL-AS1 -0.956317893 3.058591242 -10.72670151 2.43E-23 1.37E-22 41.00295646

AC073517.1 0.212967077 0.151830727 10.7253471 2.46E-23 1.39E-22 40.99205074

MIR4713HG 0.432854133 0.227710244 10.72504172 2.46E-23 1.39E-22 40.98959189

AC009318.4 -0.704805414 1.180229452 -10.71886731 2.59E-23 1.46E-22 40.93988473

AC025159.1 -0.654749995 2.248858168 -10.71720819 2.62E-23 1.48E-22 40.92653044

AP000812.1 0.21359319 0.116862937 10.71684105 2.63E-23 1.49E-22 40.92357546

AC012467.2 0.381068609 2.001585175 10.71639827 2.64E-23 1.49E-22 40.92001172

AC011611.4 -0.180064143 0.087414852 -10.71402788 2.69E-23 1.52E-22 40.90093485

AL096701.3 -0.404773605 0.707722333 -10.71011655 2.78E-23 1.57E-22 40.86946112

AL078644.2 0.351991294 0.568858814 10.70731016 2.84E-23 1.60E-22 40.84688217

A1BG-AS1 -0.539276537 1.253453415 -10.70084684 3.00E-23 1.69E-22 40.79489235

AP006284.1 -1.117204515 3.126511393 -10.6991751 3.04E-23 1.71E-22 40.78144777

LINC00628 0.283680455 0.200231322 10.69281717 3.20E-23 1.80E-22 40.73032503

AC091564.3 -0.262067666 0.127224702 -10.69214158 3.21E-23 1.81E-22 40.72489363

AC114284.1 0.336377075 0.378549132 10.69150631 3.23E-23 1.82E-22 40.71978659

AL360181.1 0.466149106 0.308752503 10.68827335 3.32E-23 1.86E-22 40.69379846

FAM83C-AS1 0.331224484 0.208097606 10.68590924 3.38E-23 1.90E-22 40.67479704

AC010649.1 -0.361863528 0.194101327 -10.681947 3.49E-23 1.96E-22 40.64295551

SCARNA9 -0.620864638 1.433008753 -10.681447 3.50E-23 1.97E-22 40.63893778

AC120053.1 0.589006095 2.520890168 10.68040646 3.53E-23 1.98E-22 40.63057694

AL136040.1 0.327996804 0.710735179 10.68018467 3.54E-23 1.99E-22 40.62879492

LINC02732 0.417890142 0.268395185 10.6801304 3.54E-23 1.99E-22 40.62835884

AL158151.1 0.313832029 0.358272813 10.67936608 3.56E-23 2.00E-22 40.62221774

AP001574.1 0.286678521 0.16872702 10.67276068 3.76E-23 2.11E-22 40.56915466

AC106820.2 -0.077656213 0.072898103 -10.65929467 4.19E-23 2.35E-22 40.46102934

AC016747.4 -0.263647265 0.288377894 -10.65744812 4.25E-23 2.38E-22 40.44620781

AC009041.1 0.274481992 0.167695823 10.65500377 4.34E-23 2.43E-22 40.42658998

LINC00599 -0.199686483 0.108166369 -10.65215407 4.44E-23 2.48E-22 40.40372169

AL139351.1 0.406106692 0.232232562 10.64988825 4.52E-23 2.53E-22 40.38554122

RMDN2-AS1 -0.196575704 0.146806201 -10.64070463 4.87E-23 2.72E-22 40.31187335

LINC02696 -0.692252175 0.765471707 -10.63889566 4.94E-23 2.76E-22 40.29736613

AC090587.2 0.36560568 0.486766042 10.63842014 4.96E-23 2.77E-22 40.29355288

AL596223.1 0.155314553 0.089660466 10.63743557 5.00E-23 2.79E-22 40.28565778

LINC00994 -0.097475779 0.081318735 -10.63732254 5.01E-23 2.79E-22 40.28475139

AC087741.2 1.165565177 1.489186425 10.63344531 5.16E-23 2.88E-22 40.25366418

AC139100.1 -0.324180794 0.395596021 -10.62835386 5.38E-23 3.00E-22 40.21285013

AC087301.1 0.336655237 0.285055835 10.62687996 5.44E-23 3.04E-22 40.20103693

LINC01376 -0.381125841 0.737164794 -10.62469926 5.54E-23 3.09E-22 40.18356028

AC008750.1 0.370971688 0.308207297 10.61655366 5.92E-23 3.30E-22 40.11829539

AC109322.1 0.493038259 1.175408132 10.61571153 5.96E-23 3.32E-22 40.11154947

AC109454.2 0.427738681 0.471445174 10.60791739 6.34E-23 3.53E-22 40.04912678

AC091182.2 0.502404443 0.294932115 10.60508541 6.49E-23 3.61E-22 40.02645141

RSF1-IT2 0.229555917 0.208727577 10.60359151 6.57E-23 3.65E-22 40.01449109

AC004492.1 -0.46813559 1.375319906 -10.59888957 6.82E-23 3.79E-22 39.97685257

AC005332.6 0.447010955 4.040408871 10.59011794 7.32E-23 4.07E-22 39.90665927

PPP4R1-AS1 0.489997604 0.472361567 10.58830086 7.43E-23 4.13E-22 39.89212215

AL163636.1 0.277615336 0.238472995 10.57768319 8.09E-23 4.49E-22 39.80720306

AL139022.2 0.393373199 0.466316786 10.57590724 8.21E-23 4.56E-22 39.79300334

LINC01607 -0.576012909 0.854491515 -10.57352229 8.37E-23 4.64E-22 39.77393634

AL512413.1 0.815138518 0.956824729 10.572878 8.41E-23 4.66E-22 39.76878578

LINC01184 -0.439566063 3.083909503 -10.57253531 8.43E-23 4.68E-22 39.76604632

AL133375.1 -0.212083468 0.102959118 -10.57043365 8.58E-23 4.75E-22 39.74924679

AC008734.1 -0.334350192 0.32968678 -10.56432914 9.01E-23 4.99E-22 39.70046007

KIZ-AS1 -0.087592877 0.100847899 -10.56326664 9.09E-23 5.03E-22 39.69197014

AC007370.2 -0.078384227 0.048843157 -10.56273266 9.13E-23 5.05E-22 39.68770356

AC016597.1 -0.216006955 0.190453712 -10.55876458 9.42E-23 5.21E-22 39.65600108

LINC01943 0.514916432 0.930586913 10.55147556 9.99E-23 5.53E-22 39.59778231

AC087442.1 0.394756878 0.249287479 10.55010971 1.01E-22 5.58E-22 39.58687534

BEAN1-AS1 -0.508371602 0.743159837 -10.54523506 1.05E-22 5.80E-22 39.54795461

LINC01475 0.317746489 0.267625446 10.54157777 1.08E-22 5.98E-22 39.51875963

AC138969.3 -0.127757524 0.063647085 -10.54029201 1.09E-22 6.04E-22 39.5084971

AC131159.2 0.37476323 0.624194962 10.53856571 1.11E-22 6.12E-22 39.49471924

AC106037.2 0.582998898 0.556866281 10.5360748 1.13E-22 6.24E-22 39.474841

HIF1A-AS1 0.204137804 0.168662868 10.53511747 1.14E-22 6.28E-22 39.46720184

AC007622.2 -0.213911059 0.402675569 -10.53204118 1.17E-22 6.44E-22 39.44265651

AP000697.1 0.388779927 0.204163963 10.53200773 1.17E-22 6.44E-22 39.44238969

AC027307.2 0.574719898 3.327639879 10.53044366 1.18E-22 6.52E-22 39.42991162

AC002470.1 0.601837906 0.998291061 10.53033078 1.18E-22 6.52E-22 39.42901109

AC008443.6 -0.289112277 0.326174188 -10.53020398 1.19E-22 6.52E-22 39.42799949

LINC00865 0.499486466 1.08407905 10.52205659 1.27E-22 6.96E-22 39.36301609

AC138028.2 -0.140462547 0.286850957 -10.52011851 1.29E-22 7.07E-22 39.34756176

AL031666.3 -0.160622159 0.146415071 -10.51613272 1.33E-22 7.30E-22 39.31578353

AC099778.1 0.341968765 0.927955602 10.51397778 1.35E-22 7.42E-22 39.29860503

AC035139.1 0.292074504 0.155963136 10.50582484 1.44E-22 7.92E-22 39.23362864

LINC01433 0.233453162 0.155064907 10.5055103 1.44E-22 7.93E-22 39.23112236

AP002336.2 0.354710256 0.588383248 10.50455718 1.46E-22 7.99E-22 39.22352814

AC131011.1 0.260194971 0.230852106 10.50439326 1.46E-22 8.00E-22 39.22222208

TMPO-AS1 0.366740795 0.870682843 10.50346947 1.47E-22 8.06E-22 39.21486192

AC051619.4 0.329096037 0.215511941 10.50290658 1.48E-22 8.09E-22 39.21037732

AC126755.3 -0.127313071 0.063875771 -10.50174596 1.49E-22 8.16E-22 39.20113098

AC079210.1 0.210411272 0.112615315 10.49953933 1.52E-22 8.30E-22 39.18355274

AC106017.1 -0.172331152 0.084200574 -10.49284237 1.60E-22 8.76E-22 39.13021583

TDRG1 -0.183414574 0.127308745 -10.48543089 1.70E-22 9.29E-22 39.07120857

AC244517.5 -0.226592159 0.127257311 -10.4850655 1.70E-22 9.31E-22 39.06830007

AC090241.2 -0.099833314 0.066674782 -10.48444892 1.71E-22 9.36E-22 39.06339212

AC109309.1 0.567195337 0.29704155 10.48411958 1.71E-22 9.38E-22 39.06077069

AC008114.1 0.257994284 0.168255507 10.4831024 1.73E-22 9.45E-22 39.05267448

AC121338.1 0.071695008 0.043971528 10.46318429 2.03E-22 1.11E-21 38.89421897

AC091078.1 -0.13435593 0.121162525 -10.46182909 2.05E-22 1.12E-21 38.88344353

AL356512.1 -0.304862663 0.67747421 -10.45944118 2.09E-22 1.14E-21 38.86445859

AL162724.1 0.23865212 0.165534108 10.44640704 2.32E-22 1.27E-21 38.76087071

AP001029.1 0.631099956 0.438523468 10.44183797 2.41E-22 1.31E-21 38.72457419

L3MBTL4-AS1 0.240188184 0.26757341 10.44163684 2.41E-22 1.31E-21 38.72297661

MIAT 0.75450796 0.998803938 10.43903565 2.46E-22 1.34E-21 38.70231673

SCAMP1-AS1 0.520230986 2.745796718 10.43862147 2.47E-22 1.34E-21 38.69902731

AP006248.4 -0.246068197 0.126956892 -10.43749359 2.49E-22 1.36E-21 38.6900702

SOS1-IT1 0.358712846 1.127583394 10.4362546 2.52E-22 1.37E-21 38.68023124

AC027796.1 -0.322629118 0.32837571 -10.41848712 2.90E-22 1.58E-21 38.53920425

LINC02785 0.307846817 0.214992716 10.41633709 2.95E-22 1.60E-21 38.52214713

AC022445.1 0.189744746 0.122424409 10.41612147 2.95E-22 1.61E-21 38.52043665

AL603832.1 0.322765496 0.308596817 10.41569608 2.96E-22 1.61E-21 38.5170621

AC073323.1 0.606439064 0.336155773 10.4148106 2.99E-22 1.62E-21 38.51003794

AL033381.2 0.450777787 0.252130229 10.41382461 3.01E-22 1.63E-21 38.50221687

AC093520.1 0.148175984 0.087445269 10.41337167 3.02E-22 1.64E-21 38.49862421

LINC02361 0.490665342 1.086473081 10.41336556 3.02E-22 1.64E-21 38.49857567

AL109924.1 0.180222006 0.108787882 10.41091938 3.08E-22 1.67E-21 38.47917417

AC008735.1 -0.371975749 0.760526825 -10.40822145 3.15E-22 1.71E-21 38.45777873

AL359504.2 -0.455211361 0.968859401 -10.40499962 3.23E-22 1.75E-21 38.43223231

AC145207.2 -0.640457034 0.310919541 -10.39910405 3.38E-22 1.83E-21 38.38549601

LMO7DN-IT1 0.2859826 0.166342712 10.39181483 3.59E-22 1.94E-21 38.32773088

MIR4458HG -0.629937938 2.013295617 -10.38888278 3.67E-22 1.99E-21 38.30450109

LINC02158 0.19981718 0.207760678 10.38777989 3.70E-22 2.00E-21 38.29576411

BAALC-AS1 -0.389717961 1.094911819 -10.37871265 3.98E-22 2.15E-21 38.22395257

AC091180.5 0.266209128 0.193161876 10.37282742 4.17E-22 2.26E-21 38.17735972

AC116552.1 0.525958436 0.57281838 10.3704852 4.25E-22 2.30E-21 38.15882046

AC112496.1 0.388244475 0.473572951 10.36664941 4.38E-22 2.37E-21 38.12846385

AL121974.1 1.178662833 0.861694746 10.36397337 4.48E-22 2.42E-21 38.10728902

AC079209.1 0.198257905 0.148324657 10.36012691 4.62E-22 2.49E-21 38.07685795

MIR193BHG -0.804589343 1.661437088 -10.35852209 4.68E-22 2.52E-21 38.06416326

LINC01449 0.144316441 0.094148925 10.35513927 4.80E-22 2.59E-21 38.03740733

LINC02802 -0.751604008 1.53561822 -10.35487339 4.81E-22 2.60E-21 38.03530457

AC005740.3 0.235862181 0.183722794 10.35464217 4.82E-22 2.60E-21 38.033476

AC007991.2 0.547208676 0.282453687 10.35282629 4.89E-22 2.64E-21 38.0191158

SNAP47-AS1 -0.111476986 0.098006101 -10.3506816 4.98E-22 2.68E-21 38.00215704

AC112503.2 -0.33399137 0.572409652 -10.34735267 5.11E-22 2.75E-21 37.97583775

AC007569.1 0.246705655 0.157875744 10.34669439 5.14E-22 2.77E-21 37.97063377

LINC01827 0.225020772 0.129011906 10.34014312 5.41E-22 2.91E-21 37.91885257

AC025809.1 0.170559089 0.110981162 10.33667477 5.56E-22 2.99E-21 37.89144578

RNASEH2B-AS1 -0.243534111 0.350283518 -10.33578804 5.60E-22 3.01E-21 37.8844396

AC010175.1 0.502091528 0.377037762 10.33555782 5.61E-22 3.02E-21 37.88262066

AL450344.3 0.224267793 0.149002415 10.33382744 5.69E-22 3.06E-21 37.86894981

AC073957.2 -0.51186379 0.248492007 -10.32809583 5.96E-22 3.20E-21 37.82367586

AC005699.1 0.292423039 0.190417588 10.32734533 5.99E-22 3.22E-21 37.81774862

LINC02253 1.235622461 0.753485724 10.32567352 6.07E-22 3.26E-21 37.80454603

LINC00092 -0.424316271 0.870892707 -10.32527363 6.09E-22 3.27E-21 37.80138817

AL096677.1 0.116112603 0.090889213 10.31941141 6.38E-22 3.42E-21 37.75510281

HCG21 0.507389797 0.294942436 10.31390486 6.67E-22 3.58E-21 37.71163827

AL359538.1 -0.134653941 0.107492096 -10.31314747 6.71E-22 3.60E-21 37.70566098

AC020978.3 0.301988239 0.343158473 10.3111844 6.81E-22 3.65E-21 37.6901695

AC018845.3 0.320605894 0.370952446 10.30449109 7.18E-22 3.85E-21 37.63736138

AC133552.5 -0.759275067 2.512911148 -10.30403797 7.21E-22 3.86E-21 37.63378705

AC020661.1 0.282196812 0.394355985 10.30327578 7.25E-22 3.88E-21 37.62777489

ZFHX4-AS1 0.259347343 0.20652429 10.30191407 7.33E-22 3.92E-21 37.61703429

ADAMTS9-AS1 -0.506010229 0.657686014 -10.29789273 7.57E-22 4.05E-21 37.58532005

WWC2-AS2 0.221182173 0.439992481 10.29268433 7.89E-22 4.22E-21 37.54425379

AL365356.1 -0.19023012 0.188411446 -10.28690828 8.26E-22 4.41E-21 37.49872457

AL360093.1 -0.20958383 0.216853299 -10.28475609 8.40E-22 4.49E-21 37.48176363

AP005205.2 -0.464190507 0.524136044 -10.28414321 8.44E-22 4.51E-21 37.47693397

AC013356.4 -0.212480484 0.103151855 -10.28129425 8.64E-22 4.61E-21 37.45448546

AP002761.1 0.481884745 0.334693509 10.27982117 8.74E-22 4.66E-21 37.44287955

AC004233.2 -0.78771136 2.581922212 -10.27917853 8.78E-22 4.68E-21 37.43781666

AC025575.2 0.750847156 0.503803438 10.27854686 8.83E-22 4.71E-21 37.43284037

AL645608.7 -0.869040593 1.880402388 -10.27344234 9.19E-22 4.90E-21 37.39263302

AC060780.1 0.541791672 1.958612148 10.27273958 9.24E-22 4.92E-21 37.38709837

AC116914.1 0.199349019 0.122537846 10.26482601 9.84E-22 5.24E-21 37.32478778

RRN3P2 0.42418926 0.888377673 10.26093689 1.01E-21 5.40E-21 37.29417462

IGFL2-AS1 1.103371924 0.686570133 10.25824675 1.04E-21 5.52E-21 37.27300272

SBF2-AS1 -0.404874056 1.657839103 -10.25492445 1.06E-21 5.66E-21 37.24685979

PLUT 0.361903633 0.434447638 10.25393282 1.07E-21 5.71E-21 37.23905763

RASSF10-DT 0.403897644 0.237311628 10.25354413 1.08E-21 5.72E-21 37.23599944

CATIP-AS2 0.162228962 0.108028898 10.25236668 1.09E-21 5.77E-21 37.22673592

AC136475.8 -0.437588669 0.28443629 -10.24953677 1.11E-21 5.90E-21 37.20447402

AC011933.3 0.283175437 0.180383918 10.24335537 1.17E-21 6.19E-21 37.15585853

AL139353.2 -0.299192012 0.506947009 -10.2404562 1.19E-21 6.34E-21 37.13306252

AC136698.1 -0.1189079 0.05772563 -10.23818636 1.22E-21 6.45E-21 37.11521728

AC134682.1 -0.115104948 0.171464499 -10.23297486 1.27E-21 6.72E-21 37.07425296

LINC01565 -0.107429822 0.056936999 -10.23103469 1.29E-21 6.82E-21 37.05900534

AC135983.1 -0.198632934 0.162151697 -10.22887294 1.31E-21 6.93E-21 37.0420181

AC022613.1 -0.837219145 2.385717871 -10.2243287 1.36E-21 7.18E-21 37.00631538

AL591178.1 0.302294497 0.191138066 10.22140224 1.39E-21 7.35E-21 36.98332755

AL513550.1 0.442591705 1.338299636 10.20456223 1.59E-21 8.39E-21 36.85111455

TMEM254-AS1 -0.424254139 0.925798012 -10.19255676 1.74E-21 9.22E-21 36.75692921

DSG2-AS1 0.298198903 0.381320109 10.19146277 1.76E-21 9.30E-21 36.74834957

AL357568.1 -0.079593695 0.050547944 -10.18689569 1.82E-21 9.64E-21 36.71253749

AC015726.2 0.304075228 0.157353048 10.18558454 1.84E-21 9.74E-21 36.70225792

AP003973.2 0.254635254 0.190842407 10.18420989 1.86E-21 9.84E-21 36.69148122

CARS-AS1 -0.085048738 0.085440632 -10.1794795 1.93E-21 1.02E-20 36.65440286

AC092384.3 -0.327177129 0.301747858 -10.17804073 1.95E-21 1.03E-20 36.64312717

AC120498.2 0.698487775 0.577839893 10.17278926 2.04E-21 1.08E-20 36.60197839

AC007285.2 0.264002916 0.219964816 10.17115228 2.06E-21 1.09E-20 36.58915387

AC034243.1 -0.958844805 1.184179107 -10.17109929 2.06E-21 1.09E-20 36.58873876

AC008649.2 0.22554503 0.120727489 10.163798 2.19E-21 1.15E-20 36.53155226

ELF3-AS1 0.692504777 1.917472868 10.16337341 2.19E-21 1.16E-20 36.52822743

CKMT2-AS1 -0.461992508 2.177398555 -10.16074008 2.24E-21 1.18E-20 36.50760806

ZNF197-AS1 0.279901994 0.421275942 10.15855927 2.28E-21 1.20E-20 36.49053414

AC012615.2 -0.765656216 0.371699376 -10.15550991 2.33E-21 1.23E-20 36.46666355

AF127936.1 0.315122765 0.274972474 10.1536071 2.37E-21 1.25E-20 36.45177014

AC068189.1 0.105883437 0.056979284 10.15180163 2.40E-21 1.26E-20 36.43764004

AP005899.1 0.486922124 0.720249327 10.14913636 2.46E-21 1.29E-20 36.41678337

AC023813.3 0.180430466 0.140014256 10.14227886 2.59E-21 1.36E-20 36.36313458

AC110995.1 0.306190577 0.294353381 10.14187393 2.60E-21 1.37E-20 36.35996726

AC002398.2 -0.759441802 0.844183241 -10.13674581 2.71E-21 1.42E-20 36.31986164

AC010487.2 0.721470593 1.117175069 10.13442526 2.76E-21 1.45E-20 36.3017169

AC025031.4 -0.150104685 0.29640853 -10.12596665 2.95E-21 1.55E-20 36.23559655

AC008080.1 -0.145153765 0.174236124 -10.12472299 2.98E-21 1.56E-20 36.2258774

AC244453.3 0.275035427 0.190464275 10.1174982 3.15E-21 1.65E-20 36.16942908

AC009123.1 -0.118988832 0.074090608 -10.11648674 3.17E-21 1.66E-20 36.16152811

U52111.1 0.411061718 0.621353103 10.11276354 3.27E-21 1.71E-20 36.13244823

AL358075.2 0.400255085 0.27247088 10.11202889 3.29E-21 1.72E-20 36.12671103

AL603839.3 0.480043805 0.904874525 10.11117558 3.31E-21 1.73E-20 36.12004733

AC120349.1 0.443377601 0.233047211 10.10870013 3.38E-21 1.77E-20 36.10071779

BSN-DT -0.263117266 0.260090915 -10.10658029 3.43E-21 1.80E-20 36.08416708

AL450998.3 -0.329840854 0.802782947 -10.10376613 3.51E-21 1.84E-20 36.06219838

AL354733.1 -0.261186532 0.155912735 -10.10302702 3.53E-21 1.85E-20 36.05642909

AC009495.3 0.411612454 0.843764922 10.09761789 3.68E-21 1.92E-20 36.01421368

TMEM26-AS1 0.151057122 0.113343827 10.09493808 3.76E-21 1.96E-20 35.9933037

SPRY4-AS1 0.325037898 0.393495522 10.09478522 3.77E-21 1.97E-20 35.99211106

AC087273.1 -0.356434332 0.202356883 -10.09315826 3.81E-21 1.99E-20 35.97941781

AL021937.4 0.186688314 0.134835225 10.08613967 4.03E-21 2.10E-20 35.92467276

AC145207.8 0.388121016 0.489804287 10.08436041 4.09E-21 2.13E-20 35.91079783

AC104655.1 0.383352232 0.544878359 10.07675146 4.34E-21 2.26E-20 35.85147705

AC010280.2 0.21665445 0.164470217 10.07597951 4.36E-21 2.28E-20 35.84546013

Z97055.2 -0.227600373 0.377105789 -10.07578829 4.37E-21 2.28E-20 35.84396972

AL023584.2 0.203717395 0.108271259 10.07530104 4.39E-21 2.29E-20 35.84017207

AP001062.2 0.306162132 0.158426832 10.0746819 4.41E-21 2.30E-20 35.83534654

LINC00592 0.203645188 0.130927743 10.07462208 4.41E-21 2.30E-20 35.83488033

AL596247.1 0.214929578 0.136703864 10.0731529 4.46E-21 2.32E-20 35.82343042

AL136164.2 -0.202302463 0.129717534 -10.0622347 4.86E-21 2.53E-20 35.73836911

AC131254.1 0.054964849 0.032239242 10.06150779 4.89E-21 2.54E-20 35.73270766

SYNGAP1-AS1 -0.333038987 0.303080931 -10.05937019 4.97E-21 2.58E-20 35.71606059

AL592546.2 0.263399855 0.237406944 10.05638779 5.09E-21 2.64E-20 35.6928377

LINC01132 0.235761665 0.333542083 10.05571958 5.12E-21 2.66E-20 35.6876351

AP005230.1 0.37492499 0.218585168 10.05349551 5.21E-21 2.70E-20 35.67032012

LINC02461 0.433412062 0.242171399 10.04944865 5.37E-21 2.79E-20 35.63881953

AC007728.2 0.311991512 0.33542647 10.04897495 5.39E-21 2.80E-20 35.63513271

TAPT1-AS1 -0.484846683 1.773611208 -10.04797785 5.44E-21 2.82E-20 35.62737262

AC048341.2 0.925252418 2.748116656 10.03824843 5.87E-21 3.04E-20 35.55167356

MIR646HG -0.244826695 0.285541359 -10.03737784 5.91E-21 3.06E-20 35.54490198

RERG-IT1 0.36373567 0.271802034 10.03538783 6.00E-21 3.11E-20 35.52942452

AC036176.3 0.332087109 0.178834254 10.03466503 6.03E-21 3.12E-20 35.52380325

AC015909.1 0.148301158 0.086026049 10.03248202 6.14E-21 3.18E-20 35.50682734

AP001107.9 -0.825752949 2.779323965 -10.03092751 6.21E-21 3.22E-20 35.49474014

AL139147.1 0.207873294 0.151426669 10.02999518 6.26E-21 3.24E-20 35.48749119

AL359878.2 0.228471226 0.133125356 10.02800887 6.36E-21 3.29E-20 35.47204865

FP236315.1 -0.229898373 0.126023987 -10.02240519 6.64E-21 3.43E-20 35.42849215

AC010654.1 0.352191366 0.82189971 10.02116628 6.71E-21 3.47E-20 35.41886403

RNF216-IT1 0.194797228 0.10401364 10.01859259 6.84E-21 3.53E-20 35.39886499

LINC01003 0.496402518 2.321544089 10.0180041 6.87E-21 3.55E-20 35.39429245

AC009097.2 0.506236362 0.526040752 10.01466739 7.06E-21 3.64E-20 35.36836926

LINC00707 0.555382301 0.30260815 10.00882422 7.39E-21 3.81E-20 35.32298454

AP000866.1 -0.26537301 0.576928068 -10.00881103 7.39E-21 3.81E-20 35.32288211

AL445288.1 -0.209912265 0.101905075 -10.00582185 7.56E-21 3.90E-20 35.29967038

AC013264.1 0.51258487 0.307799076 10.00186944 7.80E-21 4.02E-20 35.26898473

AC027237.3 0.341392748 0.621966295 9.999329838 7.96E-21 4.10E-20 35.24927131

AC092127.1 -0.065047392 0.04747699 -9.997706757 8.06E-21 4.15E-20 35.23667374

AC005550.2 0.913370173 0.540523505 9.993293041 8.34E-21 4.29E-20 35.2024223

AP001092.1 0.190287546 0.11963673 9.992490403 8.39E-21 4.32E-20 35.19619454

AL365361.1 0.648298332 0.774814751 9.990476771 8.53E-21 4.39E-20 35.18057173

AC026367.2 -0.517518407 1.104111416 -9.988572389 8.65E-21 4.45E-20 35.16579815

FOXC2-AS1 0.555701884 0.309744503 9.988196088 8.68E-21 4.46E-20 35.1628791

AC008878.4 -0.513176558 0.24912931 -9.987455375 8.73E-21 4.49E-20 35.15713342

GK-IT1 0.253900436 0.162837555 9.984575611 8.93E-21 4.59E-20 35.13479744

AC012588.1 -0.29746258 0.144407698 -9.982364009 9.08E-21 4.66E-20 35.11764624

AL117335.1 -0.374386457 0.751414418 -9.981063004 9.18E-21 4.71E-20 35.1075578

AP001033.1 0.488943661 0.649116533 9.972990561 9.77E-21 5.02E-20 35.0449774

AC099850.1 0.235389079 0.589633018 9.971763301 9.87E-21 5.06E-20 35.03546569

AC092171.3 0.494500119 1.023797327 9.97098242 9.93E-21 5.09E-20 35.02941392

ITGB5-AS1 0.209850063 0.130405697 9.970183605 9.99E-21 5.12E-20 35.02322342

AC100793.2 -0.487422494 0.35142363 -9.968783456 1.01E-20 5.18E-20 35.0123735

Z69733.1 0.322500816 0.411258758 9.967746054 1.02E-20 5.22E-20 35.00433508

AC080188.1 0.16909824 0.10178824 9.9650938 1.04E-20 5.32E-20 34.98378591

LINC01785 -0.159830015 0.128278176 -9.963630411 1.05E-20 5.38E-20 34.97244915

AC009159.3 0.461968668 0.89277554 9.961452974 1.07E-20 5.47E-20 34.95558238

LINC00243 0.184486553 0.167680957 9.957798961 1.10E-20 5.63E-20 34.92728241

AL139352.1 0.557142057 0.617088906 9.956889514 1.11E-20 5.67E-20 34.92023972

AC108451.2 0.66083487 0.461344746 9.956789064 1.11E-20 5.67E-20 34.91946187

AC010998.3 1.206029503 0.723216651 9.954902054 1.13E-20 5.75E-20 34.90485022

AL031768.1 -0.387822954 0.672142588 -9.949090471 1.18E-20 6.01E-20 34.85985908

AL353622.2 0.264831462 0.191797346 9.94871771 1.18E-20 6.03E-20 34.8569738

MTUS2-AS1 -0.143154008 0.143534559 -9.948037168 1.19E-20 6.06E-20 34.85170635

LINC01356 0.245089906 0.203072806 9.945066585 1.22E-20 6.20E-20 34.82871614

AC104462.1 0.176188428 0.117856613 9.93881375 1.28E-20 6.51E-20 34.780336

AC022092.1 0.129451432 0.069384958 9.938556604 1.28E-20 6.52E-20 34.77834674

AL034405.1 0.102383709 0.058927562 9.936797771 1.30E-20 6.61E-20 34.76474133

KLF7-IT1 0.318582461 0.35438629 9.935831646 1.31E-20 6.65E-20 34.75726845

LINC01936 0.318386736 0.49190558 9.935091419 1.31E-20 6.69E-20 34.75154315

AC073046.1 0.585660271 1.027560038 9.933216111 1.33E-20 6.78E-20 34.73703959

CEBPB-AS1 -0.272556043 0.842381947 -9.933157568 1.33E-20 6.79E-20 34.73658684

AC068338.2 -0.482351898 1.63496196 -9.932676556 1.34E-20 6.81E-20 34.73286697

AC004803.1 -0.463650148 1.292087469 -9.924584517 1.43E-20 7.25E-20 34.6703027

RFX3-AS1 -0.402293272 0.826140544 -9.922802182 1.45E-20 7.35E-20 34.65652621

TRPC7-AS1 0.735032648 0.432073553 9.922759278 1.45E-20 7.35E-20 34.65619461

AL627171.1 -0.183875897 0.303983913 -9.920511261 1.47E-20 7.47E-20 34.63882066

AC007952.4 -0.47255431 0.283713137 -9.91499893 1.54E-20 7.80E-20 34.59622745

BX088651.2 -0.083230684 0.04120348 -9.913834321 1.55E-20 7.87E-20 34.58723033

AC130469.1 0.341829736 0.318651387 9.911722016 1.58E-20 7.99E-20 34.5709133

AC004832.5 0.461073952 0.686653723 9.911670109 1.58E-20 8.00E-20 34.57051236

ARMC2-AS1 0.334088792 0.172796073 9.907754536 1.62E-20 8.24E-20 34.54027074

AC090164.2 0.746061216 0.418326856 9.906555811 1.64E-20 8.31E-20 34.53101381

AC005606.2 -0.503248151 0.257891628 -9.904237922 1.67E-20 8.46E-20 34.51311611

RXYLT1-AS1 0.225102652 0.204754607 9.903385932 1.68E-20 8.52E-20 34.506538

FMR1-IT1 0.479896033 0.711361423 9.899970242 1.73E-20 8.74E-20 34.48016907

LINC01619 -0.493593675 0.799727609 -9.897070211 1.77E-20 8.94E-20 34.45778494

AC007364.1 0.28944503 0.508253679 9.896309685 1.78E-20 8.99E-20 34.45191537

AC007952.3 -0.139520578 0.071401146 -9.892424534 1.83E-20 9.26E-20 34.42193452

AC005912.2 -0.077120586 0.059191381 -9.890281376 1.86E-20 9.41E-20 34.40539904

AC007952.8 -0.162890916 0.081009289 -9.888866083 1.88E-20 9.51E-20 34.39448048

AC025171.2 0.393867968 1.190795187 9.887768928 1.90E-20 9.59E-20 34.38601685

LINC02586 0.475686876 0.677600119 9.883977386 1.95E-20 9.87E-20 34.35677234

AC005921.2 -0.616291941 0.299188231 -9.877338286 2.06E-20 1.04E-19 34.30557936

AL359644.1 0.153651211 0.082555881 9.875665483 2.09E-20 1.05E-19 34.29268368

STEAP3-AS1 -0.137355036 0.229616077 -9.872414303 2.14E-20 1.08E-19 34.26762373

AC025287.3 -0.440295169 1.3564455 -9.870365755 2.17E-20 1.10E-19 34.25183597

AC103691.1 -0.723927377 2.383747797 -9.866031647 2.25E-20 1.13E-19 34.21843988

AL137779.1 0.232698734 0.241316711 9.865498947 2.26E-20 1.14E-19 34.21433578

AF064858.1 0.300302439 0.41831832 9.863893688 2.28E-20 1.15E-19 34.20196904

AC068987.3 0.305975769 0.569176993 9.859821915 2.36E-20 1.19E-19 34.17060559

LINC00322 0.182077607 0.100583609 9.855616997 2.44E-20 1.23E-19 34.13822416

AC008035.1 0.531502221 1.097927367 9.855051482 2.45E-20 1.23E-19 34.1338698

ARAP1-AS2 -0.213546497 0.471251931 -9.852962239 2.49E-20 1.25E-19 34.11778424

AC012462.1 0.145327814 0.081557777 9.85030937 2.54E-20 1.28E-19 34.09736195

AC114730.2 -0.723259966 0.52659716 -9.845277395 2.64E-20 1.33E-19 34.05863328

AC018638.7 -0.529065046 1.421343277 -9.841490002 2.72E-20 1.37E-19 34.02949084

AC011468.5 -0.432693562 1.138561646 -9.840161612 2.75E-20 1.38E-19 34.01927092

LINC01669 -0.314989437 0.155094755 -9.836369653 2.83E-20 1.42E-19 33.99010184

AC063944.1 -0.207258688 0.200483215 -9.836223115 2.83E-20 1.42E-19 33.98897474

AL513318.2 -0.376754282 0.431128589 -9.83499279 2.86E-20 1.43E-19 33.97951208

LINC01266 -0.167503095 0.192417349 -9.829994031 2.97E-20 1.49E-19 33.94107251

AC002128.2 0.41904581 0.674022093 9.828846619 3.00E-20 1.50E-19 33.93225066

AC011731.1 0.327216876 0.28980048 9.827603417 3.03E-20 1.52E-19 33.92269298

LINC02156 0.295403166 0.156471677 9.826823327 3.05E-20 1.53E-19 33.91669604

LINC00520 0.521602428 0.320646922 9.82605687 3.06E-20 1.53E-19 33.91080415

AC243960.3 0.31306396 0.374979845 9.824359894 3.10E-20 1.55E-19 33.89776012

AC012313.7 -0.355396429 0.172532561 -9.820637739 3.19E-20 1.60E-19 33.86915371

LINC02354 0.12818236 0.084739264 9.81530872 3.33E-20 1.67E-19 33.82820843

ATP2B1-AS1 0.467103948 0.78059905 9.807996562 3.52E-20 1.76E-19 33.77204609

AC093690.1 -0.141905499 0.298912612 -9.806961468 3.55E-20 1.78E-19 33.76409776

AC092687.3 0.674326751 1.541530168 9.801188803 3.71E-20 1.86E-19 33.71977901

AL592114.3 0.25015376 0.173116864 9.793895592 3.93E-20 1.96E-19 33.66380748

AP001610.2 0.280274562 0.205946853 9.787859554 4.12E-20 2.06E-19 33.61750181

LINC01691 0.182398597 0.099493382 9.787782931 4.12E-20 2.06E-19 33.61691409

SKAP1-AS1 0.120656185 0.072559592 9.786783255 4.15E-20 2.07E-19 33.60924663

AC004241.3 0.429763025 0.837882932 9.781417124 4.33E-20 2.16E-19 33.56809623

AC012087.2 -0.130783226 0.069409021 -9.780868198 4.35E-20 2.17E-19 33.56388749

LINC02453 -0.501723712 0.729286597 -9.777962523 4.44E-20 2.22E-19 33.5416112

AC018552.3 0.130678944 0.077445191 9.775114788 4.54E-20 2.26E-19 33.51978274

AC067817.2 0.427306604 0.579609092 9.774648797 4.56E-20 2.27E-19 33.51621117

AC118755.2 0.2837505 0.164198922 9.771777001 4.66E-20 2.32E-19 33.4942025

AL135924.2 0.471231588 0.349072437 9.769184371 4.76E-20 2.37E-19 33.47433642

DOCK9-DT 0.567025935 1.627475624 9.76850539 4.78E-20 2.38E-19 33.4691342

AC061975.4 -0.138307598 0.067143507 -9.762331247 5.01E-20 2.49E-19 33.42183853

AC068700.1 0.122787083 0.128001049 9.762183233 5.02E-20 2.50E-19 33.42070491

AC011446.2 -0.560861209 0.272278544 -9.758341071 5.17E-20 2.57E-19 33.39128165

AC023051.1 0.145988721 0.123429697 9.75695308 5.23E-20 2.60E-19 33.38065404

AC034111.1 -0.065015166 0.053625074 -9.753641274 5.36E-20 2.66E-19 33.35529953

AC078962.1 0.235522136 0.135800038 9.749561069 5.53E-20 2.75E-19 33.32406902

LINC00621 0.132365312 0.091779382 9.748468968 5.58E-20 2.77E-19 33.31571116

AC011477.4 -0.343720914 0.460877383 -9.744418427 5.76E-20 2.86E-19 33.28471698

AL390783.1 -0.14506197 0.094292788 -9.742700527 5.83E-20 2.89E-19 33.27157405

AC111152.2 0.24984602 0.137995984 9.737003286 6.10E-20 3.02E-19 33.22799624

AC083906.3 0.28322417 0.155394569 9.736640061 6.11E-20 3.03E-19 33.22521845

AC006504.1 -0.322767055 0.601315272 -9.733283046 6.27E-20 3.11E-19 33.19954818

AL158151.4 0.427626093 0.489834287 9.733263878 6.27E-20 3.11E-19 33.19940161

AL845472.1 -0.090709787 0.096062915 -9.729645838 6.45E-20 3.20E-19 33.171741

PRICKLE2-DT 0.242968201 0.12591135 9.72009961 6.94E-20 3.44E-19 33.09878625

AC007552.2 -0.279076147 0.259811728 -9.719304203 6.99E-20 3.46E-19 33.09270937

LINC02236 0.17554095 0.108546517 9.718580496 7.02E-20 3.48E-19 33.08718053

AC006064.3 0.435925233 0.409925718 9.715606257 7.19E-20 3.56E-19 33.06446095

AL121987.1 0.258624004 0.147917651 9.715007599 7.22E-20 3.57E-19 33.0598884

AC090559.1 0.640441956 1.217030692 9.714729093 7.24E-20 3.58E-19 33.05776123

AC004080.2 0.534009479 0.277926921 9.713266746 7.32E-20 3.62E-19 33.0465927

AC005632.3 0.380654596 0.765362281 9.700149095 8.09E-20 4.00E-19 32.94645063

TMEM44-AS1 0.535802608 2.699840726 9.695762831 8.37E-20 4.13E-19 32.91298243

AC023034.1 -0.211256072 0.133755456 -9.693790872 8.50E-20 4.20E-19 32.89793874

AC023154.1 -0.518869555 0.295116036 -9.690000245 8.75E-20 4.32E-19 32.86902568

AC007485.1 0.72017193 0.770581033 9.689445103 8.79E-20 4.34E-19 32.86479187

AC104134.1 -0.190769766 0.160884817 -9.681254106 9.36E-20 4.62E-19 32.80233899

AC093734.1 0.174517383 0.090691017 9.680366607 9.42E-20 4.65E-19 32.795574

AL359979.2 0.186417262 0.125297035 9.675149148 9.81E-20 4.83E-19 32.75581088

AC020763.1 0.130024863 0.08362966 9.667454091 1.04E-19 5.13E-19 32.69718791

AC091152.2 -0.113179972 0.165623558 -9.665806774 1.05E-19 5.19E-19 32.68464169

LINC01887 -0.41188554 0.266230165 -9.661963638 1.09E-19 5.34E-19 32.6553765

AC092143.3 0.316450092 0.340018144 9.6598264 1.10E-19 5.43E-19 32.63910448

AC006262.2 0.268027656 0.142928311 9.659485816 1.11E-19 5.44E-19 32.63651161

AC000032.1 -0.469535433 0.359978867 -9.658485803 1.11E-19 5.48E-19 32.6288988

SLC25A34-AS1 0.233501314 0.177982489 9.658070822 1.12E-19 5.50E-19 32.6257398

PAXBP1-AS1 -0.398322972 1.075471844 -9.654182456 1.15E-19 5.66E-19 32.59614379

MELTF-AS1 -0.724798706 2.874069702 -9.653200054 1.16E-19 5.70E-19 32.58866739

AC244153.1 0.925276127 1.08722868 9.651550469 1.18E-19 5.77E-19 32.57611449

AC079305.1 0.378534239 0.492463257 9.651195559 1.18E-19 5.79E-19 32.57341388

AP001922.5 0.380327084 0.657591365 9.650129799 1.19E-19 5.83E-19 32.56530455

LINC00189 0.207530779 0.172630775 9.648795725 1.20E-19 5.89E-19 32.55515436

MED4-AS1 0.227710761 0.251081283 9.647159864 1.22E-19 5.96E-19 32.54270914

GDNF-AS1 -0.143701519 0.173056615 -9.646766817 1.22E-19 5.98E-19 32.53971912

MLIP-IT1 0.130020328 0.073225758 9.641632992 1.27E-19 6.22E-19 32.50067104

AC000036.1 -0.0785872 0.056208862 -9.637865135 1.31E-19 6.40E-19 32.47202016

AC011473.3 -0.329430083 0.268301864 -9.630280618 1.38E-19 6.78E-19 32.41436676

AL139082.1 -0.100916675 0.147048423 -9.628547839 1.40E-19 6.86E-19 32.40119877

NFIA-AS2 0.327592593 0.319410844 9.628481316 1.40E-19 6.87E-19 32.40069327

GK-AS1 0.38938512 0.587731771 9.626143094 1.43E-19 6.99E-19 32.38292653

AL138963.1 0.348061823 0.284929196 9.621392374 1.48E-19 7.24E-19 32.34683632

SLC25A5-AS1 -0.347759039 1.092308888 -9.620855485 1.49E-19 7.27E-19 32.34275833

LINC02821 -0.352772951 0.181569177 -9.620427836 1.49E-19 7.29E-19 32.33951017

LINC02611 0.336754316 0.605088793 9.618380647 1.52E-19 7.40E-19 32.32396216

AC026740.1 0.69735581 1.275699821 9.615931416 1.54E-19 7.54E-19 32.30536322

AC004471.2 -0.131216191 0.162964191 -9.612259811 1.59E-19 7.75E-19 32.27748693

AC093157.2 -0.337540551 0.724400113 -9.61068029 1.61E-19 7.85E-19 32.26549646

LINC02054 -0.159822069 0.112426197 -9.610445403 1.61E-19 7.86E-19 32.26371348

AL359922.2 -0.288783414 0.471379178 -9.601183674 1.73E-19 8.43E-19 32.19342961

AC008897.3 0.437430185 0.467337083 9.599702772 1.75E-19 8.52E-19 32.1821952

AP001626.1 -0.155611575 0.215733749 -9.595139465 1.81E-19 8.82E-19 32.14758336

AL592148.3 0.265390772 1.116645915 9.592475686 1.85E-19 9.00E-19 32.12738348

AC007639.1 0.247985382 0.253593866 9.588673946 1.90E-19 9.26E-19 32.09855985

DNM3OS 0.557723068 1.419919415 9.588054546 1.91E-19 9.31E-19 32.09386438

AC006213.5 0.503887534 0.655151986 9.582524499 1.99E-19 9.70E-19 32.05195059

SFTA1P 0.477053069 0.351723959 9.580530886 2.02E-19 9.85E-19 32.03684385

AC005394.1 -0.123882197 0.075929696 -9.578881346 2.05E-19 9.97E-19 32.02434573

AC092142.1 0.435149807 0.433593201 9.577831984 2.07E-19 1.00E-18 32.01639564

AL049871.1 0.194232683 0.197345869 9.573211647 2.14E-19 1.04E-18 31.98139741

AC104463.2 0.560187445 0.692215798 9.571314042 2.17E-19 1.06E-18 31.96702623

AL590648.2 -0.078679304 0.040528743 -9.570332405 2.19E-19 1.06E-18 31.95959261

LINC01402 0.333864626 0.2380474 9.568507082 2.22E-19 1.08E-18 31.94577121

AC080023.1 0.248637279 0.258620638 9.566915973 2.25E-19 1.09E-18 31.93372453

C9orf147 -0.323673525 0.48200143 -9.56480479 2.28E-19 1.11E-18 31.91774203

AP005131.1 -0.063130939 0.088207561 -9.561989648 2.33E-19 1.13E-18 31.89643345

AC025539.1 0.188391422 0.155387251 9.561385289 2.34E-19 1.14E-18 31.89185937

CECR3 -0.10160222 0.077699824 -9.560117337 2.37E-19 1.15E-18 31.88226343

AC073333.1 0.242929228 0.230504989 9.559993769 2.37E-19 1.15E-18 31.8813283

PDZRN3-AS1 -0.153098194 0.093982119 -9.557422893 2.42E-19 1.17E-18 31.86187416

TMSB15B-AS1 0.259171734 0.290308754 9.557246821 2.42E-19 1.17E-18 31.86054191

AC012485.2 0.471237167 0.521451331 9.554572096 2.47E-19 1.19E-18 31.84030537

AP005131.7 0.349883876 0.457267475 9.544371646 2.67E-19 1.29E-18 31.76316052

SPATA8 -0.211956892 0.140076908 -9.543067425 2.69E-19 1.30E-18 31.75330029

AC090739.1 -0.423575991 1.195697751 -9.541593812 2.73E-19 1.32E-18 31.74216035

AC087386.1 -0.171473514 0.08772716 -9.540452437 2.75E-19 1.33E-18 31.7335327

LINC01644 0.507708112 0.29722314 9.532170344 2.93E-19 1.41E-18 31.67094626

SSSCA1-AS1 -0.390184447 1.582943062 -9.53117555 2.95E-19 1.42E-18 31.66343089

LINC01215 0.641848462 0.396902771 9.528718688 3.01E-19 1.45E-18 31.64487197

AC018695.4 -0.364513591 0.913734954 -9.528272211 3.02E-19 1.46E-18 31.64149963

AC019117.2 0.767418262 0.429718905 9.526566463 3.06E-19 1.47E-18 31.62861655

XXYLT1-AS1 0.143375529 0.074667447 9.526346414 3.06E-19 1.48E-18 31.62695467

AC007996.1 0.438919905 1.504216791 9.52093902 3.19E-19 1.54E-18 31.58612342

GAS8-AS1 -0.427776843 0.746138649 -9.520465415 3.20E-19 1.54E-18 31.58254788

AC112484.1 -0.376428687 0.81232109 -9.520050915 3.21E-19 1.55E-18 31.57941863

LINC01283 0.081626359 0.04749842 9.518347023 3.25E-19 1.57E-18 31.56655603

AP000640.1 0.256720584 0.472554497 9.514862069 3.34E-19 1.61E-18 31.54025244

LINC02122 0.690515836 0.361745053 9.513325324 3.38E-19 1.63E-18 31.52865525

TAF1A-AS1 0.424833326 1.193095876 9.512228686 3.41E-19 1.64E-18 31.52038003

PEX5L-AS1 -0.121261175 0.068883873 -9.508151749 3.52E-19 1.69E-18 31.48962038

AC125603.2 -0.154867643 0.126282221 -9.508070229 3.52E-19 1.69E-18 31.48900541

AC025470.2 0.131534827 0.098793085 9.504300392 3.62E-19 1.74E-18 31.46056974

AC008937.1 -0.152967227 0.127764197 -9.50028075 3.73E-19 1.79E-18 31.43025705

AC136624.1 -0.221278943 0.141871013 -9.496714554 3.83E-19 1.84E-18 31.40337012

AC008687.2 0.436963427 0.258602721 9.493583819 3.93E-19 1.89E-18 31.37977116

AC037198.2 -0.782745929 1.227266914 -9.491990556 3.97E-19 1.91E-18 31.36776314

AC107204.1 -0.148548277 0.301859012 -9.491895644 3.98E-19 1.91E-18 31.36704785

AL731577.2 -0.538472287 1.125788707 -9.485414622 4.18E-19 2.00E-18 31.31821448

KLHL6-AS1 0.207410889 0.121897685 9.481344008 4.31E-19 2.07E-18 31.28755307

AC093904.3 0.471865615 0.255400522 9.477827182 4.43E-19 2.12E-18 31.2610692

AL391121.1 0.529429274 2.334071839 9.475925835 4.49E-19 2.15E-18 31.24675328

AC079380.1 0.094221118 0.055811533 9.474232495 4.55E-19 2.18E-18 31.23400493

TTTY2 -0.107069796 0.052066759 -9.473174956 4.59E-19 2.20E-18 31.2260439

CACTIN-AS1 -0.209464966 0.535991977 -9.467613771 4.78E-19 2.29E-18 31.1841885

LINC01087 -0.11139942 0.107427161 -9.466377805 4.83E-19 2.31E-18 31.17488814

AC136618.1 -0.181014761 0.095945723 -9.463829163 4.92E-19 2.35E-18 31.15571245

AC138512.1 0.207792595 0.134911743 9.462944528 4.96E-19 2.37E-18 31.14905727

AC113189.2 -0.101380663 0.050060892 -9.46111631 5.02E-19 2.40E-18 31.13530459

AC073655.2 -0.430972927 1.024874624 -9.459794524 5.07E-19 2.43E-18 31.12536248

LHFPL3-AS2 -1.08429802 2.121646146 -9.457584159 5.16E-19 2.47E-18 31.10873856

AC008808.2 0.760146947 0.670491429 9.457023187 5.18E-19 2.47E-18 31.1045199

AC015987.1 0.357519446 0.505694359 9.446646552 5.61E-19 2.68E-18 31.02651146

AL136084.2 -0.428210638 0.397677717 -9.446235419 5.62E-19 2.68E-18 31.02342172

AC010997.4 -0.297410596 0.67162439 -9.444005951 5.72E-19 2.73E-18 31.00666823

AC048382.4 -0.112223194 0.061165144 -9.43441223 6.15E-19 2.93E-18 30.93460207

SLFNL1-AS1 -0.190927558 0.535628652 -9.433194402 6.21E-19 2.96E-18 30.92545706

AC015853.1 -0.319744626 0.384726511 -9.425026446 6.60E-19 3.15E-18 30.86413954

AL021408.1 0.318620704 0.305405258 9.4194991 6.89E-19 3.28E-18 30.82266304

AC093904.4 0.407525818 0.225342729 9.414424379 7.16E-19 3.41E-18 30.78459559

LINC00427 0.171155439 0.123101468 9.412133622 7.28E-19 3.47E-18 30.76741569

AC090844.2 -0.329328406 0.394439705 -9.411763822 7.30E-19 3.47E-18 30.76464255

AC007255.1 0.479702023 1.073716817 9.411726738 7.30E-19 3.47E-18 30.76436445

AL135818.1 0.292077943 0.576203753 9.41159096 7.31E-19 3.48E-18 30.76334627

AC012462.3 0.245717794 0.127326112 9.410506155 7.37E-19 3.50E-18 30.75521174

AL139246.5 0.642383488 1.772840234 9.408452811 7.49E-19 3.56E-18 30.73981602

NRAD1 -0.180121449 0.157761463 -9.408194734 7.50E-19 3.56E-18 30.73788113

AL022157.1 0.308275297 0.601903865 9.405622522 7.65E-19 3.63E-18 30.71859811

AC004943.2 0.307961553 1.016966673 9.405404995 7.66E-19 3.64E-18 30.71696753

AC092574.2 -0.218467874 0.273283129 -9.403468884 7.77E-19 3.69E-18 30.70245539

AC110285.3 -0.358658202 0.3490551 -9.401851946 7.87E-19 3.73E-18 30.69033697

AL162731.1 -0.16246228 0.095323359 -9.401803692 7.87E-19 3.73E-18 30.68997534

AC079467.1 -0.191874753 0.135282428 -9.400248078 7.97E-19 3.78E-18 30.67831772

AC092868.2 0.094796786 0.053932855 9.396424215 8.20E-19 3.89E-18 30.6496669

CASC9 1.035215183 0.723581569 9.39577362 8.24E-19 3.90E-18 30.64479292

AC023855.1 0.095036661 0.049795413 9.394008615 8.35E-19 3.96E-18 30.63157123

CSE1L-AS1 -0.317035163 0.247494174 -9.38297022 9.08E-19 4.30E-18 30.54891573

SRD5A3-AS1 -0.334787839 0.914605426 -9.381773242 9.16E-19 4.33E-18 30.53995621

AC010173.1 0.188345125 0.309380576 9.381007065 9.21E-19 4.36E-18 30.53422165

AC097716.1 0.241416998 0.125113202 9.379701421 9.30E-19 4.40E-18 30.52444999

LINC02591 -0.103552458 0.055972223 -9.378690691 9.37E-19 4.43E-18 30.51688607

AC007998.3 0.408508471 0.581901473 9.376595776 9.52E-19 4.50E-18 30.50121006

LINC00239 0.646473855 1.947185669 9.376146497 9.56E-19 4.52E-18 30.49784843

BACH1-AS1 0.186600297 0.201353184 9.375437226 9.61E-19 4.54E-18 30.49254166

AC020891.2 0.304713735 0.161926344 9.371046601 9.93E-19 4.69E-18 30.45969624

AC026471.3 0.638254945 0.870121906 9.364505457 1.04E-18 4.92E-18 30.41078011

LINC02599 -0.095500209 0.105532621 -9.362114115 1.06E-18 5.01E-18 30.39290218

SLC6A1-AS1 0.138832122 0.095816639 9.360523127 1.07E-18 5.07E-18 30.38100927

LINC00462 0.48596809 0.272242607 9.357843638 1.10E-18 5.17E-18 30.36098235

AC008124.1 0.370093599 1.963391047 9.35559083 1.12E-18 5.26E-18 30.34414714

AL513008.1 0.297650174 0.170202111 9.353536997 1.13E-18 5.34E-18 30.32880097

AP000251.1 0.301004504 0.24833792 9.349659533 1.17E-18 5.50E-18 30.29983413

AP000350.6 -0.348960028 0.514563397 -9.348500657 1.18E-18 5.54E-18 30.29117805

AC074124.1 0.334762321 0.202947751 9.346760779 1.19E-18 5.61E-18 30.27818346

AL512363.1 0.344416558 0.302833207 9.345742104 1.20E-18 5.66E-18 30.27057597

LENG8-AS1 -0.613819681 2.884171724 -9.345487082 1.20E-18 5.66E-18 30.26867153

AC145350.2 -0.116433111 0.061542965 -9.343302011 1.22E-18 5.76E-18 30.25235529

AC091906.1 0.207309717 0.110128928 9.33566516 1.30E-18 6.10E-18 30.19534762

AC090971.2 0.134032444 0.076942379 9.335620893 1.30E-18 6.10E-18 30.19501725

AP000438.1 -0.311679158 0.324652225 -9.33403193 1.31E-18 6.17E-18 30.18315945

LINC00973 0.716217929 0.384354854 9.326124584 1.39E-18 6.54E-18 30.12416794

AC004148.1 -0.614309564 2.50297739 -9.32524374 1.40E-18 6.58E-18 30.11759838

LINC00294 0.37729497 2.792589678 9.322028015 1.44E-18 6.74E-18 30.09361783

AC091057.1 -0.375572699 1.092917917 -9.321966249 1.44E-18 6.74E-18 30.09315726

AC009163.4 -0.184455717 0.225349176 -9.317987952 1.48E-18 6.95E-18 30.06349694

AC244197.2 -0.758173422 1.411143746 -9.317725655 1.48E-18 6.96E-18 30.06154164

LINC01143 -0.363646698 0.418724954 -9.314048905 1.53E-18 7.15E-18 30.03413667

AC129510.1 -0.488160513 1.861965315 -9.304572243 1.64E-18 7.68E-18 29.96353129

AC138894.2 -0.479392439 0.234733488 -9.303160126 1.66E-18 7.75E-18 29.95301406

FRMD6-AS2 0.113664651 0.059380163 9.301268106 1.68E-18 7.86E-18 29.93892408

HOXA11-AS 0.569608517 0.37443599 9.300871485 1.68E-18 7.88E-18 29.93597063

LINC01781 0.80865019 0.459715134 9.297553286 1.73E-18 8.08E-18 29.91126457

AC008781.3 0.286814136 0.229810537 9.295020341 1.76E-18 8.23E-18 29.89240876

AC026367.3 -0.453015781 1.318681354 -9.294366814 1.77E-18 8.27E-18 29.88754425

AL512329.2 0.573721677 0.724450707 9.291217664 1.81E-18 8.47E-18 29.86410654

AC040904.1 0.301909267 0.189809028 9.289023917 1.84E-18 8.60E-18 29.84778226

LINC02642 0.135188216 0.097129786 9.288692215 1.85E-18 8.62E-18 29.84531418

AL008718.2 0.172727834 0.167489839 9.283176501 1.92E-18 8.98E-18 29.80428129

AL451064.1 -0.370309755 0.561905327 -9.279341021 1.98E-18 9.24E-18 29.77575672

GATA6-AS1 -0.874390684 2.872740387 -9.278485825 1.99E-18 9.30E-18 29.76939756

AC010285.1 -0.264203938 0.29270582 -9.276660773 2.02E-18 9.42E-18 29.75582782

LINC01687 -0.211689625 0.115703823 -9.270784272 2.11E-18 9.84E-18 29.71214536

AC010335.1 0.091460229 0.058971438 9.270603793 2.11E-18 9.85E-18 29.71080404

AC027319.1 0.330932198 0.283204615 9.261133102 2.27E-18 1.06E-17 29.64044021

AC004865.2 0.394979141 0.704599185 9.261049806 2.27E-18 1.06E-17 29.63982153

AC023302.1 0.473519076 0.532649657 9.26050925 2.28E-18 1.06E-17 29.63580672

ZEB1-AS1 0.413198655 2.113379034 9.26034049 2.28E-18 1.06E-17 29.63455334

AL121985.1 -0.144431681 0.161013461 -9.260239297 2.28E-18 1.06E-17 29.63380178

GRK5-IT1 0.160375617 0.091120831 9.260018591 2.29E-18 1.06E-17 29.63216262

PAX8-AS1 -1.338835476 2.505628715 -9.259569409 2.30E-18 1.07E-17 29.62882667

HOTTIP 0.573515764 0.316134063 9.251775183 2.43E-18 1.13E-17 29.57095659

HM13-AS1 0.287043445 0.277968057 9.250595172 2.46E-18 1.14E-17 29.56219787

AC243960.1 0.66231139 0.889745911 9.248056188 2.50E-18 1.16E-17 29.54335434

AC068134.2 0.409899177 0.226146124 9.246360101 2.53E-18 1.18E-17 29.53076826

LINC02104 0.351839124 0.346510864 9.246298557 2.54E-18 1.18E-17 29.53031159

AC061992.2 0.404767306 0.566111919 9.245791476 2.55E-18 1.18E-17 29.526549

AC079354.2 0.21599054 0.124065533 9.24459334 2.57E-18 1.19E-17 29.5176592

AC090409.1 0.327537266 0.470185484 9.241047137 2.64E-18 1.22E-17 29.49135154

AC095032.1 -0.254558735 0.18584067 -9.240970336 2.64E-18 1.22E-17 29.49078185

AC040174.2 0.524225063 0.4736672 9.239269927 2.67E-18 1.24E-17 29.47816951

AL513542.1 0.385704512 0.361636415 9.232073912 2.82E-18 1.31E-17 29.42481038

AL158834.2 -0.427588589 0.637117693 -9.230811097 2.85E-18 1.32E-17 29.41544906

AC116337.3 0.195616941 0.119157974 9.229480155 2.88E-18 1.33E-17 29.40558355

AL022068.1 -0.428485459 0.566175606 -9.227430849 2.92E-18 1.35E-17 29.3903949

ST7-AS2 -0.118032341 0.081109261 -9.225801832 2.96E-18 1.37E-17 29.37832272

AC244205.1 0.28220025 0.158673439 9.219208861 3.10E-18 1.44E-17 29.32947718

LINC01612 0.723181127 0.564348473 9.218757755 3.12E-18 1.44E-17 29.32613582

MACC1-AS1 0.133828684 0.077450017 9.216227143 3.17E-18 1.47E-17 29.30739334

LINC02387 0.360093744 0.217201781 9.215451921 3.19E-18 1.48E-17 29.30165244

LINC01730 0.472254847 0.447439353 9.213348253 3.24E-18 1.50E-17 29.28607518

AL161431.1 1.041085405 0.668721598 9.212743238 3.26E-18 1.51E-17 29.28159556

LINC02776 0.190305711 0.128466801 9.207837541 3.38E-18 1.56E-17 29.2452796

AC021127.1 0.22438136 0.133272005 9.200130015 3.58E-18 1.65E-17 29.18824581

U91328.2 -0.423206663 0.206501218 -9.199182523 3.61E-18 1.66E-17 29.18123659

AC022182.2 0.410667897 0.270171746 9.198847424 3.61E-18 1.67E-17 29.17875775

AC103760.1 0.487719738 1.089435428 9.194862703 3.72E-18 1.72E-17 29.14928559

AC009088.3 -0.808925397 0.392705054 -9.190684893 3.84E-18 1.77E-17 29.11839357

OLMALINC 0.608598841 2.279334289 9.18772857 3.93E-18 1.81E-17 29.09653873

AC139100.2 -0.524841043 1.186892647 -9.183857518 4.04E-18 1.86E-17 29.06792812

AP001059.2 -0.196988879 0.096003157 -9.176929944 4.26E-18 1.96E-17 29.01674524

AL031985.3 0.343774774 1.24996948 9.175716604 4.30E-18 1.98E-17 29.00778314

AC011476.3 -0.490197341 1.754921813 -9.173717982 4.36E-18 2.01E-17 28.99302229

AC144652.1 0.422446418 1.179086414 9.163492599 4.70E-18 2.17E-17 28.91753303

AL672310.1 -0.37350041 0.196783838 -9.162768847 4.73E-18 2.18E-17 28.91219184

AC063965.2 0.353846004 0.535060157 9.160562951 4.81E-18 2.21E-17 28.89591421

AL118522.1 0.398187205 0.535782222 9.160262906 4.82E-18 2.22E-17 28.89370031

AC079949.1 0.572250524 0.504462337 9.15978796 4.84E-18 2.22E-17 28.890196

AL589765.2 -0.184360468 0.089500569 -9.156794198 4.95E-18 2.27E-17 28.86810952

AC073283.1 0.444604674 0.55218949 9.147553611 5.30E-18 2.43E-17 28.79996475

AC104561.1 -0.28300271 0.360463666 -9.144895902 5.40E-18 2.48E-17 28.7803732

AC024560.1 -0.537457125 0.725218041 -9.142843904 5.49E-18 2.52E-17 28.76524906

AC019193.2 0.215518716 0.305618315 9.134462456 5.84E-18 2.68E-17 28.70349552

AC007666.1 -0.108470022 0.140369265 -9.133508753 5.88E-18 2.70E-17 28.69647094

AC025280.1 0.274337878 0.166858509 9.130519482 6.01E-18 2.76E-17 28.67445607

AC009974.1 0.333832337 0.43746585 9.129073858 6.08E-18 2.79E-17 28.66381116

AC087354.1 -0.101572335 0.059737684 -9.128440589 6.11E-18 2.80E-17 28.65914838

AC104984.2 0.158446782 0.100232235 9.126176027 6.21E-18 2.84E-17 28.64247596

C7orf69 0.090858079 0.051716846 9.122737729 6.37E-18 2.92E-17 28.61716693

AL049869.3 0.408875412 0.679624211 9.117774602 6.61E-18 3.03E-17 28.58064402

AL590867.1 -0.090919285 0.04539549 -9.112035538 6.90E-18 3.16E-17 28.53842621

AC007687.1 -0.503947624 0.469041992 -9.101771652 7.44E-18 3.41E-17 28.46296327

TRIM52-AS1 -0.63711174 3.950120229 -9.10170411 7.45E-18 3.41E-17 28.46246685

LINC02305 -0.245799524 0.155456263 -9.098549001 7.62E-18 3.49E-17 28.43928015

AC068228.1 0.261610024 0.141959743 9.098274792 7.64E-18 3.49E-17 28.43726524

ARHGAP31-AS1 0.347430408 0.644072078 9.098237306 7.64E-18 3.49E-17 28.4369898

AC008403.2 -0.173087461 0.091133253 -9.095407579 7.80E-18 3.56E-17 28.41619892

AC004160.2 0.228710049 0.140563087 9.094676617 7.84E-18 3.58E-17 28.41082896

LACTB2-AS1 -0.137806902 0.241742235 -9.087381586 8.28E-18 3.78E-17 28.35725099

AC009061.1 -0.407263873 0.475419782 -9.086818223 8.32E-18 3.79E-17 28.3531145

AC106881.1 0.152567954 0.256680332 9.085773992 8.38E-18 3.82E-17 28.34544764

AC106820.4 -0.150506592 0.297250548 -9.085709894 8.38E-18 3.82E-17 28.34497705

HAGLROS 0.314150247 0.463108716 9.084299885 8.47E-18 3.86E-17 28.33462551

C10orf71-AS1 -0.159909627 0.085161535 -9.082200797 8.60E-18 3.92E-17 28.31921694

HOTAIR 0.538345853 0.310836096 9.081099606 8.67E-18 3.95E-17 28.3111344

AL078581.2 0.520251288 1.13072247 9.078921483 8.82E-18 4.02E-17 28.29514916

AC010255.1 0.19905338 0.125361215 9.072060443 9.28E-18 4.22E-17 28.24481135

AC114488.2 0.39014464 0.524691681 9.069251174 9.47E-18 4.31E-17 28.22420714

AC046168.2 0.252883516 0.224349645 9.065794123 9.72E-18 4.42E-17 28.19885723

MEF2C-AS1 -0.263743918 0.3012615 -9.05764951 1.03E-17 4.69E-17 28.13915773

AL162171.2 -0.160135656 0.218475035 -9.055960292 1.04E-17 4.75E-17 28.12677999

AC093620.1 0.586565967 1.030504651 9.0548587 1.05E-17 4.79E-17 28.11870884

TTTY4 -0.059645081 0.028955599 -9.053129763 1.07E-17 4.85E-17 28.10604248

AC096586.2 -0.401182229 1.304018837 -9.052997553 1.07E-17 4.85E-17 28.10507396

AL161729.3 -0.366601433 0.627950305 -9.052850023 1.07E-17 4.86E-17 28.10399321

AC092117.1 0.44452706 0.715834091 9.052717142 1.07E-17 4.86E-17 28.1030198

AC108134.4 0.513779015 0.743381988 9.051177856 1.08E-17 4.91E-17 28.0917444

AC005520.2 0.632268403 2.290815276 9.049369316 1.10E-17 4.98E-17 28.0784982

AC010247.1 0.273576197 0.237023984 9.045141933 1.13E-17 5.13E-17 28.04754213

AL080276.2 -0.428850536 0.327885485 -9.043761183 1.14E-17 5.18E-17 28.03743317

AL121574.1 0.223390567 0.158697143 9.042878989 1.15E-17 5.22E-17 28.03097481

AC096540.1 0.316327343 0.498398021 9.040568452 1.17E-17 5.30E-17 28.01406168

AC023115.1 0.121986916 0.089949477 9.039296744 1.18E-17 5.35E-17 28.00475391

AP003043.1 -0.240218193 0.235407125 -9.035281828 1.22E-17 5.51E-17 27.9753736

AC254562.2 -0.215149523 0.260879749 -9.034221294 1.23E-17 5.55E-17 27.96761418

LINC01960 0.175224847 0.159154996 9.030720382 1.26E-17 5.70E-17 27.94200365

MIR5689HG 0.089046178 0.051643205 9.030439376 1.26E-17 5.71E-17 27.93994824

SNHG25 -1.062236329 2.587048038 -9.030357254 1.26E-17 5.71E-17 27.93934757

TTTY4B -0.059500326 0.02903008 -9.028854422 1.28E-17 5.77E-17 27.92835592

POT1-AS1 0.235912433 0.538717041 9.02696722 1.29E-17 5.85E-17 27.9145546

AL035071.1 0.627334592 2.72476718 9.021027733 1.35E-17 6.11E-17 27.87113005

AL390726.2 -0.244371143 0.233586024 -9.020529099 1.36E-17 6.13E-17 27.86748525

SILC1 -0.160203077 0.194424372 -9.015967225 1.40E-17 6.34E-17 27.83414575

LINC01107 -0.070482813 0.054970772 -9.008324763 1.49E-17 6.70E-17 27.77831572

AC104248.1 0.253719315 0.148370528 9.002261491 1.55E-17 7.01E-17 27.73404284

AL390037.1 0.267867329 0.163313126 8.998916345 1.59E-17 7.18E-17 27.70962509

TTTY4C -0.059333872 0.029115727 -8.99528908 1.64E-17 7.37E-17 27.68315435

AL117379.1 0.59996229 1.896584139 8.990716631 1.69E-17 7.62E-17 27.64979535

AC004837.2 0.297440555 0.425320555 8.990623598 1.69E-17 7.63E-17 27.64911672

AC106738.1 0.218094106 0.115530714 8.982519463 1.80E-17 8.09E-17 27.5900179

AC010457.1 0.378172557 0.346819088 8.976419619 1.88E-17 8.46E-17 27.54555698

AC005342.2 0.199662015 0.131179551 8.975316136 1.89E-17 8.53E-17 27.53751584

AC079834.2 0.249979031 0.312207921 8.973439438 1.92E-17 8.64E-17 27.52384166

AF064860.2 -0.375661855 0.752780775 -8.96960575 1.98E-17 8.89E-17 27.49591376

AC010735.2 0.651533049 0.950546973 8.969293961 1.98E-17 8.90E-17 27.49364274

IDI2-AS1 -0.272285277 0.305946489 -8.968775649 1.99E-17 8.93E-17 27.48986756

AC009779.2 0.514753276 3.167986968 8.96874941 1.99E-17 8.93E-17 27.48967645

LINC01545 -0.33901755 0.467205417 -8.968087152 2.00E-17 8.98E-17 27.48485303

AP000919.3 0.088599833 0.053979416 8.966909427 2.01E-17 9.05E-17 27.47627584

AL450322.2 0.09081006 0.049996876 8.964835177 2.05E-17 9.19E-17 27.46117111

AC005759.1 -0.471561586 0.397652779 -8.960569186 2.11E-17 9.48E-17 27.43011289

FZD10-AS1 -0.407530377 0.767094327 -8.958873854 2.14E-17 9.59E-17 27.4177727

AC023946.1 -0.231959208 0.112608096 -8.958373847 2.15E-17 9.62E-17 27.41413347

STAM-AS1 -0.230919328 0.414520052 -8.957159003 2.16E-17 9.71E-17 27.40529191

AL669970.3 0.189447897 0.105095242 8.952864794 2.23E-17 1.00E-16 27.37404489

AC091849.2 -0.323325121 0.433137724 -8.947089651 2.33E-17 1.04E-16 27.33203647

LINC01374 0.20976343 0.169147502 8.945394504 2.36E-17 1.06E-16 27.31970916

AP000786.1 -0.566777626 0.899236822 -8.945359785 2.36E-17 1.06E-16 27.31945669

LINC00484 -0.121757707 0.137939736 -8.94421067 2.38E-17 1.07E-16 27.31110104

AC002310.1 -0.330803234 1.403818582 -8.93966825 2.46E-17 1.10E-16 27.27807788

BX284668.5 -0.761671998 4.241737472 -8.939545944 2.46E-17 1.10E-16 27.27718887

ERLNC1 -0.113856544 0.099068156 -8.938043179 2.49E-17 1.11E-16 27.26626624

SEPTIN7-AS1 -0.241589648 0.758733476 -8.937918412 2.49E-17 1.11E-16 27.26535944

MAPK6-DT 0.111041463 0.079501406 8.936530353 2.52E-17 1.13E-16 27.25527164

AC234772.2 0.287406275 0.231356683 8.932495826 2.59E-17 1.16E-16 27.225956

AC020913.1 0.102820331 0.060836068 8.931417333 2.61E-17 1.17E-16 27.21812086

LINC00426 0.427943974 0.603450457 8.930206705 2.64E-17 1.18E-16 27.20932648

AC246817.1 0.158874794 0.092785814 8.925581152 2.73E-17 1.22E-16 27.17573183

AC091736.1 -0.286484016 0.139077988 -8.924995972 2.74E-17 1.22E-16 27.17148253

AL136084.3 0.69365202 1.473089278 8.924510433 2.75E-17 1.23E-16 27.16795691

AC079921.2 0.445047148 0.808782612 8.920106881 2.84E-17 1.27E-16 27.13598708

LEF1-AS1 0.227578858 0.332309086 8.919646233 2.85E-17 1.27E-16 27.13264335

LINC00528 0.283084327 0.409463949 8.918052332 2.88E-17 1.29E-16 27.12107441

INKA2-AS1 -0.169333522 0.179620159 -8.91737772 2.90E-17 1.29E-16 27.11617829

PSPC1-AS2 0.488137739 1.292168058 8.914948038 2.95E-17 1.31E-16 27.09854635

AL356234.2 0.507456121 0.400347768 8.914477087 2.96E-17 1.32E-16 27.09512906

AC104170.1 0.484033089 0.639393873 8.91343111 2.98E-17 1.33E-16 27.08753969

AC138466.1 0.211795898 0.15269967 8.91043614 3.05E-17 1.36E-16 27.06581194

AL162431.1 0.265658158 0.632538724 8.909956953 3.06E-17 1.36E-16 27.06233599

AC008655.2 -0.595644346 0.859315346 -8.908318451 3.10E-17 1.38E-16 27.0504514

LINC01629 0.355819223 0.197877334 8.905336246 3.17E-17 1.41E-16 27.028824

AC079760.2 0.150465776 0.11245059 8.899267125 3.31E-17 1.47E-16 26.98482378

AC000068.1 -0.300900771 0.699158031 -8.898449186 3.33E-17 1.48E-16 26.97889528

LINC00538 -0.070271152 0.052480937 -8.897419345 3.35E-17 1.49E-16 26.97143138

AP000943.1 0.244414442 0.144525324 8.896023122 3.39E-17 1.50E-16 26.96131293

AC008750.4 0.186448352 0.102167974 8.893776561 3.44E-17 1.53E-16 26.94503416

LINC01063 0.413457039 0.482900034 8.893681971 3.45E-17 1.53E-16 26.94434881

AC073475.1 -0.160873113 0.151248457 -8.891217705 3.51E-17 1.56E-16 26.92649561

LINC00824 -0.123490498 0.083112139 -8.890642756 3.52E-17 1.56E-16 26.92233064

Z97200.1 0.323995873 0.294014271 8.88935057 3.56E-17 1.58E-16 26.91297058

AL596325.2 -0.51982448 1.366170971 -8.88756923 3.60E-17 1.60E-16 26.90006868

AC127496.2 0.357400145 0.293904407 8.885320653 3.66E-17 1.62E-16 26.88378498

LINC01852 0.295669946 0.791053787 8.884764225 3.68E-17 1.63E-16 26.87975585

MIR9-3HG -0.290944909 0.439550752 -8.88211528 3.75E-17 1.66E-16 26.86057684

AC011825.4 0.242863087 0.215990349 8.877382682 3.88E-17 1.72E-16 26.82632058

AL355312.3 -0.340255149 0.478686548 -8.87077338 4.08E-17 1.80E-16 26.77849919

LINC02675 -0.228483023 0.221389798 -8.870546497 4.08E-17 1.81E-16 26.77685798

AP001010.1 0.260138826 0.291133712 8.865806523 4.23E-17 1.87E-16 26.74257635

AC018690.1 -0.364062913 1.491127421 -8.864579502 4.26E-17 1.89E-16 26.73370385

AC087854.1 -0.224384836 0.680788493 -8.863519261 4.30E-17 1.90E-16 26.72603794

AC104971.3 0.465568831 0.246361026 8.861342831 4.37E-17 1.93E-16 26.71030341

MIR924HG 0.308180992 0.351502702 8.860945575 4.38E-17 1.93E-16 26.7074317

AL136984.1 -0.078687937 0.073518624 -8.85740679 4.49E-17 1.98E-16 26.6818539

AC017074.1 0.42099758 0.876839268 8.856019702 4.54E-17 2.00E-16 26.67182999

AC108718.1 0.170112892 0.090446738 8.853636456 4.62E-17 2.04E-16 26.65460955

AL133284.1 -0.053977298 0.032970352 -8.852880827 4.64E-17 2.05E-16 26.64915027

AL353693.1 0.330965656 0.171189111 8.85240418 4.66E-17 2.06E-16 26.64570673

KCTD21-AS1 -0.312362905 0.857057637 -8.847187709 4.84E-17 2.13E-16 26.60802793

AL445649.1 0.375059522 0.335774477 8.842842491 5.00E-17 2.20E-16 26.57665288

GSN-AS1 0.148427987 0.342883958 8.842477714 5.01E-17 2.21E-16 26.57401941

AC006441.1 0.201801841 0.188014951 8.84070575 5.07E-17 2.24E-16 26.5612279

LINC01970 -0.134818369 0.205760509 -8.840274188 5.09E-17 2.24E-16 26.55811276

AC010201.1 -0.463758967 1.100952944 -8.839738015 5.11E-17 2.25E-16 26.55424266

AC016933.1 -0.186774855 0.124953425 -8.839022681 5.14E-17 2.26E-16 26.54907959

SMAD5-AS1 -0.106978293 0.134478252 -8.837611806 5.19E-17 2.28E-16 26.53889708

AC002347.2 0.199400652 0.107516177 8.833486965 5.35E-17 2.35E-16 26.50913333

LINC02626 0.178090236 0.109105845 8.832332302 5.39E-17 2.37E-16 26.50080315

MMP2-AS1 0.616427102 0.639956061 8.830131768 5.48E-17 2.41E-16 26.48492956

SNHG11 -0.467536652 3.241979009 -8.827265809 5.60E-17 2.46E-16 26.46425966

LINC01358 -0.428258716 0.432577501 -8.826076488 5.65E-17 2.48E-16 26.45568326

AP001042.1 -0.230913597 0.217720021 -8.823860533 5.74E-17 2.52E-16 26.43970557

AC087521.2 -0.256972458 0.319669956 -8.816471555 6.05E-17 2.66E-16 26.38644713

AC012306.2 -0.43812302 2.41656264 -8.816397416 6.06E-17 2.66E-16 26.38591289

AC100847.1 0.326917897 0.420741643 8.814289354 6.15E-17 2.70E-16 26.37072359

AC010422.1 0.222189907 0.241804669 8.811876092 6.26E-17 2.75E-16 26.35333803

LINC02446 0.520547209 0.489667791 8.811583718 6.27E-17 2.75E-16 26.35123192

AP001020.1 -0.221087184 0.107330108 -8.811411533 6.28E-17 2.75E-16 26.34999161

LINC01281 0.221305447 0.116333299 8.810423915 6.33E-17 2.77E-16 26.34287774

AC093865.1 0.136826196 0.073741194 8.807318574 6.47E-17 2.83E-16 26.32051306

AP001330.1 -0.156799016 0.109362947 -8.805347801 6.56E-17 2.87E-16 26.30632214

AC096921.2 0.373865081 1.28555013 8.804716695 6.60E-17 2.89E-16 26.30177816

AC211486.5 -0.101610587 0.054867407 -8.802857401 6.68E-17 2.93E-16 26.2883924

AC084357.2 0.379528215 0.411808824 8.801169386 6.77E-17 2.96E-16 26.27624128

AC110741.1 0.22591534 0.137091795 8.799481066 6.85E-17 3.00E-16 26.26408944

AL354824.1 0.33587523 0.198667546 8.79601541 7.03E-17 3.07E-16 26.23914967

AC007879.4 0.245582948 0.164695902 8.795913319 7.03E-17 3.07E-16 26.23841509

AP001001.1 -0.277993749 0.557986146 -8.793487631 7.16E-17 3.13E-16 26.22096302

AC103691.2 -0.29117203 0.270504356 -8.788229294 7.43E-17 3.25E-16 26.18314139

AL138762.1 0.245928838 0.528332469 8.787423783 7.48E-17 3.27E-16 26.17734885

SMILR 0.422652535 0.224122539 8.784335029 7.65E-17 3.34E-16 26.15514033

AC023137.1 0.204555109 0.113962123 8.780518294 7.86E-17 3.43E-16 26.12770435

AL356215.1 -0.561116169 0.733721343 -8.777854026 8.02E-17 3.50E-16 26.10855716

AC099518.4 0.327767219 0.421344925 8.776044966 8.12E-17 3.54E-16 26.09555816

AC138207.7 -0.177389312 0.200167552 -8.775995948 8.13E-17 3.54E-16 26.09520597

AL133342.1 0.433737814 0.522649333 8.775100996 8.18E-17 3.56E-16 26.08877594

CD44-AS1 -0.742522115 1.791729605 -8.77351975 8.27E-17 3.60E-16 26.07741606

BOLA3-AS1 -0.456808032 1.715227718 -8.772876027 8.31E-17 3.62E-16 26.07279184

FOXD1-AS1 -0.198413693 0.096322919 -8.772458154 8.34E-17 3.63E-16 26.06979014

AP006623.1 -0.346834562 1.025913106 -8.769113543 8.54E-17 3.72E-16 26.04576813

AC008243.1 0.148435004 0.124148167 8.767037859 8.67E-17 3.77E-16 26.03086285

RAET1E-AS1 -0.358878552 0.264816485 -8.761319717 9.04E-17 3.93E-16 25.98981302

TDRKH-AS1 -0.408480436 1.094974713 -8.75975594 9.14E-17 3.98E-16 25.97858981

ZMYM4-AS1 0.120503138 0.088685955 8.758720578 9.21E-17 4.00E-16 25.97115974

PARAL1 0.523402243 0.410879684 8.75816472 9.25E-17 4.02E-16 25.96717096

AC136475.5 -0.32689539 0.525984526 -8.754357537 9.51E-17 4.13E-16 25.93985533

AL844908.2 0.407052177 0.575945435 8.754112757 9.52E-17 4.14E-16 25.93809935

ZBTB40-IT1 0.371854778 0.395235172 8.747745431 9.97E-17 4.33E-16 25.89243295

AC100793.3 -0.353806198 0.332342622 -8.74464373 1.02E-16 4.43E-16 25.87019523

AC087857.1 0.729183422 0.402969931 8.743186573 1.03E-16 4.47E-16 25.85974983

LINC01894 0.181593741 0.116171502 8.73497567 1.09E-16 4.75E-16 25.80091198

AC080129.2 0.519527902 0.702941362 8.731212016 1.12E-16 4.87E-16 25.77395406

AC099343.2 0.3637321 0.81915705 8.731013001 1.13E-16 4.88E-16 25.77252878

AC092757.3 0.407561584 0.645780527 8.725555419 1.17E-16 5.08E-16 25.73345146

AC008608.2 0.635566041 1.661843215 8.722012975 1.20E-16 5.21E-16 25.70809522

LINC02576 0.375181809 0.319056923 8.717371584 1.24E-16 5.38E-16 25.67488281

TRBV11-2 0.50102482 0.510566872 8.716547425 1.25E-16 5.41E-16 25.66898655

AF015262.1 0.171571385 0.091493796 8.710772562 1.30E-16 5.64E-16 25.62768158

AC079610.1 0.381692037 0.999593994 8.707721945 1.33E-16 5.76E-16 25.60586894

AC007336.2 0.297365559 0.193957176 8.700163953 1.41E-16 6.09E-16 25.55184849

AL161757.2 0.15325105 0.106777611 8.697713093 1.43E-16 6.19E-16 25.5343375

AC027808.1 -0.236564772 0.174362836 -8.696503825 1.44E-16 6.25E-16 25.52569864

AC103724.4 0.323756204 0.278758097 8.692135636 1.49E-16 6.44E-16 25.49449922

ZNF341-AS1 0.329275706 0.434908985 8.690957868 1.50E-16 6.50E-16 25.48608882

AC002306.1 0.07742978 0.049386411 8.688977754 1.52E-16 6.59E-16 25.47195055

AC008875.1 0.322097079 0.456595753 8.688889671 1.53E-16 6.59E-16 25.47132167

AC100827.3 -0.169194396 0.08213797 -8.686562621 1.55E-16 6.70E-16 25.45470897

AC127521.1 0.460439039 1.151222076 8.685148542 1.57E-16 6.77E-16 25.44461531

AC107027.3 0.285975027 1.054368134 8.6847432 1.57E-16 6.78E-16 25.44172219

AL133453.1 -0.499937019 0.242701976 -8.681018722 1.61E-16 6.97E-16 25.41514279

AL158068.2 0.096825294 0.058185053 8.679286236 1.64E-16 7.05E-16 25.40278155

AC009055.2 -0.092917853 0.048914496 -8.674467222 1.69E-16 7.30E-16 25.36840631

AC137630.2 -0.332008902 0.535794535 -8.671472205 1.73E-16 7.46E-16 25.34704826

DLGAP1-AS2 0.554099816 1.567872352 8.6705025 1.74E-16 7.51E-16 25.34013411

AC091212.1 0.20871701 0.113776535 8.656928175 1.92E-16 8.27E-16 25.2433991

LINC01767 -0.385499272 0.7011938 -8.655341731 1.94E-16 8.37E-16 25.23209993

AC114489.1 0.194984254 0.101221935 8.65445498 1.96E-16 8.42E-16 25.22578478

AC090229.1 0.24516952 0.343621969 8.654430846 1.96E-16 8.42E-16 25.22561291

AC098479.1 0.538459243 0.425471578 8.653363446 1.97E-16 8.48E-16 25.2180118

AP005432.1 -0.061857963 0.071428266 -8.652022377 1.99E-16 8.56E-16 25.20846271

AC008870.3 0.376648471 0.346652168 8.649212782 2.03E-16 8.73E-16 25.18846004

AC011676.3 0.254751976 0.139266436 8.648559 2.04E-16 8.77E-16 25.18380609

AL133406.2 -0.357158895 0.956612513 -8.648502675 2.04E-16 8.77E-16 25.18340515

AL391422.2 0.185086619 0.114105221 8.648101 2.05E-16 8.79E-16 25.18054596

AC145285.3 0.212741419 0.242509919 8.640435202 2.16E-16 9.29E-16 25.12599568

AC021087.1 0.344941511 0.365875719 8.639357665 2.18E-16 9.36E-16 25.11833036

AL590369.1 0.31865479 0.442635945 8.637565548 2.21E-16 9.47E-16 25.10558305

AC008667.1 0.162622498 0.136262579 8.63722874 2.21E-16 9.49E-16 25.10318753

AC133528.1 0.520367532 0.923453848 8.634868117 2.25E-16 9.65E-16 25.08639946

OPA1-AS1 0.148665406 0.118253 8.630554747 2.32E-16 9.95E-16 25.05573163

PPP1R12A-AS1 0.164416725 0.407049864 8.630298022 2.33E-16 9.97E-16 25.05390664

AP001781.1 -0.181826792 0.21224167 -8.629510219 2.34E-16 1.00E-15 25.04830658

OSER1-DT -0.513879443 3.323640846 -8.629029565 2.35E-16 1.01E-15 25.04489003

AC117386.2 0.351594681 0.225235711 8.627583376 2.37E-16 1.02E-15 25.03461108

AL008635.1 0.342750188 0.429168746 8.624672185 2.42E-16 1.04E-15 25.01392282

MYLK-AS1 0.365729902 0.916674669 8.621869671 2.47E-16 1.06E-15 24.99401112

AL645608.6 0.347865931 0.212668161 8.620173981 2.50E-16 1.07E-15 24.98196536

AC009119.1 0.219130227 0.199356701 8.618356987 2.53E-16 1.08E-15 24.96905957

PICSAR 0.623172519 0.341901765 8.607449353 2.74E-16 1.17E-15 24.89162145

HLA-F-AS1 -0.469073898 1.583364681 -8.606712126 2.76E-16 1.18E-15 24.88638983

AC007546.1 -0.34321709 0.63175094 -8.605408009 2.78E-16 1.19E-15 24.87713607

GRID1-AS1 -0.094841337 0.060134684 -8.6036698 2.82E-16 1.20E-15 24.86480348

AL022313.2 0.203517879 0.127140895 8.60366436 2.82E-16 1.20E-15 24.86476488

AC087392.1 0.292334335 0.365533058 8.600379438 2.88E-16 1.23E-15 24.84146275

SLC25A30-AS1 0.206877654 0.248796125 8.593333698 3.03E-16 1.29E-15 24.79150203

NHS-AS1 0.178037557 0.095194646 8.592691644 3.05E-16 1.30E-15 24.78695059

AC073896.3 0.443018504 1.014635047 8.586681558 3.18E-16 1.36E-15 24.74435654

AP003032.1 -0.627421757 0.752213611 -8.584842137 3.22E-16 1.37E-15 24.73132424

LINC00924 0.378800616 0.620477236 8.582267864 3.28E-16 1.40E-15 24.71308854

AC137834.2 -0.208563067 0.203178803 -8.577971935 3.39E-16 1.44E-15 24.68266479

SLC26A4-AS1 -0.127629275 0.106736062 -8.577752711 3.39E-16 1.44E-15 24.68111251

AL137186.2 -0.307861052 1.290803594 -8.576250373 3.43E-16 1.46E-15 24.67047546

AC008443.4 0.432752052 0.418631977 8.572385177 3.52E-16 1.50E-15 24.64311414

AC066613.2 0.069794421 0.037839598 8.570476326 3.57E-16 1.52E-15 24.62960452

PIK3IP1-AS1 0.433018296 0.827490084 8.568602399 3.62E-16 1.54E-15 24.61634396

AC079610.2 0.332442076 0.17194878 8.562788395 3.77E-16 1.61E-15 24.57521401

LINC01396 0.067467743 0.035610242 8.56273204 3.77E-16 1.61E-15 24.57481543

AC008957.1 0.327889048 0.341085959 8.562407046 3.78E-16 1.61E-15 24.57251687

AC012409.1 -0.327352555 0.476295708 -8.560820108 3.83E-16 1.63E-15 24.56129388

AC124057.1 -0.292837945 0.1421626 -8.557607398 3.92E-16 1.66E-15 24.53857737

LINC02772 -0.314690852 0.282282202 -8.556839197 3.94E-16 1.67E-15 24.53314638

LINC02065 0.330576292 0.182336685 8.554334059 4.01E-16 1.70E-15 24.51543785

SFTA3 -0.072949851 0.052770766 -8.549418066 4.15E-16 1.76E-15 24.48069705

LINC01169 0.433200007 0.229080065 8.545804333 4.26E-16 1.81E-15 24.45516744

AC108868.1 0.092366805 0.051687773 8.544830591 4.29E-16 1.82E-15 24.44828953

SMCR5 -0.076734767 0.087932372 -8.543144415 4.34E-16 1.84E-15 24.43638063

AC008147.1 0.269521611 0.190286645 8.538536822 4.49E-16 1.90E-15 24.40384652

FP325332.1 -0.093853592 0.131035957 -8.535751332 4.58E-16 1.94E-15 24.38418376

AC092368.3 -0.456158483 2.118685418 -8.535378104 4.59E-16 1.94E-15 24.38154947

TTTY14 -1.0774995 1.177272383 -8.533779184 4.64E-16 1.97E-15 24.3702649

LINC01971 -0.27654852 0.282001941 -8.531432638 4.72E-16 2.00E-15 24.35370637

AC068594.1 0.149090785 0.083268572 8.530807397 4.74E-16 2.01E-15 24.34929482

AC092376.2 0.169718711 0.335481197 8.530681342 4.75E-16 2.01E-15 24.34840543

AC073641.1 0.142936157 0.105054839 8.529725231 4.78E-16 2.02E-15 24.34165981

AC011481.2 0.371254914 0.644588961 8.528879936 4.81E-16 2.03E-15 24.33569644

TUG1 0.450984548 5.307468172 8.526524454 4.89E-16 2.07E-15 24.31908106

CLCA4-AS1 0.237870825 0.137016341 8.517852533 5.20E-16 2.20E-15 24.25793573

LINC00582 0.261959633 0.214171905 8.516963036 5.23E-16 2.21E-15 24.25166621

AC102953.2 0.362707574 1.218582808 8.514601635 5.32E-16 2.25E-15 24.23502423

COPDA1 0.455287679 0.254371327 8.512693207 5.39E-16 2.28E-15 24.22157676

Z98884.1 0.387453268 0.596254811 8.507200151 5.61E-16 2.37E-15 24.18288168

AC034206.1 0.214701456 0.14174167 8.506989466 5.62E-16 2.37E-15 24.18139786

RAB11B-AS1 -0.562893037 2.786287389 -8.504695632 5.71E-16 2.41E-15 24.16524434

AC022858.1 0.080313037 0.049118545 8.503787902 5.75E-16 2.42E-15 24.15885275

AC012555.1 -0.268926874 0.347459343 -8.502599337 5.80E-16 2.44E-15 24.1504844

AC104109.2 0.259471976 0.602574246 8.501169739 5.86E-16 2.47E-15 24.14042

AL109914.1 0.398702629 0.206042146 8.500558048 5.88E-16 2.48E-15 24.13611403

AC100803.4 -0.558094316 0.906064253 -8.49737886 6.02E-16 2.53E-15 24.1137375

AL354892.3 -0.235418888 0.781580387 -8.497288672 6.02E-16 2.54E-15 24.1131028

LINC01526 0.119021556 0.068502847 8.496639004 6.05E-16 2.55E-15 24.10853085

AC091868.2 0.221918767 0.174984332 8.493915429 6.17E-16 2.60E-15 24.08936656

KRT7-AS 0.944958443 2.592467416 8.493708243 6.17E-16 2.60E-15 24.08790887

AP000424.1 0.331999783 0.346119737 8.493429604 6.19E-16 2.60E-15 24.0859485

AC005520.5 -0.700942327 0.340283041 -8.491952277 6.25E-16 2.63E-15 24.07555543

AL138995.1 0.31143219 0.864438147 8.491384889 6.28E-16 2.64E-15 24.07156414

AL513548.3 -0.05593449 0.045465047 -8.490856237 6.30E-16 2.65E-15 24.06784549

TNFRSF14-AS1 -0.671634666 2.625103882 -8.488474838 6.41E-16 2.69E-15 24.05109612

AP001542.3 -0.666019765 2.61635344 -8.480881431 6.76E-16 2.84E-15 23.99770902

AC044781.1 0.097692578 0.058191692 8.473440929 7.13E-16 2.99E-15 23.94542722

AL049629.1 0.69482578 1.228404599 8.467095176 7.46E-16 3.13E-15 23.90086155

AL807752.3 -0.126108506 0.061221273 -8.465125745 7.56E-16 3.17E-15 23.88703484

AL118516.1 0.597016897 3.02605031 8.463237684 7.67E-16 3.22E-15 23.87378138

MAFTRR -0.220067997 0.302114534 -8.462547369 7.70E-16 3.23E-15 23.86893612

AC018552.2 0.133606131 0.077319355 8.460383918 7.82E-16 3.28E-15 23.85375272

LINC00677 0.180750747 0.150532453 8.459414509 7.88E-16 3.30E-15 23.8469501

AC098487.1 0.39535255 1.098648828 8.458211866 7.95E-16 3.33E-15 23.83851151

AC138150.1 -0.496952443 0.702742923 -8.456648497 8.03E-16 3.37E-15 23.827543

AL031283.3 0.302404472 0.368388597 8.45531774 8.11E-16 3.40E-15 23.81820752

AL672291.1 0.17200802 0.091246039 8.45446045 8.16E-16 3.42E-15 23.812194

AL117350.1 -0.233663993 0.347794388 -8.453193036 8.23E-16 3.45E-15 23.80330437

AL391834.2 0.447404022 2.443791918 8.451188296 8.35E-16 3.49E-15 23.78924492

SMIM15-AS1 0.191558497 0.262593789 8.448554646 8.51E-16 3.56E-15 23.77077818

AL353764.1 0.31336629 0.34027902 8.445784478 8.68E-16 3.63E-15 23.75135828

AC078860.2 0.220956199 0.140302283 8.444485764 8.76E-16 3.66E-15 23.74225525

LIF-AS1 0.1753282 0.103434818 8.441022089 8.97E-16 3.75E-15 23.71798192

AL354707.1 -0.653818161 1.77034386 -8.432948057 9.50E-16 3.97E-15 23.66142471

AP001266.1 -0.211264618 0.102561595 -8.428261054 9.82E-16 4.10E-15 23.62860936

ZEB2-AS1 -0.203318601 0.3113111 -8.427513304 9.88E-16 4.12E-15 23.62337521

LINC01160 -0.249427319 0.522536637 -8.425310007 1.00E-15 4.19E-15 23.60795419

AC135048.3 0.384745969 0.506999685 8.422481033 1.02E-15 4.27E-15 23.58815791

AL359182.1 0.368419903 0.436315087 8.421675824 1.03E-15 4.29E-15 23.5825241

AL136987.1 0.296755345 0.153586712 8.417784768 1.06E-15 4.41E-15 23.55530454

AC018413.1 -0.432876238 1.085778351 -8.417478495 1.06E-15 4.42E-15 23.55316238

AC097381.3 0.223658938 0.197810601 8.414888543 1.08E-15 4.50E-15 23.5350496

AC024941.2 0.396200633 0.691706264 8.414336196 1.08E-15 4.52E-15 23.53118725

AL031658.1 -0.135582108 0.237854862 -8.4130482 1.09E-15 4.56E-15 23.52218142

LINC02487 0.318519256 0.457091411 8.411599417 1.11E-15 4.60E-15 23.51205244

AL353803.4 -0.073566842 0.064282322 -8.406005381 1.15E-15 4.79E-15 23.47295324

AC007314.1 0.113218068 0.080227403 8.404895062 1.16E-15 4.82E-15 23.46519476

AC022558.1 0.287828058 0.429346147 8.403911595 1.17E-15 4.86E-15 23.45832324

AL031058.1 0.497212764 1.677855774 8.403717553 1.17E-15 4.86E-15 23.45696752

ANO1-AS1 -0.226448736 0.109932955 -8.401457219 1.19E-15 4.94E-15 23.44117672

AC007283.1 -0.345370683 0.167665411 -8.400347042 1.20E-15 4.97E-15 23.433422

AC090617.4 0.115430619 0.09058492 8.398248587 1.21E-15 5.05E-15 23.41876588

LRRC8C-DT -0.301761862 0.847384631 -8.397704888 1.22E-15 5.07E-15 23.41496895

LINC00907 -0.195010799 0.177795481 -8.396068419 1.23E-15 5.12E-15 23.40354161

AC005154.4 0.317100397 0.402502157 8.393012712 1.26E-15 5.23E-15 23.38220777

FRGCA 0.318432148 0.234905219 8.390895033 1.28E-15 5.31E-15 23.3674259

AP000941.1 -0.268089557 0.687625036 -8.388923189 1.30E-15 5.38E-15 23.35366421

AL157838.1 0.344265199 0.677184047 8.384763721 1.34E-15 5.54E-15 23.32464186

LIVAR 0.15268854 0.085715753 8.38441019 1.34E-15 5.55E-15 23.32217557

LINC02701 0.258792981 0.32270765 8.380992525 1.37E-15 5.69E-15 23.29833686

AC006272.1 0.189727488 0.196431335 8.377278554 1.41E-15 5.84E-15 23.27243865

LINC00494 0.380520691 0.261328111 8.374081265 1.44E-15 5.97E-15 23.25014944

AC011893.1 0.108170674 0.06097223 8.373962041 1.44E-15 5.97E-15 23.2493184

AP003472.1 0.183710264 0.104251868 8.371266453 1.47E-15 6.08E-15 23.23053122

AC005993.1 0.605382605 0.312386203 8.368562315 1.50E-15 6.20E-15 23.21168847

AC009126.1 0.244291598 0.798720369 8.362854551 1.56E-15 6.45E-15 23.1719293

AC012629.2 -0.423751973 0.403083381 -8.360451649 1.59E-15 6.56E-15 23.15519652

MAGI2-AS3 -0.579233541 2.359665124 -8.359507211 1.60E-15 6.60E-15 23.14862073

AC011595.2 0.17203198 0.111441789 8.352339009 1.68E-15 6.94E-15 23.09872706

LINC02073 0.103465648 0.078283942 8.351939016 1.68E-15 6.96E-15 23.09594378

AC090181.2 0.499604252 1.091107153 8.348990685 1.72E-15 7.10E-15 23.07543104

AC010809.3 -0.197744955 0.262164931 -8.346227457 1.75E-15 7.24E-15 23.0562105

AC011479.2 0.328685179 0.413229048 8.345416358 1.76E-15 7.28E-15 23.05056943

AL020995.1 -0.075100943 0.061281 -8.344200112 1.78E-15 7.34E-15 23.04211131

AC023794.3 0.221101725 0.155532204 8.343813676 1.78E-15 7.36E-15 23.03942409

AP000763.4 -0.129972944 0.063097323 -8.343217565 1.79E-15 7.39E-15 23.03527899

AC010768.1 0.07899234 0.047728389 8.340358511 1.83E-15 7.53E-15 23.01540108

AC093627.6 0.482425767 0.443983656 8.336640214 1.87E-15 7.73E-15 22.98955593

AC008080.4 -0.121902341 0.111898222 -8.336239051 1.88E-15 7.75E-15 22.98676798

AC021504.1 -0.256586097 0.20504596 -8.330771385 1.95E-15 8.05E-15 22.94877841

AL591806.1 0.157052365 0.123933896 8.321260924 2.09E-15 8.61E-15 22.88273872

AL713965.1 0.240600754 0.124693216 8.32032609 2.10E-15 8.66E-15 22.87625003

LINC01731 0.17492356 0.097600174 8.318565445 2.13E-15 8.76E-15 22.86403069

AC040934.1 0.193147392 0.220346111 8.313024904 2.21E-15 9.11E-15 22.82558909

AC026355.3 0.186117202 0.097635311 8.312454609 2.22E-15 9.14E-15 22.82163321

AC138956.1 -0.361531202 0.999952799 -8.311410587 2.24E-15 9.21E-15 22.81439177

AC110792.2 -0.074176015 0.063323478 -8.305621637 2.33E-15 9.59E-15 22.77425002

AC099522.2 0.425884129 1.08146569 8.303699051 2.36E-15 9.71E-15 22.76092252

AC005306.1 -0.591691786 1.026000098 -8.301746479 2.39E-15 9.84E-15 22.74738927

AC092112.1 0.179403634 0.131169472 8.291393996 2.57E-15 1.06E-14 22.67567172

AC118658.1 -0.304478358 0.273727721 -8.289104858 2.62E-15 1.07E-14 22.6598216

AC114488.3 0.141253186 0.082963949 8.284736946 2.70E-15 1.11E-14 22.62958602

AC010503.1 -0.247053115 0.119935662 -8.284190289 2.71E-15 1.11E-14 22.6258027

AFDN-DT -0.413485411 1.42696899 -8.282250216 2.75E-15 1.13E-14 22.61237711

CCAT2 -0.061651903 0.045887713 -8.279274575 2.80E-15 1.15E-14 22.59178932

AL606760.1 -0.327608135 1.077422143 -8.277877903 2.83E-15 1.16E-14 22.58212775

AC012593.1 0.505361998 0.299890737 8.277268253 2.84E-15 1.17E-14 22.57791081

AC005746.1 -0.173715183 0.214439721 -8.273359263 2.92E-15 1.20E-14 22.55087724

AP001381.1 0.153978886 0.128353204 8.272105481 2.95E-15 1.21E-14 22.54220822

AL583785.1 0.807021342 0.957955245 8.270965665 2.97E-15 1.22E-14 22.53432795

LINC02154 0.421391628 0.232915114 8.264303159 3.11E-15 1.27E-14 22.48828033

AL645933.2 0.568346787 2.069677221 8.260517863 3.20E-15 1.31E-14 22.46212945

GPR176-DT -0.053687094 0.04665317 -8.260459702 3.20E-15 1.31E-14 22.4617277

AC124276.1 0.151049286 0.081704869 8.259831689 3.21E-15 1.31E-14 22.45738984

AC005034.4 -0.327944773 0.518446253 -8.251654452 3.40E-15 1.39E-14 22.40092752

AL022323.2 -0.064373623 0.05649706 -8.251064535 3.41E-15 1.40E-14 22.3968557

AC005281.1 0.261872954 0.208699526 8.249430679 3.45E-15 1.41E-14 22.38557924

AC016745.2 -0.059887777 0.055387313 -8.248405574 3.48E-15 1.42E-14 22.37850499

AC025754.1 0.126767041 0.082925447 8.24809542 3.49E-15 1.42E-14 22.37636473

AC105384.1 0.167795881 0.134304638 8.247193329 3.51E-15 1.43E-14 22.37014004

LINC02324 -0.127191294 0.13901139 -8.245907938 3.54E-15 1.45E-14 22.36127127

AL359711.2 0.249729134 0.580884667 8.24428962 3.58E-15 1.46E-14 22.35010671

AC019080.4 0.181044314 0.163880576 8.238571877 3.72E-15 1.52E-14 22.31067253

PROSER2-AS1 -0.108069858 0.2797489 -8.234880852 3.82E-15 1.56E-14 22.28522596

CRPPA-AS1 -0.112399431 0.093735044 -8.231757658 3.91E-15 1.59E-14 22.26370008

SMG7-AS1 -0.177051313 0.536605509 -8.230691536 3.94E-15 1.61E-14 22.25635334

AP003068.1 -0.600057932 1.146174637 -8.230464883 3.94E-15 1.61E-14 22.25479154

AC021028.1 0.424391093 0.815435106 8.225345612 4.08E-15 1.67E-14 22.21952372

AC007556.1 0.403582446 0.211861918 8.22344774 4.14E-15 1.69E-14 22.20645258

AC018645.3 0.453898735 3.395152194 8.223187373 4.15E-15 1.69E-14 22.20465952

AL139339.2 -0.283142845 0.270208926 -8.221285461 4.20E-15 1.71E-14 22.19156287

AP005436.2 0.131433668 0.068522942 8.219002811 4.27E-15 1.74E-14 22.17584712

LINC02273 0.315001474 0.25568508 8.215986216 4.36E-15 1.77E-14 22.15508275

AADACL2-AS1 0.18900976 0.103939626 8.215799723 4.37E-15 1.78E-14 22.15379921

AC079915.1 -0.061120412 0.057197288 -8.215375477 4.38E-15 1.78E-14 22.15087942

AL135787.1 0.134096195 0.080518053 8.213921546 4.42E-15 1.80E-14 22.14087379

AC093151.2 -0.462066792 0.791858617 -8.212472652 4.47E-15 1.82E-14 22.130904

AC090125.1 0.382384983 0.199255618 8.212384217 4.47E-15 1.82E-14 22.13029551

AL583722.3 0.144139425 0.109598111 8.21219148 4.48E-15 1.82E-14 22.12896939

AC116651.1 0.258448766 0.465103808 8.207484001 4.63E-15 1.88E-14 22.09658629

AC018647.1 -0.171959421 0.252899368 -8.20675603 4.65E-15 1.89E-14 22.09157963

AC005618.2 -0.165889004 0.080533317 -8.206303609 4.66E-15 1.89E-14 22.08846824

AC105914.2 0.174587879 0.099152908 8.200552695 4.85E-15 1.97E-14 22.048928

LINC02499 -0.183460139 0.111941718 -8.200021928 4.87E-15 1.98E-14 22.04527967

AL590006.1 0.127272203 0.113630512 8.198104109 4.94E-15 2.00E-14 22.03209848

AC108136.1 0.317435771 0.165300588 8.197825941 4.95E-15 2.01E-14 22.0301868

LINC01771 0.131074082 0.085707036 8.195298809 5.03E-15 2.04E-14 22.01282135

AL592494.1 0.181868365 0.118407846 8.192284099 5.14E-15 2.08E-14 21.99211017

AC099329.1 -0.620290993 0.396273855 -8.192071769 5.15E-15 2.09E-14 21.99065165

AC007128.2 0.295522296 0.162464177 8.191938973 5.15E-15 2.09E-14 21.98973947

AC073525.1 -0.091243723 0.061743282 -8.191629761 5.16E-15 2.09E-14 21.98761552

AC147651.2 -0.648845457 1.186961178 -8.18733535 5.32E-15 2.15E-14 21.95812318

AC010198.1 -0.172196903 0.229805289 -8.18717299 5.33E-15 2.16E-14 21.95700836

AP003170.3 0.373984975 0.253338409 8.179765834 5.61E-15 2.27E-14 21.90616404

AC130324.1 0.208604973 0.235656733 8.175820519 5.76E-15 2.33E-14 21.87909519

AC006435.3 -0.484560574 0.235237248 -8.169716329 6.01E-15 2.43E-14 21.83723162

AC121247.1 0.171461529 0.255320939 8.167635727 6.10E-15 2.47E-14 21.82296731

AC092535.4 -0.302173481 0.311466004 -8.166922722 6.13E-15 2.48E-14 21.81807961

AL121790.2 0.362295545 0.230489542 8.165058398 6.21E-15 2.51E-14 21.80530091

LUCAT1 0.303588606 0.299413898 8.16467135 6.23E-15 2.52E-14 21.8026482

AL162591.2 -0.181080724 0.087908367 -8.162854515 6.31E-15 2.55E-14 21.7901973

AC090589.2 0.137541627 0.092756118 8.162052057 6.34E-15 2.56E-14 21.78469858

LINC02208 -0.062633797 0.045230789 -8.161121039 6.38E-15 2.58E-14 21.77831939

AC069234.3 -0.416648546 0.202268327 -8.157243916 6.56E-15 2.65E-14 21.75175924

AC027088.3 0.093389888 0.04894809 8.156995392 6.57E-15 2.65E-14 21.75005702

AC091588.1 0.442797502 0.646847438 8.156633943 6.58E-15 2.65E-14 21.74758141

AC246785.3 -0.374175789 0.335101315 -8.153315587 6.74E-15 2.72E-14 21.72485698

AC036214.2 0.327415033 1.043348591 8.151937868 6.80E-15 2.74E-14 21.71542407

FO680682.1 0.102593684 0.063131251 8.150444809 6.87E-15 2.77E-14 21.70520265

AC009495.2 -0.275148488 0.353550002 -8.147302194 7.02E-15 2.83E-14 21.68369259

AC104596.1 -0.301006454 0.69860492 -8.145966613 7.09E-15 2.85E-14 21.67455273

AC022167.1 -0.488982688 0.23738403 -8.145327842 7.12E-15 2.87E-14 21.67018174

AC087294.1 -0.174921544 0.384154008 -8.145233216 7.13E-15 2.87E-14 21.66953425

AC010255.2 0.337479104 0.200335505 8.144965775 7.14E-15 2.87E-14 21.66770429

AC107068.1 -0.248895571 1.234601316 -8.144949117 7.14E-15 2.87E-14 21.6675903

AC005090.1 0.304552158 0.173459183 8.142040438 7.28E-15 2.93E-14 21.64769034

AL391097.2 0.169095783 0.089832749 8.141029326 7.34E-15 2.95E-14 21.64077385

AL365226.2 -2.00857092 4.60590249 -8.140684885 7.35E-15 2.95E-14 21.63841784

LINC01713 0.138772361 0.079557891 8.140056061 7.39E-15 2.97E-14 21.63411681

AC109361.1 -0.061468685 0.094584306 -8.138530857 7.46E-15 3.00E-14 21.62368564

AC011840.4 -0.084891896 0.041212047 -8.137501373 7.52E-15 3.02E-14 21.61664554

AC103706.1 0.579032416 1.694693674 8.134301972 7.69E-15 3.08E-14 21.59477037

AC079779.3 -0.103733024 0.053513075 -8.133515989 7.73E-15 3.10E-14 21.58939728

AL590714.1 0.114846425 0.063790395 8.131559154 7.83E-15 3.14E-14 21.57602161

AGAP1-IT1 0.516135679 0.598117092 8.131245357 7.85E-15 3.15E-14 21.5738769

LINC01949 -0.051699462 0.030346943 -8.129686556 7.93E-15 3.18E-14 21.56322377

MAST4-AS1 0.364412532 0.948989224 8.126755441 8.10E-15 3.25E-14 21.54319574

AC098829.1 0.129204825 0.125667949 8.121848139 8.38E-15 3.36E-14 21.50967557

LINC01315 -0.5327663 1.857125859 -8.119317205 8.52E-15 3.41E-14 21.49239295

AC009716.1 0.25246302 0.288292245 8.117062167 8.66E-15 3.47E-14 21.47699737

AC008115.2 0.139807518 0.076726043 8.116693202 8.68E-15 3.48E-14 21.47447866

AC092123.1 0.278585671 0.792093604 8.116341311 8.70E-15 3.48E-14 21.47207656

AATBC 0.254090167 0.433754265 8.116116269 8.72E-15 3.49E-14 21.4705404

AC009320.1 0.091713169 0.04808536 8.114838535 8.79E-15 3.52E-14 21.46181905

SMC5-AS1 0.222206944 0.769351553 8.108222787 9.20E-15 3.68E-14 21.41667717

AC069544.1 0.371464208 1.250591619 8.107752884 9.23E-15 3.69E-14 21.41347179

AP000704.1 -0.389884144 1.017516565 -8.107250508 9.27E-15 3.70E-14 21.41004503

AC130462.2 0.14383484 0.129213466 8.106121502 9.34E-15 3.73E-14 21.4023445

AC108863.2 -0.254204367 0.123407345 -8.102120398 9.60E-15 3.83E-14 21.3750603

AP000894.2 -0.490946633 0.791321693 -8.10083375 9.69E-15 3.87E-14 21.36628837

HCG15 0.236501219 0.442924728 8.099049355 9.81E-15 3.91E-14 21.35412453

AC025430.1 0.170267451 0.182539577 8.097682798 9.90E-15 3.95E-14 21.34481024

IL1R1-AS1 0.106302859 0.058888695 8.08904838 1.05E-14 4.19E-14 21.28598374

AP000820.1 0.082159277 0.043169549 8.086887852 1.07E-14 4.25E-14 21.27127068

AC005089.1 -0.194576968 0.317239939 -8.086823778 1.07E-14 4.25E-14 21.27083438

AC141586.2 0.190225591 0.175866203 8.085311957 1.08E-14 4.30E-14 21.26054062

LINC02205 0.220060322 0.114124448 8.078362132 1.13E-14 4.51E-14 21.21323713

AC010260.1 0.236495494 0.398537413 8.077958967 1.13E-14 4.52E-14 21.21049386

LINC01300 0.566899431 0.302096402 8.073201887 1.17E-14 4.67E-14 21.17813219

AC090340.1 0.532531416 0.374005191 8.07168471 1.18E-14 4.71E-14 21.1678138

AL359513.1 0.524162549 0.829487648 8.0696731 1.20E-14 4.78E-14 21.15413477

AC104958.1 -0.12991974 0.107364328 -8.068042827 1.21E-14 4.83E-14 21.14305056

AL132642.1 0.218610355 0.133718338 8.06354082 1.25E-14 4.98E-14 21.11244939

AP003721.2 0.108120585 0.056527548 8.060510117 1.28E-14 5.09E-14 21.09185554

HDHD5-AS1 -0.429123117 0.846810905 -8.055807316 1.32E-14 5.25E-14 21.05991013

AL590428.1 0.111083374 0.110469095 8.051709991 1.36E-14 5.40E-14 21.03208798

AL162457.2 0.264149247 0.150994768 8.051419285 1.36E-14 5.41E-14 21.03011436

AL356740.3 0.459586697 0.497449933 8.051385498 1.36E-14 5.41E-14 21.02988498

SREBF2-AS1 0.299476737 1.485492184 8.050451121 1.37E-14 5.44E-14 21.02354179

AC044802.1 0.114242559 0.160215774 8.048831906 1.39E-14 5.50E-14 21.01255063

AP001783.1 0.460649194 0.409001201 8.043148261 1.44E-14 5.72E-14 20.97398226

EXOSC10-AS1 0.346632095 0.633482138 8.041846105 1.45E-14 5.77E-14 20.96514864

NCMAP-DT -0.223105644 0.466247109 -8.03973075 1.47E-14 5.85E-14 20.95080049

AC069224.1 -0.375233234 1.121456666 -8.03837207 1.49E-14 5.90E-14 20.94158611

AP001442.1 -0.108931686 0.080256568 -8.036788016 1.50E-14 5.97E-14 20.93084462

KDM2B-DT 0.254320686 0.434025713 8.036659947 1.51E-14 5.97E-14 20.92997625

AL590385.2 0.107123998 0.063521207 8.033649558 1.54E-14 6.09E-14 20.90956697

AC006001.2 0.458204493 1.301623453 8.033082608 1.54E-14 6.12E-14 20.90572386

AC019294.2 -0.121775165 0.145486038 -8.032626117 1.55E-14 6.13E-14 20.90262963

ZDHHC20-IT1 0.293598551 0.411450822 8.032295548 1.55E-14 6.15E-14 20.90038902

AC091806.1 0.247622086 0.168657166 8.032121822 1.55E-14 6.15E-14 20.89921151

AC008443.2 -0.115517792 0.056079851 -8.031491495 1.56E-14 6.18E-14 20.89493936

AL035446.1 0.617829066 0.97778714 8.02832376 1.59E-14 6.31E-14 20.87347292

AC022809.1 0.09881913 0.051741625 8.027924766 1.60E-14 6.33E-14 20.87076952

LINC02690 0.224908411 0.145188173 8.026057724 1.62E-14 6.41E-14 20.85812048

AC103591.3 -0.882821241 1.591896261 -8.022424128 1.66E-14 6.56E-14 20.83350898

AC069503.3 0.159919413 0.083179852 8.022023464 1.67E-14 6.58E-14 20.83079562

AF131215.6 -0.518129714 2.055237349 -8.021948156 1.67E-14 6.58E-14 20.83028563

AC004471.1 0.362482366 0.501356898 8.021592733 1.67E-14 6.60E-14 20.82787875

LINC01229 -0.15333295 0.139982329 -8.015730227 1.74E-14 6.87E-14 20.78818901

AC096992.2 0.255914531 1.147603492 8.014238346 1.76E-14 6.93E-14 20.77809199

AC027801.3 0.132356655 0.068997851 8.013129232 1.77E-14 6.98E-14 20.77058638

OSTN-AS1 0.215021017 0.126549583 8.011162581 1.79E-14 7.08E-14 20.75727936

STK32A-AS1 -0.609827186 1.13313869 -8.009318748 1.82E-14 7.17E-14 20.7448054

LINC01929 0.477005487 0.277807116 8.009289728 1.82E-14 7.17E-14 20.7446091

U91328.1 -0.296683955 1.36121742 -8.005905191 1.86E-14 7.33E-14 20.72171709

AL355482.1 -0.07822123 0.066453614 -8.005152866 1.87E-14 7.37E-14 20.71662949

AL691432.2 0.516222947 2.681522542 8.004365611 1.88E-14 7.40E-14 20.71130602

AC080162.1 0.313070061 0.524842468 8.004348383 1.88E-14 7.40E-14 20.71118953

PITPNM2-AS1 -0.064952608 0.054124948 -8.001082709 1.92E-14 7.57E-14 20.68911071

LHFPL3-AS1 -0.135530561 0.106087022 -7.99764058 1.97E-14 7.75E-14 20.66584558

AL512328.1 0.451518848 0.394301293 7.993079309 2.03E-14 7.99E-14 20.63502681

LGALS8-AS1 -0.169682212 0.297984434 -7.99243504 2.04E-14 8.02E-14 20.6306747

AC112229.4 -0.160411171 0.098731804 -7.981931517 2.19E-14 8.62E-14 20.5597562

AL049569.1 0.166382118 0.140213318 7.98184931 2.19E-14 8.62E-14 20.55920141

STX17-AS1 -0.477854769 1.758613413 -7.981219612 2.20E-14 8.66E-14 20.55495183

AP000763.3 0.28608216 0.282582676 7.977232051 2.26E-14 8.89E-14 20.52804672

AL354920.1 -0.535318275 2.060086686 -7.975781669 2.29E-14 8.98E-14 20.5182629

LINC02783 0.219539538 0.115665839 7.975178419 2.29E-14 9.01E-14 20.51419393

AL589743.4 -0.323376126 0.395268179 -7.973393915 2.32E-14 9.12E-14 20.50215854

AL035661.1 -0.904020536 4.507980446 -7.973242951 2.33E-14 9.13E-14 20.50114046

AL592431.1 0.109871462 0.063920475 7.971174321 2.36E-14 9.25E-14 20.48719131

AC012676.1 -0.234393807 0.615478189 -7.967544591 2.42E-14 9.48E-14 20.46272139

AC087477.5 0.199281243 0.162700872 7.965894689 2.44E-14 9.59E-14 20.45160107

AC087627.1 0.103796339 0.095379888 7.964838089 2.46E-14 9.66E-14 20.44448042

AC005479.1 0.343234316 0.481273027 7.964788706 2.46E-14 9.66E-14 20.44414764

AL627309.4 -0.148972331 0.074270192 -7.963360758 2.49E-14 9.75E-14 20.43452548

AC007671.1 0.13254334 0.129458899 7.962915015 2.49E-14 9.77E-14 20.4315221

AL034345.2 0.088187703 0.046271385 7.962522224 2.50E-14 9.80E-14 20.42887561

AC078850.1 -0.266259578 0.359658636 -7.962480204 2.50E-14 9.80E-14 20.4285925

AC245297.3 -0.5213863 2.494989348 -7.960310422 2.54E-14 9.94E-14 20.41397491

LINC02723 0.217663909 0.144961866 7.960100335 2.54E-14 9.95E-14 20.41255972

AP002449.1 0.268532295 0.355860969 7.955815262 2.62E-14 1.02E-13 20.38370021

DNAAF4-CCPG1 -0.193624971 0.099499337 -7.953714736 2.66E-14 1.04E-13 20.3695573

MKLN1-AS 0.334459091 1.444973156 7.948836773 2.75E-14 1.07E-13 20.33672374

AL135791.1 -0.238273358 0.544395026 -7.947679899 2.77E-14 1.08E-13 20.32893886

LINC01978 0.630435668 1.153485563 7.947153036 2.78E-14 1.09E-13 20.32539373

AC079922.2 0.357265139 1.422084688 7.946045865 2.80E-14 1.09E-13 20.31794438

LMO7DN 0.154734761 0.143706703 7.941710922 2.88E-14 1.13E-13 20.28878459

LINC00589 0.369717438 0.202807959 7.941169062 2.89E-14 1.13E-13 20.28514044

AL133410.2 -0.130757198 0.063478051 -7.93768469 2.96E-14 1.16E-13 20.26171122

AC004528.2 -0.834148652 0.404950065 -7.937051444 2.98E-14 1.16E-13 20.25745398

GSEC 0.376953418 1.001877499 7.931537175 3.09E-14 1.21E-13 20.22039208

CNNM3-DT 0.504032794 1.506967258 7.931394544 3.09E-14 1.21E-13 20.21943368

AC114803.1 0.331692383 0.171563037 7.931380253 3.09E-14 1.21E-13 20.21933766

AC009283.1 0.486909101 3.275929188 7.926286216 3.20E-14 1.25E-13 20.18511644

MIR2117HG 0.189206634 0.112032366 7.92587431 3.21E-14 1.25E-13 20.18234996

AL591468.1 0.181626511 0.101420913 7.921964929 3.30E-14 1.28E-13 20.15609837

AL157931.1 0.277534542 0.143696938 7.921701091 3.30E-14 1.29E-13 20.15432701

LINC02761 -0.545285735 1.32475165 -7.919536564 3.35E-14 1.31E-13 20.13979635

AC009948.3 0.35528387 0.973630952 7.915726802 3.44E-14 1.34E-13 20.11422772

AC125257.2 -0.888452555 0.431312715 -7.912466022 3.52E-14 1.37E-13 20.09235026

DOCK8-AS1 -0.262260292 0.623053825 -7.911681579 3.54E-14 1.38E-13 20.08708814

PTPRD-AS1 0.236279433 0.372381772 7.908176603 3.62E-14 1.41E-13 20.06358086

HOTAIRM1 0.65552347 2.549854116 7.908155229 3.62E-14 1.41E-13 20.06343753

TRMT2B-AS1 0.109188208 0.057076877 7.905475329 3.69E-14 1.43E-13 20.04546879

AL023806.2 -0.25112111 0.121910531 -7.905468379 3.69E-14 1.43E-13 20.04542219

BHLHE40-AS1 -0.576793067 1.384686652 -7.904122162 3.72E-14 1.45E-13 20.03639739

AP000442.1 -0.334614081 1.552832163 -7.898828162 3.86E-14 1.50E-13 20.00091768

AL035045.1 0.093416256 0.056177421 7.897722634 3.89E-14 1.51E-13 19.99351064

AC002551.1 0.07794068 0.050512853 7.895321483 3.95E-14 1.53E-13 19.97742542

AC074131.1 0.150500959 0.078333729 7.89415229 3.98E-14 1.55E-13 19.96959426

AC022973.3 0.097694369 0.067792719 7.892744338 4.02E-14 1.56E-13 19.96016498

FAM66E -0.080343584 0.058608768 -7.89113453 4.07E-14 1.58E-13 19.94938526

AC007216.4 0.271930057 0.510462898 7.889519467 4.11E-14 1.59E-13 19.93857189

LINC01572 -0.153070756 0.299754853 -7.888634598 4.14E-14 1.60E-13 19.93264805

AC090044.1 -0.35568256 0.468028534 -7.885587688 4.22E-14 1.64E-13 19.91225375

AC022182.1 0.191681472 0.185838923 7.884250112 4.26E-14 1.65E-13 19.9033025

Z97192.3 -0.092221641 0.130031303 -7.883538766 4.28E-14 1.66E-13 19.89854249

ARNTL2-AS1 0.140003947 0.099028652 7.88223743 4.32E-14 1.67E-13 19.88983532

AP006216.1 0.058064168 0.033978989 7.876000577 4.50E-14 1.74E-13 19.84811866

AC039056.2 0.622386862 0.91446646 7.875459461 4.52E-14 1.75E-13 19.84450036

LINC01546 0.131298293 0.093724311 7.871420338 4.65E-14 1.80E-13 19.81749724

LINC02000 0.095570526 0.054211019 7.866506083 4.80E-14 1.86E-13 19.78465649

AC005670.1 0.225698434 0.134750746 7.8616021 4.97E-14 1.92E-13 19.75189857

LINC01429 0.149896965 0.105060306 7.861188857 4.98E-14 1.93E-13 19.74913882

AC010132.4 -0.147695973 0.311359975 -7.858775537 5.06E-14 1.96E-13 19.73302397

MIF-AS1 -0.431885627 1.268389727 -7.857940271 5.09E-14 1.97E-13 19.72744732

AL158198.1 0.11158837 0.065889575 7.856647605 5.14E-14 1.98E-13 19.71881765

AC010524.1 0.169610414 0.189985137 7.854492746 5.21E-14 2.01E-13 19.70443428

AC011944.1 0.154611461 0.163538143 7.853731506 5.24E-14 2.02E-13 19.69935377

C8orf87 0.095923577 0.054553402 7.852913942 5.27E-14 2.03E-13 19.69389774

AC117834.1 0.061207751 0.041167261 7.851706727 5.31E-14 2.05E-13 19.68584208

LINC01934 0.136376319 0.121442004 7.848158045 5.44E-14 2.10E-13 19.66216696

AC036108.3 -0.354865263 0.655240219 -7.848133233 5.44E-14 2.10E-13 19.66200146

AC104564.1 0.20008052 0.141194877 7.840530925 5.73E-14 2.21E-13 19.61130765

ACTN1-AS1 0.242903563 0.348674898 7.840367023 5.73E-14 2.21E-13 19.6102151

AC034198.2 -0.339116194 0.673098289 -7.84008141 5.74E-14 2.21E-13 19.60831127

AC090617.2 0.108632145 0.063755516 7.839132146 5.78E-14 2.23E-13 19.60198402

AC036214.1 -0.091418658 0.101254495 -7.837503484 5.84E-14 2.25E-13 19.59112955

SYNE1-AS1 -0.220985358 0.226490169 -7.837262159 5.85E-14 2.25E-13 19.58952134

PRR34-AS1 -0.586637366 3.417304949 -7.837088426 5.86E-14 2.26E-13 19.58836358

AC107308.1 0.413330296 0.255336992 7.836639341 5.88E-14 2.26E-13 19.58537098

AC009806.1 -0.331760056 0.708923202 -7.83662927 5.88E-14 2.26E-13 19.58530386

AC093567.1 0.13204669 0.138262711 7.834665634 5.96E-14 2.29E-13 19.57222002

AL357153.1 0.148088283 0.108156183 7.834228538 5.97E-14 2.30E-13 19.56930792

AL157834.1 0.18534003 0.190145498 7.832992116 6.02E-14 2.32E-13 19.56107104

AC107884.2 0.146833299 0.125468743 7.830927849 6.11E-14 2.35E-13 19.54732118

AC006064.5 -0.439365789 0.213296756 -7.830109397 6.14E-14 2.36E-13 19.54187026

LINC01397 0.09275685 0.056190343 7.827690516 6.24E-14 2.40E-13 19.52576274

AC005021.1 -0.42783948 0.834994427 -7.827074875 6.27E-14 2.41E-13 19.5216637

ATXN2-AS 0.283915278 0.514536645 7.826952992 6.28E-14 2.41E-13 19.5208522

TEX26-AS1 -0.158554593 0.168194978 -7.823190947 6.44E-14 2.47E-13 19.49580899

AC027020.2 -0.479497478 1.444537487 -7.820523051 6.55E-14 2.51E-13 19.4780544

AC063943.1 0.336442382 0.409760746 7.819316757 6.61E-14 2.53E-13 19.47002802

AL031847.1 0.544376138 1.425103959 7.818639958 6.64E-14 2.55E-13 19.46552514

LINC00632 -0.228177299 0.310377195 -7.818121186 6.66E-14 2.55E-13 19.46207383

AL512353.1 -0.239768276 0.662896706 -7.8143191 6.83E-14 2.62E-13 19.43678403

AC090015.1 0.18563746 0.111929974 7.814193709 6.84E-14 2.62E-13 19.43595013

AC010761.3 -0.47890581 1.049959991 -7.813939242 6.85E-14 2.62E-13 19.43425786

AL121845.4 0.37614397 0.572007454 7.81386126 6.85E-14 2.62E-13 19.43373927

LINC00518 0.096360399 0.056022133 7.813748801 6.86E-14 2.63E-13 19.4329914

AC138305.1 0.386607328 0.519807101 7.811053623 6.99E-14 2.67E-13 19.41507037

AC090772.1 -0.294920093 0.508765734 -7.810195704 7.03E-14 2.69E-13 19.40936673

AC022486.1 -0.047827564 0.023301758 -7.809585976 7.05E-14 2.70E-13 19.40531338

AL451042.2 0.443117856 1.039089815 7.806799017 7.19E-14 2.75E-13 19.38678904

AC140125.2 -0.138957414 0.133785782 -7.805275167 7.26E-14 2.78E-13 19.37666228

FIGNL2-DT 0.153916294 0.136224204 7.804023652 7.32E-14 2.80E-13 19.36834636

AL161716.1 0.180487892 0.093763052 7.801093107 7.47E-14 2.85E-13 19.34887745

SNHG3 -0.62913358 3.703363325 -7.797234971 7.67E-14 2.93E-13 19.32325393

LINC02067 -0.06547979 0.062469663 -7.797190559 7.67E-14 2.93E-13 19.32295903

AC092666.1 -0.165599367 0.120592214 -7.796186798 7.72E-14 2.95E-13 19.3162941

AC009133.5 -0.180724378 0.088022367 -7.795916347 7.73E-14 2.95E-13 19.31449842

AC079174.2 -0.344686388 0.985584325 -7.795895208 7.74E-14 2.95E-13 19.31435807

LINC02163 0.448983638 0.231913479 7.793192176 7.88E-14 3.01E-13 19.29641352

AC130456.1 0.154250151 0.132712675 7.792382238 7.92E-14 3.02E-13 19.29103745

AC023794.6 0.254979961 0.338408191 7.790385949 8.03E-14 3.06E-13 19.27778849

AC006435.2 -0.468834574 1.628864777 -7.788316129 8.14E-14 3.10E-13 19.26405403

AL391095.1 -0.217289405 0.389679953 -7.787584936 8.18E-14 3.12E-13 19.25920275

AL442125.2 0.341437676 0.552900829 7.786476351 8.24E-14 3.14E-13 19.25184818

LINC00113 0.227983066 0.123953186 7.78566675 8.29E-14 3.16E-13 19.24647758

AC011294.1 0.276509944 0.201906377 7.784497182 8.35E-14 3.18E-13 19.2387198

AC027277.2 0.159950149 0.111447345 7.783910669 8.38E-14 3.19E-13 19.23482974

FOXP1-AS1 0.09511477 0.066705638 7.783306445 8.42E-14 3.20E-13 19.23082243

Z97056.1 0.158927147 0.107378379 7.779598136 8.63E-14 3.28E-13 19.20623309

LINC01770 0.704815405 2.216453908 7.775664589 8.86E-14 3.37E-13 19.1801592

AC092894.1 0.132857673 0.085602576 7.775004569 8.90E-14 3.38E-13 19.1757851

LINC01659 -0.419104407 0.609529604 -7.773391309 9.00E-14 3.42E-13 19.16509475

HAR1A 0.487322189 0.596493314 7.771646229 9.10E-14 3.46E-13 19.15353265

LINC02099 0.053537996 0.046229314 7.770948183 9.15E-14 3.48E-13 19.14890823

AP000238.1 0.375036507 0.714954599 7.770536167 9.17E-14 3.48E-13 19.14617883

LINC02178 0.383723615 0.200987143 7.764072678 9.58E-14 3.64E-13 19.10337482

EIF2AK3-DT -0.248762971 1.070614103 -7.763750299 9.60E-14 3.64E-13 19.10124054

AC145285.6 -0.303026006 0.92360004 -7.761401791 9.75E-14 3.70E-13 19.08569434

AL022341.1 0.396866032 0.67758309 7.756845769 1.01E-13 3.82E-13 19.05554468

LINC02828 0.176736126 0.134197817 7.756799838 1.01E-13 3.82E-13 19.05524079

AP001021.3 0.192637186 0.204840102 7.756354753 1.01E-13 3.83E-13 19.0522961

MIR194-2HG 0.643475172 1.285883628 7.753501921 1.03E-13 3.90E-13 19.03342453

LINC00653 -0.416391504 1.488427463 -7.752201191 1.04E-13 3.93E-13 19.02482177

AC024145.1 0.197474913 0.328290486 7.747313379 1.07E-13 4.06E-13 18.99250387

AL513218.1 -0.476008163 1.219045439 -7.745111885 1.09E-13 4.12E-13 18.9779524

AC090970.2 0.205491118 0.303355298 7.742596425 1.11E-13 4.19E-13 18.96132924

AC026462.3 0.402888144 0.455809171 7.73969066 1.13E-13 4.27E-13 18.9421315

AC022558.2 -0.1211856 0.091733571 -7.737117793 1.15E-13 4.34E-13 18.92513737

AC009878.1 0.48796928 0.585304238 7.736042532 1.16E-13 4.37E-13 18.9180363

IATPR 0.094552161 0.054245629 7.732504789 1.18E-13 4.48E-13 18.8946778

AC027307.1 0.318654021 0.403225654 7.731474836 1.19E-13 4.51E-13 18.88787879

AC008619.1 0.263726372 0.214980804 7.727569881 1.22E-13 4.63E-13 18.86210685

AL359853.1 0.543231347 0.354868187 7.726755226 1.23E-13 4.65E-13 18.85673144

AC010632.1 0.147805125 0.14259436 7.726716713 1.23E-13 4.65E-13 18.85647733

EXOC3-AS1 0.447802673 1.974167964 7.725379158 1.24E-13 4.69E-13 18.84765252

AC010894.4 0.077009631 0.049421357 7.724312635 1.25E-13 4.72E-13 18.84061667

AC108063.1 0.161774962 0.157164927 7.721952472 1.27E-13 4.80E-13 18.82504911

MIR4435-2HG -0.592339298 3.372887297 -7.720491475 1.28E-13 4.84E-13 18.81541411

AL161733.1 0.197660744 0.104832984 7.717557299 1.31E-13 4.94E-13 18.79606762

SMIM10L2B-AS1 -0.114430567 0.076657883 -7.716609723 1.32E-13 4.97E-13 18.78982089

AP003696.1 -0.111955016 0.092082804 -7.712190188 1.36E-13 5.11E-13 18.76069299

AL139260.1 0.317883695 0.918617831 7.711063402 1.37E-13 5.15E-13 18.75326855

AC013391.3 -0.115865698 0.070153332 -7.708670709 1.39E-13 5.23E-13 18.73750551

HHATL-AS1 -0.136926177 0.116904989 -7.708657665 1.39E-13 5.23E-13 18.73741958

AC007879.1 0.055720077 0.030847039 7.705836608 1.41E-13 5.33E-13 18.71883894

AC027117.2 -0.553466276 0.890852763 -7.705549932 1.42E-13 5.34E-13 18.71695105

AL139420.1 0.197993914 0.107816501 7.704684207 1.43E-13 5.37E-13 18.71125013

AC090517.2 0.285106092 0.87594537 7.701919789 1.45E-13 5.47E-13 18.69304911

AL049775.2 0.072103247 0.037995371 7.701421148 1.46E-13 5.48E-13 18.68976653

AL158835.2 0.343156707 0.441152847 7.701361182 1.46E-13 5.48E-13 18.68937178

AC027097.2 0.296355822 0.717085889 7.701124469 1.46E-13 5.49E-13 18.68781355

AC093525.3 -0.321762905 0.209369802 -7.700052033 1.47E-13 5.53E-13 18.68075435

AL137244.1 -0.255853114 0.690612942 -7.699858071 1.47E-13 5.53E-13 18.67947769

AC010320.4 -0.071043168 0.079237856 -7.697897945 1.49E-13 5.61E-13 18.66657737

AC026689.1 0.291366394 0.463308856 7.696290554 1.51E-13 5.67E-13 18.65600027

AC068733.1 -0.052510571 0.02941149 -7.694559454 1.53E-13 5.73E-13 18.64461087

AC007991.4 0.156818012 0.087824128 7.694480087 1.53E-13 5.73E-13 18.64408873

AC130456.5 -0.331849134 0.161101171 -7.692616947 1.54E-13 5.80E-13 18.6318327

AC034102.3 0.160218886 0.107120889 7.690781318 1.56E-13 5.87E-13 18.6197597

U73169.1 0.242009778 0.309978308 7.690342267 1.57E-13 5.89E-13 18.61687234

AC093801.1 0.132830539 0.075515437 7.686349959 1.61E-13 6.04E-13 18.59062286

AC068051.1 0.052660138 0.029797151 7.682409981 1.65E-13 6.20E-13 18.56472687

LINC01714 0.292507851 0.20973673 7.679332176 1.69E-13 6.33E-13 18.54450414

AC092431.1 0.159781779 0.147755383 7.672467441 1.77E-13 6.62E-13 18.49942002

LINC00705 0.178378573 0.152267654 7.668283319 1.82E-13 6.81E-13 18.47195479

LINC01926 0.071083622 0.039186268 7.665789157 1.85E-13 6.92E-13 18.45558776

AC004551.1 0.098210028 0.055322861 7.664825855 1.86E-13 6.96E-13 18.44926745

AL137003.1 0.333158168 1.436561395 7.664637126 1.86E-13 6.97E-13 18.44802925

AC139768.1 0.345287076 1.921430053 7.663498169 1.88E-13 7.02E-13 18.44055731

AC122134.1 0.039837965 0.021393758 7.663440129 1.88E-13 7.02E-13 18.44017656

AC079949.2 0.410111726 0.598053877 7.662890447 1.88E-13 7.05E-13 18.43657077

AC005332.5 -0.381395042 2.130178705 -7.662606488 1.89E-13 7.06E-13 18.43470813

AL121956.1 0.057125471 0.030288783 7.662482361 1.89E-13 7.06E-13 18.43389392

AC090618.1 0.108692952 0.082455796 7.658640556 1.94E-13 7.24E-13 18.40869854

AC106876.1 0.751711833 1.740941616 7.658133718 1.94E-13 7.26E-13 18.40537526

AC010729.2 0.05202665 0.029472737 7.657647441 1.95E-13 7.29E-13 18.40218694

RNF217-AS1 -0.076165565 0.176603455 -7.657391494 1.95E-13 7.30E-13 18.40050885

AC074033.1 0.299234075 0.319310769 7.656736472 1.96E-13 7.33E-13 18.39621447

AC011379.1 -0.06830734 0.120922056 -7.655130049 1.98E-13 7.40E-13 18.38568371

AL592078.1 -0.15636028 0.075907454 -7.649745733 2.05E-13 7.67E-13 18.35039872

AC124283.3 -0.257187848 0.468806731 -7.648842839 2.07E-13 7.72E-13 18.34448352

LINC00501 0.224721256 0.183432457 7.646514744 2.10E-13 7.83E-13 18.32923356

LINC01170 0.147212685 0.096507144 7.645979029 2.11E-13 7.86E-13 18.32572487

AL031717.1 -0.352577504 1.040957011 -7.643792565 2.14E-13 7.97E-13 18.31140636

AC026992.1 -0.166106838 0.157772999 -7.642890667 2.15E-13 8.02E-13 18.30550094

AC004543.1 0.23754704 0.123121974 7.642662568 2.15E-13 8.03E-13 18.30400747

PLCE1-AS1 -0.146167746 0.211483404 -7.640969937 2.18E-13 8.12E-13 18.29292608

AC010531.3 0.221989357 0.28204442 7.638896926 2.21E-13 8.23E-13 18.27935677

AL137230.1 -0.287710966 0.305317836 -7.635215494 2.26E-13 8.43E-13 18.25526565

AC253536.3 0.364214904 0.723547405 7.632694825 2.30E-13 8.57E-13 18.23877526

AP000254.2 0.403667855 2.498229479 7.628416215 2.37E-13 8.81E-13 18.21079316

DIRC3-AS1 0.084834181 0.057596774 7.627423657 2.38E-13 8.87E-13 18.20430341

AL513123.1 0.193114832 0.100260053 7.62634116 2.40E-13 8.93E-13 18.1972263

AC002378.1 0.120264863 0.062776203 7.623256086 2.45E-13 9.11E-13 18.17706072

AC007922.2 0.123555408 0.07317142 7.622204194 2.47E-13 9.17E-13 18.17018635

LINC02310 0.072426654 0.038161775 7.621111778 2.48E-13 9.24E-13 18.16304787

AC097504.2 0.273711833 0.380076758 7.62076697 2.49E-13 9.26E-13 18.16079484

ROR1-AS1 0.178110856 0.216448768 7.615835312 2.57E-13 9.56E-13 18.12857858

AC104532.2 0.35870551 0.741720805 7.615065163 2.59E-13 9.61E-13 18.12354888

AC024270.2 0.13235848 0.118591553 7.612395056 2.63E-13 9.78E-13 18.10611374

AC018697.1 0.074240403 0.044184144 7.611872256 2.64E-13 9.81E-13 18.10270049

AL359979.1 0.059707918 0.034542645 7.611081116 2.66E-13 9.86E-13 18.09753562

AC005609.5 -0.140516555 0.10040808 -7.61099484 2.66E-13 9.86E-13 18.09697241

AL355388.2 0.306670201 0.754463023 7.609413292 2.68E-13 9.96E-13 18.08664868

AC084871.1 0.163652358 0.149074662 7.609281892 2.69E-13 9.97E-13 18.08579102

AL137798.1 0.168910515 0.131213131 7.604755755 2.77E-13 1.03E-12 18.05625497

AC007750.1 0.222000218 0.389714178 7.602471889 2.81E-13 1.04E-12 18.04135597

GATA3-AS1 0.134619285 0.092278567 7.59990505 2.86E-13 1.06E-12 18.02461478

AL136307.1 0.225223587 0.153970448 7.599744168 2.86E-13 1.06E-12 18.02356562

LRP1-AS 0.1597392 0.131522786 7.593977114 2.97E-13 1.10E-12 17.98596752

AC020907.4 -0.515120539 1.857314141 -7.593323092 2.99E-13 1.11E-12 17.98170493

AC092757.2 0.321580958 0.652196846 7.592561092 3.00E-13 1.11E-12 17.97673893

AC109449.1 -0.228092956 0.664217741 -7.592102696 3.01E-13 1.11E-12 17.9737517

AC010969.1 -0.148761809 0.169419805 -7.591805505 3.02E-13 1.12E-12 17.97181506

AC087289.1 -0.197472829 0.389755797 -7.587738053 3.10E-13 1.15E-12 17.94531508

AC116366.1 -0.301652562 1.303104041 -7.587340013 3.11E-13 1.15E-12 17.94272234

AL133467.1 0.295513496 0.305735297 7.586705015 3.12E-13 1.15E-12 17.93858632

AP000757.2 -0.751826827 1.747423514 -7.585741678 3.14E-13 1.16E-12 17.93231214

LINC02765 0.280331623 0.158206498 7.585355285 3.15E-13 1.16E-12 17.92979573

LINC01506 0.31973131 0.309971703 7.585268297 3.15E-13 1.16E-12 17.92922923

AL034346.1 0.141338104 0.152114919 7.585209961 3.15E-13 1.16E-12 17.92884933

AC026523.1 -0.244325953 0.182268213 -7.584018851 3.18E-13 1.17E-12 17.92109285

IL21R-AS1 -0.131527348 0.150617335 -7.583873234 3.18E-13 1.17E-12 17.92014465

AC090912.3 0.336444861 0.268178517 7.582991738 3.20E-13 1.18E-12 17.91440501

AC018695.6 0.432928388 0.404210315 7.581114967 3.24E-13 1.19E-12 17.90218646

AC110760.2 -0.090578775 0.082755998 -7.578613152 3.29E-13 1.21E-12 17.88590197

AP003071.3 0.202446284 0.142697412 7.577225527 3.32E-13 1.22E-12 17.87687147

LINC02006 -0.094937747 0.097947463 -7.575102571 3.37E-13 1.24E-12 17.86305781

AC015819.2 0.387967834 0.235728007 7.572859225 3.42E-13 1.26E-12 17.8484638

LINC00668 -1.117556214 0.852311154 -7.570443599 3.47E-13 1.28E-12 17.83275247

MESTIT1 -0.13393719 0.325334345 -7.569019434 3.51E-13 1.29E-12 17.82349132

AL008633.1 0.182273175 0.114624676 7.567922086 3.53E-13 1.30E-12 17.81635627

C9orf139 -0.208213039 0.491712332 -7.563666728 3.63E-13 1.34E-12 17.78869453

XIST -2.195855913 2.542737271 -7.563207072 3.64E-13 1.34E-12 17.78570723

AC018809.1 -0.361303748 1.08631861 -7.562763415 3.65E-13 1.34E-12 17.78282402

AC011586.2 0.307553245 0.159142608 7.558961504 3.75E-13 1.38E-12 17.75812139

ZBED5-AS1 -0.454619427 3.329653539 -7.55741499 3.78E-13 1.39E-12 17.74807558

AC025434.1 0.081802023 0.04298573 7.557391781 3.79E-13 1.39E-12 17.74792483

WDR86-AS1 0.421831235 0.710429314 7.5568388 3.80E-13 1.40E-12 17.74433315

IER3-AS1 0.423412114 1.17979882 7.556553621 3.81E-13 1.40E-12 17.74248095

AC006249.1 0.129547992 0.070208568 7.556104317 3.82E-13 1.40E-12 17.73956288

AC012038.2 0.089003531 0.046691157 7.554744238 3.85E-13 1.41E-12 17.73073042

AL354836.1 0.629209356 2.844857613 7.552521822 3.91E-13 1.44E-12 17.7163003

AL807752.5 0.193585308 0.26192459 7.552198752 3.92E-13 1.44E-12 17.71420287

AC114763.1 0.171036569 0.416095172 7.550829388 3.95E-13 1.45E-12 17.70531339

CLRN1-AS1 -0.051599389 0.033680325 -7.550457603 3.96E-13 1.45E-12 17.70290008

AC087855.2 -0.22064296 0.169415153 -7.548891662 4.00E-13 1.47E-12 17.69273626

AL713998.1 -0.060061693 0.039084902 -7.547314352 4.05E-13 1.48E-12 17.68250016

AC127070.1 -0.369348868 0.789247805 -7.547205605 4.05E-13 1.48E-12 17.6817945

AC004870.2 0.676247566 1.004877291 7.545879337 4.08E-13 1.50E-12 17.67318884

THAP9-AS1 -0.481433511 4.177765568 -7.545114007 4.10E-13 1.50E-12 17.6682234

AC106795.3 -0.305159287 0.327777866 -7.543295295 4.15E-13 1.52E-12 17.65642507

LINC00504 -0.072469266 0.104815483 -7.540237078 4.24E-13 1.55E-12 17.63659042

AC099066.1 0.074253004 0.041205934 7.537716936 4.31E-13 1.58E-12 17.62024988

AC012213.3 0.266759116 0.571929721 7.53748187 4.32E-13 1.58E-12 17.61872592

AL139021.1 0.18523559 0.204037065 7.53604169 4.36E-13 1.59E-12 17.60938979

AP003469.4 -0.503206214 2.585625158 -7.535135224 4.38E-13 1.60E-12 17.60351418

AL137247.2 -0.254328171 0.126857478 -7.52862849 4.57E-13 1.67E-12 17.56135311

LINC00111 0.151577845 0.095499446 7.524861867 4.69E-13 1.71E-12 17.53695881

AP000851.1 0.242993384 0.133075042 7.522447823 4.76E-13 1.74E-12 17.521329

AC116903.2 0.063644335 0.053803248 7.520062738 4.84E-13 1.77E-12 17.50589021

AC011921.1 0.221877052 0.410459146 7.519748818 4.85E-13 1.77E-12 17.50385845

WDR7-OT1 -0.178612121 0.096436729 -7.516046094 4.97E-13 1.81E-12 17.47989818

AC022150.1 -0.107290469 0.053208436 -7.513833556 5.04E-13 1.84E-12 17.46558493

AC009093.5 0.187208382 0.118043107 7.513086659 5.07E-13 1.85E-12 17.46075381

AP002993.1 0.190295136 0.098809221 7.511761408 5.11E-13 1.86E-12 17.45218261

MYO16-AS1 0.508142216 0.265281056 7.511639997 5.11E-13 1.87E-12 17.45139743

LINC02298 0.521586998 1.897863398 7.511123982 5.13E-13 1.87E-12 17.44806038

AC090589.3 0.406467767 1.665147212 7.509117882 5.20E-13 1.90E-12 17.43508857

AL138962.1 0.230672812 0.152112237 7.507224249 5.26E-13 1.92E-12 17.42284628

AL353622.1 -0.458193675 1.695543683 -7.507152708 5.27E-13 1.92E-12 17.42238381

AL022344.1 0.356484657 0.810605487 7.505607675 5.32E-13 1.94E-12 17.41239692

AC104695.4 0.873281443 1.265062815 7.503114498 5.41E-13 1.97E-12 17.39628445

GEMIN7-AS1 0.342401055 0.959916515 7.502911593 5.41E-13 1.97E-12 17.39497332

AC092803.1 0.319434703 1.200398301 7.502515478 5.43E-13 1.98E-12 17.39241379

AL136115.1 0.196820409 0.208708154 7.501331394 5.47E-13 1.99E-12 17.38476328

SALRNA2 -0.060135143 0.047586451 -7.49999655 5.52E-13 2.01E-12 17.37613974

AC007342.5 0.363623482 0.542868014 7.498683538 5.57E-13 2.03E-12 17.36765831

AC023490.4 0.110370574 0.057685246 7.497171378 5.62E-13 2.05E-12 17.3578918

CLMAT3 0.0841421 0.087741625 7.495547501 5.68E-13 2.07E-12 17.34740534

AC021739.3 0.216207753 0.270696579 7.491772681 5.82E-13 2.12E-12 17.32303508

AL355304.1 0.160253081 0.168603793 7.490457234 5.88E-13 2.14E-12 17.31454462

LINC00345 0.14341316 0.082185321 7.488482505 5.95E-13 2.16E-12 17.30180089

AC019080.6 0.30367525 1.094900383 7.487414267 5.99E-13 2.18E-12 17.29490812

AC009812.3 -0.090993714 0.215476177 -7.486884582 6.01E-13 2.18E-12 17.29149061

AL392048.1 0.171504473 0.196504849 7.485343704 6.08E-13 2.21E-12 17.28154989

AC006504.7 0.371129572 1.531432223 7.483572853 6.15E-13 2.23E-12 17.27012736

AC021594.1 0.152228641 0.092655394 7.478998192 6.33E-13 2.30E-12 17.2406284

ARHGAP26-AS1 0.132050478 0.089732521 7.47406678 6.54E-13 2.37E-12 17.20884352

AL606468.1 0.123655438 0.080702257 7.469956788 6.72E-13 2.44E-12 17.18236454

AP000253.1 -0.071949741 0.121179952 -7.465311262 6.93E-13 2.51E-12 17.15244797

LAMP5-AS1 0.060560899 0.047266569 7.464437959 6.97E-13 2.53E-12 17.14682551

AC112206.2 -0.12676844 0.118210386 -7.462741952 7.04E-13 2.55E-12 17.13590772

AC007193.2 -0.238471115 0.276903627 -7.455970783 7.36E-13 2.67E-12 17.09233711

C5orf66-AS1 0.378662103 0.253376513 7.452790126 7.52E-13 2.72E-12 17.0718803

LINC02255 0.162926449 0.084727077 7.450984684 7.60E-13 2.75E-12 17.06027117

AC007001.1 0.044651668 0.025509568 7.449723248 7.67E-13 2.78E-12 17.05216124

EIPR1-IT1 0.165377208 0.158848611 7.449352134 7.69E-13 2.78E-12 17.04977549

AL450263.1 0.408029373 0.865816135 7.444489595 7.93E-13 2.87E-12 17.01852401

AC015914.1 0.114976038 0.12435791 7.442220633 8.05E-13 2.91E-12 17.00394646

LINC01465 0.228599264 0.660964288 7.442038715 8.06E-13 2.92E-12 17.00277782

AC020891.3 0.097292758 0.050956253 7.44006243 8.17E-13 2.95E-12 16.99008349

LINC00467 -0.384552965 2.445812092 -7.439096739 8.22E-13 2.97E-12 16.98388142

AC018761.1 0.167726067 0.087196647 7.434300711 8.48E-13 3.06E-12 16.95308796

LINC02725 0.442606743 0.245008426 7.432952637 8.55E-13 3.09E-12 16.94443507

AL035448.1 -0.14328232 0.333887971 -7.428857755 8.78E-13 3.17E-12 16.91815819

P3H2-AS1 0.303550792 0.220333917 7.426522735 8.92E-13 3.22E-12 16.90317904

AP002383.2 -0.285612199 0.147722638 -7.423941214 9.07E-13 3.27E-12 16.88662255

USP12-AS2 0.272943751 0.245544316 7.423187786 9.11E-13 3.29E-12 16.88179126

LINC02513 0.093101751 0.051726083 7.421787969 9.20E-13 3.32E-12 16.87281598

GPRACR 0.066006141 0.054459454 7.418853283 9.37E-13 3.38E-12 16.85400348

AC004771.1 0.32419085 0.786728398 7.414671069 9.63E-13 3.47E-12 16.82720313

FAM99B -0.140102832 0.083567294 -7.4115224 9.83E-13 3.55E-12 16.80703313

AC019186.1 0.300448042 0.565439478 7.406965087 1.01E-12 3.65E-12 16.77785051

AL137847.2 0.262614754 0.402763095 7.406847513 1.01E-12 3.65E-12 16.77709781

CTBP1-DT -0.32048541 2.681832377 -7.406666181 1.01E-12 3.66E-12 16.77593693

AL158801.2 0.117328621 0.094108726 7.406530243 1.02E-12 3.66E-12 16.77506668

TRAM2-AS1 0.289989332 2.306987834 7.405200198 1.02E-12 3.69E-12 16.76655259

AC129492.2 0.229670498 0.14270222 7.403940607 1.03E-12 3.72E-12 16.75849052

AC007923.4 0.061152865 0.036757063 7.402822134 1.04E-12 3.75E-12 16.75133252

AL132655.1 -0.077656884 0.072351397 -7.400454446 1.06E-12 3.80E-12 16.73618236

LINC01825 0.074938566 0.04223522 7.400150203 1.06E-12 3.81E-12 16.73423585

AL157756.1 -0.161353332 0.243848604 -7.397595123 1.08E-12 3.87E-12 16.71789107

AL096678.1 0.187170861 0.197007005 7.395947322 1.09E-12 3.91E-12 16.70735229

AC138035.2 -0.15091386 0.073263407 -7.393323152 1.11E-12 3.98E-12 16.6905725

C5orf64 -0.092824246 0.073037195 -7.39209247 1.12E-12 4.01E-12 16.68270461

AC011747.1 0.139502943 0.07836684 7.390449327 1.13E-12 4.05E-12 16.67220129

Z95115.1 0.342200323 1.639329573 7.390366729 1.13E-12 4.05E-12 16.67167335

AC020922.4 0.163747152 0.11781936 7.385956974 1.16E-12 4.17E-12 16.64349385

AC009121.3 0.135509316 0.142555463 7.382162787 1.19E-12 4.27E-12 16.61925776

AC007728.3 0.127386992 0.127527595 7.375555631 1.24E-12 4.46E-12 16.57707487

AC010997.5 -0.426422821 0.605898647 -7.373619617 1.26E-12 4.51E-12 16.56471973

LINC01341 -0.295034579 0.664087407 -7.367193421 1.31E-12 4.70E-12 16.5237263

AL031727.2 0.104878514 0.054859389 7.366583307 1.32E-12 4.72E-12 16.51983567

AL033384.1 0.19388773 0.197023549 7.366197272 1.32E-12 4.73E-12 16.51737408

C9orf170 -0.071938499 0.110242488 -7.365361776 1.33E-12 4.76E-12 16.51204678

AC008629.1 -0.225024155 0.234007093 -7.3643663 1.34E-12 4.78E-12 16.5057

AC091117.1 -0.217137227 0.105412542 -7.364053136 1.34E-12 4.79E-12 16.50370351

AL031599.1 0.036703903 0.02113705 7.362244066 1.35E-12 4.85E-12 16.49217151

TTTY2B -0.116126223 0.056474926 -7.358069303 1.39E-12 4.98E-12 16.46556714

AC019118.1 0.101738814 0.060548357 7.357822673 1.39E-12 4.99E-12 16.4639958

AC130650.1 0.084840904 0.047867117 7.356380466 1.41E-12 5.03E-12 16.45480787

AC010655.4 -0.271020888 0.209382725 -7.347300665 1.49E-12 5.33E-12 16.3969929

AC022034.3 0.144598792 0.101973891 7.346304646 1.50E-12 5.37E-12 16.39065398

AC103681.1 0.105282202 0.063671849 7.340965692 1.55E-12 5.55E-12 16.3566862

FAM224A -0.079275706 0.039025701 -7.340952432 1.55E-12 5.55E-12 16.35660186

PRRT3-AS1 -0.47438379 1.799135386 -7.335855987 1.61E-12 5.74E-12 16.32419381

AL354861.2 0.216212844 0.120724044 7.335231648 1.61E-12 5.76E-12 16.3202248

CLEC12A-AS1 -0.043393275 0.040256746 -7.332336404 1.64E-12 5.87E-12 16.30182252

AC008149.1 0.107336549 0.095687601 7.329401704 1.67E-12 5.98E-12 16.28317486

AC006480.2 -0.24275588 0.830407733 -7.328849737 1.68E-12 6.00E-12 16.27966817

LINC00605 0.280572015 0.589160779 7.327444297 1.69E-12 6.05E-12 16.27074014

AL357054.2 0.137210768 0.098892276 7.322449514 1.75E-12 6.25E-12 16.23902102

AC087241.2 0.0970661 0.08246215 7.321196664 1.76E-12 6.30E-12 16.23106733

AC009269.5 0.320539476 0.444444406 7.319495223 1.78E-12 6.36E-12 16.22026736

AC013480.1 0.081159146 0.042654947 7.318925543 1.79E-12 6.39E-12 16.2166517

AP002812.5 -0.198883358 0.421835278 -7.313498874 1.85E-12 6.61E-12 16.1822199

AC023300.2 0.275028718 0.190053274 7.306687735 1.94E-12 6.91E-12 16.13903012

LINC00390 0.13553375 0.097850213 7.30552221 1.95E-12 6.96E-12 16.13164241

CHKB-DT 0.373061579 1.150572242 7.302607951 1.99E-12 7.09E-12 16.11317408

AL009181.1 -0.132768601 0.141588687 -7.301461092 2.00E-12 7.14E-12 16.10590765

AL031666.2 0.146800802 0.18573026 7.297726042 2.05E-12 7.31E-12 16.08224836

AP002884.3 -0.085929812 0.087314441 -7.294416569 2.10E-12 7.46E-12 16.06129225

ZNF8-ERVK3-1 -0.113705366 0.304439547 -7.294131881 2.10E-12 7.48E-12 16.05948988

AP003351.1 0.201381492 0.109739967 7.29327908 2.11E-12 7.51E-12 16.05409109

AC020658.4 0.069481447 0.045163675 7.29305345 2.11E-12 7.52E-12 16.05266278

AC009902.2 0.217588696 0.315457252 7.291969072 2.13E-12 7.57E-12 16.04579877

AL450384.1 -0.307214912 0.552128496 -7.290909135 2.14E-12 7.62E-12 16.0390902

LINC02700 0.233275893 0.144543349 7.289800564 2.16E-12 7.68E-12 16.03207457

AC119674.1 -0.15462735 0.304640381 -7.28881924 2.17E-12 7.72E-12 16.02586488

ZFY-AS1 -0.927513513 1.067222757 -7.287031472 2.20E-12 7.81E-12 16.01455369

AC005082.1 0.290013571 0.339066677 7.280240344 2.30E-12 8.16E-12 15.97160481

AC011453.1 0.044305552 0.027560721 7.279203324 2.31E-12 8.21E-12 15.965049

AC034213.1 0.16700115 0.116629083 7.277196214 2.34E-12 8.31E-12 15.95236245

AC020928.2 -0.057213826 0.052401241 -7.276868212 2.35E-12 8.33E-12 15.95028945

AC117382.2 -0.052918871 0.050236184 -7.276134278 2.36E-12 8.37E-12 15.94565119

AC022239.1 -0.253862138 0.212969274 -7.274875473 2.38E-12 8.43E-12 15.93769668

AC004012.1 -0.315549244 0.45429583 -7.274725552 2.38E-12 8.44E-12 15.93674938

AC004448.2 -0.106961628 0.072107431 -7.273519779 2.40E-12 8.50E-12 15.92913105

KCNMA1-AS3 0.056205644 0.034461363 7.271637819 2.43E-12 8.60E-12 15.91724227

AC084036.1 -0.57396299 3.094039997 -7.271518754 2.43E-12 8.61E-12 15.91649019

AC104534.1 0.471622494 0.898022477 7.271281653 2.43E-12 8.62E-12 15.91499254

AP000322.1 -0.133574018 0.078622563 -7.269892143 2.45E-12 8.69E-12 15.90621645

AL049870.3 0.325043583 0.435339016 7.269536156 2.46E-12 8.71E-12 15.90396824

AL451070.1 0.080714703 0.059680491 7.269349313 2.46E-12 8.72E-12 15.90278829

AL354726.1 0.13157333 0.120687192 7.267462129 2.49E-12 8.82E-12 15.89087152

LINC02574 0.210489506 0.148838095 7.266240289 2.51E-12 8.89E-12 15.88315733

AC120036.4 -0.195991768 0.563338889 -7.264429313 2.54E-12 8.99E-12 15.87172533

Z69666.1 -0.149477548 0.244043496 -7.263801572 2.55E-12 9.02E-12 15.86776313

LINC01693 0.210333236 0.112506724 7.262281119 2.58E-12 9.11E-12 15.8581673

LINC00942 0.207028479 0.199842545 7.261683038 2.59E-12 9.14E-12 15.85439312

AL078590.2 0.251881128 0.433453249 7.261363117 2.59E-12 9.16E-12 15.85237436

AL133343.2 0.584913206 0.423925128 7.260934396 2.60E-12 9.18E-12 15.84966916

AL161645.1 -0.245098642 0.399487202 -7.257888331 2.65E-12 9.36E-12 15.830452

AC092919.1 0.337724045 0.234827086 7.253816058 2.72E-12 9.61E-12 15.8047699

AC083801.2 0.120052372 0.074686491 7.250213799 2.78E-12 9.83E-12 15.7820608

AC025287.2 0.149029177 0.180989359 7.246601379 2.85E-12 1.01E-11 15.75929596

LINC00658 -0.11171174 0.067354544 -7.246533467 2.85E-12 1.01E-11 15.75886807

ASH1L-AS1 0.379112887 1.904288754 7.244676451 2.88E-12 1.02E-11 15.7471688

AL121987.2 -0.155002875 0.478968427 -7.244180164 2.89E-12 1.02E-11 15.74404255

AC104365.1 0.217503517 0.116392329 7.239708373 2.98E-12 1.05E-11 15.71588055

BANCR 0.148277309 0.089816577 7.239025835 2.99E-12 1.05E-11 15.71158326

LINC01124 0.40640933 0.602523655 7.237455301 3.02E-12 1.06E-11 15.70169622

STK4-AS1 -0.136626429 0.321741663 -7.234633437 3.08E-12 1.08E-11 15.68393561

AC119424.1 0.161906362 0.115777879 7.231111699 3.15E-12 1.11E-11 15.66177719

Z84485.1 -0.295542969 0.91392571 -7.230466198 3.16E-12 1.11E-11 15.65771662

LINC02521 0.040114737 0.024428855 7.230217986 3.16E-12 1.11E-11 15.6561553

DOCK4-AS1 -0.049965698 0.03893545 -7.228132925 3.21E-12 1.13E-11 15.64304123

AC008543.3 0.162144375 0.175986733 7.224306354 3.28E-12 1.16E-11 15.61898112

AC093799.1 -0.444014121 1.141009073 -7.219575985 3.39E-12 1.19E-11 15.5892512

AC027312.1 0.124590312 0.084949656 7.218928864 3.40E-12 1.20E-11 15.58518522

AC018766.1 -0.318000833 0.712337553 -7.21695342 3.44E-12 1.21E-11 15.5727748

AC244517.4 -0.173442762 0.084597283 -7.213081556 3.53E-12 1.24E-11 15.54845768

LINC01765 0.360564754 0.259605505 7.212112029 3.55E-12 1.25E-11 15.5423701

SUCLG2-AS1 -0.222075131 0.644979776 -7.211983735 3.55E-12 1.25E-11 15.5415646

AC004825.2 0.364962504 1.034997049 7.211689758 3.56E-12 1.25E-11 15.53971888

AC105036.3 -0.366105639 0.912317059 -7.2109514 3.58E-12 1.26E-11 15.5350834

AC005165.1 -0.229191099 0.382700796 -7.210303632 3.59E-12 1.26E-11 15.53101694

AL023802.1 -0.047045505 0.032200518 -7.20593934 3.69E-12 1.30E-11 15.50362644

AL158829.1 0.061618488 0.039297564 7.205442834 3.70E-12 1.30E-11 15.50051111

AC087501.1 0.07462428 0.05327661 7.204222853 3.73E-12 1.31E-11 15.49285703

AC114812.2 0.099845533 0.065333957 7.203539274 3.75E-12 1.32E-11 15.48856872

CSRP3-AS1 0.054332702 0.028851806 7.203206387 3.76E-12 1.32E-11 15.48648051

AC018467.1 0.044484312 0.026320389 7.201868532 3.79E-12 1.33E-11 15.47808887

AP002852.1 0.167231874 0.133945181 7.201711018 3.79E-12 1.33E-11 15.47710095

AP003692.1 -0.172126013 0.083561167 -7.201435716 3.80E-12 1.33E-11 15.47537429

AC011997.1 0.16390992 0.092934822 7.200790402 3.82E-12 1.34E-11 15.47132717

AC011346.1 -0.11736736 0.376236225 -7.199681688 3.84E-12 1.35E-11 15.46437445

AC138625.1 0.113846728 0.079254721 7.192082291 4.03E-12 1.41E-11 15.41673998

LINC01918 0.229092667 0.230633121 7.191972716 4.04E-12 1.41E-11 15.41605342

BX927359.1 -0.115262688 0.055956007 -7.191129808 4.06E-12 1.42E-11 15.41077226

AC011290.1 0.154880296 0.091892712 7.191104755 4.06E-12 1.42E-11 15.41061529

ITFG2-AS1 0.187221057 0.302042606 7.189803616 4.09E-12 1.43E-11 15.40246405

GRM7-AS1 0.097924011 0.058647412 7.188357356 4.13E-12 1.44E-11 15.39340493

AC083964.1 0.323236897 1.098910899 7.187999125 4.14E-12 1.45E-11 15.39116125

AC008982.2 0.35622688 0.775988191 7.187246601 4.16E-12 1.45E-11 15.38644828

AC067852.1 -0.120292138 0.06389181 -7.18359094 4.26E-12 1.49E-11 15.36355849

IGFBP7-AS1 0.136221016 0.20118867 7.179338324 4.37E-12 1.53E-11 15.33694172

AC023790.2 -0.08262144 0.182905574 -7.178027406 4.41E-12 1.54E-11 15.32873913

LINC01500 0.070245015 0.051463039 7.17716885 4.44E-12 1.55E-11 15.32336763

AL121944.1 0.393187931 1.875529381 7.173879591 4.53E-12 1.58E-11 15.30279301

AP003354.1 -0.182344021 0.221709148 -7.172857424 4.56E-12 1.59E-11 15.29640067

AC131025.2 -0.134906971 0.065492621 -7.171895026 4.59E-12 1.60E-11 15.29038274

AL662844.3 -0.372049376 0.917193391 -7.171640602 4.59E-12 1.60E-11 15.28879191

AL035250.1 0.387385078 0.284698033 7.169940027 4.64E-12 1.62E-11 15.27815984

AL589765.7 -0.284985745 0.343433558 -7.169249106 4.66E-12 1.63E-11 15.2738407

AC079612.1 0.018506322 0.010417883 7.167507376 4.72E-12 1.64E-11 15.26295403

AC093001.1 0.484226447 0.253731688 7.165656286 4.77E-12 1.66E-11 15.25138595

AC233728.1 0.491605032 0.759280199 7.163857929 4.83E-12 1.68E-11 15.24014954

AC079336.2 0.141078875 0.222721572 7.162303133 4.87E-12 1.70E-11 15.23043661

AC009292.2 0.105251518 0.076950068 7.162296548 4.87E-12 1.70E-11 15.23039548

AC090753.1 0.235041102 0.121832581 7.156184234 5.07E-12 1.76E-11 15.19222654

AL590093.1 0.100059417 0.07224448 7.155646739 5.08E-12 1.77E-11 15.18887126

AC007406.4 -0.147743905 0.106211036 -7.155166352 5.10E-12 1.78E-11 15.18587262

AC122719.2 0.087822177 0.059312293 7.152499212 5.19E-12 1.80E-11 15.16922672

AC105052.4 -0.268032162 0.218174528 -7.152412372 5.19E-12 1.81E-11 15.16868482

AC012404.1 0.049984403 0.026614454 7.152311799 5.19E-12 1.81E-11 15.16805723

AL022328.3 -0.418923533 1.865373686 -7.152274536 5.19E-12 1.81E-11 15.1678247

AP001429.1 -0.254859125 0.412190615 -7.151767313 5.21E-12 1.81E-11 15.16465966

AC020915.1 0.284816817 0.684519901 7.151593968 5.22E-12 1.81E-11 15.16357804

AC020907.3 0.096715854 0.079742737 7.150275483 5.26E-12 1.83E-11 15.1553517

AL590399.5 -0.05413206 0.028367469 -7.150028387 5.27E-12 1.83E-11 15.15381014

AC137932.2 0.313044411 0.556025733 7.149763343 5.28E-12 1.83E-11 15.15215664

AC134772.1 -0.093637073 0.0684312 -7.147453372 5.36E-12 1.86E-11 15.13774766

AP000879.2 0.059341479 0.033571383 7.147213587 5.36E-12 1.86E-11 15.13625215

AL807757.2 0.243821059 0.260636314 7.144962602 5.44E-12 1.89E-11 15.12221477

AC136475.9 -0.348297425 0.608983899 -7.142427815 5.53E-12 1.92E-11 15.1064115

AP002478.1 0.132708624 0.075348401 7.141500343 5.56E-12 1.93E-11 15.10063016

AC067930.2 0.201133773 0.307882871 7.140402991 5.60E-12 1.94E-11 15.09379061

AC107294.3 0.316743605 0.741603752 7.13958277 5.63E-12 1.95E-11 15.08867886

AL049647.1 0.047354409 0.031114281 7.13944582 5.63E-12 1.95E-11 15.08782541

AC245036.5 -0.065736038 0.031912546 -7.138889671 5.65E-12 1.96E-11 15.08435969

AC004039.1 -0.173770105 0.284778293 -7.135944808 5.76E-12 2.00E-11 15.06601174

LINC01031 0.062042616 0.041364515 7.135392912 5.78E-12 2.00E-11 15.06257378

UBOX5-AS1 0.15818504 0.264010454 7.131936865 5.91E-12 2.05E-11 15.04104929

AC104113.1 0.377627479 1.162067559 7.131787365 5.91E-12 2.05E-11 15.04011837

DPP9-AS1 -0.119138793 0.376797261 -7.130101719 5.98E-12 2.07E-11 15.02962302

AC124016.2 0.457248497 1.236718823 7.125045212 6.17E-12 2.14E-11 14.9981507

AL662890.1 0.193015561 0.13730021 7.124997645 6.17E-12 2.14E-11 14.99785472

AC096920.1 0.093164113 0.048831921 7.123910561 6.22E-12 2.15E-11 14.99109077

LINC00861 0.421084297 0.54924087 7.123315435 6.24E-12 2.16E-11 14.98738816

AC007036.1 0.137341124 0.172047366 7.122634257 6.27E-12 2.17E-11 14.98315046

TSIX -0.048633846 0.046336106 -7.119768835 6.38E-12 2.21E-11 14.96532755

AC098617.1 -0.068203685 0.043611266 -7.117958955 6.46E-12 2.23E-11 14.95407285

OTUD6B-AS1 -0.339440438 3.165412854 -7.117771368 6.46E-12 2.23E-11 14.95290647

AC090116.1 0.22614379 0.126710547 7.116823412 6.50E-12 2.25E-11 14.94701259

AC019171.1 0.237847743 0.530445069 7.114210589 6.61E-12 2.28E-11 14.93077048

AC005532.1 0.24933893 0.264767228 7.112886671 6.67E-12 2.30E-11 14.9225423

AL449106.1 0.157814174 0.342117654 7.112801291 6.67E-12 2.30E-11 14.92201169

AL138963.4 -0.133898094 0.065002846 -7.112246455 6.69E-12 2.31E-11 14.91856374

AC135782.1 0.181209725 0.103792347 7.111385859 6.73E-12 2.32E-11 14.91321608

AL117378.1 -0.101364928 0.052656716 -7.107556349 6.89E-12 2.38E-11 14.8894257

NEURL1-AS1 -0.206374054 0.212202138 -7.107550227 6.89E-12 2.38E-11 14.88938768

LINC02507 0.074970477 0.055356236 7.105453923 6.99E-12 2.41E-11 14.87636867

AL672032.1 0.064847415 0.040469949 7.103278525 7.08E-12 2.44E-11 14.86286148

AC093512.1 -0.13367532 0.064894697 -7.103226287 7.08E-12 2.44E-11 14.86253717

AP000845.1 -0.100968443 0.237819018 -7.101882261 7.14E-12 2.46E-11 14.85419361

AC069148.1 0.282987482 0.436914239 7.101686479 7.15E-12 2.46E-11 14.85297831

AC084116.3 0.100453408 0.055670137 7.101168768 7.18E-12 2.47E-11 14.8497648

AC068481.1 -0.169971214 0.210775842 -7.097973003 7.32E-12 2.52E-11 14.82993204

AL034374.1 0.169612031 0.211483787 7.095692679 7.43E-12 2.56E-11 14.81578451

GNA14-AS1 0.059892021 0.034586549 7.089214397 7.74E-12 2.66E-11 14.7756106

PRICKLE2-AS3 -0.067165483 0.063659708 -7.088843164 7.76E-12 2.67E-11 14.77330929

LINC02613 0.142184255 0.1859798 7.085828371 7.91E-12 2.72E-11 14.75462363

PKIA-AS1 0.045581652 0.041005796 7.082781081 8.06E-12 2.77E-11 14.73574256

IL10RB-DT -0.346990535 1.467646829 -7.081332971 8.13E-12 2.79E-11 14.72677217

AC245128.3 0.347418385 0.481759316 7.079355855 8.23E-12 2.83E-11 14.71452702

AC104118.1 -0.214501156 0.712747906 -7.07803476 8.30E-12 2.85E-11 14.70634632

RPL34-AS1 -0.110052954 0.163222582 -7.077609859 8.33E-12 2.86E-11 14.70371542

AC020779.2 0.235745953 0.271875301 7.06903662 8.79E-12 3.02E-11 14.65065682

AC009229.1 0.087008581 0.060481083 7.067402714 8.88E-12 3.05E-11 14.64055024

AL022476.1 0.14067282 0.27930521 7.066896705 8.91E-12 3.06E-11 14.63742066

AL513534.2 0.193832634 0.699848557 7.065730747 8.97E-12 3.08E-11 14.63021004

AL359697.1 0.307925702 0.536794292 7.064284419 9.05E-12 3.10E-11 14.62126676

EP300-AS1 0.352831473 0.817686198 7.063788535 9.08E-12 3.11E-11 14.6182008

LINC01521 0.225510414 0.753045229 7.063766619 9.08E-12 3.11E-11 14.61806531

AC246787.2 0.254259153 0.134219371 7.063167796 9.12E-12 3.12E-11 14.61436312

AC117465.1 0.043569885 0.024510152 7.061046905 9.24E-12 3.17E-11 14.60125274

AP005131.5 0.06632482 0.038928019 7.060222656 9.29E-12 3.18E-11 14.59615841

AC011466.1 0.179937012 0.224206081 7.057550812 9.44E-12 3.23E-11 14.5796479

AC136285.2 0.063997209 0.033824532 7.056558996 9.50E-12 3.25E-11 14.57352022

AC147067.2 0.457408535 0.889334822 7.056553506 9.50E-12 3.25E-11 14.57348631

AC010636.1 -0.224890254 0.252219498 -7.056047073 9.53E-12 3.26E-11 14.57035768

AC012531.2 -0.111325493 0.09518579 -7.054768448 9.61E-12 3.29E-11 14.5624594

AC126175.2 -0.25620114 0.25560885 -7.054588776 9.62E-12 3.29E-11 14.56134963

AC006487.1 -0.102730332 0.121466965 -7.054209788 9.64E-12 3.30E-11 14.5590088

U62631.1 0.396027082 0.258139989 7.052851259 9.73E-12 3.33E-11 14.55061861

AP001160.1 -0.374863131 1.309230408 -7.050973195 9.84E-12 3.36E-11 14.53902178

AC090227.3 0.080236304 0.042180113 7.050858462 9.85E-12 3.37E-11 14.5383134

LINC01165 -0.075379235 0.089628696 -7.048886123 9.97E-12 3.41E-11 14.52613706

CYYR1-AS1 -0.055521877 0.044632784 -7.048581473 9.99E-12 3.41E-11 14.52425652

TBC1D3P1-DHX40P1 -0.053726548 0.030052391 -7.04247845 1.04E-11 3.54E-11 14.48659654

AC110792.3 0.402229628 0.857126709 7.041718497 1.04E-11 3.56E-11 14.4819088

AC117500.2 -0.174387106 0.277950687 -7.041593908 1.04E-11 3.56E-11 14.48114031

LINC00377 -0.164641781 0.170708604 -7.039556825 1.06E-11 3.61E-11 14.46857667

ZFAS1 -0.590728472 6.543760317 -7.039459032 1.06E-11 3.61E-11 14.4679736

AL450322.1 0.135319105 0.080666434 7.036932797 1.07E-11 3.67E-11 14.45239707

LINC00702 0.308566563 0.719828364 7.03687696 1.08E-11 3.67E-11 14.45205283

LINC01569 -0.367653665 2.004335813 -7.033607752 1.10E-11 3.74E-11 14.43190151

AL445363.2 -0.083544786 0.048450306 -7.032460124 1.11E-11 3.77E-11 14.42482923

AC006019.1 -0.046073936 0.024118905 -7.03207905 1.11E-11 3.78E-11 14.42248104

AP003548.1 0.108585323 0.092176636 7.030628395 1.12E-11 3.81E-11 14.41354293

AC139530.1 -0.409250414 2.200160826 -7.030511403 1.12E-11 3.81E-11 14.41282216

APOBEC3B-AS1 -0.102417927 0.06742072 -7.028175233 1.14E-11 3.87E-11 14.3984311

LINC01517 -0.073532944 0.043510502 -7.028095503 1.14E-11 3.87E-11 14.39794002

AC003986.3 0.077956119 0.045081836 7.027604427 1.14E-11 3.88E-11 14.39491541

GPC5-AS1 0.782996627 0.560830613 7.027419338 1.14E-11 3.88E-11 14.39377546

PABPC5-AS1 0.090350307 0.055240081 7.026780374 1.15E-11 3.90E-11 14.38984032

AC092944.1 -0.241684128 0.697010156 -7.02539777 1.16E-11 3.93E-11 14.38132628

AL035458.2 -0.301156981 0.522196704 -7.02519977 1.16E-11 3.93E-11 14.3801071

AC108865.1 0.189641387 0.12504109 7.02431829 1.16E-11 3.95E-11 14.37467974

AC130650.2 -0.251051322 0.703706295 -7.023752967 1.17E-11 3.97E-11 14.37119925

SAPCD1-AS1 -0.546810908 1.2285243 -7.02225596 1.18E-11 4.00E-11 14.36198375

AC104116.1 -0.465002843 0.225742652 -7.021778223 1.18E-11 4.01E-11 14.35904314

LINC02091 -0.185869203 0.287120386 -7.019666834 1.20E-11 4.07E-11 14.34604869

AC132872.1 0.454569149 2.95970465 7.018948545 1.20E-11 4.08E-11 14.34162868

AC010395.1 0.04121575 0.022102676 7.015454157 1.23E-11 4.17E-11 14.3201307

AL135902.2 0.167674806 0.132526698 7.014761872 1.23E-11 4.19E-11 14.31587262

AP001099.1 -0.181688869 0.225501602 -7.01242408 1.25E-11 4.25E-11 14.30149572

NKX2-2-AS1 -0.146625292 0.071181457 -7.01132745 1.26E-11 4.28E-11 14.29475294

AL033523.1 0.080410903 0.063305645 7.010883399 1.27E-11 4.29E-11 14.29202285

AP000894.4 0.47234787 1.626605787 7.00590054 1.31E-11 4.42E-11 14.26139641

AC012354.1 -0.279725908 0.349819193 -7.003804148 1.32E-11 4.48E-11 14.24851611

AL121820.2 0.605689863 1.319051746 7.002002396 1.34E-11 4.53E-11 14.2374484

AL391839.2 0.145765403 0.103251042 7.001101984 1.34E-11 4.56E-11 14.2319182

AC011726.3 0.092342323 0.078753299 7.000411132 1.35E-11 4.57E-11 14.22767544

AL109614.1 0.390327043 0.800908397 6.999661157 1.36E-11 4.60E-11 14.22306995

AC008507.2 -0.095068705 0.112564261 -6.999196563 1.36E-11 4.61E-11 14.22021713

AL049795.1 -0.294973825 0.640141259 -6.997650353 1.37E-11 4.65E-11 14.2107237

AC090282.1 0.023854975 0.013169952 6.995020256 1.40E-11 4.73E-11 14.19457904

AL359555.1 0.093447167 0.051260962 6.993237418 1.41E-11 4.78E-11 14.18363781

LINC00442 -0.058330225 0.030903254 -6.992324074 1.42E-11 4.80E-11 14.17803345

AC012615.1 -0.424348794 2.595841338 -6.990703145 1.44E-11 4.85E-11 14.16808864

AL158211.2 -0.116857276 0.056730124 -6.989369959 1.45E-11 4.89E-11 14.1599105

AC004817.3 0.225872584 0.15149378 6.987879596 1.46E-11 4.94E-11 14.15076958

AC105429.1 -0.208927386 0.473264255 -6.985935967 1.48E-11 5.00E-11 14.13885082

CYMP-AS1 0.349789116 0.209288635 6.983758631 1.50E-11 5.06E-11 14.12550187

LINC02012 0.244940197 0.411618723 6.983413671 1.50E-11 5.07E-11 14.12338725

AC134312.3 0.194082225 0.116499126 6.983139782 1.50E-11 5.08E-11 14.12170836

AC007879.2 0.142268411 0.11176608 6.983057752 1.51E-11 5.08E-11 14.12120554

SAP30L-AS1 -0.232634408 0.786678491 -6.982904943 1.51E-11 5.08E-11 14.12026888

CCDC183-AS1 -0.331233049 1.411150806 -6.980607636 1.53E-11 5.16E-11 14.10618908

SAMSN1-AS1 0.055542867 0.0341324 6.975077213 1.58E-11 5.34E-11 14.07230836

Z85994.1 0.125533746 0.068478202 6.974735593 1.59E-11 5.35E-11 14.07021617

AC012645.4 0.393747634 0.81693426 6.974570002 1.59E-11 5.35E-11 14.06920207

AL133325.3 -0.772812769 1.354488944 -6.974061197 1.59E-11 5.37E-11 14.06608619

AC011297.1 -0.464781257 0.550205252 -6.972831193 1.60E-11 5.41E-11 14.05855445

AC011461.1 -0.360397408 0.7476642 -6.972518277 1.61E-11 5.42E-11 14.05663852

AL355916.2 0.197542235 0.284599031 6.970286695 1.63E-11 5.49E-11 14.04297678

AC008752.1 0.145703446 0.112015046 6.970169918 1.63E-11 5.49E-11 14.04226197

AC007218.1 0.059043702 0.031275779 6.968589265 1.65E-11 5.55E-11 14.03258733

AC011816.2 -0.202237895 0.337494283 -6.96853351 1.65E-11 5.55E-11 14.03224609

AC007032.1 -0.267411781 0.657612842 -6.967088443 1.66E-11 5.59E-11 14.02340283

AC084782.1 -0.056412234 0.035658445 -6.964729428 1.69E-11 5.68E-11 14.0089695

AC008734.2 -0.138935558 0.103341416 -6.961873345 1.72E-11 5.78E-11 13.99149986

AC004000.1 0.240794489 0.278281996 6.960798715 1.73E-11 5.81E-11 13.98492813

AC044810.2 -0.096338799 0.080149066 -6.960779027 1.73E-11 5.81E-11 13.98480773

LINC02015 -0.192150411 0.369404285 -6.959295107 1.75E-11 5.87E-11 13.97573432

AC138028.3 0.07679193 0.040407862 6.956932201 1.77E-11 5.95E-11 13.96128936

AC104779.1 0.122785636 0.096064532 6.955714097 1.78E-11 5.99E-11 13.95384427

AC138230.1 0.288614018 0.781654781 6.954950274 1.79E-11 6.02E-11 13.94917626

ZNF32-AS1 0.330853482 0.798379332 6.953234254 1.81E-11 6.09E-11 13.93869042

AC104667.1 -0.096483367 0.170011512 -6.952676678 1.82E-11 6.10E-11 13.93528374

AC078883.1 -0.399391772 1.605724169 -6.952374921 1.82E-11 6.11E-11 13.93344015

AL137003.2 0.238998858 1.571039342 6.948292514 1.87E-11 6.27E-11 13.90850452

AC092134.1 -0.278963066 0.586297588 -6.94762588 1.88E-11 6.29E-11 13.90443371

LINC01412 0.220923248 0.203186384 6.944107114 1.92E-11 6.43E-11 13.88295124

MIR1-1HG -0.110877807 0.076011422 -6.94284442 1.93E-11 6.48E-11 13.87524435

AC027449.1 0.328180476 0.650370347 6.942066325 1.94E-11 6.51E-11 13.87049575

AC103719.1 0.051658221 0.029147825 6.94201019 1.94E-11 6.51E-11 13.87015318

AC022400.1 0.347649318 1.03194034 6.941578347 1.95E-11 6.53E-11 13.86751789

AC026333.3 -0.065965639 0.040558885 -6.94042959 1.96E-11 6.57E-11 13.8605083

AL161725.2 -0.244269001 0.317581098 -6.939509246 1.97E-11 6.61E-11 13.85489309

AC016396.2 0.102026262 0.1017347 6.939066376 1.98E-11 6.63E-11 13.85219125

LINC01494 0.065093311 0.035541531 6.938561109 1.99E-11 6.64E-11 13.84910889

LINC01006 -0.398514152 2.701312526 -6.93799664 1.99E-11 6.67E-11 13.84566558

ARHGEF26-AS1 -0.230222595 0.294917775 -6.937679152 2.00E-11 6.68E-11 13.84372897

AC008759.3 0.396456798 0.524801692 6.937587951 2.00E-11 6.68E-11 13.84317267

AL359091.4 0.319012575 0.907069318 6.936363527 2.01E-11 6.73E-11 13.83570464

LINC01748 0.394649456 0.24386578 6.936069533 2.02E-11 6.74E-11 13.83391165

AL662791.1 0.341521617 0.599275909 6.935231523 2.03E-11 6.77E-11 13.82880117

BASP1-AS1 0.052887952 0.046975212 6.935213163 2.03E-11 6.77E-11 13.82868921

LINC00968 0.230373656 0.291292654 6.9347533 2.03E-11 6.79E-11 13.82588501

AC023389.1 0.109290399 0.112030116 6.931772418 2.07E-11 6.92E-11 13.80771128

AL590438.1 0.19851446 0.260357005 6.931008388 2.08E-11 6.95E-11 13.80305412

AC020978.4 -0.44598985 1.068170872 -6.928912462 2.11E-11 7.04E-11 13.79028034

AL356108.1 0.085460099 0.064056011 6.928672514 2.11E-11 7.05E-11 13.78881814

LINC00840 0.127872052 0.192122022 6.92786235 2.12E-11 7.08E-11 13.78388145

PARD3-AS1 0.43752907 0.761614428 6.926117865 2.14E-11 7.15E-11 13.773253

AL512656.1 0.20326038 0.227603895 6.925148618 2.16E-11 7.20E-11 13.76734863

AL020993.1 0.233110256 0.179283607 6.924445934 2.17E-11 7.23E-11 13.76306848

AL138902.1 0.068867877 0.052121667 6.923106225 2.18E-11 7.28E-11 13.75490902

LINC01258 0.028349593 0.016448528 6.91120241 2.35E-11 7.84E-11 13.68246154

AC005006.1 0.214163399 0.125651336 6.909208098 2.38E-11 7.93E-11 13.67033322

UFL1-AS1 0.107888043 0.074995434 6.907116948 2.41E-11 8.04E-11 13.65761882

AC108676.1 0.25887201 0.269012225 6.907092507 2.41E-11 8.04E-11 13.65747023

AC002511.2 -0.62370588 1.045879738 -6.906590949 2.42E-11 8.06E-11 13.65442116

ZMIZ1-AS1 0.222740713 0.672034315 6.904393162 2.45E-11 8.17E-11 13.6410623

U62317.3 0.219648082 0.273436157 6.904226101 2.46E-11 8.17E-11 13.64004698

AC122710.2 0.146282463 0.123795653 6.904177242 2.46E-11 8.17E-11 13.63975005

AL354824.2 0.229455042 0.147650647 6.901597972 2.50E-11 8.30E-11 13.62407688

AC027338.1 -0.147869194 0.212319293 -6.901454417 2.50E-11 8.31E-11 13.62320469

PEX5L-AS2 -0.08429096 0.045093016 -6.900488471 2.51E-11 8.36E-11 13.61733626

AC105046.1 0.122334129 0.163261764 6.89590313 2.59E-11 8.59E-11 13.58948736

NECTIN3-AS1 -0.198155786 0.314783271 -6.895367335 2.59E-11 8.62E-11 13.58623414

P4HA2-AS1 0.128848685 0.100726043 6.895251349 2.60E-11 8.63E-11 13.58552993

AC026782.2 0.061919496 0.034370348 6.892949246 2.63E-11 8.75E-11 13.57155446

AC090948.1 0.301348717 0.891420426 6.89181703 2.65E-11 8.81E-11 13.56468237

MID1IP1-AS1 0.493990354 1.460466231 6.891776806 2.65E-11 8.81E-11 13.56443824

AC111182.1 -0.272218913 0.223026773 -6.88944019 2.69E-11 8.93E-11 13.5502587

AL590096.1 0.192054917 0.253949108 6.888756164 2.70E-11 8.97E-11 13.54610844

AC079298.3 0.2637748 0.413862385 6.888491581 2.71E-11 8.98E-11 13.54450318

AL359094.1 -0.088703736 0.077076309 -6.888142853 2.71E-11 9.00E-11 13.5423875

AC027601.3 0.193400637 0.43957989 6.887069872 2.73E-11 9.05E-11 13.53587835

AC007950.2 -0.067868521 0.12667585 -6.886002671 2.75E-11 9.11E-11 13.52940504

AC005280.1 0.238207153 0.282372154 6.884753407 2.77E-11 9.18E-11 13.52182834

AL451042.1 0.355093733 0.814346742 6.884188442 2.78E-11 9.21E-11 13.51840221

TCL6 -0.237756 0.297004749 -6.884080157 2.78E-11 9.21E-11 13.51774556

AP005264.3 -0.179224314 0.140048158 -6.880822174 2.84E-11 9.40E-11 13.4979925

RBAKDN -0.265658475 0.294450863 -6.879033708 2.87E-11 9.50E-11 13.48715209

AL390038.1 -0.03050112 0.015913636 -6.878847318 2.87E-11 9.51E-11 13.48602245

AC026310.2 0.146895089 0.100631891 6.878673203 2.88E-11 9.52E-11 13.48496723

Z83844.2 -0.092991792 0.172831513 -6.877376572 2.90E-11 9.59E-11 13.47710962

AC016571.1 0.127032237 0.15002415 6.877181382 2.90E-11 9.60E-11 13.47592686

AC025918.1 -0.264545325 0.463473282 -6.876756143 2.91E-11 9.62E-11 13.4733502

AL355490.2 0.160055892 0.171094291 6.876393425 2.92E-11 9.64E-11 13.47115248

AC105219.4 0.21482341 0.42176783 6.873793156 2.96E-11 9.80E-11 13.45539988

AC092535.5 0.719543745 3.225898959 6.873032398 2.98E-11 9.84E-11 13.45079202

AC011306.1 -0.135446772 0.178963643 -6.871792349 3.00E-11 9.91E-11 13.44328195

AC129492.4 -0.223600654 0.267006643 -6.87179118 3.00E-11 9.91E-11 13.44327486

LINC02448 0.224716998 0.118739012 6.86938044 3.04E-11 1.01E-10 13.42867771

AC145207.4 -0.620685417 0.301321111 -6.868353383 3.06E-11 1.01E-10 13.42246

AL162595.1 -0.359014653 1.385783324 -6.86801281 3.07E-11 1.01E-10 13.42039836

AC008115.4 0.28343557 0.441479062 6.86575215 3.11E-11 1.03E-10 13.40671554

Z95114.1 0.016044742 0.010864715 6.864013453 3.15E-11 1.04E-10 13.39619426

LINC02011 0.021487394 0.012831593 6.857426247 3.28E-11 1.08E-10 13.35635182

AL031848.1 0.214714134 0.111373647 6.85607155 3.30E-11 1.09E-10 13.34816159

AC008669.1 0.310811269 1.089073671 6.853176994 3.36E-11 1.11E-10 13.33066579

AL355353.1 0.544976266 2.505054055 6.852374719 3.38E-11 1.11E-10 13.32581752

AC135050.6 -0.397669958 4.746182015 -6.852325536 3.38E-11 1.11E-10 13.32552032

AL139039.3 0.067254299 0.046613796 6.849808595 3.43E-11 1.13E-10 13.31031296

AC012414.4 -0.039846558 0.019344107 -6.846055354 3.51E-11 1.16E-10 13.28764376

AC022098.3 0.255324308 0.312543596 6.844535503 3.55E-11 1.17E-10 13.27846669

AL359258.1 -0.030330625 0.016001361 -6.839390365 3.66E-11 1.21E-10 13.24741114

AC010148.1 -0.162308755 0.240433292 -6.839224764 3.67E-11 1.21E-10 13.24641188

SNHG31 0.155389754 0.231166544 6.837843215 3.70E-11 1.22E-10 13.23807617

AC131097.2 0.302060925 0.298583435 6.836868509 3.72E-11 1.22E-10 13.23219595

AC022164.1 0.061370717 0.036227952 6.835098934 3.76E-11 1.24E-10 13.22152206

AC104809.1 -0.053217001 0.051295901 -6.832694792 3.82E-11 1.26E-10 13.2070239

AC009167.1 -0.053085377 0.026146642 -6.830578886 3.87E-11 1.27E-10 13.19426715

AC017015.2 0.097160276 0.053472493 6.829250021 3.90E-11 1.28E-10 13.18625699

AC092839.2 0.102807833 0.073347185 6.828072393 3.93E-11 1.29E-10 13.17915945

C2-AS1 0.123643176 0.112029628 6.826408304 3.97E-11 1.30E-10 13.1691316

AC105206.2 0.301449521 1.218861646 6.826315366 3.97E-11 1.30E-10 13.16857161

LINC01079 -0.428479737 0.482591489 -6.822486872 4.06E-11 1.33E-10 13.1455083

AC093010.3 0.441676356 4.764522501 6.820491426 4.11E-11 1.35E-10 13.1334914

AC090970.1 0.14497412 0.109448656 6.819360192 4.14E-11 1.36E-10 13.12668011

AC093425.1 -0.121695876 0.102090665 -6.818532108 4.16E-11 1.37E-10 13.12169467

Z99943.1 0.084399613 0.080656497 6.817562757 4.19E-11 1.37E-10 13.11585932

AC120498.3 -0.324882029 0.417949912 -6.816341528 4.22E-11 1.38E-10 13.1085086

AC122108.2 0.272792492 0.218060836 6.81386016 4.28E-11 1.40E-10 13.09357605

AL031963.3 -0.30748123 0.846090979 -6.813232178 4.30E-11 1.41E-10 13.08979759

AL445490.1 0.335636441 0.362844536 6.810784578 4.36E-11 1.43E-10 13.07507334

LINC02520 0.130209215 0.067892919 6.809915928 4.39E-11 1.44E-10 13.06984869

AC137770.1 0.177446652 0.177094905 6.806590467 4.48E-11 1.47E-10 13.04985181

FAM3D-AS1 0.22434781 0.145600648 6.80039928 4.65E-11 1.52E-10 13.01264238

AL391834.1 -0.405011308 1.849796433 -6.799002052 4.69E-11 1.54E-10 13.00424852

AP003119.1 0.094434341 0.070931338 6.798321131 4.71E-11 1.54E-10 13.00015836

AC112512.1 -0.061243288 0.062721796 -6.791565677 4.91E-11 1.61E-10 12.95959656

ARRDC3-AS1 -0.184811736 0.546031315 -6.791367836 4.91E-11 1.61E-10 12.95840913

LINC02147 0.125291665 0.134028167 6.790686289 4.93E-11 1.61E-10 12.95431871

AC021739.2 -0.29375677 1.076257515 -6.784841867 5.11E-11 1.67E-10 12.91925531

AC131956.2 0.081952627 0.053395546 6.784836157 5.11E-11 1.67E-10 12.91922107

AL118508.1 -0.471301296 0.854563668 -6.784465267 5.13E-11 1.68E-10 12.9169967

AC135048.2 -0.127969094 0.062124524 -6.784422604 5.13E-11 1.68E-10 12.91674084

AC079322.1 -0.213262029 0.702088224 -6.781457901 5.22E-11 1.71E-10 12.89896386

AC060814.4 -0.039394971 0.019406198 -6.779522 5.28E-11 1.73E-10 12.88735899

DNM1P35 0.115230359 0.285138568 6.778410141 5.32E-11 1.74E-10 12.88069503

AC007292.1 -0.340152299 1.494605293 -6.77784717 5.34E-11 1.74E-10 12.87732117

AL355990.2 0.137295094 0.083511221 6.776135186 5.39E-11 1.76E-10 12.86706263

AL035461.2 0.390701685 1.581368693 6.772251974 5.52E-11 1.80E-10 12.84380101

AC015923.1 0.063952196 0.03503664 6.76972984 5.61E-11 1.83E-10 12.82869812

AL358933.1 -0.069401552 0.087469872 -6.767147261 5.70E-11 1.86E-10 12.81323772

LINC01675 0.066392611 0.040008746 6.767121104 5.70E-11 1.86E-10 12.81308116

AC012531.1 0.169735548 0.159364272 6.7660734 5.73E-11 1.87E-10 12.80681047

AC106037.3 0.20485025 0.22596159 6.764739915 5.78E-11 1.89E-10 12.79883041

AL512283.1 -0.066444163 0.067168653 -6.763127111 5.84E-11 1.90E-10 12.7891804

PSORS1C3 0.981697188 2.092353101 6.761593976 5.89E-11 1.92E-10 12.78000872

LINC00654 0.272904415 1.143186968 6.753416757 6.19E-11 2.02E-10 12.73111695

AC017006.1 0.048980695 0.026098011 6.751987003 6.25E-11 2.04E-10 12.72257307

FP325318.1 -0.055835349 0.038129001 -6.750974949 6.29E-11 2.05E-10 12.7165261

AC012613.1 0.0451267 0.024114996 6.749068625 6.36E-11 2.07E-10 12.70513781

LINC01224 0.246621861 0.227410144 6.746907963 6.44E-11 2.10E-10 12.69223309

AC011978.2 0.165729887 0.639996966 6.746086015 6.48E-11 2.11E-10 12.68732478

DLGAP1-AS1 0.514459423 2.749558705 6.745087094 6.52E-11 2.12E-10 12.68136027

AF001548.1 0.306725903 0.245126465 6.743434829 6.58E-11 2.14E-10 12.67149616

AC093788.1 -0.353137709 1.475813243 -6.742973184 6.60E-11 2.15E-10 12.66874045

TTC21B-AS1 -0.066704925 0.053682949 -6.741270588 6.67E-11 2.17E-10 12.65857833

LINC02300 0.430265556 0.224717568 6.740269878 6.71E-11 2.18E-10 12.65260641

AL121957.1 0.116036882 0.083982779 6.739688946 6.73E-11 2.19E-10 12.6491399

AC026341.1 0.076464443 0.05704978 6.738428869 6.78E-11 2.21E-10 12.64162162

AC131971.1 -0.125556333 0.266562029 -6.735973486 6.89E-11 2.24E-10 12.62697462

AC005162.3 -0.127931025 0.252142693 -6.734404013 6.95E-11 2.26E-10 12.61761444

AC090948.3 0.347977912 0.914960278 6.732967171 7.01E-11 2.28E-10 12.60904672

DPH6-DT -0.286071683 0.53192854 -6.729656508 7.16E-11 2.32E-10 12.58931097

AL356310.1 0.146353084 0.141275975 6.72943805 7.17E-11 2.33E-10 12.58800895

AP003037.1 -0.368391685 0.178841305 -6.726590511 7.29E-11 2.37E-10 12.57104038

LINC00974 0.027592991 0.020818156 6.725544233 7.34E-11 2.38E-10 12.56480696

AP001160.2 -0.292269711 0.765952965 -6.720124307 7.58E-11 2.46E-10 12.53252854

AC096537.1 0.447907812 0.843938327 6.719612259 7.61E-11 2.47E-10 12.52948006

AC004870.3 0.040266364 0.021614184 6.719376709 7.62E-11 2.47E-10 12.52807778

LINC02055 -0.309839917 0.234371819 -6.717648694 7.70E-11 2.50E-10 12.51779164

AC005614.1 -0.039927321 0.034604216 -6.716240304 7.76E-11 2.52E-10 12.5094096

AL035665.1 0.12100339 0.133645457 6.714994791 7.82E-11 2.53E-10 12.50199804

SEC62-AS1 -0.322460035 0.568907906 -6.713609114 7.89E-11 2.56E-10 12.49375366

LINC00304 -0.128287435 0.16220355 -6.713495007 7.89E-11 2.56E-10 12.49307482

AC015982.2 -0.378199913 1.686967473 -6.713155173 7.91E-11 2.56E-10 12.49105313

HGC6.3 -0.106750521 0.08204599 -6.707853295 8.17E-11 2.64E-10 12.45952219

AC005911.1 0.362088024 0.861200162 6.704996294 8.31E-11 2.69E-10 12.44253918

AC006262.1 0.381595969 0.663236087 6.702976351 8.41E-11 2.72E-10 12.43053528

AC083805.1 -0.053266323 0.074407655 -6.7028917 8.42E-11 2.72E-10 12.43003229

AL139022.1 -0.166031165 0.307486783 -6.702757638 8.42E-11 2.72E-10 12.42923571

TPRG1-AS1 0.302128082 0.763658223 6.70178444 8.47E-11 2.74E-10 12.42345343

AL118511.1 0.206030426 0.451831266 6.701570148 8.48E-11 2.74E-10 12.4221803

AP003031.1 0.073401758 0.052037454 6.700109586 8.56E-11 2.77E-10 12.41350376

LINC01391 -0.047662262 0.033117232 -6.698328409 8.65E-11 2.80E-10 12.40292456

NUP50-DT 0.378273535 2.995549365 6.697409394 8.70E-11 2.81E-10 12.39746697

AC069213.1 0.187406314 0.283864293 6.695449067 8.80E-11 2.84E-10 12.38582745

AC011933.2 -0.044374398 0.045726787 -6.69522729 8.82E-11 2.85E-10 12.3845108

AC011825.3 -0.11378387 0.055609586 -6.693247282 8.92E-11 2.88E-10 12.37275738

AC211476.2 -0.192281355 0.435021117 -6.692143652 8.98E-11 2.90E-10 12.36620734

LINC02637 -0.248024714 0.547878505 -6.69055477 9.07E-11 2.93E-10 12.35677879

FER1L6-AS2 0.090635827 0.063476889 6.685558438 9.35E-11 3.02E-10 12.32714138

AC092068.3 -0.050865585 0.04867522 -6.68541211 9.35E-11 3.02E-10 12.32627364

CDKN2A-DT 0.155849728 0.097743074 6.685041819 9.38E-11 3.02E-10 12.32407785

AP001025.1 0.266140682 0.148891498 6.684725578 9.39E-11 3.03E-10 12.32220265

AC007787.1 -0.047425037 0.034228416 -6.684689729 9.40E-11 3.03E-10 12.32199008

AC125618.1 -0.183755742 0.194148373 -6.684013811 9.43E-11 3.04E-10 12.31798236

ACOXL-AS1 -0.156634079 0.202839378 -6.68277852 9.50E-11 3.06E-10 12.31065875

RMRP -0.404545743 0.223253626 -6.682077963 9.54E-11 3.07E-10 12.30650585

AC010745.1 0.072125622 0.038006884 6.67956867 9.69E-11 3.12E-10 12.29163353

AC021546.1 0.125852447 0.136375813 6.677891375 9.79E-11 3.15E-10 12.28169477

AL359881.1 0.500828193 0.814174409 6.676270079 9.89E-11 3.18E-10 12.27208966

LINC01431 -0.395088435 1.03909038 -6.675626309 9.92E-11 3.19E-10 12.26827625

LINC02539 0.05769025 0.044968443 6.674295192 1.00E-10 3.22E-10 12.26039218

FMR1-AS1 -0.053222418 0.054072527 -6.671530176 1.02E-10 3.27E-10 12.24401916

AC108681.1 0.106998584 0.055950239 6.669442 1.03E-10 3.31E-10 12.2316575

AC073257.2 0.26487503 0.485467307 6.667912608 1.04E-10 3.34E-10 12.22260565

AC005703.4 0.090684696 0.059738888 6.666443749 1.05E-10 3.37E-10 12.21391356

AL449423.1 0.13631782 0.102292192 6.664477052 1.06E-10 3.41E-10 12.2022778

AP000317.1 0.101035888 0.104642751 6.661325182 1.08E-10 3.48E-10 12.1836356

DPYD-IT1 0.067564407 0.038434908 6.657087398 1.11E-10 3.56E-10 12.15858131

GNAS-AS1 -0.171020523 0.254700732 -6.655413944 1.12E-10 3.60E-10 12.14869104

AC005884.1 0.252757301 0.342983631 6.653380124 1.13E-10 3.64E-10 12.13667355

AC023983.1 0.107607214 0.109256284 6.65297564 1.14E-10 3.65E-10 12.13428386

AC027228.1 0.079741691 0.048528236 6.651665891 1.15E-10 3.68E-10 12.12654664

RSF1-IT1 0.077119828 0.072382581 6.650879618 1.15E-10 3.70E-10 12.12190237

AL354928.1 0.158279516 0.211903525 6.650119302 1.16E-10 3.71E-10 12.11741183

LINC02069 -0.053963355 0.033596474 -6.646384571 1.18E-10 3.80E-10 12.09535967

LINC00165 0.172366449 0.10187196 6.643682344 1.20E-10 3.86E-10 12.07941001

AC112484.3 -0.358409858 0.93233471 -6.6429547 1.21E-10 3.87E-10 12.07511601

AL590652.1 -0.234235013 0.670308083 -6.641984783 1.22E-10 3.89E-10 12.06939287

LINC02422 0.421800643 0.270387221 6.640997465 1.22E-10 3.92E-10 12.06356771

AL133467.3 -0.043584927 0.025821065 -6.640031052 1.23E-10 3.94E-10 12.05786653

AC080013.3 0.292613114 0.70823377 6.638644729 1.24E-10 3.97E-10 12.0496893

LINC02575 0.367304112 0.347381937 6.637520161 1.25E-10 4.00E-10 12.043057

LINC02128 -0.138697054 0.140665256 -6.637217777 1.25E-10 4.00E-10 12.04127379

AC007128.1 0.297095741 0.284028082 6.635737516 1.26E-10 4.04E-10 12.03254537

AC002467.1 0.345507203 1.782169833 6.635204499 1.27E-10 4.05E-10 12.02940277

AC119403.1 -0.239962125 0.714971858 -6.633366179 1.28E-10 4.09E-10 12.01856581

AL121972.1 -0.169284971 0.235112976 -6.632774277 1.28E-10 4.11E-10 12.01507701

AP001610.3 0.180771544 0.096283547 6.632522966 1.29E-10 4.11E-10 12.01359581

AC103769.1 0.143343164 0.090698459 6.632192397 1.29E-10 4.12E-10 12.01164753

AC104564.4 0.145384829 0.083773108 6.630382846 1.30E-10 4.16E-10 12.00098388

ITGA6-AS1 0.225443119 0.437447189 6.627191815 1.33E-10 4.24E-10 11.98218467

AP001189.1 0.331675103 0.983911866 6.622856122 1.36E-10 4.36E-10 11.95665317

PTPRJ-AS1 0.116743604 0.079934814 6.622070708 1.37E-10 4.37E-10 11.9520295

AL645939.4 0.364496266 0.393476977 6.620477673 1.38E-10 4.42E-10 11.94265273

AC083843.2 -0.104857049 0.113878615 -6.619334647 1.39E-10 4.45E-10 11.93592584

LINC02223 0.091344822 0.055264904 6.619161155 1.39E-10 4.45E-10 11.93490489

AP004247.2 -0.088537328 0.122478082 -6.618923637 1.40E-10 4.45E-10 11.9335072

AC068790.8 -0.196547773 0.309811881 -6.618201444 1.40E-10 4.47E-10 11.92925764

AC005498.2 -0.299157963 0.473239127 -6.617183969 1.41E-10 4.50E-10 11.92327118

AC138430.1 -0.069101855 0.074057815 -6.61552823 1.42E-10 4.54E-10 11.91353093

AC090796.1 0.257691037 0.244498482 6.615357279 1.43E-10 4.55E-10 11.91252538

AC034231.1 0.375206433 1.220472256 6.614961643 1.43E-10 4.56E-10 11.91019829

AC079793.1 0.08207234 0.055736956 6.61461858 1.43E-10 4.56E-10 11.90818052

LINC02587 0.254010005 0.254927906 6.61040105 1.47E-10 4.68E-10 11.88338112

GPC5-IT1 0.26765016 0.142663659 6.607302705 1.50E-10 4.77E-10 11.86517042

AC027088.1 -0.047877301 0.023242752 -6.601101359 1.55E-10 4.95E-10 11.8287415

AC096564.1 -0.072637978 0.093249639 -6.599940945 1.56E-10 4.98E-10 11.82192776

AC083809.1 0.271568218 0.204332496 6.597375058 1.59E-10 5.06E-10 11.80686462

LINC02263 0.145858285 0.075944911 6.597180256 1.59E-10 5.06E-10 11.80572122

AC036222.1 0.03203187 0.01737725 6.597065671 1.59E-10 5.06E-10 11.80504866

AF111167.2 0.248463128 0.601428205 6.59552793 1.61E-10 5.11E-10 11.7960238

AL158847.1 0.119817428 0.138839459 6.595069502 1.61E-10 5.12E-10 11.79333364

AC087284.1 -0.137239241 0.169355655 -6.594278803 1.62E-10 5.14E-10 11.78869398

AC104964.1 0.625011531 1.19777531 6.594151256 1.62E-10 5.15E-10 11.78794561

AC090206.1 -0.058392057 0.06628684 -6.592257156 1.64E-10 5.20E-10 11.77683334

LINC01891 0.122569687 0.063962115 6.592097519 1.64E-10 5.21E-10 11.7758969

AL391095.3 -0.169662568 0.261186877 -6.591220148 1.65E-10 5.23E-10 11.77075049

AC069294.1 -0.270692355 0.322573471 -6.588845923 1.67E-10 5.31E-10 11.75682663

AC007638.2 -0.097745425 0.130349341 -6.587403948 1.69E-10 5.35E-10 11.74837194

AC116096.1 -0.05231322 0.042565915 -6.587202568 1.69E-10 5.36E-10 11.74719131

TPRG1-AS2 0.095768078 0.061607006 6.585266076 1.71E-10 5.42E-10 11.73583965

NKAIN3-IT1 0.176040387 0.214438067 6.585017222 1.71E-10 5.43E-10 11.73438106

AC244502.3 0.058366419 0.032807949 6.581538649 1.75E-10 5.54E-10 11.71399687

AC092902.2 0.205849658 0.135363828 6.581314605 1.75E-10 5.54E-10 11.71268427

ZNF436-AS1 -0.397797047 1.772149067 -6.580026456 1.76E-10 5.59E-10 11.70513813

U47924.2 -0.342047871 1.662031937 -6.578027745 1.78E-10 5.65E-10 11.69343169

AC018695.3 0.083143084 0.066184572 6.572487279 1.84E-10 5.84E-10 11.66099564

AL035427.1 0.102971836 0.064164894 6.572045392 1.85E-10 5.85E-10 11.65840957

AC016292.1 0.077933882 0.040995436 6.571561183 1.85E-10 5.87E-10 11.65557598

AC136469.1 0.086189696 0.065581473 6.571161616 1.86E-10 5.88E-10 11.65323783

AC016229.2 0.108122207 0.070557122 6.570910246 1.86E-10 5.89E-10 11.65176695

LINC00866 0.275477769 0.344537612 6.57056179 1.86E-10 5.90E-10 11.64972804

AL139393.1 0.101934627 0.075820345 6.568883073 1.88E-10 5.96E-10 11.6399066

COL5A1-AS1 0.147778629 0.111091792 6.567767582 1.90E-10 6.00E-10 11.63338142

C10orf91 0.234226754 0.271792141 6.567154333 1.90E-10 6.02E-10 11.62979452

LINC01098 0.121831203 0.069957464 6.567139336 1.90E-10 6.02E-10 11.62970681

AC022167.4 0.123533746 0.122920806 6.566280736 1.91E-10 6.05E-10 11.6246853

AC106739.1 0.335845539 0.752568773 6.566216614 1.91E-10 6.05E-10 11.62431031

AC139722.1 0.100957897 0.057190963 6.565374174 1.92E-10 6.08E-10 11.61938385

AC092807.1 0.110736501 0.064699542 6.565163567 1.92E-10 6.08E-10 11.61815233

LINC02132 0.192224269 0.110035095 6.562387283 1.96E-10 6.18E-10 11.60192092

AL157392.2 0.149446868 0.116125067 6.56123696 1.97E-10 6.22E-10 11.59519717

LINC00278 0.335578433 0.337896452 6.560756305 1.98E-10 6.24E-10 11.59238797

AC011773.3 0.130897796 0.188594031 6.560601757 1.98E-10 6.25E-10 11.59148474

AL589765.1 0.386567341 0.256343724 6.559373009 1.99E-10 6.29E-10 11.58430414

AC022034.4 0.227254141 0.242816196 6.555661665 2.04E-10 6.43E-10 11.56262202

HCFC1-AS1 0.180249308 0.160300489 6.55556553 2.04E-10 6.43E-10 11.56206051

AC104117.5 -0.196894428 0.258518467 -6.554644863 2.05E-10 6.47E-10 11.55668341

AL138826.1 0.395904817 0.249156991 6.554195529 2.05E-10 6.48E-10 11.55405931

AC005722.3 -0.0452202 0.049094326 -6.55312619 2.07E-10 6.52E-10 11.54781495

AC073332.1 -0.287905105 0.786744542 -6.550549065 2.10E-10 6.62E-10 11.53276921

ZFX-AS1 0.097962553 0.075023119 6.54974263 2.11E-10 6.65E-10 11.52806204

AC079336.1 0.085575944 0.066483846 6.548177318 2.13E-10 6.71E-10 11.51892657

AC106882.1 0.051938204 0.036561738 6.547399147 2.14E-10 6.74E-10 11.51438565

LINC01268 0.254143703 0.618086049 6.545817428 2.16E-10 6.80E-10 11.505157

AC211433.2 0.184285878 0.233420618 6.545108986 2.17E-10 6.83E-10 11.50102412

KCNAB1-AS1 -0.142104573 0.092833852 -6.544933502 2.17E-10 6.84E-10 11.50000044

AC004232.1 -0.089587434 0.043491567 -6.544210392 2.18E-10 6.86E-10 11.49578243

TTTY6 -0.065479156 0.031787838 -6.542548498 2.20E-10 6.93E-10 11.48608972

WASIR2 0.245946155 0.261901909 6.539482349 2.24E-10 7.06E-10 11.46821197

AL158212.2 0.317245239 0.649775756 6.538984531 2.25E-10 7.08E-10 11.46530996

AGBL5-AS1 0.167735886 0.193889148 6.537291743 2.27E-10 7.15E-10 11.45544322

AC103764.1 -0.03779128 0.023804572 -6.53658376 2.28E-10 7.17E-10 11.4513172

AC116447.1 0.236186968 0.48708414 6.53455842 2.31E-10 7.26E-10 11.43951573

AC068338.3 -0.407427955 1.646347645 -6.532946608 2.33E-10 7.33E-10 11.43012588

AC093458.1 -0.498751197 0.681321877 -6.531735505 2.35E-10 7.38E-10 11.4230716

AC079384.1 -0.130759235 0.098690291 -6.531448447 2.35E-10 7.39E-10 11.42139974

AC104964.2 0.224172657 0.240956444 6.530949197 2.36E-10 7.41E-10 11.41849217

AL356804.1 -0.212965357 0.250400735 -6.528985548 2.39E-10 7.50E-10 11.4070578

AC104971.1 0.120782857 0.268356043 6.523393239 2.47E-10 7.75E-10 11.37450835

AL356488.3 0.1474111 0.507120273 6.522458133 2.48E-10 7.79E-10 11.36906779

AL358072.1 -0.277599038 0.769090879 -6.521128603 2.50E-10 7.85E-10 11.36133346

LINC02694 0.03282662 0.027046313 6.520874279 2.50E-10 7.86E-10 11.35985411

AC021678.2 0.0664591 0.043801965 6.518127272 2.55E-10 7.99E-10 11.34387821

AC010320.1 0.117364531 0.151735667 6.517360617 2.56E-10 8.02E-10 11.33942048

AC005083.1 -0.500315195 2.915802569 -6.515473434 2.59E-10 8.11E-10 11.32844914

AC136604.3 -0.29107798 0.725086768 -6.515293389 2.59E-10 8.12E-10 11.32740257

AC008496.2 0.134614973 0.095804401 6.514923826 2.59E-10 8.13E-10 11.32525441

AC127496.3 0.064050154 0.047191198 6.513152295 2.62E-10 8.22E-10 11.31495836

AC093206.1 -0.144025592 0.120853489 -6.512909145 2.63E-10 8.23E-10 11.31354536

PCAT19 0.377865086 1.512800155 6.507443354 2.71E-10 8.49E-10 11.28179315

NAV2-AS5 -0.040144336 0.021012851 -6.506955266 2.72E-10 8.52E-10 11.27895873

AL139300.2 0.105874171 0.112676217 6.506310258 2.73E-10 8.55E-10 11.27521331

STARD7-AS1 -0.22122606 2.027878627 -6.502545085 2.79E-10 8.74E-10 11.25335551

AL390778.2 0.236222405 0.13541546 6.501703909 2.81E-10 8.78E-10 11.24847361

LINC00565 0.247973021 0.396774487 6.501682518 2.81E-10 8.78E-10 11.24834947

AP006219.1 0.061158439 0.032363886 6.501576122 2.81E-10 8.78E-10 11.24773202

AP005120.1 -0.181511141 0.101061601 -6.501477634 2.81E-10 8.79E-10 11.24716047

PINK1-AS -0.234447961 1.779248027 -6.499349549 2.85E-10 8.90E-10 11.23481233

AC084026.1 -0.132872899 0.122011011 -6.499192609 2.85E-10 8.90E-10 11.23390182

MCPH1-AS1 -0.171940039 0.555628849 -6.497608244 2.88E-10 8.98E-10 11.22471083

INO80-AS1 0.079951242 0.064017281 6.492307015 2.97E-10 9.27E-10 11.1939708

AC131097.3 0.214706828 0.354415656 6.491382829 2.98E-10 9.32E-10 11.18861376

AC018682.1 0.154937047 0.379039788 6.490878295 2.99E-10 9.34E-10 11.18568949

Z73429.1 0.223494228 0.291057546 6.490413995 3.00E-10 9.37E-10 11.18299857

AC106801.1 0.043142008 0.034630206 6.490161466 3.00E-10 9.38E-10 11.18153506

AL356124.1 0.147457032 0.234753372 6.489808623 3.01E-10 9.39E-10 11.17949027

AP006287.2 -0.249201555 0.408154625 -6.48558769 3.09E-10 9.63E-10 11.15503588

AC004771.2 -0.219809746 0.382718804 -6.479288996 3.20E-10 9.99E-10 11.1185669

AL133255.1 -0.209867906 0.282597854 -6.476576374 3.26E-10 1.02E-09 11.10286956

ADCY6-DT -0.395495545 1.250779623 -6.475281769 3.28E-10 1.02E-09 11.09537977

AC103974.1 0.280639154 1.106924795 6.475194284 3.28E-10 1.02E-09 11.09487368

AC023590.1 0.184837692 0.222590098 6.475144765 3.28E-10 1.02E-09 11.09458722

SALRNA1 0.030088438 0.019244399 6.47109415 3.36E-10 1.05E-09 11.07116082

AC016747.2 -0.29698686 0.492640606 -6.469485622 3.39E-10 1.06E-09 11.06186122

AP001160.3 0.327366608 1.879555006 6.468598382 3.41E-10 1.06E-09 11.05673246

AC136443.4 -0.051027286 0.027205602 -6.466052462 3.46E-10 1.08E-09 11.04201865

LINC01982 0.162062511 0.08428255 6.465941297 3.47E-10 1.08E-09 11.04137629

AL023803.1 0.418774686 0.429890922 6.465145806 3.48E-10 1.08E-09 11.03677985

AL139407.1 -0.251537292 0.885401261 -6.464389182 3.50E-10 1.09E-09 11.0324084

AC022028.2 0.068367198 0.058531707 6.462751035 3.53E-10 1.10E-09 11.02294525

LINC00402 0.194145171 0.113875867 6.461946884 3.55E-10 1.10E-09 11.01830056

AC078880.3 -0.159933783 0.243501943 -6.45815775 3.63E-10 1.13E-09 10.99642104

AL136964.1 0.145867996 0.160168057 6.458120469 3.63E-10 1.13E-09 10.99620582

AC121154.1 0.292590528 0.151443768 6.455877101 3.68E-10 1.14E-09 10.98325678

TTTY6B -0.059433501 0.028852884 -6.448309641 3.85E-10 1.19E-09 10.93960232

TM4SF1-AS1 0.5690535 1.487078079 6.445090778 3.92E-10 1.22E-09 10.9210458

AC013731.1 0.267234513 0.798205103 6.440146847 4.03E-10 1.25E-09 10.89255854

NBR2 0.382937417 2.621014726 6.438788201 4.07E-10 1.26E-09 10.88473293

AC044840.1 0.078038577 0.050918687 6.437392051 4.10E-10 1.27E-09 10.87669265

AC022872.1 0.035153935 0.022197773 6.436269236 4.13E-10 1.28E-09 10.87022747

AL137796.1 0.155337321 0.167273275 6.435207156 4.15E-10 1.29E-09 10.86411282

AL807761.4 -0.113413401 0.062005886 -6.43512137 4.16E-10 1.29E-09 10.86361897

AL136298.1 0.077940124 0.056311645 6.432995783 4.21E-10 1.31E-09 10.85138396

AL603962.1 -0.175102054 0.085005931 -6.431704995 4.24E-10 1.31E-09 10.84395565

AC006299.1 -0.138177394 0.210012431 -6.431329712 4.25E-10 1.32E-09 10.84179617

AC010967.1 -0.429309607 0.856920977 -6.429958968 4.28E-10 1.33E-09 10.83390936

AC130456.4 0.068844066 0.052872564 6.427569358 4.34E-10 1.35E-09 10.8201635

AC099314.1 0.082720999 0.065368059 6.420792558 4.52E-10 1.40E-09 10.78120282

AC009133.1 -0.455725585 2.669680268 -6.419742973 4.55E-10 1.41E-09 10.77517151

AC008897.2 0.042433155 0.025271765 6.418852196 4.57E-10 1.42E-09 10.77005338

AC055822.1 0.445051444 1.523792257 6.416942826 4.62E-10 1.43E-09 10.75908459

AC090983.1 -0.044854967 0.037698457 -6.416727193 4.63E-10 1.43E-09 10.75784601

AL158825.2 0.216855225 0.399891405 6.416471499 4.64E-10 1.43E-09 10.75637735

LINC01977 0.336796982 0.710240186 6.416107563 4.65E-10 1.44E-09 10.75428705

AC103858.2 -0.190736711 0.254935456 -6.415933344 4.65E-10 1.44E-09 10.75328645

AC113189.3 -0.179637507 0.087207736 -6.415680266 4.66E-10 1.44E-09 10.75183296

AC022898.2 -0.078886229 0.098155951 -6.408730849 4.85E-10 1.50E-09 10.71193839

AC011498.2 -0.137044262 0.066530201 -6.404426351 4.97E-10 1.54E-09 10.68724458

AC007406.3 0.225270848 0.471124987 6.404060181 4.99E-10 1.54E-09 10.68514455

SOCS2-AS1 -0.292536867 1.064836136 -6.40179236 5.05E-10 1.56E-09 10.67214046

AC009303.2 -0.079042407 0.06715908 -6.401673965 5.06E-10 1.56E-09 10.67146167

IGBP1-AS1 0.245343377 0.678543825 6.401476348 5.06E-10 1.56E-09 10.67032868

LINC01301 -0.158568068 0.351437258 -6.401373645 5.06E-10 1.56E-09 10.66973988

AC026202.3 -0.067098887 0.048736838 -6.401059537 5.07E-10 1.57E-09 10.6679391

LINC02398 -0.247080037 0.307711242 -6.399139849 5.13E-10 1.58E-09 10.6569351

AC109347.2 -0.395357594 1.213251752 -6.397844898 5.17E-10 1.60E-09 10.64951366

CCDC148-AS1 0.030352357 0.016513082 6.397297615 5.19E-10 1.60E-09 10.64637751

AL683807.2 0.060385797 0.033825356 6.395905888 5.23E-10 1.61E-09 10.6384033

AC064853.1 0.06936553 0.039638248 6.392341735 5.34E-10 1.65E-09 10.61798793

MIR302CHG -0.204579251 0.363034432 -6.390443369 5.40E-10 1.66E-09 10.60711779

AC015712.4 -0.203945028 0.182172842 -6.390282843 5.40E-10 1.67E-09 10.60619873

AL078599.2 0.034795973 0.018799477 6.389587289 5.43E-10 1.67E-09 10.60221667

AC124067.3 0.119343628 0.067584752 6.388912074 5.45E-10 1.68E-09 10.59835138

PAPPA-AS2 -0.058964111 0.045153352 -6.387774214 5.48E-10 1.69E-09 10.59183838

AC092354.1 0.25014358 0.330070433 6.387506654 5.49E-10 1.69E-09 10.59030703

Z92544.1 -0.117713767 0.184959376 -6.387213651 5.50E-10 1.69E-09 10.58863011

AP000911.1 -0.157042749 0.311050301 -6.386736061 5.52E-10 1.70E-09 10.5858969

MIR202HG -0.172403439 0.220899923 -6.386494721 5.52E-10 1.70E-09 10.58451578

AC004542.2 -0.296921011 0.144144785 -6.385497555 5.56E-10 1.71E-09 10.57880976

FER1L6-AS1 0.066665077 0.035197243 6.384869761 5.58E-10 1.72E-09 10.57521773

FAM66A -0.190083698 0.324974286 -6.383579242 5.62E-10 1.73E-09 10.56783468

JAZF1-AS1 -0.091719463 0.126151351 -6.382828504 5.64E-10 1.74E-09 10.56354025

AC087623.2 0.249801953 0.819973174 6.382556275 5.65E-10 1.74E-09 10.56198312

AC090921.1 0.123390267 0.12497209 6.382361786 5.66E-10 1.74E-09 10.5608707

LINC02280 0.092742542 0.129740064 6.379781119 5.75E-10 1.77E-09 10.54611247

FO393419.2 0.056515395 0.038194129 6.37962212 5.75E-10 1.77E-09 10.54520334

AL121890.4 0.121724305 0.169318142 6.377501041 5.82E-10 1.79E-09 10.53307714

CYP4F26P 0.177037954 0.143090096 6.377425482 5.82E-10 1.79E-09 10.53264523

AC090505.2 0.074178998 0.039063417 6.376324747 5.86E-10 1.80E-09 10.52635364

AC107982.3 0.262611041 0.547778124 6.373362905 5.96E-10 1.83E-09 10.50942858

AC011503.2 0.194013012 0.393782911 6.366313257 6.21E-10 1.91E-09 10.46916922

AC019077.1 0.202742085 0.273379404 6.364204997 6.29E-10 1.93E-09 10.45713613

AL135936.1 0.039913889 0.021432823 6.362653692 6.35E-10 1.95E-09 10.44828392

CD200R1L-AS1 0.082416809 0.050292009 6.36240371 6.36E-10 1.95E-09 10.4468576

AC090192.2 0.163000947 0.084765408 6.361258929 6.40E-10 1.96E-09 10.44032643

AC012442.2 0.262999224 0.54005098 6.360922301 6.41E-10 1.97E-09 10.43840609

FLNC-AS1 0.110360952 0.061677431 6.360393877 6.43E-10 1.97E-09 10.43539177

AC100830.1 0.089696043 0.169637087 6.360268075 6.44E-10 1.97E-09 10.43467419

AL161909.2 0.078551038 0.055436905 6.358804562 6.49E-10 1.99E-09 10.42632697

AC009133.2 -0.447301827 0.218905812 -6.358773795 6.49E-10 1.99E-09 10.4261515

MANEA-DT -0.150989633 0.59387627 -6.3579593 6.52E-10 2.00E-09 10.42150667

AL121772.3 0.360844054 1.342861148 6.357114724 6.56E-10 2.01E-09 10.41669078

LINC00336 0.114172687 0.170375369 6.352022209 6.75E-10 2.07E-09 10.38766328

AC010731.2 0.091239248 0.058768171 6.351988216 6.76E-10 2.07E-09 10.38746959

AP000785.1 -0.349902622 0.516302147 -6.35023893 6.82E-10 2.09E-09 10.37750288

SMAD9-IT1 0.150476708 0.149818043 6.348970589 6.88E-10 2.10E-09 10.37027775

AC009686.1 0.057560658 0.035362935 6.348871175 6.88E-10 2.10E-09 10.36971149

AC126178.1 0.136889358 0.102534728 6.348785894 6.88E-10 2.10E-09 10.36922573

AC243830.2 -0.27944577 0.630913156 -6.346281067 6.98E-10 2.14E-09 10.35496062

AL358394.1 0.227002732 0.127099949 6.345970306 7.00E-10 2.14E-09 10.35319113

AL022328.1 -0.361658525 1.887696468 -6.344454473 7.06E-10 2.16E-09 10.34456088

AL590705.1 -0.290171391 0.71321989 -6.343799522 7.08E-10 2.16E-09 10.34083248

AL354979.1 0.056352403 0.029891012 6.342641791 7.13E-10 2.18E-09 10.33424268

EGLN3-AS1 0.196670729 0.102089686 6.337263636 7.36E-10 2.25E-09 10.30364272

AC105053.1 -0.06067962 0.069555059 -6.336589065 7.39E-10 2.26E-09 10.29980608

LINC02308 0.118052889 0.10194251 6.336512345 7.39E-10 2.26E-09 10.29936975

AL596202.1 -0.17206574 0.94789514 -6.334687074 7.47E-10 2.28E-09 10.28899018

AC008551.1 0.166009191 0.121889106 6.332828765 7.55E-10 2.30E-09 10.27842517

AL121721.1 0.185557509 0.100219021 6.330701468 7.64E-10 2.33E-09 10.26633389

LINC02251 0.113220781 0.070471436 6.330276034 7.66E-10 2.34E-09 10.26391617

AC138696.2 0.565011742 1.405750428 6.329663382 7.69E-10 2.35E-09 10.26043472

AL031587.2 0.080468333 0.062218716 6.325766036 7.87E-10 2.40E-09 10.23829392

AC011455.1 0.087194001 0.078435244 6.322804978 8.00E-10 2.44E-09 10.22147938

AC091053.2 -0.126202837 0.129404992 -6.321805709 8.05E-10 2.45E-09 10.21580638

AC090673.1 0.146496644 0.090826609 6.321758957 8.05E-10 2.45E-09 10.21554097

AL626787.1 0.153604906 0.205118093 6.314398865 8.40E-10 2.56E-09 10.17377872

PPM1K-DT -0.11813965 0.176285797 -6.311780893 8.53E-10 2.60E-09 10.15893324

AL133297.1 0.207346357 0.326288595 6.311553979 8.54E-10 2.60E-09 10.15764673

AL606760.3 -0.040479146 0.04249461 -6.308929265 8.67E-10 2.64E-09 10.14276835

U91328.3 -0.284528109 0.742538487 -6.307896932 8.72E-10 2.66E-09 10.13691783

AL121772.1 0.347079052 0.869563608 6.30733581 8.75E-10 2.66E-09 10.13373812

AC016542.1 0.17533689 0.238604845 6.30351131 8.95E-10 2.72E-09 10.11207176

AL355922.1 0.216500409 0.228240842 6.303032065 8.97E-10 2.73E-09 10.1093575

AC004231.3 0.101635348 0.053190667 6.301304326 9.06E-10 2.76E-09 10.09957361

AL133243.3 -0.173281159 0.660122502 -6.299787835 9.14E-10 2.78E-09 10.09098773

AC005674.1 0.181064816 0.274573682 6.298750741 9.20E-10 2.80E-09 10.08511698

AC007389.5 0.163360088 0.245788637 6.298383385 9.22E-10 2.80E-09 10.08303765

LINC02636 0.110753104 0.090263619 6.296088362 9.34E-10 2.84E-09 10.07004938

AC114781.2 0.114398251 0.087572775 6.291039998 9.62E-10 2.92E-09 10.04149229

AC123768.1 0.084357414 0.061422204 6.29079949 9.63E-10 2.93E-09 10.04013226

AC008764.7 -0.064927977 0.03152026 -6.28915048 9.72E-10 2.95E-09 10.03080852

AC018742.1 0.194434671 0.185941083 6.287469491 9.82E-10 2.98E-09 10.02130596

AC123595.1 -0.390997677 0.467485123 -6.282440252 1.01E-09 3.07E-09 9.99288792

AL359091.1 -0.042719596 0.03413835 -6.27691415 1.04E-09 3.17E-09 9.961683135

AC117490.2 0.156046708 0.287308452 6.274430256 1.06E-09 3.21E-09 9.947664186

AC016027.2 -0.049496594 0.059461978 -6.274312021 1.06E-09 3.21E-09 9.946996987

HOXA-AS3 0.200068308 0.190507533 6.272861169 1.07E-09 3.24E-09 9.938810624

AC104964.4 -0.285060942 0.985046844 -6.27180758 1.07E-09 3.26E-09 9.93286674

AL645941.3 -0.138536169 0.06725447 -6.271674849 1.08E-09 3.26E-09 9.932117987

LINC01944 0.058079496 0.03243455 6.265568525 1.11E-09 3.38E-09 9.897685015

AL355512.1 0.230888883 0.199952393 6.26457941 1.12E-09 3.39E-09 9.892109998

AC022144.1 0.43112571 1.528357827 6.262786992 1.13E-09 3.43E-09 9.882009047

AC011503.1 -0.08060387 0.065335539 -6.262094983 1.14E-09 3.44E-09 9.878109935

AC004941.1 -0.035034982 0.027476843 -6.262048317 1.14E-09 3.44E-09 9.877847006

AC006566.1 0.223812546 0.345612759 6.261301448 1.14E-09 3.46E-09 9.873639195

AL020998.1 0.081963733 0.043068935 6.260562242 1.15E-09 3.47E-09 9.869474944

AC091544.7 -0.22803419 0.2325293 -6.259140242 1.16E-09 3.50E-09 9.861465338

AC092723.1 0.054520296 0.028948329 6.257281124 1.17E-09 3.53E-09 9.850995788

AC073352.2 -0.130603772 0.083517297 -6.257162126 1.17E-09 3.54E-09 9.850325738

AC134312.4 0.112619197 0.087847593 6.256879415 1.17E-09 3.54E-09 9.848733905

HMGN3-AS1 0.200208004 0.96076444 6.254979378 1.18E-09 3.58E-09 9.838037021

AL157400.2 0.078012547 0.057152248 6.254473005 1.19E-09 3.59E-09 9.835186662

AL034347.1 0.049845042 0.032309352 6.252620324 1.20E-09 3.63E-09 9.824759544

AL451164.1 0.036752666 0.019806264 6.252316189 1.20E-09 3.63E-09 9.823048069

Z95624.1 -0.081168663 0.060871214 -6.251672735 1.21E-09 3.65E-09 9.819427345

LINC01778 0.154555667 0.160401515 6.249188521 1.22E-09 3.70E-09 9.805451419

AC131235.2 0.32062711 0.766475308 6.247899895 1.23E-09 3.72E-09 9.798203486

KIAA0087 -0.101523309 0.125115691 -6.247019305 1.24E-09 3.74E-09 9.793251255

AL356481.3 0.267552377 0.925097808 6.242462172 1.27E-09 3.84E-09 9.76763188

C11orf44 0.028225977 0.02001238 6.242273202 1.27E-09 3.84E-09 9.766569842

LINC00112 0.107997797 0.079339409 6.241178634 1.28E-09 3.87E-09 9.760418745

AL133297.2 0.401019247 0.450985163 6.236165457 1.32E-09 3.98E-09 9.732257369

AL139089.1 0.344908785 1.367791762 6.235559951 1.32E-09 3.99E-09 9.728857176

AC104072.1 0.319932429 0.252665831 6.234872417 1.33E-09 4.01E-09 9.724996678

LINC02385 0.054086515 0.03419121 6.231421197 1.36E-09 4.09E-09 9.705623225

AP001625.2 0.416039773 1.058422849 6.230658074 1.36E-09 4.10E-09 9.701340581

AL078459.1 0.099257718 0.194571429 6.229168866 1.37E-09 4.14E-09 9.692984351

AL353804.2 0.200712053 0.271850068 6.228445866 1.38E-09 4.15E-09 9.688928036

AC097658.2 0.06845282 0.048114514 6.22686602 1.39E-09 4.19E-09 9.680065778

Z92544.2 -0.176681886 0.77292714 -6.226743784 1.39E-09 4.19E-09 9.679380162

AC008063.1 0.132290153 0.099930598 6.226495302 1.39E-09 4.20E-09 9.677986472

AC007285.1 0.15246575 0.31364053 6.226155916 1.40E-09 4.20E-09 9.676082984

AC125421.2 -0.130558545 0.081131253 -6.226101721 1.40E-09 4.21E-09 9.675779033

LINC01976 0.494965302 0.516789591 6.224755625 1.41E-09 4.24E-09 9.668230166

AC027243.2 0.063621948 0.055439631 6.223095392 1.42E-09 4.28E-09 9.658921417

AL359643.3 0.21720428 0.934526074 6.219856147 1.45E-09 4.36E-09 9.640765019

AC025262.1 0.074084545 0.065108674 6.217430517 1.47E-09 4.42E-09 9.627173979

LINC02284 -0.129884547 0.146505653 -6.215948471 1.48E-09 4.45E-09 9.618872007

AC091117.2 0.03905996 0.024164692 6.212236796 1.51E-09 4.55E-09 9.59808726

AC004156.1 -0.054529706 0.121366756 -6.211425562 1.52E-09 4.57E-09 9.593545809

AC025181.2 0.339781825 2.693311488 6.205052686 1.58E-09 4.74E-09 9.557885614

AL645941.1 -0.314632635 0.580186375 -6.204347943 1.58E-09 4.75E-09 9.55394393

AC068831.1 -0.141913641 0.421781275 -6.203672243 1.59E-09 4.77E-09 9.550165023

LINC01275 0.17233 0.184000506 6.202978561 1.60E-09 4.79E-09 9.546285894

AL049780.2 0.268735638 0.500751833 6.201890405 1.61E-09 4.82E-09 9.540201533

AC138028.4 -0.324590423 1.732170085 -6.196556786 1.65E-09 4.97E-09 9.510391215

AL355607.1 0.266498335 0.234820636 6.195180829 1.67E-09 5.00E-09 9.502704133

TRPM2-AS 0.354790161 0.364657958 6.190614775 1.71E-09 5.13E-09 9.477204638

AC087269.1 0.089648782 0.05894572 6.190273645 1.72E-09 5.14E-09 9.475300173

AL133387.1 0.11895045 0.091682418 6.189813444 1.72E-09 5.16E-09 9.47273109

AIRN -0.039020942 0.026904284 -6.188769758 1.73E-09 5.19E-09 9.466905258

AC120114.1 0.173583902 0.532775075 6.188021899 1.74E-09 5.21E-09 9.462731202

LINC01865 -0.070169797 0.054432796 -6.187523885 1.74E-09 5.22E-09 9.459951843

AC087762.1 0.077080707 0.05882453 6.184681982 1.77E-09 5.30E-09 9.444094921

AC073862.1 -0.093120226 0.045206614 -6.182162472 1.80E-09 5.38E-09 9.430041721

AC234917.1 -0.224660693 0.225758257 -6.180842013 1.81E-09 5.42E-09 9.422678362

AL050344.1 0.032481946 0.01760883 6.178377054 1.84E-09 5.50E-09 9.408936207

LINC02812 0.149008919 0.126545599 6.176257957 1.86E-09 5.56E-09 9.397125743

AD001527.1 0.252155233 0.55290494 6.173558267 1.89E-09 5.65E-09 9.382084127

AC004884.2 0.135433928 0.183912394 6.172075746 1.90E-09 5.69E-09 9.373826335

LINC00685 0.445555029 1.644659104 6.169663603 1.93E-09 5.77E-09 9.360393847

SIDT1-AS1 0.109365667 0.081921001 6.166509038 1.96E-09 5.87E-09 9.342833377

SMAD1-AS2 -0.053479586 0.043111369 -6.160476381 2.03E-09 6.08E-09 9.309271498

AC073072.1 0.172706174 0.200012265 6.16030593 2.03E-09 6.08E-09 9.308323597

AC016825.1 -0.263997539 0.137121517 -6.155304855 2.09E-09 6.26E-09 9.280521341

AC016876.1 -0.386724887 3.284289603 -6.152417077 2.13E-09 6.36E-09 9.264475679

AC246817.2 0.102133862 0.089726004 6.151206973 2.14E-09 6.40E-09 9.257753647

AL138889.1 -0.030977403 0.021453047 -6.149821251 2.16E-09 6.45E-09 9.25005737

AL133499.1 -0.099266179 0.057066608 -6.145526105 2.21E-09 6.61E-09 9.226211031

AC253536.6 -0.399065339 1.342421469 -6.145040436 2.22E-09 6.62E-09 9.223515474

AC007938.3 -0.298458575 1.387904961 -6.144651334 2.22E-09 6.64E-09 9.221356001

AC016394.1 0.326100119 1.941225022 6.143428061 2.24E-09 6.68E-09 9.214567697

LINC01799 -0.257586287 0.271035752 -6.143167113 2.24E-09 6.69E-09 9.213119759

AL357033.1 0.155225804 0.197358576 6.14128378 2.27E-09 6.76E-09 9.20267106

AC103810.5 -0.208816069 0.329224666 -6.140407221 2.28E-09 6.79E-09 9.197808799

AC040977.2 -0.179393227 0.088251618 -6.139291213 2.29E-09 6.83E-09 9.191619126

AC005609.2 -0.070755782 0.034349456 -6.139038861 2.30E-09 6.84E-09 9.190219642

ZSCAN16-AS1 0.366529794 2.49300691 6.138194756 2.31E-09 6.87E-09 9.18553877

AC009065.3 -0.142479507 0.165895266 -6.137315281 2.32E-09 6.91E-09 9.180662302

AL512343.2 0.198439063 0.382084945 6.137033003 2.32E-09 6.92E-09 9.17909726

AC015712.5 -0.072229037 0.035393255 -6.134848641 2.35E-09 7.00E-09 9.166988403

LINC01633 0.102276811 0.065872112 6.133399353 2.37E-09 7.06E-09 9.158956283

AC116563.1 0.101026331 0.075007255 6.13102463 2.40E-09 7.15E-09 9.145798587

AL592429.2 0.124528409 0.084412961 6.130129854 2.41E-09 7.19E-09 9.140841937

SYNJ2-IT1 0.055525968 0.035157261 6.12928276 2.43E-09 7.22E-09 9.136149956

AL021707.8 0.671892076 1.70925337 6.127022372 2.46E-09 7.31E-09 9.123632413

AL136456.1 0.090733591 0.05238938 6.126738768 2.46E-09 7.32E-09 9.12206214

ELN-AS1 0.4530198 2.249399796 6.123224741 2.51E-09 7.47E-09 9.102610293

CLSTN2-AS1 -0.040245294 0.033507207 -6.12143924 2.54E-09 7.54E-09 9.092730112

LINC00434 -0.186352412 0.170945921 -6.121359351 2.54E-09 7.54E-09 9.092288095

FARSA-AS1 0.153122131 0.124817793 6.119777284 2.56E-09 7.61E-09 9.083535656

AC099342.1 0.088811184 0.046592187 6.119495073 2.56E-09 7.62E-09 9.081974573

AL050327.1 0.13890171 0.101083918 6.118116116 2.58E-09 7.68E-09 9.074347561

AC004522.3 -0.16071547 0.19618255 -6.117152345 2.60E-09 7.72E-09 9.069017756

MIR210HG 0.698263644 2.7077163 6.116592245 2.61E-09 7.74E-09 9.065920631

SMYD3-IT1 0.059611316 0.033614468 6.116379574 2.61E-09 7.75E-09 9.064744701

LINC01013 0.111333424 0.151157947 6.115601115 2.62E-09 7.78E-09 9.060440637

EMX2OS -0.130733931 0.211103838 -6.115581973 2.62E-09 7.78E-09 9.060334804

ESRG -0.03037401 0.020499654 -6.113355158 2.65E-09 7.88E-09 9.048025299

LINC01010 0.120552363 0.173183718 6.113223849 2.66E-09 7.88E-09 9.047299553

AC245033.2 0.045295924 0.027251429 6.113047536 2.66E-09 7.89E-09 9.04632509

AC018648.1 -0.281959628 0.867893767 -6.112229966 2.67E-09 7.92E-09 9.041806766

AC087752.4 -0.295995418 1.01726214 -6.112144838 2.67E-09 7.92E-09 9.041336337

AC100791.1 0.143195175 0.074574648 6.111927556 2.68E-09 7.93E-09 9.040135618

AC015853.2 0.106997779 0.057185094 6.111841777 2.68E-09 7.94E-09 9.039661601

AC002428.2 -0.120106918 0.070770059 -6.106527847 2.76E-09 8.18E-09 9.010307371

AL359397.1 0.166592364 0.212144306 6.106033751 2.77E-09 8.20E-09 9.007579021

LINC02734 0.107891019 0.106678149 6.105141392 2.78E-09 8.24E-09 9.002651946

AC024270.4 0.165072591 0.255383457 6.104061853 2.80E-09 8.28E-09 8.996692154

AC104985.1 0.303938697 0.236993698 6.103670519 2.80E-09 8.30E-09 8.99453193

LINC02830 0.177282719 0.133340018 6.103665735 2.80E-09 8.30E-09 8.994505522

AC114956.2 0.163006608 0.288044539 6.103386958 2.81E-09 8.31E-09 8.992966699

OSMR-AS1 0.196873952 0.548799748 6.101412639 2.84E-09 8.40E-09 8.982070266

AC022960.1 0.131621573 0.131813656 6.101382741 2.84E-09 8.40E-09 8.981905283

AC097467.3 -0.091273307 0.063733353 -6.10058604 2.85E-09 8.44E-09 8.97750904

CPEB2-DT -0.051294026 0.069305512 -6.10029014 2.86E-09 8.45E-09 8.975876367

AC011442.1 0.314264889 0.501980416 6.100064946 2.86E-09 8.46E-09 8.974633864

AL031733.2 0.159388584 0.130478639 6.099876833 2.86E-09 8.47E-09 8.973595984

AC008280.3 0.258262499 1.170010347 6.097965458 2.90E-09 8.56E-09 8.963051781

AC004828.1 0.079596494 0.043170971 6.095742592 2.93E-09 8.66E-09 8.950792567

LINC01583 0.047179224 0.027920957 6.095608801 2.93E-09 8.67E-09 8.950054816

AC005845.1 0.074179223 0.075902232 6.095524633 2.94E-09 8.67E-09 8.949590705

AC008549.2 -0.059491056 0.031309816 -6.094889439 2.95E-09 8.70E-09 8.946088342

LINC02519 0.236055675 0.476608327 6.094693215 2.95E-09 8.71E-09 8.945006451

AL365205.4 0.166400468 0.10029372 6.092222201 2.99E-09 8.83E-09 8.931384793

AC004917.1 0.057964926 0.060872094 6.091545281 3.00E-09 8.86E-09 8.927653991

AL450327.1 -0.033757712 0.021200378 -6.090585154 3.02E-09 8.91E-09 8.922362886

AL355303.1 0.067719589 0.042445851 6.090397787 3.02E-09 8.91E-09 8.921330418

AC004817.1 0.042047203 0.024296611 6.090205958 3.03E-09 8.92E-09 8.920273389

CACNA1C-IT2 0.088245253 0.046300996 6.089722387 3.03E-09 8.94E-09 8.917608894

AC007731.3 0.053674285 0.036422939 6.087708169 3.07E-09 9.04E-09 8.906512314

PLCXD2-AS1 0.092975435 0.058952135 6.087381695 3.07E-09 9.06E-09 8.904714004

ELOVL2-AS1 -0.115151775 0.108586017 -6.084367063 3.13E-09 9.21E-09 8.888112257

AC005180.1 0.18891753 0.218343272 6.082368162 3.16E-09 9.31E-09 8.877107843

AC040160.1 0.140068288 0.565343568 6.082005742 3.17E-09 9.33E-09 8.875112949

AC027807.2 0.254607044 0.406827726 6.08123472 3.18E-09 9.37E-09 8.870869278

C1orf229 -0.135120145 0.4038164 -6.079561396 3.21E-09 9.46E-09 8.861660862

AL136418.1 0.338404409 0.327233267 6.079526193 3.21E-09 9.46E-09 8.861467161

AC012574.2 0.192573318 0.102157057 6.07908075 3.22E-09 9.48E-09 8.859016213

AC084026.2 0.276066412 0.455920997 6.077636066 3.25E-09 9.55E-09 8.851068173

AC121761.1 0.285259152 1.080093725 6.07118809 3.37E-09 9.90E-09 8.815612672

LINC02836 0.032843899 0.017795067 6.070199853 3.39E-09 9.96E-09 8.810181327

AC092650.1 0.019408232 0.015356339 6.069093604 3.41E-09 1.00E-08 8.80410224

LINC01927 0.201862274 0.125371826 6.06901128 3.41E-09 1.00E-08 8.803649885

AC090825.1 0.278326339 0.638367645 6.067326701 3.44E-09 1.01E-08 8.794394562

AC007608.1 0.092520348 0.162231845 6.067205666 3.44E-09 1.01E-08 8.793729656

AC007879.3 0.191496977 0.316143443 6.066501827 3.46E-09 1.02E-08 8.789863334

AC093382.1 0.166128772 0.22322953 6.064811436 3.49E-09 1.03E-08 8.780579177

LHX1-DT 0.253357604 0.151142391 6.064511551 3.50E-09 1.03E-08 8.778932333

SNHG32 -0.453339389 6.840416505 -6.06012322 3.58E-09 1.05E-08 8.754840937

AC233296.1 0.051326651 0.035758247 6.059310758 3.60E-09 1.06E-08 8.750382166

AC007114.1 0.409688228 2.313541893 6.058065491 3.62E-09 1.06E-08 8.743549103

AC138123.1 -0.144538431 0.207485898 -6.057623095 3.63E-09 1.07E-08 8.741121847

GFOD1-AS1 0.109458107 0.084916986 6.056773714 3.65E-09 1.07E-08 8.736462026

AC005529.1 0.078130368 0.045603605 6.055565285 3.68E-09 1.08E-08 8.729833328

BX255923.2 -0.062512325 0.054277871 -6.055137278 3.68E-09 1.08E-08 8.727485802

AC022960.2 -0.110276869 0.136003074 -6.05482956 3.69E-09 1.08E-08 8.725798116

AC073316.3 -0.187084716 0.340836167 -6.054449026 3.70E-09 1.08E-08 8.723711161

AP001178.3 -0.098977853 0.158319089 -6.0540007 3.71E-09 1.09E-08 8.721252559

AP003469.3 0.07765246 0.05855714 6.053585969 3.72E-09 1.09E-08 8.718978321

AC024075.1 0.358881007 1.737102769 6.053298368 3.72E-09 1.09E-08 8.717401291

AC023908.3 -0.148583781 0.566796532 -6.052827444 3.73E-09 1.09E-08 8.714819158

LINC01592 0.034031096 0.023650273 6.051353642 3.76E-09 1.10E-08 8.706739178

AC023669.1 0.035495328 0.01915932 6.051335455 3.76E-09 1.10E-08 8.706639482

HOXD-AS2 0.241378606 0.437888475 6.050891165 3.77E-09 1.10E-08 8.70420402

AC011754.1 -0.624103721 0.679927117 -6.045852091 3.88E-09 1.14E-08 8.676591479

AP000919.4 0.188174954 0.490650275 6.043229237 3.94E-09 1.15E-08 8.662226408

PCAT1 0.150007132 0.205116537 6.039907338 4.01E-09 1.17E-08 8.64403998

AL158850.1 -0.046088114 0.027824296 -6.039723681 4.02E-09 1.17E-08 8.643034747

ANKRD10-IT1 0.530816314 4.252626623 6.038143175 4.05E-09 1.18E-08 8.634384981

AC069234.5 -0.277906164 0.686977016 -6.033616168 4.16E-09 1.22E-08 8.609619785

AJ009632.2 0.155571886 0.221935121 6.030949075 4.22E-09 1.23E-08 8.595036359

AL137129.1 -0.144688671 0.085388938 -6.027713086 4.30E-09 1.26E-08 8.577349253

AC133919.1 0.061103932 0.034408654 6.027560896 4.30E-09 1.26E-08 8.576517611

AP003680.1 -0.047044911 0.073651623 -6.026237774 4.33E-09 1.27E-08 8.569288113

AC009269.2 0.093885545 0.081951209 6.023784441 4.39E-09 1.28E-08 8.555886578

AC020916.2 0.156345008 0.187401013 6.02353088 4.40E-09 1.28E-08 8.554501732

AC108751.4 0.261571896 0.515662766 6.02273067 4.42E-09 1.29E-08 8.550131623

AC010422.4 0.233478243 0.430748346 6.022282485 4.43E-09 1.29E-08 8.547684196

AC013275.1 -0.855384246 3.711374758 -6.02113105 4.46E-09 1.30E-08 8.541397179

AL031283.2 -0.06406711 0.049614731 -6.01934535 4.50E-09 1.31E-08 8.531648892

AC131212.1 0.130985333 0.150296511 6.019120515 4.51E-09 1.31E-08 8.530421663

AL162430.2 0.159655163 0.210182738 6.013371456 4.66E-09 1.36E-08 8.499053908

AC007570.1 0.099217462 0.136792959 6.012616926 4.67E-09 1.36E-08 8.494938875

AC132192.1 0.162922846 0.269100482 6.011674658 4.70E-09 1.37E-08 8.489800548

LINC02421 -0.060741593 0.141143179 -6.011065884 4.72E-09 1.37E-08 8.48648116

AC090115.1 0.106992749 0.130889142 6.010261702 4.74E-09 1.38E-08 8.482096712

AC027243.1 0.107896613 0.080995412 6.008327302 4.79E-09 1.39E-08 8.471552194

DSCR9 -0.102009432 0.202669836 -6.008249534 4.79E-09 1.39E-08 8.471128334

AC097717.1 -0.117489495 0.084714437 -6.00784528 4.80E-09 1.40E-08 8.468925094

AC097359.2 0.205862147 1.189578641 6.004550345 4.89E-09 1.42E-08 8.45097172

AC020703.1 0.040927044 0.021954127 6.003208075 4.93E-09 1.43E-08 8.443660276

WWTR1-IT1 0.149090283 0.186436634 6.003023573 4.93E-09 1.44E-08 8.442655382

LINC00626 0.108527319 0.075509657 6.000752774 4.99E-09 1.45E-08 8.430289486

AC024267.5 -0.13834719 0.254765711 -5.99919026 5.04E-09 1.47E-08 8.421782838

AC006116.8 -0.108382924 0.19480006 -5.997947024 5.07E-09 1.48E-08 8.415015693

LINC00997 0.277941246 2.205142114 5.996466789 5.12E-09 1.49E-08 8.406959995

AC007365.1 0.133197866 0.221703022 5.994394435 5.17E-09 1.50E-08 8.39568459

AL157895.1 0.166621593 0.326059898 5.992985399 5.22E-09 1.52E-08 8.388020019

AC008915.1 -0.094151918 0.134862648 -5.991833873 5.25E-09 1.53E-08 8.381757276

LINC02269 0.074890722 0.043754583 5.99059919 5.29E-09 1.54E-08 8.375043353

AL021153.1 0.069142953 0.040815183 5.990494037 5.29E-09 1.54E-08 8.374471611

SPATA13-AS1 0.059929897 0.037221284 5.988421693 5.35E-09 1.55E-08 8.363205363

AC226119.1 -0.108668893 0.068434316 -5.986302917 5.41E-09 1.57E-08 8.351689959

AC110015.1 0.115908611 0.0786194 5.984793153 5.46E-09 1.58E-08 8.343486507

AL133243.2 0.22796387 1.266730899 5.984618676 5.46E-09 1.59E-08 8.342538581

LINC00544 0.042666059 0.034098659 5.984021651 5.48E-09 1.59E-08 8.339295125

METTL14-DT -0.101960046 0.134177463 -5.983859302 5.49E-09 1.59E-08 8.338413177

LINC02262 0.053907321 0.028632932 5.982353804 5.53E-09 1.60E-08 8.330235614

AC010737.1 0.068875453 0.048134949 5.98024806 5.60E-09 1.62E-08 8.318800434

LINC01993 0.097689918 0.105687507 5.976488195 5.72E-09 1.66E-08 8.298390715

AC011284.1 -0.091975821 0.092544795 -5.976243859 5.72E-09 1.66E-08 8.297064745

AL138828.1 -0.182320667 0.118879302 -5.975523656 5.75E-09 1.67E-08 8.293156572

LINC01508 0.150937909 0.158422437 5.974877279 5.77E-09 1.67E-08 8.289649347

AP002761.2 0.07493262 0.059667148 5.972731107 5.84E-09 1.69E-08 8.278006474

AC010547.3 0.060077337 0.041997562 5.972468622 5.85E-09 1.69E-08 8.27658274

AC093274.1 -0.029795516 0.025399839 -5.972156762 5.86E-09 1.70E-08 8.274891254

AC104170.2 0.104192559 0.164815403 5.971687881 5.87E-09 1.70E-08 8.27234825

AL354766.2 0.399983784 0.216822417 5.969761502 5.93E-09 1.72E-08 8.261902114

LINC02804 -0.285159675 0.533668179 -5.969721989 5.94E-09 1.72E-08 8.261687877

AC005632.5 0.103688723 0.06864351 5.969206241 5.95E-09 1.72E-08 8.258891622

AC023632.2 0.128892527 0.363995056 5.968871949 5.96E-09 1.72E-08 8.257079275

AC145146.1 0.07219168 0.038040873 5.968245743 5.98E-09 1.73E-08 8.253684561

AC132872.3 -0.46126199 2.405439659 -5.96666071 6.04E-09 1.75E-08 8.245093259

AC006460.1 0.12966354 0.132236029 5.966608057 6.04E-09 1.75E-08 8.244807899

AC087239.1 0.343515148 0.778480646 5.966483613 6.04E-09 1.75E-08 8.24413346

GTSCR1 0.166595298 0.086614828 5.964570392 6.11E-09 1.76E-08 8.233766007

LINC01587 -0.176510937 0.240409093 -5.963382445 6.15E-09 1.78E-08 8.227330064

AP003064.2 0.094832144 0.089802501 5.961027904 6.23E-09 1.80E-08 8.214576935

AC025188.1 0.102929982 0.143805157 5.959824757 6.27E-09 1.81E-08 8.208061791

NFIA-AS1 -0.209272616 0.137936278 -5.957003198 6.37E-09 1.84E-08 8.192787002

LCMT1-AS1 -0.209436668 0.551496526 -5.955379877 6.43E-09 1.86E-08 8.184001652

AC105020.3 -0.099026268 0.075458932 -5.953416922 6.50E-09 1.88E-08 8.173380818

AL023284.4 0.468349792 2.950367423 5.951710485 6.56E-09 1.89E-08 8.164150221

AC012360.2 0.300736514 0.839919334 5.949244606 6.65E-09 1.92E-08 8.150815393

AC003102.1 0.314685408 1.679864095 5.94396637 6.85E-09 1.97E-08 8.122287162

PIK3CD-AS1 0.060448396 0.068201574 5.943088934 6.88E-09 1.98E-08 8.117546722

AC020661.4 -0.03986361 0.029685412 -5.941527344 6.94E-09 2.00E-08 8.109111477

RASSF1-AS1 -0.201685022 0.68151987 -5.937773852 7.08E-09 2.04E-08 8.0888436

AC009262.1 0.072882928 0.041799181 5.937660553 7.09E-09 2.04E-08 8.088231976

AL132671.1 0.079447825 0.041774413 5.93517584 7.19E-09 2.07E-08 8.074821119

AC131238.1 -0.341069571 0.354640225 -5.935156684 7.19E-09 2.07E-08 8.074717742

FENDRR 0.39703887 0.853993735 5.935048268 7.19E-09 2.07E-08 8.074132692

FP565260.5 -0.0881125 0.062576148 -5.934615537 7.21E-09 2.08E-08 8.071797595

IGF2-AS -0.393161962 0.73898372 -5.934244082 7.22E-09 2.08E-08 8.069793273

AP001180.4 -0.077474856 0.061438735 -5.932495966 7.29E-09 2.10E-08 8.060362024

AP001330.4 0.336158189 1.672070491 5.930610158 7.37E-09 2.12E-08 8.050190451

AC074194.1 -0.111398824 0.166396807 -5.928065224 7.48E-09 2.15E-08 8.03646789

LINC01840 0.104079732 0.063736817 5.927354144 7.50E-09 2.16E-08 8.032634526

AC005920.1 -0.177503911 0.349659023 -5.924903175 7.61E-09 2.19E-08 8.019424455

AL139385.1 0.215789794 0.41014601 5.924065641 7.64E-09 2.20E-08 8.014911391

AL355870.1 0.271340155 0.24082046 5.921171945 7.76E-09 2.23E-08 7.999322663

LINC00896 -0.348432628 0.80707395 -5.920773288 7.78E-09 2.24E-08 7.997175531

AL359880.1 0.295870265 0.338963886 5.920707587 7.78E-09 2.24E-08 7.996821684

AC027682.6 -0.289745608 1.206197595 -5.920365271 7.80E-09 2.24E-08 7.994978109

AL008638.1 0.153835357 0.080049393 5.918663998 7.87E-09 2.26E-08 7.985817032

LINC02753 0.046016014 0.033918814 5.917753881 7.91E-09 2.27E-08 7.980917087

AL021368.1 -0.160208083 0.248621631 -5.917617923 7.92E-09 2.27E-08 7.980185157

ARHGAP27P1-BPTFP1-KPNA2P3 -0.424593878 2.72407453 -5.917163386 7.94E-09 2.28E-08 7.977738269

C1QTNF1-AS1 0.21292484 0.28713786 5.915583561 8.01E-09 2.30E-08 7.96923485

AC137932.1 0.352073578 1.06492886 5.915356516 8.02E-09 2.30E-08 7.968012932

AL355096.1 0.184793999 0.159443569 5.914767868 8.04E-09 2.31E-08 7.964845099

LINC01147 0.037243162 0.022300899 5.91348132 8.10E-09 2.32E-08 7.957922395

LINC00992 -0.442388579 1.709597811 -5.913234311 8.11E-09 2.33E-08 7.956593416

AC092828.1 -0.050204584 0.128214624 -5.911933859 8.17E-09 2.34E-08 7.949597378

DEPDC1-AS1 0.052666593 0.031364502 5.910722431 8.23E-09 2.36E-08 7.94308139

C15orf54 -0.058867167 0.080933472 -5.908189733 8.34E-09 2.39E-08 7.929462117

AL117336.1 0.43150994 1.177506878 5.908070202 8.35E-09 2.39E-08 7.928819472

AC067945.3 0.235414691 0.437251263 5.907138465 8.39E-09 2.40E-08 7.92381045

AP001528.1 -0.249122387 1.050637823 -5.906664932 8.41E-09 2.41E-08 7.921264981

AC010359.2 0.276580386 0.893683966 5.905282062 8.48E-09 2.43E-08 7.913832339

AC137056.1 -0.095296381 0.051031876 -5.902467779 8.61E-09 2.46E-08 7.898710532

AC092279.1 -0.273506262 1.296959376 -5.902017619 8.63E-09 2.47E-08 7.896292261

C18orf65 0.107308592 0.252712012 5.898754074 8.79E-09 2.51E-08 7.878764913

AL513327.2 0.120024637 0.098743387 5.895770477 8.93E-09 2.56E-08 7.862747984

AC068756.1 0.221397701 0.126792655 5.891010744 9.17E-09 2.62E-08 7.837209865

AL138820.1 -0.058502994 0.183247924 -5.889898727 9.22E-09 2.64E-08 7.831245819

AP001471.1 0.120436041 0.148611513 5.888328509 9.30E-09 2.66E-08 7.822825875

AL137856.1 0.072397406 0.081416216 5.888292414 9.31E-09 2.66E-08 7.822632346

AC011601.1 0.095787014 0.052269369 5.887755184 9.33E-09 2.67E-08 7.819752006

AC023906.3 -0.094758037 0.046001714 -5.886814311 9.38E-09 2.68E-08 7.814708068

LINC00342 0.693682833 4.238488368 5.88643827 9.40E-09 2.69E-08 7.812692329

AC010761.2 -0.203317771 0.106020145 -5.882925054 9.58E-09 2.74E-08 7.793865077

AC024933.1 -0.328721055 1.029538786 -5.88233163 9.62E-09 2.75E-08 7.790685837

NLGN4Y-AS1 0.06400285 0.039841535 5.881684378 9.65E-09 2.76E-08 7.787218512

AL359643.2 -0.074048128 0.255946635 -5.881601661 9.65E-09 2.76E-08 7.786775422

AC013270.1 0.167756647 0.18513244 5.881230856 9.67E-09 2.76E-08 7.784789186

AJ271736.1 -0.070757541 0.048788956 -5.880107048 9.73E-09 2.78E-08 7.778770071

AL353596.1 0.10366964 0.136677796 5.879144495 9.78E-09 2.79E-08 7.773615392

OTX2-AS1 -0.042170695 0.026655109 -5.879019854 9.79E-09 2.79E-08 7.772947962

AC106772.1 0.205617472 0.201992408 5.878725956 9.81E-09 2.80E-08 7.771374238

AL929236.1 0.128310196 0.143270323 5.877220238 9.89E-09 2.82E-08 7.76331264

AC116345.1 0.186906798 0.148221468 5.876473256 9.93E-09 2.83E-08 7.75931393

AC103563.7 0.15873915 0.097723429 5.875897887 9.96E-09 2.84E-08 7.756234175

AL031674.1 0.044414521 0.02599231 5.875367703 9.99E-09 2.85E-08 7.753396503

AC026691.1 0.206414024 0.669842008 5.874891689 1.00E-08 2.85E-08 7.750848934

U62317.2 0.402530272 3.173569897 5.874665695 1.00E-08 2.86E-08 7.749639503

AC147651.1 -0.307027955 0.976372444 -5.874482246 1.00E-08 2.86E-08 7.748657784

AC244250.1 0.102057687 0.080282691 5.873567501 1.01E-08 2.87E-08 7.743762935

AC079760.1 0.260782676 0.135077519 5.87258854 1.01E-08 2.89E-08 7.738525153

AC091114.1 0.025512557 0.01739956 5.87182355 1.02E-08 2.90E-08 7.734432687

LINC01435 -0.134114154 0.083502549 -5.87143987 1.02E-08 2.90E-08 7.732380282

AL033527.2 -0.171766078 0.228707558 -5.869165144 1.03E-08 2.94E-08 7.720214407

AL357033.2 0.16010098 0.13882213 5.868183125 1.04E-08 2.96E-08 7.714963489

AC027514.1 -0.062725116 0.073426698 -5.867335885 1.04E-08 2.97E-08 7.710433817

C1orf143 0.04315866 0.032968778 5.860080619 1.09E-08 3.09E-08 7.671666256

LINC01305 -0.065826527 0.08460256 -5.859468095 1.09E-08 3.10E-08 7.66839511

AC024559.1 0.069861854 0.042171217 5.859283516 1.09E-08 3.10E-08 7.667409433

LINC01952 0.201839317 0.159522811 5.857992632 1.10E-08 3.12E-08 7.660516646

WWC2-AS1 -0.170230737 0.183783233 -5.856818372 1.11E-08 3.14E-08 7.654247656

LINC02857 0.165427257 0.126187989 5.856645823 1.11E-08 3.14E-08 7.653326561

AC006019.2 -0.033915616 0.025081763 -5.855794186 1.11E-08 3.16E-08 7.648780703

AL157871.2 0.203026388 0.401721227 5.855171108 1.12E-08 3.17E-08 7.645455187

AC112187.3 0.223627954 0.385180762 5.855057164 1.12E-08 3.17E-08 7.644847076

AC021242.3 -0.138453441 0.342214312 -5.853214366 1.13E-08 3.20E-08 7.635013475

LYPLAL1-DT 0.148908228 0.31594568 5.852702544 1.13E-08 3.21E-08 7.632282724

AC145285.2 -0.174422234 0.833711395 -5.851771016 1.14E-08 3.23E-08 7.627313194

AF064860.1 0.223789374 0.416262323 5.850220699 1.15E-08 3.25E-08 7.619043974

AL158154.3 0.026885045 0.026935768 5.84902119 1.15E-08 3.27E-08 7.612647154

AP002856.2 0.031482684 0.019262277 5.845768604 1.17E-08 3.33E-08 7.595306962

AC005264.1 0.061824028 0.114926335 5.844056559 1.19E-08 3.36E-08 7.58618287

PRR7-AS1 0.230237452 0.534453394 5.842828237 1.19E-08 3.38E-08 7.579638063

AC012417.1 -0.082722769 0.098383759 -5.838368771 1.22E-08 3.47E-08 7.555886375

AL139420.2 0.10608364 0.06168569 5.837596929 1.23E-08 3.48E-08 7.551776949

AC104035.1 -0.223342113 0.413691318 -5.837183267 1.23E-08 3.49E-08 7.549574725

AC105389.2 0.113059653 0.122159466 5.835120715 1.24E-08 3.53E-08 7.538596159

AC099518.3 -0.097822935 0.08986957 -5.833748315 1.25E-08 3.55E-08 7.5312929

LINC01752 0.272603539 0.530615346 5.833554576 1.26E-08 3.55E-08 7.530262025

LINC02832 0.038902116 0.031467449 5.8321221 1.27E-08 3.58E-08 7.52264078

AC026904.1 0.115446235 0.070997659 5.8308964 1.27E-08 3.60E-08 7.516120869

INTS9-AS1 -0.133724096 0.187757969 -5.828872581 1.29E-08 3.64E-08 7.505357951

AC006130.1 -0.08366876 0.053216864 -5.828179234 1.29E-08 3.66E-08 7.50167135

AL159990.1 -0.091838014 0.044584144 -5.825187001 1.31E-08 3.72E-08 7.485765449

AC008741.1 -0.095977334 0.04659364 -5.824845478 1.32E-08 3.72E-08 7.483950433

AC005722.4 0.096096392 0.053331458 5.824580914 1.32E-08 3.73E-08 7.482544472

AC010325.2 0.067274385 0.040304706 5.822661404 1.33E-08 3.77E-08 7.47234527

AC015802.3 0.25037305 0.66818919 5.820461672 1.35E-08 3.81E-08 7.46066052

AC138123.2 0.040748679 0.024499831 5.819834704 1.35E-08 3.82E-08 7.457330795

AC103831.1 0.096938248 0.062974574 5.819025164 1.36E-08 3.84E-08 7.453031892

AL445223.1 -0.054682307 0.042296895 -5.814545361 1.39E-08 3.93E-08 7.429251649

AC102941.1 -0.045793708 0.039005057 -5.814056215 1.40E-08 3.94E-08 7.426656015

AC099524.1 0.202677794 0.202876455 5.812677866 1.41E-08 3.97E-08 7.419342819

AP000553.3 -0.144935343 0.173403224 -5.810430507 1.42E-08 4.02E-08 7.407421915

AC092068.2 -0.066483214 0.083791129 -5.809833239 1.43E-08 4.03E-08 7.404254402

LINC02721 0.076529426 0.040272795 5.80610798 1.46E-08 4.11E-08 7.384504116

AL591501.1 0.217520251 0.169759484 5.804492515 1.47E-08 4.15E-08 7.375942605

AL136309.4 -0.075938021 0.055832105 -5.803604483 1.48E-08 4.17E-08 7.371237118

AC005540.1 0.228367407 0.362692804 5.800280774 1.50E-08 4.24E-08 7.353630754

AC079584.2 0.122611889 0.089693109 5.798489431 1.52E-08 4.28E-08 7.344145081

AC022915.1 0.145010485 0.122545081 5.798153252 1.52E-08 4.29E-08 7.342365183

AC091173.1 0.022873661 0.016096735 5.797996342 1.52E-08 4.29E-08 7.341534455

AP000317.2 0.071541802 0.070472911 5.795092987 1.55E-08 4.36E-08 7.326166548

AC008267.5 -0.407158779 2.333866196 -5.794770408 1.55E-08 4.37E-08 7.324459478

AC018695.2 0.062093882 0.035304486 5.794246133 1.56E-08 4.38E-08 7.321685209

AC105206.1 -0.032430252 0.026581653 -5.794221158 1.56E-08 4.38E-08 7.321553055

AC116036.2 0.275518929 0.631287061 5.79204886 1.57E-08 4.43E-08 7.310060299

EDNRB-AS1 0.056080897 0.043803655 5.791495401 1.58E-08 4.44E-08 7.307132738

AC009511.2 0.025608612 0.014072259 5.791189598 1.58E-08 4.45E-08 7.305515269

AL353743.2 -0.138411115 0.124684234 -5.790039685 1.59E-08 4.48E-08 7.299433719

AL159990.2 -0.091324972 0.044848122 -5.789405205 1.60E-08 4.49E-08 7.296078562

AC138932.5 -0.285468817 1.027831186 -5.789026494 1.60E-08 4.50E-08 7.294076069

LINC01524 0.028755457 0.021184954 5.78752211 1.61E-08 4.53E-08 7.286122469

BMS1P14 -0.073024693 0.044754974 -5.785567008 1.63E-08 4.58E-08 7.275788486

AC115102.1 0.115777809 0.149607154 5.785199608 1.63E-08 4.59E-08 7.273846857

MAPKAPK5-AS1 -0.297820096 4.088975671 -5.784560288 1.64E-08 4.61E-08 7.270468436

AC015909.4 0.054089102 0.042848682 5.783505464 1.65E-08 4.63E-08 7.264894995

AL355102.3 -0.035144184 0.020886472 -5.781098899 1.67E-08 4.69E-08 7.252182398

AC008115.3 -0.325113421 1.529437429 -5.779486088 1.68E-08 4.73E-08 7.243665217

AC008764.2 0.359409731 2.475155676 5.778895659 1.69E-08 4.74E-08 7.240547676

BX323046.1 0.125658603 0.170253477 5.778605485 1.69E-08 4.75E-08 7.239015619

LINC01176 0.396861443 2.215789138 5.777995701 1.70E-08 4.77E-08 7.235796292

Z99289.2 0.086404668 0.106012165 5.773940417 1.74E-08 4.87E-08 7.214393701

AC005828.3 -0.088291795 0.068562098 -5.771652092 1.76E-08 4.93E-08 7.202322049

AC008443.5 -0.47125609 2.042545581 -5.77142489 1.76E-08 4.94E-08 7.201123701

AC016885.2 0.275970072 0.16029809 5.771115112 1.76E-08 4.94E-08 7.199489882

AC087190.2 0.02136163 0.013219931 5.769673049 1.78E-08 4.98E-08 7.191885143

AL138799.2 0.036838706 0.019850534 5.76737673 1.80E-08 5.04E-08 7.179778699

NEBL-AS1 -0.368825089 1.150651818 -5.765884658 1.81E-08 5.08E-08 7.171914463

DIP2A-IT1 0.087093168 0.077245302 5.765860056 1.81E-08 5.08E-08 7.171784805

AC148477.4 0.071327249 0.045713767 5.765073304 1.82E-08 5.10E-08 7.167638776

AP000755.1 0.028289614 0.015451728 5.758742183 1.88E-08 5.28E-08 7.134291944

AC117498.1 -0.234194889 0.113693441 -5.75733438 1.90E-08 5.32E-08 7.126880965

HAR1B 0.293030134 0.376930529 5.757151916 1.90E-08 5.32E-08 7.125920547

GRM5-AS1 -0.034272737 0.029738995 -5.755541573 1.92E-08 5.37E-08 7.117445407

LINC01338 0.147882648 0.104836147 5.751090141 1.96E-08 5.50E-08 7.094027942

AC020891.1 0.078704231 0.041391808 5.751084182 1.96E-08 5.50E-08 7.0939966

LINC01764 0.111312182 0.108137638 5.750879094 1.97E-08 5.50E-08 7.092918061

AL139241.1 -0.243585654 0.573790679 -5.74898358 1.99E-08 5.56E-08 7.082951246

EHMT2-AS1 0.295293378 0.487117744 5.748065474 2.00E-08 5.58E-08 7.078124716

AC011891.2 -0.077804984 0.037771598 -5.746729929 2.01E-08 5.62E-08 7.071104828

CASC16 0.089150546 0.085054601 5.746715428 2.01E-08 5.62E-08 7.071028615

AC010343.3 0.234090577 0.124425854 5.745779758 2.02E-08 5.65E-08 7.066111355

AL031963.2 0.082185986 0.059987523 5.74512465 2.03E-08 5.67E-08 7.062668937

LINC00470 -0.21381917 0.182624929 -5.743263814 2.05E-08 5.72E-08 7.05289251

AL022329.1 -0.126230597 0.126229388 -5.743119149 2.05E-08 5.73E-08 7.052132577

LINC01801 0.113645412 0.253856473 5.742070648 2.06E-08 5.76E-08 7.046625244

AC078842.1 0.047900358 0.027491023 5.741030236 2.07E-08 5.79E-08 7.041161218

AC010931.3 0.042714525 0.030676963 5.740399119 2.08E-08 5.81E-08 7.037847123

AC007014.1 0.035238616 0.023194314 5.739996308 2.08E-08 5.82E-08 7.035732055

LINC00917 -0.074490935 0.054140912 -5.739940244 2.09E-08 5.82E-08 7.035437684

AC010503.2 -0.181447155 0.088086256 -5.739670096 2.09E-08 5.83E-08 7.034019278

TRG-AS1 -0.303975054 0.833722749 -5.735948835 2.13E-08 5.94E-08 7.014486498

AC073052.2 -0.143253424 0.178947496 -5.735660019 2.13E-08 5.95E-08 7.012970946

AC072061.1 0.159671593 0.760309234 5.734181847 2.15E-08 6.00E-08 7.005215293

AC011676.2 0.11357745 0.09980844 5.733978881 2.15E-08 6.00E-08 7.004150502

AC119868.2 0.055144407 0.032998389 5.733394329 2.16E-08 6.02E-08 7.001084026

AC009495.1 0.246171215 0.438208485 5.732281858 2.17E-08 6.06E-08 6.99524887

LINC00415 0.063625821 0.043044731 5.730903256 2.19E-08 6.10E-08 6.988019102

AC011483.2 0.054421268 0.033954766 5.729496732 2.21E-08 6.15E-08 6.980644382

AC068308.1 0.047644809 0.031404316 5.728740569 2.22E-08 6.17E-08 6.976680266

AC105020.5 0.273063435 0.665048113 5.728357989 2.22E-08 6.18E-08 6.974674786

BX539320.1 0.254961674 0.60112435 5.727360745 2.23E-08 6.21E-08 6.969447777

LINC02106 0.466451493 0.60187931 5.727167942 2.23E-08 6.22E-08 6.968437294

SMCR2 0.135462818 0.16919906 5.72430517 2.27E-08 6.31E-08 6.953436784

AL391832.1 0.101946119 0.089728544 5.721883406 2.30E-08 6.39E-08 6.940751929

AC244035.1 0.213102169 0.164751968 5.721740567 2.30E-08 6.40E-08 6.940003897

AC090774.2 -0.116636966 0.102098345 -5.721418934 2.30E-08 6.41E-08 6.938319593

AC024257.4 -0.234787212 0.57086537 -5.720598455 2.31E-08 6.44E-08 6.934023334

AL157702.2 0.140185166 0.095500645 5.719308977 2.33E-08 6.48E-08 6.927272291

AC016999.1 0.148359223 0.236679707 5.717877544 2.35E-08 6.53E-08 6.919779516

AC006547.1 -0.224980349 1.729849481 -5.717225062 2.36E-08 6.55E-08 6.916364642

AC127496.5 0.200169959 0.449942075 5.716974764 2.36E-08 6.56E-08 6.915054754

AC026801.2 0.295151747 1.371838156 5.716537548 2.37E-08 6.57E-08 6.912766773

AL450344.2 0.042057362 0.02781596 5.716438194 2.37E-08 6.57E-08 6.912246863

AL137025.1 -0.045283435 0.064199248 -5.715597059 2.38E-08 6.60E-08 6.90784562

AL121852.1 0.140323069 0.201264925 5.714475087 2.39E-08 6.64E-08 6.90197572

AC009163.6 0.177339804 0.118056213 5.71297949 2.41E-08 6.69E-08 6.894152583

AC025165.5 0.270140911 1.730255841 5.711855154 2.43E-08 6.73E-08 6.888272549

AC011726.2 0.031516407 0.018308217 5.710723663 2.44E-08 6.77E-08 6.882356058

AC027702.1 0.16443792 0.613438432 5.710562245 2.44E-08 6.78E-08 6.881512091

STPG2-AS1 0.037962277 0.022412263 5.709345604 2.46E-08 6.82E-08 6.875151585

AP001476.1 -0.088395395 0.123093781 -5.708769704 2.47E-08 6.84E-08 6.872141217

AC006058.3 0.122134628 0.08745503 5.705999618 2.50E-08 6.94E-08 6.857664799

AC104066.3 -0.045199746 0.047539602 -5.705724558 2.51E-08 6.95E-08 6.856227663

AC005005.4 0.016690428 0.014372312 5.705713888 2.51E-08 6.95E-08 6.856171915

AC016168.1 0.062924741 0.041801844 5.701950731 2.56E-08 7.09E-08 6.836515852

AC016730.1 -0.17454214 0.193045824 -5.70137732 2.57E-08 7.11E-08 6.833521696

LINC01522 0.427424925 0.282598852 5.701262745 2.57E-08 7.11E-08 6.832923459

AC007938.1 -0.102607207 0.235868706 -5.700908883 2.57E-08 7.12E-08 6.831075869

FAM66D 0.172923549 0.363090299 5.700662888 2.58E-08 7.13E-08 6.829791532

AL356608.1 -0.113723719 0.12642143 -5.700548706 2.58E-08 7.13E-08 6.829195406

AC027348.1 -0.255039411 0.687711821 -5.698984414 2.60E-08 7.19E-08 6.821029467

AL139280.1 0.04365931 0.025774844 5.698125977 2.61E-08 7.22E-08 6.816549031

AP001269.4 -0.096161216 0.046682908 -5.69761087 2.62E-08 7.24E-08 6.813860807

AL163051.1 -0.205649972 0.924938926 -5.696677622 2.63E-08 7.28E-08 6.808990905

AL354993.2 0.189225962 0.11729775 5.696217004 2.64E-08 7.29E-08 6.806587541

AL355073.1 0.120938794 0.139269045 5.695418091 2.65E-08 7.32E-08 6.802419432

AC005746.3 0.089561162 0.081454598 5.694810073 2.66E-08 7.35E-08 6.799247595

AC010619.2 -0.086879351 0.136258442 -5.691660162 2.70E-08 7.47E-08 6.782819974

AL133330.2 -0.054217936 0.034616566 -5.689554122 2.73E-08 7.55E-08 6.771840611

LINC02371 0.086931777 0.100971916 5.688875761 2.74E-08 7.58E-08 6.768304845

MRVI1-AS1 -0.221287103 0.396385706 -5.682246054 2.84E-08 7.85E-08 6.733767739

1-Dec 0.052858034 0.042116874 5.681356683 2.86E-08 7.89E-08 6.729137143

LINC01726 0.025763953 0.015442492 5.677932728 2.91E-08 8.03E-08 6.711315595

AC018761.4 -0.244189055 0.118545261 -5.676708905 2.93E-08 8.08E-08 6.704947807

AL590226.1 0.284415764 0.767059166 5.675378921 2.95E-08 8.14E-08 6.698028929

AC069155.1 0.024847829 0.01368081 5.673650157 2.98E-08 8.21E-08 6.689037514

AC078942.1 0.131186581 0.159244499 5.671947405 3.00E-08 8.28E-08 6.680183604

AL138720.1 0.051872382 0.032066733 5.671327295 3.01E-08 8.31E-08 6.676959728

AC002310.2 -0.072012015 0.083134567 -5.671020837 3.02E-08 8.32E-08 6.675366598

AL136980.1 -0.165232977 0.338132163 -5.664993205 3.12E-08 8.59E-08 6.644046272

AL390860.1 -0.058524774 0.04234958 -5.664460664 3.13E-08 8.62E-08 6.641280448

Z82198.2 -0.073546077 0.035704047 -5.663024758 3.15E-08 8.68E-08 6.633823955
[truncated: 333,474 more chars]
