# Supplementary material for: Identification and Validation of Apparent Imbalanced Epi-lncRNAs Prognostic Model Based on Multi-Omics Data in Pancreatic Cancer
Source: Front Mol Biosci. 2022 May 12;9:860323. doi: 10.3389/fmolb.2022.860323 (PMC9133386; doi:10.3389/fmolb.2022.860323)
Supplement: Supplementary file 5 [file Table3.DOCX]

Supplementary Table3. 2237 epi-lncRNAs, 11855 non-epi-lncRNAs, 13518 epi-PCGs, and 6097 non-epi-PCGs identified by combining with the histone modification data and 450K methylation microarray data.

Genes Type

AL160408.2 epi.lncRNA

AP001267.1 epi.lncRNA

AL583722.2 epi.lncRNA

AL354740.1 epi.lncRNA

AC087292.1 epi.lncRNA

AC125611.3 epi.lncRNA

AL139317.3 epi.lncRNA

ZFHX2-AS1 epi.lncRNA

AC034102.4 epi.lncRNA

AC083798.2 epi.lncRNA

EDRF1-DT epi.lncRNA

AC008738.3 epi.lncRNA

LINC01106 epi.lncRNA

AL441883.1 epi.lncRNA

AP001318.2 epi.lncRNA

AC012615.3 epi.lncRNA

AP000873.2 epi.lncRNA

AC093673.1 epi.lncRNA

AL358781.1 epi.lncRNA

BX664727.3 epi.lncRNA

AL590999.1 epi.lncRNA

AC005562.1 epi.lncRNA

LINC00893 epi.lncRNA

AC074212.1 epi.lncRNA

ZSWIM8-AS1 epi.lncRNA

AC084018.2 epi.lncRNA

SNHG16 epi.lncRNA

AC105339.2 epi.lncRNA

AP006621.1 epi.lncRNA

AC017116.1 epi.lncRNA

AC022336.2 epi.lncRNA

AC135048.1 epi.lncRNA

BMS1P4 epi.lncRNA

AL513327.1 epi.lncRNA

AC016876.2 epi.lncRNA

OR2A1-AS1 epi.lncRNA

LINC00663 epi.lncRNA

AP001453.1 epi.lncRNA

AL162258.1 epi.lncRNA

BAIAP2-DT epi.lncRNA

EPB41L4A-AS1 epi.lncRNA

AC011603.2 epi.lncRNA

SPAG5-AS1 epi.lncRNA

AC025271.4 epi.lncRNA

AC021016.2 epi.lncRNA

AL591684.2 epi.lncRNA

AL354892.2 epi.lncRNA

LINC01152 epi.lncRNA

AL390195.1 epi.lncRNA

AC090114.2 epi.lncRNA

AL450306.1 epi.lncRNA

TGFB2-OT1 epi.lncRNA

PRDM16-DT epi.lncRNA

TMEM147-AS1 epi.lncRNA

AC147067.1 epi.lncRNA

AL355312.4 epi.lncRNA

ZBED3-AS1 epi.lncRNA

AC015802.1 epi.lncRNA

AC091057.2 epi.lncRNA

AL604028.1 epi.lncRNA

LINC01140 epi.lncRNA

SFTPD-AS1 epi.lncRNA

RNF139-AS1 epi.lncRNA

AL162274.1 epi.lncRNA

PRKCQ-AS1 epi.lncRNA

AC000120.1 epi.lncRNA

DTX2P1-UPK3BP1-PMS2P11 epi.lncRNA

AL589765.4 epi.lncRNA

BX284668.2 epi.lncRNA

AC016727.1 epi.lncRNA

AC002116.1 epi.lncRNA

AL391422.4 epi.lncRNA

NORAD epi.lncRNA

BMS1P4-AGAP5 epi.lncRNA

SNHG1 epi.lncRNA

AC090617.5 epi.lncRNA

AC087741.1 epi.lncRNA

AL731566.2 epi.lncRNA

DARS-AS1 epi.lncRNA

GLIDR epi.lncRNA

CD2BP2-DT epi.lncRNA

AC018904.1 epi.lncRNA

BX470102.1 epi.lncRNA

AC073349.1 epi.lncRNA

LINC02585 epi.lncRNA

AC136475.3 epi.lncRNA

MIR497HG epi.lncRNA

AC016738.1 epi.lncRNA

AC008556.1 epi.lncRNA

DANCR epi.lncRNA

AP001267.3 epi.lncRNA

AC135457.1 epi.lncRNA

ADIRF-AS1 epi.lncRNA

LINC01783 epi.lncRNA

LINC00857 epi.lncRNA

LPP-AS2 epi.lncRNA

AC240565.1 epi.lncRNA

MALAT1 epi.lncRNA

RPARP-AS1 epi.lncRNA

AC026471.4 epi.lncRNA

AL691459.1 epi.lncRNA

LINC01089 epi.lncRNA

RBM26-AS1 epi.lncRNA

AL096870.2 epi.lncRNA

SNHG21 epi.lncRNA

AC027644.3 epi.lncRNA

KDM7A-DT epi.lncRNA

HCG11 epi.lncRNA

AL031320.2 epi.lncRNA

AL161421.1 epi.lncRNA

LINC01560 epi.lncRNA

AP001107.2 epi.lncRNA

LINC00173 epi.lncRNA

AL009179.1 epi.lncRNA

AC010401.1 epi.lncRNA

AC104758.2 epi.lncRNA

C8orf49 epi.lncRNA

AC110048.2 epi.lncRNA

AL354733.3 epi.lncRNA

AC123768.3 epi.lncRNA

AC005253.1 epi.lncRNA

RGMB-AS1 epi.lncRNA

AC005381.1 epi.lncRNA

AP002748.4 epi.lncRNA

AL138756.1 epi.lncRNA

Z84484.1 epi.lncRNA

LINC00689 epi.lncRNA

AL049597.2 epi.lncRNA

AC004889.1 epi.lncRNA

AC078795.1 epi.lncRNA

SEPTIN4-AS1 epi.lncRNA

LINC02381 epi.lncRNA

SNHG9 epi.lncRNA

AC009065.1 epi.lncRNA

LINC01843 epi.lncRNA

ERVH48-1 epi.lncRNA

SNHG5 epi.lncRNA

AC106038.1 epi.lncRNA

ROCR epi.lncRNA

BX293535.1 epi.lncRNA

AC004951.4 epi.lncRNA

AC025580.1 epi.lncRNA

AC073569.2 epi.lncRNA

AC009065.5 epi.lncRNA

AC003965.2 epi.lncRNA

AC015813.1 epi.lncRNA

LINC00205 epi.lncRNA

AL161756.1 epi.lncRNA

SEPSECS-AS1 epi.lncRNA

CHMP1B-AS1 epi.lncRNA

AC008438.1 epi.lncRNA

C11orf72 epi.lncRNA

MIR1915HG epi.lncRNA

AL357140.2 epi.lncRNA

AL357033.4 epi.lncRNA

AL022323.1 epi.lncRNA

CHROMR epi.lncRNA

MATN1-AS1 epi.lncRNA

SNHG30 epi.lncRNA

NKILA epi.lncRNA

AC008736.1 epi.lncRNA

AC114291.1 epi.lncRNA

AC244090.1 epi.lncRNA

AC095057.3 epi.lncRNA

AC023157.3 epi.lncRNA

ERICD epi.lncRNA

AFAP1-AS1 epi.lncRNA

AC010615.2 epi.lncRNA

MORF4L2-AS1 epi.lncRNA

LINC00899 epi.lncRNA

AC104211.1 epi.lncRNA

AC040970.1 epi.lncRNA

AC005920.3 epi.lncRNA

MEG9 epi.lncRNA

AL357033.3 epi.lncRNA

AC010913.1 epi.lncRNA

SRP14-AS1 epi.lncRNA

AC004585.1 epi.lncRNA

GAPLINC epi.lncRNA

AC092287.1 epi.lncRNA

RRS1-AS1 epi.lncRNA

UBXN10-AS1 epi.lncRNA

AC015917.2 epi.lncRNA

AL138976.2 epi.lncRNA

AL592430.1 epi.lncRNA

HMGA2-AS1 epi.lncRNA

BNC2-AS1 epi.lncRNA

AC004707.1 epi.lncRNA

DLG5-AS1 epi.lncRNA

AC254633.1 epi.lncRNA

LINC00930 epi.lncRNA

KTN1-AS1 epi.lncRNA

AC090907.1 epi.lncRNA

AC004923.4 epi.lncRNA

AC020916.1 epi.lncRNA

LINC01269 epi.lncRNA

CAMTA1-DT epi.lncRNA

AC091544.2 epi.lncRNA

BCDIN3D-AS1 epi.lncRNA

AC245041.2 epi.lncRNA

LINC02204 epi.lncRNA

AC080013.4 epi.lncRNA

AC087645.2 epi.lncRNA

AC011445.1 epi.lncRNA

AC009831.1 epi.lncRNA

TRIM31-AS1 epi.lncRNA

MAP3K2-DT epi.lncRNA

AC022211.3 epi.lncRNA

OBSCN-AS1 epi.lncRNA

AC046143.1 epi.lncRNA

AL139288.1 epi.lncRNA

AL603756.1 epi.lncRNA

AC018521.5 epi.lncRNA

AC127459.1 epi.lncRNA

AC108488.1 epi.lncRNA

RUSC1-AS1 epi.lncRNA

TGFB2-AS1 epi.lncRNA

AC004816.1 epi.lncRNA

AL162231.4 epi.lncRNA

AC138028.1 epi.lncRNA

WEE2-AS1 epi.lncRNA

AD000671.3 epi.lncRNA

AP003096.1 epi.lncRNA

GHRLOS epi.lncRNA

AP001922.6 epi.lncRNA

AL662797.1 epi.lncRNA

AL603839.2 epi.lncRNA

AC051619.6 epi.lncRNA

MIR155HG epi.lncRNA

AC114488.1 epi.lncRNA

RNASEH1-AS1 epi.lncRNA

CFAP58-DT epi.lncRNA

AL157871.1 epi.lncRNA

AC025048.4 epi.lncRNA

FAM245A epi.lncRNA

AC079328.2 epi.lncRNA

GABPB1-AS1 epi.lncRNA

AC005332.4 epi.lncRNA

PITPNA-AS1 epi.lncRNA

AL022316.1 epi.lncRNA

XXYLT1-AS2 epi.lncRNA

FAM201A epi.lncRNA

AL450326.1 epi.lncRNA

LINC02773 epi.lncRNA

AC008610.1 epi.lncRNA

AC138207.2 epi.lncRNA

AP002761.4 epi.lncRNA

AL137026.1 epi.lncRNA

AC004263.1 epi.lncRNA

AL512598.1 epi.lncRNA

RASAL2-AS1 epi.lncRNA

LINC00346 epi.lncRNA

AC012313.1 epi.lncRNA

AC126763.1 epi.lncRNA

AC005229.4 epi.lncRNA

CRIM1-DT epi.lncRNA

AP003469.2 epi.lncRNA

AL049830.3 epi.lncRNA

AC009962.1 epi.lncRNA

AL583722.1 epi.lncRNA

AL450998.2 epi.lncRNA

AC087276.1 epi.lncRNA

AC099518.1 epi.lncRNA

RFPL3S epi.lncRNA

AC100835.2 epi.lncRNA

AC138356.1 epi.lncRNA

AC060766.7 epi.lncRNA

AC007383.3 epi.lncRNA

MINCR epi.lncRNA

BX640514.2 epi.lncRNA

AP003064.1 epi.lncRNA

AL136295.2 epi.lncRNA

AC005261.1 epi.lncRNA

OGFR-AS1 epi.lncRNA

AL353194.1 epi.lncRNA

LINC002481 epi.lncRNA

C1RL-AS1 epi.lncRNA

SBNO1-AS1 epi.lncRNA

AL441992.1 epi.lncRNA

COL18A1-AS1 epi.lncRNA

AL049836.1 epi.lncRNA

LINC01504 epi.lncRNA

AC022034.1 epi.lncRNA

AC098851.1 epi.lncRNA

AC027601.1 epi.lncRNA

LINC01857 epi.lncRNA

CD27-AS1 epi.lncRNA

AC097532.2 epi.lncRNA

AC124248.1 epi.lncRNA

AC087392.3 epi.lncRNA

Z82217.1 epi.lncRNA

AGAP2-AS1 epi.lncRNA

AC145207.6 epi.lncRNA

AC084117.1 epi.lncRNA

AC005070.3 epi.lncRNA

AC099811.3 epi.lncRNA

AC091544.4 epi.lncRNA

FAM111A-DT epi.lncRNA

AC004264.1 epi.lncRNA

AL391069.2 epi.lncRNA

ERVK9-11 epi.lncRNA

AC002116.2 epi.lncRNA

AC025043.1 epi.lncRNA

UCKL1-AS1 epi.lncRNA

LINC00941 epi.lncRNA

SLCO4A1-AS1 epi.lncRNA

LINC01637 epi.lncRNA

FAM41C epi.lncRNA

AC093484.4 epi.lncRNA

AC100771.2 epi.lncRNA

LIMS1-AS1 epi.lncRNA

AL139274.2 epi.lncRNA

MAN1B1-DT epi.lncRNA

AL162724.2 epi.lncRNA

AP001029.2 epi.lncRNA

AC025423.4 epi.lncRNA

ZNF503-AS2 epi.lncRNA

AL021392.1 epi.lncRNA

AC048344.4 epi.lncRNA

LINC02818 epi.lncRNA

AL133338.1 epi.lncRNA

AL360219.1 epi.lncRNA

HMGA1P4 epi.lncRNA

LINC02323 epi.lncRNA

LINC00460 epi.lncRNA

AC002401.4 epi.lncRNA

AC132872.2 epi.lncRNA

CCDC84-DT epi.lncRNA

AC012073.1 epi.lncRNA

AC025048.2 epi.lncRNA

ZNF236-DT epi.lncRNA

AC145098.1 epi.lncRNA

AC007383.2 epi.lncRNA

STPG3-AS1 epi.lncRNA

LINC00886 epi.lncRNA

PICART1 epi.lncRNA

ZNRF3-AS1 epi.lncRNA

AL008729.1 epi.lncRNA

AC108058.1 epi.lncRNA

AC012186.2 epi.lncRNA

AC018647.2 epi.lncRNA

AC005225.2 epi.lncRNA

TMEM99 epi.lncRNA

EBLN3P epi.lncRNA

AC068580.2 epi.lncRNA

AC073575.2 epi.lncRNA

RPS6KA2-IT1 epi.lncRNA

AP001363.1 epi.lncRNA

ERICH6-AS1 epi.lncRNA

WAKMAR2 epi.lncRNA

AC019254.1 epi.lncRNA

AC007743.1 epi.lncRNA

MAFG-DT epi.lncRNA

AC007066.2 epi.lncRNA

AC008440.1 epi.lncRNA

AC008543.5 epi.lncRNA

ACBD3-AS1 epi.lncRNA

AC005041.3 epi.lncRNA

PCCA-DT epi.lncRNA

AC093726.1 epi.lncRNA

AL354919.2 epi.lncRNA

NSMCE1-DT epi.lncRNA

OCIAD1-AS1 epi.lncRNA

AC009133.4 epi.lncRNA

AC067852.2 epi.lncRNA

AC067930.4 epi.lncRNA

AC068234.2 epi.lncRNA

LINC00641 epi.lncRNA

AL357054.4 epi.lncRNA

AC099518.6 epi.lncRNA

AP002954.1 epi.lncRNA

ZNF30-AS1 epi.lncRNA

AC006435.1 epi.lncRNA

AC022075.1 epi.lncRNA

AC097534.2 epi.lncRNA

AC016590.3 epi.lncRNA

AC034102.6 epi.lncRNA

AC023510.2 epi.lncRNA

AL109936.2 epi.lncRNA

RTCA-AS1 epi.lncRNA

AC004854.2 epi.lncRNA

AC025682.1 epi.lncRNA

AC025171.4 epi.lncRNA

AL352984.1 epi.lncRNA

DUBR epi.lncRNA

AL359317.1 epi.lncRNA

LINC01750 epi.lncRNA

AL049840.5 epi.lncRNA

FOXCUT epi.lncRNA

AP002498.1 epi.lncRNA

AC021078.1 epi.lncRNA

ARF4-AS1 epi.lncRNA

AC006111.2 epi.lncRNA

ETV5-AS1 epi.lncRNA

DIO3OS epi.lncRNA

AC105339.3 epi.lncRNA

AC092756.1 epi.lncRNA

AL139339.1 epi.lncRNA

AC112721.2 epi.lncRNA

LIX1L-AS1 epi.lncRNA

HOXB-AS2 epi.lncRNA

AC104653.1 epi.lncRNA

AC108047.1 epi.lncRNA

AC087672.2 epi.lncRNA

AC136475.1 epi.lncRNA

AP005019.1 epi.lncRNA

FO393418.1 epi.lncRNA

AC022211.1 epi.lncRNA

OSGEPL1-AS1 epi.lncRNA

LINC01394 epi.lncRNA

AL356356.1 epi.lncRNA

AL162258.2 epi.lncRNA

AC027309.1 epi.lncRNA

SNHG19 epi.lncRNA

AC012615.4 epi.lncRNA

AL513165.1 epi.lncRNA

AC079907.1 epi.lncRNA

SMC2-AS1 epi.lncRNA

LINC01547 epi.lncRNA

AC117394.2 epi.lncRNA

PRC1-AS1 epi.lncRNA

RHPN1-AS1 epi.lncRNA

AL162424.1 epi.lncRNA

AL590440.1 epi.lncRNA

AC009005.1 epi.lncRNA

LINC02289 epi.lncRNA

AC009090.3 epi.lncRNA

AL133215.2 epi.lncRNA

LINC02004 epi.lncRNA

AC092115.3 epi.lncRNA

LINC01786 epi.lncRNA

DNAJC9-AS1 epi.lncRNA

AL355803.1 epi.lncRNA

POLH-AS1 epi.lncRNA

AL355388.1 epi.lncRNA

TTC39C-AS1 epi.lncRNA

AC011472.1 epi.lncRNA

PSMB8-AS1 epi.lncRNA

AL109947.1 epi.lncRNA

URB1-AS1 epi.lncRNA

PACRG-AS1 epi.lncRNA

AC006333.2 epi.lncRNA

AL138921.1 epi.lncRNA

AC010327.6 epi.lncRNA

AC116407.2 epi.lncRNA

AC120498.9 epi.lncRNA

AL132780.1 epi.lncRNA

AL592494.2 epi.lncRNA

AC083805.3 epi.lncRNA

AC023794.2 epi.lncRNA

AL157938.3 epi.lncRNA

AC107214.1 epi.lncRNA

AC017002.3 epi.lncRNA

AC110285.2 epi.lncRNA

AC007613.1 epi.lncRNA

AP000320.1 epi.lncRNA

AC012557.2 epi.lncRNA

KANSL1-AS1 epi.lncRNA

AC007773.1 epi.lncRNA

AC012236.1 epi.lncRNA

AC097534.1 epi.lncRNA

MIR3142HG epi.lncRNA

AC131009.1 epi.lncRNA

AC096772.1 epi.lncRNA

AC026355.2 epi.lncRNA

AP000866.5 epi.lncRNA

LINC01775 epi.lncRNA

AC005841.1 epi.lncRNA

AC118344.1 epi.lncRNA

AC112220.2 epi.lncRNA

AL513365.2 epi.lncRNA

SPATA41 epi.lncRNA

AL603910.1 epi.lncRNA

AL928654.1 epi.lncRNA

AC022613.2 epi.lncRNA

AP001372.2 epi.lncRNA

AL162311.3 epi.lncRNA

AC107959.3 epi.lncRNA

AL050341.2 epi.lncRNA

AC132938.1 epi.lncRNA

AC009171.2 epi.lncRNA

AC012317.1 epi.lncRNA

AP005264.1 epi.lncRNA

LINC00525 epi.lncRNA

AC092295.2 epi.lncRNA

AC100861.1 epi.lncRNA

AL391807.1 epi.lncRNA

AC087500.1 epi.lncRNA

AC004846.1 epi.lncRNA

WWTR1-AS1 epi.lncRNA

AC006270.1 epi.lncRNA

AL121917.1 epi.lncRNA

AC109347.1 epi.lncRNA

AC013565.1 epi.lncRNA

AP001816.1 epi.lncRNA

AC092747.4 epi.lncRNA

AC073370.1 epi.lncRNA

AC010834.1 epi.lncRNA

LINC02454 epi.lncRNA

GCC2-AS1 epi.lncRNA

N4BP2L2-IT2 epi.lncRNA

AC008555.4 epi.lncRNA

AP000569.1 epi.lncRNA

LINC00664 epi.lncRNA

EZR-AS1 epi.lncRNA

AC018529.1 epi.lncRNA

SHANK2-AS1 epi.lncRNA

AC123768.2 epi.lncRNA

AC093249.2 epi.lncRNA

AL353807.2 epi.lncRNA

AC013553.3 epi.lncRNA

GASAL1 epi.lncRNA

AC100861.2 epi.lncRNA

AF129408.1 epi.lncRNA

AL133370.1 epi.lncRNA

AC016394.2 epi.lncRNA

AC233992.1 epi.lncRNA

AL356489.1 epi.lncRNA

USP46-AS1 epi.lncRNA

AC089983.1 epi.lncRNA

AL118558.3 epi.lncRNA

AP005131.2 epi.lncRNA

ILF3-DT epi.lncRNA

AP002761.3 epi.lncRNA

LINC01963 epi.lncRNA

AL445248.1 epi.lncRNA

SNHG15 epi.lncRNA

AL136309.2 epi.lncRNA

AL162171.3 epi.lncRNA

AC022154.1 epi.lncRNA

AC010761.6 epi.lncRNA

AC017100.1 epi.lncRNA

AL078581.4 epi.lncRNA

AC010680.1 epi.lncRNA

AC087491.1 epi.lncRNA

AC113143.1 epi.lncRNA

AC114956.1 epi.lncRNA

AL031848.2 epi.lncRNA

LINC00242 epi.lncRNA

AC004975.2 epi.lncRNA

AC108134.2 epi.lncRNA

AL512408.1 epi.lncRNA

AC010504.1 epi.lncRNA

AC006252.1 epi.lncRNA

AC100830.3 epi.lncRNA

AC021683.2 epi.lncRNA

ZRANB2-AS1 epi.lncRNA

PACERR epi.lncRNA

LINC02593 epi.lncRNA

AL109976.1 epi.lncRNA

AC122129.1 epi.lncRNA

AL137186.1 epi.lncRNA

LINC00638 epi.lncRNA

AC018521.7 epi.lncRNA

LINC01016 epi.lncRNA

AC069360.1 epi.lncRNA

AC006033.2 epi.lncRNA

KF459542.1 epi.lncRNA

HOMER3-AS1 epi.lncRNA

AC027237.2 epi.lncRNA

SNHG6 epi.lncRNA

AC012636.1 epi.lncRNA

AL160314.2 epi.lncRNA

MIR3945HG epi.lncRNA

AL731533.2 epi.lncRNA

AC092903.2 epi.lncRNA

HCG27 epi.lncRNA

GHET1 epi.lncRNA

AC093732.1 epi.lncRNA

LINC02019 epi.lncRNA

LINC02601 epi.lncRNA

AC092809.2 epi.lncRNA

AC004771.5 epi.lncRNA

AC008393.1 epi.lncRNA

AC007036.3 epi.lncRNA

AC009041.4 epi.lncRNA

AC018755.4 epi.lncRNA

ADAMTSL4-AS1 epi.lncRNA

AL136141.1 epi.lncRNA

LINC01910 epi.lncRNA

AL590133.2 epi.lncRNA

AC124276.2 epi.lncRNA

AC073254.1 epi.lncRNA

SNHG10 epi.lncRNA

AC123788.1 epi.lncRNA

AL445228.2 epi.lncRNA

AP001351.1 epi.lncRNA

AC092159.2 epi.lncRNA

NDUFB2-AS1 epi.lncRNA

AL513320.1 epi.lncRNA

AC064836.2 epi.lncRNA

LINC02768 epi.lncRNA

LINC01556 epi.lncRNA

AL358472.2 epi.lncRNA

AC020658.5 epi.lncRNA

AC011247.1 epi.lncRNA

AC034199.1 epi.lncRNA

AC005046.1 epi.lncRNA

AC018362.1 epi.lncRNA

LINC02560 epi.lncRNA

AL122023.1 epi.lncRNA

ELFN1-AS1 epi.lncRNA

AL591623.1 epi.lncRNA

SERPINB9P1 epi.lncRNA

AC083806.2 epi.lncRNA

H1FX-AS1 epi.lncRNA

AC009961.1 epi.lncRNA

AC004906.1 epi.lncRNA

AL137779.2 epi.lncRNA

AC005180.2 epi.lncRNA

AC008074.2 epi.lncRNA

AC010203.2 epi.lncRNA

AL035071.2 epi.lncRNA

AC087164.1 epi.lncRNA

AL359921.1 epi.lncRNA

LINC00847 epi.lncRNA

Z83843.1 epi.lncRNA

AP003071.4 epi.lncRNA

AP003774.2 epi.lncRNA

AL353803.5 epi.lncRNA

AC096751.2 epi.lncRNA

AC078889.1 epi.lncRNA

AC097639.1 epi.lncRNA

C17orf82 epi.lncRNA

AC068870.2 epi.lncRNA

LOXL1-AS1 epi.lncRNA

AC026803.1 epi.lncRNA

LINC00337 epi.lncRNA

AC006273.1 epi.lncRNA

AP003059.2 epi.lncRNA

AC090061.1 epi.lncRNA

LINC02450 epi.lncRNA

AC120498.1 epi.lncRNA

LINC02356 epi.lncRNA

AC011510.1 epi.lncRNA

AC112715.1 epi.lncRNA

RUNDC3A-AS1 epi.lncRNA

AC008494.3 epi.lncRNA

AC073517.1 epi.lncRNA

AC012467.2 epi.lncRNA

AC011611.4 epi.lncRNA

AL078644.2 epi.lncRNA

AP006284.1 epi.lncRNA

AC091564.3 epi.lncRNA

AL360181.1 epi.lncRNA

FAM83C-AS1 epi.lncRNA

AC120053.1 epi.lncRNA

AC106820.2 epi.lncRNA

RMDN2-AS1 epi.lncRNA

AC090587.2 epi.lncRNA

AC139100.1 epi.lncRNA

AC087301.1 epi.lncRNA

AC109322.1 epi.lncRNA

PPP4R1-AS1 epi.lncRNA

AL133375.1 epi.lncRNA

KIZ-AS1 epi.lncRNA

LINC01943 epi.lncRNA

HIF1A-AS1 epi.lncRNA

AC008443.6 epi.lncRNA

AL031666.3 epi.lncRNA

TMPO-AS1 epi.lncRNA

AC051619.4 epi.lncRNA

AC121338.1 epi.lncRNA

AL162724.1 epi.lncRNA

AL033381.2 epi.lncRNA

AC093520.1 epi.lncRNA

AC008735.1 epi.lncRNA

AL359504.2 epi.lncRNA

AC005740.3 epi.lncRNA

AC007569.1 epi.lncRNA

LINC01827 epi.lncRNA

RNASEH2B-AS1 epi.lncRNA

AC010175.1 epi.lncRNA

AL450344.3 epi.lncRNA

HCG21 epi.lncRNA

AC018845.3 epi.lncRNA

WWC2-AS2 epi.lncRNA

AL645608.7 epi.lncRNA

SBF2-AS1 epi.lncRNA

AC136475.8 epi.lncRNA

AC011933.3 epi.lncRNA

AC134682.1 epi.lncRNA

AL591178.1 epi.lncRNA

AL513550.1 epi.lncRNA

CARS-AS1 epi.lncRNA

AC008649.2 epi.lncRNA

AC012615.2 epi.lncRNA

AP005899.1 epi.lncRNA

AC010487.2 epi.lncRNA

AC025031.4 epi.lncRNA

AL358075.2 epi.lncRNA

AL603839.3 epi.lncRNA

AC145207.8 epi.lncRNA

AC010280.2 epi.lncRNA

AP001062.2 epi.lncRNA

AL596247.1 epi.lncRNA

SYNGAP1-AS1 epi.lncRNA

LINC01132 epi.lncRNA

AC015909.1 epi.lncRNA

AL359878.2 epi.lncRNA

RNF216-IT1 epi.lncRNA

AP000866.1 epi.lncRNA

AC013264.1 epi.lncRNA

AC092127.1 epi.lncRNA

AC012588.1 epi.lncRNA

AP001033.1 epi.lncRNA

ITGB5-AS1 epi.lncRNA

AC100793.2 epi.lncRNA

AC080188.1 epi.lncRNA

AC009159.3 epi.lncRNA

LINC00243 epi.lncRNA

AL139352.1 epi.lncRNA

AC022092.1 epi.lncRNA

AC073046.1 epi.lncRNA

CEBPB-AS1 epi.lncRNA

AC068338.2 epi.lncRNA

AC004803.1 epi.lncRNA

TRPC7-AS1 epi.lncRNA

AL627171.1 epi.lncRNA

AC007952.4 epi.lncRNA

AC004832.5 epi.lncRNA

AC090164.2 epi.lncRNA

AC025171.2 epi.lncRNA

AC005921.2 epi.lncRNA

STEAP3-AS1 epi.lncRNA

AC243960.3 epi.lncRNA

AC012313.7 epi.lncRNA

ATP2B1-AS1 epi.lncRNA

LINC02453 epi.lncRNA

DOCK9-DT epi.lncRNA

AC011446.2 epi.lncRNA

AC023051.1 epi.lncRNA

AC078962.1 epi.lncRNA

AC083906.3 epi.lncRNA

AL158151.4 epi.lncRNA

PRICKLE2-DT epi.lncRNA

AC006064.3 epi.lncRNA

AL121987.1 epi.lncRNA

AC090559.1 epi.lncRNA

TMEM44-AS1 epi.lncRNA

AC093734.1 epi.lncRNA

AC020763.1 epi.lncRNA

SLC25A34-AS1 epi.lncRNA

PAXBP1-AS1 epi.lncRNA

MELTF-AS1 epi.lncRNA

AC244153.1 epi.lncRNA

AP001922.5 epi.lncRNA

LINC02054 epi.lncRNA

AC007639.1 epi.lncRNA

DNM3OS epi.lncRNA

AC006213.5 epi.lncRNA

AP005131.1 epi.lncRNA

LINC01215 epi.lncRNA

XXYLT1-AS1 epi.lncRNA

AC112484.1 epi.lncRNA

AC008937.1 epi.lncRNA

AC093904.3 epi.lncRNA

AL391121.1 epi.lncRNA

AC010997.4 epi.lncRNA

AL021408.1 epi.lncRNA

AC012462.3 epi.lncRNA

AL022157.1 epi.lncRNA

AC079467.1 epi.lncRNA

AC092868.2 epi.lncRNA

AC097716.1 epi.lncRNA

AC007998.3 epi.lncRNA

LENG8-AS1 epi.lncRNA

AC090971.2 epi.lncRNA

LINC00294 epi.lncRNA

AC129510.1 epi.lncRNA

LINC02642 epi.lncRNA

AL121985.1 epi.lncRNA

GRK5-IT1 epi.lncRNA

HM13-AS1 epi.lncRNA

AC243960.1 epi.lncRNA

AC061992.2 epi.lncRNA

AC079354.2 epi.lncRNA

AC090409.1 epi.lncRNA

LINC02387 epi.lncRNA

LINC01730 epi.lncRNA

AL161431.1 epi.lncRNA

AC103760.1 epi.lncRNA

AC144652.1 epi.lncRNA

AL118522.1 epi.lncRNA

AC073283.1 epi.lncRNA

AC104984.2 epi.lncRNA

C7orf69 epi.lncRNA

AL049869.3 epi.lncRNA

AC008403.2 epi.lncRNA

AC114488.2 epi.lncRNA

AC046168.2 epi.lncRNA

AC096586.2 epi.lncRNA

AL161729.3 epi.lncRNA

AC092117.1 epi.lncRNA

AC108134.4 epi.lncRNA

AL121574.1 epi.lncRNA

AC096540.1 epi.lncRNA

SNHG25 epi.lncRNA

AC010735.2 epi.lncRNA

IDI2-AS1 epi.lncRNA

AC009779.2 epi.lncRNA

AP000919.3 epi.lncRNA

AL450322.2 epi.lncRNA

AC005759.1 epi.lncRNA

AP000786.1 epi.lncRNA

LINC00484 epi.lncRNA

AC002310.1 epi.lncRNA

MAPK6-DT epi.lncRNA

AC234772.2 epi.lncRNA

AL136084.3 epi.lncRNA

LINC00528 epi.lncRNA

AC104170.1 epi.lncRNA

AC138466.1 epi.lncRNA

LINC01629 epi.lncRNA

AC000068.1 epi.lncRNA

AP000943.1 epi.lncRNA

LINC01063 epi.lncRNA

AC011825.4 epi.lncRNA

AL355312.3 epi.lncRNA

AC018690.1 epi.lncRNA

AC017074.1 epi.lncRNA

AC108718.1 epi.lncRNA

AL353693.1 epi.lncRNA

LINC01970 epi.lncRNA

SMAD5-AS1 epi.lncRNA

SNHG11 epi.lncRNA

AC087521.2 epi.lncRNA

AC010422.1 epi.lncRNA

AC096921.2 epi.lncRNA

AL354824.1 epi.lncRNA

AP001001.1 epi.lncRNA

AL356215.1 epi.lncRNA

CD44-AS1 epi.lncRNA

BOLA3-AS1 epi.lncRNA

AP006623.1 epi.lncRNA

RAET1E-AS1 epi.lncRNA

TDRKH-AS1 epi.lncRNA

ZMYM4-AS1 epi.lncRNA

AC136475.5 epi.lncRNA

AL844908.2 epi.lncRNA

ZBTB40-IT1 epi.lncRNA

AF015262.1 epi.lncRNA

AC027808.1 epi.lncRNA

AC103724.4 epi.lncRNA

AC002306.1 epi.lncRNA

DLGAP1-AS2 epi.lncRNA

AC114489.1 epi.lncRNA

AC008870.3 epi.lncRNA

AL133406.2 epi.lncRNA

AC145285.3 epi.lncRNA

AC021087.1 epi.lncRNA

OPA1-AS1 epi.lncRNA

PPP1R12A-AS1 epi.lncRNA

AL645608.6 epi.lncRNA

HLA-F-AS1 epi.lncRNA

AL022313.2 epi.lncRNA

AC087392.1 epi.lncRNA

AC137834.2 epi.lncRNA

AC008443.4 epi.lncRNA

AC008957.1 epi.lncRNA

SMCR5 epi.lncRNA

AC008147.1 epi.lncRNA

AC068594.1 epi.lncRNA

AC073641.1 epi.lncRNA

LINC00582 epi.lncRNA

AC102953.2 epi.lncRNA

AC012555.1 epi.lncRNA

AC104109.2 epi.lncRNA

AC005520.5 epi.lncRNA

TNFRSF14-AS1 epi.lncRNA

AC044781.1 epi.lncRNA

AL049629.1 epi.lncRNA

LINC00677 epi.lncRNA

AL672291.1 epi.lncRNA

AL117350.1 epi.lncRNA

LIF-AS1 epi.lncRNA

ZEB2-AS1 epi.lncRNA

AC135048.3 epi.lncRNA

AC018413.1 epi.lncRNA

AC007314.1 epi.lncRNA

AL031058.1 epi.lncRNA

AP000941.1 epi.lncRNA

AL157838.1 epi.lncRNA

LIVAR epi.lncRNA

LINC02701 epi.lncRNA

MAGI2-AS3 epi.lncRNA

AC011595.2 epi.lncRNA

LINC01731 epi.lncRNA

AC040934.1 epi.lncRNA

AC026355.3 epi.lncRNA

AC099522.2 epi.lncRNA

AC092112.1 epi.lncRNA

AFDN-DT epi.lncRNA

AL606760.1 epi.lncRNA

AC005746.1 epi.lncRNA

AC124276.1 epi.lncRNA

AC005281.1 epi.lncRNA

AP003068.1 epi.lncRNA

AL139339.2 epi.lncRNA

AL583722.3 epi.lncRNA

LINC01771 epi.lncRNA

AC010198.1 epi.lncRNA

AL121790.2 epi.lncRNA

AC069234.3 epi.lncRNA

AC022167.1 epi.lncRNA

AC087294.1 epi.lncRNA

AL391097.2 epi.lncRNA

AC103706.1 epi.lncRNA

AL590714.1 epi.lncRNA

AGAP1-IT1 epi.lncRNA

MAST4-AS1 epi.lncRNA

LINC01315 epi.lncRNA

AC008115.2 epi.lncRNA

AATBC epi.lncRNA

AP000704.1 epi.lncRNA

HCG15 epi.lncRNA

AC025430.1 epi.lncRNA

AP000820.1 epi.lncRNA

LINC02205 epi.lncRNA

AL359513.1 epi.lncRNA

AL590428.1 epi.lncRNA

SREBF2-AS1 epi.lncRNA

AP001783.1 epi.lncRNA

KDM2B-DT epi.lncRNA

AC006001.2 epi.lncRNA

AC019294.2 epi.lncRNA

AC008443.2 epi.lncRNA

LINC02690 epi.lncRNA

AC004471.1 epi.lncRNA

AC027801.3 epi.lncRNA

AL691432.2 epi.lncRNA

AC080162.1 epi.lncRNA

AL049569.1 epi.lncRNA

LINC02783 epi.lncRNA

AC012676.1 epi.lncRNA

AL034345.2 epi.lncRNA

AP002449.1 epi.lncRNA

MKLN1-AS epi.lncRNA

AC079922.2 epi.lncRNA

LMO7DN epi.lncRNA

AC114803.1 epi.lncRNA

LINC02761 epi.lncRNA

AC022973.3 epi.lncRNA

ARNTL2-AS1 epi.lncRNA

LINC01546 epi.lncRNA

AC005670.1 epi.lncRNA

MIF-AS1 epi.lncRNA

AC011944.1 epi.lncRNA

AC104564.1 epi.lncRNA

SYNE1-AS1 epi.lncRNA

AC107308.1 epi.lncRNA

AC009806.1 epi.lncRNA

AC093567.1 epi.lncRNA

AL157834.1 epi.lncRNA

AC006064.5 epi.lncRNA

ATXN2-AS epi.lncRNA

AL031847.1 epi.lncRNA

AL512353.1 epi.lncRNA

AC010761.3 epi.lncRNA

AL451042.2 epi.lncRNA

SNHG3 epi.lncRNA

AC009133.5 epi.lncRNA

LINC01770 epi.lncRNA

HAR1A epi.lncRNA

LINC00653 epi.lncRNA

AL359853.1 epi.lncRNA

EXOC3-AS1 epi.lncRNA

AP003696.1 epi.lncRNA

AL139260.1 epi.lncRNA

U73169.1 epi.lncRNA

LINC01714 epi.lncRNA

AC092431.1 epi.lncRNA

LINC01926 epi.lncRNA

AC122134.1 epi.lncRNA

AC074033.1 epi.lncRNA

AL031717.1 epi.lncRNA

LINC02310 epi.lncRNA

AC104532.2 epi.lncRNA

AL359979.1 epi.lncRNA

AL355388.2 epi.lncRNA

AC092757.2 epi.lncRNA

AC116366.1 epi.lncRNA

AL133467.1 epi.lncRNA

AC018695.6 epi.lncRNA

MESTIT1 epi.lncRNA

IER3-AS1 epi.lncRNA

AC012038.2 epi.lncRNA

AC004870.2 epi.lncRNA

LINC00111 epi.lncRNA

AP000851.1 epi.lncRNA

AC011921.1 epi.lncRNA

AL022344.1 epi.lncRNA

AC092803.1 epi.lncRNA

AL136115.1 epi.lncRNA

ARHGAP26-AS1 epi.lncRNA

LINC02255 epi.lncRNA

AC020891.3 epi.lncRNA

LINC02725 epi.lncRNA

P3H2-AS1 epi.lncRNA

USP12-AS2 epi.lncRNA

AC019186.1 epi.lncRNA

AL158801.2 epi.lncRNA

TRAM2-AS1 epi.lncRNA

AL132655.1 epi.lncRNA

AL157756.1 epi.lncRNA

AL096678.1 epi.lncRNA

AC020922.4 epi.lncRNA

AC009121.3 epi.lncRNA

AC010997.5 epi.lncRNA

AL031727.2 epi.lncRNA

AL033384.1 epi.lncRNA

AC130650.1 epi.lncRNA

AC103681.1 epi.lncRNA

AC009269.5 epi.lncRNA

AC013480.1 epi.lncRNA

AC023300.2 epi.lncRNA

AL031666.2 epi.lncRNA

AC020658.4 epi.lncRNA

AC009902.2 epi.lncRNA

LINC02700 epi.lncRNA

AC117382.2 epi.lncRNA

AC004012.1 epi.lncRNA

KCNMA1-AS3 epi.lncRNA

AL354726.1 epi.lncRNA

LINC02574 epi.lncRNA

LINC01693 epi.lncRNA

LINC00942 epi.lncRNA

AL133343.2 epi.lncRNA

AL161645.1 epi.lncRNA

AC092919.1 epi.lncRNA

ASH1L-AS1 epi.lncRNA

AL121987.2 epi.lncRNA

LINC01124 epi.lncRNA

STK4-AS1 epi.lncRNA

LINC02521 epi.lncRNA

AC008543.3 epi.lncRNA

AC018766.1 epi.lncRNA

AC004825.2 epi.lncRNA

AL158829.1 epi.lncRNA

AP002852.1 epi.lncRNA

AC008982.2 epi.lncRNA

AL662844.3 epi.lncRNA

AL035250.1 epi.lncRNA

AC093001.1 epi.lncRNA

AC079336.2 epi.lncRNA

AC020915.1 epi.lncRNA

AC137932.2 epi.lncRNA

AP000879.2 epi.lncRNA

AL807757.2 epi.lncRNA

AP002478.1 epi.lncRNA

AC245036.5 epi.lncRNA

AL662890.1 epi.lncRNA

OTUD6B-AS1 epi.lncRNA

AC005532.1 epi.lncRNA

AL449106.1 epi.lncRNA

AL117378.1 epi.lncRNA

AL672032.1 epi.lncRNA

AP000845.1 epi.lncRNA

AL034374.1 epi.lncRNA

LINC01521 epi.lncRNA

AC117465.1 epi.lncRNA

AC011466.1 epi.lncRNA

AC126175.2 epi.lncRNA

AP001160.1 epi.lncRNA

LINC01165 epi.lncRNA

AL450322.1 epi.lncRNA

AC139530.1 epi.lncRNA

AC130650.2 epi.lncRNA

AP000894.4 epi.lncRNA

AL391839.2 epi.lncRNA

AL109614.1 epi.lncRNA

AL049795.1 epi.lncRNA

AL359555.1 epi.lncRNA

AC105429.1 epi.lncRNA

CCDC183-AS1 epi.lncRNA

AC008752.1 epi.lncRNA

AC007032.1 epi.lncRNA

AC008734.2 epi.lncRNA

AC104779.1 epi.lncRNA

AC104667.1 epi.lncRNA

AL137003.2 epi.lncRNA

LINC01412 epi.lncRNA

AL590438.1 epi.lncRNA

PARD3-AS1 epi.lncRNA

AL512656.1 epi.lncRNA

AL138902.1 epi.lncRNA

AC108676.1 epi.lncRNA

AL354824.2 epi.lncRNA

AC111182.1 epi.lncRNA

AL451042.1 epi.lncRNA

RBAKDN epi.lncRNA

AC026310.2 epi.lncRNA

AC016571.1 epi.lncRNA

AL355490.2 epi.lncRNA

AC092535.5 epi.lncRNA

AC129492.4 epi.lncRNA

AC145207.4 epi.lncRNA

AC008115.4 epi.lncRNA

AL139039.3 epi.lncRNA

AC131097.2 epi.lncRNA

AC104809.1 epi.lncRNA

C2-AS1 epi.lncRNA

Z99943.1 epi.lncRNA

AL445490.1 epi.lncRNA

DNM1P35 epi.lncRNA

AL358933.1 epi.lncRNA

AC012531.1 epi.lncRNA

DLGAP1-AS1 epi.lncRNA

AL356310.1 epi.lncRNA

LINC00974 epi.lncRNA

AC005614.1 epi.lncRNA

SEC62-AS1 epi.lncRNA

AC015982.2 epi.lncRNA

AC083805.1 epi.lncRNA

AL118511.1 epi.lncRNA

AC211476.2 epi.lncRNA

LINC01431 epi.lncRNA

LINC02539 epi.lncRNA

FMR1-AS1 epi.lncRNA

AC005884.1 epi.lncRNA

AC027228.1 epi.lncRNA

RSF1-IT1 epi.lncRNA

AL354928.1 epi.lncRNA

AC080013.3 epi.lncRNA

LINC02575 epi.lncRNA

AC002467.1 epi.lncRNA

AP001610.3 epi.lncRNA

AC083843.2 epi.lncRNA

AC034231.1 epi.lncRNA

AF111167.2 epi.lncRNA

AC069294.1 epi.lncRNA

U47924.2 epi.lncRNA

AC016292.1 epi.lncRNA

AC136469.1 epi.lncRNA

AC016229.2 epi.lncRNA

LINC00866 epi.lncRNA

C10orf91 epi.lncRNA

AC022167.4 epi.lncRNA

AC139722.1 epi.lncRNA

AC092807.1 epi.lncRNA

AC011773.3 epi.lncRNA

HCFC1-AS1 epi.lncRNA

AC104117.5 epi.lncRNA

AC005722.3 epi.lncRNA

ZFX-AS1 epi.lncRNA

AC079336.1 epi.lncRNA

AC106882.1 epi.lncRNA

AC211433.2 epi.lncRNA

KCNAB1-AS1 epi.lncRNA

WASIR2 epi.lncRNA

AGBL5-AS1 epi.lncRNA

AC104971.1 epi.lncRNA

AC136604.3 epi.lncRNA

AL139300.2 epi.lncRNA

STARD7-AS1 epi.lncRNA

AP006219.1 epi.lncRNA

INO80-AS1 epi.lncRNA

AC131097.3 epi.lncRNA

Z73429.1 epi.lncRNA

AL356124.1 epi.lncRNA

AP006287.2 epi.lncRNA

AP001160.3 epi.lncRNA

AC022028.2 epi.lncRNA

TM4SF1-AS1 epi.lncRNA

AC013731.1 epi.lncRNA

AC022872.1 epi.lncRNA

AC130456.4 epi.lncRNA

AC009133.1 epi.lncRNA

AC055822.1 epi.lncRNA

AC007406.3 epi.lncRNA

SOCS2-AS1 epi.lncRNA

CCDC148-AS1 epi.lncRNA

MIR302CHG epi.lncRNA

AC015712.4 epi.lncRNA

Z92544.1 epi.lncRNA

AC090921.1 epi.lncRNA

LINC02280 epi.lncRNA

CYP4F26P epi.lncRNA

AC011503.2 epi.lncRNA

AC090192.2 epi.lncRNA

FLNC-AS1 epi.lncRNA

AC100830.1 epi.lncRNA

AL121772.3 epi.lncRNA

LINC00336 epi.lncRNA

AC009686.1 epi.lncRNA

AL358394.1 epi.lncRNA

EGLN3-AS1 epi.lncRNA

AC105053.1 epi.lncRNA

LINC02308 epi.lncRNA

AL596202.1 epi.lncRNA

AC008551.1 epi.lncRNA

AL121721.1 epi.lncRNA

AC091053.2 epi.lncRNA

AC090673.1 epi.lncRNA

AL606760.3 epi.lncRNA

AL121772.1 epi.lncRNA

AC005674.1 epi.lncRNA

AC007389.5 epi.lncRNA

LINC02636 epi.lncRNA

AC114781.2 epi.lncRNA

AC123595.1 epi.lncRNA

AC117490.2 epi.lncRNA

AL645941.3 epi.lncRNA

AL020998.1 epi.lncRNA

AC091544.7 epi.lncRNA

AC073352.2 epi.lncRNA

HMGN3-AS1 epi.lncRNA

AL451164.1 epi.lncRNA

KIAA0087 epi.lncRNA

AL139089.1 epi.lncRNA

LINC02385 epi.lncRNA

AL078459.1 epi.lncRNA

Z92544.2 epi.lncRNA

AC025262.1 epi.lncRNA

LINC02284 epi.lncRNA

AC091117.2 epi.lncRNA

AC004156.1 epi.lncRNA

AC025181.2 epi.lncRNA

AC068831.1 epi.lncRNA

LINC01275 epi.lncRNA

AC138028.4 epi.lncRNA

AL133387.1 epi.lncRNA

AC004884.2 epi.lncRNA

AC016394.1 epi.lncRNA

AL357033.1 epi.lncRNA

AL512343.2 epi.lncRNA

AC015712.5 epi.lncRNA

LINC01633 epi.lncRNA

SYNJ2-IT1 epi.lncRNA

CLSTN2-AS1 epi.lncRNA

AC099342.1 epi.lncRNA

AC004522.3 epi.lncRNA

MIR210HG epi.lncRNA

LINC01013 epi.lncRNA

AC245033.2 epi.lncRNA

AC015853.2 epi.lncRNA

AL359397.1 epi.lncRNA

AC114956.2 epi.lncRNA

OSMR-AS1 epi.lncRNA

AC022960.1 epi.lncRNA

AL031733.2 epi.lncRNA

LINC01583 epi.lncRNA

CACNA1C-IT2 epi.lncRNA

AC007731.3 epi.lncRNA

AC005180.1 epi.lncRNA

C1orf229 epi.lncRNA

AC121761.1 epi.lncRNA

LINC01927 epi.lncRNA

SNHG32 epi.lncRNA

AC007114.1 epi.lncRNA

AC073316.3 epi.lncRNA

AP001178.3 epi.lncRNA

AC023908.3 epi.lncRNA

ANKRD10-IT1 epi.lncRNA

AP003680.1 epi.lncRNA

AC009269.2 epi.lncRNA

AC020916.2 epi.lncRNA

AC108751.4 epi.lncRNA

AC007570.1 epi.lncRNA

AC132192.1 epi.lncRNA

AC097717.1 epi.lncRNA

AC097359.2 epi.lncRNA

WWTR1-IT1 epi.lncRNA

AL133243.2 epi.lncRNA

METTL14-DT epi.lncRNA

AC023632.2 epi.lncRNA

AC006460.1 epi.lncRNA

AP003064.2 epi.lncRNA

LCMT1-AS1 epi.lncRNA

AC020661.4 epi.lncRNA

AL132671.1 epi.lncRNA

IGF2-AS epi.lncRNA

AC074194.1 epi.lncRNA

AC027682.6 epi.lncRNA

AC137932.1 epi.lncRNA

C18orf65 epi.lncRNA

AL137856.1 epi.lncRNA

AC010761.2 epi.lncRNA

AL359643.2 epi.lncRNA

AC013270.1 epi.lncRNA

AL353596.1 epi.lncRNA

AL929236.1 epi.lncRNA

AC103563.7 epi.lncRNA

AC026691.1 epi.lncRNA

AC147651.1 epi.lncRNA

AL357033.2 epi.lncRNA

C1orf143 epi.lncRNA

AC024559.1 epi.lncRNA

LINC01952 epi.lncRNA

WWC2-AS1 epi.lncRNA

AC021242.3 epi.lncRNA

AC005264.1 epi.lncRNA

PRR7-AS1 epi.lncRNA

AC105389.2 epi.lncRNA

AP000553.3 epi.lncRNA

AC092068.2 epi.lncRNA

AL136309.4 epi.lncRNA

AC091173.1 epi.lncRNA

AC005828.3 epi.lncRNA

AC087190.2 epi.lncRNA

NEBL-AS1 epi.lncRNA

DIP2A-IT1 epi.lncRNA

AP000755.1 epi.lncRNA

AL031963.2 epi.lncRNA

AL022329.1 epi.lncRNA

AC010931.3 epi.lncRNA

TRG-AS1 epi.lncRNA

AC073052.2 epi.lncRNA

AC244035.1 epi.lncRNA

AL157702.2 epi.lncRNA

AC016999.1 epi.lncRNA

AL137025.1 epi.lncRNA

AC005005.4 epi.lncRNA

AL163051.1 epi.lncRNA

AC005746.3 epi.lncRNA

AL133330.2 epi.lncRNA

LINC02371 epi.lncRNA

MRVI1-AS1 epi.lncRNA

AL161935.1 epi.lncRNA

HCG9 epi.lncRNA

AL158801.3 epi.lncRNA

AC015909.2 epi.lncRNA

ATP11A-AS1 epi.lncRNA

AC006205.2 epi.lncRNA

LINC01836 epi.lncRNA

LINC01856 epi.lncRNA

AL121989.1 epi.lncRNA

AC080038.1 epi.lncRNA

AL034417.2 epi.lncRNA

AL035588.1 epi.lncRNA

AP005131.3 epi.lncRNA

AL590666.3 epi.lncRNA

LINC01187 epi.lncRNA

AC010999.2 epi.lncRNA

AC026774.1 epi.lncRNA

AC099568.2 epi.lncRNA

LINC01645 epi.lncRNA

HTT-AS epi.lncRNA

ABCC5-AS1 epi.lncRNA

AL592071.1 epi.lncRNA

AL590708.1 epi.lncRNA

AC012370.1 epi.lncRNA

AC003991.2 epi.lncRNA

AC073195.1 epi.lncRNA

AL031432.3 epi.lncRNA

AC021660.2 epi.lncRNA

AC133106.1 epi.lncRNA

AL731569.1 epi.lncRNA

HMMR-AS1 epi.lncRNA

ARHGEF7-AS2 epi.lncRNA

LINC02076 epi.lncRNA

AC024909.1 epi.lncRNA

GRPEL2-AS1 epi.lncRNA

AC100786.1 epi.lncRNA

LEMD1-DT epi.lncRNA

LINC02571 epi.lncRNA

AC034236.2 epi.lncRNA

AC121757.1 epi.lncRNA

AC022217.2 epi.lncRNA

ATP2A1-AS1 epi.lncRNA

AC004832.4 epi.lncRNA

AC021054.1 epi.lncRNA

AL451069.2 epi.lncRNA

AC073508.3 epi.lncRNA

LINC01768 epi.lncRNA

AC078820.1 epi.lncRNA

LINC02598 epi.lncRNA

DNAH10OS epi.lncRNA

LINC02026 epi.lncRNA

AC009996.1 epi.lncRNA

AC002128.1 epi.lncRNA

C22orf24 epi.lncRNA

AC073842.2 epi.lncRNA

AC006947.1 epi.lncRNA

LINC01983 epi.lncRNA

AC111149.2 epi.lncRNA

AC097059.1 epi.lncRNA

AC097724.1 epi.lncRNA

AL161781.2 epi.lncRNA

AC025263.1 epi.lncRNA

AC100778.2 epi.lncRNA

DNAJB5-DT epi.lncRNA

SP2-AS1 epi.lncRNA

AC032011.1 epi.lncRNA

AC090826.2 epi.lncRNA

MRPL20-AS1 epi.lncRNA

AC016405.1 epi.lncRNA

AC046168.1 epi.lncRNA

KCNJ2-AS1 epi.lncRNA

NRSN2-AS1 epi.lncRNA

AC010883.1 epi.lncRNA

AC010168.1 epi.lncRNA

AP003174.1 epi.lncRNA

AL731563.2 epi.lncRNA

NCBP2-AS1 epi.lncRNA

AL358613.2 epi.lncRNA

AC136475.7 epi.lncRNA

AC140725.1 epi.lncRNA

LINC01962 epi.lncRNA

PACRG-AS3 epi.lncRNA

AC012170.3 epi.lncRNA

AC016888.1 epi.lncRNA

AC091729.2 epi.lncRNA

ZNF582-AS1 epi.lncRNA

AP005482.2 epi.lncRNA

AC008937.3 epi.lncRNA

AL583722.4 epi.lncRNA

SHANK2-AS2 epi.lncRNA

AC025031.1 epi.lncRNA

AL133371.3 epi.lncRNA

AC226118.1 epi.lncRNA

AL590068.1 epi.lncRNA

AC011377.1 epi.lncRNA

LINC01489 epi.lncRNA

AC069029.1 epi.lncRNA

AC063919.1 epi.lncRNA

AL162511.1 epi.lncRNA

AC087752.3 epi.lncRNA

AC004816.2 epi.lncRNA

AC010307.2 epi.lncRNA

AC068789.1 epi.lncRNA

LINC00167 epi.lncRNA

AC125603.3 epi.lncRNA

AL157402.2 epi.lncRNA

AC121338.2 epi.lncRNA

CEP83-DT epi.lncRNA

AC104938.1 epi.lncRNA

SPACA6P-AS epi.lncRNA

LINC01277 epi.lncRNA

AC138965.2 epi.lncRNA

AL356488.2 epi.lncRNA

AL162734.1 epi.lncRNA

AC007663.4 epi.lncRNA

AC006483.2 epi.lncRNA

AC006058.2 epi.lncRNA

LINC02265 epi.lncRNA

AC011611.2 epi.lncRNA

AC105935.2 epi.lncRNA

AC012447.1 epi.lncRNA

AC114271.1 epi.lncRNA

AL035252.3 epi.lncRNA

VAV3-AS1 epi.lncRNA

AC135050.4 epi.lncRNA

AL358333.3 epi.lncRNA

AC092338.2 epi.lncRNA

AL138787.2 epi.lncRNA

AL160286.2 epi.lncRNA

POU6F2-AS1 epi.lncRNA

AC034223.2 epi.lncRNA

AL499627.1 epi.lncRNA

SCAT2 epi.lncRNA

AP000439.2 epi.lncRNA

LINC01839 epi.lncRNA

AL590666.1 epi.lncRNA

LINC01424 epi.lncRNA

AC008972.1 epi.lncRNA

AC100821.2 epi.lncRNA

AC079336.4 epi.lncRNA

AC011389.2 epi.lncRNA

AC106779.1 epi.lncRNA

AC005899.4 epi.lncRNA

AC097382.1 epi.lncRNA

AF235103.1 epi.lncRNA

AC010300.1 epi.lncRNA

AC005355.1 epi.lncRNA

AL137802.1 epi.lncRNA

AC004771.4 epi.lncRNA

LINC02130 epi.lncRNA

AL133353.1 epi.lncRNA

ITCH-IT1 epi.lncRNA

AC021321.1 epi.lncRNA

AL031600.2 epi.lncRNA

AC009088.1 epi.lncRNA

AL357874.1 epi.lncRNA

AP001007.1 epi.lncRNA

AL139287.1 epi.lncRNA

AP001178.1 epi.lncRNA

LAMA5-AS1 epi.lncRNA

AC232271.1 epi.lncRNA

AC080013.1 epi.lncRNA

AC008966.1 epi.lncRNA

Z84723.1 epi.lncRNA

AL357500.1 epi.lncRNA

AC128709.3 epi.lncRNA

CARNMT1-AS1 epi.lncRNA

AC005625.1 epi.lncRNA

AL590483.1 epi.lncRNA

LINC02625 epi.lncRNA

AL356414.1 epi.lncRNA

LINC01932 epi.lncRNA

VIM-AS1 epi.lncRNA

BICRA-AS1 epi.lncRNA

FAM87B epi.lncRNA

LINC00602 epi.lncRNA

LINC02092 epi.lncRNA

AC097375.1 epi.lncRNA

AL136528.1 epi.lncRNA

AC022405.1 epi.lncRNA

AL355613.1 epi.lncRNA

AC118755.1 epi.lncRNA

AL022323.3 epi.lncRNA

AC103853.1 epi.lncRNA

AC034102.7 epi.lncRNA

BX322562.1 epi.lncRNA

LINC01273 epi.lncRNA

AC233992.3 epi.lncRNA

AC021752.1 epi.lncRNA

ZNF460-AS1 epi.lncRNA

AC093334.1 epi.lncRNA

AP002907.1 epi.lncRNA

AC090826.1 epi.lncRNA

AC126696.2 epi.lncRNA

AC017104.1 epi.lncRNA

AC022558.3 epi.lncRNA

AC073111.1 epi.lncRNA

LINC01597 epi.lncRNA

AL445531.1 epi.lncRNA

AC011491.3 epi.lncRNA

AC008406.3 epi.lncRNA

AC107982.2 epi.lncRNA

AL133343.1 epi.lncRNA

AC022893.3 epi.lncRNA

AC004408.1 epi.lncRNA

AC006329.1 epi.lncRNA

AC074138.1 epi.lncRNA

AC015871.4 epi.lncRNA

AC064805.2 epi.lncRNA

LINC00028 epi.lncRNA

AC025754.2 epi.lncRNA

AC021491.1 epi.lncRNA

DNAH17-AS1 epi.lncRNA

AC040169.1 epi.lncRNA

AC007298.2 epi.lncRNA

AL606970.1 epi.lncRNA

AC011444.2 epi.lncRNA

AP000679.1 epi.lncRNA

LINC01873 epi.lncRNA

LINC00364 epi.lncRNA

LINC01704 epi.lncRNA

AL109917.1 epi.lncRNA

AC243654.1 epi.lncRNA

AL136038.2 epi.lncRNA

AC104024.1 epi.lncRNA

AC127070.2 epi.lncRNA

LINC01800 epi.lncRNA

LINC02243 epi.lncRNA

AC003101.1 epi.lncRNA

AC002094.1 epi.lncRNA

AC092145.1 epi.lncRNA

AP003721.4 epi.lncRNA

MIS18A-AS1 epi.lncRNA

AP005482.1 epi.lncRNA

UBE2E2-AS1 epi.lncRNA

AL121900.1 epi.lncRNA

AC106028.2 epi.lncRNA

AC099508.2 epi.lncRNA

PCAT14 epi.lncRNA

AC016745.1 epi.lncRNA

AC073288.1 epi.lncRNA

AC093249.6 epi.lncRNA

LINC00987 epi.lncRNA

AL355483.2 epi.lncRNA

AC139491.2 epi.lncRNA

PSD2-AS1 epi.lncRNA

AC114947.2 epi.lncRNA

LINC00485 epi.lncRNA

AC124016.1 epi.lncRNA

TXNDC12-AS1 epi.lncRNA

CAHM epi.lncRNA

Z99572.1 epi.lncRNA

AC012307.1 epi.lncRNA

AC020922.3 epi.lncRNA

AC105254.1 epi.lncRNA

LINC02455 epi.lncRNA

AP000866.6 epi.lncRNA

LUARIS epi.lncRNA

AC010319.4 epi.lncRNA

AL445524.1 epi.lncRNA

AL645608.8 epi.lncRNA

AC066612.2 epi.lncRNA

AP001360.1 epi.lncRNA

AC013476.1 epi.lncRNA

AC022001.3 epi.lncRNA

EVX1-AS epi.lncRNA

AC009090.2 epi.lncRNA

AC068987.2 epi.lncRNA

SLC16A1-AS1 epi.lncRNA

AC022211.2 epi.lncRNA

AC007450.1 epi.lncRNA

AL022328.4 epi.lncRNA

AL023581.2 epi.lncRNA

AC124068.2 epi.lncRNA

PLCE1-AS2 epi.lncRNA

LINC02551 epi.lncRNA

SLC16A12-AS1 epi.lncRNA

AC139099.2 epi.lncRNA

GLIS2-AS1 epi.lncRNA

AL121906.1 epi.lncRNA

AL117332.1 epi.lncRNA

AC009065.7 epi.lncRNA

AL035078.1 epi.lncRNA

LINC02036 epi.lncRNA

AL391361.2 epi.lncRNA

AC012456.2 epi.lncRNA

AC013457.1 epi.lncRNA

BFSP2-AS1 epi.lncRNA

AP005137.2 epi.lncRNA

AC021851.1 epi.lncRNA

AC105760.1 epi.lncRNA

AL353708.1 epi.lncRNA

LINC02771 epi.lncRNA

AC005096.1 epi.lncRNA

AC128709.2 epi.lncRNA

FGF12-AS3 epi.lncRNA

AC233702.7 epi.lncRNA

AC092265.1 epi.lncRNA

AL356489.2 epi.lncRNA

AL157902.1 epi.lncRNA

AC027682.5 epi.lncRNA

AC011625.1 epi.lncRNA

AC023421.1 epi.lncRNA

LINC00283 epi.lncRNA

LINC01795 epi.lncRNA

AC008555.2 epi.lncRNA

AL450469.2 epi.lncRNA

IDH2-DT epi.lncRNA

AL663074.1 epi.lncRNA

LINC02697 epi.lncRNA

LINC02362 epi.lncRNA

AC135166.1 epi.lncRNA

AC017104.3 epi.lncRNA

LINC02528 epi.lncRNA

AC111170.1 epi.lncRNA

AC079171.1 epi.lncRNA

AC096637.2 epi.lncRNA

AC107959.2 epi.lncRNA

AC078883.2 epi.lncRNA

AC003965.1 epi.lncRNA

ZNF630-AS1 epi.lncRNA

AC006064.4 epi.lncRNA

ALMS1-IT1 epi.lncRNA

AL445183.2 epi.lncRNA

AC004466.2 epi.lncRNA

AP002833.2 epi.lncRNA

AL135744.1 epi.lncRNA

AC092849.1 epi.lncRNA

AL355488.1 epi.lncRNA

TIMM23B-AGAP6 epi.lncRNA

AC078962.3 epi.lncRNA

AC026461.3 epi.lncRNA

AL138789.1 epi.lncRNA

AC141424.1 epi.lncRNA

AC106786.2 epi.lncRNA

AC005828.4 epi.lncRNA

ITPRIP-AS1 epi.lncRNA

AC100871.2 epi.lncRNA

AC112907.3 epi.lncRNA

LINC02583 epi.lncRNA

AP001893.1 epi.lncRNA

LNCTAM34A epi.lncRNA

AL359095.1 epi.lncRNA

AC011595.1 epi.lncRNA

AC131009.2 epi.lncRNA

RERE-AS1 epi.lncRNA

LINC02639 epi.lncRNA

AC073912.2 epi.lncRNA

CYP51A1-AS1 epi.lncRNA

AC008781.1 epi.lncRNA

AC090559.2 epi.lncRNA

AC116609.3 epi.lncRNA

LINC01395 epi.lncRNA

AC009908.1 epi.lncRNA

AL160411.1 epi.lncRNA

AC012173.1 epi.lncRNA

KCNIP2-AS1 epi.lncRNA

AC013468.1 epi.lncRNA

AL138916.2 epi.lncRNA

AC022296.4 epi.lncRNA

CLIP1-AS1 epi.lncRNA

AC090198.1 epi.lncRNA

AC005996.1 epi.lncRNA

AC124319.2 epi.lncRNA

AL132711.1 epi.lncRNA

AC097478.3 epi.lncRNA

AC006557.3 epi.lncRNA

AL390067.1 epi.lncRNA

AC090971.1 epi.lncRNA

AC006148.2 epi.lncRNA

AL353597.1 epi.lncRNA

AC093821.1 epi.lncRNA

LINC02757 epi.lncRNA

AC121761.2 epi.lncRNA

AC079848.1 epi.lncRNA

AC067930.3 epi.lncRNA

LINC02555 epi.lncRNA

AP003717.1 epi.lncRNA

AL445685.1 epi.lncRNA

IL6R-AS1 epi.lncRNA

BMPR1B-DT epi.lncRNA

AC026979.2 epi.lncRNA

AL035634.1 epi.lncRNA

BRWD1-AS1 epi.lncRNA

AC008429.1 epi.lncRNA

AC005076.1 epi.lncRNA

LINC01270 epi.lncRNA

AC084824.5 epi.lncRNA

AC053503.1 epi.lncRNA

COL18A1-AS2 epi.lncRNA

AP001527.2 epi.lncRNA

AL139246.4 epi.lncRNA

LINC01635 epi.lncRNA

AC008915.2 epi.lncRNA

LINC01972 epi.lncRNA

AL365273.1 epi.lncRNA

AL360091.1 epi.lncRNA

AC020663.3 epi.lncRNA

AP001970.1 epi.lncRNA

AC006159.1 epi.lncRNA

AL132989.2 epi.lncRNA

AC068888.2 epi.lncRNA

AL133492.1 epi.lncRNA

AC005392.3 epi.lncRNA

AC006041.1 epi.lncRNA

LINC01447 epi.lncRNA

AC027682.1 epi.lncRNA

KIF9-AS1 epi.lncRNA

AC123567.2 epi.lncRNA

AC012506.4 epi.lncRNA

LINC02848 epi.lncRNA

LINC00686 epi.lncRNA

AC005332.2 epi.lncRNA

AC010789.1 epi.lncRNA

AC090023.2 epi.lncRNA

AL450003.1 epi.lncRNA

AL163973.3 epi.lncRNA

LINC02817 epi.lncRNA

IQCA1-AS1 epi.lncRNA

ITCH-AS1 epi.lncRNA

AL353803.1 epi.lncRNA

AC116312.1 epi.lncRNA

LINC02580 epi.lncRNA

AL136528.2 epi.lncRNA

AC004973.1 epi.lncRNA

AC022101.1 epi.lncRNA

AC097358.2 epi.lncRNA

LINC01555 epi.lncRNA

AC008464.1 epi.lncRNA

AP004609.3 epi.lncRNA

AL139035.1 epi.lncRNA

AL162411.1 epi.lncRNA

AC026785.3 epi.lncRNA

AC008154.1 epi.lncRNA

MNX1-AS1 epi.lncRNA

AC078864.1 epi.lncRNA

AL133260.2 epi.lncRNA

AC092171.1 epi.lncRNA

AP002336.3 epi.lncRNA

AC099811.4 epi.lncRNA

AC011500.3 epi.lncRNA

NAALADL2-AS2 epi.lncRNA

AL591926.2 epi.lncRNA

AL109615.2 epi.lncRNA

GAS6-DT epi.lncRNA

AC004797.1 epi.lncRNA

AL031665.1 epi.lncRNA

AC083864.3 epi.lncRNA

AC018450.1 epi.lncRNA

AL450311.1 epi.lncRNA

LINC00397 epi.lncRNA

AC064852.1 epi.lncRNA

AC096996.2 epi.lncRNA

AC006355.2 epi.lncRNA

AC103746.1 epi.lncRNA

LINC02736 epi.lncRNA

AC144450.1 epi.lncRNA

LINC02709 epi.lncRNA

AC012506.3 epi.lncRNA

AP001469.1 epi.lncRNA

ANK3-DT epi.lncRNA

AC005091.1 epi.lncRNA

AC109460.1 epi.lncRNA

AC087477.4 epi.lncRNA

LINC02037 epi.lncRNA

ATP6V0E2-AS1 epi.lncRNA

AC002480.2 epi.lncRNA

AL024498.1 epi.lncRNA

AC015849.4 epi.lncRNA

AC007950.1 epi.lncRNA

AL160408.4 epi.lncRNA

AC044839.3 epi.lncRNA

LINC02489 epi.lncRNA

AC010336.5 epi.lncRNA

AP000688.4 epi.lncRNA

SNTG2-AS1 epi.lncRNA

AC093627.4 epi.lncRNA

AC018410.2 epi.lncRNA

AP001198.1 epi.lncRNA

AC079414.3 epi.lncRNA

AL589182.1 epi.lncRNA

AC015813.4 epi.lncRNA

AC106820.5 epi.lncRNA

AC005730.2 epi.lncRNA

AC093726.2 epi.lncRNA

AC116003.1 epi.lncRNA

AC068025.1 epi.lncRNA

AC024619.3 epi.lncRNA

AC009107.1 epi.lncRNA

AP001150.1 epi.lncRNA

MIR22HG epi.lncRNA

RNU6ATAC35P epi.lncRNA

AC020659.2 epi.lncRNA

AL161937.1 epi.lncRNA

AC127459.2 epi.lncRNA

AC104316.2 epi.lncRNA

LINC00570 epi.lncRNA

AC026347.1 epi.lncRNA

AC012625.1 epi.lncRNA

AL442067.1 epi.lncRNA

AC241644.2 epi.lncRNA

AC027307.3 epi.lncRNA

AL139384.2 epi.lncRNA

AL157871.6 epi.lncRNA

AC100812.1 epi.lncRNA

INE1 epi.lncRNA

OGFRP1 epi.lncRNA

DCTN1-AS1 epi.lncRNA

AC048383.1 epi.lncRNA

RAI1-AS1 epi.lncRNA

LINC01738 epi.lncRNA

AC005614.2 epi.lncRNA

AC093496.1 epi.lncRNA

AC010223.1 epi.lncRNA

AC102953.1 epi.lncRNA

AC122129.2 epi.lncRNA

AC128709.1 epi.lncRNA

AC008438.2 epi.lncRNA

AC073573.1 epi.lncRNA

LINC01513 epi.lncRNA

AL121832.3 epi.lncRNA

AL390718.1 epi.lncRNA

AC068025.2 epi.lncRNA

AL139161.1 epi.lncRNA

AC090587.1 epi.lncRNA

CERNA3 epi.lncRNA

AL391097.1 epi.lncRNA

AC092687.2 epi.lncRNA

AC004221.1 epi.lncRNA

AC244034.2 epi.lncRNA

AL645504.1 epi.lncRNA

AL139275.1 epi.lncRNA

AC087664.1 epi.lncRNA

AC023161.1 epi.lncRNA

AC079600.3 epi.lncRNA

KIRREL1-IT1 epi.lncRNA

AC026469.1 epi.lncRNA

AC011753.2 epi.lncRNA

INTS6L-AS1 epi.lncRNA

AC025162.1 epi.lncRNA

AC091047.1 epi.lncRNA

Z97652.1 epi.lncRNA

AC024257.1 epi.lncRNA

AL021396.1 epi.lncRNA

AC005828.1 epi.lncRNA

LINC02730 epi.lncRNA

AC078929.1 epi.lncRNA

AC090617.3 epi.lncRNA

AL035420.1 epi.lncRNA

AL162741.1 epi.lncRNA

AC126407.1 epi.lncRNA

AC022973.4 epi.lncRNA

AC127024.6 epi.lncRNA

UBR5-AS1 epi.lncRNA

TBL1XR1-AS1 epi.lncRNA

AC244100.3 epi.lncRNA

CCDC39-AS1 epi.lncRNA

AC026780.2 epi.lncRNA

PIK3CD-AS2 epi.lncRNA

AC083880.1 epi.lncRNA

C6orf99 epi.lncRNA

AC114760.2 epi.lncRNA

LINC00958 epi.lncRNA

AC018529.2 epi.lncRNA

AC063926.1 epi.lncRNA

AC007448.4 epi.lncRNA

AL133279.1 epi.lncRNA

AL359378.1 epi.lncRNA

AC099811.1 epi.lncRNA

AC018943.1 epi.lncRNA

AC051649.1 epi.lncRNA

AC127024.2 epi.lncRNA

LINC01139 epi.lncRNA

AC092807.2 epi.lncRNA

AC055860.1 epi.lncRNA

AP000977.1 epi.lncRNA

LINC02029 epi.lncRNA

UBE2E1-AS1 epi.lncRNA

AC078962.4 epi.lncRNA

STK24-AS1 epi.lncRNA

AC009093.6 epi.lncRNA

AC018521.3 epi.lncRNA

GACAT2 epi.lncRNA

LINC01531 epi.lncRNA

LINC02570 epi.lncRNA

AC087257.1 epi.lncRNA

AL049870.2 epi.lncRNA

AC002546.1 epi.lncRNA

AP001271.1 epi.lncRNA

AC087672.1 epi.lncRNA

LLPH-DT epi.lncRNA

AL160270.1 epi.lncRNA

AC119677.1 epi.lncRNA

AC103853.2 epi.lncRNA

AC073592.1 epi.lncRNA

AC025178.1 epi.lncRNA

AP003119.2 epi.lncRNA

TUSC8 epi.lncRNA

PGM5-AS1 epi.lncRNA

AC104187.1 epi.lncRNA

AC010207.1 epi.lncRNA

ATP2C2-AS1 epi.lncRNA

AC011257.1 epi.lncRNA

AL133304.2 epi.lncRNA

AC024337.1 epi.lncRNA

AC007193.1 epi.lncRNA

LINC02399 epi.lncRNA

AC068898.1 epi.lncRNA

LINC01909 epi.lncRNA

AC079779.1 epi.lncRNA

AC104564.3 epi.lncRNA

AL110115.2 epi.lncRNA

AC092953.2 epi.lncRNA

AP003352.1 epi.lncRNA

AL353708.2 epi.lncRNA

LINC01134 epi.lncRNA

AP001266.2 epi.lncRNA

AC007040.1 epi.lncRNA

AL360295.1 epi.lncRNA

AC024587.2 epi.lncRNA

AC025031.2 epi.lncRNA

LINCMD1 epi.lncRNA

AL162293.1 epi.lncRNA

AC012640.4 epi.lncRNA

LINC01080 epi.lncRNA

AL365436.2 epi.lncRNA

AC091271.1 epi.lncRNA

ASAP1-IT2 epi.lncRNA

AC126323.6 epi.lncRNA

AC024575.1 epi.lncRNA

AC246793.1 epi.lncRNA

AC007834.1 epi.lncRNA

AL445647.1 epi.lncRNA

AC044839.1 epi.lncRNA

MKX-AS1 epi.lncRNA

AP000777.2 epi.lncRNA

AC021237.1 epi.lncRNA

LINC02683 epi.lncRNA

AC005225.3 epi.lncRNA

AC024267.4 epi.lncRNA

AL136131.2 epi.lncRNA

AC027419.2 epi.lncRNA

AC092574.1 epi.lncRNA

AC007216.1 epi.lncRNA

AC022447.2 epi.lncRNA

AL596087.3 epi.lncRNA

AC068620.1 epi.lncRNA

LINC01543 epi.lncRNA

AL451069.1 epi.lncRNA

AC068724.1 epi.lncRNA

AC008440.2 epi.lncRNA

AC084759.2 epi.lncRNA

LINC01922 epi.lncRNA

AC090023.1 epi.lncRNA

TNK2-AS1 epi.lncRNA

AC026780.1 epi.lncRNA

AC005523.1 epi.lncRNA

AC010401.2 epi.lncRNA

LINC02707 epi.lncRNA

AC051619.7 epi.lncRNA

AC066612.1 epi.lncRNA

AL355103.1 epi.lncRNA

AC026316.2 epi.lncRNA

AC078909.2 epi.lncRNA

AL049869.2 epi.lncRNA

AC092747.1 epi.lncRNA

AL732292.2 epi.lncRNA

AC009313.1 epi.lncRNA

SRGAP3-AS4 epi.lncRNA

AC004801.5 epi.lncRNA

AC007663.3 epi.lncRNA

AC055764.1 epi.lncRNA

UVRAG-DT epi.lncRNA

AL353801.1 epi.lncRNA

AC068234.3 epi.lncRNA

AC011443.1 epi.lncRNA

L34079.3 epi.lncRNA

AC068295.1 epi.lncRNA

AP003472.2 epi.lncRNA

AP003059.1 epi.lncRNA

AC125494.3 epi.lncRNA

AC103739.2 epi.lncRNA

LINC02003 epi.lncRNA

TRPC7-AS2 epi.lncRNA

AP000688.2 epi.lncRNA

LINC02225 epi.lncRNA

CSMD2-AS1 epi.lncRNA

AC026124.1 epi.lncRNA

AF038458.3 epi.lncRNA

AC005220.1 epi.lncRNA

LINC02710 epi.lncRNA

LINC00533 epi.lncRNA

TCEAL3-AS1 epi.lncRNA

AC024382.1 epi.lncRNA

AC127540.1 epi.lncRNA

AC114752.2 epi.lncRNA

AC008883.1 epi.lncRNA

AC018761.3 epi.lncRNA

AL049651.2 epi.lncRNA

AL138916.1 epi.lncRNA

AC126696.3 epi.lncRNA

AL121904.1 epi.lncRNA

LINC02813 epi.lncRNA

AL353747.4 epi.lncRNA

NAV2-AS4 epi.lncRNA

DDX11-AS1 epi.lncRNA

AC002550.1 epi.lncRNA

AP001065.4 epi.lncRNA

SGMS1-AS1 epi.lncRNA

AC100872.2 epi.lncRNA

AC004706.1 epi.lncRNA

AC022196.1 epi.lncRNA

AC096746.1 epi.lncRNA

AC025580.2 epi.lncRNA

EHD4-AS1 epi.lncRNA

AP003465.1 epi.lncRNA

AC053503.2 epi.lncRNA

AC025162.2 epi.lncRNA

AC092070.1 epi.lncRNA

AC066613.1 epi.lncRNA

RAD21-AS1 epi.lncRNA

AC018832.1 epi.lncRNA

AL355773.1 epi.lncRNA

AC008696.2 epi.lncRNA

LINC01011 epi.lncRNA

LINC02111 epi.lncRNA

AL049541.1 epi.lncRNA

AC084824.4 epi.lncRNA

AC068418.1 epi.lncRNA

AL157884.2 epi.lncRNA

AL049796.1 epi.lncRNA

AL049780.1 epi.lncRNA

AC243773.2 epi.lncRNA

AC019103.1 epi.lncRNA

AL390763.1 epi.lncRNA

AC116913.1 epi.lncRNA

ADGRA1-AS1 epi.lncRNA

AP003550.1 epi.lncRNA

AP000355.1 epi.lncRNA

ST20-AS1 epi.lncRNA

AC055733.2 epi.lncRNA

AC097381.1 epi.lncRNA

AL354993.1 epi.lncRNA

AP001412.1 epi.lncRNA

AP003068.2 epi.lncRNA

AC104590.1 epi.lncRNA

AC092127.2 epi.lncRNA

TMEM9B-AS1 epi.lncRNA

AC008735.3 epi.lncRNA

AC021739.4 epi.lncRNA

CCDC37-DT epi.lncRNA

AC243547.2 epi.lncRNA

AC073342.2 epi.lncRNA

AL358154.1 epi.lncRNA

AL355312.2 epi.lncRNA

BMF-AS1 epi.lncRNA

AL031710.1 epi.lncRNA

AC009097.1 epi.lncRNA

AC104667.2 epi.lncRNA

AC107896.1 epi.lncRNA

AC004994.1 epi.lncRNA

C9orf135-DT epi.lncRNA

AC078785.2 epi.lncRNA

AC002428.1 epi.lncRNA

AC010442.1 epi.lncRNA

AC103740.2 epi.lncRNA

AC073316.2 epi.lncRNA

LINC01845 epi.lncRNA

HS1BP3-IT1 epi.lncRNA

AL133383.1 epi.lncRNA

AC044849.1 epi.lncRNA

AC021066.1 epi.lncRNA

AP002478.2 epi.lncRNA

PAXIP1-AS1 epi.lncRNA

AC087439.1 epi.lncRNA

AL162385.2 epi.lncRNA

AC092614.1 epi.lncRNA

AP003419.3 epi.lncRNA

AC006449.1 epi.lncRNA

AC013726.1 epi.lncRNA

AC008453.1 epi.lncRNA

AC095030.1 epi.lncRNA

AL589986.1 epi.lncRNA

AC012379.2 epi.lncRNA

SRGAP3-AS3 epi.lncRNA

AC020980.1 epi.lncRNA

AC027544.2 epi.lncRNA

AC096637.1 epi.lncRNA

AP000350.5 epi.lncRNA

AL512604.2 epi.lncRNA

AC083864.2 epi.lncRNA

AL670729.2 epi.lncRNA

AL358334.1 epi.lncRNA

ARRDC1-AS1 epi.lncRNA

AC092119.2 epi.lncRNA

AC019349.1 epi.lncRNA

LINC01740 epi.lncRNA

RCC2-AS1 epi.lncRNA

AC016027.1 epi.lncRNA

CREB3L2-AS1 epi.lncRNA

SNHG4 epi.lncRNA

AC009704.2 epi.lncRNA

AL450468.2 epi.lncRNA

AC083862.2 epi.lncRNA

AP003396.1 epi.lncRNA

AC073283.2 epi.lncRNA

AC002368.1 epi.lncRNA

AL022313.4 epi.lncRNA

LINC02475 epi.lncRNA

AL133230.1 epi.lncRNA

AL133163.2 epi.lncRNA

PYCARD-AS1 epi.lncRNA

AC087612.1 epi.lncRNA

AC008033.3 epi.lncRNA

AL732314.6 epi.lncRNA

AC073957.1 epi.lncRNA

AC009509.2 epi.lncRNA

AC022392.1 epi.lncRNA

AC087620.1 epi.lncRNA

AL354892.1 epi.lncRNA

AL033528.2 epi.lncRNA

AL161618.1 epi.lncRNA

AL391262.1 epi.lncRNA

AC073389.2 epi.lncRNA

LINC01895 epi.lncRNA

AC021491.3 epi.lncRNA

AC026461.1 epi.lncRNA

AC093915.1 epi.lncRNA

AC034228.2 epi.lncRNA

ADGRD1-AS1 epi.lncRNA

TSPAN18-AS1 epi.lncRNA

CASC18 epi.lncRNA

AC092687.1 epi.lncRNA

AC253576.2 epi.lncRNA

AL591178.2 epi.lncRNA

AC099482.1 epi.lncRNA

AC008758.2 epi.lncRNA

LINC01149 epi.lncRNA

AC008738.5 epi.lncRNA

LINC01486 epi.lncRNA

AC010319.3 epi.lncRNA

AC073878.1 epi.lncRNA

LINC01332 epi.lncRNA

AL031658.2 epi.lncRNA

AC004217.1 epi.lncRNA

AC005901.1 epi.lncRNA

AC233280.1 epi.lncRNA

PCOLCE-AS1 epi.lncRNA

HTR3E-AS1 epi.lncRNA

USP12-AS1 epi.lncRNA

AP001208.1 epi.lncRNA

AL591848.2 epi.lncRNA

AL442125.1 epi.lncRNA

PLCB2-AS1 epi.lncRNA

PAN3-AS1 epi.lncRNA

AC008105.3 epi.lncRNA

AC124283.1 epi.lncRNA

AC139530.3 epi.lncRNA

AC090607.3 epi.lncRNA

AC022001.2 epi.lncRNA

ACTA2-AS1 epi.lncRNA

AC109454.3 epi.lncRNA

AC005498.1 epi.lncRNA

AC073575.1 epi.lncRNA

AC092296.1 epi.lncRNA

AC076968.1 epi.lncRNA

AL512288.1 epi.lncRNA

FBXO3-DT epi.lncRNA

AC244453.1 epi.lncRNA

MRPS30-DT epi.lncRNA

AL356309.2 epi.lncRNA

AC007406.2 epi.lncRNA

LINC01114 epi.lncRNA

AC083805.2 epi.lncRNA

AL022725.1 epi.lncRNA

LINGO1-AS1 epi.lncRNA

AL365214.2 epi.lncRNA

AL133244.2 epi.lncRNA

DDX59-AS1 epi.lncRNA

AL139384.1 epi.lncRNA

LINC02124 epi.lncRNA

AP000842.3 epi.lncRNA

AL117328.2 epi.lncRNA

AP000289.1 epi.lncRNA

AL353719.1 epi.lncRNA

AC140912.1 epi.lncRNA

LINC01255 epi.lncRNA

DUXAP8 epi.lncRNA

AL611929.1 epi.lncRNA

AC004253.2 epi.lncRNA

MCM8-AS1 epi.lncRNA

AL136982.7 epi.lncRNA

LINC01854 epi.lncRNA

LINC00184 epi.lncRNA

AC016705.1 epi.lncRNA

AC006058.1 epi.lncRNA

AC011092.2 epi.lncRNA

AC023906.2 epi.lncRNA

AP001961.1 epi.lncRNA

AP001790.1 epi.lncRNA

AC138956.2 epi.lncRNA

AL590640.1 epi.lncRNA

AC032019.1 epi.lncRNA

AC104076.1 epi.lncRNA

LINC02352 epi.lncRNA

AL603839.1 epi.lncRNA

AC073896.5 epi.lncRNA

LINC00265 epi.lncRNA

AP001972.1 epi.lncRNA

AC103808.5 epi.lncRNA

AC023024.1 epi.lncRNA

AC018781.1 epi.lncRNA

LINC01760 epi.lncRNA

AL133330.1 epi.lncRNA

UBAC2-AS1 epi.lncRNA

BOK-AS1 epi.lncRNA

AL135785.1 epi.lncRNA

AL035078.3 epi.lncRNA

AC112196.1 epi.lncRNA

A1BG-AS1 non.epi.lncRNA

A2M-AS1 non.epi.lncRNA

A2ML1-AS1 non.epi.lncRNA

A2ML1-AS2 non.epi.lncRNA

AADACL2-AS1 non.epi.lncRNA

ABALON non.epi.lncRNA

ABCA9-AS1 non.epi.lncRNA

ABHD15-AS1 non.epi.lncRNA

AC000032.1 non.epi.lncRNA

AC000036.1 non.epi.lncRNA

AC000058.1 non.epi.lncRNA

AC000061.1 non.epi.lncRNA

AC000067.1 non.epi.lncRNA

AC000068.2 non.epi.lncRNA

AC000068.3 non.epi.lncRNA

AC000099.1 non.epi.lncRNA

AC000123.1 non.epi.lncRNA

AC000124.1 non.epi.lncRNA

AC000372.1 non.epi.lncRNA

AC000403.1 non.epi.lncRNA

AC001226.1 non.epi.lncRNA

AC002044.1 non.epi.lncRNA

AC002056.2 non.epi.lncRNA

AC002059.1 non.epi.lncRNA

AC002064.1 non.epi.lncRNA

AC002064.2 non.epi.lncRNA

AC002066.1 non.epi.lncRNA

AC002069.2 non.epi.lncRNA

AC002070.1 non.epi.lncRNA

AC002076.1 non.epi.lncRNA

AC002091.1 non.epi.lncRNA

AC002091.2 non.epi.lncRNA

AC002094.2 non.epi.lncRNA

AC002094.4 non.epi.lncRNA

AC002101.1 non.epi.lncRNA

AC002115.1 non.epi.lncRNA

AC002128.2 non.epi.lncRNA

AC002310.2 non.epi.lncRNA

AC002347.1 non.epi.lncRNA

AC002347.2 non.epi.lncRNA

AC002351.1 non.epi.lncRNA

AC002375.1 non.epi.lncRNA

AC002378.1 non.epi.lncRNA

AC002383.1 non.epi.lncRNA

AC002386.1 non.epi.lncRNA

AC002398.1 non.epi.lncRNA

AC002398.2 non.epi.lncRNA

AC002401.1 non.epi.lncRNA

AC002401.2 non.epi.lncRNA

AC002401.3 non.epi.lncRNA

AC002428.2 non.epi.lncRNA

AC002429.2 non.epi.lncRNA

AC002451.1 non.epi.lncRNA

AC002454.1 non.epi.lncRNA

AC002456.1 non.epi.lncRNA

AC002460.1 non.epi.lncRNA

AC002463.1 non.epi.lncRNA

AC002464.1 non.epi.lncRNA

AC002465.1 non.epi.lncRNA

AC002470.1 non.epi.lncRNA

AC002472.1 non.epi.lncRNA

AC002480.1 non.epi.lncRNA

AC002511.1 non.epi.lncRNA

AC002511.2 non.epi.lncRNA

AC002519.1 non.epi.lncRNA

AC002550.2 non.epi.lncRNA

AC002551.1 non.epi.lncRNA

AC002553.1 non.epi.lncRNA

AC002553.2 non.epi.lncRNA

AC002558.2 non.epi.lncRNA

AC002558.3 non.epi.lncRNA

AC002563.1 non.epi.lncRNA

AC003001.1 non.epi.lncRNA

AC003005.2 non.epi.lncRNA

AC003009.1 non.epi.lncRNA

AC003035.1 non.epi.lncRNA

AC003035.2 non.epi.lncRNA

AC003043.1 non.epi.lncRNA

AC003070.1 non.epi.lncRNA

AC003070.2 non.epi.lncRNA

AC003084.1 non.epi.lncRNA

AC003087.1 non.epi.lncRNA

AC003092.1 non.epi.lncRNA

AC003092.2 non.epi.lncRNA

AC003098.1 non.epi.lncRNA

AC003101.2 non.epi.lncRNA

AC003102.1 non.epi.lncRNA

AC003659.1 non.epi.lncRNA

AC003666.1 non.epi.lncRNA

AC003681.1 non.epi.lncRNA

AC003682.1 non.epi.lncRNA

AC003684.1 non.epi.lncRNA

AC003685.1 non.epi.lncRNA

AC003687.1 non.epi.lncRNA

AC003688.2 non.epi.lncRNA

AC003956.1 non.epi.lncRNA

AC003958.2 non.epi.lncRNA

AC003973.1 non.epi.lncRNA

AC003975.1 non.epi.lncRNA

AC003982.1 non.epi.lncRNA

AC003984.1 non.epi.lncRNA

AC003985.1 non.epi.lncRNA

AC003986.1 non.epi.lncRNA

AC003986.2 non.epi.lncRNA

AC003986.3 non.epi.lncRNA

AC003988.1 non.epi.lncRNA

AC003991.1 non.epi.lncRNA

AC004000.1 non.epi.lncRNA

AC004009.1 non.epi.lncRNA

AC004014.1 non.epi.lncRNA

AC004023.1 non.epi.lncRNA

AC004034.1 non.epi.lncRNA

AC004039.1 non.epi.lncRNA

AC004052.1 non.epi.lncRNA

AC004053.1 non.epi.lncRNA

AC004054.1 non.epi.lncRNA

AC004063.1 non.epi.lncRNA

AC004066.2 non.epi.lncRNA

AC004067.1 non.epi.lncRNA

AC004069.1 non.epi.lncRNA

AC004076.2 non.epi.lncRNA

AC004080.1 non.epi.lncRNA

AC004080.2 non.epi.lncRNA

AC004080.4 non.epi.lncRNA

AC004080.5 non.epi.lncRNA

AC004080.6 non.epi.lncRNA

AC004083.1 non.epi.lncRNA

AC004112.1 non.epi.lncRNA

AC004130.2 non.epi.lncRNA

AC004147.1 non.epi.lncRNA

AC004147.2 non.epi.lncRNA

AC004147.3 non.epi.lncRNA

AC004147.4 non.epi.lncRNA

AC004147.5 non.epi.lncRNA

AC004148.1 non.epi.lncRNA

AC004158.1 non.epi.lncRNA

AC004160.1 non.epi.lncRNA

AC004160.2 non.epi.lncRNA

AC004223.2 non.epi.lncRNA

AC004223.4 non.epi.lncRNA

AC004224.2 non.epi.lncRNA

AC004231.1 non.epi.lncRNA

AC004231.3 non.epi.lncRNA

AC004232.1 non.epi.lncRNA

AC004233.1 non.epi.lncRNA

AC004233.2 non.epi.lncRNA

AC004233.3 non.epi.lncRNA

AC004241.1 non.epi.lncRNA

AC004241.2 non.epi.lncRNA

AC004241.3 non.epi.lncRNA

AC004241.4 non.epi.lncRNA

AC004253.1 non.epi.lncRNA

AC004257.1 non.epi.lncRNA

AC004381.1 non.epi.lncRNA

AC004415.1 non.epi.lncRNA

AC004448.1 non.epi.lncRNA

AC004448.2 non.epi.lncRNA

AC004448.3 non.epi.lncRNA

AC004449.1 non.epi.lncRNA

AC004461.2 non.epi.lncRNA

AC004466.1 non.epi.lncRNA

AC004466.3 non.epi.lncRNA

AC004470.1 non.epi.lncRNA

AC004471.2 non.epi.lncRNA

AC004477.1 non.epi.lncRNA

AC004477.2 non.epi.lncRNA

AC004477.3 non.epi.lncRNA

AC004485.1 non.epi.lncRNA

AC004490.1 non.epi.lncRNA

AC004492.1 non.epi.lncRNA

AC004494.1 non.epi.lncRNA

AC004528.1 non.epi.lncRNA

AC004528.2 non.epi.lncRNA

AC004540.1 non.epi.lncRNA

AC004540.2 non.epi.lncRNA

AC004542.1 non.epi.lncRNA

AC004542.2 non.epi.lncRNA

AC004543.1 non.epi.lncRNA

AC004551.1 non.epi.lncRNA

AC004584.1 non.epi.lncRNA

AC004584.3 non.epi.lncRNA

AC004590.1 non.epi.lncRNA

AC004593.1 non.epi.lncRNA

AC004594.1 non.epi.lncRNA

AC004596.1 non.epi.lncRNA

AC004597.1 non.epi.lncRNA

AC004637.1 non.epi.lncRNA

AC004672.1 non.epi.lncRNA

AC004672.2 non.epi.lncRNA

AC004674.1 non.epi.lncRNA

AC004687.1 non.epi.lncRNA

AC004690.2 non.epi.lncRNA

AC004691.1 non.epi.lncRNA

AC004692.2 non.epi.lncRNA

AC004702.1 non.epi.lncRNA

AC004704.1 non.epi.lncRNA

AC004771.1 non.epi.lncRNA

AC004771.2 non.epi.lncRNA

AC004771.3 non.epi.lncRNA

AC004775.1 non.epi.lncRNA

AC004801.3 non.epi.lncRNA

AC004801.4 non.epi.lncRNA

AC004801.6 non.epi.lncRNA

AC004812.2 non.epi.lncRNA

AC004817.1 non.epi.lncRNA

AC004817.2 non.epi.lncRNA

AC004817.3 non.epi.lncRNA

AC004817.4 non.epi.lncRNA

AC004828.1 non.epi.lncRNA

AC004830.2 non.epi.lncRNA

AC004832.1 non.epi.lncRNA

AC004832.6 non.epi.lncRNA

AC004835.1 non.epi.lncRNA

AC004837.2 non.epi.lncRNA

AC004837.3 non.epi.lncRNA

AC004839.2 non.epi.lncRNA

AC004846.2 non.epi.lncRNA

AC004847.1 non.epi.lncRNA

AC004852.2 non.epi.lncRNA

AC004862.1 non.epi.lncRNA

AC004865.2 non.epi.lncRNA

AC004869.1 non.epi.lncRNA

AC004869.2 non.epi.lncRNA

AC004870.3 non.epi.lncRNA

AC004870.4 non.epi.lncRNA

AC004875.1 non.epi.lncRNA

AC004877.1 non.epi.lncRNA

AC004882.1 non.epi.lncRNA

AC004882.2 non.epi.lncRNA

AC004888.1 non.epi.lncRNA

AC004895.1 non.epi.lncRNA

AC004908.1 non.epi.lncRNA

AC004908.2 non.epi.lncRNA

AC004908.3 non.epi.lncRNA

AC004917.1 non.epi.lncRNA

AC004918.1 non.epi.lncRNA

AC004918.4 non.epi.lncRNA

AC004920.1 non.epi.lncRNA

AC004921.1 non.epi.lncRNA

AC004930.1 non.epi.lncRNA

AC004941.1 non.epi.lncRNA

AC004943.1 non.epi.lncRNA

AC004943.2 non.epi.lncRNA

AC004943.3 non.epi.lncRNA

AC004944.1 non.epi.lncRNA

AC004946.1 non.epi.lncRNA

AC004946.2 non.epi.lncRNA

AC004947.1 non.epi.lncRNA

AC004948.1 non.epi.lncRNA

AC004951.1 non.epi.lncRNA

AC004969.1 non.epi.lncRNA

AC004982.1 non.epi.lncRNA

AC004982.2 non.epi.lncRNA

AC004988.1 non.epi.lncRNA

AC004990.1 non.epi.lncRNA

AC005005.3 non.epi.lncRNA

AC005006.1 non.epi.lncRNA

AC005008.2 non.epi.lncRNA

AC005009.1 non.epi.lncRNA

AC005009.2 non.epi.lncRNA

AC005013.1 non.epi.lncRNA

AC005014.2 non.epi.lncRNA

AC005014.3 non.epi.lncRNA

AC005019.2 non.epi.lncRNA

AC005021.1 non.epi.lncRNA

AC005034.2 non.epi.lncRNA

AC005034.3 non.epi.lncRNA

AC005034.4 non.epi.lncRNA

AC005034.5 non.epi.lncRNA

AC005037.1 non.epi.lncRNA

AC005042.2 non.epi.lncRNA

AC005050.1 non.epi.lncRNA

AC005062.1 non.epi.lncRNA

AC005064.1 non.epi.lncRNA

AC005072.1 non.epi.lncRNA

AC005082.1 non.epi.lncRNA

AC005083.1 non.epi.lncRNA

AC005086.2 non.epi.lncRNA

AC005089.1 non.epi.lncRNA

AC005090.1 non.epi.lncRNA

AC005100.1 non.epi.lncRNA

AC005104.1 non.epi.lncRNA

AC005154.3 non.epi.lncRNA

AC005154.4 non.epi.lncRNA

AC005160.1 non.epi.lncRNA

AC005162.1 non.epi.lncRNA

AC005162.2 non.epi.lncRNA

AC005162.3 non.epi.lncRNA

AC005165.1 non.epi.lncRNA

AC005197.1 non.epi.lncRNA

AC005208.1 non.epi.lncRNA

AC005209.1 non.epi.lncRNA

AC005224.1 non.epi.lncRNA

AC005224.2 non.epi.lncRNA

AC005224.3 non.epi.lncRNA

AC005225.1 non.epi.lncRNA

AC005234.1 non.epi.lncRNA

AC005237.1 non.epi.lncRNA

AC005244.2 non.epi.lncRNA

AC005253.2 non.epi.lncRNA

AC005256.1 non.epi.lncRNA

AC005261.2 non.epi.lncRNA

AC005261.3 non.epi.lncRNA

AC005262.2 non.epi.lncRNA

AC005277.1 non.epi.lncRNA

AC005277.2 non.epi.lncRNA

AC005280.1 non.epi.lncRNA

AC005288.1 non.epi.lncRNA

AC005291.1 non.epi.lncRNA

AC005291.2 non.epi.lncRNA

AC005301.1 non.epi.lncRNA

AC005303.1 non.epi.lncRNA

AC005304.1 non.epi.lncRNA

AC005304.2 non.epi.lncRNA

AC005304.3 non.epi.lncRNA

AC005306.1 non.epi.lncRNA

AC005307.1 non.epi.lncRNA

AC005323.1 non.epi.lncRNA

AC005323.2 non.epi.lncRNA

AC005324.1 non.epi.lncRNA

AC005324.2 non.epi.lncRNA

AC005324.5 non.epi.lncRNA

AC005329.1 non.epi.lncRNA

AC005329.2 non.epi.lncRNA

AC005329.3 non.epi.lncRNA

AC005330.1 non.epi.lncRNA

AC005332.1 non.epi.lncRNA

AC005332.3 non.epi.lncRNA

AC005332.5 non.epi.lncRNA

AC005332.6 non.epi.lncRNA

AC005332.7 non.epi.lncRNA

AC005339.1 non.epi.lncRNA

AC005342.1 non.epi.lncRNA

AC005342.2 non.epi.lncRNA

AC005344.1 non.epi.lncRNA

AC005358.1 non.epi.lncRNA

AC005358.2 non.epi.lncRNA

AC005363.2 non.epi.lncRNA

AC005379.1 non.epi.lncRNA

AC005383.1 non.epi.lncRNA

AC005387.1 non.epi.lncRNA

AC005387.2 non.epi.lncRNA

AC005391.1 non.epi.lncRNA

AC005392.1 non.epi.lncRNA

AC005392.2 non.epi.lncRNA

AC005393.1 non.epi.lncRNA

AC005394.1 non.epi.lncRNA

AC005410.2 non.epi.lncRNA

AC005476.2 non.epi.lncRNA

AC005479.1 non.epi.lncRNA

AC005479.2 non.epi.lncRNA

AC005480.1 non.epi.lncRNA

AC005481.1 non.epi.lncRNA

AC005482.1 non.epi.lncRNA

AC005486.1 non.epi.lncRNA

AC005487.1 non.epi.lncRNA

AC005498.2 non.epi.lncRNA

AC005498.3 non.epi.lncRNA

AC005515.2 non.epi.lncRNA

AC005518.1 non.epi.lncRNA

AC005519.1 non.epi.lncRNA

AC005520.2 non.epi.lncRNA

AC005520.3 non.epi.lncRNA

AC005522.1 non.epi.lncRNA

AC005523.2 non.epi.lncRNA

AC005529.1 non.epi.lncRNA

AC005534.1 non.epi.lncRNA

AC005537.1 non.epi.lncRNA

AC005538.1 non.epi.lncRNA

AC005540.1 non.epi.lncRNA

AC005544.1 non.epi.lncRNA

AC005544.2 non.epi.lncRNA

AC005546.1 non.epi.lncRNA

AC005549.1 non.epi.lncRNA

AC005550.1 non.epi.lncRNA

AC005550.2 non.epi.lncRNA

AC005552.1 non.epi.lncRNA

AC005580.1 non.epi.lncRNA

AC005586.1 non.epi.lncRNA

AC005586.2 non.epi.lncRNA

AC005592.1 non.epi.lncRNA

AC005597.1 non.epi.lncRNA

AC005606.1 non.epi.lncRNA

AC005606.2 non.epi.lncRNA

AC005609.1 non.epi.lncRNA

AC005609.2 non.epi.lncRNA

AC005609.3 non.epi.lncRNA

AC005609.4 non.epi.lncRNA

AC005609.5 non.epi.lncRNA

AC005616.1 non.epi.lncRNA

AC005618.1 non.epi.lncRNA

AC005618.2 non.epi.lncRNA

AC005618.3 non.epi.lncRNA

AC005632.3 non.epi.lncRNA

AC005632.5 non.epi.lncRNA

AC005670.3 non.epi.lncRNA

AC005674.2 non.epi.lncRNA

AC005692.1 non.epi.lncRNA

AC005692.2 non.epi.lncRNA

AC005695.1 non.epi.lncRNA

AC005695.2 non.epi.lncRNA

AC005695.3 non.epi.lncRNA

AC005696.1 non.epi.lncRNA

AC005696.2 non.epi.lncRNA

AC005696.3 non.epi.lncRNA

AC005696.4 non.epi.lncRNA

AC005697.2 non.epi.lncRNA

AC005699.1 non.epi.lncRNA

AC005703.1 non.epi.lncRNA

AC005703.2 non.epi.lncRNA

AC005703.3 non.epi.lncRNA

AC005703.4 non.epi.lncRNA

AC005722.4 non.epi.lncRNA

AC005725.1 non.epi.lncRNA

AC005726.2 non.epi.lncRNA

AC005726.3 non.epi.lncRNA

AC005726.4 non.epi.lncRNA

AC005726.5 non.epi.lncRNA

AC005730.3 non.epi.lncRNA

AC005736.1 non.epi.lncRNA

AC005736.2 non.epi.lncRNA

AC005740.4 non.epi.lncRNA

AC005746.2 non.epi.lncRNA

AC005753.1 non.epi.lncRNA

AC005757.1 non.epi.lncRNA

AC005772.1 non.epi.lncRNA

AC005774.2 non.epi.lncRNA

AC005775.1 non.epi.lncRNA

AC005776.2 non.epi.lncRNA

AC005785.1 non.epi.lncRNA

AC005786.2 non.epi.lncRNA

AC005786.3 non.epi.lncRNA

AC005789.1 non.epi.lncRNA

AC005790.1 non.epi.lncRNA

AC005808.1 non.epi.lncRNA

AC005821.1 non.epi.lncRNA

AC005823.1 non.epi.lncRNA

AC005823.2 non.epi.lncRNA

AC005828.2 non.epi.lncRNA

AC005828.5 non.epi.lncRNA

AC005832.1 non.epi.lncRNA

AC005833.2 non.epi.lncRNA

AC005837.1 non.epi.lncRNA

AC005837.4 non.epi.lncRNA

AC005838.2 non.epi.lncRNA

AC005840.2 non.epi.lncRNA

AC005840.4 non.epi.lncRNA

AC005842.1 non.epi.lncRNA

AC005845.1 non.epi.lncRNA

AC005863.1 non.epi.lncRNA

AC005871.1 non.epi.lncRNA

AC005871.2 non.epi.lncRNA

AC005884.2 non.epi.lncRNA

AC005899.1 non.epi.lncRNA

AC005899.3 non.epi.lncRNA

AC005899.5 non.epi.lncRNA

AC005899.6 non.epi.lncRNA

AC005899.7 non.epi.lncRNA

AC005899.8 non.epi.lncRNA

AC005906.2 non.epi.lncRNA

AC005908.2 non.epi.lncRNA

AC005909.1 non.epi.lncRNA

AC005911.1 non.epi.lncRNA

AC005912.2 non.epi.lncRNA

AC005920.1 non.epi.lncRNA

AC005920.2 non.epi.lncRNA

AC005920.4 non.epi.lncRNA

AC005944.1 non.epi.lncRNA

AC005954.1 non.epi.lncRNA

AC005954.2 non.epi.lncRNA

AC005962.1 non.epi.lncRNA

AC005993.1 non.epi.lncRNA

AC005999.1 non.epi.lncRNA

AC006003.1 non.epi.lncRNA

AC006004.1 non.epi.lncRNA

AC006007.1 non.epi.lncRNA

AC006013.1 non.epi.lncRNA

AC006017.1 non.epi.lncRNA

AC006019.1 non.epi.lncRNA

AC006019.2 non.epi.lncRNA

AC006019.3 non.epi.lncRNA

AC006026.3 non.epi.lncRNA

AC006027.1 non.epi.lncRNA

AC006037.1 non.epi.lncRNA

AC006042.1 non.epi.lncRNA

AC006042.2 non.epi.lncRNA

AC006042.4 non.epi.lncRNA

AC006055.1 non.epi.lncRNA

AC006058.3 non.epi.lncRNA

AC006059.1 non.epi.lncRNA

AC006063.1 non.epi.lncRNA

AC006064.1 non.epi.lncRNA

AC006064.2 non.epi.lncRNA

AC006065.3 non.epi.lncRNA

AC006065.4 non.epi.lncRNA

AC006076.1 non.epi.lncRNA

AC006111.1 non.epi.lncRNA

AC006111.3 non.epi.lncRNA

AC006116.10 non.epi.lncRNA

AC006116.4 non.epi.lncRNA

AC006116.5 non.epi.lncRNA

AC006116.6 non.epi.lncRNA

AC006116.8 non.epi.lncRNA

AC006116.9 non.epi.lncRNA

AC006130.1 non.epi.lncRNA

AC006141.1 non.epi.lncRNA

AC006145.1 non.epi.lncRNA

AC006146.1 non.epi.lncRNA

AC006148.1 non.epi.lncRNA

AC006150.1 non.epi.lncRNA

AC006153.1 non.epi.lncRNA

AC006157.1 non.epi.lncRNA

AC006159.2 non.epi.lncRNA

AC006160.1 non.epi.lncRNA

AC006205.1 non.epi.lncRNA

AC006206.1 non.epi.lncRNA

AC006206.2 non.epi.lncRNA

AC006213.1 non.epi.lncRNA

AC006213.2 non.epi.lncRNA

AC006213.3 non.epi.lncRNA

AC006213.4 non.epi.lncRNA

AC006237.1 non.epi.lncRNA

AC006238.1 non.epi.lncRNA

AC006249.1 non.epi.lncRNA

AC006262.1 non.epi.lncRNA

AC006262.2 non.epi.lncRNA

AC006262.3 non.epi.lncRNA

AC006272.1 non.epi.lncRNA

AC006288.1 non.epi.lncRNA

AC006296.1 non.epi.lncRNA

AC006296.2 non.epi.lncRNA

AC006299.1 non.epi.lncRNA

AC006305.1 non.epi.lncRNA

AC006305.2 non.epi.lncRNA

AC006305.3 non.epi.lncRNA

AC006333.1 non.epi.lncRNA

AC006357.1 non.epi.lncRNA

AC006369.1 non.epi.lncRNA

AC006369.2 non.epi.lncRNA

AC006372.1 non.epi.lncRNA

AC006372.2 non.epi.lncRNA

AC006372.3 non.epi.lncRNA

AC006378.1 non.epi.lncRNA

AC006387.1 non.epi.lncRNA

AC006435.2 non.epi.lncRNA

AC006435.3 non.epi.lncRNA

AC006441.1 non.epi.lncRNA

AC006441.3 non.epi.lncRNA

AC006441.4 non.epi.lncRNA

AC006449.2 non.epi.lncRNA

AC006449.3 non.epi.lncRNA

AC006449.5 non.epi.lncRNA

AC006449.6 non.epi.lncRNA

AC006450.1 non.epi.lncRNA

AC006450.2 non.epi.lncRNA

AC006450.3 non.epi.lncRNA

AC006455.1 non.epi.lncRNA

AC006455.4 non.epi.lncRNA

AC006455.5 non.epi.lncRNA

AC006455.8 non.epi.lncRNA

AC006458.1 non.epi.lncRNA

AC006480.2 non.epi.lncRNA

AC006482.1 non.epi.lncRNA

AC006487.1 non.epi.lncRNA

AC006487.2 non.epi.lncRNA

AC006504.1 non.epi.lncRNA

AC006504.2 non.epi.lncRNA

AC006504.5 non.epi.lncRNA

AC006504.7 non.epi.lncRNA

AC006538.1 non.epi.lncRNA

AC006538.3 non.epi.lncRNA

AC006547.1 non.epi.lncRNA

AC006547.2 non.epi.lncRNA

AC006547.3 non.epi.lncRNA

AC006557.1 non.epi.lncRNA

AC006566.1 non.epi.lncRNA

AC006581.1 non.epi.lncRNA

AC006942.1 non.epi.lncRNA

AC006946.2 non.epi.lncRNA

AC006946.3 non.epi.lncRNA

AC006960.2 non.epi.lncRNA

AC006960.3 non.epi.lncRNA

AC006967.2 non.epi.lncRNA

AC006967.3 non.epi.lncRNA

AC006994.2 non.epi.lncRNA

AC007000.3 non.epi.lncRNA

AC007001.1 non.epi.lncRNA

AC007003.1 non.epi.lncRNA

AC007009.1 non.epi.lncRNA

AC007014.1 non.epi.lncRNA

AC007014.2 non.epi.lncRNA

AC007029.1 non.epi.lncRNA

AC007036.1 non.epi.lncRNA

AC007036.2 non.epi.lncRNA

AC007038.1 non.epi.lncRNA

AC007038.2 non.epi.lncRNA

AC007064.2 non.epi.lncRNA

AC007091.1 non.epi.lncRNA

AC007098.1 non.epi.lncRNA

AC007099.1 non.epi.lncRNA

AC007099.2 non.epi.lncRNA

AC007100.1 non.epi.lncRNA

AC007106.1 non.epi.lncRNA

AC007114.2 non.epi.lncRNA

AC007126.1 non.epi.lncRNA

AC007128.1 non.epi.lncRNA

AC007128.2 non.epi.lncRNA

AC007159.1 non.epi.lncRNA

AC007163.1 non.epi.lncRNA

AC007179.1 non.epi.lncRNA

AC007179.2 non.epi.lncRNA

AC007182.1 non.epi.lncRNA

AC007192.2 non.epi.lncRNA

AC007193.2 non.epi.lncRNA

AC007193.3 non.epi.lncRNA

AC007216.2 non.epi.lncRNA

AC007216.3 non.epi.lncRNA

AC007216.4 non.epi.lncRNA

AC007218.1 non.epi.lncRNA

AC007220.1 non.epi.lncRNA

AC007223.1 non.epi.lncRNA

AC007250.1 non.epi.lncRNA

AC007255.1 non.epi.lncRNA

AC007272.1 non.epi.lncRNA

AC007277.1 non.epi.lncRNA

AC007278.1 non.epi.lncRNA

AC007278.2 non.epi.lncRNA

AC007283.1 non.epi.lncRNA

AC007285.1 non.epi.lncRNA

AC007285.2 non.epi.lncRNA

AC007292.1 non.epi.lncRNA

AC007292.2 non.epi.lncRNA

AC007292.3 non.epi.lncRNA

AC007298.1 non.epi.lncRNA

AC007308.1 non.epi.lncRNA

AC007319.1 non.epi.lncRNA

AC007326.2 non.epi.lncRNA

AC007327.2 non.epi.lncRNA

AC007333.1 non.epi.lncRNA

AC007333.2 non.epi.lncRNA

AC007336.1 non.epi.lncRNA

AC007336.2 non.epi.lncRNA

AC007342.1 non.epi.lncRNA

AC007342.4 non.epi.lncRNA

AC007342.5 non.epi.lncRNA

AC007344.1 non.epi.lncRNA

AC007347.1 non.epi.lncRNA

AC007349.1 non.epi.lncRNA

AC007349.2 non.epi.lncRNA

AC007349.3 non.epi.lncRNA

AC007349.4 non.epi.lncRNA

AC007359.1 non.epi.lncRNA

AC007362.1 non.epi.lncRNA

AC007364.1 non.epi.lncRNA

AC007365.1 non.epi.lncRNA

AC007368.1 non.epi.lncRNA

AC007370.1 non.epi.lncRNA

AC007370.2 non.epi.lncRNA

AC007375.3 non.epi.lncRNA

AC007376.2 non.epi.lncRNA

AC007378.1 non.epi.lncRNA

AC007381.1 non.epi.lncRNA

AC007384.1 non.epi.lncRNA

AC007389.1 non.epi.lncRNA

AC007389.3 non.epi.lncRNA

AC007390.2 non.epi.lncRNA

AC007391.1 non.epi.lncRNA

AC007402.1 non.epi.lncRNA

AC007403.1 non.epi.lncRNA

AC007405.1 non.epi.lncRNA

AC007405.2 non.epi.lncRNA

AC007405.3 non.epi.lncRNA

AC007406.1 non.epi.lncRNA

AC007406.4 non.epi.lncRNA

AC007406.5 non.epi.lncRNA

AC007422.1 non.epi.lncRNA

AC007423.1 non.epi.lncRNA

AC007431.1 non.epi.lncRNA

AC007431.2 non.epi.lncRNA

AC007448.3 non.epi.lncRNA

AC007463.1 non.epi.lncRNA

AC007485.1 non.epi.lncRNA

AC007493.1 non.epi.lncRNA

AC007493.2 non.epi.lncRNA

AC007494.2 non.epi.lncRNA

AC007495.1 non.epi.lncRNA

AC007496.1 non.epi.lncRNA

AC007496.2 non.epi.lncRNA

AC007497.1 non.epi.lncRNA

AC007513.1 non.epi.lncRNA

AC007527.1 non.epi.lncRNA

AC007527.2 non.epi.lncRNA

AC007529.1 non.epi.lncRNA

AC007533.1 non.epi.lncRNA

AC007540.1 non.epi.lncRNA

AC007541.1 non.epi.lncRNA

AC007546.1 non.epi.lncRNA

AC007552.2 non.epi.lncRNA

AC007556.1 non.epi.lncRNA

AC007557.1 non.epi.lncRNA

AC007557.2 non.epi.lncRNA

AC007557.3 non.epi.lncRNA

AC007557.4 non.epi.lncRNA

AC007563.1 non.epi.lncRNA

AC007563.2 non.epi.lncRNA

AC007566.1 non.epi.lncRNA

AC007595.1 non.epi.lncRNA

AC007598.1 non.epi.lncRNA

AC007598.2 non.epi.lncRNA

AC007599.2 non.epi.lncRNA

AC007601.1 non.epi.lncRNA

AC007601.2 non.epi.lncRNA

AC007603.1 non.epi.lncRNA

AC007608.1 non.epi.lncRNA

AC007608.2 non.epi.lncRNA

AC007608.3 non.epi.lncRNA

AC007610.1 non.epi.lncRNA

AC007610.2 non.epi.lncRNA

AC007611.1 non.epi.lncRNA

AC007614.1 non.epi.lncRNA

AC007619.1 non.epi.lncRNA

AC007620.2 non.epi.lncRNA

AC007620.3 non.epi.lncRNA

AC007622.2 non.epi.lncRNA

AC007631.1 non.epi.lncRNA

AC007637.1 non.epi.lncRNA

AC007638.1 non.epi.lncRNA

AC007638.2 non.epi.lncRNA

AC007652.1 non.epi.lncRNA

AC007655.1 non.epi.lncRNA

AC007656.1 non.epi.lncRNA

AC007656.2 non.epi.lncRNA

AC007663.2 non.epi.lncRNA

AC007666.1 non.epi.lncRNA

AC007671.1 non.epi.lncRNA

AC007673.1 non.epi.lncRNA

AC007679.1 non.epi.lncRNA

AC007681.1 non.epi.lncRNA

AC007684.2 non.epi.lncRNA

AC007686.2 non.epi.lncRNA

AC007686.3 non.epi.lncRNA

AC007687.1 non.epi.lncRNA

AC007688.3 non.epi.lncRNA

AC007728.1 non.epi.lncRNA

AC007728.2 non.epi.lncRNA

AC007728.3 non.epi.lncRNA

AC007731.2 non.epi.lncRNA

AC007744.1 non.epi.lncRNA

AC007749.1 non.epi.lncRNA

AC007750.1 non.epi.lncRNA

AC007780.1 non.epi.lncRNA

AC007785.1 non.epi.lncRNA

AC007785.3 non.epi.lncRNA

AC007786.1 non.epi.lncRNA

AC007786.2 non.epi.lncRNA

AC007786.3 non.epi.lncRNA

AC007787.1 non.epi.lncRNA

AC007795.1 non.epi.lncRNA

AC007823.1 non.epi.lncRNA

AC007848.1 non.epi.lncRNA

AC007848.2 non.epi.lncRNA

AC007849.1 non.epi.lncRNA

AC007861.1 non.epi.lncRNA

AC007861.2 non.epi.lncRNA

AC007876.1 non.epi.lncRNA

AC007878.1 non.epi.lncRNA

AC007879.1 non.epi.lncRNA

AC007879.2 non.epi.lncRNA

AC007879.3 non.epi.lncRNA

AC007879.4 non.epi.lncRNA

AC007881.2 non.epi.lncRNA

AC007881.3 non.epi.lncRNA

AC007906.1 non.epi.lncRNA

AC007907.1 non.epi.lncRNA

AC007920.1 non.epi.lncRNA

AC007922.1 non.epi.lncRNA

AC007922.2 non.epi.lncRNA

AC007922.3 non.epi.lncRNA

AC007923.1 non.epi.lncRNA

AC007923.4 non.epi.lncRNA

AC007938.1 non.epi.lncRNA

AC007938.2 non.epi.lncRNA

AC007938.3 non.epi.lncRNA

AC007948.1 non.epi.lncRNA

AC007950.2 non.epi.lncRNA

AC007952.1 non.epi.lncRNA

AC007952.2 non.epi.lncRNA

AC007952.3 non.epi.lncRNA

AC007952.5 non.epi.lncRNA

AC007952.6 non.epi.lncRNA

AC007952.7 non.epi.lncRNA

AC007952.8 non.epi.lncRNA

AC007952.9 non.epi.lncRNA

AC007953.1 non.epi.lncRNA

AC007953.2 non.epi.lncRNA

AC007991.2 non.epi.lncRNA

AC007991.3 non.epi.lncRNA

AC007991.4 non.epi.lncRNA

AC007993.2 non.epi.lncRNA

AC007996.1 non.epi.lncRNA

AC007998.4 non.epi.lncRNA

AC008011.2 non.epi.lncRNA

AC008014.1 non.epi.lncRNA

AC008026.3 non.epi.lncRNA

AC008035.1 non.epi.lncRNA

AC008040.1 non.epi.lncRNA

AC008050.1 non.epi.lncRNA

AC008056.1 non.epi.lncRNA

AC008056.2 non.epi.lncRNA

AC008060.1 non.epi.lncRNA

AC008060.2 non.epi.lncRNA

AC008060.3 non.epi.lncRNA

AC008060.4 non.epi.lncRNA

AC008063.1 non.epi.lncRNA

AC008063.2 non.epi.lncRNA

AC008067.1 non.epi.lncRNA

AC008073.1 non.epi.lncRNA

AC008073.2 non.epi.lncRNA

AC008079.1 non.epi.lncRNA

AC008080.1 non.epi.lncRNA

AC008080.2 non.epi.lncRNA

AC008080.3 non.epi.lncRNA

AC008080.4 non.epi.lncRNA

AC008083.2 non.epi.lncRNA

AC008083.3 non.epi.lncRNA

AC008083.4 non.epi.lncRNA

AC008088.1 non.epi.lncRNA

AC008103.1 non.epi.lncRNA

AC008105.1 non.epi.lncRNA

AC008114.1 non.epi.lncRNA

AC008115.1 non.epi.lncRNA

AC008115.3 non.epi.lncRNA

AC008121.2 non.epi.lncRNA

AC008121.3 non.epi.lncRNA

AC008124.1 non.epi.lncRNA

AC008125.1 non.epi.lncRNA

AC008127.1 non.epi.lncRNA

AC008133.1 non.epi.lncRNA

AC008147.2 non.epi.lncRNA

AC008149.1 non.epi.lncRNA

AC008154.2 non.epi.lncRNA

AC008164.1 non.epi.lncRNA

AC008175.1 non.epi.lncRNA

AC008243.1 non.epi.lncRNA

AC008250.1 non.epi.lncRNA

AC008250.2 non.epi.lncRNA

AC008264.2 non.epi.lncRNA

AC008267.5 non.epi.lncRNA

AC008268.1 non.epi.lncRNA

AC008269.1 non.epi.lncRNA

AC008269.2 non.epi.lncRNA

AC008277.1 non.epi.lncRNA

AC008278.1 non.epi.lncRNA

AC008278.2 non.epi.lncRNA

AC008280.3 non.epi.lncRNA

AC008378.1 non.epi.lncRNA

AC008395.1 non.epi.lncRNA

AC008406.1 non.epi.lncRNA

AC008406.2 non.epi.lncRNA

AC008413.2 non.epi.lncRNA

AC008415.1 non.epi.lncRNA

AC008429.3 non.epi.lncRNA

AC008434.1 non.epi.lncRNA

AC008443.1 non.epi.lncRNA

AC008443.3 non.epi.lncRNA

AC008443.5 non.epi.lncRNA

AC008456.1 non.epi.lncRNA

AC008467.1 non.epi.lncRNA

AC008474.1 non.epi.lncRNA

AC008494.2 non.epi.lncRNA

AC008496.2 non.epi.lncRNA

AC008507.2 non.epi.lncRNA

AC008514.1 non.epi.lncRNA

AC008517.1 non.epi.lncRNA

AC008522.1 non.epi.lncRNA

AC008525.1 non.epi.lncRNA

AC008525.2 non.epi.lncRNA

AC008534.1 non.epi.lncRNA

AC008537.2 non.epi.lncRNA

AC008539.1 non.epi.lncRNA

AC008543.1 non.epi.lncRNA

AC008543.4 non.epi.lncRNA

AC008549.1 non.epi.lncRNA

AC008549.2 non.epi.lncRNA

AC008554.1 non.epi.lncRNA

AC008555.1 non.epi.lncRNA

AC008555.5 non.epi.lncRNA

AC008567.2 non.epi.lncRNA

AC008568.1 non.epi.lncRNA

AC008569.1 non.epi.lncRNA

AC008569.2 non.epi.lncRNA

AC008572.1 non.epi.lncRNA

AC008574.1 non.epi.lncRNA

AC008581.1 non.epi.lncRNA

AC008588.1 non.epi.lncRNA

AC008591.1 non.epi.lncRNA

AC008592.1 non.epi.lncRNA

AC008592.2 non.epi.lncRNA

AC008592.4 non.epi.lncRNA

AC008592.5 non.epi.lncRNA

AC008601.1 non.epi.lncRNA

AC008608.2 non.epi.lncRNA

AC008609.1 non.epi.lncRNA

AC008619.1 non.epi.lncRNA

AC008622.2 non.epi.lncRNA

AC008627.1 non.epi.lncRNA

AC008629.1 non.epi.lncRNA

AC008632.1 non.epi.lncRNA

AC008635.1 non.epi.lncRNA

AC008641.1 non.epi.lncRNA

AC008649.1 non.epi.lncRNA

AC008652.1 non.epi.lncRNA

AC008655.1 non.epi.lncRNA

AC008655.2 non.epi.lncRNA

AC008659.1 non.epi.lncRNA

AC008662.1 non.epi.lncRNA

AC008662.2 non.epi.lncRNA

AC008663.1 non.epi.lncRNA

AC008663.2 non.epi.lncRNA

AC008663.3 non.epi.lncRNA

AC008667.1 non.epi.lncRNA

AC008667.2 non.epi.lncRNA

AC008667.3 non.epi.lncRNA

AC008667.4 non.epi.lncRNA

AC008669.1 non.epi.lncRNA

AC008676.1 non.epi.lncRNA

AC008680.1 non.epi.lncRNA

AC008686.1 non.epi.lncRNA

AC008687.2 non.epi.lncRNA

AC008687.3 non.epi.lncRNA

AC008691.1 non.epi.lncRNA

AC008700.1 non.epi.lncRNA

AC008705.2 non.epi.lncRNA

AC008708.1 non.epi.lncRNA

AC008708.2 non.epi.lncRNA

AC008728.1 non.epi.lncRNA

AC008731.1 non.epi.lncRNA

AC008734.1 non.epi.lncRNA

AC008735.2 non.epi.lncRNA

AC008735.4 non.epi.lncRNA

AC008736.2 non.epi.lncRNA

AC008737.1 non.epi.lncRNA

AC008737.3 non.epi.lncRNA

AC008738.1 non.epi.lncRNA

AC008738.2 non.epi.lncRNA

AC008738.4 non.epi.lncRNA

AC008738.6 non.epi.lncRNA

AC008738.7 non.epi.lncRNA

AC008739.1 non.epi.lncRNA

AC008741.1 non.epi.lncRNA

AC008742.1 non.epi.lncRNA

AC008743.1 non.epi.lncRNA

AC008747.1 non.epi.lncRNA

AC008750.1 non.epi.lncRNA

AC008750.2 non.epi.lncRNA

AC008750.3 non.epi.lncRNA

AC008750.4 non.epi.lncRNA

AC008750.5 non.epi.lncRNA

AC008750.7 non.epi.lncRNA

AC008752.2 non.epi.lncRNA

AC008753.2 non.epi.lncRNA

AC008753.3 non.epi.lncRNA

AC008758.4 non.epi.lncRNA

AC008759.2 non.epi.lncRNA

AC008759.3 non.epi.lncRNA

AC008760.1 non.epi.lncRNA

AC008760.2 non.epi.lncRNA

AC008761.1 non.epi.lncRNA

AC008761.2 non.epi.lncRNA

AC008763.1 non.epi.lncRNA

AC008764.2 non.epi.lncRNA

AC008764.3 non.epi.lncRNA

AC008764.5 non.epi.lncRNA

AC008764.6 non.epi.lncRNA

AC008764.7 non.epi.lncRNA

AC008764.8 non.epi.lncRNA

AC008770.3 non.epi.lncRNA

AC008771.1 non.epi.lncRNA

AC008780.1 non.epi.lncRNA

AC008781.2 non.epi.lncRNA

AC008781.3 non.epi.lncRNA

AC008794.1 non.epi.lncRNA

AC008799.2 non.epi.lncRNA

AC008805.2 non.epi.lncRNA

AC008808.1 non.epi.lncRNA

AC008808.2 non.epi.lncRNA

AC008825.1 non.epi.lncRNA

AC008840.1 non.epi.lncRNA

AC008852.1 non.epi.lncRNA

AC008869.1 non.epi.lncRNA

AC008870.1 non.epi.lncRNA

AC008870.2 non.epi.lncRNA

AC008870.4 non.epi.lncRNA

AC008871.1 non.epi.lncRNA

AC008875.1 non.epi.lncRNA

AC008877.1 non.epi.lncRNA

AC008878.4 non.epi.lncRNA

AC008883.2 non.epi.lncRNA

AC008885.2 non.epi.lncRNA

AC008892.1 non.epi.lncRNA

AC008894.1 non.epi.lncRNA

AC008894.2 non.epi.lncRNA

AC008897.2 non.epi.lncRNA

AC008897.3 non.epi.lncRNA

AC008906.1 non.epi.lncRNA

AC008906.2 non.epi.lncRNA

AC008914.1 non.epi.lncRNA

AC008915.1 non.epi.lncRNA

AC008937.2 non.epi.lncRNA

AC008938.1 non.epi.lncRNA

AC008940.1 non.epi.lncRNA

AC008945.1 non.epi.lncRNA

AC008946.1 non.epi.lncRNA

AC008948.1 non.epi.lncRNA

AC008957.2 non.epi.lncRNA

AC008966.2 non.epi.lncRNA

AC008969.1 non.epi.lncRNA

AC008972.2 non.epi.lncRNA

AC008991.1 non.epi.lncRNA

AC008992.1 non.epi.lncRNA

AC009019.1 non.epi.lncRNA

AC009021.1 non.epi.lncRNA

AC009021.2 non.epi.lncRNA

AC009022.1 non.epi.lncRNA

AC009032.1 non.epi.lncRNA

AC009034.1 non.epi.lncRNA

AC009035.1 non.epi.lncRNA

AC009039.1 non.epi.lncRNA

AC009041.1 non.epi.lncRNA

AC009041.2 non.epi.lncRNA

AC009041.3 non.epi.lncRNA

AC009052.1 non.epi.lncRNA

AC009053.2 non.epi.lncRNA

AC009053.3 non.epi.lncRNA

AC009054.1 non.epi.lncRNA

AC009054.2 non.epi.lncRNA

AC009055.1 non.epi.lncRNA

AC009055.2 non.epi.lncRNA

AC009060.1 non.epi.lncRNA

AC009061.1 non.epi.lncRNA

AC009061.2 non.epi.lncRNA

AC009063.1 non.epi.lncRNA

AC009063.2 non.epi.lncRNA

AC009063.3 non.epi.lncRNA

AC009065.2 non.epi.lncRNA

AC009065.3 non.epi.lncRNA

AC009065.4 non.epi.lncRNA

AC009065.6 non.epi.lncRNA

AC009065.8 non.epi.lncRNA

AC009075.1 non.epi.lncRNA

AC009081.1 non.epi.lncRNA

AC009081.2 non.epi.lncRNA

AC009084.1 non.epi.lncRNA

AC009087.1 non.epi.lncRNA

AC009088.2 non.epi.lncRNA

AC009088.3 non.epi.lncRNA

AC009090.1 non.epi.lncRNA

AC009093.1 non.epi.lncRNA

AC009093.2 non.epi.lncRNA

AC009093.4 non.epi.lncRNA

AC009093.5 non.epi.lncRNA

AC009095.1 non.epi.lncRNA

AC009097.2 non.epi.lncRNA

AC009097.3 non.epi.lncRNA

AC009097.4 non.epi.lncRNA

AC009102.1 non.epi.lncRNA

AC009102.2 non.epi.lncRNA

AC009107.2 non.epi.lncRNA

AC009108.2 non.epi.lncRNA

AC009108.3 non.epi.lncRNA

AC009110.1 non.epi.lncRNA

AC009113.1 non.epi.lncRNA

AC009113.2 non.epi.lncRNA

AC009117.2 non.epi.lncRNA

AC009118.1 non.epi.lncRNA

AC009118.2 non.epi.lncRNA

AC009118.3 non.epi.lncRNA

AC009119.1 non.epi.lncRNA

AC009119.2 non.epi.lncRNA

AC009120.2 non.epi.lncRNA

AC009120.3 non.epi.lncRNA

AC009120.4 non.epi.lncRNA

AC009120.5 non.epi.lncRNA

AC009121.2 non.epi.lncRNA

AC009123.1 non.epi.lncRNA

AC009126.1 non.epi.lncRNA

AC009127.1 non.epi.lncRNA

AC009133.2 non.epi.lncRNA

AC009133.3 non.epi.lncRNA

AC009134.1 non.epi.lncRNA

AC009135.1 non.epi.lncRNA

AC009137.1 non.epi.lncRNA

AC009137.2 non.epi.lncRNA

AC009139.1 non.epi.lncRNA

AC009139.2 non.epi.lncRNA

AC009142.1 non.epi.lncRNA

AC009145.1 non.epi.lncRNA

AC009145.2 non.epi.lncRNA

AC009145.3 non.epi.lncRNA

AC009148.1 non.epi.lncRNA

AC009152.1 non.epi.lncRNA

AC009154.1 non.epi.lncRNA

AC009154.2 non.epi.lncRNA

AC009158.1 non.epi.lncRNA

AC009159.1 non.epi.lncRNA

AC009159.2 non.epi.lncRNA

AC009163.1 non.epi.lncRNA

AC009163.4 non.epi.lncRNA

AC009163.6 non.epi.lncRNA

AC009163.7 non.epi.lncRNA

AC009166.1 non.epi.lncRNA

AC009167.1 non.epi.lncRNA

AC009185.1 non.epi.lncRNA

AC009226.1 non.epi.lncRNA

AC009227.1 non.epi.lncRNA

AC009227.2 non.epi.lncRNA

AC009228.1 non.epi.lncRNA

AC009229.1 non.epi.lncRNA

AC009229.2 non.epi.lncRNA

AC009229.3 non.epi.lncRNA

AC009237.14 non.epi.lncRNA

AC009237.15 non.epi.lncRNA

AC009248.2 non.epi.lncRNA

AC009262.1 non.epi.lncRNA

AC009264.1 non.epi.lncRNA

AC009268.2 non.epi.lncRNA

AC009269.3 non.epi.lncRNA

AC009269.4 non.epi.lncRNA

AC009271.1 non.epi.lncRNA

AC009275.1 non.epi.lncRNA

AC009276.1 non.epi.lncRNA

AC009283.1 non.epi.lncRNA

AC009292.1 non.epi.lncRNA

AC009292.2 non.epi.lncRNA

AC009299.3 non.epi.lncRNA

AC009303.2 non.epi.lncRNA

AC009303.3 non.epi.lncRNA

AC009305.1 non.epi.lncRNA

AC009309.1 non.epi.lncRNA

AC009312.1 non.epi.lncRNA

AC009318.1 non.epi.lncRNA

AC009318.2 non.epi.lncRNA

AC009318.3 non.epi.lncRNA

AC009318.4 non.epi.lncRNA

AC009320.1 non.epi.lncRNA

AC009336.1 non.epi.lncRNA

AC009365.1 non.epi.lncRNA

AC009365.3 non.epi.lncRNA

AC009365.4 non.epi.lncRNA

AC009387.1 non.epi.lncRNA

AC009396.1 non.epi.lncRNA

AC009396.2 non.epi.lncRNA

AC009396.3 non.epi.lncRNA

AC009403.1 non.epi.lncRNA

AC009404.1 non.epi.lncRNA

AC009407.1 non.epi.lncRNA

AC009411.1 non.epi.lncRNA

AC009411.2 non.epi.lncRNA

AC009432.1 non.epi.lncRNA

AC009435.1 non.epi.lncRNA

AC009446.1 non.epi.lncRNA

AC009464.1 non.epi.lncRNA

AC009468.1 non.epi.lncRNA

AC009468.2 non.epi.lncRNA

AC009478.1 non.epi.lncRNA

AC009480.1 non.epi.lncRNA

AC009486.1 non.epi.lncRNA

AC009487.1 non.epi.lncRNA

AC009487.3 non.epi.lncRNA

AC009495.1 non.epi.lncRNA

AC009495.2 non.epi.lncRNA

AC009495.3 non.epi.lncRNA

AC009498.1 non.epi.lncRNA

AC009505.1 non.epi.lncRNA

AC009506.1 non.epi.lncRNA

AC009509.1 non.epi.lncRNA

AC009509.4 non.epi.lncRNA

AC009511.1 non.epi.lncRNA

AC009511.2 non.epi.lncRNA

AC009518.1 non.epi.lncRNA

AC009522.1 non.epi.lncRNA

AC009531.1 non.epi.lncRNA

AC009542.1 non.epi.lncRNA

AC009549.1 non.epi.lncRNA

AC009554.1 non.epi.lncRNA

AC009558.1 non.epi.lncRNA

AC009558.2 non.epi.lncRNA

AC009560.1 non.epi.lncRNA

AC009562.1 non.epi.lncRNA

AC009563.1 non.epi.lncRNA

AC009567.1 non.epi.lncRNA

AC009570.1 non.epi.lncRNA

AC009597.1 non.epi.lncRNA

AC009623.1 non.epi.lncRNA

AC009623.2 non.epi.lncRNA

AC009630.1 non.epi.lncRNA

AC009630.2 non.epi.lncRNA

AC009630.3 non.epi.lncRNA

AC009646.2 non.epi.lncRNA

AC009652.1 non.epi.lncRNA

AC009654.1 non.epi.lncRNA

AC009656.1 non.epi.lncRNA

AC009686.2 non.epi.lncRNA

AC009690.2 non.epi.lncRNA

AC009704.1 non.epi.lncRNA

AC009716.1 non.epi.lncRNA

AC009720.1 non.epi.lncRNA

AC009743.1 non.epi.lncRNA

AC009754.1 non.epi.lncRNA

AC009779.5 non.epi.lncRNA

AC009803.1 non.epi.lncRNA

AC009803.2 non.epi.lncRNA

AC009812.1 non.epi.lncRNA

AC009812.3 non.epi.lncRNA

AC009812.4 non.epi.lncRNA

AC009831.3 non.epi.lncRNA

AC009852.1 non.epi.lncRNA

AC009878.1 non.epi.lncRNA

AC009884.1 non.epi.lncRNA

AC009902.3 non.epi.lncRNA

AC009930.1 non.epi.lncRNA

AC009948.2 non.epi.lncRNA

AC009948.3 non.epi.lncRNA

AC009950.1 non.epi.lncRNA

AC009955.1 non.epi.lncRNA

AC009955.2 non.epi.lncRNA

AC009955.3 non.epi.lncRNA

AC009955.4 non.epi.lncRNA

AC009965.1 non.epi.lncRNA

AC009970.1 non.epi.lncRNA

AC009974.1 non.epi.lncRNA

AC009987.1 non.epi.lncRNA

AC009988.1 non.epi.lncRNA

AC010082.1 non.epi.lncRNA

AC010096.1 non.epi.lncRNA

AC010105.1 non.epi.lncRNA

AC010132.1 non.epi.lncRNA

AC010132.4 non.epi.lncRNA

AC010136.1 non.epi.lncRNA

AC010139.1 non.epi.lncRNA

AC010145.1 non.epi.lncRNA

AC010148.1 non.epi.lncRNA

AC010149.1 non.epi.lncRNA

AC010157.1 non.epi.lncRNA

AC010163.1 non.epi.lncRNA

AC010163.2 non.epi.lncRNA

AC010168.2 non.epi.lncRNA

AC010173.1 non.epi.lncRNA

AC010177.1 non.epi.lncRNA

AC010181.1 non.epi.lncRNA

AC010181.2 non.epi.lncRNA

AC010183.1 non.epi.lncRNA

AC010183.2 non.epi.lncRNA

AC010185.1 non.epi.lncRNA

AC010186.1 non.epi.lncRNA

AC010186.3 non.epi.lncRNA

AC010186.4 non.epi.lncRNA

AC010196.1 non.epi.lncRNA

AC010197.1 non.epi.lncRNA

AC010201.1 non.epi.lncRNA

AC010201.2 non.epi.lncRNA

AC010205.1 non.epi.lncRNA

AC010210.1 non.epi.lncRNA

AC010226.1 non.epi.lncRNA

AC010230.1 non.epi.lncRNA

AC010240.3 non.epi.lncRNA

AC010245.1 non.epi.lncRNA

AC010245.2 non.epi.lncRNA

AC010247.1 non.epi.lncRNA

AC010247.2 non.epi.lncRNA

AC010255.1 non.epi.lncRNA

AC010255.2 non.epi.lncRNA

AC010260.1 non.epi.lncRNA

AC010261.1 non.epi.lncRNA

AC010261.2 non.epi.lncRNA

AC010266.1 non.epi.lncRNA

AC010266.2 non.epi.lncRNA

AC010267.1 non.epi.lncRNA

AC010271.1 non.epi.lncRNA

AC010271.2 non.epi.lncRNA

AC010273.2 non.epi.lncRNA

AC010273.3 non.epi.lncRNA

AC010275.1 non.epi.lncRNA

AC010280.1 non.epi.lncRNA

AC010280.3 non.epi.lncRNA

AC010285.1 non.epi.lncRNA

AC010307.3 non.epi.lncRNA

AC010307.4 non.epi.lncRNA

AC010319.5 non.epi.lncRNA

AC010320.1 non.epi.lncRNA

AC010320.2 non.epi.lncRNA

AC010320.3 non.epi.lncRNA

AC010320.4 non.epi.lncRNA

AC010325.2 non.epi.lncRNA

AC010326.3 non.epi.lncRNA

AC010327.4 non.epi.lncRNA

AC010327.5 non.epi.lncRNA

AC010328.1 non.epi.lncRNA

AC010328.3 non.epi.lncRNA

AC010329.1 non.epi.lncRNA

AC010331.1 non.epi.lncRNA

AC010333.1 non.epi.lncRNA

AC010333.2 non.epi.lncRNA

AC010333.3 non.epi.lncRNA

AC010335.1 non.epi.lncRNA

AC010336.1 non.epi.lncRNA

AC010336.2 non.epi.lncRNA

AC010336.3 non.epi.lncRNA

AC010336.4 non.epi.lncRNA

AC010336.6 non.epi.lncRNA

AC010343.3 non.epi.lncRNA

AC010359.2 non.epi.lncRNA

AC010387.1 non.epi.lncRNA

AC010395.1 non.epi.lncRNA

AC010406.1 non.epi.lncRNA

AC010420.1 non.epi.lncRNA

AC010422.2 non.epi.lncRNA

AC010422.4 non.epi.lncRNA

AC010424.2 non.epi.lncRNA

AC010425.1 non.epi.lncRNA

AC010445.1 non.epi.lncRNA

AC010451.1 non.epi.lncRNA

AC010451.2 non.epi.lncRNA

AC010451.3 non.epi.lncRNA

AC010457.1 non.epi.lncRNA

AC010463.2 non.epi.lncRNA

AC010463.3 non.epi.lncRNA

AC010468.2 non.epi.lncRNA

AC010478.1 non.epi.lncRNA

AC010485.1 non.epi.lncRNA

AC010486.1 non.epi.lncRNA

AC010486.2 non.epi.lncRNA

AC010486.3 non.epi.lncRNA

AC010491.1 non.epi.lncRNA

AC010501.2 non.epi.lncRNA

AC010503.1 non.epi.lncRNA

AC010503.2 non.epi.lncRNA

AC010503.4 non.epi.lncRNA

AC010511.1 non.epi.lncRNA

AC010519.1 non.epi.lncRNA

AC010524.1 non.epi.lncRNA

AC010525.1 non.epi.lncRNA

AC010528.1 non.epi.lncRNA

AC010530.1 non.epi.lncRNA

AC010531.3 non.epi.lncRNA

AC010531.4 non.epi.lncRNA

AC010531.5 non.epi.lncRNA

AC010531.6 non.epi.lncRNA

AC010531.7 non.epi.lncRNA

AC010536.1 non.epi.lncRNA

AC010536.2 non.epi.lncRNA

AC010536.3 non.epi.lncRNA

AC010538.1 non.epi.lncRNA

AC010542.1 non.epi.lncRNA

AC010542.2 non.epi.lncRNA

AC010542.4 non.epi.lncRNA

AC010542.5 non.epi.lncRNA

AC010547.1 non.epi.lncRNA

AC010547.2 non.epi.lncRNA

AC010547.3 non.epi.lncRNA

AC010551.1 non.epi.lncRNA

AC010551.2 non.epi.lncRNA

AC010551.3 non.epi.lncRNA

AC010595.1 non.epi.lncRNA

AC010601.1 non.epi.lncRNA

AC010605.1 non.epi.lncRNA

AC010608.1 non.epi.lncRNA

AC010609.1 non.epi.lncRNA

AC010618.2 non.epi.lncRNA

AC010618.3 non.epi.lncRNA

AC010619.2 non.epi.lncRNA

AC010624.1 non.epi.lncRNA

AC010624.2 non.epi.lncRNA

AC010624.3 non.epi.lncRNA

AC010632.1 non.epi.lncRNA

AC010632.2 non.epi.lncRNA

AC010634.1 non.epi.lncRNA

AC010636.1 non.epi.lncRNA

AC010636.2 non.epi.lncRNA

AC010638.1 non.epi.lncRNA

AC010641.1 non.epi.lncRNA

AC010643.1 non.epi.lncRNA

AC010649.1 non.epi.lncRNA

AC010654.1 non.epi.lncRNA

AC010655.2 non.epi.lncRNA

AC010655.4 non.epi.lncRNA

AC010680.2 non.epi.lncRNA

AC010680.3 non.epi.lncRNA

AC010680.4 non.epi.lncRNA

AC010680.5 non.epi.lncRNA

AC010719.1 non.epi.lncRNA

AC010722.1 non.epi.lncRNA

AC010723.1 non.epi.lncRNA

AC010729.1 non.epi.lncRNA

AC010729.2 non.epi.lncRNA

AC010731.2 non.epi.lncRNA

AC010731.3 non.epi.lncRNA

AC010733.2 non.epi.lncRNA

AC010735.1 non.epi.lncRNA

AC010737.1 non.epi.lncRNA

AC010745.1 non.epi.lncRNA

AC010745.2 non.epi.lncRNA

AC010745.3 non.epi.lncRNA

AC010745.4 non.epi.lncRNA

AC010746.2 non.epi.lncRNA

AC010754.1 non.epi.lncRNA

AC010760.1 non.epi.lncRNA

AC010761.1 non.epi.lncRNA

AC010761.4 non.epi.lncRNA

AC010761.5 non.epi.lncRNA

AC010768.1 non.epi.lncRNA

AC010768.2 non.epi.lncRNA

AC010768.4 non.epi.lncRNA

AC010776.1 non.epi.lncRNA

AC010776.2 non.epi.lncRNA

AC010776.3 non.epi.lncRNA

AC010809.1 non.epi.lncRNA

AC010809.2 non.epi.lncRNA

AC010809.3 non.epi.lncRNA

AC010834.2 non.epi.lncRNA

AC010834.3 non.epi.lncRNA

AC010857.1 non.epi.lncRNA

AC010864.1 non.epi.lncRNA

AC010880.1 non.epi.lncRNA

AC010884.1 non.epi.lncRNA

AC010889.1 non.epi.lncRNA

AC010891.1 non.epi.lncRNA

AC010894.2 non.epi.lncRNA

AC010894.3 non.epi.lncRNA

AC010894.4 non.epi.lncRNA

AC010904.2 non.epi.lncRNA

AC010907.1 non.epi.lncRNA

AC010907.2 non.epi.lncRNA

AC010931.2 non.epi.lncRNA

AC010967.1 non.epi.lncRNA

AC010967.2 non.epi.lncRNA

AC010969.1 non.epi.lncRNA

AC010969.2 non.epi.lncRNA

AC010973.1 non.epi.lncRNA

AC010973.2 non.epi.lncRNA

AC010974.1 non.epi.lncRNA

AC010975.1 non.epi.lncRNA

AC010976.1 non.epi.lncRNA

AC010976.2 non.epi.lncRNA

AC010978.1 non.epi.lncRNA

AC010980.1 non.epi.lncRNA

AC010982.1 non.epi.lncRNA

AC010983.1 non.epi.lncRNA

AC010991.1 non.epi.lncRNA

AC010997.2 non.epi.lncRNA

AC010997.3 non.epi.lncRNA

AC010998.1 non.epi.lncRNA

AC010998.2 non.epi.lncRNA

AC010998.3 non.epi.lncRNA

AC010999.1 non.epi.lncRNA

AC011005.4 non.epi.lncRNA

AC011008.1 non.epi.lncRNA

AC011095.1 non.epi.lncRNA

AC011120.1 non.epi.lncRNA

AC011124.1 non.epi.lncRNA

AC011124.2 non.epi.lncRNA

AC011131.1 non.epi.lncRNA

AC011193.1 non.epi.lncRNA

AC011196.1 non.epi.lncRNA

AC011228.1 non.epi.lncRNA

AC011239.1 non.epi.lncRNA

AC011243.1 non.epi.lncRNA

AC011284.1 non.epi.lncRNA

AC011287.1 non.epi.lncRNA

AC011287.2 non.epi.lncRNA

AC011290.1 non.epi.lncRNA

AC011294.1 non.epi.lncRNA

AC011297.1 non.epi.lncRNA

AC011298.1 non.epi.lncRNA

AC011306.1 non.epi.lncRNA

AC011313.1 non.epi.lncRNA

AC011330.2 non.epi.lncRNA

AC011333.1 non.epi.lncRNA

AC011337.1 non.epi.lncRNA

AC011346.1 non.epi.lncRNA

AC011352.1 non.epi.lncRNA

AC011352.3 non.epi.lncRNA

AC011362.1 non.epi.lncRNA

AC011363.1 non.epi.lncRNA

AC011365.1 non.epi.lncRNA

AC011365.2 non.epi.lncRNA

AC011369.1 non.epi.lncRNA

AC011373.1 non.epi.lncRNA

AC011374.1 non.epi.lncRNA

AC011374.2 non.epi.lncRNA

AC011379.1 non.epi.lncRNA

AC011379.2 non.epi.lncRNA

AC011383.1 non.epi.lncRNA

AC011389.1 non.epi.lncRNA

AC011396.1 non.epi.lncRNA

AC011396.2 non.epi.lncRNA

AC011396.3 non.epi.lncRNA

AC011405.1 non.epi.lncRNA

AC011410.1 non.epi.lncRNA

AC011442.1 non.epi.lncRNA

AC011444.1 non.epi.lncRNA

AC011444.3 non.epi.lncRNA

AC011445.2 non.epi.lncRNA

AC011446.1 non.epi.lncRNA

AC011447.3 non.epi.lncRNA

AC011450.1 non.epi.lncRNA

AC011451.1 non.epi.lncRNA

AC011453.1 non.epi.lncRNA

AC011455.1 non.epi.lncRNA

AC011461.1 non.epi.lncRNA

AC011462.2 non.epi.lncRNA

AC011462.3 non.epi.lncRNA

AC011462.4 non.epi.lncRNA

AC011465.1 non.epi.lncRNA

AC011466.2 non.epi.lncRNA

AC011466.3 non.epi.lncRNA

AC011467.1 non.epi.lncRNA

AC011467.3 non.epi.lncRNA

AC011468.1 non.epi.lncRNA

AC011468.2 non.epi.lncRNA

AC011468.3 non.epi.lncRNA

AC011468.5 non.epi.lncRNA

AC011471.2 non.epi.lncRNA

AC011472.2 non.epi.lncRNA

AC011472.3 non.epi.lncRNA

AC011472.4 non.epi.lncRNA

AC011473.1 non.epi.lncRNA

AC011473.2 non.epi.lncRNA

AC011473.3 non.epi.lncRNA

AC011474.1 non.epi.lncRNA

AC011474.2 non.epi.lncRNA

AC011474.3 non.epi.lncRNA

AC011476.2 non.epi.lncRNA

AC011476.3 non.epi.lncRNA

AC011477.2 non.epi.lncRNA

AC011477.3 non.epi.lncRNA

AC011477.4 non.epi.lncRNA

AC011478.1 non.epi.lncRNA

AC011479.2 non.epi.lncRNA

AC011479.3 non.epi.lncRNA

AC011481.1 non.epi.lncRNA

AC011481.2 non.epi.lncRNA

AC011483.1 non.epi.lncRNA

AC011483.2 non.epi.lncRNA

AC011484.1 non.epi.lncRNA

AC011491.2 non.epi.lncRNA

AC011495.2 non.epi.lncRNA

AC011495.3 non.epi.lncRNA

AC011497.1 non.epi.lncRNA

AC011498.1 non.epi.lncRNA

AC011498.2 non.epi.lncRNA

AC011498.3 non.epi.lncRNA

AC011498.6 non.epi.lncRNA

AC011503.1 non.epi.lncRNA

AC011507.1 non.epi.lncRNA

AC011509.1 non.epi.lncRNA

AC011509.2 non.epi.lncRNA

AC011511.2 non.epi.lncRNA

AC011511.3 non.epi.lncRNA

AC011511.5 non.epi.lncRNA

AC011516.1 non.epi.lncRNA

AC011518.1 non.epi.lncRNA

AC011523.1 non.epi.lncRNA

AC011524.1 non.epi.lncRNA

AC011524.2 non.epi.lncRNA

AC011525.1 non.epi.lncRNA

AC011525.2 non.epi.lncRNA

AC011586.1 non.epi.lncRNA

AC011586.2 non.epi.lncRNA

AC011591.1 non.epi.lncRNA

AC011601.1 non.epi.lncRNA

AC011603.1 non.epi.lncRNA

AC011603.3 non.epi.lncRNA

AC011611.3 non.epi.lncRNA

AC011632.1 non.epi.lncRNA

AC011632.2 non.epi.lncRNA

AC011676.1 non.epi.lncRNA

AC011676.2 non.epi.lncRNA

AC011676.3 non.epi.lncRNA

AC011676.4 non.epi.lncRNA

AC011700.1 non.epi.lncRNA

AC011718.1 non.epi.lncRNA

AC011726.2 non.epi.lncRNA

AC011726.3 non.epi.lncRNA

AC011731.1 non.epi.lncRNA

AC011747.1 non.epi.lncRNA

AC011752.1 non.epi.lncRNA

AC011754.1 non.epi.lncRNA

AC011773.1 non.epi.lncRNA

AC011773.2 non.epi.lncRNA

AC011773.4 non.epi.lncRNA

AC011815.1 non.epi.lncRNA

AC011815.2 non.epi.lncRNA

AC011816.2 non.epi.lncRNA

AC011824.1 non.epi.lncRNA

AC011824.2 non.epi.lncRNA

AC011824.3 non.epi.lncRNA

AC011824.4 non.epi.lncRNA

AC011825.2 non.epi.lncRNA

AC011825.3 non.epi.lncRNA

AC011840.1 non.epi.lncRNA

AC011840.2 non.epi.lncRNA

AC011840.3 non.epi.lncRNA

AC011840.4 non.epi.lncRNA

AC011853.2 non.epi.lncRNA

AC011891.2 non.epi.lncRNA

AC011893.1 non.epi.lncRNA

AC011897.1 non.epi.lncRNA

AC011899.1 non.epi.lncRNA

AC011899.2 non.epi.lncRNA

AC011899.3 non.epi.lncRNA

AC011900.1 non.epi.lncRNA

AC011901.1 non.epi.lncRNA

AC011912.1 non.epi.lncRNA

AC011933.2 non.epi.lncRNA

AC011933.4 non.epi.lncRNA

AC011939.2 non.epi.lncRNA

AC011939.3 non.epi.lncRNA

AC011978.2 non.epi.lncRNA

AC011990.1 non.epi.lncRNA

AC011995.1 non.epi.lncRNA

AC011995.2 non.epi.lncRNA

AC011997.1 non.epi.lncRNA

AC011998.3 non.epi.lncRNA

AC012020.1 non.epi.lncRNA

AC012038.1 non.epi.lncRNA

AC012050.1 non.epi.lncRNA

AC012055.1 non.epi.lncRNA

AC012055.2 non.epi.lncRNA

AC012063.1 non.epi.lncRNA

AC012065.1 non.epi.lncRNA

AC012065.3 non.epi.lncRNA

AC012065.4 non.epi.lncRNA

AC012070.1 non.epi.lncRNA

AC012074.1 non.epi.lncRNA

AC012081.1 non.epi.lncRNA

AC012085.2 non.epi.lncRNA

AC012087.2 non.epi.lncRNA

AC012100.2 non.epi.lncRNA

AC012103.1 non.epi.lncRNA

AC012123.1 non.epi.lncRNA

AC012146.1 non.epi.lncRNA

AC012146.3 non.epi.lncRNA

AC012150.1 non.epi.lncRNA

AC012150.2 non.epi.lncRNA

AC012157.2 non.epi.lncRNA

AC012170.2 non.epi.lncRNA

AC012174.1 non.epi.lncRNA

AC012178.1 non.epi.lncRNA

AC012181.1 non.epi.lncRNA

AC012181.2 non.epi.lncRNA

AC012184.1 non.epi.lncRNA

AC012184.3 non.epi.lncRNA

AC012213.1 non.epi.lncRNA

AC012213.2 non.epi.lncRNA

AC012213.3 non.epi.lncRNA

AC012213.4 non.epi.lncRNA

AC012229.1 non.epi.lncRNA

AC012254.1 non.epi.lncRNA

AC012254.3 non.epi.lncRNA

AC012291.2 non.epi.lncRNA

AC012301.1 non.epi.lncRNA

AC012306.2 non.epi.lncRNA

AC012309.2 non.epi.lncRNA

AC012313.2 non.epi.lncRNA

AC012313.3 non.epi.lncRNA

AC012313.5 non.epi.lncRNA

AC012313.6 non.epi.lncRNA

AC012313.8 non.epi.lncRNA

AC012313.9 non.epi.lncRNA

AC012321.1 non.epi.lncRNA

AC012322.1 non.epi.lncRNA

AC012339.1 non.epi.lncRNA

AC012349.1 non.epi.lncRNA

AC012354.1 non.epi.lncRNA

AC012355.1 non.epi.lncRNA

AC012358.1 non.epi.lncRNA

AC012358.3 non.epi.lncRNA

AC012360.1 non.epi.lncRNA

AC012360.2 non.epi.lncRNA

AC012360.3 non.epi.lncRNA

AC012363.1 non.epi.lncRNA

AC012363.2 non.epi.lncRNA

AC012366.1 non.epi.lncRNA

AC012368.1 non.epi.lncRNA

AC012368.2 non.epi.lncRNA

AC012370.2 non.epi.lncRNA

AC012378.1 non.epi.lncRNA

AC012379.1 non.epi.lncRNA

AC012404.1 non.epi.lncRNA

AC012404.2 non.epi.lncRNA

AC012409.1 non.epi.lncRNA

AC012409.2 non.epi.lncRNA

AC012409.3 non.epi.lncRNA

AC012409.4 non.epi.lncRNA

AC012413.1 non.epi.lncRNA

AC012414.3 non.epi.lncRNA

AC012414.4 non.epi.lncRNA

AC012414.5 non.epi.lncRNA

AC012417.1 non.epi.lncRNA

AC012435.3 non.epi.lncRNA

AC012442.1 non.epi.lncRNA

AC012442.2 non.epi.lncRNA

AC012445.1 non.epi.lncRNA

AC012451.1 non.epi.lncRNA

AC012456.1 non.epi.lncRNA

AC012462.1 non.epi.lncRNA

AC012462.2 non.epi.lncRNA

AC012464.1 non.epi.lncRNA

AC012464.2 non.epi.lncRNA

AC012464.3 non.epi.lncRNA

AC012467.1 non.epi.lncRNA

AC012485.1 non.epi.lncRNA

AC012485.2 non.epi.lncRNA

AC012494.1 non.epi.lncRNA

AC012494.2 non.epi.lncRNA

AC012499.1 non.epi.lncRNA

AC012501.2 non.epi.lncRNA

AC012506.1 non.epi.lncRNA

AC012506.2 non.epi.lncRNA

AC012507.1 non.epi.lncRNA

AC012508.1 non.epi.lncRNA

AC012508.2 non.epi.lncRNA

AC012510.1 non.epi.lncRNA

AC012511.1 non.epi.lncRNA

AC012513.1 non.epi.lncRNA

AC012531.2 non.epi.lncRNA

AC012555.2 non.epi.lncRNA

AC012557.1 non.epi.lncRNA

AC012558.1 non.epi.lncRNA

AC012568.1 non.epi.lncRNA

AC012572.1 non.epi.lncRNA

AC012574.1 non.epi.lncRNA

AC012574.2 non.epi.lncRNA

AC012593.1 non.epi.lncRNA

AC012603.1 non.epi.lncRNA

AC012613.1 non.epi.lncRNA

AC012613.2 non.epi.lncRNA

AC012615.1 non.epi.lncRNA

AC012615.5 non.epi.lncRNA

AC012615.6 non.epi.lncRNA

AC012629.1 non.epi.lncRNA

AC012629.2 non.epi.lncRNA

AC012640.1 non.epi.lncRNA

AC012640.2 non.epi.lncRNA

AC012640.3 non.epi.lncRNA

AC012645.1 non.epi.lncRNA

AC012645.2 non.epi.lncRNA

AC012645.3 non.epi.lncRNA

AC012645.4 non.epi.lncRNA

AC012653.2 non.epi.lncRNA

AC012668.1 non.epi.lncRNA

AC012668.2 non.epi.lncRNA

AC012668.3 non.epi.lncRNA

AC012676.3 non.epi.lncRNA

AC012676.4 non.epi.lncRNA

AC013248.1 non.epi.lncRNA

AC013269.1 non.epi.lncRNA

AC013269.2 non.epi.lncRNA

AC013275.1 non.epi.lncRNA

AC013286.1 non.epi.lncRNA

AC013287.1 non.epi.lncRNA

AC013356.2 non.epi.lncRNA

AC013356.3 non.epi.lncRNA

AC013356.4 non.epi.lncRNA

AC013391.1 non.epi.lncRNA

AC013391.2 non.epi.lncRNA

AC013391.3 non.epi.lncRNA

AC013400.1 non.epi.lncRNA

AC013401.1 non.epi.lncRNA

AC013402.1 non.epi.lncRNA

AC013402.2 non.epi.lncRNA

AC013402.3 non.epi.lncRNA

AC013403.2 non.epi.lncRNA

AC013436.1 non.epi.lncRNA

AC013437.1 non.epi.lncRNA

AC013448.1 non.epi.lncRNA

AC013448.2 non.epi.lncRNA

AC013451.1 non.epi.lncRNA

AC013451.2 non.epi.lncRNA

AC013452.2 non.epi.lncRNA

AC013460.1 non.epi.lncRNA

AC013472.1 non.epi.lncRNA

AC013472.2 non.epi.lncRNA

AC013472.3 non.epi.lncRNA

AC013509.1 non.epi.lncRNA

AC013549.1 non.epi.lncRNA

AC013549.3 non.epi.lncRNA

AC013549.4 non.epi.lncRNA

AC013553.4 non.epi.lncRNA

AC013562.1 non.epi.lncRNA

AC013564.1 non.epi.lncRNA

AC013565.3 non.epi.lncRNA

AC013640.1 non.epi.lncRNA

AC013643.2 non.epi.lncRNA

AC013644.1 non.epi.lncRNA

AC013652.1 non.epi.lncRNA

AC013652.2 non.epi.lncRNA

AC013714.1 non.epi.lncRNA

AC013724.1 non.epi.lncRNA

AC013727.1 non.epi.lncRNA

AC013727.2 non.epi.lncRNA

AC013733.1 non.epi.lncRNA

AC013733.2 non.epi.lncRNA

AC013762.1 non.epi.lncRNA

AC013799.1 non.epi.lncRNA

AC015468.1 non.epi.lncRNA

AC015468.2 non.epi.lncRNA

AC015468.3 non.epi.lncRNA

AC015522.1 non.epi.lncRNA

AC015540.1 non.epi.lncRNA

AC015563.1 non.epi.lncRNA

AC015563.2 non.epi.lncRNA

AC015574.1 non.epi.lncRNA

AC015631.1 non.epi.lncRNA

AC015660.1 non.epi.lncRNA

AC015660.2 non.epi.lncRNA

AC015660.3 non.epi.lncRNA

AC015674.1 non.epi.lncRNA

AC015688.6 non.epi.lncRNA

AC015712.1 non.epi.lncRNA

AC015712.2 non.epi.lncRNA

AC015712.6 non.epi.lncRNA

AC015712.7 non.epi.lncRNA

AC015722.1 non.epi.lncRNA

AC015722.2 non.epi.lncRNA

AC015726.1 non.epi.lncRNA

AC015726.2 non.epi.lncRNA

AC015727.1 non.epi.lncRNA

AC015795.1 non.epi.lncRNA

AC015802.3 non.epi.lncRNA

AC015802.4 non.epi.lncRNA

AC015802.5 non.epi.lncRNA

AC015813.3 non.epi.lncRNA

AC015818.2 non.epi.lncRNA

AC015818.4 non.epi.lncRNA

AC015818.7 non.epi.lncRNA

AC015818.8 non.epi.lncRNA

AC015819.1 non.epi.lncRNA

AC015819.2 non.epi.lncRNA

AC015819.3 non.epi.lncRNA

AC015845.1 non.epi.lncRNA

AC015845.2 non.epi.lncRNA

AC015849.1 non.epi.lncRNA

AC015849.2 non.epi.lncRNA

AC015849.3 non.epi.lncRNA

AC015853.1 non.epi.lncRNA

AC015853.3 non.epi.lncRNA

AC015878.1 non.epi.lncRNA

AC015878.2 non.epi.lncRNA

AC015908.2 non.epi.lncRNA

AC015908.3 non.epi.lncRNA

AC015908.4 non.epi.lncRNA

AC015909.3 non.epi.lncRNA

AC015909.4 non.epi.lncRNA

AC015911.2 non.epi.lncRNA

AC015911.3 non.epi.lncRNA

AC015911.5 non.epi.lncRNA

AC015911.6 non.epi.lncRNA

AC015911.9 non.epi.lncRNA

AC015912.1 non.epi.lncRNA

AC015912.3 non.epi.lncRNA

AC015914.1 non.epi.lncRNA

AC015921.1 non.epi.lncRNA

AC015922.2 non.epi.lncRNA

AC015922.3 non.epi.lncRNA

AC015923.1 non.epi.lncRNA

AC015936.1 non.epi.lncRNA

AC015936.2 non.epi.lncRNA

AC015961.1 non.epi.lncRNA

AC015961.2 non.epi.lncRNA

AC015967.1 non.epi.lncRNA

AC015969.1 non.epi.lncRNA

AC015971.1 non.epi.lncRNA

AC015977.2 non.epi.lncRNA

AC015983.2 non.epi.lncRNA

AC015987.1 non.epi.lncRNA

AC016026.1 non.epi.lncRNA

AC016027.2 non.epi.lncRNA

AC016027.3 non.epi.lncRNA

AC016044.1 non.epi.lncRNA

AC016065.1 non.epi.lncRNA

AC016074.1 non.epi.lncRNA

AC016134.1 non.epi.lncRNA

AC016152.1 non.epi.lncRNA

AC016168.1 non.epi.lncRNA

AC016168.2 non.epi.lncRNA

AC016229.1 non.epi.lncRNA

AC016245.1 non.epi.lncRNA

AC016252.1 non.epi.lncRNA

AC016266.1 non.epi.lncRNA

AC016355.1 non.epi.lncRNA

AC016382.1 non.epi.lncRNA

AC016383.1 non.epi.lncRNA

AC016396.1 non.epi.lncRNA

AC016396.2 non.epi.lncRNA

AC016405.2 non.epi.lncRNA

AC016405.3 non.epi.lncRNA

AC016493.1 non.epi.lncRNA

AC016526.1 non.epi.lncRNA

AC016526.2 non.epi.lncRNA

AC016542.1 non.epi.lncRNA

AC016550.1 non.epi.lncRNA

AC016550.2 non.epi.lncRNA

AC016550.3 non.epi.lncRNA

AC016553.1 non.epi.lncRNA

AC016556.1 non.epi.lncRNA

AC016573.1 non.epi.lncRNA

AC016575.1 non.epi.lncRNA

AC016576.1 non.epi.lncRNA

AC016586.1 non.epi.lncRNA

AC016588.1 non.epi.lncRNA

AC016590.1 non.epi.lncRNA

AC016590.2 non.epi.lncRNA

AC016595.1 non.epi.lncRNA

AC016596.1 non.epi.lncRNA

AC016597.1 non.epi.lncRNA

AC016597.2 non.epi.lncRNA

AC016598.1 non.epi.lncRNA

AC016598.2 non.epi.lncRNA

AC016629.2 non.epi.lncRNA

AC016642.1 non.epi.lncRNA

AC016644.1 non.epi.lncRNA

AC016650.1 non.epi.lncRNA

AC016683.1 non.epi.lncRNA

AC016687.2 non.epi.lncRNA

AC016687.3 non.epi.lncRNA

AC016700.3 non.epi.lncRNA

AC016705.2 non.epi.lncRNA

AC016710.1 non.epi.lncRNA

AC016716.2 non.epi.lncRNA

AC016717.2 non.epi.lncRNA

AC016722.1 non.epi.lncRNA

AC016722.2 non.epi.lncRNA

AC016723.1 non.epi.lncRNA

AC016730.1 non.epi.lncRNA

AC016735.1 non.epi.lncRNA

AC016737.1 non.epi.lncRNA

AC016737.2 non.epi.lncRNA

AC016738.2 non.epi.lncRNA

AC016745.2 non.epi.lncRNA

AC016746.1 non.epi.lncRNA

AC016747.1 non.epi.lncRNA

AC016747.2 non.epi.lncRNA

AC016747.3 non.epi.lncRNA

AC016747.4 non.epi.lncRNA

AC016751.1 non.epi.lncRNA

AC016751.2 non.epi.lncRNA

AC016766.1 non.epi.lncRNA

AC016773.1 non.epi.lncRNA

AC016813.1 non.epi.lncRNA

AC016820.1 non.epi.lncRNA

AC016822.1 non.epi.lncRNA

AC016825.1 non.epi.lncRNA

AC016831.1 non.epi.lncRNA

AC016831.5 non.epi.lncRNA

AC016831.6 non.epi.lncRNA

AC016866.1 non.epi.lncRNA

AC016866.2 non.epi.lncRNA

AC016868.1 non.epi.lncRNA

AC016876.1 non.epi.lncRNA

AC016876.3 non.epi.lncRNA

AC016877.1 non.epi.lncRNA

AC016877.3 non.epi.lncRNA

AC016885.1 non.epi.lncRNA

AC016885.2 non.epi.lncRNA

AC016903.1 non.epi.lncRNA

AC016903.2 non.epi.lncRNA

AC016907.2 non.epi.lncRNA

AC016910.1 non.epi.lncRNA

AC016924.1 non.epi.lncRNA

AC016933.1 non.epi.lncRNA

AC016949.1 non.epi.lncRNA

AC016957.2 non.epi.lncRNA

AC016968.1 non.epi.lncRNA

AC016987.1 non.epi.lncRNA

AC016987.2 non.epi.lncRNA

AC016993.1 non.epi.lncRNA

AC016994.1 non.epi.lncRNA

AC017002.1 non.epi.lncRNA

AC017002.2 non.epi.lncRNA

AC017002.5 non.epi.lncRNA

AC017006.1 non.epi.lncRNA

AC017006.2 non.epi.lncRNA

AC017007.5 non.epi.lncRNA

AC017015.2 non.epi.lncRNA

AC017033.1 non.epi.lncRNA

AC017048.1 non.epi.lncRNA

AC017048.2 non.epi.lncRNA

AC017048.3 non.epi.lncRNA

AC017053.1 non.epi.lncRNA

AC017067.1 non.epi.lncRNA

AC017071.1 non.epi.lncRNA

AC017074.2 non.epi.lncRNA

AC017076.1 non.epi.lncRNA

AC017083.1 non.epi.lncRNA

AC017083.2 non.epi.lncRNA

AC017091.1 non.epi.lncRNA

AC017101.1 non.epi.lncRNA

AC018358.1 non.epi.lncRNA

AC018359.1 non.epi.lncRNA

AC018362.2 non.epi.lncRNA

AC018371.1 non.epi.lncRNA

AC018410.1 non.epi.lncRNA

AC018437.2 non.epi.lncRNA

AC018442.2 non.epi.lncRNA

AC018445.1 non.epi.lncRNA

AC018450.2 non.epi.lncRNA

AC018462.1 non.epi.lncRNA

AC018464.1 non.epi.lncRNA

AC018467.1 non.epi.lncRNA

AC018495.1 non.epi.lncRNA

AC018511.1 non.epi.lncRNA

AC018511.2 non.epi.lncRNA

AC018521.1 non.epi.lncRNA

AC018521.2 non.epi.lncRNA

AC018521.4 non.epi.lncRNA

AC018521.6 non.epi.lncRNA

AC018541.1 non.epi.lncRNA

AC018552.2 non.epi.lncRNA

AC018552.3 non.epi.lncRNA

AC018553.1 non.epi.lncRNA

AC018557.1 non.epi.lncRNA

AC018557.2 non.epi.lncRNA

AC018558.1 non.epi.lncRNA

AC018558.2 non.epi.lncRNA

AC018558.3 non.epi.lncRNA

AC018563.1 non.epi.lncRNA

AC018607.1 non.epi.lncRNA

AC018616.1 non.epi.lncRNA

AC018618.1 non.epi.lncRNA

AC018635.2 non.epi.lncRNA

AC018638.6 non.epi.lncRNA

AC018638.7 non.epi.lncRNA

AC018643.1 non.epi.lncRNA

AC018645.3 non.epi.lncRNA

AC018647.1 non.epi.lncRNA

AC018648.1 non.epi.lncRNA

AC018653.1 non.epi.lncRNA

AC018653.3 non.epi.lncRNA

AC018680.1 non.epi.lncRNA

AC018682.1 non.epi.lncRNA

AC018682.2 non.epi.lncRNA

AC018685.1 non.epi.lncRNA

AC018685.2 non.epi.lncRNA

AC018692.1 non.epi.lncRNA

AC018693.1 non.epi.lncRNA

AC018695.2 non.epi.lncRNA

AC018695.3 non.epi.lncRNA

AC018695.4 non.epi.lncRNA

AC018697.1 non.epi.lncRNA

AC018716.1 non.epi.lncRNA

AC018730.1 non.epi.lncRNA

AC018731.1 non.epi.lncRNA

AC018742.1 non.epi.lncRNA

AC018752.1 non.epi.lncRNA

AC018755.1 non.epi.lncRNA

AC018761.1 non.epi.lncRNA

AC018761.2 non.epi.lncRNA

AC018761.4 non.epi.lncRNA

AC018767.1 non.epi.lncRNA

AC018767.2 non.epi.lncRNA

AC018767.3 non.epi.lncRNA

AC018809.1 non.epi.lncRNA

AC018809.2 non.epi.lncRNA

AC018814.1 non.epi.lncRNA

AC018816.1 non.epi.lncRNA

AC018845.1 non.epi.lncRNA

AC018861.2 non.epi.lncRNA

AC018865.1 non.epi.lncRNA

AC018866.1 non.epi.lncRNA

AC018878.1 non.epi.lncRNA

AC018904.2 non.epi.lncRNA

AC018926.1 non.epi.lncRNA

AC018926.2 non.epi.lncRNA

AC018926.3 non.epi.lncRNA

AC018946.1 non.epi.lncRNA

AC018953.1 non.epi.lncRNA

AC019055.1 non.epi.lncRNA

AC019064.1 non.epi.lncRNA

AC019068.1 non.epi.lncRNA

AC019069.1 non.epi.lncRNA

AC019077.1 non.epi.lncRNA

AC019080.1 non.epi.lncRNA

AC019080.4 non.epi.lncRNA

AC019080.5 non.epi.lncRNA

AC019080.6 non.epi.lncRNA

AC019117.1 non.epi.lncRNA

AC019117.2 non.epi.lncRNA

AC019118.1 non.epi.lncRNA

AC019118.2 non.epi.lncRNA

AC019129.2 non.epi.lncRNA

AC019130.1 non.epi.lncRNA

AC019131.1 non.epi.lncRNA

AC019131.2 non.epi.lncRNA

AC019133.1 non.epi.lncRNA

AC019155.2 non.epi.lncRNA

AC019163.1 non.epi.lncRNA

AC019171.1 non.epi.lncRNA

AC019183.1 non.epi.lncRNA

AC019185.2 non.epi.lncRNA

AC019193.2 non.epi.lncRNA

AC019193.3 non.epi.lncRNA

AC019197.1 non.epi.lncRNA

AC019205.1 non.epi.lncRNA

AC019209.1 non.epi.lncRNA

AC019209.2 non.epi.lncRNA

AC019209.3 non.epi.lncRNA

AC019211.1 non.epi.lncRNA

AC019254.2 non.epi.lncRNA

AC019257.1 non.epi.lncRNA

AC019257.2 non.epi.lncRNA

AC019270.1 non.epi.lncRNA

AC019294.3 non.epi.lncRNA

AC019330.1 non.epi.lncRNA

AC020551.1 non.epi.lncRNA

AC020558.1 non.epi.lncRNA

AC020558.2 non.epi.lncRNA

AC020594.1 non.epi.lncRNA

AC020595.1 non.epi.lncRNA

AC020611.2 non.epi.lncRNA

AC020612.3 non.epi.lncRNA

AC020634.2 non.epi.lncRNA

AC020636.1 non.epi.lncRNA

AC020637.1 non.epi.lncRNA

AC020651.1 non.epi.lncRNA

AC020656.1 non.epi.lncRNA

AC020656.2 non.epi.lncRNA

AC020658.2 non.epi.lncRNA

AC020658.3 non.epi.lncRNA

AC020659.1 non.epi.lncRNA

AC020661.1 non.epi.lncRNA

AC020661.2 non.epi.lncRNA

AC020661.3 non.epi.lncRNA

AC020663.1 non.epi.lncRNA

AC020663.2 non.epi.lncRNA

AC020687.1 non.epi.lncRNA

AC020703.1 non.epi.lncRNA

AC020704.1 non.epi.lncRNA

AC020718.1 non.epi.lncRNA

AC020719.1 non.epi.lncRNA

AC020741.1 non.epi.lncRNA

AC020743.1 non.epi.lncRNA

AC020743.2 non.epi.lncRNA

AC020743.3 non.epi.lncRNA

AC020765.2 non.epi.lncRNA

AC020779.2 non.epi.lncRNA

AC020891.1 non.epi.lncRNA

AC020891.2 non.epi.lncRNA

AC020892.1 non.epi.lncRNA

AC020892.2 non.epi.lncRNA

AC020897.1 non.epi.lncRNA

AC020900.1 non.epi.lncRNA

AC020905.1 non.epi.lncRNA

AC020907.1 non.epi.lncRNA

AC020907.2 non.epi.lncRNA

AC020907.3 non.epi.lncRNA

AC020907.4 non.epi.lncRNA

AC020908.1 non.epi.lncRNA

AC020908.2 non.epi.lncRNA

AC020908.3 non.epi.lncRNA

AC020909.2 non.epi.lncRNA

AC020909.3 non.epi.lncRNA

AC020910.2 non.epi.lncRNA

AC020910.5 non.epi.lncRNA

AC020911.1 non.epi.lncRNA

AC020911.2 non.epi.lncRNA

AC020913.1 non.epi.lncRNA

AC020913.2 non.epi.lncRNA

AC020913.3 non.epi.lncRNA

AC020914.1 non.epi.lncRNA

AC020915.2 non.epi.lncRNA

AC020917.2 non.epi.lncRNA

AC020917.3 non.epi.lncRNA

AC020922.2 non.epi.lncRNA

AC020928.1 non.epi.lncRNA

AC020928.2 non.epi.lncRNA

AC020931.1 non.epi.lncRNA

AC020934.1 non.epi.lncRNA

AC020934.2 non.epi.lncRNA

AC020978.1 non.epi.lncRNA

AC020978.2 non.epi.lncRNA

AC020978.3 non.epi.lncRNA

AC020978.4 non.epi.lncRNA

AC020978.5 non.epi.lncRNA

AC020978.6 non.epi.lncRNA

AC020978.7 non.epi.lncRNA

AC021016.3 non.epi.lncRNA

AC021028.1 non.epi.lncRNA

AC021035.1 non.epi.lncRNA

AC021074.3 non.epi.lncRNA

AC021086.1 non.epi.lncRNA

AC021087.3 non.epi.lncRNA

AC021087.4 non.epi.lncRNA

AC021088.1 non.epi.lncRNA

AC021092.1 non.epi.lncRNA

AC021097.1 non.epi.lncRNA

AC021127.1 non.epi.lncRNA

AC021134.1 non.epi.lncRNA

AC021146.12 non.epi.lncRNA

AC021151.1 non.epi.lncRNA

AC021180.1 non.epi.lncRNA

AC021188.1 non.epi.lncRNA

AC021192.1 non.epi.lncRNA

AC021205.3 non.epi.lncRNA

AC021218.1 non.epi.lncRNA

AC021231.1 non.epi.lncRNA

AC021242.2 non.epi.lncRNA

AC021351.1 non.epi.lncRNA

AC021355.1 non.epi.lncRNA

AC021382.1 non.epi.lncRNA

AC021393.1 non.epi.lncRNA

AC021422.1 non.epi.lncRNA

AC021483.1 non.epi.lncRNA

AC021483.2 non.epi.lncRNA

AC021491.2 non.epi.lncRNA

AC021491.4 non.epi.lncRNA

AC021504.1 non.epi.lncRNA

AC021506.1 non.epi.lncRNA

AC021517.1 non.epi.lncRNA

AC021534.1 non.epi.lncRNA

AC021546.1 non.epi.lncRNA

AC021549.1 non.epi.lncRNA

AC021594.1 non.epi.lncRNA

AC021594.2 non.epi.lncRNA

AC021613.1 non.epi.lncRNA

AC021678.2 non.epi.lncRNA

AC021683.1 non.epi.lncRNA

AC021683.3 non.epi.lncRNA

AC021713.1 non.epi.lncRNA

AC021733.2 non.epi.lncRNA

AC021736.1 non.epi.lncRNA

AC021739.2 non.epi.lncRNA

AC021739.3 non.epi.lncRNA

AC021739.5 non.epi.lncRNA

AC021744.1 non.epi.lncRNA

AC021755.2 non.epi.lncRNA

AC021755.3 non.epi.lncRNA

AC021785.1 non.epi.lncRNA

AC021818.1 non.epi.lncRNA

AC021860.1 non.epi.lncRNA

AC021915.1 non.epi.lncRNA

AC021915.2 non.epi.lncRNA

AC021979.1 non.epi.lncRNA

AC021979.2 non.epi.lncRNA

AC022007.1 non.epi.lncRNA

AC022017.1 non.epi.lncRNA

AC022031.1 non.epi.lncRNA

AC022031.2 non.epi.lncRNA

AC022034.3 non.epi.lncRNA

AC022034.4 non.epi.lncRNA

AC022035.1 non.epi.lncRNA

AC022039.1 non.epi.lncRNA

AC022079.1 non.epi.lncRNA

AC022079.2 non.epi.lncRNA

AC022081.1 non.epi.lncRNA

AC022087.1 non.epi.lncRNA

AC022098.1 non.epi.lncRNA

AC022098.2 non.epi.lncRNA

AC022098.3 non.epi.lncRNA

AC022098.4 non.epi.lncRNA

AC022113.1 non.epi.lncRNA

AC022118.1 non.epi.lncRNA

AC022126.1 non.epi.lncRNA

AC022140.1 non.epi.lncRNA

AC022144.1 non.epi.lncRNA

AC022145.1 non.epi.lncRNA

AC022146.2 non.epi.lncRNA

AC022148.1 non.epi.lncRNA

AC022148.2 non.epi.lncRNA

AC022150.1 non.epi.lncRNA

AC022150.2 non.epi.lncRNA

AC022150.3 non.epi.lncRNA

AC022150.4 non.epi.lncRNA

AC022164.1 non.epi.lncRNA

AC022165.1 non.epi.lncRNA

AC022166.1 non.epi.lncRNA

AC022166.2 non.epi.lncRNA

AC022167.2 non.epi.lncRNA

AC022167.3 non.epi.lncRNA

AC022182.1 non.epi.lncRNA

AC022182.2 non.epi.lncRNA

AC022188.1 non.epi.lncRNA

AC022201.1 non.epi.lncRNA

AC022201.2 non.epi.lncRNA

AC022217.3 non.epi.lncRNA

AC022239.1 non.epi.lncRNA

AC022272.1 non.epi.lncRNA

AC022274.1 non.epi.lncRNA

AC022306.2 non.epi.lncRNA

AC022306.3 non.epi.lncRNA

AC022364.1 non.epi.lncRNA

AC022367.1 non.epi.lncRNA

AC022382.1 non.epi.lncRNA

AC022395.1 non.epi.lncRNA

AC022400.1 non.epi.lncRNA

AC022400.2 non.epi.lncRNA

AC022400.4 non.epi.lncRNA

AC022424.1 non.epi.lncRNA

AC022424.2 non.epi.lncRNA

AC022431.1 non.epi.lncRNA

AC022441.1 non.epi.lncRNA

AC022441.2 non.epi.lncRNA

AC022445.1 non.epi.lncRNA

AC022447.1 non.epi.lncRNA

AC022447.3 non.epi.lncRNA

AC022447.5 non.epi.lncRNA

AC022447.6 non.epi.lncRNA

AC022447.7 non.epi.lncRNA

AC022467.1 non.epi.lncRNA

AC022469.1 non.epi.lncRNA

AC022469.2 non.epi.lncRNA

AC022486.1 non.epi.lncRNA

AC022498.1 non.epi.lncRNA

AC022509.1 non.epi.lncRNA

AC022509.2 non.epi.lncRNA

AC022509.3 non.epi.lncRNA

AC022509.4 non.epi.lncRNA

AC022511.1 non.epi.lncRNA

AC022517.1 non.epi.lncRNA

AC022523.1 non.epi.lncRNA

AC022523.3 non.epi.lncRNA

AC022535.1 non.epi.lncRNA

AC022537.1 non.epi.lncRNA

AC022540.1 non.epi.lncRNA

AC022558.1 non.epi.lncRNA

AC022558.2 non.epi.lncRNA

AC022596.1 non.epi.lncRNA

AC022601.1 non.epi.lncRNA

AC022613.1 non.epi.lncRNA

AC022613.3 non.epi.lncRNA

AC022634.2 non.epi.lncRNA

AC022655.1 non.epi.lncRNA

AC022679.1 non.epi.lncRNA

AC022679.2 non.epi.lncRNA

AC022690.2 non.epi.lncRNA

AC022695.2 non.epi.lncRNA

AC022695.3 non.epi.lncRNA

AC022706.1 non.epi.lncRNA

AC022710.1 non.epi.lncRNA

AC022726.1 non.epi.lncRNA

AC022730.3 non.epi.lncRNA

AC022730.4 non.epi.lncRNA

AC022733.1 non.epi.lncRNA

AC022733.2 non.epi.lncRNA

AC022762.1 non.epi.lncRNA

AC022762.2 non.epi.lncRNA

AC022778.1 non.epi.lncRNA

AC022784.1 non.epi.lncRNA

AC022784.2 non.epi.lncRNA

AC022784.3 non.epi.lncRNA

AC022784.5 non.epi.lncRNA

AC022784.6 non.epi.lncRNA

AC022809.1 non.epi.lncRNA

AC022816.1 non.epi.lncRNA

AC022819.1 non.epi.lncRNA

AC022858.1 non.epi.lncRNA

AC022874.1 non.epi.lncRNA

AC022893.1 non.epi.lncRNA

AC022893.2 non.epi.lncRNA

AC022898.1 non.epi.lncRNA

AC022898.2 non.epi.lncRNA

AC022903.1 non.epi.lncRNA

AC022915.1 non.epi.lncRNA

AC022915.2 non.epi.lncRNA

AC022915.3 non.epi.lncRNA

AC022916.1 non.epi.lncRNA

AC022916.2 non.epi.lncRNA

AC022916.4 non.epi.lncRNA

AC022929.1 non.epi.lncRNA

AC022929.2 non.epi.lncRNA

AC022960.2 non.epi.lncRNA

AC022966.1 non.epi.lncRNA

AC022973.5 non.epi.lncRNA

AC023034.1 non.epi.lncRNA

AC023043.1 non.epi.lncRNA

AC023043.4 non.epi.lncRNA

AC023078.1 non.epi.lncRNA

AC023090.1 non.epi.lncRNA

AC023115.1 non.epi.lncRNA

AC023128.1 non.epi.lncRNA

AC023136.1 non.epi.lncRNA

AC023137.1 non.epi.lncRNA

AC023154.1 non.epi.lncRNA

AC023158.1 non.epi.lncRNA

AC023158.2 non.epi.lncRNA

AC023194.3 non.epi.lncRNA

AC023200.1 non.epi.lncRNA

AC023202.1 non.epi.lncRNA

AC023282.1 non.epi.lncRNA

AC023300.1 non.epi.lncRNA

AC023301.1 non.epi.lncRNA

AC023302.1 non.epi.lncRNA

AC023310.4 non.epi.lncRNA

AC023347.1 non.epi.lncRNA

AC023355.1 non.epi.lncRNA

AC023389.1 non.epi.lncRNA

AC023394.1 non.epi.lncRNA

AC023394.2 non.epi.lncRNA

AC023403.1 non.epi.lncRNA

AC023421.2 non.epi.lncRNA

AC023442.2 non.epi.lncRNA

AC023442.3 non.epi.lncRNA

AC023449.2 non.epi.lncRNA

AC023469.1 non.epi.lncRNA

AC023469.2 non.epi.lncRNA

AC023481.1 non.epi.lncRNA

AC023490.2 non.epi.lncRNA

AC023490.4 non.epi.lncRNA

AC023509.1 non.epi.lncRNA

AC023509.2 non.epi.lncRNA

AC023509.3 non.epi.lncRNA

AC023510.1 non.epi.lncRNA

AC023511.1 non.epi.lncRNA

AC023590.1 non.epi.lncRNA

AC023595.1 non.epi.lncRNA

AC023632.5 non.epi.lncRNA

AC023644.1 non.epi.lncRNA

AC023669.1 non.epi.lncRNA

AC023669.2 non.epi.lncRNA

AC023762.1 non.epi.lncRNA

AC023790.2 non.epi.lncRNA

AC023794.1 non.epi.lncRNA

AC023794.3 non.epi.lncRNA

AC023794.5 non.epi.lncRNA

AC023794.6 non.epi.lncRNA

AC023796.1 non.epi.lncRNA

AC023796.2 non.epi.lncRNA

AC023813.1 non.epi.lncRNA

AC023813.3 non.epi.lncRNA

AC023813.4 non.epi.lncRNA

AC023824.1 non.epi.lncRNA

AC023824.3 non.epi.lncRNA

AC023824.4 non.epi.lncRNA

AC023824.5 non.epi.lncRNA

AC023825.2 non.epi.lncRNA

AC023830.1 non.epi.lncRNA

AC023830.2 non.epi.lncRNA

AC023830.3 non.epi.lncRNA

AC023855.1 non.epi.lncRNA

AC023866.1 non.epi.lncRNA

AC023866.2 non.epi.lncRNA

AC023886.1 non.epi.lncRNA

AC023905.1 non.epi.lncRNA

AC023906.3 non.epi.lncRNA

AC023906.4 non.epi.lncRNA

AC023906.5 non.epi.lncRNA

AC023908.1 non.epi.lncRNA

AC023908.2 non.epi.lncRNA

AC023946.1 non.epi.lncRNA

AC023983.1 non.epi.lncRNA

AC023983.2 non.epi.lncRNA

AC024022.1 non.epi.lncRNA

AC024028.1 non.epi.lncRNA

AC024060.2 non.epi.lncRNA

AC024075.1 non.epi.lncRNA

AC024075.2 non.epi.lncRNA

AC024075.3 non.epi.lncRNA

AC024084.1 non.epi.lncRNA

AC024132.1 non.epi.lncRNA

AC024132.2 non.epi.lncRNA

AC024132.3 non.epi.lncRNA

AC024145.1 non.epi.lncRNA

AC024224.2 non.epi.lncRNA

AC024230.1 non.epi.lncRNA

AC024243.1 non.epi.lncRNA

AC024257.3 non.epi.lncRNA

AC024257.4 non.epi.lncRNA

AC024257.5 non.epi.lncRNA

AC024267.1 non.epi.lncRNA

AC024267.3 non.epi.lncRNA

AC024267.5 non.epi.lncRNA

AC024267.6 non.epi.lncRNA

AC024270.2 non.epi.lncRNA

AC024270.3 non.epi.lncRNA

AC024270.4 non.epi.lncRNA

AC024337.2 non.epi.lncRNA

AC024361.1 non.epi.lncRNA

AC024361.2 non.epi.lncRNA

AC024361.3 non.epi.lncRNA

AC024451.4 non.epi.lncRNA

AC024475.1 non.epi.lncRNA

AC024475.3 non.epi.lncRNA

AC024559.2 non.epi.lncRNA

AC024560.1 non.epi.lncRNA

AC024560.3 non.epi.lncRNA

AC024563.1 non.epi.lncRNA

AC024579.1 non.epi.lncRNA

AC024581.1 non.epi.lncRNA

AC024588.1 non.epi.lncRNA

AC024592.1 non.epi.lncRNA

AC024592.2 non.epi.lncRNA

AC024603.1 non.epi.lncRNA

AC024610.1 non.epi.lncRNA

AC024610.2 non.epi.lncRNA

AC024614.1 non.epi.lncRNA

AC024614.2 non.epi.lncRNA

AC024619.1 non.epi.lncRNA

AC024619.2 non.epi.lncRNA

AC024651.1 non.epi.lncRNA

AC024651.2 non.epi.lncRNA

AC024681.2 non.epi.lncRNA

AC024884.2 non.epi.lncRNA

AC024896.1 non.epi.lncRNA

AC024901.1 non.epi.lncRNA

AC024933.1 non.epi.lncRNA

AC024940.1 non.epi.lncRNA

AC024940.6 non.epi.lncRNA

AC024941.1 non.epi.lncRNA

AC024941.2 non.epi.lncRNA

AC024958.1 non.epi.lncRNA

AC025030.2 non.epi.lncRNA

AC025031.3 non.epi.lncRNA

AC025034.1 non.epi.lncRNA

AC025038.1 non.epi.lncRNA

AC025040.1 non.epi.lncRNA

AC025048.1 non.epi.lncRNA

AC025154.1 non.epi.lncRNA

AC025154.2 non.epi.lncRNA

AC025159.1 non.epi.lncRNA

AC025164.1 non.epi.lncRNA

AC025165.1 non.epi.lncRNA

AC025165.2 non.epi.lncRNA

AC025165.4 non.epi.lncRNA

AC025165.5 non.epi.lncRNA

AC025166.1 non.epi.lncRNA

AC025171.1 non.epi.lncRNA

AC025171.3 non.epi.lncRNA

AC025171.5 non.epi.lncRNA

AC025174.1 non.epi.lncRNA

AC025175.1 non.epi.lncRNA

AC025176.1 non.epi.lncRNA

AC025180.1 non.epi.lncRNA

AC025183.1 non.epi.lncRNA

AC025183.2 non.epi.lncRNA

AC025183.3 non.epi.lncRNA

AC025187.1 non.epi.lncRNA

AC025188.1 non.epi.lncRNA

AC025211.1 non.epi.lncRNA

AC025244.1 non.epi.lncRNA

AC025244.2 non.epi.lncRNA

AC025252.1 non.epi.lncRNA

AC025253.1 non.epi.lncRNA

AC025254.1 non.epi.lncRNA

AC025257.1 non.epi.lncRNA

AC025259.1 non.epi.lncRNA

AC025259.3 non.epi.lncRNA

AC025265.1 non.epi.lncRNA

AC025265.2 non.epi.lncRNA

AC025265.3 non.epi.lncRNA

AC025271.2 non.epi.lncRNA

AC025271.3 non.epi.lncRNA

AC025272.1 non.epi.lncRNA

AC025277.1 non.epi.lncRNA

AC025278.1 non.epi.lncRNA

AC025279.1 non.epi.lncRNA

AC025280.1 non.epi.lncRNA

AC025280.2 non.epi.lncRNA

AC025284.1 non.epi.lncRNA

AC025287.1 non.epi.lncRNA

AC025287.2 non.epi.lncRNA

AC025287.3 non.epi.lncRNA

AC025300.1 non.epi.lncRNA

AC025366.1 non.epi.lncRNA

AC025370.1 non.epi.lncRNA

AC025419.1 non.epi.lncRNA

AC025423.1 non.epi.lncRNA

AC025428.1 non.epi.lncRNA

AC025431.1 non.epi.lncRNA

AC025434.1 non.epi.lncRNA

AC025437.1 non.epi.lncRNA

AC025437.2 non.epi.lncRNA

AC025437.3 non.epi.lncRNA

AC025437.4 non.epi.lncRNA

AC025437.5 non.epi.lncRNA

AC025442.1 non.epi.lncRNA

AC025465.1 non.epi.lncRNA

AC025465.2 non.epi.lncRNA

AC025465.3 non.epi.lncRNA

AC025465.4 non.epi.lncRNA

AC025470.2 non.epi.lncRNA

AC025475.1 non.epi.lncRNA

AC025521.1 non.epi.lncRNA

AC025524.1 non.epi.lncRNA

AC025539.1 non.epi.lncRNA

AC025566.1 non.epi.lncRNA

AC025569.1 non.epi.lncRNA

AC025572.1 non.epi.lncRNA

AC025575.1 non.epi.lncRNA

AC025575.2 non.epi.lncRNA

AC025576.1 non.epi.lncRNA

AC025576.2 non.epi.lncRNA

AC025580.3 non.epi.lncRNA

AC025627.1 non.epi.lncRNA

AC025674.2 non.epi.lncRNA

AC025741.1 non.epi.lncRNA

AC025752.1 non.epi.lncRNA

AC025754.1 non.epi.lncRNA

AC025766.1 non.epi.lncRNA

AC025773.1 non.epi.lncRNA

AC025809.1 non.epi.lncRNA

AC025810.1 non.epi.lncRNA

AC025811.1 non.epi.lncRNA

AC025822.1 non.epi.lncRNA

AC025822.2 non.epi.lncRNA

AC025857.2 non.epi.lncRNA

AC025871.1 non.epi.lncRNA

AC025871.2 non.epi.lncRNA

AC025887.1 non.epi.lncRNA

AC025917.1 non.epi.lncRNA

AC025918.1 non.epi.lncRNA

AC025946.1 non.epi.lncRNA

AC025947.1 non.epi.lncRNA

AC026116.1 non.epi.lncRNA

AC026124.2 non.epi.lncRNA

AC026150.1 non.epi.lncRNA

AC026150.3 non.epi.lncRNA

AC026167.1 non.epi.lncRNA

AC026191.1 non.epi.lncRNA

AC026202.1 non.epi.lncRNA

AC026202.2 non.epi.lncRNA

AC026202.3 non.epi.lncRNA

AC026254.2 non.epi.lncRNA

AC026304.1 non.epi.lncRNA

AC026316.3 non.epi.lncRNA

AC026320.1 non.epi.lncRNA

AC026333.3 non.epi.lncRNA

AC026333.4 non.epi.lncRNA

AC026336.2 non.epi.lncRNA

AC026336.3 non.epi.lncRNA

AC026341.1 non.epi.lncRNA

AC026353.1 non.epi.lncRNA

AC026356.1 non.epi.lncRNA

AC026356.2 non.epi.lncRNA

AC026358.1 non.epi.lncRNA

AC026362.1 non.epi.lncRNA

AC026367.1 non.epi.lncRNA

AC026367.2 non.epi.lncRNA

AC026367.3 non.epi.lncRNA

AC026368.1 non.epi.lncRNA

AC026369.1 non.epi.lncRNA

AC026369.2 non.epi.lncRNA

AC026369.3 non.epi.lncRNA

AC026391.1 non.epi.lncRNA

AC026401.1 non.epi.lncRNA

AC026401.2 non.epi.lncRNA

AC026401.3 non.epi.lncRNA

AC026412.3 non.epi.lncRNA

AC026414.1 non.epi.lncRNA

AC026415.1 non.epi.lncRNA

AC026427.1 non.epi.lncRNA

AC026458.2 non.epi.lncRNA

AC026461.2 non.epi.lncRNA

AC026462.2 non.epi.lncRNA

AC026462.3 non.epi.lncRNA

AC026462.4 non.epi.lncRNA

AC026464.2 non.epi.lncRNA

AC026470.2 non.epi.lncRNA

AC026470.3 non.epi.lncRNA

AC026471.1 non.epi.lncRNA

AC026471.2 non.epi.lncRNA

AC026471.3 non.epi.lncRNA

AC026471.5 non.epi.lncRNA

AC026474.1 non.epi.lncRNA

AC026495.1 non.epi.lncRNA

AC026523.1 non.epi.lncRNA

AC026583.1 non.epi.lncRNA

AC026585.1 non.epi.lncRNA

AC026620.1 non.epi.lncRNA

AC026688.2 non.epi.lncRNA

AC026689.1 non.epi.lncRNA

AC026704.1 non.epi.lncRNA

AC026719.1 non.epi.lncRNA

AC026725.1 non.epi.lncRNA

AC026726.1 non.epi.lncRNA

AC026740.1 non.epi.lncRNA

AC026741.1 non.epi.lncRNA

AC026746.1 non.epi.lncRNA

AC026765.2 non.epi.lncRNA

AC026765.3 non.epi.lncRNA

AC026770.1 non.epi.lncRNA

AC026771.1 non.epi.lncRNA

AC026782.1 non.epi.lncRNA

AC026782.2 non.epi.lncRNA

AC026785.2 non.epi.lncRNA

AC026786.2 non.epi.lncRNA

AC026787.1 non.epi.lncRNA

AC026790.1 non.epi.lncRNA

AC026801.2 non.epi.lncRNA

AC026803.2 non.epi.lncRNA

AC026888.1 non.epi.lncRNA

AC026894.1 non.epi.lncRNA

AC026904.1 non.epi.lncRNA

AC026904.2 non.epi.lncRNA

AC026954.3 non.epi.lncRNA

AC026979.1 non.epi.lncRNA

AC026979.3 non.epi.lncRNA

AC026991.1 non.epi.lncRNA

AC026992.1 non.epi.lncRNA

AC026992.2 non.epi.lncRNA

AC027013.1 non.epi.lncRNA

AC027018.1 non.epi.lncRNA

AC027020.1 non.epi.lncRNA

AC027020.2 non.epi.lncRNA

AC027031.1 non.epi.lncRNA

AC027031.2 non.epi.lncRNA

AC027045.1 non.epi.lncRNA

AC027088.1 non.epi.lncRNA

AC027088.2 non.epi.lncRNA

AC027088.3 non.epi.lncRNA

AC027097.1 non.epi.lncRNA

AC027097.2 non.epi.lncRNA

AC027117.1 non.epi.lncRNA

AC027117.2 non.epi.lncRNA

AC027130.1 non.epi.lncRNA

AC027228.2 non.epi.lncRNA

AC027237.3 non.epi.lncRNA

AC027237.4 non.epi.lncRNA

AC027243.1 non.epi.lncRNA

AC027243.2 non.epi.lncRNA

AC027271.1 non.epi.lncRNA

AC027277.1 non.epi.lncRNA

AC027277.2 non.epi.lncRNA

AC027279.1 non.epi.lncRNA

AC027279.2 non.epi.lncRNA

AC027281.1 non.epi.lncRNA

AC027287.2 non.epi.lncRNA

AC027288.1 non.epi.lncRNA

AC027288.3 non.epi.lncRNA

AC027290.1 non.epi.lncRNA

AC027306.1 non.epi.lncRNA

AC027307.1 non.epi.lncRNA

AC027307.2 non.epi.lncRNA

AC027312.1 non.epi.lncRNA

AC027315.1 non.epi.lncRNA

AC027319.1 non.epi.lncRNA

AC027335.1 non.epi.lncRNA

AC027338.1 non.epi.lncRNA

AC027338.2 non.epi.lncRNA

AC027343.1 non.epi.lncRNA

AC027343.2 non.epi.lncRNA

AC027343.3 non.epi.lncRNA

AC027348.1 non.epi.lncRNA

AC027373.1 non.epi.lncRNA

AC027449.1 non.epi.lncRNA

AC027451.1 non.epi.lncRNA

AC027458.1 non.epi.lncRNA

AC027514.1 non.epi.lncRNA

AC027514.2 non.epi.lncRNA

AC027541.1 non.epi.lncRNA

AC027575.2 non.epi.lncRNA

AC027601.2 non.epi.lncRNA

AC027601.3 non.epi.lncRNA

AC027601.4 non.epi.lncRNA

AC027607.1 non.epi.lncRNA

AC027627.1 non.epi.lncRNA

AC027682.2 non.epi.lncRNA

AC027682.3 non.epi.lncRNA

AC027682.4 non.epi.lncRNA

AC027688.1 non.epi.lncRNA

AC027688.2 non.epi.lncRNA

AC027698.1 non.epi.lncRNA

AC027702.1 non.epi.lncRNA

AC027763.2 non.epi.lncRNA

AC027779.1 non.epi.lncRNA

AC027796.1 non.epi.lncRNA

AC027796.4 non.epi.lncRNA

AC027796.5 non.epi.lncRNA

AC027801.1 non.epi.lncRNA

AC027801.4 non.epi.lncRNA

AC027804.1 non.epi.lncRNA

AC027807.2 non.epi.lncRNA

AC027808.2 non.epi.lncRNA

AC032019.2 non.epi.lncRNA

AC032044.1 non.epi.lncRNA

AC034102.3 non.epi.lncRNA

AC034102.5 non.epi.lncRNA

AC034102.8 non.epi.lncRNA

AC034111.1 non.epi.lncRNA

AC034114.2 non.epi.lncRNA

AC034154.1 non.epi.lncRNA

AC034187.1 non.epi.lncRNA

AC034195.1 non.epi.lncRNA

AC034198.2 non.epi.lncRNA

AC034206.1 non.epi.lncRNA

AC034213.1 non.epi.lncRNA

AC034223.1 non.epi.lncRNA

AC034226.1 non.epi.lncRNA

AC034228.1 non.epi.lncRNA

AC034228.3 non.epi.lncRNA

AC034229.1 non.epi.lncRNA

AC034229.2 non.epi.lncRNA

AC034229.3 non.epi.lncRNA

AC034229.4 non.epi.lncRNA

AC034232.2 non.epi.lncRNA

AC034234.1 non.epi.lncRNA

AC034236.3 non.epi.lncRNA

AC034238.1 non.epi.lncRNA

AC034238.2 non.epi.lncRNA

AC034238.3 non.epi.lncRNA

AC034243.1 non.epi.lncRNA

AC034245.1 non.epi.lncRNA

AC035139.1 non.epi.lncRNA

AC035140.1 non.epi.lncRNA

AC036101.1 non.epi.lncRNA

AC036103.1 non.epi.lncRNA

AC036108.1 non.epi.lncRNA

AC036108.2 non.epi.lncRNA

AC036108.3 non.epi.lncRNA

AC036176.1 non.epi.lncRNA

AC036176.3 non.epi.lncRNA

AC036214.1 non.epi.lncRNA

AC036214.2 non.epi.lncRNA

AC036222.1 non.epi.lncRNA

AC036222.2 non.epi.lncRNA

AC037198.1 non.epi.lncRNA

AC037198.2 non.epi.lncRNA

AC037441.1 non.epi.lncRNA

AC037441.2 non.epi.lncRNA

AC037450.1 non.epi.lncRNA

AC037459.2 non.epi.lncRNA

AC037459.3 non.epi.lncRNA

AC037486.1 non.epi.lncRNA

AC037487.1 non.epi.lncRNA

AC037487.2 non.epi.lncRNA

AC037487.4 non.epi.lncRNA

AC039056.1 non.epi.lncRNA

AC039056.2 non.epi.lncRNA

AC040160.1 non.epi.lncRNA

AC040162.3 non.epi.lncRNA

AC040168.1 non.epi.lncRNA

AC040169.3 non.epi.lncRNA

AC040173.1 non.epi.lncRNA

AC040174.1 non.epi.lncRNA

AC040174.2 non.epi.lncRNA

AC040896.1 non.epi.lncRNA

AC040904.1 non.epi.lncRNA

AC040914.1 non.epi.lncRNA

AC040936.1 non.epi.lncRNA

AC040963.1 non.epi.lncRNA

AC040977.1 non.epi.lncRNA

AC040977.2 non.epi.lncRNA

AC044784.1 non.epi.lncRNA

AC044798.1 non.epi.lncRNA

AC044798.2 non.epi.lncRNA

AC044802.1 non.epi.lncRNA

AC044802.2 non.epi.lncRNA

AC044810.2 non.epi.lncRNA

AC044810.3 non.epi.lncRNA

AC044839.2 non.epi.lncRNA

AC044840.1 non.epi.lncRNA

AC044873.1 non.epi.lncRNA

AC044893.1 non.epi.lncRNA

AC046130.1 non.epi.lncRNA

AC046130.2 non.epi.lncRNA

AC046134.2 non.epi.lncRNA

AC046143.2 non.epi.lncRNA

AC046158.1 non.epi.lncRNA

AC046158.2 non.epi.lncRNA

AC046195.1 non.epi.lncRNA

AC046195.2 non.epi.lncRNA

AC048337.1 non.epi.lncRNA

AC048341.1 non.epi.lncRNA

AC048341.2 non.epi.lncRNA

AC048344.1 non.epi.lncRNA

AC048352.1 non.epi.lncRNA

AC048380.1 non.epi.lncRNA

AC048382.1 non.epi.lncRNA

AC048382.2 non.epi.lncRNA

AC048382.4 non.epi.lncRNA

AC048382.5 non.epi.lncRNA

AC048382.6 non.epi.lncRNA

AC048387.1 non.epi.lncRNA

AC051618.1 non.epi.lncRNA

AC051619.5 non.epi.lncRNA

AC051619.8 non.epi.lncRNA

AC053503.4 non.epi.lncRNA

AC053503.5 non.epi.lncRNA

AC053513.1 non.epi.lncRNA

AC053513.2 non.epi.lncRNA

AC053527.2 non.epi.lncRNA

AC055716.3 non.epi.lncRNA

AC055717.1 non.epi.lncRNA

AC055717.2 non.epi.lncRNA

AC055720.1 non.epi.lncRNA

AC055720.2 non.epi.lncRNA

AC055736.1 non.epi.lncRNA

AC055758.1 non.epi.lncRNA

AC055758.2 non.epi.lncRNA

AC055764.2 non.epi.lncRNA

AC055811.1 non.epi.lncRNA

AC055811.3 non.epi.lncRNA

AC055854.1 non.epi.lncRNA

AC055855.1 non.epi.lncRNA

AC055855.2 non.epi.lncRNA

AC055874.1 non.epi.lncRNA

AC055878.1 non.epi.lncRNA

AC058791.1 non.epi.lncRNA

AC060234.1 non.epi.lncRNA

AC060234.2 non.epi.lncRNA

AC060234.3 non.epi.lncRNA

AC060765.1 non.epi.lncRNA

AC060765.2 non.epi.lncRNA

AC060766.4 non.epi.lncRNA

AC060766.6 non.epi.lncRNA

AC060780.1 non.epi.lncRNA

AC060809.1 non.epi.lncRNA

AC060814.3 non.epi.lncRNA

AC060814.4 non.epi.lncRNA

AC060814.5 non.epi.lncRNA

AC060834.2 non.epi.lncRNA

AC061709.2 non.epi.lncRNA

AC061975.1 non.epi.lncRNA

AC061975.4 non.epi.lncRNA

AC061975.6 non.epi.lncRNA

AC061975.7 non.epi.lncRNA

AC062004.1 non.epi.lncRNA

AC062015.1 non.epi.lncRNA

AC062020.1 non.epi.lncRNA

AC062021.1 non.epi.lncRNA

AC062028.1 non.epi.lncRNA

AC062032.1 non.epi.lncRNA

AC062037.2 non.epi.lncRNA

AC062039.1 non.epi.lncRNA

AC063923.2 non.epi.lncRNA

AC063938.1 non.epi.lncRNA

AC063943.1 non.epi.lncRNA

AC063944.1 non.epi.lncRNA

AC063944.3 non.epi.lncRNA

AC063947.1 non.epi.lncRNA

AC063948.1 non.epi.lncRNA

AC063949.2 non.epi.lncRNA

AC063962.1 non.epi.lncRNA

AC063965.2 non.epi.lncRNA

AC063976.1 non.epi.lncRNA

AC063977.3 non.epi.lncRNA

AC063977.6 non.epi.lncRNA

AC063979.2 non.epi.lncRNA

AC063980.1 non.epi.lncRNA

AC064799.2 non.epi.lncRNA

AC064801.1 non.epi.lncRNA

AC064802.1 non.epi.lncRNA

AC064805.1 non.epi.lncRNA

AC064807.1 non.epi.lncRNA

AC064807.2 non.epi.lncRNA

AC064807.3 non.epi.lncRNA

AC064807.4 non.epi.lncRNA

AC064834.1 non.epi.lncRNA

AC064836.3 non.epi.lncRNA

AC064853.1 non.epi.lncRNA

AC064871.2 non.epi.lncRNA

AC064874.1 non.epi.lncRNA

AC064875.1 non.epi.lncRNA

AC066595.1 non.epi.lncRNA

AC066613.2 non.epi.lncRNA

AC067747.1 non.epi.lncRNA

AC067750.1 non.epi.lncRNA

AC067751.1 non.epi.lncRNA

AC067773.1 non.epi.lncRNA

AC067817.1 non.epi.lncRNA

AC067817.2 non.epi.lncRNA

AC067838.1 non.epi.lncRNA

AC067852.1 non.epi.lncRNA

AC067852.3 non.epi.lncRNA

AC067863.1 non.epi.lncRNA

AC067930.1 non.epi.lncRNA

AC067930.2 non.epi.lncRNA

AC067945.2 non.epi.lncRNA

AC067945.3 non.epi.lncRNA

AC067956.1 non.epi.lncRNA

AC067960.1 non.epi.lncRNA

AC067968.2 non.epi.lncRNA

AC067969.1 non.epi.lncRNA

AC067969.2 non.epi.lncRNA

AC068014.1 non.epi.lncRNA

AC068020.1 non.epi.lncRNA

AC068051.1 non.epi.lncRNA

AC068057.1 non.epi.lncRNA

AC068058.1 non.epi.lncRNA

AC068075.1 non.epi.lncRNA

AC068112.1 non.epi.lncRNA

AC068134.1 non.epi.lncRNA

AC068134.2 non.epi.lncRNA

AC068135.2 non.epi.lncRNA

AC068189.1 non.epi.lncRNA

AC068196.1 non.epi.lncRNA

AC068228.1 non.epi.lncRNA

AC068254.1 non.epi.lncRNA

AC068286.1 non.epi.lncRNA

AC068305.2 non.epi.lncRNA

AC068308.1 non.epi.lncRNA

AC068338.3 non.epi.lncRNA

AC068385.1 non.epi.lncRNA

AC068389.1 non.epi.lncRNA

AC068389.2 non.epi.lncRNA

AC068389.3 non.epi.lncRNA

AC068397.1 non.epi.lncRNA

AC068397.2 non.epi.lncRNA

AC068408.1 non.epi.lncRNA

AC068413.1 non.epi.lncRNA

AC068418.2 non.epi.lncRNA

AC068446.2 non.epi.lncRNA

AC068473.1 non.epi.lncRNA

AC068473.2 non.epi.lncRNA

AC068473.3 non.epi.lncRNA

AC068473.4 non.epi.lncRNA

AC068473.5 non.epi.lncRNA

AC068481.1 non.epi.lncRNA

AC068483.1 non.epi.lncRNA

AC068489.1 non.epi.lncRNA

AC068491.2 non.epi.lncRNA

AC068491.3 non.epi.lncRNA

AC068492.1 non.epi.lncRNA

AC068506.1 non.epi.lncRNA

AC068533.3 non.epi.lncRNA

AC068535.1 non.epi.lncRNA

AC068580.1 non.epi.lncRNA

AC068580.3 non.epi.lncRNA

AC068620.2 non.epi.lncRNA

AC068631.1 non.epi.lncRNA

AC068633.1 non.epi.lncRNA

AC068643.1 non.epi.lncRNA

AC068643.2 non.epi.lncRNA

AC068658.1 non.epi.lncRNA

AC068672.1 non.epi.lncRNA

AC068672.2 non.epi.lncRNA

AC068672.3 non.epi.lncRNA

AC068700.1 non.epi.lncRNA

AC068721.1 non.epi.lncRNA

AC068722.1 non.epi.lncRNA

AC068722.2 non.epi.lncRNA

AC068726.1 non.epi.lncRNA

AC068733.1 non.epi.lncRNA

AC068756.1 non.epi.lncRNA

AC068759.1 non.epi.lncRNA

AC068768.1 non.epi.lncRNA

AC068790.2 non.epi.lncRNA

AC068790.3 non.epi.lncRNA

AC068790.4 non.epi.lncRNA

AC068790.5 non.epi.lncRNA

AC068790.6 non.epi.lncRNA

AC068790.7 non.epi.lncRNA

AC068790.8 non.epi.lncRNA

AC068790.9 non.epi.lncRNA

AC068792.1 non.epi.lncRNA

AC068831.4 non.epi.lncRNA

AC068831.6 non.epi.lncRNA

AC068870.1 non.epi.lncRNA

AC068875.1 non.epi.lncRNA

AC068880.2 non.epi.lncRNA

AC068880.3 non.epi.lncRNA

AC068888.1 non.epi.lncRNA

AC068898.2 non.epi.lncRNA

AC068944.1 non.epi.lncRNA

AC068985.1 non.epi.lncRNA

AC068987.1 non.epi.lncRNA

AC068987.3 non.epi.lncRNA

AC068993.2 non.epi.lncRNA

AC069023.1 non.epi.lncRNA

AC069061.2 non.epi.lncRNA

AC069079.1 non.epi.lncRNA

AC069113.2 non.epi.lncRNA

AC069114.1 non.epi.lncRNA

AC069120.1 non.epi.lncRNA

AC069133.1 non.epi.lncRNA

AC069148.1 non.epi.lncRNA

AC069155.1 non.epi.lncRNA

AC069185.1 non.epi.lncRNA

AC069200.1 non.epi.lncRNA

AC069209.1 non.epi.lncRNA

AC069213.1 non.epi.lncRNA

AC069222.1 non.epi.lncRNA

AC069224.1 non.epi.lncRNA

AC069228.1 non.epi.lncRNA

AC069234.1 non.epi.lncRNA

AC069234.2 non.epi.lncRNA

AC069234.4 non.epi.lncRNA

AC069234.5 non.epi.lncRNA

AC069257.1 non.epi.lncRNA

AC069272.1 non.epi.lncRNA

AC069277.1 non.epi.lncRNA

AC069280.1 non.epi.lncRNA

AC069281.1 non.epi.lncRNA

AC069281.2 non.epi.lncRNA

AC069285.2 non.epi.lncRNA

AC069287.1 non.epi.lncRNA

AC069287.2 non.epi.lncRNA

AC069287.3 non.epi.lncRNA

AC069307.1 non.epi.lncRNA

AC069366.2 non.epi.lncRNA

AC069431.1 non.epi.lncRNA

AC069437.1 non.epi.lncRNA

AC069439.2 non.epi.lncRNA

AC069444.1 non.epi.lncRNA

AC069503.1 non.epi.lncRNA

AC069503.3 non.epi.lncRNA

AC069542.1 non.epi.lncRNA

AC069544.1 non.epi.lncRNA

AC069549.1 non.epi.lncRNA

AC072022.1 non.epi.lncRNA

AC072039.2 non.epi.lncRNA

AC072061.1 non.epi.lncRNA

AC072062.1 non.epi.lncRNA

AC073023.1 non.epi.lncRNA

AC073050.1 non.epi.lncRNA

AC073062.1 non.epi.lncRNA

AC073071.1 non.epi.lncRNA

AC073072.1 non.epi.lncRNA

AC073073.2 non.epi.lncRNA

AC073094.1 non.epi.lncRNA

AC073114.1 non.epi.lncRNA

AC073115.1 non.epi.lncRNA

AC073115.2 non.epi.lncRNA

AC073127.1 non.epi.lncRNA

AC073130.1 non.epi.lncRNA

AC073130.2 non.epi.lncRNA

AC073167.1 non.epi.lncRNA

AC073172.1 non.epi.lncRNA

AC073176.1 non.epi.lncRNA

AC073188.2 non.epi.lncRNA

AC073188.3 non.epi.lncRNA

AC073188.4 non.epi.lncRNA

AC073188.5 non.epi.lncRNA

AC073188.6 non.epi.lncRNA

AC073218.1 non.epi.lncRNA

AC073257.1 non.epi.lncRNA

AC073257.2 non.epi.lncRNA

AC073263.1 non.epi.lncRNA

AC073263.2 non.epi.lncRNA

AC073284.1 non.epi.lncRNA

AC073288.2 non.epi.lncRNA

AC073311.1 non.epi.lncRNA

AC073314.1 non.epi.lncRNA

AC073316.1 non.epi.lncRNA

AC073320.1 non.epi.lncRNA

AC073321.1 non.epi.lncRNA

AC073323.1 non.epi.lncRNA

AC073325.1 non.epi.lncRNA

AC073326.1 non.epi.lncRNA

AC073332.1 non.epi.lncRNA

AC073333.1 non.epi.lncRNA

AC073336.1 non.epi.lncRNA

AC073342.1 non.epi.lncRNA

AC073343.2 non.epi.lncRNA

AC073346.1 non.epi.lncRNA

AC073352.1 non.epi.lncRNA

AC073359.1 non.epi.lncRNA

AC073359.2 non.epi.lncRNA

AC073365.1 non.epi.lncRNA

AC073367.1 non.epi.lncRNA

AC073389.1 non.epi.lncRNA

AC073389.3 non.epi.lncRNA

AC073409.1 non.epi.lncRNA

AC073429.1 non.epi.lncRNA

AC073429.2 non.epi.lncRNA

AC073475.1 non.epi.lncRNA

AC073476.3 non.epi.lncRNA

AC073487.1 non.epi.lncRNA

AC073525.1 non.epi.lncRNA

AC073529.1 non.epi.lncRNA

AC073530.1 non.epi.lncRNA

AC073534.2 non.epi.lncRNA

AC073539.1 non.epi.lncRNA

AC073539.3 non.epi.lncRNA

AC073569.1 non.epi.lncRNA

AC073569.3 non.epi.lncRNA

AC073571.1 non.epi.lncRNA

AC073578.1 non.epi.lncRNA

AC073578.2 non.epi.lncRNA

AC073591.1 non.epi.lncRNA

AC073611.1 non.epi.lncRNA

AC073626.1 non.epi.lncRNA

AC073636.1 non.epi.lncRNA

AC073651.1 non.epi.lncRNA

AC073655.1 non.epi.lncRNA

AC073655.2 non.epi.lncRNA

AC073657.1 non.epi.lncRNA

AC073834.1 non.epi.lncRNA

AC073842.1 non.epi.lncRNA

AC073848.1 non.epi.lncRNA

AC073862.1 non.epi.lncRNA

AC073863.1 non.epi.lncRNA

AC073864.1 non.epi.lncRNA

AC073869.3 non.epi.lncRNA

AC073878.2 non.epi.lncRNA

AC073896.2 non.epi.lncRNA

AC073896.3 non.epi.lncRNA

AC073896.4 non.epi.lncRNA

AC073912.1 non.epi.lncRNA

AC073934.1 non.epi.lncRNA

AC073941.1 non.epi.lncRNA

AC073957.2 non.epi.lncRNA

AC073957.3 non.epi.lncRNA

AC073964.1 non.epi.lncRNA

AC073987.1 non.epi.lncRNA

AC074011.1 non.epi.lncRNA

AC074029.3 non.epi.lncRNA

AC074031.1 non.epi.lncRNA

AC074032.1 non.epi.lncRNA

AC074035.1 non.epi.lncRNA

AC074044.1 non.epi.lncRNA

AC074050.2 non.epi.lncRNA

AC074050.3 non.epi.lncRNA

AC074050.4 non.epi.lncRNA

AC074052.2 non.epi.lncRNA

AC074091.2 non.epi.lncRNA

AC074117.1 non.epi.lncRNA

AC074124.1 non.epi.lncRNA

AC074131.1 non.epi.lncRNA

AC074135.1 non.epi.lncRNA

AC074135.2 non.epi.lncRNA

AC074183.1 non.epi.lncRNA

AC074237.1 non.epi.lncRNA

AC074254.1 non.epi.lncRNA

AC074257.1 non.epi.lncRNA

AC074286.1 non.epi.lncRNA

AC074344.2 non.epi.lncRNA

AC074351.1 non.epi.lncRNA

AC074366.1 non.epi.lncRNA

AC074389.1 non.epi.lncRNA

AC074389.2 non.epi.lncRNA

AC074389.3 non.epi.lncRNA

AC076966.2 non.epi.lncRNA

AC077690.1 non.epi.lncRNA

AC078776.1 non.epi.lncRNA

AC078777.1 non.epi.lncRNA

AC078778.1 non.epi.lncRNA

AC078785.1 non.epi.lncRNA

AC078788.1 non.epi.lncRNA

AC078788.2 non.epi.lncRNA

AC078789.1 non.epi.lncRNA

AC078795.2 non.epi.lncRNA

AC078795.3 non.epi.lncRNA

AC078802.1 non.epi.lncRNA

AC078814.1 non.epi.lncRNA

AC078828.1 non.epi.lncRNA

AC078842.1 non.epi.lncRNA

AC078842.2 non.epi.lncRNA

AC078845.1 non.epi.lncRNA

AC078846.1 non.epi.lncRNA

AC078850.1 non.epi.lncRNA

AC078850.2 non.epi.lncRNA

AC078851.1 non.epi.lncRNA

AC078852.1 non.epi.lncRNA

AC078852.2 non.epi.lncRNA

AC078860.2 non.epi.lncRNA

AC078865.1 non.epi.lncRNA

AC078878.2 non.epi.lncRNA

AC078880.1 non.epi.lncRNA

AC078880.2 non.epi.lncRNA

AC078880.3 non.epi.lncRNA

AC078880.4 non.epi.lncRNA

AC078880.5 non.epi.lncRNA

AC078881.1 non.epi.lncRNA

AC078882.1 non.epi.lncRNA

AC078883.1 non.epi.lncRNA

AC078905.1 non.epi.lncRNA

AC078906.1 non.epi.lncRNA

AC078909.1 non.epi.lncRNA

AC078922.1 non.epi.lncRNA

AC078923.1 non.epi.lncRNA

AC078925.1 non.epi.lncRNA

AC078942.1 non.epi.lncRNA

AC078950.1 non.epi.lncRNA

AC078955.1 non.epi.lncRNA

AC078962.2 non.epi.lncRNA

AC078980.1 non.epi.lncRNA

AC078993.1 non.epi.lncRNA

AC079015.1 non.epi.lncRNA

AC079031.1 non.epi.lncRNA

AC079031.2 non.epi.lncRNA

AC079035.1 non.epi.lncRNA

AC079054.1 non.epi.lncRNA

AC079061.1 non.epi.lncRNA

AC079062.1 non.epi.lncRNA

AC079070.1 non.epi.lncRNA

AC079075.1 non.epi.lncRNA

AC079089.1 non.epi.lncRNA

AC079117.1 non.epi.lncRNA

AC079145.1 non.epi.lncRNA

AC079148.1 non.epi.lncRNA

AC079154.1 non.epi.lncRNA

AC079160.1 non.epi.lncRNA

AC079163.1 non.epi.lncRNA

AC079174.1 non.epi.lncRNA

AC079174.2 non.epi.lncRNA

AC079193.2 non.epi.lncRNA

AC079203.2 non.epi.lncRNA

AC079209.1 non.epi.lncRNA

AC079209.2 non.epi.lncRNA

AC079210.1 non.epi.lncRNA

AC079226.1 non.epi.lncRNA

AC079226.2 non.epi.lncRNA

AC079248.1 non.epi.lncRNA

AC079296.1 non.epi.lncRNA

AC079298.1 non.epi.lncRNA

AC079298.2 non.epi.lncRNA

AC079298.3 non.epi.lncRNA

AC079305.1 non.epi.lncRNA

AC079305.3 non.epi.lncRNA

AC079310.1 non.epi.lncRNA

AC079313.1 non.epi.lncRNA

AC079313.2 non.epi.lncRNA

AC079322.1 non.epi.lncRNA

AC079329.1 non.epi.lncRNA

AC079336.3 non.epi.lncRNA

AC079336.5 non.epi.lncRNA

AC079340.1 non.epi.lncRNA

AC079340.2 non.epi.lncRNA

AC079341.1 non.epi.lncRNA

AC079341.2 non.epi.lncRNA

AC079349.1 non.epi.lncRNA

AC079352.1 non.epi.lncRNA

AC079360.1 non.epi.lncRNA

AC079362.1 non.epi.lncRNA

AC079380.1 non.epi.lncRNA

AC079384.1 non.epi.lncRNA

AC079385.1 non.epi.lncRNA

AC079385.2 non.epi.lncRNA

AC079385.3 non.epi.lncRNA

AC079411.1 non.epi.lncRNA

AC079414.1 non.epi.lncRNA

AC079465.1 non.epi.lncRNA

AC079466.1 non.epi.lncRNA

AC079466.2 non.epi.lncRNA

AC079584.1 non.epi.lncRNA

AC079584.2 non.epi.lncRNA

AC079598.1 non.epi.lncRNA

AC079601.1 non.epi.lncRNA

AC079610.1 non.epi.lncRNA

AC079610.2 non.epi.lncRNA

AC079612.1 non.epi.lncRNA

AC079612.2 non.epi.lncRNA

AC079630.1 non.epi.lncRNA

AC079684.1 non.epi.lncRNA

AC079742.1 non.epi.lncRNA

AC079760.1 non.epi.lncRNA

AC079760.2 non.epi.lncRNA

AC079763.1 non.epi.lncRNA

AC079766.1 non.epi.lncRNA

AC079779.2 non.epi.lncRNA

AC079779.3 non.epi.lncRNA

AC079779.4 non.epi.lncRNA

AC079790.1 non.epi.lncRNA

AC079793.1 non.epi.lncRNA

AC079799.1 non.epi.lncRNA

AC079801.1 non.epi.lncRNA

AC079807.1 non.epi.lncRNA

AC079834.2 non.epi.lncRNA

AC079858.1 non.epi.lncRNA

AC079866.2 non.epi.lncRNA

AC079896.1 non.epi.lncRNA

AC079906.1 non.epi.lncRNA

AC079907.2 non.epi.lncRNA

AC079910.1 non.epi.lncRNA

AC079915.1 non.epi.lncRNA

AC079921.1 non.epi.lncRNA

AC079921.2 non.epi.lncRNA

AC079942.1 non.epi.lncRNA

AC079943.1 non.epi.lncRNA

AC079943.2 non.epi.lncRNA

AC079949.1 non.epi.lncRNA

AC079949.2 non.epi.lncRNA

AC079950.1 non.epi.lncRNA

AC079988.1 non.epi.lncRNA

AC080011.1 non.epi.lncRNA

AC080013.5 non.epi.lncRNA

AC080013.6 non.epi.lncRNA

AC080023.1 non.epi.lncRNA

AC080037.2 non.epi.lncRNA

AC080038.2 non.epi.lncRNA

AC080075.1 non.epi.lncRNA

AC080078.1 non.epi.lncRNA

AC080078.2 non.epi.lncRNA

AC080112.1 non.epi.lncRNA

AC080129.1 non.epi.lncRNA

AC080129.2 non.epi.lncRNA

AC082650.1 non.epi.lncRNA

AC082651.1 non.epi.lncRNA

AC082651.3 non.epi.lncRNA

AC082651.4 non.epi.lncRNA

AC083795.2 non.epi.lncRNA

AC083799.1 non.epi.lncRNA

AC083801.2 non.epi.lncRNA

AC083806.3 non.epi.lncRNA

AC083809.1 non.epi.lncRNA

AC083836.1 non.epi.lncRNA

AC083841.1 non.epi.lncRNA

AC083841.2 non.epi.lncRNA

AC083841.3 non.epi.lncRNA

AC083843.1 non.epi.lncRNA

AC083843.3 non.epi.lncRNA

AC083864.1 non.epi.lncRNA

AC083867.2 non.epi.lncRNA

AC083900.1 non.epi.lncRNA

AC083902.1 non.epi.lncRNA

AC083949.1 non.epi.lncRNA

AC083964.1 non.epi.lncRNA

AC083967.1 non.epi.lncRNA

AC083973.1 non.epi.lncRNA

AC084018.1 non.epi.lncRNA

AC084024.1 non.epi.lncRNA

AC084024.3 non.epi.lncRNA

AC084026.1 non.epi.lncRNA

AC084026.2 non.epi.lncRNA

AC084030.1 non.epi.lncRNA

AC084036.1 non.epi.lncRNA

AC084048.1 non.epi.lncRNA

AC084064.1 non.epi.lncRNA

AC084082.1 non.epi.lncRNA

AC084083.1 non.epi.lncRNA

AC084116.1 non.epi.lncRNA

AC084116.2 non.epi.lncRNA

AC084116.3 non.epi.lncRNA

AC084125.1 non.epi.lncRNA

AC084125.2 non.epi.lncRNA

AC084125.3 non.epi.lncRNA

AC084125.4 non.epi.lncRNA

AC084128.1 non.epi.lncRNA

AC084149.1 non.epi.lncRNA

AC084211.1 non.epi.lncRNA

AC084262.1 non.epi.lncRNA

AC084291.1 non.epi.lncRNA

AC084346.1 non.epi.lncRNA

AC084357.2 non.epi.lncRNA

AC084361.1 non.epi.lncRNA

AC084365.1 non.epi.lncRNA

AC084375.1 non.epi.lncRNA

AC084398.2 non.epi.lncRNA

AC084706.1 non.epi.lncRNA

AC084734.1 non.epi.lncRNA

AC084756.1 non.epi.lncRNA

AC084757.2 non.epi.lncRNA

AC084757.3 non.epi.lncRNA

AC084757.4 non.epi.lncRNA

AC084768.1 non.epi.lncRNA

AC084781.1 non.epi.lncRNA

AC084781.2 non.epi.lncRNA

AC084782.1 non.epi.lncRNA

AC084782.2 non.epi.lncRNA

AC084782.3 non.epi.lncRNA

AC084809.1 non.epi.lncRNA

AC084809.2 non.epi.lncRNA

AC084816.1 non.epi.lncRNA

AC084819.1 non.epi.lncRNA

AC084824.3 non.epi.lncRNA

AC084838.1 non.epi.lncRNA

AC084855.1 non.epi.lncRNA

AC084855.2 non.epi.lncRNA

AC084864.1 non.epi.lncRNA

AC084866.1 non.epi.lncRNA

AC084866.2 non.epi.lncRNA

AC084871.1 non.epi.lncRNA

AC084876.1 non.epi.lncRNA

AC084879.2 non.epi.lncRNA

AC084880.2 non.epi.lncRNA

AC084880.3 non.epi.lncRNA

AC084880.4 non.epi.lncRNA

AC084882.1 non.epi.lncRNA

AC087045.2 non.epi.lncRNA

AC087071.1 non.epi.lncRNA

AC087071.2 non.epi.lncRNA

AC087190.1 non.epi.lncRNA

AC087190.3 non.epi.lncRNA

AC087203.3 non.epi.lncRNA

AC087222.1 non.epi.lncRNA

AC087235.1 non.epi.lncRNA

AC087235.2 non.epi.lncRNA

AC087239.1 non.epi.lncRNA

AC087241.2 non.epi.lncRNA

AC087242.1 non.epi.lncRNA

AC087260.1 non.epi.lncRNA

AC087269.1 non.epi.lncRNA

AC087273.1 non.epi.lncRNA

AC087273.2 non.epi.lncRNA

AC087276.2 non.epi.lncRNA

AC087276.3 non.epi.lncRNA

AC087277.1 non.epi.lncRNA

AC087277.2 non.epi.lncRNA

AC087284.1 non.epi.lncRNA

AC087286.1 non.epi.lncRNA

AC087286.2 non.epi.lncRNA

AC087286.3 non.epi.lncRNA

AC087286.4 non.epi.lncRNA

AC087289.1 non.epi.lncRNA

AC087289.2 non.epi.lncRNA

AC087289.4 non.epi.lncRNA

AC087289.5 non.epi.lncRNA

AC087311.2 non.epi.lncRNA

AC087318.1 non.epi.lncRNA

AC087341.1 non.epi.lncRNA

AC087354.1 non.epi.lncRNA

AC087362.1 non.epi.lncRNA

AC087362.2 non.epi.lncRNA

AC087379.1 non.epi.lncRNA

AC087379.2 non.epi.lncRNA

AC087386.1 non.epi.lncRNA

AC087392.2 non.epi.lncRNA

AC087392.4 non.epi.lncRNA

AC087392.5 non.epi.lncRNA

AC087393.2 non.epi.lncRNA

AC087399.1 non.epi.lncRNA

AC087430.1 non.epi.lncRNA

AC087439.2 non.epi.lncRNA

AC087442.1 non.epi.lncRNA

AC087457.1 non.epi.lncRNA

AC087463.1 non.epi.lncRNA

AC087463.2 non.epi.lncRNA

AC087473.1 non.epi.lncRNA

AC087477.2 non.epi.lncRNA

AC087477.3 non.epi.lncRNA

AC087477.5 non.epi.lncRNA

AC087482.1 non.epi.lncRNA

AC087500.2 non.epi.lncRNA

AC087501.1 non.epi.lncRNA

AC087501.2 non.epi.lncRNA

AC087501.3 non.epi.lncRNA

AC087501.4 non.epi.lncRNA

AC087516.1 non.epi.lncRNA

AC087516.2 non.epi.lncRNA

AC087518.1 non.epi.lncRNA

AC087521.1 non.epi.lncRNA

AC087521.3 non.epi.lncRNA

AC087565.1 non.epi.lncRNA

AC087588.2 non.epi.lncRNA

AC087623.1 non.epi.lncRNA

AC087623.2 non.epi.lncRNA

AC087623.3 non.epi.lncRNA

AC087627.1 non.epi.lncRNA

AC087633.1 non.epi.lncRNA

AC087636.1 non.epi.lncRNA

AC087664.2 non.epi.lncRNA

AC087667.1 non.epi.lncRNA

AC087683.2 non.epi.lncRNA

AC087721.1 non.epi.lncRNA

AC087741.2 non.epi.lncRNA

AC087742.1 non.epi.lncRNA

AC087749.1 non.epi.lncRNA

AC087749.2 non.epi.lncRNA

AC087752.4 non.epi.lncRNA

AC087761.1 non.epi.lncRNA

AC087762.1 non.epi.lncRNA

AC087854.1 non.epi.lncRNA

AC087855.1 non.epi.lncRNA

AC087855.2 non.epi.lncRNA

AC087857.1 non.epi.lncRNA

AC087863.2 non.epi.lncRNA

AC087878.1 non.epi.lncRNA

AC087893.2 non.epi.lncRNA

AC087897.2 non.epi.lncRNA

AC089984.1 non.epi.lncRNA

AC089987.2 non.epi.lncRNA

AC089998.1 non.epi.lncRNA

AC089998.2 non.epi.lncRNA

AC089998.4 non.epi.lncRNA

AC089999.1 non.epi.lncRNA

AC089999.2 non.epi.lncRNA

AC090001.1 non.epi.lncRNA

AC090015.1 non.epi.lncRNA

AC090044.1 non.epi.lncRNA

AC090049.1 non.epi.lncRNA

AC090099.1 non.epi.lncRNA

AC090103.1 non.epi.lncRNA

AC090109.1 non.epi.lncRNA

AC090115.1 non.epi.lncRNA

AC090116.1 non.epi.lncRNA

AC090124.1 non.epi.lncRNA

AC090125.1 non.epi.lncRNA

AC090136.3 non.epi.lncRNA

AC090138.1 non.epi.lncRNA

AC090150.1 non.epi.lncRNA

AC090150.2 non.epi.lncRNA

AC090152.1 non.epi.lncRNA

AC090155.1 non.epi.lncRNA

AC090155.2 non.epi.lncRNA

AC090159.1 non.epi.lncRNA

AC090164.3 non.epi.lncRNA

AC090181.1 non.epi.lncRNA

AC090181.2 non.epi.lncRNA

AC090186.1 non.epi.lncRNA

AC090193.1 non.epi.lncRNA

AC090197.1 non.epi.lncRNA

AC090200.1 non.epi.lncRNA

AC090204.1 non.epi.lncRNA

AC090206.1 non.epi.lncRNA

AC090213.1 non.epi.lncRNA

AC090220.1 non.epi.lncRNA

AC090227.3 non.epi.lncRNA

AC090229.1 non.epi.lncRNA

AC090236.1 non.epi.lncRNA

AC090236.2 non.epi.lncRNA

AC090241.2 non.epi.lncRNA

AC090241.3 non.epi.lncRNA

AC090246.1 non.epi.lncRNA

AC090260.1 non.epi.lncRNA

AC090282.1 non.epi.lncRNA

AC090283.1 non.epi.lncRNA

AC090286.1 non.epi.lncRNA

AC090337.1 non.epi.lncRNA

AC090340.1 non.epi.lncRNA

AC090358.1 non.epi.lncRNA

AC090365.1 non.epi.lncRNA

AC090371.1 non.epi.lncRNA

AC090371.2 non.epi.lncRNA

AC090376.1 non.epi.lncRNA

AC090377.1 non.epi.lncRNA

AC090386.1 non.epi.lncRNA

AC090398.1 non.epi.lncRNA

AC090403.1 non.epi.lncRNA

AC090409.2 non.epi.lncRNA

AC090415.2 non.epi.lncRNA

AC090425.2 non.epi.lncRNA

AC090425.3 non.epi.lncRNA

AC090457.1 non.epi.lncRNA

AC090502.2 non.epi.lncRNA

AC090503.1 non.epi.lncRNA

AC090503.2 non.epi.lncRNA

AC090505.2 non.epi.lncRNA

AC090506.1 non.epi.lncRNA

AC090510.1 non.epi.lncRNA

AC090510.2 non.epi.lncRNA

AC090510.3 non.epi.lncRNA

AC090515.2 non.epi.lncRNA

AC090515.4 non.epi.lncRNA

AC090515.5 non.epi.lncRNA

AC090517.2 non.epi.lncRNA

AC090518.1 non.epi.lncRNA

AC090519.2 non.epi.lncRNA

AC090527.1 non.epi.lncRNA

AC090527.3 non.epi.lncRNA

AC090531.1 non.epi.lncRNA

AC090539.1 non.epi.lncRNA

AC090541.1 non.epi.lncRNA

AC090572.2 non.epi.lncRNA

AC090572.3 non.epi.lncRNA

AC090578.1 non.epi.lncRNA

AC090578.2 non.epi.lncRNA

AC090579.1 non.epi.lncRNA

AC090589.2 non.epi.lncRNA

AC090589.3 non.epi.lncRNA

AC090607.1 non.epi.lncRNA

AC090607.4 non.epi.lncRNA

AC090607.5 non.epi.lncRNA

AC090617.1 non.epi.lncRNA

AC090617.2 non.epi.lncRNA

AC090617.4 non.epi.lncRNA

AC090618.1 non.epi.lncRNA

AC090621.1 non.epi.lncRNA

AC090625.2 non.epi.lncRNA

AC090630.1 non.epi.lncRNA

AC090651.1 non.epi.lncRNA

AC090679.2 non.epi.lncRNA

AC090680.1 non.epi.lncRNA

AC090692.1 non.epi.lncRNA

AC090696.1 non.epi.lncRNA

AC090707.1 non.epi.lncRNA

AC090709.1 non.epi.lncRNA

AC090735.1 non.epi.lncRNA

AC090739.1 non.epi.lncRNA

AC090753.1 non.epi.lncRNA

AC090771.2 non.epi.lncRNA

AC090772.1 non.epi.lncRNA

AC090772.2 non.epi.lncRNA

AC090772.3 non.epi.lncRNA

AC090774.2 non.epi.lncRNA

AC090791.1 non.epi.lncRNA

AC090796.1 non.epi.lncRNA

AC090802.1 non.epi.lncRNA

AC090809.1 non.epi.lncRNA

AC090821.2 non.epi.lncRNA

AC090825.1 non.epi.lncRNA

AC090844.2 non.epi.lncRNA

AC090844.3 non.epi.lncRNA

AC090857.2 non.epi.lncRNA

AC090877.2 non.epi.lncRNA

AC090907.2 non.epi.lncRNA

AC090912.1 non.epi.lncRNA

AC090912.2 non.epi.lncRNA

AC090912.3 non.epi.lncRNA

AC090948.1 non.epi.lncRNA

AC090948.2 non.epi.lncRNA

AC090948.3 non.epi.lncRNA

AC090952.1 non.epi.lncRNA

AC090957.1 non.epi.lncRNA

AC090970.1 non.epi.lncRNA

AC090970.2 non.epi.lncRNA

AC090971.5 non.epi.lncRNA

AC090983.1 non.epi.lncRNA

AC090985.2 non.epi.lncRNA

AC090987.1 non.epi.lncRNA

AC090993.1 non.epi.lncRNA

AC091027.1 non.epi.lncRNA

AC091027.2 non.epi.lncRNA

AC091043.1 non.epi.lncRNA

AC091044.1 non.epi.lncRNA

AC091045.1 non.epi.lncRNA

AC091053.1 non.epi.lncRNA

AC091057.1 non.epi.lncRNA

AC091057.3 non.epi.lncRNA

AC091059.1 non.epi.lncRNA

AC091062.1 non.epi.lncRNA

AC091073.1 non.epi.lncRNA

AC091078.1 non.epi.lncRNA

AC091078.2 non.epi.lncRNA

AC091096.1 non.epi.lncRNA

AC091100.1 non.epi.lncRNA

AC091114.1 non.epi.lncRNA

AC091117.1 non.epi.lncRNA

AC091132.1 non.epi.lncRNA

AC091132.2 non.epi.lncRNA

AC091132.4 non.epi.lncRNA

AC091132.5 non.epi.lncRNA

AC091133.1 non.epi.lncRNA

AC091133.3 non.epi.lncRNA

AC091133.4 non.epi.lncRNA

AC091138.1 non.epi.lncRNA

AC091151.1 non.epi.lncRNA

AC091152.2 non.epi.lncRNA

AC091153.2 non.epi.lncRNA

AC091153.3 non.epi.lncRNA

AC091153.4 non.epi.lncRNA

AC091163.1 non.epi.lncRNA

AC091163.2 non.epi.lncRNA

AC091167.5 non.epi.lncRNA

AC091179.1 non.epi.lncRNA

AC091180.2 non.epi.lncRNA

AC091180.3 non.epi.lncRNA

AC091180.4 non.epi.lncRNA

AC091180.5 non.epi.lncRNA

AC091182.1 non.epi.lncRNA

AC091182.2 non.epi.lncRNA

AC091185.1 non.epi.lncRNA

AC091198.1 non.epi.lncRNA

AC091212.1 non.epi.lncRNA

AC091230.1 non.epi.lncRNA

AC091231.1 non.epi.lncRNA

AC091435.2 non.epi.lncRNA

AC091488.1 non.epi.lncRNA

AC091489.1 non.epi.lncRNA

AC091493.1 non.epi.lncRNA

AC091534.1 non.epi.lncRNA

AC091544.5 non.epi.lncRNA

AC091544.6 non.epi.lncRNA

AC091563.1 non.epi.lncRNA

AC091564.2 non.epi.lncRNA

AC091564.4 non.epi.lncRNA

AC091564.5 non.epi.lncRNA

AC091564.6 non.epi.lncRNA

AC091588.1 non.epi.lncRNA

AC091588.2 non.epi.lncRNA

AC091588.3 non.epi.lncRNA

AC091614.1 non.epi.lncRNA

AC091685.1 non.epi.lncRNA

AC091691.2 non.epi.lncRNA

AC091705.1 non.epi.lncRNA

AC091729.1 non.epi.lncRNA

AC091729.3 non.epi.lncRNA

AC091730.1 non.epi.lncRNA

AC091736.1 non.epi.lncRNA

AC091770.1 non.epi.lncRNA

AC091806.1 non.epi.lncRNA

AC091814.1 non.epi.lncRNA

AC091819.1 non.epi.lncRNA

AC091819.2 non.epi.lncRNA

AC091820.1 non.epi.lncRNA

AC091820.2 non.epi.lncRNA

AC091826.2 non.epi.lncRNA

AC091832.1 non.epi.lncRNA

AC091849.2 non.epi.lncRNA

AC091860.2 non.epi.lncRNA

AC091868.2 non.epi.lncRNA

AC091885.2 non.epi.lncRNA

AC091887.1 non.epi.lncRNA

AC091891.1 non.epi.lncRNA

AC091906.1 non.epi.lncRNA

AC091917.1 non.epi.lncRNA

AC091917.2 non.epi.lncRNA

AC091917.3 non.epi.lncRNA

AC091932.1 non.epi.lncRNA

AC091939.1 non.epi.lncRNA

AC091944.1 non.epi.lncRNA

AC091946.1 non.epi.lncRNA

AC091946.2 non.epi.lncRNA

AC091953.1 non.epi.lncRNA

AC091962.1 non.epi.lncRNA

AC091965.1 non.epi.lncRNA

AC091965.4 non.epi.lncRNA

AC091979.1 non.epi.lncRNA

AC091979.2 non.epi.lncRNA

AC091980.2 non.epi.lncRNA

AC091982.1 non.epi.lncRNA

AC091982.3 non.epi.lncRNA

AC091987.1 non.epi.lncRNA

AC092040.1 non.epi.lncRNA

AC092042.1 non.epi.lncRNA

AC092059.1 non.epi.lncRNA

AC092067.1 non.epi.lncRNA

AC092068.1 non.epi.lncRNA

AC092068.3 non.epi.lncRNA

AC092069.1 non.epi.lncRNA

AC092070.3 non.epi.lncRNA

AC092070.4 non.epi.lncRNA

AC092071.1 non.epi.lncRNA

AC092078.1 non.epi.lncRNA

AC092078.2 non.epi.lncRNA

AC092100.1 non.epi.lncRNA

AC092111.1 non.epi.lncRNA

AC092111.2 non.epi.lncRNA

AC092114.1 non.epi.lncRNA

AC092115.2 non.epi.lncRNA

AC092118.1 non.epi.lncRNA

AC092118.2 non.epi.lncRNA

AC092119.3 non.epi.lncRNA

AC092120.1 non.epi.lncRNA

AC092123.1 non.epi.lncRNA

AC092125.1 non.epi.lncRNA

AC092130.1 non.epi.lncRNA

AC092131.1 non.epi.lncRNA

AC092132.1 non.epi.lncRNA

AC092134.1 non.epi.lncRNA

AC092138.1 non.epi.lncRNA

AC092138.2 non.epi.lncRNA

AC092139.1 non.epi.lncRNA

AC092140.1 non.epi.lncRNA

AC092140.2 non.epi.lncRNA

AC092142.1 non.epi.lncRNA

AC092142.2 non.epi.lncRNA

AC092143.2 non.epi.lncRNA

AC092143.3 non.epi.lncRNA

AC092155.1 non.epi.lncRNA

AC092159.1 non.epi.lncRNA

AC092159.3 non.epi.lncRNA

AC092162.2 non.epi.lncRNA

AC092164.1 non.epi.lncRNA

AC092168.1 non.epi.lncRNA

AC092168.2 non.epi.lncRNA

AC092171.2 non.epi.lncRNA

AC092171.3 non.epi.lncRNA

AC092171.4 non.epi.lncRNA

AC092171.5 non.epi.lncRNA

AC092198.1 non.epi.lncRNA

AC092279.1 non.epi.lncRNA

AC092295.1 non.epi.lncRNA

AC092296.2 non.epi.lncRNA

AC092296.4 non.epi.lncRNA

AC092301.1 non.epi.lncRNA

AC092316.1 non.epi.lncRNA

AC092325.1 non.epi.lncRNA

AC092327.2 non.epi.lncRNA

AC092329.1 non.epi.lncRNA

AC092332.1 non.epi.lncRNA

AC092335.1 non.epi.lncRNA

AC092336.1 non.epi.lncRNA

AC092337.1 non.epi.lncRNA

AC092338.1 non.epi.lncRNA

AC092343.1 non.epi.lncRNA

AC092353.1 non.epi.lncRNA

AC092354.1 non.epi.lncRNA

AC092354.2 non.epi.lncRNA

AC092364.1 non.epi.lncRNA

AC092368.3 non.epi.lncRNA

AC092375.2 non.epi.lncRNA

AC092376.1 non.epi.lncRNA

AC092376.2 non.epi.lncRNA

AC092378.1 non.epi.lncRNA

AC092384.1 non.epi.lncRNA

AC092384.2 non.epi.lncRNA

AC092384.3 non.epi.lncRNA

AC092422.1 non.epi.lncRNA

AC092435.1 non.epi.lncRNA

AC092435.2 non.epi.lncRNA

AC092435.3 non.epi.lncRNA

AC092436.3 non.epi.lncRNA

AC092440.1 non.epi.lncRNA

AC092447.4 non.epi.lncRNA

AC092447.5 non.epi.lncRNA

AC092447.8 non.epi.lncRNA

AC092468.1 non.epi.lncRNA

AC092484.1 non.epi.lncRNA

AC092490.1 non.epi.lncRNA

AC092490.2 non.epi.lncRNA

AC092535.2 non.epi.lncRNA

AC092535.4 non.epi.lncRNA

AC092542.1 non.epi.lncRNA

AC092546.1 non.epi.lncRNA

AC092567.1 non.epi.lncRNA

AC092574.2 non.epi.lncRNA

AC092580.2 non.epi.lncRNA

AC092590.1 non.epi.lncRNA

AC092598.1 non.epi.lncRNA

AC092608.1 non.epi.lncRNA

AC092611.1 non.epi.lncRNA

AC092620.1 non.epi.lncRNA

AC092620.2 non.epi.lncRNA

AC092625.1 non.epi.lncRNA

AC092634.4 non.epi.lncRNA

AC092637.1 non.epi.lncRNA

AC092650.1 non.epi.lncRNA

AC092652.1 non.epi.lncRNA

AC092652.2 non.epi.lncRNA

AC092653.1 non.epi.lncRNA

AC092660.1 non.epi.lncRNA

AC092666.1 non.epi.lncRNA

AC092667.1 non.epi.lncRNA

AC092669.1 non.epi.lncRNA

AC092673.1 non.epi.lncRNA

AC092674.1 non.epi.lncRNA

AC092675.1 non.epi.lncRNA

AC092675.2 non.epi.lncRNA

AC092681.2 non.epi.lncRNA

AC092681.3 non.epi.lncRNA

AC092683.1 non.epi.lncRNA

AC092683.2 non.epi.lncRNA

AC092687.3 non.epi.lncRNA

AC092691.1 non.epi.lncRNA

AC092691.3 non.epi.lncRNA

AC092718.1 non.epi.lncRNA

AC092718.2 non.epi.lncRNA

AC092718.4 non.epi.lncRNA

AC092718.5 non.epi.lncRNA

AC092718.6 non.epi.lncRNA

AC092720.1 non.epi.lncRNA

AC092720.2 non.epi.lncRNA

AC092723.1 non.epi.lncRNA

AC092725.1 non.epi.lncRNA

AC092745.1 non.epi.lncRNA

AC092746.1 non.epi.lncRNA

AC092755.1 non.epi.lncRNA

AC092755.2 non.epi.lncRNA

AC092757.3 non.epi.lncRNA

AC092794.1 non.epi.lncRNA

AC092794.2 non.epi.lncRNA

AC092800.1 non.epi.lncRNA

AC092801.1 non.epi.lncRNA

AC092802.1 non.epi.lncRNA

AC092802.2 non.epi.lncRNA

AC092802.3 non.epi.lncRNA

AC092809.4 non.epi.lncRNA

AC092810.3 non.epi.lncRNA

AC092813.1 non.epi.lncRNA

AC092813.2 non.epi.lncRNA

AC092818.1 non.epi.lncRNA

AC092819.1 non.epi.lncRNA

AC092828.1 non.epi.lncRNA

AC092832.2 non.epi.lncRNA

AC092834.1 non.epi.lncRNA

AC092839.1 non.epi.lncRNA

AC092839.2 non.epi.lncRNA

AC092848.1 non.epi.lncRNA

AC092849.2 non.epi.lncRNA

AC092894.1 non.epi.lncRNA

AC092902.2 non.epi.lncRNA

AC092910.3 non.epi.lncRNA

AC092916.1 non.epi.lncRNA

AC092924.1 non.epi.lncRNA

AC092925.1 non.epi.lncRNA

AC092941.1 non.epi.lncRNA

AC092941.2 non.epi.lncRNA

AC092944.1 non.epi.lncRNA

AC092954.1 non.epi.lncRNA

AC092954.2 non.epi.lncRNA

AC092957.1 non.epi.lncRNA

AC092958.1 non.epi.lncRNA

AC092958.2 non.epi.lncRNA

AC092966.1 non.epi.lncRNA

AC092969.1 non.epi.lncRNA

AC092979.1 non.epi.lncRNA

AC092994.1 non.epi.lncRNA

AC093010.2 non.epi.lncRNA

AC093010.3 non.epi.lncRNA

AC093019.2 non.epi.lncRNA

AC093025.1 non.epi.lncRNA

AC093072.1 non.epi.lncRNA

AC093074.1 non.epi.lncRNA

AC093083.1 non.epi.lncRNA

AC093110.1 non.epi.lncRNA

AC093117.1 non.epi.lncRNA

AC093151.2 non.epi.lncRNA

AC093151.3 non.epi.lncRNA

AC093152.1 non.epi.lncRNA

AC093157.1 non.epi.lncRNA

AC093157.2 non.epi.lncRNA

AC093158.1 non.epi.lncRNA

AC093159.1 non.epi.lncRNA

AC093206.1 non.epi.lncRNA

AC093227.1 non.epi.lncRNA

AC093248.1 non.epi.lncRNA

AC093274.1 non.epi.lncRNA

AC093278.2 non.epi.lncRNA

AC093281.1 non.epi.lncRNA

AC093281.2 non.epi.lncRNA

AC093283.1 non.epi.lncRNA

AC093292.1 non.epi.lncRNA

AC093297.1 non.epi.lncRNA

AC093297.2 non.epi.lncRNA

AC093305.1 non.epi.lncRNA

AC093326.1 non.epi.lncRNA

AC093330.1 non.epi.lncRNA

AC093382.1 non.epi.lncRNA

AC093388.1 non.epi.lncRNA

AC093390.1 non.epi.lncRNA

AC093422.2 non.epi.lncRNA

AC093423.2 non.epi.lncRNA

AC093424.1 non.epi.lncRNA

AC093425.1 non.epi.lncRNA

AC093426.1 non.epi.lncRNA

AC093458.1 non.epi.lncRNA

AC093459.1 non.epi.lncRNA

AC093462.1 non.epi.lncRNA

AC093484.1 non.epi.lncRNA

AC093484.2 non.epi.lncRNA

AC093484.3 non.epi.lncRNA

AC093495.1 non.epi.lncRNA

AC093503.1 non.epi.lncRNA

AC093503.2 non.epi.lncRNA

AC093510.1 non.epi.lncRNA

AC093510.2 non.epi.lncRNA

AC093512.1 non.epi.lncRNA

AC093515.1 non.epi.lncRNA

AC093523.1 non.epi.lncRNA

AC093525.3 non.epi.lncRNA

AC093525.4 non.epi.lncRNA

AC093525.5 non.epi.lncRNA

AC093525.6 non.epi.lncRNA

AC093525.7 non.epi.lncRNA

AC093534.2 non.epi.lncRNA

AC093535.1 non.epi.lncRNA

AC093536.2 non.epi.lncRNA

AC093579.1 non.epi.lncRNA

AC093581.1 non.epi.lncRNA

AC093582.1 non.epi.lncRNA

AC093583.1 non.epi.lncRNA

AC093585.1 non.epi.lncRNA

AC093591.2 non.epi.lncRNA

AC093599.1 non.epi.lncRNA

AC093607.1 non.epi.lncRNA

AC093620.1 non.epi.lncRNA

AC093627.1 non.epi.lncRNA

AC093627.2 non.epi.lncRNA

AC093627.3 non.epi.lncRNA

AC093627.5 non.epi.lncRNA

AC093627.6 non.epi.lncRNA

AC093627.7 non.epi.lncRNA

AC093635.1 non.epi.lncRNA

AC093639.1 non.epi.lncRNA

AC093642.1 non.epi.lncRNA

AC093655.1 non.epi.lncRNA

AC093673.2 non.epi.lncRNA

AC093677.2 non.epi.lncRNA

AC093689.1 non.epi.lncRNA

AC093690.1 non.epi.lncRNA

AC093702.1 non.epi.lncRNA

AC093714.1 non.epi.lncRNA

AC093714.2 non.epi.lncRNA

AC093722.1 non.epi.lncRNA

AC093725.1 non.epi.lncRNA

AC093725.2 non.epi.lncRNA

AC093730.1 non.epi.lncRNA

AC093732.2 non.epi.lncRNA

AC093752.2 non.epi.lncRNA

AC093752.3 non.epi.lncRNA

AC093763.1 non.epi.lncRNA

AC093766.1 non.epi.lncRNA

AC093772.1 non.epi.lncRNA

AC093788.1 non.epi.lncRNA

AC093791.1 non.epi.lncRNA

AC093797.1 non.epi.lncRNA

AC093799.1 non.epi.lncRNA

AC093801.1 non.epi.lncRNA

AC093802.1 non.epi.lncRNA

AC093802.2 non.epi.lncRNA

AC093810.1 non.epi.lncRNA

AC093817.1 non.epi.lncRNA

AC093831.1 non.epi.lncRNA

AC093833.1 non.epi.lncRNA

AC093835.1 non.epi.lncRNA

AC093843.1 non.epi.lncRNA

AC093849.1 non.epi.lncRNA

AC093857.1 non.epi.lncRNA

AC093864.1 non.epi.lncRNA

AC093865.1 non.epi.lncRNA

AC093866.1 non.epi.lncRNA

AC093871.1 non.epi.lncRNA

AC093879.1 non.epi.lncRNA

AC093879.2 non.epi.lncRNA

AC093893.1 non.epi.lncRNA

AC093895.1 non.epi.lncRNA

AC093903.1 non.epi.lncRNA

AC093904.1 non.epi.lncRNA

AC093904.2 non.epi.lncRNA

AC093904.4 non.epi.lncRNA

AC093909.2 non.epi.lncRNA

AC093909.4 non.epi.lncRNA

AC093911.1 non.epi.lncRNA

AC093916.1 non.epi.lncRNA

AC094104.1 non.epi.lncRNA

AC094104.2 non.epi.lncRNA

AC094105.1 non.epi.lncRNA

AC094105.2 non.epi.lncRNA

AC094108.1 non.epi.lncRNA

AC095032.1 non.epi.lncRNA

AC095032.2 non.epi.lncRNA

AC095033.1 non.epi.lncRNA

AC095050.1 non.epi.lncRNA

AC095055.1 non.epi.lncRNA

AC095056.1 non.epi.lncRNA

AC095057.2 non.epi.lncRNA

AC095059.1 non.epi.lncRNA

AC095060.1 non.epi.lncRNA

AC095350.1 non.epi.lncRNA

AC096531.2 non.epi.lncRNA

AC096536.1 non.epi.lncRNA

AC096536.2 non.epi.lncRNA

AC096537.1 non.epi.lncRNA

AC096541.1 non.epi.lncRNA

AC096543.1 non.epi.lncRNA

AC096543.2 non.epi.lncRNA

AC096554.1 non.epi.lncRNA

AC096559.1 non.epi.lncRNA

AC096564.1 non.epi.lncRNA

AC096564.2 non.epi.lncRNA

AC096570.1 non.epi.lncRNA

AC096570.2 non.epi.lncRNA

AC096576.2 non.epi.lncRNA

AC096576.3 non.epi.lncRNA

AC096577.1 non.epi.lncRNA

AC096586.1 non.epi.lncRNA

AC096588.1 non.epi.lncRNA

AC096631.1 non.epi.lncRNA

AC096639.1 non.epi.lncRNA

AC096642.1 non.epi.lncRNA

AC096644.3 non.epi.lncRNA

AC096649.1 non.epi.lncRNA

AC096659.1 non.epi.lncRNA

AC096666.1 non.epi.lncRNA

AC096669.1 non.epi.lncRNA

AC096677.1 non.epi.lncRNA

AC096677.2 non.epi.lncRNA

AC096708.2 non.epi.lncRNA

AC096708.3 non.epi.lncRNA

AC096711.1 non.epi.lncRNA

AC096711.2 non.epi.lncRNA

AC096719.1 non.epi.lncRNA

AC096721.1 non.epi.lncRNA

AC096732.1 non.epi.lncRNA

AC096733.1 non.epi.lncRNA

AC096733.2 non.epi.lncRNA

AC096734.1 non.epi.lncRNA

AC096734.2 non.epi.lncRNA

AC096736.1 non.epi.lncRNA

AC096736.2 non.epi.lncRNA

AC096736.3 non.epi.lncRNA

AC096741.1 non.epi.lncRNA

AC096759.1 non.epi.lncRNA

AC096759.2 non.epi.lncRNA

AC096773.1 non.epi.lncRNA

AC096920.1 non.epi.lncRNA

AC096992.2 non.epi.lncRNA

AC097065.2 non.epi.lncRNA

AC097103.1 non.epi.lncRNA

AC097110.1 non.epi.lncRNA

AC097173.2 non.epi.lncRNA

AC097359.3 non.epi.lncRNA

AC097372.1 non.epi.lncRNA

AC097375.2 non.epi.lncRNA

AC097375.3 non.epi.lncRNA

AC097376.3 non.epi.lncRNA

AC097381.2 non.epi.lncRNA

AC097381.3 non.epi.lncRNA

AC097382.2 non.epi.lncRNA

AC097451.1 non.epi.lncRNA

AC097460.1 non.epi.lncRNA

AC097467.1 non.epi.lncRNA

AC097467.3 non.epi.lncRNA

AC097468.1 non.epi.lncRNA

AC097468.2 non.epi.lncRNA

AC097468.3 non.epi.lncRNA

AC097478.1 non.epi.lncRNA

AC097478.2 non.epi.lncRNA

AC097480.1 non.epi.lncRNA

AC097487.1 non.epi.lncRNA

AC097491.1 non.epi.lncRNA

AC097494.1 non.epi.lncRNA

AC097501.1 non.epi.lncRNA

AC097504.2 non.epi.lncRNA

AC097505.1 non.epi.lncRNA

AC097512.1 non.epi.lncRNA

AC097515.1 non.epi.lncRNA

AC097518.1 non.epi.lncRNA

AC097521.1 non.epi.lncRNA

AC097532.1 non.epi.lncRNA

AC097535.1 non.epi.lncRNA

AC097537.1 non.epi.lncRNA

AC097625.1 non.epi.lncRNA

AC097634.1 non.epi.lncRNA

AC097634.2 non.epi.lncRNA

AC097634.3 non.epi.lncRNA

AC097641.1 non.epi.lncRNA

AC097641.2 non.epi.lncRNA

AC097652.1 non.epi.lncRNA

AC097658.2 non.epi.lncRNA

AC097662.1 non.epi.lncRNA

AC097713.1 non.epi.lncRNA

AC098479.1 non.epi.lncRNA

AC098483.1 non.epi.lncRNA

AC098484.1 non.epi.lncRNA

AC098484.2 non.epi.lncRNA

AC098487.1 non.epi.lncRNA

AC098587.1 non.epi.lncRNA

AC098617.1 non.epi.lncRNA

AC098649.1 non.epi.lncRNA

AC098657.2 non.epi.lncRNA

AC098679.1 non.epi.lncRNA

AC098680.1 non.epi.lncRNA

AC098798.1 non.epi.lncRNA

AC098818.2 non.epi.lncRNA

AC098820.1 non.epi.lncRNA

AC098820.2 non.epi.lncRNA

AC098820.3 non.epi.lncRNA

AC098828.2 non.epi.lncRNA

AC098829.1 non.epi.lncRNA

AC098848.1 non.epi.lncRNA

AC098850.1 non.epi.lncRNA

AC098850.2 non.epi.lncRNA

AC098850.4 non.epi.lncRNA

AC098859.2 non.epi.lncRNA

AC098868.1 non.epi.lncRNA

AC098869.2 non.epi.lncRNA

AC098872.1 non.epi.lncRNA

AC098934.4 non.epi.lncRNA

AC099063.1 non.epi.lncRNA

AC099066.1 non.epi.lncRNA

AC099066.2 non.epi.lncRNA

AC099313.1 non.epi.lncRNA

AC099314.1 non.epi.lncRNA

AC099328.2 non.epi.lncRNA

AC099329.1 non.epi.lncRNA

AC099329.2 non.epi.lncRNA

AC099343.2 non.epi.lncRNA

AC099343.3 non.epi.lncRNA

AC099344.1 non.epi.lncRNA

AC099344.2 non.epi.lncRNA

AC099398.1 non.epi.lncRNA

AC099487.1 non.epi.lncRNA

AC099489.3 non.epi.lncRNA

AC099499.1 non.epi.lncRNA

AC099506.1 non.epi.lncRNA

AC099508.1 non.epi.lncRNA

AC099509.1 non.epi.lncRNA

AC099509.2 non.epi.lncRNA

AC099511.1 non.epi.lncRNA

AC099518.2 non.epi.lncRNA

AC099518.3 non.epi.lncRNA

AC099518.4 non.epi.lncRNA

AC099518.5 non.epi.lncRNA

AC099520.1 non.epi.lncRNA

AC099520.2 non.epi.lncRNA

AC099521.1 non.epi.lncRNA

AC099524.1 non.epi.lncRNA

AC099541.1 non.epi.lncRNA

AC099542.1 non.epi.lncRNA

AC099542.2 non.epi.lncRNA

AC099550.1 non.epi.lncRNA

AC099552.1 non.epi.lncRNA

AC099552.2 non.epi.lncRNA

AC099552.3 non.epi.lncRNA

AC099554.1 non.epi.lncRNA

AC099560.1 non.epi.lncRNA

AC099567.1 non.epi.lncRNA

AC099568.1 non.epi.lncRNA

AC099654.1 non.epi.lncRNA

AC099668.1 non.epi.lncRNA

AC099677.4 non.epi.lncRNA

AC099681.1 non.epi.lncRNA

AC099681.2 non.epi.lncRNA

AC099681.3 non.epi.lncRNA

AC099684.1 non.epi.lncRNA

AC099684.2 non.epi.lncRNA

AC099684.3 non.epi.lncRNA

AC099754.1 non.epi.lncRNA

AC099778.1 non.epi.lncRNA

AC099786.1 non.epi.lncRNA

AC099786.2 non.epi.lncRNA

AC099786.3 non.epi.lncRNA

AC099788.1 non.epi.lncRNA

AC099791.2 non.epi.lncRNA

AC099792.1 non.epi.lncRNA

AC099794.1 non.epi.lncRNA

AC099811.5 non.epi.lncRNA

AC099850.1 non.epi.lncRNA

AC099850.3 non.epi.lncRNA

AC100763.1 non.epi.lncRNA

AC100768.1 non.epi.lncRNA

AC100774.1 non.epi.lncRNA

AC100775.1 non.epi.lncRNA

AC100778.1 non.epi.lncRNA

AC100778.3 non.epi.lncRNA

AC100781.1 non.epi.lncRNA

AC100782.1 non.epi.lncRNA

AC100786.2 non.epi.lncRNA

AC100788.1 non.epi.lncRNA

AC100791.1 non.epi.lncRNA

AC100791.2 non.epi.lncRNA

AC100791.3 non.epi.lncRNA

AC100793.3 non.epi.lncRNA

AC100793.4 non.epi.lncRNA

AC100797.1 non.epi.lncRNA

AC100800.1 non.epi.lncRNA

AC100801.1 non.epi.lncRNA

AC100802.1 non.epi.lncRNA

AC100803.2 non.epi.lncRNA

AC100803.3 non.epi.lncRNA

AC100803.4 non.epi.lncRNA

AC100807.2 non.epi.lncRNA

AC100810.1 non.epi.lncRNA

AC100814.1 non.epi.lncRNA

AC100823.1 non.epi.lncRNA

AC100826.1 non.epi.lncRNA

AC100827.3 non.epi.lncRNA

AC100827.4 non.epi.lncRNA

AC100830.2 non.epi.lncRNA

AC100832.2 non.epi.lncRNA

AC100835.1 non.epi.lncRNA

AC100836.1 non.epi.lncRNA

AC100839.1 non.epi.lncRNA

AC100839.2 non.epi.lncRNA

AC100843.1 non.epi.lncRNA

AC100844.1 non.epi.lncRNA

AC100847.1 non.epi.lncRNA

AC100849.1 non.epi.lncRNA

AC100849.2 non.epi.lncRNA

AC100858.2 non.epi.lncRNA

AC100858.3 non.epi.lncRNA

AC100860.1 non.epi.lncRNA

AC100863.1 non.epi.lncRNA

AC102797.1 non.epi.lncRNA

AC102941.1 non.epi.lncRNA

AC102945.1 non.epi.lncRNA

AC103409.1 non.epi.lncRNA

AC103563.2 non.epi.lncRNA

AC103564.1 non.epi.lncRNA

AC103591.3 non.epi.lncRNA

AC103681.2 non.epi.lncRNA

AC103691.1 non.epi.lncRNA

AC103691.2 non.epi.lncRNA

AC103702.1 non.epi.lncRNA

AC103702.2 non.epi.lncRNA

AC103719.1 non.epi.lncRNA

AC103724.3 non.epi.lncRNA

AC103726.1 non.epi.lncRNA

AC103726.2 non.epi.lncRNA

AC103739.1 non.epi.lncRNA

AC103740.1 non.epi.lncRNA

AC103764.1 non.epi.lncRNA

AC103769.1 non.epi.lncRNA

AC103770.1 non.epi.lncRNA

AC103794.1 non.epi.lncRNA

AC103796.1 non.epi.lncRNA

AC103808.1 non.epi.lncRNA

AC103808.2 non.epi.lncRNA

AC103808.3 non.epi.lncRNA

AC103808.4 non.epi.lncRNA

AC103808.6 non.epi.lncRNA

AC103809.1 non.epi.lncRNA

AC103810.2 non.epi.lncRNA

AC103810.5 non.epi.lncRNA

AC103831.1 non.epi.lncRNA

AC103843.1 non.epi.lncRNA

AC103855.1 non.epi.lncRNA

AC103855.2 non.epi.lncRNA

AC103855.3 non.epi.lncRNA

AC103858.1 non.epi.lncRNA

AC103858.2 non.epi.lncRNA

AC103876.1 non.epi.lncRNA

AC103879.1 non.epi.lncRNA

AC103923.1 non.epi.lncRNA

AC103925.1 non.epi.lncRNA

AC103952.1 non.epi.lncRNA

AC103957.1 non.epi.lncRNA

AC103957.2 non.epi.lncRNA

AC103974.1 non.epi.lncRNA

AC103982.1 non.epi.lncRNA

AC103987.2 non.epi.lncRNA

AC103993.1 non.epi.lncRNA

AC103996.2 non.epi.lncRNA

AC103996.3 non.epi.lncRNA

AC104002.1 non.epi.lncRNA

AC104002.2 non.epi.lncRNA

AC104002.3 non.epi.lncRNA

AC104009.1 non.epi.lncRNA

AC104012.1 non.epi.lncRNA

AC104024.2 non.epi.lncRNA

AC104031.1 non.epi.lncRNA

AC104035.1 non.epi.lncRNA

AC104041.1 non.epi.lncRNA

AC104051.1 non.epi.lncRNA

AC104051.2 non.epi.lncRNA

AC104063.1 non.epi.lncRNA

AC104066.3 non.epi.lncRNA

AC104071.1 non.epi.lncRNA

AC104072.1 non.epi.lncRNA

AC104078.1 non.epi.lncRNA

AC104078.2 non.epi.lncRNA

AC104083.1 non.epi.lncRNA

AC104088.1 non.epi.lncRNA

AC104109.4 non.epi.lncRNA

AC104113.1 non.epi.lncRNA

AC104116.1 non.epi.lncRNA

AC104117.3 non.epi.lncRNA

AC104118.1 non.epi.lncRNA

AC104119.1 non.epi.lncRNA

AC104123.1 non.epi.lncRNA

AC104126.1 non.epi.lncRNA

AC104129.1 non.epi.lncRNA

AC104134.1 non.epi.lncRNA

AC104135.1 non.epi.lncRNA

AC104137.1 non.epi.lncRNA

AC104170.2 non.epi.lncRNA

AC104211.2 non.epi.lncRNA

AC104211.3 non.epi.lncRNA

AC104232.1 non.epi.lncRNA

AC104232.2 non.epi.lncRNA

AC104237.1 non.epi.lncRNA

AC104237.2 non.epi.lncRNA

AC104237.3 non.epi.lncRNA

AC104248.1 non.epi.lncRNA

AC104257.1 non.epi.lncRNA

AC104260.1 non.epi.lncRNA

AC104316.1 non.epi.lncRNA

AC104350.1 non.epi.lncRNA

AC104365.1 non.epi.lncRNA

AC104365.2 non.epi.lncRNA

AC104365.3 non.epi.lncRNA

AC104389.2 non.epi.lncRNA

AC104393.1 non.epi.lncRNA

AC104407.1 non.epi.lncRNA

AC104411.1 non.epi.lncRNA

AC104417.2 non.epi.lncRNA

AC104423.1 non.epi.lncRNA

AC104435.2 non.epi.lncRNA

AC104445.1 non.epi.lncRNA

AC104457.2 non.epi.lncRNA

AC104458.1 non.epi.lncRNA

AC104461.1 non.epi.lncRNA

AC104462.1 non.epi.lncRNA

AC104462.2 non.epi.lncRNA

AC104463.1 non.epi.lncRNA

AC104463.2 non.epi.lncRNA

AC104472.1 non.epi.lncRNA

AC104506.1 non.epi.lncRNA

AC104521.1 non.epi.lncRNA

AC104534.1 non.epi.lncRNA

AC104561.1 non.epi.lncRNA

AC104561.3 non.epi.lncRNA

AC104564.2 non.epi.lncRNA

AC104564.4 non.epi.lncRNA

AC104564.5 non.epi.lncRNA

AC104574.1 non.epi.lncRNA

AC104574.2 non.epi.lncRNA

AC104596.1 non.epi.lncRNA

AC104619.2 non.epi.lncRNA

AC104623.1 non.epi.lncRNA

AC104640.1 non.epi.lncRNA

AC104653.2 non.epi.lncRNA

AC104655.1 non.epi.lncRNA

AC104662.1 non.epi.lncRNA

AC104663.1 non.epi.lncRNA

AC104664.1 non.epi.lncRNA

AC104688.1 non.epi.lncRNA

AC104695.1 non.epi.lncRNA

AC104695.2 non.epi.lncRNA

AC104695.3 non.epi.lncRNA

AC104695.4 non.epi.lncRNA

AC104699.1 non.epi.lncRNA

AC104758.4 non.epi.lncRNA

AC104758.5 non.epi.lncRNA

AC104770.1 non.epi.lncRNA

AC104777.1 non.epi.lncRNA

AC104777.2 non.epi.lncRNA

AC104781.1 non.epi.lncRNA

AC104785.1 non.epi.lncRNA

AC104791.1 non.epi.lncRNA

AC104793.1 non.epi.lncRNA

AC104794.2 non.epi.lncRNA

AC104794.3 non.epi.lncRNA

AC104794.4 non.epi.lncRNA

AC104794.5 non.epi.lncRNA

AC104803.1 non.epi.lncRNA

AC104806.2 non.epi.lncRNA

AC104809.2 non.epi.lncRNA

AC104819.3 non.epi.lncRNA

AC104823.1 non.epi.lncRNA

AC104825.1 non.epi.lncRNA

AC104827.1 non.epi.lncRNA

AC104831.1 non.epi.lncRNA

AC104836.1 non.epi.lncRNA

AC104958.1 non.epi.lncRNA

AC104958.2 non.epi.lncRNA

AC104961.1 non.epi.lncRNA

AC104964.1 non.epi.lncRNA

AC104964.2 non.epi.lncRNA

AC104964.3 non.epi.lncRNA

AC104964.4 non.epi.lncRNA

AC104971.2 non.epi.lncRNA

AC104971.3 non.epi.lncRNA

AC104982.2 non.epi.lncRNA

AC104984.1 non.epi.lncRNA

AC104984.3 non.epi.lncRNA

AC104984.4 non.epi.lncRNA

AC104984.5 non.epi.lncRNA

AC104985.1 non.epi.lncRNA

AC104985.2 non.epi.lncRNA

AC104996.1 non.epi.lncRNA

AC105001.1 non.epi.lncRNA

AC105020.1 non.epi.lncRNA

AC105020.2 non.epi.lncRNA

AC105020.3 non.epi.lncRNA

AC105020.4 non.epi.lncRNA

AC105020.5 non.epi.lncRNA

AC105020.6 non.epi.lncRNA

AC105031.2 non.epi.lncRNA

AC105036.3 non.epi.lncRNA

AC105046.1 non.epi.lncRNA

AC105052.4 non.epi.lncRNA

AC105094.1 non.epi.lncRNA

AC105094.2 non.epi.lncRNA

AC105105.1 non.epi.lncRNA

AC105105.2 non.epi.lncRNA

AC105105.3 non.epi.lncRNA

AC105105.4 non.epi.lncRNA

AC105118.1 non.epi.lncRNA

AC105133.1 non.epi.lncRNA

AC105137.2 non.epi.lncRNA

AC105150.1 non.epi.lncRNA

AC105177.1 non.epi.lncRNA

AC105180.1 non.epi.lncRNA

AC105206.1 non.epi.lncRNA

AC105206.2 non.epi.lncRNA

AC105219.1 non.epi.lncRNA

AC105219.2 non.epi.lncRNA

AC105219.3 non.epi.lncRNA

AC105219.4 non.epi.lncRNA

AC105227.1 non.epi.lncRNA

AC105265.3 non.epi.lncRNA

AC105265.4 non.epi.lncRNA

AC105271.1 non.epi.lncRNA

AC105275.1 non.epi.lncRNA

AC105275.2 non.epi.lncRNA

AC105285.1 non.epi.lncRNA

AC105290.1 non.epi.lncRNA

AC105343.1 non.epi.lncRNA

AC105345.1 non.epi.lncRNA

AC105345.2 non.epi.lncRNA

AC105362.1 non.epi.lncRNA

AC105383.1 non.epi.lncRNA

AC105384.1 non.epi.lncRNA

AC105389.1 non.epi.lncRNA

AC105389.3 non.epi.lncRNA

AC105390.1 non.epi.lncRNA

AC105393.1 non.epi.lncRNA

AC105393.2 non.epi.lncRNA

AC105398.1 non.epi.lncRNA

AC105402.3 non.epi.lncRNA

AC105411.1 non.epi.lncRNA

AC105415.1 non.epi.lncRNA

AC105430.1 non.epi.lncRNA

AC105446.1 non.epi.lncRNA

AC105450.1 non.epi.lncRNA

AC105460.1 non.epi.lncRNA

AC105460.2 non.epi.lncRNA

AC105914.2 non.epi.lncRNA

AC105924.1 non.epi.lncRNA

AC105935.1 non.epi.lncRNA

AC105940.1 non.epi.lncRNA

AC105940.2 non.epi.lncRNA

AC105942.1 non.epi.lncRNA

AC105999.1 non.epi.lncRNA

AC106017.1 non.epi.lncRNA

AC106017.2 non.epi.lncRNA

AC106028.3 non.epi.lncRNA

AC106028.4 non.epi.lncRNA

AC106037.2 non.epi.lncRNA

AC106037.3 non.epi.lncRNA

AC106052.1 non.epi.lncRNA

AC106053.1 non.epi.lncRNA

AC106706.1 non.epi.lncRNA

AC106707.1 non.epi.lncRNA

AC106712.1 non.epi.lncRNA

AC106729.1 non.epi.lncRNA

AC106730.1 non.epi.lncRNA

AC106736.1 non.epi.lncRNA

AC106738.1 non.epi.lncRNA

AC106738.2 non.epi.lncRNA

AC106739.1 non.epi.lncRNA

AC106744.1 non.epi.lncRNA

AC106744.2 non.epi.lncRNA

AC106745.1 non.epi.lncRNA

AC106754.1 non.epi.lncRNA

AC106771.1 non.epi.lncRNA

AC106772.1 non.epi.lncRNA

AC106772.2 non.epi.lncRNA

AC106782.5 non.epi.lncRNA

AC106785.2 non.epi.lncRNA

AC106786.1 non.epi.lncRNA

AC106791.1 non.epi.lncRNA

AC106791.2 non.epi.lncRNA

AC106793.1 non.epi.lncRNA

AC106795.2 non.epi.lncRNA

AC106795.3 non.epi.lncRNA

AC106795.5 non.epi.lncRNA

AC106796.1 non.epi.lncRNA

AC106798.1 non.epi.lncRNA

AC106799.1 non.epi.lncRNA

AC106799.2 non.epi.lncRNA

AC106799.3 non.epi.lncRNA

AC106801.1 non.epi.lncRNA

AC106820.3 non.epi.lncRNA

AC106820.4 non.epi.lncRNA

AC106821.1 non.epi.lncRNA

AC106822.1 non.epi.lncRNA

AC106864.1 non.epi.lncRNA

AC106870.1 non.epi.lncRNA

AC106870.2 non.epi.lncRNA

AC106871.1 non.epi.lncRNA

AC106872.9 non.epi.lncRNA

AC106873.1 non.epi.lncRNA

AC106874.1 non.epi.lncRNA

AC106875.1 non.epi.lncRNA

AC106875.2 non.epi.lncRNA

AC106876.1 non.epi.lncRNA

AC106881.1 non.epi.lncRNA

AC106886.2 non.epi.lncRNA

AC106886.3 non.epi.lncRNA

AC106892.1 non.epi.lncRNA

AC106894.1 non.epi.lncRNA

AC106895.1 non.epi.lncRNA

AC106895.2 non.epi.lncRNA

AC106897.1 non.epi.lncRNA

AC106900.1 non.epi.lncRNA

AC107021.1 non.epi.lncRNA

AC107021.2 non.epi.lncRNA

AC107023.1 non.epi.lncRNA

AC107027.1 non.epi.lncRNA

AC107027.3 non.epi.lncRNA

AC107029.1 non.epi.lncRNA

AC107029.2 non.epi.lncRNA

AC107031.1 non.epi.lncRNA

AC107032.2 non.epi.lncRNA

AC107057.1 non.epi.lncRNA

AC107068.1 non.epi.lncRNA

AC107072.1 non.epi.lncRNA

AC107072.2 non.epi.lncRNA

AC107074.1 non.epi.lncRNA

AC107079.1 non.epi.lncRNA

AC107081.2 non.epi.lncRNA

AC107204.1 non.epi.lncRNA

AC107208.1 non.epi.lncRNA

AC107214.2 non.epi.lncRNA

AC107220.1 non.epi.lncRNA

AC107223.1 non.epi.lncRNA

AC107241.1 non.epi.lncRNA

AC107294.1 non.epi.lncRNA

AC107294.2 non.epi.lncRNA

AC107294.3 non.epi.lncRNA

AC107373.1 non.epi.lncRNA

AC107373.2 non.epi.lncRNA

AC107375.1 non.epi.lncRNA

AC107391.1 non.epi.lncRNA

AC107396.1 non.epi.lncRNA

AC107398.2 non.epi.lncRNA

AC107398.3 non.epi.lncRNA

AC107419.1 non.epi.lncRNA

AC107463.1 non.epi.lncRNA

AC107464.1 non.epi.lncRNA

AC107464.2 non.epi.lncRNA

AC107464.3 non.epi.lncRNA

AC107871.2 non.epi.lncRNA

AC107882.1 non.epi.lncRNA

AC107884.1 non.epi.lncRNA

AC107884.2 non.epi.lncRNA

AC107886.1 non.epi.lncRNA

AC107892.1 non.epi.lncRNA

AC107905.1 non.epi.lncRNA

AC107909.1 non.epi.lncRNA

AC107909.2 non.epi.lncRNA

AC107926.1 non.epi.lncRNA

AC107952.2 non.epi.lncRNA

AC107953.2 non.epi.lncRNA

AC107958.2 non.epi.lncRNA

AC107958.3 non.epi.lncRNA

AC107959.1 non.epi.lncRNA

AC107973.1 non.epi.lncRNA

AC107976.1 non.epi.lncRNA

AC107980.1 non.epi.lncRNA

AC107982.3 non.epi.lncRNA

AC107993.1 non.epi.lncRNA

AC108002.1 non.epi.lncRNA

AC108002.2 non.epi.lncRNA

AC108010.1 non.epi.lncRNA

AC108025.1 non.epi.lncRNA

AC108037.1 non.epi.lncRNA

AC108051.1 non.epi.lncRNA

AC108063.1 non.epi.lncRNA

AC108066.1 non.epi.lncRNA

AC108067.1 non.epi.lncRNA

AC108097.1 non.epi.lncRNA

AC108099.1 non.epi.lncRNA

AC108102.1 non.epi.lncRNA

AC108112.1 non.epi.lncRNA

AC108125.1 non.epi.lncRNA

AC108134.1 non.epi.lncRNA

AC108134.3 non.epi.lncRNA

AC108136.1 non.epi.lncRNA

AC108142.1 non.epi.lncRNA

AC108156.1 non.epi.lncRNA

AC108159.1 non.epi.lncRNA

AC108174.1 non.epi.lncRNA

AC108199.1 non.epi.lncRNA

AC108206.1 non.epi.lncRNA

AC108210.1 non.epi.lncRNA

AC108448.1 non.epi.lncRNA

AC108449.1 non.epi.lncRNA

AC108449.2 non.epi.lncRNA

AC108449.3 non.epi.lncRNA

AC108451.1 non.epi.lncRNA

AC108451.2 non.epi.lncRNA

AC108462.1 non.epi.lncRNA

AC108463.2 non.epi.lncRNA

AC108463.3 non.epi.lncRNA

AC108467.1 non.epi.lncRNA

AC108471.2 non.epi.lncRNA

AC108472.1 non.epi.lncRNA

AC108482.1 non.epi.lncRNA

AC108515.1 non.epi.lncRNA

AC108516.1 non.epi.lncRNA

AC108516.2 non.epi.lncRNA

AC108517.1 non.epi.lncRNA

AC108673.2 non.epi.lncRNA

AC108673.3 non.epi.lncRNA

AC108681.1 non.epi.lncRNA

AC108693.2 non.epi.lncRNA

AC108704.1 non.epi.lncRNA

AC108721.1 non.epi.lncRNA

AC108727.1 non.epi.lncRNA

AC108727.2 non.epi.lncRNA

AC108733.1 non.epi.lncRNA

AC108747.1 non.epi.lncRNA

AC108749.1 non.epi.lncRNA

AC108752.1 non.epi.lncRNA

AC108860.2 non.epi.lncRNA

AC108861.1 non.epi.lncRNA

AC108863.2 non.epi.lncRNA

AC108865.1 non.epi.lncRNA

AC108865.2 non.epi.lncRNA

AC108868.1 non.epi.lncRNA

AC108868.2 non.epi.lncRNA

AC108879.1 non.epi.lncRNA

AC108935.1 non.epi.lncRNA

AC109309.1 non.epi.lncRNA

AC109347.2 non.epi.lncRNA

AC109349.1 non.epi.lncRNA

AC109361.1 non.epi.lncRNA

AC109361.2 non.epi.lncRNA

AC109439.2 non.epi.lncRNA

AC109446.3 non.epi.lncRNA

AC109449.1 non.epi.lncRNA

AC109454.2 non.epi.lncRNA

AC109460.2 non.epi.lncRNA

AC109460.3 non.epi.lncRNA

AC109460.4 non.epi.lncRNA

AC109462.1 non.epi.lncRNA

AC109462.2 non.epi.lncRNA

AC109464.1 non.epi.lncRNA

AC109464.2 non.epi.lncRNA

AC109466.1 non.epi.lncRNA

AC109471.1 non.epi.lncRNA

AC109479.1 non.epi.lncRNA

AC109479.3 non.epi.lncRNA

AC109492.1 non.epi.lncRNA

AC109495.1 non.epi.lncRNA

AC109587.1 non.epi.lncRNA

AC109597.1 non.epi.lncRNA

AC109597.2 non.epi.lncRNA

AC109630.1 non.epi.lncRNA

AC109809.1 non.epi.lncRNA

AC109811.1 non.epi.lncRNA

AC109927.1 non.epi.lncRNA

AC109927.2 non.epi.lncRNA

AC109992.2 non.epi.lncRNA

AC110011.1 non.epi.lncRNA

AC110015.1 non.epi.lncRNA

AC110023.1 non.epi.lncRNA

AC110058.1 non.epi.lncRNA

AC110285.1 non.epi.lncRNA

AC110285.3 non.epi.lncRNA

AC110285.4 non.epi.lncRNA

AC110285.5 non.epi.lncRNA

AC110288.1 non.epi.lncRNA

AC110296.1 non.epi.lncRNA

AC110491.1 non.epi.lncRNA

AC110491.2 non.epi.lncRNA

AC110597.1 non.epi.lncRNA

AC110597.3 non.epi.lncRNA

AC110603.1 non.epi.lncRNA

AC110609.1 non.epi.lncRNA

AC110615.1 non.epi.lncRNA

AC110619.1 non.epi.lncRNA

AC110716.1 non.epi.lncRNA

AC110716.2 non.epi.lncRNA

AC110741.1 non.epi.lncRNA

AC110751.1 non.epi.lncRNA

AC110760.1 non.epi.lncRNA

AC110760.2 non.epi.lncRNA

AC110767.1 non.epi.lncRNA

AC110769.1 non.epi.lncRNA

AC110769.2 non.epi.lncRNA

AC110772.1 non.epi.lncRNA

AC110772.2 non.epi.lncRNA

AC110792.2 non.epi.lncRNA

AC110792.3 non.epi.lncRNA

AC110800.1 non.epi.lncRNA

AC110813.1 non.epi.lncRNA

AC110921.1 non.epi.lncRNA

AC110995.1 non.epi.lncRNA

AC111000.4 non.epi.lncRNA

AC111149.1 non.epi.lncRNA

AC111152.2 non.epi.lncRNA

AC111170.2 non.epi.lncRNA

AC111194.1 non.epi.lncRNA

AC111194.2 non.epi.lncRNA

AC111198.1 non.epi.lncRNA

AC112176.1 non.epi.lncRNA

AC112178.1 non.epi.lncRNA

AC112187.3 non.epi.lncRNA

AC112198.3 non.epi.lncRNA

AC112204.2 non.epi.lncRNA

AC112206.2 non.epi.lncRNA

AC112211.1 non.epi.lncRNA

AC112219.1 non.epi.lncRNA

AC112219.2 non.epi.lncRNA

AC112229.2 non.epi.lncRNA

AC112229.4 non.epi.lncRNA

AC112236.1 non.epi.lncRNA

AC112236.2 non.epi.lncRNA

AC112242.1 non.epi.lncRNA

AC112250.2 non.epi.lncRNA

AC112481.1 non.epi.lncRNA

AC112484.3 non.epi.lncRNA

AC112487.1 non.epi.lncRNA

AC112491.1 non.epi.lncRNA

AC112493.1 non.epi.lncRNA

AC112495.1 non.epi.lncRNA

AC112496.1 non.epi.lncRNA

AC112503.1 non.epi.lncRNA

AC112503.2 non.epi.lncRNA

AC112512.1 non.epi.lncRNA

AC112693.1 non.epi.lncRNA

AC112695.1 non.epi.lncRNA

AC112719.2 non.epi.lncRNA

AC112721.1 non.epi.lncRNA

AC112722.1 non.epi.lncRNA

AC112907.1 non.epi.lncRNA

AC113133.1 non.epi.lncRNA

AC113137.1 non.epi.lncRNA

AC113139.1 non.epi.lncRNA

AC113145.1 non.epi.lncRNA

AC113146.1 non.epi.lncRNA

AC113167.1 non.epi.lncRNA

AC113189.1 non.epi.lncRNA

AC113189.2 non.epi.lncRNA

AC113189.3 non.epi.lncRNA

AC113189.4 non.epi.lncRNA

AC113192.1 non.epi.lncRNA

AC113192.3 non.epi.lncRNA

AC113194.1 non.epi.lncRNA

AC113208.4 non.epi.lncRNA

AC113331.1 non.epi.lncRNA

AC113346.1 non.epi.lncRNA

AC113346.2 non.epi.lncRNA

AC113347.4 non.epi.lncRNA

AC113349.1 non.epi.lncRNA

AC113349.2 non.epi.lncRNA

AC113355.1 non.epi.lncRNA

AC113361.1 non.epi.lncRNA

AC113382.1 non.epi.lncRNA

AC113382.2 non.epi.lncRNA

AC113383.1 non.epi.lncRNA

AC113385.1 non.epi.lncRNA

AC113386.1 non.epi.lncRNA

AC113391.1 non.epi.lncRNA

AC113391.2 non.epi.lncRNA

AC113398.2 non.epi.lncRNA

AC113404.1 non.epi.lncRNA

AC113410.2 non.epi.lncRNA

AC113414.1 non.epi.lncRNA

AC113418.1 non.epi.lncRNA

AC113423.1 non.epi.lncRNA

AC113423.2 non.epi.lncRNA

AC113607.1 non.epi.lncRNA

AC113608.2 non.epi.lncRNA

AC113615.1 non.epi.lncRNA

AC114284.1 non.epi.lncRNA

AC114296.1 non.epi.lncRNA

AC114316.1 non.epi.lncRNA

AC114321.1 non.epi.lncRNA

AC114341.1 non.epi.lncRNA

AC114401.1 non.epi.lncRNA

AC114485.1 non.epi.lncRNA

AC114488.3 non.epi.lncRNA

AC114498.1 non.epi.lncRNA

AC114501.3 non.epi.lncRNA

AC114546.1 non.epi.lncRNA

AC114546.2 non.epi.lncRNA

AC114550.1 non.epi.lncRNA

AC114550.2 non.epi.lncRNA

AC114550.3 non.epi.lncRNA

AC114684.1 non.epi.lncRNA

AC114689.3 non.epi.lncRNA

AC114730.1 non.epi.lncRNA

AC114730.2 non.epi.lncRNA

AC114730.3 non.epi.lncRNA

AC114737.1 non.epi.lncRNA

AC114741.1 non.epi.lncRNA

AC114752.1 non.epi.lncRNA

AC114757.1 non.epi.lncRNA

AC114763.1 non.epi.lncRNA

AC114763.2 non.epi.lncRNA

AC114776.1 non.epi.lncRNA

AC114808.1 non.epi.lncRNA

AC114808.2 non.epi.lncRNA

AC114810.1 non.epi.lncRNA

AC114811.2 non.epi.lncRNA

AC114812.2 non.epi.lncRNA

AC114814.1 non.epi.lncRNA

AC114814.3 non.epi.lncRNA

AC114928.1 non.epi.lncRNA

AC114939.1 non.epi.lncRNA

AC114954.1 non.epi.lncRNA

AC114956.3 non.epi.lncRNA

AC114980.1 non.epi.lncRNA

AC115099.1 non.epi.lncRNA

AC115102.1 non.epi.lncRNA

AC115284.1 non.epi.lncRNA

AC115522.1 non.epi.lncRNA

AC115618.1 non.epi.lncRNA

AC115618.2 non.epi.lncRNA

AC115619.1 non.epi.lncRNA

AC115622.1 non.epi.lncRNA

AC115676.1 non.epi.lncRNA

AC115837.2 non.epi.lncRNA

AC115989.1 non.epi.lncRNA

AC116003.2 non.epi.lncRNA

AC116003.3 non.epi.lncRNA

AC116021.1 non.epi.lncRNA

AC116025.1 non.epi.lncRNA

AC116025.2 non.epi.lncRNA

AC116035.1 non.epi.lncRNA

AC116036.2 non.epi.lncRNA

AC116038.1 non.epi.lncRNA

AC116049.2 non.epi.lncRNA

AC116096.1 non.epi.lncRNA

AC116158.1 non.epi.lncRNA

AC116158.2 non.epi.lncRNA

AC116337.3 non.epi.lncRNA

AC116345.1 non.epi.lncRNA

AC116345.3 non.epi.lncRNA

AC116348.1 non.epi.lncRNA

AC116348.2 non.epi.lncRNA

AC116348.3 non.epi.lncRNA

AC116351.1 non.epi.lncRNA

AC116351.2 non.epi.lncRNA

AC116362.1 non.epi.lncRNA

AC116366.2 non.epi.lncRNA

AC116407.1 non.epi.lncRNA

AC116424.1 non.epi.lncRNA

AC116447.1 non.epi.lncRNA

AC116456.1 non.epi.lncRNA

AC116535.1 non.epi.lncRNA

AC116552.1 non.epi.lncRNA

AC116563.1 non.epi.lncRNA

AC116609.1 non.epi.lncRNA

AC116609.2 non.epi.lncRNA

AC116612.1 non.epi.lncRNA

AC116616.1 non.epi.lncRNA

AC116634.1 non.epi.lncRNA

AC116651.1 non.epi.lncRNA

AC116666.1 non.epi.lncRNA

AC116667.1 non.epi.lncRNA

AC116903.1 non.epi.lncRNA

AC116903.2 non.epi.lncRNA

AC116914.1 non.epi.lncRNA

AC116914.2 non.epi.lncRNA

AC117373.1 non.epi.lncRNA

AC117377.1 non.epi.lncRNA

AC117383.1 non.epi.lncRNA

AC117386.1 non.epi.lncRNA

AC117386.2 non.epi.lncRNA

AC117395.1 non.epi.lncRNA

AC117422.1 non.epi.lncRNA

AC117430.1 non.epi.lncRNA

AC117453.1 non.epi.lncRNA

AC117460.1 non.epi.lncRNA

AC117462.1 non.epi.lncRNA

AC117473.1 non.epi.lncRNA

AC117498.1 non.epi.lncRNA

AC117500.1 non.epi.lncRNA

AC117500.2 non.epi.lncRNA

AC117500.3 non.epi.lncRNA

AC117503.1 non.epi.lncRNA

AC117505.1 non.epi.lncRNA

AC117522.2 non.epi.lncRNA

AC117569.1 non.epi.lncRNA

AC117834.1 non.epi.lncRNA

AC117834.2 non.epi.lncRNA

AC117944.1 non.epi.lncRNA

AC117945.1 non.epi.lncRNA

AC118345.1 non.epi.lncRNA

AC118553.1 non.epi.lncRNA

AC118555.1 non.epi.lncRNA

AC118653.1 non.epi.lncRNA

AC118658.1 non.epi.lncRNA

AC118754.1 non.epi.lncRNA

AC118754.2 non.epi.lncRNA

AC118755.2 non.epi.lncRNA

AC118757.1 non.epi.lncRNA

AC118758.3 non.epi.lncRNA

AC119150.1 non.epi.lncRNA

AC119396.2 non.epi.lncRNA

AC119403.1 non.epi.lncRNA

AC119424.1 non.epi.lncRNA

AC119428.1 non.epi.lncRNA

AC119674.1 non.epi.lncRNA

AC119800.1 non.epi.lncRNA

AC119868.2 non.epi.lncRNA

AC120024.1 non.epi.lncRNA

AC120036.4 non.epi.lncRNA

AC120042.1 non.epi.lncRNA

AC120042.2 non.epi.lncRNA

AC120045.1 non.epi.lncRNA

AC120049.1 non.epi.lncRNA

AC120114.1 non.epi.lncRNA

AC120114.2 non.epi.lncRNA

AC120193.1 non.epi.lncRNA

AC120349.1 non.epi.lncRNA

AC120498.10 non.epi.lncRNA

AC120498.2 non.epi.lncRNA

AC120498.3 non.epi.lncRNA

AC120498.4 non.epi.lncRNA

AC120498.6 non.epi.lncRNA

AC120498.8 non.epi.lncRNA

AC121154.1 non.epi.lncRNA

AC121161.2 non.epi.lncRNA

AC121247.1 non.epi.lncRNA

AC121320.1 non.epi.lncRNA

AC121333.1 non.epi.lncRNA

AC121342.1 non.epi.lncRNA

AC121493.1 non.epi.lncRNA

AC121764.1 non.epi.lncRNA

AC121764.2 non.epi.lncRNA

AC121764.3 non.epi.lncRNA

AC122108.1 non.epi.lncRNA

AC122108.2 non.epi.lncRNA

AC122136.1 non.epi.lncRNA

AC122138.1 non.epi.lncRNA

AC122683.1 non.epi.lncRNA

AC122685.1 non.epi.lncRNA

AC122688.2 non.epi.lncRNA

AC122694.1 non.epi.lncRNA

AC122707.1 non.epi.lncRNA

AC122710.1 non.epi.lncRNA

AC122710.2 non.epi.lncRNA

AC122719.1 non.epi.lncRNA

AC122719.2 non.epi.lncRNA

AC123023.1 non.epi.lncRNA

AC123768.1 non.epi.lncRNA

AC123768.4 non.epi.lncRNA

AC123777.1 non.epi.lncRNA

AC123786.1 non.epi.lncRNA

AC123905.1 non.epi.lncRNA

AC123912.1 non.epi.lncRNA

AC123912.2 non.epi.lncRNA

AC123912.4 non.epi.lncRNA

AC124016.2 non.epi.lncRNA

AC124017.1 non.epi.lncRNA

AC124045.1 non.epi.lncRNA

AC124057.1 non.epi.lncRNA

AC124067.1 non.epi.lncRNA

AC124067.2 non.epi.lncRNA

AC124067.3 non.epi.lncRNA

AC124067.4 non.epi.lncRNA

AC124069.1 non.epi.lncRNA

AC124242.1 non.epi.lncRNA

AC124254.2 non.epi.lncRNA

AC124283.2 non.epi.lncRNA

AC124283.3 non.epi.lncRNA

AC124290.1 non.epi.lncRNA

AC124290.2 non.epi.lncRNA

AC124301.1 non.epi.lncRNA

AC124312.2 non.epi.lncRNA

AC124312.3 non.epi.lncRNA

AC124319.1 non.epi.lncRNA

AC124784.1 non.epi.lncRNA

AC124798.1 non.epi.lncRNA

AC124804.1 non.epi.lncRNA

AC124852.1 non.epi.lncRNA

AC124854.1 non.epi.lncRNA

AC124861.1 non.epi.lncRNA

AC124893.1 non.epi.lncRNA

AC124947.1 non.epi.lncRNA

AC125257.1 non.epi.lncRNA

AC125257.2 non.epi.lncRNA

AC125421.1 non.epi.lncRNA

AC125421.2 non.epi.lncRNA

AC125437.1 non.epi.lncRNA

AC125494.1 non.epi.lncRNA

AC125494.2 non.epi.lncRNA

AC125603.1 non.epi.lncRNA

AC125603.2 non.epi.lncRNA

AC125603.4 non.epi.lncRNA

AC125611.4 non.epi.lncRNA

AC125613.1 non.epi.lncRNA

AC125616.1 non.epi.lncRNA

AC125618.1 non.epi.lncRNA

AC125793.1 non.epi.lncRNA

AC125807.2 non.epi.lncRNA

AC126118.1 non.epi.lncRNA

AC126121.2 non.epi.lncRNA

AC126121.3 non.epi.lncRNA

AC126177.3 non.epi.lncRNA

AC126178.1 non.epi.lncRNA

AC126283.1 non.epi.lncRNA

AC126323.2 non.epi.lncRNA

AC126323.3 non.epi.lncRNA

AC126323.5 non.epi.lncRNA

AC126335.1 non.epi.lncRNA

AC126365.1 non.epi.lncRNA

AC126614.1 non.epi.lncRNA

AC126696.1 non.epi.lncRNA

AC126755.2 non.epi.lncRNA

AC126755.3 non.epi.lncRNA

AC126768.1 non.epi.lncRNA

AC126768.2 non.epi.lncRNA

AC126773.3 non.epi.lncRNA

AC126773.4 non.epi.lncRNA

AC126773.6 non.epi.lncRNA

AC127002.2 non.epi.lncRNA

AC127024.3 non.epi.lncRNA

AC127024.4 non.epi.lncRNA

AC127024.5 non.epi.lncRNA

AC127029.2 non.epi.lncRNA

AC127070.1 non.epi.lncRNA

AC127164.1 non.epi.lncRNA

AC127496.1 non.epi.lncRNA

AC127496.2 non.epi.lncRNA

AC127496.3 non.epi.lncRNA

AC127496.4 non.epi.lncRNA

AC127496.5 non.epi.lncRNA

AC127496.6 non.epi.lncRNA

AC127502.2 non.epi.lncRNA

AC127521.1 non.epi.lncRNA

AC127522.1 non.epi.lncRNA

AC127526.1 non.epi.lncRNA

AC127526.2 non.epi.lncRNA

AC127537.1 non.epi.lncRNA

AC127894.1 non.epi.lncRNA

AC128685.1 non.epi.lncRNA

AC128687.2 non.epi.lncRNA

AC128707.1 non.epi.lncRNA

AC129102.1 non.epi.lncRNA

AC129492.1 non.epi.lncRNA

AC129492.2 non.epi.lncRNA

AC129507.1 non.epi.lncRNA

AC129507.2 non.epi.lncRNA

AC129507.3 non.epi.lncRNA

AC129507.4 non.epi.lncRNA

AC129510.2 non.epi.lncRNA

AC129807.1 non.epi.lncRNA

AC129926.1 non.epi.lncRNA

AC130324.1 non.epi.lncRNA

AC130324.2 non.epi.lncRNA

AC130324.3 non.epi.lncRNA

AC130343.1 non.epi.lncRNA

AC130343.2 non.epi.lncRNA

AC130352.1 non.epi.lncRNA

AC130371.1 non.epi.lncRNA

AC130371.2 non.epi.lncRNA

AC130404.1 non.epi.lncRNA

AC130415.1 non.epi.lncRNA

AC130456.1 non.epi.lncRNA

AC130456.2 non.epi.lncRNA

AC130456.3 non.epi.lncRNA

AC130456.5 non.epi.lncRNA

AC130462.2 non.epi.lncRNA

AC130469.1 non.epi.lncRNA

AC130651.1 non.epi.lncRNA

AC130710.1 non.epi.lncRNA

AC130895.1 non.epi.lncRNA

AC131009.3 non.epi.lncRNA

AC131011.1 non.epi.lncRNA

AC131025.1 non.epi.lncRNA

AC131025.2 non.epi.lncRNA

AC131094.1 non.epi.lncRNA

AC131097.1 non.epi.lncRNA

AC131157.1 non.epi.lncRNA

AC131159.1 non.epi.lncRNA

AC131159.2 non.epi.lncRNA

AC131182.1 non.epi.lncRNA

AC131210.1 non.epi.lncRNA

AC131211.1 non.epi.lncRNA

AC131212.1 non.epi.lncRNA

AC131235.2 non.epi.lncRNA

AC131235.3 non.epi.lncRNA

AC131238.1 non.epi.lncRNA

AC131254.1 non.epi.lncRNA

AC131254.2 non.epi.lncRNA

AC131254.3 non.epi.lncRNA

AC131274.1 non.epi.lncRNA

AC131391.1 non.epi.lncRNA

AC131571.1 non.epi.lncRNA

AC131649.1 non.epi.lncRNA

AC131902.1 non.epi.lncRNA

AC131934.1 non.epi.lncRNA

AC131953.1 non.epi.lncRNA

AC131956.1 non.epi.lncRNA

AC131956.2 non.epi.lncRNA

AC131971.1 non.epi.lncRNA

AC132192.2 non.epi.lncRNA

AC132216.1 non.epi.lncRNA

AC132217.1 non.epi.lncRNA

AC132219.1 non.epi.lncRNA

AC132803.1 non.epi.lncRNA

AC132807.1 non.epi.lncRNA

AC132807.2 non.epi.lncRNA

AC132825.2 non.epi.lncRNA

AC132825.3 non.epi.lncRNA

AC132872.1 non.epi.lncRNA

AC132872.3 non.epi.lncRNA

AC132938.2 non.epi.lncRNA

AC132938.3 non.epi.lncRNA

AC133065.1 non.epi.lncRNA

AC133480.1 non.epi.lncRNA

AC133485.2 non.epi.lncRNA

AC133485.3 non.epi.lncRNA

AC133485.5 non.epi.lncRNA

AC133528.1 non.epi.lncRNA

AC133540.1 non.epi.lncRNA

AC133550.2 non.epi.lncRNA

AC133550.3 non.epi.lncRNA

AC133552.5 non.epi.lncRNA

AC133565.1 non.epi.lncRNA

AC133634.1 non.epi.lncRNA

AC133644.2 non.epi.lncRNA

AC133785.1 non.epi.lncRNA

AC133865.1 non.epi.lncRNA

AC133919.1 non.epi.lncRNA

AC133919.2 non.epi.lncRNA

AC133961.1 non.epi.lncRNA

AC134043.2 non.epi.lncRNA

AC134312.1 non.epi.lncRNA

AC134312.2 non.epi.lncRNA

AC134312.3 non.epi.lncRNA

AC134312.4 non.epi.lncRNA

AC134312.5 non.epi.lncRNA

AC134312.6 non.epi.lncRNA

AC134349.1 non.epi.lncRNA

AC134407.1 non.epi.lncRNA

AC134508.1 non.epi.lncRNA

AC134772.1 non.epi.lncRNA

AC134775.1 non.epi.lncRNA

AC134978.1 non.epi.lncRNA

AC135012.1 non.epi.lncRNA

AC135012.2 non.epi.lncRNA

AC135012.3 non.epi.lncRNA

AC135048.2 non.epi.lncRNA

AC135050.1 non.epi.lncRNA

AC135050.3 non.epi.lncRNA

AC135050.5 non.epi.lncRNA

AC135050.6 non.epi.lncRNA

AC135068.7 non.epi.lncRNA

AC135178.2 non.epi.lncRNA

AC135178.4 non.epi.lncRNA

AC135178.5 non.epi.lncRNA

AC135178.6 non.epi.lncRNA

AC135279.4 non.epi.lncRNA

AC135388.1 non.epi.lncRNA

AC135507.1 non.epi.lncRNA

AC135586.2 non.epi.lncRNA

AC135776.1 non.epi.lncRNA

AC135776.2 non.epi.lncRNA

AC135782.1 non.epi.lncRNA

AC135782.2 non.epi.lncRNA

AC135782.3 non.epi.lncRNA

AC135803.1 non.epi.lncRNA

AC135895.1 non.epi.lncRNA

AC135983.1 non.epi.lncRNA

AC136188.1 non.epi.lncRNA

AC136285.1 non.epi.lncRNA

AC136285.2 non.epi.lncRNA

AC136424.1 non.epi.lncRNA

AC136424.2 non.epi.lncRNA

AC136431.1 non.epi.lncRNA

AC136443.3 non.epi.lncRNA

AC136443.4 non.epi.lncRNA

AC136475.2 non.epi.lncRNA

AC136475.4 non.epi.lncRNA

AC136475.9 non.epi.lncRNA

AC136489.1 non.epi.lncRNA

AC136601.1 non.epi.lncRNA

AC136604.2 non.epi.lncRNA

AC136618.1 non.epi.lncRNA

AC136624.1 non.epi.lncRNA

AC136624.2 non.epi.lncRNA

AC136628.1 non.epi.lncRNA

AC136628.4 non.epi.lncRNA

AC136698.1 non.epi.lncRNA

AC136777.1 non.epi.lncRNA

AC136944.1 non.epi.lncRNA

AC136944.2 non.epi.lncRNA

AC136944.4 non.epi.lncRNA

AC137056.1 non.epi.lncRNA

AC137579.1 non.epi.lncRNA

AC137579.2 non.epi.lncRNA

AC137590.1 non.epi.lncRNA

AC137590.2 non.epi.lncRNA

AC137630.1 non.epi.lncRNA

AC137630.2 non.epi.lncRNA

AC137630.3 non.epi.lncRNA

AC137630.4 non.epi.lncRNA

AC137735.1 non.epi.lncRNA

AC137761.1 non.epi.lncRNA

AC137770.1 non.epi.lncRNA

AC137810.1 non.epi.lncRNA

AC137894.1 non.epi.lncRNA

AC137932.3 non.epi.lncRNA

AC138024.1 non.epi.lncRNA

AC138028.2 non.epi.lncRNA

AC138028.3 non.epi.lncRNA

AC138028.5 non.epi.lncRNA

AC138028.6 non.epi.lncRNA

AC138035.1 non.epi.lncRNA

AC138035.2 non.epi.lncRNA

AC138057.1 non.epi.lncRNA

AC138089.1 non.epi.lncRNA

AC138123.1 non.epi.lncRNA

AC138123.2 non.epi.lncRNA

AC138150.1 non.epi.lncRNA

AC138150.2 non.epi.lncRNA

AC138207.1 non.epi.lncRNA

AC138207.4 non.epi.lncRNA

AC138207.5 non.epi.lncRNA

AC138207.7 non.epi.lncRNA

AC138230.1 non.epi.lncRNA

AC138304.1 non.epi.lncRNA

AC138305.1 non.epi.lncRNA

AC138305.2 non.epi.lncRNA

AC138331.1 non.epi.lncRNA

AC138356.3 non.epi.lncRNA

AC138360.1 non.epi.lncRNA

AC138393.3 non.epi.lncRNA

AC138430.1 non.epi.lncRNA

AC138466.3 non.epi.lncRNA

AC138474.1 non.epi.lncRNA

AC138512.1 non.epi.lncRNA

AC138625.1 non.epi.lncRNA

AC138625.2 non.epi.lncRNA

AC138627.1 non.epi.lncRNA

AC138646.1 non.epi.lncRNA

AC138649.1 non.epi.lncRNA

AC138655.1 non.epi.lncRNA

AC138696.2 non.epi.lncRNA

AC138761.1 non.epi.lncRNA

AC138781.1 non.epi.lncRNA

AC138811.1 non.epi.lncRNA

AC138819.1 non.epi.lncRNA

AC138869.2 non.epi.lncRNA

AC138869.3 non.epi.lncRNA

AC138894.2 non.epi.lncRNA

AC138904.1 non.epi.lncRNA

AC138907.1 non.epi.lncRNA

AC138907.2 non.epi.lncRNA

AC138907.3 non.epi.lncRNA

AC138907.7 non.epi.lncRNA

AC138915.3 non.epi.lncRNA

AC138932.2 non.epi.lncRNA

AC138932.5 non.epi.lncRNA

AC138956.1 non.epi.lncRNA

AC138965.1 non.epi.lncRNA

AC138969.3 non.epi.lncRNA

AC139099.1 non.epi.lncRNA

AC139100.2 non.epi.lncRNA

AC139149.1 non.epi.lncRNA

AC139256.2 non.epi.lncRNA

AC139426.2 non.epi.lncRNA

AC139491.1 non.epi.lncRNA

AC139491.3 non.epi.lncRNA

AC139491.4 non.epi.lncRNA

AC139497.1 non.epi.lncRNA

AC139712.3 non.epi.lncRNA

AC139718.1 non.epi.lncRNA

AC139718.2 non.epi.lncRNA

AC139720.1 non.epi.lncRNA

AC139720.2 non.epi.lncRNA

AC139768.1 non.epi.lncRNA

AC139769.2 non.epi.lncRNA

AC139792.1 non.epi.lncRNA

AC139795.2 non.epi.lncRNA

AC139795.3 non.epi.lncRNA

AC139887.1 non.epi.lncRNA

AC139887.2 non.epi.lncRNA

AC139887.3 non.epi.lncRNA

AC139887.4 non.epi.lncRNA

AC140118.1 non.epi.lncRNA

AC140125.1 non.epi.lncRNA

AC140125.2 non.epi.lncRNA

AC140125.3 non.epi.lncRNA

AC140481.1 non.epi.lncRNA

AC140481.2 non.epi.lncRNA

AC140658.1 non.epi.lncRNA

AC140658.2 non.epi.lncRNA

AC140847.1 non.epi.lncRNA

AC140847.2 non.epi.lncRNA

AC141002.1 non.epi.lncRNA

AC141257.1 non.epi.lncRNA

AC141257.2 non.epi.lncRNA

AC141273.1 non.epi.lncRNA

AC141586.2 non.epi.lncRNA

AC141586.3 non.epi.lncRNA

AC141930.1 non.epi.lncRNA

AC142086.1 non.epi.lncRNA

AC142086.4 non.epi.lncRNA

AC142472.1 non.epi.lncRNA

AC144521.1 non.epi.lncRNA

AC144548.1 non.epi.lncRNA

AC144568.2 non.epi.lncRNA

AC144831.1 non.epi.lncRNA

AC144833.1 non.epi.lncRNA

AC144836.1 non.epi.lncRNA

AC145124.1 non.epi.lncRNA

AC145141.1 non.epi.lncRNA

AC145141.2 non.epi.lncRNA

AC145146.1 non.epi.lncRNA

AC145207.1 non.epi.lncRNA

AC145207.2 non.epi.lncRNA

AC145207.3 non.epi.lncRNA

AC145207.5 non.epi.lncRNA

AC145207.7 non.epi.lncRNA

AC145285.2 non.epi.lncRNA

AC145285.6 non.epi.lncRNA

AC145343.1 non.epi.lncRNA

AC145350.2 non.epi.lncRNA

AC145350.4 non.epi.lncRNA

AC145423.2 non.epi.lncRNA

AC145423.3 non.epi.lncRNA

AC145543.1 non.epi.lncRNA

AC145625.1 non.epi.lncRNA

AC146944.4 non.epi.lncRNA

AC147067.2 non.epi.lncRNA

AC147651.2 non.epi.lncRNA

AC147651.3 non.epi.lncRNA

AC147651.4 non.epi.lncRNA

AC148476.1 non.epi.lncRNA

AC148477.1 non.epi.lncRNA

AC148477.2 non.epi.lncRNA

AC148477.3 non.epi.lncRNA

AC148477.4 non.epi.lncRNA

AC156455.1 non.epi.lncRNA

AC159540.1 non.epi.lncRNA

AC159540.2 non.epi.lncRNA

AC174065.1 non.epi.lncRNA

AC209005.1 non.epi.lncRNA

AC209154.1 non.epi.lncRNA

AC211433.1 non.epi.lncRNA

AC211486.2 non.epi.lncRNA

AC211486.5 non.epi.lncRNA

AC215217.1 non.epi.lncRNA

AC217774.1 non.epi.lncRNA

AC217774.2 non.epi.lncRNA

AC226101.1 non.epi.lncRNA

AC226119.1 non.epi.lncRNA

AC231533.1 non.epi.lncRNA

AC231981.1 non.epi.lncRNA

AC233263.6 non.epi.lncRNA

AC233266.2 non.epi.lncRNA

AC233296.1 non.epi.lncRNA

AC233701.1 non.epi.lncRNA

AC233702.5 non.epi.lncRNA

AC233728.1 non.epi.lncRNA

AC233976.1 non.epi.lncRNA

AC234582.1 non.epi.lncRNA

AC234582.2 non.epi.lncRNA

AC234771.1 non.epi.lncRNA

AC234775.3 non.epi.lncRNA

AC234781.1 non.epi.lncRNA

AC234917.1 non.epi.lncRNA

AC235097.1 non.epi.lncRNA

AC236972.3 non.epi.lncRNA

AC237221.1 non.epi.lncRNA

AC239584.1 non.epi.lncRNA

AC239585.1 non.epi.lncRNA

AC239585.2 non.epi.lncRNA

AC239727.1 non.epi.lncRNA

AC239799.1 non.epi.lncRNA

AC239800.2 non.epi.lncRNA

AC239802.1 non.epi.lncRNA

AC239802.2 non.epi.lncRNA

AC239803.1 non.epi.lncRNA

AC239804.1 non.epi.lncRNA

AC239809.3 non.epi.lncRNA

AC239868.1 non.epi.lncRNA

AC240565.2 non.epi.lncRNA

AC241644.1 non.epi.lncRNA

AC241644.3 non.epi.lncRNA

AC242426.2 non.epi.lncRNA

AC242842.1 non.epi.lncRNA

AC242988.1 non.epi.lncRNA

AC243547.1 non.epi.lncRNA

AC243562.3 non.epi.lncRNA

AC243571.1 non.epi.lncRNA

AC243571.2 non.epi.lncRNA

AC243585.1 non.epi.lncRNA

AC243585.2 non.epi.lncRNA

AC243654.3 non.epi.lncRNA

AC243772.2 non.epi.lncRNA

AC243773.1 non.epi.lncRNA

AC243829.1 non.epi.lncRNA

AC243829.2 non.epi.lncRNA

AC243829.4 non.epi.lncRNA

AC243829.5 non.epi.lncRNA

AC243830.1 non.epi.lncRNA

AC243830.2 non.epi.lncRNA

AC243830.3 non.epi.lncRNA

AC243919.2 non.epi.lncRNA

AC243961.1 non.epi.lncRNA

AC243964.3 non.epi.lncRNA

AC243965.1 non.epi.lncRNA

AC243965.2 non.epi.lncRNA

AC243967.2 non.epi.lncRNA

AC243967.3 non.epi.lncRNA

AC243972.1 non.epi.lncRNA

AC244035.2 non.epi.lncRNA

AC244090.3 non.epi.lncRNA

AC244093.3 non.epi.lncRNA

AC244093.4 non.epi.lncRNA

AC244093.5 non.epi.lncRNA

AC244100.2 non.epi.lncRNA

AC244100.4 non.epi.lncRNA

AC244102.1 non.epi.lncRNA

AC244131.2 non.epi.lncRNA

AC244197.2 non.epi.lncRNA

AC244205.1 non.epi.lncRNA

AC244230.1 non.epi.lncRNA

AC244250.1 non.epi.lncRNA

AC244394.2 non.epi.lncRNA

AC244453.2 non.epi.lncRNA

AC244453.3 non.epi.lncRNA

AC244502.1 non.epi.lncRNA

AC244502.3 non.epi.lncRNA

AC244517.1 non.epi.lncRNA

AC244517.11 non.epi.lncRNA

AC244517.12 non.epi.lncRNA

AC244517.2 non.epi.lncRNA

AC244517.4 non.epi.lncRNA

AC244517.5 non.epi.lncRNA

AC244517.6 non.epi.lncRNA

AC244517.7 non.epi.lncRNA

AC244517.8 non.epi.lncRNA

AC244517.9 non.epi.lncRNA

AC245008.1 non.epi.lncRNA

AC245014.1 non.epi.lncRNA

AC245014.3 non.epi.lncRNA

AC245041.1 non.epi.lncRNA

AC245052.1 non.epi.lncRNA

AC245052.4 non.epi.lncRNA

AC245052.7 non.epi.lncRNA

AC245056.1 non.epi.lncRNA

AC245060.2 non.epi.lncRNA

AC245060.5 non.epi.lncRNA

AC245060.6 non.epi.lncRNA

AC245088.2 non.epi.lncRNA

AC245100.1 non.epi.lncRNA

AC245100.6 non.epi.lncRNA

AC245123.1 non.epi.lncRNA

AC245128.1 non.epi.lncRNA

AC245128.3 non.epi.lncRNA

AC245140.1 non.epi.lncRNA

AC245140.2 non.epi.lncRNA

AC245164.1 non.epi.lncRNA

AC245177.1 non.epi.lncRNA

AC245187.1 non.epi.lncRNA

AC245297.2 non.epi.lncRNA

AC245297.3 non.epi.lncRNA

AC245427.1 non.epi.lncRNA

AC245452.1 non.epi.lncRNA

AC245452.5 non.epi.lncRNA

AC245519.1 non.epi.lncRNA

AC245748.2 non.epi.lncRNA

AC245748.3 non.epi.lncRNA

AC245884.1 non.epi.lncRNA

AC245884.10 non.epi.lncRNA

AC245884.11 non.epi.lncRNA

AC245884.8 non.epi.lncRNA

AC245884.9 non.epi.lncRNA

AC246680.1 non.epi.lncRNA

AC246785.3 non.epi.lncRNA

AC246787.2 non.epi.lncRNA

AC246817.1 non.epi.lncRNA

AC246817.2 non.epi.lncRNA

AC247036.1 non.epi.lncRNA

AC253536.3 non.epi.lncRNA

AC253536.6 non.epi.lncRNA

AC254562.2 non.epi.lncRNA

AC254562.3 non.epi.lncRNA

AC254629.1 non.epi.lncRNA

ACAP2-IT1 non.epi.lncRNA

ACOXL-AS1 non.epi.lncRNA

ACTN1-AS1 non.epi.lncRNA

ACVR2B-AS1 non.epi.lncRNA

AD001527.1 non.epi.lncRNA

ADAMTS19-AS1 non.epi.lncRNA

ADAMTS9-AS1 non.epi.lncRNA

ADAMTS9-AS2 non.epi.lncRNA

ADARB2-AS1 non.epi.lncRNA

ADCY6-DT non.epi.lncRNA

ADD3-AS1 non.epi.lncRNA

ADGRL3-AS1 non.epi.lncRNA

ADIPOQ-AS1 non.epi.lncRNA

ADNP-AS1 non.epi.lncRNA

ADORA2A-AS1 non.epi.lncRNA

ADPGK-AS1 non.epi.lncRNA

AF001548.1 non.epi.lncRNA

AF001548.2 non.epi.lncRNA

AF001550.1 non.epi.lncRNA

AF015720.1 non.epi.lncRNA

AF038458.2 non.epi.lncRNA

AF064858.1 non.epi.lncRNA

AF064858.2 non.epi.lncRNA

AF064860.1 non.epi.lncRNA

AF064860.2 non.epi.lncRNA

AF067845.1 non.epi.lncRNA

AF067845.2 non.epi.lncRNA

AF096876.1 non.epi.lncRNA

AF099810.1 non.epi.lncRNA

AF106564.1 non.epi.lncRNA

AF107885.2 non.epi.lncRNA

AF111167.1 non.epi.lncRNA

AF111169.1 non.epi.lncRNA

AF111169.2 non.epi.lncRNA

AF111169.3 non.epi.lncRNA

AF117829.1 non.epi.lncRNA

AF121898.1 non.epi.lncRNA

AF123462.1 non.epi.lncRNA

AF124730.1 non.epi.lncRNA

AF127577.1 non.epi.lncRNA

AF127577.2 non.epi.lncRNA

AF127577.3 non.epi.lncRNA

AF127577.4 non.epi.lncRNA

AF127577.5 non.epi.lncRNA

AF127936.1 non.epi.lncRNA

AF127936.2 non.epi.lncRNA

AF129075.1 non.epi.lncRNA

AF129075.2 non.epi.lncRNA

AF130359.1 non.epi.lncRNA

AF130417.1 non.epi.lncRNA

AF131215.2 non.epi.lncRNA

AF131215.3 non.epi.lncRNA

AF131215.4 non.epi.lncRNA

AF131215.5 non.epi.lncRNA

AF131215.6 non.epi.lncRNA

AF131215.7 non.epi.lncRNA

AF131216.1 non.epi.lncRNA

AF131216.3 non.epi.lncRNA

AF131216.4 non.epi.lncRNA

AF165147.1 non.epi.lncRNA

AF178030.1 non.epi.lncRNA

AF181450.1 non.epi.lncRNA

AF186192.1 non.epi.lncRNA

AF186192.3 non.epi.lncRNA

AF196972.1 non.epi.lncRNA

AF212831.1 non.epi.lncRNA

AF213884.3 non.epi.lncRNA

AF230666.1 non.epi.lncRNA

AF230666.2 non.epi.lncRNA

AF233439.1 non.epi.lncRNA

AF233439.2 non.epi.lncRNA

AF240627.1 non.epi.lncRNA

AF241725.1 non.epi.lncRNA

AF241728.1 non.epi.lncRNA

AF250324.1 non.epi.lncRNA

AF254983.1 non.epi.lncRNA

AF274573.1 non.epi.lncRNA

AF279873.3 non.epi.lncRNA

AF279873.4 non.epi.lncRNA

AF287957.1 non.epi.lncRNA

AFF2-IT1 non.epi.lncRNA

AGAP11 non.epi.lncRNA

AGBL1-AS1 non.epi.lncRNA

AGBL4-IT1 non.epi.lncRNA

AGBL5-IT1 non.epi.lncRNA

AIRN non.epi.lncRNA

AJ003147.1 non.epi.lncRNA

AJ003147.2 non.epi.lncRNA

AJ006995.1 non.epi.lncRNA

AJ009632.1 non.epi.lncRNA

AJ009632.2 non.epi.lncRNA

AJ011931.1 non.epi.lncRNA

AJ011932.1 non.epi.lncRNA

AJ239318.1 non.epi.lncRNA

AJ239322.1 non.epi.lncRNA

AJ239322.2 non.epi.lncRNA

AJ239328.1 non.epi.lncRNA

AJ271736.1 non.epi.lncRNA

AKT3-IT1 non.epi.lncRNA

AL008582.1 non.epi.lncRNA

AL008626.1 non.epi.lncRNA

AL008628.1 non.epi.lncRNA

AL008633.1 non.epi.lncRNA

AL008635.1 non.epi.lncRNA

AL008636.1 non.epi.lncRNA

AL008638.1 non.epi.lncRNA

AL008638.2 non.epi.lncRNA

AL008638.3 non.epi.lncRNA

AL008638.4 non.epi.lncRNA

AL008638.5 non.epi.lncRNA

AL008638.6 non.epi.lncRNA

AL008718.2 non.epi.lncRNA

AL008718.3 non.epi.lncRNA

AL008719.1 non.epi.lncRNA

AL008721.1 non.epi.lncRNA

AL008721.2 non.epi.lncRNA

AL008723.1 non.epi.lncRNA

AL008723.2 non.epi.lncRNA

AL008726.1 non.epi.lncRNA

AL008727.1 non.epi.lncRNA

AL008729.2 non.epi.lncRNA

AL008733.1 non.epi.lncRNA

AL009050.1 non.epi.lncRNA

AL009178.2 non.epi.lncRNA

AL009181.1 non.epi.lncRNA

AL020993.1 non.epi.lncRNA

AL020994.1 non.epi.lncRNA

AL020994.2 non.epi.lncRNA

AL020994.3 non.epi.lncRNA

AL020995.1 non.epi.lncRNA

AL020996.1 non.epi.lncRNA

AL020996.3 non.epi.lncRNA

AL020997.2 non.epi.lncRNA

AL020997.3 non.epi.lncRNA

AL021026.1 non.epi.lncRNA

AL021153.1 non.epi.lncRNA

AL021154.1 non.epi.lncRNA

AL021328.1 non.epi.lncRNA

AL021368.1 non.epi.lncRNA

AL021368.2 non.epi.lncRNA

AL021368.3 non.epi.lncRNA

AL021391.1 non.epi.lncRNA

AL021393.1 non.epi.lncRNA

AL021395.1 non.epi.lncRNA

AL021578.1 non.epi.lncRNA

AL021707.1 non.epi.lncRNA

AL021707.2 non.epi.lncRNA

AL021707.3 non.epi.lncRNA

AL021707.4 non.epi.lncRNA

AL021707.5 non.epi.lncRNA

AL021707.6 non.epi.lncRNA

AL021707.7 non.epi.lncRNA

AL021707.8 non.epi.lncRNA

AL021807.1 non.epi.lncRNA

AL021877.2 non.epi.lncRNA

AL021878.2 non.epi.lncRNA

AL021920.1 non.epi.lncRNA

AL021937.1 non.epi.lncRNA

AL021937.3 non.epi.lncRNA

AL021937.4 non.epi.lncRNA

AL021940.1 non.epi.lncRNA

AL022067.1 non.epi.lncRNA

AL022068.1 non.epi.lncRNA

AL022069.1 non.epi.lncRNA

AL022097.1 non.epi.lncRNA

AL022238.2 non.epi.lncRNA

AL022238.3 non.epi.lncRNA

AL022310.1 non.epi.lncRNA

AL022311.1 non.epi.lncRNA

AL022313.3 non.epi.lncRNA

AL022314.1 non.epi.lncRNA

AL022315.1 non.epi.lncRNA

AL022318.1 non.epi.lncRNA

AL022322.1 non.epi.lncRNA

AL022322.2 non.epi.lncRNA

AL022323.2 non.epi.lncRNA

AL022323.4 non.epi.lncRNA

AL022323.5 non.epi.lncRNA

AL022324.3 non.epi.lncRNA

AL022326.1 non.epi.lncRNA

AL022328.1 non.epi.lncRNA

AL022328.2 non.epi.lncRNA

AL022328.3 non.epi.lncRNA

AL022329.2 non.epi.lncRNA

AL022329.3 non.epi.lncRNA

AL022332.1 non.epi.lncRNA

AL022334.2 non.epi.lncRNA

AL022337.1 non.epi.lncRNA

AL022341.1 non.epi.lncRNA

AL022341.2 non.epi.lncRNA

AL022396.1 non.epi.lncRNA

AL022476.1 non.epi.lncRNA

AL022724.2 non.epi.lncRNA

AL023284.4 non.epi.lncRNA

AL023495.1 non.epi.lncRNA

AL023581.1 non.epi.lncRNA

AL023583.1 non.epi.lncRNA

AL023584.1 non.epi.lncRNA

AL023584.2 non.epi.lncRNA

AL023653.1 non.epi.lncRNA

AL023693.1 non.epi.lncRNA

AL023754.1 non.epi.lncRNA

AL023755.1 non.epi.lncRNA

AL023802.1 non.epi.lncRNA

AL023803.1 non.epi.lncRNA

AL023803.2 non.epi.lncRNA

AL023803.3 non.epi.lncRNA

AL023806.1 non.epi.lncRNA

AL023806.2 non.epi.lncRNA

AL023881.1 non.epi.lncRNA

AL024474.2 non.epi.lncRNA

AL024497.1 non.epi.lncRNA

AL024497.2 non.epi.lncRNA

AL024507.2 non.epi.lncRNA

AL024508.1 non.epi.lncRNA

AL024508.2 non.epi.lncRNA

AL031005.1 non.epi.lncRNA

AL031008.1 non.epi.lncRNA

AL031055.1 non.epi.lncRNA

AL031056.1 non.epi.lncRNA

AL031118.1 non.epi.lncRNA

AL031123.1 non.epi.lncRNA

AL031123.2 non.epi.lncRNA

AL031186.1 non.epi.lncRNA

AL031275.1 non.epi.lncRNA

AL031280.1 non.epi.lncRNA

AL031282.2 non.epi.lncRNA

AL031283.1 non.epi.lncRNA

AL031283.2 non.epi.lncRNA

AL031283.3 non.epi.lncRNA

AL031289.1 non.epi.lncRNA

AL031386.1 non.epi.lncRNA

AL031428.1 non.epi.lncRNA

AL031429.1 non.epi.lncRNA

AL031429.2 non.epi.lncRNA

AL031432.1 non.epi.lncRNA

AL031584.3 non.epi.lncRNA

AL031587.1 non.epi.lncRNA

AL031587.2 non.epi.lncRNA

AL031587.3 non.epi.lncRNA

AL031587.4 non.epi.lncRNA

AL031593.1 non.epi.lncRNA

AL031595.2 non.epi.lncRNA

AL031595.3 non.epi.lncRNA

AL031598.1 non.epi.lncRNA

AL031599.1 non.epi.lncRNA

AL031600.1 non.epi.lncRNA

AL031600.3 non.epi.lncRNA

AL031651.1 non.epi.lncRNA

AL031651.2 non.epi.lncRNA

AL031656.1 non.epi.lncRNA

AL031658.1 non.epi.lncRNA

AL031663.2 non.epi.lncRNA

AL031663.3 non.epi.lncRNA

AL031666.1 non.epi.lncRNA

AL031667.3 non.epi.lncRNA

AL031668.1 non.epi.lncRNA

AL031670.1 non.epi.lncRNA

AL031673.1 non.epi.lncRNA

AL031674.1 non.epi.lncRNA

AL031676.1 non.epi.lncRNA

AL031687.1 non.epi.lncRNA

AL031705.1 non.epi.lncRNA

AL031709.1 non.epi.lncRNA

AL031710.2 non.epi.lncRNA

AL031714.1 non.epi.lncRNA

AL031716.1 non.epi.lncRNA

AL031722.1 non.epi.lncRNA

AL031726.1 non.epi.lncRNA

AL031728.1 non.epi.lncRNA

AL031767.1 non.epi.lncRNA

AL031768.1 non.epi.lncRNA

AL031770.1 non.epi.lncRNA

AL031772.1 non.epi.lncRNA

AL031773.1 non.epi.lncRNA

AL031775.1 non.epi.lncRNA

AL031775.2 non.epi.lncRNA

AL031779.1 non.epi.lncRNA

AL031846.2 non.epi.lncRNA

AL031848.1 non.epi.lncRNA

AL031963.1 non.epi.lncRNA

AL031963.3 non.epi.lncRNA

AL031985.3 non.epi.lncRNA

AL032819.1 non.epi.lncRNA

AL032821.1 non.epi.lncRNA

AL033379.1 non.epi.lncRNA

AL033380.1 non.epi.lncRNA

AL033381.1 non.epi.lncRNA

AL033384.2 non.epi.lncRNA

AL033397.1 non.epi.lncRNA

AL033397.2 non.epi.lncRNA

AL033504.1 non.epi.lncRNA

AL033519.4 non.epi.lncRNA

AL033523.1 non.epi.lncRNA

AL033527.2 non.epi.lncRNA

AL033527.3 non.epi.lncRNA

AL033539.1 non.epi.lncRNA

AL033543.1 non.epi.lncRNA

AL034346.1 non.epi.lncRNA

AL034347.1 non.epi.lncRNA

AL034349.1 non.epi.lncRNA

AL034376.1 non.epi.lncRNA

AL034376.2 non.epi.lncRNA

AL034380.1 non.epi.lncRNA

AL034397.2 non.epi.lncRNA

AL034397.3 non.epi.lncRNA

AL034405.1 non.epi.lncRNA

AL034428.1 non.epi.lncRNA

AL034546.1 non.epi.lncRNA

AL034548.1 non.epi.lncRNA

AL034550.1 non.epi.lncRNA

AL034550.2 non.epi.lncRNA

AL035045.1 non.epi.lncRNA

AL035071.1 non.epi.lncRNA

AL035078.2 non.epi.lncRNA

AL035250.2 non.epi.lncRNA

AL035252.2 non.epi.lncRNA

AL035258.1 non.epi.lncRNA

AL035401.1 non.epi.lncRNA

AL035404.2 non.epi.lncRNA

AL035409.1 non.epi.lncRNA

AL035415.1 non.epi.lncRNA

AL035416.1 non.epi.lncRNA

AL035420.2 non.epi.lncRNA

AL035420.3 non.epi.lncRNA

AL035425.1 non.epi.lncRNA

AL035425.3 non.epi.lncRNA

AL035427.1 non.epi.lncRNA

AL035443.1 non.epi.lncRNA

AL035446.1 non.epi.lncRNA

AL035448.1 non.epi.lncRNA

AL035454.1 non.epi.lncRNA

AL035458.2 non.epi.lncRNA

AL035461.2 non.epi.lncRNA

AL035530.1 non.epi.lncRNA

AL035530.2 non.epi.lncRNA

AL035541.1 non.epi.lncRNA

AL035587.1 non.epi.lncRNA

AL035604.1 non.epi.lncRNA

AL035658.1 non.epi.lncRNA

AL035661.1 non.epi.lncRNA

AL035665.1 non.epi.lncRNA

AL035670.1 non.epi.lncRNA

AL035681.1 non.epi.lncRNA

AL035691.1 non.epi.lncRNA

AL035696.1 non.epi.lncRNA

AL035696.3 non.epi.lncRNA

AL035701.1 non.epi.lncRNA

AL035706.1 non.epi.lncRNA

AL049536.1 non.epi.lncRNA

AL049539.1 non.epi.lncRNA

AL049541.2 non.epi.lncRNA

AL049543.1 non.epi.lncRNA

AL049548.1 non.epi.lncRNA

AL049552.1 non.epi.lncRNA

AL049555.1 non.epi.lncRNA

AL049594.1 non.epi.lncRNA

AL049612.1 non.epi.lncRNA

AL049637.1 non.epi.lncRNA

AL049646.1 non.epi.lncRNA

AL049646.2 non.epi.lncRNA

AL049647.1 non.epi.lncRNA

AL049648.1 non.epi.lncRNA

AL049649.1 non.epi.lncRNA

AL049651.1 non.epi.lncRNA

AL049712.1 non.epi.lncRNA

AL049747.1 non.epi.lncRNA

AL049748.1 non.epi.lncRNA

AL049749.1 non.epi.lncRNA

AL049765.1 non.epi.lncRNA

AL049775.1 non.epi.lncRNA

AL049775.2 non.epi.lncRNA

AL049775.3 non.epi.lncRNA

AL049780.2 non.epi.lncRNA

AL049794.1 non.epi.lncRNA

AL049794.2 non.epi.lncRNA

AL049795.2 non.epi.lncRNA

AL049812.2 non.epi.lncRNA

AL049812.3 non.epi.lncRNA

AL049820.1 non.epi.lncRNA

AL049828.1 non.epi.lncRNA

AL049829.2 non.epi.lncRNA

AL049833.1 non.epi.lncRNA

AL049833.2 non.epi.lncRNA

AL049835.1 non.epi.lncRNA

AL049836.2 non.epi.lncRNA

AL049838.1 non.epi.lncRNA

AL049840.2 non.epi.lncRNA

AL049840.3 non.epi.lncRNA

AL049840.4 non.epi.lncRNA

AL049840.6 non.epi.lncRNA

AL049870.3 non.epi.lncRNA

AL049871.1 non.epi.lncRNA

AL049874.3 non.epi.lncRNA

AL050303.1 non.epi.lncRNA

AL050309.1 non.epi.lncRNA

AL050320.1 non.epi.lncRNA

AL050327.1 non.epi.lncRNA

AL050331.2 non.epi.lncRNA

AL050338.1 non.epi.lncRNA

AL050343.2 non.epi.lncRNA

AL050343.3 non.epi.lncRNA

AL050344.1 non.epi.lncRNA

AL050349.1 non.epi.lncRNA

AL050402.1 non.epi.lncRNA

AL050403.1 non.epi.lncRNA

AL050403.2 non.epi.lncRNA

AL050404.1 non.epi.lncRNA

AL078581.2 non.epi.lncRNA

AL078581.3 non.epi.lncRNA

AL078587.1 non.epi.lncRNA

AL078590.1 non.epi.lncRNA

AL078590.2 non.epi.lncRNA

AL078599.2 non.epi.lncRNA

AL078602.1 non.epi.lncRNA

AL078604.2 non.epi.lncRNA

AL078605.1 non.epi.lncRNA

AL078612.1 non.epi.lncRNA

AL078612.2 non.epi.lncRNA

AL078623.1 non.epi.lncRNA

AL078645.1 non.epi.lncRNA

AL079301.1 non.epi.lncRNA

AL079303.1 non.epi.lncRNA

AL079304.1 non.epi.lncRNA

AL079307.1 non.epi.lncRNA

AL079307.2 non.epi.lncRNA

AL079343.1 non.epi.lncRNA

AL080248.1 non.epi.lncRNA

AL080250.1 non.epi.lncRNA

AL080273.1 non.epi.lncRNA

AL080276.2 non.epi.lncRNA

AL080284.1 non.epi.lncRNA

AL080312.2 non.epi.lncRNA

AL080313.1 non.epi.lncRNA

AL080313.2 non.epi.lncRNA

AL080316.1 non.epi.lncRNA

AL080317.1 non.epi.lncRNA

AL080317.2 non.epi.lncRNA

AL096677.1 non.epi.lncRNA

AL096677.2 non.epi.lncRNA

AL096701.3 non.epi.lncRNA

AL096772.1 non.epi.lncRNA

AL096799.1 non.epi.lncRNA

AL096803.2 non.epi.lncRNA

AL096803.4 non.epi.lncRNA

AL096828.1 non.epi.lncRNA

AL096828.2 non.epi.lncRNA

AL096828.3 non.epi.lncRNA

AL096865.1 non.epi.lncRNA

AL096869.1 non.epi.lncRNA

AL096869.2 non.epi.lncRNA

AL096869.3 non.epi.lncRNA

AL096870.1 non.epi.lncRNA

AL109610.1 non.epi.lncRNA

AL109613.1 non.epi.lncRNA

AL109615.3 non.epi.lncRNA

AL109659.1 non.epi.lncRNA

AL109659.2 non.epi.lncRNA

AL109741.1 non.epi.lncRNA

AL109761.1 non.epi.lncRNA

AL109763.1 non.epi.lncRNA

AL109767.1 non.epi.lncRNA

AL109804.1 non.epi.lncRNA

AL109807.1 non.epi.lncRNA

AL109811.1 non.epi.lncRNA

AL109811.2 non.epi.lncRNA

AL109838.1 non.epi.lncRNA

AL109910.1 non.epi.lncRNA

AL109910.2 non.epi.lncRNA

AL109914.1 non.epi.lncRNA

AL109923.1 non.epi.lncRNA

AL109924.1 non.epi.lncRNA

AL109924.2 non.epi.lncRNA

AL109930.1 non.epi.lncRNA

AL109933.1 non.epi.lncRNA

AL109935.1 non.epi.lncRNA

AL109945.1 non.epi.lncRNA

AL109946.1 non.epi.lncRNA

AL109954.1 non.epi.lncRNA

AL109954.2 non.epi.lncRNA

AL109955.1 non.epi.lncRNA

AL110115.1 non.epi.lncRNA

AL110292.1 non.epi.lncRNA

AL110503.1 non.epi.lncRNA

AL110504.1 non.epi.lncRNA

AL110505.1 non.epi.lncRNA

AL117190.1 non.epi.lncRNA

AL117190.2 non.epi.lncRNA

AL117209.1 non.epi.lncRNA

AL117327.1 non.epi.lncRNA

AL117329.1 non.epi.lncRNA

AL117335.1 non.epi.lncRNA

AL117336.1 non.epi.lncRNA

AL117336.2 non.epi.lncRNA

AL117339.4 non.epi.lncRNA

AL117341.1 non.epi.lncRNA

AL117348.1 non.epi.lncRNA

AL117372.1 non.epi.lncRNA

AL117379.1 non.epi.lncRNA

AL117381.1 non.epi.lncRNA

AL117382.1 non.epi.lncRNA

AL117382.2 non.epi.lncRNA

AL118505.1 non.epi.lncRNA

AL118506.1 non.epi.lncRNA

AL118508.1 non.epi.lncRNA

AL118511.2 non.epi.lncRNA

AL118516.1 non.epi.lncRNA

AL118523.1 non.epi.lncRNA

AL118556.1 non.epi.lncRNA

AL118558.4 non.epi.lncRNA

AL121578.3 non.epi.lncRNA

AL121583.1 non.epi.lncRNA

AL121584.1 non.epi.lncRNA

AL121601.1 non.epi.lncRNA

AL121603.2 non.epi.lncRNA

AL121612.1 non.epi.lncRNA

AL121652.1 non.epi.lncRNA

AL121655.1 non.epi.lncRNA

AL121658.1 non.epi.lncRNA

AL121672.1 non.epi.lncRNA

AL121672.2 non.epi.lncRNA

AL121672.3 non.epi.lncRNA

AL121694.1 non.epi.lncRNA

AL121718.1 non.epi.lncRNA

AL121748.1 non.epi.lncRNA

AL121749.1 non.epi.lncRNA

AL121757.1 non.epi.lncRNA

AL121757.2 non.epi.lncRNA

AL121757.3 non.epi.lncRNA

AL121759.1 non.epi.lncRNA

AL121761.1 non.epi.lncRNA

AL121768.1 non.epi.lncRNA

AL121772.2 non.epi.lncRNA

AL121781.1 non.epi.lncRNA

AL121782.1 non.epi.lncRNA

AL121787.1 non.epi.lncRNA

AL121790.1 non.epi.lncRNA

AL121796.1 non.epi.lncRNA

AL121809.1 non.epi.lncRNA

AL121809.2 non.epi.lncRNA

AL121820.1 non.epi.lncRNA

AL121820.2 non.epi.lncRNA

AL121821.2 non.epi.lncRNA

AL121823.1 non.epi.lncRNA

AL121827.1 non.epi.lncRNA

AL121827.2 non.epi.lncRNA

AL121829.1 non.epi.lncRNA

AL121829.2 non.epi.lncRNA

AL121830.1 non.epi.lncRNA

AL121832.1 non.epi.lncRNA

AL121832.2 non.epi.lncRNA

AL121839.2 non.epi.lncRNA

AL121845.1 non.epi.lncRNA

AL121845.4 non.epi.lncRNA

AL121852.1 non.epi.lncRNA

AL121872.1 non.epi.lncRNA

AL121885.1 non.epi.lncRNA

AL121885.2 non.epi.lncRNA

AL121885.3 non.epi.lncRNA

AL121890.2 non.epi.lncRNA

AL121890.3 non.epi.lncRNA

AL121890.4 non.epi.lncRNA

AL121890.5 non.epi.lncRNA

AL121892.1 non.epi.lncRNA

AL121894.1 non.epi.lncRNA

AL121894.2 non.epi.lncRNA

AL121895.1 non.epi.lncRNA

AL121895.2 non.epi.lncRNA

AL121899.1 non.epi.lncRNA

AL121901.1 non.epi.lncRNA

AL121902.1 non.epi.lncRNA

AL121906.2 non.epi.lncRNA

AL121908.1 non.epi.lncRNA

AL121910.1 non.epi.lncRNA

AL121917.2 non.epi.lncRNA

AL121928.1 non.epi.lncRNA

AL121929.1 non.epi.lncRNA

AL121929.2 non.epi.lncRNA

AL121944.1 non.epi.lncRNA

AL121956.1 non.epi.lncRNA

AL121956.4 non.epi.lncRNA

AL121957.1 non.epi.lncRNA

AL121970.1 non.epi.lncRNA

AL121972.1 non.epi.lncRNA

AL121974.1 non.epi.lncRNA

AL121988.1 non.epi.lncRNA

AL121990.1 non.epi.lncRNA

AL121992.1 non.epi.lncRNA

AL121992.2 non.epi.lncRNA

AL121992.3 non.epi.lncRNA

AL121999.1 non.epi.lncRNA

AL122008.1 non.epi.lncRNA

AL122008.3 non.epi.lncRNA

AL122008.4 non.epi.lncRNA

AL122010.1 non.epi.lncRNA

AL122017.1 non.epi.lncRNA

AL122034.1 non.epi.lncRNA

AL122035.1 non.epi.lncRNA

AL122035.2 non.epi.lncRNA

AL122058.1 non.epi.lncRNA

AL122125.1 non.epi.lncRNA

AL132639.2 non.epi.lncRNA

AL132639.3 non.epi.lncRNA

AL132642.1 non.epi.lncRNA

AL132655.2 non.epi.lncRNA

AL132655.3 non.epi.lncRNA

AL132656.2 non.epi.lncRNA

AL132656.3 non.epi.lncRNA

AL132657.1 non.epi.lncRNA

AL132657.2 non.epi.lncRNA

AL132708.1 non.epi.lncRNA

AL132709.7 non.epi.lncRNA

AL132712.1 non.epi.lncRNA

AL132719.1 non.epi.lncRNA

AL132765.2 non.epi.lncRNA

AL132780.2 non.epi.lncRNA

AL132796.1 non.epi.lncRNA

AL132796.2 non.epi.lncRNA

AL132800.1 non.epi.lncRNA

AL132801.1 non.epi.lncRNA

AL132819.1 non.epi.lncRNA

AL132822.1 non.epi.lncRNA

AL132855.1 non.epi.lncRNA

AL132875.2 non.epi.lncRNA

AL132989.1 non.epi.lncRNA

AL132996.1 non.epi.lncRNA

AL133153.2 non.epi.lncRNA

AL133163.3 non.epi.lncRNA

AL133166.1 non.epi.lncRNA

AL133167.1 non.epi.lncRNA

AL133215.1 non.epi.lncRNA

AL133215.3 non.epi.lncRNA

AL133216.1 non.epi.lncRNA

AL133227.1 non.epi.lncRNA

AL133232.1 non.epi.lncRNA

AL133240.1 non.epi.lncRNA

AL133243.1 non.epi.lncRNA

AL133243.3 non.epi.lncRNA

AL133245.1 non.epi.lncRNA

AL133247.1 non.epi.lncRNA

AL133255.1 non.epi.lncRNA

AL133268.2 non.epi.lncRNA

AL133268.3 non.epi.lncRNA

AL133279.2 non.epi.lncRNA

AL133279.3 non.epi.lncRNA

AL133284.1 non.epi.lncRNA

AL133297.1 non.epi.lncRNA

AL133297.2 non.epi.lncRNA

AL133299.1 non.epi.lncRNA

AL133304.1 non.epi.lncRNA

AL133304.3 non.epi.lncRNA

AL133319.1 non.epi.lncRNA

AL133320.2 non.epi.lncRNA

AL133325.2 non.epi.lncRNA

AL133325.3 non.epi.lncRNA

AL133329.1 non.epi.lncRNA

AL133342.1 non.epi.lncRNA

AL133346.1 non.epi.lncRNA

AL133351.1 non.epi.lncRNA

AL133351.2 non.epi.lncRNA

AL133355.1 non.epi.lncRNA

AL133367.1 non.epi.lncRNA

AL133368.2 non.epi.lncRNA

AL133371.1 non.epi.lncRNA

AL133371.2 non.epi.lncRNA

AL133372.2 non.epi.lncRNA

AL133372.3 non.epi.lncRNA

AL133387.2 non.epi.lncRNA

AL133396.2 non.epi.lncRNA

AL133410.1 non.epi.lncRNA

AL133410.2 non.epi.lncRNA

AL133415.1 non.epi.lncRNA

AL133419.1 non.epi.lncRNA

AL133445.2 non.epi.lncRNA

AL133453.1 non.epi.lncRNA

AL133456.1 non.epi.lncRNA

AL133457.1 non.epi.lncRNA

AL133464.1 non.epi.lncRNA

AL133465.1 non.epi.lncRNA

AL133467.2 non.epi.lncRNA

AL133467.3 non.epi.lncRNA

AL133467.4 non.epi.lncRNA

AL133480.1 non.epi.lncRNA

AL133481.1 non.epi.lncRNA

AL133481.3 non.epi.lncRNA

AL133485.2 non.epi.lncRNA

AL133499.1 non.epi.lncRNA

AL133520.1 non.epi.lncRNA

AL133523.1 non.epi.lncRNA

AL133551.1 non.epi.lncRNA

AL133553.1 non.epi.lncRNA

AL135746.1 non.epi.lncRNA

AL135786.2 non.epi.lncRNA

AL135787.1 non.epi.lncRNA

AL135790.1 non.epi.lncRNA

AL135791.1 non.epi.lncRNA

AL135818.1 non.epi.lncRNA

AL135818.2 non.epi.lncRNA

AL135838.1 non.epi.lncRNA

AL135878.1 non.epi.lncRNA

AL135902.1 non.epi.lncRNA

AL135902.2 non.epi.lncRNA

AL135905.1 non.epi.lncRNA

AL135908.1 non.epi.lncRNA

AL135910.1 non.epi.lncRNA

AL135923.1 non.epi.lncRNA

AL135923.2 non.epi.lncRNA

AL135924.2 non.epi.lncRNA

AL135925.1 non.epi.lncRNA

AL135934.1 non.epi.lncRNA

AL135936.1 non.epi.lncRNA

AL135937.1 non.epi.lncRNA

AL135960.1 non.epi.lncRNA

AL135999.1 non.epi.lncRNA

AL135999.2 non.epi.lncRNA

AL135999.3 non.epi.lncRNA

AL136018.1 non.epi.lncRNA

AL136038.3 non.epi.lncRNA

AL136038.5 non.epi.lncRNA

AL136040.1 non.epi.lncRNA

AL136084.2 non.epi.lncRNA

AL136090.1 non.epi.lncRNA

AL136097.2 non.epi.lncRNA

AL136099.1 non.epi.lncRNA

AL136114.1 non.epi.lncRNA

AL136115.2 non.epi.lncRNA

AL136131.3 non.epi.lncRNA

AL136140.1 non.epi.lncRNA

AL136146.2 non.epi.lncRNA

AL136162.1 non.epi.lncRNA

AL136164.1 non.epi.lncRNA

AL136164.2 non.epi.lncRNA

AL136172.1 non.epi.lncRNA

AL136181.1 non.epi.lncRNA

AL136221.1 non.epi.lncRNA

AL136234.1 non.epi.lncRNA

AL136295.6 non.epi.lncRNA

AL136295.7 non.epi.lncRNA

AL136298.1 non.epi.lncRNA

AL136301.1 non.epi.lncRNA

AL136304.1 non.epi.lncRNA

AL136307.1 non.epi.lncRNA

AL136309.3 non.epi.lncRNA

AL136317.2 non.epi.lncRNA

AL136320.1 non.epi.lncRNA

AL136322.1 non.epi.lncRNA

AL136359.1 non.epi.lncRNA

AL136360.2 non.epi.lncRNA

AL136361.1 non.epi.lncRNA

AL136366.1 non.epi.lncRNA

AL136368.1 non.epi.lncRNA

AL136369.1 non.epi.lncRNA

AL136369.2 non.epi.lncRNA

AL136376.1 non.epi.lncRNA

AL136418.1 non.epi.lncRNA

AL136439.1 non.epi.lncRNA

AL136456.1 non.epi.lncRNA

AL136460.1 non.epi.lncRNA

AL136501.1 non.epi.lncRNA

AL136526.1 non.epi.lncRNA

AL136531.1 non.epi.lncRNA

AL136537.1 non.epi.lncRNA

AL136962.1 non.epi.lncRNA

AL136964.1 non.epi.lncRNA

AL136972.1 non.epi.lncRNA

AL136979.1 non.epi.lncRNA

AL136980.1 non.epi.lncRNA

AL136982.1 non.epi.lncRNA

AL136982.2 non.epi.lncRNA

AL136982.3 non.epi.lncRNA

AL136982.6 non.epi.lncRNA

AL136984.1 non.epi.lncRNA

AL136985.1 non.epi.lncRNA

AL136985.2 non.epi.lncRNA

AL136987.1 non.epi.lncRNA

AL136988.1 non.epi.lncRNA

AL136988.2 non.epi.lncRNA

AL137001.1 non.epi.lncRNA

AL137002.1 non.epi.lncRNA

AL137003.1 non.epi.lncRNA

AL137009.1 non.epi.lncRNA

AL137024.1 non.epi.lncRNA

AL137026.2 non.epi.lncRNA

AL137027.1 non.epi.lncRNA

AL137058.2 non.epi.lncRNA

AL137060.3 non.epi.lncRNA

AL137071.1 non.epi.lncRNA

AL137076.1 non.epi.lncRNA

AL137078.1 non.epi.lncRNA

AL137078.2 non.epi.lncRNA

AL137127.1 non.epi.lncRNA

AL137129.1 non.epi.lncRNA

AL137186.2 non.epi.lncRNA

AL137191.1 non.epi.lncRNA

AL137220.1 non.epi.lncRNA

AL137224.1 non.epi.lncRNA

AL137230.1 non.epi.lncRNA

AL137230.2 non.epi.lncRNA

AL137244.1 non.epi.lncRNA

AL137246.1 non.epi.lncRNA

AL137246.2 non.epi.lncRNA

AL137247.2 non.epi.lncRNA

AL137779.1 non.epi.lncRNA

AL137782.1 non.epi.lncRNA

AL137786.1 non.epi.lncRNA

AL137789.1 non.epi.lncRNA

AL137796.1 non.epi.lncRNA

AL137798.1 non.epi.lncRNA

AL137802.2 non.epi.lncRNA

AL137804.1 non.epi.lncRNA

AL137847.1 non.epi.lncRNA

AL137847.2 non.epi.lncRNA

AL138478.1 non.epi.lncRNA

AL138686.1 non.epi.lncRNA

AL138689.1 non.epi.lncRNA

AL138690.1 non.epi.lncRNA

AL138720.1 non.epi.lncRNA

AL138724.1 non.epi.lncRNA

AL138731.1 non.epi.lncRNA

AL138733.1 non.epi.lncRNA

AL138733.2 non.epi.lncRNA

AL138749.1 non.epi.lncRNA

AL138759.1 non.epi.lncRNA

AL138760.1 non.epi.lncRNA

AL138762.1 non.epi.lncRNA

AL138767.1 non.epi.lncRNA

AL138767.2 non.epi.lncRNA

AL138767.3 non.epi.lncRNA

AL138781.1 non.epi.lncRNA

AL138799.2 non.epi.lncRNA

AL138799.4 non.epi.lncRNA

AL138808.1 non.epi.lncRNA

AL138812.1 non.epi.lncRNA

AL138820.1 non.epi.lncRNA

AL138826.1 non.epi.lncRNA

AL138828.1 non.epi.lncRNA

AL138830.1 non.epi.lncRNA

AL138830.2 non.epi.lncRNA

AL138831.1 non.epi.lncRNA

AL138831.2 non.epi.lncRNA

AL138885.2 non.epi.lncRNA

AL138889.1 non.epi.lncRNA

AL138895.1 non.epi.lncRNA

AL138899.1 non.epi.lncRNA

AL138900.1 non.epi.lncRNA

AL138918.1 non.epi.lncRNA

AL138921.2 non.epi.lncRNA

AL138930.1 non.epi.lncRNA

AL138955.1 non.epi.lncRNA

AL138957.1 non.epi.lncRNA

AL138960.1 non.epi.lncRNA

AL138962.1 non.epi.lncRNA

AL138963.1 non.epi.lncRNA

AL138963.4 non.epi.lncRNA

AL138966.2 non.epi.lncRNA

AL138995.1 non.epi.lncRNA

AL138999.1 non.epi.lncRNA

AL139002.1 non.epi.lncRNA

AL139008.2 non.epi.lncRNA

AL139011.1 non.epi.lncRNA

AL139020.1 non.epi.lncRNA

AL139021.1 non.epi.lncRNA

AL139021.2 non.epi.lncRNA

AL139022.1 non.epi.lncRNA

AL139022.2 non.epi.lncRNA

AL139023.1 non.epi.lncRNA

AL139041.1 non.epi.lncRNA

AL139042.1 non.epi.lncRNA

AL139081.1 non.epi.lncRNA

AL139082.1 non.epi.lncRNA

AL139095.4 non.epi.lncRNA

AL139099.1 non.epi.lncRNA

AL139099.2 non.epi.lncRNA

AL139118.1 non.epi.lncRNA

AL139120.1 non.epi.lncRNA

AL139123.1 non.epi.lncRNA

AL139124.1 non.epi.lncRNA

AL139130.1 non.epi.lncRNA

AL139142.1 non.epi.lncRNA

AL139147.1 non.epi.lncRNA

AL139193.1 non.epi.lncRNA

AL139193.2 non.epi.lncRNA

AL139220.2 non.epi.lncRNA

AL139230.1 non.epi.lncRNA

AL139231.1 non.epi.lncRNA

AL139237.1 non.epi.lncRNA

AL139240.1 non.epi.lncRNA

AL139241.1 non.epi.lncRNA

AL139246.1 non.epi.lncRNA

AL139246.3 non.epi.lncRNA

AL139246.5 non.epi.lncRNA

AL139254.1 non.epi.lncRNA

AL139260.2 non.epi.lncRNA

AL139275.2 non.epi.lncRNA

AL139280.1 non.epi.lncRNA

AL139286.2 non.epi.lncRNA

AL139289.1 non.epi.lncRNA

AL139289.2 non.epi.lncRNA

AL139294.1 non.epi.lncRNA

AL139316.1 non.epi.lncRNA

AL139317.1 non.epi.lncRNA

AL139327.2 non.epi.lncRNA

AL139348.1 non.epi.lncRNA

AL139349.1 non.epi.lncRNA

AL139350.1 non.epi.lncRNA

AL139351.1 non.epi.lncRNA

AL139353.2 non.epi.lncRNA

AL139354.1 non.epi.lncRNA

AL139383.1 non.epi.lncRNA

AL139385.1 non.epi.lncRNA

AL139390.1 non.epi.lncRNA

AL139393.1 non.epi.lncRNA

AL139393.2 non.epi.lncRNA

AL139393.3 non.epi.lncRNA

AL139397.1 non.epi.lncRNA

AL139407.1 non.epi.lncRNA

AL139420.1 non.epi.lncRNA

AL139420.2 non.epi.lncRNA

AL139423.1 non.epi.lncRNA

AL139424.1 non.epi.lncRNA

AL139807.1 non.epi.lncRNA

AL139819.1 non.epi.lncRNA

AL139824.1 non.epi.lncRNA

AL157359.1 non.epi.lncRNA

AL157359.2 non.epi.lncRNA

AL157371.1 non.epi.lncRNA

AL157373.1 non.epi.lncRNA

AL157373.2 non.epi.lncRNA

AL157378.1 non.epi.lncRNA

AL157387.1 non.epi.lncRNA

AL157388.1 non.epi.lncRNA

AL157392.1 non.epi.lncRNA

AL157392.2 non.epi.lncRNA

AL157392.3 non.epi.lncRNA

AL157392.4 non.epi.lncRNA

AL157394.1 non.epi.lncRNA

AL157395.1 non.epi.lncRNA

AL157396.1 non.epi.lncRNA

AL157400.1 non.epi.lncRNA

AL157400.2 non.epi.lncRNA

AL157400.3 non.epi.lncRNA

AL157400.4 non.epi.lncRNA

AL157402.1 non.epi.lncRNA

AL157413.1 non.epi.lncRNA

AL157414.1 non.epi.lncRNA

AL157414.2 non.epi.lncRNA

AL157688.1 non.epi.lncRNA

AL157700.1 non.epi.lncRNA

AL157709.1 non.epi.lncRNA

AL157762.1 non.epi.lncRNA

AL157778.1 non.epi.lncRNA

AL157786.1 non.epi.lncRNA

AL157813.1 non.epi.lncRNA

AL157817.1 non.epi.lncRNA

AL157823.2 non.epi.lncRNA

AL157831.2 non.epi.lncRNA

AL157832.1 non.epi.lncRNA

AL157832.2 non.epi.lncRNA

AL157834.2 non.epi.lncRNA

AL157871.2 non.epi.lncRNA

AL157871.4 non.epi.lncRNA

AL157871.5 non.epi.lncRNA

AL157882.1 non.epi.lncRNA

AL157895.1 non.epi.lncRNA

AL157896.1 non.epi.lncRNA

AL157904.1 non.epi.lncRNA

AL157911.1 non.epi.lncRNA

AL157912.1 non.epi.lncRNA

AL157931.1 non.epi.lncRNA

AL157932.1 non.epi.lncRNA

AL157935.1 non.epi.lncRNA

AL157937.1 non.epi.lncRNA

AL157938.2 non.epi.lncRNA

AL157944.1 non.epi.lncRNA

AL157955.1 non.epi.lncRNA

AL158013.1 non.epi.lncRNA

AL158042.1 non.epi.lncRNA

AL158055.1 non.epi.lncRNA

AL158058.1 non.epi.lncRNA

AL158063.1 non.epi.lncRNA

AL158066.1 non.epi.lncRNA

AL158068.2 non.epi.lncRNA

AL158069.1 non.epi.lncRNA

AL158070.1 non.epi.lncRNA

AL158071.1 non.epi.lncRNA

AL158071.3 non.epi.lncRNA

AL158071.4 non.epi.lncRNA

AL158071.5 non.epi.lncRNA

AL158090.1 non.epi.lncRNA

AL158147.1 non.epi.lncRNA

AL158151.1 non.epi.lncRNA

AL158151.2 non.epi.lncRNA

AL158151.3 non.epi.lncRNA

AL158152.1 non.epi.lncRNA

AL158154.2 non.epi.lncRNA

AL158154.3 non.epi.lncRNA

AL158163.1 non.epi.lncRNA

AL158163.2 non.epi.lncRNA

AL158166.1 non.epi.lncRNA

AL158166.2 non.epi.lncRNA

AL158168.1 non.epi.lncRNA

AL158196.1 non.epi.lncRNA

AL158198.1 non.epi.lncRNA

AL158206.1 non.epi.lncRNA

AL158207.2 non.epi.lncRNA

AL158211.1 non.epi.lncRNA

AL158211.2 non.epi.lncRNA

AL158211.3 non.epi.lncRNA

AL158211.4 non.epi.lncRNA

AL158212.1 non.epi.lncRNA

AL158212.2 non.epi.lncRNA

AL158212.3 non.epi.lncRNA

AL158817.1 non.epi.lncRNA

AL158825.2 non.epi.lncRNA

AL158828.1 non.epi.lncRNA

AL158832.1 non.epi.lncRNA

AL158834.1 non.epi.lncRNA

AL158834.2 non.epi.lncRNA

AL158835.1 non.epi.lncRNA

AL158835.2 non.epi.lncRNA

AL158835.3 non.epi.lncRNA

AL158837.1 non.epi.lncRNA

AL158839.1 non.epi.lncRNA

AL158840.1 non.epi.lncRNA

AL158847.1 non.epi.lncRNA

AL158850.1 non.epi.lncRNA

AL159166.1 non.epi.lncRNA

AL159169.2 non.epi.lncRNA

AL159169.3 non.epi.lncRNA

AL159174.1 non.epi.lncRNA

AL159972.1 non.epi.lncRNA

AL159990.1 non.epi.lncRNA

AL159990.2 non.epi.lncRNA

AL160004.1 non.epi.lncRNA

AL160006.1 non.epi.lncRNA

AL160153.1 non.epi.lncRNA

AL160163.1 non.epi.lncRNA

AL160191.1 non.epi.lncRNA

AL160236.2 non.epi.lncRNA

AL160237.1 non.epi.lncRNA

AL160262.1 non.epi.lncRNA

AL160271.2 non.epi.lncRNA

AL160286.3 non.epi.lncRNA

AL160290.1 non.epi.lncRNA

AL160291.1 non.epi.lncRNA

AL160313.1 non.epi.lncRNA

AL160313.2 non.epi.lncRNA

AL160408.1 non.epi.lncRNA

AL160408.3 non.epi.lncRNA

AL160408.5 non.epi.lncRNA

AL160412.1 non.epi.lncRNA

AL160413.1 non.epi.lncRNA

AL161452.1 non.epi.lncRNA

AL161457.1 non.epi.lncRNA

AL161457.2 non.epi.lncRNA

AL161616.2 non.epi.lncRNA

AL161629.1 non.epi.lncRNA

AL161630.1 non.epi.lncRNA

AL161636.1 non.epi.lncRNA

AL161638.1 non.epi.lncRNA

AL161638.2 non.epi.lncRNA

AL161640.1 non.epi.lncRNA

AL161644.1 non.epi.lncRNA

AL161646.1 non.epi.lncRNA

AL161668.1 non.epi.lncRNA

AL161668.2 non.epi.lncRNA

AL161668.3 non.epi.lncRNA

AL161668.4 non.epi.lncRNA

AL161669.1 non.epi.lncRNA

AL161669.3 non.epi.lncRNA

AL161716.1 non.epi.lncRNA

AL161719.1 non.epi.lncRNA

AL161725.1 non.epi.lncRNA

AL161725.2 non.epi.lncRNA

AL161729.1 non.epi.lncRNA

AL161729.2 non.epi.lncRNA

AL161729.4 non.epi.lncRNA

AL161733.1 non.epi.lncRNA

AL161734.1 non.epi.lncRNA

AL161740.1 non.epi.lncRNA

AL161747.2 non.epi.lncRNA

AL161752.1 non.epi.lncRNA

AL161757.2 non.epi.lncRNA

AL161757.3 non.epi.lncRNA

AL161757.4 non.epi.lncRNA

AL161757.5 non.epi.lncRNA

AL161772.1 non.epi.lncRNA

AL161782.1 non.epi.lncRNA

AL161785.1 non.epi.lncRNA

AL161785.2 non.epi.lncRNA

AL161785.3 non.epi.lncRNA

AL161793.1 non.epi.lncRNA

AL161804.1 non.epi.lncRNA

AL161891.1 non.epi.lncRNA

AL161896.1 non.epi.lncRNA

AL161908.1 non.epi.lncRNA

AL161908.2 non.epi.lncRNA

AL161909.2 non.epi.lncRNA

AL161912.1 non.epi.lncRNA

AL161912.2 non.epi.lncRNA

AL161938.1 non.epi.lncRNA

AL162151.1 non.epi.lncRNA

AL162171.1 non.epi.lncRNA

AL162171.2 non.epi.lncRNA

AL162231.2 non.epi.lncRNA

AL162253.1 non.epi.lncRNA

AL162274.2 non.epi.lncRNA

AL162311.1 non.epi.lncRNA

AL162377.1 non.epi.lncRNA

AL162384.1 non.epi.lncRNA

AL162385.1 non.epi.lncRNA

AL162386.2 non.epi.lncRNA

AL162390.1 non.epi.lncRNA

AL162391.1 non.epi.lncRNA

AL162394.1 non.epi.lncRNA

AL162399.1 non.epi.lncRNA

AL162400.1 non.epi.lncRNA

AL162400.2 non.epi.lncRNA

AL162408.1 non.epi.lncRNA

AL162412.1 non.epi.lncRNA

AL162413.1 non.epi.lncRNA

AL162414.1 non.epi.lncRNA

AL162419.1 non.epi.lncRNA

AL162425.1 non.epi.lncRNA

AL162426.1 non.epi.lncRNA

AL162427.1 non.epi.lncRNA

AL162430.2 non.epi.lncRNA

AL162431.1 non.epi.lncRNA

AL162457.1 non.epi.lncRNA

AL162457.2 non.epi.lncRNA

AL162464.1 non.epi.lncRNA

AL162464.2 non.epi.lncRNA

AL162497.1 non.epi.lncRNA

AL162574.1 non.epi.lncRNA

AL162574.2 non.epi.lncRNA

AL162582.1 non.epi.lncRNA

AL162584.1 non.epi.lncRNA

AL162586.1 non.epi.lncRNA

AL162591.2 non.epi.lncRNA

AL162595.1 non.epi.lncRNA

AL162595.2 non.epi.lncRNA

AL162632.1 non.epi.lncRNA

AL162632.3 non.epi.lncRNA

AL162713.1 non.epi.lncRNA

AL162725.2 non.epi.lncRNA

AL162726.1 non.epi.lncRNA

AL162726.4 non.epi.lncRNA

AL162727.1 non.epi.lncRNA

AL162727.2 non.epi.lncRNA

AL162731.1 non.epi.lncRNA

AL162742.1 non.epi.lncRNA

AL162872.1 non.epi.lncRNA

AL163192.1 non.epi.lncRNA

AL163195.2 non.epi.lncRNA

AL163636.1 non.epi.lncRNA

AL163932.1 non.epi.lncRNA

AL163952.1 non.epi.lncRNA

AL163953.1 non.epi.lncRNA

AL163973.2 non.epi.lncRNA

AL163974.1 non.epi.lncRNA

AL352955.1 non.epi.lncRNA

AL352979.2 non.epi.lncRNA

AL353052.1 non.epi.lncRNA

AL353052.2 non.epi.lncRNA

AL353135.1 non.epi.lncRNA

AL353150.1 non.epi.lncRNA

AL353152.1 non.epi.lncRNA

AL353581.1 non.epi.lncRNA

AL353593.1 non.epi.lncRNA

AL353593.2 non.epi.lncRNA

AL353600.1 non.epi.lncRNA

AL353604.1 non.epi.lncRNA

AL353608.1 non.epi.lncRNA

AL353608.2 non.epi.lncRNA

AL353608.3 non.epi.lncRNA

AL353611.1 non.epi.lncRNA

AL353612.1 non.epi.lncRNA

AL353613.1 non.epi.lncRNA

AL353614.1 non.epi.lncRNA

AL353615.1 non.epi.lncRNA

AL353622.1 non.epi.lncRNA

AL353622.2 non.epi.lncRNA

AL353626.1 non.epi.lncRNA

AL353633.1 non.epi.lncRNA

AL353651.1 non.epi.lncRNA

AL353653.1 non.epi.lncRNA

AL353658.1 non.epi.lncRNA

AL353658.2 non.epi.lncRNA

AL353680.1 non.epi.lncRNA

AL353681.1 non.epi.lncRNA

AL353689.2 non.epi.lncRNA

AL353699.1 non.epi.lncRNA

AL353708.3 non.epi.lncRNA

AL353732.1 non.epi.lncRNA

AL353740.1 non.epi.lncRNA

AL353742.1 non.epi.lncRNA

AL353743.2 non.epi.lncRNA

AL353746.1 non.epi.lncRNA

AL353747.2 non.epi.lncRNA

AL353747.3 non.epi.lncRNA

AL353751.1 non.epi.lncRNA

AL353753.1 non.epi.lncRNA

AL353764.1 non.epi.lncRNA

AL353768.1 non.epi.lncRNA

AL353770.3 non.epi.lncRNA

AL353770.4 non.epi.lncRNA

AL353771.1 non.epi.lncRNA

AL353780.1 non.epi.lncRNA

AL353784.1 non.epi.lncRNA

AL353795.2 non.epi.lncRNA

AL353796.1 non.epi.lncRNA

AL353801.2 non.epi.lncRNA

AL353801.3 non.epi.lncRNA

AL353803.2 non.epi.lncRNA

AL353803.4 non.epi.lncRNA

AL353804.1 non.epi.lncRNA

AL353804.2 non.epi.lncRNA

AL353811.1 non.epi.lncRNA

AL353997.2 non.epi.lncRNA

AL354674.1 non.epi.lncRNA

AL354694.1 non.epi.lncRNA

AL354696.1 non.epi.lncRNA

AL354696.2 non.epi.lncRNA

AL354707.1 non.epi.lncRNA

AL354710.2 non.epi.lncRNA

AL354712.1 non.epi.lncRNA

AL354714.1 non.epi.lncRNA

AL354714.3 non.epi.lncRNA

AL354718.2 non.epi.lncRNA

AL354733.1 non.epi.lncRNA

AL354733.2 non.epi.lncRNA

AL354754.1 non.epi.lncRNA

AL354760.1 non.epi.lncRNA

AL354766.2 non.epi.lncRNA

AL354771.1 non.epi.lncRNA

AL354793.1 non.epi.lncRNA

AL354794.1 non.epi.lncRNA

AL354809.1 non.epi.lncRNA

AL354810.1 non.epi.lncRNA

AL354811.1 non.epi.lncRNA

AL354813.1 non.epi.lncRNA

AL354821.1 non.epi.lncRNA

AL354836.1 non.epi.lncRNA

AL354861.2 non.epi.lncRNA

AL354861.3 non.epi.lncRNA

AL354863.1 non.epi.lncRNA

AL354864.1 non.epi.lncRNA

AL354872.2 non.epi.lncRNA

AL354892.3 non.epi.lncRNA

AL354893.2 non.epi.lncRNA

AL354919.1 non.epi.lncRNA

AL354920.1 non.epi.lncRNA

AL354936.1 non.epi.lncRNA

AL354949.1 non.epi.lncRNA

AL354950.1 non.epi.lncRNA

AL354950.2 non.epi.lncRNA

AL354956.1 non.epi.lncRNA

AL354977.1 non.epi.lncRNA

AL354977.2 non.epi.lncRNA

AL354979.1 non.epi.lncRNA

AL354984.1 non.epi.lncRNA

AL354984.2 non.epi.lncRNA

AL354989.1 non.epi.lncRNA

AL354993.2 non.epi.lncRNA

AL354994.1 non.epi.lncRNA

AL355001.1 non.epi.lncRNA

AL355001.2 non.epi.lncRNA

AL355073.1 non.epi.lncRNA

AL355073.2 non.epi.lncRNA

AL355075.1 non.epi.lncRNA

AL355075.2 non.epi.lncRNA

AL355075.3 non.epi.lncRNA

AL355075.4 non.epi.lncRNA

AL355076.2 non.epi.lncRNA

AL355095.1 non.epi.lncRNA

AL355096.1 non.epi.lncRNA

AL355097.1 non.epi.lncRNA

AL355102.1 non.epi.lncRNA

AL355102.3 non.epi.lncRNA

AL355102.4 non.epi.lncRNA

AL355102.5 non.epi.lncRNA

AL355112.1 non.epi.lncRNA

AL355140.1 non.epi.lncRNA

AL355297.2 non.epi.lncRNA

AL355297.3 non.epi.lncRNA

AL355297.4 non.epi.lncRNA

AL355300.1 non.epi.lncRNA

AL355303.1 non.epi.lncRNA

AL355304.1 non.epi.lncRNA

AL355306.2 non.epi.lncRNA

AL355310.1 non.epi.lncRNA

AL355310.2 non.epi.lncRNA

AL355310.3 non.epi.lncRNA

AL355314.1 non.epi.lncRNA

AL355314.2 non.epi.lncRNA

AL355336.1 non.epi.lncRNA

AL355353.1 non.epi.lncRNA

AL355376.1 non.epi.lncRNA

AL355385.1 non.epi.lncRNA

AL355390.1 non.epi.lncRNA

AL355390.2 non.epi.lncRNA

AL355432.1 non.epi.lncRNA

AL355472.2 non.epi.lncRNA

AL355472.3 non.epi.lncRNA

AL355482.1 non.epi.lncRNA

AL355482.2 non.epi.lncRNA

AL355483.1 non.epi.lncRNA

AL355483.3 non.epi.lncRNA

AL355490.1 non.epi.lncRNA

AL355497.2 non.epi.lncRNA

AL355499.1 non.epi.lncRNA

AL355512.1 non.epi.lncRNA

AL355516.1 non.epi.lncRNA

AL355526.1 non.epi.lncRNA

AL355537.1 non.epi.lncRNA

AL355574.1 non.epi.lncRNA

AL355578.1 non.epi.lncRNA

AL355581.1 non.epi.lncRNA

AL355592.1 non.epi.lncRNA

AL355596.1 non.epi.lncRNA

AL355601.1 non.epi.lncRNA

AL355607.1 non.epi.lncRNA

AL355607.2 non.epi.lncRNA

AL355612.1 non.epi.lncRNA

AL355674.1 non.epi.lncRNA

AL355802.3 non.epi.lncRNA

AL355810.1 non.epi.lncRNA

AL355816.1 non.epi.lncRNA

AL355816.2 non.epi.lncRNA

AL355834.1 non.epi.lncRNA

AL355835.1 non.epi.lncRNA

AL355838.1 non.epi.lncRNA

AL355870.1 non.epi.lncRNA

AL355916.1 non.epi.lncRNA

AL355916.2 non.epi.lncRNA

AL355922.1 non.epi.lncRNA

AL355922.3 non.epi.lncRNA

AL355922.4 non.epi.lncRNA

AL355974.1 non.epi.lncRNA

AL355974.2 non.epi.lncRNA

AL355987.2 non.epi.lncRNA

AL355987.4 non.epi.lncRNA

AL355990.1 non.epi.lncRNA

AL355990.2 non.epi.lncRNA

AL355994.3 non.epi.lncRNA

AL355997.1 non.epi.lncRNA

AL356010.2 non.epi.lncRNA

AL356017.1 non.epi.lncRNA

AL356019.1 non.epi.lncRNA

AL356019.2 non.epi.lncRNA

AL356020.1 non.epi.lncRNA

AL356022.1 non.epi.lncRNA

AL356055.1 non.epi.lncRNA

AL356056.1 non.epi.lncRNA

AL356056.2 non.epi.lncRNA

AL356108.1 non.epi.lncRNA

AL356124.2 non.epi.lncRNA

AL356130.1 non.epi.lncRNA

AL356133.2 non.epi.lncRNA

AL356134.1 non.epi.lncRNA

AL356157.1 non.epi.lncRNA

AL356234.2 non.epi.lncRNA

AL356259.1 non.epi.lncRNA

AL356276.1 non.epi.lncRNA

AL356277.2 non.epi.lncRNA

AL356277.3 non.epi.lncRNA

AL356289.1 non.epi.lncRNA

AL356299.2 non.epi.lncRNA

AL356309.1 non.epi.lncRNA

AL356311.1 non.epi.lncRNA

AL356361.2 non.epi.lncRNA

AL356364.1 non.epi.lncRNA

AL356387.1 non.epi.lncRNA

AL356417.1 non.epi.lncRNA

AL356417.2 non.epi.lncRNA

AL356421.2 non.epi.lncRNA

AL356441.1 non.epi.lncRNA

AL356475.1 non.epi.lncRNA

AL356481.1 non.epi.lncRNA

AL356481.2 non.epi.lncRNA

AL356481.3 non.epi.lncRNA

AL356488.3 non.epi.lncRNA

AL356512.1 non.epi.lncRNA

AL356515.1 non.epi.lncRNA

AL356580.1 non.epi.lncRNA

AL356599.1 non.epi.lncRNA

AL356608.1 non.epi.lncRNA

AL356652.1 non.epi.lncRNA

AL356740.1 non.epi.lncRNA

AL356740.2 non.epi.lncRNA

AL356740.3 non.epi.lncRNA

AL356752.1 non.epi.lncRNA

AL356753.1 non.epi.lncRNA

AL356756.1 non.epi.lncRNA

AL356804.1 non.epi.lncRNA

AL356805.1 non.epi.lncRNA

AL356859.1 non.epi.lncRNA

AL356961.1 non.epi.lncRNA

AL356966.1 non.epi.lncRNA

AL357052.1 non.epi.lncRNA

AL357054.2 non.epi.lncRNA

AL357054.3 non.epi.lncRNA

AL357078.2 non.epi.lncRNA

AL357079.1 non.epi.lncRNA

AL357093.1 non.epi.lncRNA

AL357093.2 non.epi.lncRNA

AL357127.1 non.epi.lncRNA

AL357140.1 non.epi.lncRNA

AL357146.1 non.epi.lncRNA

AL357153.1 non.epi.lncRNA

AL357153.2 non.epi.lncRNA

AL357153.3 non.epi.lncRNA

AL357172.1 non.epi.lncRNA

AL357315.1 non.epi.lncRNA

AL357375.1 non.epi.lncRNA

AL357497.1 non.epi.lncRNA

AL357507.1 non.epi.lncRNA

AL357514.1 non.epi.lncRNA

AL357518.1 non.epi.lncRNA

AL357552.2 non.epi.lncRNA

AL357558.1 non.epi.lncRNA

AL357558.2 non.epi.lncRNA

AL357568.1 non.epi.lncRNA

AL357568.2 non.epi.lncRNA

AL357673.2 non.epi.lncRNA

AL357793.1 non.epi.lncRNA

AL357793.2 non.epi.lncRNA

AL357832.1 non.epi.lncRNA

AL357833.1 non.epi.lncRNA

AL357873.1 non.epi.lncRNA

AL357874.2 non.epi.lncRNA

AL357936.1 non.epi.lncRNA

AL357992.1 non.epi.lncRNA

AL358072.1 non.epi.lncRNA

AL358074.1 non.epi.lncRNA

AL358075.1 non.epi.lncRNA

AL358115.1 non.epi.lncRNA

AL358134.1 non.epi.lncRNA

AL358176.1 non.epi.lncRNA

AL358176.4 non.epi.lncRNA

AL358215.1 non.epi.lncRNA

AL358216.1 non.epi.lncRNA

AL358292.1 non.epi.lncRNA

AL358332.1 non.epi.lncRNA

AL358333.1 non.epi.lncRNA

AL358333.2 non.epi.lncRNA

AL358334.2 non.epi.lncRNA

AL358334.3 non.epi.lncRNA

AL358335.2 non.epi.lncRNA

AL358393.1 non.epi.lncRNA

AL358394.2 non.epi.lncRNA

AL358394.3 non.epi.lncRNA

AL358452.1 non.epi.lncRNA

AL358472.3 non.epi.lncRNA

AL358473.1 non.epi.lncRNA

AL358473.2 non.epi.lncRNA

AL358612.1 non.epi.lncRNA

AL358613.1 non.epi.lncRNA

AL358779.1 non.epi.lncRNA

AL358781.2 non.epi.lncRNA

AL358852.1 non.epi.lncRNA

AL358876.2 non.epi.lncRNA

AL358937.1 non.epi.lncRNA

AL358944.1 non.epi.lncRNA

AL358972.1 non.epi.lncRNA

AL359076.1 non.epi.lncRNA

AL359081.1 non.epi.lncRNA

AL359091.1 non.epi.lncRNA

AL359091.3 non.epi.lncRNA

AL359091.4 non.epi.lncRNA

AL359091.5 non.epi.lncRNA

AL359094.1 non.epi.lncRNA

AL359094.2 non.epi.lncRNA

AL359182.1 non.epi.lncRNA

AL359195.1 non.epi.lncRNA

AL359198.1 non.epi.lncRNA

AL359220.1 non.epi.lncRNA

AL359232.1 non.epi.lncRNA

AL359233.1 non.epi.lncRNA

AL359237.1 non.epi.lncRNA

AL359238.1 non.epi.lncRNA

AL359258.1 non.epi.lncRNA

AL359258.2 non.epi.lncRNA

AL359313.1 non.epi.lncRNA

AL359317.2 non.epi.lncRNA

AL359382.1 non.epi.lncRNA

AL359397.2 non.epi.lncRNA

AL359399.1 non.epi.lncRNA

AL359458.1 non.epi.lncRNA

AL359475.1 non.epi.lncRNA

AL359502.1 non.epi.lncRNA

AL359532.1 non.epi.lncRNA

AL359538.1 non.epi.lncRNA

AL359541.1 non.epi.lncRNA

AL359547.1 non.epi.lncRNA

AL359551.1 non.epi.lncRNA

AL359636.1 non.epi.lncRNA

AL359636.2 non.epi.lncRNA

AL359643.3 non.epi.lncRNA

AL359644.1 non.epi.lncRNA

AL359649.1 non.epi.lncRNA

AL359682.1 non.epi.lncRNA

AL359693.1 non.epi.lncRNA

AL359697.1 non.epi.lncRNA

AL359706.1 non.epi.lncRNA

AL359710.1 non.epi.lncRNA

AL359711.2 non.epi.lncRNA

AL359715.1 non.epi.lncRNA

AL359715.2 non.epi.lncRNA

AL359715.3 non.epi.lncRNA

AL359745.1 non.epi.lncRNA

AL359771.1 non.epi.lncRNA

AL359792.1 non.epi.lncRNA

AL359851.1 non.epi.lncRNA

AL359853.2 non.epi.lncRNA

AL359878.1 non.epi.lncRNA

AL359880.1 non.epi.lncRNA

AL359881.1 non.epi.lncRNA

AL359881.2 non.epi.lncRNA

AL359881.3 non.epi.lncRNA

AL359915.1 non.epi.lncRNA

AL359918.2 non.epi.lncRNA

AL359921.2 non.epi.lncRNA

AL359922.2 non.epi.lncRNA

AL359924.1 non.epi.lncRNA

AL359962.1 non.epi.lncRNA

AL359962.2 non.epi.lncRNA

AL359979.2 non.epi.lncRNA

AL360007.1 non.epi.lncRNA

AL360012.1 non.epi.lncRNA

AL360013.2 non.epi.lncRNA

AL360014.1 non.epi.lncRNA

AL360089.1 non.epi.lncRNA

AL360091.2 non.epi.lncRNA

AL360091.3 non.epi.lncRNA

AL360093.1 non.epi.lncRNA

AL360157.1 non.epi.lncRNA

AL360169.1 non.epi.lncRNA

AL360169.2 non.epi.lncRNA

AL360175.1 non.epi.lncRNA

AL360181.2 non.epi.lncRNA

AL360181.4 non.epi.lncRNA

AL360182.2 non.epi.lncRNA

AL360227.1 non.epi.lncRNA

AL360267.1 non.epi.lncRNA

AL360268.1 non.epi.lncRNA

AL360268.2 non.epi.lncRNA

AL360270.1 non.epi.lncRNA

AL360270.2 non.epi.lncRNA

AL360270.3 non.epi.lncRNA

AL360294.1 non.epi.lncRNA

AL365181.1 non.epi.lncRNA

AL365181.2 non.epi.lncRNA

AL365181.3 non.epi.lncRNA

AL365181.4 non.epi.lncRNA

AL365184.1 non.epi.lncRNA

AL365184.2 non.epi.lncRNA

AL365194.1 non.epi.lncRNA

AL365203.2 non.epi.lncRNA

AL365204.1 non.epi.lncRNA

AL365204.2 non.epi.lncRNA

AL365205.3 non.epi.lncRNA

AL365205.4 non.epi.lncRNA

AL365226.1 non.epi.lncRNA

AL365226.2 non.epi.lncRNA

AL365255.1 non.epi.lncRNA

AL365258.1 non.epi.lncRNA

AL365259.1 non.epi.lncRNA

AL365271.1 non.epi.lncRNA

AL365295.1 non.epi.lncRNA

AL365318.1 non.epi.lncRNA

AL365330.1 non.epi.lncRNA

AL365356.1 non.epi.lncRNA

AL365361.1 non.epi.lncRNA

AL365434.1 non.epi.lncRNA

AL365434.2 non.epi.lncRNA

AL365440.2 non.epi.lncRNA

AL389885.1 non.epi.lncRNA

AL389889.1 non.epi.lncRNA

AL390037.1 non.epi.lncRNA

AL390038.1 non.epi.lncRNA

AL390061.1 non.epi.lncRNA

AL390066.1 non.epi.lncRNA

AL390195.2 non.epi.lncRNA

AL390198.1 non.epi.lncRNA

AL390208.1 non.epi.lncRNA

AL390243.1 non.epi.lncRNA

AL390254.1 non.epi.lncRNA

AL390294.1 non.epi.lncRNA

AL390719.2 non.epi.lncRNA

AL390726.2 non.epi.lncRNA

AL390726.3 non.epi.lncRNA

AL390728.5 non.epi.lncRNA

AL390728.6 non.epi.lncRNA

AL390730.1 non.epi.lncRNA

AL390730.2 non.epi.lncRNA

AL390755.1 non.epi.lncRNA

AL390760.1 non.epi.lncRNA

AL390774.2 non.epi.lncRNA

AL390778.1 non.epi.lncRNA

AL390778.2 non.epi.lncRNA

AL390783.1 non.epi.lncRNA

AL390786.1 non.epi.lncRNA

AL390816.1 non.epi.lncRNA

AL390816.2 non.epi.lncRNA

AL390835.1 non.epi.lncRNA

AL390838.1 non.epi.lncRNA

AL390856.1 non.epi.lncRNA

AL390860.1 non.epi.lncRNA

AL390866.1 non.epi.lncRNA

AL390955.2 non.epi.lncRNA

AL390961.1 non.epi.lncRNA

AL390961.2 non.epi.lncRNA

AL390962.1 non.epi.lncRNA

AL390964.1 non.epi.lncRNA

AL391001.1 non.epi.lncRNA

AL391056.1 non.epi.lncRNA

AL391069.1 non.epi.lncRNA

AL391069.3 non.epi.lncRNA

AL391095.1 non.epi.lncRNA

AL391095.2 non.epi.lncRNA

AL391095.3 non.epi.lncRNA

AL391119.1 non.epi.lncRNA

AL391152.1 non.epi.lncRNA

AL391244.1 non.epi.lncRNA

AL391244.2 non.epi.lncRNA

AL391261.2 non.epi.lncRNA

AL391261.4 non.epi.lncRNA

AL391335.1 non.epi.lncRNA

AL391336.1 non.epi.lncRNA

AL391415.1 non.epi.lncRNA

AL391421.1 non.epi.lncRNA

AL391422.2 non.epi.lncRNA

AL391422.3 non.epi.lncRNA

AL391427.1 non.epi.lncRNA

AL391497.1 non.epi.lncRNA

AL391645.1 non.epi.lncRNA

AL391684.1 non.epi.lncRNA

AL391704.1 non.epi.lncRNA

AL391704.2 non.epi.lncRNA

AL391832.1 non.epi.lncRNA

AL391832.2 non.epi.lncRNA

AL391832.3 non.epi.lncRNA

AL391834.1 non.epi.lncRNA

AL391834.2 non.epi.lncRNA

AL391839.1 non.epi.lncRNA

AL391840.1 non.epi.lncRNA

AL391845.1 non.epi.lncRNA

AL391845.2 non.epi.lncRNA

AL391863.1 non.epi.lncRNA

AL391863.2 non.epi.lncRNA

AL391869.1 non.epi.lncRNA

AL391883.1 non.epi.lncRNA

AL391987.3 non.epi.lncRNA

AL391987.4 non.epi.lncRNA

AL391988.1 non.epi.lncRNA

AL392023.1 non.epi.lncRNA

AL392023.2 non.epi.lncRNA

AL392046.1 non.epi.lncRNA

AL392048.1 non.epi.lncRNA

AL392089.1 non.epi.lncRNA

AL392172.1 non.epi.lncRNA

AL392183.1 non.epi.lncRNA

AL392185.1 non.epi.lncRNA

AL441943.1 non.epi.lncRNA

AL441943.2 non.epi.lncRNA

AL441989.1 non.epi.lncRNA

AL442067.2 non.epi.lncRNA

AL442071.1 non.epi.lncRNA

AL442125.2 non.epi.lncRNA

AL442128.2 non.epi.lncRNA

AL442163.1 non.epi.lncRNA

AL442224.1 non.epi.lncRNA

AL442638.1 non.epi.lncRNA

AL442663.3 non.epi.lncRNA

AL445070.1 non.epi.lncRNA

AL445072.1 non.epi.lncRNA

AL445074.1 non.epi.lncRNA

AL445123.1 non.epi.lncRNA

AL445183.1 non.epi.lncRNA

AL445190.1 non.epi.lncRNA

AL445193.2 non.epi.lncRNA

AL445209.1 non.epi.lncRNA

AL445218.1 non.epi.lncRNA

AL445222.1 non.epi.lncRNA

AL445223.1 non.epi.lncRNA

AL445224.1 non.epi.lncRNA

AL445228.1 non.epi.lncRNA

AL445231.1 non.epi.lncRNA

AL445235.1 non.epi.lncRNA

AL445237.1 non.epi.lncRNA

AL445250.1 non.epi.lncRNA

AL445288.1 non.epi.lncRNA

AL445307.1 non.epi.lncRNA

AL445309.1 non.epi.lncRNA

AL445363.1 non.epi.lncRNA

AL445363.2 non.epi.lncRNA

AL445423.1 non.epi.lncRNA

AL445426.1 non.epi.lncRNA

AL445430.1 non.epi.lncRNA

AL445430.2 non.epi.lncRNA

AL445433.2 non.epi.lncRNA

AL445465.1 non.epi.lncRNA

AL445465.2 non.epi.lncRNA

AL445471.1 non.epi.lncRNA

AL445471.2 non.epi.lncRNA

AL445489.1 non.epi.lncRNA

AL445493.2 non.epi.lncRNA

AL445493.3 non.epi.lncRNA

AL445584.2 non.epi.lncRNA

AL445623.1 non.epi.lncRNA

AL445644.1 non.epi.lncRNA

AL445645.1 non.epi.lncRNA

AL445649.1 non.epi.lncRNA

AL445931.1 non.epi.lncRNA

AL445933.2 non.epi.lncRNA

AL445985.1 non.epi.lncRNA

AL449043.1 non.epi.lncRNA

AL449403.1 non.epi.lncRNA

AL449403.2 non.epi.lncRNA

AL449423.1 non.epi.lncRNA

AL449983.1 non.epi.lncRNA

AL450226.1 non.epi.lncRNA

AL450263.1 non.epi.lncRNA

AL450267.1 non.epi.lncRNA

AL450270.1 non.epi.lncRNA

AL450313.1 non.epi.lncRNA

AL450327.1 non.epi.lncRNA

AL450332.1 non.epi.lncRNA

AL450344.1 non.epi.lncRNA

AL450344.2 non.epi.lncRNA

AL450345.1 non.epi.lncRNA

AL450384.1 non.epi.lncRNA

AL450384.2 non.epi.lncRNA

AL450423.1 non.epi.lncRNA

AL450442.1 non.epi.lncRNA

AL450468.1 non.epi.lncRNA

AL450469.1 non.epi.lncRNA

AL450992.1 non.epi.lncRNA

AL450998.3 non.epi.lncRNA

AL451047.1 non.epi.lncRNA

AL451048.1 non.epi.lncRNA

AL451049.1 non.epi.lncRNA

AL451050.2 non.epi.lncRNA

AL451062.1 non.epi.lncRNA

AL451064.1 non.epi.lncRNA

AL451065.1 non.epi.lncRNA

AL451067.1 non.epi.lncRNA

AL451069.3 non.epi.lncRNA

AL451070.1 non.epi.lncRNA

AL451074.5 non.epi.lncRNA

AL451081.1 non.epi.lncRNA

AL451085.1 non.epi.lncRNA

AL451085.2 non.epi.lncRNA

AL451105.2 non.epi.lncRNA

AL451127.1 non.epi.lncRNA

AL451129.1 non.epi.lncRNA

AL451137.2 non.epi.lncRNA

AL451140.1 non.epi.lncRNA

AL451164.2 non.epi.lncRNA

AL451165.2 non.epi.lncRNA

AL499616.1 non.epi.lncRNA

AL499627.2 non.epi.lncRNA

AL500522.1 non.epi.lncRNA

AL512274.1 non.epi.lncRNA

AL512283.1 non.epi.lncRNA

AL512303.1 non.epi.lncRNA

AL512306.2 non.epi.lncRNA

AL512306.3 non.epi.lncRNA

AL512310.10 non.epi.lncRNA

AL512310.11 non.epi.lncRNA

AL512310.2 non.epi.lncRNA

AL512310.9 non.epi.lncRNA

AL512328.1 non.epi.lncRNA

AL512329.2 non.epi.lncRNA

AL512330.1 non.epi.lncRNA

AL512347.1 non.epi.lncRNA

AL512356.1 non.epi.lncRNA

AL512357.1 non.epi.lncRNA

AL512363.1 non.epi.lncRNA

AL512366.1 non.epi.lncRNA

AL512380.1 non.epi.lncRNA

AL512413.1 non.epi.lncRNA

AL512422.1 non.epi.lncRNA

AL512444.1 non.epi.lncRNA

AL512506.1 non.epi.lncRNA

AL512604.3 non.epi.lncRNA

AL512622.1 non.epi.lncRNA

AL512624.2 non.epi.lncRNA

AL512625.1 non.epi.lncRNA

AL512625.2 non.epi.lncRNA

AL512631.1 non.epi.lncRNA

AL512631.2 non.epi.lncRNA

AL512634.1 non.epi.lncRNA

AL512641.1 non.epi.lncRNA

AL512649.2 non.epi.lncRNA

AL512652.1 non.epi.lncRNA

AL512658.1 non.epi.lncRNA

AL512770.1 non.epi.lncRNA

AL512785.1 non.epi.lncRNA

AL512791.1 non.epi.lncRNA

AL512791.2 non.epi.lncRNA

AL513008.1 non.epi.lncRNA

AL513122.2 non.epi.lncRNA

AL513123.1 non.epi.lncRNA

AL513128.1 non.epi.lncRNA

AL513164.1 non.epi.lncRNA

AL513185.1 non.epi.lncRNA

AL513188.1 non.epi.lncRNA

AL513190.1 non.epi.lncRNA

AL513210.1 non.epi.lncRNA

AL513217.1 non.epi.lncRNA

AL513218.1 non.epi.lncRNA

AL513285.1 non.epi.lncRNA

AL513304.1 non.epi.lncRNA

AL513314.1 non.epi.lncRNA

AL513314.2 non.epi.lncRNA

AL513318.2 non.epi.lncRNA

AL513323.1 non.epi.lncRNA

AL513324.1 non.epi.lncRNA

AL513327.2 non.epi.lncRNA

AL513327.3 non.epi.lncRNA

AL513348.1 non.epi.lncRNA

AL513412.1 non.epi.lncRNA

AL513422.1 non.epi.lncRNA

AL513477.2 non.epi.lncRNA

AL513523.3 non.epi.lncRNA

AL513523.4 non.epi.lncRNA

AL513534.2 non.epi.lncRNA

AL513542.1 non.epi.lncRNA

AL513548.1 non.epi.lncRNA

AL513548.3 non.epi.lncRNA

AL583785.1 non.epi.lncRNA

AL583803.1 non.epi.lncRNA

AL583804.1 non.epi.lncRNA

AL583805.2 non.epi.lncRNA

AL583808.1 non.epi.lncRNA

AL583810.1 non.epi.lncRNA

AL583810.2 non.epi.lncRNA

AL583810.3 non.epi.lncRNA

AL583824.1 non.epi.lncRNA

AL583839.1 non.epi.lncRNA

AL583854.1 non.epi.lncRNA

AL583856.2 non.epi.lncRNA

AL583859.1 non.epi.lncRNA

AL589642.1 non.epi.lncRNA

AL589642.2 non.epi.lncRNA

AL589678.1 non.epi.lncRNA

AL589684.1 non.epi.lncRNA

AL589739.1 non.epi.lncRNA

AL589740.1 non.epi.lncRNA

AL589743.1 non.epi.lncRNA

AL589743.4 non.epi.lncRNA

AL589745.1 non.epi.lncRNA

AL589745.2 non.epi.lncRNA

AL589765.1 non.epi.lncRNA

AL589765.2 non.epi.lncRNA

AL589765.5 non.epi.lncRNA

AL589765.6 non.epi.lncRNA

AL589765.7 non.epi.lncRNA

AL589843.1 non.epi.lncRNA

AL589863.1 non.epi.lncRNA

AL589923.1 non.epi.lncRNA

AL589935.1 non.epi.lncRNA

AL589946.1 non.epi.lncRNA

AL589986.2 non.epi.lncRNA

AL589987.2 non.epi.lncRNA

AL589990.1 non.epi.lncRNA

AL590004.3 non.epi.lncRNA

AL590006.1 non.epi.lncRNA

AL590068.2 non.epi.lncRNA

AL590093.1 non.epi.lncRNA

AL590096.1 non.epi.lncRNA

AL590133.1 non.epi.lncRNA

AL590226.1 non.epi.lncRNA

AL590226.2 non.epi.lncRNA

AL590227.1 non.epi.lncRNA

AL590235.1 non.epi.lncRNA

AL590302.1 non.epi.lncRNA

AL590302.2 non.epi.lncRNA

AL590369.1 non.epi.lncRNA

AL590378.1 non.epi.lncRNA

AL590385.1 non.epi.lncRNA

AL590385.2 non.epi.lncRNA

AL590399.1 non.epi.lncRNA

AL590399.3 non.epi.lncRNA

AL590399.5 non.epi.lncRNA

AL590408.1 non.epi.lncRNA

AL590422.1 non.epi.lncRNA

AL590432.1 non.epi.lncRNA

AL590483.2 non.epi.lncRNA

AL590490.1 non.epi.lncRNA

AL590491.2 non.epi.lncRNA

AL590502.1 non.epi.lncRNA

AL590560.3 non.epi.lncRNA

AL590609.3 non.epi.lncRNA

AL590617.2 non.epi.lncRNA

AL590648.2 non.epi.lncRNA

AL590652.1 non.epi.lncRNA

AL590666.2 non.epi.lncRNA

AL590677.1 non.epi.lncRNA

AL590705.1 non.epi.lncRNA

AL590705.2 non.epi.lncRNA

AL590705.3 non.epi.lncRNA

AL590723.1 non.epi.lncRNA

AL590727.1 non.epi.lncRNA

AL590729.1 non.epi.lncRNA

AL590730.1 non.epi.lncRNA

AL590764.1 non.epi.lncRNA

AL590783.1 non.epi.lncRNA

AL590787.1 non.epi.lncRNA

AL590822.1 non.epi.lncRNA

AL590822.2 non.epi.lncRNA

AL590867.1 non.epi.lncRNA

AL591030.1 non.epi.lncRNA

AL591043.2 non.epi.lncRNA

AL591074.1 non.epi.lncRNA

AL591167.1 non.epi.lncRNA

AL591212.1 non.epi.lncRNA

AL591222.1 non.epi.lncRNA

AL591242.1 non.epi.lncRNA

AL591368.1 non.epi.lncRNA

AL591468.1 non.epi.lncRNA

AL591501.1 non.epi.lncRNA

AL591503.1 non.epi.lncRNA

AL591504.1 non.epi.lncRNA

AL591643.1 non.epi.lncRNA

AL591684.1 non.epi.lncRNA

AL591686.1 non.epi.lncRNA

AL591686.2 non.epi.lncRNA

AL591719.2 non.epi.lncRNA

AL591721.1 non.epi.lncRNA

AL591767.1 non.epi.lncRNA

AL591770.1 non.epi.lncRNA

AL591806.1 non.epi.lncRNA

AL591846.2 non.epi.lncRNA

AL591848.3 non.epi.lncRNA

AL591848.4 non.epi.lncRNA

AL591895.1 non.epi.lncRNA

AL591896.1 non.epi.lncRNA

AL592043.1 non.epi.lncRNA

AL592078.1 non.epi.lncRNA

AL592114.3 non.epi.lncRNA

AL592146.1 non.epi.lncRNA

AL592148.3 non.epi.lncRNA

AL592161.1 non.epi.lncRNA

AL592164.1 non.epi.lncRNA

AL592166.1 non.epi.lncRNA

AL592182.1 non.epi.lncRNA

AL592182.2 non.epi.lncRNA

AL592211.1 non.epi.lncRNA

AL592301.1 non.epi.lncRNA

AL592309.2 non.epi.lncRNA

AL592402.1 non.epi.lncRNA

AL592424.1 non.epi.lncRNA

AL592429.2 non.epi.lncRNA

AL592431.1 non.epi.lncRNA

AL592431.2 non.epi.lncRNA

AL592435.1 non.epi.lncRNA

AL592435.2 non.epi.lncRNA

AL592463.1 non.epi.lncRNA

AL592464.1 non.epi.lncRNA

AL592464.2 non.epi.lncRNA

AL592466.1 non.epi.lncRNA

AL592494.1 non.epi.lncRNA

AL592528.1 non.epi.lncRNA

AL592546.2 non.epi.lncRNA

AL596087.2 non.epi.lncRNA

AL596094.1 non.epi.lncRNA

AL596188.1 non.epi.lncRNA

AL596211.1 non.epi.lncRNA

AL596218.1 non.epi.lncRNA

AL596223.1 non.epi.lncRNA

AL596223.2 non.epi.lncRNA

AL596244.1 non.epi.lncRNA

AL596325.1 non.epi.lncRNA

AL596325.2 non.epi.lncRNA

AL596330.1 non.epi.lncRNA

AL596442.1 non.epi.lncRNA

AL596442.2 non.epi.lncRNA

AL596451.1 non.epi.lncRNA

AL603832.1 non.epi.lncRNA

AL603832.2 non.epi.lncRNA

AL603840.1 non.epi.lncRNA

AL603962.1 non.epi.lncRNA

AL606468.1 non.epi.lncRNA

AL606469.1 non.epi.lncRNA

AL606489.1 non.epi.lncRNA

AL606490.3 non.epi.lncRNA

AL606491.1 non.epi.lncRNA

AL606516.1 non.epi.lncRNA

AL606519.1 non.epi.lncRNA

AL606534.1 non.epi.lncRNA

AL606534.2 non.epi.lncRNA

AL606534.3 non.epi.lncRNA

AL606537.1 non.epi.lncRNA

AL606748.1 non.epi.lncRNA

AL606760.2 non.epi.lncRNA

AL606804.1 non.epi.lncRNA

AL606807.1 non.epi.lncRNA

AL606834.1 non.epi.lncRNA

AL606834.2 non.epi.lncRNA

AL606970.2 non.epi.lncRNA

AL606970.3 non.epi.lncRNA

AL606970.4 non.epi.lncRNA

AL607028.1 non.epi.lncRNA

AL626787.1 non.epi.lncRNA

AL627309.1 non.epi.lncRNA

AL627309.2 non.epi.lncRNA

AL627309.3 non.epi.lncRNA

AL627309.4 non.epi.lncRNA

AL627309.5 non.epi.lncRNA

AL627443.1 non.epi.lncRNA

AL645465.1 non.epi.lncRNA

AL645568.1 non.epi.lncRNA

AL645608.1 non.epi.lncRNA

AL645608.2 non.epi.lncRNA

AL645608.4 non.epi.lncRNA

AL645608.5 non.epi.lncRNA

AL645634.1 non.epi.lncRNA

AL645634.2 non.epi.lncRNA

AL645728.1 non.epi.lncRNA

AL645924.1 non.epi.lncRNA

AL645929.2 non.epi.lncRNA

AL645933.2 non.epi.lncRNA

AL645937.2 non.epi.lncRNA

AL645939.4 non.epi.lncRNA

AL645940.1 non.epi.lncRNA

AL645941.1 non.epi.lncRNA

AL646090.1 non.epi.lncRNA

AL662789.1 non.epi.lncRNA

AL662791.1 non.epi.lncRNA

AL662791.2 non.epi.lncRNA

AL662844.4 non.epi.lncRNA

AL662864.1 non.epi.lncRNA

AL662884.1 non.epi.lncRNA

AL662889.1 non.epi.lncRNA

AL669831.2 non.epi.lncRNA

AL669841.1 non.epi.lncRNA

AL669970.1 non.epi.lncRNA

AL669970.2 non.epi.lncRNA

AL669970.3 non.epi.lncRNA

AL670729.1 non.epi.lncRNA

AL671710.1 non.epi.lncRNA

AL672277.1 non.epi.lncRNA

AL672310.1 non.epi.lncRNA

AL683807.1 non.epi.lncRNA

AL683807.2 non.epi.lncRNA

AL683813.1 non.epi.lncRNA

AL683887.1 non.epi.lncRNA

AL691403.1 non.epi.lncRNA

AL691403.2 non.epi.lncRNA

AL691420.1 non.epi.lncRNA

AL691426.1 non.epi.lncRNA

AL691447.2 non.epi.lncRNA

AL691482.3 non.epi.lncRNA

AL691515.1 non.epi.lncRNA

AL691515.2 non.epi.lncRNA

AL713851.1 non.epi.lncRNA

AL713851.2 non.epi.lncRNA

AL713866.1 non.epi.lncRNA

AL713923.1 non.epi.lncRNA

AL713965.1 non.epi.lncRNA

AL713998.1 non.epi.lncRNA

AL731533.1 non.epi.lncRNA

AL731537.1 non.epi.lncRNA

AL731537.2 non.epi.lncRNA

AL731557.1 non.epi.lncRNA

AL731563.3 non.epi.lncRNA

AL731566.3 non.epi.lncRNA

AL731567.1 non.epi.lncRNA

AL731568.1 non.epi.lncRNA

AL731571.1 non.epi.lncRNA

AL731575.1 non.epi.lncRNA

AL731577.1 non.epi.lncRNA

AL731577.2 non.epi.lncRNA

AL731661.1 non.epi.lncRNA

AL731684.1 non.epi.lncRNA

AL732314.4 non.epi.lncRNA

AL732323.1 non.epi.lncRNA

AL732372.1 non.epi.lncRNA

AL732437.1 non.epi.lncRNA

AL772155.1 non.epi.lncRNA

AL772202.1 non.epi.lncRNA

AL772337.2 non.epi.lncRNA

AL772363.1 non.epi.lncRNA

AL773545.1 non.epi.lncRNA

AL807752.2 non.epi.lncRNA

AL807752.3 non.epi.lncRNA

AL807752.4 non.epi.lncRNA

AL807752.5 non.epi.lncRNA

AL807757.1 non.epi.lncRNA

AL807761.3 non.epi.lncRNA

AL807761.4 non.epi.lncRNA

AL831784.1 non.epi.lncRNA

AL844175.1 non.epi.lncRNA

AL844908.1 non.epi.lncRNA

AL845311.1 non.epi.lncRNA

AL845472.1 non.epi.lncRNA

AL845552.2 non.epi.lncRNA

AL928654.2 non.epi.lncRNA

AL928921.1 non.epi.lncRNA

AL928970.1 non.epi.lncRNA

AL929288.1 non.epi.lncRNA

AL929472.2 non.epi.lncRNA

AL929472.3 non.epi.lncRNA

AL929601.1 non.epi.lncRNA

AL929601.2 non.epi.lncRNA

AL929601.3 non.epi.lncRNA

AL935212.1 non.epi.lncRNA

AL953897.1 non.epi.lncRNA

AL954642.1 non.epi.lncRNA

ALDH1L1-AS1 non.epi.lncRNA

ALDH1L1-AS2 non.epi.lncRNA

ALG13-AS1 non.epi.lncRNA

ALG1L9P non.epi.lncRNA

ALG9-IT1 non.epi.lncRNA

ALKBH3-AS1 non.epi.lncRNA

ALOX12-AS1 non.epi.lncRNA

AMMECR1-IT1 non.epi.lncRNA

ANKRD33B-AS1 non.epi.lncRNA

ANKRD34C-AS1 non.epi.lncRNA

ANKRD44-IT1 non.epi.lncRNA

ANKRD62P1-PARP4P3 non.epi.lncRNA

ANO1-AS1 non.epi.lncRNA

ANO3-AS1 non.epi.lncRNA

AOAH-IT1 non.epi.lncRNA

AP000146.1 non.epi.lncRNA

AP000221.1 non.epi.lncRNA

AP000223.1 non.epi.lncRNA

AP000229.1 non.epi.lncRNA

AP000233.1 non.epi.lncRNA

AP000233.2 non.epi.lncRNA

AP000235.1 non.epi.lncRNA

AP000238.1 non.epi.lncRNA

AP000240.1 non.epi.lncRNA

AP000251.1 non.epi.lncRNA

AP000253.1 non.epi.lncRNA

AP000254.2 non.epi.lncRNA

AP000255.1 non.epi.lncRNA

AP000265.1 non.epi.lncRNA

AP000266.1 non.epi.lncRNA

AP000281.2 non.epi.lncRNA

AP000282.1 non.epi.lncRNA

AP000290.1 non.epi.lncRNA

AP000302.1 non.epi.lncRNA

AP000317.1 non.epi.lncRNA

AP000317.2 non.epi.lncRNA

AP000322.1 non.epi.lncRNA

AP000322.2 non.epi.lncRNA

AP000345.1 non.epi.lncRNA

AP000345.2 non.epi.lncRNA

AP000346.1 non.epi.lncRNA

AP000350.6 non.epi.lncRNA

AP000350.7 non.epi.lncRNA

AP000356.1 non.epi.lncRNA

AP000365.1 non.epi.lncRNA

AP000402.1 non.epi.lncRNA

AP000424.1 non.epi.lncRNA

AP000424.2 non.epi.lncRNA

AP000426.1 non.epi.lncRNA

AP000431.1 non.epi.lncRNA

AP000431.2 non.epi.lncRNA

AP000432.1 non.epi.lncRNA

AP000438.1 non.epi.lncRNA

AP000439.1 non.epi.lncRNA

AP000439.3 non.epi.lncRNA

AP000442.1 non.epi.lncRNA

AP000446.1 non.epi.lncRNA

AP000459.1 non.epi.lncRNA

AP000459.2 non.epi.lncRNA

AP000462.1 non.epi.lncRNA

AP000462.2 non.epi.lncRNA

AP000462.3 non.epi.lncRNA

AP000470.1 non.epi.lncRNA

AP000472.1 non.epi.lncRNA

AP000474.1 non.epi.lncRNA

AP000477.1 non.epi.lncRNA

AP000477.2 non.epi.lncRNA

AP000487.1 non.epi.lncRNA

AP000487.2 non.epi.lncRNA

AP000525.1 non.epi.lncRNA

AP000527.1 non.epi.lncRNA

AP000532.2 non.epi.lncRNA

AP000534.1 non.epi.lncRNA

AP000542.2 non.epi.lncRNA

AP000542.3 non.epi.lncRNA

AP000547.2 non.epi.lncRNA

AP000550.1 non.epi.lncRNA

AP000550.2 non.epi.lncRNA

AP000552.2 non.epi.lncRNA

AP000553.1 non.epi.lncRNA

AP000553.2 non.epi.lncRNA

AP000561.1 non.epi.lncRNA

AP000593.3 non.epi.lncRNA

AP000640.1 non.epi.lncRNA

AP000640.2 non.epi.lncRNA

AP000654.1 non.epi.lncRNA

AP000662.1 non.epi.lncRNA

AP000688.1 non.epi.lncRNA

AP000688.3 non.epi.lncRNA

AP000692.1 non.epi.lncRNA

AP000692.2 non.epi.lncRNA

AP000695.1 non.epi.lncRNA

AP000695.2 non.epi.lncRNA

AP000696.1 non.epi.lncRNA

AP000697.1 non.epi.lncRNA

AP000708.1 non.epi.lncRNA

AP000721.2 non.epi.lncRNA

AP000722.1 non.epi.lncRNA

AP000753.1 non.epi.lncRNA

AP000753.2 non.epi.lncRNA

AP000755.2 non.epi.lncRNA

AP000757.1 non.epi.lncRNA

AP000757.2 non.epi.lncRNA

AP000759.1 non.epi.lncRNA

AP000763.3 non.epi.lncRNA

AP000763.4 non.epi.lncRNA

AP000766.1 non.epi.lncRNA

AP000769.2 non.epi.lncRNA

AP000777.1 non.epi.lncRNA

AP000777.3 non.epi.lncRNA

AP000781.1 non.epi.lncRNA

AP000785.1 non.epi.lncRNA

AP000787.1 non.epi.lncRNA

AP000790.1 non.epi.lncRNA

AP000793.1 non.epi.lncRNA

AP000802.1 non.epi.lncRNA

AP000808.1 non.epi.lncRNA

AP000812.1 non.epi.lncRNA

AP000821.1 non.epi.lncRNA

AP000829.1 non.epi.lncRNA

AP000842.1 non.epi.lncRNA

AP000842.2 non.epi.lncRNA

AP000844.1 non.epi.lncRNA

AP000844.2 non.epi.lncRNA

AP000851.2 non.epi.lncRNA

AP000855.1 non.epi.lncRNA

AP000857.2 non.epi.lncRNA

AP000864.1 non.epi.lncRNA

AP000866.2 non.epi.lncRNA

AP000873.3 non.epi.lncRNA

AP000873.4 non.epi.lncRNA

AP000879.1 non.epi.lncRNA

AP000880.1 non.epi.lncRNA

AP000892.1 non.epi.lncRNA

AP000892.3 non.epi.lncRNA

AP000893.2 non.epi.lncRNA

AP000894.2 non.epi.lncRNA

AP000894.3 non.epi.lncRNA

AP000897.1 non.epi.lncRNA

AP000897.2 non.epi.lncRNA

AP000907.2 non.epi.lncRNA

AP000911.1 non.epi.lncRNA

AP000915.1 non.epi.lncRNA

AP000915.2 non.epi.lncRNA

AP000919.2 non.epi.lncRNA

AP000919.4 non.epi.lncRNA

AP000936.1 non.epi.lncRNA

AP000942.2 non.epi.lncRNA

AP000943.2 non.epi.lncRNA

AP000944.1 non.epi.lncRNA

AP000959.1 non.epi.lncRNA

AP000962.1 non.epi.lncRNA

AP000997.2 non.epi.lncRNA

AP000997.3 non.epi.lncRNA

AP001007.2 non.epi.lncRNA

AP001007.3 non.epi.lncRNA

AP001010.1 non.epi.lncRNA

AP001011.1 non.epi.lncRNA

AP001020.1 non.epi.lncRNA

AP001020.2 non.epi.lncRNA

AP001020.3 non.epi.lncRNA

AP001021.1 non.epi.lncRNA

AP001021.2 non.epi.lncRNA

AP001021.3 non.epi.lncRNA

AP001025.1 non.epi.lncRNA

AP001029.1 non.epi.lncRNA

AP001033.2 non.epi.lncRNA

AP001042.1 non.epi.lncRNA

AP001042.2 non.epi.lncRNA

AP001043.1 non.epi.lncRNA

AP001046.1 non.epi.lncRNA

AP001048.1 non.epi.lncRNA

AP001056.1 non.epi.lncRNA

AP001057.1 non.epi.lncRNA

AP001059.1 non.epi.lncRNA

AP001059.2 non.epi.lncRNA

AP001059.3 non.epi.lncRNA

AP001062.1 non.epi.lncRNA

AP001062.3 non.epi.lncRNA

AP001063.1 non.epi.lncRNA

AP001065.3 non.epi.lncRNA

AP001085.1 non.epi.lncRNA

AP001092.1 non.epi.lncRNA

AP001094.1 non.epi.lncRNA

AP001094.2 non.epi.lncRNA

AP001094.3 non.epi.lncRNA

AP001099.1 non.epi.lncRNA

AP001107.1 non.epi.lncRNA

AP001107.3 non.epi.lncRNA

AP001107.4 non.epi.lncRNA

AP001107.5 non.epi.lncRNA

AP001107.6 non.epi.lncRNA

AP001107.7 non.epi.lncRNA

AP001107.8 non.epi.lncRNA

AP001107.9 non.epi.lncRNA

AP001109.1 non.epi.lncRNA

AP001116.1 non.epi.lncRNA

AP001117.1 non.epi.lncRNA

AP001120.1 non.epi.lncRNA

AP001120.2 non.epi.lncRNA

AP001122.1 non.epi.lncRNA

AP001136.1 non.epi.lncRNA

AP001160.2 non.epi.lncRNA

AP001160.4 non.epi.lncRNA

AP001172.1 non.epi.lncRNA

AP001172.2 non.epi.lncRNA

AP001178.2 non.epi.lncRNA

AP001180.1 non.epi.lncRNA

AP001180.2 non.epi.lncRNA

AP001180.4 non.epi.lncRNA

AP001189.1 non.epi.lncRNA

AP001189.3 non.epi.lncRNA

AP001189.4 non.epi.lncRNA

AP001189.5 non.epi.lncRNA

AP001198.2 non.epi.lncRNA

AP001205.1 non.epi.lncRNA

AP001207.3 non.epi.lncRNA

AP001266.1 non.epi.lncRNA

AP001267.2 non.epi.lncRNA

AP001267.4 non.epi.lncRNA

AP001269.1 non.epi.lncRNA

AP001269.2 non.epi.lncRNA

AP001269.4 non.epi.lncRNA

AP001318.1 non.epi.lncRNA

AP001330.1 non.epi.lncRNA

AP001330.4 non.epi.lncRNA

AP001341.1 non.epi.lncRNA

AP001347.1 non.epi.lncRNA

AP001350.1 non.epi.lncRNA

AP001363.2 non.epi.lncRNA

AP001372.1 non.epi.lncRNA

AP001372.3 non.epi.lncRNA

AP001381.1 non.epi.lncRNA

AP001429.1 non.epi.lncRNA

AP001432.1 non.epi.lncRNA

AP001434.1 non.epi.lncRNA

AP001437.1 non.epi.lncRNA

AP001439.1 non.epi.lncRNA

AP001442.1 non.epi.lncRNA

AP001453.2 non.epi.lncRNA

AP001458.1 non.epi.lncRNA

AP001462.1 non.epi.lncRNA

AP001464.1 non.epi.lncRNA

AP001468.1 non.epi.lncRNA

AP001469.2 non.epi.lncRNA

AP001469.3 non.epi.lncRNA

AP001471.1 non.epi.lncRNA

AP001476.1 non.epi.lncRNA

AP001476.2 non.epi.lncRNA

AP001476.3 non.epi.lncRNA

AP001486.2 non.epi.lncRNA

AP001496.1 non.epi.lncRNA

AP001496.2 non.epi.lncRNA

AP001505.1 non.epi.lncRNA

AP001527.1 non.epi.lncRNA

AP001528.1 non.epi.lncRNA

AP001542.3 non.epi.lncRNA

AP001547.1 non.epi.lncRNA

AP001554.1 non.epi.lncRNA

AP001574.1 non.epi.lncRNA

AP001595.1 non.epi.lncRNA

AP001596.1 non.epi.lncRNA

AP001596.2 non.epi.lncRNA

AP001599.1 non.epi.lncRNA

AP001604.1 non.epi.lncRNA

AP001605.1 non.epi.lncRNA

AP001610.2 non.epi.lncRNA

AP001615.1 non.epi.lncRNA

AP001619.1 non.epi.lncRNA

AP001619.2 non.epi.lncRNA

AP001625.1 non.epi.lncRNA

AP001625.2 non.epi.lncRNA

AP001625.3 non.epi.lncRNA

AP001626.1 non.epi.lncRNA

AP001627.1 non.epi.lncRNA

AP001628.1 non.epi.lncRNA

AP001628.2 non.epi.lncRNA

AP001630.1 non.epi.lncRNA

AP001631.1 non.epi.lncRNA

AP001636.3 non.epi.lncRNA

AP001652.1 non.epi.lncRNA

AP001767.3 non.epi.lncRNA

AP001775.2 non.epi.lncRNA

AP001781.1 non.epi.lncRNA

AP001793.1 non.epi.lncRNA

AP001825.1 non.epi.lncRNA

AP001830.1 non.epi.lncRNA

AP001830.2 non.epi.lncRNA

AP001831.1 non.epi.lncRNA

AP001893.3 non.epi.lncRNA

AP001922.1 non.epi.lncRNA

AP001922.2 non.epi.lncRNA

AP001922.3 non.epi.lncRNA

AP001972.2 non.epi.lncRNA

AP001972.3 non.epi.lncRNA

AP001972.4 non.epi.lncRNA

AP001978.1 non.epi.lncRNA

AP001993.1 non.epi.lncRNA

AP001999.1 non.epi.lncRNA

AP002008.1 non.epi.lncRNA

AP002008.3 non.epi.lncRNA

AP002026.1 non.epi.lncRNA

AP002075.1 non.epi.lncRNA

AP002336.1 non.epi.lncRNA

AP002336.2 non.epi.lncRNA

AP002340.1 non.epi.lncRNA

AP002358.1 non.epi.lncRNA

AP002360.1 non.epi.lncRNA

AP002370.2 non.epi.lncRNA

AP002383.2 non.epi.lncRNA

AP002387.2 non.epi.lncRNA

AP002409.1 non.epi.lncRNA

AP002414.5 non.epi.lncRNA

AP002428.1 non.epi.lncRNA

AP002433.1 non.epi.lncRNA

AP002439.1 non.epi.lncRNA

AP002490.1 non.epi.lncRNA

AP002505.1 non.epi.lncRNA

AP002518.1 non.epi.lncRNA

AP002518.2 non.epi.lncRNA

AP002748.2 non.epi.lncRNA

AP002754.1 non.epi.lncRNA

AP002754.2 non.epi.lncRNA

AP002761.1 non.epi.lncRNA

AP002761.2 non.epi.lncRNA

AP002765.1 non.epi.lncRNA

AP002768.1 non.epi.lncRNA

AP002770.1 non.epi.lncRNA

AP002784.1 non.epi.lncRNA

AP002802.1 non.epi.lncRNA

AP002807.1 non.epi.lncRNA

AP002812.2 non.epi.lncRNA

AP002812.3 non.epi.lncRNA

AP002812.5 non.epi.lncRNA

AP002815.1 non.epi.lncRNA

AP002833.1 non.epi.lncRNA

AP002840.1 non.epi.lncRNA

AP002840.2 non.epi.lncRNA

AP002856.1 non.epi.lncRNA

AP002856.2 non.epi.lncRNA

AP002856.3 non.epi.lncRNA

AP002856.4 non.epi.lncRNA

AP002884.1 non.epi.lncRNA

AP002884.3 non.epi.lncRNA

AP002892.1 non.epi.lncRNA

AP002892.2 non.epi.lncRNA

AP002893.1 non.epi.lncRNA

AP002957.1 non.epi.lncRNA

AP002989.1 non.epi.lncRNA

AP002992.1 non.epi.lncRNA

AP002993.1 non.epi.lncRNA

AP003025.1 non.epi.lncRNA

AP003027.1 non.epi.lncRNA

AP003031.1 non.epi.lncRNA

AP003032.1 non.epi.lncRNA

AP003032.2 non.epi.lncRNA

AP003037.1 non.epi.lncRNA

AP003043.1 non.epi.lncRNA

AP003049.2 non.epi.lncRNA

AP003065.1 non.epi.lncRNA

AP003066.1 non.epi.lncRNA

AP003068.3 non.epi.lncRNA

AP003071.1 non.epi.lncRNA

AP003071.2 non.epi.lncRNA

AP003071.3 non.epi.lncRNA

AP003086.1 non.epi.lncRNA

AP003086.2 non.epi.lncRNA

AP003100.1 non.epi.lncRNA

AP003108.1 non.epi.lncRNA

AP003110.1 non.epi.lncRNA

AP003115.1 non.epi.lncRNA

AP003117.1 non.epi.lncRNA

AP003117.2 non.epi.lncRNA

AP003119.1 non.epi.lncRNA

AP003119.3 non.epi.lncRNA

AP003121.1 non.epi.lncRNA

AP003122.2 non.epi.lncRNA

AP003123.1 non.epi.lncRNA

AP003128.1 non.epi.lncRNA

AP003168.2 non.epi.lncRNA

AP003170.3 non.epi.lncRNA

AP003170.4 non.epi.lncRNA

AP003171.1 non.epi.lncRNA

AP003174.2 non.epi.lncRNA

AP003306.1 non.epi.lncRNA

AP003306.2 non.epi.lncRNA

AP003351.1 non.epi.lncRNA

AP003354.1 non.epi.lncRNA

AP003355.2 non.epi.lncRNA

AP003385.3 non.epi.lncRNA

AP003390.1 non.epi.lncRNA

AP003392.1 non.epi.lncRNA

AP003392.3 non.epi.lncRNA

AP003392.4 non.epi.lncRNA

AP003392.5 non.epi.lncRNA

AP003393.1 non.epi.lncRNA

AP003396.3 non.epi.lncRNA

AP003396.5 non.epi.lncRNA

AP003400.1 non.epi.lncRNA

AP003419.2 non.epi.lncRNA

AP003465.2 non.epi.lncRNA

AP003467.1 non.epi.lncRNA

AP003469.1 non.epi.lncRNA

AP003469.3 non.epi.lncRNA

AP003469.4 non.epi.lncRNA

AP003471.1 non.epi.lncRNA

AP003472.1 non.epi.lncRNA

AP003486.1 non.epi.lncRNA

AP003498.1 non.epi.lncRNA

AP003500.1 non.epi.lncRNA

AP003501.1 non.epi.lncRNA

AP003501.2 non.epi.lncRNA

AP003548.1 non.epi.lncRNA

AP003555.1 non.epi.lncRNA

AP003555.2 non.epi.lncRNA

AP003557.1 non.epi.lncRNA

AP003559.1 non.epi.lncRNA

AP003692.1 non.epi.lncRNA

AP003715.1 non.epi.lncRNA

AP003718.1 non.epi.lncRNA

AP003721.1 non.epi.lncRNA

AP003721.2 non.epi.lncRNA

AP003721.3 non.epi.lncRNA

AP003774.1 non.epi.lncRNA

AP003785.1 non.epi.lncRNA

AP003900.1 non.epi.lncRNA

AP003969.1 non.epi.lncRNA

AP003973.2 non.epi.lncRNA

AP004147.1 non.epi.lncRNA

AP004247.2 non.epi.lncRNA

AP004550.1 non.epi.lncRNA

AP004608.1 non.epi.lncRNA

AP004609.1 non.epi.lncRNA

AP005057.1 non.epi.lncRNA

AP005059.1 non.epi.lncRNA

AP005059.2 non.epi.lncRNA

AP005062.1 non.epi.lncRNA

AP005120.1 non.epi.lncRNA

AP005121.1 non.epi.lncRNA

AP005121.2 non.epi.lncRNA

AP005131.4 non.epi.lncRNA

AP005131.5 non.epi.lncRNA

AP005131.6 non.epi.lncRNA

AP005131.7 non.epi.lncRNA

AP005136.3 non.epi.lncRNA

AP005137.1 non.epi.lncRNA

AP005202.1 non.epi.lncRNA

AP005202.2 non.epi.lncRNA

AP005203.1 non.epi.lncRNA

AP005205.1 non.epi.lncRNA

AP005205.2 non.epi.lncRNA

AP005209.1 non.epi.lncRNA

AP005210.1 non.epi.lncRNA

AP005229.1 non.epi.lncRNA

AP005229.2 non.epi.lncRNA

AP005230.1 non.epi.lncRNA

AP005233.1 non.epi.lncRNA

AP005233.2 non.epi.lncRNA

AP005242.1 non.epi.lncRNA

AP005242.3 non.epi.lncRNA

AP005262.2 non.epi.lncRNA

AP005263.1 non.epi.lncRNA

AP005264.3 non.epi.lncRNA

AP005264.4 non.epi.lncRNA

AP005271.1 non.epi.lncRNA

AP005273.1 non.epi.lncRNA

AP005328.1 non.epi.lncRNA

AP005329.1 non.epi.lncRNA

AP005329.2 non.epi.lncRNA

AP005329.3 non.epi.lncRNA

AP005357.1 non.epi.lncRNA

AP005380.1 non.epi.lncRNA

AP005432.1 non.epi.lncRNA

AP005432.2 non.epi.lncRNA

AP005436.1 non.epi.lncRNA

AP005436.2 non.epi.lncRNA

AP005436.3 non.epi.lncRNA

AP005482.3 non.epi.lncRNA

AP005530.1 non.epi.lncRNA

AP005671.1 non.epi.lncRNA

AP005717.1 non.epi.lncRNA

AP005901.2 non.epi.lncRNA

AP006216.1 non.epi.lncRNA

AP006216.2 non.epi.lncRNA

AP006248.4 non.epi.lncRNA

AP006259.1 non.epi.lncRNA

AP006285.1 non.epi.lncRNA

AP006295.1 non.epi.lncRNA

AP006333.1 non.epi.lncRNA

AP006333.2 non.epi.lncRNA

AP006437.1 non.epi.lncRNA

AP006545.1 non.epi.lncRNA

AP006545.2 non.epi.lncRNA

AP006547.1 non.epi.lncRNA

AP006565.1 non.epi.lncRNA

AP006621.2 non.epi.lncRNA

AP006621.3 non.epi.lncRNA

AP006621.4 non.epi.lncRNA

AP006748.1 non.epi.lncRNA

AP007216.1 non.epi.lncRNA

AP007216.2 non.epi.lncRNA

AP4B1-AS1 non.epi.lncRNA

APCDD1L-DT non.epi.lncRNA

APOA1-AS non.epi.lncRNA

APOBEC3B-AS1 non.epi.lncRNA

APTR non.epi.lncRNA

AQP4-AS1 non.epi.lncRNA

ARAP1-AS1 non.epi.lncRNA

ARAP1-AS2 non.epi.lncRNA

ARHGAP22-IT1 non.epi.lncRNA

ARHGAP26-IT1 non.epi.lncRNA

ARHGAP27P1-BPTFP1-KPNA2P3 non.epi.lncRNA

ARHGAP29-AS1 non.epi.lncRNA

ARHGAP31-AS1 non.epi.lncRNA

ARHGAP5-AS1 non.epi.lncRNA

ARHGEF19-AS1 non.epi.lncRNA

ARHGEF26-AS1 non.epi.lncRNA

ARHGEF3-AS1 non.epi.lncRNA

ARHGEF38-IT1 non.epi.lncRNA

ARHGEF7-AS1 non.epi.lncRNA

ARHGEF7-IT1 non.epi.lncRNA

ARHGEF9-IT1 non.epi.lncRNA

ARLNC1 non.epi.lncRNA

ARMC2-AS1 non.epi.lncRNA

ARMCX3-AS1 non.epi.lncRNA

ARMCX5-GPRASP2 non.epi.lncRNA

ARPP21-AS1 non.epi.lncRNA

ARRDC3-AS1 non.epi.lncRNA

ARSD-AS1 non.epi.lncRNA

ASB16-AS1 non.epi.lncRNA

ASH1L-IT1 non.epi.lncRNA

ASMTL-AS1 non.epi.lncRNA

ASTN2-AS1 non.epi.lncRNA

ATG10-AS1 non.epi.lncRNA

ATG10-IT1 non.epi.lncRNA

ATP11AUN non.epi.lncRNA

ATP13A4-AS1 non.epi.lncRNA

ATP13A5-AS1 non.epi.lncRNA

ATP1A1-AS1 non.epi.lncRNA

ATP1B3-AS1 non.epi.lncRNA

ATP2B2-IT1 non.epi.lncRNA

ATP2B2-IT2 non.epi.lncRNA

ATP6V1B1-AS1 non.epi.lncRNA

ATXN8OS non.epi.lncRNA

AZIN1-AS1 non.epi.lncRNA

B3GALT5-AS1 non.epi.lncRNA

B4GALT1-AS1 non.epi.lncRNA

B4GALT4-AS1 non.epi.lncRNA

BAALC-AS1 non.epi.lncRNA

BAALC-AS2 non.epi.lncRNA

BACE1-AS non.epi.lncRNA

BACE2-IT1 non.epi.lncRNA

BACH1-AS1 non.epi.lncRNA

BACH1-IT1 non.epi.lncRNA

BACH1-IT2 non.epi.lncRNA

BACH1-IT3 non.epi.lncRNA

BANCR non.epi.lncRNA

BARX1-DT non.epi.lncRNA

BASP1-AS1 non.epi.lncRNA

BBOX1-AS1 non.epi.lncRNA

BCAR4 non.epi.lncRNA

BCRP3 non.epi.lncRNA

BDNF-AS non.epi.lncRNA

BEAN1-AS1 non.epi.lncRNA

BGLT3 non.epi.lncRNA

BHLHE40-AS1 non.epi.lncRNA

BIRC6-AS1 non.epi.lncRNA

BLACE non.epi.lncRNA

BMP7-AS1 non.epi.lncRNA

BMS1P14 non.epi.lncRNA

BPESC1 non.epi.lncRNA

BRWD1-AS2 non.epi.lncRNA

BRWD1-IT1 non.epi.lncRNA

BSN-AS1 non.epi.lncRNA

BSN-DT non.epi.lncRNA

BTBD9-AS1 non.epi.lncRNA

BTG3-AS1 non.epi.lncRNA

BVES-AS1 non.epi.lncRNA

BX005019.1 non.epi.lncRNA

BX005040.1 non.epi.lncRNA

BX005040.3 non.epi.lncRNA

BX005214.1 non.epi.lncRNA

BX005214.2 non.epi.lncRNA

BX005266.2 non.epi.lncRNA

BX072579.1 non.epi.lncRNA

BX072579.2 non.epi.lncRNA

BX088651.2 non.epi.lncRNA

BX088651.4 non.epi.lncRNA

BX119904.2 non.epi.lncRNA

BX255923.1 non.epi.lncRNA

BX255923.2 non.epi.lncRNA

BX255925.1 non.epi.lncRNA

BX284613.2 non.epi.lncRNA

BX284656.1 non.epi.lncRNA

BX284656.2 non.epi.lncRNA

BX284668.1 non.epi.lncRNA

BX284668.5 non.epi.lncRNA

BX284668.6 non.epi.lncRNA

BX322234.1 non.epi.lncRNA

BX322234.2 non.epi.lncRNA

BX322557.1 non.epi.lncRNA

BX322559.1 non.epi.lncRNA

BX323046.1 non.epi.lncRNA

BX324167.1 non.epi.lncRNA

BX324167.2 non.epi.lncRNA

BX470209.1 non.epi.lncRNA

BX470209.2 non.epi.lncRNA

BX537318.1 non.epi.lncRNA

BX539320.1 non.epi.lncRNA

BX546450.1 non.epi.lncRNA

BX546450.2 non.epi.lncRNA

BX571846.1 non.epi.lncRNA

BX640514.1 non.epi.lncRNA

BX640515.1 non.epi.lncRNA

BX649601.1 non.epi.lncRNA

BX649632.1 non.epi.lncRNA

BX664718.1 non.epi.lncRNA

BX664730.1 non.epi.lncRNA

BX927359.1 non.epi.lncRNA

C10orf25 non.epi.lncRNA

C10orf71-AS1 non.epi.lncRNA

C11orf40 non.epi.lncRNA

C11orf44 non.epi.lncRNA

C12orf77 non.epi.lncRNA

C12orf80 non.epi.lncRNA

C15orf32 non.epi.lncRNA

C15orf54 non.epi.lncRNA

C15orf56 non.epi.lncRNA

C16orf97 non.epi.lncRNA

C17orf102 non.epi.lncRNA

C17orf112 non.epi.lncRNA

C17orf77 non.epi.lncRNA

C1orf137 non.epi.lncRNA

C1orf147 non.epi.lncRNA

C1orf195 non.epi.lncRNA

C1orf220 non.epi.lncRNA

C1QTNF1-AS1 non.epi.lncRNA

C1QTNF9-AS1 non.epi.lncRNA

C20orf187 non.epi.lncRNA

C20orf197 non.epi.lncRNA

C20orf78 non.epi.lncRNA

C21orf62-AS1 non.epi.lncRNA

C21orf91-OT1 non.epi.lncRNA

C22orf34 non.epi.lncRNA

C2CD4D-AS1 non.epi.lncRNA

C2orf27A non.epi.lncRNA

C3orf35 non.epi.lncRNA

C3orf67-AS1 non.epi.lncRNA

C3orf79 non.epi.lncRNA

C4A-AS1 non.epi.lncRNA

C4B-AS1 non.epi.lncRNA

C5orf17 non.epi.lncRNA

C5orf56 non.epi.lncRNA

C5orf60 non.epi.lncRNA

C5orf64 non.epi.lncRNA

C5orf64-AS1 non.epi.lncRNA

C5orf66 non.epi.lncRNA

C5orf66-AS1 non.epi.lncRNA

C5orf66-AS2 non.epi.lncRNA

C6orf47-AS1 non.epi.lncRNA

C7orf65 non.epi.lncRNA

C7orf66 non.epi.lncRNA

C8orf31 non.epi.lncRNA

C8orf34-AS1 non.epi.lncRNA

C8orf37-AS1 non.epi.lncRNA

C8orf87 non.epi.lncRNA

C9orf106 non.epi.lncRNA

C9orf139 non.epi.lncRNA

C9orf147 non.epi.lncRNA

C9orf163 non.epi.lncRNA

C9orf170 non.epi.lncRNA

C9orf62 non.epi.lncRNA

CA3-AS1 non.epi.lncRNA

CACNA1C-AS1 non.epi.lncRNA

CACNA1C-AS2 non.epi.lncRNA

CACNA1C-AS3 non.epi.lncRNA

CACNA1C-AS4 non.epi.lncRNA

CACNA1C-IT1 non.epi.lncRNA

CACNA1C-IT3 non.epi.lncRNA

CACNA1G-AS1 non.epi.lncRNA

CACNA2D3-AS1 non.epi.lncRNA

CACTIN-AS1 non.epi.lncRNA

CADM2-AS1 non.epi.lncRNA

CADM2-AS2 non.epi.lncRNA

CADM3-AS1 non.epi.lncRNA

CALML3-AS1 non.epi.lncRNA

CAMTA1-IT1 non.epi.lncRNA

CAPN10-DT non.epi.lncRNA

CARD8-AS1 non.epi.lncRNA

CARMN non.epi.lncRNA

CASC11 non.epi.lncRNA

CASC15 non.epi.lncRNA

CASC16 non.epi.lncRNA

CASC17 non.epi.lncRNA

CASC19 non.epi.lncRNA

CASC2 non.epi.lncRNA

CASC20 non.epi.lncRNA

CASC22 non.epi.lncRNA

CASC23 non.epi.lncRNA

CASC6 non.epi.lncRNA

CASC8 non.epi.lncRNA

CASC9 non.epi.lncRNA

CASK-AS1 non.epi.lncRNA

CATIP-AS1 non.epi.lncRNA

CATIP-AS2 non.epi.lncRNA

CBR3-AS1 non.epi.lncRNA

CCAT2 non.epi.lncRNA

CCDC13-AS1 non.epi.lncRNA

CCDC144NL-AS1 non.epi.lncRNA

CCDC18-AS1 non.epi.lncRNA

CCDC26 non.epi.lncRNA

CCDC39 non.epi.lncRNA

CCND2-AS1 non.epi.lncRNA

CCNT2-AS1 non.epi.lncRNA

CCR5AS non.epi.lncRNA

CD200R1L-AS1 non.epi.lncRNA

CD81-AS1 non.epi.lncRNA

CDC37L1-DT non.epi.lncRNA

CDC42-IT1 non.epi.lncRNA

CDH18-AS1 non.epi.lncRNA

CDH23-AS1 non.epi.lncRNA

CDKN2A-DT non.epi.lncRNA

CDKN2B-AS1 non.epi.lncRNA

CDRT7 non.epi.lncRNA

CDRT8 non.epi.lncRNA

CEBPA-DT non.epi.lncRNA

CECR3 non.epi.lncRNA

CELF2-AS1 non.epi.lncRNA

CELF2-AS2 non.epi.lncRNA

CELF2-DT non.epi.lncRNA

CERNA1 non.epi.lncRNA

CERS3-AS1 non.epi.lncRNA

CERS6-AS1 non.epi.lncRNA

CFAP44-AS1 non.epi.lncRNA

CFLAR-AS1 non.epi.lncRNA

CFTR-AS1 non.epi.lncRNA

CHKB-DT non.epi.lncRNA

CHL1-AS1 non.epi.lncRNA

CHL1-AS2 non.epi.lncRNA

CHODL-AS1 non.epi.lncRNA

CHRM3-AS1 non.epi.lncRNA

CHRM3-AS2 non.epi.lncRNA

CIRBP-AS1 non.epi.lncRNA

CISTR non.epi.lncRNA

CKMT2-AS1 non.epi.lncRNA

CLCA4-AS1 non.epi.lncRNA

CLDN10-AS1 non.epi.lncRNA

CLEC12A-AS1 non.epi.lncRNA

CLLU1 non.epi.lncRNA

CLMAT3 non.epi.lncRNA

CLRN1-AS1 non.epi.lncRNA

CLYBL-AS1 non.epi.lncRNA

CLYBL-AS2 non.epi.lncRNA

CNNM3-DT non.epi.lncRNA

CNOT10-AS1 non.epi.lncRNA

CNTFR-AS1 non.epi.lncRNA

CNTN4-AS1 non.epi.lncRNA

CNTN4-AS2 non.epi.lncRNA

COA6-AS1 non.epi.lncRNA

COL4A2-AS1 non.epi.lncRNA

COL4A2-AS2 non.epi.lncRNA

COL5A1-AS1 non.epi.lncRNA

COLCA1 non.epi.lncRNA

COPDA1 non.epi.lncRNA

COX10-AS1 non.epi.lncRNA

CPB2-AS1 non.epi.lncRNA

CPEB1-AS1 non.epi.lncRNA

CPEB2-DT non.epi.lncRNA

CPNE8-AS1 non.epi.lncRNA

CPS1-IT1 non.epi.lncRNA

CR381653.1 non.epi.lncRNA

CR382287.2 non.epi.lncRNA

CR383656.10 non.epi.lncRNA

CR383656.12 non.epi.lncRNA

CR392039.4 non.epi.lncRNA

CR559946.1 non.epi.lncRNA

CR559946.2 non.epi.lncRNA

CR786580.1 non.epi.lncRNA

CR936218.1 non.epi.lncRNA

CR936218.2 non.epi.lncRNA

CRNDE non.epi.lncRNA

CRPPA-AS1 non.epi.lncRNA

CRTC3-AS1 non.epi.lncRNA

CRYZL2P-SEC16B non.epi.lncRNA

CSE1L-AS1 non.epi.lncRNA

CSNK1G2-AS1 non.epi.lncRNA

CSPG4P1Y non.epi.lncRNA

CSPG4P2Y non.epi.lncRNA

CSRP3-AS1 non.epi.lncRNA

CSTF3-DT non.epi.lncRNA

CT66 non.epi.lncRNA

CT69 non.epi.lncRNA

CT70 non.epi.lncRNA

CTBP1-AS non.epi.lncRNA

CTBP1-DT non.epi.lncRNA

CU104787.1 non.epi.lncRNA

CU633904.1 non.epi.lncRNA

CU633906.1 non.epi.lncRNA

CU633906.2 non.epi.lncRNA

CU633967.1 non.epi.lncRNA

CU634019.1 non.epi.lncRNA

CU634019.2 non.epi.lncRNA

CU634019.5 non.epi.lncRNA

CU638689.1 non.epi.lncRNA

CU638689.4 non.epi.lncRNA

CU638689.5 non.epi.lncRNA

CU639417.4 non.epi.lncRNA

CU639417.5 non.epi.lncRNA

CXXC4-AS1 non.epi.lncRNA

CXXC5-AS1 non.epi.lncRNA

CYB561D2 non.epi.lncRNA

CYMP-AS1 non.epi.lncRNA

CYP1B1-AS1 non.epi.lncRNA

CYP4A22-AS1 non.epi.lncRNA

CYTOR non.epi.lncRNA

CYYR1-AS1 non.epi.lncRNA

DAB1-AS1 non.epi.lncRNA

DACT3-AS1 non.epi.lncRNA

DANT1 non.epi.lncRNA

DANT2 non.epi.lncRNA

DAOA-AS1 non.epi.lncRNA

DAPK1-IT1 non.epi.lncRNA

DBET non.epi.lncRNA

DBH-AS1 non.epi.lncRNA

DCST1-AS1 non.epi.lncRNA

DCUN1D2-AS non.epi.lncRNA

DCXR-DT non.epi.lncRNA

DDC-AS1 non.epi.lncRNA

DDIT4-AS1 non.epi.lncRNA

DDN-AS1 non.epi.lncRNA

DDR1-DT non.epi.lncRNA

DDX39B-AS1 non.epi.lncRNA

1-Dec non.epi.lncRNA

DENND5B-AS1 non.epi.lncRNA

DENND6A-AS1 non.epi.lncRNA

DENND6A-DT non.epi.lncRNA

DEPDC1-AS1 non.epi.lncRNA

DGCR10 non.epi.lncRNA

DGCR11 non.epi.lncRNA

DGCR9 non.epi.lncRNA

DGUOK-AS1 non.epi.lncRNA

DHCR24-DT non.epi.lncRNA

DHRS4-AS1 non.epi.lncRNA

DHRSX-IT1 non.epi.lncRNA

DHX33-DT non.epi.lncRNA

DIAPH2-AS1 non.epi.lncRNA

DIAPH3-AS1 non.epi.lncRNA

DIAPH3-AS2 non.epi.lncRNA

DICER1-AS1 non.epi.lncRNA

DIO2-AS1 non.epi.lncRNA

DIRC3 non.epi.lncRNA

DIRC3-AS1 non.epi.lncRNA

DISC1-IT1 non.epi.lncRNA

DISC1FP1 non.epi.lncRNA

DLEU1 non.epi.lncRNA

DLEU2 non.epi.lncRNA

DLEU2L non.epi.lncRNA

DLG1-AS1 non.epi.lncRNA

DLG3-AS1 non.epi.lncRNA

DLGAP1-AS3 non.epi.lncRNA

DLGAP1-AS4 non.epi.lncRNA

DLGAP1-AS5 non.epi.lncRNA

DLGAP2-AS1 non.epi.lncRNA

DLGAP4-AS1 non.epi.lncRNA

DLX2-DT non.epi.lncRNA

DLX6-AS1 non.epi.lncRNA

DM1-AS non.epi.lncRNA

DMD-AS3 non.epi.lncRNA

DNAAF4-CCPG1 non.epi.lncRNA

DNAJB8-AS1 non.epi.lncRNA

DNAJC27-AS1 non.epi.lncRNA

DNAJC3-DT non.epi.lncRNA

DNM3-IT1 non.epi.lncRNA

DNMBP-AS1 non.epi.lncRNA

DOCK4-AS1 non.epi.lncRNA

DOCK8-AS1 non.epi.lncRNA

DOCK9-AS1 non.epi.lncRNA

DPH6-DT non.epi.lncRNA

DPP10-AS1 non.epi.lncRNA

DPP10-AS2 non.epi.lncRNA

DPP10-AS3 non.epi.lncRNA

DPP9-AS1 non.epi.lncRNA

DPYD-AS1 non.epi.lncRNA

DPYD-AS2 non.epi.lncRNA

DPYD-IT1 non.epi.lncRNA

DRAIC non.epi.lncRNA

DSCAM-AS1 non.epi.lncRNA

DSCAM-IT1 non.epi.lncRNA

DSCAS non.epi.lncRNA

DSCR10 non.epi.lncRNA

DSCR4 non.epi.lncRNA

DSCR4-IT1 non.epi.lncRNA

DSCR8 non.epi.lncRNA

DSCR9 non.epi.lncRNA

DSG1-AS1 non.epi.lncRNA

DSG2-AS1 non.epi.lncRNA

E2F3-IT1 non.epi.lncRNA

EAF1-AS1 non.epi.lncRNA

EDNRB-AS1 non.epi.lncRNA

EDRF1-AS1 non.epi.lncRNA

EFCAB14-AS1 non.epi.lncRNA

EFCAB6-AS1 non.epi.lncRNA

EGFLAM-AS1 non.epi.lncRNA

EGFLAM-AS2 non.epi.lncRNA

EGFLAM-AS3 non.epi.lncRNA

EGFLAM-AS4 non.epi.lncRNA

EGFR-AS1 non.epi.lncRNA

EGOT non.epi.lncRNA

EHHADH-AS1 non.epi.lncRNA

EHMT2-AS1 non.epi.lncRNA

EIF1AX-AS1 non.epi.lncRNA

EIF1B-AS1 non.epi.lncRNA

EIF2AK3-DT non.epi.lncRNA

EIF3J-DT non.epi.lncRNA

EIPR1-IT1 non.epi.lncRNA

ELDR non.epi.lncRNA

ELF3-AS1 non.epi.lncRNA

ELMO1-AS1 non.epi.lncRNA

ELN-AS1 non.epi.lncRNA

ELOA-AS1 non.epi.lncRNA

ELOVL2-AS1 non.epi.lncRNA

EMC1-AS1 non.epi.lncRNA

EML2-AS1 non.epi.lncRNA

EMX2OS non.epi.lncRNA

ENO1-AS1 non.epi.lncRNA

ENOX1-AS1 non.epi.lncRNA

ENOX1-AS2 non.epi.lncRNA

ENTPD1-AS1 non.epi.lncRNA

ENTPD3-AS1 non.epi.lncRNA

EP300-AS1 non.epi.lncRNA

EPB41L4A-DT non.epi.lncRNA

EPCAM-DT non.epi.lncRNA

EPHA1-AS1 non.epi.lncRNA

EPHA5-AS1 non.epi.lncRNA

EPN2-AS1 non.epi.lncRNA

ERC2-IT1 non.epi.lncRNA

ERI3-IT1 non.epi.lncRNA

ERICH3-AS1 non.epi.lncRNA

ERLNC1 non.epi.lncRNA

ERVE-1 non.epi.lncRNA

ERVH-1 non.epi.lncRNA

ERVK-28 non.epi.lncRNA

ERVK13-1 non.epi.lncRNA

ERVMER61-1 non.epi.lncRNA

ESRG non.epi.lncRNA

ETS1-AS1 non.epi.lncRNA

EWSAT1 non.epi.lncRNA

EXOSC10-AS1 non.epi.lncRNA

EXTL3-AS1 non.epi.lncRNA

F10-AS1 non.epi.lncRNA

F11-AS1 non.epi.lncRNA

FALEC non.epi.lncRNA

FAM106A non.epi.lncRNA

FAM138A non.epi.lncRNA

FAM138B non.epi.lncRNA

FAM138C non.epi.lncRNA

FAM138D non.epi.lncRNA

FAM138E non.epi.lncRNA

FAM13A-AS1 non.epi.lncRNA

FAM153CP non.epi.lncRNA

FAM155A-IT1 non.epi.lncRNA

FAM157C non.epi.lncRNA

FAM160A1-DT non.epi.lncRNA

FAM167A-AS1 non.epi.lncRNA

FAM170B-AS1 non.epi.lncRNA

FAM181A-AS1 non.epi.lncRNA

FAM182A non.epi.lncRNA

FAM182B non.epi.lncRNA

FAM197Y6 non.epi.lncRNA

FAM197Y7 non.epi.lncRNA

FAM198B-AS1 non.epi.lncRNA

FAM215A non.epi.lncRNA

FAM215B non.epi.lncRNA

FAM222A-AS1 non.epi.lncRNA

FAM223A non.epi.lncRNA

FAM223B non.epi.lncRNA

FAM224A non.epi.lncRNA

FAM224B non.epi.lncRNA

FAM225A non.epi.lncRNA

FAM225B non.epi.lncRNA

FAM226B non.epi.lncRNA

FAM230A non.epi.lncRNA

FAM230B non.epi.lncRNA

FAM230C non.epi.lncRNA

FAM230D non.epi.lncRNA

FAM230E non.epi.lncRNA

FAM230F non.epi.lncRNA

FAM230G non.epi.lncRNA

FAM230H non.epi.lncRNA

FAM230I non.epi.lncRNA

FAM230J non.epi.lncRNA

FAM242A non.epi.lncRNA

FAM242C non.epi.lncRNA

FAM242D non.epi.lncRNA

FAM242E non.epi.lncRNA

FAM242F non.epi.lncRNA

FAM27C non.epi.lncRNA

FAM27E3 non.epi.lncRNA

FAM27E5 non.epi.lncRNA

FAM30A non.epi.lncRNA

FAM3D-AS1 non.epi.lncRNA

FAM41AY1 non.epi.lncRNA

FAM41AY2 non.epi.lncRNA

FAM53B-AS1 non.epi.lncRNA

FAM66A non.epi.lncRNA

FAM66B non.epi.lncRNA

FAM66C non.epi.lncRNA

FAM66D non.epi.lncRNA

FAM66E non.epi.lncRNA

FAM74A1 non.epi.lncRNA

FAM74A7 non.epi.lncRNA

FAM83A-AS1 non.epi.lncRNA

FAM85B non.epi.lncRNA

FAM87A non.epi.lncRNA

FAM99A non.epi.lncRNA

FAM99B non.epi.lncRNA

FANK1-AS1 non.epi.lncRNA

FAR1-IT1 non.epi.lncRNA

FARP1-AS1 non.epi.lncRNA

FARSA-AS1 non.epi.lncRNA

FBXL19-AS1 non.epi.lncRNA

FBXO36-IT1 non.epi.lncRNA

FBXW7-AS1 non.epi.lncRNA

FENDRR non.epi.lncRNA

FER1L6-AS1 non.epi.lncRNA

FER1L6-AS2 non.epi.lncRNA

FEZF1-AS1 non.epi.lncRNA

FGD5-AS1 non.epi.lncRNA

FGF10-AS1 non.epi.lncRNA

FGF12-AS1 non.epi.lncRNA

FGF12-AS2 non.epi.lncRNA

FGF13-AS1 non.epi.lncRNA

FGF14-AS1 non.epi.lncRNA

FGF14-AS2 non.epi.lncRNA

FGF14-IT1 non.epi.lncRNA

FIGNL2-DT non.epi.lncRNA

FILNC1 non.epi.lncRNA

FIRRE non.epi.lncRNA

FLG-AS1 non.epi.lncRNA

FLJ45513 non.epi.lncRNA

FLNB-AS1 non.epi.lncRNA

FLVCR1-DT non.epi.lncRNA

FMR1-IT1 non.epi.lncRNA

FNDC1-IT1 non.epi.lncRNA

FO393401.1 non.epi.lncRNA

FO393408.1 non.epi.lncRNA

FO393415.1 non.epi.lncRNA

FO393415.3 non.epi.lncRNA

FO393419.2 non.epi.lncRNA

FO680682.1 non.epi.lncRNA

FO704657.1 non.epi.lncRNA

FOCAD-AS1 non.epi.lncRNA

FOXC2-AS1 non.epi.lncRNA

FOXD1-AS1 non.epi.lncRNA

FOXD2-AS1 non.epi.lncRNA

FOXD3-AS1 non.epi.lncRNA

FOXG1-AS1 non.epi.lncRNA

FOXN3-AS1 non.epi.lncRNA

FOXN3-AS2 non.epi.lncRNA

FOXP1-AS1 non.epi.lncRNA

FOXP1-IT1 non.epi.lncRNA

FOXP4-AS1 non.epi.lncRNA

FP236240.1 non.epi.lncRNA

FP236241.1 non.epi.lncRNA

FP236315.1 non.epi.lncRNA

FP236315.3 non.epi.lncRNA

FP236383.3 non.epi.lncRNA

FP236383.4 non.epi.lncRNA

FP236383.5 non.epi.lncRNA

FP325318.1 non.epi.lncRNA

FP325330.1 non.epi.lncRNA

FP325330.2 non.epi.lncRNA

FP325330.3 non.epi.lncRNA

FP325332.1 non.epi.lncRNA

FP325335.1 non.epi.lncRNA

FP475955.1 non.epi.lncRNA

FP475955.2 non.epi.lncRNA

FP475955.3 non.epi.lncRNA

FP565260.5 non.epi.lncRNA

FP671120.4 non.epi.lncRNA

FP671120.6 non.epi.lncRNA

FP671120.7 non.epi.lncRNA

FP700111.1 non.epi.lncRNA

FREM2-AS1 non.epi.lncRNA

FRG1-DT non.epi.lncRNA

FRGCA non.epi.lncRNA

FRMD6-AS1 non.epi.lncRNA

FRMD6-AS2 non.epi.lncRNA

FRMPD3-AS1 non.epi.lncRNA

FRMPD4-AS1 non.epi.lncRNA

FRY-AS1 non.epi.lncRNA

FSIP2-AS1 non.epi.lncRNA

FSIP2-AS2 non.epi.lncRNA

FTCD-AS1 non.epi.lncRNA

FTX non.epi.lncRNA

FUT8-AS1 non.epi.lncRNA

FZD10-AS1 non.epi.lncRNA

FZD4-DT non.epi.lncRNA

G2E3-AS1 non.epi.lncRNA

GABRG3-AS1 non.epi.lncRNA

GACAT1 non.epi.lncRNA

GACAT3 non.epi.lncRNA

GARS-DT non.epi.lncRNA

GAS1RR non.epi.lncRNA

GAS5 non.epi.lncRNA

GAS5-AS1 non.epi.lncRNA

GAS6-AS1 non.epi.lncRNA

GAS8-AS1 non.epi.lncRNA

GATA2-AS1 non.epi.lncRNA

GATA3-AS1 non.epi.lncRNA

GATA6-AS1 non.epi.lncRNA

GAU1 non.epi.lncRNA

GCSIR non.epi.lncRNA

GDNF-AS1 non.epi.lncRNA

GEMIN7-AS1 non.epi.lncRNA

GFOD1-AS1 non.epi.lncRNA

GIHCG non.epi.lncRNA

GK-AS1 non.epi.lncRNA

GK-IT1 non.epi.lncRNA

GLIS3-AS1 non.epi.lncRNA

GLYCTK-AS1 non.epi.lncRNA

GMDS-DT non.epi.lncRNA

GNA14-AS1 non.epi.lncRNA

GNAS-AS1 non.epi.lncRNA

GNG12-AS1 non.epi.lncRNA

GOLGA8M non.epi.lncRNA

GORAB-AS1 non.epi.lncRNA

GPC5-AS1 non.epi.lncRNA

GPC5-AS2 non.epi.lncRNA

GPC5-IT1 non.epi.lncRNA

GPC6-AS1 non.epi.lncRNA

GPC6-AS2 non.epi.lncRNA

GPR1-AS non.epi.lncRNA

GPR158-AS1 non.epi.lncRNA

GPR176-DT non.epi.lncRNA

GPR50-AS1 non.epi.lncRNA

GPRACR non.epi.lncRNA

GPRC5D-AS1 non.epi.lncRNA

GRID1-AS1 non.epi.lncRNA

GRIK1-AS1 non.epi.lncRNA

GRM5-AS1 non.epi.lncRNA

GRM7-AS1 non.epi.lncRNA

GRM7-AS2 non.epi.lncRNA

GRM7-AS3 non.epi.lncRNA

GRTP1-AS1 non.epi.lncRNA

GSEC non.epi.lncRNA

GSN-AS1 non.epi.lncRNA

GTF3C2-AS1 non.epi.lncRNA

GTSCR1 non.epi.lncRNA

GTSE1-DT non.epi.lncRNA

GUSBP11 non.epi.lncRNA

GYG2-AS1 non.epi.lncRNA

H19 non.epi.lncRNA

HAGLR non.epi.lncRNA

HAGLROS non.epi.lncRNA

HAND2-AS1 non.epi.lncRNA

HAO2-IT1 non.epi.lncRNA

HAR1B non.epi.lncRNA

HAS2-AS1 non.epi.lncRNA

HCCAT5 non.epi.lncRNA

HCG14 non.epi.lncRNA

HCG17 non.epi.lncRNA

HCG18 non.epi.lncRNA

HCG20 non.epi.lncRNA

HCG22 non.epi.lncRNA

HCG24 non.epi.lncRNA

HCG25 non.epi.lncRNA

HCP5 non.epi.lncRNA

HDAC11-AS1 non.epi.lncRNA

HDAC2-AS2 non.epi.lncRNA

HDAC4-AS1 non.epi.lncRNA

HDHD5-AS1 non.epi.lncRNA

HECW1-IT1 non.epi.lncRNA

HECW2-AS1 non.epi.lncRNA

HEIH non.epi.lncRNA

HELLPAR non.epi.lncRNA

HEXA-AS1 non.epi.lncRNA

HEXD-IT1 non.epi.lncRNA

HGC6.3 non.epi.lncRNA

HHATL-AS1 non.epi.lncRNA

HHIP-AS1 non.epi.lncRNA

HID1-AS1 non.epi.lncRNA

HIF1A-AS3 non.epi.lncRNA

HIPK1-AS1 non.epi.lncRNA

HLA-DQB1-AS1 non.epi.lncRNA

HLCS-IT1 non.epi.lncRNA

HLTF-AS1 non.epi.lncRNA

HLX-AS1 non.epi.lncRNA

HM13-IT1 non.epi.lncRNA

HMBOX1-IT1 non.epi.lncRNA

HNF1A-AS1 non.epi.lncRNA

HNF4A-AS1 non.epi.lncRNA

HORMAD2-AS1 non.epi.lncRNA

HOTAIR non.epi.lncRNA

HOTAIRM1 non.epi.lncRNA

HOTTIP non.epi.lncRNA

HOXA-AS2 non.epi.lncRNA

HOXA-AS3 non.epi.lncRNA

HOXA10-AS non.epi.lncRNA

HOXA11-AS non.epi.lncRNA

HOXB-AS1 non.epi.lncRNA

HOXB-AS3 non.epi.lncRNA

HOXB-AS4 non.epi.lncRNA

HOXC-AS1 non.epi.lncRNA

HOXC-AS2 non.epi.lncRNA

HOXC-AS3 non.epi.lncRNA

HOXC13-AS non.epi.lncRNA

HOXD-AS2 non.epi.lncRNA

HPN-AS1 non.epi.lncRNA

HPYR1 non.epi.lncRNA

HS6ST2-AS1 non.epi.lncRNA

hsa-mir-1253 non.epi.lncRNA

hsa-mir-423 non.epi.lncRNA

HSD11B1-AS1 non.epi.lncRNA

HSD17B3-AS1 non.epi.lncRNA

HTR2A-AS1 non.epi.lncRNA

HTR5A-AS1 non.epi.lncRNA

HUNK-AS1 non.epi.lncRNA

HYI-AS1 non.epi.lncRNA

IATPR non.epi.lncRNA

IBA57-DT non.epi.lncRNA

ID2-AS1 non.epi.lncRNA

IDH1-AS1 non.epi.lncRNA

IFNG-AS1 non.epi.lncRNA

IFT74-AS1 non.epi.lncRNA

IGBP1-AS1 non.epi.lncRNA

IGBP1-AS2 non.epi.lncRNA

IGF2BP2-AS1 non.epi.lncRNA

IGFBP7-AS1 non.epi.lncRNA

IGFL2-AS1 non.epi.lncRNA

IGSF11-AS1 non.epi.lncRNA

IGSF21-AS1 non.epi.lncRNA

IL10RB-DT non.epi.lncRNA

IL12A-AS1 non.epi.lncRNA

IL1R1-AS1 non.epi.lncRNA

IL20RB-AS1 non.epi.lncRNA

IL21-AS1 non.epi.lncRNA

IL21R-AS1 non.epi.lncRNA

INE2 non.epi.lncRNA

INHBA-AS1 non.epi.lncRNA

INKA2-AS1 non.epi.lncRNA

INSYN1-AS1 non.epi.lncRNA

INTS6-AS1 non.epi.lncRNA

INTS9-AS1 non.epi.lncRNA

IPO9-AS1 non.epi.lncRNA

IQCF5-AS1 non.epi.lncRNA

IQCH-AS1 non.epi.lncRNA

IQCJ-SCHIP1-AS1 non.epi.lncRNA

IRAIN non.epi.lncRNA

ISM1-AS1 non.epi.lncRNA

ITFG1-AS1 non.epi.lncRNA

ITFG2-AS1 non.epi.lncRNA

ITGA6-AS1 non.epi.lncRNA

ITGA9-AS1 non.epi.lncRNA

ITGB1-DT non.epi.lncRNA

ITGB2-AS1 non.epi.lncRNA

ITIH4-AS1 non.epi.lncRNA

ITPK1-AS1 non.epi.lncRNA

ITPKB-AS1 non.epi.lncRNA

ITPKB-IT1 non.epi.lncRNA

ITPR1-DT non.epi.lncRNA

JADRR non.epi.lncRNA

JAKMIP2-AS1 non.epi.lncRNA

JARID2-AS1 non.epi.lncRNA

JAZF1-AS1 non.epi.lncRNA

JMJD1C-AS1 non.epi.lncRNA

JPX non.epi.lncRNA

JRKL-AS1 non.epi.lncRNA

KAZN-AS1 non.epi.lncRNA

KBTBD11-OT1 non.epi.lncRNA

KC877392.1 non.epi.lncRNA

KC877982.1 non.epi.lncRNA

KCNAB1-AS2 non.epi.lncRNA

KCND3-AS1 non.epi.lncRNA

KCND3-IT1 non.epi.lncRNA

KCNH1-IT1 non.epi.lncRNA

KCNIP4-IT1 non.epi.lncRNA

KCNJ6-AS1 non.epi.lncRNA

KCNK15-AS1 non.epi.lncRNA

KCNK4-TEX40 non.epi.lncRNA

KCNMA1-AS1 non.epi.lncRNA

KCNMA1-AS2 non.epi.lncRNA

KCNMB2-AS1 non.epi.lncRNA

KCNQ1-AS1 non.epi.lncRNA

KCNQ1DN non.epi.lncRNA

KCNQ1OT1 non.epi.lncRNA

KCNQ5-AS1 non.epi.lncRNA

KCNQ5-IT1 non.epi.lncRNA

KCTD21-AS1 non.epi.lncRNA

KDM4A-AS1 non.epi.lncRNA

KDM5C-IT1 non.epi.lncRNA

KF456478.1 non.epi.lncRNA

KHDRBS2-OT non.epi.lncRNA

KIAA1614-AS1 non.epi.lncRNA

KIAA2012-AS1 non.epi.lncRNA

KIF25-AS1 non.epi.lncRNA

KIF26B-AS1 non.epi.lncRNA

KIRREL3-AS1 non.epi.lncRNA

KIRREL3-AS2 non.epi.lncRNA

KIRREL3-AS3 non.epi.lncRNA

KLF3-AS1 non.epi.lncRNA

KLF7-IT1 non.epi.lncRNA

KLHL6-AS1 non.epi.lncRNA

KLHL7-DT non.epi.lncRNA

KMT2E-AS1 non.epi.lncRNA

KRBOX1-AS1 non.epi.lncRNA

KRT7-AS non.epi.lncRNA

KRT73-AS1 non.epi.lncRNA

KRTAP5-AS1 non.epi.lncRNA

L29074.1 non.epi.lncRNA

L34079.2 non.epi.lncRNA

L3MBTL4-AS1 non.epi.lncRNA

LACTB2-AS1 non.epi.lncRNA

LAMC1-AS1 non.epi.lncRNA

LAMP5-AS1 non.epi.lncRNA

LAMTOR5-AS1 non.epi.lncRNA

LANCL1-AS1 non.epi.lncRNA

LAPTM4A-DT non.epi.lncRNA

LARGE-AS1 non.epi.lncRNA

LARGE-IT1 non.epi.lncRNA

LARS2-AS1 non.epi.lncRNA

LATS2-AS1 non.epi.lncRNA

LBX1-AS1 non.epi.lncRNA

LBX2-AS1 non.epi.lncRNA

LCMT1-AS2 non.epi.lncRNA

LDLRAD4-AS1 non.epi.lncRNA

LEF1-AS1 non.epi.lncRNA

LEMD1-AS1 non.epi.lncRNA

LERFS non.epi.lncRNA

LGALS8-AS1 non.epi.lncRNA

LGALSL-DT non.epi.lncRNA

LHFPL3-AS1 non.epi.lncRNA

LHFPL3-AS2 non.epi.lncRNA

LHX1-DT non.epi.lncRNA

LHX5-AS1 non.epi.lncRNA

LIFR-AS1 non.epi.lncRNA

LILRB1-AS1 non.epi.lncRNA

LIMD1-AS1 non.epi.lncRNA

LIN28B-AS1 non.epi.lncRNA

LINC-PINT non.epi.lncRNA

LINC-ROR non.epi.lncRNA

LINC00029 non.epi.lncRNA

LINC00051 non.epi.lncRNA

LINC00052 non.epi.lncRNA

LINC00092 non.epi.lncRNA

LINC00102 non.epi.lncRNA

LINC00106 non.epi.lncRNA

LINC00112 non.epi.lncRNA

LINC00113 non.epi.lncRNA

LINC00114 non.epi.lncRNA

LINC00115 non.epi.lncRNA

LINC00158 non.epi.lncRNA

LINC00159 non.epi.lncRNA

LINC00160 non.epi.lncRNA

LINC00161 non.epi.lncRNA

LINC00163 non.epi.lncRNA

LINC00165 non.epi.lncRNA

LINC00174 non.epi.lncRNA

LINC00189 non.epi.lncRNA

LINC00200 non.epi.lncRNA

LINC00207 non.epi.lncRNA

LINC00208 non.epi.lncRNA

LINC00210 non.epi.lncRNA

LINC00211 non.epi.lncRNA

LINC00221 non.epi.lncRNA

LINC00222 non.epi.lncRNA

LINC00226 non.epi.lncRNA

LINC00229 non.epi.lncRNA

LINC00235 non.epi.lncRNA

LINC00237 non.epi.lncRNA

LINC00239 non.epi.lncRNA

LINC00240 non.epi.lncRNA

LINC00244 non.epi.lncRNA

LINC00251 non.epi.lncRNA

LINC00254 non.epi.lncRNA

LINC00261 non.epi.lncRNA

LINC00266-1 non.epi.lncRNA

LINC00266-4P non.epi.lncRNA

LINC00269 non.epi.lncRNA

LINC00271 non.epi.lncRNA

LINC00272 non.epi.lncRNA

LINC00273 non.epi.lncRNA

LINC00276 non.epi.lncRNA

LINC00278 non.epi.lncRNA

LINC00280 non.epi.lncRNA

LINC00290 non.epi.lncRNA

LINC00293 non.epi.lncRNA

LINC00297 non.epi.lncRNA

LINC00298 non.epi.lncRNA

LINC00299 non.epi.lncRNA

LINC00303 non.epi.lncRNA

LINC00304 non.epi.lncRNA

LINC00305 non.epi.lncRNA

LINC00307 non.epi.lncRNA

LINC00308 non.epi.lncRNA

LINC00309 non.epi.lncRNA

LINC00310 non.epi.lncRNA

LINC00311 non.epi.lncRNA

LINC00313 non.epi.lncRNA

LINC00314 non.epi.lncRNA

LINC00315 non.epi.lncRNA

LINC00316 non.epi.lncRNA

LINC00317 non.epi.lncRNA

LINC00319 non.epi.lncRNA

LINC00320 non.epi.lncRNA

LINC00322 non.epi.lncRNA

LINC00323 non.epi.lncRNA

LINC00324 non.epi.lncRNA

LINC00326 non.epi.lncRNA

LINC00327 non.epi.lncRNA

LINC00330 non.epi.lncRNA

LINC00331 non.epi.lncRNA

LINC00332 non.epi.lncRNA

LINC00333 non.epi.lncRNA

LINC00334 non.epi.lncRNA

LINC00339 non.epi.lncRNA

LINC00342 non.epi.lncRNA

LINC00343 non.epi.lncRNA

LINC00345 non.epi.lncRNA

LINC00347 non.epi.lncRNA

LINC00348 non.epi.lncRNA

LINC00349 non.epi.lncRNA

LINC00350 non.epi.lncRNA

LINC00351 non.epi.lncRNA

LINC00352 non.epi.lncRNA

LINC00353 non.epi.lncRNA

LINC00354 non.epi.lncRNA

LINC00355 non.epi.lncRNA

LINC00358 non.epi.lncRNA

LINC00362 non.epi.lncRNA

LINC00363 non.epi.lncRNA

LINC00365 non.epi.lncRNA

LINC00366 non.epi.lncRNA

LINC00368 non.epi.lncRNA

LINC00370 non.epi.lncRNA

LINC00373 non.epi.lncRNA

LINC00374 non.epi.lncRNA

LINC00375 non.epi.lncRNA

LINC00376 non.epi.lncRNA

LINC00377 non.epi.lncRNA

LINC00378 non.epi.lncRNA

LINC00379 non.epi.lncRNA

LINC00380 non.epi.lncRNA

LINC00381 non.epi.lncRNA

LINC00382 non.epi.lncRNA

LINC00383 non.epi.lncRNA

LINC00384 non.epi.lncRNA

LINC00385 non.epi.lncRNA

LINC00387 non.epi.lncRNA

LINC00388 non.epi.lncRNA

LINC00390 non.epi.lncRNA

LINC00391 non.epi.lncRNA

LINC00392 non.epi.lncRNA

LINC00393 non.epi.lncRNA

LINC00395 non.epi.lncRNA

LINC00396 non.epi.lncRNA

LINC00398 non.epi.lncRNA

LINC00399 non.epi.lncRNA

LINC00400 non.epi.lncRNA

LINC00402 non.epi.lncRNA

LINC00404 non.epi.lncRNA

LINC00407 non.epi.lncRNA

LINC00408 non.epi.lncRNA

LINC00410 non.epi.lncRNA

LINC00411 non.epi.lncRNA

LINC00412 non.epi.lncRNA

LINC00415 non.epi.lncRNA

LINC00421 non.epi.lncRNA

LINC00423 non.epi.lncRNA

LINC00424 non.epi.lncRNA

LINC00426 non.epi.lncRNA

LINC00427 non.epi.lncRNA

LINC00428 non.epi.lncRNA

LINC00430 non.epi.lncRNA

LINC00433 non.epi.lncRNA

LINC00434 non.epi.lncRNA

LINC00437 non.epi.lncRNA

LINC00440 non.epi.lncRNA

LINC00442 non.epi.lncRNA

LINC00443 non.epi.lncRNA

LINC00445 non.epi.lncRNA

LINC00446 non.epi.lncRNA

LINC00448 non.epi.lncRNA

LINC00449 non.epi.lncRNA

LINC00452 non.epi.lncRNA

LINC00454 non.epi.lncRNA

LINC00456 non.epi.lncRNA

LINC00457 non.epi.lncRNA

LINC00458 non.epi.lncRNA

LINC00459 non.epi.lncRNA

LINC00461 non.epi.lncRNA

LINC00462 non.epi.lncRNA

LINC00463 non.epi.lncRNA

LINC00466 non.epi.lncRNA

LINC00467 non.epi.lncRNA

LINC00469 non.epi.lncRNA

LINC00470 non.epi.lncRNA

LINC00471 non.epi.lncRNA

LINC00472 non.epi.lncRNA

LINC00474 non.epi.lncRNA

LINC00476 non.epi.lncRNA

LINC00477 non.epi.lncRNA

LINC00479 non.epi.lncRNA

LINC00482 non.epi.lncRNA

LINC00486 non.epi.lncRNA

LINC00487 non.epi.lncRNA

LINC00488 non.epi.lncRNA

LINC00489 non.epi.lncRNA

LINC00491 non.epi.lncRNA

LINC00492 non.epi.lncRNA

LINC00494 non.epi.lncRNA

LINC00498 non.epi.lncRNA

LINC00499 non.epi.lncRNA

LINC00500 non.epi.lncRNA

LINC00501 non.epi.lncRNA

LINC00502 non.epi.lncRNA

LINC00504 non.epi.lncRNA

LINC00507 non.epi.lncRNA

LINC00508 non.epi.lncRNA

LINC00511 non.epi.lncRNA

LINC00513 non.epi.lncRNA

LINC00517 non.epi.lncRNA

LINC00518 non.epi.lncRNA

LINC00519 non.epi.lncRNA

LINC00520 non.epi.lncRNA

LINC00523 non.epi.lncRNA

LINC00524 non.epi.lncRNA

LINC00526 non.epi.lncRNA

LINC00529 non.epi.lncRNA

LINC00534 non.epi.lncRNA

LINC00535 non.epi.lncRNA

LINC00536 non.epi.lncRNA

LINC00538 non.epi.lncRNA

LINC00539 non.epi.lncRNA

LINC00543 non.epi.lncRNA

LINC00544 non.epi.lncRNA

LINC00545 non.epi.lncRNA

LINC00547 non.epi.lncRNA

LINC00550 non.epi.lncRNA

LINC00551 non.epi.lncRNA

LINC00554 non.epi.lncRNA

LINC00555 non.epi.lncRNA

LINC00556 non.epi.lncRNA

LINC00557 non.epi.lncRNA

LINC00558 non.epi.lncRNA

LINC00559 non.epi.lncRNA

LINC00560 non.epi.lncRNA

LINC00561 non.epi.lncRNA

LINC00562 non.epi.lncRNA

LINC00563 non.epi.lncRNA

LINC00564 non.epi.lncRNA

LINC00565 non.epi.lncRNA

LINC00566 non.epi.lncRNA

LINC00567 non.epi.lncRNA

LINC00571 non.epi.lncRNA

LINC00572 non.epi.lncRNA

LINC00574 non.epi.lncRNA

LINC00575 non.epi.lncRNA

LINC00578 non.epi.lncRNA

LINC00581 non.epi.lncRNA

LINC00583 non.epi.lncRNA

LINC00587 non.epi.lncRNA

LINC00588 non.epi.lncRNA

LINC00589 non.epi.lncRNA

LINC00592 non.epi.lncRNA

LINC00595 non.epi.lncRNA

LINC00596 non.epi.lncRNA

LINC00598 non.epi.lncRNA

LINC00599 non.epi.lncRNA

LINC00601 non.epi.lncRNA

LINC00603 non.epi.lncRNA

LINC00604 non.epi.lncRNA

LINC00605 non.epi.lncRNA

LINC00606 non.epi.lncRNA

LINC00607 non.epi.lncRNA

LINC00608 non.epi.lncRNA

LINC00609 non.epi.lncRNA

LINC00612 non.epi.lncRNA

LINC00613 non.epi.lncRNA

LINC00615 non.epi.lncRNA

LINC00616 non.epi.lncRNA

LINC00618 non.epi.lncRNA

LINC00620 non.epi.lncRNA

LINC00621 non.epi.lncRNA

LINC00622 non.epi.lncRNA

LINC00623 non.epi.lncRNA

LINC00624 non.epi.lncRNA

LINC00626 non.epi.lncRNA

LINC00628 non.epi.lncRNA

LINC00629 non.epi.lncRNA

LINC00630 non.epi.lncRNA

LINC00632 non.epi.lncRNA

LINC00635 non.epi.lncRNA

LINC00636 non.epi.lncRNA

LINC00637 non.epi.lncRNA

LINC00639 non.epi.lncRNA

LINC00640 non.epi.lncRNA

LINC00642 non.epi.lncRNA

LINC00644 non.epi.lncRNA

LINC00645 non.epi.lncRNA

LINC00648 non.epi.lncRNA

LINC00649 non.epi.lncRNA

LINC00652 non.epi.lncRNA

LINC00654 non.epi.lncRNA

LINC00656 non.epi.lncRNA

LINC00658 non.epi.lncRNA

LINC00659 non.epi.lncRNA

LINC00661 non.epi.lncRNA

LINC00662 non.epi.lncRNA

LINC00665 non.epi.lncRNA

LINC00667 non.epi.lncRNA

LINC00668 non.epi.lncRNA

LINC00670 non.epi.lncRNA

LINC00671 non.epi.lncRNA

LINC00676 non.epi.lncRNA

LINC00678 non.epi.lncRNA

LINC00681 non.epi.lncRNA

LINC00682 non.epi.lncRNA

LINC00683 non.epi.lncRNA

LINC00685 non.epi.lncRNA

LINC00687 non.epi.lncRNA

LINC00690 non.epi.lncRNA

LINC00691 non.epi.lncRNA

LINC00692 non.epi.lncRNA

LINC00698 non.epi.lncRNA

LINC00700 non.epi.lncRNA

LINC00701 non.epi.lncRNA

LINC00702 non.epi.lncRNA

LINC00703 non.epi.lncRNA

LINC00705 non.epi.lncRNA

LINC00706 non.epi.lncRNA

LINC00707 non.epi.lncRNA

LINC00708 non.epi.lncRNA

LINC00709 non.epi.lncRNA

LINC00710 non.epi.lncRNA

LINC00824 non.epi.lncRNA

LINC00836 non.epi.lncRNA

LINC00837 non.epi.lncRNA

LINC00838 non.epi.lncRNA

LINC00839 non.epi.lncRNA

LINC00840 non.epi.lncRNA

LINC00841 non.epi.lncRNA

LINC00844 non.epi.lncRNA

LINC00845 non.epi.lncRNA

LINC00850 non.epi.lncRNA

LINC00851 non.epi.lncRNA

LINC00852 non.epi.lncRNA

LINC00853 non.epi.lncRNA

LINC00858 non.epi.lncRNA

LINC00861 non.epi.lncRNA

LINC00862 non.epi.lncRNA

LINC00863 non.epi.lncRNA

LINC00865 non.epi.lncRNA

LINC00867 non.epi.lncRNA

LINC00868 non.epi.lncRNA

LINC00870 non.epi.lncRNA

LINC00871 non.epi.lncRNA

LINC00877 non.epi.lncRNA

LINC00879 non.epi.lncRNA

LINC00880 non.epi.lncRNA

LINC00881 non.epi.lncRNA

LINC00882 non.epi.lncRNA

LINC00884 non.epi.lncRNA

LINC00885 non.epi.lncRNA

LINC00887 non.epi.lncRNA

LINC00891 non.epi.lncRNA

LINC00892 non.epi.lncRNA

LINC00894 non.epi.lncRNA

LINC00895 non.epi.lncRNA

LINC00896 non.epi.lncRNA

LINC00898 non.epi.lncRNA

LINC00900 non.epi.lncRNA

LINC00901 non.epi.lncRNA

LINC00903 non.epi.lncRNA

LINC00905 non.epi.lncRNA

LINC00906 non.epi.lncRNA

LINC00907 non.epi.lncRNA

LINC00909 non.epi.lncRNA

LINC00910 non.epi.lncRNA

LINC00911 non.epi.lncRNA

LINC00917 non.epi.lncRNA

LINC00919 non.epi.lncRNA

LINC00920 non.epi.lncRNA

LINC00921 non.epi.lncRNA

LINC00922 non.epi.lncRNA

LINC00923 non.epi.lncRNA

LINC00924 non.epi.lncRNA

LINC00926 non.epi.lncRNA

LINC00927 non.epi.lncRNA

LINC00928 non.epi.lncRNA

LINC00929 non.epi.lncRNA

LINC00934 non.epi.lncRNA

LINC00937 non.epi.lncRNA

LINC00939 non.epi.lncRNA

LINC00940 non.epi.lncRNA

LINC00943 non.epi.lncRNA

LINC00944 non.epi.lncRNA

LINC00945 non.epi.lncRNA

LINC00951 non.epi.lncRNA

LINC00954 non.epi.lncRNA

LINC00955 non.epi.lncRNA

LINC00957 non.epi.lncRNA

LINC00960 non.epi.lncRNA

LINC00963 non.epi.lncRNA

LINC00964 non.epi.lncRNA

LINC00967 non.epi.lncRNA

LINC00968 non.epi.lncRNA

LINC00970 non.epi.lncRNA

LINC00971 non.epi.lncRNA

LINC00972 non.epi.lncRNA

LINC00973 non.epi.lncRNA

LINC00989 non.epi.lncRNA

LINC00992 non.epi.lncRNA

LINC00994 non.epi.lncRNA

LINC00996 non.epi.lncRNA

LINC00997 non.epi.lncRNA

LINC01001 non.epi.lncRNA

LINC01003 non.epi.lncRNA

LINC01004 non.epi.lncRNA

LINC01005 non.epi.lncRNA

LINC01006 non.epi.lncRNA

LINC01007 non.epi.lncRNA

LINC01010 non.epi.lncRNA

LINC01014 non.epi.lncRNA

LINC01015 non.epi.lncRNA

LINC01017 non.epi.lncRNA

LINC01018 non.epi.lncRNA

LINC01019 non.epi.lncRNA

LINC01020 non.epi.lncRNA

LINC01022 non.epi.lncRNA

LINC01023 non.epi.lncRNA

LINC01028 non.epi.lncRNA

LINC01029 non.epi.lncRNA

LINC01030 non.epi.lncRNA

LINC01031 non.epi.lncRNA

LINC01033 non.epi.lncRNA

LINC01034 non.epi.lncRNA

LINC01035 non.epi.lncRNA

LINC01038 non.epi.lncRNA

LINC01039 non.epi.lncRNA

LINC01040 non.epi.lncRNA

LINC01043 non.epi.lncRNA

LINC01044 non.epi.lncRNA

LINC01046 non.epi.lncRNA

LINC01047 non.epi.lncRNA

LINC01048 non.epi.lncRNA

LINC01049 non.epi.lncRNA

LINC01050 non.epi.lncRNA

LINC01052 non.epi.lncRNA

LINC01053 non.epi.lncRNA

LINC01054 non.epi.lncRNA

LINC01055 non.epi.lncRNA

LINC01056 non.epi.lncRNA

LINC01058 non.epi.lncRNA

LINC01060 non.epi.lncRNA

LINC01065 non.epi.lncRNA

LINC01066 non.epi.lncRNA

LINC01067 non.epi.lncRNA

LINC01068 non.epi.lncRNA

LINC01069 non.epi.lncRNA

LINC01070 non.epi.lncRNA

LINC01072 non.epi.lncRNA

LINC01074 non.epi.lncRNA

LINC01075 non.epi.lncRNA

LINC01076 non.epi.lncRNA

LINC01077 non.epi.lncRNA

LINC01078 non.epi.lncRNA

LINC01079 non.epi.lncRNA

LINC01081 non.epi.lncRNA

LINC01082 non.epi.lncRNA

LINC01087 non.epi.lncRNA

LINC01088 non.epi.lncRNA

LINC01090 non.epi.lncRNA

LINC01091 non.epi.lncRNA

LINC01093 non.epi.lncRNA

LINC01094 non.epi.lncRNA

LINC01095 non.epi.lncRNA

LINC01096 non.epi.lncRNA

LINC01097 non.epi.lncRNA

LINC01098 non.epi.lncRNA

LINC01099 non.epi.lncRNA

LINC01100 non.epi.lncRNA

LINC01102 non.epi.lncRNA

LINC01103 non.epi.lncRNA

LINC01104 non.epi.lncRNA

LINC01107 non.epi.lncRNA

LINC01108 non.epi.lncRNA

LINC01109 non.epi.lncRNA

LINC01111 non.epi.lncRNA

LINC01115 non.epi.lncRNA

LINC01116 non.epi.lncRNA

LINC01117 non.epi.lncRNA

LINC01118 non.epi.lncRNA

LINC01119 non.epi.lncRNA

LINC01120 non.epi.lncRNA

LINC01121 non.epi.lncRNA

LINC01122 non.epi.lncRNA

LINC01123 non.epi.lncRNA

LINC01126 non.epi.lncRNA

LINC01127 non.epi.lncRNA

LINC01128 non.epi.lncRNA

LINC01133 non.epi.lncRNA

LINC01135 non.epi.lncRNA

LINC01136 non.epi.lncRNA

LINC01137 non.epi.lncRNA

LINC01138 non.epi.lncRNA

LINC01141 non.epi.lncRNA

LINC01142 non.epi.lncRNA

LINC01143 non.epi.lncRNA

LINC01144 non.epi.lncRNA

LINC01146 non.epi.lncRNA

LINC01147 non.epi.lncRNA

LINC01148 non.epi.lncRNA

LINC01150 non.epi.lncRNA

LINC01151 non.epi.lncRNA

LINC01153 non.epi.lncRNA

LINC01154 non.epi.lncRNA

LINC01159 non.epi.lncRNA

LINC01160 non.epi.lncRNA

LINC01162 non.epi.lncRNA

LINC01163 non.epi.lncRNA

LINC01164 non.epi.lncRNA

LINC01166 non.epi.lncRNA

LINC01167 non.epi.lncRNA

LINC01168 non.epi.lncRNA

LINC01169 non.epi.lncRNA

LINC01170 non.epi.lncRNA

LINC01173 non.epi.lncRNA

LINC01176 non.epi.lncRNA

LINC01177 non.epi.lncRNA

LINC01179 non.epi.lncRNA

LINC01180 non.epi.lncRNA

LINC01181 non.epi.lncRNA

LINC01182 non.epi.lncRNA

LINC01184 non.epi.lncRNA

LINC01185 non.epi.lncRNA

LINC01186 non.epi.lncRNA

LINC01189 non.epi.lncRNA

LINC01191 non.epi.lncRNA

LINC01192 non.epi.lncRNA

LINC01193 non.epi.lncRNA

LINC01194 non.epi.lncRNA

LINC01195 non.epi.lncRNA

LINC01197 non.epi.lncRNA

LINC01198 non.epi.lncRNA

LINC01201 non.epi.lncRNA

LINC01202 non.epi.lncRNA

LINC01203 non.epi.lncRNA

LINC01204 non.epi.lncRNA

LINC01205 non.epi.lncRNA

LINC01206 non.epi.lncRNA

LINC01208 non.epi.lncRNA

LINC01209 non.epi.lncRNA

LINC01210 non.epi.lncRNA

LINC01213 non.epi.lncRNA

LINC01214 non.epi.lncRNA

LINC01216 non.epi.lncRNA

LINC01217 non.epi.lncRNA

LINC01218 non.epi.lncRNA

LINC01219 non.epi.lncRNA

LINC01220 non.epi.lncRNA

LINC01221 non.epi.lncRNA

LINC01222 non.epi.lncRNA

LINC01224 non.epi.lncRNA

LINC01227 non.epi.lncRNA

LINC01228 non.epi.lncRNA

LINC01229 non.epi.lncRNA

LINC01230 non.epi.lncRNA

LINC01231 non.epi.lncRNA

LINC01232 non.epi.lncRNA

LINC01233 non.epi.lncRNA

LINC01234 non.epi.lncRNA

LINC01235 non.epi.lncRNA

LINC01237 non.epi.lncRNA

LINC01238 non.epi.lncRNA

LINC01239 non.epi.lncRNA

LINC01241 non.epi.lncRNA

LINC01242 non.epi.lncRNA

LINC01243 non.epi.lncRNA

LINC01247 non.epi.lncRNA

LINC01248 non.epi.lncRNA

LINC01249 non.epi.lncRNA

LINC01250 non.epi.lncRNA

LINC01251 non.epi.lncRNA

LINC01252 non.epi.lncRNA

LINC01254 non.epi.lncRNA

LINC01256 non.epi.lncRNA

LINC01257 non.epi.lncRNA

LINC01258 non.epi.lncRNA

LINC01259 non.epi.lncRNA

LINC01262 non.epi.lncRNA

LINC01264 non.epi.lncRNA

LINC01266 non.epi.lncRNA

LINC01267 non.epi.lncRNA

LINC01268 non.epi.lncRNA

LINC01271 non.epi.lncRNA

LINC01276 non.epi.lncRNA

LINC01278 non.epi.lncRNA

LINC01280 non.epi.lncRNA

LINC01281 non.epi.lncRNA

LINC01282 non.epi.lncRNA

LINC01283 non.epi.lncRNA

LINC01284 non.epi.lncRNA

LINC01285 non.epi.lncRNA

LINC01287 non.epi.lncRNA

LINC01288 non.epi.lncRNA

LINC01289 non.epi.lncRNA

LINC01290 non.epi.lncRNA

LINC01291 non.epi.lncRNA

LINC01293 non.epi.lncRNA

LINC01297 non.epi.lncRNA

LINC01298 non.epi.lncRNA

LINC01299 non.epi.lncRNA

LINC01300 non.epi.lncRNA

LINC01301 non.epi.lncRNA

LINC01303 non.epi.lncRNA

LINC01304 non.epi.lncRNA

LINC01305 non.epi.lncRNA

LINC01307 non.epi.lncRNA

LINC01309 non.epi.lncRNA

LINC01310 non.epi.lncRNA

LINC01311 non.epi.lncRNA

LINC01312 non.epi.lncRNA

LINC01318 non.epi.lncRNA

LINC01320 non.epi.lncRNA

LINC01322 non.epi.lncRNA

LINC01323 non.epi.lncRNA

LINC01324 non.epi.lncRNA

LINC01326 non.epi.lncRNA

LINC01327 non.epi.lncRNA

LINC01331 non.epi.lncRNA

LINC01333 non.epi.lncRNA

LINC01335 non.epi.lncRNA

LINC01337 non.epi.lncRNA

LINC01338 non.epi.lncRNA

LINC01339 non.epi.lncRNA

LINC01340 non.epi.lncRNA

LINC01341 non.epi.lncRNA

LINC01342 non.epi.lncRNA

LINC01343 non.epi.lncRNA

LINC01344 non.epi.lncRNA

LINC01345 non.epi.lncRNA

LINC01346 non.epi.lncRNA

LINC01348 non.epi.lncRNA

LINC01349 non.epi.lncRNA

LINC01350 non.epi.lncRNA

LINC01351 non.epi.lncRNA

LINC01352 non.epi.lncRNA

LINC01353 non.epi.lncRNA

LINC01354 non.epi.lncRNA

LINC01355 non.epi.lncRNA

LINC01356 non.epi.lncRNA

LINC01357 non.epi.lncRNA

LINC01358 non.epi.lncRNA

LINC01359 non.epi.lncRNA

LINC01360 non.epi.lncRNA

LINC01361 non.epi.lncRNA

LINC01362 non.epi.lncRNA

LINC01363 non.epi.lncRNA

LINC01364 non.epi.lncRNA

LINC01365 non.epi.lncRNA

LINC01366 non.epi.lncRNA

LINC01370 non.epi.lncRNA

LINC01374 non.epi.lncRNA

LINC01375 non.epi.lncRNA

LINC01376 non.epi.lncRNA

LINC01378 non.epi.lncRNA

LINC01381 non.epi.lncRNA

LINC01385 non.epi.lncRNA

LINC01386 non.epi.lncRNA

LINC01387 non.epi.lncRNA

LINC01388 non.epi.lncRNA

LINC01389 non.epi.lncRNA

LINC01391 non.epi.lncRNA

LINC01392 non.epi.lncRNA

LINC01393 non.epi.lncRNA

LINC01396 non.epi.lncRNA

LINC01397 non.epi.lncRNA

LINC01398 non.epi.lncRNA

LINC01400 non.epi.lncRNA

LINC01402 non.epi.lncRNA

LINC01405 non.epi.lncRNA

LINC01409 non.epi.lncRNA

LINC01410 non.epi.lncRNA

LINC01411 non.epi.lncRNA

LINC01413 non.epi.lncRNA

LINC01414 non.epi.lncRNA

LINC01415 non.epi.lncRNA

LINC01416 non.epi.lncRNA

LINC01419 non.epi.lncRNA

LINC01422 non.epi.lncRNA

LINC01423 non.epi.lncRNA

LINC01425 non.epi.lncRNA

LINC01426 non.epi.lncRNA

LINC01427 non.epi.lncRNA

LINC01428 non.epi.lncRNA

LINC01429 non.epi.lncRNA

LINC01432 non.epi.lncRNA

LINC01433 non.epi.lncRNA

LINC01435 non.epi.lncRNA

LINC01436 non.epi.lncRNA

LINC01438 non.epi.lncRNA

LINC01440 non.epi.lncRNA

LINC01441 non.epi.lncRNA

LINC01442 non.epi.lncRNA

LINC01443 non.epi.lncRNA

LINC01444 non.epi.lncRNA

LINC01445 non.epi.lncRNA

LINC01446 non.epi.lncRNA

LINC01448 non.epi.lncRNA

LINC01449 non.epi.lncRNA

LINC01450 non.epi.lncRNA

LINC01455 non.epi.lncRNA

LINC01456 non.epi.lncRNA

LINC01460 non.epi.lncRNA

LINC01465 non.epi.lncRNA

LINC01467 non.epi.lncRNA

LINC01470 non.epi.lncRNA

LINC01471 non.epi.lncRNA

LINC01473 non.epi.lncRNA

LINC01474 non.epi.lncRNA

LINC01475 non.epi.lncRNA

LINC01476 non.epi.lncRNA

LINC01477 non.epi.lncRNA

LINC01478 non.epi.lncRNA

LINC01479 non.epi.lncRNA

LINC01480 non.epi.lncRNA

LINC01481 non.epi.lncRNA

LINC01482 non.epi.lncRNA

LINC01483 non.epi.lncRNA

LINC01484 non.epi.lncRNA

LINC01485 non.epi.lncRNA

LINC01487 non.epi.lncRNA

LINC01490 non.epi.lncRNA

LINC01491 non.epi.lncRNA

LINC01492 non.epi.lncRNA

LINC01493 non.epi.lncRNA

LINC01494 non.epi.lncRNA

LINC01495 non.epi.lncRNA

LINC01496 non.epi.lncRNA

LINC01497 non.epi.lncRNA

LINC01498 non.epi.lncRNA

LINC01499 non.epi.lncRNA

LINC01500 non.epi.lncRNA

LINC01501 non.epi.lncRNA

LINC01502 non.epi.lncRNA

LINC01503 non.epi.lncRNA

LINC01505 non.epi.lncRNA

LINC01506 non.epi.lncRNA

LINC01507 non.epi.lncRNA

LINC01508 non.epi.lncRNA

LINC01509 non.epi.lncRNA

LINC01510 non.epi.lncRNA

LINC01511 non.epi.lncRNA

LINC01514 non.epi.lncRNA

LINC01515 non.epi.lncRNA

LINC01516 non.epi.lncRNA

LINC01517 non.epi.lncRNA

LINC01518 non.epi.lncRNA

LINC01519 non.epi.lncRNA

LINC01520 non.epi.lncRNA

LINC01522 non.epi.lncRNA

LINC01523 non.epi.lncRNA

LINC01524 non.epi.lncRNA

LINC01525 non.epi.lncRNA

LINC01526 non.epi.lncRNA

LINC01527 non.epi.lncRNA

LINC01532 non.epi.lncRNA

LINC01533 non.epi.lncRNA

LINC01534 non.epi.lncRNA

LINC01535 non.epi.lncRNA

LINC01537 non.epi.lncRNA

LINC01538 non.epi.lncRNA

LINC01539 non.epi.lncRNA

LINC01541 non.epi.lncRNA

LINC01544 non.epi.lncRNA

LINC01545 non.epi.lncRNA

LINC01548 non.epi.lncRNA

LINC01549 non.epi.lncRNA

LINC01550 non.epi.lncRNA

LINC01551 non.epi.lncRNA

LINC01553 non.epi.lncRNA

LINC01554 non.epi.lncRNA

LINC01558 non.epi.lncRNA

LINC01559 non.epi.lncRNA

LINC01561 non.epi.lncRNA

LINC01562 non.epi.lncRNA

LINC01563 non.epi.lncRNA

LINC01564 non.epi.lncRNA

LINC01565 non.epi.lncRNA

LINC01566 non.epi.lncRNA

LINC01567 non.epi.lncRNA

LINC01568 non.epi.lncRNA

LINC01569 non.epi.lncRNA

LINC01570 non.epi.lncRNA

LINC01571 non.epi.lncRNA

LINC01572 non.epi.lncRNA

LINC01574 non.epi.lncRNA

LINC01578 non.epi.lncRNA

LINC01579 non.epi.lncRNA

LINC01580 non.epi.lncRNA

LINC01581 non.epi.lncRNA

LINC01582 non.epi.lncRNA

LINC01584 non.epi.lncRNA

LINC01585 non.epi.lncRNA

LINC01586 non.epi.lncRNA

LINC01587 non.epi.lncRNA

LINC01588 non.epi.lncRNA

LINC01589 non.epi.lncRNA

LINC01591 non.epi.lncRNA

LINC01592 non.epi.lncRNA

LINC01593 non.epi.lncRNA

LINC01594 non.epi.lncRNA

LINC01595 non.epi.lncRNA

LINC01596 non.epi.lncRNA

LINC01600 non.epi.lncRNA

LINC01602 non.epi.lncRNA

LINC01603 non.epi.lncRNA

LINC01605 non.epi.lncRNA

LINC01606 non.epi.lncRNA

LINC01607 non.epi.lncRNA

LINC01608 non.epi.lncRNA

LINC01609 non.epi.lncRNA

LINC01611 non.epi.lncRNA

LINC01612 non.epi.lncRNA

LINC01613 non.epi.lncRNA

LINC01614 non.epi.lncRNA

LINC01615 non.epi.lncRNA

LINC01616 non.epi.lncRNA

LINC01618 non.epi.lncRNA

LINC01619 non.epi.lncRNA

LINC01620 non.epi.lncRNA

LINC01621 non.epi.lncRNA

LINC01623 non.epi.lncRNA

LINC01624 non.epi.lncRNA

LINC01625 non.epi.lncRNA

LINC01626 non.epi.lncRNA

LINC01627 non.epi.lncRNA

LINC01628 non.epi.lncRNA

LINC01630 non.epi.lncRNA

LINC01632 non.epi.lncRNA

LINC01634 non.epi.lncRNA

LINC01638 non.epi.lncRNA

LINC01639 non.epi.lncRNA

LINC01640 non.epi.lncRNA

LINC01641 non.epi.lncRNA

LINC01643 non.epi.lncRNA

LINC01644 non.epi.lncRNA

LINC01646 non.epi.lncRNA

LINC01647 non.epi.lncRNA

LINC01648 non.epi.lncRNA

LINC01649 non.epi.lncRNA

LINC01650 non.epi.lncRNA

LINC01651 non.epi.lncRNA

LINC01653 non.epi.lncRNA

LINC01654 non.epi.lncRNA

LINC01655 non.epi.lncRNA

LINC01656 non.epi.lncRNA

LINC01657 non.epi.lncRNA

LINC01659 non.epi.lncRNA

LINC01661 non.epi.lncRNA

LINC01664 non.epi.lncRNA

LINC01665 non.epi.lncRNA

LINC01666 non.epi.lncRNA

LINC01669 non.epi.lncRNA

LINC01670 non.epi.lncRNA

LINC01671 non.epi.lncRNA

LINC01672 non.epi.lncRNA

LINC01673 non.epi.lncRNA

LINC01674 non.epi.lncRNA

LINC01675 non.epi.lncRNA

LINC01676 non.epi.lncRNA

LINC01677 non.epi.lncRNA

LINC01678 non.epi.lncRNA

LINC01679 non.epi.lncRNA

LINC01680 non.epi.lncRNA

LINC01681 non.epi.lncRNA

LINC01682 non.epi.lncRNA

LINC01683 non.epi.lncRNA

LINC01684 non.epi.lncRNA

LINC01685 non.epi.lncRNA

LINC01686 non.epi.lncRNA

LINC01687 non.epi.lncRNA

LINC01688 non.epi.lncRNA

LINC01689 non.epi.lncRNA

LINC01690 non.epi.lncRNA

LINC01691 non.epi.lncRNA

LINC01692 non.epi.lncRNA

LINC01694 non.epi.lncRNA

LINC01695 non.epi.lncRNA

LINC01696 non.epi.lncRNA

LINC01697 non.epi.lncRNA

LINC01698 non.epi.lncRNA

LINC01699 non.epi.lncRNA

LINC01700 non.epi.lncRNA

LINC01701 non.epi.lncRNA

LINC01702 non.epi.lncRNA

LINC01703 non.epi.lncRNA

LINC01705 non.epi.lncRNA

LINC01706 non.epi.lncRNA

LINC01707 non.epi.lncRNA

LINC01708 non.epi.lncRNA

LINC01709 non.epi.lncRNA

LINC01710 non.epi.lncRNA

LINC01711 non.epi.lncRNA

LINC01712 non.epi.lncRNA

LINC01713 non.epi.lncRNA

LINC01715 non.epi.lncRNA

LINC01716 non.epi.lncRNA

LINC01717 non.epi.lncRNA

LINC01718 non.epi.lncRNA

LINC01719 non.epi.lncRNA

LINC01720 non.epi.lncRNA

LINC01721 non.epi.lncRNA

LINC01722 non.epi.lncRNA

LINC01723 non.epi.lncRNA

LINC01724 non.epi.lncRNA

LINC01725 non.epi.lncRNA

LINC01726 non.epi.lncRNA

LINC01727 non.epi.lncRNA

LINC01728 non.epi.lncRNA

LINC01729 non.epi.lncRNA

LINC01732 non.epi.lncRNA

LINC01733 non.epi.lncRNA

LINC01734 non.epi.lncRNA

LINC01735 non.epi.lncRNA

LINC01736 non.epi.lncRNA

LINC01737 non.epi.lncRNA

LINC01739 non.epi.lncRNA

LINC01741 non.epi.lncRNA

LINC01742 non.epi.lncRNA

LINC01743 non.epi.lncRNA

LINC01744 non.epi.lncRNA

LINC01745 non.epi.lncRNA

LINC01746 non.epi.lncRNA

LINC01747 non.epi.lncRNA

LINC01748 non.epi.lncRNA

LINC01749 non.epi.lncRNA

LINC01751 non.epi.lncRNA

LINC01752 non.epi.lncRNA

LINC01753 non.epi.lncRNA

LINC01754 non.epi.lncRNA

LINC01755 non.epi.lncRNA

LINC01756 non.epi.lncRNA

LINC01757 non.epi.lncRNA

LINC01758 non.epi.lncRNA

LINC01761 non.epi.lncRNA

LINC01762 non.epi.lncRNA

LINC01763 non.epi.lncRNA

LINC01764 non.epi.lncRNA

LINC01765 non.epi.lncRNA

LINC01766 non.epi.lncRNA

LINC01767 non.epi.lncRNA

LINC01772 non.epi.lncRNA

LINC01774 non.epi.lncRNA

LINC01776 non.epi.lncRNA

LINC01777 non.epi.lncRNA

LINC01778 non.epi.lncRNA

LINC01779 non.epi.lncRNA

LINC01780 non.epi.lncRNA

LINC01781 non.epi.lncRNA

LINC01782 non.epi.lncRNA

LINC01784 non.epi.lncRNA

LINC01785 non.epi.lncRNA

LINC01787 non.epi.lncRNA

LINC01788 non.epi.lncRNA

LINC01789 non.epi.lncRNA

LINC01790 non.epi.lncRNA

LINC01791 non.epi.lncRNA

LINC01792 non.epi.lncRNA

LINC01793 non.epi.lncRNA

LINC01794 non.epi.lncRNA

LINC01796 non.epi.lncRNA

LINC01797 non.epi.lncRNA

LINC01798 non.epi.lncRNA

LINC01799 non.epi.lncRNA

LINC01801 non.epi.lncRNA

LINC01802 non.epi.lncRNA

LINC01803 non.epi.lncRNA

LINC01804 non.epi.lncRNA

LINC01805 non.epi.lncRNA

LINC01806 non.epi.lncRNA

LINC01807 non.epi.lncRNA

LINC01808 non.epi.lncRNA

LINC01809 non.epi.lncRNA

LINC01810 non.epi.lncRNA

LINC01811 non.epi.lncRNA

LINC01812 non.epi.lncRNA

LINC01813 non.epi.lncRNA

LINC01814 non.epi.lncRNA

LINC01815 non.epi.lncRNA

LINC01816 non.epi.lncRNA

LINC01817 non.epi.lncRNA

LINC01818 non.epi.lncRNA

LINC01819 non.epi.lncRNA

LINC01820 non.epi.lncRNA

LINC01821 non.epi.lncRNA

LINC01822 non.epi.lncRNA

LINC01823 non.epi.lncRNA

LINC01824 non.epi.lncRNA

LINC01825 non.epi.lncRNA

LINC01826 non.epi.lncRNA

LINC01828 non.epi.lncRNA

LINC01829 non.epi.lncRNA

LINC01831 non.epi.lncRNA

LINC01832 non.epi.lncRNA

LINC01833 non.epi.lncRNA

LINC01834 non.epi.lncRNA

LINC01837 non.epi.lncRNA

LINC01838 non.epi.lncRNA

LINC01840 non.epi.lncRNA

LINC01841 non.epi.lncRNA

LINC01842 non.epi.lncRNA

LINC01844 non.epi.lncRNA

LINC01846 non.epi.lncRNA

LINC01847 non.epi.lncRNA

LINC01848 non.epi.lncRNA

LINC01849 non.epi.lncRNA

LINC01850 non.epi.lncRNA

LINC01851 non.epi.lncRNA

LINC01852 non.epi.lncRNA

LINC01853 non.epi.lncRNA

LINC01855 non.epi.lncRNA

LINC01858 non.epi.lncRNA

LINC01859 non.epi.lncRNA

LINC01861 non.epi.lncRNA

LINC01863 non.epi.lncRNA

LINC01864 non.epi.lncRNA

LINC01865 non.epi.lncRNA

LINC01866 non.epi.lncRNA

LINC01867 non.epi.lncRNA

LINC01868 non.epi.lncRNA

LINC01870 non.epi.lncRNA

LINC01871 non.epi.lncRNA

LINC01872 non.epi.lncRNA

LINC01874 non.epi.lncRNA

LINC01875 non.epi.lncRNA

LINC01876 non.epi.lncRNA

LINC01877 non.epi.lncRNA

LINC01878 non.epi.lncRNA

LINC01879 non.epi.lncRNA

LINC01880 non.epi.lncRNA

LINC01883 non.epi.lncRNA

LINC01884 non.epi.lncRNA

LINC01885 non.epi.lncRNA

LINC01886 non.epi.lncRNA

LINC01887 non.epi.lncRNA

LINC01888 non.epi.lncRNA

LINC01889 non.epi.lncRNA

LINC01890 non.epi.lncRNA

LINC01891 non.epi.lncRNA

LINC01892 non.epi.lncRNA

LINC01893 non.epi.lncRNA

LINC01894 non.epi.lncRNA

LINC01896 non.epi.lncRNA

LINC01897 non.epi.lncRNA

LINC01898 non.epi.lncRNA

LINC01899 non.epi.lncRNA

LINC01900 non.epi.lncRNA

LINC01901 non.epi.lncRNA

LINC01903 non.epi.lncRNA

LINC01904 non.epi.lncRNA

LINC01905 non.epi.lncRNA

LINC01906 non.epi.lncRNA

LINC01907 non.epi.lncRNA

LINC01908 non.epi.lncRNA

LINC01911 non.epi.lncRNA

LINC01912 non.epi.lncRNA

LINC01913 non.epi.lncRNA

LINC01914 non.epi.lncRNA

LINC01915 non.epi.lncRNA

LINC01916 non.epi.lncRNA

LINC01917 non.epi.lncRNA

LINC01918 non.epi.lncRNA

LINC01919 non.epi.lncRNA

LINC01920 non.epi.lncRNA

LINC01921 non.epi.lncRNA

LINC01923 non.epi.lncRNA

LINC01924 non.epi.lncRNA

LINC01925 non.epi.lncRNA

LINC01928 non.epi.lncRNA

LINC01929 non.epi.lncRNA

LINC01931 non.epi.lncRNA

LINC01933 non.epi.lncRNA

LINC01934 non.epi.lncRNA

LINC01935 non.epi.lncRNA

LINC01936 non.epi.lncRNA

LINC01937 non.epi.lncRNA

LINC01938 non.epi.lncRNA

LINC01939 non.epi.lncRNA

LINC01940 non.epi.lncRNA

LINC01941 non.epi.lncRNA

LINC01942 non.epi.lncRNA

LINC01944 non.epi.lncRNA

LINC01945 non.epi.lncRNA

LINC01946 non.epi.lncRNA

LINC01947 non.epi.lncRNA

LINC01948 non.epi.lncRNA

LINC01949 non.epi.lncRNA

LINC01950 non.epi.lncRNA

LINC01951 non.epi.lncRNA

LINC01953 non.epi.lncRNA

LINC01954 non.epi.lncRNA

LINC01955 non.epi.lncRNA

LINC01956 non.epi.lncRNA

LINC01957 non.epi.lncRNA

LINC01960 non.epi.lncRNA

LINC01961 non.epi.lncRNA

LINC01964 non.epi.lncRNA

LINC01965 non.epi.lncRNA

LINC01966 non.epi.lncRNA

LINC01967 non.epi.lncRNA

LINC01968 non.epi.lncRNA

LINC01971 non.epi.lncRNA

LINC01973 non.epi.lncRNA

LINC01974 non.epi.lncRNA

LINC01975 non.epi.lncRNA

LINC01976 non.epi.lncRNA

LINC01977 non.epi.lncRNA

LINC01978 non.epi.lncRNA

LINC01979 non.epi.lncRNA

LINC01980 non.epi.lncRNA

LINC01981 non.epi.lncRNA

LINC01982 non.epi.lncRNA

LINC01985 non.epi.lncRNA

LINC01986 non.epi.lncRNA

LINC01987 non.epi.lncRNA

LINC01989 non.epi.lncRNA

LINC01990 non.epi.lncRNA

LINC01991 non.epi.lncRNA

LINC01992 non.epi.lncRNA

LINC01993 non.epi.lncRNA

LINC01994 non.epi.lncRNA

LINC01995 non.epi.lncRNA

LINC01996 non.epi.lncRNA

LINC01997 non.epi.lncRNA

LINC01998 non.epi.lncRNA

LINC01999 non.epi.lncRNA

LINC02000 non.epi.lncRNA

LINC02002 non.epi.lncRNA

LINC02005 non.epi.lncRNA

LINC02006 non.epi.lncRNA

LINC02008 non.epi.lncRNA

LINC02010 non.epi.lncRNA

LINC02011 non.epi.lncRNA

LINC02012 non.epi.lncRNA

LINC02013 non.epi.lncRNA

LINC02014 non.epi.lncRNA

LINC02015 non.epi.lncRNA

LINC02016 non.epi.lncRNA

LINC02017 non.epi.lncRNA

LINC02018 non.epi.lncRNA

LINC02020 non.epi.lncRNA

LINC02021 non.epi.lncRNA

LINC02022 non.epi.lncRNA

LINC02023 non.epi.lncRNA

LINC02024 non.epi.lncRNA

LINC02025 non.epi.lncRNA

LINC02027 non.epi.lncRNA

LINC02028 non.epi.lncRNA

LINC02030 non.epi.lncRNA

LINC02031 non.epi.lncRNA

LINC02032 non.epi.lncRNA

LINC02033 non.epi.lncRNA

LINC02035 non.epi.lncRNA

LINC02038 non.epi.lncRNA

LINC02039 non.epi.lncRNA

LINC02040 non.epi.lncRNA

LINC02041 non.epi.lncRNA

LINC02042 non.epi.lncRNA

LINC02043 non.epi.lncRNA

LINC02044 non.epi.lncRNA

LINC02045 non.epi.lncRNA

LINC02046 non.epi.lncRNA

LINC02047 non.epi.lncRNA

LINC02048 non.epi.lncRNA

LINC02049 non.epi.lncRNA

LINC02050 non.epi.lncRNA

LINC02051 non.epi.lncRNA

LINC02052 non.epi.lncRNA

LINC02053 non.epi.lncRNA

LINC02055 non.epi.lncRNA

LINC02056 non.epi.lncRNA

LINC02057 non.epi.lncRNA

LINC02058 non.epi.lncRNA

LINC02059 non.epi.lncRNA

LINC02060 non.epi.lncRNA

LINC02061 non.epi.lncRNA

LINC02062 non.epi.lncRNA

LINC02063 non.epi.lncRNA

LINC02064 non.epi.lncRNA

LINC02065 non.epi.lncRNA

LINC02066 non.epi.lncRNA

LINC02067 non.epi.lncRNA

LINC02068 non.epi.lncRNA

LINC02069 non.epi.lncRNA

LINC02070 non.epi.lncRNA

LINC02071 non.epi.lncRNA

LINC02073 non.epi.lncRNA

LINC02074 non.epi.lncRNA

LINC02075 non.epi.lncRNA

LINC02077 non.epi.lncRNA

LINC02078 non.epi.lncRNA

LINC02079 non.epi.lncRNA

LINC02080 non.epi.lncRNA

LINC02082 non.epi.lncRNA

LINC02084 non.epi.lncRNA

LINC02085 non.epi.lncRNA

LINC02086 non.epi.lncRNA

LINC02087 non.epi.lncRNA

LINC02088 non.epi.lncRNA

LINC02089 non.epi.lncRNA

LINC02090 non.epi.lncRNA

LINC02091 non.epi.lncRNA

LINC02093 non.epi.lncRNA

LINC02094 non.epi.lncRNA

LINC02096 non.epi.lncRNA

LINC02097 non.epi.lncRNA

LINC02098 non.epi.lncRNA

LINC02099 non.epi.lncRNA

LINC02100 non.epi.lncRNA

LINC02101 non.epi.lncRNA

LINC02102 non.epi.lncRNA

LINC02103 non.epi.lncRNA

LINC02104 non.epi.lncRNA

LINC02105 non.epi.lncRNA

LINC02106 non.epi.lncRNA

LINC02108 non.epi.lncRNA

LINC02109 non.epi.lncRNA

LINC02110 non.epi.lncRNA

LINC02112 non.epi.lncRNA

LINC02113 non.epi.lncRNA

LINC02114 non.epi.lncRNA

LINC02115 non.epi.lncRNA

LINC02116 non.epi.lncRNA

LINC02117 non.epi.lncRNA

LINC02118 non.epi.lncRNA

LINC02119 non.epi.lncRNA

LINC02120 non.epi.lncRNA

LINC02121 non.epi.lncRNA

LINC02122 non.epi.lncRNA

LINC02123 non.epi.lncRNA

LINC02125 non.epi.lncRNA

LINC02126 non.epi.lncRNA

LINC02127 non.epi.lncRNA

LINC02128 non.epi.lncRNA

LINC02129 non.epi.lncRNA

LINC02131 non.epi.lncRNA

LINC02132 non.epi.lncRNA

LINC02133 non.epi.lncRNA

LINC02134 non.epi.lncRNA

LINC02135 non.epi.lncRNA

LINC02136 non.epi.lncRNA

LINC02137 non.epi.lncRNA

LINC02138 non.epi.lncRNA

LINC02139 non.epi.lncRNA

LINC02140 non.epi.lncRNA

LINC02141 non.epi.lncRNA

LINC02142 non.epi.lncRNA

LINC02143 non.epi.lncRNA

LINC02144 non.epi.lncRNA

LINC02145 non.epi.lncRNA

LINC02146 non.epi.lncRNA

LINC02147 non.epi.lncRNA

LINC02148 non.epi.lncRNA

LINC02149 non.epi.lncRNA

LINC02150 non.epi.lncRNA

LINC02151 non.epi.lncRNA

LINC02152 non.epi.lncRNA

LINC02153 non.epi.lncRNA

LINC02154 non.epi.lncRNA

LINC02155 non.epi.lncRNA

LINC02156 non.epi.lncRNA

LINC02157 non.epi.lncRNA

LINC02158 non.epi.lncRNA

LINC02159 non.epi.lncRNA

LINC02160 non.epi.lncRNA

LINC02161 non.epi.lncRNA

LINC02162 non.epi.lncRNA

LINC02163 non.epi.lncRNA

LINC02164 non.epi.lncRNA

LINC02165 non.epi.lncRNA

LINC02166 non.epi.lncRNA

LINC02167 non.epi.lncRNA

LINC02168 non.epi.lncRNA

LINC02169 non.epi.lncRNA

LINC02171 non.epi.lncRNA

LINC02172 non.epi.lncRNA

LINC02173 non.epi.lncRNA

LINC02174 non.epi.lncRNA

LINC02175 non.epi.lncRNA

LINC02176 non.epi.lncRNA

LINC02177 non.epi.lncRNA

LINC02178 non.epi.lncRNA

LINC02179 non.epi.lncRNA

LINC02180 non.epi.lncRNA

LINC02181 non.epi.lncRNA

LINC02182 non.epi.lncRNA

LINC02184 non.epi.lncRNA

LINC02185 non.epi.lncRNA

LINC02186 non.epi.lncRNA

LINC02188 non.epi.lncRNA

LINC02189 non.epi.lncRNA

LINC02190 non.epi.lncRNA

LINC02191 non.epi.lncRNA

LINC02192 non.epi.lncRNA

LINC02193 non.epi.lncRNA

LINC02195 non.epi.lncRNA

LINC02196 non.epi.lncRNA

LINC02197 non.epi.lncRNA

LINC02198 non.epi.lncRNA

LINC02199 non.epi.lncRNA

LINC02200 non.epi.lncRNA

LINC02201 non.epi.lncRNA

LINC02202 non.epi.lncRNA

LINC02203 non.epi.lncRNA

LINC02206 non.epi.lncRNA

LINC02207 non.epi.lncRNA

LINC02208 non.epi.lncRNA

LINC02209 non.epi.lncRNA

LINC02211 non.epi.lncRNA

LINC02212 non.epi.lncRNA

LINC02213 non.epi.lncRNA

LINC02214 non.epi.lncRNA

LINC02215 non.epi.lncRNA

LINC02216 non.epi.lncRNA

LINC02217 non.epi.lncRNA

LINC02219 non.epi.lncRNA

LINC02220 non.epi.lncRNA

LINC02221 non.epi.lncRNA

LINC02222 non.epi.lncRNA

LINC02223 non.epi.lncRNA

LINC02224 non.epi.lncRNA

LINC02226 non.epi.lncRNA

LINC02227 non.epi.lncRNA

LINC02228 non.epi.lncRNA

LINC02229 non.epi.lncRNA

LINC02230 non.epi.lncRNA

LINC02231 non.epi.lncRNA

LINC02232 non.epi.lncRNA

LINC02233 non.epi.lncRNA

LINC02234 non.epi.lncRNA

LINC02235 non.epi.lncRNA

LINC02236 non.epi.lncRNA

LINC02237 non.epi.lncRNA

LINC02238 non.epi.lncRNA

LINC02239 non.epi.lncRNA

LINC02240 non.epi.lncRNA

LINC02241 non.epi.lncRNA

LINC02242 non.epi.lncRNA

LINC02244 non.epi.lncRNA

LINC02245 non.epi.lncRNA

LINC02246 non.epi.lncRNA

LINC02247 non.epi.lncRNA

LINC02248 non.epi.lncRNA

LINC02249 non.epi.lncRNA

LINC02250 non.epi.lncRNA

LINC02251 non.epi.lncRNA

LINC02252 non.epi.lncRNA

LINC02253 non.epi.lncRNA

LINC02254 non.epi.lncRNA

LINC02256 non.epi.lncRNA

LINC02257 non.epi.lncRNA

LINC02258 non.epi.lncRNA

LINC02259 non.epi.lncRNA

LINC02260 non.epi.lncRNA

LINC02261 non.epi.lncRNA

LINC02262 non.epi.lncRNA

LINC02263 non.epi.lncRNA

LINC02264 non.epi.lncRNA

LINC02266 non.epi.lncRNA

LINC02267 non.epi.lncRNA

LINC02268 non.epi.lncRNA

LINC02269 non.epi.lncRNA

LINC02270 non.epi.lncRNA

LINC02271 non.epi.lncRNA

LINC02272 non.epi.lncRNA

LINC02273 non.epi.lncRNA

LINC02274 non.epi.lncRNA

LINC02275 non.epi.lncRNA

LINC02276 non.epi.lncRNA

LINC02277 non.epi.lncRNA

LINC02278 non.epi.lncRNA

LINC02279 non.epi.lncRNA

LINC02281 non.epi.lncRNA

LINC02282 non.epi.lncRNA

LINC02283 non.epi.lncRNA

LINC02285 non.epi.lncRNA

LINC02286 non.epi.lncRNA

LINC02287 non.epi.lncRNA

LINC02288 non.epi.lncRNA

LINC02290 non.epi.lncRNA

LINC02291 non.epi.lncRNA

LINC02292 non.epi.lncRNA

LINC02293 non.epi.lncRNA

LINC02294 non.epi.lncRNA

LINC02295 non.epi.lncRNA

LINC02296 non.epi.lncRNA

LINC02297 non.epi.lncRNA

LINC02298 non.epi.lncRNA

LINC02299 non.epi.lncRNA

LINC02300 non.epi.lncRNA

LINC02301 non.epi.lncRNA

LINC02302 non.epi.lncRNA

LINC02303 non.epi.lncRNA

LINC02304 non.epi.lncRNA

LINC02305 non.epi.lncRNA

LINC02306 non.epi.lncRNA

LINC02307 non.epi.lncRNA

LINC02309 non.epi.lncRNA

LINC02311 non.epi.lncRNA

LINC02312 non.epi.lncRNA

LINC02313 non.epi.lncRNA

LINC02314 non.epi.lncRNA

LINC02315 non.epi.lncRNA

LINC02316 non.epi.lncRNA

LINC02317 non.epi.lncRNA

LINC02318 non.epi.lncRNA

LINC02319 non.epi.lncRNA

LINC02320 non.epi.lncRNA

LINC02321 non.epi.lncRNA

LINC02322 non.epi.lncRNA

LINC02324 non.epi.lncRNA

LINC02325 non.epi.lncRNA

LINC02326 non.epi.lncRNA

LINC02327 non.epi.lncRNA

LINC02328 non.epi.lncRNA

LINC02329 non.epi.lncRNA

LINC02330 non.epi.lncRNA

LINC02331 non.epi.lncRNA

LINC02332 non.epi.lncRNA

LINC02333 non.epi.lncRNA

LINC02334 non.epi.lncRNA

LINC02335 non.epi.lncRNA

LINC02336 non.epi.lncRNA

LINC02337 non.epi.lncRNA

LINC02338 non.epi.lncRNA

LINC02339 non.epi.lncRNA

LINC02340 non.epi.lncRNA

LINC02342 non.epi.lncRNA

LINC02343 non.epi.lncRNA

LINC02344 non.epi.lncRNA

LINC02345 non.epi.lncRNA

LINC02346 non.epi.lncRNA

LINC02347 non.epi.lncRNA

LINC02349 non.epi.lncRNA

LINC02351 non.epi.lncRNA

LINC02353 non.epi.lncRNA

LINC02354 non.epi.lncRNA

LINC02355 non.epi.lncRNA

LINC02358 non.epi.lncRNA

LINC02360 non.epi.lncRNA

LINC02361 non.epi.lncRNA

LINC02363 non.epi.lncRNA

LINC02364 non.epi.lncRNA

LINC02365 non.epi.lncRNA

LINC02366 non.epi.lncRNA

LINC02367 non.epi.lncRNA

LINC02368 non.epi.lncRNA

LINC02369 non.epi.lncRNA

LINC02370 non.epi.lncRNA

LINC02372 non.epi.lncRNA

LINC02373 non.epi.lncRNA

LINC02374 non.epi.lncRNA

LINC02375 non.epi.lncRNA

LINC02376 non.epi.lncRNA

LINC02377 non.epi.lncRNA

LINC02378 non.epi.lncRNA

LINC02379 non.epi.lncRNA

LINC02380 non.epi.lncRNA

LINC02382 non.epi.lncRNA

LINC02383 non.epi.lncRNA

LINC02384 non.epi.lncRNA

LINC02386 non.epi.lncRNA

LINC02388 non.epi.lncRNA

LINC02389 non.epi.lncRNA

LINC02390 non.epi.lncRNA

LINC02391 non.epi.lncRNA

LINC02392 non.epi.lncRNA

LINC02393 non.epi.lncRNA

LINC02394 non.epi.lncRNA

LINC02395 non.epi.lncRNA

LINC02396 non.epi.lncRNA

LINC02397 non.epi.lncRNA

LINC02398 non.epi.lncRNA

LINC02400 non.epi.lncRNA

LINC02401 non.epi.lncRNA

LINC02402 non.epi.lncRNA

LINC02403 non.epi.lncRNA

LINC02404 non.epi.lncRNA

LINC02405 non.epi.lncRNA

LINC02406 non.epi.lncRNA

LINC02408 non.epi.lncRNA

LINC02409 non.epi.lncRNA

LINC02410 non.epi.lncRNA

LINC02411 non.epi.lncRNA

LINC02412 non.epi.lncRNA

LINC02413 non.epi.lncRNA

LINC02414 non.epi.lncRNA

LINC02415 non.epi.lncRNA

LINC02416 non.epi.lncRNA

LINC02417 non.epi.lncRNA

LINC02418 non.epi.lncRNA

LINC02419 non.epi.lncRNA

LINC02420 non.epi.lncRNA

LINC02421 non.epi.lncRNA

LINC02422 non.epi.lncRNA

LINC02423 non.epi.lncRNA

LINC02424 non.epi.lncRNA

LINC02425 non.epi.lncRNA

LINC02426 non.epi.lncRNA

LINC02427 non.epi.lncRNA

LINC02428 non.epi.lncRNA

LINC02429 non.epi.lncRNA

LINC02430 non.epi.lncRNA

LINC02431 non.epi.lncRNA

LINC02432 non.epi.lncRNA

LINC02433 non.epi.lncRNA

LINC02434 non.epi.lncRNA

LINC02435 non.epi.lncRNA

LINC02436 non.epi.lncRNA

LINC02437 non.epi.lncRNA

LINC02438 non.epi.lncRNA

LINC02439 non.epi.lncRNA

LINC02440 non.epi.lncRNA

LINC02441 non.epi.lncRNA

LINC02442 non.epi.lncRNA

LINC02443 non.epi.lncRNA

LINC02444 non.epi.lncRNA

LINC02445 non.epi.lncRNA

LINC02446 non.epi.lncRNA

LINC02447 non.epi.lncRNA

LINC02448 non.epi.lncRNA

LINC02449 non.epi.lncRNA

LINC02451 non.epi.lncRNA

LINC02457 non.epi.lncRNA

LINC02458 non.epi.lncRNA

LINC02459 non.epi.lncRNA

LINC02460 non.epi.lncRNA

LINC02461 non.epi.lncRNA

LINC02462 non.epi.lncRNA

LINC02463 non.epi.lncRNA

LINC02464 non.epi.lncRNA

LINC02465 non.epi.lncRNA

LINC02466 non.epi.lncRNA

LINC02468 non.epi.lncRNA

LINC02469 non.epi.lncRNA

LINC02470 non.epi.lncRNA

LINC02471 non.epi.lncRNA

LINC02472 non.epi.lncRNA

LINC02473 non.epi.lncRNA

LINC02474 non.epi.lncRNA

LINC02476 non.epi.lncRNA

LINC02477 non.epi.lncRNA

LINC02479 non.epi.lncRNA

LINC02480 non.epi.lncRNA

LINC02482 non.epi.lncRNA

LINC02483 non.epi.lncRNA

LINC02484 non.epi.lncRNA

LINC02485 non.epi.lncRNA

LINC02487 non.epi.lncRNA

LINC02488 non.epi.lncRNA

LINC02490 non.epi.lncRNA

LINC02492 non.epi.lncRNA

LINC02493 non.epi.lncRNA

LINC02494 non.epi.lncRNA

LINC02496 non.epi.lncRNA

LINC02497 non.epi.lncRNA

LINC02499 non.epi.lncRNA

LINC02500 non.epi.lncRNA

LINC02501 non.epi.lncRNA

LINC02502 non.epi.lncRNA

LINC02503 non.epi.lncRNA

LINC02504 non.epi.lncRNA

LINC02505 non.epi.lncRNA

LINC02506 non.epi.lncRNA

LINC02507 non.epi.lncRNA

LINC02508 non.epi.lncRNA

LINC02509 non.epi.lncRNA

LINC02510 non.epi.lncRNA

LINC02511 non.epi.lncRNA

LINC02512 non.epi.lncRNA

LINC02513 non.epi.lncRNA

LINC02514 non.epi.lncRNA

LINC02515 non.epi.lncRNA

LINC02516 non.epi.lncRNA

LINC02517 non.epi.lncRNA

LINC02518 non.epi.lncRNA

LINC02519 non.epi.lncRNA

LINC02520 non.epi.lncRNA

LINC02522 non.epi.lncRNA

LINC02523 non.epi.lncRNA

LINC02525 non.epi.lncRNA

LINC02526 non.epi.lncRNA

LINC02527 non.epi.lncRNA

LINC02529 non.epi.lncRNA

LINC02530 non.epi.lncRNA

LINC02531 non.epi.lncRNA

LINC02532 non.epi.lncRNA

LINC02533 non.epi.lncRNA

LINC02534 non.epi.lncRNA

LINC02535 non.epi.lncRNA

LINC02536 non.epi.lncRNA

LINC02538 non.epi.lncRNA

LINC02540 non.epi.lncRNA

LINC02541 non.epi.lncRNA

LINC02542 non.epi.lncRNA

LINC02543 non.epi.lncRNA

LINC02544 non.epi.lncRNA

LINC02545 non.epi.lncRNA

LINC02546 non.epi.lncRNA

LINC02547 non.epi.lncRNA

LINC02548 non.epi.lncRNA

LINC02549 non.epi.lncRNA

LINC02550 non.epi.lncRNA

LINC02552 non.epi.lncRNA

LINC02553 non.epi.lncRNA

LINC02554 non.epi.lncRNA

LINC02556 non.epi.lncRNA

LINC02557 non.epi.lncRNA

LINC02558 non.epi.lncRNA

LINC02559 non.epi.lncRNA

LINC02561 non.epi.lncRNA

LINC02562 non.epi.lncRNA

LINC02563 non.epi.lncRNA

LINC02564 non.epi.lncRNA

LINC02565 non.epi.lncRNA

LINC02567 non.epi.lncRNA

LINC02568 non.epi.lncRNA

LINC02569 non.epi.lncRNA

LINC02572 non.epi.lncRNA

LINC02573 non.epi.lncRNA

LINC02576 non.epi.lncRNA

LINC02577 non.epi.lncRNA

LINC02578 non.epi.lncRNA

LINC02579 non.epi.lncRNA

LINC02582 non.epi.lncRNA

LINC02584 non.epi.lncRNA

LINC02586 non.epi.lncRNA

LINC02587 non.epi.lncRNA

LINC02588 non.epi.lncRNA

LINC02590 non.epi.lncRNA

LINC02591 non.epi.lncRNA

LINC02594 non.epi.lncRNA

LINC02595 non.epi.lncRNA

LINC02596 non.epi.lncRNA

LINC02599 non.epi.lncRNA

LINC02600 non.epi.lncRNA

LINC02603 non.epi.lncRNA

LINC02604 non.epi.lncRNA

LINC02605 non.epi.lncRNA

LINC02607 non.epi.lncRNA

LINC02608 non.epi.lncRNA

LINC02609 non.epi.lncRNA

LINC02610 non.epi.lncRNA

LINC02611 non.epi.lncRNA

LINC02612 non.epi.lncRNA

LINC02613 non.epi.lncRNA

LINC02614 non.epi.lncRNA

LINC02615 non.epi.lncRNA

LINC02616 non.epi.lncRNA

LINC02617 non.epi.lncRNA

LINC02619 non.epi.lncRNA

LINC02620 non.epi.lncRNA

LINC02621 non.epi.lncRNA

LINC02622 non.epi.lncRNA

LINC02623 non.epi.lncRNA

LINC02624 non.epi.lncRNA

LINC02626 non.epi.lncRNA

LINC02627 non.epi.lncRNA

LINC02628 non.epi.lncRNA

LINC02629 non.epi.lncRNA

LINC02630 non.epi.lncRNA

LINC02631 non.epi.lncRNA

LINC02632 non.epi.lncRNA

LINC02633 non.epi.lncRNA

LINC02634 non.epi.lncRNA

LINC02637 non.epi.lncRNA

LINC02640 non.epi.lncRNA

LINC02641 non.epi.lncRNA

LINC02643 non.epi.lncRNA

LINC02644 non.epi.lncRNA

LINC02645 non.epi.lncRNA

LINC02646 non.epi.lncRNA

LINC02647 non.epi.lncRNA

LINC02648 non.epi.lncRNA

LINC02649 non.epi.lncRNA

LINC02650 non.epi.lncRNA

LINC02651 non.epi.lncRNA

LINC02652 non.epi.lncRNA

LINC02653 non.epi.lncRNA

LINC02654 non.epi.lncRNA

LINC02655 non.epi.lncRNA

LINC02656 non.epi.lncRNA

LINC02657 non.epi.lncRNA

LINC02658 non.epi.lncRNA

LINC02659 non.epi.lncRNA

LINC02660 non.epi.lncRNA

LINC02661 non.epi.lncRNA

LINC02662 non.epi.lncRNA

LINC02663 non.epi.lncRNA

LINC02664 non.epi.lncRNA

LINC02665 non.epi.lncRNA

LINC02667 non.epi.lncRNA

LINC02668 non.epi.lncRNA

LINC02669 non.epi.lncRNA

LINC02670 non.epi.lncRNA

LINC02671 non.epi.lncRNA

LINC02672 non.epi.lncRNA

LINC02673 non.epi.lncRNA

LINC02674 non.epi.lncRNA

LINC02675 non.epi.lncRNA

LINC02676 non.epi.lncRNA

LINC02677 non.epi.lncRNA

LINC02678 non.epi.lncRNA

LINC02679 non.epi.lncRNA

LINC02681 non.epi.lncRNA

LINC02682 non.epi.lncRNA

LINC02684 non.epi.lncRNA

LINC02685 non.epi.lncRNA

LINC02686 non.epi.lncRNA

LINC02687 non.epi.lncRNA

LINC02688 non.epi.lncRNA

LINC02691 non.epi.lncRNA

LINC02692 non.epi.lncRNA

LINC02693 non.epi.lncRNA

LINC02694 non.epi.lncRNA

LINC02695 non.epi.lncRNA

LINC02696 non.epi.lncRNA

LINC02698 non.epi.lncRNA

LINC02699 non.epi.lncRNA

LINC02702 non.epi.lncRNA

LINC02703 non.epi.lncRNA

LINC02704 non.epi.lncRNA

LINC02705 non.epi.lncRNA

LINC02706 non.epi.lncRNA

LINC02708 non.epi.lncRNA

LINC02711 non.epi.lncRNA

LINC02712 non.epi.lncRNA

LINC02713 non.epi.lncRNA

LINC02714 non.epi.lncRNA

LINC02715 non.epi.lncRNA

LINC02716 non.epi.lncRNA

LINC02718 non.epi.lncRNA

LINC02719 non.epi.lncRNA

LINC02720 non.epi.lncRNA

LINC02721 non.epi.lncRNA

LINC02722 non.epi.lncRNA

LINC02723 non.epi.lncRNA

LINC02724 non.epi.lncRNA

LINC02726 non.epi.lncRNA

LINC02727 non.epi.lncRNA

LINC02728 non.epi.lncRNA

LINC02729 non.epi.lncRNA

LINC02731 non.epi.lncRNA

LINC02732 non.epi.lncRNA

LINC02733 non.epi.lncRNA

LINC02734 non.epi.lncRNA

LINC02735 non.epi.lncRNA

LINC02737 non.epi.lncRNA

LINC02739 non.epi.lncRNA

LINC02740 non.epi.lncRNA

LINC02741 non.epi.lncRNA

LINC02742 non.epi.lncRNA

LINC02743 non.epi.lncRNA

LINC02744 non.epi.lncRNA

LINC02745 non.epi.lncRNA

LINC02747 non.epi.lncRNA

LINC02748 non.epi.lncRNA

LINC02749 non.epi.lncRNA

LINC02750 non.epi.lncRNA

LINC02751 non.epi.lncRNA

LINC02752 non.epi.lncRNA

LINC02753 non.epi.lncRNA

LINC02754 non.epi.lncRNA

LINC02755 non.epi.lncRNA

LINC02756 non.epi.lncRNA

LINC02758 non.epi.lncRNA

LINC02759 non.epi.lncRNA

LINC02760 non.epi.lncRNA

LINC02762 non.epi.lncRNA

LINC02763 non.epi.lncRNA

LINC02764 non.epi.lncRNA

LINC02765 non.epi.lncRNA

LINC02766 non.epi.lncRNA

LINC02769 non.epi.lncRNA

LINC02770 non.epi.lncRNA

LINC02772 non.epi.lncRNA

LINC02774 non.epi.lncRNA

LINC02776 non.epi.lncRNA

LINC02777 non.epi.lncRNA

LINC02778 non.epi.lncRNA

LINC02779 non.epi.lncRNA

LINC02782 non.epi.lncRNA

LINC02784 non.epi.lncRNA

LINC02785 non.epi.lncRNA

LINC02787 non.epi.lncRNA

LINC02789 non.epi.lncRNA

LINC02790 non.epi.lncRNA

LINC02791 non.epi.lncRNA

LINC02794 non.epi.lncRNA

LINC02796 non.epi.lncRNA

LINC02798 non.epi.lncRNA

LINC02799 non.epi.lncRNA

LINC02800 non.epi.lncRNA

LINC02802 non.epi.lncRNA

LINC02803 non.epi.lncRNA

LINC02804 non.epi.lncRNA

LINC02805 non.epi.lncRNA

LINC02806 non.epi.lncRNA

LINC02809 non.epi.lncRNA

LINC02810 non.epi.lncRNA

LINC02812 non.epi.lncRNA

LINC02814 non.epi.lncRNA

LINC02816 non.epi.lncRNA

LINC02819 non.epi.lncRNA

LINC02820 non.epi.lncRNA

LINC02821 non.epi.lncRNA

LINC02823 non.epi.lncRNA

LINC02824 non.epi.lncRNA

LINC02825 non.epi.lncRNA

LINC02826 non.epi.lncRNA

LINC02827 non.epi.lncRNA

LINC02828 non.epi.lncRNA

LINC02829 non.epi.lncRNA

LINC02830 non.epi.lncRNA

LINC02831 non.epi.lncRNA

LINC02832 non.epi.lncRNA

LINC02833 non.epi.lncRNA

LINC02836 non.epi.lncRNA

LINC02837 non.epi.lncRNA

LINC02839 non.epi.lncRNA

LINC02840 non.epi.lncRNA

LINC02841 non.epi.lncRNA

LINC02842 non.epi.lncRNA

LINC02843 non.epi.lncRNA

LINC02844 non.epi.lncRNA

LINC02845 non.epi.lncRNA

LINC02846 non.epi.lncRNA

LINC02847 non.epi.lncRNA

LINC02850 non.epi.lncRNA

LINC02851 non.epi.lncRNA

LINC02853 non.epi.lncRNA

LINC02854 non.epi.lncRNA

LINC02855 non.epi.lncRNA

LINC02857 non.epi.lncRNA

LINC02860 non.epi.lncRNA

LINC2194 non.epi.lncRNA

LINGO1-AS2 non.epi.lncRNA

LINP1 non.epi.lncRNA

LIPC-AS1 non.epi.lncRNA

LIPE-AS1 non.epi.lncRNA

LIX1-AS1 non.epi.lncRNA

LMCD1-AS1 non.epi.lncRNA

LMF1-AS1 non.epi.lncRNA

LMLN-AS1 non.epi.lncRNA

LMNB1-DT non.epi.lncRNA

LMO7-AS1 non.epi.lncRNA

LMO7DN-IT1 non.epi.lncRNA

LNCAROD non.epi.lncRNA

LNCARSR non.epi.lncRNA

LNCNEF non.epi.lncRNA

LNCOC1 non.epi.lncRNA

LNCOG non.epi.lncRNA

LNCPRESS1 non.epi.lncRNA

LNCPRESS2 non.epi.lncRNA

LNCSRLR non.epi.lncRNA

LNX1-AS1 non.epi.lncRNA

LNX1-AS2 non.epi.lncRNA

LOH12CR2 non.epi.lncRNA

LPGAT1-AS1 non.epi.lncRNA

LPP-AS1 non.epi.lncRNA

LRIG2-DT non.epi.lncRNA

LRP1-AS non.epi.lncRNA

LRP4-AS1 non.epi.lncRNA

LRRC2-AS1 non.epi.lncRNA

LRRC3-DT non.epi.lncRNA

LRRC52-AS1 non.epi.lncRNA

LRRC8C-DT non.epi.lncRNA

LSAMP-AS1 non.epi.lncRNA

LSINCT5 non.epi.lncRNA

LUADT1 non.epi.lncRNA

LUCAT1 non.epi.lncRNA

LUNAR1 non.epi.lncRNA

LURAP1L-AS1 non.epi.lncRNA

LY6E-DT non.epi.lncRNA

LY86-AS1 non.epi.lncRNA

LYPLAL1-AS1 non.epi.lncRNA

LYPLAL1-DT non.epi.lncRNA

LYST-AS1 non.epi.lncRNA

LZTS1-AS1 non.epi.lncRNA

MACC1-AS1 non.epi.lncRNA

MACORIS non.epi.lncRNA

MACROD2-AS1 non.epi.lncRNA

MACROD2-IT1 non.epi.lncRNA

MAFA-AS1 non.epi.lncRNA

MAFTRR non.epi.lncRNA

MAGEA10-MAGEA5 non.epi.lncRNA

MAGEA4-AS1 non.epi.lncRNA

MAGEA8-AS1 non.epi.lncRNA

MAGI1-AS1 non.epi.lncRNA

MAGI1-IT1 non.epi.lncRNA

MAGI2-AS1 non.epi.lncRNA

MAGI2-AS2 non.epi.lncRNA

MAL2-AS1 non.epi.lncRNA

MALINC1 non.epi.lncRNA

MAMDC2-AS1 non.epi.lncRNA

MANCR non.epi.lncRNA

MANEA-DT non.epi.lncRNA

MAP3K14-AS1 non.epi.lncRNA

MAP3K20-AS1 non.epi.lncRNA

MAP4K3-DT non.epi.lncRNA

MAPKAPK5-AS1 non.epi.lncRNA

MAPT-AS1 non.epi.lncRNA

MAPT-IT1 non.epi.lncRNA

MARCH10-DT non.epi.lncRNA

MAST4-IT1 non.epi.lncRNA

MBNL1-AS1 non.epi.lncRNA

MCCC1-AS1 non.epi.lncRNA

MCF2L-AS1 non.epi.lncRNA

MCHR2-AS1 non.epi.lncRNA

MCM3AP-AS1 non.epi.lncRNA

MCPH1-AS1 non.epi.lncRNA

MCTP1-AS1 non.epi.lncRNA

MDC1-AS1 non.epi.lncRNA

MDS2 non.epi.lncRNA

MED14OS non.epi.lncRNA

MED4-AS1 non.epi.lncRNA

MEF2C-AS1 non.epi.lncRNA

MEF2C-AS2 non.epi.lncRNA

MEG3 non.epi.lncRNA

MEG8 non.epi.lncRNA

MEIS1-AS2 non.epi.lncRNA

MEIS1-AS3 non.epi.lncRNA

MGAT3-AS1 non.epi.lncRNA

MHENCR non.epi.lncRNA

MIAT non.epi.lncRNA

MIATNB non.epi.lncRNA

MID1IP1-AS1 non.epi.lncRNA

MIMT1 non.epi.lncRNA

MIR1-1HG non.epi.lncRNA

MIR1-1HG-AS1 non.epi.lncRNA

MIR100HG non.epi.lncRNA

MIR122HG non.epi.lncRNA

MIR124-2HG non.epi.lncRNA

MIR1302-2HG non.epi.lncRNA

MIR133A1HG non.epi.lncRNA

MIR137HG non.epi.lncRNA

MIR17HG non.epi.lncRNA

MIR181A1HG non.epi.lncRNA

MIR181A2HG non.epi.lncRNA

MIR193BHG non.epi.lncRNA

MIR194-2HG non.epi.lncRNA

MIR200CHG non.epi.lncRNA

MIR202HG non.epi.lncRNA

MIR2052HG non.epi.lncRNA

MIR205HG non.epi.lncRNA

MIR2117HG non.epi.lncRNA

MIR217HG non.epi.lncRNA

MIR222HG non.epi.lncRNA

MIR29B2CHG non.epi.lncRNA

MIR3150BHG non.epi.lncRNA

MIR31HG non.epi.lncRNA

MIR34AHG non.epi.lncRNA

MIR3659HG non.epi.lncRNA

MIR3663HG non.epi.lncRNA

MIR3681HG non.epi.lncRNA

MIR378D2HG non.epi.lncRNA

MIR381HG non.epi.lncRNA

MIR3936HG non.epi.lncRNA

MIR3976HG non.epi.lncRNA

MIR4290HG non.epi.lncRNA

MIR4300HG non.epi.lncRNA

MIR4307HG non.epi.lncRNA

MIR4422HG non.epi.lncRNA

MIR4435-2HG non.epi.lncRNA

MIR4453HG non.epi.lncRNA

MIR4458HG non.epi.lncRNA

MIR4500HG non.epi.lncRNA

MIR4527HG non.epi.lncRNA

MIR4713HG non.epi.lncRNA

MIR503HG non.epi.lncRNA

MIR548XHG non.epi.lncRNA

MIR5689HG non.epi.lncRNA

MIR583HG non.epi.lncRNA

MIR600HG non.epi.lncRNA

MIR646HG non.epi.lncRNA

MIR663AHG non.epi.lncRNA

MIR670HG non.epi.lncRNA

MIR7-3HG non.epi.lncRNA

MIR7515HG non.epi.lncRNA

MIR762HG non.epi.lncRNA

MIR9-3HG non.epi.lncRNA

MIR924HG non.epi.lncRNA

MIR99AHG non.epi.lncRNA

MIRLET7BHG non.epi.lncRNA

MKNK1-AS1 non.epi.lncRNA

MLIP-AS1 non.epi.lncRNA

MLIP-IT1 non.epi.lncRNA

MME-AS1 non.epi.lncRNA

MMP2-AS1 non.epi.lncRNA

MMP25-AS1 non.epi.lncRNA

MNX1-AS2 non.epi.lncRNA

MORC1-AS1 non.epi.lncRNA

MORC2-AS1 non.epi.lncRNA

MPPED2-AS1 non.epi.lncRNA

MPRIP-AS1 non.epi.lncRNA

MRGPRF-AS1 non.epi.lncRNA

MRGPRG-AS1 non.epi.lncRNA

MRPL23-AS1 non.epi.lncRNA

MRTFA-AS1 non.epi.lncRNA

MSC-AS1 non.epi.lncRNA

MTOR-AS1 non.epi.lncRNA

MTUS2-AS1 non.epi.lncRNA

MTUS2-AS2 non.epi.lncRNA

MUC20-OT1 non.epi.lncRNA

MUC5B-AS1 non.epi.lncRNA

MYB-AS1 non.epi.lncRNA

MYCBP2-AS1 non.epi.lncRNA

MYCBP2-AS2 non.epi.lncRNA

MYCNOS non.epi.lncRNA

MYCNUT non.epi.lncRNA

MYHAS non.epi.lncRNA

MYLK-AS1 non.epi.lncRNA

MYLK-AS2 non.epi.lncRNA

MYO16-AS1 non.epi.lncRNA

MYO16-AS2 non.epi.lncRNA

MYO3B-AS1 non.epi.lncRNA

MYOSLID non.epi.lncRNA

MYRF-AS1 non.epi.lncRNA

MYT1L-AS1 non.epi.lncRNA

MZF1-AS1 non.epi.lncRNA

NAALADL2-AS1 non.epi.lncRNA

NAALADL2-AS3 non.epi.lncRNA

NADK2-AS1 non.epi.lncRNA

NAGPA-AS1 non.epi.lncRNA

NALCN-AS1 non.epi.lncRNA

NALT1 non.epi.lncRNA

NAMA non.epi.lncRNA

NANOGP11 non.epi.lncRNA

NAPA-AS1 non.epi.lncRNA

NARF-AS1 non.epi.lncRNA

NARF-IT1 non.epi.lncRNA

NAV2-AS1 non.epi.lncRNA

NAV2-AS2 non.epi.lncRNA

NAV2-AS3 non.epi.lncRNA

NAV2-AS5 non.epi.lncRNA

NAV2-IT1 non.epi.lncRNA

NBAT1 non.epi.lncRNA

NBR2 non.epi.lncRNA

NCAM1-AS1 non.epi.lncRNA

NCF4-AS1 non.epi.lncRNA

NCK1-DT non.epi.lncRNA

NCKAP5-AS1 non.epi.lncRNA

NCKAP5-AS2 non.epi.lncRNA

NCKAP5-IT1 non.epi.lncRNA

NCMAP-DT non.epi.lncRNA

NCOA7-AS1 non.epi.lncRNA

NDFIP2-AS1 non.epi.lncRNA

NDP-AS1 non.epi.lncRNA

NDST1-AS1 non.epi.lncRNA

NDUFA6-DT non.epi.lncRNA

NDUFV2-AS1 non.epi.lncRNA

NEAT1 non.epi.lncRNA

NECTIN1-AS1 non.epi.lncRNA

NECTIN3-AS1 non.epi.lncRNA

NEGR1-IT1 non.epi.lncRNA

NEURL1-AS1 non.epi.lncRNA

NEXN-AS1 non.epi.lncRNA

NFIA-AS1 non.epi.lncRNA

NFIA-AS2 non.epi.lncRNA

NFYC-AS1 non.epi.lncRNA

NGF-AS1 non.epi.lncRNA

NHS-AS1 non.epi.lncRNA

NIFK-AS1 non.epi.lncRNA

NKAIN3-IT1 non.epi.lncRNA

NKX2-1-AS1 non.epi.lncRNA

NKX2-2-AS1 non.epi.lncRNA

NLGN1-AS1 non.epi.lncRNA

NLGN4Y-AS1 non.epi.lncRNA

NMBR-AS1 non.epi.lncRNA

NNT-AS1 non.epi.lncRNA

NOL4L-DT non.epi.lncRNA

NOP14-AS1 non.epi.lncRNA

NOP53-AS1 non.epi.lncRNA

NPHP3-AS1 non.epi.lncRNA

NPSR1-AS1 non.epi.lncRNA

NPTN-IT1 non.epi.lncRNA

NR2F1-AS1 non.epi.lncRNA

NR2F2-AS1 non.epi.lncRNA

NRAD1 non.epi.lncRNA

NRAV non.epi.lncRNA

NREP-AS1 non.epi.lncRNA

NRG1-IT1 non.epi.lncRNA

NRG1-IT3 non.epi.lncRNA

NRG3-AS1 non.epi.lncRNA

NRIR non.epi.lncRNA

NTM-AS1 non.epi.lncRNA

NTM-IT non.epi.lncRNA

NTRK3-AS1 non.epi.lncRNA

NUCB1-AS1 non.epi.lncRNA

NUP50-DT non.epi.lncRNA

NUTM2A-AS1 non.epi.lncRNA

NUTM2B-AS1 non.epi.lncRNA

OBI1-AS1 non.epi.lncRNA

ODC1-DT non.epi.lncRNA

ODF2-AS1 non.epi.lncRNA

OIP5-AS1 non.epi.lncRNA

OLMALINC non.epi.lncRNA

OOEP-AS1 non.epi.lncRNA

OPCML-IT1 non.epi.lncRNA

OPCML-IT2 non.epi.lncRNA

OR7E11P non.epi.lncRNA

OSBPL10-AS1 non.epi.lncRNA

OSER1-DT non.epi.lncRNA

OSTM1-AS1 non.epi.lncRNA

OSTN-AS1 non.epi.lncRNA

OTX2-AS1 non.epi.lncRNA

OVAAL non.epi.lncRNA

OVCH1-AS1 non.epi.lncRNA

OVOL1-AS1 non.epi.lncRNA

OXCT1-AS1 non.epi.lncRNA

P4HA2-AS1 non.epi.lncRNA

P4HA3-AS1 non.epi.lncRNA

PABPC1L2B-AS1 non.epi.lncRNA

PABPC4-AS1 non.epi.lncRNA

PABPC5-AS1 non.epi.lncRNA

PACRG-AS2 non.epi.lncRNA

PANCR non.epi.lncRNA

PANDAR non.epi.lncRNA

PANTR1 non.epi.lncRNA

PAPPA-AS1 non.epi.lncRNA

PAPPA-AS2 non.epi.lncRNA

PAQR9-AS1 non.epi.lncRNA

PARAL1 non.epi.lncRNA

PARD6G-AS1 non.epi.lncRNA

PART1 non.epi.lncRNA

PAUPAR non.epi.lncRNA

PAX8-AS1 non.epi.lncRNA

PAXIP1-AS2 non.epi.lncRNA

PCA3 non.epi.lncRNA

PCAT1 non.epi.lncRNA

PCAT18 non.epi.lncRNA

PCAT19 non.epi.lncRNA

PCAT4 non.epi.lncRNA

PCAT5 non.epi.lncRNA

PCAT6 non.epi.lncRNA

PCAT7 non.epi.lncRNA

PCBP1-AS1 non.epi.lncRNA

PCCA-AS1 non.epi.lncRNA

PCDH9-AS1 non.epi.lncRNA

PCDH9-AS2 non.epi.lncRNA

PCDH9-AS3 non.epi.lncRNA

PCDH9-AS4 non.epi.lncRNA

PCED1B-AS1 non.epi.lncRNA

PCF11-AS1 non.epi.lncRNA

PCGEM1 non.epi.lncRNA

PCOTH non.epi.lncRNA

PCSK6-AS1 non.epi.lncRNA

PCYT1B-AS1 non.epi.lncRNA

PDCD4-AS1 non.epi.lncRNA

PDYN-AS1 non.epi.lncRNA

PDZRN3-AS1 non.epi.lncRNA

PEX5L-AS1 non.epi.lncRNA

PEX5L-AS2 non.epi.lncRNA

PGM5P3-AS1 non.epi.lncRNA

PGM5P4-AS1 non.epi.lncRNA

PHACTR2-AS1 non.epi.lncRNA

PHEX-AS1 non.epi.lncRNA

PHKA1-AS1 non.epi.lncRNA

PHKA2-AS1 non.epi.lncRNA

PICSAR non.epi.lncRNA

PIK3CD-AS1 non.epi.lncRNA

PIK3IP1-AS1 non.epi.lncRNA

PINCR non.epi.lncRNA

PINK1-AS non.epi.lncRNA

PISRT1 non.epi.lncRNA

PITPNM2-AS1 non.epi.lncRNA

PITRM1-AS1 non.epi.lncRNA

PKIA-AS1 non.epi.lncRNA

PKN2-AS1 non.epi.lncRNA

PKNOX2-AS1 non.epi.lncRNA

PKP4-AS1 non.epi.lncRNA

PLA2G4C-AS1 non.epi.lncRNA

PLA2G4E-AS1 non.epi.lncRNA

PLAC4 non.epi.lncRNA

PLBD1-AS1 non.epi.lncRNA

PLCB1-IT1 non.epi.lncRNA

PLCE1-AS1 non.epi.lncRNA

PLCG1-AS1 non.epi.lncRNA

PLCH1-AS1 non.epi.lncRNA

PLCH1-AS2 non.epi.lncRNA

PLCL2-AS1 non.epi.lncRNA

PLCXD2-AS1 non.epi.lncRNA

PLS1-AS1 non.epi.lncRNA

PLS3-AS1 non.epi.lncRNA

PLSCR5-AS1 non.epi.lncRNA

PLUT non.epi.lncRNA

POC1B-AS1 non.epi.lncRNA

POLR2J4 non.epi.lncRNA

POT1-AS1 non.epi.lncRNA

POTEH-AS1 non.epi.lncRNA

POU6F2-AS2 non.epi.lncRNA

PPEF1-AS1 non.epi.lncRNA

PPFIA2-AS1 non.epi.lncRNA

PPM1K-DT non.epi.lncRNA

PPP1R14B-AS1 non.epi.lncRNA

PPP1R26-AS1 non.epi.lncRNA

PPP2R2B-IT1 non.epi.lncRNA

PPP3CB-AS1 non.epi.lncRNA

PRICKLE2-AS1 non.epi.lncRNA

PRICKLE2-AS2 non.epi.lncRNA

PRICKLE2-AS3 non.epi.lncRNA

PRKAG2-AS1 non.epi.lncRNA

PRKAR2A-AS1 non.epi.lncRNA

PRKCA-AS1 non.epi.lncRNA

PRKCZ-AS1 non.epi.lncRNA

PRKG1-AS1 non.epi.lncRNA

PRKX-AS1 non.epi.lncRNA

PRMT5-AS1 non.epi.lncRNA

PRNT non.epi.lncRNA

PRORY non.epi.lncRNA

PROSER2-AS1 non.epi.lncRNA

PROX1-AS1 non.epi.lncRNA

PRR29-AS1 non.epi.lncRNA

PRR34 non.epi.lncRNA

PRR34-AS1 non.epi.lncRNA

PRRT3-AS1 non.epi.lncRNA

PRRX2-AS1 non.epi.lncRNA

PSG8-AS1 non.epi.lncRNA

PSMA3-AS1 non.epi.lncRNA

PSMD6-AS1 non.epi.lncRNA

PSMD6-AS2 non.epi.lncRNA

PSMG3-AS1 non.epi.lncRNA

PSORS1C3 non.epi.lncRNA

PSPC1-AS2 non.epi.lncRNA

PTCHD1-AS non.epi.lncRNA

PTCSC2 non.epi.lncRNA

PTCSC3 non.epi.lncRNA

PTENP1-AS non.epi.lncRNA

PTOV1-AS1 non.epi.lncRNA

PTOV1-AS2 non.epi.lncRNA

PTPRD-AS1 non.epi.lncRNA

PTPRD-AS2 non.epi.lncRNA

PTPRG-AS1 non.epi.lncRNA

PTPRJ-AS1 non.epi.lncRNA

PURPL non.epi.lncRNA

PVT1 non.epi.lncRNA

PWRN1 non.epi.lncRNA

PWRN2 non.epi.lncRNA

PWRN4 non.epi.lncRNA

PXN-AS1 non.epi.lncRNA

RAB11B-AS1 non.epi.lncRNA

RAB30-DT non.epi.lncRNA

RABGAP1L-DT non.epi.lncRNA

RABGAP1L-IT1 non.epi.lncRNA

RAD51-AS1 non.epi.lncRNA

RAMP2-AS1 non.epi.lncRNA

RAP2C-AS1 non.epi.lncRNA

RAPGEF4-AS1 non.epi.lncRNA

RARA-AS1 non.epi.lncRNA

RASA2-IT1 non.epi.lncRNA

RASA3-IT1 non.epi.lncRNA

RASGRF2-AS1 non.epi.lncRNA

RASSF1-AS1 non.epi.lncRNA

RASSF10-DT non.epi.lncRNA

RASSF8-AS1 non.epi.lncRNA

RB1-DT non.epi.lncRNA

RBFADN non.epi.lncRNA

RBM15-AS1 non.epi.lncRNA

RBM5-AS1 non.epi.lncRNA

RBMS3-AS1 non.epi.lncRNA

RBMS3-AS2 non.epi.lncRNA

RBMS3-AS3 non.epi.lncRNA

RBPMS-AS1 non.epi.lncRNA

RC3H1-IT1 non.epi.lncRNA

RDH10-AS1 non.epi.lncRNA

RERG-AS1 non.epi.lncRNA

RERG-IT1 non.epi.lncRNA

REV3L-IT1 non.epi.lncRNA

RFPL1S non.epi.lncRNA

RFX3-AS1 non.epi.lncRNA

RGPD4-AS1 non.epi.lncRNA

RHOA-IT1 non.epi.lncRNA

RHOXF1-AS1 non.epi.lncRNA

RIC3-DT non.epi.lncRNA

RMRP non.epi.lncRNA

RMST non.epi.lncRNA

RN7SL832P non.epi.lncRNA

RNF144A-AS1 non.epi.lncRNA

RNF157-AS1 non.epi.lncRNA

RNF185-AS1 non.epi.lncRNA

RNF213-AS1 non.epi.lncRNA

RNF217-AS1 non.epi.lncRNA

RNFT1-DT non.epi.lncRNA

ROPN1L-AS1 non.epi.lncRNA

ROR1-AS1 non.epi.lncRNA

RORA-AS1 non.epi.lncRNA

RORA-AS2 non.epi.lncRNA

RORB-AS1 non.epi.lncRNA

RPL34-AS1 non.epi.lncRNA

RPP38-DT non.epi.lncRNA

RPS6KA2-AS1 non.epi.lncRNA

RPS6KB2-AS1 non.epi.lncRNA

RRM1-AS1 non.epi.lncRNA

RRN3P2 non.epi.lncRNA

RSF1-IT2 non.epi.lncRNA

RUNX2-AS1 non.epi.lncRNA

RUVBL1-AS1 non.epi.lncRNA

RXYLT1-AS1 non.epi.lncRNA

SACS-AS1 non.epi.lncRNA

SALRNA1 non.epi.lncRNA

SALRNA2 non.epi.lncRNA

SALRNA3 non.epi.lncRNA

SAMD12-AS1 non.epi.lncRNA

SAMMSON non.epi.lncRNA

SAMSN1-AS1 non.epi.lncRNA

SAP30L-AS1 non.epi.lncRNA

SAPCD1-AS1 non.epi.lncRNA

SATB1-AS1 non.epi.lncRNA

SATB2-AS1 non.epi.lncRNA

SCAANT1 non.epi.lncRNA

SCAMP1-AS1 non.epi.lncRNA

SCARNA9 non.epi.lncRNA

SCAT1 non.epi.lncRNA

SCAT8 non.epi.lncRNA

SCEL-AS1 non.epi.lncRNA

SCGB1B2P non.epi.lncRNA

SCHLAP1 non.epi.lncRNA

SCN1A-AS1 non.epi.lncRNA

SCOC-AS1 non.epi.lncRNA

SDCBP2-AS1 non.epi.lncRNA

SEC23A-AS1 non.epi.lncRNA

SEC24B-AS1 non.epi.lncRNA

SEMA3B-AS1 non.epi.lncRNA

SEMA3F-AS1 non.epi.lncRNA

SEMA5A-AS1 non.epi.lncRNA

SEMA6A-AS1 non.epi.lncRNA

SEMA6A-AS2 non.epi.lncRNA

SENCR non.epi.lncRNA

SEPTIN7-AS1 non.epi.lncRNA

SEPTIN9-DT non.epi.lncRNA

SERTAD4-AS1 non.epi.lncRNA

SFTA1P non.epi.lncRNA

SFTA3 non.epi.lncRNA

SGO1-AS1 non.epi.lncRNA

SH3BP5-AS1 non.epi.lncRNA

SH3PXD2A-AS1 non.epi.lncRNA

SH3RF3-AS1 non.epi.lncRNA

SH3TC2-DT non.epi.lncRNA

SHANK2-AS3 non.epi.lncRNA

SIAH2-AS1 non.epi.lncRNA

SIDT1-AS1 non.epi.lncRNA

SILC1 non.epi.lncRNA

SIRLNT non.epi.lncRNA

SIRPG-AS1 non.epi.lncRNA

SIX3-AS1 non.epi.lncRNA

SKAP1-AS1 non.epi.lncRNA

SLC12A5-AS1 non.epi.lncRNA

SLC12A9-AS1 non.epi.lncRNA

SLC14A2-AS1 non.epi.lncRNA

SLC25A21-AS1 non.epi.lncRNA

SLC25A25-AS1 non.epi.lncRNA

SLC25A30-AS1 non.epi.lncRNA

SLC25A48-AS1 non.epi.lncRNA

SLC25A5-AS1 non.epi.lncRNA

SLC26A4-AS1 non.epi.lncRNA

SLC2A1-AS1 non.epi.lncRNA

SLC39A12-AS1 non.epi.lncRNA

SLC5A4-AS1 non.epi.lncRNA

SLC6A1-AS1 non.epi.lncRNA

SLC7A11-AS1 non.epi.lncRNA

SLC8A1-AS1 non.epi.lncRNA

SLC9A3-AS1 non.epi.lncRNA

SLC9A9-AS1 non.epi.lncRNA

SLC9A9-AS2 non.epi.lncRNA

SLFNL1-AS1 non.epi.lncRNA

SLIT1-AS1 non.epi.lncRNA

SLIT2-IT1 non.epi.lncRNA

SLX1A-SULT1A3 non.epi.lncRNA

SLX1B-SULT1A4 non.epi.lncRNA

SMAD1-AS1 non.epi.lncRNA

SMAD1-AS2 non.epi.lncRNA

SMAD9-IT1 non.epi.lncRNA

SMARCA5-AS1 non.epi.lncRNA

SMC5-AS1 non.epi.lncRNA

SMCR2 non.epi.lncRNA

SMG7-AS1 non.epi.lncRNA

SMILR non.epi.lncRNA

SMIM10L2B-AS1 non.epi.lncRNA

SMIM15-AS1 non.epi.lncRNA

SMIM2-AS1 non.epi.lncRNA

SMIM2-IT1 non.epi.lncRNA

SMIM25 non.epi.lncRNA

SMYD3-IT1 non.epi.lncRNA

SNAI3-AS1 non.epi.lncRNA

SNAP25-AS1 non.epi.lncRNA

SNAP47-AS1 non.epi.lncRNA

SNCA-AS1 non.epi.lncRNA

SND1-IT1 non.epi.lncRNA

SNHG12 non.epi.lncRNA

SNHG14 non.epi.lncRNA

SNHG17 non.epi.lncRNA

SNHG18 non.epi.lncRNA

SNHG20 non.epi.lncRNA

SNHG22 non.epi.lncRNA

SNHG26 non.epi.lncRNA

SNHG27 non.epi.lncRNA

SNHG28 non.epi.lncRNA

SNHG29 non.epi.lncRNA

SNHG31 non.epi.lncRNA

SNHG7 non.epi.lncRNA

SNHG8 non.epi.lncRNA

SNRK-AS1 non.epi.lncRNA

SORCS3-AS1 non.epi.lncRNA

SOS1-IT1 non.epi.lncRNA

SOX1-OT non.epi.lncRNA

SOX2-OT non.epi.lncRNA

SOX21-AS1 non.epi.lncRNA

SOX5-AS1 non.epi.lncRNA

SOX9-AS1 non.epi.lncRNA

SPAG16-DT non.epi.lncRNA

SPANXA2-OT1 non.epi.lncRNA

SPART-AS1 non.epi.lncRNA

SPATA13-AS1 non.epi.lncRNA

SPATA17-AS1 non.epi.lncRNA

SPATA3-AS1 non.epi.lncRNA

SPATA42 non.epi.lncRNA

SPATA8 non.epi.lncRNA

SPATA8-AS1 non.epi.lncRNA

SPDYE11 non.epi.lncRNA

SPIN4-AS1 non.epi.lncRNA

SPINT1-AS1 non.epi.lncRNA

SPON1-AS1 non.epi.lncRNA

SPRY4-AS1 non.epi.lncRNA

SRD5A3-AS1 non.epi.lncRNA

SRGAP2-AS1 non.epi.lncRNA

SRGAP3-AS1 non.epi.lncRNA

SRGAP3-AS2 non.epi.lncRNA

SRRM2-AS1 non.epi.lncRNA

SSBP3-AS1 non.epi.lncRNA

SSSCA1-AS1 non.epi.lncRNA

SSTR5-AS1 non.epi.lncRNA

ST3GAL5-AS1 non.epi.lncRNA

ST3GAL6-AS1 non.epi.lncRNA

ST6GAL2-IT1 non.epi.lncRNA

ST7-AS1 non.epi.lncRNA

ST7-AS2 non.epi.lncRNA

ST7-OT4 non.epi.lncRNA

ST8SIA6-AS1 non.epi.lncRNA

STAG3L5P-PVRIG2P-PILRB non.epi.lncRNA

STAM-AS1 non.epi.lncRNA

STARD13-AS non.epi.lncRNA

STARD13-IT1 non.epi.lncRNA

STARD4-AS1 non.epi.lncRNA

STAU2-AS1 non.epi.lncRNA

STEAP2-AS1 non.epi.lncRNA

STIM2-AS1 non.epi.lncRNA

STK32A-AS1 non.epi.lncRNA

STPG2-AS1 non.epi.lncRNA

STRA6LP non.epi.lncRNA

STX17-AS1 non.epi.lncRNA

STX18-AS1 non.epi.lncRNA

STX18-IT1 non.epi.lncRNA

STXBP5-AS1 non.epi.lncRNA

SUCLA2-AS1 non.epi.lncRNA

SUCLG2-AS1 non.epi.lncRNA

SUGT1P4-STRA6LP non.epi.lncRNA

SYNPR-AS1 non.epi.lncRNA

SYP-AS1 non.epi.lncRNA

SZT2-AS1 non.epi.lncRNA

TAB2-AS1 non.epi.lncRNA

TAB3-AS1 non.epi.lncRNA

TAB3-AS2 non.epi.lncRNA

TAF1A-AS1 non.epi.lncRNA

TAPT1-AS1 non.epi.lncRNA

TARID non.epi.lncRNA

TAT-AS1 non.epi.lncRNA

TBC1D22A-AS1 non.epi.lncRNA

TBC1D3P1-DHX40P1 non.epi.lncRNA

TBC1D8-AS1 non.epi.lncRNA

TBILA non.epi.lncRNA

TBX18-AS1 non.epi.lncRNA

TBX2-AS1 non.epi.lncRNA

TBX5-AS1 non.epi.lncRNA

TCERG1L-AS1 non.epi.lncRNA

TCF4-AS1 non.epi.lncRNA

TCF4-AS2 non.epi.lncRNA

TCF7L1-IT1 non.epi.lncRNA

TCL6 non.epi.lncRNA

TDRG1 non.epi.lncRNA

TEMN3-AS1 non.epi.lncRNA

TENM3-AS1 non.epi.lncRNA

TERC non.epi.lncRNA

TESC-AS1 non.epi.lncRNA

TET2-AS1 non.epi.lncRNA

TEX26-AS1 non.epi.lncRNA

TEX36-AS1 non.epi.lncRNA

TEX41 non.epi.lncRNA

TFAP2A-AS1 non.epi.lncRNA

TGFA-IT1 non.epi.lncRNA

TGFB3-AS1 non.epi.lncRNA

TH2LCRR non.epi.lncRNA

THAP7-AS1 non.epi.lncRNA

THAP9-AS1 non.epi.lncRNA

THOC7-AS1 non.epi.lncRNA

THORLNC non.epi.lncRNA

THRA1/BTR non.epi.lncRNA

THRB-AS1 non.epi.lncRNA

THRB-IT1 non.epi.lncRNA

THRIL non.epi.lncRNA

THSD4-AS1 non.epi.lncRNA

THUMPD3-AS1 non.epi.lncRNA

TIPARP-AS1 non.epi.lncRNA

TLR8-AS1 non.epi.lncRNA

TLX1NB non.epi.lncRNA

TM4SF19-AS1 non.epi.lncRNA

TMC3-AS1 non.epi.lncRNA

TMCC1-AS1 non.epi.lncRNA

TMCO1-AS1 non.epi.lncRNA

TMED2-DT non.epi.lncRNA

TMEM108-AS1 non.epi.lncRNA

TMEM132D-AS1 non.epi.lncRNA

TMEM132D-AS2 non.epi.lncRNA

TMEM161B-AS1 non.epi.lncRNA

TMEM202-AS1 non.epi.lncRNA

TMEM212-AS1 non.epi.lncRNA

TMEM212-IT1 non.epi.lncRNA

TMEM220-AS1 non.epi.lncRNA

TMEM246-AS1 non.epi.lncRNA

TMEM252-DT non.epi.lncRNA

TMEM254-AS1 non.epi.lncRNA

TMEM26-AS1 non.epi.lncRNA

TMEM51-AS1 non.epi.lncRNA

TMEM72-AS1 non.epi.lncRNA

TMEM78 non.epi.lncRNA

TMEM92-AS1 non.epi.lncRNA

TMLHE-AS1 non.epi.lncRNA

TMSB15B-AS1 non.epi.lncRNA

TNFRSF10A-AS1 non.epi.lncRNA

TNKS2-AS1 non.epi.lncRNA

TNR-IT1 non.epi.lncRNA

TNRC6C-AS1 non.epi.lncRNA

TOB1-AS1 non.epi.lncRNA

TOLLIP-AS1 non.epi.lncRNA

TONSL-AS1 non.epi.lncRNA

TP53TG1 non.epi.lncRNA

TPM1-AS non.epi.lncRNA

TPRG1-AS1 non.epi.lncRNA

TPRG1-AS2 non.epi.lncRNA

TPT1-AS1 non.epi.lncRNA

TRAF3IP2-AS1 non.epi.lncRNA

TRAPPC12-AS1 non.epi.lncRNA

TRBV11-2 non.epi.lncRNA

TRDN-AS1 non.epi.lncRNA

TRERNA1 non.epi.lncRNA

TRHDE-AS1 non.epi.lncRNA

TRIM36-IT1 non.epi.lncRNA

TRIM52-AS1 non.epi.lncRNA

TRMT2B-AS1 non.epi.lncRNA

TRPM2-AS non.epi.lncRNA

TSBP1-AS1 non.epi.lncRNA

TSC22D1-AS1 non.epi.lncRNA

TSIX non.epi.lncRNA

TSPAN9-IT1 non.epi.lncRNA

TSPEAR-AS1 non.epi.lncRNA

TSPEAR-AS2 non.epi.lncRNA

TSPOAP1-AS1 non.epi.lncRNA

TTC21B-AS1 non.epi.lncRNA

TTC28-AS1 non.epi.lncRNA

TTC3-AS1 non.epi.lncRNA

TTC39A-AS1 non.epi.lncRNA

TTLL10-AS1 non.epi.lncRNA

TTLL11-IT1 non.epi.lncRNA

TTLL7-IT1 non.epi.lncRNA

TTN-AS1 non.epi.lncRNA

TTTY1 non.epi.lncRNA

TTTY10 non.epi.lncRNA

TTTY12 non.epi.lncRNA

TTTY13 non.epi.lncRNA

TTTY14 non.epi.lncRNA

TTTY17A non.epi.lncRNA

TTTY17B non.epi.lncRNA

TTTY17C non.epi.lncRNA

TTTY18 non.epi.lncRNA

TTTY19 non.epi.lncRNA

TTTY1B non.epi.lncRNA

TTTY2 non.epi.lncRNA

TTTY20 non.epi.lncRNA

TTTY21 non.epi.lncRNA

TTTY21B non.epi.lncRNA

TTTY22 non.epi.lncRNA

TTTY23 non.epi.lncRNA

TTTY2B non.epi.lncRNA

TTTY3 non.epi.lncRNA

TTTY3B non.epi.lncRNA

TTTY4 non.epi.lncRNA

TTTY4B non.epi.lncRNA

TTTY4C non.epi.lncRNA

TTTY5 non.epi.lncRNA

TTTY6 non.epi.lncRNA

TTTY6B non.epi.lncRNA

TTTY7 non.epi.lncRNA

TTTY7B non.epi.lncRNA

TTTY8 non.epi.lncRNA

TTTY8B non.epi.lncRNA

TTTY9B non.epi.lncRNA

TUB-AS1 non.epi.lncRNA

TUBA3FP non.epi.lncRNA

TUG1 non.epi.lncRNA

TUSC7 non.epi.lncRNA

TYMSOS non.epi.lncRNA

U47924.1 non.epi.lncRNA

U47924.3 non.epi.lncRNA

U51244.1 non.epi.lncRNA

U52111.1 non.epi.lncRNA

U52112.1 non.epi.lncRNA

U62317.1 non.epi.lncRNA

U62317.2 non.epi.lncRNA

U62317.3 non.epi.lncRNA

U62317.4 non.epi.lncRNA

U62631.1 non.epi.lncRNA

U73166.1 non.epi.lncRNA

U91319.1 non.epi.lncRNA

U91324.1 non.epi.lncRNA

U91328.1 non.epi.lncRNA

U91328.2 non.epi.lncRNA

U91328.3 non.epi.lncRNA

U95743.1 non.epi.lncRNA

UBA6-AS1 non.epi.lncRNA

UBE2D3-AS1 non.epi.lncRNA

UBE2Q1-AS1 non.epi.lncRNA

UBE2R2-AS1 non.epi.lncRNA

UBL7-AS1 non.epi.lncRNA

UBOX5-AS1 non.epi.lncRNA

UBXN7-AS1 non.epi.lncRNA

UCA1 non.epi.lncRNA

UCHL1-AS1 non.epi.lncRNA

UFL1-AS1 non.epi.lncRNA

UGDH-AS1 non.epi.lncRNA

UMLILO non.epi.lncRNA

UMODL1-AS1 non.epi.lncRNA

UNC5B-AS1 non.epi.lncRNA

UPK1A-AS1 non.epi.lncRNA

UPP2-IT1 non.epi.lncRNA

USP2-AS1 non.epi.lncRNA

USP27X-AS1 non.epi.lncRNA

USP3-AS1 non.epi.lncRNA

USP30-AS1 non.epi.lncRNA

UST-AS1 non.epi.lncRNA

UXT-AS1 non.epi.lncRNA

VAC14-AS1 non.epi.lncRNA

VASH1-AS1 non.epi.lncRNA

VCAN-AS1 non.epi.lncRNA

VENTXP1 non.epi.lncRNA

VIPR1-AS1 non.epi.lncRNA

VLDLR-AS1 non.epi.lncRNA

VPS13A-AS1 non.epi.lncRNA

VPS13B-DT non.epi.lncRNA

VPS33B-DT non.epi.lncRNA

VPS9D1-AS1 non.epi.lncRNA

VSTM2A-OT1 non.epi.lncRNA

VWA8-AS1 non.epi.lncRNA

VWC2L-IT1 non.epi.lncRNA

WAC-AS1 non.epi.lncRNA

WARS2-AS1 non.epi.lncRNA

WARS2-IT1 non.epi.lncRNA

WASF3-AS1 non.epi.lncRNA

WASHC5-AS1 non.epi.lncRNA

WASIR1 non.epi.lncRNA

WDFY3-AS1 non.epi.lncRNA

WDFY3-AS2 non.epi.lncRNA

WDR11-AS1 non.epi.lncRNA

WDR7-OT1 non.epi.lncRNA

WDR86-AS1 non.epi.lncRNA

WNT5A-AS1 non.epi.lncRNA

WSPAR non.epi.lncRNA

WT1-AS non.epi.lncRNA

WWC3-AS1 non.epi.lncRNA

WWOX-AS1 non.epi.lncRNA

XACT non.epi.lncRNA

XIAP-AS1 non.epi.lncRNA

XIRP2-AS1 non.epi.lncRNA

XIST non.epi.lncRNA

YEATS2-AS1 non.epi.lncRNA

YTHDF3-AS1 non.epi.lncRNA

Z68323.1 non.epi.lncRNA

Z68871.1 non.epi.lncRNA

Z69666.1 non.epi.lncRNA

Z69706.1 non.epi.lncRNA

Z69720.1 non.epi.lncRNA

Z69733.1 non.epi.lncRNA

Z73495.1 non.epi.lncRNA

Z82173.1 non.epi.lncRNA

Z82180.1 non.epi.lncRNA

Z82185.1 non.epi.lncRNA

Z82186.1 non.epi.lncRNA

Z82188.2 non.epi.lncRNA

Z82196.1 non.epi.lncRNA

Z82196.2 non.epi.lncRNA

Z82198.1 non.epi.lncRNA

Z82198.2 non.epi.lncRNA

Z82202.1 non.epi.lncRNA

Z82214.1 non.epi.lncRNA

Z82214.2 non.epi.lncRNA

Z82243.1 non.epi.lncRNA

Z82246.1 non.epi.lncRNA

Z82249.1 non.epi.lncRNA

Z83839.2 non.epi.lncRNA

Z83844.2 non.epi.lncRNA

Z83847.1 non.epi.lncRNA

Z84468.1 non.epi.lncRNA

Z84485.1 non.epi.lncRNA

Z84488.1 non.epi.lncRNA

Z85994.1 non.epi.lncRNA

Z86062.2 non.epi.lncRNA

Z93022.1 non.epi.lncRNA

Z93241.1 non.epi.lncRNA

Z93403.1 non.epi.lncRNA

Z93930.2 non.epi.lncRNA

Z93930.3 non.epi.lncRNA

Z94057.1 non.epi.lncRNA

Z94160.1 non.epi.lncRNA

Z94160.2 non.epi.lncRNA

Z94721.1 non.epi.lncRNA

Z94721.2 non.epi.lncRNA

Z95114.1 non.epi.lncRNA

Z95114.2 non.epi.lncRNA

Z95114.4 non.epi.lncRNA

Z95115.1 non.epi.lncRNA

Z95331.1 non.epi.lncRNA

Z95624.1 non.epi.lncRNA

Z97055.2 non.epi.lncRNA

Z97056.1 non.epi.lncRNA

Z97192.1 non.epi.lncRNA

Z97192.2 non.epi.lncRNA

Z97192.3 non.epi.lncRNA

Z97198.1 non.epi.lncRNA

Z97200.1 non.epi.lncRNA

Z97205.1 non.epi.lncRNA

Z97205.2 non.epi.lncRNA

Z97353.2 non.epi.lncRNA

Z97653.1 non.epi.lncRNA

Z97832.2 non.epi.lncRNA

Z97986.1 non.epi.lncRNA

Z97987.1 non.epi.lncRNA

Z97989.1 non.epi.lncRNA

Z98200.1 non.epi.lncRNA

Z98257.1 non.epi.lncRNA

Z98259.1 non.epi.lncRNA

Z98742.1 non.epi.lncRNA

Z98747.1 non.epi.lncRNA

Z98749.1 non.epi.lncRNA

Z98752.1 non.epi.lncRNA

Z98884.1 non.epi.lncRNA

Z98884.2 non.epi.lncRNA

Z98885.2 non.epi.lncRNA

Z98885.3 non.epi.lncRNA

Z98949.1 non.epi.lncRNA

Z98949.3 non.epi.lncRNA

Z99127.1 non.epi.lncRNA

Z99289.1 non.epi.lncRNA

Z99289.2 non.epi.lncRNA

Z99289.3 non.epi.lncRNA

Z99716.1 non.epi.lncRNA

Z99755.2 non.epi.lncRNA

Z99756.1 non.epi.lncRNA

Z99758.1 non.epi.lncRNA

Z99774.1 non.epi.lncRNA

Z99916.1 non.epi.lncRNA

Z99916.3 non.epi.lncRNA

ZBED5-AS1 non.epi.lncRNA

ZBTB11-AS1 non.epi.lncRNA

ZBTB20-AS1 non.epi.lncRNA

ZBTB20-AS2 non.epi.lncRNA

ZBTB20-AS3 non.epi.lncRNA

ZBTB20-AS4 non.epi.lncRNA

ZBTB20-AS5 non.epi.lncRNA

ZBTB44-DT non.epi.lncRNA

ZBTB46-AS1 non.epi.lncRNA

ZCCHC23 non.epi.lncRNA

ZDHHC20-IT1 non.epi.lncRNA

ZEB1-AS1 non.epi.lncRNA

ZFAS1 non.epi.lncRNA

ZFAT-AS1 non.epi.lncRNA

ZFHX4-AS1 non.epi.lncRNA

ZFPM2-AS1 non.epi.lncRNA

ZFY-AS1 non.epi.lncRNA

ZIC4-AS1 non.epi.lncRNA

ZIM2-AS1 non.epi.lncRNA

ZKSCAN2-DT non.epi.lncRNA

ZKSCAN7-AS1 non.epi.lncRNA

ZMIZ1-AS1 non.epi.lncRNA

ZMYND10-AS1 non.epi.lncRNA

ZNF114-AS1 non.epi.lncRNA

ZNF197-AS1 non.epi.lncRNA

ZNF213-AS1 non.epi.lncRNA

ZNF25-DT non.epi.lncRNA

ZNF252P-AS1 non.epi.lncRNA

ZNF295-AS1 non.epi.lncRNA

ZNF32-AS1 non.epi.lncRNA

ZNF32-AS2 non.epi.lncRNA

ZNF32-AS3 non.epi.lncRNA

ZNF337-AS1 non.epi.lncRNA

ZNF341-AS1 non.epi.lncRNA

ZNF346-IT1 non.epi.lncRNA

ZNF350-AS1 non.epi.lncRNA

ZNF385D-AS1 non.epi.lncRNA

ZNF385D-AS2 non.epi.lncRNA

ZNF426-DT non.epi.lncRNA

ZNF433-AS1 non.epi.lncRNA

ZNF436-AS1 non.epi.lncRNA

ZNF451-AS1 non.epi.lncRNA

ZNF503-AS1 non.epi.lncRNA

ZNF528-AS1 non.epi.lncRNA

ZNF529-AS1 non.epi.lncRNA

ZNF561-AS1 non.epi.lncRNA

ZNF571-AS1 non.epi.lncRNA

ZNF649-AS1 non.epi.lncRNA

ZNF667-AS1 non.epi.lncRNA

ZNF674-AS1 non.epi.lncRNA

ZNF687-AS1 non.epi.lncRNA

ZNF710-AS1 non.epi.lncRNA

ZNF790-AS1 non.epi.lncRNA

ZNF793-AS1 non.epi.lncRNA

ZNF8-ERVK3-1 non.epi.lncRNA

ZNF84-DT non.epi.lncRNA

ZNRF3-IT1 non.epi.lncRNA

ZRANB2-AS2 non.epi.lncRNA

ZSCAN16-AS1 non.epi.lncRNA

TOMM6 epi.PCG

XBP1 epi.PCG

POLR2F epi.PCG

EEF1G epi.PCG

U2AF1 epi.PCG

FAM156A epi.PCG

OVCA2 epi.PCG

AC026464.4 epi.PCG

AC010132.3 epi.PCG

TEN1 epi.PCG

EIF3CL epi.PCG

TREX1 epi.PCG

AC011462.1 epi.PCG

AP003419.1 epi.PCG

CMC4 epi.PCG

PTRH1 epi.PCG

ZNF410 epi.PCG

ATRIP epi.PCG

ANKHD1 epi.PCG

TOP3B epi.PCG

EIF3C epi.PCG

ISY1-RAB43 epi.PCG

AP003108.2 epi.PCG

ACAD11 epi.PCG

CBWD5 epi.PCG

INO80B-WBP1 epi.PCG

C1QTNF5 epi.PCG

SNX15 epi.PCG

MATR3 epi.PCG

POLR2J3 epi.PCG

MEMO1 epi.PCG

NDST2 epi.PCG

ANKHD1-EIF4EBP3 epi.PCG

GTF2H2C epi.PCG

C17orf49 epi.PCG

ZNRF3 epi.PCG

DDX47 epi.PCG

ARPIN-AP3S2 epi.PCG

ADIRF epi.PCG

PIK3R2 epi.PCG

NACA epi.PCG

SCLY epi.PCG

RTEL1 epi.PCG

OGFOD2 epi.PCG

EIF4A1 epi.PCG

SARNP epi.PCG

ZNF23 epi.PCG

BBS1 epi.PCG

WDR73 epi.PCG

RBM34 epi.PCG

NOTCH2NLA epi.PCG

KREMEN1 epi.PCG

SPSB3 epi.PCG

COMMD3-BMI1 epi.PCG

MTCP1 epi.PCG

ALG9 epi.PCG

AC011511.4 epi.PCG

GTF2IRD2B epi.PCG

AARSD1 epi.PCG

CNBD2 epi.PCG

RASA4B epi.PCG

ZBTB9 epi.PCG

GET4 epi.PCG

HTD2 epi.PCG

CBWD2 epi.PCG

RBM4 epi.PCG

CYB5D1 epi.PCG

NBPF10 epi.PCG

UBE2V1 epi.PCG

HNRNPUL2-BSCL2 epi.PCG

DNAJC25-GNG10 epi.PCG

GPS2 epi.PCG

SPNS1 epi.PCG

RPL17 epi.PCG

TTC4 epi.PCG

DNAH14 epi.PCG

RNASEK epi.PCG

ASB3 epi.PCG

C18orf32 epi.PCG

ZSCAN32 epi.PCG

CCZ1B epi.PCG

MIA2 epi.PCG

MTMR3 epi.PCG

ANKRD36B epi.PCG

DIABLO epi.PCG

NHEJ1 epi.PCG

GPR89A epi.PCG

STIMATE epi.PCG

TICAM2 epi.PCG

GLS2 epi.PCG

FAM47E-STBD1 epi.PCG

AC027796.3 epi.PCG

ZACN epi.PCG

DTWD1 epi.PCG

CAPN3 epi.PCG

AS3MT epi.PCG

ANKRD36 epi.PCG

STRADA epi.PCG

CCZ1 epi.PCG

ARF6 epi.PCG

CELF6 epi.PCG

TXNDC5 epi.PCG

PRPF40B epi.PCG

ABHD16A epi.PCG

AL049839.2 epi.PCG

MISP epi.PCG

GPR89B epi.PCG

AL662899.2 epi.PCG

CMC2 epi.PCG

TTC7B epi.PCG

WDR27 epi.PCG

BLOC1S5-TXNDC5 epi.PCG

FMC1-LUC7L2 epi.PCG

PYURF epi.PCG

SARM1 epi.PCG

PSMD9 epi.PCG

SH3KBP1 epi.PCG

COX20 epi.PCG

RGPD8 epi.PCG

IFI30 epi.PCG

AC022400.5 epi.PCG

PSMA2 epi.PCG

KCNMB3 epi.PCG

RAP2B epi.PCG

CMC1 epi.PCG

BRSK2 epi.PCG

CHKB epi.PCG

NPHP3 epi.PCG

PSMC1 epi.PCG

ZNF280D epi.PCG

FGFR1OP epi.PCG

CEMP1 epi.PCG

CAMK2N1 epi.PCG

LUC7L2 epi.PCG

RABIF epi.PCG

KLC1 epi.PCG

TUT4 epi.PCG

ELF4 epi.PCG

GSS epi.PCG

GOLGA8B epi.PCG

CDK3 epi.PCG

BEGAIN epi.PCG

EML5 epi.PCG

CHKB-CPT1B epi.PCG

RILPL2 epi.PCG

ZC3H14 epi.PCG

STIMATE-MUSTN1 epi.PCG

HAUS7 epi.PCG

CYB5A epi.PCG

GTF2H4 epi.PCG

PPT2-EGFL8 epi.PCG

PRORP epi.PCG

MRPL38 epi.PCG

WDR92 epi.PCG

ARPC4-TTLL3 epi.PCG

CDRT4 epi.PCG

A1BG epi.PCG

MTPN epi.PCG

GTF2H2 epi.PCG

ZNF493 epi.PCG

VPS18 epi.PCG

ECHDC2 epi.PCG

ZNF564 epi.PCG

BRF1 epi.PCG

SRXN1 epi.PCG

CDK11A epi.PCG

RABGEF1 epi.PCG

ACADL epi.PCG

RPL36A epi.PCG

SLC2A11 epi.PCG

PSMA6 epi.PCG

EEF1D epi.PCG

EFNB2 epi.PCG

APEX2 epi.PCG

MKNK1 epi.PCG

FUNDC2 epi.PCG

ILK epi.PCG

UBQLN2 epi.PCG

STX6 epi.PCG

RNF19B epi.PCG

BAG5 epi.PCG

H3F3A epi.PCG

RNPC3 epi.PCG

ARHGAP8 epi.PCG

FAM204A epi.PCG

RYBP epi.PCG

PDXP epi.PCG

BUB1B-PAK6 epi.PCG

NAGA epi.PCG

HEMK1 epi.PCG

B4GALT5 epi.PCG

NDUFA7 epi.PCG

NPIPB2 epi.PCG

EDRF1 epi.PCG

BCKDHA epi.PCG

RPS29 epi.PCG

USP15 epi.PCG

FRMD4A epi.PCG

NBDY epi.PCG

SLC46A1 epi.PCG

MYEF2 epi.PCG

RIC3 epi.PCG

SETD6 epi.PCG

PSMD6 epi.PCG

SKAP2 epi.PCG

ARHGDIG epi.PCG

METTL22 epi.PCG

ST7L epi.PCG

OSTF1 epi.PCG

TRIM34 epi.PCG

COA1 epi.PCG

ANKRD23 epi.PCG

GPKOW epi.PCG

TTLL3 epi.PCG

ANP32E epi.PCG

HYI epi.PCG

TM9SF1 epi.PCG

ITGA2 epi.PCG

ZNFX1 epi.PCG

FNBP4 epi.PCG

ZNF177 epi.PCG

ZNF333 epi.PCG

MTERF4 epi.PCG

KSR1 epi.PCG

GPR137B epi.PCG

RAP1B epi.PCG

EMB epi.PCG

FAHD2A epi.PCG

S100A6 epi.PCG

SLC27A5 epi.PCG

FARP1 epi.PCG

SARS2 epi.PCG

ABRAXAS2 epi.PCG

ZFPL1 epi.PCG

MTG1 epi.PCG

NRG4 epi.PCG

RBM28 epi.PCG

ZBTB7A epi.PCG

VIPR2 epi.PCG

SLC35F6 epi.PCG

PSMG4 epi.PCG

RALA epi.PCG

C15orf40 epi.PCG

MPPE1 epi.PCG

SPTLC2 epi.PCG

NUDT6 epi.PCG

GIT2 epi.PCG

CORO2A epi.PCG

NDUFV2 epi.PCG

LRRC24 epi.PCG

PILRB epi.PCG

UBE2T epi.PCG

GVQW3 epi.PCG

ADHFE1 epi.PCG

CLK3 epi.PCG

EFCAB2 epi.PCG

MSTO1 epi.PCG

POLI epi.PCG

BSCL2 epi.PCG

NBPF26 epi.PCG

CCNK epi.PCG

SGCB epi.PCG

MARCKSL1 epi.PCG

RALGAPA1 epi.PCG

LIX1L epi.PCG

PPP3CA epi.PCG

FAM47E epi.PCG

VDR epi.PCG

TUBE1 epi.PCG

SYAP1 epi.PCG

SERPINA3 epi.PCG

ELK3 epi.PCG

RAP2A epi.PCG

BBIP1 epi.PCG

DPH1 epi.PCG

SLC31A2 epi.PCG

SMAD2 epi.PCG

DTNA epi.PCG

APOL1 epi.PCG

HYPK epi.PCG

PI4K2A epi.PCG

ABCA5 epi.PCG

ALKBH6 epi.PCG

ERVK3-1 epi.PCG

IL13RA1 epi.PCG

SLC7A6OS epi.PCG

AC015813.2 epi.PCG

AHR epi.PCG

AC006254.1 epi.PCG

TOMM5 epi.PCG

ACAD8 epi.PCG

AC026464.6 epi.PCG

CKS2 epi.PCG

SMS epi.PCG

FAM133B epi.PCG

CCDC189 epi.PCG

MARVELD1 epi.PCG

FAM20B epi.PCG

PHC1 epi.PCG

CCDC117 epi.PCG

MICOS10 epi.PCG

TNFRSF21 epi.PCG

RP2 epi.PCG

ENDOD1 epi.PCG

PCSK7 epi.PCG

TMED3 epi.PCG

APOL6 epi.PCG

CYP2E1 epi.PCG

BMPR2 epi.PCG

ACTR3C epi.PCG

FUCA1 epi.PCG

PIGL epi.PCG

RABL2A epi.PCG

PIGP epi.PCG

INTS6 epi.PCG

MRPS24 epi.PCG

DPYSL3 epi.PCG

ZNF596 epi.PCG

SPEF2 epi.PCG

CNKSR3 epi.PCG

ZNF625-ZNF20 epi.PCG

PTBP3 epi.PCG

SLC2A14 epi.PCG

AL163636.2 epi.PCG

SERTAD1 epi.PCG

SDHC epi.PCG

PIK3C3 epi.PCG

MAN2C1 epi.PCG

F2R epi.PCG

HNRNPA0 epi.PCG

ATXN3 epi.PCG

SPX epi.PCG

PMM2 epi.PCG

SNX12 epi.PCG

FAM219B epi.PCG

HLA-A epi.PCG

WBP1 epi.PCG

CCDC155 epi.PCG

FLRT2 epi.PCG

RPS27L epi.PCG

RFESD epi.PCG

RAB22A epi.PCG

SOWAHC epi.PCG

SREK1 epi.PCG

SRP19 epi.PCG

CHFR epi.PCG

RNASE4 epi.PCG

GNAQ epi.PCG

PLEKHG1 epi.PCG

KDM4C epi.PCG

NRBF2 epi.PCG

ACTB epi.PCG

YWHAG epi.PCG

ADAMTS17 epi.PCG

NPHS1 epi.PCG

WBP1L epi.PCG

ANKAR epi.PCG

AC012651.1 epi.PCG

CDR2L epi.PCG

FAM153B epi.PCG

PAK2 epi.PCG

RAB8B epi.PCG

AC244197.3 epi.PCG

EFNB1 epi.PCG

ZNF98 epi.PCG

GIMAP5 epi.PCG

CCPG1 epi.PCG

CLK4 epi.PCG

ANAPC5 epi.PCG

MRNIP epi.PCG

LRRC28 epi.PCG

GTF2I epi.PCG

TSSK3 epi.PCG

MAPKAPK5 epi.PCG

BPNT1 epi.PCG

IKBKG epi.PCG

REEP3 epi.PCG

MB21D2 epi.PCG

TMEM266 epi.PCG

RNF212 epi.PCG

INAFM2 epi.PCG

ZNF585A epi.PCG

HINFP epi.PCG

ZDHHC20 epi.PCG

CTTNBP2NL epi.PCG

LYRM9 epi.PCG

KPNA3 epi.PCG

HIST1H4I epi.PCG

DDX39B epi.PCG

STK38 epi.PCG

RPL17-C18orf32 epi.PCG

CCDC71 epi.PCG

TWSG1 epi.PCG

MRPL14 epi.PCG

CARD11 epi.PCG

SLC25A45 epi.PCG

THOC1 epi.PCG

KLHDC2 epi.PCG

LRCH4 epi.PCG

HRH1 epi.PCG

B3GALT4 epi.PCG

TVP23C-CDRT4 epi.PCG

IFRD1 epi.PCG

TWF2 epi.PCG

SLC9A6 epi.PCG

BCL10 epi.PCG

SPRED1 epi.PCG

TPX2 epi.PCG

CASP3 epi.PCG

FBXO9 epi.PCG

C5orf15 epi.PCG

CFAP44 epi.PCG

RCC2 epi.PCG

ACTR2 epi.PCG

ENOSF1 epi.PCG

BATF epi.PCG

TM2D3 epi.PCG

IGFN1 epi.PCG

B3GNT3 epi.PCG

NCBP3 epi.PCG

ARIH1 epi.PCG

RBM14-RBM4 epi.PCG

CSTF2 epi.PCG

ZNF814 epi.PCG

ADPRHL2 epi.PCG

NPEPL1 epi.PCG

AL132780.3 epi.PCG

GNA13 epi.PCG

LGALS3BP epi.PCG

TAF10 epi.PCG

ZNF717 epi.PCG

WDFY2 epi.PCG

ABCB6 epi.PCG

E2F3 epi.PCG

CCDC66 epi.PCG

CAMTA1 epi.PCG

TMEM134 epi.PCG

MRPS18C epi.PCG

MON2 epi.PCG

GNA15 epi.PCG

SAMD9 epi.PCG

C22orf39 epi.PCG

POMP epi.PCG

HMCES epi.PCG

RGS10 epi.PCG

MTHFD2L epi.PCG

SFT2D2 epi.PCG

OLFML2B epi.PCG

UNC5B epi.PCG

HYDIN epi.PCG

SERTAD3 epi.PCG

UBTD2 epi.PCG

RLIM epi.PCG

RALB epi.PCG

SLC24A3 epi.PCG

NACC1 epi.PCG

DMKN epi.PCG

UBA5 epi.PCG

SPRY4 epi.PCG

GRB10 epi.PCG

SELPLG epi.PCG

COL5A2 epi.PCG

N4BP2L2 epi.PCG

PAN2 epi.PCG

FAM214A epi.PCG

GM2A epi.PCG

YWHAB epi.PCG

DERL2 epi.PCG

ZNF19 epi.PCG

ING5 epi.PCG

CMTM6 epi.PCG

SYNE1 epi.PCG

DYRK4 epi.PCG

RPAP2 epi.PCG

RBPJL epi.PCG

IRF9 epi.PCG

CCDC32 epi.PCG

CTHRC1 epi.PCG

MSH5 epi.PCG

HOMER2 epi.PCG

AFAP1 epi.PCG

APOBEC3C epi.PCG

FAM227B epi.PCG

PRDM5 epi.PCG

TP53RK epi.PCG

MRRF epi.PCG

EIF3F epi.PCG

MCM6 epi.PCG

HMGN4 epi.PCG

HDAC10 epi.PCG

MTO1 epi.PCG

FEM1B epi.PCG

ZNF703 epi.PCG

ABCC6 epi.PCG

DCAF8 epi.PCG

DPP10 epi.PCG

RNF31 epi.PCG

CHMP4B epi.PCG

TBC1D2 epi.PCG

BGN epi.PCG

POLR3E epi.PCG

ZNF706 epi.PCG

ARL4C epi.PCG

ZSWIM1 epi.PCG

FBXW12 epi.PCG

TMEM138 epi.PCG

MAN2A2 epi.PCG

TPD52L1 epi.PCG

CWC22 epi.PCG

RBBP9 epi.PCG

PHYKPL epi.PCG

CHMP1B epi.PCG

NME1-NME2 epi.PCG

STARD5 epi.PCG

ZNF7 epi.PCG

CECR2 epi.PCG

LAMP5 epi.PCG

DPH6 epi.PCG

PGRMC1 epi.PCG

DBNDD2 epi.PCG

NDUFAF5 epi.PCG

SPSB4 epi.PCG

TOMM34 epi.PCG

PTPN1 epi.PCG

GOSR2 epi.PCG

AOPEP epi.PCG

TRIM50 epi.PCG

AC135050.2 epi.PCG

ABHD13 epi.PCG

ADORA2A epi.PCG

OSBPL11 epi.PCG

TTYH3 epi.PCG

TLCD4-RWDD3 epi.PCG

MICAL3 epi.PCG

SCCPDH epi.PCG

ATP9B epi.PCG

UPF3A epi.PCG

TIMM23 epi.PCG

ATP6AP2 epi.PCG

EDNRA epi.PCG

FAM221A epi.PCG

PNPLA7 epi.PCG

MRPL46 epi.PCG

TSPAN1 epi.PCG

LPAR5 epi.PCG

KPNA4 epi.PCG

NDUFA9 epi.PCG

SLC6A6 epi.PCG

LAMC2 epi.PCG

DPYSL2 epi.PCG

MCUB epi.PCG

SNAPC1 epi.PCG

ASB7 epi.PCG

CXCL16 epi.PCG

NQO2 epi.PCG

ZNF26 epi.PCG

DRG2 epi.PCG

SGPP2 epi.PCG

AC010522.1 epi.PCG

GDPD5 epi.PCG

ATP5MF-PTCD1 epi.PCG

SECISBP2 epi.PCG

KCTD5 epi.PCG

ZSCAN5A epi.PCG

CERS4 epi.PCG

ALAS1 epi.PCG

KLHDC4 epi.PCG

TFPT epi.PCG

PEA15 epi.PCG

FAM187A epi.PCG

NTPCR epi.PCG

MFSD14B epi.PCG

INO80B epi.PCG

UBR4 epi.PCG

TEPSIN epi.PCG

PANX1 epi.PCG

NEDD4L epi.PCG

ICMT epi.PCG

BIK epi.PCG

EGF epi.PCG

DNAJC24 epi.PCG

PDCL epi.PCG

INHBA epi.PCG

RBM5 epi.PCG

ANO1 epi.PCG

FNDC10 epi.PCG

POLR1D epi.PCG

EIF4E epi.PCG

HMBOX1 epi.PCG

ARL8A epi.PCG

PURB epi.PCG

TAMM41 epi.PCG

ZNF709 epi.PCG

UHMK1 epi.PCG

L3HYPDH epi.PCG

ACVR1 epi.PCG

LRCH1 epi.PCG

AZI2 epi.PCG

SPTSSA epi.PCG

STX16-NPEPL1 epi.PCG

WDR13 epi.PCG

GPR153 epi.PCG

TXNL1 epi.PCG

ZNF629 epi.PCG

PBDC1 epi.PCG

TRMU epi.PCG

PDE4DIP epi.PCG

MMP9 epi.PCG

ZNF224 epi.PCG

FAM92A epi.PCG

LPCAT3 epi.PCG

ASF1B epi.PCG

UQCRB epi.PCG

ARL17B epi.PCG

CAMSAP2 epi.PCG

SMU1 epi.PCG

ZNF625 epi.PCG

MOB1A epi.PCG

ERICH6B epi.PCG

EFNA5 epi.PCG

GJB3 epi.PCG

MORN1 epi.PCG

LTO1 epi.PCG

YAF2 epi.PCG

FKBP11 epi.PCG

RNF26 epi.PCG

LBH epi.PCG

NDUFAF6 epi.PCG

MRPS35 epi.PCG

FTL epi.PCG

B3GALNT2 epi.PCG

DESI2 epi.PCG

CLASRP epi.PCG

COL3A1 epi.PCG

NAA16 epi.PCG

PTAFR epi.PCG

EDIL3 epi.PCG

ITFG2 epi.PCG

FSCN1 epi.PCG

ATM epi.PCG

CEP95 epi.PCG

FER1L5 epi.PCG

DPH7 epi.PCG

MREG epi.PCG

MAPRE1 epi.PCG

NDUFA10 epi.PCG

LHFPL5 epi.PCG

CNTNAP3B epi.PCG

CCDC57 epi.PCG

C9orf64 epi.PCG

TTC17 epi.PCG

WASL epi.PCG

SLC35E2A epi.PCG

MAATS1 epi.PCG

PERP epi.PCG

NXT1 epi.PCG

LMNB1 epi.PCG

ARHGEF4 epi.PCG

TICAM1 epi.PCG

PLA2G6 epi.PCG

PIGF epi.PCG

TSEN2 epi.PCG

LMNB2 epi.PCG

COL1A1 epi.PCG

B3GAT2 epi.PCG

COBLL1 epi.PCG

GNAI3 epi.PCG

KIF11 epi.PCG

SDC1 epi.PCG

VKORC1L1 epi.PCG

HTRA3 epi.PCG

FXYD1 epi.PCG

CENPW epi.PCG

TVP23C epi.PCG

GCSH epi.PCG

AOC1 epi.PCG

CCDC110 epi.PCG

ZNF416 epi.PCG

HLA-DRA epi.PCG

SKP1 epi.PCG

ANKRD62 epi.PCG

BASP1 epi.PCG

OSGIN2 epi.PCG

SHISA2 epi.PCG

NRAS epi.PCG

RTP4 epi.PCG

IGFBP3 epi.PCG

NT5E epi.PCG

PPIP5K2 epi.PCG

DENND6A epi.PCG

RPL39 epi.PCG

TIMM21 epi.PCG

CAVIN1 epi.PCG

RNASEH2B epi.PCG

STAG3 epi.PCG

TPST2 epi.PCG

MDP1 epi.PCG

HK1 epi.PCG

KLF16 epi.PCG

DNHD1 epi.PCG

NBPF1 epi.PCG

PTPRCAP epi.PCG

MRPL47 epi.PCG

ZNF273 epi.PCG

SERPINB9 epi.PCG

RND3 epi.PCG

MRPS33 epi.PCG

MCOLN3 epi.PCG

FAM184A epi.PCG

CLCN7 epi.PCG

PLIN3 epi.PCG

CCDC30 epi.PCG

TOR4A epi.PCG

MYO19 epi.PCG

ZNF526 epi.PCG

MPI epi.PCG

PAQR8 epi.PCG

UBE2C epi.PCG

VAMP3 epi.PCG

ZNF519 epi.PCG

TCHP epi.PCG

COQ8A epi.PCG

DPH3 epi.PCG

POLE epi.PCG

YTHDF1 epi.PCG

MRGBP epi.PCG

IFIT5 epi.PCG

FBXL8 epi.PCG

THBS2 epi.PCG

RAB31 epi.PCG

CRYZL1 epi.PCG

ADAT2 epi.PCG

CYP46A1 epi.PCG

CEP83 epi.PCG

L3MBTL1 epi.PCG

TBCD epi.PCG

TMEM218 epi.PCG

ACTN2 epi.PCG

SIX5 epi.PCG

DEPTOR epi.PCG

SH3YL1 epi.PCG

STK33 epi.PCG

LTBP1 epi.PCG

TNFRSF1B epi.PCG

ACAA1 epi.PCG

UBE3D epi.PCG

RAC2 epi.PCG

PFKP epi.PCG

DYRK2 epi.PCG

CNOT6 epi.PCG

ANTXR1 epi.PCG

METTL3 epi.PCG

TRMT10C epi.PCG

MMD epi.PCG

RPP21 epi.PCG

ZNF20 epi.PCG

FAM13A epi.PCG

SPAST epi.PCG

SLC16A1 epi.PCG

CNOT1 epi.PCG

ARMT1 epi.PCG

ASPHD2 epi.PCG

OBSCN epi.PCG

ICE2 epi.PCG

COMMD3 epi.PCG

RPS15A epi.PCG

CEP55 epi.PCG

SDC3 epi.PCG

TXNL4A epi.PCG

TMPRSS4 epi.PCG

ERICH1 epi.PCG

BCS1L epi.PCG

AXL epi.PCG

TMEM120B epi.PCG

ZMPSTE24 epi.PCG

SULF2 epi.PCG

KCTD11 epi.PCG

RAB14 epi.PCG

PHF13 epi.PCG

TATDN1 epi.PCG

TOP2A epi.PCG

ZNF260 epi.PCG

LYRM4 epi.PCG

BTBD10 epi.PCG

SHPK epi.PCG

RALBP1 epi.PCG

STK25 epi.PCG

NKTR epi.PCG

COQ10B epi.PCG

ZNF839 epi.PCG

HNRNPH1 epi.PCG

PTBP2 epi.PCG

FZD7 epi.PCG

WDPCP epi.PCG

MCM3 epi.PCG

ANAPC1 epi.PCG

MGAT2 epi.PCG

PJA1 epi.PCG

CDC42EP2 epi.PCG

TAF1D epi.PCG

AKT2 epi.PCG

FGF12 epi.PCG

SF3B4 epi.PCG

CNPY2 epi.PCG

TRIM52 epi.PCG

HOXB7 epi.PCG

C11orf98 epi.PCG

PRAG1 epi.PCG

MAML2 epi.PCG

BNIP3 epi.PCG

HOOK2 epi.PCG

AD000671.1 epi.PCG

TTC21A epi.PCG

ZNF558 epi.PCG

ENY2 epi.PCG

NSMCE3 epi.PCG

COQ10A epi.PCG

LRMDA epi.PCG

PARP6 epi.PCG

CDC5L epi.PCG

MMP11 epi.PCG

FAM153A epi.PCG

TMTC3 epi.PCG

AP002495.1 epi.PCG

MRPS10 epi.PCG

PAM16 epi.PCG

MRPS11 epi.PCG

NQO1 epi.PCG

AGBL3 epi.PCG

MAP1A epi.PCG

KIF26B epi.PCG

KPNA2 epi.PCG

SLC22A5 epi.PCG

ZMAT2 epi.PCG

ACTR3B epi.PCG

CDYL epi.PCG

CSTB epi.PCG

FOXQ1 epi.PCG

RBM20 epi.PCG

TOP1 epi.PCG

CDH23 epi.PCG

KCNN4 epi.PCG

SHPRH epi.PCG

COL5A1 epi.PCG

NAGK epi.PCG

AL121845.3 epi.PCG

CDCA8 epi.PCG

NDUFS7 epi.PCG

SLC25A27 epi.PCG

KIF4A epi.PCG

TIMP1 epi.PCG

CENPT epi.PCG

AC010547.4 epi.PCG

CXXC1 epi.PCG

CCDC149 epi.PCG

PLEKHO2 epi.PCG

ERG28 epi.PCG

MRPL22 epi.PCG

PTCD3 epi.PCG

PYGB epi.PCG

MYOF epi.PCG

DEXI epi.PCG

VASP epi.PCG

PTTG1IP epi.PCG

FBXO34 epi.PCG

SULF1 epi.PCG

MYADM epi.PCG

ENO3 epi.PCG

ZNHIT3 epi.PCG

PDCD4 epi.PCG

TTYH1 epi.PCG

HENMT1 epi.PCG

LSMEM1 epi.PCG

ELK1 epi.PCG

MINDY4 epi.PCG

TMEM87B epi.PCG

INTS10 epi.PCG

ATP6V1G2-DDX39B epi.PCG

PMFBP1 epi.PCG

MMP28 epi.PCG

ZDHHC5 epi.PCG

PROB1 epi.PCG

MCF2L epi.PCG

TMEM45B epi.PCG

ZNF326 epi.PCG

CDCA4 epi.PCG

MCM9 epi.PCG

DMTF1 epi.PCG

PRRG4 epi.PCG

DNTTIP1 epi.PCG

CSAD epi.PCG

SH3PXD2A epi.PCG

MKI67 epi.PCG

ZNF577 epi.PCG

PPAN epi.PCG

SKIL epi.PCG

NSDHL epi.PCG

HSD17B4 epi.PCG

TGFBR1 epi.PCG

LRRC8E epi.PCG

GRAMD1C epi.PCG

ELP2 epi.PCG

GNS epi.PCG

RTEL1-TNFRSF6B epi.PCG

ZNF559-ZNF177 epi.PCG

POP4 epi.PCG

ACSL5 epi.PCG

ZMYM6 epi.PCG

INTS11 epi.PCG

MRPL44 epi.PCG

HLA-DQB2 epi.PCG

RIOX2 epi.PCG

KRT19 epi.PCG

DCK epi.PCG

HLA-E epi.PCG

BNIP3L epi.PCG

PLAU epi.PCG

CDC20 epi.PCG

METAP1D epi.PCG

PDZD11 epi.PCG

ZBTB2 epi.PCG

RPS10-NUDT3 epi.PCG

UMOD epi.PCG

PHKA2 epi.PCG

ALDH1L1 epi.PCG

GTF2H5 epi.PCG

CEACAM6 epi.PCG

ABT1 epi.PCG

CCDC159 epi.PCG

RTRAF epi.PCG

PYGO2 epi.PCG

PRELID3B epi.PCG

RFLNA epi.PCG

CKAP2 epi.PCG

RPAIN epi.PCG

IPPK epi.PCG

ARNTL2 epi.PCG

ZSCAN2 epi.PCG

ARMCX4 epi.PCG

MPV17 epi.PCG

VXN epi.PCG

C12orf76 epi.PCG

ACSM3 epi.PCG

ZNF543 epi.PCG

SERINC3 epi.PCG

CEP63 epi.PCG

PSTK epi.PCG

SFXN2 epi.PCG

CHSY1 epi.PCG

CHN2 epi.PCG

CBFA2T3 epi.PCG

COL8A2 epi.PCG

DEGS1 epi.PCG

PEX5L epi.PCG

FXYD2 epi.PCG

RRAS epi.PCG

PRR5-ARHGAP8 epi.PCG

ADAM19 epi.PCG

DNAH7 epi.PCG

TMCC3 epi.PCG

FOXS1 epi.PCG

HECA epi.PCG

CARD8 epi.PCG

PFDN2 epi.PCG

RBBP5 epi.PCG

CLIC1 epi.PCG

NAB2 epi.PCG

KCTD12 epi.PCG

MDK epi.PCG

BST2 epi.PCG

TTLL7 epi.PCG

GNG12 epi.PCG

S100A16 epi.PCG

C19orf33 epi.PCG

DENND10 epi.PCG

ACBD3 epi.PCG

ZNF451 epi.PCG

COG8 epi.PCG

EIF3M epi.PCG

PRMT7 epi.PCG

OGG1 epi.PCG

SCART1 epi.PCG

FUNDC1 epi.PCG

ZSWIM8 epi.PCG

AP2B1 epi.PCG

NADSYN1 epi.PCG

GDF10 epi.PCG

ACSL4 epi.PCG

CYP51A1 epi.PCG

AL157935.2 epi.PCG

LARP1B epi.PCG

CDS1 epi.PCG

SIDT2 epi.PCG

CMTM3 epi.PCG

ARL3 epi.PCG

PKD1 epi.PCG

PASK epi.PCG

NAA10 epi.PCG

IFNGR2 epi.PCG

ACAT1 epi.PCG

ZNF286A epi.PCG

ZNF181 epi.PCG

SH3RF1 epi.PCG

SYNE2 epi.PCG

RAD51C epi.PCG

GSTZ1 epi.PCG

GRB2 epi.PCG

SLC45A3 epi.PCG

HK2 epi.PCG

BMT2 epi.PCG

FBXO28 epi.PCG

STK26 epi.PCG

DPY19L1 epi.PCG

ZNF765 epi.PCG

INTS8 epi.PCG

SMIM7 epi.PCG

FAN1 epi.PCG

SMIM15 epi.PCG

SMIM11A epi.PCG

PELO epi.PCG

ZCCHC3 epi.PCG

PPP1R3B epi.PCG

PTPRJ epi.PCG

GPSM3 epi.PCG

KIAA0232 epi.PCG

TBC1D30 epi.PCG

AC009690.1 epi.PCG

APBB3 epi.PCG

S100A11 epi.PCG

TRAPPC12 epi.PCG

IL3RA epi.PCG

ARID5B epi.PCG

SPCS2 epi.PCG

ASPSCR1 epi.PCG

C16orf91 epi.PCG

MASP2 epi.PCG

LUZP1 epi.PCG

TSR2 epi.PCG

GJB2 epi.PCG

GOLGA8A epi.PCG

USP8 epi.PCG

SORBS2 epi.PCG

PPP2R2D epi.PCG

PLPP5 epi.PCG

PCBP2 epi.PCG

SEMA7A epi.PCG

PRSS16 epi.PCG

ATXN2 epi.PCG

HLA-B epi.PCG

REPS1 epi.PCG

RAB32 epi.PCG

PDRG1 epi.PCG

GRPEL2 epi.PCG

STK36 epi.PCG

CARS2 epi.PCG

CEACAM1 epi.PCG

EMC6 epi.PCG

BMS1 epi.PCG

RNF215 epi.PCG

HPSE epi.PCG

PRDM1 epi.PCG

POC1A epi.PCG

ANKRD50 epi.PCG

MIPOL1 epi.PCG

MGLL epi.PCG

OBSL1 epi.PCG

SMARCE1 epi.PCG

FAHD2B epi.PCG

UBAP1 epi.PCG

TMEM43 epi.PCG

PNISR epi.PCG

TRIM3 epi.PCG

SUGT1 epi.PCG

PACS2 epi.PCG

SEMA3C epi.PCG

ENOPH1 epi.PCG

PROCR epi.PCG

ZKSCAN5 epi.PCG

IZUMO4 epi.PCG

ICAM3 epi.PCG

PLCXD2 epi.PCG

USP44 epi.PCG

CEP164 epi.PCG

NDC80 epi.PCG

RFTN1 epi.PCG

NGLY1 epi.PCG

TIPRL epi.PCG

PABPC4 epi.PCG

ZNF90 epi.PCG

NDUFA13 epi.PCG

HM13 epi.PCG

PAFAH1B2 epi.PCG

MTA1 epi.PCG

LRRC42 epi.PCG

SRSF10 epi.PCG

TMSB15B epi.PCG

SPG7 epi.PCG

CAP1 epi.PCG

GSK3B epi.PCG

ZYG11B epi.PCG

GNMT epi.PCG

UBE2F epi.PCG

NBPF15 epi.PCG

ZNF775 epi.PCG

SEPHS2 epi.PCG

TSGA10 epi.PCG

IGFLR1 epi.PCG

HERC2 epi.PCG

LIG3 epi.PCG

KCNN2 epi.PCG

ITGAE epi.PCG

CRIPT epi.PCG

TRAF3 epi.PCG

PPIE epi.PCG

PGAM2 epi.PCG

IL27RA epi.PCG

AKIRIN2 epi.PCG

KIF5B epi.PCG

PDCD2 epi.PCG

C1orf216 epi.PCG

GJA1 epi.PCG

SLC30A1 epi.PCG

EIF2AK2 epi.PCG

CETN2 epi.PCG

PRNP epi.PCG

GSTO2 epi.PCG

GPRC5A epi.PCG

MSLN epi.PCG

BTBD1 epi.PCG

CBR3 epi.PCG

GALNT5 epi.PCG

ZNF431 epi.PCG

CYHR1 epi.PCG

CCNA2 epi.PCG

APOBEC3D epi.PCG

FERMT1 epi.PCG

RCOR3 epi.PCG

CBFB epi.PCG

KIF2C epi.PCG

WDR59 epi.PCG

RAB3IP epi.PCG

SAMD8 epi.PCG

FAM220A epi.PCG

DYNLL2 epi.PCG

PLEKHB2 epi.PCG

LEO1 epi.PCG

MEIS1 epi.PCG

CHRM3 epi.PCG

RAB20 epi.PCG

C5orf63 epi.PCG

HS3ST1 epi.PCG

SEC11C epi.PCG

MTHFS epi.PCG

BPHL epi.PCG

TRIM21 epi.PCG

GCC1 epi.PCG

MAF epi.PCG

PCNX4 epi.PCG

JARID2 epi.PCG

NIT2 epi.PCG

GRK4 epi.PCG

ANAPC4 epi.PCG

CUL1 epi.PCG

PDK2 epi.PCG

ZNF780A epi.PCG

ZNF385A epi.PCG

LRRC37B epi.PCG

RABGGTB epi.PCG

GLE1 epi.PCG

WNK2 epi.PCG

TKFC epi.PCG

ARMCX6 epi.PCG

TTC9 epi.PCG

HBS1L epi.PCG

CCDC141 epi.PCG

FBRSL1 epi.PCG

IMPA2 epi.PCG

GIMAP1-GIMAP5 epi.PCG

SNRPB epi.PCG

TRERF1 epi.PCG

SYTL1 epi.PCG

CCDC85C epi.PCG

RBIS epi.PCG

FBXO16 epi.PCG

ARHGEF18 epi.PCG

GATB epi.PCG

RAB43 epi.PCG

C11orf1 epi.PCG

NFE2L1 epi.PCG

BROX epi.PCG

MAP4K4 epi.PCG

TRIM38 epi.PCG

GUCA1C epi.PCG

FAM222B epi.PCG

ATP6V1A epi.PCG

FAM193B epi.PCG

CTC1 epi.PCG

FOXL1 epi.PCG

WBP4 epi.PCG

ARFIP1 epi.PCG

LPAR3 epi.PCG

FAAP20 epi.PCG

PTGER2 epi.PCG

ABCG1 epi.PCG

SARDH epi.PCG

C16orf95 epi.PCG

TP53INP2 epi.PCG

COL8A1 epi.PCG

ARL6IP5 epi.PCG

SRSF11 epi.PCG

NOMO1 epi.PCG

RHBDL2 epi.PCG

TMEM262 epi.PCG

FNTA epi.PCG

FAM83E epi.PCG

NRP2 epi.PCG

MAT1A epi.PCG

RYR2 epi.PCG

ACSF3 epi.PCG

EIF2B4 epi.PCG

STK4 epi.PCG

SYPL2 epi.PCG

GRN epi.PCG

IFNAR1 epi.PCG

KIFC1 epi.PCG

ZNF384 epi.PCG

COL4A1 epi.PCG

SLC1A2 epi.PCG

TMEM116 epi.PCG

PABPC1L epi.PCG

ILRUN epi.PCG

SCAPER epi.PCG

ERI2 epi.PCG

ACER3 epi.PCG

SATB1 epi.PCG

ATP6V1F epi.PCG

WDR90 epi.PCG

CDCP1 epi.PCG

EPN1 epi.PCG

COL4A2 epi.PCG

SOX4 epi.PCG

WDR19 epi.PCG

MDFI epi.PCG

TRMT12 epi.PCG

FOXF2 epi.PCG

LSM12 epi.PCG

PPP1R9B epi.PCG

ZSWIM4 epi.PCG

DLGAP5 epi.PCG

VMA21 epi.PCG

RHOG epi.PCG

RPL37A epi.PCG

WDR61 epi.PCG

MSANTD3-TMEFF1 epi.PCG

ANK3 epi.PCG

ETV6 epi.PCG

RCHY1 epi.PCG

IER5 epi.PCG

E2F1 epi.PCG

GPC6 epi.PCG

ALG13 epi.PCG

CBL epi.PCG

TNFAIP6 epi.PCG

METTL17 epi.PCG

FBF1 epi.PCG

USP34 epi.PCG

GIMAP2 epi.PCG

GTF3C4 epi.PCG

IFIT2 epi.PCG

ZNF599 epi.PCG

ZNF852 epi.PCG

PTTG1 epi.PCG

RXYLT1 epi.PCG

NUDT15 epi.PCG

NOL12 epi.PCG

ZCCHC8 epi.PCG

BAG4 epi.PCG

SYT13 epi.PCG

WDR91 epi.PCG

ABCA10 epi.PCG

EZR epi.PCG

PLEKHF2 epi.PCG

CDH3 epi.PCG

PAIP2B epi.PCG

CDA epi.PCG

EXOC7 epi.PCG

PRPF6 epi.PCG

PNLIPRP1 epi.PCG

NUSAP1 epi.PCG

MMGT1 epi.PCG

RACGAP1 epi.PCG

ATF7IP2 epi.PCG

HPN epi.PCG

INTS5 epi.PCG

IFI6 epi.PCG

EPB41L4B epi.PCG

NDUFA8 epi.PCG

LEF1 epi.PCG

SPCS1 epi.PCG

HDAC6 epi.PCG

FSBP epi.PCG

TIMP3 epi.PCG

NRDE2 epi.PCG

PFN1 epi.PCG

SLC16A10 epi.PCG

GARNL3 epi.PCG

OAS3 epi.PCG

LETMD1 epi.PCG

MTA3 epi.PCG

HMGA1 epi.PCG

HOXB5 epi.PCG

TRIOBP epi.PCG

LRRC8A epi.PCG

KIF20A epi.PCG

PLAUR epi.PCG

CXCR4 epi.PCG

IL12RB2 epi.PCG

DMXL2 epi.PCG

MRPL15 epi.PCG

CCDC14 epi.PCG

AP1S1 epi.PCG

CYP2S1 epi.PCG

STYK1 epi.PCG

NCEH1 epi.PCG

CDK1 epi.PCG

H2AFY2 epi.PCG

MAGIX epi.PCG

MICU1 epi.PCG

POLN epi.PCG

SLC44A4 epi.PCG

SNX9 epi.PCG

CRKL epi.PCG

HIP1 epi.PCG

COA8 epi.PCG

SHOC2 epi.PCG

NBPF12 epi.PCG

BAZ1B epi.PCG

SKI epi.PCG

CFAP70 epi.PCG

TGS1 epi.PCG

ETV3 epi.PCG

TMEM185B epi.PCG

FBXW7 epi.PCG

TLCD2 epi.PCG

DST epi.PCG

SYT11 epi.PCG

BUD13 epi.PCG

BAZ2B epi.PCG

CD109 epi.PCG

LXN epi.PCG

AHI1 epi.PCG

SOGA1 epi.PCG

MBOAT2 epi.PCG

CXorf40A epi.PCG

BPGM epi.PCG

BOLA3 epi.PCG

MTERF2 epi.PCG

NR2C1 epi.PCG

GON4L epi.PCG

LRRC20 epi.PCG

SLFN5 epi.PCG

N4BP3 epi.PCG

PLXDC2 epi.PCG

ANKMY1 epi.PCG

NUCB2 epi.PCG

CCNB2 epi.PCG

PPP1R14C epi.PCG

PRR15 epi.PCG

CLHC1 epi.PCG

CYP27A1 epi.PCG

TIGAR epi.PCG

KRR1 epi.PCG

AMHR2 epi.PCG

GANC epi.PCG

HDAC2 epi.PCG

ZNF638 epi.PCG

TWISTNB epi.PCG

ETS2 epi.PCG

COL1A2 epi.PCG

TPRG1L epi.PCG

MARCKS epi.PCG

RBM39 epi.PCG

GRB14 epi.PCG

SPARC epi.PCG

POGLUT2 epi.PCG

ACBD5 epi.PCG

FAM228B epi.PCG

KDM2B epi.PCG

ENTPD1 epi.PCG

POGLUT3 epi.PCG

RNF139 epi.PCG

PAK6 epi.PCG

CDCA5 epi.PCG

RBM18 epi.PCG

ACBD6 epi.PCG

CSTF2T epi.PCG

CYB561A3 epi.PCG

ATPAF2 epi.PCG

MPLKIP epi.PCG

SEM1 epi.PCG

ZNF76 epi.PCG

MARK3 epi.PCG

BAK1 epi.PCG

CHST12 epi.PCG

RNF214 epi.PCG

SFI1 epi.PCG

ZNF354B epi.PCG

NAA60 epi.PCG

AVPI1 epi.PCG

PHF20L1 epi.PCG

LNPK epi.PCG

ZNF433 epi.PCG

RAB7A epi.PCG

HERC1 epi.PCG

DUS1L epi.PCG

WDR12 epi.PCG

NDC1 epi.PCG

CSGALNACT2 epi.PCG

ACYP2 epi.PCG

RBM15 epi.PCG

NCAPH epi.PCG

SHROOM3 epi.PCG

VAMP7 epi.PCG

TMEM258 epi.PCG

MLYCD epi.PCG

PTPMT1 epi.PCG

SAP30BP epi.PCG

RARB epi.PCG

VEGFC epi.PCG

INTS4 epi.PCG

LSM3 epi.PCG

EIF3L epi.PCG

NUFIP2 epi.PCG

GNPDA1 epi.PCG

TGOLN2 epi.PCG

ALKAL2 epi.PCG

DIPK1A epi.PCG

RNF130 epi.PCG

PBXIP1 epi.PCG

COMP epi.PCG

UBXN10 epi.PCG

LRRC27 epi.PCG

F11 epi.PCG

EGLN2 epi.PCG

GLO1 epi.PCG

TRPV6 epi.PCG

DNAJB6 epi.PCG

WDR82 epi.PCG

FGFR1 epi.PCG

MUL1 epi.PCG

PATL1 epi.PCG

HEXA epi.PCG

ADAMTS14 epi.PCG

SUPT20H epi.PCG

SLC2A1 epi.PCG

PAX2 epi.PCG

YBX3 epi.PCG

KCNIP3 epi.PCG

WDR44 epi.PCG

SERINC4 epi.PCG

PRPSAP1 epi.PCG

LSM5 epi.PCG

KLF5 epi.PCG

LTBP2 epi.PCG

PPHLN1 epi.PCG

SLPI epi.PCG

TEN1-CDK3 epi.PCG

RAB35 epi.PCG

PELI1 epi.PCG

SLC22A3 epi.PCG

KRT17 epi.PCG

IDH3A epi.PCG

KIAA1328 epi.PCG

DNASE1 epi.PCG

SMIM10L1 epi.PCG

DUSP6 epi.PCG

AL133352.1 epi.PCG

TTN epi.PCG

GSR epi.PCG

CDK5RAP3 epi.PCG

TRIM66 epi.PCG

HDDC2 epi.PCG

NSMCE4A epi.PCG

ERCC5 epi.PCG

PCM1 epi.PCG

HGS epi.PCG

SLC16A4 epi.PCG

RCOR1 epi.PCG

LUC7L epi.PCG

FASTKD5 epi.PCG

MMP12 epi.PCG

ABI1 epi.PCG

CTSS epi.PCG

TMEM173 epi.PCG

RBM17 epi.PCG

PRDX5 epi.PCG

INS-IGF2 epi.PCG

ZNF184 epi.PCG

LIN37 epi.PCG

IGFBP4 epi.PCG

FEN1 epi.PCG

VCPIP1 epi.PCG

LIPH epi.PCG

TTLL1 epi.PCG

MFHAS1 epi.PCG

C5orf51 epi.PCG

ONECUT1 epi.PCG

ARHGAP31 epi.PCG

FOXP2 epi.PCG

HTATSF1 epi.PCG

PLA2G7 epi.PCG

CENPS epi.PCG

IRAK2 epi.PCG

CDKN2D epi.PCG

SNX18 epi.PCG

HNRNPAB epi.PCG

KHDRBS1 epi.PCG

PLAAT4 epi.PCG

NOXO1 epi.PCG

OSBPL10 epi.PCG

SUMO1 epi.PCG

ZWINT epi.PCG

EGR2 epi.PCG

IGFBP5 epi.PCG

NEMF epi.PCG

MEA1 epi.PCG

SCD epi.PCG

CCDC103 epi.PCG

PRTFDC1 epi.PCG

DIP2A epi.PCG

AP002990.1 epi.PCG

BUB1 epi.PCG

RIPOR3 epi.PCG

IRF2 epi.PCG

UBE2L6 epi.PCG

ZNF207 epi.PCG

S100P epi.PCG

FNDC1 epi.PCG

CALR epi.PCG

CDC73 epi.PCG

NAB1 epi.PCG

IL1RN epi.PCG

C1orf54 epi.PCG

UQCC2 epi.PCG

APOL2 epi.PCG

FZD2 epi.PCG

TUBGCP4 epi.PCG

RNF220 epi.PCG

CATSPERG epi.PCG

SLC25A48 epi.PCG

KIF9 epi.PCG

PEX11B epi.PCG

KRBA2 epi.PCG

NES epi.PCG

CD55 epi.PCG

CCL18 epi.PCG

CCDC7 epi.PCG

ZNF438 epi.PCG

RPGR epi.PCG

XXYLT1 epi.PCG

TNPO3 epi.PCG

TSC2 epi.PCG

FGGY epi.PCG

OGDH epi.PCG

HARS epi.PCG

GBP4 epi.PCG

PLPP4 epi.PCG

CTSZ epi.PCG

ARL15 epi.PCG

WBP2NL epi.PCG

AAR2 epi.PCG

SOCS4 epi.PCG

RAB7B epi.PCG

CORO1C epi.PCG

MDN1 epi.PCG

CHRD epi.PCG

CTCF epi.PCG

DNAJC7 epi.PCG

ZBTB6 epi.PCG

HBEGF epi.PCG

USP5 epi.PCG

AMIGO2 epi.PCG

CGAS epi.PCG

IQGAP3 epi.PCG

SRF epi.PCG

MED17 epi.PCG

PSAP epi.PCG

TRIM32 epi.PCG

SH3RF3 epi.PCG

ZNF322 epi.PCG

ARHGAP42 epi.PCG

ORMDL1 epi.PCG

GCOM1 epi.PCG

PROS1 epi.PCG

ZNF777 epi.PCG

MSN epi.PCG

PCDH7 epi.PCG

MSH5-SAPCD1 epi.PCG

MED29 epi.PCG

EHMT1 epi.PCG

TRIM41 epi.PCG

TAGLN2 epi.PCG

PHF23 epi.PCG

JUP epi.PCG

CENPC epi.PCG

LMF1 epi.PCG

FAIM epi.PCG

TAF7 epi.PCG

LAPTM5 epi.PCG

RAB10 epi.PCG

IFI35 epi.PCG

CYP2R1 epi.PCG

GMPPB epi.PCG

CD93 epi.PCG

ZNF665 epi.PCG

DNAJC17 epi.PCG

SFRP4 epi.PCG

GCNT3 epi.PCG

SLC17A5 epi.PCG

SLC37A3 epi.PCG

AC010422.6 epi.PCG

GNE epi.PCG

C3orf38 epi.PCG

PRAF2 epi.PCG

KLF15 epi.PCG

TRAM2 epi.PCG

CTDSPL epi.PCG

KIRREL2 epi.PCG

AKIRIN1 epi.PCG

AGGF1 epi.PCG

LY96 epi.PCG

NDUFB1 epi.PCG

GEM epi.PCG

RNF145 epi.PCG

GALT epi.PCG

KCNK1 epi.PCG

SNX27 epi.PCG

NFKBIE epi.PCG

ZNF598 epi.PCG

LAPTM4B epi.PCG

SOCS5 epi.PCG

STRADB epi.PCG

PAQR7 epi.PCG

UNG epi.PCG

TPGS1 epi.PCG

ZNF10 epi.PCG

RNGTT epi.PCG

ZNF281 epi.PCG

ELOVL5 epi.PCG

DALRD3 epi.PCG

ATP9A epi.PCG

HNRNPH2 epi.PCG

SPIN2B epi.PCG

NEK2 epi.PCG

TNFAIP8L1 epi.PCG

ARF3 epi.PCG

SEMA6D epi.PCG

CHST14 epi.PCG

EIF2D epi.PCG

NYNRIN epi.PCG

SMC2 epi.PCG

TRMT44 epi.PCG

SUFU epi.PCG

CLCNKA epi.PCG

RUNX1 epi.PCG

BHLHE41 epi.PCG

PPARD epi.PCG

ECT2 epi.PCG

ZNF677 epi.PCG

POM121 epi.PCG

TPGS2 epi.PCG

CD74 epi.PCG

DCT epi.PCG

TYRO3 epi.PCG

TMEM150A epi.PCG

HAGH epi.PCG

AL031708.1 epi.PCG

GPATCH11 epi.PCG

PGM3 epi.PCG

NFS1 epi.PCG

HTT epi.PCG

MOCS3 epi.PCG

VCAN epi.PCG

ABHD17C epi.PCG

LIF epi.PCG

AFMID epi.PCG

SERTAD4 epi.PCG

DSG2 epi.PCG

PARP3 epi.PCG

PDGFB epi.PCG

PTPRA epi.PCG

SLC25A3 epi.PCG

HPS6 epi.PCG

CST7 epi.PCG

FOXF1 epi.PCG

GMIP epi.PCG

MRPL42 epi.PCG

S100A10 epi.PCG

LRTOMT epi.PCG

SRPX2 epi.PCG

SLC44A1 epi.PCG

GPD2 epi.PCG

SAPCD2 epi.PCG

HMOX1 epi.PCG

MOB3A epi.PCG

BRCC3 epi.PCG

KBTBD2 epi.PCG

PEX1 epi.PCG

SLC51A epi.PCG

GLI2 epi.PCG

FRZB epi.PCG

FADS3 epi.PCG

SENP3 epi.PCG

TRAFD1 epi.PCG

WDR74 epi.PCG

RNASEL epi.PCG

VRK3 epi.PCG

TMEM158 epi.PCG

OTUD7A epi.PCG

TGFB1 epi.PCG

MAP3K2 epi.PCG

EPC1 epi.PCG

PIP4K2A epi.PCG

PMAIP1 epi.PCG

ZC3H8 epi.PCG

FXYD5 epi.PCG

APMAP epi.PCG

TRIM15 epi.PCG

MAP6D1 epi.PCG

TNFRSF10A epi.PCG

SYCP2L epi.PCG

NPHP1 epi.PCG

CCN4 epi.PCG

ADAM9 epi.PCG

ZNF578 epi.PCG

VAPA epi.PCG

FGD1 epi.PCG

CPXM1 epi.PCG

TRIM56 epi.PCG

LAMA4 epi.PCG

PLCB3 epi.PCG

PCED1B epi.PCG

SRGN epi.PCG

UBTD1 epi.PCG

ZFP1 epi.PCG

HEXD epi.PCG

STXBP6 epi.PCG

TEAD3 epi.PCG

ZCCHC17 epi.PCG

LNPEP epi.PCG

C11orf68 epi.PCG

NME2 epi.PCG

PPP1R8 epi.PCG

MFN2 epi.PCG

GTF2A1 epi.PCG

VPS51 epi.PCG

DTX3L epi.PCG

AHNAK epi.PCG

RUFY3 epi.PCG

SLC35G1 epi.PCG

NEURL4 epi.PCG

SYNDIG1 epi.PCG

UNKL epi.PCG

OAS2 epi.PCG

BCL11B epi.PCG

EPB41L5 epi.PCG

USP3 epi.PCG

KANSL1 epi.PCG

PCDH1 epi.PCG

PSMB8 epi.PCG

DTL epi.PCG

SPDEF epi.PCG

MR1 epi.PCG

CRABP2 epi.PCG

DDX55 epi.PCG

FMOD epi.PCG

AFDN epi.PCG

ZDHHC11B epi.PCG

ZKSCAN4 epi.PCG

HAAO epi.PCG

PPT1 epi.PCG

AAK1 epi.PCG

ABCD4 epi.PCG

ZNF655 epi.PCG

AURKA epi.PCG

CNPY4 epi.PCG

HNRNPA1P48 epi.PCG

XPNPEP1 epi.PCG

ANLN epi.PCG

PCNA epi.PCG

IFIT3 epi.PCG

PHLDB1 epi.PCG

SLC26A10 epi.PCG

PARP4 epi.PCG

RHOA epi.PCG

SMIM13 epi.PCG

NOP10 epi.PCG

HYKK epi.PCG

BCAS2 epi.PCG

CUL3 epi.PCG

SPTY2D1 epi.PCG

RWDD1 epi.PCG

PRRX2 epi.PCG

ASNSD1 epi.PCG

SPRYD4 epi.PCG

AEBP1 epi.PCG

GAL3ST4 epi.PCG

NDRG3 epi.PCG

PRCD epi.PCG

ZNF789 epi.PCG

RIT1 epi.PCG

RSRC2 epi.PCG

TSN epi.PCG

TTC1 epi.PCG

PPOX epi.PCG

STRN epi.PCG

GPX8 epi.PCG

DGKH epi.PCG

PLAT epi.PCG

HOXC4 epi.PCG

NPAS2 epi.PCG

CASTOR1 epi.PCG

TCEAL9 epi.PCG

SLC22A18AS epi.PCG

MYL12B epi.PCG

VSTM4 epi.PCG

SRRM2 epi.PCG

COL17A1 epi.PCG

GOLM1 epi.PCG

PINX1 epi.PCG

CCDC196 epi.PCG

ADARB2 epi.PCG

ITGB5 epi.PCG

HAPLN3 epi.PCG

ACCS epi.PCG

KCTD20 epi.PCG

CCNL1 epi.PCG

FAM76B epi.PCG

ALDH1B1 epi.PCG

USP38 epi.PCG

CAMK2N2 epi.PCG

F2RL2 epi.PCG

MPRIP epi.PCG

GGNBP2 epi.PCG

TFDP1 epi.PCG

BTBD9 epi.PCG

ADAMTS12 epi.PCG

RAB29 epi.PCG

TOR1B epi.PCG

DDHD2 epi.PCG

PPP1R16A epi.PCG

MRC2 epi.PCG

USHBP1 epi.PCG

DKK3 epi.PCG

CXorf38 epi.PCG

ARL2BP epi.PCG

HLA-DOA epi.PCG

EPS8L3 epi.PCG

SLC25A36 epi.PCG

DUSP5 epi.PCG

GMNN epi.PCG

SNN epi.PCG

ETS1 epi.PCG

TAPBP epi.PCG

LPA epi.PCG

LY86 epi.PCG

TMEM54 epi.PCG

LCP1 epi.PCG

RANBP17 epi.PCG

ADAMTS2 epi.PCG

OAS1 epi.PCG

DDX11 epi.PCG

RAD54B epi.PCG

PRRG1 epi.PCG

METTL7B epi.PCG

TMED6 epi.PCG

GORASP1 epi.PCG

MFAP1 epi.PCG

SFRP2 epi.PCG

PPP1CB epi.PCG

RUNX2 epi.PCG

MRPS22 epi.PCG

ORAI1 epi.PCG

MZT2A epi.PCG

CAPN5 epi.PCG

WNT2 epi.PCG

PIM2 epi.PCG

ZSWIM3 epi.PCG

ZSCAN30 epi.PCG

SGK3 epi.PCG

PSMC6 epi.PCG

LATS2 epi.PCG

SP2 epi.PCG

ARL6IP1 epi.PCG

MTUS2 epi.PCG

PPP3R1 epi.PCG

HMMR epi.PCG

RPA2 epi.PCG

INCA1 epi.PCG

MAPK6 epi.PCG

CPD epi.PCG

PLEKHH3 epi.PCG

IGSF6 epi.PCG

FAM168A epi.PCG

ZNF444 epi.PCG

TAF6L epi.PCG

B3GLCT epi.PCG

DACT1 epi.PCG

PDCL3 epi.PCG

WNT7B epi.PCG

CRADD epi.PCG

CELSR1 epi.PCG

ZNF140 epi.PCG

SH3BGRL epi.PCG

HS2ST1 epi.PCG

LHX4 epi.PCG

PRR11 epi.PCG

MELK epi.PCG

AL139353.1 epi.PCG

SLC4A5 epi.PCG

ZNF208 epi.PCG

TAF13 epi.PCG

GALNT1 epi.PCG

STOM epi.PCG

AD000671.2 epi.PCG

SYBU epi.PCG

BAG3 epi.PCG

GID8 epi.PCG

FXR1 epi.PCG

KIF3C epi.PCG

CCDC51 epi.PCG

HJURP epi.PCG

BRD8 epi.PCG

TRAF2 epi.PCG

TRMT2A epi.PCG

AC093323.1 epi.PCG

ERC2 epi.PCG

CSE1L epi.PCG

CUZD1 epi.PCG

YWHAH epi.PCG

TSPAN15 epi.PCG

TJAP1 epi.PCG

EED epi.PCG

PCF11 epi.PCG

PRSS50 epi.PCG

EFCAB14 epi.PCG

GPR176 epi.PCG

UBE2L3 epi.PCG

TNS4 epi.PCG

KIRREL1 epi.PCG

IWS1 epi.PCG

FRA10AC1 epi.PCG

BRD9 epi.PCG

ZNF169 epi.PCG

TMBIM4 epi.PCG

ITSN2 epi.PCG

MTF1 epi.PCG

TMC5 epi.PCG

DIPK2A epi.PCG

PLEC epi.PCG

IGFBP7 epi.PCG

CRACR2B epi.PCG

OARD1 epi.PCG

NRG2 epi.PCG

CEMIP epi.PCG

GOLIM4 epi.PCG

POLR3K epi.PCG

AKAP9 epi.PCG

DYM epi.PCG

CCNB1 epi.PCG

FAM83F epi.PCG

ZBTB12 epi.PCG

SORT1 epi.PCG

LRRFIP2 epi.PCG

SPATA5 epi.PCG

MMP1 epi.PCG

CROCC epi.PCG

ALG12 epi.PCG

CD86 epi.PCG

MAGEF1 epi.PCG

RNF168 epi.PCG

PGD epi.PCG

BCL2L2-PABPN1 epi.PCG

TBRG1 epi.PCG

POMT2 epi.PCG

TRAF5 epi.PCG

BRI3 epi.PCG

GNB4 epi.PCG

UHRF2 epi.PCG

SLC35C1 epi.PCG

TPRN epi.PCG

CRIM1 epi.PCG

FBXO3 epi.PCG

HIF1A epi.PCG

JRKL epi.PCG

PIGG epi.PCG

THOC2 epi.PCG

SLC33A1 epi.PCG

KCTD17 epi.PCG

RTL8C epi.PCG

OGT epi.PCG

AKAP11 epi.PCG

FGD5 epi.PCG

N4BP2L1 epi.PCG

PXDN epi.PCG

CYP27B1 epi.PCG

NCOA3 epi.PCG

THAP12 epi.PCG

TK1 epi.PCG

CCDC84 epi.PCG

EXOC2 epi.PCG

RBM23 epi.PCG

ZNF496 epi.PCG

FARSB epi.PCG

CSNK2B epi.PCG

ZNF469 epi.PCG

TUT1 epi.PCG

ZSCAN18 epi.PCG

CORO7 epi.PCG

SNTG2 epi.PCG

PGM2L1 epi.PCG

B4GALNT3 epi.PCG

FAM43A epi.PCG

AOX1 epi.PCG

MTFR1L epi.PCG

ACAD10 epi.PCG

RPL18A epi.PCG

ZNF429 epi.PCG

CD83 epi.PCG

MACF1 epi.PCG

AC114490.2 epi.PCG

DEF8 epi.PCG

EAF1 epi.PCG

FOSL1 epi.PCG

MCM2 epi.PCG

GAPDH epi.PCG

BCAR3 epi.PCG

ZNF771 epi.PCG

CD300A epi.PCG

NME6 epi.PCG

ARMC1 epi.PCG

TRAF3IP1 epi.PCG

WRAP73 epi.PCG

AP3S2 epi.PCG

FSTL1 epi.PCG

PIGU epi.PCG

CLCN4 epi.PCG

RBM25 epi.PCG

ADSS epi.PCG

ZNF141 epi.PCG

SKA3 epi.PCG

TCF19 epi.PCG

ATXN7L3B epi.PCG

ANKRD20A2 epi.PCG

TMEM92 epi.PCG

AASS epi.PCG

ITM2C epi.PCG

DOP1B epi.PCG

RHNO1 epi.PCG

POLL epi.PCG

FLCN epi.PCG

SH3BGRL2 epi.PCG

SUSD6 epi.PCG

RPL37 epi.PCG

SOCS6 epi.PCG

ZNF426 epi.PCG

RCN3 epi.PCG

GLIPR2 epi.PCG

KLK10 epi.PCG

DCAF7 epi.PCG

NBL1 epi.PCG

DCAF12 epi.PCG

ZNF148 epi.PCG

NUDT22 epi.PCG

NOSIP epi.PCG

DENR epi.PCG

MAL2 epi.PCG

TNRC6A epi.PCG

ESYT2 epi.PCG

MALSU1 epi.PCG

ATG12 epi.PCG

ITGA1 epi.PCG

SCFD1 epi.PCG

BANP epi.PCG

TRAK1 epi.PCG

RPS6KA4 epi.PCG

ESM1 epi.PCG

ZNF185 epi.PCG

NUFIP1 epi.PCG

DIS3L2 epi.PCG

CREBL2 epi.PCG

PM20D1 epi.PCG

JPT1 epi.PCG

CEACAM5 epi.PCG

ABLIM3 epi.PCG

RBP1 epi.PCG

BCL2A1 epi.PCG

SGSH epi.PCG

KPNA6 epi.PCG

WDR45 epi.PCG

PGM2 epi.PCG

SHCBP1 epi.PCG

MET epi.PCG

ILKAP epi.PCG

LAYN epi.PCG

IFT80 epi.PCG

SNIP1 epi.PCG

SLC25A22 epi.PCG

FHL2 epi.PCG

TMEM154 epi.PCG

RASL12 epi.PCG

FAM83D epi.PCG

C15orf48 epi.PCG

UGCG epi.PCG

SLIRP epi.PCG

RPL31 epi.PCG

TMEM184B epi.PCG

RPL26 epi.PCG

BCAT2 epi.PCG

GEMIN5 epi.PCG

NDUFC2 epi.PCG

MDM1 epi.PCG

ETV7 epi.PCG

ZNF398 epi.PCG

RBM6 epi.PCG

ZBTB41 epi.PCG

ABRACL epi.PCG

CAB39 epi.PCG

ATG2B epi.PCG

MTMR2 epi.PCG

KIFC3 epi.PCG

PMEPA1 epi.PCG

SLC16A13 epi.PCG

HAUS3 epi.PCG

DOK5 epi.PCG

STOX2 epi.PCG

PIP4K2C epi.PCG

SSR2 epi.PCG

RRM2 epi.PCG

CD14 epi.PCG

CDNF epi.PCG

SDC4 epi.PCG

CLIC3 epi.PCG

TTC14 epi.PCG

FOXC1 epi.PCG

GCNA epi.PCG

HLA-DQA2 epi.PCG

STK17B epi.PCG

PHF12 epi.PCG

ITPRIP epi.PCG

HECTD4 epi.PCG

PBK epi.PCG

STRA6 epi.PCG

NUCKS1 epi.PCG

ATP4A epi.PCG

SPINDOC epi.PCG

INTU epi.PCG

CLIP3 epi.PCG

ATP6V1G1 epi.PCG

1-Mar epi.PCG

SF3B3 epi.PCG

ERCC6L2 epi.PCG

TM2D1 epi.PCG

HLA-DPB1 epi.PCG

FLYWCH2 epi.PCG
[truncated: 295,909 more chars]
